# Misregulation of *AUXIN RESPONSE FACTOR 8* Underlies the Developmental Abnormalities Caused by Three Distinct Viral Silencing Suppressors in Arabidopsis

**DOI:** 10.1371/journal.ppat.1002035

**Published:** 2011-05-12

**Authors:** Florence Jay, Yu Wang, Agnès Yu, Ludivine Taconnat, Sandra Pelletier, Vincent Colot, Jean-Pierre Renou, Olivier Voinnet

**Affiliations:** 1 Institut de Biologie Moléculaire des Plantes, Centre National de la Recherche Scientifique, Université de Strasbourg, Strasbourg Cedex, France; 2 Swiss Federal Institute of Technology (ETH), Zurich, Switzerland; 3 Institute for Bioinformatics and Systems Biology, Helmholtz Zentrum München, German Research Center for Environmental Health (GmbH), Neuherberg, Germany; 4 Unité de Recherche en Génomique Végétale, Evry Cedex, France; University of Sydney, Australia

## Abstract

In Arabidopsis, micro (mi)RNAs and *trans*-acting (ta-si)RNAs synthesized directly or indirectly through the DICER-LIKE-1 (DCL1) ribonuclease have roles in patterning and hormonal responses, while DCL2,3,4-dependent small-interfering (si)RNAs are mainly involved in silencing of transposable elements and antiviral defense. Viral suppressors of RNA silencing (VSRs) produced by phytoviruses to counter plant defense may perturb plant developmental programs because of the collision of their inhibitory effects with the regulatory action of endogenous miRNAs and ta-siRNAs. This could explain the similar developmental aberrations displayed by Arabidopsis miRNA/ta-siRNA pathway mutants, including *dcl1*, and by some VSR-expressing plants. Nonetheless, the molecular bases for these morphological aberrations have remained mysterious, and their contribution to viral disease symptoms/virulence unexplored. The extent of VSR inhibitory actions to other types of endogenous small RNAs remains also unclear. Here, we present an in-depth analysis of transgenic Arabidopsis expressing constitutively HcPro, P19 and P15, three unrelated VSRs. We show that VSR expression has comparable, yet modest effects on known miRNA and ta-siRNA target RNA levels, similar to those observed using an hypomorphic *dcl1* mutation. However, by combining results of transcriptome studies with deep-sequencing data from immuno-precipitated small RNAs, additional, novel endogenous targets of miRNA and ta-siRNA were identified, unraveling an unsuspected complexity in the origin and scope-of-action of these molecules. Other stringent analyses pinpointed misregulation of the miR167 target AUXIN RESPONSE FACTOR 8 (ARF8) as a major cause for the developmental aberrations exhibited by VSR transgenic plants, but also for the phenotypes induced during normal viral infection caused by the HcPro-encoding *Turnip mosaic virus* (TuMV). Neither RNA silencing, its suppression by VSRs, nor the virulence/accumulation of TuMV was altered by mutations in *ARF8*. These findings have important implications for our understanding of viral disease symptoms and small RNA-directed regulation of plant growth/development.

## Author Summary

In the plant and animal RNA silencing pathways, small RNA molecules known as micro (mi)RNA and short-interfering (si)RNAs have key roles in development and antiviral defense, respectively. In turn, viruses counteract this defense by deploying specific virulence factors, referred to as Viral Suppressors of RNA silencing (VSRs), which target distinct steps of the host silencing machinery. In the model plant species *Arabidopsis thaliana*, transgenic expression of distinct VSRs often incurs a set of strikingly recurrent developmental anomalies that resemble those triggered by viral infections. While these defects have been assumed to result from a general interference of VSRs with silencing-based mechanisms controlling cellular growth, their exact molecular basis has remained largely elusive. Here, we address this issue by demonstrating that misregulation of a single transcript encoding the AUXIN RESPONSE FACTOR 8, a target of miR167, underlies most, if not all, of the defects caused by VSR expression, both in transgenic and in an authentic infection context. Our study also highlights the value of VSRs as generic tools for the discovery or validation of endogenous RNA silencing targets. These results also have implications for our understanding of small RNA-based regulations in plants, and shed light on the possible origin of some of the symptoms elicited by viral diseases.

## Introduction

RNA silencing in Arabidopsis entails the activities of four distinct paralogs of the RNaseIII Dicer, producing small RNAs with specialized functions [1]. DICER-LIKE 1 (DCL1) predominantly synthesizes microRNAs (miRNAs), 19-to-24-nucleotide (nt) in length, from non-coding primary transcripts called pri-miRNAs containing imperfect stem–loop structures. Stepwise nuclear pri-miRNA processing produces mature miRNAs that are then 2′-O methylated by HUA ENHANCER 1 (HEN1) and exported to the cytoplasm [2], [3]. One miRNA strand is stabilized in an RNA-induced silencing complex (RISC) containing, chiefly, the ARGONAUTE 1 (AGO1) silencing effector protein, whereas the passenger miRNA strand, or miRNA*, is degraded. The miRNA-loaded AGO1 then guides post-transcriptional gene silencing (PTGS) of complementary or partially complementary mRNAs by inhibiting their stability and/or translation [4]. Hypomorphic mutations in *DCL1*, *HEN1* or *AGO1* cause severe developmental abnormalities, highlighting the important role for miRNAs in plant development. Accordingly, many miRNA targets are mRNAs encoding transcription factors required for patterning, control of cell identity and elongation, including transcripts for AUXIN RESPONSE FACTORs (ARFs), which modulate plant responses to the hormone auxin [5]. Nonetheless, other classes of miRNAs regulate non-developmental processes including basal metabolism and plant adaptation to biotic or abiotic stress [4].

Unlike miRNAs, populations of *cis*-acting, 24nt-long siRNAs produced by DCL3 direct cytosine methylation and other chromatin modifications at the endogenous loci that generate them, including transposable elements, DNA repeats, and complex gene arrays [6]. DCL4 generates 21nt-long siRNA populations that guide PTGS of endogenous transcripts, including *trans*-acting siRNAs (ta-siRNAs), the biogenesis of which is initiated by miRNA-directed cleavage of specific, often non-coding precursor transcripts. This promotes complementary strand synthesis mediated by the RNA-DEPENDENT RNA POLYMERASE RDR6 that generates long dsRNA processed by DCL4 [7], [8]. The ta-siRNAs then guide AGO1 to repress target mRNAs including those of ARF3 and ARF4, which are important determinants of leaf development during post-embryonic growth [9], [10]. Other classes of endogenous siRNAs are similarly loaded into AGO1, presumably also to direct endogenous PTGS. These include DCL4-dependent, 21nt-long, and DCL2-dependent, 22nt-long, siRNA populations that are produced from small hairpins or extensively base-paired RNA formed upon transcription of inverted-repeat (IR) loci. These hairpin and IR loci may also attract DCL3 activity, leading to the accumulation of corresponding 24nt-long siRNAs [11]. DCL4, and to a lesser extent DCL2 and DCL3, additionally has a key role in antiviral defense by dicing dsRNA produced during replication of phytovirus genomes (reviewed in [12]). The resulting siRNAs are methylated by HEN1 and incorporated into one or several AGO proteins directing PTGS of viral RNA as part of antiviral RISCs. AGO1 and AGO7 are good candidates as antiviral RISC effectors because hypomorphic *ago1* and null *ago7* mutants are hyper-susceptible to several viruses [13], [14].

As expected from the never-ending molecular arms race that characterizes nearly all host-parasite interactions, phytoviruses have evolved a vast array of proteins, called viral suppressors of RNA silencing (VSRs), in order to multiply and invade plants systemically [12]. Studies in transgenic plants expressing RNAi constructs (as a surrogate to virus infection) have shown that VSRs may target many steps of antiviral silencing, including small RNA processing, stability and activity *via* AGO effectors (reviewed in [15]). For instance, homo-dimers of the tombusviral P19 protein sequester viral- or hairpin-derived siRNA duplexes in a size-dependent manner to prevent their effective loading into antiviral RISCs [16]. Many antiviral silencing factors are components of cellular pathways regulating host gene expression, including, and of note, HEN1, which methylates and protects all endogenous classes of small RNAs, as well as AGO1 and AGO7, effectors of miRNAs, ta-siRNAs and IR-derived siRNAs. Consequently, some VSRs are expected to interfere with endogenous silencing pathways as part of their counter-defensive action and, thus, to perturb plant developmental programs.

This hypothesis has been supported by various studies of Arabidopsis plants expressing constitutively distinct types of VSRs: in many cases, such plants display morphological abnormalities in leaves and inflorescences, reduced stature and fertility reminiscent of defects exhibited by hypomorphic miRNA mutants [17], [18], [19]. Furthermore, transgenic plants expressing VSRs show alterations of ta-siRNA/miRNA and ta-siRNA/miRNA target levels. For instance, P19 sequesters and thereby stabilizes host miRNAs/miRNA* duplexes, preventing the activity of the mature miRNA strand [18]. Other transgenically expressed VSRs, such as the potyviral HcPro, cause a consistent elevation in mature miRNA steady state levels, possibly as a consequence of perturbed HEN1 activity [17], [18], [19]. Arabidopsis plants stably expressing the P15 protein of pecluviruses, by contrast, do not display altered mature miRNA levels, but, like HcPro and P19 transgenics, they accumulate ectopically miRNA and ta-siRNA target transcripts, suggesting a general perturbation in miRNA-RISC activity [18].

The above and other studies have prompted the popular assumption that the developmental phenotype of VSR transgenic plants is an unintended consequence of the primary inhibition of the antiviral silencing machinery at some steps colliding with the host miRNA/ta-siRNA pathways. This assumption, however, may be only partly true because it assumes that the miRNA pathway does not contribute actively to antiviral defense, and that, as a corollary, plant viruses do not rewire endogenous silencing pathways in order to thrive in their hosts. However, miRNAs and other cellular small RNAs have recently emerged as key regulators of Arabidopsis basal and race-specific resistance against many pathogens, including viruses (reviewed in [12], [20]). Therefore, inhibition of endogenous small RNA pathways by VSRs might also reflect a deliberate viral strategy to inhibit such immune systems. By extension, it could be argued that the onset of developmental or hormonal defects as a consequence of suppressed miRNA or endogenous siRNA activities might optimize the replication and spread of at least some viruses. Conversely, suppression of endogenous silencing pathways may be inconsequential to other virus types, and this may explain why some VSRs have narrower impacts in transgenic Arabidopsis, merely inhibiting RNAi and antiviral defense. For instance, the P6 protein of *Caulimoviridae* targets the DCL4-interacting protein DRB4 during siRNA biogenesis, without noticeable incidence on miRNA regulation in transgenic plants [21]. A related issue is whether the inhibition (targeted or fortuitous) of endogenous small RNA functions observed with certain VSR transgenes recapitulates some of the disease symptoms normally elicited by viruses during authentic infections. Indeed, those studies have mostly involved, so far, constitutive or inducible VSR expression in a much broader tissue range than is expected from natural infections (discussed in [12], [22]).

An additional question pertains to the exact molecular underpinnings of the morphological abnormalities induced by transgenic expression of P19, HcPro, P15 or other VSRs in Arabidopsis. The broad ectopic accumulation of miRNA targets seen in those plants would intuitively argue in favor of pleitropy owing to many compromised regulatory and developmental pathways. This idea is challenged, however, by the surprising recurrence and discrete nature of the observed defects, independent of the VSR under study (though their strength may vary depending on VSR expression levels). Hence, rosette leaves are invariably narrow, serrated and curled, the rosette diameter and leaf area are reduced, as are the weight of total aerial tissue and the length of primary bolts. P19, HcPro and P15 plants also display inflorescences with typically narrow and unusually long sepals; organs within internal whorls are usually exposed prior to opening, and flowers fail to release pollen, resulting in male sterility [17], [18], [19]. These recurrent and discrete anomalies thus suggest that misregulation of only a discrete number of endogenous genes accounts for the VSR phenotype. The identity of these targets remains unknown, however, as does the nature of the possible endogenous small RNA pathway(s) (i.e. miRNA, ta-siRNA, endogenous IR-derived siRNAs) involved. Moreover, although an effect of VSRs at the level of AGO action is usually invoked to unify these observations, additional actions of VSRs on chromatin or primary miRNA/ta-siRNA transcription have never been formally ruled out. For instance, histone acetylation/deacetylation was recently identified as a broad-spectrum chromatin-based mechanism regulating miRNA production in Arabidopsis [23]. This overall lack of understanding of the VSR effects in transgenic settings has limited the use of these factors as tools for the identification of potentially novel endogenous small RNAs and their associated targets, both in Arabidopsis and other plant species. It was indeed anticipated that VSRs could be possibly used as weak alleles of RNA silencing mutations, but with a broader output because of the likely simultaneous interference of these factors with multiple endogenous silencing pathways [24].

Through a systematic, comparative analysis of Arabidopsis lines over-expressing the tombusviral P19, potyviral HcPro or pecluviral P15 VSRs, the present study addresses many of the outstanding issues raised above. This analysis notably uncovers the as yet unexplained molecular feature that underlies the post-embryonic developmental phenotype exhibited in common by the three VSR transgenic plants. Moreover, this study establishes that the same molecular bases account for the developmental, but not metabolic, symptoms normally elicited by an authentic virus infection. Finally, our work demonstrates that virus-induced developmental aberrations, on the one hand, and pathogen virulence as a consequence of antiviral silencing suppression, on the other, can be uncoupled. These findings not only shed light on hitherto unsolved issues of viral diseases, but they also challenge current views on the roles and impact of endogenous small RNAs on plant growth and development.

## Results

### VSRs do not impact chromatin-level silencing or primary miRNA transcription, and have only modest effects on the accumulation of known miRNA and ta-siRNA target transcripts

The systematic analysis reported in this study involved previously characterized Arabidopsis lines expressing the potyviral HcPro, tombusviral P19 and pecluviral P15 VSRs under the constitutive 35S promoter from *Cauliflower mosaic virus* [18, material and method]. These lines contain an additional transgene encoding an RNAi inverted-duplication of the *CHALCONE SYNTHASE* gene (*CHS*), which prevents pigmentation of the seed coat. The VSR transgenics, by contrast, have a brown seed coat owing to RNAi suppression [18].

We first investigated the possibility that VSRs could affect chromatin-level silencing of repeat elements mediated by small RNAs, or accumulation of known primary miRNA transcripts (pri-miRNAs). To this end, transcript levels and two histone modifications were analyzed along the Arabidopsis chromosome 4 using a custom-made tiling array (GSE24692; [25]). One percent or less of the 21000 probes on the tiling array reported statistically significant differences in transcript accumulation in leaves or inflorescences between WT plants and VSR transgenic plants (Figure S1). Likewise, histone marks were largely unaffected by VSR expression indicating that these proteins interfere with RNA silencing at the post-transcriptional level, consistent with previous studies showing that none of the three VSRs prevent accumulation of mature miRNAs [17], [18], [19].

**Figure 1:**
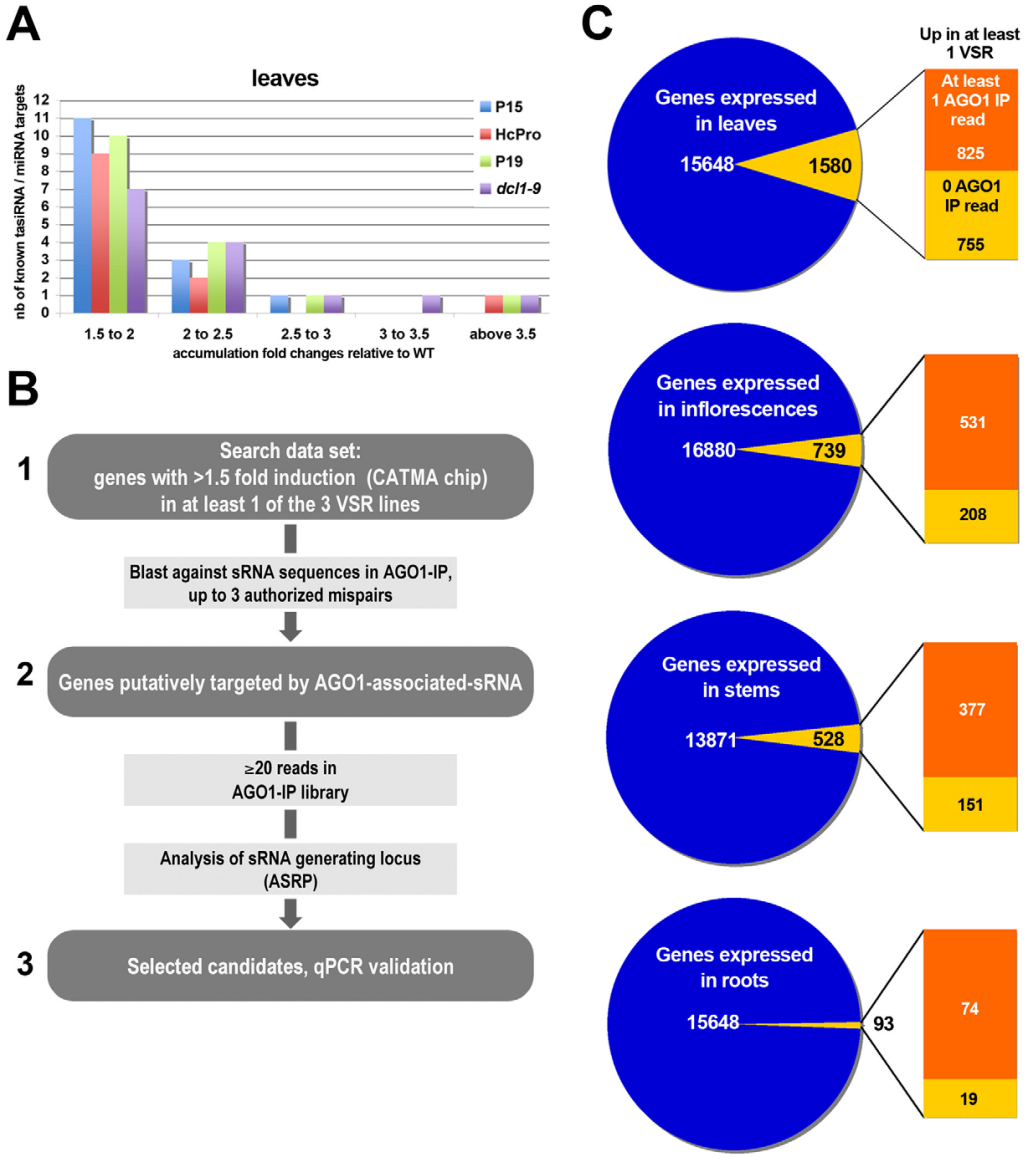
VSRs incur modest yet consistent expression changes to known miRNA and ta-siRNA target transcripts in Arabidopsis. (**A**) CATMA gene chip analysis of the ranges in expression changes of known miRNA and ta-siRNA target transcripts that are up-regulated in leaves of transgenic VSR plants and *dcl1-9* mutant, as compared to WT plants. Data for other organs are available in Figure S3-S5. nb: number. (**B**) Diagram summarizing the strategy used in this study for *de novo* RNA silencing target identification through AGO1-IP small RNA deep sequencing data. (**C**) Proportions of the total number of genes expressed in each organ analyzed, which were found up-regulated by at least 1.5 fold in at least one VSR transgenic background compared to non-transgenic Arabidopsis. Also indicated is the fraction of up-regulated genes with at least one matching AGO1-IP small RNA read, as assessed by computer-based prediction (see methods).

We conclude that these factors likely interfere with Arabidopsis silencing pathways downstream of Dicer, presumably by inhibiting RISC-mediated repression of target transcripts, which may occur, at least partly, at the mRNA stability level. Consequently, we decided to analyze the changes in mRNA accumulation observed between WT and VSR-expressing plants, using a microarray approach (Data deposited at the Gene Expression Omnibus [GEO], accession GSE24693). In order to define a threshold value for such changes, we first examined, in inflorescences, stems, leaves and roots of the VSR transgenic plants, the average expression changes of all known Arabidopsis miRNA and ta-siRNA target transcripts, as available in the miRbase (http://www.mirbase.org) and ASRP (http://asrp.cgrb.oregonstate.edu; [26]) depositories. We found that more than 90% of all known miRNA and tasiRNA target transcripts did not differentially accumulate in WT versus VSR plants: their accumulation was within the 0.8–1.2 fold range in all four organs of the VSR transgenic plants (Figure S2). A similar value was obtained upon analysis of *dcl1-9* plants, which display vastly reduced miRNA levels (Figure S2). Strikingly, in leaves, only 30% of all target transcripts were found to over-accumulate in at least one VSR transgenic line, as compared to WT plants, and this figure was reduced to 11% in the *dcl1-9* mutant (Figure S3; results for the other organs are presented in Figure S4A-S6A). Moreover, for those over-accumulating target mRNAs, expression changes were mostly in the 1.5-2 fold range (Figure 1A; Figure S4B-S6B). These results are in line with those of two separate microarray studies involving additional alleles of the *dcl1* mutation in at least two distinct Arabidopsis ecotypes [27], [28]. We conclude that expression of P19, P15 or HcPro, like the *dcl1-9* mutation, incurs only modest changes to the accumulation of some miRNA and ta-siRNA target transcripts. We further propose from this analysis that variations in gene expression above the 1.5 fold threshold can be ascribed to putative effects of VSRs interfering with endogenous PTGS pathways.

### VSRs interfere with the activity of many types of endogenous, AGO1-dependent small RNAs

**Figure 2:**
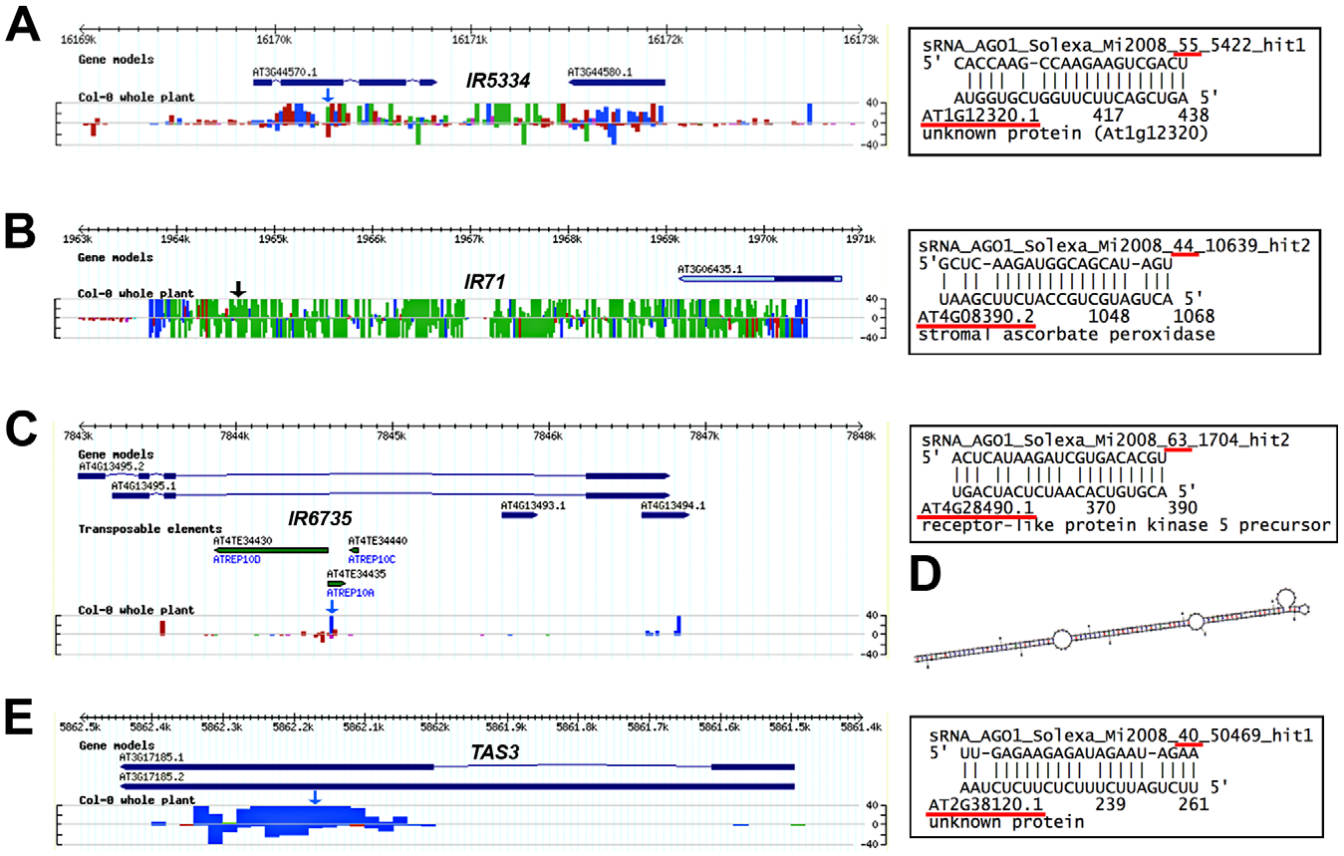
Using the AGO1-IP read filter in conjunction with VSR microarray data uncovers possibly novel *IR*- and*TAS*-derived siRNA target transcripts with altered accumulation by VSRs. (**A-E**) Left panels provide ASRP genome browser views of the small RNA loci of origin. Colored arrows indicate the position and length of the small RNA. Blue, green and red labels indicate 21nt-long, 22nt-long and 24nt-long siRNA species, respectively. A black color signifies small RNAs with length diverging from the above. The right panels depict predicted target sites alongside the small RNA identification number (as in [30]), AGO1-IP read value (underlined in red) and number of loci of origin (hit). The gene identification number of the predicted target is underlined in red. (**A–C**) Inverted-repeat (IR)-derived siRNAs and their predicted targets, At1g12320 (in leaves; A), At4g08390 (in stems and leaves; B) and At4g28490 (in stems and leaves; C). (**D**) Predicted secondary structure of the transposon-derived *IR6735*. (**E**) A 21nt-long siRNA derived from the *TAS3* locus predicted to target the At2g38120 transcript in leaves. For each example, statistically significant up-regulation of gene expression was validated in two independent qRT-PCR analyses of total RNA extracted from the indicated tissues.

**Figure 3:**
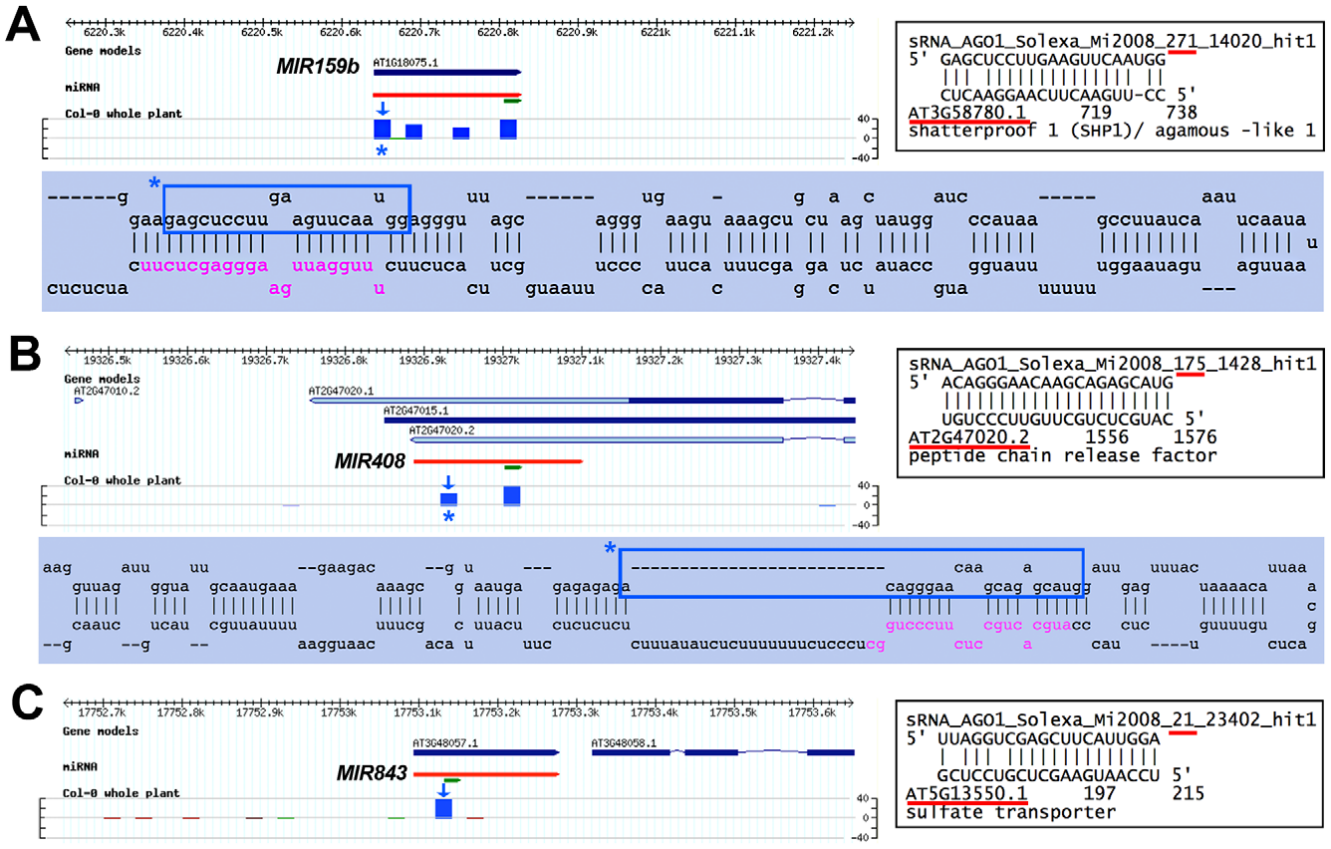
Using the AGO1-IP read filter in conjunction with VSR microarray data uncovers possibly novel miRNA* and orphan miRNA target transcripts whose accumulation is altered by VSRs. As in Figure 3, the upper panels in A–C provide ASRP genome browser views of the small RNA loci of origin. (**A-B**) Abundantly AGO1-loaded miRNA passenger strands (miR*) for miR159b and miR408, alongside their predicted targets, At3g58780 (stems and leaves; A) and At2g47020 (flowers; B). The blue panels show the predicted stem-loop structures of the corresponding miRNA precursors, in which the miRNA* sequence is boxed in blue. (**C**) The sulfate transporter At5g13550 transcript is a putative target for the Arabidopsis-specific miR843 in stems. For each example, statistically significant up-regulation of gene expression was validated in two independent qRT-PCR analyses of total RNA extracted from the indicated tissues.

Beside their effect on ta-siRNA and miRNA activities, VSRs might also compromise the action of additional species of AGO1-bound small RNA, including endogenous siRNAs, natural antisense (nat-) or long siRNAs [29], or even heterochromatic small RNAs, and this may contribute to the developmental phenotype displayed by HcPro, P15 and P19 transgenic plants. To investigate this aspect exhaustively and in an unbiased manner, we exploited available small RNA deep-sequencing data from AGO1-immno-precipitates (IPs) obtained from a mixture of Arabidopsis tissues including those investigated in the present study [30]. In each organ, we selected mRNA (i) displaying ≥1.5 fold expression changes compared to WT in at least one of the three VSR lines and (ii) exhibiting high complementarity (not more than three authorized mispairs) to one or more AGO1-loaded small RNA (Figure 1B, step 1-2). We found that more than half of the transcripts that are up-regulated in at least one VSR have at least one matching AGO1-IP small RNA in the various organs analyzed (Figure 1C). This approach was further refined by taking into account the number of unique small RNA reads from AGO1-IP deep-sequencing data ([30]; Figure 1B, step 2–3). Based on an analysis of all AGO1-loaded sRNAs mapped on all their predicted targets, a conservative threshold of ≥20 AGO1 reads was chosen in order to identify small RNAs that might reliably engage the transcripts identified in step 1–2 into regulatory interactions (Figure S7). Some of the results of this refined study are presented in Figure 2, 3 and Figure S8 (showing mostly small RNAs mapping to unique genomic regions) and were all validated by two independent qRT-PCR analyses of RNA extracted from the VSR transgenic versus WT tissues (Table S1). The reader is referred to Table S2 and Text S1 for the complete list of putative target transcripts, their matching small RNAs, and corresponding AGO1-IP read values.

This analysis notably uncovered that VSR expression enhances the accumulation of several potential *trans*-targets of AGO1-bound siRNAs, 21–22nt in size, that originate from long dsRNA formed upon transcription of inverted gene-duplications (i.e. IRs). Although IRs are commonly detected along the Arabidopsis genome and frequently associated to siRNA production [31], their targets (if any) are difficult to identify because of the shear amount and diversity of siRNAs generated at these loci. Figure 2A shows, for instance, that VSR expression elevates the levels of a putative target (At1g12320, encoding an unknown protein) of a 21nt-long siRNA mapping to *IR5334*, which is on chromosome 3 and produces heterogeneous populations of 21nt, 22nt and 24nt siRNAs. Similar findings were made for At4g08390 (encoding a stromal ascorbate peroxidase; Figure 2B), a putative target of a 20nt siRNA derived from the >7kb-long *IR71* (Chromosome 3), and for At4g28490 (encoding a receptor-like protein kinase 5 precursor), which is likely regulated by a 21nt siRNA derived from *IR6735* (Figure 2C–D). The analysis also revealed that VSR expression enhances the accumulation of a putative novel target of a *TAS3-derived small RNAs (*At2g38120, Figure 3E). *TAS* loci typically produce populations of phased, 21nt-long siRNAs that are loaded into AGO1, many of which have as yet unidentified functions.

Figure 3A–B illustrates additional striking cases in which VSRs cause increased accumulation of transcripts that are likely regulated *via* miRNA* strands upon their efficient loading into AGO1. This is the case of the MADS box gene *SHATTERPROOF 1* (*SHP1*), involved notably in seed dispersal through regulation of valve dehiscence and also lateral root initiation [32], [33]. The *SHP1* open-reading frame displays near-perfect complementarity to miR159b*, which is nearly as abundant as miR159b itself (Figure 3A). Similarly, VSR transgenic plants displayed elevated levels of the At2g47020 transcript, which is antisense and, therefore, perfectly complementary to miR408* (Figure 3B). This configuration likely allows *cis* regulation of At2g47020 expression by miR408*, reminiscent of several natural-antisense transcripts/miRNA pairs that have been documented in rice [34], but, as yet, not in Arabidopsis. Consistent with regulatory roles for both miR159b* and miR408* and with their interference by VSR expression, SHP1 and At2g47020 levels were similarly up-regulated in corresponding organs of *dcl1-9* mutant plants (http://urgv.evry.inra.fr/CATdb/; Project: GEN-107). The sulphate transporter mRNA At5g13550 was also up-regulated in VSR transgenic plants (Figure 3C) and was identified as a likely target of miR843, an Arabidopsis-specific miRNA with previously unassigned targets or functions.

These and additional examples presented in Figure S8 show that VSR expression interferes with AGO1-dependent regulatory functions that extend beyond conventional miRNA-mediated repression and may involve a large variety of endogenous small RNA species including possible *trans*-acting siRNAs derived from repeats and transposable elements. Consequently, applying the microarray/AGO1-IP approach to individual VSR lines could not singularize alterations to one specific RNA silencing pathway, which could have shed light on the developmental phenotype shared by P15, HcPro and P19 transgenic plants. We thus sought to design an alternative method to address this issue independently of AGO1-IP small RNA read values.

### The *arf8* mutation abolishes all VSR-induced developmental defects

**Figure 4:**
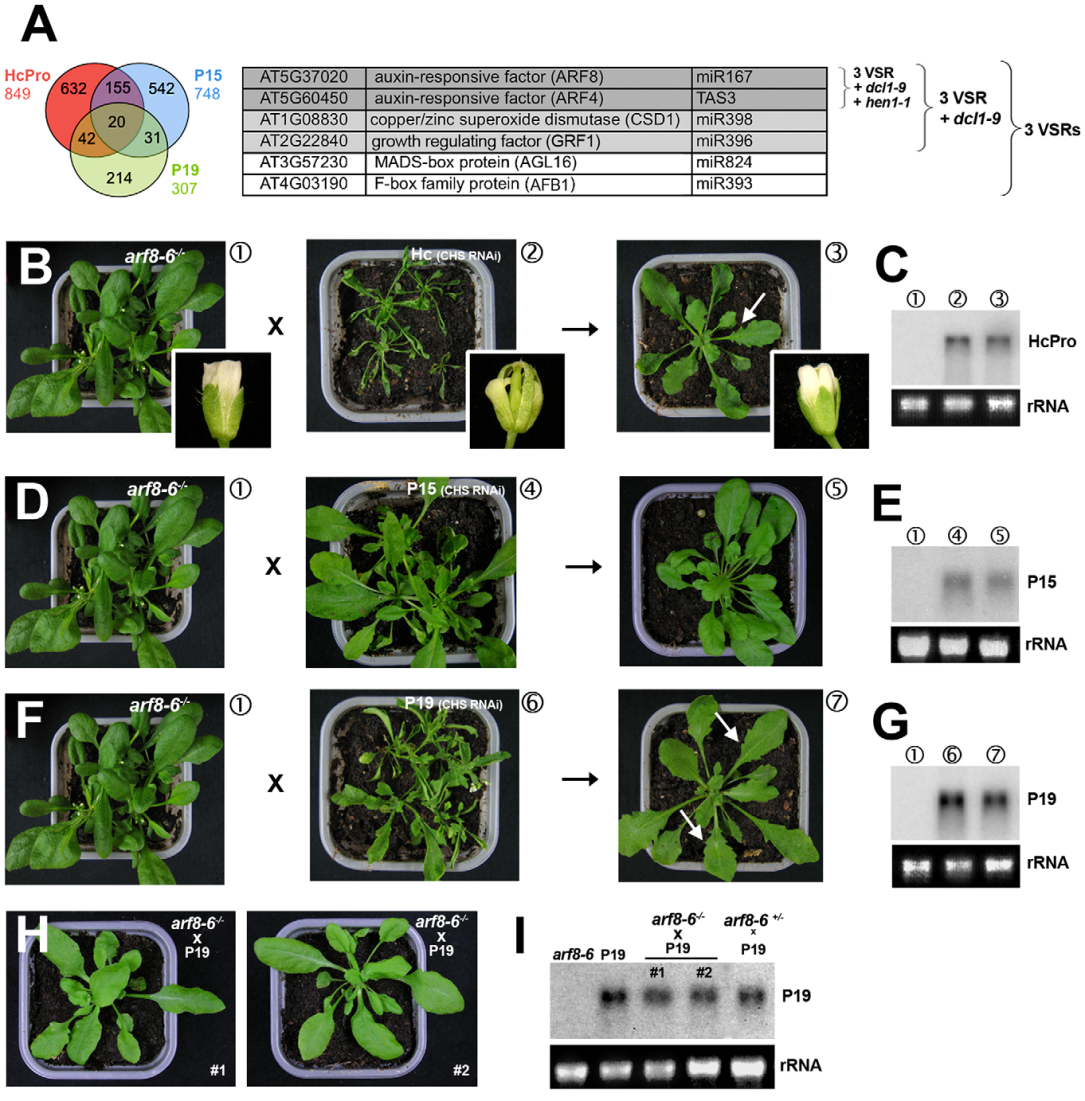
Heterozygous and homozygous *arf8* mutant backgrounds respectively attenuate and alleviate the developmental phenotypes incurred by VSRs. (**A**) The Venn diagram on the left shows that only a modest number of transcripts are up-regulated in common in leaves of the three VSR transgenics. The table shows that refining the analysis with additional filters based on transcripts up-regulated in *dcl1-9* (pale grey) and *hen1-1* (dark grey) backgrounds singularizes ARF4 and ARF8, respectively direct targets of miR390 and miR167, as strong candidates for the underlying developmental defects seen in VSR transgenics. (**B–C**) Strong reduction of leaf and inflorescence defects (inlays) caused by HcPro in F1 progenies of crosses between *arf8* mutants and HcPro transgenics carrying the *CHS* RNAi transgene (B). The Northern blot in (C) shows comparable accumulation of HcPro transcripts in the various backgrounds involved in the crosses. (**D–E**) same as (B–C) for P15 transgenics with the *CHS* RNAi background. (**F–G**) Same as (B–C) for P19 transgenics with the *CHS* RNAi background. Arrows indicate the presence of slight leaf serration in F1 progeny plants. (**H–I**) Complete alleviation of developmental defects and sterility of P19 transgenic plants (*CHS* RNAi background) in the homozygous *arf8* mutant background. Northern analysis in (I) confirms comparable P19 levels in the various backgrounds indicated. Plants #1 and #2 where retrieved through independent genotyping in populations of P19 plants with homozygous or heterozygous *arf8* mutant genotype. rRNA: ethidium bromide staining of ribosomal RNA provides a control for equal RNA loading.

We reasoned that the recurrent phenotypic abnormalities observed in VSR plants are mostly manifested in leaves and, therefore, likely accounted for by the ectopic expression of one or several silencing-regulated genes up-regulated in common in the three VSR lines. Based on this hypothesis, we found that only a subset of 20 transcripts had this stringent attribute in VSR leaves (Figure 4A, diagram; Table S3), among which approximately half were involved in basic metabolism or enzymatic processes that were unlikely to account for the leaf developmental phenotype (Table S3). Among the remaining nine candidate transcripts, six were direct or indirect targets of known miRNAs (Figure 4A, table), of which four were also up-regulated in leaves of the *dcl1-9* mutant plants. Given the importance of miRNAs in plant development, we decided to focus on this subset of candidates, which was further refined using a final filter based on organ-specific analyses of the *hen1-1* mutant (Figure 4A, table). Because HEN1 methylates and thereby protects all plant small RNA classes from degradation, *hen1* mutants accumulate miRNAs to low levels [3]. Applying the same procedure to the other organs of VSR transgenic plants (Table S4) identified gene sets that, as in leaves, were enriched for transcripts targeted by the miR398 family, involved in copper/zinc homeostasis, and for mRNAs encoding the Auxin response factors ARF8 (targeted by miR167; [35]), ARF4 and ARF3/ETTIN (both targeted by miR390-dependent *TAS3;* [9], [10]).

Based on the role of auxin in plant organogenesis [36], the three ARFs ectopically accumulating in the VSR lines were further investigated. We reasoned that a key contribution of those factors to the developmental defects of VSR lines would be diagnosed by an attenuation of the phenotype following introgression of either the *arf8*, *arf4* or *arf*3 mutations. In other words, it was predicted that some of the above mutations would act as general, second-site suppressors of the VSR phenotype. As for most miRNA target genes (Figure 1A), expression changes for *ARF8* and *ARF4* were within the 1.5–2 fold range in the leaves of the three VSR lines, while those of *ARF3* were below the 1.5 fold threshold in leaves of P15 and HcPro plants (Figure S9A). It was thus anticipated that the effects of mutations in at least *arf8* or *arf4* would be possibly manifested in the heterozygous state. Analysis of F1 progenies from the respective crosses to VSRs (in the *CHS* RNAi background) did not reveal any effect of the *arf4–2* or *arf3–2* heterozygous mutations (Figure S9B-D and data not shown). VSR transgenic plants with the heterozygous *arf8–6* background [37], by contrast, displayed dramatically attenuated developmental defects (Figure 4B, 4D and 4F), which could not be attributed to changes in expression levels of the cognate silencing suppressor mRNAs as compared to those found in the parental VSR lines (Figure 4C, 4E and 4G). In addition, as expected, *ARF8* expression levels were reduced in these F1 heterozygous mutant plants (Figure S9E and data not shown). This *arf8*-dependent phenotype attenuation was not only observed in leaves, but also in inflorescences (Figure 4B) such that fertility of all three VSR lines was restored to near WT levels. While those VSRs with initially strong phenotypes in parental lines (HcPro and P19) still exhibited a low degree of leaf serration in the *arf8–6* heterozygous background (Figure 4B, 4F, arrows), they were essentially undistinguishable from WT plants when the *arf8–6* mutation was brought to homozygocity, as exemplified with the independently genotyped [P19 x *arf8–6^-/-^*] plants presented in Figure 4H–I. All these effects were highly specific for *arf8* because they were not observed with mutations in *ARF6*, a close paralog of *ARF8* also regulated by miR167, which has been implicated in similar developmental processes (Figure S9C–D). We conclude that heterozygous or homozygous *arf8* is sufficient to respectively attenuate or abolish the developmental defects caused by the three VSRs, strongly suggesting that all these defects have a sole and common ARF8-dependent origin.

### The *arf8* mutation does not suppress miRNA-directed gene silencing or RNAi, nor does it interfere with VSR-mediated inhibition of the two processes

**Figure 5:**
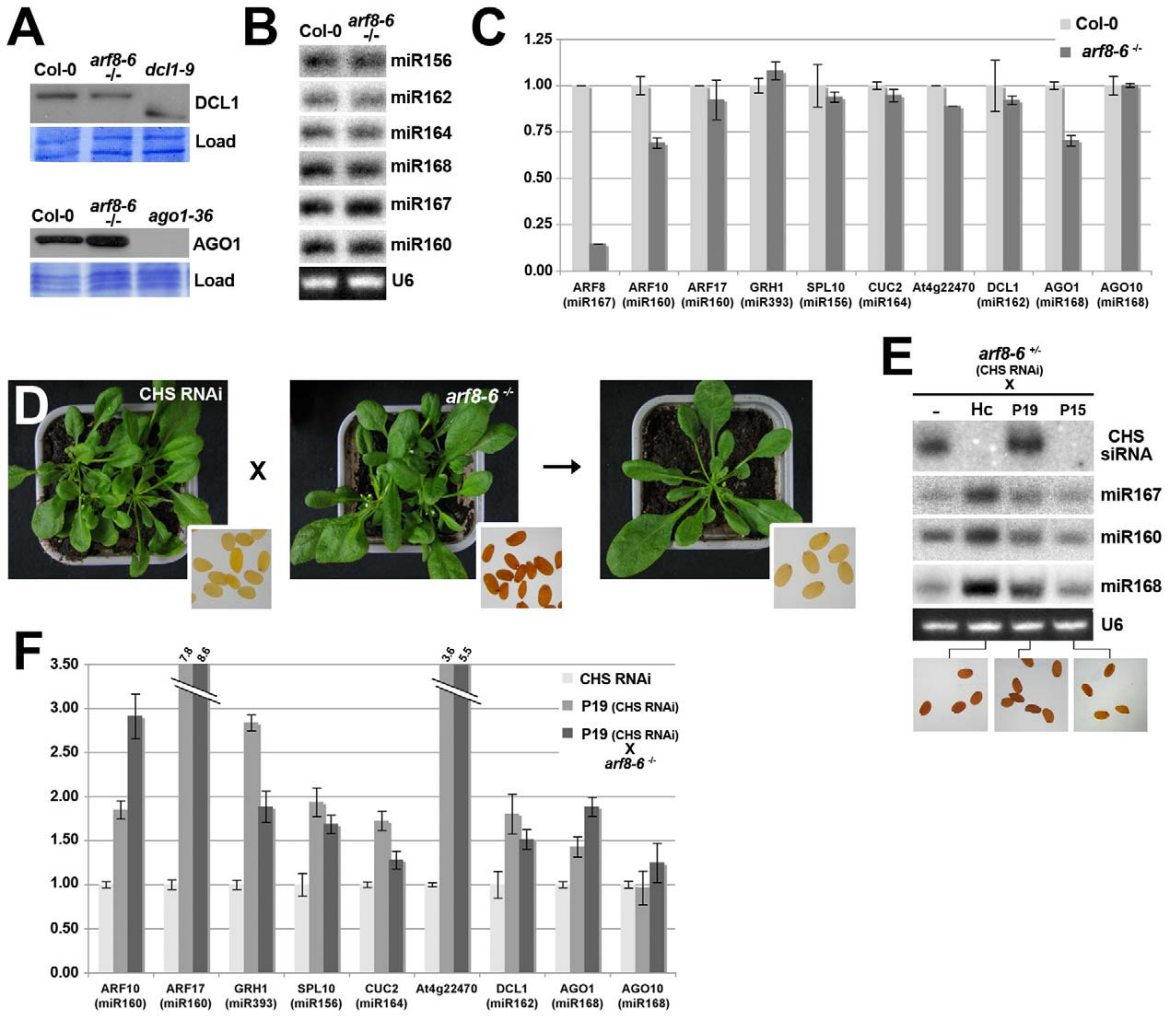
RNAi and miRNA-mediated gene silencing are not compromised by the *arf8* mutation. (**A**) Western analysis of DCL1 and AGO1 accumulation in *arf8* homozygous seedlings compared to WT seedlings. Negative controls were plants with the *dcl1–9* genotype, which accumulate a truncated form of the DCL1 protein, and null *ago1–36* mutants. Load: coomassie staining provides a control for equal loading of total proteins. (**B**) Northern analysis of various endogenous miRNAs in Col-0 or homozygous *arf8* mutant seedlings. (**C**) qRT-PCR analysis of transcript levels from various targets of the miRNAs studied in (B), showing intact miRNA-mediated repression in *arf8* mutants as compared to WT plants. (**D**) RNAi of *CHS*, diagnosed by a loss-of-seed pigmentation (inlays), remains unaltered in plants with the *arf8* ^-/-^ genotype. (**E**) Northern analysis of CHS siRNAs and endogenous miRNA accumulation in VSR transgenics with the heterozygous *arf8* mutant background (as depicted in Figure 5B-I). Note the strong decrease in siRNA levels caused by HcPro and P15 as well as the slight shift in electrophoretic migration and enhanced accumulation incurred to miRNAs by P19 and HcPro, respectively. The inlays at the bottom show that RNAi of *CHS* remains suppressed by the three VSRs in the *arf8* mutant background, as diagnosed by the dark-brown seed coloration. (**F**) qRT-PCR analysis of transcript levels from various targets of the miRNAs studied in (B) in the P19 transgenics carrying the homozygous *arf8* mutation (*CHS* RNAi background), as depicted in Figure 5H. Reference plants used in the analysis were line *CHS* RNAi and its P19 transgenic derivative (P19 *CHS* RNAi) with a wild type background. Off-scale values for ARF17 and At4g22470 (a novel small target shown in Figure 4A) are indicated by double-dashed lines. U6: oligonucleotide hybridization of the ubiquitous U6 small nucleolar RNA provides a control for equal RNA loading.

A possible cause of the effects of *arf8* on the VSR phenotype is that ARF8 might itself influence small RNA biogenesis or activity. We ruled out this possibility, however, for three reasons. First, the protein levels of the miRNA-processing enzyme DCL1 were not changed dramatically in *arf8–6^-/-^* mutant as compared to WT plants, as were the levels of AGO1, the main effector of miRNA and siRNA actions (Figure 5A). Likewise, inspection of available transcriptome data for *arf8–3^-/-^ arf6–2^-/-^* double mutant plants did not reveal any significant changes in the transcript levels of major PTGS effector proteins and endogenous suppressors of silencing, as compared to WT plants, with the notable exception of AGO7 (Table S5). Second, accumulation of a variety of miRNAs -including miR162 and miR168 regulating, respectively, the levels of AGO1 and DCL1 transcripts- was nearly the same in *arf8–6^-/-^* mutant plants as it was in WT plants (Figure 5B). Third, accumulation of the endogenous targets of those miRNAs was largely unaffected in *arf8–6^-/-^* mutant compared to WT plants (Figure 5C). Using crosses to the *CHS* RNAi line [18], we also confirmed that the *arf8–6* mutation did not affect PTGS mediated by siRNAs derived from long dsRNA, as the seed coat of all progeny plants remained pale, an indicator of *CHS* silencing (Figure 5D; [18]). Suppression of *CHS* RNAi, manifested as brown seed coats, was, however, still observed in the VSR lines with the *arf8–6* heterozygous mutation, which nonetheless exhibited strongly attenuated developmental phenotypes (Figure 4B, 4D and 4F; Figure 5E). Moreover, the known effects of VSRs on CHS siRNA and endogenous miRNA accumulation were still observed in those crosses: as expected, both P15 and HcPro caused a strong reduction in 21nt CHS siRNA levels, while these remained unaffected by P19 (Figure 5E). Also as reported previously [18], HcPro and P19 (but not P15) caused respectively an increased accumulation and a slight mobility shift of endogenous miRNAs (Figure 5E). qRT-PCR analyses confirmed, additionally, that VSRs in both the heterozygous and homozygous *arf8–6* mutant background still displayed enhanced accumulation (a 1.5–2 fold range on average) of several miRNA target transcripts, as observed in the parental VSR lines (Figure 5F and data not shown). We conclude that suppression of developmental defects by the *arf8–6* mutation in the VSR transgenic plants is merely accounted for by the correction of ARF8 transcript levels, independently of any other effects on RNA silencing. Therefore, ectopic ARF8 accumulation, diagnosed by a ∼2 fold elevation in transcript levels, is responsible for many of the severe developmental anomalies exhibited by the VSR transgenic plants.

### The *arf8* mutation does not suppress the developmental defects incurred by the P6 VSR, but eliminates those caused by *Turnip mosaic virus* infection

**Figure 6:**
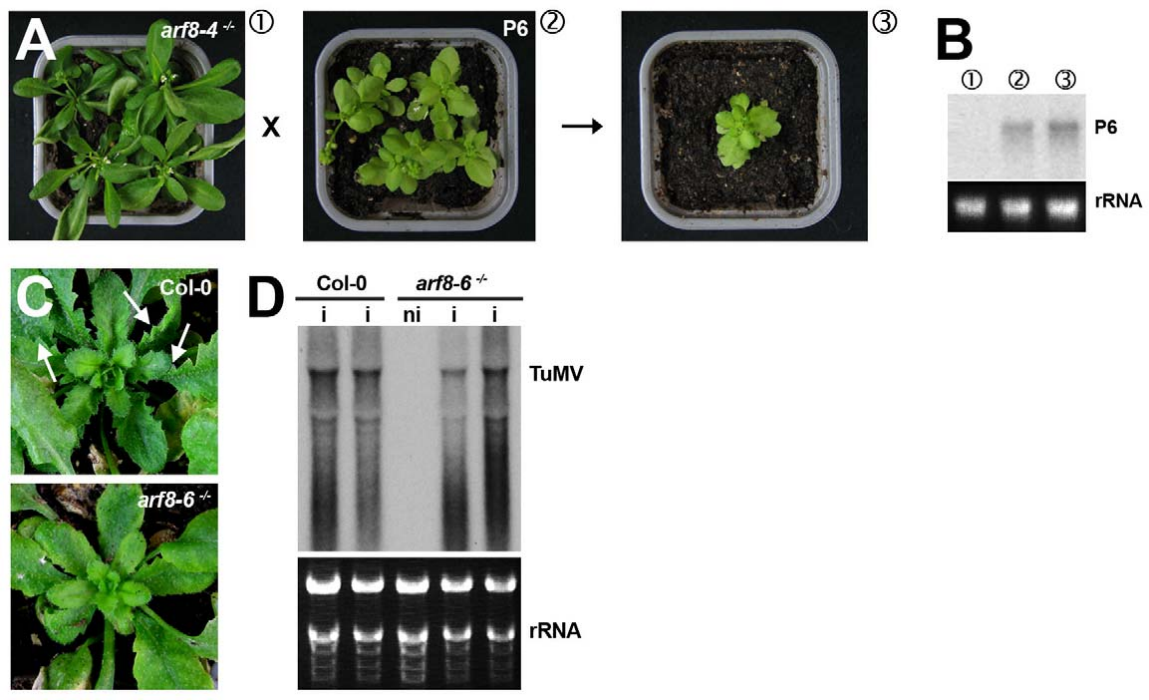
The *arf8* mutation does not alter the developmental phenotypes caused by the P6 VSR of *Cauliflower mosaic virus* but strongly reduces those incurred by*Turnip mosaic virus* infection. (**A–B**) F1 progenies of crosses between *arf8–4* mutant and P6 transgenic plants (ecotype Ler) exhibit the typical dwarfism, chlorosis and pointy leaf phenotype incurred by P6 expression. The Northern analysis in (B) shows comparable accumulation of P6 transcripts in the various backgrounds involved in these crosses. (**C**) The leaf serrations caused by TuMV infection of Col-0 plants (upper panel) are strongly reduced in plants with the *arf8–6* ^-/-^ mutant background. Note the persistence of chlorosis in both cases. (**D**) Comparative Northern analysis of TuMV RNA accumulation in Col-0 versus *arf8–6* ^-/-^ mutant plants. The tracks contain RNA isolated in two independent infections. i: infected; ni: non-infected. rRNA: ethidium bromide staining of ribosomal RNA provides a control for equal RNA loading.

As a further test of the specificity of the *arf8* effects, we used transgenic plants expressing the P6 VSR from *Cauliflower mosaic virus* (CaMV). We previously showed that, unlike HcPro, P15 and P19, the P6 protein does not compromise the miRNA pathway in Arabidopsis but targets the nuclear dsRNA-binding protein DRB4, an accessory factor of DCL4, the main dicer required for RNAi and antiviral defense [21]. Nonetheless, P6 transgenic plants exhibit developmental (i.e. dwarfism, pointy leaves) as well as metabolic (i.e. chlorotic sectors) anomalies that do not overlap with those of HcPro, P19 or P15 plants (Figure 6A). We used an *arf8–4* null mutation in the Ler ecotype and analyzed the phenotype of progenies from crosses to the P6 reference transgenic line, also in the Ler ecotype. We found that expression of P6 was unchanged in the crosses compared to the parental lines, as were the developmental anomalies incurred by P6, suggesting that *arf8* only suppresses those developmental phenotypes that are caused by VSRs targeting miRNA pathway components (Figure 6A–B).

As a final proof of the biological relevance of *ARF8* during compromised miRNA-directed gene regulation, we used *Turnip mosaic virus* (TuMV), which unlike tombusviruses (producing P19) or pecluviruses (producing P15) is known to infect Arabidopsis. TuMV is the potyvirus that naturally encodes the HcPro allele expressed in the VSR transgenic plants employed in the present study. We and others have previously shown that in addition to chlorosis, TuMV infection causes leaf serration and defects in flower architecture highly reminiscent of those found in stable transgenic HcPro plants [18], [19]. Such morphological changes are, in fact, commonly associated to phytovirus infections but their molecular bases have remained poorly understood. Based on the results implicating *ARF8* ectopic expression as a major cause for this phenotype in the VSR lines, we predicted that *arf8–6^-/-^* plants would sustain normal TuMV infection but would fail to display the developmental anomalies normally associated to the disease. The results of several independent infections were consistent with this prediction: while infected *arf8–6^-/-^* plants remained as chlorotic and accumulated as much TuMV RNA as WT plants, leaf serration was hardly discernable (Figure 6C–D). We conclude that ARF8 ectopic accumulation, presumably as a result of HcPro-mediated suppression of miR167 underlies most, if not all, of the developmental symptoms associated to the authentic TuMV infection.

## Discussion

### The surprisingly modest effects of VSRs and *dcl1-9* mutation on small RNA target transcript accumulation

The present analysis indicates that up-regulation of small RNA targets at the post-transcriptional level, incurred in common by VSR expression and/or by the *dcl1–9* mutation, concerns only a discrete subset of transcripts in Arabidopsis, with strikingly modest effects, mostly in the 1.5–2 fold range. This was not only observed for experimentally established (Figure 1A; Figure S4–S6), but also newly identified, putative targets. Although, the selection against high VSR expression and the hypomorphic nature of *dcl1–9* might contribute to these effects, they are unlikely to form their sole basis. Indeed, modest changes in silencing target transcript levels were also noticed in studies of distinct alleles of *dcl1* in various ecotypes, displaying developmental alterations ranging from moderate to severe; the same was observed in comparative analyses of transgenic Arabidopsis expressing other types of VSRs that also impinge on miRNA and siRNA functions [27], [28]. More compellingly, a recent study of miRNA target mimics expressed under the strong 35S promoter also revealed a generally modest effect on miRNA target transcript levels, despite the generation of sometimes dramatic developmental phenotypes [28]. Collectively, these observations highlight an apparent discrepancy between the expected or observed biological outcome of miRNA action on the one hand, and the overall level of variation of target transcripts, on the other, which is in most cases within the range of haplo-sufficiency.

One aspect that could help reconcile, at least in part, this apparent discrepancy is the tissue- or even cell-type specific expression of small RNAs and/or their targets. *In situ*-hybridization and reporter gene fusion analyses indeed show that several, perhaps many, plant miRNAs display exquisitely defined expression patterns [38], [39]. However, the above-mentioned analyses and the present one employed RNA extracted from whole organs, and this may artificially dilute spatially restricted, yet biologically highly significant, effects of some miRNAs on some target transcripts. According to this idea, a much higher spatial, and even temporal resolution might be required in future microarray-based analyses of plant small RNA action [4]. A second, non-mutually exclusive possibility is that plant miRNA- and siRNA-mediated gene regulation entails a much wider translational inhibition component than is usually thought, such that only modest small RNA effects are manifested at the transcript level. Indeed, use of appropriate genetic background indicates that most, if not all, plant miRNAs (and, possibly, siRNAs) regulate their targets through a combination of slicing-based or translation-based inhibitory mechanisms whose respective prevalence is essentially unpredictable based on the position (5′ UTR, CDS, 3′ UTR), pairing degree, or multiplicity of small RNA binding sites [40]. In support of this idea, many Arabidopsis miRNAs are found on polysomes in association with AGO1 [41]. It is, in fact, striking that the amplitude of target mRNA expression changes (1.5–2.5 fold) uncovered in this and other studies of Arabidopsis small RNAs falls within the range of variations typically observed for miRNA-repressed transcripts in metazoans. This modest, yet quantifiable reduction of transcript accumulation by metazoan small RNAs is not accounted for by slicing but, rather, by mRNA decay following deadenylation and decapping, which is coupled to translational repression [42], [43], [44]. In plants, the bulk of target mRNA degradation is commonly ascribed to slicing, typically diagnosed by 5′ RACE analyses [45]. Yet, hardly ever is this technique used quantitatively, so that the real contribution of slicing as opposed to other mechanisms of miRNA-induced transcript turnover (i.e. mRNA decay) is difficult to evaluate. mRNA decay as a consequence of small RNA-directed translational repression is yet to be described in plants, but it certainly deserves careful attention in future investigation of small RNA/target interactions in those organisms.

### Silencing target discovery through analysis of VSR transgenic plants

This study incidentally unraveled that combining comparative microarray analyses of individual VSR transgenic plants and target site predictions from AGO-IP reads is an original approach to the discovery of endogenous transcripts regulated *via* small RNAs at the post-transcriptional level. The approach was notably useful in uncovering somewhat poorly predictable instances of PTGS-based regulations, emphasizing the flexibility and intricate nature of the various RNA silencing pathways in Arabidopsis. For instance, some heterochromatic loci normally associated to the production of 24-nt siRNAs, might be sources of AGO1-loaded *trans*-acting siRNAs, 21–22-nt in length (Figure 2C, Figure S8) while 5′-A- or 5′-G-terminal miRNA passenger strands may exert *cis* or *trans* regulatory effects upon their association with AGO1, which is prominently loaded with 5′-U-terminal small RNAs [30], [46], [47]. Hence, our observation with miR159b* and *SHP1* (Figure 3A) possibly extends the range of transcription factors controlled by the *MIR159b* locus (at least in Arabidopsis), which normally targets MYB-related genes through the mature miR159 species. The prospect of miRNA passenger strands being used for regulatory purposes has not received much attention so far in plants, yet this phenomenon appears to be common in metazoans. In Drosophila, developmentally regulated mechanisms seem to determine the selection/usage of one or the other miRNA strand, and to engage them into distinct regulatory networks, possibly in a cell- or tissue-specific manner [48], [49]. Finally, the AGO1-IP approach applied to single VSR lines could also identify potential *trans*-targets of *IR*-derived siRNAs. In particular, we recently showed that *IR71*-derived siRNA populations can move between distant tissues through the vasculature, presumably to orchestrate gene regulation at a distance both at the transcriptional and post-transcriptional levels [11]. At4g08390, encoding a stromal ascorbate peroxidase, is obviously a strong candidate for this type of regulation; moreover, its presumed function -detoxifying hydrogen peroxide, a molecule involved in defense reactions- is consistent with our recent finding that *IR71* transcription and ensuing siRNA production are strongly induced by viral and bacterial pathogens [11].

Undoubtedly, many additional occurrences will be uncovered through analysis of the non-exhaustive depository found in Figure S7 and Figure S8 such that the approach and its possible refinements (Text S2) will likely complement the tools already available for the discovery or validation of endogenous silencing targets and associated small RNAs in Arabidopsis. Although the method was restricted here to the analysis of sequencing reads from AGO1-IPs [30], it could, in principle, be adapted to small RNAs that are loaded into other types of AGOs with demonstrated or suspected functions in PTGS, and whose action is also likely inhibited by VSRs. These include Arabidopsis AGO10 and AGO5, which belong to the same genetic clade as AGO1, as well as AGO7, which directs cleavage of specific non-coding RNAs to initiate phased TAS3 ta-siRNA production [50]. One advantage of the method is that it does not rely on specific mutations in RNA silencing pathway components (i.e *AGO1* or *DCL1*) but, rather, on the broad-spectrum inhibitory effects of VSRs upon the activity of PTGS-associated small RNAs, independently of their origin and of their AGO effector proteins. This likely explains our finding that introgression of the *arf8–6^-/-^* or *arf8–6^+/−^* mutation into hypomorphic (*ago1–27*) or null (*ago1–36*) mutant alleles of *AGO1* has no detectable effects on the developmental abnormalities exhibited by those plants (data not shown). Presumably, miR167 regulatory functions are, in this case, rescued by the function of an alternative AGO (e.g. AGO10) that is also affected by VSRs. This hypothesis predicts that the developmental defects of mutants in *DCL1*, which fail to accumulate most miRNAs, should, by contrast, be sensitive to *arf8^-/-^*. Indeed, introducing the *arf8* homozygous mutation into the *dcl1–7* hypomorphic allele (ecotype Col-0) was reported to rescue the pleitropic phenotype and viability of this allele, although the molecular bases for this phenomenon was not explained at the time [29].

### Abrogation of silencing mutant developmental phenotypes by single mutations in miRNA target genes

We show, in this study, that the post-embryonic developmental anomalies of VSR plants can be largely ascribed to the misregulation of ARF8, presumably *via* an effect on miR167 activity. In support of this result, *arf8–6* mutant plants expressing ectopically a miR167-resistant allele of *ARF8* (mARF8) are hardly viable, and the few T1 individuals that survive transformation, presumably because of low transgene expression levels, display strong sterility reminiscent of that seen in HcPro, P19 and P15 plants [51, Jason Reed, personal communication]. Moreover, and as explained in the previous section, the *arf8* mutation also attenuates the pleiotropy and fertility defects of *dcl1–7* mutant Arabidopsis [29]. Regulation of ARF8 by miR167 appears, therefore, central to Arabidopsis developmental biology. Recently, a mutation in an ethylene-induced transcription factor, RAV2, was also shown to partially suppress the developmental phenotype of HcPro transgenic Arabidopsis plants [52]. Unlike in *arf8* mutant plants, however, this effect was only evident in homozygous *rav2* mutants, and it was accompanied by a strong inhibition of RNAi suppression by HcPro. While *ARF8* was not part of the set of genes previously found to be up-regulated in *rav2* mutant plants, analyses of available transcriptome data for the Arabidopsis *arf8–3^-/-^ arf6–2^ -/-^* double mutant revealed that *RAV2* expression is induced, rather than repressed, in the tissues analyzed (Table S6). Therefore, the developmental anomalies of HcPro transgenic plants may result from defects in at least two parallel pathways with distinct molecular bases.

Abrogation of the VSR or *dcl1–7* phenotypes by the *arf8* mutation echoes previous findings that most developmental abnormalities of mutant plants deficient for *SERRATE* (a gene involved in maturation of some, albeit not all Arabidopsis miRNAs) can be rescued by mutations in only two targets of miR-165/miR-166, PHABULOSA and PHAVOLUTA, which encode HD-ZIPII transcription factors specifying adaxial cell fates [53]. Thus, the establishment of key developmental programs might require the action of only a small subset of miRNAs and of their targets in Arabidopsis, raising the important issue of the biological significance of additional targets predicted for these and other miRNAs. An in-depth meta-analysis of the transcriptome and protein outputs of over-expressed miRNAs in various mammalian cell cultures similarly raised the question of whether metazoan miRNA-directed regulation of most predicted targets might be biologically neutral [54]. While the neutrality hypothesis certainly deserves attention in plants, an alternative idea holds that many plant miRNAs (and thus their targets) might mainly confer robustness to redundant, miRNA-independent gene repression programs based on transcriptional or epigenetic control, for instance. According to this idea, the function of such miRNAs would only become apparent under at least two conditions. The first condition would entail the prior genetic ablation of the redundant layers of gene expression control [55]. The second circumstance that might reveal functions of plant miRNAs in safeguarding unwanted gene expression is stress. Indeed, most miRNA studies in Arabidopsis have been conducted so far under ideal laboratory growth conditions, where the environmental cues or stimuli that might be required to induce unstable transcriptional patterns are usually nonexistent. Stress application and genetic inactivation of major transcriptional/epigenetic ‘hubs’ in VSR plants, miRNA pathway mutants, or individual *MIRNA* gene knockouts, are thus attractive prospects in future studies of Arabidopsis small RNAs and of their targets.

### VSRs, post-transcriptional gene silencing and viral disease symptoms

One important aspect that had remained unclear from previous studies of antiviral silencing is whether hindrance of the host miRNA pathway is actually a mere consequence of the primary inhibition of antiviral silencing by VSRs or, on the contrary, a deliberate attempt of plant viruses to perturb plant developmental or hormonal pathways to optimize their replication and/or spread. This question is of particular pertinence in the frame of auxin signaling (which is modulated by ARF8), as this hormone has been previously implicated as a negative regulator of basal defense in plants [56]. Moreover, the interaction of the *Tobacco mosaic virus* (TMV) replicase protein, which displays VSR activities, with the PAP1 Aux/IAA protein correlated with viral disease symptoms [57]. The results of TuMV infections in *arf8^-/-^* mutant Arabidopsis, however, show that neither the virus replication nor the chlorotic symptom intensity was altered in those plants, despite a strong reduction of the developmental anomalies normally linked to the infection. These experiments therefore demonstrate in an authentic infection context, that the onset of morphological symptoms often associated with viral diseases, on the one hand, and pathogen virulence as a consequence of antiviral silencing suppression, on the other, can be uncoupled.

Given the high evolutionary conservation of ARF8 and of its riboregulator, miR167, the question thus arises of whether leaf serration and flower defects seen in Arabidopsis are an expected, generic outcome of virus infection in other plant species. It might not be the case for at least three reasons. First, miR167 and its targets may not have a similar weight in shaping organ morphology as they do in Arabidopsis, given the differences in stature and physiology of many plants. Second, the contribution of small RNAs to ARF8 regulation, as opposed to transcriptional control programs (as evoked in the previous section), may vary between species. Third, paralogous proteins not necessarily regulated by small RNAs might ensure redundant ARF8 functions in some species. Supporting these ideas, expression of the same or related VSR alleles as those used in the present study induces developmental phenotypes in tobacco that do not necessarily overlap with those seen in Arabidopsis [58]. A last puzzling observation is that the *arf8* mutation did not suppress the chlorotic symptoms associated with TuMV infection, leaving open the question of whether chlorosis, a widespread yet very poorly understood outcome of virus infection, is indeed related to virulence through VSR function. Accumulation of VSR-deficient viruses, including HcPro-deficient TuMV, can be rescued in Arabidopsis *dcl2-dcl4* double-mutants. Thus, incorporating the *arf8* mutation in this background might provide an interesting ground to study the potential VSR-dependency of chlorosis without the complication of developmental symptoms caused by viruses.

## Materials and Methods

### Plant material and growth conditions

P15, P19 and HcPro expressing lines (in the *CHS* RNAi background) were described previously [18], as were the P6 transgenic line in the WT background [21]. P15, P19 and HcPro lines are moderate expressors and carry the corresponding VSR transgenes in the heterozygous condition, as previously described [18]. The *dcl1–9*, *hen1-1, hen1–6* and *arf8–6* mutants were as described [37], [59], [60]. The *arf8–4* (WISC DsLox 324F09), *arf4–2* (Salk_070506), *arf6* (GABI_859B08) and *arf3–2* (SALK_005658) mutants were as reported in [37]. For microarray analyses all plants were grown *in vitro* in sterile Magenta glass boxes containing 1x MS medium (Duchefa), 1% sucrose and 0.8% agar. Homozygous mutant plants were selected based on their developmental phenotypes and grown at 21–22°C with an 8 h photoperiod (60 µE m^−2^ sec^−1^). All tissues (inflorescences, stems, leaves and roots) were harvested at once at the flowering stage.

### TuMV infections

TuMV sap was extracted from 10 dpi infected turnip leaves (1 g tissue/2 mL 5 mM NaP pH 7.5) and used to inoculate three-week-old *Arabidopsis* rosettes as described previously [18]. TuMV-infected systemic leaves were collected at 14 dpi and subjected to molecular analyses.

### RNA gel blot analyses

Total RNA was extracted from Arabidopsis tissues using Tri-Reagent (Sigma, St. Louis, MO) according to the manufacturer's instructions. Northern analyses of low molecular weight RNA were performed with 30 µg of total RNA, as described previously [18]. DNA oligonucleotides complementary to miRNA sequences were end-labelled with [γ-32P]ATP using T4 PNK (New England Biolabs, Beverly, MA). Northern analyses of high molecular weight RNA were performed with 5–10 µg of total RNA. Probes were DNA fragments labelled by random priming incorporation of [α-32P]dCTP (Amersham). RNA gel blots were subsequently exposed to x-ray films.

### Protein gel blot analyses

For DCL1, protein extraction was performed as previously reported [61]. For AGO1, analyses were carried out using protein crude extracts in Tris-HCl 25M, pH 7.5. In both cases, 100 to 200 µg of proteins were resolved on a 8% SDS-polyacrylamide gel, and subjected to western blotting. Antibodies for AGO1 and DCL1 were described previously in [62].

### Real-time RT-PCR analyses

Total RNA was extracted using the RNeasy Plant Mini kit (Qiagen) according to the manufacturer's instructions. RNA samples were reverse transcribed into cDNA using SuperScript III reverse transcriptase (Invitrogen) after DNase treatment with RQ1 RNase-freeDNase (Promega). The cDNA was quantified using LightCycler 480 SYBR Green I Master mix (ROCHE) and gene-specific primers (see table below). PCR was performed in 384-well optical reaction plates heated at 95°C for 10min, followed by 45 cycles of denaturation at 95°C for 15s, annealing at 60°C for 20s, and elongation at 72°C for 40s. A melting curve was performed at the end of the amplification by steps of 1°C (from 50°C to 95°C). The reference gene set was defined using the NormFinder algorithm (http://www.mdl.dk/publicationsnormfinder.htm). These were Actin2 (At3g18780), At4g34270 and At4g26410 in stems; At4g34270 in leaves; Actin2 and At4g26410 in inflorescences. The sequences of DNA oligonucleotides used for qPCR validations were as shown in Table 1.

**Table 1:** Primer sequences used in this study.

AGI	Name	Sequences		Notes
		LP	RP	
AT3G18780	Actin2	GCACCCTGTTCTTCTTACCG	AACCCTCGTAGATTGGCACA	House keeping gene
AT4G34270	Tip4.1 like	GTGAAAACTGTTGGAGAGAAGCAA	TCAACTGGATACCCTTTCGCA	House keeping gene
AT4G26410	Expressed-10	GAGCTGAAGTGGCTTCAATGAC	GGTCCGACATACCCATGATCC	House keeping gene
AT1G12320		CTCCCTTGAACTTTCCAGAGACTA	ACCACAACAGCTCCTCTGTTTC	*IR5334* putative target
AT4G08390		ATGAAGATCTCCTTGTCCTACCC	TGCAGCATACTTTTCAGCATAGA	*IR71* putative target
AT2G38120		GCTCCACCGGTTCTAACCTA	ATGTCAATAACACTTGAGCCACTT	*TAS3* locus putative target
AT3G58780	SHP1	GAATTCAAATAGGCATATTGTTGG	CAAGACGTCCTTCTAGGTTTTTG	miR159* putative target
AT2G47020		GCTGATGAGAGGGATTGCAT	AGAAGCCTCTTCACCACCAG	miR408* putative target
AT5G13550		CAGCATCCTGACACCTCCAATG	CCGGAGAAGATATCGTCGAA	miR843 putative target
AT4G22470		TGTGGTCGTAGGATTCCACA	GGAAGTGGTGGTGGTGAGAT	Helitron derived siRNA putative *Trans-*target
AT1G67750		GTTGTCCCTGGTATGTCAATTTTT	CAAAGGGAATCCACACATAACTTT	TAS2 derived putative *Trans-*target
AT2G26560		GAAGTAGCTGGTTGGGGACTATT	TATAGTTGGCTTCGGAATGAAGA	PPR derived siRNA putative target
AT3G59210		TTCAGTTGTGTTTGAAGAGGGTAA	CACGCAAGATTAAGCAAAGATAAT	*IR5337* putative target
AT4G28490		TTGGTTCACATAACTTCCACAACT	GTGTTTGCATTGAAAGAGAGAATG	*IR6735* putative target
AT5G37020	ARF8	AGATGTTTGCTATCGAAGGGTTGTTG	CCATGGGTCATCACCAAGGAGAAG	miR167 target. From Vazquez *et al.*, 2004.
AT2G33860	ARF3	CAACACTTGTTCGGATGGTG	CCCACACCAAATGTTCCTCT	TAS3 target. From Hunter *et al.*, 2006.
AT5G60450	ARF4	ATACTACCCCACCCGGAAAC	TGAGACTGCATCGCAAAATC	TAS3 target. From Hunter *et al.*, 2006.
AT2G28350	ARF10	TGGCGAGTCCATGTGTTATC	CAACAAAGACGGAGATGGTG	miR160 target. From Liu *et al.*, 2006.
AT1G77850	ARF17	AGCACCTGATCCAAGTCCTTCTATG	TGGTGAATAGCTGGGGAGGATTTC	miR160 target. From Vazquez et al., 2004.
AT4G03190	GRH1	AAGAAGCTTGAGATACGAGACTGC	ACTTACAAAGCAAGATGACATCCA	miR393 target.
AT1G27370	SPL10	GTGGGAGAATGCTCAGGAGGC	GAGTGTGTTTGATCCCTTGTGAATCC	miR156 target. From Vazquez *et al.*, 2004.
AT5G53950	CUC2	AGCCGTAGCACCAACACAA	GGGACAGTGGAGAAACAGGA	miR164 target
AT1G01040	DCL1	CAGAGTTCGCGATTCTTTTTG	AGGGTTCAACATCAACATCCA	miR162 target
AT1G48410	AGO1	AAGGAGGTCGAGGAGGGTATGG	CAAATTGCTGAGCCAGAACAGTAGG	miR168 target. From Vazquez *et al.*, 2004.
AT5G43810	AGO10	TGCACTCGGTCGGTCTCTAT	TGCTCGAAATGCTGCAAGA	

### Transcriptome studies

Microarray analysis was carried out at the Unité de Recherche en Génomique Végétale (Evry, France), using the CATMA gene arrays containing 24576 gene-specific tags corresponding to 22089 genes from Arabidopsis [63], [64] and a custom-made tiling array covering chromosome 4 at 1 kb resolution [25]. Two independent biological replicates were produced. Total RNA was extracted using trizol according to the supplier's instructions. For each comparison, one technical replication with fluorochrome reversal was performed for each biological replicate (i.e. four hybridizations per comparison). Labelling of cRNAs with Cy3-dUTP or Cy5-dUTP (Perkin-Elmer-NEN Life Science Products), hybridization to the slides, and scanning were performed as described in [65].

### Statistical analysis of transcriptome data

Experiments were designed with the statistics group of the Unité de Recherche en Génomique Végétale. Normalization and statistical analysis was based on two-dye swap method (i.e. four arrays, each containing 24576 GSTs and 384 controls) as described in [66]. To determine differentially expressed genes, a paired t-test was performed on the log ratios, assuming that the variance of the log ratios was the same for all genes. Spots displaying extreme variance (too small or too large) were excluded. The raw p-values were adjusted by the FDR method, which controls the Family Wise Error Rate, (with a type I error equal to 5%) in order to keep a strong control of the false positives in a multiple-comparison context (as described in [67].) We considered as being differentially expressed the genes with a pFDR ≤0.05, as described in [66]. An exhaustive description of the statistical procedures used for microarray analyses can be found in Text S3.

### Chromatin analyses

Chromatin was extracted from leaves of three weeks old plants and chromatin immupoprecipitation was performed using two biological replicates, as described previously [68]. H3K4me2 and H3K9me2 antibodies were purchased from Millipore (Ref. 07-030 and 07-441, respectively). Immunoprecipitated samples were differentially labeled and hybridized onto a custom made tiling array covering Arabidopsis chromosome 4 and the results were analyzed as described previously [25].

### Bioinformatic analyses

AGO1 associated siRNA sequences were downloaded from GEO (www.ncbi.nlm.nih.gov/geo/), accession number GSE10036, and were mapped to *Arabidopsis thaliana* genome (TAIR8 release) with Vmatch (www.vmatch.de). mRNA sequences were calculated from the MIPS *Arabidopsis thaliana* Genome Database (MAtDB), based on TAIR8 release. Each AGO1 associated siRNA was then subjected to BLAST analysis against a given set of mRNA sequences. The results were parsed by a python script, using the Biopython library. A transcript is considered as a putative target when its reverse complement sequence presents (i) ≤three mismatches with an AGO1-IP sRNA and (ii) no more than two mismatches between position one and 12. All the transcripts for one gene were searched for target sites independently. The abundances of all siRNAs matching each target site were then summed for each mRNA.

### Accession numbers

The datasets corresponding to the gene expression profiling experiments in VSR transgenics, *hen1* and *dcl1* mutants of Arabidopsis are accessible at the Gene Expression Omnibus [GEO] under accession number GSE24693.

The datasets corresponding to the Arabidopsis chromosome 4 TILLING array experiments are accessible at the Gene Expression Omnibus [GEO] under accession number GSE26739 for transcript analysis and GSE24692 for chromatin modifications.

Both datasets are also accessible at CATdb (http://urgv.evry.inra.fr/CATdb/; Project: GEN107) according to the “Minimum Information About a Microarray Experiment” standards.

## Supporting Information

Figure S1 VSR expression has little or no incidence on chromatin-level silencing of repeat elements. (A) The few probes (0.5%–2% of total) reporting differential accumulation of transcripts between WT and VSR transgenics or *dcl1-9* and *hen1-1* mutants mainly correspond to genes. (Y axis: relative percentage of differentially hybridizing probes corresponding to transposable elements (TEs), genes and other sequences, respectively; L: leaves; In: inflorescences). (B) Two examples of repeat element sequences with altered H3K9me2 and/or H3K4me2 levels in *hen1-6* mutant plants compared to WT. Chromatin immunoprecipitation results are indicated as log ratio (IP/INPUT) for WT and as log ratio (IP_xx_/IP_WT_) for all of six comparisons performed. Note that HcPro and P19 transgenics show no equivalent changes relative to WT. Annotation is indicated at the top. Small RNA deep sequencing data were obtained from 3 week-old WT leaves (Colot *et al*., unpublished).
(PDF)

Figure S2 CATMA gene expression analysis of the ranges of accumulation changes of all known ta-siRNA and miRNA target transcripts of Arabidopsis in VSR transgenic plants as compared to *dcl1-9* mutant plants. The analysis was carried out in leaves, stems, inflorescences and roots. Note that accumulation changes for the majority of targets are within the 0.8-1.6 fold range.
(PSD)

Figure S3 Proportions of known miRNA and ta-siRNA target transcripts up-regulated in leaves of at least one VSR transgenic background (two upper charts), relative to that found in the *dcl1-9* background alone (third chart), as assessed by CATMA gene expression analysis. Depicted on the right-end panels is the fraction of up-regulated genes (>1.5 fold change) in at least one VSR plant with at least one matching AGO1-IP small RNA read, as assessed by computer-based prediction (see methods).
(PSD)

Figure S4 (**A**) Same as Figure S2, but in inflorescences of transgenic VSR plants and *dcl1-9* mutant plants. (**B**) Ranges in expression changes of known miRNA and ta-siRNA target transcripts in leaves of transgenic VSR plants and *dcl1-9* mutant plants.
(PSD)

Figure S5 (**A**) Same as Figure S2, but in stems of transgenic VSR plants and *dcl1-9* mutant plants. (**B**) Ranges in expression changes of known miRNA and ta-siRNA target transcripts in leaves of transgenic VSR plants and *dcl1-9* mutant plants.
(PSD)

Figure S6 (**A**) Same as Figure S2, but in roots of transgenic VSR plants and *dcl1-9* mutant plants. (**B**) Ranges in expression changes of known miRNA and ta-siRNA target transcripts in leaves of transgenic VSR plants and *dcl1-9* mutant plants.
(PSD)

Figure S7 Ratio between up-regulated transcripts (>1.5 fold change) in VSR transgenic lines versus transcripts with unchanged expression (Y axis) as a function of the number of unique deep-sequencing reads of small RNA, which match all transcripts, isolated from AGO1-IPs (X axis). The logarithmic regression line is presented in black. The blue bar figures the threshold that was used throughout this study. The load number from the unchanged expression transcripts was obtained by calculating the average of 100 independent randomly sampled sets of those mRNA.
(PSD)

Figure S8 Identification of novel putative small RNA target transcripts up-regulated in common in the three VSR transgenic lines (A-C) or mapping to previously predicted stem-loop structures scattered along the Arabidopsis genome (D-E). The presentation principles are the same as those in Figures 3-4. (**A**) A 22nt-long siRNA derived from a unique genomic region annotated as a rolling-circle Helitron transposon, which typically generates heterochromatic siRNAs, 24nt in size. The extensin-like transcript At4g22470, predicted as a target, over-accumulates in stems, leaves and flowers of the three VSR transgenics. (**B**) The pectate-lyase-like transcript At1g67750 (up-regulated in stems of the three VSR transgenics) is predicted as a target for a 20nt-long siRNA derived from the *TAS2* locus. (**C**) A 21nt-long siRNA derived from a population of small RNAs produced from a PPR transcript (At1g63130) that is normally silenced by *TAS3*-derived ta-siRNAs. The siRNA produced from At1g63130 has two identifiable complementary sites within the open-reading frame of its predicted target, the latex allergen-related transcript At2g26560 (up-regulated in inflorescences of the three VSR transgenics). (**D-E**) Stem-loop derived, 21nt siRNAs showing extensive complementarity to its predicted target transcript, At3g59210, up-regulated in inflorescences of the three VSR transgenics. The predicted secondary structure of the stem-loop from which the siRNAs originates is shown in (E).
(PDF)

Figure S9 Suppression of pleotropic defects in VSR plants is *arf8*-specific. (**A)** CATMA gene chip analysis of the accumulation changes of ARF3, ARF4 and ARF8 in leaves of P19, HcPro and P15 transgenic plants, as compared to leaves of WT Arabidopsis. (**B**) Absence of reduction of developmental anomalies in F1 and F2 progenies of crosses between *arf6* mutants and HcPro transgenics carrying the *CHS* RNAi transgene. (**C**) Absence of reduction of developmental anomalies in F1 and F2 progenies of crosses between *arf4–2* mutants and HcPro transgenics carrying the *CHS* RNAi transgene. (**D**) Absence of reduction of developmental anomalies in F1 progenies of crosses between *arf6* (left panel) or *arf4–2* (right panel) mutants and P19 transgenics carrying the *CHS* RNAi transgene. (**E**) qRT-PCR analysis of ARF8 transcript accumulation in the reference line CHS-RNAi, the *arf8-6* mutant and an F1 progeny of a cross between CHS-RNAi and *arf8–6*. Analyses carried out in two independent F1 progenies of HcPro x *arf8–6* and P19 x *arf8–6* plants show the expected reduction in *ARF8* transcript accumulation as compared to the reference HcPro and P19 lines (CHS-RNAi background in both cases). The right column contains qRT-PCR standard deviation values.
(PSD)

Table S1 Quantitative qRT-PCR validation of the variations in gene expression for the targets depicted in Figures 3 and 4, and in Figure S7. Note that most variations only occurred within specific organs. The values in the table are from one analysis; similar results were obtained in a second, independent experiment. SD: standard deviation. Shaded boxes represent non-applicable values.
(XLS)

Table S2 Transcripts exhibiting high complementarity to AGO1-loaded small RNAs and displaying ≥1.5 fold accumulation change compared to WT, in at least one of the three VSR lines, as assessed by CATMA gene expression analysis. Data are presented per organ. Data entries in the table are as follows: ^(1)^ Indicates if the transcript is a known Arabidopsis miRNA target, as annotated in the ASRP depository. ^(2)^ Indicates if the transcript is a known Arabidopsis ta-siRNA target, as annotated in the ASRP depository. ^(3)^ Indicates if the small RNA sequence matches perfectly to an Arabidopsis genomic hairpin, as annotated in the database from [31]. ^(4)^ Indicates if the small RNA sequence matches exactly that of a known Arabidopsis miRNAs, as annotated in the miRBase or ASRP depositories. ^(5)^ The abundance and nomenclature for AGO1-IP reads was kept the same as in [30]. ^(6)^ Number of times that the sequence of the AGO1-IP small RNA can be mapped to the genome. ^(7)^ Complementary sequence of the Arabidopsis target transcript that matches the small RNA. ^(8)^ Percentage of overall nucleotide complementarity between the small RNA and the target transcript. ^(9)^ P-value for the percentage of overall nucleotide complementarity between the small RNA and the target transcript. ^(10)^ Starting position of the small RNA target site within the transcript. ^(11)^ Starting position of the match to target within the small RNA sequence. ^(12)^ Extent of complementarity between the target transcript and the small RNA, in nucleotides. ^(13)^ Length of the small RNA-target complementary region. ^(14)^ Quality score attributed to the small RNA-target complementary region, with a score of 0 representing complete complementarity (see materials and methods for calculation rules). #VALUE entries correspond to non-determined or non-applicable values.
(XLS)

Table S3 List of the 20 transcripts up-regulated by at least 1.5 fold in common in leaves of P19, HcPro and P15 transgenic plants as compared to leaves of WT plants. The genes were identified by CATMA gene expression analysis accumulation of their transcripts further confirmed by qRT-PCR analysis. gene identification and putative function of each targeted transcript are also indicated.
(XLS)

Table S4 List of up-regulated transcripts by at least 1.5 fold in common in stems, roots and inflorescence of P19, HcPro and P15 transgenic plants as compared to leaves of WT plants. Pale grey and dark grey fill colors indicate when transcripts are also up-regulated in *dcl1-9* and *hen1-1*, respectively. The genes were identified by CATMA gene expression analysis and accumulation of their transcripts further confirmed by qRT-PCR analysis.
(XLS)

Table S5 Analysis of the accumulation of the transcripts for major Arabidopsis effectors and endogenous suppressors of PTGS in stems and inflorescences of the Arabidopsis *arf8–3^-/-^ arf-6–2^-/-^* double mutant, as compared to WT plants (Col-0). The data were extracted from the ARRAYEXPRESS website (http://www.ebi.ac.uk/microarray-as/ae/; Experiment ID: E-GEOD-2848).
(XLS)

Table S6 Analysis of the accumulation of the transcripts for the endogenous silencing suppressor gene *RAV2* in stems and inflorescences of the Arabidopsis *arf8–3^-/-^ arf–6–2^-/-^* double mutant. The data were extracted from the ARRAYEXPRESS website (http://www.ebi.ac.uk/microarray-as/ae/; Experiment ID: E-GEOD-2848).
(XLS)

Text S1 Predicted complementary sites between AGO1-IP small RNAs and the Arabidopsis transcripts. The results are presented per organ. The small RNA identification number [30], deep sequencing AGO1-IP read value and number of loci of origin (hit) are indicated.
(PDF)

Text S2 Limits and possible implements to the VSR microarray/AGO1-IP approach to silencing target identification in Arabidopsis.
(DOC)

Text S3 Details on statistical analysis of microarray data.
(DOC)
