# Expression of Concern: Misregulation of *AUXIN RESPONSE FACTOR 8* Underlies the Developmental Abnormalities Caused by Three Distinct Viral Silencing Suppressors in Arabidopsis

**DOI:** 10.1371/journal.ppat.1005234

**Published:** 2015-10-09

**Authors:** The PLOS Pathogens Editors

Concerns have been raised regarding the preparation of Figures 4, 5, and 6 and verification of the genotypes of the three transgenic plants—P15, P19, and HcPro—which were originally described in an article by Dunoyer et al. (2004, *Plant* Cell) that was retracted from the published literature. These concerns were rigorously investigated, the authors have been contacted, and efforts to fully address these concerns are currently underway.

This Expression of Concern should not be considered as a statement regarding the validity of the work, but rather as a notification to readers. *PLOS Pathogens* will provide additional information as it becomes available.
