# Correction: Misregulation of *AUXIN RESPONSE FACTOR 8* Underlies the Developmental Abnormalities Caused by Three Distinct Viral Silencing Suppressors in Arabidopsis

**DOI:** 10.1371/journal.ppat.1005627

**Published:** 2016-05-05

**Authors:** Florence Jay, Yu Wang, Agnès Yu, Ludivine Taconnat, Sandra Pelletier, Vincent Colot, Jean-Pierre Renou, Olivier Voinnet

Errors in Figs 4, 5 and 6 were introduced during preparation for publication. Olivier Voinnet, the corresponding author, assumes full responsibility for these errors. All the erroneous figures have been re-prepared from the original raw material and the seeds of the HcPro, P15 and P19 transgenic lines are available upon reasonable request to the journal.

**Fig 4:**
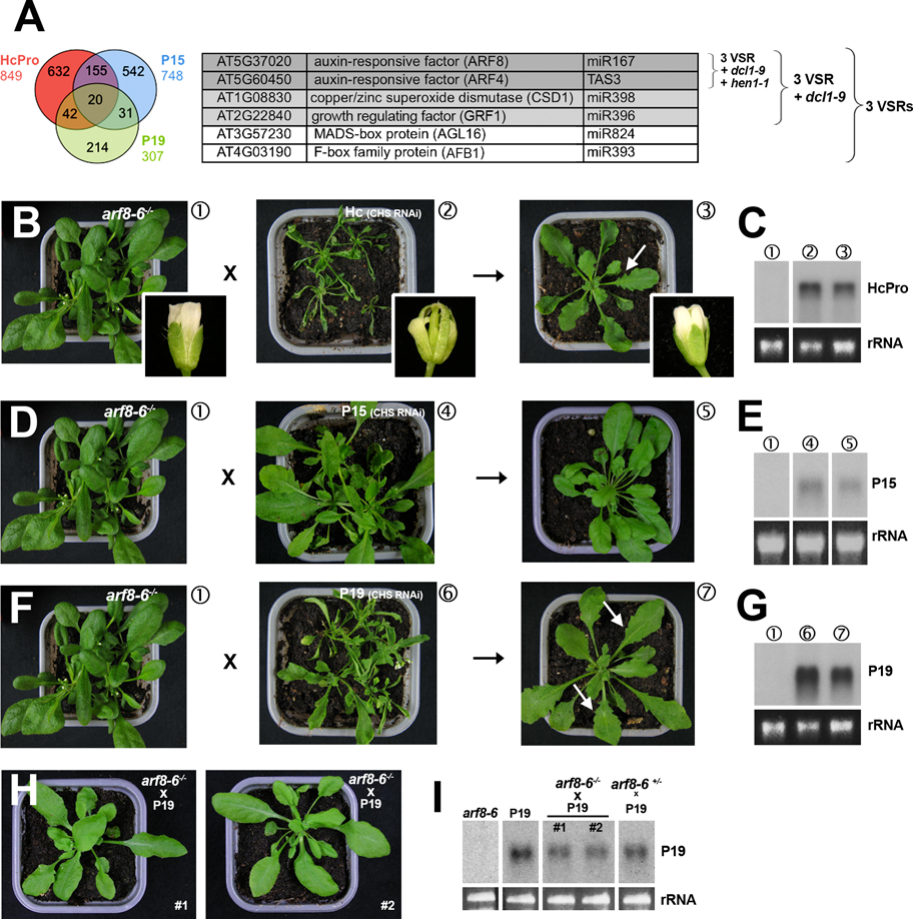
Heterozygous and homozygous *arf8* mutant backgrounds respectively attenuate and alleviate the developmental phenotypes incurred by VSRs. (**A**) The Venn diagram on the left shows that only a modest number of transcripts are up-regulated in common in leaves of the three VSR transgenics. The Table shows that refining the analysis with additional filters based on transcripts up-regulated in *dcl1-9* (pale grey) and *hen1-1* (dark grey) backgrounds singularizes ARF4 and ARF8, respectively direct targets of miR390 and miR167, as strong candidates for the underlying developmental defects seen in VSR transgenics. (**B**, **C**) Strong reduction of leaf and influorescence defects (inlays) caused by HcPro in F1 progenies of crosses between *arf8* mutants and HcPro transgenics carrying the *CHS* RNAi transgene (B). The Northern blot in (C) shows comparable accumulation of HcPro transcripts in the various backgrounds involved in the crosses. (**D**, **E**) same as (B–C) for P15 transgenics in the *CHS* RNAi background. (**F**, **G**) Same as (B, C) for P19 transgenics in the *CHS* RNAi background. Arrows indicate the presence of slight leaf serration in F1 progeny plants. (**H**, **I**) Complete reversion of developmental defects and sterility of P19 transgenic plants (*CHS* RNAi background) in the homozygous *arf8* mutant background. The Northern analysis in (I) confirms comparable P19 levels in the various backgrounds indicated. Plants #1 and #2 where retrieved through independent genotyping in populations of P19 plants with homozygous or heterozygous *arf8* mutant genotype. rRNA: ethidium bromide staining of ribosomal RNA provides a control for equal RNA loading. Note that the rRNA in panel 4I corresponds to a pre-loading control from a preparatory agarose gel.

**Fig 5:**
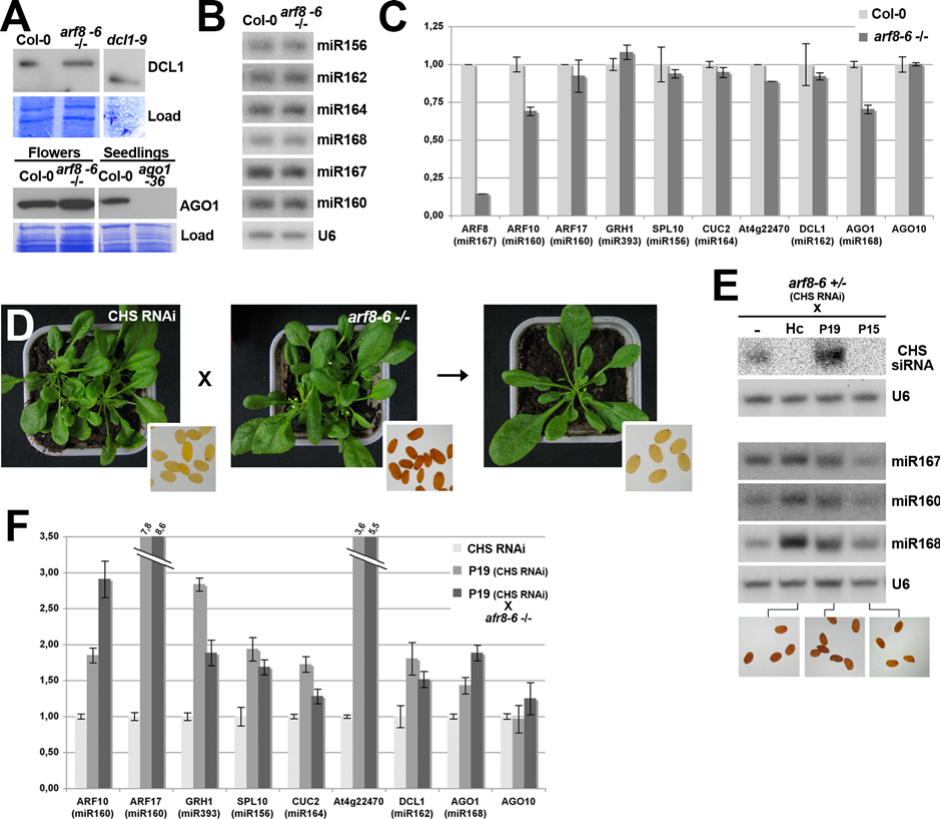
RNAi and miRNA-mediated gene silencing are not compromised by the *arf8* mutation. (**A**) Western analysis of DCL1 accumulation in *arf8* homozygous seedlings compared to WT seedlings and AGO1 accumulation in *arf8* homozygous flowers compared to WT flowers. Due to the strong developmental defects (*dcl1*) or developmental arrest (*ago1*) exhibited by miRNA deficient mutants, the negative controls used in this analysis were from seedlings with the hypomorphic *dcl1–9* genotype, which accumulate a truncated, non-functional form of the DCL1 protein, and from seedlings with the null *ago1–36* genotype. Load: Coomassie staining provides a control for equal loading of total proteins. (**B**) Northern analysis of various endogenous miRNAs in Col-0 or homozygous *arf8* mutant seedlings. (**C**) qRT-PCR analysis of transcript levels from various targets of the miRNAs studied in (B), showing intact miRNA-mediated repression in *arf8* mutants as compared to WT plants. (**D**) RNAi of *CHS*, diagnosed by a loss-of-seed pigmentation (inlays), remains unaltered in plants with the *arf8* ^*-/-*^ genotype. (**E**) Northern analysis of CHS siRNAs and endogenous miRNA accumulation in VSR transgenics with the heterozygous *arf8* mutant background (as depicted in Fig 5B-I). Note the strong decrease in CHS siRNA levels caused by HcPro and P15 as well as the slight shift in electrophoretic mobility and increased accumulation incurred to miRNAs by P19 and HcPro, respectively. The inlays at the bottom show that RNAi of *CHS* remains suppressed by the three VSRs in the *arf8* mutant background, as diagnosed by the dark-brown seed coloration. (**F**) qRT-PCR analysis of transcript levels from various targets of the miRNAs studied in (B) in the P19 transgenics carrying the homozygous *arf8* mutation (*CHS* RNAi background), as depicted in Fig 5H. Reference plants used in the analysis were line *CHS* RNAi and its P19 transgenic derivative (P19 *CHS* RNAi) with a wild type background. Off-scale values for ARF17 and At4g22470 (a novel small RNA target shown in Fig 4A) are indicated by double-dashed lines. U6: oligonucleotide hybridization of the ubiquitous U6 small nucleolar RNA provides a control for equal RNA loading.

**Fig 6:**
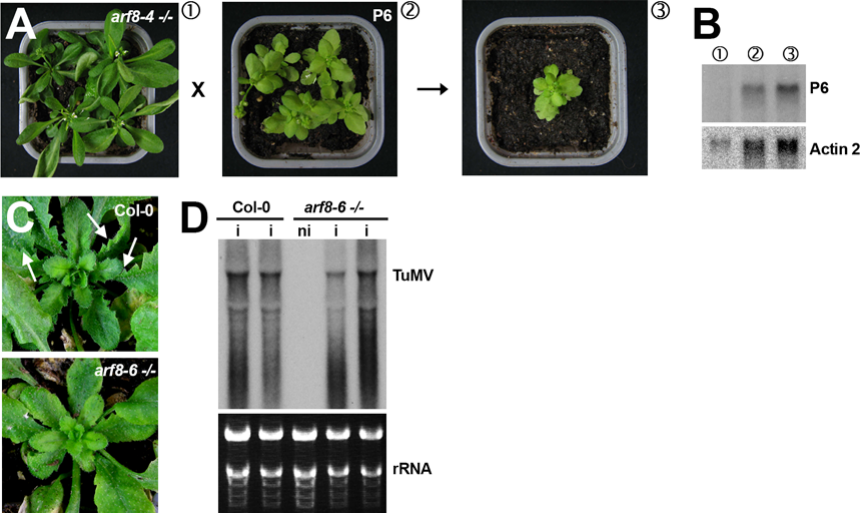
The *arf8* mutation does not alter the developmental phenotypes caused by the P6 VSR of *Cauliflower mosaic virus* but strongly reduces those incurred by *Turnip mosaic virus* infection. (**A**, **B**) F1 progenies of crosses between *arf8–4* ^*-/-*^ mutant and P6 transgenic plants (ecotype Ler) exhibit the typical dwarfism, chlorosis and pointy leaf phenotype caused by P6 expression. The Northern analysis in (B) shows comparable accumulation of P6 transcripts in the various backgrounds involved in these crosses. Actin 2: *ACTIN 2* transcript accumulation provides a control for equal RNA loading. (**C**) The leaf serrations caused by TuMV infection of Col-0 plants (upper panel) are strongly reduced in plants with the *arf8–6* ^*-/-*^ mutant background. Note the persistence of chlorosis in both cases. (**D**) Comparative Northern analysis of TuMV RNA accumulation in Col-0 versus *arf8–6* ^*-/-*^ mutant plants. The tracks contain RNA isolated in two independent infections. i: infected; ni: non-infected. rRNA: ethidium bromide staining of ribosomal RNA provides a control for equal RNA loading.

In Fig 4C, the first track of the blot was incorrectly mounted and used as the control track *arf8-6* ^-/-^. This track has now been replaced by the correct *arf8-6* ^-/-^ track, alongside its corresponding rRNA loading control track, into the corrected Fig 4C. The blot-splicing operation is now indicated with a white space.

In Fig 4E, the original blot was flipped horizontally for mounting this panel, tracks were spliced without notification, and incorrect/un-flipped rRNA tracks were used as loading control. Fig 4E has been now corrected by splicing out the appropriate tracks from the original un-flipped Northern blot, alongside their corresponding rRNA loading control tracks. The blot-splicing operations are now indicated with white spaces.

In Fig 4I, incorrect tracks were mounted and an inappropriate ethidium bromide staining was used. Fig 4I has now been corrected by splicing out the appropriate tracks from the original Northern blot, and by indicating blot-splicing operations with white spaces. Furthermore, since the corresponding original ethidium bromide staining was of poor quality, a pre-loading control from a preparatory agarose gel was used to correct the rRNA loading controls inserted into the corrected Fig 4I.

In Fig 5A, incorrect tracks were mounted and spliced without notification. Fig 5A has now been corrected by splicing out the appropriate tracks from the original Western blots, alongside their corresponding coomassie loading control tracks, and by indicating blot-splicing operations with white spaces. Furthermore, for the AGO1 panel, flowers or seedlings were analyzed, which was not properly indicated. Into the correct Fig 5A, the plant tissues used for these analyses are now clearly specified, both on the panel and in the figure caption, and the “Col-0 seedlings” track has been added, alongside its coomassie loading control track, as was the corresponding control for the *ago1-36* mutant track.

In Fig 5B, Col-0 and *arf8-6* ^-/-^ tracks together with their U6 loading controls were inverted during the mounting process. Fig 5B has now been corrected by flipping all the original panels horizontally.

In Fig 5E, several mounting errors occurred. First, the labels for the miR167 and miR160 hybridizations have been inverted; this inversion is now corrected by assigning the correct label to the corresponding hybridizations, into the corrected Fig 5E. Second, the different hybridizations were presented as if they originated from a single membrane, because the corresponding rRNA loading control for the separate CHS siRNA experiment was omitted. To correct Fig 5E, the original membrane used for the CHS RNAi hybridization was re-probed and the new signal was used, alongside its corresponding U6 loading control, and clearly separated from the other hybridizations.

In Fig 6B, the *arf8-4* ^-/-^ track was stretched towards the left during the mounting process for the panel P6 and the wrong ethidium bromide staining was used as rRNA loading control. To correct Fig 6B, the panel P6 was replaced by a new scan of the original Northern blot and the original membrane was probed for the Actin2 housekeeping mRNA, whose hybridization signal is now used as the loading control into the corrected Fig 6B.

Fig 4G was correct. However, for full transparency, this figure has been re-mounted in order to use the exact same raw material as Fig 4C, since both were spliced out from the same original blot.

The authors confirm that these changes do not alter the results of their study and have provided the relevant underlying data as Supporting Information.

## Supporting Information

S1 Fig Underlying data for Fig 4. (**A**) Original film (left) and ethidium bromide staining (right) used for mounting Fig 4C and Fig 4G. Both figures were mounted from the same blot, hybridized with a mix of random-labeled PCR products corresponding to the 35S terminator and HcPro, allowing simultaneous detection of the P19 and HcPro transcripts (bottom lane), respectively. Blue rectangles indicate the part of the blot used for mounting these figures; samples were loaded according to the track labels; surrounded numbers correspond to the annotated samples on the original Fig 4B and 4F. (**B**) Original film (left) and ethidium bromide staining (right) used for mounting Fig 4E. The blot was hybridized with random-labeled PCR products corresponding to the 35S terminator, allowing detection of the P15 transcripts. Samples were loaded according to the track labels; surrounded numbers correspond to the annotated samples on the original Fig 4D. (**C**) Original scan (left) and inappropriate ethidium bromide staining (right) used for mounting Fig 4I. The blot was hybridized with random-labeled PCR products corresponding to the 35S terminator, allowing detection of the P19 transcripts. Samples were loaded according to the track labels. (**D**) Original film (left) and original ethidium bromide staining (right) corresponding to samples presented in Fig 4I. The blot was hybridized with random-labeled PCR products corresponding to the 35S terminator, allowing detection of the P19 transcripts. Blue rectangles indicate the part of the blot with the samples used in Fig 4I. (**E**) Left panel: original pre-loading control corresponding to the ethidium bromide staining of 1 μg of total RNA loaded on a 1% agarose gel to check quality and equal loading prior loading of the high molecular Northern blot. Right panel: original film of the original membrane used for mounting Fig 4I, re-probed with non-specific random-labeled PCR products that provides an independent loading control. Blue rectangle indicates the part of the blot with the samples used in Fig 4I. Samples used for Fig 4I are labeled on both panels.
(PPTX)

S2 Fig Underlying data for Fig 5. (**A**) Original films and coomassie stainings used for mounting Fig 5A. Left panels: Western analysis of DCL1. Right panels: Western analysis of AGO1. (**B**) Original films used for mounting Fig 5B. Fig 5B was mounted from films obtained by sequentially stripping and re-hybridizing a single Northern blot membrane with several distinct miRNA probes. U6 and miR168 were hybridized at the same time. The long film exposure (2h30) was selected for miR168, and the short exposure (1h) was selected for U6, to avoid a saturated loading signal. Col-0 and *arf8-6* ^-/-^ are respectively on tracks #7 and #6. rRNA, not used for the mounting of Fig 5B, provides an additional loading control. (**C**) Original films used for mounting Fig 5E. Fig 5E was mounted from films obtained by sequentially stripping and re-hybridizing two Northern blot membranes with several distinct miRNA/siRNA probes. U6 and miR168 were hybridized at the same time. The long film exposure (4h30) was selected for miR168, and the short exposure (1h30) was selected for U6, to avoid a saturated loading signal. *arf8-6* ^+/-^ (CHS-RNAi) control (-), crossed with Hc, P19 or P15 are respectively on tracks #2, #3, #4 and #5. Left panels: films of the two membranes re-hybridized with CHS siRNA in June 2015.
(PPTX)

S3 Fig Underlying data for Fig 6. (**A**) Original film (left) used for mounting Fig 6B and original ethidium bromide staining of the corresponding high molecular Northern gel (right). The blot was hybridized with a mix of random-labeled PCR products corresponding to the 35S terminator and HcPro, allowing detection of the P6 transcripts (upper lane). Blue rectangle indicates the part of the blot used for mounting this figure; samples were loaded according to the track labels; surrounded numbers correspond to the annotated samples on the original Fig 6B. (**B**) Crop and uncropped original scans of the unrelated ethidium bromide staining used for mounting Fig 6B. Blue rectangles indicate the three tracks erroneously used for mounting Fig 6B. (**C**) Scan obtained by re-probing the original membrane used in Fig 6B to detect the Actin2 housekeeping mRNA. Samples were loaded according to the track labels; surrounded numbers correspond to the annotated samples on the original Fig 6B. (**D**) Original pre-loading control corresponding to the ethidium bromide stainings of 1 μg of total RNA loaded on a 1% agarose gel to check quality and equal loading prior loading of the high molecular Northern blot used in Fig 6B. (**E**) Original film (left) and ethidium bromide staining (right) used for mounting Fig 6D. The blot was hybridized with random-labeled PCR products corresponding to HcPro. Samples were loaded according to the tracks labels.
(PPTX)

S4 Fig Genotyping of the suppressor lines used in Jay et al., 2011. (**A**) Total gDNA loaded on 1.2% agarose gel. (**B**) Primers used for PCR and sequencing. (**C**) ACTIN2 amplification (PCR of 28 cycles using primers Actin2-For and Actin2-Rev) loaded on 1.2% agarose gel. (**D**) PCR amplification of the *CHS* RNAi transgene (PCR of 30 cycles using primers 35S-For and CHS-Rev) loaded on 1.2% agarose gel. E) P15, P19 and HC-Pro transgene amplification (PCR of 40 cycles using primers 35S-For and T35S-Rev), loaded on 1.2% agarose gel, used for sequencing. Bands extracted for sequencing are indicated for each line. The HC-Pro line presents two bands, one corresponding to the HC-Pro sequence, the second corresponding to the hygromycin resistance gene (HptII), also amplified with the primers used.
(PDF)

S5 Fig Western blot analysis of the suppressor lines used in Jay et al., 2011. P15 was detected from 20 μg of total proteins from seedlings. The P15 antibody was used at a 1/10 000 dilution. P19 was detected from 100 μg of total proteins from seedlings. The P19 antibody was used at a 1/5000 dilution. Hc-Pro was detected from 20 μg of total proteins from seedlings. The Hc-Pro antibody was used at a 1/8000 dilution. The red arrow indicates the position of the P19 signal.
(PDF)

S6 Text Materials and methods used for the analysis of the suppressor lines used in Jay et al., 2011, corresponding to S4 Fig, S5 Fig, S7 File and S8 File. (PDF)

S7 File Raw sequencing data of the genomic DNA of the suppressor lines used in Jay et al., 2011, downloaded from GATC, in the ab1 format. (ZIP)

S8 Fig Contigs of the raw sequences from the S7 File, for P15, P19 and HcPro from the suppressor lines used in Jay et al., 2011, using the CLC Genomics Workbench software. The hygromycin selection gene present in the HcPro transgenic line was also assembled.
(ZIP)

S9 File Detailed steps of the mounting of the corrected panels from Jay et al., 2011. (PDF)
