# Supplementary material for: Misregulation of AUXIN RESPONSE FACTOR 8 Underlies the Developmental Abnormalities Caused by Three Distinct Viral Silencing Suppressors in Arabidopsis
Source: PLoS Pathog. 2011 May 12;7(5):e1002035. doi: 10.1371/journal.ppat.1002035 (PMC3093370; doi:10.1371/journal.ppat.1002035)
Supplement: Figure S1 — VSR expression has little or no incidence on chromatin-level silencing of repeat elements. (A) The few probes (0.5%–2% of total) reporting differential accumulation of transcripts between WT and VSR transgenics or dcl1-9 and hen1-1 mutants mainly correspond to genes. (Y axis: relative percentage of differentially hybridizing probes corresponding to transposable elements (TEs), genes and other sequences, respectively; L: leaves; In: inflorescences). (B) Two examples of repeat element sequences with altered H3K9me2 and/or H3K4me2 levels in hen1-6 mutant plants compared to WT. Chromatin immunoprecipitation results are indicated as log ratio (IP/INPUT) for WT and as log ratio (IPxx/IPWT) for all of six comparisons performed. Note that HcPro and P19 transgenics show no equivalent changes relative to WT. Annotation is indicated at the top. Small RNA deep sequencing data were obtained from 3 week-old WT leaves (Colot et al., unpublished). (PDF) [file ppat.1002035.s001.pdf]

Figure S1. Arabidopsis Chromosome IV Tiling array analysis

One percent or less of the ~21000 probes present on the tiling array reported statistically significant differences in transcript accumulation in leaves or inflorescences between WT plants and transgenic plants expressing VSRs, a fraction slightly lower than between WT and *dcl1-9* or *hen1-1* mutant plants (data deposited on Gene expression omnibus (GEO): accession number GSE26739). In addition, most of these changes in transcript accumulation concerned genes and not repeat elements (A). We therefore conclude that VSRs do not detectably affect silencing of repeat elements across the Arabidopsis genome. Similarly, no difference could be detected reliably in the profiles of histone H3 dimethylated at lysine 9 (H3K9me2, a mark associated with silent repeat elements) or dimethylated at lysine 4 (H3K4me2, a typical euchromatic mark) along the entire chromosome between WT and HcPro- or WT and P19-expressing plants (B; GEO accession number GSE24692). In contrast, a small number of repeat loci exhibited statistically significant gain of H3K4me2 and/or loss of H3K9me2 in *hen1-1* mutant plants (GEO accession number GSE24692), consistent with the role of HEN1 in stabilizing all classes of small RNAs, including 24nt-long siRNA associated with repeat elements (B). Nonetheless, these chromatin changes were not associated with detectable transcript up-regulation of the corresponding repeat elements (data not shown).

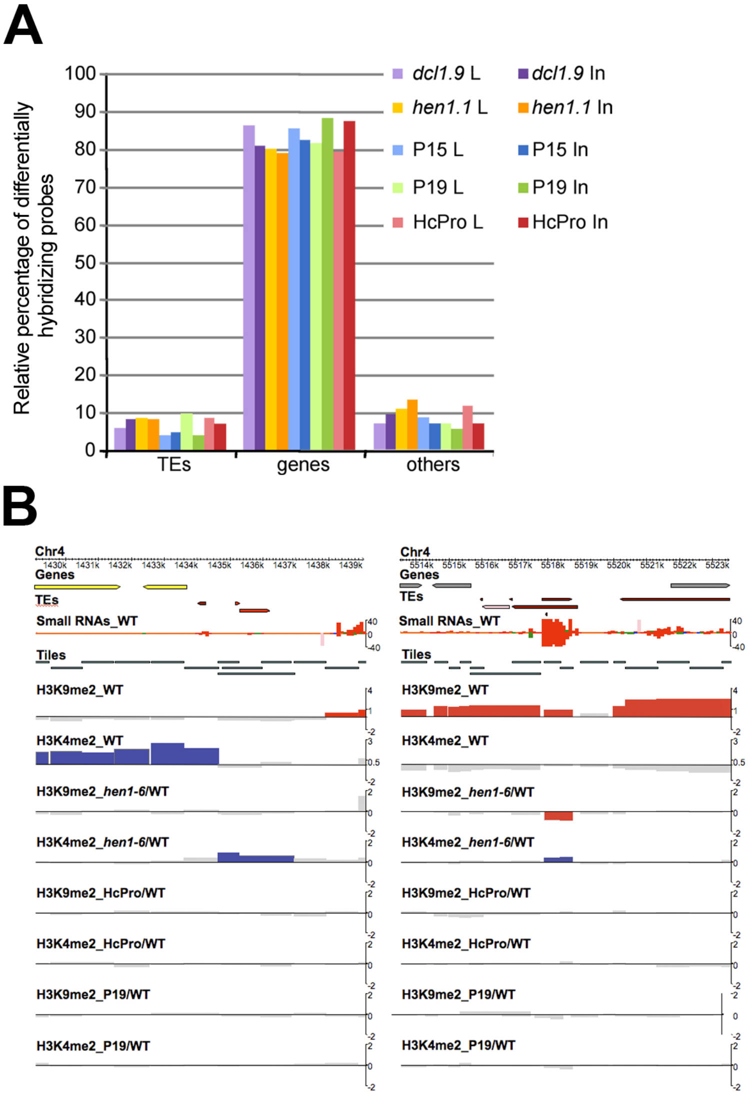
A
Relative percentage of differentially hybridizing probes
100
90
80
70
60
50
40
30
20
10
0
TEs
genes
others
dcl1.9 L
dcl1.9 In
hen1.1 L
hen1.1 In
P15 L
P15 In
P19 L
P19 In
HcPro L
HcPro In
B
Chr4
1430k 1431k 1432k 1433k 1434k 1435k 1436k 1437k 1438k 1439k
5514k 5515k 5516k 5517k 5518k 5519k 5520k 5521k 5522k 5523k
Genes
TEs
Small RNAs_WT
Tiles
H3K9me2_WT
H3K4me2_WT
H3K9me2_hen1-6/WT
H3K4me2_hen1-6/WT
H3K9me2_HcPro/WT
H3K4me2_HcPro/WT
H3K9me2_P19/WT
H3K4me2_P19/WT
