# Supplementary material for: Misregulation of AUXIN RESPONSE FACTOR 8 Underlies the Developmental Abnormalities Caused by Three Distinct Viral Silencing Suppressors in Arabidopsis
Source: PLoS Pathog. 2011 May 12;7(5):e1002035. doi: 10.1371/journal.ppat.1002035 (PMC3093370; doi:10.1371/journal.ppat.1002035)
Supplement: Figure S8 — Identification of novel putative small RNA target transcripts up-regulated in common in the three VSR transgenic lines (A-C) or mapping to previously predicted stem-loop structures scattered along the Arabidopsis genome (D-E). The presentation principles are the same as those in Figures 3-4. (A) A 22nt-long siRNA derived from a unique genomic region annotated as a rolling-circle Helitron transposon, which typically generates heterochromatic siRNAs, 24nt in size. The extensin-like transcript At4g22470, predicted as a target, over-accumulates in stems, leaves and flowers of the three VSR transgenics. (B) The pectate-lyase-like transcript At1g67750 (up-regulated in stems of the three VSR transgenics) is predicted as a target for a 20nt-long siRNA derived from the TAS2 locus. (C) A 21nt-long siRNA derived from a population of small RNAs produced from a PPR transcript (At1g63130) that is normally silenced by TAS3-derived ta-siRNAs. The siRNA produced from At1g63130 has two identifiable complementary sites within the open-reading frame of its predicted target, the latex allergen-related transcript At2g26560 (up-regulated in inflorescences of the three VSR transgenics). (D-E) Stem-loop derived, 21nt siRNAs showing extensive complementarity to its predicted target transcript, At3g59210, up-regulated in inflorescences of the three VSR transgenics. The predicted secondary structure of the stem-loop from which the siRNAs originates is shown in (E). (PDF) [file ppat.1002035.s008.pdf]

Figure S8. Potential trans-targets of siRNAs derived from transposable elements, repeats and TASIRNA-target loci

The AGO1-IP approach was combined to the use of a second, high-stringency filter for genes that are up-regulated (>1.5 fold) in common in the three VSR lines. This allowed identification and qRT-PCR-based validation of At4g22470 (encoding an extensin-like protein) as being a possible target for a 22nt-long siRNA derived from a unique *HELITRON* locus on chromosome 4 (A). This siRNA is clearly nested into a larger population of 24nt siRNAs generally thought to promote heterochromatin formation and TGS of transposable elements in *cis,* via AGO4 or AGO6. This example thus illustrates how detailed analyses might unravel additional *trans*-targeting potential for such heterochromatic loci *via* the production of AGO1-loaded siRNA variants. The same high-stringency filter allowed identification of putative novel targets for *TAS* loci, including *TAS2* (B), and notably unraveled a potential for *trans*-targeting by 21nt-long siRNAs produced from known *TAS3* target transcripts. These include members of the pentatricopeptide-repeat (PPR) protein gene family (e.g. At1g63130), of which some were previously shown to support second waves of RDR6/DCL4-dependent siRNA production following their primary cleavage by discrete ta-siRNA species, as in the example depicted in panel C. In this case, two distinct *PPR*-derived siRNAs show extensive complementary target sites within the ORF of the latex allergen-like protein At2g26560, which they likely contribute to regulate post-transcriptionally as assessed by qRT-PCR. Combining the AGO1-IP approach to a query of a genomic hairpin database was also successful in identifying novel transposable elements-derived IRs as sources of 20-21nt-long siRNAs with a potential to inhibit gene expression in *trans* (D-E).

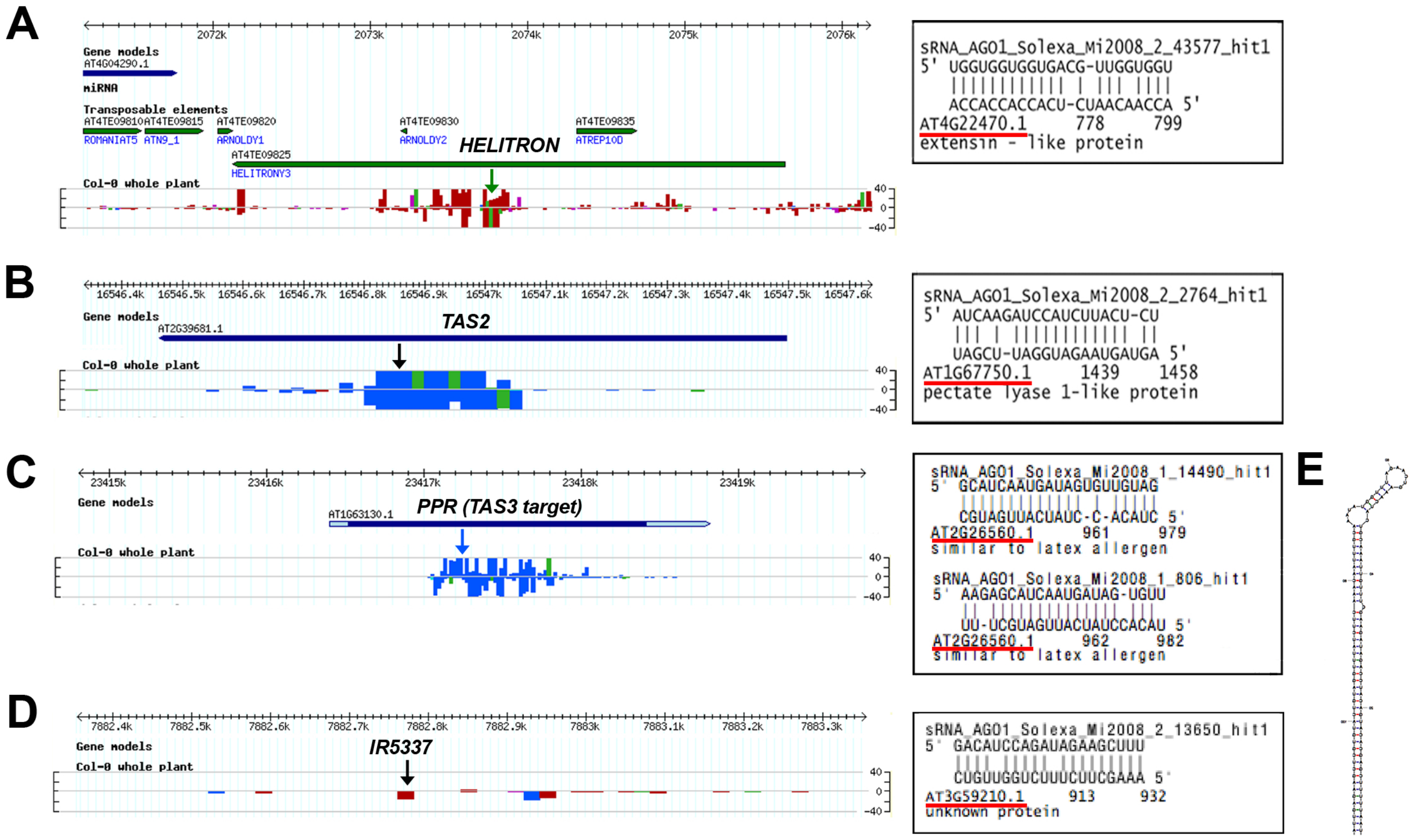

A
2072k
2073k
2074k
2075k
2076k
Gene models
AT4G04290.1
miRNA
Transposable elements
AT4TE09810
AT4TE09815
AT4TE09820
AT4TE09830
AT4TE09835
ROMANIAT5
ATN9_1
ARNOLDY1
ARNOLDY2
ATREP10D
HELITRON
AT4TE09825
HELITRONY3
Col-0 whole plant
40
0
-40
sRNA_AGO1_Solexa_Mi2008_2_43577_hit1
5' UGGUGGUGGUGACG-UUGGUGGU
ACCACCACCACU-CUAACAACCA 5'
AT4G22470.1 778 799
extensin - like protein
B
16546.4k
16546.5k
16546.6k
16546.7k
16546.8k
16546.9k
16547k
16547.1k
16547.2k
16547.3k
16547.4k
16547.5k
16547.6k
Gene models
TAS2
AT2G39681.1
Col-0 whole plant
sRNA_AGO1_Solexa_Mi2008_2_2764_hit1
5' AUCAAGAUCCAUCUUACU-CU
UAGCU-UAGGUAGAAUGAUGA 5'
AT1G67750.1 1439 1458
pectate lyase 1-like protein
C
23415k
23416k
23417k
23418k
23419k
Gene models
PPR (TAS3 target)
AT1G63130.1
Col-0 whole plant
sRNA_AGO1_Solexa_Mi2008_1_14490_hit1
5' GCAUCAAUGAUAGUGUUGUAG
CGUAGUUACUAUC-C-ACAUC 5'
AT2G26560.1 961 979
similar to latex allergen
sRNA_AGO1_Solexa_Mi2008_1_806_hit1
5' AAGAGCAUCAAUGAUAG-UGUU
UU-UCGUAGUUACUAUCCACAU 5'
AT2G26560.1 962 982
similar to latex allergen
E
D
7882.4k
7882.5k
7882.6k
7882.7k
7882.8k
7882.9k
7883k
7883.1k
7883.2k
7883.3k
Gene models
IR5337
Col-0 whole plant
sRNA_AGO1_Solexa_Mi2008_2_13650_hit1
5' GACAUCCAGAUAGAAGCUUU
CUGUUGGUCUUUCUUCGAAA 5'
AT3G59210.1 913 932
unknown protein
