# Supplementary material for: Misregulation of AUXIN RESPONSE FACTOR 8 Underlies the Developmental Abnormalities Caused by Three Distinct Viral Silencing Suppressors in Arabidopsis
Source: PLoS Pathog. 2011 May 12;7(5):e1002035. doi: 10.1371/journal.ppat.1002035 (PMC3093370; doi:10.1371/journal.ppat.1002035)
Supplement: Text S1 — Predicted complementary sites between AGO1-IP small RNAs and the Arabidopsis transcripts. The results are presented per organ. The small RNA identification number [30], deep sequencing AGO1-IP read value and number of loci of origin (hit) are indicated. (PDF) [file ppat.1002035.s016.pdf]

```
sRNA_AGO1_Solexa_Mi2008_1_24183_hit1
5' UAUAAAAGAAACGCACCAUUGUU
   ||||||||| |||| ||| ||||
   AUAUUUUCUGUGCGAGGU-ACAA 5'
AT1G01340.1     2016    2037
CaM-regulated potassium ion channel (ACBK1)

sRNA_AGO1_Solexa_Mi2008_1_7085_hit1
5' CAUCUCUCAGACGCUUUAU
    ||||||||||||  ||||
   AUAGAGAGUCUGC--AAUA 5'
AT1G01480.2     1399    1415
1-aminocyclopropane-1-carboxylate synthase (ACC2)

sRNA_AGO1_Solexa_Mi2008_1_7085_hit1
5' CAUCUCUCAGACGCUUUAU
    ||||||||||||  ||||
   AUAGAGAGUCUGC--AAUA 5'
AT1G01480.1     1584    1600
1-aminocyclopropane-1-carboxylate synthase (ACC2)

sRNA_AGO1_Solexa_Mi2008_1_14075_hit1
5' GAGGCAAAGAUGAAAAGGA
   | || ||||||||||||||
   CGCC-UUUCUACUUUUCCU 5'
AT1G01480.1     191     208
1-aminocyclopropane-1-carboxylate synthase (ACC2)

sRNA_AGO1_Solexa_Mi2008_4_14108_hit2
5' GAGGUAAAGAUGAAAAGGA
   | || ||||||||||||||
   CGCC-UUUCUACUUUUCCU 5'
AT1G01480.1     191     208
1-aminocyclopropane-1-carboxylate synthase (ACC2)

sRNA_AGO1_Solexa_Mi2008_23_6457_hit4
5' CAGG-AGAACAUGAUCGUUU
    ||| |||||||||| ||||
   AUCCGUCUUGUACUAACAAA 5'
AT1G01480.2     293     312
1-aminocyclopropane-1-carboxylate synthase (ACC2)

sRNA_AGO1_Solexa_Mi2008_23_6457_hit4
5' CAGG-AGAACAUGAUCGUUU
    ||| |||||||||| ||||
   AUCCGUCUUGUACUAACAAA 5'
AT1G01480.1     478     497
1-aminocyclopropane-1-carboxylate synthase (ACC2)

sRNA_AGO1_Solexa_Mi2008_1_12622_hit1
5' CUUCGAUUGGGUGAGAU-UG
   |||||||||||||| || ||
   GAAGCUAACCCACU-UACAC 5'
AT1G01590.1     1726    1744
ferric-chelate reductase FRO1

sRNA_AGO1_Solexa_Mi2008_1_45244_hit1
5' UGUGGAAAAGGUGUAUGG-UAG
   |||||||||||||| ||| |||
   ACACCUUUUCCACAAACCGAUC 5'
AT1G01740.1     409     430
protein kinase, putative
```

```
sRNA_AGO1_Solexa_Mi2008_1_30935_hit6
5' UCGAGUGAACGGUGAGGUCG
     ||||||||||||||||:
   CUCUCACUUGCCACUCCAGU 5'
AT1G02190.2     1157    1176
CER1-like protein

sRNA_AGO1_Solexa_Mi2008_2_43441_hit1
5' UGGUGGAGAGCUAUCUGUGU
   |||||||||||  ||||||
   ACCACCUCUCG--AGACACU 5'
AT1G02190.2     510     527
CER1-like protein

sRNA_AGO1_Solexa_Mi2008_1_31046_hit2
5' UCGAGUGGUGGUGAGAAGUGGU
   ||||||||||||||||| ||||
   AGCUCACCACCACUCUU-ACCA 5'
AT1G02190.2     548     568
CER1-like protein

sRNA_AGO1_Solexa_Mi2008_1_52312_hit1
5' UUG-GCAUGCAUCAUAAGGAUA
   ||| |||| ||||||||||||
   AACACGUA-GUAGUAUUCCUAA 5'
AT1G02220.1     1116    1136
hypothetical protein

sRNA_AGO1_Solexa_Mi2008_1_3_hit25
5' AAAA-AAAAAAAAAGAAAGA
    ||| ||||||||||||| |
   CUUUAUUUUUUUUUCUUUGU 5'
AT1G02840.2     1068    1087
ribonucleoprotein SF-2 like protein

sRNA_AGO1_Solexa_Mi2008_3_23994_hit1
5' UAGUGGUGUCAAUGUAACAUG
   |||||||| ||||||||||||
   AUCACCAC-GUUACAUUGUAC 5'
AT1G02850.5     479     498
Similar to beta-glucosidases (At1g02850)

sRNA_AGO1_Solexa_Mi2008_1_54567_hit1
5' UUUAUCUGCAUCAAGAA-A-AGA
   ||||||| ||||||||| | |||
   AAAUAGAAGUAGUUCUUGUCUCU 5'
AT1G03370.1     2844    2866
unknown protein

sRNA_AGO1_Solexa_Mi2008_1_38019_hit2
5' UGCAGAGGAAAGAUGUUGUG
   ||||||||||||  ||||||
   ACGUCUCCUUUCC-CAACAC 5'
AT1G03550.1     1106    1124

sRNA_AGO1_Solexa_Mi2008_7_14062_hit1
5' GAGGAGGAGGAGGUGAACA
   ||||||||||||| |||
   CUCCUCCUCCUCCUCUUUC 5'
AT1G03610.1     352     370
unknown protein

sRNA_AGO1_Solexa_Mi2008_1_29316_hit1
```

```
5' UCCAC-AAGGG-GUUAUGCAAUGAAG
   :|||| ||||| ||||||||||||||
   GGGUGAUUCCCACAAUACGUUACUUC 5'
AT1G03610.1     466     491
unknown protein

sRNA_AGO1_Solexa_Mi2008_58_10471_hit1
5' CUAUCCAUAGUACCAGGCUCA
   |||||||||||| || ||||
   GAUAGGUAUCAUAGU-CGAGA 5'
AT1G03740.2     2574    2593

sRNA_AGO1_Solexa_Mi2008_1_41621_hit1
5' UGGCUGAGCCAACAAUGGUCC
   |||||||||||||||||||||
   ACCGACUCGGUUGUUACCAGG 5'
AT1G03740.1     2660    2680

sRNA_AGO1_Solexa_Mi2008_1_41621_hit1
5' UGGCUGAGCCAACAAUGGUCC
   |||||||||||||||||||||
   ACCGACUCGGUUGUUACCAGG 5'
AT1G03740.2     2742    2762

sRNA_AGO1_Solexa_Mi2008_1_51111_hit1
5' UUGAUGAUGAUGAUGGUUUGA
    ||||||||||||||||||:
   UACUACUACUACUACCAAAUA 5'
AT1G03850.2     158     178
unknown protein

sRNA_AGO1_Solexa_Mi2008_9_14254_hit8
5' GAUGAUGAUGAUGAUGAUCUU
   |||||||||||||||| | ||
   CUACUACUACUACUACCA-AA 5'
AT1G03850.2     161     180
unknown protein

sRNA_AGO1_Solexa_Mi2008_9_14254_hit8
5' GAUGAUGAUGAUGAUGAUCUU
   ||||||||||||||||||  |
   CUACUACUACUACUACUACCA 5'
AT1G03850.2     162     182
unknown protein

sRNA_AGO1_Solexa_Mi2008_6_14255_hit369
5' GAUGAUGAUGAUGAUGAUGAUGAU
   :||||||||||||||||||||| |
   UUACUACUACUACUACUACUACCA 5'
AT1G03850.2     162     185
unknown protein

sRNA_AGO1_Solexa_Mi2008_1_14256_hit8
5' GAUGAUGAUGAUGAUGAUGAUG-UU
    ||||||||||||||||||||| ||
   AUACUACUACUACUACUACUACCAA 5'
AT1G03850.2     162     186
unknown protein

sRNA_AGO1_Solexa_Mi2008_7_3078_hit1
5' AUGAUGAUGA-GAAUGAUGAU
   |||||||||| | |||||| |
   UACUACUACUAC-UACUACCA 5'
```

```
AT1G03850.2     163     182
unknown protein

sRNA_AGO1_Solexa_Mi2008_1_36323_hit13
5' UGAGGAUGAUGAUGAUGAUGA
   ||| ||||||||||||||||
   ACUACUACUACUACUACUACC 5'
AT1G03850.2     163     183
unknown protein

sRNA_AGO1_Solexa_Mi2008_1_3079_hit472
5' AUGAUGAUGAUGAUGAUGAUGA
   |||||||||||||||||||||
   UACUACUACUACUACUACUACC 5'
AT1G03850.2     163     184
unknown protein

sRNA_AGO1_Solexa_Mi2008_1_45039_hit3
5' UGUGAUGAUGAUGAUGAUGAUGA
    :||||||||||||||||||||
   UUACUACUACUACUACUACUACC 5'
AT1G03850.2     163     185
unknown protein

sRNA_AGO1_Solexa_Mi2008_1_56092_hit3
5' UUUG-UGAUGAUGAUGAUGAUGA
   |||| |||||||||||||||||
   AAACUACUACUACUACUACUACC 5'
AT1G03850.2     164     186
unknown protein

sRNA_AGO1_Solexa_Mi2008_6_14255_hit369
5' GAUGAUGAUGAUGAUGAUGAUGAU
   |||:||||||||||||||||||||
   CUAUUACUACUACUACUACUACUA 5'
AT1G03850.2     165     188
unknown protein

sRNA_AGO1_Solexa_Mi2008_1_14256_hit8
5' GAUGAUGAUGAUGAUGAUGAUGUU
   |||:|||||||||||||||||| |
   CUAUUACUACUACUACUACUACUA 5'
AT1G03850.2     165     188
unknown protein

sRNA_AGO1_Solexa_Mi2008_7_3078_hit1
5' AUGAUGAUGA-GAAUGAUGAU
   |||||||||| || |||||||
   UACUACUACUACU-ACUACUA 5'
AT1G03850.2     166     185
unknown protein

sRNA_AGO1_Solexa_Mi2008_1_3079_hit472
5' AUGAUGAUGAUGAUGAUGAUGA
   ||:|||||||||||||||||||
   UAUUACUACUACUACUACUACU 5'
AT1G03850.2     166     187
unknown protein

sRNA_AGO1_Solexa_Mi2008_6_14255_hit369
5' GAUGAUGAUGAUGAUGAUGAUGAU
    |||||:|||||||||||||||||
   GUACUAUUACUACUACUACUACUA 5'
```

```
AT1G03850.2     168     191
unknown protein

sRNA_AGO1_Solexa_Mi2008_1_3079_hit472
5' AUGAUGAUGAUGAUGAUGAUGA
   |||||:||||||||||||||||
   UACUAUUACUACUACUACUACU 5'
AT1G03850.2     169     190
unknown protein

sRNA_AGO1_Solexa_Mi2008_1_45039_hit3
5' UGUGAUGAUGAUGAUGAUGAUGA
   ::||||:||||||||||||||||
   GUACUAUUACUACUACUACUACU 5'
AT1G03850.2     169     191
unknown protein

sRNA_AGO1_Solexa_Mi2008_4_14169_hit1
5' GAUAAUGAUGAUGAAAGAUGA
   ||||||||||||||  |||||
   CUAUUACUACUACUA-CUACU 5'
AT1G03850.2     170     189
unknown protein

sRNA_AGO1_Solexa_Mi2008_2_28918_hit1
5' UCAUCUCAGAACCUACCCGUA
   ||||||||||||||| |||||
   AGUAGAGUCUUGGAUAGGCAU 5'
AT1G03850.1     661     681
unknown protein

sRNA_AGO1_Solexa_Mi2008_1_35367_hit1
5' UGACC-AGAACGCAGAUGCGA
   ||||| ||||||||| |||||
   ACUGGGUCUUGCGUCGACGCU 5'
AT1G03910.1     1181    1201
hypothetical protein

sRNA_AGO1_Solexa_Mi2008_1_7045_hit20
5' CAUCGCCUGCACCUAUGUUGU
    |||||||| ||||||||:|||
   AUAGCGGACAUGGAUACAGCAA 5'
AT1G03910.1     2105    2126
hypothetical protein

sRNA_AGO1_Solexa_Mi2008_2_13419_hit2
5' GAAGAAGAAGAAGACACUU
   |||||||||||||||  :
   CUUCUUCUUCUUCUGUUCG 5'
AT1G04310.1     206     224
putative ethylene receptor ERS2

sRNA_AGO1_Solexa_Mi2008_1_13420_hit2
5' GAAGAAGAAGAAGACUCUU
   |||||||||||||  |||
   CUUCUUCUUCUUC--AGAU 5'
AT1G04310.1     90      106
putative ethylene receptor ERS2

sRNA_AGO1_Solexa_Mi2008_1_13305_hit1
5' CUUUUUGGGCUU-CUGAUACUC
   |||||||||||| || ||||||
   GAAAAACCCGAAUGA-UAUGAG 5'
```

```
AT1G05380.1     1094    1114
unknown protein

sRNA_AGO1_Solexa_Mi2008_1_13305_hit1
5' CUUUUUGGGCUU-CUGAUACUC
   |||||||||||| || ||||||
   GAAAAACCCGAAUGA-UAUGAG 5'
AT1G05380.2     1115    1135
unknown protein

sRNA_AGO1_Solexa_Mi2008_1_38059_hit1
5' UGCAGCAAUCACAAAUGGU
   |||||||||||||| ||:
   ACGUCGUUAGUGUU-ACUU 5'
AT1G05380.1     1944    1961
unknown protein

sRNA_AGO1_Solexa_Mi2008_1_38059_hit1
5' UGCAGCAAUCACAAAUGGU
   |||||||||||||| ||:
   ACGUCGUUAGUGUU-ACUU 5'
AT1G05380.2     1965    1982
unknown protein

sRNA_AGO1_Solexa_Mi2008_1_44558_hit1
5' UGUC-A-UUGAUGGAGAACAAGAU
   |||| | |||||||||| ||||||
   ACAGGUGAACUACCUCUCGUUCUA 5'
AT1G05380.1     2103    2126
unknown protein

sRNA_AGO1_Solexa_Mi2008_1_44558_hit1
5' UGUC-A-UUGAUGGAGAACAAGAU
   |||| | |||||||||| ||||||
   ACAGGUGAACUACCUCUCGUUCUA 5'
AT1G05380.2     2124    2147
unknown protein

sRNA_AGO1_Solexa_Mi2008_1_37628_hit1
5' UGCAAAAACGGAUAAUCU-AGC
   ||||||| |||||||||| ||
   ACGUUUUCGCCUAUUAGAGUCC 5'
AT1G05460.1     1631    1652
unknown protein

sRNA_AGO1_Solexa_Mi2008_1_1123_hit2
5' AAUGGAGAAGCAGAUCGAAGA
   |||||||||||| || ||||
   UUACCUCUUCGU-UA-CUUCG 5'
AT1G05460.1     71      89
unknown protein

sRNA_AGO1_Solexa_Mi2008_2_27439_hit1
5' UCAAUG-CAUGUGGCUGUCAACA
   |||| | |||||:||||||||||
   AGUU-CAGUACAUCGACAGUUGU 5'
AT1G05570.1     2658    2679
putative glucan synthase

sRNA_AGO1_Solexa_Mi2008_7_3892_hit1
5' CAAAACCAGAAGGA-UGAUAAAG
   |||||||||| ||| ||| ||||
   GUUUUGGUCUGCCUAACU-UUUC 5'
```

```
AT1G05570.1     5113    5134
putative glucan synthase

sRNA_AGO1_Solexa_Mi2008_1_3891_hit1
5' CAAAACCAGAAGGA-UGAUAAA
   |||||||||| ||| ||| |||
   GUUUUGGUCUGCCUAACU-UUU 5'
AT1G05570.1     5114    5134
putative glucan synthase

sRNA_AGO1_Solexa_Mi2008_30_44251_hit5
5' UGUAGGUAACAACAUUUGGUC
   |||| ||||||||||||||
   ACAUACAUUGUUGUAAACCCC 5'
AT1G05670.1     2691    2711
UDP glycosyltransferase UGT74E1

sRNA_AGO1_Solexa_Mi2008_1_54576_hit1
5' UUUAUGAGCUCUAAAACGACGUC
   ||| || ||| ||||||||||||
   AAA-ACGCGA-AUUUUGCUGCAG 5'
AT1G05710.3     136     156
unknown protein

sRNA_AGO1_Solexa_Mi2008_1_34211_hit3
5' UGAAAGAGAGAUGAGAGCUUU
   |||||||||||| ||| |||
   ACUUUCUCUCUAGUCU-GAAC 5'
AT1G05710.4     149     168
unknown protein

sRNA_AGO1_Solexa_Mi2008_1_34211_hit3
5' UGAAAGAGAGAUGAGAGCUUU
   |||||||||||| ||| |||
   ACUUUCUCUCUAGUCU-GAAC 5'
AT1G05710.1     260     279
unknown protein

sRNA_AGO1_Solexa_Mi2008_1_34211_hit3
5' UGAAAGAGAGAUGAGAGCUUU
   |||||||||||| ||| |||
   ACUUUCUCUCUAGUCU-GAAC 5'
AT1G05710.3     521     540
unknown protein

sRNA_AGO1_Solexa_Mi2008_2_41651_hit1
5' UGGCUUAACACAACGUAU-GUAG
   ||||||||||||| | || ||||
   ACCGAAUUGUGUUUC-UAGCAUC 5'
AT1G05710.4     536     557
unknown protein

sRNA_AGO1_Solexa_Mi2008_2_41651_hit1
5' UGGCUUAACACAACGUAU-GUAG
   ||||||||||||| | || ||||
   ACCGAAUUGUGUUUC-UAGCAUC 5'
AT1G05710.1     647     668
unknown protein

sRNA_AGO1_Solexa_Mi2008_2_41651_hit1
5' UGGCUUAACACAACGUAU-GUAG
   ||||||||||||| | || ||||
   ACCGAAUUGUGUUUC-UAGCAUC 5'
```

```
AT1G05710.3     908     929
unknown protein

sRNA_AGO1_Solexa_Mi2008_2_41651_hit1
5' UGGCUUAACACAACGUAU-GUAG
   ||||||||||||| | || ||||
   ACCGAAUUGUGUUUC-UAGCAUC 5'
AT1G05710.2     974     995
unknown protein

sRNA_AGO1_Solexa_Mi2008_1_44921_hit1
5' UGUGAAUGAUGCUAUAUUGUG
     |||||| ||||||||||||
   UGACUUACAACGAUAUAACAC 5'
AT1G06150.1     2946    2966
unknown protein

sRNA_AGO1_Solexa_Mi2008_1_34211_hit3
5' UGAAAGAGAGAUGAGA-GCUUU
   |||||||||||||| | :||||
   ACUUUCUCUCUACU-UGUGAAA 5'
AT1G06150.1     42      62
unknown protein

sRNA_AGO1_Solexa_Mi2008_3_39710_hit4
5' UGGAAGAAAUUGUAGUAUU
   ::||| |||||||||||||
   GUCUUGUUUAACAUCAUAA 5'
AT1G06840.1     1188    1206
receptor protein kinase, putative

sRNA_AGO1_Solexa_Mi2008_1_25303_hit1
5' UAUGAGAGAGAAUUGUUGGCAA
   ||||| | |||||||||||||
   AUACU-U-UCUUAACAACCGUA 5'
AT1G06840.1     1457    1476
receptor protein kinase, putative

sRNA_AGO1_Solexa_Mi2008_1_28646_hit2
5' UCAGUAUCGCUGCGGGCUUC-CA
   ||||| |||||||| ||||| ||
   AGUCAAAGCGACGCACGAAGUGU 5'
AT1G07000.1     1454    1476
unknown protein

sRNA_AGO1_Solexa_Mi2008_9_14254_hit8
5' GAUGAUGAUGAUGAUGAUCUU
    ||||||||||||||| |||
   AUACUACUACUACUACGAGAC 5'
AT1G07530.1     1084    1104
transcription factor scarecrow-like 14, putative

sRNA_AGO1_Solexa_Mi2008_1_39866_hit1
5' UGGAAUUGGGCAACAAGAUGAC
   |||| || ||||||||||||||
   ACCU-AAGCCGUUGUUCUACUG 5'
AT1G07530.1     134     154
transcription factor scarecrow-like 14, putative

sRNA_AGO1_Solexa_Mi2008_1_48560_hit1
5' UUC-AAUGAUAAAGCGGUGU
   ||| || |||||||||||||
   AAGCUU-CUAUUUCGCCACA 5'
```

```
AT1G07530.1     1589    1607
transcription factor scarecrow-like 14, putative

sRNA_AGO1_Solexa_Mi2008_1_45985_hit1
5' UGUUGAUGUUGUUGUCGUUAG
   ||||||||||||||| |||
   ACAACUACAACAACAACAACA 5'
AT1G07530.1     403     423
transcription factor scarecrow-like 14, putative

sRNA_AGO1_Solexa_Mi2008_1_52302_hit3
5' UUGGCAGGGUCAUCCUUGGAG
   ||||||||||||||||||||
   AACCGUCCCAGUAGGAACCUC 5'
AT1G07930.1     1072    1092
elongation factor 1-alpha

sRNA_AGO1_Solexa_Mi2008_1_27291_hit3
5' UCAAGGUUGGUGGACCUCUCA
   ||||||||||||||||||||
   AGUUCCAACCACCUGGAGAGU 5'
AT1G07930.2     730     750
elongation factor 1-alpha

sRNA_AGO1_Solexa_Mi2008_1_52302_hit3
5' UUGGCAGGGUCAUCCUUGGAG
   ||||||||||||||||||||
   AACCGUCCCAGUAGGAACCUC 5'
AT1G07940.1     1064    1084
elongation factor 1-alpha

sRNA_AGO1_Solexa_Mi2008_1_52302_hit3
5' UUGGCAGGGUCAUCCUUGGAG
   ||||||||||||||||||||
   AACCGUCCCAGUAGGAACCUC 5'
AT1G07940.2     1191    1211
elongation factor 1-alpha

sRNA_AGO1_Solexa_Mi2008_1_351_hit2
5' AAAGAAGAAAAACAGAUCU
     ||||||||||||| ||
   AGUCUUCUUUUUGUCU-GA 5'
AT1G07940.1     1754    1771
elongation factor 1-alpha

sRNA_AGO1_Solexa_Mi2008_1_351_hit2
5' AAAGAAGAAAAACAGAUCU
     ||||||||||||| ||
   AGUCUUCUUUUUGUCU-GA 5'
AT1G07940.2     1881    1898
elongation factor 1-alpha

sRNA_AGO1_Solexa_Mi2008_1_27291_hit3
5' UCAAGGUUGGUGGACCUCUCA
   ||||||||||||||||||||
   AGUUCCAACCACCUGGAGAGU 5'
AT1G07940.1     722     742
elongation factor 1-alpha

sRNA_AGO1_Solexa_Mi2008_1_27291_hit3
5' UCAAGGUUGGUGGACCUCUCA
   ||||||||||||||||||||
   AGUUCCAACCACCUGGAGAGU 5'
```

```
AT1G07940.2     849     869
elongation factor 1-alpha

sRNA_AGO1_Solexa_Mi2008_1_3_hit25
5' AA-AAAAAAA-AAAAGAAAGA
   || ||||||| ||||||||:|
   UUAUUUUUUUCUUUUCUUUUU 5'
AT1G08050.1     2250    2270
unknown protein

sRNA_AGO1_Solexa_Mi2008_1_172_hit2
5' AAAAGAAGAAGAUA-AAGCA
   |||||||||||| | ||| |
   UUUUCUUCUUCU-UCUUCUU 5'
AT1G08050.1     2       20
unknown protein

sRNA_AGO1_Solexa_Mi2008_2_16280_hit2
5' UAAAAAGAAGAAGAUA-AAGCA
   |||||||||||||| | ||| |
   AUUUUUCUUCUUCU-UCUUCUU 5'
AT1G08050.1     2       22
unknown protein

sRNA_AGO1_Solexa_Mi2008_1_2_hit38
5' AAAAAAAAAAAAAAAC-CAU
   |||||||| ||||||| |||
   UUUUUUUU-UUUUUUGAGUA 5'
AT1G08650.1     1145    1163
putative calcium-dependent protein kinase (U90439)

sRNA_AGO1_Solexa_Mi2008_3_32841_hit1
5' UCUACUGAGGUUGAGUGGG
   :||||||||||||||||
   GGAUGACUCCAACUCACGA 5'
AT1G08700.1     1066    1084
presenilin like protein

sRNA_AGO1_Solexa_Mi2008_1_40235_hit1
5' UGGA-GACGAAGAUGAUGGC-UU
   |||| ||||||||:|||||| ||
   ACCUGCUGCUUCUGCUACCGCAA 5'
AT1G08700.1     331     353
presenilin like protein

sRNA_AGO1_Solexa_Mi2008_1_50433_hit1
5' UUGACUCACCGUGACGCAUUA
   :||||||||||||||||||||
   GACUGAGUGGCACUGCGUAAU 5'
AT1G08940.1     42      62
unknown protein

sRNA_AGO1_Solexa_Mi2008_2_41914_hit2
5' UGGGAUCUUGUGAU-GGAU-UGGU
   |||||||||||||| || | ||||
   ACCCUAGAACACUAGCCCACACCA 5'
AT1G08940.1     93      116
unknown protein

sRNA_AGO1_Solexa_Mi2008_1_21117_hit1
5' UAGACUAAGGCUCCGAAUG-GU
   :|||||||||| ||||||| ||
   GUCUGAUUCCGUGGCUUACACA 5'
```

```
AT1G09560.1     269     290
germin-like protein

sRNA_AGO1_Solexa_Mi2008_1_351_hit2
5' AAAGAAGA-AAAACAGAUCU
   |||||||| |||| ||||||
   UUUCUUCUAUUUU-UCUAGA 5'
AT1G09560.1     771     789
germin-like protein

sRNA_AGO1_Solexa_Mi2008_9_6904_hit1
5' CAUAGGGCUUUCUGCGAUGCA
   || ||||||||||||:|||||
   GU-UCCCGAAAGACGUUACGU 5'
AT1G09570.2     544     563
putative phytochrome A

sRNA_AGO1_Solexa_Mi2008_9_6904_hit1
5' CAUAGGGCUUUCUGCGAUGCA
   || ||||||||||||:|||||
   GU-UCCCGAAAGACGUUACGU 5'
AT1G09570.1     635     654
putative phytochrome A

sRNA_AGO1_Solexa_Mi2008_2_6628_hit1
5' CAGGUGAUGGUGUAAUCAA
   ||| |||||||||||:|||
   GUC-ACUACCACAUUGGUU 5'
AT1G10010.1     383     400
putative amino acid permease

sRNA_AGO1_Solexa_Mi2008_3_54736_hit4
5' UUUCAGUGAACAGAUUG-AGUG
   :||||||||| |||||| ||||
   GAAGUCACUUUUCUAACAUCAC 5'
AT1G10090.1     2163    2184
hypothetical protein

sRNA_AGO1_Solexa_Mi2008_1_26464_hit13
5' UAUUUCAGGAAAUCAGA-UGGCA
   | |||| |||||||||| |||||
   ACAAAGACCUUUAGUCUUACCGU 5'
AT1G10450.1     1510    1532
unknown protein

sRNA_AGO1_Solexa_Mi2008_1_369_hit5
5' AAAGAAUGCCAAUGAUGAGGU
   |||||||||||| |||| ||:
   UUUCUUACGGUUUCUAC-CCG 5'
AT1G10450.1     1854    1873
unknown protein

sRNA_AGO1_Solexa_Mi2008_1_40683_hit1
5' UGGAGUCAGACG-AGCAAGUGA
   :|||||||| || |||||||||
   GCCUCAGUC-GCCUCGUUCACU 5'
AT1G10450.1     851     871
unknown protein

sRNA_AGO1_Solexa_Mi2008_1_13075_hit1
5' CUUGUGAUUUCAGAUAUGCUU
    |||||||||| ||||| |||
   UAACACUAAAGGCUAUA-GAA 5'
```

```
AT1G10450.1     977     996
unknown protein

sRNA_AGO1_Solexa_Mi2008_16_2965_hit6
5' AUGAAG-GCCCAUCACGUG-UUU
    ||||| |||||||||||| |||
   CACUUCUCGGGUAGUGCACCAAA 5'
AT1G11000.1     1097    1119
membrane protein Mlo4

sRNA_AGO1_Solexa_Mi2008_2_35370_hit1
5' UGACC-AGAUCCCAUCUGUGUA
   || || |||||||||||| |||
   AC-GGCUCUAGGGUAGACUCAU 5'
AT1G11000.1     1330    1350
membrane protein Mlo4

sRNA_AGO1_Solexa_Mi2008_2_47511_hit2
5' UUAGCAACCUGGACAUUGAG
   |||||||||||||||||
   AAUCGUUGGACCUGUAAAGG 5'
AT1G12000.1     1296    1315
putative pyrophosphate-fructose-6-phosphate 1-phosphotransferase

sRNA_AGO1_Solexa_Mi2008_1_50661_hit2
5' UUGAGGAGUACUC-AGCACCAA
   ||||||||||||| ||| |||
   AACUCCUCAUGAGGUCGCGGUA 5'
AT1G12000.1     1487    1508
putative pyrophosphate-fructose-6-phosphate 1-phosphotransferase

sRNA_AGO1_Solexa_Mi2008_1_53320_hit2
5' UUGUAGAGAAACAUGAGAGAAA
   ||| | |||||||:||||||||
   AAC-U-UCUUUGUGCUCUCUUU 5'
AT1G12000.1     1960    1979
putative pyrophosphate-fructose-6-phosphate 1-phosphotransferase

sRNA_AGO1_Solexa_Mi2008_1_5136_hit2
5' CAAUGACAUUGAGUGCAAGUG
   |||||||||| ||||| ||||
   GUUACUGUAAGUCACGAUCAC 5'
AT1G12210.1     1054    1074
NBS/LRR disease resistance protein

sRNA_AGO1_Solexa_Mi2008_1_33147_hit1
5' UCUC-C-AAUGACAUUAAGUGCU
   |||| | ||||||||| ||||||
   AGAGUGGUUACUGUAAGUCACGA 5'
AT1G12210.1     1059    1081
NBS/LRR disease resistance protein

sRNA_AGO1_Solexa_Mi2008_1_28067_hit1
5' UCAGAAUCUUCAGGAAACAGAG
   |||:||||||||||||| ||||
   AGUUUUAGAAGUCCUUUAUCUC 5'
AT1G12210.1     1243    1264
NBS/LRR disease resistance protein

sRNA_AGO1_Solexa_Mi2008_17_20513_hit1
5' UACUCCGCCCAUACCAUACAUA
   ||||||:||||||||||||| |
   AUGAGGUGGGUAUGGUAUGUCU 5'
```

```
AT1G12210.1     539     560
NBS/LRR disease resistance protein

sRNA_AGO1_Solexa_Mi2008_1_30136_hit1
5' UCCUACUCCGCCCAUACCAUAC
    ||||||||:|||||||||||
   CGGAUGAGGUGGGUAUGGUAUG 5'
AT1G12210.1     542     563
NBS/LRR disease resistance protein

sRNA_AGO1_Solexa_Mi2008_1_10997_hit1
5' CUCGAGAUAGGUUUUGGAC
   ||| |||||||||||||
   GAG-UCUAUCCAAAACCUA 5'
AT1G12210.1     85      102
NBS/LRR disease resistance protein

sRNA_AGO1_Solexa_Mi2008_55_5422_hit1
5' CACCAAG-CCAAGAAGUCGACU
    |||| | |||||||||||||
   AUGGUGCUGGUUCUUCAGCUGA 5'
AT1G12320.1     417     438
unknown protein (At1g12320)

sRNA_AGO1_Solexa_Mi2008_1_49711_hit26
5' UUCUCAACAAUG-CAUCAUA
   |||||||||||| ||| ||
   AAGAGUUGUUACAGUA-UAG 5'
AT1G12800.1     794     812
unknown protein

sRNA_AGO1_Solexa_Mi2008_1_6621_hit1
5' CA-GG-UGAAGAAGAUAACGU
   || || ||||||||| |||||
   GUACCUACUUCUUCUCUUGCA 5'
AT1G13340.1     296     316
unknown protein

sRNA_AGO1_Solexa_Mi2008_2_116_hit3
5' AAAAAGAUAAGAGA-GAUAG
   |||||||||||||| || ||
   UUUUUCUAUUCUCUUCUCUC 5'
AT1G13390.1     15      34
hypothetical protein

sRNA_AGO1_Solexa_Mi2008_7_1811_hit1
5' AGAAGGGUGAAAGUAAGAGCA
   |||||| |||||| |||||||
   UCUUCC-ACUUUCUUUCUCGU 5'
AT1G13390.2     261     280
hypothetical protein

sRNA_AGO1_Solexa_Mi2008_1_27462_hit1
5' UCAAUGGAGGCAAUGAAUCGGU
   ||||||||||||||||||||||
   AGUUACCUCCGUUACUUAGCCA 5'
AT1G13640.1     104     125
unknown protein

sRNA_AGO1_Solexa_Mi2008_1_29847_hit2
5' UCCGAAUGGUGAUUGCGUUUUGC
   ||||| |||| ||||||||| ||
   AGGCU-ACCAGUAACGCAAA-CG 5'
```

```
AT1G14010.1     181     201
transmembrane like protein

sRNA_AGO1_Solexa_Mi2008_3_4568_hit12
5' CAAGAAGAAG-AUGAGAACAAUG
   |||||||||| | ||||||||||
   GUUCUUCUUCCU-CUCUUGUUAC 5'
AT1G14330.1     222     243
unknown protein

sRNA_AGO1_Solexa_Mi2008_2_6214_hit2
5' CAGAUUGGUCUU-UUAUGUGUU
   |||||||||||| || | ||||
   GUCUAACCAGAACAAGA-ACAA 5'
AT1G14330.1     775     795
unknown protein

sRNA_AGO1_Solexa_Mi2008_1_43234_hit1
5' UGGUCUUUGCAGUUGUCAGUU
   ||||||:||||||| ||||||
   ACCAGAGACGUCAAAAGUCAA 5'
AT1G14380.2     1529    1549
unknown protein

sRNA_AGO1_Solexa_Mi2008_1_43234_hit1
5' UGGUCUUUGCAGUUGUCAGUU
   ||||||:||||||| ||||||
   ACCAGAGACGUCAAAAGUCAA 5'
AT1G14380.3     1663    1683
unknown protein

sRNA_AGO1_Solexa_Mi2008_1_43234_hit1
5' UGGUCUUUGCAGUUGUCAGUU
   ||||||:||||||| ||||||
   ACCAGAGACGUCAAAAGUCAA 5'
AT1G14380.1     1715    1735
unknown protein

sRNA_AGO1_Solexa_Mi2008_2_37991_hit1
5' UGCAGAAGGCUAAAAGAAGUU
   :||||| |||||| |||||||
   GCGUCU-CCGAUUCUCUUCAA 5'
AT1G14380.2     1878    1897
unknown protein

sRNA_AGO1_Solexa_Mi2008_2_37991_hit1
5' UGCAGAAGGCUAAAAGAAGUU
   :||||| |||||| |||||||
   GCGUCU-CCGAUUCUCUUCAA 5'
AT1G14380.3     2012    2031
unknown protein

sRNA_AGO1_Solexa_Mi2008_2_37991_hit1
5' UGCAGAAGGCUAAAAGAAGUU
   :||||| |||||| |||||||
   GCGUCU-CCGAUUCUCUUCAA 5'
AT1G14380.1     2064    2083
unknown protein

sRNA_AGO1_Solexa_Mi2008_3_5252_hit2
5' CACAAAACCGCAGCUACAU-UAG
   || ||||| |||||||||| |||
   GU-UUUUGACGUCGAUGUACAUC 5'
```

```
AT1G14480.1     268     289
hypothetical protein

sRNA_AGO1_Solexa_Mi2008_1_49033_hit1
5' UUCCCA-CAAA-CUUGAUCGCU
   |||| | |||| ||||||||||
   AAGGCUUGUUUUGAACUAGCGA 5'
AT1G14540.1     877     898
peroxidase, putative

sRNA_AGO1_Solexa_Mi2008_1_167_hit1
5' AAAAGA-AAGAGAUGGAGAGAU
    ||||| ||||||||||||||
   CUUUCUGUUCUCUACCUCUCUC 5'
AT1G14710.1     65      86
unknown protein

sRNA_AGO1_Solexa_Mi2008_1_10354_hit2
5' CUAG-UCCCAACUCGGAACCAG
   |||| |||||||||| |||||||
   GAUCGAGGGUUGAGACUUGGUC 5'
AT1G14850.1     2219    2240
nucleoporin, putative

sRNA_AGO1_Solexa_Mi2008_1_8623_hit1
5' CGAGGUGGCUAUGAGCGGUCG
   |||||||||||||||||||||
   GCUCCACCGAUACUCGCCAGC 5'
AT1G14920.1     1745    1765
signal response protein (GAI)

sRNA_AGO1_Solexa_Mi2008_9_14254_hit8
5' GAUGAUGAUGAUGAUGAUCUU
   ||||||||||||||||||||:
   CUACUACUACUACUACUAGAG 5'
AT1G14920.1     195     215
signal response protein (GAI)

sRNA_AGO1_Solexa_Mi2008_6_14255_hit369
5' GAUGAUGAUGAUGAUGAUGAUGAU
   |||||||||||||||||||||  :
   CUACUACUACUACUACUACUAGAG 5'
AT1G14920.1     195     218
signal response protein (GAI)

sRNA_AGO1_Solexa_Mi2008_1_14256_hit8
5' GAUGAUGAUGAUGAUGAUGAUGUU
   ||||||||||||||||||||| |:
   CUACUACUACUACUACUACUAGAG 5'
AT1G14920.1     195     218
signal response protein (GAI)

sRNA_AGO1_Solexa_Mi2008_1_36323_hit13
5' UGAGGAUGAUGAUGAUGAUGA
   ||| |||||||||||||||
   ACUACUACUACUACUACUAGA 5'
AT1G14920.1     196     216
signal response protein (GAI)

sRNA_AGO1_Solexa_Mi2008_1_3079_hit472
5' AUGAUGAUGAUGAUGAUGAUGA
   |||||||||||||||||||
   UACUACUACUACUACUACUAGA 5'
```

```
AT1G14920.1     196     217
signal response protein (GAI)

sRNA_AGO1_Solexa_Mi2008_1_45039_hit3
5' UG-UGAUGAUGAUGAUGAUGAUGA
   || |||||||||||||||||||
   ACUACUACUACUACUACUACUAGA 5'
AT1G14920.1     197     220
signal response protein (GAI)

sRNA_AGO1_Solexa_Mi2008_9_14254_hit8
5' GAUGAUGAUGAUGAUGAUCUU
   ||||||||||||||||||  |
   CUACUACUACUACUACUACUA 5'
AT1G14920.1     198     218
signal response protein (GAI)

sRNA_AGO1_Solexa_Mi2008_6_14255_hit369
5' GAUGAUGAUGAUGAUGAUGAUGAU
     ||||||||||||||||||||||
   GAACUACUACUACUACUACUACUA 5'
AT1G14920.1     198     221
signal response protein (GAI)

sRNA_AGO1_Solexa_Mi2008_7_3078_hit1
5' AUGAUGAUGA-GAAUGAUGAU
   |||||||||| || |||||||
   UACUACUACUACU-ACUACUA 5'
AT1G14920.1     199     218
signal response protein (GAI)

sRNA_AGO1_Solexa_Mi2008_1_36323_hit13
5' UGAGGAUGAUGAUGAUGAUGA
   ||| |||||||||||||||||
   ACUACUACUACUACUACUACU 5'
AT1G14920.1     199     219
signal response protein (GAI)

sRNA_AGO1_Solexa_Mi2008_1_3079_hit472
5' AUGAUGAUGAUGAUGAUGAUGA
    |||||||||||||||||||||
   AACUACUACUACUACUACUACU 5'
AT1G14920.1     199     220
signal response protein (GAI)

sRNA_AGO1_Solexa_Mi2008_1_45039_hit3
5' UGUGAUGAUGAUGAUGAUGAUGA
   : |||||||||||||||||||||
   GAACUACUACUACUACUACUACU 5'
AT1G14920.1     199     221
signal response protein (GAI)

sRNA_AGO1_Solexa_Mi2008_1_56092_hit3
5' UUUG-UGAUGAUGAUGAUGAUGA
    ||| ||||||||||||||||||
   CAACUACUACUACUACUACUACU 5'
AT1G14920.1     200     222
signal response protein (GAI)

sRNA_AGO1_Solexa_Mi2008_7_3078_hit1
5' AUGAUGAUGA-GAAUGAUGAU
   |||||||||| || |||||||
   UACUACUACUACU-ACUACUA 5'
```

```
AT1G14920.1     202     221
signal response protein (GAI)

sRNA_AGO1_Solexa_Mi2008_1_13731_hit1
5' GACGAAGAAGCCGAAUCGA
   |||||||||||||||||||
   CUGCUUCUUCGGCUUAGCU 5'
AT1G14920.1     534     552
signal response protein (GAI)

sRNA_AGO1_Solexa_Mi2008_2_30679_hit3
5' UCGAGACGGGCAA-GCUGACGA
   |||||||||| || ||||||||
   AGCUCUGCCC-UUGCGACUGCU 5'
AT1G15010.1     349     369
unknown protein

sRNA_AGO1_Solexa_Mi2008_4_14108_hit2
5' GAGGUAAA-GAUGAAAAGGA
    |:||||| |||||||||||
   AUUCAUUUUCUACUUUUCCU 5'
AT1G15125.1     124     143
putative protein

sRNA_AGO1_Solexa_Mi2008_4_4868_hit1
5' CAAG-GGAGCUUGGAUGAUGA
   |||| || |||||||||||||
   GUUCUCC-CGAACCUACUACU 5'
AT1G15125.1     194     213
putative protein

sRNA_AGO1_Solexa_Mi2008_4_4953_hit1
5' CAAGGUUGAGGUUCCAAAUAG
    ||| |||||:||||||||||
   AUUC-AACUCUAAGGUUUAUC 5'
AT1G15670.1     1501    1520
unknown protein

sRNA_AGO1_Solexa_Mi2008_3_53982_hit1
5' UUUAAGGU-GGAAAGUUGAGAUUCC
   |||||| | ||||||||||||||||
   AAAUUC-AUCCUUUCAACUCUAAGG 5'
AT1G15670.1     1507    1530
unknown protein

sRNA_AGO1_Solexa_Mi2008_8_54395_hit3
5' UUUAGGGU-GGAAAGUUGAGAUUCC
   ||||:| | ||||||||||||||||
   AAAUUC-AUCCUUUCAACUCUAAGG 5'
AT1G15670.1     1507    1530
unknown protein

sRNA_AGO1_Solexa_Mi2008_1_18369_hit1
5' UAAGGU-GGAAAGUUGAGAUU
   |||| | ||||||||||||||
   AUUC-AUCCUUUCAACUCUAA 5'
AT1G15670.1     1509    1528
unknown protein

sRNA_AGO1_Solexa_Mi2008_4_25271_hit1
5' UAUGAAUGAGAUGAUG-GAUGAA
   |||||||||| ||||| || |||
   AUACUUACUCCACUACUCU-CUU 5'
```

```
AT1G15670.1     77      98
unknown protein

sRNA_AGO1_Solexa_Mi2008_1_25270_hit1
5' UAUGAAUGAGAUGAUG-GAUGA
   |||||||||| ||||| || ||
   AUACUUACUCCACUACUCU-CU 5'
AT1G15670.1     78      98
unknown protein

sRNA_AGO1_Solexa_Mi2008_1_17808_hit1
5' UAAGACCGG-AGCAGCGGAAAG
   :||||| || |||||||||||
   GUUCUG-CCGUCGUCGCCUUUC 5'
AT1G15930.2     34      54
unknown protein

sRNA_AGO1_Solexa_Mi2008_3_36272_hit2
5' UGAGGAGACACCGGUGCCA-AAA
   |||||||||||||||| || |||
   ACUCCUCUGUGGCCAC-GUCUUU 5'
AT1G16010.1     1189    1210
unknown protein

sRNA_AGO1_Solexa_Mi2008_4_36271_hit2
5' UGAGGAGACACCGGUGCCA-AA
   |||||||||||||||| || ||
   ACUCCUCUGUGGCCAC-GUCUU 5'
AT1G16010.1     1190    1210
unknown protein

sRNA_AGO1_Solexa_Mi2008_3_36272_hit2
5' UGAGGAGACACCGGUGCCA-AAA
   |||||||||||||||| || |||
   ACUCCUCUGUGGCCAC-GUCUUU 5'
AT1G16010.2     1278    1299
unknown protein

sRNA_AGO1_Solexa_Mi2008_4_36271_hit2
5' UGAGGAGACACCGGUGCCA-AA
   |||||||||||||||| || ||
   ACUCCUCUGUGGCCAC-GUCUU 5'
AT1G16010.2     1279    1299
unknown protein

sRNA_AGO1_Solexa_Mi2008_3_354_hit1
5' AAAGAAGAAGAUGAUGAGCGU
    |||||||||| |||||| ||
   GUUCUUCUUCUUCUACUC-CA 5'
AT1G17140.2     138     157
unknown protein

sRNA_AGO1_Solexa_Mi2008_3_354_hit1
5' AAAGAAGAAGAUGAUGAGCGU
    |||||||||| |||||| ||
   GUUCUUCUUCUUCUACUC-CA 5'
AT1G17140.1     176     195
unknown protein

sRNA_AGO1_Solexa_Mi2008_3_768_hit1
5' AAGAAGAGAGAGA-AUUGAG
   ||||||||||||| || |||
   UUCUUCUCUCUCUCUAUCUC 5'
```

```
AT1G17140.1     17      36
unknown protein

sRNA_AGO1_Solexa_Mi2008_1_55885_hit1
5' UUUGGGUUGGCUGAUUUUCGAGU
   |||| ||||||||||| | ||||
   AAACGCAACCGACUAACA-CUCA 5'
AT1G17140.1     91      112
unknown protein

sRNA_AGO1_Solexa_Mi2008_2_39987_hit1
5' UGGACCAUGAGGAUGGAGGCU
   ||||||||| ||| |||||||
   ACCUGGUACCCCUCCCUCCGA 5'
AT1G17420.1     1694    1714
lipoxygenase

sRNA_AGO1_Solexa_Mi2008_5_47646_hit1
5' UUAGCUUGGAUUGGUUAGGUUG
   ||| ||||||||||||||:|||
   AAU-GAACCUAACCAAUUCAAG 5'
AT1G17500.1     1032    1052
P-type ATPase, putative

sRNA_AGO1_Solexa_Mi2008_5_39580_hit1
5' UGGAAAGAAAGUGAUCA-AUUUU
   ||||||||| ||||||| ||| |
   ACCUUUCUUCCACUAGUUUAACA 5'
AT1G17500.1     203     225
P-type ATPase, putative

sRNA_AGO1_Solexa_Mi2008_1_29728_hit1
5' UC-CCGACACAUGCGAUUUGCA
   || ||| || |||||||||||
   AGUGGCAGU-UACGCUAAACGU 5'
AT1G17500.1     3179    3199
P-type ATPase, putative

sRNA_AGO1_Solexa_Mi2008_1_34544_hit1
5' UGAAGACGAAGAUGAUGAUGA-UA
   |||||| |||||:|||||||| ||
   ACUUCUUCUUCUGCUACUACUUAU 5'
AT1G17615.1     4       27

sRNA_AGO1_Solexa_Mi2008_1_29151_hit1
5' UCAUUUGUAUAAGAAAGCUAAUU
   || |||||| ||||||||||||
   AG-AAACAU-UUCUUUCGAUUAC 5'
AT1G17615.1     90      110

sRNA_AGO1_Solexa_Mi2008_1_7085_hit1
5' CAUCUCUCAGACGCUUUAU
   |||| ||||||||||||
   GUAG-GAGUCUGCGAAAGC 5'
AT1G18160.1     2764    2781
MAP kinase, putative

sRNA_AGO1_Solexa_Mi2008_2_35255_hit21
5' UGACAGAGAGGUAUGAGCUUA
   ||| ||||||||||| |||||
   ACU-UCUCUCCAUACACGAAU 5'
AT1G18160.1     2924    2943
MAP kinase, putative
```

```
sRNA_AGO1_Solexa_Mi2008_10_30419_hit1
5' UCGAACGCUUCACGGAUCUCUA
   ||||| || ||||||||||||
   AGCUUUCGCAGUGCCUAGAGAC 5'
AT1G18210.2     314     335
unknown protein

sRNA_AGO1_Solexa_Mi2008_1_14930_hit1
5' GGAUUGAAGGGAGCUCUAC
     ||||| |||||||||||
   GGUAACUACCCUCGAGAUG 5'
AT1G18250.2     130     148
putative protein

sRNA_AGO1_Solexa_Mi2008_1_14930_hit1
5' GGAUUGAAGGGAGCUCUAC
     ||||| |||||||||||
   GGUAACUACCCUCGAGAUG 5'
AT1G18250.1     25      43
putative protein

sRNA_AGO1_Solexa_Mi2008_1_40_hit1
5' AAAAAACACAACACAAAAAU
   |||||| | ||||||||||:
   UUUUUU-U-UUGUGUUUUUG 5'
AT1G18570.1     1571    1588
unknown protein

sRNA_AGO1_Solexa_Mi2008_1_40_hit1
5' AAAAAACACAACACAAAAAU
   ||||||||||| | |||||
   UUUUUUGUGUU-U-UUUUUU 5'
AT1G18570.1     264     281
unknown protein

sRNA_AGO1_Solexa_Mi2008_1_35874_hit2
5' UGAGAGACA-AAAUGAGAAGGU
   ||||||||| ||| ||||||||
   ACUCUCUGUGUUU-CUCUUCCA 5'
AT1G18570.1     48      68
unknown protein

sRNA_AGO1_Solexa_Mi2008_4_42709_hit1
5' UGGGUGAUGAUGAUGAUUGAU
    |:||||||||||||||||||
   UCUCACUACUACUACUAACUA 5'
AT1G18740.1     0       20
unknown protein

sRNA_AGO1_Solexa_Mi2008_1_36884_hit32
5' UGAGUUGAUGAGUCAA-UUGGU
   |||||||||||||||| |||||
   ACUCAACUACUCAGUUGAACCA 5'
AT1G18740.1     190     211
unknown protein

sRNA_AGO1_Solexa_Mi2008_1_21856_hit3
5' UAGAUGGGAAUCUCUCUGAUG
   |||||||||||||||||||||
   AUCUACCCUUAGAGAGACUAC 5'
AT1G18740.1     1939    1959
unknown protein
```

```
sRNA_AGO1_Solexa_Mi2008_3_37174_hit2
5' UGAUGACUAAUGUCUAGAUGG
   |||||||||||||||||||||
   ACUACUGAUUACAGAUCUACC 5'
AT1G18740.1     1953    1973
unknown protein

sRNA_AGO1_Solexa_Mi2008_1_21856_hit3
5' UAGAUGGGAAUCUCUCUGAUG
   |||||||||||||||||||||
   AUCUACCCUUAGAGAGACUAC 5'
AT1G18740.1     2102    2122
unknown protein

sRNA_AGO1_Solexa_Mi2008_3_37174_hit2
5' UGAUGACUAAUGUCUAGAUGG
   |||||||||||||||||||||
   ACUACUGAUUACAGAUCUACC 5'
AT1G18740.1     2116    2136
unknown protein

sRNA_AGO1_Solexa_Mi2008_1_37815_hit1
5' UGCAAGUGAUGAUAAAAAGA
   ||||||||||||| ||||||
   ACGUUCACUACUACUUUUCU 5'
AT1G18740.1     2173    2192
unknown protein

sRNA_AGO1_Solexa_Mi2008_1_80_hit1
5' AAAAAAUCAACUCUGAGGCA
   ||||||||||||||||||||
   UUUUUUAGUUGAGACUCCGU 5'
AT1G18740.1     2310    2329
unknown protein

sRNA_AGO1_Solexa_Mi2008_1_33_hit1
5' AAAAAAAUCAACUCUGAGGCA
   |||||||||||||||||||||
   UUUUUUUAGUUGAGACUCCGU 5'
AT1G18740.1     2310    2330
unknown protein

sRNA_AGO1_Solexa_Mi2008_1_13420_hit2
5' GAAGAAGAAGAAGACUCUU
    || |||||||||||| ||
   AUU-UUCUUCUUCUGA-AA 5'
AT1G18740.1     45      61
unknown protein

sRNA_AGO1_Solexa_Mi2008_1_50511_hit7
5' UUGAGAGACAGGGAAGAUGAUGAU
   |||||||| || || |||||||||
   AACUCUCUCUCACUACUACUACUA 5'
AT1G18740.1     4       27
unknown protein

sRNA_AGO1_Solexa_Mi2008_2_50515_hit4
5' UUGAGAGAUAGGGAAGAUGAUGAU
   |||||||| || || |||||||||
   AACUCUCUCUCACUACUACUACUA 5'
AT1G18740.1     4       27
unknown protein
```

```
sRNA_AGO1_Solexa_Mi2008_1_11343_hit1
5' CUGAAAUUACAAAAUACAAAUU
   ||||||:|| |||||||||||
   GACUUUGAUCUUUUAUGUUUAC 5'
AT1G18850.1     1397    1418
unknown protein

sRNA_AGO1_Solexa_Mi2008_4_14108_hit2
5' GAGGUAAAGAUGAAAAGGA
    ||||||||||||||||
   GUCCAUUUCUACUUUUCGA 5'
AT1G18850.1     3       21
unknown protein

sRNA_AGO1_Solexa_Mi2008_1_7584_hit16
5' CCA-AAUGCAGAAACCCAUCUU
   ||| ||| ||||||||||| ||
   GGUAUUAGGUCUUUGGGUA-AA 5'
AT1G18890.1     1474    1494
calcium-dependent protein kinase (ATCDPK1)

sRNA_AGO1_Solexa_Mi2008_3_29603_hit1
5' UCCAUUUGGGUGACACAUCAUC
   |||||| |:|||||||||||||
   AGGUAAUCUCACUGUGUAGUAG 5'
AT1G18890.1     750     771
calcium-dependent protein kinase (ATCDPK1)

sRNA_AGO1_Solexa_Mi2008_1_18115_hit1
5' UAAGGAAGAGAGAUGGUGAGGU
   :||| ||||||||||||||||
   GUUC-UUCUCUCUACCACUCCC 5'
AT1G19025.1     234     254
unknown protein

sRNA_AGO1_Solexa_Mi2008_4_3848_hit1
5' CAAAAAAGAGAGCAGAUUGUG
    ||||||||||||||||||
   AUUUUUUCUCUCGUCUAACCA 5'
AT1G19050.1     65      85
putative protein

sRNA_AGO1_Solexa_Mi2008_1_42552_hit1
5' UGGGGUUGGUUGG-UUGGUUGG
   ||||||||||||| |||||||
   ACCCCAACCAACCAAACCAACG 5'
AT1G19180.1     27      48
unknown protein

sRNA_AGO1_Solexa_Mi2008_1_42552_hit1
5' UGGGGUUGGUUGG-UUGGUUGG
   ||||||||||||| |||||||
   ACCCCAACCAACCAAACCAACG 5'
AT1G19180.2     7       28
unknown protein

sRNA_AGO1_Solexa_Mi2008_1_34362_hit1
5' UGAACAGUUGAUGAUAC-AAU
   ||||||| ||||||||| ||:
   ACUUGUCUACUACUAUGAUUG 5'
AT1G19440.1     397     417
very-long-chain fatty acid condensing enzyme CUT1 like protein
```

```
sRNA_AGO1_Solexa_Mi2008_4_1061_hit1
5' AAUGAACAGUUGAUGAUAC
     ||||||| |||||||||
   CGACUUGUCUACUACUAUG 5'
AT1G19440.1     400     418
very-long-chain fatty acid condensing enzyme CUT1 like protein

sRNA_AGO1_Solexa_Mi2008_1_27730_hit4
5' UCACCCUUCUUCGCCAUGGCC
   ||| ||| |||||||||||||
   AGUUGGA-GAAGCGGUACCGG 5'
AT1G19715.1     1359    1378

sRNA_AGO1_Solexa_Mi2008_1_27730_hit4
5' UCACCCUUCUUCGCCAUGGCC
   ||| ||| |||||||||||||
   AGUUGGA-GAAGCGGUACCGG 5'
AT1G19715.2     1390    1409

sRNA_AGO1_Solexa_Mi2008_2_21996_hit1
5' UAGCAACA-UUGGAUCUUUCUU
   | |||| | |||||||||||||
   AGCGUU-UCAACCUAGAAAGAA 5'
AT1G19715.1     696     716

sRNA_AGO1_Solexa_Mi2008_2_21996_hit1
5' UAGCAACA-UUGGAUCUUUCUU
   | |||| | |||||||||||||
   AGCGUU-UCAACCUAGAAAGAA 5'
AT1G19715.2     727     747

sRNA_AGO1_Solexa_Mi2008_3_2080_hit1
5' AGCGAUGAUGAC-AAUGAUGA
    ||||||||||| || |||||
   ACGCUACUACUGCUU-CUACU 5'
AT1G19770.1     237     256
unknown protein

sRNA_AGO1_Solexa_Mi2008_1_23826_hit1
5' UAGUGAAGAAGAGCUAUGCAUG
   :||||||||||||| ||| ||
   GUCACUUCUUCUCGACACG-AC 5'
AT1G19770.1     582     602
unknown protein

sRNA_AGO1_Solexa_Mi2008_1_13420_hit2
5' GAAGAAGAAGAAGACUCUU
   ||||||||||||| |||:
   CUUCUUCUUCUUCAGAGGC 5'
AT1G20350.1     529     547
unknown protein

sRNA_AGO1_Solexa_Mi2008_17_5534_hit1
5' CACCGUGCGCCGUCGACGC
     |||| ||||||||||||
   UGGGCA-GCGGCAGCUGCG 5'
AT1G20480.1     58      75
unknown protein

sRNA_AGO1_Solexa_Mi2008_1_14334_hit2
5' GAUGGUGAGGGACGACGAU-UU
   |||||||||||| || ||| ||
```

```
   CUACCACUCCCUUCU-CUACAA 5'
AT1G20620.4     155     175
unknown protein

sRNA_AGO1_Solexa_Mi2008_1_14334_hit2
5' GAUGGUGAGGGACGACGAU-UU
   |||||||||||| || ||| ||
   CUACCACUCCCUUCU-CUACAA 5'
AT1G20620.5     157     177
unknown protein

sRNA_AGO1_Solexa_Mi2008_7_6425_hit1
5' CAGGAAGGAAACAAUAGAG
    |||||||||||| | |||
   AUCCUUCCUUUGU-A-CUC 5'
AT1G20620.5     1681    1697
unknown protein

sRNA_AGO1_Solexa_Mi2008_7_6425_hit1
5' CAGGAAGGAAACAAUAGAG
    ||||||||||| || |||
   AUCCUUCCUUUG-UA-CUC 5'
AT1G20620.4     1700    1716
unknown protein

sRNA_AGO1_Solexa_Mi2008_7_6425_hit1
5' CAGGAAGGAAACAAUAGAG
    ||||||||||| || |||
   AUCCUUCCUUUG-UA-CUC 5'
AT1G20620.1     1702    1718
unknown protein

sRNA_AGO1_Solexa_Mi2008_7_6425_hit1
5' CAGGAAGGAAACAAUAGAG
    |||||||||||| | |||
   AUCCUUCCUUUGU-A-CUC 5'
AT1G20620.2     1801    1817
unknown protein

sRNA_AGO1_Solexa_Mi2008_1_8041_hit2
5' CCGGAGACGGAGUG-UGGGC
   |||||||||||||| |||:
   GGCCUCUGCCUCACAACCUA 5'
AT1G20823.1     113     132
ring-H2 finger like protein

sRNA_AGO1_Solexa_Mi2008_9_7942_hit2
5' CCCGGAGACGGAGUG-UGGG
   ||||||||||||||| |||:
   GGGCCUCUGCCUCACAACCU 5'
AT1G20823.1     114     133
ring-H2 finger like protein

sRNA_AGO1_Solexa_Mi2008_5_13314_hit2
5' GAAAAAUGGAAGAUGGCUU
   ||||||||||||| || ||
   CUUUUUACCUUCUUCC-AA 5'
AT1G20823.1     17      34
ring-H2 finger like protein

sRNA_AGO1_Solexa_Mi2008_1_43389_hit1
5' UGGUGAUGGUUGUUGGUGGUC
   |||| |||||||||| ||||
```

```
   ACCA-UACCAACAACAACCAU 5'
AT1G21050.1     241     260
unknown protein

sRNA_AGO1_Solexa_Mi2008_11_43802_hit1
5' UGGU-UGAUGAUGAAGAUGAGGA
   |||| ||| ||||||||:|||||
   ACCAGACUCCUACUUCUGCUCCU 5'
AT1G21050.1     292     314
unknown protein

sRNA_AGO1_Solexa_Mi2008_1_36931_hit2
5' UGAUAACCGUAGA-GCCGAUGG
   ||||||||| ||| ||||||||
   ACUAUUGGC-UCUUCGGCUACC 5'
AT1G21270.1     1076    1096
putative protein

sRNA_AGO1_Solexa_Mi2008_2_35299_hit10
5' UGACAUCAACAUUUAAUGGCC
   |||||||||||||| ||||||
   ACUGUAGUUGUAAACUACCGG 5'
AT1G21270.1     1213    1233
putative protein

sRNA_AGO1_Solexa_Mi2008_3_11843_hit2
5' CUGCUUCGGGUUCCU-UAUG-GG
    |||||||||||||| |||| ||
   AACGAAGCCCAAGGAUAUACUCC 5'
AT1G21450.1     291     313
scarecrow-like 1 (SCL1)

sRNA_AGO1_Solexa_Mi2008_1_45157_hit2
5' UGUGCCAUGGAUUGUGCUCUU
   |||||||| |||||||||| :
   ACACGGUAACUAACACGAGUG 5'
AT1G21460.1     284     304

sRNA_AGO1_Solexa_Mi2008_1_45156_hit2
5' UGUGCCAUGGAUUGUGCUC
   |||||||| ||||||||||
   ACACGGUAACUAACACGAG 5'
AT1G21460.1     286     304

sRNA_AGO1_Solexa_Mi2008_1_36898_hit1
5' UGAGU-UGGAUGAUGAUGAUGA
   :|||| ||||||||||||| ||
   GCUCAGACCUACUACUACU-CU 5'
AT1G21750.2     1041    1061
putative disulfide isomerase

sRNA_AGO1_Solexa_Mi2008_1_36898_hit1
5' UGAGU-UGGAUGAUGAUGAUGA
   :|||| ||||||||||||| ||
   GCUCAGACCUACUACUACU-CU 5'
AT1G21750.1     1042    1062
putative disulfide isomerase

sRNA_AGO1_Solexa_Mi2008_3_13421_hit1
5' GAAGAAGAAGAUGAUGUUGAU
   ||||||||||| |||| ||||
   CUUCUUCUUCUUCUACCACUA 5'
AT1G21910.1     181     201
```

```
TINY like protein

sRNA_AGO1_Solexa_Mi2008_6_14255_hit369
5' GAUGAUGAUGAUGAUGAUGAUGAU
   |||||||||||||| ||:|| |||
   CUACUACUACUACUUCUGCUUCUA 5'
AT1G22850.1     15      38
unknown protein

sRNA_AGO1_Solexa_Mi2008_1_37225_hit4
5' UGAUGAUGAUGAUGAUGAAGAAG
    |||||||||||||| || ||||
   UCUACUACUACUACUUCUGCUUC 5'
AT1G22850.1     17      39
unknown protein

sRNA_AGO1_Solexa_Mi2008_1_7931_hit2
5' CCCGAUGAUGAUGAUG-AGAC
   || ||||||||||||| ||||
   GGACUACUACUACUACUUCUG 5'
AT1G22850.1     22      42
unknown protein

sRNA_AGO1_Solexa_Mi2008_1_13759_hit1
5' GACGAUGAUGAUGAUGACA
   || ||||||||||||||
   CU-CUACUACUACUACUUC 5'
AT1G22850.1     24      41
unknown protein

sRNA_AGO1_Solexa_Mi2008_5_13760_hit2
5' GACGAUGAUGAUGAUGAGC
   || ||||||||||||||:
   CU-CUACUACUACUACUUC 5'
AT1G22850.1     24      41
unknown protein

sRNA_AGO1_Solexa_Mi2008_1_2280_hit1
5' AGUGGAUGAUGAUGAUGAUG
     |||| |||||||||||||
   AAACCU-CUACUACUACUAC 5'
AT1G22850.1     27      45
unknown protein

sRNA_AGO1_Solexa_Mi2008_1_56259_hit1
5' UUUUAAGGCACUUUUCAGGGAUG
   |||||||||  |||||||||||
   AAAAUUCCG--AAAAGUCCCUAG 5'
AT1G23280.1     1009    1029
mak16-like protein-related

sRNA_AGO1_Solexa_Mi2008_12_5493_hit2
5' CACCCAUUUGAUACAUAGU
   |||||||||||| |||| |
   GUGGGUAAACUA-GUAUAA 5'
AT1G23850.1     1142    1159
unknown protein

sRNA_AGO1_Solexa_Mi2008_1_55587_hit1
5' UUUGGAGUGAAAGGU-GAAGAU
   ||||||||||||| | |||||
   AAACCUCACUUUCGAGCUUCUU 5'
AT1G23850.1     317     338
```

```
unknown protein

sRNA_AGO1_Solexa_Mi2008_1_7688_hit1
5' CCACCACCACCUCCAGCG-CCAC
   |||||||||||||| || || |
   GGUGGUGGUGGAGGU-GCUGGAG 5'
AT1G23860.2     298     319
9G8-like splicing factor / SRZ-21

sRNA_AGO1_Solexa_Mi2008_1_7688_hit1
5' CCACCACCACCUCCAGCG-CCAC
   |||||||||||||| || || |
   GGUGGUGGUGGAGGU-GCUGGAG 5'
AT1G23860.1     300     321
9G8-like splicing factor / SRZ-21

sRNA_AGO1_Solexa_Mi2008_1_7688_hit1
5' CCACCACCACCUCCAGCG-CCAC
   |||||||||||||| || || |
   GGUGGUGGUGGAGGU-GCUGGAG 5'
AT1G23860.3     303     324
9G8-like splicing factor / SRZ-21

sRNA_AGO1_Solexa_Mi2008_1_35588_hit10
5' UGACGGUUACCAGGCUAUUAC
   ||| ||||||||||| ||||
   ACUUCCAAUGGUCCG-UAAUA 5'
AT1G24090.1     1162    1181
unknown protein

sRNA_AGO1_Solexa_Mi2008_1_3079_hit472
5' AUGAUGAUGAUGAUGAUGAUGA
   |||||||| |||||| |||||
   UACUACUAAUACUACAACUACC 5'
AT1G24575.1     89      110
unknown protein

sRNA_AGO1_Solexa_Mi2008_1_3079_hit472
5' AUGAUGAUGAUGAUGAUGAUGA
    |||||||||| |||||| |||
   CACUACUACUAAUACUACAACU 5'
AT1G24575.1     92      113
unknown protein

sRNA_AGO1_Solexa_Mi2008_1_45039_hit3
5' UGUGAUGAUGAUGAUGAUGAUGA
    ||||||||||| |||||| |||
   CCACUACUACUAAUACUACAACU 5'
AT1G24575.1     92      114
unknown protein

sRNA_AGO1_Solexa_Mi2008_2_36760_hit1
5' UGA-GUCCAUGUGUCGUUCCGGU
   ||| |||||||| ||||||||||
   ACUACAGGUACAGAGCAAGGCCA 5'
AT1G25440.1     1130    1152
unknown protein

sRNA_AGO1_Solexa_Mi2008_1_36759_hit1
5' UGA-GUCCAUGUGUCGUUCCGG
   ||| |||||||| |||||||||
   ACUACAGGUACAGAGCAAGGCC 5'
AT1G25440.1     1131    1152
```

```
unknown protein

sRNA_AGO1_Solexa_Mi2008_1_36838_hit1
5' UGAGUGGUUGCUAUGCUUUAC
   |||||||||||||||||||||
   ACUCACCAACGAUACGAAAUG 5'
AT1G25440.1     374     394
unknown protein

sRNA_AGO1_Solexa_Mi2008_714_35241_hit6
5' UGACAGAAGAGAGUGAG-CACA
   ||| ||||||||||||| || |
   ACUCUCUUCUCUCACUCCGUUU 5'
AT1G25500.3     2       23
unknown protein

sRNA_AGO1_Solexa_Mi2008_2091_35240_hit6
5' UGACAGAAGAGAGUGAG-CAC
   ||| ||||||||||||| ||
   ACUCUCUUCUCUCACUCCGUU 5'
AT1G25500.3     3       23
unknown protein

sRNA_AGO1_Solexa_Mi2008_1778_50260_hit1
5' UUGACAGAAGAGAGUGAG-CAC
   |||| ||||||||||||| ||
   AACUCUCUUCUCUCACUCCGUU 5'
AT1G25500.3     3       24
unknown protein

sRNA_AGO1_Solexa_Mi2008_30_35239_hit6
5' UGACAGAAGAGAGUGAG-CA
   ||| ||||||||||||| ||
   ACUCUCUUCUCUCACUCCGU 5'
AT1G25500.3     4       23
unknown protein

sRNA_AGO1_Solexa_Mi2008_1_11426_hit3
5' CUGACAGAAGAGAGUGAG-CA
    ||| ||||||||||||| ||
   AACUCUCUUCUCUCACUCCGU 5'
AT1G25500.3     4       24
unknown protein

sRNA_AGO1_Solexa_Mi2008_16_50259_hit1
5' UUGACAGAAGAGAGUGAG-CA
   |||| ||||||||||||| ||
   AACUCUCUUCUCUCACUCCGU 5'
AT1G25500.3     4       24
unknown protein

sRNA_AGO1_Solexa_Mi2008_1_50258_hit1
5' UUGACAGAAGAGAGUGAGC
    ||| |||||||||||||
   UACUCUCUUCUCUCACUCC 5'
AT1G25500.3     5       23
unknown protein

sRNA_AGO1_Solexa_Mi2008_1_50864_hit1
5' UUGAGGUUGCCUCUGUAA-CAA
   ||||||||| |||||||| |||
   AACUCCAACAGAGACAUUAGUU 5'
AT1G26560.1     1589    1610
```

```
beta-glucosidase like protein

sRNA_AGO1_Solexa_Mi2008_1_32987_hit1
5' UCUAUCAAAAGGUGGG-U-UGGU
   | |||||||||||||| | ||||
   AAAUAGUUUUCCACCCUAGACCA 5'
AT1G27980.1     1058    1080
unknown protein

sRNA_AGO1_Solexa_Mi2008_1_50609_hit1
5' UUGAGCGAGAAACAGAGAGAA
   :||||||||||||||||||
   GACUCGCUCUUUGUCUCUCUG 5'
AT1G27980.1     186     206
unknown protein

sRNA_AGO1_Solexa_Mi2008_1_40835_hit1
5' UGGAUCAUCUCCUCGUGCA
     |||||||||||| ||||
   UACUAGUAGAGGAGAACGU 5'
AT1G27990.1     416     434
hypothetical protein

sRNA_AGO1_Solexa_Mi2008_1_34261_hit1
5' UGA-A-AGUGGAAGAUAAAUA
   ||| | |||||||||||| ||
   ACUCUCUCACCUUCUAUUAAU 5'
AT1G28110.2     102     122
serine carboxypeptidase II (At1g28110)

sRNA_AGO1_Solexa_Mi2008_2_34881_hit1
5' UGAAGUGAAGACGAGA-UAG-GAUGG
   ||||||||||| |||| ||| |||||
   ACUUCACUUCUCCUCUGAUCACUACC 5'
AT1G28110.2     10      35
serine carboxypeptidase II (At1g28110)

sRNA_AGO1_Solexa_Mi2008_1_43831_hit1
5' UGGUUGUGGCUC-UGGUGGUAA
   |||| ||||| | |||||||||
   ACCACCACCG-GCACCACCAUU 5'
AT1G28290.2     158     178
proline-rich protein, putative

sRNA_AGO1_Solexa_Mi2008_1_35934_hit2
5' UGAG-AGUGAUGUGGGUGGUGGU
   |||| || | |||||||||||||
   ACUCCUCCC-ACACCCACCACCA 5'
AT1G28290.2     172     193
proline-rich protein, putative

sRNA_AGO1_Solexa_Mi2008_1_43831_hit1
5' UGGUUGUGGCUC-UGGUGGUAA
   |||| ||||| | |||||||||
   ACCACCACCG-GCACCACCAUU 5'
AT1G28290.1     276     296
proline-rich protein, putative

sRNA_AGO1_Solexa_Mi2008_1_35934_hit2
5' UGAG-AGUGAUGUGGGUGGUGGU
   |||| || | |||||||||||||
   ACUCCUCCC-ACACCCACCACCA 5'
AT1G28290.1     290     311
```

proline-rich protein, putative

sRNA_AGO1_Solexa_Mi2008_20_49676_hit3
5' UUCU-A-CCAUCCGAUCAACAAG
   |||| | |||||||||||||||
   AAGACUUGGUAGGCUAGUUGUUA 5'
AT1G28330.3   590   612
dormancy-associated protein

sRNA_AGO1_Solexa_Mi2008_2_54961_hit1
5' UUUCU-A-CCAUCCGAUCAACAA
   :|||| | |||||||||||||||
   GAAGACUUGGUAGGCUAGUUGUU 5'
AT1G28330.3   591   613
dormancy-associated protein

sRNA_AGO1_Solexa_Mi2008_10_54960_hit1
5' UUUCU-A-CCAUCCGAUCAACA
   :|||| | ||||||||||||||
   GAAGACUUGGUAGGCUAGUUGU 5'
AT1G28330.3   592   613
dormancy-associated protein

sRNA_AGO1_Solexa_Mi2008_1_1776_hit10
5' AGAAAAAAAAAAAAAAAAAAU
   | ||||||||||||||||||
   UGGUUUUUUUUUUUUUUUUUA 5'
AT1G28370.1   692   712
putative ethylene responsive element binding factor 4 protein

sRNA_AGO1_Solexa_Mi2008_1_3_hit25
5' AAAAAAAAAAAAAGAAAGA
   ||||||||||||| |||:
   UUUUUUUUUUUUU-UUUUA 5'
AT1G28370.1   693   710
putative ethylene responsive element binding factor 4 protein

sRNA_AGO1_Solexa_Mi2008_3_8177_hit1
5' CCUGA-UAAAGUAAAAUCUCCAU
   ||||| ||||||| ||||||||
   GGACUUAUUUCAUAAUAGAGGUA 5'
AT1G28370.1   950   972
putative ethylene responsive element binding factor 4 protein

sRNA_AGO1_Solexa_Mi2008_2_40270_hit1
5' UGGA-GAGCUAGAGUUGAUGGA
   |||| ||| |||||||||||||
   ACCUUCUC-AUCUCAACUACCU 5'
AT1G28440.1   373   393
unknown protein

sRNA_AGO1_Solexa_Mi2008_1_18621_hit1
5' UAAUACUAAACAUAUUCAUGG
   |||| ||||| ||||||||||
   AUUA-GAUUUCUAUAAGUACC 5'
AT1G29330.1   205   224
ER lumen protein retaining receptor

sRNA_AGO1_Solexa_Mi2008_1_3_hit25
5' AAAAAAAAAA-AAAGAAAGA
   |||||||||| ||| |||||
   UUUUUUUUUUCUUU-UUUCU 5'
AT1G30040.2   891   909

```
unknown protein

sRNA_AGO1_Solexa_Mi2008_9_11228_hit1
5' CUCUCCUGCAGUAGAUUCGUC
   |||||||||||||||||||||
   GAGAGGACGUCAUCUAAGCAG 5'
AT1G30330.2     1023    1043
putative protein

sRNA_AGO1_Solexa_Mi2008_1_39438_hit1
5' UGCUGUUGAGAGAUGAUGCUC
   ||||||||||||| | ||||
   ACGACAACUCUCU-CGACGAC 5'
AT1G30330.2     1555    1574
putative protein

sRNA_AGO1_Solexa_Mi2008_9_11228_hit1
5' CUCUCCUGCAGUAGAUUCGUC
   |||||||||||||||||||||
   GAGAGGACGUCAUCUAAGCAG 5'
AT1G30330.1     1780    1800
putative protein

sRNA_AGO1_Solexa_Mi2008_1_39236_hit1
5' UGCUCCAAUACGGAUUGAGUG
   |||||||||||||||||||||
   ACGAGGUUAUGCCUAACUCAC 5'
AT1G30330.2     2015    2035
putative protein

sRNA_AGO1_Solexa_Mi2008_1_39438_hit1
5' UGCUGUUGAGAGAUGAUGCUC
   ||||||||||||| | ||||
   ACGACAACUCUCU-CGACGAC 5'
AT1G30330.1     2312    2331
putative protein

sRNA_AGO1_Solexa_Mi2008_4_2102_hit1
5' AGCUGCCAGCAUGAUCUAU
   |||||||||| ||||||
   UCGACGGUCGGACUAGAGU 5'
AT1G30330.2     2518    2536
putative protein

sRNA_AGO1_Solexa_Mi2008_13_873_hit2
5' AAGCUGCCAGCAUGAUCUA
   ||||||||||| ||||||
   UUCGACGGUCGGACUAGAG 5'
AT1G30330.2     2519    2537
putative protein

sRNA_AGO1_Solexa_Mi2008_1_874_hit1
5' AAGCUGCCAGCAUGAUCUG
   ||||||||||| ||||||
   UUCGACGGUCGGACUAGAG 5'
AT1G30330.2     2519    2537
putative protein

sRNA_AGO1_Solexa_Mi2008_1_4776_hit1
5' CAAGCUGCCAGCCUGAUCUA
   ||||||||||||||||||
   GUUCGACGGUCGGACUAGAG 5'
AT1G30330.2     2519    2538
```

```
putative protein

sRNA_AGO1_Solexa_Mi2008_1519_13483_hit2
5' GAAGCUGCCAGCAUGAUCUA
    ||||||||||| ||||||
   GUUCGACGGUCGGACUAGAG 5'
AT1G30330.2     2519    2538
putative protein

sRNA_AGO1_Solexa_Mi2008_3_13485_hit1
5' GAAGCUGCCAGCAUGAUCUG
    ||||||||||| ||||||
   GUUCGACGGUCGGACUAGAG 5'
AT1G30330.2     2519    2538
putative protein

sRNA_AGO1_Solexa_Mi2008_54_13482_hit3
5' GAAGCUGCCAGCAUGAUCU
    ||||||||||| ||||||
   GUUCGACGGUCGGACUAGA 5'
AT1G30330.2     2520    2538
putative protein

sRNA_AGO1_Solexa_Mi2008_7845_34690_hit3
5' UGAAGCUGCCAGCAUGAUCU
     ||||||||||| ||||||
   UGUUCGACGGUCGGACUAGA 5'
AT1G30330.2     2520    2539
putative protein

sRNA_AGO1_Solexa_Mi2008_17_46569_hit1
5' UUAAGCUGCCAGCAUGAUCU
    :||||||||||| ||||||
   UGUUCGACGGUCGGACUAGA 5'
AT1G30330.2     2520    2539
putative protein

sRNA_AGO1_Solexa_Mi2008_6340_34689_hit3
5' UGAAGCUGCCAGCAUGAUC
     ||||||||||| |||||
   UGUUCGACGGUCGGACUAG 5'
AT1G30330.2     2521    2539
putative protein

sRNA_AGO1_Solexa_Mi2008_8_46568_hit1
5' UUAAGCUGCCAGCAUGAUC
    :||||||||||| |||||
   UGUUCGACGGUCGGACUAG 5'
AT1G30330.2     2521    2539
putative protein

sRNA_AGO1_Solexa_Mi2008_1_39236_hit1
5' UGCUCCAAUACGGAUUGAGUG
   |||||||||||||||||||||
   ACGAGGUUAUGCCUAACUCAC 5'
AT1G30330.1     2772    2792
putative protein

sRNA_AGO1_Solexa_Mi2008_2_15965_hit1
5' GUGGAUUAUACAGUUUUUGU
   : |||||||| |||||||||
   UCCCUAAUAUAUCAAAAACA 5'
AT1G30330.2     2950    2969
```

```
putative protein

sRNA_AGO1_Solexa_Mi2008_1_351_hit2
5' AAAGAAGAAAAACAGAUCU
   ||| |||||| ||||||||
   UUU-UUCUUUGUGUCUAGA 5'
AT1G30330.1     325     342
putative protein

sRNA_AGO1_Solexa_Mi2008_4_2102_hit1
5' AGCUGCCAGCAUGAUCUAU
   |||||||||| ||||||
   UCGACGGUCGGACUAGAGU 5'
AT1G30330.1     3275    3293
putative protein

sRNA_AGO1_Solexa_Mi2008_13_873_hit2
5' AAGCUGCCAGCAUGAUCUA
   ||||||||||| ||||||
   UUCGACGGUCGGACUAGAG 5'
AT1G30330.1     3276    3294
putative protein

sRNA_AGO1_Solexa_Mi2008_1_874_hit1
5' AAGCUGCCAGCAUGAUCUG
   ||||||||||| ||||||
   UUCGACGGUCGGACUAGAG 5'
AT1G30330.1     3276    3294
putative protein

sRNA_AGO1_Solexa_Mi2008_1_4776_hit1
5' CAAGCUGCCAGCCUGAUCUA
   ||||||||||||||||||
   GUUCGACGGUCGGACUAGAG 5'
AT1G30330.1     3276    3295
putative protein

sRNA_AGO1_Solexa_Mi2008_1519_13483_hit2
5' GAAGCUGCCAGCAUGAUCUA
    ||||||||||| ||||||
   GUUCGACGGUCGGACUAGAG 5'
AT1G30330.1     3276    3295
putative protein

sRNA_AGO1_Solexa_Mi2008_3_13485_hit1
5' GAAGCUGCCAGCAUGAUCUG
    ||||||||||| ||||||
   GUUCGACGGUCGGACUAGAG 5'
AT1G30330.1     3276    3295
putative protein

sRNA_AGO1_Solexa_Mi2008_54_13482_hit3
5' GAAGCUGCCAGCAUGAUCU
    ||||||||||| ||||||
   GUUCGACGGUCGGACUAGA 5'
AT1G30330.1     3277    3295
putative protein

sRNA_AGO1_Solexa_Mi2008_7845_34690_hit3
5' UGAAGCUGCCAGCAUGAUCU
     ||||||||||| ||||||
   UGUUCGACGGUCGGACUAGA 5'
AT1G30330.1     3277    3296
```

```
putative protein

sRNA_AGO1_Solexa_Mi2008_17_46569_hit1
5' UUAAGCUGCCAGCAUGAUCU
    :||||||||||| ||||||
   UGUUCGACGGUCGGACUAGA 5'
AT1G30330.1     3277    3296
putative protein

sRNA_AGO1_Solexa_Mi2008_6340_34689_hit3
5' UGAAGCUGCCAGCAUGAUC
     ||||||||||| |||||
   UGUUCGACGGUCGGACUAG 5'
AT1G30330.1     3278    3296
putative protein

sRNA_AGO1_Solexa_Mi2008_8_46568_hit1
5' UUAAGCUGCCAGCAUGAUC
    :||||||||||| |||||
   UGUUCGACGGUCGGACUAG 5'
AT1G30330.1     3278    3296
putative protein

sRNA_AGO1_Solexa_Mi2008_2_15965_hit1
5' GUGGAUUAUACAGUUUUUGU
   : |||||||| |||||||||
   UCCCUAAUAUAUCAAAAACA 5'
AT1G30330.1     3707    3726
putative protein

sRNA_AGO1_Solexa_Mi2008_1_9282_hit3
5' CGGUUUUUUCGGGU-UUUUUC
   | ||||||| |||| ||||||
   GACAAAAAA-CCCAAAAAAAG 5'
AT1G30490.1     17      36
HD-Zip protein

sRNA_AGO1_Solexa_Mi2008_1_39355_hit2
5' UGCUGAUGUG-UGGGCUUUUGG
   |||||||||| |||| |||||
   ACGACUACACAACCC-AAAACG 5'
AT1G30490.1     664     684
HD-Zip protein

sRNA_AGO1_Solexa_Mi2008_2_8957_hit1
5' CGGACCAGGCUUCAUCCCCCC
   |||||||||||||||||
   GCCUGGUCCGAAGUAGGGUUA 5'
AT1G30490.1     793     813
HD-Zip protein

sRNA_AGO1_Solexa_Mi2008_153_14822_hit2
5' GGACCAGGCUUCAUCCCCC
   ||||||||||||||||
   CCUGGUCCGAAGUAGGGUU 5'
AT1G30490.1     794     812
HD-Zip protein

sRNA_AGO1_Solexa_Mi2008_219_8956_hit2
5' CGGACCAGGCUUCAUCCCCC
   |||||||||||||||||
   GCCUGGUCCGAAGUAGGGUU 5'
AT1G30490.1     794     813
```

```
HD-Zip protein

sRNA_AGO1_Solexa_Mi2008_1063_31727_hit2
5' UCGGACCAGGCUUCAUCCCCC
   :||||||||||||||||||
   GGCCUGGUCCGAAGUAGGGUU 5'
AT1G30490.1     794     814
HD-Zip protein

sRNA_AGO1_Solexa_Mi2008_4_8955_hit2
5' CGGACCAGGCUUCAUCCCC
   ||||||||||||||||||
   GCCUGGUCCGAAGUAGGGU 5'
AT1G30490.1     795     813
HD-Zip protein

sRNA_AGO1_Solexa_Mi2008_8_8960_hit4
5' CGGACCAGGCUUCAUUCCCCC
   |||||||||||||| ||||
   GCCUGGUCCGAAGU-AGGGUU 5'
AT1G30490.1     795     814
HD-Zip protein

sRNA_AGO1_Solexa_Mi2008_47_31726_hit2
5' UCGGACCAGGCUUCAUCCCC
   :||||||||||||||||||
   GGCCUGGUCCGAAGUAGGGU 5'
AT1G30490.1     795     814
HD-Zip protein

sRNA_AGO1_Solexa_Mi2008_310_14823_hit7
5' GGACCAGGCUUCAUUCCCC
   ||||||||||||| ||||
   CCUGGUCCGAAGU-AGGGU 5'
AT1G30490.1     796     813
HD-Zip protein

sRNA_AGO1_Solexa_Mi2008_641_8959_hit7
5' CGGACCAGGCUUCAUUCCCC
   |||||||||||||| ||||
   GCCUGGUCCGAAGU-AGGGU 5'
AT1G30490.1     796     814
HD-Zip protein

sRNA_AGO1_Solexa_Mi2008_2_31725_hit2
5' UCGGACCAGGCUUCAUCCC
   :||||||||||||||||||
   GGCCUGGUCCGAAGUAGGG 5'
AT1G30490.1     796     814
HD-Zip protein

sRNA_AGO1_Solexa_Mi2008_4138_31731_hit7
5' UCGGACCAGGCUUCAUUCCCC
    |||||||||||||| ||||
   CGCCUGGUCCGAAGU-AGGGU 5'
AT1G30490.1     796     815
HD-Zip protein

sRNA_AGO1_Solexa_Mi2008_1_8958_hit7
5' CGGACCAGGCUUCAUUCCC
   |||||||||||||| ||||
   GCCUGGUCCGAAGU-AGGG 5'
AT1G30490.1     797     814
```

```
HD-Zip protein

sRNA_AGO1_Solexa_Mi2008_15_11085_hit1
5' CUCGGACCAGGCUUCAUCC
    :||||||||||||||||| 
   AGGCCUGGUCCGAAGUAGG 5'
AT1G30490.1     797     815
HD-Zip protein

sRNA_AGO1_Solexa_Mi2008_98_31730_hit7
5' UCGGACCAGGCUUCAUUCCC
    ||||||||||||| ||||
   CGCCUGGUCCGAAGU-AGGG 5'
AT1G30490.1     797     815
HD-Zip protein

sRNA_AGO1_Solexa_Mi2008_1_11087_hit3
5' CUCGGACCAGGCUUCAUUCCC
     ||||||||||||| ||||
   UCGCCUGGUCCGAAGU-AGGG 5'
AT1G30490.1     797     816
HD-Zip protein

sRNA_AGO1_Solexa_Mi2008_33_15725_hit3
5' GUCGGACCAGGCUUCAUUCCC
   : ||||||||||||| ||||
   UCGCCUGGUCCGAAGU-AGGG 5'
AT1G30490.1     797     816
HD-Zip protein

sRNA_AGO1_Solexa_Mi2008_68_49390_hit1
5' UUCGGACCAGGCUUCAUUCCC
     ||||||||||||| ||||
   UCGCCUGGUCCGAAGU-AGGG 5'
AT1G30490.1     797     816
HD-Zip protein

sRNA_AGO1_Solexa_Mi2008_6_31729_hit7
5' UCGGACCAGGCUUCAUUCC
    ||||||||||||| |||
   CGCCUGGUCCGAAGU-AGG 5'
AT1G30490.1     798     815
HD-Zip protein

sRNA_AGO1_Solexa_Mi2008_1_49389_hit1
5' UUCGGACCAGGCUUCAUUCC
     ||||||||||||| |||
   UCGCCUGGUCCGAAGU-AGG 5'
AT1G30490.1     798     816
HD-Zip protein

sRNA_AGO1_Solexa_Mi2008_2_49996_hit1
5' UUGAACAGAGUGAACAAAUUA
   |||||||||||  ||||||
   AACUUGUCUCAC--GUUUAAA 5'
AT1G30680.1     1518    1536
unknown protein

sRNA_AGO1_Solexa_Mi2008_1_24435_hit5
5' UAUAGAGAACCACCAUGAUUG
    |||||||| ||||||||||
   UUAUCUCUUUGUGGUACUAAG 5'
AT1G30680.1     2394    2414
```

```
unknown protein

sRNA_AGO1_Solexa_Mi2008_1_38600_hit1
5' UGCCGCAACAAAAGGAUGGU
   ||||||||||||||| ||
   ACGGCGUUGUUUUCCU-CCU 5'
AT1G30680.1     2529    2547
unknown protein

sRNA_AGO1_Solexa_Mi2008_1_26531_hit1
5' UAUUUUUGCAGAAAACUUUAAU
   |||||| || |||||||||||
   AUAAAA-CGACUUUUGAAAUUA 5'
AT1G30730.1     1600    1620
putative reticuline oxidase-like protein

sRNA_AGO1_Solexa_Mi2008_2_45154_hit1
5' UGUGCAUUUGGUGGUGUUUUC
   ||||| ||||||||||||||
   ACACG-AAACCACCACAAAAG 5'
AT1G30755.1     1422    1441

sRNA_AGO1_Solexa_Mi2008_1_52018_hit20
5' UUGGACACUGAAGACGA-GUUG
   |||||||||||| ||:| ||||
   AACCUGUGACUU-UGUUACAAC 5'
AT1G31910.1     2171    2191
hypothetical protein

sRNA_AGO1_Solexa_Mi2008_9_19222_hit1
5' UACAAUGUUCUCAUCAGGUCU
   |||||||||||||||||||||
   AUGUUACAAGAGUAGUCCAGA 5'
AT1G32120.1     1116    1136
hypothetical protein

sRNA_AGO1_Solexa_Mi2008_1_4815_hit1
5' CAAGGACCGAAUGAUGAUGU-GA
   ||||||||| |||| ||||| ||
   GUUCCUGGCAUACUUCUACAGCU 5'
AT1G33050.3     1176    1198
unknown protein

sRNA_AGO1_Solexa_Mi2008_3_53631_hit1
5' UUGUGGGCGUCCUGAAGCUUUC
   ||||||||||||||||||||||
   AACACCCGCAGGACUUCGAAAG 5'
AT1G33420.1     1306    1327
unknown protein

sRNA_AGO1_Solexa_Mi2008_1_50005_hit1
5' UUGAACCAGGAUUGCAACGUG
   |||||||||||||||||||||
   AACUUGGUCCUAACGUUGCAC 5'
AT1G33420.1     1455    1475
unknown protein

sRNA_AGO1_Solexa_Mi2008_1_8165_hit1
5' CCUCGUGCAAGCCUUAGUGUC
   |||||||||||||||||||||
   GGAGCACGUUCGGAAUCACAG 5'
AT1G33420.1     638     658
unknown protein
```

```
sRNA_AGO1_Solexa_Mi2008_3_35894_hit1
5' UGAGAGCAGAGAAAGAGAGU
   |||||| |||||||||||
   ACUCUC-UCUCUUUCUCUCU 5'
AT1G33700.2     22      40
unknown protein

sRNA_AGO1_Solexa_Mi2008_37_6798_hit10
5' CAUAAAAGAGGGAU-GCAAC
   |||||||||||| | ||||
   GUAUUUUCUCCC-AGCGUUU 5'
AT1G33700.1     3016    3034
unknown protein

sRNA_AGO1_Solexa_Mi2008_37_6798_hit10
5' CAUAAAAGAGGGAU-GCAAC
   |||||||||||| | ||||
   GUAUUUUCUCCC-AGCGUUU 5'
AT1G33700.2     3022    3040
unknown protein

sRNA_AGO1_Solexa_Mi2008_7_55923_hit1
5' UUUGGUCUUCAG-AAAGUUUUC
   |||||||||||| || |||||
   AAACCAGAAGUCCUUGCAAAAC 5'
AT1G33700.1     91      112
unknown protein

sRNA_AGO1_Solexa_Mi2008_2_38645_hit1
5' UGCCGUAACAUAACAGGUGGGU
   ||| ||| | ||||||||||||
   ACG-CAUCG-AUUGUCCACCCA 5'
AT1G33700.1     920     939
unknown protein

sRNA_AGO1_Solexa_Mi2008_2_38645_hit1
5' UGCCGUAACAUAACAGGUGGGU
   ||| ||| | ||||||||||||
   ACG-CAUCG-AUUGUCCACCCA 5'
AT1G33700.2     926     945
unknown protein

sRNA_AGO1_Solexa_Mi2008_7_55923_hit1
5' UUUGGUCUUCA-GAAAGUUUUC
   ||||||||||| ||| |||||
   AAACCAGAAGUCCUUGCAAAAC 5'
AT1G33700.2     97      118
unknown protein

sRNA_AGO1_Solexa_Mi2008_1_45719_hit1
5' UGUGUGUGUGUGUUGGUGUGU
   ||||||||||||||  ||||
   ACACACACACACAA--ACACU 5'
AT1G33760.1     13      31
TINY-like protein

sRNA_AGO1_Solexa_Mi2008_1_2738_hit1
5' AUAUUCU-CAGAUCGUUGGCCAU
    |||||| | |||||||||||||
   CAUAAGAAGACUAGCAACCGGUA 5'
AT1G33960.1     63      85
AIG1
```

```
sRNA_AGO1_Solexa_Mi2008_1_19645_hit1
5' UACAUUUGCUUG-A-GCCCUCUUG
   |||||||||||| | || ||||||
   AUGUAAACGAACUUACGAGAGAAC 5'
AT1G34370.3     347     370
zinc finger protein, putative

sRNA_AGO1_Solexa_Mi2008_1_19645_hit1
5' UACAUUUGCUUGA--GCCCUCUUG
   |||||||||||||  || ||||||
   AUGUAAACGAACUUACGAGAGAAC 5'
AT1G34370.1     350     373
zinc finger protein, putative

sRNA_AGO1_Solexa_Mi2008_1_19645_hit1
5' UACAUUUGCUUG-A-GCCCUCUUG
   |||||||||||| | || ||||||
   AUGUAAACGAACUUACGAGAGAAC 5'
AT1G34370.2     360     383
zinc finger protein, putative

sRNA_AGO1_Solexa_Mi2008_1_36323_hit13
5' UGAGGAUGAUGAUGAU-GAUGA
   ||| |||||||||||| || ||
   ACUACUACUACUACUAUCU-CU 5'
AT1G34370.3     38      58
zinc finger protein, putative

sRNA_AGO1_Solexa_Mi2008_1_3079_hit472
5' AUGAUGAUGAUGAUGAU-GAUGA
    |||||||||||||||| || ||
   GACUACUACUACUACUAUCU-CU 5'
AT1G34370.3     38      59
zinc finger protein, putative

sRNA_AGO1_Solexa_Mi2008_1_45039_hit3
5' UGUGAUGAUGAUGAUGAU-GAUGA
   |||||||||||||||||| || ||
   ACACUACUACUACUACUAUCU-CU 5'
AT1G34370.3     38      60
zinc finger protein, putative

sRNA_AGO1_Solexa_Mi2008_1_36323_hit13
5' UGAG-GAUGAUGAUGAUGAU-GA
   | || ||||||||||||||| ||
   AAUCACUACUACUACUACUAUCU 5'
AT1G34370.3     40      62
zinc finger protein, putative

sRNA_AGO1_Solexa_Mi2008_1_56092_hit3
5' UUUGUGAUGAUGAUGAUGAU-GA
   |||||||||||||||||||| ||
   AAACACUACUACUACUACUAUCU 5'
AT1G34370.3     40      62
zinc finger protein, putative

sRNA_AGO1_Solexa_Mi2008_1_36323_hit13
5' UGAGGAUGAUGAUGAU-GAUGA
   ||| |||||||||||| || ||
   ACUACUACUACUACUAUCU-CU 5'
AT1G34370.1     41      61
zinc finger protein, putative
```

```
sRNA_AGO1_Solexa_Mi2008_1_3079_hit472
5' AUGAUGAUGAUGAUGAU-GAUGA
    |||||||||||||||| || ||
   GACUACUACUACUACUAUCU-CU 5'
AT1G34370.1     41      62
zinc finger protein, putative

sRNA_AGO1_Solexa_Mi2008_1_45039_hit3
5' UGUGAUGAUGAUGAUGAU-GAUGA
   ||||||||||||||||| || ||
   ACACUACUACUACUACUAUCU-CU 5'
AT1G34370.1     41      63
zinc finger protein, putative

sRNA_AGO1_Solexa_Mi2008_1_2280_hit1
5' AGUGGAUGAUGAUGAUGAUG
   |||| ||||||||||||||:
   UCAC-UACUACUACUACUAU 5'
AT1G34370.3     42      60
zinc finger protein, putative

sRNA_AGO1_Solexa_Mi2008_1_36323_hit13
5' UGAG-GAUGAUGAUGAUGAU-GA
   | || ||||||||||||||| ||
   AAUCACUACUACUACUACUAUCU 5'
AT1G34370.1     43      65
zinc finger protein, putative

sRNA_AGO1_Solexa_Mi2008_1_56092_hit3
5' UUUGUGAUGAUGAUGAUGAU-GA
   |||||||||||||||||||| ||
   AAACACUACUACUACUACUAUCU 5'
AT1G34370.1     43      65
zinc finger protein, putative

sRNA_AGO1_Solexa_Mi2008_1_2280_hit1
5' AGUGGAUGAUGAUGAUGAUG
   |||| ||||||||||||||:
   UCAC-UACUACUACUACUAU 5'
AT1G34370.1     45      63
zinc finger protein, putative

sRNA_AGO1_Solexa_Mi2008_1_40229_hit3
5' UGGAGACAAGUGAUGAUGAUGAU
     |||| |||||||||||||||
   UACUCU-UUCACUACUACUACUA 5'
AT1G34370.3     46      67
zinc finger protein, putative

sRNA_AGO1_Solexa_Mi2008_1_36323_hit13
5' UGAGGAUGAUGAUGAU-GAUGA
   ||| |||||||||||| || ||
   ACUACUACUACUACUAUCU-CU 5'
AT1G34370.2     47      67
zinc finger protein, putative

sRNA_AGO1_Solexa_Mi2008_1_3079_hit472
5' AUGAUGAUGAUGAUGAU-GAUGA
    |||||||||||||||| || ||
   GACUACUACUACUACUAUCU-CU 5'
AT1G34370.2     47      68
zinc finger protein, putative
```

```
sRNA_AGO1_Solexa_Mi2008_1_45039_hit3
5' UGUGAUGAUGAUGAUGAU-GAUGA
   ||||||||||||||||| || ||
   ACACUACUACUACUACUAUCU-CU 5'
AT1G34370.2     47      69
zinc finger protein, putative

sRNA_AGO1_Solexa_Mi2008_1_40229_hit3
5' UGGAGACAAGUGAUGAUGAUGAU
     |||| |||||||||||||||
   UACUCU-UUCACUACUACUACUA 5'
AT1G34370.1     49      70
zinc finger protein, putative

sRNA_AGO1_Solexa_Mi2008_1_36323_hit13
5' UGAG-GAUGAUGAUGAUGAU-GA
   | || ||||||||||||||| ||
   AAUCACUACUACUACUACUAUCU 5'
AT1G34370.2     49      71
zinc finger protein, putative

sRNA_AGO1_Solexa_Mi2008_1_56092_hit3
5' UUUGUGAUGAUGAUGAUGAU-GA
   |||||||||||||||||||| ||
   AAACACUACUACUACUACUAUCU 5'
AT1G34370.2     49      71
zinc finger protein, putative

sRNA_AGO1_Solexa_Mi2008_1_2280_hit1
5' AGUGGAUGAUGAUGAUGAUG
   |||| ||||||||||||||:
   UCAC-UACUACUACUACUAU 5'
AT1G34370.2     51      69
zinc finger protein, putative

sRNA_AGO1_Solexa_Mi2008_1_40229_hit3
5' UGGAGACAAGUGAUGAUGAUGAU
     |||| |||||||||||||||
   UACUCU-UUCACUACUACUACUA 5'
AT1G34370.2     55      76
zinc finger protein, putative

sRNA_AGO1_Solexa_Mi2008_2_378_hit1
5' AAAGAGAAGGGAU-AUUUUU
   ||| ||||||||| |||||
   UUU-UCUUCCCUAUUAAAAC 5'
AT1G34750.1     276     294
protein phosphatase type 2C, putative

sRNA_AGO1_Solexa_Mi2008_1_28030_hit4
5' UCAGAAGAAGCCAC-GUCAGAUGA
   ||||||||||  || |||||||||
   AGUCUUCUUCU-UGGCAGUCUACU 5'
AT1G35210.1     259     281
unknown protein

sRNA_AGO1_Solexa_Mi2008_1_919_hit1
5' AAGGCUAAUGUUGUGAUCUUC
    |||| | ||||||||||||
   GUCCG-U-ACAACACUAGAAG 5'
AT1G35310.1     137     155
unknown protein
```

```
sRNA_AGO1_Solexa_Mi2008_1_14083_hit2
5' GAGGCCAAUGUUGUGAUCUA
   ||||| | |||||||||||
   CUCCG-U-ACAACACUAGAA 5'
AT1G35310.1     138     155
unknown protein

sRNA_AGO1_Solexa_Mi2008_2_9476_hit2
5' CUAAAAAUGGUGUAAUUU-GA
    |||||| |||||||||| ||
   AAUUUUU-CCACAUUAAAGCU 5'
AT1G35580.1     1994    2013
invertase, putative

sRNA_AGO1_Solexa_Mi2008_2_9476_hit2
5' CUAAAAAUGGUGUAAUUU-GA
    |||||| |||||||||| ||
   AAUUUUU-CCACAUUAAAGCU 5'
AT1G35580.2     2006    2025
invertase, putative

sRNA_AGO1_Solexa_Mi2008_1_26497_hit1
5' UAUUUGUGA-GAGUUCGUGAAA
   | ||| ||| ||||||||||||
   AGAAA-ACUACUCAAGCACUUU 5'
AT1G35710.1     129     149
protein kinase, putative

sRNA_AGO1_Solexa_Mi2008_1_19304_hit2
5' UACACGUUCAUCGGAAAUGCU
   |||||||||||||||||||||
   AUGUGCAAGUAGCCUUUACGA 5'
AT1G35710.1     3219    3239
protein kinase, putative

sRNA_AGO1_Solexa_Mi2008_2_5215_hit1
5' CAAUUAG-UGGAGAAGUUCA
    |||| | ||||||||||||
   AUUAA-CUACCUCUUCAAGU 5'
AT1G35710.1     3368    3386
protein kinase, putative

sRNA_AGO1_Solexa_Mi2008_10_17146_hit1
5' UAACAACAACAAC-AAAGGUGAA
   ||||||||||||| |||| ||||
   AUUGUUGUUGUUGUUUUCGACUU 5'
AT1G35710.1     3487    3509
protein kinase, putative

sRNA_AGO1_Solexa_Mi2008_1_18621_hit1
5' UAAUACUAAACAUAUUCAUGG
   ||||||||| |||||||||
   AUUAUGAUUGGUAUAAGUAAA 5'
AT1G36060.1     1809    1829
putative AP2 domain containing protein RAP2.4 gi|2281633

sRNA_AGO1_Solexa_Mi2008_1_6293_hit1
5' CAGCCAG-CGUGGAAGCACU
    ||| || ||||||||||||
   AUCG-UCUGCACCUUCGUGA 5'
AT1G36060.1     736     754
putative AP2 domain containing protein RAP2.4 gi|2281633
```

```
sRNA_AGO1_Solexa_Mi2008_3_31107_hit1
5' UCGAGUUUUCGGGUU-UUGAA
   :|:|||||||||||| |||||
   GGUUCAAAAGCCCAACAACUU 5'
AT1G36060.1     929     949
putative AP2 domain containing protein RAP2.4 gi|2281633

sRNA_AGO1_Solexa_Mi2008_1_28845_hit2
5' UCAUCACUAGGAAGCGUUGGA
   |||| ||:||||||||||||
   AGUAAUGGUCCUUCGCAACCU 5'
AT1G37130.1     1592    1612
nitrate reductase (At1g37130)

sRNA_AGO1_Solexa_Mi2008_12_19915_hit2
5' UACCGUCCUAGUCUCAAGCCAUA
   ||| ||||||||||||| |||
   AUG-CAGGAUCAGAGUUCGUAA 5'
AT1G42990.1     537     557
bZip transcription factor AtbZip60

sRNA_AGO1_Solexa_Mi2008_1_43567_hit2
5' UGGUGGUGGAGCGGUUGA-GAA
   |||||||||||| ||||| |||
   ACCACCACCUCG-CAACUCCUU 5'
AT1G43700.1     305     325
VirE2-interacting protein VIP1 / bZip factor AtbZip51

sRNA_AGO1_Solexa_Mi2008_2_35306_hit1
5' UGACAUCGAUGA-UUGGUUUCG
   |||||| || || |||||||||
   ACUGUAUCU-CUUAACCAAAGC 5'
AT1G44750.2     336     356
Unknown protein (At1g44750)

sRNA_AGO1_Solexa_Mi2008_2_35306_hit1
5' UGACAUCGAUGA-UUGGUUUCG
   |||||| || || |||||||||
   ACUGUAUCU-CUUAACCAAAGC 5'
AT1G44750.3     88      108
Unknown protein (At1g44750)

sRNA_AGO1_Solexa_Mi2008_2_44202_hit1
5' UGUAGGACGAAUGCUUUGGUA
   |||||||||||||||||||||
   ACAUCCUGCUUACGAAACCAU 5'
AT1G48410.1     1250    1270
Argonaute protein (AGO1)

sRNA_AGO1_Solexa_Mi2008_2_44202_hit1
5' UGUAGGACGAAUGCUUUGGUA
   |||||||||||||||||||||
   ACAUCCUGCUUACGAAACCAU 5'
AT1G48410.2     1256    1276
Argonaute protein (AGO1)

sRNA_AGO1_Solexa_Mi2008_13_6596_hit1
5' CAGGUAACCUUCAGCAAAGCA
   |||||||||||||||||||||
   GUCCAUUGGAAGUCGUUUCGU 5'
AT1G48410.1     1711    1731
Argonaute protein (AGO1)
```

```
sRNA_AGO1_Solexa_Mi2008_13_6596_hit1
5' CAGGUAACCUUCAGCAAAGCA
   |||||||||||||||||||||
   GUCCAUUGGAAGUCGUUUCGU 5'
AT1G48410.2     1717    1737
Argonaute protein (AGO1)

sRNA_AGO1_Solexa_Mi2008_16_26870_hit1
5' UCAACAGAAGCCAGAGAAGUA
   |||||||||||||||||||||
   AGUUGUCUUCGGUCUCUUCAU 5'
AT1G48410.1     1831    1851
Argonaute protein (AGO1)

sRNA_AGO1_Solexa_Mi2008_16_26870_hit1
5' UCAACAGAAGCCAGAGAAGUA
   |||||||||||||||||||||
   AGUUGUCUUCGGUCUCUUCAU 5'
AT1G48410.2     1837    1857
Argonaute protein (AGO1)

sRNA_AGO1_Solexa_Mi2008_3_40523_hit1
5' UGGAGGAGGCAGUAUACGAGC
   |||||||||||||||||||||
   ACCUCCUCCGUCAUAUGCUCG 5'
AT1G48410.1     1853    1873
Argonaute protein (AGO1)

sRNA_AGO1_Solexa_Mi2008_3_40523_hit1
5' UGGAGGAGGCAGUAUACGAGC
   |||||||||||||||||||||
   ACCUCCUCCGUCAUAUGCUCG 5'
AT1G48410.2     1859    1879
Argonaute protein (AGO1)

sRNA_AGO1_Solexa_Mi2008_2_28543_hit1
5' UCAGGGCGAGCACUGACUGGU
   |||||||||||||||||||||
   AGUCCCGCUCGUGACUGACCA 5'
AT1G48410.1     2098    2118
Argonaute protein (AGO1)

sRNA_AGO1_Solexa_Mi2008_2_28543_hit1
5' UCAGGGCGAGCACUGACUGGU
   |||||||||||||||||||||
   AGUCCCGCUCGUGACUGACCA 5'
AT1G48410.2     2104    2124
Argonaute protein (AGO1)

sRNA_AGO1_Solexa_Mi2008_2_36850_hit22
5' UGAGUGUGGUCCUCCUCUUCC
   |||| | ||||||||||||||
   ACUC-CUCCAGGAGGAGAAGG 5'
AT1G48410.2     417     436
Argonaute protein (AGO1)

sRNA_AGO1_Solexa_Mi2008_1_55475_hit2
5' UUUG-CUC-GCGGUGGUCCAGAC
   |||| ||| |||||||||||||
   AAACAGAGACGCCACCAGGUCUU 5'
AT1G48410.2     485     507
Argonaute protein (AGO1)
```

```
sRNA_AGO1_Solexa_Mi2008_1_39168_hit1
5' UGCUAGAAGAAGGUAUAGGCU
   |||||||||||||||||||||
   ACGAUCUUCUUCCAUAUCCGA 5'
AT1G48410.2     669     689
Argonaute protein (AGO1)

sRNA_AGO1_Solexa_Mi2008_5_6229_hit1
5' CAGCAAAGAAAUGGUUAGCC
   ||||||||||||||||||||
   GUCGUUUCUUUACCAAUCGG 5'
AT1G48410.2     751     770
Argonaute protein (AGO1)

sRNA_AGO1_Solexa_Mi2008_1_28240_hit1
5' UCAGCAAAGAAAUGGUUAGCC
   |||||||||||||||||||||
   AGUCGUUUCUUUACCAAUCGG 5'
AT1G48410.2     751     771
Argonaute protein (AGO1)

sRNA_AGO1_Solexa_Mi2008_1_28621_hit15
5' UC-AGGUUGUCUGGUU-GAGUG
   || ||||||||||||| ||| |
   AGAUCCAACAGACCAAACUCUC 5'
AT1G49470.1     293     314
unknown protein

sRNA_AGO1_Solexa_Mi2008_1_18744_hit1
5' UAAUGAGGUGGAGGAACAUGGA
   ||||||||||||||||||||||
   AUUACUCCACCUCCUUGUACCU 5'
AT1G49470.1     321     342
unknown protein

sRNA_AGO1_Solexa_Mi2008_2_16084_hit1
5' GUU-AGAGGAAGAGGUAAAA
   :|| ||||||||||| ||||
   UAAGUCUCCUUCUCC-UUUU 5'
AT1G50380.1     115     133
oligopeptidase-like protein

sRNA_AGO1_Solexa_Mi2008_7_42241_hit1
5' UGGG-CUUCAUUUCUAUCAGUU
    ||| |||||||||| ||||||
   UCCCAGAAGUAAAGAGAGUCAA 5'
AT1G50380.1     2144    2165
oligopeptidase-like protein

sRNA_AGO1_Solexa_Mi2008_1_3_hit25
5' AA-AAAAAA-AAAAAGAAAGA
   || |||||| |||||||||:|
   UUGUUUUUUCUUUUUCUUUUU 5'
AT1G50380.1     2435    2455
oligopeptidase-like protein

sRNA_AGO1_Solexa_Mi2008_1_53557_hit1
5' UUGUGAGUAAUA--GUGUAUGUGA
   ||||||||||||  |||| |||
   AACACUCAUUAUCGCACACACA 5'
AT1G50420.1     -1      22
scarecrow 3 -like protein
```

```
sRNA_AGO1_Solexa_Mi2008_1_12127_hit1
5' CUGGUGGUUAUCUUGUGAGUA
   |||||||||||||||||||||
   GACCACCAAUAGAACACUCAU 5'
AT1G50420.1     13      33
scarecrow 3 -like protein

sRNA_AGO1_Solexa_Mi2008_1_14022_hit2
5' GAGCUCU-UUC-UUGAUUCUA
   | ||||| ||| |||||||||
   CCCGAGAGAAGGAACUAAGAU 5'
AT1G50420.1     1613    1633
scarecrow 3 -like protein

sRNA_AGO1_Solexa_Mi2008_4_44854_hit1
5' UGUCUUCCAGGCAUUGAUGAU
   ||||| ||| ||||||||||
   ACAGA-GGUACGUAACUACUC 5'
AT1G51380.1     942     961
putative RNA helicase

sRNA_AGO1_Solexa_Mi2008_10_6379_hit1
5' CAGCUUAGGACAAUUUGACU
   ||||||||||| | |||||
   GUCGAAUCCUG-U-AACUGU 5'
AT1G51890.1     2013    2030
Putative protein kinase

sRNA_AGO1_Solexa_Mi2008_2_9562_hit1
5' CUAAC-AAACGCUACACCGUCGU
   ||||| ||| ||||||||:||||
   GAUUGCUUU-CGAUGUGGUAGCA 5'
AT1G52030.1     1749    1770
unknown protein

sRNA_AGO1_Solexa_Mi2008_2_9562_hit1
5' CUAAC-AAACGCUACACCGUCGU
   ||||| ||| ||||||||:||||
   GAUUGCUUU-CGAUGUGGUAGCA 5'
AT1G52030.2     1948    1969
unknown protein

sRNA_AGO1_Solexa_Mi2008_5_14577_hit1
5' GCC-GAGAGAUGGAAACGUU
    || ||:|||||||||||||
   AGGACUUUCUACCUUUGCAA 5'
AT1G52030.2     340     359
unknown protein

sRNA_AGO1_Solexa_Mi2008_1_1209_hit1
5' AAUUGGUGCUGACCACUCGUC
   |||||||||||||||||||||
   UUAACCACGACUGGUGAGCAG 5'
AT1G52150.3     1127    1147
unknown protein

sRNA_AGO1_Solexa_Mi2008_8_8960_hit4
5' CGGACCAGGCUUCAUUCCCCC
   ||||||||||||||||||
   GCCUGGUCCGAAGUAAGGUCC 5'
AT1G52150.3     1266    1286
unknown protein
```

```
sRNA_AGO1_Solexa_Mi2008_310_14823_hit7
5' GGACCAGGCUUCAUUCCCC
   |||||||||||||||||
   CCUGGUCCGAAGUAAGGUC 5'
AT1G52150.3     1267    1285
unknown protein

sRNA_AGO1_Solexa_Mi2008_641_8959_hit7
5' CGGACCAGGCUUCAUUCCCC
   ||||||||||||||||||
   GCCUGGUCCGAAGUAAGGUC 5'
AT1G52150.3     1267    1286
unknown protein

sRNA_AGO1_Solexa_Mi2008_4138_31731_hit7
5' UCGGACCAGGCUUCAUUCCCC
   :||||||||||||||||||
   GGCCUGGUCCGAAGUAAGGUC 5'
AT1G52150.3     1267    1287
unknown protein

sRNA_AGO1_Solexa_Mi2008_1_8958_hit7
5' CGGACCAGGCUUCAUUCCC
   ||||||||||||||||||
   GCCUGGUCCGAAGUAAGGU 5'
AT1G52150.3     1268    1286
unknown protein

sRNA_AGO1_Solexa_Mi2008_4_8955_hit2
5' CGGACCAGGCUUCAU-CCCC
   ||||||||||||||| ||
   GCCUGGUCCGAAGUAAGGUC 5'
AT1G52150.3     1268    1287
unknown protein

sRNA_AGO1_Solexa_Mi2008_98_31730_hit7
5' UCGGACCAGGCUUCAUUCCC
   :||||||||||||||||||
   GGCCUGGUCCGAAGUAAGGU 5'
AT1G52150.3     1268    1287
unknown protein

sRNA_AGO1_Solexa_Mi2008_1_11087_hit3
5' CUCGGACCAGGCUUCAUUCCC
    :||||||||||||||||||
   AGGCCUGGUCCGAAGUAAGGU 5'
AT1G52150.3     1268    1288
unknown protein

sRNA_AGO1_Solexa_Mi2008_33_15725_hit3
5' GUCGGACCAGGCUUCAUUCCC
    :||||||||||||||||||
   AGGCCUGGUCCGAAGUAAGGU 5'
AT1G52150.3     1268    1288
unknown protein

sRNA_AGO1_Solexa_Mi2008_68_49390_hit1
5' UUCGGACCAGGCUUCAUUCCC
   |:||||||||||||||||||
   AGGCCUGGUCCGAAGUAAGGU 5'
AT1G52150.3     1268    1288
unknown protein
```

```
sRNA_AGO1_Solexa_Mi2008_6_31729_hit7
5' UCGGACCAGGCUUCAUUCC
   :||||||||||||||||||
   GGCCUGGUCCGAAGUAAGG 5'
AT1G52150.3     1269    1287
unknown protein

sRNA_AGO1_Solexa_Mi2008_2_31725_hit2
5' UCGGACCAGGCUUCAU-CCC
    ||||||||||||||| ||
   CGCCUGGUCCGAAGUAAGGU 5'
AT1G52150.3     1269    1288
unknown protein

sRNA_AGO1_Solexa_Mi2008_1_49389_hit1
5' UUCGGACCAGGCUUCAUUCC
   |:||||||||||||||||||
   AGGCCUGGUCCGAAGUAAGG 5'
AT1G52150.3     1269    1288
unknown protein

sRNA_AGO1_Solexa_Mi2008_15_11085_hit1
5' CUCGGACCAGGCUUCAU-CC
     ||||||||||||||| ||
   UCGCCUGGUCCGAAGUAAGG 5'
AT1G52150.3     1270    1289
unknown protein

sRNA_AGO1_Solexa_Mi2008_1_28246_hit1
5' UCAGCAACAUCAACAACAACU
    |||||||||||| ||||||
   UGUCGUUGUAGUUCUUGUUGU 5'
AT1G52150.1     3310    3330
unknown protein

sRNA_AGO1_Solexa_Mi2008_1_28246_hit1
5' UCAGCAACAUCAACAACAACU
    |||||||||||| ||||||
   UGUCGUUGUAGUUCUUGUUGU 5'
AT1G52150.2     3313    3333
unknown protein

sRNA_AGO1_Solexa_Mi2008_1_351_hit2
5' AAAGAA-GAAAAACAGAUCU
    ||||| |||||||||| ||
   CUUCUUUCUUUUUGUCU-GA 5'
AT1G52150.3     332     350
unknown protein

sRNA_AGO1_Solexa_Mi2008_1_28246_hit1
5' UCAGCAACAUCAACAACAACU
    |||||||||||| ||||||
   UGUCGUUGUAGUUCUUGUUGU 5'
AT1G52150.3     3389    3409
unknown protein

sRNA_AGO1_Solexa_Mi2008_1_34620_hit1
5' UGAAGAUGAUGAUGAAGUU
   ||||||:||||||||||
   ACUUCUGCUACUACUUCUU 5'
AT1G52160.1     154     172
unknown protein
```

```
sRNA_AGO1_Solexa_Mi2008_2_50720_hit1
5' UUGAGGGAGACAGAGAUAAG
    ||||||||||||||| |||
   UACUCCCUCUGUCUCU-UUC 5'
AT1G52190.1     87      105
peptide transporter like protein

sRNA_AGO1_Solexa_Mi2008_1_29992_hit1
5' UCCGGCUCCGGUGGAAGAUUGA
   |||||| |||||||| ||| ||
   AGGCCGCGGCCACCU-CUA-CU 5'
AT1G52290.1     115     134
protein kinase, putative

sRNA_AGO1_Solexa_Mi2008_1_21321_hit1
5' UAGAGCGUCGCCGUGUCUG-UG
   :||| |||||||||||||| ||
   GUCU-GCAGCGGCACAGACCAC 5'
AT1G52290.1     149     169
protein kinase, putative

sRNA_AGO1_Solexa_Mi2008_1_1813_hit1
5' AGAAGGUCCGUUGAUGGAUGA
   |||||||||| |||||||||
   UCUUCCAGGCUACUACCUACC 5'
AT1G52290.1     183     203
protein kinase, putative

sRNA_AGO1_Solexa_Mi2008_1_27045_hit1
5' UCAAGAAGGUCCGUUGAUGGAU
   : ||||||||||| ||||||||
   GCUUCUUCCAGGCUACUACCUA 5'
AT1G52290.1     185     206
protein kinase, putative

sRNA_AGO1_Solexa_Mi2008_2_27044_hit1
5' UCAAGAAGGUCCGUUGAUGG
   : ||||||||||| ||||||
   GCUUCUUCCAGGCUACUACC 5'
AT1G52290.1     187     206
protein kinase, putative

sRNA_AGO1_Solexa_Mi2008_1_39699_hit1
5' UGGAACUGA-UGAUGAUGAUGA
   ||||||| | ||||||||||||
   ACCUUGA-UGACUACUACUACU 5'
AT1G52310.1     898     918
protein kinase, putative

sRNA_AGO1_Solexa_Mi2008_1_34289_hit2
5' UGAAAUCGAUGUUGU-AAGUCC
   :||||||||||| || ||||||
   GCUUUAGCUACAUCAUUUCAGG 5'
AT1G52400.2     1567    1588
beta-glucosidase, putative

sRNA_AGO1_Solexa_Mi2008_3_21212_hit1
5' UAGAGAGAGAAAAAGAUG-GC
   ||||||||||| |||||| ||
   AUCUCUCUCUUCUUCUACUCG 5'
AT1G52740.1     48      68
putative histone H2A
```

```
sRNA_AGO1_Solexa_Mi2008_1_4317_hit1
5' CAACAAACAGCAUU-ACAGCAAG
   ||||||||| || | ||||||||
   GUUGUUUGU-GUUACUGUCGUUC 5'
AT1G52780.1     3207    3228
unknown protein

sRNA_AGO1_Solexa_Mi2008_2_19339_hit1
5' UACAGAACGACAAGGGAGAG
    ||||||||| ||||||||:
   UUGUCUUGCUCUUCCCUCUU 5'
AT1G52780.1     3285    3304
unknown protein

sRNA_AGO1_Solexa_Mi2008_1_34058_hit3
5' UGAAAAACUCUCGCGGUUAUUU
   ||||||||||||||  |||||
   ACUUUUUGAGAGCG--AAUAAC 5'
AT1G53200.1     2004    2023
hypothetical protein; similar to EST gb|AI997566.1

sRNA_AGO1_Solexa_Mi2008_1_17452_hit1
5' UAACGCCGCAAUGA-GAAGACG
   |||||||||||||| || |||
   AUUGCGGCGUUACUGCUACUGG 5'
AT1G53710.1     1637    1658
cell division control protein like protein

sRNA_AGO1_Solexa_Mi2008_1_17452_hit1
5' UAACGCCGCAAUGA-GAAGACG
   |||||||||||||| || |||
   AUUGCGGCGUUACUGCUACUGG 5'
AT1G53710.2     1642    1663
cell division control protein like protein

sRNA_AGO1_Solexa_Mi2008_20_3894_hit2
5' CAAAACCCGUUGAUCAAUGAA
   ||||||| |:||||||||||
   GUUUUGG-CGACUAGUUACUG 5'
AT1G53710.2     795     814
cell division control protein like protein

sRNA_AGO1_Solexa_Mi2008_20_3894_hit2
5' CAAAACCCGUUGAUCAAUGAA
   ||||||| |:||||||||||
   GUUUUGG-CGACUAGUUACUG 5'
AT1G53710.1     870     889
cell division control protein like protein

sRNA_AGO1_Solexa_Mi2008_1_5984_hit23
5' CAGACAGAAAAUAAAAC-AC
   |||| |||||||||||| ||
   GUCU-UCUUUUAUUUUGCUG 5'
AT1G53780.1     1410    1428
hypothetical protein

sRNA_AGO1_Solexa_Mi2008_1_2_hit38
5' AAAAA-AAAAAAAAAACCAU
   ||||| ||||||||||| ||
   UUUUUGUUUUUUUUUUGAUA 5'
AT1G53780.1     1505    1524
hypothetical protein
```

```
sRNA_AGO1_Solexa_Mi2008_1_19021_hit2
5' UACAAAAUACAAAU-UAUGCAUA
   ||||||||||  || ||||||||
   AUGUUUUAUG-GUAGAUACGUAU 5'
AT1G53790.2     900     921
null

sRNA_AGO1_Solexa_Mi2008_3_39478_hit1
5' UGCUUC-UCAGAUGUCUUGAA
   |||||| |||| |||||||||
   ACGAAGGAGUCGACAGAACUU 5'
AT1G53910.3     584     604
unknown protein

sRNA_AGO1_Solexa_Mi2008_3_39478_hit1
5' UGCUUC-UCAGAUGUCUUGAA
   |||||| |||| |||||||||
   ACGAAGGAGUCGACAGAACUU 5'
AT1G53910.2     590     610
unknown protein

sRNA_AGO1_Solexa_Mi2008_10_12779_hit2
5' CUUGAGGGUGUAAAUGUUCGA
   |||||||||||||||||||||
   GAACUCCCACAUUUACAAGCU 5'
AT1G53910.3     99      119
unknown protein

sRNA_AGO1_Solexa_Mi2008_1_19666_hit1
5' UACCAACAGCAGAAUC-CACCAU
   |||||||||||:|||| || |||
   AUGGUUGUCGUUUUAGUGU-GUA 5'
AT1G54100.2     487     508
unknown protein

sRNA_AGO1_Solexa_Mi2008_1_19666_hit1
5' UACCAACAGCAGAAUC-CACCAU
   |||||||||||:|||| || |||
   AUGGUUGUCGUUUUAGUGU-GUA 5'
AT1G54100.1     538     559
unknown protein

sRNA_AGO1_Solexa_Mi2008_7_353_hit2
5' AAAGAAGAAGAU-AA-AGCAU
   |||||||||||| || || ||
   UUUCUUCUUCUACUUAUCCUA 5'
AT1G54115.1     76      96

sRNA_AGO1_Solexa_Mi2008_1_352_hit1
5' AAAGAAGAAGAU-AAAAGGA
   |||||||||||| || ||||
   UUUCUUCUUCUACUUAUCCU 5'
AT1G54115.1     77      96

sRNA_AGO1_Solexa_Mi2008_6_13422_hit1
5' GAAGAAGAAGAUGAUUAAGGA
    ||||||||||||| | ||||
   AUUCUUCUUCUACUUA-UCCU 5'
AT1G54115.1     77      96

sRNA_AGO1_Solexa_Mi2008_1_12560_hit1
5' CUUCACAAGGUAC-UGAUGGA
```

```
    |||||||||||| ||||||
   UAAGUGUUCCAUGUACUACCA 5'
AT1G54320.1     1134    1154
unknown protein

sRNA_AGO1_Solexa_Mi2008_12_46414_hit1
5' UUAACGCCGUGAUUGUU-UGGU
   |||||| |||||||||| ||:|
   AAUUGCUGCACUAACAACACUA 5'
AT1G55020.1     340     361
unknown protein

sRNA_AGO1_Solexa_Mi2008_1_28726_hit1
5' UCAGUGUGGAAGCUAAGUCUC
   ||| |||||||| |||||||
   AGU-ACACCUUCUAUUCAGAA 5'
AT1G55020.1     577     596
unknown protein

sRNA_AGO1_Solexa_Mi2008_1_46444_hit1
5' UUAACUCUGUGAUUGUUU-GGU
   |||||||||||||| ||| ||:
   AAUUGAGACACUAAGAAAUCCG 5'
AT1G56010.1     1000    1021
NAC1

sRNA_AGO1_Solexa_Mi2008_1_46444_hit1
5' UUAACUCUGUGAUUGUUU-GGU
   |||||||||||||| ||| ||:
   AAUUGAGACACUAAGAAAUCCG 5'
AT1G56010.2     1025    1046
NAC1

sRNA_AGO1_Solexa_Mi2008_1_13477_hit1
5' GAAGCAGGGCACGUG--CAUU
   ||||||||| |||||  ||||
   CUUCGUCCCAUGCACGAGUAA 5'
AT1G56010.1     773     793
NAC1

sRNA_AGO1_Solexa_Mi2008_2_14844_hit1
5' GGAGAAGCAGGGCACGUG--CAUU
   |||||||||||| |||||  ||||
   CCUCUUCGUCCCAUGCACGAGUAA 5'
AT1G56010.1     773     796
NAC1

sRNA_AGO1_Solexa_Mi2008_91_40198_hit1
5' UGGAGAAGCAGGGCACGUG--CAUU
   ||||||||||||| |||||  ||||
   ACCUCUUCGUCCCAUGCACGAGUAA 5'
AT1G56010.1     773     797
NAC1

sRNA_AGO1_Solexa_Mi2008_2_13910_hit1
5' GAGAAGCAGGGCACGUGCAU
   ||||||||||| |||||| :
   CUCUUCGUCCCAUGCACGAG 5'
AT1G56010.1     775     794
NAC1

sRNA_AGO1_Solexa_Mi2008_1_14842_hit1
5' GGAGAAGCAGGGCACGUGCAA
```

```
   |||||||||||| ||||||
   CCUCUUCGUCCCAUGCACGAG 5'
AT1G56010.1     775     795
NAC1

sRNA_AGO1_Solexa_Mi2008_4_14843_hit1
5' GGAGAAGCAGGGCACGUGCAU
   |||||||||||| |||||| :
   CCUCUUCGUCCCAUGCACGAG 5'
AT1G56010.1     775     795
NAC1

sRNA_AGO1_Solexa_Mi2008_1_14846_hit1
5' GGAGAAGCAGGGCACGUGCGA
   |||||||||||| ||||||
   CCUCUUCGUCCCAUGCACGAG 5'
AT1G56010.1     775     795
NAC1

sRNA_AGO1_Solexa_Mi2008_3_40196_hit1
5' UGGAGAAGCAGGGCACGUGCAA
   ||||||||||||| ||||||
   ACCUCUUCGUCCCAUGCACGAG 5'
AT1G56010.1     775     796
NAC1

sRNA_AGO1_Solexa_Mi2008_284_40197_hit1
5' UGGAGAAGCAGGGCACGUGCAU
   ||||||||||||| |||||| :
   ACCUCUUCGUCCCAUGCACGAG 5'
AT1G56010.1     775     796
NAC1

sRNA_AGO1_Solexa_Mi2008_10440_13909_hit2
5' GAGAAGCAGGGCACGUGCA
   ||||||||||| ||||||
   CUCUUCGUCCCAUGCACGA 5'
AT1G56010.1     776     794
NAC1

sRNA_AGO1_Solexa_Mi2008_110_13911_hit1
5' GAGAAGCAGGGCACGUGCG
   ||||||||||| ||||||
   CUCUUCGUCCCAUGCACGA 5'
AT1G56010.1     776     794
NAC1

sRNA_AGO1_Solexa_Mi2008_41322_14841_hit2
5' GGAGAAGCAGGGCACGUGCA
   |||||||||||| ||||||
   CCUCUUCGUCCCAUGCACGA 5'
AT1G56010.1     776     795
NAC1

sRNA_AGO1_Solexa_Mi2008_422_14845_hit1
5' GGAGAAGCAGGGCACGUGCG
   |||||||||||| ||||||
   CCUCUUCGUCCCAUGCACGA 5'
AT1G56010.1     776     795
NAC1

sRNA_AGO1_Solexa_Mi2008_370931_40195_hit2
5' UGGAGAAGCAGGGCACGUGCA
```

```
   ||||||||||||| ||||||
   ACCUCUUCGUCCCAUGCACGA 5'
AT1G56010.1     776     796
NAC1

sRNA_AGO1_Solexa_Mi2008_7589_40199_hit1
5' UGGAGAAGCAGGGCACGUGCG
   ||||||||||||| ||||||
   ACCUCUUCGUCCCAUGCACGA 5'
AT1G56010.1     776     796
NAC1

sRNA_AGO1_Solexa_Mi2008_2_40202_hit1
5' UGGAGAAGCAGGGUACGUGCU
   |||||||||||||||||||||
   ACCUCUUCGUCCCAUGCACGA 5'
AT1G56010.1     776     796
NAC1

sRNA_AGO1_Solexa_Mi2008_1214_3232_hit1
5' AUGGAGAAGCAGGGCACGUGCA
    ||||||||||||| ||||||
   AACCUCUUCGUCCCAUGCACGA 5'
AT1G56010.1     776     797
NAC1

sRNA_AGO1_Solexa_Mi2008_24_3233_hit1
5' AUGGAGAAGCAGGGCACGUGCG
    ||||||||||||| ||||||
   AACCUCUUCGUCCCAUGCACGA 5'
AT1G56010.1     776     797
NAC1

sRNA_AGO1_Solexa_Mi2008_9_52081_hit1
5' UUGGAGAAGCAGGGCACGUGCA
   |||||||||||||| ||||||
   AACCUCUUCGUCCCAUGCACGA 5'
AT1G56010.1     776     797
NAC1

sRNA_AGO1_Solexa_Mi2008_23_14840_hit3
5' GGAGAAGCAGGGCACGUGC
   |||||||||||| ||||||
   CCUCUUCGUCCCAUGCACG 5'
AT1G56010.1     777     795
NAC1

sRNA_AGO1_Solexa_Mi2008_1_40190_hit1
5' UGGAGAAGCAGGGCACGUAA
   ||||||||||||| ||||
   ACCUCUUCGUCCCAUGCACG 5'
AT1G56010.1     777     796
NAC1

sRNA_AGO1_Solexa_Mi2008_404_40194_hit3
5' UGGAGAAGCAGGGCACGUGC
   ||||||||||||| ||||||
   ACCUCUUCGUCCCAUGCACG 5'
AT1G56010.1     777     796
NAC1

sRNA_AGO1_Solexa_Mi2008_2_40201_hit1
5' UGGAGAAGCAGGGUACGUGC
```

```
   |||||||||||||||||||
   ACCUCUUCGUCCCAUGCACG 5'
AT1G56010.1     777     796
NAC1

sRNA_AGO1_Solexa_Mi2008_9_40189_hit2
5' UGGAGAAGCAGGGCACGUA
   ||||||||||||| ||||
   ACCUCUUCGUCCCAUGCAC 5'
AT1G56010.1     778     796
NAC1

sRNA_AGO1_Solexa_Mi2008_366_40193_hit3
5' UGGAGAAGCAGGGCACGUG
   ||||||||||||| |||||
   ACCUCUUCGUCCCAUGCAC 5'
AT1G56010.1     778     796
NAC1

sRNA_AGO1_Solexa_Mi2008_1_40200_hit1
5' UGGAGAAGCAGGGUACGUG
   |||||||||||||||||||
   ACCUCUUCGUCCCAUGCAC 5'
AT1G56010.1     778     796
NAC1

sRNA_AGO1_Solexa_Mi2008_2_3231_hit2
5' AUGGAGAAGCAGGGCACGUG
    ||||||||||||| |||||
   AACCUCUUCGUCCCAUGCAC 5'
AT1G56010.1     778     797
NAC1

sRNA_AGO1_Solexa_Mi2008_1_13477_hit1
5' GAAGCAGGGCACGUG--CAUU
   ||||||||| |||||  ||||
   CUUCGUCCCAUGCACGAGUAA 5'
AT1G56010.2     798     818
NAC1

sRNA_AGO1_Solexa_Mi2008_2_14844_hit1
5' GGAGAAGCAGGGCACGUG--CAUU
   |||||||||||| |||||  ||||
   CCUCUUCGUCCCAUGCACGAGUAA 5'
AT1G56010.2     798     821
NAC1

sRNA_AGO1_Solexa_Mi2008_91_40198_hit1
5' UGGAGAAGCAGGGCACGUG--CAUU
   ||||||||||||| |||||  ||||
   ACCUCUUCGUCCCAUGCACGAGUAA 5'
AT1G56010.2     798     822
NAC1

sRNA_AGO1_Solexa_Mi2008_2_13910_hit1
5' GAGAAGCAGGGCACGUGCAU
   ||||||||||| |||||| :
   CUCUUCGUCCCAUGCACGAG 5'
AT1G56010.2     800     819
NAC1

sRNA_AGO1_Solexa_Mi2008_1_14842_hit1
5' GGAGAAGCAGGGCACGUGCAA
```

```
   |||||||||||| ||||||
   CCUCUUCGUCCCAUGCACGAG 5'
AT1G56010.2     800     820
NAC1

sRNA_AGO1_Solexa_Mi2008_4_14843_hit1
5' GGAGAAGCAGGGCACGUGCAU
   |||||||||||| |||||| :
   CCUCUUCGUCCCAUGCACGAG 5'
AT1G56010.2     800     820
NAC1

sRNA_AGO1_Solexa_Mi2008_1_14846_hit1
5' GGAGAAGCAGGGCACGUGCGA
   |||||||||||| ||||||
   CCUCUUCGUCCCAUGCACGAG 5'
AT1G56010.2     800     820
NAC1

sRNA_AGO1_Solexa_Mi2008_3_40196_hit1
5' UGGAGAAGCAGGGCACGUGCAA
   ||||||||||||| ||||||
   ACCUCUUCGUCCCAUGCACGAG 5'
AT1G56010.2     800     821
NAC1

sRNA_AGO1_Solexa_Mi2008_284_40197_hit1
5' UGGAGAAGCAGGGCACGUGCAU
   ||||||||||||| |||||| :
   ACCUCUUCGUCCCAUGCACGAG 5'
AT1G56010.2     800     821
NAC1

sRNA_AGO1_Solexa_Mi2008_10440_13909_hit2
5' GAGAAGCAGGGCACGUGCA
   ||||||||||| ||||||
   CUCUUCGUCCCAUGCACGA 5'
AT1G56010.2     801     819
NAC1

sRNA_AGO1_Solexa_Mi2008_110_13911_hit1
5' GAGAAGCAGGGCACGUGCG
   ||||||||||| ||||||
   CUCUUCGUCCCAUGCACGA 5'
AT1G56010.2     801     819
NAC1

sRNA_AGO1_Solexa_Mi2008_41322_14841_hit2
5' GGAGAAGCAGGGCACGUGCA
   |||||||||||| ||||||
   CCUCUUCGUCCCAUGCACGA 5'
AT1G56010.2     801     820
NAC1

sRNA_AGO1_Solexa_Mi2008_422_14845_hit1
5' GGAGAAGCAGGGCACGUGCG
   |||||||||||| ||||||
   CCUCUUCGUCCCAUGCACGA 5'
AT1G56010.2     801     820
NAC1

sRNA_AGO1_Solexa_Mi2008_370931_40195_hit2
5' UGGAGAAGCAGGGCACGUGCA
```

```
   ||||||||||||| ||||||
   ACCUCUUCGUCCCAUGCACGA 5'
AT1G56010.2     801     821
NAC1

sRNA_AGO1_Solexa_Mi2008_7589_40199_hit1
5' UGGAGAAGCAGGGCACGUGCG
   ||||||||||||| ||||||
   ACCUCUUCGUCCCAUGCACGA 5'
AT1G56010.2     801     821
NAC1

sRNA_AGO1_Solexa_Mi2008_2_40202_hit1
5' UGGAGAAGCAGGGUACGUGCU
   ||||||||||||||||||||
   ACCUCUUCGUCCCAUGCACGA 5'
AT1G56010.2     801     821
NAC1

sRNA_AGO1_Solexa_Mi2008_1214_3232_hit1
5' AUGGAGAAGCAGGGCACGUGCA
    ||||||||||||| ||||||
   AACCUCUUCGUCCCAUGCACGA 5'
AT1G56010.2     801     822
NAC1

sRNA_AGO1_Solexa_Mi2008_24_3233_hit1
5' AUGGAGAAGCAGGGCACGUGCG
    ||||||||||||| ||||||
   AACCUCUUCGUCCCAUGCACGA 5'
AT1G56010.2     801     822
NAC1

sRNA_AGO1_Solexa_Mi2008_9_52081_hit1
5' UUGGAGAAGCAGGGCACGUGCA
   |||||||||||||| ||||||
   AACCUCUUCGUCCCAUGCACGA 5'
AT1G56010.2     801     822
NAC1

sRNA_AGO1_Solexa_Mi2008_23_14840_hit3
5' GGAGAAGCAGGGCACGUGC
   |||||||||||| ||||||
   CCUCUUCGUCCCAUGCACG 5'
AT1G56010.2     802     820
NAC1

sRNA_AGO1_Solexa_Mi2008_1_40190_hit1
5' UGGAGAAGCAGGGCACGUAA
   ||||||||||||| ||||
   ACCUCUUCGUCCCAUGCACG 5'
AT1G56010.2     802     821
NAC1

sRNA_AGO1_Solexa_Mi2008_404_40194_hit3
5' UGGAGAAGCAGGGCACGUGC
   ||||||||||||| ||||||
   ACCUCUUCGUCCCAUGCACG 5'
AT1G56010.2     802     821
NAC1

sRNA_AGO1_Solexa_Mi2008_2_40201_hit1
5' UGGAGAAGCAGGGUACGUGC
```

```
   ||||||||||||||||||||
   ACCUCUUCGUCCCAUGCACG 5'
AT1G56010.2     802     821
NAC1

sRNA_AGO1_Solexa_Mi2008_9_40189_hit2
5' UGGAGAAGCAGGGCACGUA
   ||||||||||||| ||||
   ACCUCUUCGUCCCAUGCAC 5'
AT1G56010.2     803     821
NAC1

sRNA_AGO1_Solexa_Mi2008_366_40193_hit3
5' UGGAGAAGCAGGGCACGUG
   ||||||||||||| |||||
   ACCUCUUCGUCCCAUGCAC 5'
AT1G56010.2     803     821
NAC1

sRNA_AGO1_Solexa_Mi2008_1_40200_hit1
5' UGGAGAAGCAGGGUACGUG
   |||||||||||||||||||
   ACCUCUUCGUCCCAUGCAC 5'
AT1G56010.2     803     821
NAC1

sRNA_AGO1_Solexa_Mi2008_2_3231_hit2
5' AUGGAGAAGCAGGGCACGUG
    ||||||||||||| |||||
   AACCUCUUCGUCCCAUGCAC 5'
AT1G56010.2     803     822
NAC1

sRNA_AGO1_Solexa_Mi2008_1_48023_hit1
5' UU-AG-UUCAAGUUGGGUAAA
   || || |||||||| ||||||
   AACUCUAAGUUCAAACCAUUU 5'
AT1G56140.1     1224    1244
receptor-like protein kinase, putative

sRNA_AGO1_Solexa_Mi2008_1_5211_hit1
5' CAA-UUAAGUUUGAAAAUGACU
   ||| |||||||||||||:|||
   GUUCAAUUCAAACUUUUGCUGC 5'
AT1G56140.1     863     884
receptor-like protein kinase, putative

sRNA_AGO1_Solexa_Mi2008_1_30079_hit1
5' UCCGUGCCGCAACAACGUUCG
   |||||||||||||| || |||
   AGGCACGGCGUUGUGGC-AGC 5'
AT1G56230.1     113     132
hypothetical protein

sRNA_AGO1_Solexa_Mi2008_1_3712_hit1
5' AUUGCUAUUUCAGUUGGAUCUU
    ||||| |||||||||||||||
   CAACGAAAAAGUCAACCUAGAA 5'
AT1G56230.1     1950    1971
hypothetical protein

sRNA_AGO1_Solexa_Mi2008_3_6705_hit1
5' CAGUCUCAGGUGAAUCAG-AU
```

```
   ||||||||:||||||||| ||
   GUCAGAGUUCACUUAGUCCUA 5'
AT1G56230.1     544     564
hypothetical protein

sRNA_AGO1_Solexa_Mi2008_1_1445_hit1
5' ACAGUCUCAGGUGAAUCAG-AU
   |||||||||:||||||||| ||
   UGUCAGAGUUCACUUAGUCCUA 5'
AT1G56230.1     544     565
hypothetical protein

sRNA_AGO1_Solexa_Mi2008_1_4378_hit1
5' CAACAGUCUCAGGUGAAUCAG
    ||||||||||:|||||||||
   UUUGUCAGAGUUCACUUAGUC 5'
AT1G56230.1     546     566
hypothetical protein

sRNA_AGO1_Solexa_Mi2008_1_49627_hit1
5' UUCGUUCGGAGCCGCACUUUA
   ||| ||||||| ||||||||
   AAG-AAGCCUCUGCGUGAAAC 5'
AT1G57620.1     246     265
unknown protein

sRNA_AGO1_Solexa_Mi2008_1_31526_hit1
5' UCGCGGACCACGGGACCAUUUU
   ||||||||||||||||||
   AGCGCCUGGUGCCCUGGUA___ 5'
AT1G57650.1     -3      18
null

sRNA_AGO1_Solexa_Mi2008_1_40990_hit1
5' UGGAUUAAAGAAGGUGUUGUU
   ||||||||||||||||||||
   ACCUAAUUUCUUCCACAACAA 5'
AT1G58030.1     37      57

sRNA_AGO1_Solexa_Mi2008_7_14756_hit2
5' GCUACCUU-AAGAGAGUCA-UAG
   | |||||| |||||||||| |||
   CCAUGGAAAUUCUCUCAGUUAUC 5'
AT1G58340.1     972     994
putative protein

sRNA_AGO1_Solexa_Mi2008_2_31038_hit1
5' UCGAGUGGUGAUCGUGUGGUUC
   |||||||:||||| |||||||
   AGCUCACUACUAGAACACCAAA 5'
AT1G59590.1     79      100
unknown protein

sRNA_AGO1_Solexa_Mi2008_4_31037_hit1
5' UCGAGUGGUGAUCGUGUGGUU
   |||||||:||||| |||||||
   AGCUCACUACUAGAACACCAA 5'
AT1G59590.1     80      100
unknown protein

sRNA_AGO1_Solexa_Mi2008_3_7132_hit1
5' CAUGAGCAGCUUG-AU-AUGGU
   ||||||||||||| || ||| |
```

```
   GUACUCGUCGAACUUAGUACGA 5'
AT1G59820.1     2822    2843
hypothetical protein

sRNA_AGO1_Solexa_Mi2008_1_1474_hit1
5' ACAUGAGCAGCUUG-AU-AUGGU
   ||||||||||||| || ||| |
   UGUACUCGUCGAACUUAGUACGA 5'
AT1G59820.1     2822    2844
hypothetical protein

sRNA_AGO1_Solexa_Mi2008_1_35436_hit1
5' UGACCGUGGUACCUGUAUGUG
   |||||||||||||||||||||
   ACUGGCACCAUGGACAUACAC 5'
AT1G59900.1     924     944
pyruvate dehydrogenase E1 alpha subunit, putative

sRNA_AGO1_Solexa_Mi2008_1_3401_hit1
5' AUGGUGGUGUCUUCAGC-CUCUU
   ||||||||||| |||||  ||||
   UACCACCACAG-AGUCGCAAGAA 5'
AT1G60710.1     1128    1149
unknown protein

sRNA_AGO1_Solexa_Mi2008_1_13420_hit2
5' GAAGAAGAAGAAGACUCUU
   |||||||| ||||||| ||
   CUUCUUCUGCUUCUGA-AA 5'
AT1G61250.1     65      82
unknown protein

sRNA_AGO1_Solexa_Mi2008_1_55863_hit1
5' UUUGGGUGGCGAUUUGUGGUGUA
   :||| |||| ||||||||||||
   GAACACACCCCUAAACACCACAU 5'
AT1G61380.1     671     693
S-like receptor protein kinase

sRNA_AGO1_Solexa_Mi2008_2_40937_hit1
5' UG-GAUGGCGAUUUGUGGUGUA
   || | ||| ||||||||||||
   ACAC-ACCCCUAAACACCACAU 5'
AT1G61380.1     672     692
S-like receptor protein kinase

sRNA_AGO1_Solexa_Mi2008_1_34051_hit1
5' UGA-AAAAAAAAGAGAGGGAC
   ||| ||||||||||||||
   ACUGUUUUUUUCUCUCCCAU 5'
AT1G61730.1     1323    1343
unknown protein

sRNA_AGO1_Solexa_Mi2008_1_11311_hit1
5' CUGA-AAAAAAAAGAGAGGG
    ||| ||||||||||||||
   AACUGUUUUUUUCUCUCCC 5'
AT1G61730.1     1325    1344
unknown protein

sRNA_AGO1_Solexa_Mi2008_1_35311_hit3
5' UGAC-AUGAUGUUGAUGUGCAUA
   |||| ||||||||||||| ||
```

```
   ACUGGUACUACAACUACAC-UAC 5'
AT1G62360.1     104     125
homeobox transcription factor shootmeristemless (stm)

sRNA_AGO1_Solexa_Mi2008_1_35310_hit3
5' UGAC-AUGAUGUUGAUGUGCA
   |||| |||||||||||||| 
   ACUGGUACUACAACUACACUA 5'
AT1G62360.1     105     125
homeobox transcription factor shootmeristemless (stm)

sRNA_AGO1_Solexa_Mi2008_1_2990_hit3
5' AUGAC-AUGAUGUUGAUGUGCAU
   ||||| ||||||||||||| ||
   UACUGGUACUACAACUACAC-UA 5'
AT1G62360.1     105     126
homeobox transcription factor shootmeristemless (stm)

sRNA_AGO1_Solexa_Mi2008_2_13419_hit2
5' GAAGAAGAAGAAG-ACACUU
   ||||||||||||| | ||||
   CUUCUUCUUCUUCGU-UGAA 5'
AT1G62360.1     239     257
homeobox transcription factor shootmeristemless (stm)

sRNA_AGO1_Solexa_Mi2008_1_3891_hit1
5' CAAAACCAGAAGGAUGAUAAA
   |||||| |||||||||| |||
   GUUUUGCUCUUCCUACU-UUU 5'
AT1G62570.1     1421    1440
similar to glutamate synthase

sRNA_AGO1_Solexa_Mi2008_30_14853_hit1
5' GGAGAGAGCAAAGUUGAGA
   | ||||||||||||| |||
   CGUCUCUCGUUUCAAAUCU 5'
AT1G63010.1     190     208
tetracycline resistance efflux protein like protein

sRNA_AGO1_Solexa_Mi2008_30_14853_hit1
5' GGAGAGAGCAAAGUUGAGA
   | ||||||||||||| |||
   CGUCUCUCGUUUCAAAUCU 5'
AT1G63010.4     310     328
tetracycline resistance efflux protein like protein

sRNA_AGO1_Solexa_Mi2008_30_14853_hit1
5' GGAGAGAGCAAAGUUGAGA
   | ||||||||||||| |||
   CGUCUCUCGUUUCAAAUCU 5'
AT1G63010.2     409     427
tetracycline resistance efflux protein like protein

sRNA_AGO1_Solexa_Mi2008_30_14853_hit1
5' GGAGAGAGCAAAGUUGAGA
   | ||||||||||||| |||
   CGUCUCUCGUUUCAAAUCU 5'
AT1G63010.3     410     428
tetracycline resistance efflux protein like protein

sRNA_AGO1_Solexa_Mi2008_1_50002_hit1
5' UUGAACAUUGUGGUG-UGAGUG
   ||||||||||||| | |||||
```

```
   AACUUGUAACACCUCUACUCAA 5'
AT1G63840.1     399     420
putative RING zinc finger protein

sRNA_AGO1_Solexa_Mi2008_1_3384_hit1
5' AUGGUGAAGAUGUUUCUAGU
    |||||| |||||||||||
   CACCACUACUACAAAGAUCU 5'
AT1G63840.1     73      92
putative RING zinc finger protein

sRNA_AGO1_Solexa_Mi2008_10_12740_hit16
5' CUUGACACGUGGCACGAUC
      ||||||||||||||||
   CUCCUGUGCACCGUGCUAG 5'
AT1G64065.1     146     164

sRNA_AGO1_Solexa_Mi2008_2_8245_hit1
5' CGAA-AAGAAGAAGAUGAUGAG
   |||| |||||||||| ||||||
   GCUUCUUCUUCUUCUUCUACUC 5'
AT1G64065.1     229     250

sRNA_AGO1_Solexa_Mi2008_1_27754_hit1
5' UCACCUCAUGAUGAACUUGGA
    |||| | |||||||||||||
   CGUGGUG-ACUACUUGAACCU 5'
AT1G64660.1     798     817
similar to O-succinylhomoserine sulfhydrylase

sRNA_AGO1_Solexa_Mi2008_1_16704_hit12
5' UAAAGAGGGUGCUGCAA-AGUU
   | ||||||||||||| | ||||
   AGUUCUCCCACGACGAUCUCAA 5'
AT1G64970.1     125     146
unknown protein

sRNA_AGO1_Solexa_Mi2008_1_13420_hit2
5' GAAGAAGAAGAAGACUCUU
   ||||||||||||| || ||
   CUUCUUCUUCUUC-GA-AA 5'
AT1G65690.1     14      30
unknown protein

sRNA_AGO1_Solexa_Mi2008_2_13419_hit2
5' GAAGAAGAAGAAGACA-CUU
   |||||||||||||| | |||
   CUUCUUCUUCUUCU-UCGAA 5'
AT1G65690.1     15      33
unknown protein

sRNA_AGO1_Solexa_Mi2008_3_13421_hit1
5' GAAGAAGAAGAUGAUGUUGAU
   :||||||||||||||| |||
   UUUCUUCUUCUACUACUACUU 5'
AT1G66090.1     101     121
unknown protein

sRNA_AGO1_Solexa_Mi2008_6_29484_hit1
5' UCCAGGACCAAACCACGUUGU
   |||||||||| ||||| |||
   AGGUCCUGGUAUGGUG-AACU 5'
AT1G66090.1     1049    1068
```

```
unknown protein

sRNA_AGO1_Solexa_Mi2008_1_48999_hit1
5' UUCCAGGACCAAACCACGUUG
   ||||||||||| ||||| |||
   AAGGUCCUGGUAUGGUG-AAC 5'
AT1G66090.1     1050    1069
unknown protein

sRNA_AGO1_Solexa_Mi2008_1_1763_hit2
5' ACUUCCAGGACCAAACCACGU
   ||||||||||||| ||||| |
   UGAAGGUCCUGGUAUGGUGAA 5'
AT1G66090.1     1050    1070
unknown protein

sRNA_AGO1_Solexa_Mi2008_26_20692_hit2
5' UACUUCCAGGACCAAACCACGU
   |||||||||||||| ||||| |
   AUGAAGGUCCUGGUAUGGUGAA 5'
AT1G66090.1     1050    1071
unknown protein

sRNA_AGO1_Solexa_Mi2008_12_20691_hit2
5' UACUUCCAGGACCAAACCACG
   |||||||||||||| |||||
   AUGAAGGUCCUGGUAUGGUGA 5'
AT1G66090.1     1051    1071
unknown protein

sRNA_AGO1_Solexa_Mi2008_2_8245_hit1
5' CGA-AAAGAAGAAGAUGAUGAG
    || ||||||||||||||||
   ACUAUUUCUUCUUCUACUACUA 5'
AT1G66090.1     105     126
unknown protein

sRNA_AGO1_Solexa_Mi2008_3_27133_hit1
5' UCAAG-CACCAGCUCGAAGAAGC
   ||||| ||||||||||| |||||
   AGUUCUGUGGUCGAGCUACUUCG 5'
AT1G66090.1     346     368
unknown protein

sRNA_AGO1_Solexa_Mi2008_1_27132_hit1
5' UCAAG-CACCAGCUCGAAGAAG
   ||||| ||||||||||| ||||
   AGUUCUGUGGUCGAGCUACUUC 5'
AT1G66090.1     347     368
unknown protein

sRNA_AGO1_Solexa_Mi2008_3_39171_hit1
5' UGCUAGAGAGUAGGCUU-UGU
   |||||||||||||| || |||
   ACGAUCUCUCAUCC-AAUACA 5'
AT1G66090.1     795     814
unknown protein

sRNA_AGO1_Solexa_Mi2008_1_34620_hit1
5' UGAAGAUGAUGAUGAAGUU
    ||||||||||||||||
   UCUUCUACUACUACUUCUU 5'
AT1G66090.1     98      116
```

```
unknown protein

sRNA_AGO1_Solexa_Mi2008_2_11307_hit2
5' CUCUUUGAACCAUUUGAU-CA
    |||||||||||||| || ||
   AAGAAACUUGGUAAA-UACGU 5'
AT1G66860.1     618     637
unknown protein

sRNA_AGO1_Solexa_Mi2008_1_46069_hit17
5' UGUUUAAGUGUUUCAG--GUUUG
   ||||||||||||| ||  |||||
   ACAAAUUCACAAAAUCUGCAAAC 5'
AT1G66880.1     220     242
wall-associated kinase like protein

sRNA_AGO1_Solexa_Mi2008_1_45181_hit3
5' UGUGCGGUUGAUAU-UGUCGUU
   :||||||||||||| ||| |||
   GCACGCCAACUAUACACA-CAA 5'
AT1G66880.1     3360    3380
wall-associated kinase like protein

sRNA_AGO1_Solexa_Mi2008_1_6424_hit2
5' CAG-GAAGCUCUGGCAAU-GAU
   ||| ||||||||:||||| |||
   GUCGCUUCGAGAUCGUUACCUA 5'
AT1G66880.1     710     731
wall-associated kinase like protein

sRNA_AGO1_Solexa_Mi2008_1_45073_hit1
5' UGUGAUUGGUGAAUGCAAGGG
   |||| |||||||||| |||||
   ACAC-AACCACUUACAUUCCC 5'
AT1G66880.1     851     870
wall-associated kinase like protein

sRNA_AGO1_Solexa_Mi2008_1_2_hit38
5' AAAAAAAAAA-AAAAACCAU
   |||||||||| ||||||  |
   UUUUUUUUUUCUUUUUGUCA 5'
AT1G66940.3     991     1010
unknown protein

sRNA_AGO1_Solexa_Mi2008_1_341_hit1
5' AAAGAAACAGAGAGGAAGAU
   ||| ||||||||||| |||
   UUU-UUUGUCUCUCC-UCUU 5'
AT1G66970.1     2364    2381
Glycerophosphodiesterase-like (GPDL3)

sRNA_AGO1_Solexa_Mi2008_2_13419_hit2
5' GAAGAAGAAGAAGACACUU
   ||||||||||||||| :
   CUUCUUCUUCUUCUGUUGC 5'
AT1G67050.1     712     730
unknown protein

sRNA_AGO1_Solexa_Mi2008_3_330_hit1
5' AAACUCAGAAGAACAUUCU
   |||||||||||||| || :
   UUUGAGUCUUCUUG-AACG 5'
AT1G67310.1     646     663
```

```
Calmodulin-binding transcription activator 4 (CAMTA4)

sRNA_AGO1_Solexa_Mi2008_1_11311_hit1
5' CUGAA-AAAAAAAGAGAGGG
    |||| |||||||||||||
   UACUUCUUUUUUUCUCUCCA 5'
AT1G67360.2     1026    1045
unknown protein

sRNA_AGO1_Solexa_Mi2008_3_492_hit1
5' AAAGGACCAGGACAGAAUCAA
   |||| ||||||| |||||||
   UUUC-UGGUCCUCUCUUAGUC 5'
AT1G67360.1     332     351
unknown protein

sRNA_AGO1_Solexa_Mi2008_3_492_hit1
5' AAAGGACCAGGACAGAAUCAA
   |||| ||||||| |||||||
   UUUC-UGGUCCUCUCUUAGUC 5'
AT1G67360.2     430     449
unknown protein

sRNA_AGO1_Solexa_Mi2008_1_11311_hit1
5' CUGAA-AAAAAAAGAGAGGG
    |||| |||||||||||||
   UACUUCUUUUUUUCUCUCCA 5'
AT1G67360.1     928     947
unknown protein

sRNA_AGO1_Solexa_Mi2008_1_21468_hit1
5' UAGAGGUAUACAUCGGAGAUGGUC
   ||||| ||||||| |||||| |||
   AUCUCGAUAUGUAACCUCUAACAG 5'
AT1G67580.1     2956    2979
putative protein kinase

sRNA_AGO1_Solexa_Mi2008_1_1919_hit6
5' AGAGCUAUACAUUGGAGAUGGU
   ||||||||||||||||||| ||
   UCUCGAUAUGUAACCUCUAACA 5'
AT1G67580.1     2957    2978
putative protein kinase

sRNA_AGO1_Solexa_Mi2008_1_21332_hit1
5' UAGAGCUAUACAUUGGAGA-UGAUC
   ||||||||||||||||||| || ||
   AUCUCGAUAUGUAACCUCUAAC-AG 5'
AT1G67580.1     2957    2980
putative protein kinase

sRNA_AGO1_Solexa_Mi2008_1_54254_hit3
5' UUUAGAGGUG-GAUAUAGAGCUAUA
   ||||||| |: ||||||||||||||
   AAAUCUC-AUUCUAUAUCUCGAUAU 5'
AT1G67580.1     2971    2994
putative protein kinase

sRNA_AGO1_Solexa_Mi2008_1_54253_hit3
5' UUUAGAGGUG-GAUAUAGAGCUA
   ||||||| |: ||||||||||||
   AAAUCUC-AUUCUAUAUCUCGAU 5'
AT1G67580.1     2973    2994
```

putative protein kinase

```
sRNA_AGO1_Solexa_Mi2008_1_820_hit13
5' AAGAUAAAUAGAAAU-GAAU
   | :||||||||||| ||||
   UAUUAUUUAUCUUUAUCUUA 5'
AT1G67580.1     3061    3080
putative protein kinase

sRNA_AGO1_Solexa_Mi2008_8_8512_hit2
5' CGAGAGGAACCG-UUGAUUC
    ||||||||||| || ||||
   UCUCUCCUUGGCCAA-UAAG 5'
AT1G67800.4     1510    1528
unknown protein

sRNA_AGO1_Solexa_Mi2008_8_8512_hit2
5' CGAGAGGAACC-GUUGAUUC
    |||||||||| ||| ||||
   UCUCUCCUUGGCCAA-UAAG 5'
AT1G67800.2     1585    1603
unknown protein

sRNA_AGO1_Solexa_Mi2008_8_8512_hit2
5' CGAGAGGAACC-GUUGAUUC
    |||||||||| ||| ||||
   UCUCUCCUUGGCCAA-UAAG 5'
AT1G67800.1     1723    1741
unknown protein

sRNA_AGO1_Solexa_Mi2008_2_21192_hit1
5' UAGAGACAAAA-ACAACACAGA
   ||||||||||| | ||| ||||
   AUCUCUGUUUUGU-UUGGGUCU 5'
AT1G67850.2     173     193
At1g67850/F12A21_2

sRNA_AGO1_Solexa_Mi2008_2_21192_hit1
5' UAGAGACAAAA-ACAACACAGA
   ||||||||||| | ||| ||||
   AUCUCUGUUUUGU-UUGGGUCU 5'
AT1G67850.1     187     207
At1g67850/F12A21_2

sRNA_AGO1_Solexa_Mi2008_2_47860_hit1
5' UUAGGU-C-CCAUGAACUCCGCA
   |||||| | |||||||||||||
   AAUCCAAGAGGUACUUGAGGCGU 5'
AT1G68390.1     1184    1206
hypothetical protein

sRNA_AGO1_Solexa_Mi2008_7_14062_hit1
5' GAGGAGGAGGAGGUGAACA
   ||||||||||||| |||
   CUCCUCCUCCUCCUCUUCC 5'
AT1G68390.1     341     359
hypothetical protein

sRNA_AGO1_Solexa_Mi2008_1_11574_hit1
5' CUG-AUCGGCCGCAUCCAA-GCC
   ||| ||||||||| ||||| |||
   GACAUAGCCGGCGCAGGUUGCGG 5'
AT1G68390.1     879     901
```

```
hypothetical protein

sRNA_AGO1_Solexa_Mi2008_1_2360_hit1
5' AUAACAAGUCCUGUUGGGUC
   | |||||||||| |||||||
   UCUUGUUCAGGAAAACCCAG 5'
AT1G68550.1     319     338
putative AP2 domain transcription factor

sRNA_AGO1_Solexa_Mi2008_1_2360_hit1
5' AUAACAAGUCCUGUUGGGUC
   | |||||||||| |||||||
   UCUUGUUCAGGAAAACCCAG 5'
AT1G68550.2     329     348
putative AP2 domain transcription factor

sRNA_AGO1_Solexa_Mi2008_2_50219_hit1
5' UUGACAACUGCAAUGUGAUCCUUA
   |||||||||||||| |  ||||||
   AACUGUUGACGUUA-AGGAGGAAU 5'
AT1G68570.1     1821    1843
peptide transporter like

sRNA_AGO1_Solexa_Mi2008_1_559_hit1
5' AAAGUCAAACAACACAUGGUCU
   |||| ||| ||||||||||||
   UUUC-GUU-GUUGUGUACCAGC 5'
AT1G68570.1     547     566
peptide transporter like

sRNA_AGO1_Solexa_Mi2008_1_20838_hit1
5' UAGAACCAAUUAUUUAUUGAA
   || ||| |||||||||||||
   AU-UUGCUUAAUAAAUAACUA 5'
AT1G68620.1     1274    1293
putative carboxylesterase

sRNA_AGO1_Solexa_Mi2008_1_4194_hit1
5' CAAAGGUGUG-AUCAGGAUGGU
   ||||||| || |||||||||||
   GUUUCCA-ACCUAGUCCUACCA 5'
AT1G68620.1     675     695
putative carboxylesterase

sRNA_AGO1_Solexa_Mi2008_1_18801_hit1
5' UAAUGGAGUUG-AUCACAUGA
   ||||||||| | |||||||||
   AUUACCUCA-CGUAGUGUACU 5'
AT1G68875.1     301     320

sRNA_AGO1_Solexa_Mi2008_4_21672_hit1
5' UAGAGUUGGGUAUGAUAGCUU
   :||||||||||||| || |||
   GUCUCAACCCAUAC-AU-GAA 5'
AT1G69180.1     582     600
transcription factor CRC

sRNA_AGO1_Solexa_Mi2008_1_35840_hit2
5' UGAGACAAUGGUUUG-C-AUA
   ||||||||||||||| | ||
   ACUCUGUUACCAAACCGGUAG 5'
AT1G69530.2     783     803
expansin-like protein (AtEXPA1)
```

```
sRNA_AGO1_Solexa_Mi2008_1_35840_hit2
5' UGAGACAAUGGUUU-G-CAUA
   |||||||||||||| | |||
   ACUCUGUUACCAAACCGGUAG 5'
AT1G69530.4     868     888
expansin-like protein (AtEXPA1)

sRNA_AGO1_Solexa_Mi2008_2_53124_hit14
5' UUGGUGUGUUUUCAGG-UGUA-GA
    ||||||||||||||| |||| ||
   CACCACACAAAAGUCCUACAUACU 5'
AT1G69930.1     668     691
glutathione transferase like protein

sRNA_AGO1_Solexa_Mi2008_1_56587_hit16
5' UUUUGGUGUGUUUUCAGG-UGUA
   |  ||||||||||||||| ||||
   ACCACCACACAAAAGUCCUACAU 5'
AT1G69930.1     671     693
glutathione transferase like protein

sRNA_AGO1_Solexa_Mi2008_2_13419_hit2
5' GAAGAAGAAGAAGACAC-UU
   |||||||||| |||||| ||
   CUUCUUCUUC-UCUGUGCAA 5'
AT1G69930.1     990     1008
glutathione transferase like protein

sRNA_AGO1_Solexa_Mi2008_1_52644_hit1
5' UUGGGGAUGUUACAGUUUGUUUU
   ||||| ||||| |||||| ||||
   AACCC-UACAAAGUCAAA-AAAA 5'
AT1G70160.1     91      111
unknown protein

sRNA_AGO1_Solexa_Mi2008_1_45596_hit2
5' UGUGUACA-GGCUAAGAUCUGG
    ||||| | |||||||||||||
   CCACAU-UACCGAUUCUAGACC 5'
AT1G72070.1     206     226
hypothetical protein

sRNA_AGO1_Solexa_Mi2008_3_11483_hit1
5' CUGAGCGGUGGUCCAUUGGUC
   ||||||||||||||||||||
   GACUCGCCACCAGGUAACCAG 5'
AT1G72370.2     812     832
40S ribosomal protein SA (laminin receptor-like protein)

sRNA_AGO1_Solexa_Mi2008_1_20232_hit22
5' UACGGAUAAGCCAUCAGAGUG
   ||||| |||||||||| |||
   AUGCCAAUUCGGUAGU-UCAA 5'
AT1G72520.1     1523    1542
putative lipoxygenase

sRNA_AGO1_Solexa_Mi2008_11_14524_hit19
5' GCCAUUUGUUCUUGAGUAG
   ||||||||||||| | ||
   CGGUAAACAAGAA-U-AUG 5'
AT1G72520.1     2064    2080
putative lipoxygenase
```

```
sRNA_AGO1_Solexa_Mi2008_1_4981_hit1
5' CAAGUA-UUUAAGCCCAUUUA
   |||||| ||||||||||| |
   GUUCAUUAAAUUCGGGUACAC 5'
AT1G72520.1     2150    2170
putative lipoxygenase

sRNA_AGO1_Solexa_Mi2008_1_20416_hit2
5' UACGUUGAUGAUAUGGUCA
   || ||||||||||||||
   AU-CAACUACUAUACCAUC 5'
AT1G72520.1     63      80
putative lipoxygenase

sRNA_AGO1_Solexa_Mi2008_1_10750_hit1
5' CUC-AGUGCCUUCAUCUUCGUC
   ||| |||||||||||||  |||
   GAGAUCACGGAAGUAGAC-CAG 5'
AT1G72680.1     849     869
putative cinnamyl-alcohol dehydrogenase

sRNA_AGO1_Solexa_Mi2008_1_3_hit25
5' AAAAAA-A-AAAAAAGAAAGA
   |||||| | |||||||||||
   UUUUUUGUCUUUUUUCUUUCC 5'
AT1G72730.1     1428    1448
Eukaryotic initiation factor 4A like protein

sRNA_AGO1_Solexa_Mi2008_1_54673_hit1
5' UUUCAAGUG-GUCCAAACAUAUCAG
   ||||| ||| ||||||| |||||||
   AAAGU-CACACAGGUUUCUAUAGUC 5'
AT1G72830.3     1238    1261
unknown protein

sRNA_AGO1_Solexa_Mi2008_1_54673_hit1
5' UUUCAAGUG-GUCCAAACAUAUCAG
   ||||| ||| ||||||| |||||||
   AAAGU-CACACAGGUUUCUAUAGUC 5'
AT1G72830.1     1314    1337
unknown protein

sRNA_AGO1_Solexa_Mi2008_1_54673_hit1
5' UUUCAAGUG-GUCCAAACAUAUCAG
   ||||| ||| ||||||| |||||||
   AAAGU-CACACAGGUUUCUAUAGUC 5'
AT1G72830.2     1317    1340
unknown protein

sRNA_AGO1_Solexa_Mi2008_17_12252_hit1
5' CUGUGGUGGGAACAUGAACUC
   || |||||||||||||||| |
   GA-ACCACCCUUGUACUUGUG 5'
AT1G72830.3     1391    1410
unknown protein

sRNA_AGO1_Solexa_Mi2008_26_14509_hit4
5' GCCAAGGAUGACUUGCCGG
   |||||||||||||| ||
   CGGUUCCUACUGAA-GGGG 5'
AT1G72830.3     1413    1430
unknown protein
```

```
sRNA_AGO1_Solexa_Mi2008_4_2048_hit4
5' AGCCAAGGAUGACUUGCCGG
   ||||||||||||||| ||
   UCGGUUCCUACUGAA-GGGG 5'
AT1G72830.3     1413    1431
unknown protein

sRNA_AGO1_Solexa_Mi2008_400_22176_hit7
5' UAGCCAAGGAUGACUUGCCUG
   |||||||||||||||| ||:
   AUCGGUUCCUACUGAA-GGGG 5'
AT1G72830.3     1413    1432
unknown protein

sRNA_AGO1_Solexa_Mi2008_2_15465_hit6
5' GUAGCCAAGGAUGACUUGCCUG
   ||||||||||||||||| ||:
   CAUCGGUUCCUACUGAA-GGGG 5'
AT1G72830.3     1413    1433
unknown protein

sRNA_AGO1_Solexa_Mi2008_78_6282_hit3
5' CAGCCAAGGAUGACUUGCCG
    ||||||||||||||| ||
   UUCGGUUCCUACUGAA-GGG 5'
AT1G72830.3     1414    1432
unknown protein

sRNA_AGO1_Solexa_Mi2008_9_22175_hit7
5' UAGCCAAGGAUGACUUGCCU
   |||||||||||||||| ||:
   AUCGGUUCCUACUGAA-GGG 5'
AT1G72830.3     1414    1432
unknown protein

sRNA_AGO1_Solexa_Mi2008_20_6281_hit3
5' CAGCCAAGGAUGACUUGCC
    ||||||||||||||| ||
   UUCGGUUCCUACUGAA-GG 5'
AT1G72830.3     1415    1432
unknown protein

sRNA_AGO1_Solexa_Mi2008_7_22174_hit7
5' UAGCCAAGGAUGACUUGCC
   |||||||||||||||| ||
   AUCGGUUCCUACUGAA-GG 5'
AT1G72830.3     1415    1432
unknown protein

sRNA_AGO1_Solexa_Mi2008_1_28302_hit1
5' UCAGCCAAGGAUGACUUGCC
   : ||||||||||||||| ||
   GUUCGGUUCCUACUGAA-GG 5'
AT1G72830.3     1415    1433
unknown protein

sRNA_AGO1_Solexa_Mi2008_4_36086_hit4
5' UGAGCCAAGGAUGACUUGCC
   ::||||||||||||||| ||
   GUUCGGUUCCUACUGAA-GG 5'
AT1G72830.3     1415    1433
unknown protein
```

```
sRNA_AGO1_Solexa_Mi2008_17_12252_hit1
5' CUGUGGUGGGAACAUGAACUC
   || |||||||||||||||| |
   GA-ACCACCCUUGUACUUGUG 5'
AT1G72830.1     1467    1486
unknown protein

sRNA_AGO1_Solexa_Mi2008_17_12252_hit1
5' CUGUGGUGGGAACAUGAACUC
   || |||||||||||||||| |
   GA-ACCACCCUUGUACUUGUG 5'
AT1G72830.2     1470    1489
unknown protein

sRNA_AGO1_Solexa_Mi2008_26_14509_hit4
5' GCCAAGGAUGACUUGCCGG
   |||||||||||||| ||
   CGGUUCCUACUGAA-GGGG 5'
AT1G72830.1     1489    1506
unknown protein

sRNA_AGO1_Solexa_Mi2008_4_2048_hit4
5' AGCCAAGGAUGACUUGCCGG
   ||||||||||||||| ||
   UCGGUUCCUACUGAA-GGGG 5'
AT1G72830.1     1489    1507
unknown protein

sRNA_AGO1_Solexa_Mi2008_400_22176_hit7
5' UAGCCAAGGAUGACUUGCCUG
   |||||||||||||||| ||:
   AUCGGUUCCUACUGAA-GGGG 5'
AT1G72830.1     1489    1508
unknown protein

sRNA_AGO1_Solexa_Mi2008_2_15465_hit6
5' GUAGCCAAGGAUGACUUGCCUG
   ||||||||||||||||| ||:
   CAUCGGUUCCUACUGAA-GGGG 5'
AT1G72830.1     1489    1509
unknown protein

sRNA_AGO1_Solexa_Mi2008_78_6282_hit3
5' CAGCCAAGGAUGACUUGCCG
    ||||||||||||||| ||
   UUCGGUUCCUACUGAA-GGG 5'
AT1G72830.1     1490    1508
unknown protein

sRNA_AGO1_Solexa_Mi2008_9_22175_hit7
5' UAGCCAAGGAUGACUUGCCU
   |||||||||||||||| ||:
   AUCGGUUCCUACUGAA-GGG 5'
AT1G72830.1     1490    1508
unknown protein

sRNA_AGO1_Solexa_Mi2008_20_6281_hit3
5' CAGCCAAGGAUGACUUGCC
    ||||||||||||||| ||
   UUCGGUUCCUACUGAA-GG 5'
AT1G72830.1     1491    1508
unknown protein
```

```
sRNA_AGO1_Solexa_Mi2008_7_22174_hit7
5' UAGCCAAGGAUGACUUGCC
   |||||||||||||||| ||
   AUCGGUUCCUACUGAA-GG 5'
AT1G72830.1     1491    1508
unknown protein

sRNA_AGO1_Solexa_Mi2008_1_28302_hit1
5' UCAGCCAAGGAUGACUUGCC
   : ||||||||||||||| ||
   GUUCGGUUCCUACUGAA-GG 5'
AT1G72830.1     1491    1509
unknown protein

sRNA_AGO1_Solexa_Mi2008_4_36086_hit4
5' UGAGCCAAGGAUGACUUGCC
   ::||||||||||||||| ||
   GUUCGGUUCCUACUGAA-GG 5'
AT1G72830.1     1491    1509
unknown protein

sRNA_AGO1_Solexa_Mi2008_26_14509_hit4
5' GCCAAGGAUGACUUGCCGG
   |||||||||||||| ||
   CGGUUCCUACUGAA-GGGG 5'
AT1G72830.2     1492    1509
unknown protein

sRNA_AGO1_Solexa_Mi2008_4_2048_hit4
5' AGCCAAGGAUGACUUGCCGG
   ||||||||||||||| ||
   UCGGUUCCUACUGAA-GGGG 5'
AT1G72830.2     1492    1510
unknown protein

sRNA_AGO1_Solexa_Mi2008_400_22176_hit7
5' UAGCCAAGGAUGACUUGCCUG
   |||||||||||||||| ||:
   AUCGGUUCCUACUGAA-GGGG 5'
AT1G72830.2     1492    1511
unknown protein

sRNA_AGO1_Solexa_Mi2008_2_15465_hit6
5' GUAGCCAAGGAUGACUUGCCUG
   ||||||||||||||||| ||:
   CAUCGGUUCCUACUGAA-GGGG 5'
AT1G72830.2     1492    1512
unknown protein

sRNA_AGO1_Solexa_Mi2008_78_6282_hit3
5' CAGCCAAGGAUGACUUGCCG
    ||||||||||||||| ||
   UUCGGUUCCUACUGAA-GGG 5'
AT1G72830.2     1493    1511
unknown protein

sRNA_AGO1_Solexa_Mi2008_9_22175_hit7
5' UAGCCAAGGAUGACUUGCCU
   |||||||||||||||| ||:
   AUCGGUUCCUACUGAA-GGG 5'
AT1G72830.2     1493    1511
unknown protein
```

```
sRNA_AGO1_Solexa_Mi2008_20_6281_hit3
5' CAGCCAAGGAUGACUUGCC
    ||||||||||||||| ||
   UUCGGUUCCUACUGAA-GG 5'
AT1G72830.2     1494    1511
unknown protein

sRNA_AGO1_Solexa_Mi2008_7_22174_hit7
5' UAGCCAAGGAUGACUUGCC
   |||||||||||||||| ||
   AUCGGUUCCUACUGAA-GG 5'
AT1G72830.2     1494    1511
unknown protein

sRNA_AGO1_Solexa_Mi2008_1_28302_hit1
5' UCAGCCAAGGAUGACUUGCC
   : ||||||||||||||| ||
   GUUCGGUUCCUACUGAA-GG 5'
AT1G72830.2     1494    1512
unknown protein

sRNA_AGO1_Solexa_Mi2008_4_36086_hit4
5' UGAGCCAAGGAUGACUUGCC
   ::||||||||||||||| ||
   GUUCGGUUCCUACUGAA-GG 5'
AT1G72830.2     1494    1512
unknown protein

sRNA_AGO1_Solexa_Mi2008_5_24767_hit1
5' UAUCAAAAGAAUCAU-UGCACU
   ||||| ||||||||| | ||||
   AUAGUCUUCUUAGUAUA-GUGA 5'
AT1G72830.3     1579    1599
unknown protein

sRNA_AGO1_Solexa_Mi2008_5_24767_hit1
5' UAUCAAAAGAAUCAU-UGCACU
   ||||| ||||||||| | ||||
   AUAGUCUUCUUAGUAUA-GUGA 5'
AT1G72830.1     1655    1675
unknown protein

sRNA_AGO1_Solexa_Mi2008_5_24767_hit1
5' UAUCAAAAGAAUCAU-UGCACU
   ||||| ||||||||| | ||||
   AUAGUCUUCUUAGUAUA-GUGA 5'
AT1G72830.2     1658    1678
unknown protein

sRNA_AGO1_Solexa_Mi2008_1_2_hit38
5' AAAAAAAAAA-AAAAACCAU
   |||||||||| ||||| |||
   UUUUUUUUUUGUUUUU-GUA 5'
AT1G72940.1     1448    1466
unknown protein

sRNA_AGO1_Solexa_Mi2008_1_1763_hit2
5' ACUUCCAGGACCAAACCACGU
   |||||| |||||||||| |||
   UGAAGGGCCUGGUUUGG-GCA 5'
AT1G72940.1     930     949
unknown protein
```

```
sRNA_AGO1_Solexa_Mi2008_26_20692_hit2
5' UACUUCCAGGACCAAACCACGU
    |||||| |||||||||| |||
   CUGAAGGGCCUGGUUUGG-GCA 5'
AT1G72940.1     930     950
unknown protein

sRNA_AGO1_Solexa_Mi2008_12_20691_hit2
5' UACUUCCAGGACCAAACCACG
    |||||| |||||||||| ||
   CUGAAGGGCCUGGUUUGG-GC 5'
AT1G72940.1     931     950
unknown protein

sRNA_AGO1_Solexa_Mi2008_1_38353_hit4
5' UGCCAAGGAGUCGCAACUCCGA
   ||||| | |||||||| |||||
   ACGGU-C-UCAGCGUUCAGGCU 5'
AT1G73260.1     631     650
putative trypsin inhibitor (At1g73260)

sRNA_AGO1_Solexa_Mi2008_10_30419_hit1
5' UCGAACGCUUCACGGAUCUCUA
   ||||||||||||||||||||||
   AGCUUGCGAAGUGCCUAGAGAU 5'
AT1G73630.1     274     295
calmodulin like protein

sRNA_AGO1_Solexa_Mi2008_1_3_hit25
5' AAA-AAAAAAAAAAGAAAGA
   ||| |||||||||||||| |
   UUUCUUUUUUUUUUCUUUGU 5'
AT1G73630.1     612     631
calmodulin like protein

sRNA_AGO1_Solexa_Mi2008_1_3_hit25
5' AAAAAAA-AAAAAAGAAAGA
   ||||||| ||||||:|||||
   UUUUUUUCUUUUUUUUUUCU 5'
AT1G73630.1     616     635
calmodulin like protein

sRNA_AGO1_Solexa_Mi2008_1_3_hit25
5' AAAAAAAA-AAAAAGAAAGA
   |||||||| ||||| |||:|
   UUUUUUUUCUUUUU-UUUUU 5'
AT1G73630.1     618     636
calmodulin like protein

sRNA_AGO1_Solexa_Mi2008_1_3_hit25
5' AAAAAAAAA-AAAAGAAAGA
   ||||||||| |||| |||:|
   UUUUUUUUUCUUUU-UUUUU 5'
AT1G73630.1     619     637
calmodulin like protein

sRNA_AGO1_Solexa_Mi2008_1_1776_hit10
5' AGAAAAAAAAAA-AAAAAAAAU
    ||||||||||| ||||||||
   GCUUUUUUUUUUCUUUUUUUUU 5'
AT1G73630.1     619     640
calmodulin like protein
```

```
sRNA_AGO1_Solexa_Mi2008_1_3_hit25
5' AAAAAAAAAA-AAAGAAAGA
   |||||||||| ||| |||:|
   UUUUUUUUUUCUUU-UUUUU 5'
AT1G73630.1     620     638
calmodulin like protein

sRNA_AGO1_Solexa_Mi2008_10_14179_hit22
5' GAUAGGUGUGUAUGUGAGAAG
   ||||||||| |||||| |||
   CUAUCCACAAAUACAC-CUUG 5'
AT1G74210.1     1263    1282
glycerophosphodiester phosphodiesterase like protein

sRNA_AGO1_Solexa_Mi2008_16_36976_hit22
5' UGAUAGGUGUGUAUGUGAGAA
   |||||||||| |||||| |||
   ACUAUCCACAAAUACAC-CUU 5'
AT1G74210.1     1264    1283
glycerophosphodiester phosphodiesterase like protein

sRNA_AGO1_Solexa_Mi2008_4_36977_hit1
5' UGAUAGGUGUGUAUGUGGGAA
   |||||||||| ||||| ||||
   ACUAUCCACAAAUACA-CCUU 5'
AT1G74210.1     1264    1283
glycerophosphodiester phosphodiesterase like protein

sRNA_AGO1_Solexa_Mi2008_1_51006_hit22
5' UUGAUAGGUGUGUAUGUGAGAA
    |||||||||| |||||| |||
   CACUAUCCACAAAUACAC-CUU 5'
AT1G74210.1     1264    1284
glycerophosphodiester phosphodiesterase like protein

sRNA_AGO1_Solexa_Mi2008_1_51005_hit24
5' UUGAUAGGUGUGUAUGUGAGA
    |||||||||| |||||| ||
   CACUAUCCACAAAUACAC-CU 5'
AT1G74210.1     1265    1284
glycerophosphodiester phosphodiesterase like protein

sRNA_AGO1_Solexa_Mi2008_3_53844_hit1
5' UUGUUUCGUGGAGAAUA-AAU
   ||||||||| ||||||| |||
   AACAAAGCAGCUCUUAUGUUA 5'
AT1G74210.1     357     377
glycerophosphodiester phosphodiesterase like protein

sRNA_AGO1_Solexa_Mi2008_1_36898_hit1
5' UGAGUUGGAUGAUGAUGAUGA
   ||||| | |||||||||||||
   ACUCA-C-UACUACUACUACU 5'
AT1G74450.1     100     118
unknown protein

sRNA_AGO1_Solexa_Mi2008_1_3079_hit472
5' AUGAUGA-UGAUGAUGAUGAUGA
    ||| || |||||||||||||||
   GACU-CUCACUACUACUACUACU 5'
AT1G74450.1     100     121
unknown protein
```

```
sRNA_AGO1_Solexa_Mi2008_1_36323_hit13
5' UGAG-GA-UGAUGAUGAUGA
   |||| || |||||||||||||||
   ACUCUCUCACUACUACUACUACU 5'
AT1G74450.1     100     122
unknown protein

sRNA_AGO1_Solexa_Mi2008_1_14208_hit2
5' GAUCGAUAAACCUCUGCAUC
   ||||||||||||||| || |
   CUAGCUAUUUGGAGA-GUCG 5'
AT1G74450.1     376     394
unknown protein

sRNA_AGO1_Solexa_Mi2008_3_8704_hit1
5' CGAUCGAUAAACCUCUGCAU
    ||||||||||||||| ||
   ACUAGCUAUUUGGAGA-GUC 5'
AT1G74450.1     377     395
unknown protein

sRNA_AGO1_Solexa_Mi2008_2_21756_hit1
5' UAGAUCGAUAAACCUCUGCAU
   :|||||||||||||||| ||
   GUCUAGCUAUUUGGAGA-GUC 5'
AT1G74450.1     377     396
unknown protein

sRNA_AGO1_Solexa_Mi2008_1_37225_hit4
5' UGA-UGAUGAUGAUGAUGAAGAAG
   ||| |||||||||||||||| ||
   ACUCACUACUACUACUACUU-UUA 5'
AT1G74450.1     96      118
unknown protein

sRNA_AGO1_Solexa_Mi2008_1_2280_hit1
5' AGUGGAUGAUGAUGAUGAUG
   |||| ||||||||||||| :
   UCAC-UACUACUACUACUUU 5'
AT1G74450.1     98      116
unknown protein

sRNA_AGO1_Solexa_Mi2008_2_52_hit1
5' AAAAAAGAGAGAUACAGAGAU
   ||||||||||||||  |||||
   UUUUUUCUCUCUAU--CUCUA 5'
AT1G74470.1     1520    1538
geranylgeranyl reductase

sRNA_AGO1_Solexa_Mi2008_1_1240_hit2
5' ACAA-AUAUUUAUAUGAUAU
    ||| || ||||||||||||
   AGUUCUA-AAAUAUACUAUA 5'
AT1G74670.1     524     542
GAST1-like protein

sRNA_AGO1_Solexa_Mi2008_1_22575_hit1
5' UAGGAAAACAAGAAGAU-GAUGAAG
   |||||| ||||||| || |||||||
   AUCCUUGUGUUCUU-UAACUACUUC 5'
AT1G74790.1     154     177
Hedgehog-Interacting-Protein-like (HIPL1)
```

```
sRNA_AGO1_Solexa_Mi2008_1_39551_hit2
5' UGGAAA-AUCAGGUUUAGUGAU
   |||||| ||| |||||||||||
   ACCUUUAUAGCCCAAAUCACUA 5'
AT1G74790.1     1692    1713
Hedgehog-Interacting-Protein-like (HIPL1)

sRNA_AGO1_Solexa_Mi2008_2_116_hit3
5' AA-A-AAGAUAAGAGAGAUAG
   || | |||| |||||||||||
   UUCUGUUCUCUUCUCUCUAUC 5'
AT1G74790.1     25      45
Hedgehog-Interacting-Protein-like (HIPL1)

sRNA_AGO1_Solexa_Mi2008_15_6297_hit2
5' CA-GCCAUUUGGGUUCUGCCUA
   || |||||||||||| ||||||
   GUACGGUAAACCCAA-ACGGAU 5'
AT1G75080.2     1227    1247
unknown protein

sRNA_AGO1_Solexa_Mi2008_1_35248_hit1
5' UGACAGAAUCGGCGC-A-AAAUGA
   ||||||||||||| | | ||||||
   ACUGUCUUAGCCGAGUUCUUUACU 5'
AT1G75080.1     1539    1562
unknown protein

sRNA_AGO1_Solexa_Mi2008_1_21352_hit9
5' UAGAGGAAAUGUUGUU-GUUGGU
   ||||||| ||||||||  |||||
   AUCUCCUGUACAACAAUAAACCA 5'
AT1G75080.2     634     656
unknown protein

sRNA_AGO1_Solexa_Mi2008_1_1991_hit1
5' AGAUAUUAUAUA-A-AAUAGU
   |||||||||||| | || |||
   UCUAUAAUAUAUAUCUUUUCA 5'
AT1G75500.1     1591    1611
nodulin-like protein

sRNA_AGO1_Solexa_Mi2008_5_14242_hit1
5' GAUGA-GACGCAAGGCGAUGAU
   || || || |||||||||||||
   CU-CUUCUUCGUUCCGCUACUA 5'
AT1G75500.1     319     339
nodulin-like protein

sRNA_AGO1_Solexa_Mi2008_1_21095_hit1
5' UAGACGCCGGAGUUUGCUGGU
   |||||| ||||||||| ||||
   AUCUGCCGCCUCAAAC-ACCA 5'
AT1G75500.1     691     710
nodulin-like protein

sRNA_AGO1_Solexa_Mi2008_1_45719_hit1
5' UGUGUGUGUGUGU-UG-GUGUGU
   ||||||||||||| || ||||||
   ACACACACACACAAACACACACA 5'
AT1G75500.1     87      109
nodulin-like protein
```

```
sRNA_AGO1_Solexa_Mi2008_1_39825_hit1
5' UGGAAUAGAAGAGACCUG-G-UC
   |||||||||||||| ||| | ||
   ACCUUAUCUUCUCUUGACUCGAG 5'
AT1G75670.2     210     232
unknown protein

sRNA_AGO1_Solexa_Mi2008_6_14366_hit1
5' GAUGUUGAGGUAUUAUGAUGA
    ||||| || |||||||||||
   AUACAA-UC-AUAAUACUACU 5'
AT1G75670.2     816     834
unknown protein

sRNA_AGO1_Solexa_Mi2008_6_13422_hit1
5' GAAGAAGAAGAUGAUUAAGGA
   ||||||||||||  |||||||
   CUUCUUCUUCUA--AAUUCCU 5'
AT1G75800.1     80      98
thaumatin, putative

sRNA_AGO1_Solexa_Mi2008_1_27366_hit1
5' UCAAUAAGUCACGGCGU-CUCAUC
   |||| ||||||||||||  |||||
   AGUUCUUCAGUGCCGCACAAGUAG 5'
AT1G76130.1     496     519
alpha-amylase like protein

sRNA_AGO1_Solexa_Mi2008_3_779_hit1
5' AAGAAUAUAAGUGUAGUUA
   |||||| |||||||||||
   UUCUUACAUUCACAUCAAG 5'
AT1G76160.1     439     457
unknown protein

sRNA_AGO1_Solexa_Mi2008_1_44461_hit2
5' UGUCAAGAUGGUCCAACAUGA
   ||| ||||| |||||||||||
   ACA-UUCUAACAGGUUGUACU 5'
AT1G76160.1     872     891
unknown protein

sRNA_AGO1_Solexa_Mi2008_1_49672_hit1
5' UUCUACAGCAAAAAAUCUAUAA
   || |||||||||||| |||||
   AA-AUGUCGUUUUUUUGAUAUA 5'
AT1G76590.1     1135    1155
unknown protein

sRNA_AGO1_Solexa_Mi2008_3_4962_hit3
5' CAAGUAAUAACAUUUAACCU
   |||||||| |||||||||
   GUUCAUUAGUGUAAAUUGCC 5'
AT1G76590.1     773     792
unknown protein

sRNA_AGO1_Solexa_Mi2008_21_8641_hit2
5' CGAGUCAGCAUUCCAGAACCUA
   ||| |||| ||||||||||||
   GCUAAGUC-UAAGGUCUUGGAC 5'
AT1G76600.1     202     222
unknown protein
```

```
sRNA_AGO1_Solexa_Mi2008_1_37225_hit4
5' UGAUGAUGAUGAUGAUGAAGAAG
    |||||||||||||| |||||||
   UCUACUACUACUACUUCUUCUUC 5'
AT1G76600.1     226     248
unknown protein

sRNA_AGO1_Solexa_Mi2008_3_13421_hit1
5' GAAGAAGAAGAUGAUGUUGAU
    ||||||||||||||| ||||
   AUUCUUCUUCUACUACUACUA 5'
AT1G76600.1     236     256
unknown protein

sRNA_AGO1_Solexa_Mi2008_1_53872_hit1
5' UUUAA-AAUGAAGAUGAUGAUGA
   ||||| || |||||||||||||
   AAAUUCUU-CUUCUACUACUACU 5'
AT1G76600.1     238     259
unknown protein

sRNA_AGO1_Solexa_Mi2008_2_368_hit1
5' AAAGAAUGAGAGAAUGA-GAU
    |||||||||||| ||| |||
   GUUCUUACUCUCUCACUACUA 5'
AT1G77640.1     101     121
hypothetical protein

sRNA_AGO1_Solexa_Mi2008_19_11133_hit1
5' CUC-GGUGGUGAAGAUGCAG
    || ||||||||||||||||
   AAGACCACCACUUCUACGUC 5'
AT1G77640.1     544     563
hypothetical protein

sRNA_AGO1_Solexa_Mi2008_1_351_hit2
5' AAAGAAGAAAA-ACAGAUCU
    |||||||||| | ||||||
   GUUCUUCUUUUGU-UCUAGA 5'
AT1G77680.1     121     139
putative 3'-5' exoribonuclease, 3' partial

sRNA_AGO1_Solexa_Mi2008_9_5201_hit2
5' CAAUGUCUUGCUGUAAUGCUG
   ||| |||||||| |||||||
   GUU-CAGAACGAGAUUACGAA 5'
AT1G77680.1     1491    1510
putative 3'-5' exoribonuclease, 3' partial

sRNA_AGO1_Solexa_Mi2008_1_6621_hit1
5' CAGGUGAAG-AAGAUAACGU
   ||||||||| ||| |||||
   GUCCACUUCUUUC-AUUGCC 5'
AT1G77680.1     631     649
putative 3'-5' exoribonuclease, 3' partial

sRNA_AGO1_Solexa_Mi2008_2_8245_hit1
5' CGAA-AAGAAGAAGAUGAUGAG
   |||| |||||||||| |||||:
   GCUUAUUCUUCUUCUUCUACUU 5'
AT1G77680.1     82      103
putative 3'-5' exoribonuclease, 3' partial
```

```
sRNA_AGO1_Solexa_Mi2008_2_10380_hit1
5' CUAGUGGACCUUUGAUAUCUA
   |||||||||||||||||||||
   GAUCACCUGGAAACUAUAGAU 5'
AT1G77760.1     2356    2376
nitrate reductase 1 (NR1)

sRNA_AGO1_Solexa_Mi2008_1_39739_hit2
5' UGGAAGAU-GGAGUGAUGCCUC
   ||| |||| ||||||||||||
   ACCAUCUAUCCUCACUACGGAC 5'
AT1G77760.1     2465    2486
nitrate reductase 1 (NR1)

sRNA_AGO1_Solexa_Mi2008_93_9914_hit1
5' CUACCAGCCGUUGA-UCAUGCU
   ||| |||||||||| |||| ||
   GAUAGUCGGCAACUAAGUA-GA 5'
AT1G77760.1     384     404
nitrate reductase 1 (NR1)

sRNA_AGO1_Solexa_Mi2008_3_13312_hit1
5' GAAAAAGGUGGAGAGAUAAG
    |||||| |||||||| |||
   AUUUUUC-ACCUCUCU-UUC 5'
AT1G77840.1     260     277
putative eukaryotic translation initiation factor 5 (EIF-5) sp|P48724; similar to
ESTs emb|F19992, gb|N96933, emb|Z33699, emb|F19991

sRNA_AGO1_Solexa_Mi2008_1_20688_hit1
5' UACUUCACGGUGGUUGAUGUC
   |||||||||||||||||||||
   AUGAAGUGCCACCAACUACAG 5'
AT1G77850.1     119     139
putative auxin response factor protein (T32E8.16)

sRNA_AGO1_Solexa_Mi2008_10_11960_hit1
5' CUGGCUCCCUGUAUGCCAU
   |||||||||||:||||||
   GACCGAGGGACGUACGGUC 5'
AT1G77850.1     1407    1425
putative auxin response factor protein (T32E8.16)

sRNA_AGO1_Solexa_Mi2008_4_38719_hit2
5' UGCCUGGCUCCCUGUAUGCCAC
   ||||||||||||||:||||||
   ACGGACCGAGGGACGUACGGUC 5'
AT1G77850.1     1407    1428
putative auxin response factor protein (T32E8.16)

sRNA_AGO1_Solexa_Mi2008_2_38720_hit1
5' UGCCUGGCUCCCUGUAUGCCAU
   ||||||||||||||:||||||
   ACGGACCGAGGGACGUACGGUC 5'
AT1G77850.1     1407    1428
putative auxin response factor protein (T32E8.16)

sRNA_AGO1_Solexa_Mi2008_35_8196_hit3
5' CCUGGCUCCCUGUAUGCCA
   ||||||||||||:||||||
   GGACCGAGGGACGUACGGU 5'
AT1G77850.1     1408    1426
```

```
putative auxin response factor protein (T32E8.16)

sRNA_AGO1_Solexa_Mi2008_141_14646_hit3
5' GCCUGGCUCCCUGUAUGCCA
   |||||||||||||:||||||
   CGGACCGAGGGACGUACGGU 5'
AT1G77850.1     1408    1427
putative auxin response factor protein (T32E8.16)

sRNA_AGO1_Solexa_Mi2008_12_38715_hit1
5' UGCCUGGCUCCCUGCAUGCCA
   |||||||||||||||||||||
   ACGGACCGAGGGACGUACGGU 5'
AT1G77850.1     1408    1428
putative auxin response factor protein (T32E8.16)

sRNA_AGO1_Solexa_Mi2008_7192_38718_hit3
5' UGCCUGGCUCCCUGUAUGCCA
   ||||||||||||||:||||||
   ACGGACCGAGGGACGUACGGU 5'
AT1G77850.1     1408    1428
putative auxin response factor protein (T32E8.16)

sRNA_AGO1_Solexa_Mi2008_37_3172_hit2
5' AUGCCUGGCUCCCUGUAUGCCA
    ||||||||||||||:||||||
   AACGGACCGAGGGACGUACGGU 5'
AT1G77850.1     1408    1429
putative auxin response factor protein (T32E8.16)

sRNA_AGO1_Solexa_Mi2008_83_38717_hit3
5' UGCCUGGCUCCCUGUAUGCC
   ||||||||||||||:|||||
   ACGGACCGAGGGACGUACGG 5'
AT1G77850.1     1409    1428
putative auxin response factor protein (T32E8.16)

sRNA_AGO1_Solexa_Mi2008_183_3171_hit2
5' AUGCCUGGCUCCCUGUAUGCC
    ||||||||||||||:|||||
   AACGGACCGAGGGACGUACGG 5'
AT1G77850.1     1409    1429
putative auxin response factor protein (T32E8.16)

sRNA_AGO1_Solexa_Mi2008_1_25516_hit2
5' UAU-GCCUGGCUCCCUGUAUGCCA
   ||| |||||||||||||:||||||
   AUAACGGACCGAGGGACGUACGGU 5'
AT1G77850.1     1409    1432
putative auxin response factor protein (T32E8.16)

sRNA_AGO1_Solexa_Mi2008_296_38716_hit3
5' UGCCUGGCUCCCUGUAUGC
   ||||||||||||||:||||
   ACGGACCGAGGGACGUACG 5'
AT1G77850.1     1410    1428
putative auxin response factor protein (T32E8.16)

sRNA_AGO1_Solexa_Mi2008_5_3170_hit2
5' AUGCCUGGCUCCCUGUAUGC
    ||||||||||||||:||||
   AACGGACCGAGGGACGUACG 5'
AT1G77850.1     1410    1429
```

```
putative auxin response factor protein (T32E8.16)

sRNA_AGO1_Solexa_Mi2008_4_25515_hit2
5' UAU-GCCUGGCUCCCUGUAUGCC
   ||| ||||||||||||:|||||
   AUAACGGACCGAGGGACGUACGG 5'
AT1G77850.1     1410    1432
putative auxin response factor protein (T32E8.16)

sRNA_AGO1_Solexa_Mi2008_7_48235_hit1
5' UUAU-GCCUGGCUCCCUGUAUG
   |||| ||||||||||||:|||
   AAUAACGGACCGAGGGACGUAC 5'
AT1G77850.1     1412    1433
putative auxin response factor protein (T32E8.16)

sRNA_AGO1_Solexa_Mi2008_31_5258_hit1
5' CA-CAAAGAACCACCAC-UCU
   || |||||| ||||||| |||
   GUCGUUUCUAGGUGGUGAAGA 5'
AT1G77850.1     1859    1879
putative auxin response factor protein (T32E8.16)

sRNA_AGO1_Solexa_Mi2008_1_1991_hit1
5' AGAUAUUAUAUAAAAUAGU
   ||| |||||||| ||||||
   UCU-UAAUAUAUAUUAUCA 5'
AT1G77850.1     1911    1928
putative auxin response factor protein (T32E8.16)

sRNA_AGO1_Solexa_Mi2008_1_3_hit25
5' AAAAAAAAAA-AAAGAAAGA
   |||||||||| ||||||||
   UUUUUUUUUUCUUUCUUUCA 5'
AT1G77850.1     1989    2008
putative auxin response factor protein (T32E8.16)

sRNA_AGO1_Solexa_Mi2008_1_3_hit25
5' AAAAAAAAAAAAAGAAAGA
    | |||||||||||||||
   CUAUUUUUUUUUUCUUUCU 5'
AT1G77850.1     1992    2010
putative auxin response factor protein (T32E8.16)

sRNA_AGO1_Solexa_Mi2008_1_8247_hit1
5' CGAA-AAGAGGUAGCUACUGUU
    ||| ||||||||| ||||||
   ACUUCUUCUCCAUCCAUGACAA 5'
AT1G77850.1     27      48
putative auxin response factor protein (T32E8.16)

sRNA_AGO1_Solexa_Mi2008_1_56164_hit1
5' UUUGUGUGUGUGUAGUGAAUUA
   ||||||||||||||||||||||
   AAACACACACACAUCACUUAAU 5'
AT1G78080.1     17      38
putative AP2 domain containing protein (At1g78080)

sRNA_AGO1_Solexa_Mi2008_4_21464_hit1
5' UAGAGGU-AGAGAUAGAUAU
   ||||| | |||||||||||
   AUCUC-ACUCUCUAUCUAUC 5'
AT1G78580.1     41      59
```

```
trehalose-6-phosphate synthase

sRNA_AGO1_Solexa_Mi2008_1_50384_hit1
5' UUGACGGACGAAGUAAGUGGA
   |||||||||||||||||||||
   AACUGCCUGCUUCAUUCACCU 5'
AT1G78830.1     1016    1036
unknown protein (At1g78830)

sRNA_AGO1_Solexa_Mi2008_1_22802_hit1
5' UAGGAUCGGCAAGUACAUGGU
   |||||||||||||||||||||
   AUCCUAGCCGUUCAUGUACCA 5'
AT1G78895.1     750     770
unknown protein

sRNA_AGO1_Solexa_Mi2008_1_16975_hit2
5' UAAAUAAGUAAAUG-UUUAUCUA
   |||||||||||||| || || ||
   AUUUAUUCAUUUACUAAUUA-AU 5'
AT1G78970.2     2342    2363
unknown protein

sRNA_AGO1_Solexa_Mi2008_1_16975_hit2
5' UAAAUAAGUAAAUG-UUUAUCUA
   |||||||||||||| || || ||
   AUUUAUUCAUUUACUAAUUA-AU 5'
AT1G78970.1     2507    2528
unknown protein

sRNA_AGO1_Solexa_Mi2008_2_15621_hit2
5' GUCAUAUGCUUGUCUCAAAGA
    |||:| ||||||||||||||
   AAGUGU-CGAACAGAGUUUCU 5'
AT1G79560.1     2073    2092
FtsH like cell division protein

sRNA_AGO1_Solexa_Mi2008_1_5313_hit2
5' CACACGCAAU-AAAGUAGACAAG
   |||||||||| |||| ||||||
   GUGUGCGUUAGUUUC-UCUGUUA 5'
AT1G80160.1     658     679
unknown protein

sRNA_AGO1_Solexa_Mi2008_1_5313_hit2
5' CACACGCAAU-AAAGUAGACAAG
   |||||||||| |||| ||||||
   GUGUGCGUUAGUUUC-UCUGUUA 5'
AT1G80160.2     756     777
unknown protein

sRNA_AGO1_Solexa_Mi2008_1_14331_hit2
5' GAUGGUGAACUAU-GCCUGA
   ||||||||||||| | |||
   CUACCACUUGAUAGC-GACG 5'
AT1G80170.1     1163    1181
putative polygalacturonase

sRNA_AGO1_Solexa_Mi2008_3_8768_hit1
5' CGCAGUUG-CAGAGAUGAUGUC
   |||||| | ||||||||||| |
   GCGUCA-CAGUCUCUACUACCG 5'
AT1G80170.1     916     936
```

```
putative polygalacturonase

sRNA_AGO1_Solexa_Mi2008_17_5479_hit1
5' CACCAUGGAAAGGCUGAAG
     |||||| ||||||||||
   UCGGUACCCUUCCGACUUC 5'
AT1G80300.1     167     185
adenine nucleotide translocase

sRNA_AGO1_Solexa_Mi2008_2_17207_hit1
5' UAACACCACCAAGAC-UCCU
     ||||||||||||| ||||
   CGUGUGGUGGUUCUGUAGGA 5'
AT1G80300.1     508     527
adenine nucleotide translocase

sRNA_AGO1_Solexa_Mi2008_4_297_hit1
5' AAACAUGAUUGGUA-ACAAUA
   |||||||||||| | |||||
   UUUGUACUAACC-UGUGUUAC 5'
AT1G80950.1     745     764
unknown protein

sRNA_AGO1_Solexa_Mi2008_2_49752_hit2
5' UUCUCUACAAACUUUUCCCACA
   |||||| || |||||||||||
   AAGAGAAGUAUGAAAAGGGUGA 5'
AT2G01100.3     1184    1205
unknown protein

sRNA_AGO1_Solexa_Mi2008_1_26541_hit2
5' UCAAAAACAAAGAGAGUUAAUA
    |||||||| ||||||:|||||
   CGUUUUUGUGUCUCUCGAUUAU 5'
AT2G01100.3     189     210
unknown protein

sRNA_AGO1_Solexa_Mi2008_1_23619_hit1
5' UAGUAAUCUACAUGAUGGUAU
   :|||| ||||||||||||||
   GUCAU-AGAUGUACUACCAUU 5'
AT2G01100.3     440     459
unknown protein

sRNA_AGO1_Solexa_Mi2008_2_49752_hit2
5' UUCUCUACAAACUUUUCCCACA
   |||||| || |||||||||||
   AAGAGAAGUAUGAAAAGGGUGA 5'
AT2G01100.1     768     789
unknown protein

sRNA_AGO1_Solexa_Mi2008_2_49752_hit2
5' UUCUCUACAAACUUUUCCCACA
   |||||| || |||||||||||
   AAGAGAAGUAUGAAAAGGGUGA 5'
AT2G01100.2     803     824
unknown protein

sRNA_AGO1_Solexa_Mi2008_8_4655_hit1
5' CAAGAGUGUUUGAGAGAUA
   |||||||||||| ||||
   GUUCUCACAAACCCUCUCA 5'
AT2G01420.2     453     471
```

```
auxin transporter splice variant b (PIN4)

sRNA_AGO1_Solexa_Mi2008_1_2542_hit2
5' AUAGAUAUAACACCAGGAU
   |||||||| ||||||| ||
   UAUCUAUACUGUGGUC-UA 5'
AT2G02955.1     1479    1496

sRNA_AGO1_Solexa_Mi2008_1_12105_hit1
5' CUGGUGAUGAUGAUUGU-GGUA
   ||||||||||||||| | |||
   GACCACUACUACUAAGAGCCAC 5'
AT2G02955.1     1763    1784

sRNA_AGO1_Solexa_Mi2008_6_402_hit22
5' AAAGAGGUCGAG-UAGAAUGA
   |||||||||||| ||| |||
   UUUCUCCAGCUCAAUC-UACG 5'
AT2G02955.1     2065    2084

sRNA_AGO1_Solexa_Mi2008_3_240_hit2
5' AAAAGUCCAAA-AAAAAUUGUU
   ||||||| ||| |||| |||||
   UUUUCAG-UUUCUUUUAAACAA 5'
AT2G03120.1     1355    1375
unknown protein

sRNA_AGO1_Solexa_Mi2008_1_7931_hit2
5' CCCGAUGAUGAUGAUGAG-AC
   |  ||||||||||||||| ||
   GAUCUACUACUACUACUCCUG 5'
AT2G03240.1     128     148
unknown protein

sRNA_AGO1_Solexa_Mi2008_1_25289_hit1
5' UAUGACGAUGAUGAUGACGGA
   :|||| ||||||||||| |||
   GUACUACUACUACUACU-CCU 5'
AT2G03240.1     129     148
unknown protein

sRNA_AGO1_Solexa_Mi2008_1_56092_hit3
5' UUUGUGAUGAUGAUGAUGAUGA
   |  |||||||||||||||||||
   AUUCACUACUACUACUACUACU 5'
AT2G03240.1     131     152
unknown protein

sRNA_AGO1_Solexa_Mi2008_1_36323_hit13
5' UGAG-GAUGAUGAUGAUGAUGA
     || |||||||||||||||||
   UAUCACUACUACUACUACUACU 5'
AT2G03240.1     132     153
unknown protein

sRNA_AGO1_Solexa_Mi2008_1_2280_hit1
5' AGUGGAUGAUGAUGAUGAUG
   |||| |||||||||||||||
   UCAC-UACUACUACUACUAC 5'
AT2G03240.1     133     151
unknown protein

sRNA_AGO1_Solexa_Mi2008_1_13537_hit1
```

```
5' GAAGUUCCGUUAAUGAUUGAU
   ||||||| ||||||| |||||
   CUUCAAGCCAAUUACGAACUA 5'
AT2G03240.1     1609    1629
unknown protein

sRNA_AGO1_Solexa_Mi2008_17_13290_hit4
5' CUUUUGUCGGAA-GAUUCAGGA
   |||||||| ||| |||| ||||
   GAAAACAG-CUUACUAACUCCU 5'
AT2G03510.1     1124    1144
unknown protein

sRNA_AGO1_Solexa_Mi2008_2_34047_hit4
5' UCUUUUGUCGGAA-GAUUCAGGA
   ||||||||| ||| |||| ||||
   AGAAAACAG-CUUACUAACUCCU 5'
AT2G03510.1     1124    1145
unknown protein

sRNA_AGO1_Solexa_Mi2008_1_34046_hit4
5' UCUUUUGUCGGAA-GAUUCAGG
   ||||||||| ||| |||| |||
   AGAAAACAG-CUUACUAACUCC 5'
AT2G03510.1     1125    1145
unknown protein

sRNA_AGO1_Solexa_Mi2008_61_11700_hit1
5' CUGCAGCAC-UUGGUGAAGUA
    |||||| | |||||||||||
   UACGUCG-GUAACCACUUCAU 5'
AT2G03590.1     1602    1621
unknown protein

sRNA_AGO1_Solexa_Mi2008_1_6827_hit1
5' CAUAAGCCCAAUCAUAAUGUG
   ||||||||||| |||| ||||
   GUAUUCGGGUUCGUAUAACAC 5'
AT2G03590.1     667     687
unknown protein

sRNA_AGO1_Solexa_Mi2008_1_8179_hit1
5' CCUGAUGAUGAUGCUG-CAU
    |||||||||||| || |||
   AGACUACUACUACUACUGUA 5'
AT2G03760.1     209     228
putative steroid sulfotransferase

sRNA_AGO1_Solexa_Mi2008_1_55155_hit1
5' UUUGA-GAUGAUGAUGACUAU
   | ||| |||||||||||| ||
   ACACUACUACUACUACUG-UA 5'
AT2G03760.1     209     228
putative steroid sulfotransferase

sRNA_AGO1_Solexa_Mi2008_1_13759_hit1
5' GAC-GAUGAUGAUGAUGACA
    || ||||||||||||||||
   AUGACUACUACUACUACUGU 5'
AT2G03760.1     210     229
putative steroid sulfotransferase

sRNA_AGO1_Solexa_Mi2008_1_39699_hit1
```

```
5' UGGAACUGAUGAUGAUGAUGA
    |||||||||||||||||||
   UCCUUGACUACUACUACUACU 5'
AT2G03760.1     211     231
putative steroid sulfotransferase

sRNA_AGO1_Solexa_Mi2008_1_49502_hit1
5' UUCGGGUUUUCUCGUGCUUUGA
   |||||||||||||| |||||
   AAGCCCAAAAGAGC-CGAAAGA 5'
AT2G03770.1     624     644
putative steroid sulfotransferase

sRNA_AGO1_Solexa_Mi2008_1_4698_hit2
5' CAAGCAAA-GAAUCUAAAAU
    ||||||| |||||||||||
   UUUCGUUUUCUUAGAUUUUA 5'
AT2G03770.1     956     975
putative steroid sulfotransferase

sRNA_AGO1_Solexa_Mi2008_1_45719_hit1
5' UGUGUGUGUGUGUUGGUGUGU
   |||||||||||| || |||||
   ACACACACACAC-ACAACACA 5'
AT2G03890.2     11      30
unknown protein

sRNA_AGO1_Solexa_Mi2008_1_45719_hit1
5' UGUGUGUGUGUGUUG-GUGUGU
   ||||||||||||||| || |||
   ACACACACACACAACACA-ACA 5'
AT2G03890.1     11      31
unknown protein

sRNA_AGO1_Solexa_Mi2008_1_45719_hit1
5' UGUGUGUGUGUGUUGGUGUGU
   ||||||||||||| | |||||
   ACACACACACACA-CAACACA 5'
AT2G03890.1     14      33
unknown protein

sRNA_AGO1_Solexa_Mi2008_1_32589_hit1
5' UCGUGUCACUAAGAUGUGUGU
   |||||||||||||||||||||
   AGCACAGUGAUUCUACACACA 5'
AT2G03890.2     1876    1896
unknown protein

sRNA_AGO1_Solexa_Mi2008_1_32589_hit1
5' UCGUGUCACUAAGAUGUGUGU
   |||||||||||||||||||||
   AGCACAGUGAUUCUACACACA 5'
AT2G03890.1     2239    2259
unknown protein

sRNA_AGO1_Solexa_Mi2008_1_19345_hit2
5' UACAGAAGAAGGAA-CAAAAUAA
   |||||||||||:|| ||||| ||
   AUGUCUUCUUCUUUGGUUUU-UU 5'
AT2G03890.2     520     541
unknown protein

sRNA_AGO1_Solexa_Mi2008_1_19345_hit2
```

```
5' UACAGAAGAAGGAA-CAAAAUAA
   |||||||||||:|| ||||| ||
   AUGUCUUCUUCUUUGGUUUU-UU 5'
AT2G03890.1     523     544
unknown protein

sRNA_AGO1_Solexa_Mi2008_1_45719_hit1
5' UGUGUGUGUGUGUUG-GUGUGU
   ||||||||||||||| || |||
   ACACACACACACAACACA-ACA 5'
AT2G03890.2     8       28
unknown protein

sRNA_AGO1_Solexa_Mi2008_1_55155_hit1
5' UUUGA-GAUGAUGAUGACUAU
   :|||| ||||||||||||||:
   GAACUACUACUACUACUGAUG 5'
AT2G05940.1     1242    1262
putative protein kinase

sRNA_AGO1_Solexa_Mi2008_9_14254_hit8
5' GAUGAUGAUGAUGAUGAUCUU
   ||||||||||||||||| ||
   CUACUACUACUACUACU-GAU 5'
AT2G05940.1     1243    1262
putative protein kinase

sRNA_AGO1_Solexa_Mi2008_7_3078_hit1
5' AUGAUGAUGA-GAAUGAUGAU
   |||||||||| || ||||||:
   UACUACUACUACU-ACUACUG 5'
AT2G05940.1     1245    1264
putative protein kinase

sRNA_AGO1_Solexa_Mi2008_1_7931_hit2
5' CCCGAUGAUGAUGAUGA-GAC
   |  |||||||||||||| |||
   GUUCUACUACUACUACUACUG 5'
AT2G05940.1     1245    1265
putative protein kinase

sRNA_AGO1_Solexa_Mi2008_1_56092_hit3
5' UUUGU-GAUGAUGAUGAUGAUGA
   :|||| |||||||||||||||||
   GAACAACUACUACUACUACUACU 5'
AT2G05940.1     1246    1268
putative protein kinase

sRNA_AGO1_Solexa_Mi2008_2_56614_hit1
5' UUUUGUGAGGUGCUAGUUU
   |||||||||||| | ||||
   AAAACACUCCAC-A-CAAA 5'
AT2G13100.1     54      70

sRNA_AGO1_Solexa_Mi2008_1_4748_hit1
5' CAAGCGAGACUAGGAUGUGUC
   |||||||||||| || |||||
   GUUCGCUCUGAU-CU-CACAG 5'
AT2G13810.1     1124    1142
putative aspartate aminotransferase

sRNA_AGO1_Solexa_Mi2008_2_11107_hit1
5' CUCGGCUGG-UUGAUGGAUCAU
```

   ||||||||| || ||||||||:
   GAGCCGACCUAA-UACCUAGUG 5'
AT2G14835.2   249   269
unknown protein

sRNA_AGO1_Solexa_Mi2008_1_22750_hit3
5' UAGGAGGAGGU-GCUGGUGGAG
   :|||||||| | ||||||||||
   GUCCUCCUC-AUCGACCACCUC 5'
AT2G14890.2   303   323
arabinogalactan-protein AGP9

sRNA_AGO1_Solexa_Mi2008_1_22750_hit3
5' UAGGAGGAGGUGCUGGUGGAG
    |||||||| |||||||||||
   CUCCUCCUCAACGACCACCUC 5'
AT2G14890.2   356   376
arabinogalactan-protein AGP9

sRNA_AGO1_Solexa_Mi2008_1_51908_hit6
5' UUGGAAAAGUGACUGCUGAUUUAGU
   |||:||||||||||| |||||||||
   AACUUUUUCACUGACAACUAAAUCA 5'
AT2G15050.1   437   461
putative lipid transfer protein

sRNA_AGO1_Solexa_Mi2008_2_34868_hit1
5' UGAAGUC-CGGUUCUCGCAUGGU
   ||||||| |||||||||||| |
   ACUUCAGUGCCAAGAGCGUAGGA 5'
AT2G15490.2   355   377
putative glucosyltransferase

sRNA_AGO1_Solexa_Mi2008_1_48518_hit2
5' UUCAAGGACAAGUCACAUGAGC
   ||||||:|||||||| |||||
   AAGUUCUUGUUCAGUUUACUCA 5'
AT2G15490.2   398   419
putative glucosyltransferase

sRNA_AGO1_Solexa_Mi2008_3_11240_hit1
5' CUCUCUCAUGUUAUACAGA
    || |||||||||||| ||
   AAG-GAGUACAAUAUG-CU 5'
AT2G15490.2   593   609
putative glucosyltransferase

sRNA_AGO1_Solexa_Mi2008_1_21700_hit1
5' UAGAUACGCAACAACACUGAG
   |||||||| |||||||| |||
   AUCUAUGCCUUGUUGUG-CUC 5'
AT2G15560.1   797   816
unknown protein

sRNA_AGO1_Solexa_Mi2008_1_45020_hit16
5' UGUGAU-CAGCAAAGACCAGCUG
   |||| | ||| ||||||||||||
   ACACAAUGUC-UUUCUGGUCGAC 5'
AT2G16640.1   3270   3291
putative chloroplast outer membrane protein

sRNA_AGO1_Solexa_Mi2008_31_7103_hit13
5' CAUGAACGUUUGUUGUGUC

```
    |||||||||| ||||||
   UUACUUGCAAAAAACACAA 5'
AT2G16650.1     1822    1840
hypothetical protein

sRNA_AGO1_Solexa_Mi2008_1_3_hit25
5' AAAAAAAAAAAA-AGAAAGA
    | ||||||||| |||||||
   AUGUUUUUUUUUAUCUUUCU 5'
AT2G16720.1     904     923
DNA-binding protein

sRNA_AGO1_Solexa_Mi2008_3_46668_hit1
5' UUAAGUGUCUCAGAUCAAGUUUUG
   |||| || ||||||||| ||||||
   AAUU-ACUGAGUCUAGUCCAAAAC 5'
AT2G16900.2     1195    1217
unknown protein

sRNA_AGO1_Solexa_Mi2008_3_46668_hit1
5' UUAAGUGUCUCAGAUCAAGUUUUG
   |||| || ||||||||| ||||||
   AAUU-ACUGAGUCUAGUCCAAAAC 5'
AT2G16900.1     1612    1634
unknown protein

sRNA_AGO1_Solexa_Mi2008_3_46668_hit1
5' UUAAGUGUCUCAGAUCAAGUUUUG
   |||| || ||||||||| ||||||
   AAUU-ACUGAGUCUAGUCCAAAAC 5'
AT2G16900.3     1654    1676
unknown protein

sRNA_AGO1_Solexa_Mi2008_1_3_hit25
5' AAAA-AA-AAAAAAAGAAAGA
   | || || ||||||||||||
   UGUUAUUGUUUUUUUCUUUCU 5'
AT2G16900.3     191     211
unknown protein

sRNA_AGO1_Solexa_Mi2008_2_51_hit1
5' AAAAAAGA-AGAGAAACAAAGA
     |||||| ||||||||||||
   CCUUUUCUCUCUCUUUGUUUCU 5'
AT2G16900.3     1       22
unknown protein

sRNA_AGO1_Solexa_Mi2008_1_10880_hit1
5' CUCCC-GUAUUCUCGAUAAG
   ||||| | |||||||||||:
   GAGGGGC-UAAGAGCUAUUU 5'
AT2G17040.1     159     177
NAM (no apical meristem)-like protein

sRNA_AGO1_Solexa_Mi2008_1_2_hit38
5' AAAAA-AAAA-AAAAAACCAU
   ||||| |||| ||||||||||
   UUUUUGUUUUGUUUUUUGGUA 5'
AT2G17130.2     1319    1339
NAD+ dependent isocitrate dehydrogenase subunit 2 like, IDH2

sRNA_AGO1_Solexa_Mi2008_1_2_hit38
5' AAAAA-AAAA-AAAAAACCAU
```

```
   ||||| |||| ||||||||||
   UUUUUGUUUUGUUUUUUGGUA 5'
AT2G17130.1     1331    1351
NAD+ dependent isocitrate dehydrogenase subunit 2 like, IDH2

sRNA_AGO1_Solexa_Mi2008_1_34613_hit1
5' UGAAGAUGAAGAUGAGUUGU
    ||||||||||| ||||||:
   UCUUCUACUUCUUCUCAACG 5'
AT2G17130.2     13      32
NAD+ dependent isocitrate dehydrogenase subunit 2 like, IDH2

sRNA_AGO1_Solexa_Mi2008_1_13411_hit2
5' GAAGAAAACGCGUGUGGUC
   |||||||||||| ||||:
   CUUCUUUUGCGC-CACCGA 5'
AT2G17420.1     102     119
putative thioredoxin reductase

sRNA_AGO1_Solexa_Mi2008_1_19461_hit1
5' UACAGCUGUGUCAUGGUACCU
   ||||||||||||||||||||
   AUGUCGACACAGUACCAUGGC 5'
AT2G17500.4     1216    1236
unknown protein

sRNA_AGO1_Solexa_Mi2008_1_19461_hit1
5' UACAGCUGUGUCAUGGUACCU
   ||||||||||||||||||||
   AUGUCGACACAGUACCAUGGC 5'
AT2G17500.3     1223    1243
unknown protein

sRNA_AGO1_Solexa_Mi2008_1_19461_hit1
5' UACAGCUGUGUCAUGGUACCU
   ||||||||||||||||||||
   AUGUCGACACAGUACCAUGGC 5'
AT2G17500.1     1278    1298
unknown protein

sRNA_AGO1_Solexa_Mi2008_1_19461_hit1
5' UACAGCUGUGUCAUGGUACCU
   ||||||||||||||||||||
   AUGUCGACACAGUACCAUGGC 5'
AT2G17500.2     1295    1315
unknown protein

sRNA_AGO1_Solexa_Mi2008_9_14254_hit8
5' GAUGAUGAUGAUGAUGAUCUU
   || ||||||||||||||||
   CU-CUACUACUACUACUAGUC 5'
AT2G17520.1     154     173
endoribonuclease/protein kinase IRE1 (IRE1)

sRNA_AGO1_Solexa_Mi2008_1_13759_hit1
5' GACGAUGAUGAUGAUGA-CA
   || |||||||||||||| ||
   CU-CUACUACUACUACUAGU 5'
AT2G17520.1     155     173
endoribonuclease/protein kinase IRE1 (IRE1)

sRNA_AGO1_Solexa_Mi2008_1_3079_hit472
5' AU-GAUGAUGAUGAUGAUGAUGA
```

```
   || || ||||||||||||||| |
   UAUCU-CUACUACUACUACUAGU 5'
AT2G17520.1     155     176
endoribonuclease/protein kinase IRE1 (IRE1)

sRNA_AGO1_Solexa_Mi2008_5_13760_hit2
5' GACGAUGAUGAUGAUGAGC
   || |||||||||||||| |
   CU-CUACUACUACUACUAG 5'
AT2G17520.1     156     173
endoribonuclease/protein kinase IRE1 (IRE1)

sRNA_AGO1_Solexa_Mi2008_1_3079_hit472
5' AUGAU-GAUGAUGAUGAUGAUGA
    |||| || ||||||||||||||
   GACUAUCU-CUACUACUACUACU 5'
AT2G17520.1     158     179
endoribonuclease/protein kinase IRE1 (IRE1)

sRNA_AGO1_Solexa_Mi2008_1_36898_hit1
5' UGAGUU-G-GAUGAUGAUGAUGA
   |||| | | ||||||||||||||
   ACUCUAUCUCUACUACUACUACU 5'
AT2G17520.1     158     180
endoribonuclease/protein kinase IRE1 (IRE1)

sRNA_AGO1_Solexa_Mi2008_1_50157_hit1
5' UUGAAUCA-GAGAGGUGACGGGU
   || || || ||||||||||||||
   AA-UUCGUACUCUCCACUGCCCA 5'
AT2G17520.1     1834    1855
endoribonuclease/protein kinase IRE1 (IRE1)

sRNA_AGO1_Solexa_Mi2008_1_29379_hit1
5' UCCACGGGCCGGUUCUGUUGC
    |||||| | |||||||||||
   CGGUGCC-G-CCAAGACAACG 5'
AT2G17670.2     164     182
unknown protein

sRNA_AGO1_Solexa_Mi2008_1_7550_hit15
5' CAUUUUUUGUUU-CUGU-GAUUG
   |||||||||||| || | |||||
   GUAAAAAACAAAGAAAGCUAAC 5'
AT2G17670.2     50      72
unknown protein

sRNA_AGO1_Solexa_Mi2008_1_7006_hit1
5' CAUCAUCAUCAUCAC-AGAAG
   ||||||||||||||| || |
   GUAGUAGUAGUAGUGAUCGUA 5'
AT2G18193.1     644     664
AAA-type ATPase like protein

sRNA_AGO1_Solexa_Mi2008_8_4218_hit28
5' CAAAG-UGGCUGCAAAAAUGUA
   ||||| ||||||||||||||
   GUUUCUACCGACGUUUUUACUC 5'
AT2G18900.1     2544    2565
unknown protein

sRNA_AGO1_Solexa_Mi2008_8_5939_hit1
5' CAGAAGCAGCAGAGGAA-AAGAA
```

    ||||| |||||||||| |||||
   AUCUUCUUCGUCUCCUUCUUCUU 5'
AT2G20120.1   269   291
unknown protein

sRNA_AGO1_Solexa_Mi2008_8_5939_hit1
5' CAGAAGCAGCAGAGGAA-AAGAA
    ||||| |||||||||| |||||
   AUCUUCUUCGUCUCCUUCUUCUU 5'
AT2G20130.1   273   295
unknown protein

sRNA_AGO1_Solexa_Mi2008_6_14255_hit369
5' GAUGAUGAU-GAUGAUGAUGAUGAU
   ||||||||| | ||||||||||||
   CUACUACUAAC-ACUACUACUACUU 5'
AT2G20130.1   34   57
unknown protein

sRNA_AGO1_Solexa_Mi2008_1_36323_hit13
5' UGAGGAU-GAUGAUGAUGAUGA
   ||| ||| | ||||||||||||
   ACUACUAAC-ACUACUACUACU 5'
AT2G20130.1   35   55
unknown protein

sRNA_AGO1_Solexa_Mi2008_1_3079_hit472
5' AUGAUGAU-GAUGAUGAUGAUGA
   |||||||| | ||||||||||||
   UACUACUAAC-ACUACUACUACU 5'
AT2G20130.1   35   56
unknown protein

sRNA_AGO1_Solexa_Mi2008_1_34559_hit1
5' UGAAGA-GAUUGUGAUGAUGUU
   ||| || ||||||||||||| |
   ACUACUACUAACACUACUACUA 5'
AT2G20130.1   37   58
unknown protein

sRNA_AGO1_Solexa_Mi2008_6_14255_hit369
5' GAUGAUGAUGA-UGAUGAUGAUGAU
   ||||||||||| || ||||||||||
   CUACUACUACUAAC-ACUACUACUA 5'
AT2G20130.1   37   60
unknown protein

sRNA_AGO1_Solexa_Mi2008_1_14256_hit8
5' GAUGAUGAUGAU-GAUGAUGAUGUU
   |||||||||||| | |||||||| |
   CUACUACUACUAAC-ACUACUACUA 5'
AT2G20130.1   37   60
unknown protein

sRNA_AGO1_Solexa_Mi2008_1_36323_hit13
5' UGAGGAUGAU-GAUGAUGAUGA
   ||| |||||| | |||||||||
   ACUACUACUAAC-ACUACUACU 5'
AT2G20130.1   38   58
unknown protein

sRNA_AGO1_Solexa_Mi2008_1_3079_hit472
5' AUGAUGAUGAU-GAUGAUGAUGA

```
   ||||||||||| | |||||||||
   UACUACUACUAAC-ACUACUACU 5'
AT2G20130.1     38      59
unknown protein

sRNA_AGO1_Solexa_Mi2008_6_14255_hit369
5' GAUGAUGAUGAUGA-UGAUGAUGAU
   :||||||||||||| || |||||||
   UUACUACUACUACUAAC-ACUACUA 5'
AT2G20130.1     40      63
unknown protein

sRNA_AGO1_Solexa_Mi2008_1_36323_hit13
5' UGAGGAUGAUGAU-GAUGAUGA
   ||| ||||||||| | ||||||
   ACUACUACUACUAAC-ACUACU 5'
AT2G20130.1     41      61
unknown protein

sRNA_AGO1_Solexa_Mi2008_1_3079_hit472
5' AUGAUGAUGAUGAU-GAUGAUGA
   |||||||||||||| | ||||||
   UACUACUACUACUAAC-ACUACU 5'
AT2G20130.1     41      62
unknown protein

sRNA_AGO1_Solexa_Mi2008_1_51445_hit1
5' UUGCAUGAUGAUGAUUGAUGU
   ||| ||||||||||||| ||
   AAC-UACUACUACUAAC-ACU 5'
AT2G20130.1     44      62
unknown protein

sRNA_AGO1_Solexa_Mi2008_1_28233_hit3
5' UCAGCAAAACCAAGGUCAUGCU
   |||||||||||||| | || ||
   AGUCGUUUUGGUUCAA-UA-GA 5'
AT2G20720.1     1082    1101
unknown protein

sRNA_AGO1_Solexa_Mi2008_1_13467_hit1
5' GAAGAUUAAUCCAU-AUU-AU
   :||||||||||||| ||| ||
   UUUCUAAUUAGGUAUUAAGUA 5'
AT2G20720.1     401     421
unknown protein

sRNA_AGO1_Solexa_Mi2008_1_56662_hit4
5' UUUUUGAAGGCAAGA-GA-GAGA
   ||||| ||||||||| || ||||
   AAAAAGUUCCGUUCUACUACUCU 5'
AT2G21050.1     48      70
AUX1-like amino acid permease

sRNA_AGO1_Solexa_Mi2008_1_48515_hit1
5' UUCAAGGAACGGAUU-UUGUUA
   |||||||||| |||| |||||
   AAGUUCCUUGGCUAAGAACAAG 5'
AT2G21520.1     144     165
putative phosphatidylinositol/phosphatidylcholine transfer protein

sRNA_AGO1_Solexa_Mi2008_1_48515_hit1
5' UUCAAGGAACGGAUU-UUGUUA
```

```
   |||||||||| |||| |||||
   AAGUUCCUUGGCUAAGAACAAG 5'
AT2G21520.2     162     183
putative phosphatidylinositol/phosphatidylcholine transfer protein

sRNA_AGO1_Solexa_Mi2008_2_13369_hit1
5' GAA-AUUGUAAAGAGGUUCUGA
   ||| ||| |||||||||||||
   CUUGUAAGAUUUCUCCAAGACC 5'
AT2G21580.2     518     539
40S ribosomal protein S25

sRNA_AGO1_Solexa_Mi2008_2_13369_hit1
5' GAA-AUUGUAAAGAGGUUCUGA
   ||| ||| |||||||||||||
   CUUGUAAGAUUUCUCCAAGACC 5'
AT2G21580.1     551     572
40S ribosomal protein S25

sRNA_AGO1_Solexa_Mi2008_1_52954_hit1
5' UUGGUCCAUGGUGUUUU-GACA
   ||| ||||||||||||| || |
   AAC-AGGUACCACAAAAACUUU 5'
AT2G21640.1     238     258
unknown protein

sRNA_AGO1_Solexa_Mi2008_12_23988_hit1
5' UAGUGGUG-CGUUGUUGAGAUC
    ||||||| | |||||||||||
   UUCACCACAG-AACAACUCUAG 5'
AT2G21870.2     50      70
putative ATP synthase

sRNA_AGO1_Solexa_Mi2008_1_7667_hit9
5' CCACAAAUCCUUAAGCUCUUCC
   ||| ||| |||| |||||||||
   GGU-UUU-GGAAAUCGAGAAGG 5'
AT2G22100.1     618     637
putative RNA-binding protein

sRNA_AGO1_Solexa_Mi2008_2_11061_hit1
5' CUCGCCAUUGUUGAUCGGU
    ||||||||||||| |||:
   UAGCGGUAACAACU-GCCG 5'
AT2G22100.1     8       25
putative RNA-binding protein

sRNA_AGO1_Solexa_Mi2008_1_23262_hit1
5' UAGGGUCUUGAAACGUUGUGAG
   ||||| |||||| |||||||||
   AUCCC-GAACUUCGCAACACUC 5'
AT2G22330.1     129     149
putative cytochrome P450

sRNA_AGO1_Solexa_Mi2008_1_21384_hit1
5' UAGAGGAGGCGCGUUGACGGU
   ||||||||||||||||||||
   AUCUCCUCCGCGCAACUGCCA 5'
AT2G22500.1     438     458
putative mitochondrial dicarboxylate carrier protein

sRNA_AGO1_Solexa_Mi2008_2_4570_hit2
5' CAAGAAGAGAUUCCAU-AGU
```

```
   |||||||||| ||||| |||
   GUUCUUCUCUUAGGUAGUCA 5'
AT2G22840.1     233     252
unknown protein

sRNA_AGO1_Solexa_Mi2008_3_5131_hit1
5' CAAUGAAAAAGGGCCUAUU-CUC
   |||||||||||| ||| || |||
   GUUACUUUUUCCAGGAAAAUGAG 5'
AT2G22840.1     471     493
unknown protein

sRNA_AGO1_Solexa_Mi2008_9_5355_hit1
5' CACAG-CUUUCUUGAACUUU
   ||||| |||||||||||  |
   GUGUCCGAAAGAACUUGCUA 5'
AT2G22840.1     780     799
unknown protein

sRNA_AGO1_Solexa_Mi2008_138_7679_hit1
5' CCACAG-CUUUCUUGAACUG
   |||||| ||||||||||| :
   GGUGUCCGAAAGAACUUGCU 5'
AT2G22840.1     781     800
unknown protein

sRNA_AGO1_Solexa_Mi2008_54_7680_hit1
5' CCACAG-CUUUCUUGAACUU
   |||||| |||||||||||
   GGUGUCCGAAAGAACUUGCU 5'
AT2G22840.1     781     800
unknown protein

sRNA_AGO1_Solexa_Mi2008_18_29330_hit1
5' UCCACAG-CUUUCUUGAACUG
   ||||||| ||||||||||| :
   AGGUGUCCGAAAGAACUUGCU 5'
AT2G22840.1     781     801
unknown protein

sRNA_AGO1_Solexa_Mi2008_11_29331_hit1
5' UCCACAG-CUUUCUUGAACUU
   ||||||| |||||||||||
   AGGUGUCCGAAAGAACUUGCU 5'
AT2G22840.1     781     801
unknown protein

sRNA_AGO1_Solexa_Mi2008_1449_48967_hit1
5' UUCCACAG-CUUUCUUGAACUG
   |||||||| ||||||||||| :
   AAGGUGUCCGAAAGAACUUGCU 5'
AT2G22840.1     781     802
unknown protein

sRNA_AGO1_Solexa_Mi2008_809_48968_hit1
5' UUCCACAG-CUUUCUUGAACUU
   |||||||| |||||||||||
   AAGGUGUCCGAAAGAACUUGCU 5'
AT2G22840.1     781     802
unknown protein

sRNA_AGO1_Solexa_Mi2008_9_12589_hit1
5' CUUCCACAG-CUUUCUUGAACUG
```

   ||||||||| ||||||||||| :
   GAAGGUGUCCGAAAGAACUUGCU 5'
AT2G22840.1   781   803
unknown protein

sRNA_AGO1_Solexa_Mi2008_8_48966_hit2
5' UUCCACAG-CUUUCUUGAACU
   |||||||| |||||||||||
   AAGGUGUCCGAAAGAACUUGC 5'
AT2G22840.1   782   802
unknown protein

sRNA_AGO1_Solexa_Mi2008_5_12588_hit1
5' CUUCCACAG-CUUUCUUGAACU
   ||||||||| |||||||||||
   GAAGGUGUCCGAAAGAACUUGC 5'
AT2G22840.1   782   803
unknown protein

sRNA_AGO1_Solexa_Mi2008_1_42987_hit1
5' UGGUAUGAUGAUGUGUUACUCA
   |||||||||| |||||| | ||
   ACCAUACUACAACACAA-G-GU 5'
AT2G22840.1   949   968
unknown protein

sRNA_AGO1_Solexa_Mi2008_1_11584_hit1
5' CUGAUGACAUAAAUUUACAU
    :||||||||||| ||||||
   AGCUACUGUAUUU-AAUGUA 5'
AT2G22860.1   364   382
unknown protein

sRNA_AGO1_Solexa_Mi2008_8_7746_hit1
5' CCAGAAGCCUCUAGGAUGUU
   ||||||||||||||||||||
   GGUCUUCGGAGAUCCUACAA 5'
AT2G23520.1   2738   2757
hypothetical protein

sRNA_AGO1_Solexa_Mi2008_1_21596_hit4
5' UAGAGUGA-ACAAUGAUGAG
    ||||| | |||||||||||
   CUCUCA-UCUGUUACUACUC 5'
AT2G23810.1   942   960
similar to senescence-associated protein

sRNA_AGO1_Solexa_Mi2008_2_44783_hit3
5' UGUCGUUGGCUAAGUCCGUUC
    ||| |||||||||||| |||
   CCAGAAACCGAUUCAGG-AAG 5'
AT2G24200.2   202   221
putative leucine aminopeptidase

sRNA_AGO1_Solexa_Mi2008_2_44783_hit3
5' UGUCGUUGGCUAAGUCCGUUC
    ||| |||||||||||| |||
   CCAGAAACCGAUUCAGG-AAG 5'
AT2G24200.1   228   247
putative leucine aminopeptidase

sRNA_AGO1_Solexa_Mi2008_6_14255_hit369
5' GAUGAUGAUGAUGAUGAUGAUGAU

```
   ||||||||||||||||||:||| :
   CUACUACUACUACUACUAUUACCG 5'
AT2G25110.1     157     180
unknown protein

sRNA_AGO1_Solexa_Mi2008_1_14256_hit8
5' GAUGAUGAUGAUGAUGAUGAUGUU
   ||||||||||||||||||:||| :
   CUACUACUACUACUACUAUUACCG 5'
AT2G25110.1     157     180
unknown protein

sRNA_AGO1_Solexa_Mi2008_1_3079_hit472
5' AUGAUGAUGAUGAUGAUGAUGA
   |||||||||||||||||:|||
   UACUACUACUACUACUAUUACC 5'
AT2G25110.1     158     179
unknown protein

sRNA_AGO1_Solexa_Mi2008_9_14254_hit8
5' GAUGAUGAUGAUGAUGAUCUU
   ||||||||||||||||||  |
   CUACUACUACUACUACUAUUA 5'
AT2G25110.1     160     180
unknown protein

sRNA_AGO1_Solexa_Mi2008_1_34544_hit1
5' UGAAGACGAAGAUGAUGAUGAUA
   |||||| || |||||||||||||
   ACUUCUACUACUACUACUACUAU 5'
AT2G25110.1     162     184
unknown protein

sRNA_AGO1_Solexa_Mi2008_1_2280_hit1
5' AG-UGGAUGAUGAUGAUGAUG
   || || ||||||||||||||:
   UCUAC-UACUACUACUACUAU 5'
AT2G25110.1     163     182
unknown protein

sRNA_AGO1_Solexa_Mi2008_6_14255_hit369
5' GAUGAUGAUGAUGAUGAUGAUGAU
   ||||| ||||||||||||||||||
   CUACUUCUACUACUACUACUACUA 5'
AT2G25110.1     163     186
unknown protein

sRNA_AGO1_Solexa_Mi2008_1_14256_hit8
5' GAUGAUGAUGAUGAUGAUGAUGUU
   ||||| |||||||||||||||| |
   CUACUUCUACUACUACUACUACUA 5'
AT2G25110.1     163     186
unknown protein

sRNA_AGO1_Solexa_Mi2008_7_3078_hit1
5' AUGAUGAUGA-GAAUGAUGAU
    ||||||||| || |||||||
   AACUACUACUACU-ACUACUA 5'
AT2G25110.1     164     183
unknown protein

sRNA_AGO1_Solexa_Mi2008_1_36323_hit13
5' UGAGGAUGAUGAUGAUGAUGA
```

```
   |||:|||||||||||||||||
   ACUUCUACUACUACUACUACU 5'
AT2G25110.1     164     184
unknown protein

sRNA_AGO1_Solexa_Mi2008_1_3079_hit472
5' AUGAUGAUGAUGAUGAUGAUGA
   |||| |||||||||||||||||
   UACUUCUACUACUACUACUACU 5'
AT2G25110.1     164     185
unknown protein

sRNA_AGO1_Solexa_Mi2008_1_36898_hit1
5' UGA-GUUGGAUGAUGAUGAUGA
   ||| | || |||||||||||||
   ACUUCUAC-UACUACUACUACU 5'
AT2G25110.1     165     185
unknown protein

sRNA_AGO1_Solexa_Mi2008_1_45039_hit3
5' UG-UGAUGAUGAUGAUGAUGAUGA
   || ||| |||||||||||||||||
   ACUACUUCUACUACUACUACUACU 5'
AT2G25110.1     165     188
unknown protein

sRNA_AGO1_Solexa_Mi2008_6_14255_hit369
5' GAUGAUGAUGAUGAUGAUGAUGAU
   |||||||| |||||||||||||||
   CUACUACUUCUACUACUACUACUA 5'
AT2G25110.1     166     189
unknown protein

sRNA_AGO1_Solexa_Mi2008_1_14256_hit8
5' GAUGAUGAUGAUGAUGAUGAUGUU
   |||||||| ||||||||||||| |
   CUACUACUUCUACUACUACUACUA 5'
AT2G25110.1     166     189
unknown protein

sRNA_AGO1_Solexa_Mi2008_1_3079_hit472
5' AUGAUGAUGAUGAUGAUGAUGA
   ||||||| ||||||||||||||
   UACUACUUCUACUACUACUACU 5'
AT2G25110.1     167     188
unknown protein

sRNA_AGO1_Solexa_Mi2008_1_38238_hit1
5' UGCAUGAACAUGAUGAUGAUGG
   || ||||| ||||||||||||:
   AC-UACUUCUACUACUACUACU 5'
AT2G25110.1     168     188
unknown protein

sRNA_AGO1_Solexa_Mi2008_3_14271_hit18
5' GAUGCUGAAUAUGAUGAUGA-GA
   |||| |||| |||||||||| ||
   CUACUACUUCUACUACUACUACU 5'
AT2G25110.1     168     190
unknown protein

sRNA_AGO1_Solexa_Mi2008_1_56092_hit3
5' UUUG-UGAUGAUGAUGAUGA
```

```
   :||| ||| |||||||||||||
   GAACUACUUCUACUACUACUACU 5'
AT2G25110.1     168     190
unknown protein

sRNA_AGO1_Solexa_Mi2008_1_3079_hit472
5' AUGAUGAUGAUGAUGAUGAUGA
     |||||||| ||||||||||
   CUCUACUACUUCUACUACUACU 5'
AT2G25110.1     170     191
unknown protein

sRNA_AGO1_Solexa_Mi2008_1_41920_hit17
5' UGG-GAUGAUGAAGUUGAUGAU
   ||| |||||||||| |||||||
   ACCUCUACUACUUCUACUACUA 5'
AT2G25110.1     173     194
unknown protein

sRNA_AGO1_Solexa_Mi2008_3_49087_hit1
5' UUCCGAGGUGGUGUGGUCU
   ||||||||||||||  |||
   AAGGCUCCACCACAG-AGA 5'
AT2G25450.1     1089    1106
putative dioxygenase

sRNA_AGO1_Solexa_Mi2008_9_52832_hit1
5' UUGGGUUGGU-UCGGGUAUAGAA
   |||| || || |||||||||||
   AACCAAAACAGAGCCCAUAUCUU 5'
AT2G25450.1     450     472
putative dioxygenase

sRNA_AGO1_Solexa_Mi2008_1_2638_hit1
5' AUAGGUUGUGCAGUUUGAGCU
   ||||| | ||||||||||||
   UAUCCUAGACGUCAAACUCGA 5'
AT2G25490.1     1962    1982
putative glucose regulated repressor protein

sRNA_AGO1_Solexa_Mi2008_2_8128_hit2
5' CCUAGAACGGUUCGGGGGUGUU
   ||||||||| ||||||| | ||
   GGAUCUUGCAAAGCCCC-A-AA 5'
AT2G25680.1     46      65
hypothetical protein

sRNA_AGO1_Solexa_Mi2008_1_8490_hit1
5' CGAGAAGAAUGAUGUCCUCU
   |||||||||||||| |||
   GCUCUUCUUACUAC-GGAAC 5'
AT2G26430.2     1301    1319
cyclin like protein

sRNA_AGO1_Solexa_Mi2008_1_8490_hit1
5' CGAGAAGAAUGAUGUCCUCU
   |||||||||||||| |||
   GCUCUUCUUACUAC-GGAAC 5'
AT2G26430.1     1308    1326
cyclin like protein

sRNA_AGO1_Solexa_Mi2008_1_8490_hit1
5' CGAGAAGAAUGAUGUCCUCU
```

```
   |||||||||||||| |||
   GCUCUUCUUACUAC-GGAAC 5'
AT2G26430.3     1392    1410
cyclin like protein

sRNA_AGO1_Solexa_Mi2008_2_26713_hit3
5' UC-AAAGCCAAAUCAAUAUCAC
   || ||||| ||||||||||||
   AGCUUUCGCUUUAGUUAUAGUA 5'
AT2G26530.2     431     452
AR781, similar to yeast pheromone receptor

sRNA_AGO1_Solexa_Mi2008_1_10600_hit3
5' CUC-AAAGCCAAAUCAAUAUCAC
   ||| ||||| ||||||||||||
   GAGCUUUCGCUUUAGUUAUAGUA 5'
AT2G26530.2     431     453
AR781, similar to yeast pheromone receptor

sRNA_AGO1_Solexa_Mi2008_3_26712_hit3
5' UC-AAAGCCAAAUCAAUAUCA
   || ||||| ||||||||||||
   AGCUUUCGCUUUAGUUAUAGU 5'
AT2G26530.2     432     452
AR781, similar to yeast pheromone receptor

sRNA_AGO1_Solexa_Mi2008_2_26713_hit3
5' UC-AAAGCCAAAUCAAUAUCAC
   || ||||| ||||||||||||
   AGCUUUCGCUUUAGUUAUAGUA 5'
AT2G26530.1     449     470
AR781, similar to yeast pheromone receptor

sRNA_AGO1_Solexa_Mi2008_1_10600_hit3
5' CUC-AAAGCCAAAUCAAUAUCAC
   ||| ||||| ||||||||||||
   GAGCUUUCGCUUUAGUUAUAGUA 5'
AT2G26530.1     449     471
AR781, similar to yeast pheromone receptor

sRNA_AGO1_Solexa_Mi2008_3_26712_hit3
5' UC-AAAGCCAAAUCAAUAUCA
   || ||||| ||||||||||||
   AGCUUUCGCUUUAGUUAUAGU 5'
AT2G26530.1     450     470
AR781, similar to yeast pheromone receptor

sRNA_AGO1_Solexa_Mi2008_1_14490_hit1
5' GCAUCAAUGAUAGUGUUGUAG
   ||||||||||||| | |||||
   CGUAGUUACUAUC-C-ACAUC 5'
AT2G26560.1     961     979
similar to latex allergen from Hevea brasiliensis

sRNA_AGO1_Solexa_Mi2008_1_806_hit1
5' AAGAGCAUCAAUGAUAG-UGUU
   || |||||||||||||| |||
   UU-UCGUAGUUACUAUCCACAU 5'
AT2G26560.1     962     982
similar to latex allergen from Hevea brasiliensis

sRNA_AGO1_Solexa_Mi2008_1_34059_hit1
5' UGAA-AAAGAGUGUCAUGAUGA
```

```
    ||| |||||:|||||||||||
   CCUUGUUUCUUACAGUACUACU 5'
AT2G26650.1     474     495
K+ transporter, AKT1

sRNA_AGO1_Solexa_Mi2008_1_10002_hit2
5' CUACUAUAUGAAUUAAGCUUA
   || ||||||||||:||||||
   GA-GAUAUACUUAGUUCGAAA 5'
AT2G27230.1     2422    2441
unknown protein

sRNA_AGO1_Solexa_Mi2008_1_10002_hit2
5' CUACUAUAUGAAUUAAGCUUA
   || ||||||||||:||||||
   GA-GAUAUACUUAGUUCGAAA 5'
AT2G27230.2     2459    2478
unknown protein

sRNA_AGO1_Solexa_Mi2008_2_830_hit1
5' AAGAUGGAGAAGAUACGCAA
   ||||||||||||| | ||||
   UUCUACCUCUUCU-UUCGUU 5'
AT2G27230.2     61      79
unknown protein

sRNA_AGO1_Solexa_Mi2008_2_830_hit1
5' AAGAUGGAGAAGAUACGCAA
   ||||||||||||| | ||||
   UUCUACCUCUUCUUU-CGUU 5'
AT2G27230.1     62      80
unknown protein

sRNA_AGO1_Solexa_Mi2008_2_16073_hit2
5' GUUA-AAGAAGAUGGAGAAA
    ||| ||||||||||||||
   AAAUCUUCUUCUACCUCUUC 5'
AT2G27230.2     68      87
unknown protein

sRNA_AGO1_Solexa_Mi2008_2_16073_hit2
5' GUUA-AAGAAGAUGGAGAAA
    ||| ||||||||||||||
   AAAUCUUCUUCUACCUCUUC 5'
AT2G27230.1     69      88
unknown protein

sRNA_AGO1_Solexa_Mi2008_18_5055_hit1
5' CAAGUUGAGCUUCAUGU-U-GAAA
   ||||||||||||||||| | |||
   GUUCAACUCGAAGUACAUAACUUC 5'
AT2G27430.1     86      109
unknown protein

sRNA_AGO1_Solexa_Mi2008_1_22500_hit1
5' UAGCUGAA-GAGGAAGAUGAG
   ||| |||| |||||||||| |
   AUC-ACUUUCUCCUUCUACAC 5'
AT2G27840.1     69      88
putative histone deacetylase HD2d

sRNA_AGO1_Solexa_Mi2008_1_19345_hit2
5' UACAGAAGAAGGAACAAAAUAA
```

```
   || | ||||| |||||||||||
   AU-U-UUCUUACUUGUUUUAUU 5'
AT2G28355.1     16      35
unknown protein

sRNA_AGO1_Solexa_Mi2008_1_11584_hit1
5' CUGAUGACAUAAA-UUUACAU
   ||||||||||||| |||| ||
   GACUACUGUAUUUGAAAU-UA 5'
AT2G28510.1     1045    1064
DOF zinc finger like protein

sRNA_AGO1_Solexa_Mi2008_3_35894_hit1
5' UGAGAGCAGAGAAAGAGAGU
   |||||| ||||||||||||
   ACUCUC-UCUCUUUCUCUCU 5'
AT2G28510.1     24      42
DOF zinc finger like protein

sRNA_AGO1_Solexa_Mi2008_8_13576_hit1
5' GACAAACUAAUGUUAUAUAGU
   ||||||||| ||||||| |||
   CUGUUUGAUAACAAUAU-UCA 5'
AT2G28510.1     709     728
DOF zinc finger like protein

sRNA_AGO1_Solexa_Mi2008_1_28927_hit1
5' UCAUCUGGGGACAA-ACAAGCA
   ||||||||:||||| | |||||
   AGUAGACCUCUGUUCU-UUCGU 5'
AT2G29120.1     1541    1561
glutamate receptor like protein

sRNA_AGO1_Solexa_Mi2008_1_7990_hit1
5' CCGAUGGAGAUGGUCUUACAAG
   ||||||||||||| | | ||||
   GGCUACCUCUACC-GCA-GUUC 5'
AT2G29720.1     332     351
Hydroxylase/Oxygenase (CTF2B)

sRNA_AGO1_Solexa_Mi2008_1_29892_hit1
5' UCCGAUGGAGAUGGUCUUACAA
   |||||||||||||| | | |||
   AGGCUACCUCUACC-GCA-GUU 5'
AT2G29720.1     333     352
Hydroxylase/Oxygenase (CTF2B)

sRNA_AGO1_Solexa_Mi2008_3_29723_hit1
5' UCCCCUUACAAUGUCGAG-UAA
   | ||||| |||||||||| |||
   AUGGGAAGGUUACAGCUCAAUU 5'
AT2G30140.2     1103    1124
putative glucosyltransferase

sRNA_AGO1_Solexa_Mi2008_3_29723_hit1
5' UCCCCUUACAAUGUCGAGU-AA
   | ||||| ||||||||||| ||
   AUGGGAAGGUUACAGCUCAAUU 5'
AT2G30140.1     1106    1127
putative glucosyltransferase

sRNA_AGO1_Solexa_Mi2008_6_11244_hit6
5' CUCUGAACCACAACGCUUU
```

```
    ||||||||||||| |||
   CAGACUUGGUGUUGAGAAC 5'
AT2G30250.1     1049    1067
putative WRKY-type DNA binding protein

sRNA_AGO1_Solexa_Mi2008_24_502_hit1
5' AAAGGAGGUGGUGGUUGAU
    ||| |||||||||||||
   GUUC-UCCACCACCAACUC 5'
AT2G30250.1     255     272
putative WRKY-type DNA binding protein

sRNA_AGO1_Solexa_Mi2008_1_8683_hit2
5' CGA-GUU-UCUAUUGGAAGUGGU
   ||| ||| |||||||||||||
   GCUACAAGAGAUAACCUUCACCU 5'
AT2G30440.1     1270    1292
signal peptidase I like protein

sRNA_AGO1_Solexa_Mi2008_2_28674_hit1
5' UCAGUGAAAGGAAACUUCUUG
   | || |||||||||||||||
   AAUC-CUUUCCUUUGAAGAAC 5'
AT2G30440.1     27      46
signal peptidase I like protein

sRNA_AGO1_Solexa_Mi2008_1_7006_hit1
5' CAUCAUCAUCAUCACAGAAG
   |||||||||||||||| ||
   GUAGUAGUAGUAGUGU-UUG 5'
AT2G30440.1     752     770
signal peptidase I like protein

sRNA_AGO1_Solexa_Mi2008_1_55686_hit1
5' UUUGGCGGGAAAA-UG-UUUUUGA
   ||||||||||||| || |||:|||
   AAACCGCCCUUUUAACUAAAGACU 5'
AT2G30550.1     113     136
lipase like protein

sRNA_AGO1_Solexa_Mi2008_1_55685_hit1
5' UUUGGCGGGAAAACUUCAGUUUCU
   ||||||||||||| || | |||||
   AAACCGCCCUUUU-AACU-AAAGA 5'
AT2G30550.1     115     136
lipase like protein

sRNA_AGO1_Solexa_Mi2008_1_56517_hit30
5' UUUUGGCGGGAAAAUAUGAUUUU
   ||||||||||||||| ||||||:
   AAAACCGCCCUUUUA-ACUAAAG 5'
AT2G30550.1     116     137
lipase like protein

sRNA_AGO1_Solexa_Mi2008_1_55683_hit64
5' UUUGGCGGGAAAACAUGAUUU
   |||||||||||||  ||||||
   AAACCGCCCUUUUA-ACUAAA 5'
AT2G30550.1     117     136
lipase like protein

sRNA_AGO1_Solexa_Mi2008_1_55686_hit1
5' UUUGGCGGGAAAA-UG-UUUUUGA
```

```
   ||||||||||||| || |||:|||
   AAACCGCCCUUUUAACUAAAGACU 5'
AT2G30550.2     124     147
lipase like protein

sRNA_AGO1_Solexa_Mi2008_1_55685_hit1
5' UUUGGCGGGAAAACUUCAGUUUCU
   ||||||||||||| || | |||||
   AAACCGCCCUUUU-AACU-AAAGA 5'
AT2G30550.2     126     147
lipase like protein

sRNA_AGO1_Solexa_Mi2008_1_56517_hit30
5' UUUUGGCGGGAAAAUAUGAUUUU
   ||||||||||||||| ||||||:
   AAAACCGCCCUUUUA-ACUAAAG 5'
AT2G30550.2     127     148
lipase like protein

sRNA_AGO1_Solexa_Mi2008_1_55683_hit64
5' UUUGGCGGGAAAACAUGAUUU
   |||||||||||||  ||||||
   AAACCGCCCUUUUA-ACUAAA 5'
AT2G30550.2     128     147
lipase like protein

sRNA_AGO1_Solexa_Mi2008_5_6024_hit1
5' CAG-AGAAUGAUGAUCCUGU
   ||| ||||||||||||||
   GUCCUCUUACUACUAGGAGU 5'
AT2G30550.2     1560    1579
lipase like protein

sRNA_AGO1_Solexa_Mi2008_1_30970_hit3
5' UCGAG-UGAGAGAGAUGAUGGU
    |||| ||||||||||| ||||
   CGCUCGACUCUCUCUACCACCA 5'
AT2G30550.2     1586    1607
lipase like protein

sRNA_AGO1_Solexa_Mi2008_1_26191_hit1
5' UAUUAGGCUGCGAGAGAUC
   | ||||||||||||| |||
   AAAAUCCGACGCUCUAUAG 5'
AT2G30990.3     1313    1331
hypothetical protein

sRNA_AGO1_Solexa_Mi2008_1_26191_hit1
5' UAUUAGGCUGCGAGAGAUC
   | ||||||||||||| |||
   AAAAUCCGACGCUCUAUAG 5'
AT2G30990.2     1403    1421
hypothetical protein

sRNA_AGO1_Solexa_Mi2008_1_26191_hit1
5' UAUUAGGCUGCGAGAGAUC
   | ||||||||||||| |||
   AAAAUCCGACGCUCUAUAG 5'
AT2G30990.1     1433    1451
hypothetical protein

sRNA_AGO1_Solexa_Mi2008_2_32368_hit1
5' UCGGUUUGUUUGCAGAAUCACU
```

```
   ||| ||| |:|||||||||||
   AGC-AAA-AGACGUCUUAGUGA 5'
AT2G31010.1     1030    1049
putative protein kinase

sRNA_AGO1_Solexa_Mi2008_1_52000_hit7
5' UUGGAAUGUGGUGAACUUGA
   ||||| | |||||||||||
   AACCU-A-ACCACUUGAACU 5'
AT2G31020.1     2259    2276
putative oxysterol-binding protein

sRNA_AGO1_Solexa_Mi2008_1_41488_hit1
5' UGGCGGCGCUGGUGG-UGGU
    ||||| |||||||| ||||
   CCCGCCUCGACCACCAACCA 5'
AT2G31020.1     427     446
putative oxysterol-binding protein

sRNA_AGO1_Solexa_Mi2008_1_29031_hit1
5' UCAUGUCCUAAGUUGUUGGUC
   ||||||| ||||||||||||
   AGUACAG-AUUCAACAACCAG 5'
AT2G31020.1     648     667
putative oxysterol-binding protein

sRNA_AGO1_Solexa_Mi2008_2_21913_hit1
5' UAGAUGU-UGAAGUUGUUGGUC
   :|||||| | |||||||||||
   GUCUACAGA-UUCAACAACCAG 5'
AT2G31020.1     648     668
putative oxysterol-binding protein

sRNA_AGO1_Solexa_Mi2008_1_43489_hit1
5' UGGUGGCGUUGUUGGUCUUGAA
   |||||| ||||||| ||||||
   ACCACCACAACAACAAGAACUC 5'
AT2G31070.1     1037    1058
unknown protein

sRNA_AGO1_Solexa_Mi2008_1_51213_hit1
5' UUGAUGUUCUGAUGAG-AUCCU
   ||||||||||||||| ||  |
   AACUACAAGACUACUCCUACUA 5'
AT2G31070.1     1215    1236
unknown protein

sRNA_AGO1_Solexa_Mi2008_2_51261_hit1
5' UUGAUUGAUGUUCAGAUGAUGAU
   ||| ||||||||| ||||| |||
   AAC-AACUACAAGACUACUCCUA 5'
AT2G31070.1     1218    1239
unknown protein

sRNA_AGO1_Solexa_Mi2008_6_14255_hit369
5' GAUGAUGAUGAUGAUGAUGAUGAU
   |||||||||| ||| |||||||||
   CUACUACUACCACUCCUACUACUA 5'
AT2G31070.1     1279    1302
unknown protein

sRNA_AGO1_Solexa_Mi2008_1_3079_hit472
5' AUGAUGAUGAUGAUGAUGA
```

```
   ||||||||| ||| ||||||||
   UACUACUACCACUCCUACUACU 5'
AT2G31070.1     1280    1301
unknown protein

sRNA_AGO1_Solexa_Mi2008_1_45039_hit3
5' UG-UGAUGAUGAUGAUGAUGAUGA
   || |||||||| ||| ||||||||
   ACUACUACUACCACUCCUACUACU 5'
AT2G31070.1     1281    1304
unknown protein

sRNA_AGO1_Solexa_Mi2008_6_14255_hit369
5' GAUGAUGAUGAUGAUGAUGAUGAU
   ||||||||||||| ||| ||||||
   CUACUACUACUACCACUCCUACUA 5'
AT2G31070.1     1282    1305
unknown protein

sRNA_AGO1_Solexa_Mi2008_1_3079_hit472
5' AUGAUGAUGAUGAUGAUGAUGA
   |||||||||||| ||| |||||
   UACUACUACUACCACUCCUACU 5'
AT2G31070.1     1283    1304
unknown protein

sRNA_AGO1_Solexa_Mi2008_1_36898_hit1
5' UGAGU-UGGAUGAUGAUGAUGA
   ||| | || ||||||||||||
   ACUAAGAC-UACUACUACUACU 5'
AT2G31070.1     1452    1472
unknown protein

sRNA_AGO1_Solexa_Mi2008_2_411_hit5
5' AAAGAU-GAAGAGAGAAAGAGA
   |||| | ||||||||||||||
   UUUC-AUCUUCUCUCUUUCUCU 5'
AT2G31090.1     40      60
unknown protein

sRNA_AGO1_Solexa_Mi2008_1_19407_hit2
5' UACAGAGUCGCCGGAGAUGGU
   || ||||||||||||| |||
   AU-UCUCAGCGGCCUCAACCU 5'
AT2G31190.1     26      45
unknown protein

sRNA_AGO1_Solexa_Mi2008_1_38236_hit3
5' UGCAU-CUGUUGUUGGUGUUGC
   ||| | |||:||||||||||||
   ACG-ACGACGACAACCACAACG 5'
AT2G31370.4     1254    1274
bZIP transcription factor PosF21 / AtbZip59

sRNA_AGO1_Solexa_Mi2008_1_38236_hit3
5' UGCAU-CUGUUGUUGGUGUUGC
   ||| | |||:||||||||||||
   ACG-ACGACGACAACCACAACG 5'
AT2G31370.3     1299    1319
bZIP transcription factor PosF21 / AtbZip59

sRNA_AGO1_Solexa_Mi2008_1_38236_hit3
5' UGCAU-CUGUUGUUGGUGUUGC
```

```
   ||| | |||:||||||||||||
   ACG-ACGACGACAACCACAACG 5'
AT2G31370.1     1351    1371
bZIP transcription factor PosF21 / AtbZip59

sRNA_AGO1_Solexa_Mi2008_5_12942_hit1
5' CUUGGGACAGAGUGUG-CUUAC
   ||||| |||||||||| ||| |
   GAACC-UGUCUCACACAGAAGG 5'
AT2G31560.2     95      115
unknown protein

sRNA_AGO1_Solexa_Mi2008_1_7415_hit1
5' CAUGUUUGGAUACUUGUUCUG
   ||||||||| |||||| |||
   GUACAAACCAAUGAAC-AGAA 5'
AT2G31890.1     2071    2090
unknown protein

sRNA_AGO1_Solexa_Mi2008_1_37142_hit1
5' UGAUCUUUAGUCG-GAUUUGUG
   |||||||| || | ||||||||
   ACUAGAAAACA-CACUAAACAC 5'
AT2G31980.1     75      95
putative cysteine proteinase inhibitor B (cystatin B)

sRNA_AGO1_Solexa_Mi2008_2_35642_hit1
5' UGACUAG-GUUGAUGAUCGU
   ||||| | |||||||||||
   ACUGA-CGCAACUACUAGCC 5'
AT2G31990.1     195     213
hypothetical protein

sRNA_AGO1_Solexa_Mi2008_2_14225_hit1
5' GAUGAA-AGAAAGGAAAAGA
   || ||| ||||||||||||
   CU-CUUCUCUUUCCUUUUCA 5'
AT2G31990.1     94      112
hypothetical protein

sRNA_AGO1_Solexa_Mi2008_1_43600_hit1
5' UGGUGGUUGUCAUUGUGGUAA
   |||||||||||||||||||||
   ACCACCAACAGUAACACCAUU 5'
AT2G32100.1     158     178
Unknown protein (At2g32100; F22D22.15)

sRNA_AGO1_Solexa_Mi2008_5_15110_hit2
5' GGCGUGCCGGAGUGGUUAU
   |||| |||||||||||| |
   CCGC-CGGCCUCACCAACA 5'
AT2G32100.1     220     237
Unknown protein (At2g32100; F22D22.15)

sRNA_AGO1_Solexa_Mi2008_10_15948_hit29
5' GUGGACGUGCCGGAGUGGUUA
   |||| || ||||||||||||
   CACC-GC-CGGCCUCACCAAC 5'
AT2G32100.1     221     239
Unknown protein (At2g32100; F22D22.15)

sRNA_AGO1_Solexa_Mi2008_1_36772_hit1
5' UGAGUCGGUGAUUGCAUUGGA
```

```
   ||||||||||||||||||||
   ACUCAGCCACUAACGUAACCU 5'
AT2G32100.1     336     356
Unknown protein (At2g32100; F22D22.15)

sRNA_AGO1_Solexa_Mi2008_3_15340_hit5
5' GGUUUGCUAAUUCUCGGAA
   |||||||||||| ||:|||
   CCAAACGAUUAA-AGUCUU 5'
AT2G32250.3     218     235
Mutator-like transposase

sRNA_AGO1_Solexa_Mi2008_1_56441_hit1
5' UUUUGAAUGGUAACUGUCAAAGU
   |||||||||||||||: ||| ||
   AAAACUUACCAUUGAU-GUU-CA 5'
AT2G32250.4     2938    2958
Mutator-like transposase

sRNA_AGO1_Solexa_Mi2008_3_15340_hit5
5' GGUUUGCUAAUUCUCGGAA
   |||||||||||| ||:|||
   CCAAACGAUUAA-AGUCUU 5'
AT2G32250.4     329     346
Mutator-like transposase

sRNA_AGO1_Solexa_Mi2008_1_27099_hit1
5' UCAAGAUGCUUGAGGUUGAUG
   ||||||||||||||||||||
   AGUUCUACGAACUCCAACUAC 5'
AT2G32680.1     1016    1036
putative disease resistance protein

sRNA_AGO1_Solexa_Mi2008_1_32535_hit1
5' UCGUGCGUGAUGAUGCUUUCC
   ||||||||||||||||||||
   AGCACGCACUACUACGAAAGG 5'
AT2G32680.1     1951    1971
putative disease resistance protein

sRNA_AGO1_Solexa_Mi2008_1_48497_hit1
5' UUCAA-GAGGCAACUGUUUGGA
    |||| ||||| ||||||||||
   CAGUUUCUCCGAUGACAAACCU 5'
AT2G32680.1     561     582
putative disease resistance protein

sRNA_AGO1_Solexa_Mi2008_1_10941_hit1
5' CUCCUUGGUAAUUGAUUUGG
   ||||||||||||:|| ||||
   GAGGAACCAUUAGCU-AACC 5'
AT2G32920.1     409     427
putative protein disulfide isomerase

sRNA_AGO1_Solexa_Mi2008_2_9291_hit50
5' CGUAAGAAUUGUAUCCUUGUU
    |||| |||||||||||| ||
   UCAUU-UUAACAUAGGAA-AA 5'
AT2G32920.1     8       26
putative protein disulfide isomerase

sRNA_AGO1_Solexa_Mi2008_1_50341_hit1
5' UUGACCCAGAAAGGAGAAUG-GU
```

```
   |||| |||||||||| |||| ||
   AACUCGGUCUUUCCU-UUACACA 5'
AT2G33020.1     1730    1751
putative leucine-rich repeat disease resistance protein

sRNA_AGO1_Solexa_Mi2008_1_48998_hit1
5' UUCCAG-GAAGUGAUUAUCGGAA
   |||||| |||||||||||| |||
   AAGGUCUCUUCACUAAUAGGCUU 5'
AT2G33020.1     492     514
putative leucine-rich repeat disease resistance protein

sRNA_AGO1_Solexa_Mi2008_3_18454_hit1
5' UAAGUAUGAGUGGGAUCUUGG
    |||| | |||||||||||||
   CUUCA-AGUCACCCUAGAACC 5'
AT2G33060.1     1041    1060
putative leucine-rich repeat disease resistance protein

sRNA_AGO1_Solexa_Mi2008_9_24533_hit1
5' UAUAGGGCCAUGGAAUUUGUU
   |||||||||||||||||||||
   AUAUCCCGGUACCUUAAACAA 5'
AT2G33060.1     1723    1743
putative leucine-rich repeat disease resistance protein

sRNA_AGO1_Solexa_Mi2008_1_23572_hit2
5' UAGGUUGCCCAGUUAUUUGUG
   |||:||||||||| |||||||
   AUCUAACGGGUCACUAAACAC 5'
AT2G33060.1     2258    2278
putative leucine-rich repeat disease resistance protein

sRNA_AGO1_Solexa_Mi2008_1_48998_hit1
5' UUCCAG-GAAGUGAUUAUCGGAA
   |||||| ||||||||||| ||||
   AAGGUCUCUUCACUAAUAUCCUU 5'
AT2G33060.1     706     728
putative leucine-rich repeat disease resistance protein

sRNA_AGO1_Solexa_Mi2008_1_27099_hit1
5' UCAAGAUGCUUGAGGUUGAUG
   || ||||||||||||||||||
   AGCUCUACGAACUCCAACUAC 5'
AT2G33060.1     900     920
putative leucine-rich repeat disease resistance protein

sRNA_AGO1_Solexa_Mi2008_4_22426_hit32
5' UAGC-GUUAGGGUUUAGGGUUUA
   |||| || ||||||||||||||
   AUCGUCA-UCCCAAAUCCCAAAA 5'
AT2G33210.1     12      33
mitochondrial chaperonin (HSP60)

sRNA_AGO1_Solexa_Mi2008_1_23310_hit418
5' UAGGGUUUAGGGUUU-AGGGUUU
   ||||||||||||||| |  ||||
   AUCCCAAAUCCCAAAAU-GCAAA 5'
AT2G33210.1     6       27
mitochondrial chaperonin (HSP60)

sRNA_AGO1_Solexa_Mi2008_1_52615_hit7
5' UUGGGCUUGUAGUUGUUGUAC
```

```
    ||||||||||||||| |||
   CACCCGAACAUCAACAUCAUU 5'
AT2G33850.1     708     728
unknown protein

sRNA_AGO1_Solexa_Mi2008_4_52616_hit19
5' UUGGGCUUGUAGUUGUUGUAG
    ||||||||||||||| |||:
   CACCCGAACAUCAACAUCAUU 5'
AT2G33850.1     708     728
unknown protein

sRNA_AGO1_Solexa_Mi2008_1_39748_hit1
5' UGGAAGCAGAUGGUUCGUU
   ||||| ||||||||||| |
   ACCUUGGUCUACCAAGCUA 5'
AT2G33860.1     1523    1541
auxin response transcription factor 3 (ETTIN/ARF3)

sRNA_AGO1_Solexa_Mi2008_7_33918_hit1
5' UCUUGACCUUGUAAGACCCCA
   |||||||||||:|||||||
   AGAACUGGAACGUUCUGGGAG 5'
AT2G33860.1     1672    1692
auxin response transcription factor 3 (ETTIN/ARF3)

sRNA_AGO1_Solexa_Mi2008_7_49853_hit1
5' UUCUUGACCUUGUAAGACCCCA
   ||||||||||||:|||||||
   AAGAACUGGAACGUUCUGGGAG 5'
AT2G33860.1     1672    1693
auxin response transcription factor 3 (ETTIN/ARF3)

sRNA_AGO1_Solexa_Mi2008_7_12746_hit1
5' CUUGACCUUGUAAGACCCC
   ||||||||||:|||||||
   GAACUGGAACGUUCUGGGA 5'
AT2G33860.1     1673    1691
auxin response transcription factor 3 (ETTIN/ARF3)

sRNA_AGO1_Solexa_Mi2008_7_49852_hit1
5' UUCUUGACCUUGUAAGACCCC
   ||||||||||||:|||||||
   AAGAACUGGAACGUUCUGGGA 5'
AT2G33860.1     1673    1693
auxin response transcription factor 3 (ETTIN/ARF3)

sRNA_AGO1_Solexa_Mi2008_4_55012_hit1
5' UUUCUUGACCUUGUAAGACCCC
   |||||||||||||:|||||||
   AAAGAACUGGAACGUUCUGGGA 5'
AT2G33860.1     1673    1694
auxin response transcription factor 3 (ETTIN/ARF3)

sRNA_AGO1_Solexa_Mi2008_1_55011_hit1
5' UUUCUUGACCUUGUAAGACCC
   |||||||||||||:|||||||
   AAAGAACUGGAACGUUCUGGG 5'
AT2G33860.1     1674    1694
auxin response transcription factor 3 (ETTIN/ARF3)

sRNA_AGO1_Solexa_Mi2008_1_3_hit25
5' AAAAAAAAAAAA--AGAAAGA
```

```
   ||||||||||||  |||||:|
   UUUUUUUUUUUUGAUCUUUUU 5'
AT2G33860.1     173     193
auxin response transcription factor 3 (ETTIN/ARF3)

sRNA_AGO1_Solexa_Mi2008_1_2_hit38
5' AAA-AAAAAAAAAAAACCAU
   ||| ||||||||||||| |
   UUUCUUUUUUUUUUUUGAUC 5'
AT2G33860.1     178     197
auxin response transcription factor 3 (ETTIN/ARF3)

sRNA_AGO1_Solexa_Mi2008_7_12746_hit1
5' CUUGACCUUGUAAGACCCC
   ||||||||||:||||||
   GAACUGGAACGUUCUGGAA 5'
AT2G33860.1     1793    1811
auxin response transcription factor 3 (ETTIN/ARF3)

sRNA_AGO1_Solexa_Mi2008_7_49852_hit1
5' UUCUUGACCUUGUAAGACCCC
   ||||||||||||:||||||
   AAGAACUGGAACGUUCUGGAA 5'
AT2G33860.1     1793    1813
auxin response transcription factor 3 (ETTIN/ARF3)

sRNA_AGO1_Solexa_Mi2008_4_55012_hit1
5' UUUCUUGACCUUGUAAGACCCC
   |||||||||||||:||||||
   AAAGAACUGGAACGUUCUGGAA 5'
AT2G33860.1     1793    1814
auxin response transcription factor 3 (ETTIN/ARF3)

sRNA_AGO1_Solexa_Mi2008_1_55011_hit1
5' UUUCUUGACCUUGUAAGACCC
   |||||||||||||:||||||
   AAAGAACUGGAACGUUCUGGA 5'
AT2G33860.1     1794    1814
auxin response transcription factor 3 (ETTIN/ARF3)

sRNA_AGO1_Solexa_Mi2008_1_42435_hit1
5' UGGGGGGAGGAUACGUGUACU
   ||||||||||||||||||||
   ACCCCCCUCCUAUGCACAUGA 5'
AT2G33860.1     1901    1921
auxin response transcription factor 3 (ETTIN/ARF3)

sRNA_AGO1_Solexa_Mi2008_2_52_hit1
5' AAAAA-AGAGAGAUACAGAGAU
   ||||| ||||||| ||||||||
   UUUUUCUCUCUCUUUGUCUCUA 5'
AT2G33860.1     345     366
auxin response transcription factor 3 (ETTIN/ARF3)

sRNA_AGO1_Solexa_Mi2008_4_13855_hit1
5' GACUGAC-GACAAGAAAGAU
   ||||| | ||||||||||||
   CUGAC-GUCUGUUCUUUCUA 5'
AT2G34250.1     321     339
putative protein transport protein SEC61 alpha subunit

sRNA_AGO1_Solexa_Mi2008_4_13855_hit1
5' GACUGAC-GACAAGAAAGAU
```

```
   ||||| | ||||||||||||
   CUGAC-GUCUGUUCUUUCUA 5'
AT2G34250.2     364     382
putative protein transport protein SEC61 alpha subunit

sRNA_AGO1_Solexa_Mi2008_1_55343_hit1
5' UUUGAUUUUGUGCAAUGGCCGUUU
   ||| ||:|||||||||||| ||||
   AAAGUAGAACACGUUACCGUCAAA 5'
AT2G34357.1     2095    2118
nodulin-like protein

sRNA_AGO1_Solexa_Mi2008_2_7736_hit1
5' CCAGAAAAGAAGAAACAAU
     |||||||||||||||||
   CAUCUUUUCUUCUUUGUUA 5'
AT2G34357.1     2484    2502
nodulin-like protein

sRNA_AGO1_Solexa_Mi2008_1_18763_hit1
5' UAAUGCACAUGCUGGUGUCCC
   ||| |||||||||||| |||
   AUUCCGUGUACGACCA-AGGU 5'
AT2G34357.1     24      43
nodulin-like protein

sRNA_AGO1_Solexa_Mi2008_1_13442_hit1
5' GAAGAGAUUUAGGGAAGAUUG
   :||||| ||||||||| ||||
   UUUCUCCAAAUCCCUU-UAAC 5'
AT2G34500.1     32      51
putative cytochrome P450

sRNA_AGO1_Solexa_Mi2008_1_56384_hit12
5' UUUUCCCGCGCAAAGUGCUCGC
   |||||||||||||| ||| ||
   AAAAGGGCGCGUUU-ACG-GCU 5'
AT2G34500.1     932     951
putative cytochrome P450

sRNA_AGO1_Solexa_Mi2008_6_37563_hit1
5' UGAUUCUCUGUGUAAGCGAAA
   :||| |||||:||||||||||
   GCUA-GAGACGCAUUCGCUUU 5'
AT2G34650.1     399     418
protein kinase PINOID

sRNA_AGO1_Solexa_Mi2008_1_13420_hit2
5' GAAGAAGAAGAAGACUCUU
   |||||||||||||| | ||
   CUUCUUCUUCUUCU-A-AA 5'
AT2G34650.1     46      62
protein kinase PINOID

sRNA_AGO1_Solexa_Mi2008_11_26463_hit1
5' UAUUU-CAAGCUUCAGAUGCUCU
   ||||| || ||||||||| ||||
   AUAAAAGUCCGAAGUCUA-GAGA 5'
AT2G34650.1     841     862
protein kinase PINOID

sRNA_AGO1_Solexa_Mi2008_2_8957_hit1
5' CGGACCAGGCUUCAUCCCCCC
```

```
   ||||||||||||||||||
   GCCUGGUCCGAAGUAGGGUUA 5'
AT2G34710.1     868     888
homeodomain transcription factor (ATHB-14)

sRNA_AGO1_Solexa_Mi2008_153_14822_hit2
5' GGACCAGGCUUCAUCCCC
   |||||||||||||||||
   CCUGGUCCGAAGUAGGGUU 5'
AT2G34710.1     869     887
homeodomain transcription factor (ATHB-14)

sRNA_AGO1_Solexa_Mi2008_219_8956_hit2
5' CGGACCAGGCUUCAUCCCCC
   ||||||||||||||||||
   GCCUGGUCCGAAGUAGGGUU 5'
AT2G34710.1     869     888
homeodomain transcription factor (ATHB-14)

sRNA_AGO1_Solexa_Mi2008_1063_31727_hit2
5' UCGGACCAGGCUUCAUCCCCC
   :||||||||||||||||||
   GGCCUGGUCCGAAGUAGGGUU 5'
AT2G34710.1     869     889
homeodomain transcription factor (ATHB-14)

sRNA_AGO1_Solexa_Mi2008_4_8955_hit2
5' CGGACCAGGCUUCAUCCCC
   ||||||||||||||||||
   GCCUGGUCCGAAGUAGGGU 5'
AT2G34710.1     870     888
homeodomain transcription factor (ATHB-14)

sRNA_AGO1_Solexa_Mi2008_8_8960_hit4
5' CGGACCAGGCUUCAUUCCCCC
   |||||||||||||| ||||
   GCCUGGUCCGAAGU-AGGGUU 5'
AT2G34710.1     870     889
homeodomain transcription factor (ATHB-14)

sRNA_AGO1_Solexa_Mi2008_47_31726_hit2
5' UCGGACCAGGCUUCAUCCCC
   :||||||||||||||||||
   GGCCUGGUCCGAAGUAGGGU 5'
AT2G34710.1     870     889
homeodomain transcription factor (ATHB-14)

sRNA_AGO1_Solexa_Mi2008_310_14823_hit7
5' GGACCAGGCUUCAUUCCCC
   ||||||||||||| ||||
   CCUGGUCCGAAGU-AGGGU 5'
AT2G34710.1     871     888
homeodomain transcription factor (ATHB-14)

sRNA_AGO1_Solexa_Mi2008_641_8959_hit7
5' CGGACCAGGCUUCAUUCCCC
   |||||||||||||| ||||
   GCCUGGUCCGAAGU-AGGGU 5'
AT2G34710.1     871     889
homeodomain transcription factor (ATHB-14)

sRNA_AGO1_Solexa_Mi2008_2_31725_hit2
5' UCGGACCAGGCUUCAUCCC
```

```
   :||||||||||||||||||
   GGCCUGGUCCGAAGUAGGG 5'
AT2G34710.1     871     889
homeodomain transcription factor (ATHB-14)

sRNA_AGO1_Solexa_Mi2008_4138_31731_hit7
5' UCGGACCAGGCUUCAUUCCCC
    |||||||||||||| ||||
   CGCCUGGUCCGAAGU-AGGGU 5'
AT2G34710.1     871     890
homeodomain transcription factor (ATHB-14)

sRNA_AGO1_Solexa_Mi2008_1_8958_hit7
5' CGGACCAGGCUUCAUUCCC
   |||||||||||||| ||||
   GCCUGGUCCGAAGU-AGGG 5'
AT2G34710.1     872     889
homeodomain transcription factor (ATHB-14)

sRNA_AGO1_Solexa_Mi2008_15_11085_hit1
5' CUCGGACCAGGCUUCAUCC
    :|||||||||||||||||
   AGGCCUGGUCCGAAGUAGG 5'
AT2G34710.1     872     890
homeodomain transcription factor (ATHB-14)

sRNA_AGO1_Solexa_Mi2008_98_31730_hit7
5' UCGGACCAGGCUUCAUUCCC
    |||||||||||||| ||||
   CGCCUGGUCCGAAGU-AGGG 5'
AT2G34710.1     872     890
homeodomain transcription factor (ATHB-14)

sRNA_AGO1_Solexa_Mi2008_1_11087_hit3
5' CUCGGACCAGGCUUCAUUCCC
     |||||||||||||| ||||
   UCGCCUGGUCCGAAGU-AGGG 5'
AT2G34710.1     872     891
homeodomain transcription factor (ATHB-14)

sRNA_AGO1_Solexa_Mi2008_33_15725_hit3
5' GUCGGACCAGGCUUCAUUCCC
   : |||||||||||||| ||||
   UCGCCUGGUCCGAAGU-AGGG 5'
AT2G34710.1     872     891
homeodomain transcription factor (ATHB-14)

sRNA_AGO1_Solexa_Mi2008_68_49390_hit1
5' UUCGGACCAGGCUUCAUUCCC
     |||||||||||||| ||||
   UCGCCUGGUCCGAAGU-AGGG 5'
AT2G34710.1     872     891
homeodomain transcription factor (ATHB-14)

sRNA_AGO1_Solexa_Mi2008_6_31729_hit7
5' UCGGACCAGGCUUCAUUCC
    |||||||||||||| |||
   CGCCUGGUCCGAAGU-AGG 5'
AT2G34710.1     873     890
homeodomain transcription factor (ATHB-14)

sRNA_AGO1_Solexa_Mi2008_1_49389_hit1
5' UUCGGACCAGGCUUCAUUCC
```

```
  |||||||||||||| |||
   UCGCCUGGUCCGAAGU-AGG 5'
AT2G34710.1     873     891
homeodomain transcription factor (ATHB-14)

sRNA_AGO1_Solexa_Mi2008_2_10634_hit2
5' CUCAAGAGAAUCAACAUCCUC
   |||||||:|||||||||||
   GAGUUCUUUUAGUUGUAGGCC 5'
AT2G34730.1     105     125
putative myosin heavy chain

sRNA_AGO1_Solexa_Mi2008_3_15619_hit2
5' GUCAUAUGCUUGUCUCAAA
    |||||||||| ||||||
   AAGUAUACGAAGAGAGUUG 5'
AT2G34730.1     1496    1514
putative myosin heavy chain

sRNA_AGO1_Solexa_Mi2008_4_4686_hit27
5' CAAGAUGUGUACAGAAGCAAC
   ||| ||| ||||||||||||
   GUUGUAC-CAUGUCUUCGUUU 5'
AT2G34730.1     282     301
putative myosin heavy chain

sRNA_AGO1_Solexa_Mi2008_3_22089_hit3
5' UAGCA-GCAAGGGUAAAAUGGU
   ||||| |||||||| ||||||
   AUCGUUCGUUCCCAGUUUACCC 5'
AT2G34750.1     1748    1769

sRNA_AGO1_Solexa_Mi2008_1_40637_hit1
5' UGGAGGUGGAACGAGAG-UGCU
   |||||||||||||| || ||||
   ACCUCCACCUUGCU-UCAACGA 5'
AT2G34750.1     606     626

sRNA_AGO1_Solexa_Mi2008_3_12851_hit1
5' CUUGCCAAGUAGAAGAUCUUU
   ||||| ||||||||| |||||
   GAACGAUUCAUCUUCGAGAAA 5'
AT2G34940.1     743     763
putative vacuolar sorting receptor

sRNA_AGO1_Solexa_Mi2008_2_39871_hit2
5' UGGAAUUUUCGUGUUGGUUCA
    :||||||| |||||||||||
   UUCUUAAAAACACAACCAAGU 5'
AT2G35020.1     685     705
putative UDP-N-acetylglucosamine pyrophosphorylase

sRNA_AGO1_Solexa_Mi2008_1_39870_hit2
5' UGGAAUUUUCGUGUUGGUUC
    :||||||| ||||||||||
   UUCUUAAAAACACAACCAAG 5'
AT2G35020.1     686     705
putative UDP-N-acetylglucosamine pyrophosphorylase

sRNA_AGO1_Solexa_Mi2008_2_13419_hit2
5' GAAGAAGAAGAAGACACUU
   |||||||||||||| ||
   CUUCUUCUUCUUCU-UGUC 5'
```

```
AT2G35480.1     887     904
unknown protein

sRNA_AGO1_Solexa_Mi2008_4_21443_hit1
5' UAGAGGGUGAAAU-UCUAAUGAG
   |||||| |||| | |||||||||
   AUCUCCGACUU-AGAGAUUACUC 5'
AT2G35930.1     453     474
unknown protein

sRNA_AGO1_Solexa_Mi2008_1_56003_hit1
5' UUUGGUUCGGGUAUUGGAUU-GGUU
   |||||||||||| ||||||| ||||
   AAACCAAGCCCA-AACCUAAUCCAA 5'
AT2G36270.1     849     872
bZip transcription factor AtbZip39

sRNA_AGO1_Solexa_Mi2008_1_10194_hit1
5' CUAGCCACCGUCUGAAGAUCC
    |||| ||||:||||||||||
   UAUCG-UGGCGGACUUCUAGG 5'
AT2G36300.1     189     208
unknown protein

sRNA_AGO1_Solexa_Mi2008_6_1263_hit1
5' ACAAGAAGAGAGACGAGUAGU
   ||||||||||||| ||| |||
   UGUUCUUCUCUCUUCUC-UCA 5'
AT2G36300.1     882     901
unknown protein

sRNA_AGO1_Solexa_Mi2008_2_43059_hit1
5' UGGUCAUCUUAGCAUAGUGCU
   |||||||||||||||||||||
   ACCAGUAGAAUCGUAUCACGA 5'
AT2G36530.1     1008    1028
enolase (2-phospho-D-glycerate hydroylase)

sRNA_AGO1_Solexa_Mi2008_2_7736_hit1
5' CCAGA-AAAGAAGAAACAAU
   |||:| |||||||||||||
   GGUUUGUUUCUUCUUUGUUU 5'
AT2G36530.1     1512    1531
enolase (2-phospho-D-glycerate hydroylase)

sRNA_AGO1_Solexa_Mi2008_1_13536_hit1
5' GAAGUUCAAGUCCAUAGGUCC
   ||||||||||||  ||||||
   CUUCAAGUUCAGC-AUCCAGA 5'
AT2G36530.1     865     884
enolase (2-phospho-D-glycerate hydroylase)

sRNA_AGO1_Solexa_Mi2008_1_37996_hit1
5' UGCAGACAAGAGUG-AUACCAGA
   ||||||||||||||  ||||| |
   ACGUCUGUUCUCACACAUGGUAU 5'
AT2G36850.1     1481    1503
putative glucan synthase

sRNA_AGO1_Solexa_Mi2008_1_16322_hit2
5' UAAAACAACCCACG-GU-GCCA
    ||||||||||||| || ||||
   CUUUUGUUGGGUGCUCAUCGGU 5'
```

```
AT2G36850.1     4638    4659
putative glucan synthase

sRNA_AGO1_Solexa_Mi2008_1_49540_hit1
5' UUCGGUGUUGGUUCGACUC
   ||||||||||||  ||||
   AAGCCACAACCA--CUGAA 5'
AT2G37080.1     1755    1771
putative myosin heavy chain

sRNA_AGO1_Solexa_Mi2008_32_5888_hit1
5' CAGAAAGGUAGUUCACGAG
   || |||| ||||||||||
   GU-UUUC-AUCAAGUGCUA 5'
AT2G37080.1     2143    2159
putative myosin heavy chain

sRNA_AGO1_Solexa_Mi2008_1_50617_hit1
5' UUGAGCUAUGGAUAUCAUCUUC
   ||||||||||||||||||||||
   AACUCGAUACCUAUAGUAGAAG 5'
AT2G37080.1     94      115
putative myosin heavy chain

sRNA_AGO1_Solexa_Mi2008_1_352_hit1
5' AAAGAAGAAGAUAAAAGGA
   ||||||||||| ||||| |
   UUUCUUCUUCU-UUUUCAU 5'
AT2G37110.1     62      79
unknown protein

sRNA_AGO1_Solexa_Mi2008_1_37677_hit4
5' UGCAAAGACCAUCAAUGAUUG
   ||||| |||||||||| |||:
   ACGUUCCUGGUAGUUA-UAAU 5'
AT2G37130.1     1055    1074
putative peroxidase ATP2a

sRNA_AGO1_Solexa_Mi2008_1_37677_hit4
5' UGCAAAGACCAUCAAUGAUUG
   ||||| |||||||||| |||:
   ACGUUCCUGGUAGUUA-UAAU 5'
AT2G37130.2     955     974
putative peroxidase ATP2a

sRNA_AGO1_Solexa_Mi2008_2_7000_hit28
5' CAUCAGGUACUUCCAGCGUUCU
   |||||||| |||||| || |||
   GUAGUCCAGGAAGGU-GC-AGA 5'
AT2G37180.1     80      99
aquaporin (plasma membrane intrinsic protein 2C)

sRNA_AGO1_Solexa_Mi2008_2_411_hit5
5' AAAGAUGAAGAGAGAAAGAGA
   ||||||||||||| || |||
   UUUCUACUUCUCU-UUACUCA 5'
AT2G37400.1     91      110
unknown protein

sRNA_AGO1_Solexa_Mi2008_1_19866_hit1
5' UACCGAUUGUGUCCCUCGCAA
    ||||||||||| |||| |||
   CUGGCUAACACAAGGAG-GUU 5'
```

```
AT2G37410.2     254     273
translocase like protein

sRNA_AGO1_Solexa_Mi2008_1_19866_hit1
5' UACCGAUUGUGUCCCUCGCAA
    ||||||||||| |||| |||
   CUGGCUAACACAAGGAG-GUU 5'
AT2G37410.1     299     318
translocase like protein

sRNA_AGO1_Solexa_Mi2008_1_34620_hit1
5' UGAAGAUGAUGAUGAAGUU
   ||| |||||||||||||:|
   ACUACUACUACUACUUCGA 5'
AT2G37410.2     698     716
translocase like protein

sRNA_AGO1_Solexa_Mi2008_1_37225_hit4
5' UGAUGAUGAUGAUGAUGAAGAAG
   |||||||||||||||||| ||||
   ACUACUACUACUACUACUACUUC 5'
AT2G37410.2     700     722
translocase like protein

sRNA_AGO1_Solexa_Mi2008_9_14254_hit8
5' GAUGAUGAUGAUGAUGAUCUU
   ||||||||||||||||||
   CUACUACUACUACUACUACUU 5'
AT2G37410.2     701     721
translocase like protein

sRNA_AGO1_Solexa_Mi2008_6_14255_hit369
5' GAUGAUGAUGAUGAUGAUGAUGAU
    ||||||||||||||||||||||
   GUACUACUACUACUACUACUACUU 5'
AT2G37410.2     701     724
translocase like protein

sRNA_AGO1_Solexa_Mi2008_1_14256_hit8
5' GAUGAUGAUGAUGAUGAUGAUGUU
    |||||||||||||||||||||
   GUACUACUACUACUACUACUACUU 5'
AT2G37410.2     701     724
translocase like protein

sRNA_AGO1_Solexa_Mi2008_7_3078_hit1
5' AUGAUGAUGA-GAAUGAUGAU
   |||||||||| || ||||||
   UACUACUACUACU-ACUACUU 5'
AT2G37410.2     702     721
translocase like protein

sRNA_AGO1_Solexa_Mi2008_1_36323_hit13
5' UGAGGAUGAUGAUGAUGAUGA
   ||| ||||||||||||||||
   ACUACUACUACUACUACUACU 5'
AT2G37410.2     702     722
translocase like protein

sRNA_AGO1_Solexa_Mi2008_1_3079_hit472
5' AUGAUGAUGAUGAUGAUGAUGA
   |||||||||||||||||||||
   UACUACUACUACUACUACUACU 5'
```

```
AT2G37410.2     702     723
translocase like protein

sRNA_AGO1_Solexa_Mi2008_1_45039_hit3
5' UGUGAUGAUGAUGAUGAUGAUGA
   ::|||||||||||||||||||||
   GUACUACUACUACUACUACUACU 5'
AT2G37410.2     702     724
translocase like protein

sRNA_AGO1_Solexa_Mi2008_1_56092_hit3
5' UUUG-UGAUGAUGAUGAUGAUGA
    ||| ||||||||||||||||||
   CAACUACUACUACUACUACUACU 5'
AT2G37410.2     703     725
translocase like protein

sRNA_AGO1_Solexa_Mi2008_7_3078_hit1
5' AUGAUGAUGA-GAAUGAUGAU
   |||||||||| | ||||||||
   UACUACUACUAC-UACUACUA 5'
AT2G37410.2     705     724
translocase like protein

sRNA_AGO1_Solexa_Mi2008_1_34620_hit1
5' UGAAGAUGAUGAUGAAGUU
   ||| |||||||||||||:|
   ACUACUACUACUACUUCGA 5'
AT2G37410.1     743     761
translocase like protein

sRNA_AGO1_Solexa_Mi2008_1_37225_hit4
5' UGAUGAUGAUGAUGAUGAAGAAG
   |||||||||||||||||| ||||
   ACUACUACUACUACUACUACUUC 5'
AT2G37410.1     745     767
translocase like protein

sRNA_AGO1_Solexa_Mi2008_9_14254_hit8
5' GAUGAUGAUGAUGAUGAUCUU
   ||||||||||||||||||
   CUACUACUACUACUACUACUU 5'
AT2G37410.1     746     766
translocase like protein

sRNA_AGO1_Solexa_Mi2008_6_14255_hit369
5' GAUGAUGAUGAUGAUGAUGAUGAU
    ||||||||||||||||||||||
   GUACUACUACUACUACUACUACUU 5'
AT2G37410.1     746     769
translocase like protein

sRNA_AGO1_Solexa_Mi2008_1_14256_hit8
5' GAUGAUGAUGAUGAUGAUGAUGUU
    |||||||||||||||||||||
   GUACUACUACUACUACUACUACUU 5'
AT2G37410.1     746     769
translocase like protein

sRNA_AGO1_Solexa_Mi2008_7_3078_hit1
5' AUGAUGAUGA-GAAUGAUGAU
   |||||||||| | |||||||
   UACUACUACUAC-UACUACUU 5'
```

```
AT2G37410.1     747     766
translocase like protein

sRNA_AGO1_Solexa_Mi2008_1_36323_hit13
5' UGAGGAUGAUGAUGAUGAUGA
   ||| ||||||||||||||||
   ACUACUACUACUACUACUACU 5'
AT2G37410.1     747     767
translocase like protein

sRNA_AGO1_Solexa_Mi2008_1_3079_hit472
5' AUGAUGAUGAUGAUGAUGAUGA
   |||||||||||||||||||||
   UACUACUACUACUACUACUACU 5'
AT2G37410.1     747     768
translocase like protein

sRNA_AGO1_Solexa_Mi2008_1_45039_hit3
5' UGUGAUGAUGAUGAUGAUGAUGA
   ::|||||||||||||||||||||
   GUACUACUACUACUACUACUACU 5'
AT2G37410.1     747     769
translocase like protein

sRNA_AGO1_Solexa_Mi2008_1_56092_hit3
5' UUUG-UGAUGAUGAUGAUGAUGA
    ||| |||||||||||||||||
   CAACUACUACUACUACUACUACU 5'
AT2G37410.1     748     770
translocase like protein

sRNA_AGO1_Solexa_Mi2008_7_3078_hit1
5' AUGAUGAUGA-GAAUGAUGAU
   |||||||||| || |||||||
   UACUACUACUACU-ACUACUA 5'
AT2G37410.1     750     769
translocase like protein

sRNA_AGO1_Solexa_Mi2008_1_3728_hit1
5' AUUGGAUGCCAUG-GAAAUAGA
   |||||||| || | ||||||||
   UAACCUACUGU-CUCUUUAUCU 5'
AT2G37410.2     853     873
translocase like protein

sRNA_AGO1_Solexa_Mi2008_1_3728_hit1
5' AUUGGAUGCCAUG-GAAAUAGA
   |||||||| || | ||||||||
   UAACCUACUGU-CUCUUUAUCU 5'
AT2G37410.1     898     918
translocase like protein

sRNA_AGO1_Solexa_Mi2008_1_10933_hit5
5' CUCCUGAAUCUUCCGACAA
   ||||| | |||||||||||
   GAGGA-U-AGAAGGCUGUU 5'
AT2G37640.1     527     543
Expansin (AtEXPA3)

sRNA_AGO1_Solexa_Mi2008_2_25192_hit1
5' UAUCUUAUGCGUCCUAUCUUCC
    ||||||| | |||||||||||
   UUAGAAUAGGGAGGAUAGAAGG 5'
```

```
AT2G37640.1     531     552
Expansin (AtEXPA3)

sRNA_AGO1_Solexa_Mi2008_1_1205_hit1
5' AAUUGAAUGGACCUUUCUC
     |||||| ||||||||||
   AGAACUUAACUGGAAAGAG 5'
AT2G37710.1     580     598
putative receptor-like protein kinase

sRNA_AGO1_Solexa_Mi2008_2_50007_hit1
5' UUGAACCGCACAUGUACCGCU
   |||| |||||| ||||||||:
   AACU-GGCGUGAACAUGGCGG 5'
AT2G37800.1     206     225
hypothetical protein

sRNA_AGO1_Solexa_Mi2008_1_14_hit1
5' AAAA-AAACAGAGAACAAGAAGA
   |||| |||||||| | |||||||
   UUUUCUUUGUCUCGUAUUCUUCU 5'
AT2G38120.1     107     129
unknown protein

sRNA_AGO1_Solexa_Mi2008_3_13907_hit1
5' GAGAAGAGAUAGAAU-AGAAU
   ||||||||| ||||| ||||
   CUCUUCUCUUUCUUAGUCUUC 5'
AT2G38120.1     238     258
unknown protein

sRNA_AGO1_Solexa_Mi2008_13_35786_hit1
5' UGAGAAGAGAUAGAAU-AGAAU
   |||||||||| ||||| ||||
   ACUCUUCUCUUUCUUAGUCUUC 5'
AT2G38120.1     238     259
unknown protein

sRNA_AGO1_Solexa_Mi2008_13_13906_hit2
5' GAGAAGAGAUAGAAU-AGAA
   ||||||||| ||||| ||||
   CUCUUCUCUUUCUUAGUCUU 5'
AT2G38120.1     239     258
unknown protein

sRNA_AGO1_Solexa_Mi2008_4_35785_hit2
5' UGAGAAGAGAUAGAAU-AGAA
   |||||||||| ||||| ||||
   ACUCUUCUCUUUCUUAGUCUU 5'
AT2G38120.1     239     259
unknown protein

sRNA_AGO1_Solexa_Mi2008_40_50469_hit1
5' UU-GAGAAGAGAUAGAAU-AGAA
   || ||||||||| ||||| ||||
   AAUCUCUUCUCUUUCUUAGUCUU 5'
AT2G38120.1     239     261
unknown protein

sRNA_AGO1_Solexa_Mi2008_1_35784_hit2
5' UGAGAAGAGAUAGAAU-AGA
   |||||||||| ||||| |||
   ACUCUUCUCUUUCUUAGUCU 5'
```

```
AT2G38120.1     240     259
unknown protein

sRNA_AGO1_Solexa_Mi2008_1_34613_hit1
5' UGAAG-AUGAAGAUGAGUUGU
   ||||| |||| |||||||||
   ACUUCCUACUCCUACUCAACU 5'
AT2G38180.1     314     334
unknown protein

sRNA_AGO1_Solexa_Mi2008_1_174_hit2
5' AAAAGAAGAAGAUAAAGCAUAU
   |||||||||||| ||| ||||
   UUUUCUUCUUCUUUUUAGUAUC 5'
AT2G38290.1     1514    1535
putative ammonium transporter

sRNA_AGO1_Solexa_Mi2008_1_174_hit2
5' AAAAGAAGAAGAUAAAGCAUAU
   |||||||||||| ||| ||||
   UUUUCUUCUUCUUUUUAGUAUC 5'
AT2G38290.2     1593    1614
putative ammonium transporter

sRNA_AGO1_Solexa_Mi2008_1_17161_hit1
5' UAACAAGACCUGGCCACACAUC
   |||||||||||||| | || ||
   AUUGUUCUGGACCG-UAUG-AG 5'
AT2G38290.2     194     213
putative ammonium transporter

sRNA_AGO1_Solexa_Mi2008_1_4535_hit1
5' CAAGAAAAAACGAAGAUUUAU
    ||||||||||||| | ||||
   AUUCUUUUUUGCUU-UUAAUA 5'
AT2G38940.1     1802    1821
phosphate transporter (AtPT2)

sRNA_AGO1_Solexa_Mi2008_1_15927_hit2
5' GUGCUUGAAAUUGUCGGGAGG
   |||||||||||| |||:|||
   CACGAACUUUAA-AGCUCUCA 5'
AT2G39530.1     191     210
unknown protein

sRNA_AGO1_Solexa_Mi2008_1_5984_hit23
5' CAGA-CAGAAAAUAAAACAC
    ||| ||||||||||||| |
   AUCUUGUCUUUUAUUUUGAG 5'
AT2G39550.1     1303    1322
putative geranylgeranyl transferase type I beta subunit

sRNA_AGO1_Solexa_Mi2008_1_51471_hit6
5' UUGCCAAGAACA-UCUUCGUUA
   |||||||||||| || ||||||
   AACGGUUCUUGUAAGUAGCAAU 5'
AT2G39580.1     2569    2590
unknown protein

sRNA_AGO1_Solexa_Mi2008_1_36795_hit1
5' UG-AGUGAGAGAUUAAGAUCAU
   || || ||||||||||||| ||
   ACGUCUCUCUCUAAUUCUA-UA 5'
```

```
AT2G39580.1     4024    4044
unknown protein

sRNA_AGO1_Solexa_Mi2008_3_13421_hit1
5' GAAGAAGAAGAUGAUGUUGAU
   ||||||||||||| || |||
   CUUCUUCUUCUACAAC-ACUU 5'
AT2G39725.1     65      84
unknown protein

sRNA_AGO1_Solexa_Mi2008_1_17143_hit1
5' UAACAAAUGAUGUGGCACGUG
   |||||||||| ||||| ||||
   AUUGUUUACUUCACCGCGCAC 5'
AT2G39800.1     117     137
delta-1-pyrroline 5-carboxylase synthetase (P5C1)

sRNA_AGO1_Solexa_Mi2008_1_2355_hit1
5' AUAACAAAUGACGUGGCACGUG
    |||||||||| ||||| ||||
   GAUUGUUUACUUCACCGCGCAC 5'
AT2G39800.1     117     138
delta-1-pyrroline 5-carboxylase synthetase (P5C1)

sRNA_AGO1_Solexa_Mi2008_1_3_hit25
5' AAAAAAA-AAAA-AAGAAAGA
   ||||||| |||| |||||||
   UUUUUUUCUUUUGUUCUUUCG 5'
AT2G39820.1     789     809
putative translation initiation factor

sRNA_AGO1_Solexa_Mi2008_9_4565_hit2
5' CAAGAAGAAAUUAAGUACU
   |||||||||||| | ||||
   GUUCUUCUUUAA-UGAUGA 5'
AT2G39820.1     862     879
putative translation initiation factor

sRNA_AGO1_Solexa_Mi2008_2_47421_hit1
5' UUAGAGUGUUGAAUGUGAA
   |||||||||||| || |||
   AAUCUCACAACU-ACUCUU 5'
AT2G40750.1     213     230
unknown protein

sRNA_AGO1_Solexa_Mi2008_1_45467_hit2
5' UGUGGUAGCCACUGUA-GUGA
   ||||||||||| |||| ||||
   ACACCAUCGGUUACAUACACU 5'
AT2G40750.1     722     742
unknown protein

sRNA_AGO1_Solexa_Mi2008_4_48898_hit1
5' UUCAUGUGUUGGUGAAGGAAG
   |||||||:|||||| |||||:
   AAGUACAUAACCAC-UCCUUU 5'
AT2G40770.1     3899    3918
putative SNF2/SWI2 family transcription factor

sRNA_AGO1_Solexa_Mi2008_4_14762_hit1
5' GCUCAAAGUAUAGAA-ACGCU
   :|||||||||||||| || ||
   UGAGUUUCAUAUCUUCUG-GA 5'
```

```
AT2G40770.1     5146    5165
putative SNF2/SWI2 family transcription factor

sRNA_AGO1_Solexa_Mi2008_1_38224_hit4
5' UGCAUCAUUGGUGGUGUGAG
   ||| | ||||||||||||||
   ACG-A-UAACCACCACACUC 5'
AT2G40940.1     174     191
ethylene response sensor (ERS)

sRNA_AGO1_Solexa_Mi2008_1_4591_hit1
5' CAAGACAAGGAGUUUUACC
   ||||||||:||||||||||
   GUUCUGUUUCUCAAAAUGG 5'
AT2G40940.1     753     771
ethylene response sensor (ERS)

sRNA_AGO1_Solexa_Mi2008_1_3647_hit1
5' AUUGAAC-GAGAUGAUGAUU-GG
   ||||| | |||||||||||| ||
   UAACUAGGCUCUACUACUAAUCC 5'
AT2G40970.1     304     326
unknown protein

sRNA_AGO1_Solexa_Mi2008_1_35931_hit1
5' UGAGAGUG-AGGAGAUGAUGCGU
   |||||| | ||||:||||||||
   ACUCUC-CAUCCUUUACUACGCA 5'
AT2G41190.1     1126    1147
unknown protein

sRNA_AGO1_Solexa_Mi2008_1_20735_hit4
5' UACUUUUUCG-GGCAUUUUUGUG
   |||||||||| || |||| ||||
   AUGAAAAAGCUCC-UAAACACAC 5'
AT2G41190.1     27      48
unknown protein

sRNA_AGO1_Solexa_Mi2008_51_8316_hit10
5' CGAAGAUGGGUUUUCACG-GCGA
   ||| ||||:||||||||| ||||
   GCU-CUACUCAAAAGUGCUCGCU 5'
AT2G41220.1     4201    4222
ferredoxin-dependent glutamate synthase (GLU2)

sRNA_AGO1_Solexa_Mi2008_1_42_hit14
5' AAAAAACAGAAAACAAUGGAU
   |||||| | ||||||||||||
   UUUUUU-U-UUUUGUUACCUA 5'
AT2G41220.1     5432    5450
ferredoxin-dependent glutamate synthase (GLU2)

sRNA_AGO1_Solexa_Mi2008_5_154_hit2
5' AAA-A-CAUCAUUUACAAUCGU
   ||| | ||||||||||||| |
   UUUCUCGUAGUAAAUGUUAGAA 5'
AT2G41220.1     637     658
ferredoxin-dependent glutamate synthase (GLU2)

sRNA_AGO1_Solexa_Mi2008_1_5010_hit1
5' CAAGUGC-AUUAAGAACAUCAU
   ||||||| | ||||| ||||||
   GUUCACGAU-AUUCUCGUAGUA 5'
```

AT2G41220.1   647   667
ferredoxin-dependent glutamate synthase (GLU2)

sRNA_AGO1_Solexa_Mi2008_2_411_hit5
5' AA-AGAUGA-AGAGAGAAAGAGA
   || ||| || ||||||||||||
   UUCUCUCCUAUCUCUCUUUCUCU 5'
AT2G41640.1   295   317
unknown protein

sRNA_AGO1_Solexa_Mi2008_1_13292_hit1
5' CUUUUGUGUUG-UACUUGGUU
   ||||||||||| || |||||
   GAAAACACAACAAU-AACCAC 5'
AT2G41640.1   559   578
unknown protein

sRNA_AGO1_Solexa_Mi2008_1_45992_hit2
5' UGUUGCAAUGUUGUGAAGUUA
   ||||| ||||:||||||||||
   ACAACAUUACGACACUUCAAU 5'
AT2G41640.1   785   805
unknown protein

sRNA_AGO1_Solexa_Mi2008_1_10568_hit2
5' CUAUUUCAAGUUCAAGCA-CUCC
   || |||| |||||||||| ||||
   GA-AAAGGUCAAGUUCGUCGAGG 5'
AT2G41650.1   148   169
unknown protein

sRNA_AGO1_Solexa_Mi2008_3_53389_hit2
5' UUGUCAGGUGGGGAGU-UUG
   ||||||| |||||||| ||
   AACAGUCAACCCCUCAUAAA 5'
AT2G42300.2   231   250
putative bHLH transcription factor (bHLH048)

sRNA_AGO1_Solexa_Mi2008_3_53389_hit2
5' UUGUCAGGUGGGGAGU-UUG
   ||||||| |||||||| ||
   AACAGUCAACCCCUCAUAAA 5'
AT2G42300.1   232   251
putative bHLH transcription factor (bHLH048)

sRNA_AGO1_Solexa_Mi2008_2_411_hit5
5' AAAGAUGAAGAGAGAAAGA-GA
   ||||||||||| | |||| ||
   UUUCUACUUCUCGC-UUCUGCU 5'
AT2G42360.1   243   263
putative RING zinc finger protein

sRNA_AGO1_Solexa_Mi2008_1_189_hit14
5' AAAAGAGGAGAAGA-CAAAGUGU
    ||||||||||||| |||| |||
   GUUUCUCCUCUUCUCGUUU-ACA 5'
AT2G42890.1   154   175
MEI2-like protein (MEI2)

sRNA_AGO1_Solexa_Mi2008_3_21212_hit1
5' UAGAGAGAGAAAAAGAUGGC
   :||||||||||||| ||||
   GUCUCUCUCUUUUU-UACCU 5'

```
AT2G42890.1     25      43
MEI2-like protein (MEI2)

sRNA_AGO1_Solexa_Mi2008_1_189_hit14
5' AAAAGAGGAGAAGACAAAGU-GU
   |||||| |||||||| |||| ||
   UUUUCUACUCUUCUG-UUCAGCA 5'
AT2G43020.1     316     337
putative amine oxidase

sRNA_AGO1_Solexa_Mi2008_1_41735_hit1
5' UGGGAAGCA-AUCGUGAUGGUUU
   ||||||| | || ||||||||||
   ACCCUUC-UCUACCACUACCAAA 5'
AT2G43200.1     107     128
hypothetical protein

sRNA_AGO1_Solexa_Mi2008_6_15650_hit4
5' GUCCCACAUUGGA-AGUUUGA
    |||||||||||| | |||||
   AAGGGUGUAACCUAU-AAACU 5'
AT2G43200.1     1454    1473
hypothetical protein

sRNA_AGO1_Solexa_Mi2008_1_43556_hit2
5' UGGUGGUGAUGGUUGUAGUAG
   |||||||| |||| |||||||
   ACCACCACCACCAUCAUCAUC 5'
AT2G43200.1     222     242
hypothetical protein

sRNA_AGO1_Solexa_Mi2008_11_14876_hit4
5' GGAGG-UGGUGGUGGUGGUGGU
   :|||| ||:|||||||||||||
   UCUCCUACUACCACCACCACCA 5'
AT2G43200.1     231     252
hypothetical protein

sRNA_AGO1_Solexa_Mi2008_2_10859_hit1
5' CUCCAUCUCUCUC-UCUGCUU
   ||||||||||||| ||| |||
   GAGGUAGAGAGAGAAGA-GAA 5'
AT2G44180.1     422     441
methionine aminopeptidase-like protein

sRNA_AGO1_Solexa_Mi2008_4_54658_hit2
5' UUUCAAGAUUCCAGGCUUCAAU
   : |||| ||||||||||||||
   GUAGUUGUAAGGUCCGAAGUUA 5'
AT2G44180.1     521     542
methionine aminopeptidase-like protein

sRNA_AGO1_Solexa_Mi2008_1_40813_hit2
5' UGGAUAUAAGAGCUGGGC-UGU
   ||||| |||||||||| | |||
   ACCUAAAUUCUCGACC-GAACA 5'
AT2G44240.1     268     288
unknown protein

sRNA_AGO1_Solexa_Mi2008_1_56092_hit3
5' UUUGUGAUGAUGAUGAUGAUGA
   ||| |||||||||||||||
   AAAGACUACUACUACUACUAAG 5'
```

```
AT2G44360.1     16      37
unknown protein

sRNA_AGO1_Solexa_Mi2008_2_15682_hit2
5' GUCCUUUGAUCAGUGA-UCA
   |:||||||||||| || |||
   CGGGAAAACUAGUC-CUCAGU 5'
AT2G44730.1     1141    1160
unknown protein

sRNA_AGO1_Solexa_Mi2008_1_1776_hit10
5' AGAAAAAAAAAAAAAAA-A-AAU
   ||||| ||||||||||| | |||
   UCUUUCUUUUUUUUUUUCUGUUA 5'
AT2G45210.1     109     131
putative auxin-regulated protein

sRNA_AGO1_Solexa_Mi2008_24_21651_hit2
5' UAGAGUGUGUCUUGGUAACCG
   ||||||||||||||||| |
   AUCUCACACAGAACCAUUCGA 5'
AT2G45220.1     1030    1050
pectinesterase like protein

sRNA_AGO1_Solexa_Mi2008_1_17412_hit1
5' UAACCUGAGGGAGGAUGCUAUU
   ||||||||||:||||| |||||
   AUUGGACUCCUUCCUAAGAUAA 5'
AT2G45380.2     468     489
unknown protein

sRNA_AGO1_Solexa_Mi2008_1_1219_hit2
5' ACAAAAUCUGGUCAUGUGGUU
   ||||||||||||||||| |||
   UGUUUUAGACCAGUACA-CAA 5'
AT2G45560.2     308     327
cytochrome P450  like protein

sRNA_AGO1_Solexa_Mi2008_1_27216_hit1
5' UCAAGGGAAGCGUACUU-GUG
   ||||||||||||| ||| |||
   AGUUCCCUUCGCA-GAACCAC 5'
AT2G45560.2     366     385
cytochrome P450  like protein

sRNA_AGO1_Solexa_Mi2008_1_1219_hit2
5' ACAAAAUCUGGUCAUGUGGUU
   ||||||||||||||||| |||
   UGUUUUAGACCAGUACA-CAA 5'
AT2G45560.1     444     463
cytochrome P450  like protein

sRNA_AGO1_Solexa_Mi2008_1_27216_hit1
5' UCAAGGGAAGCGUACUU-GUG
   ||||||||||||| ||| |||
   AGUUCCCUUCGCA-GAACCAC 5'
AT2G45560.1     502     521
cytochrome P450  like protein

sRNA_AGO1_Solexa_Mi2008_6_733_hit1
5' AACUGUUCAUUAAGUAGAA
   || |||||||||||| ||
   UU-ACAAGUAAUUCA-CUA 5'
```

```
AT2G46680.2     811     827
homeodomain transcription factor  (ATHB-7)

sRNA_AGO1_Solexa_Mi2008_6_733_hit1
5' AACUGUUCAUUAAGUAGAA
   || |||||||||||| ||
   UU-ACAAGUAAUUCA-CUA 5'
AT2G46680.1     817     833
homeodomain transcription factor  (ATHB-7)

sRNA_AGO1_Solexa_Mi2008_1_13420_hit2
5' GAAGAAGAAGAAGACUCUU
   :||||||||||| || ||
   UUUCUUCUUCUUC-GA-AA 5'
AT2G46710.1     107     123
putative rac GTPase activating protein

sRNA_AGO1_Solexa_Mi2008_1_4612_hit91
5' CAAGACUUUUUUGGUGGAUUU
    ||| ||||:|||||||||||
   AUUC-GAAAGAACCACCUAAA 5'
AT2G46710.1     1446    1465
putative rac GTPase activating protein

sRNA_AGO1_Solexa_Mi2008_3_51264_hit6
5' UUGAUUGCGCAAGA-GCUUGUAU
   |||||||| || || ||||||||
   AACUAACGGGU-CUUCGAACAUA 5'
AT2G47000.1     2273    2294
putative ABC transporter

sRNA_AGO1_Solexa_Mi2008_1_14208_hit2
5' GAUCGAUAAA-CCUCUGCAUC
   ||||| |||| |||||||| |
   CUAGC-AUUUUGGAGACGUUG 5'
AT2G47000.1     3267    3286
putative ABC transporter

sRNA_AGO1_Solexa_Mi2008_3_8704_hit1
5' CGAUCGAUAAA-CCUCUGCAU
   |||||| |||| ||||||||
   GCUAGC-AUUUUGGAGACGUU 5'
AT2G47000.1     3268    3287
putative ABC transporter

sRNA_AGO1_Solexa_Mi2008_1_3_hit25
5' AAAAAAAAAAAAAGA-AAGA
   ||||||||||||||| ||
   UUUUUUUUUUUUUCUAUUAC 5'
AT2G47130.1     903     922
putative alcohol dehydrogenase

sRNA_AGO1_Solexa_Mi2008_1_50059_hit1
5' UUGAAGA-UGAUGACUCUGUUU
   | ||||| |||||||||||||
   ACCUUCUUACUACUGAGACAAA 5'
AT2G47360.1     190     211
unknown protein

sRNA_AGO1_Solexa_Mi2008_1_341_hit1
5' AAAGAAACAGAGAGGAAGAU
    |||||||||||| |||||:
   GUUCUUUGUCUCU-CUUCUG 5'
```

```
AT2G47360.1     210     228
unknown protein

sRNA_AGO1_Solexa_Mi2008_1_13420_hit2
5' GAAGAAGAAGAAGACUCUU
   |||||||||||||| | ||
   CUUCUUCUUCUUCU-A-AA 5'
AT2G47450.1     182     198
CAO chloroplast signal recognition particle chromo protein

sRNA_AGO1_Solexa_Mi2008_3_53795_hit2
5' UUGUUG-CCGUUGGGGAAGAAU
   |||||| || ||||| ||||||
   AACAACAGGAAACCC-UUCUUA 5'
AT2G47470.4     743     763
putative protein disulfide-isomerase

sRNA_AGO1_Solexa_Mi2008_7_15838_hit1
5' GUGAAGAUGAAGAAUAAUGUU
   || |||||||||||:||||||
   CA-UUCUACUUCUUGUUACAA 5'
AT2G47510.2     1680    1699
putative fumarase

sRNA_AGO1_Solexa_Mi2008_7_15838_hit1
5' GUGAAGAUGAAGAAUAAUGUU
   || |||||||||||:||||||
   CA-UUCUACUUCUUGUUACAA 5'
AT2G47510.1     1736    1755
putative fumarase

sRNA_AGO1_Solexa_Mi2008_1_41846_hit1
5' UGG-GAGCUGAGAGAA-ACGUCG
   ||| |||||||||||| ||| ||
   ACCUCUCGACUCUCUUCUGCUGC 5'
AT2G47510.2     40      62
putative fumarase

sRNA_AGO1_Solexa_Mi2008_1_41846_hit1
5' UGG-GAGCUGAGAGAA-ACGUCG
   ||| |||||||||||| ||| ||
   ACCUCUCGACUCUCUUCUGCUGC 5'
AT2G47510.1     84      106
putative fumarase

sRNA_AGO1_Solexa_Mi2008_6_44397_hit1
5' UGUAUGG-GCCACAUAGGUGGG
   |||| || |||||| |||||||
   ACAU-CCUCGGUGUCUCCACCC 5'
AT2G47770.1     489     509
unknown protein

sRNA_AGO1_Solexa_Mi2008_8_2829_hit1
5' AUCCAUGGUCCUCAGAGAGUC
    ||||||:|||||||| ||||
   GAGGUACUAGGAGUCU-UCAG 5'
AT2G47880.1     71      90
putative glutaredoxin

sRNA_AGO1_Solexa_Mi2008_1_21766_hit2
5' UAGAUCUGAC-AAUCAUUUUGA
   ||| ||| || |||||||||||
   AUC-AGAGUGGUUAGUAAAACU 5'
```

AT2G48160.1   2423   2443

sRNA_AGO1_Solexa_Mi2008_1_40978_hit1
5' UGGAUGUGGAUGAUGGUAUGAG
   ||||||||||||| || |||||
   ACCUACACCUACU-CC-UACUC 5'
AT2G48160.1   3926   3945

sRNA_AGO1_Solexa_Mi2008_1_83_hit1
5' AAAAAAUGCAAAGUA-AUCCUA
   |||||||:||||||| || |||
   UUUUUUAUGUUUCAUAUA-GAU 5'
AT3G01290.1   1069   1089
unknown protein

sRNA_AGO1_Solexa_Mi2008_1_46644_hit28
5' UUAAGUACUUUUUCGGGCAUUU
   ||||||||||||| | | ||||
   AAUUCAUGAAAAA-C-CUUAAA 5'
AT3G01290.1   133   152
unknown protein

sRNA_AGO1_Solexa_Mi2008_1_3384_hit1
5' AUGGUGAAGAUGU-UUC-UAGU
   ||||||||||||| ||| || |
   UACCACUUCUACAGAAGCAUGA 5'
AT3G01420.1   291   312
feebly like protein

sRNA_AGO1_Solexa_Mi2008_1_37225_hit4
5' UGAUGAUGAUGAUGAUGAAGAAG
    |||||:||||| ||||||||||
   CCUACUGCUACUUCUACUUCUUC 5'
AT3G01830.1   614   636
unknown protein

sRNA_AGO1_Solexa_Mi2008_6_33450_hit2
5' UCUGAUGGUGUGCAUCCCAAU
   |||||||||||| | |||||:
   AGACUACCACAC-UCGGGUUG 5'
AT3G01970.1   737   756
putative WRKY-like transcriptional regulator protein

sRNA_AGO1_Solexa_Mi2008_1_3901_hit3
5' CAAAACGAAGAAGAU-GAUGAG
   ||||||| ||||||| |||||
   GUUUUGCCUCUUCUAUCUACUA 5'
AT3G02230.1   1190   1211
reversibly glycosylated polypeptide-1

sRNA_AGO1_Solexa_Mi2008_1_14378_hit1
5' GAUUCUUGUCUUGAUU-UUGUU
   |||||||||||:| || |||||
   CUAAGAACAGAGC-AACAACAA 5'
AT3G02650.1   963   983
hypothetical protein

sRNA_AGO1_Solexa_Mi2008_17_6989_hit1
5' CAUC-AGAAGAAUCAUAGUCCGC
   |||| ||| ||||||| ||||||
   GUAGAUCU-CUUAGUAACAGGCG 5'
AT3G02870.2   167   188
myo-inositol monophosphatase like protein

```
sRNA_AGO1_Solexa_Mi2008_1_30071_hit1
5' UCCGUCUGCGGUGGUCCGAUU
   |||||||||||||||||||||
   AGGCAGACGCCACCAGGCUAA 5'
AT3G03450.1     1086    1106
RGA1-like protein

sRNA_AGO1_Solexa_Mi2008_1_55233_hit1
5' UUUGAGUAUUCGGGUCGGGUAUU
   ||| || ||||||||||||| ||
   AAAGUC-UAAGCCCAGCCCA-AA 5'
AT3G03450.1     1233    1253
RGA1-like protein

sRNA_AGO1_Solexa_Mi2008_1_6039_hit2
5' CAGAGAGUCUGAUAUCAAGAACA
   ||||||||||||||| ||| |||
   GUCUCUCAGACUAUACUUC-UGU 5'
AT3G03910.1     12      33
putative glutamate dehydrogenase

sRNA_AGO1_Solexa_Mi2008_2_13276_hit1
5' CUUUUCUCGAGCCUGUGAGUC
   |||||||||||| | ||||||
   GAAAAGAGCUCGAA-ACUCAG 5'
AT3G03910.1     132     151
putative glutamate dehydrogenase

sRNA_AGO1_Solexa_Mi2008_1_51028_hit2
5' UUGAUCAAUGGGUUUUGCA-UG
   |||||||||||||||| || ||
   AACUAGUUACCCAAAAGGUCAC 5'
AT3G03910.1     575     596
putative glutamate dehydrogenase

sRNA_AGO1_Solexa_Mi2008_1_48488_hit1
5' UUCAACUGCGGUAACAUCAGAU
   :||||||||||||| || |||
   GAGUUGACGCCAUUGAAGACUA 5'
AT3G04070.2     932     953
NAM-like protein (no apical meristem)

sRNA_AGO1_Solexa_Mi2008_1_48488_hit1
5' UUCAACUGCGGUAACAUCAGAU
   :||||||||||||| || |||
   GAGUUGACGCCAUUGAAGACUA 5'
AT3G04070.1     980     1001
NAM-like protein (no apical meristem)

sRNA_AGO1_Solexa_Mi2008_1_8652_hit2
5' CGAGUGAGUUAGGUGAG-GA
    ||||||| |||||||| ||
   ACUCACUCUAUCCACUCACU 5'
AT3G04120.1     47      66
glyceraldehyde-3-phosphate dehydrogenase C subunit (GapC)

sRNA_AGO1_Solexa_Mi2008_1_47577_hit5
5' UUAGCCAGUGAUUGUGUGGAC
   :|||| |||||||| ||||||
   GAUCG-UCACUAACUCACCUG 5'
AT3G04120.1     671     690
glyceraldehyde-3-phosphate dehydrogenase C subunit (GapC)
```

```
sRNA_AGO1_Solexa_Mi2008_6_29484_hit1
5' UCCAGGACCAAACCACGUUGU
   ||||||||||||||| ||:|
   AGGUCCUGGUUUGGUG-AAUA 5'
AT3G04210.1     1164    1183
disease resistance like protein

sRNA_AGO1_Solexa_Mi2008_1_48999_hit1
5' UUCCAGGACCAAACCACGUUG
   |||||||||||||||| ||:
   AAGGUCCUGGUUUGGUG-AAU 5'
AT3G04210.1     1165    1184
disease resistance like protein

sRNA_AGO1_Solexa_Mi2008_1_1763_hit2
5' ACUUCCAGGACCAAACCACGU
   |||||||||||||||||| |
   UGAAGGUCCUGGUUUGGUGAA 5'
AT3G04210.1     1165    1185
disease resistance like protein

sRNA_AGO1_Solexa_Mi2008_26_20692_hit2
5' UACUUCCAGGACCAAACCACGU
    |||||||||||||||||| |
   CUGAAGGUCCUGGUUUGGUGAA 5'
AT3G04210.1     1165    1186
disease resistance like protein

sRNA_AGO1_Solexa_Mi2008_12_20691_hit2
5' UACUUCCAGGACCAAACCACG
    ||||||||||||||||||
   CUGAAGGUCCUGGUUUGGUGA 5'
AT3G04210.1     1166    1186
disease resistance like protein

sRNA_AGO1_Solexa_Mi2008_2_3990_hit1
5' CAAA-CAGCAUCAGGUGGUGGU
    ||| || |||||||||||||
   AUUUCGUAGUAGUCCACCACCA 5'
AT3G04210.1     1261    1282
disease resistance like protein

sRNA_AGO1_Solexa_Mi2008_1_4534_hit3
5' CAAGA-A-AAAAAGAGAUGAG
   ||||| | ||||||||||| |
   GUUCUCUAUUUUUCUCUACAC 5'
AT3G04520.1     14      34
L-allo-threonine aldolase like protein

sRNA_AGO1_Solexa_Mi2008_2_44800_hit1
5' UGUCUACGU-UGAUGAUGUUGU
   ||||||||| | |||||||||
   ACAGAUGCAUA-UACUACAACC 5'
AT3G04520.1     965     985
L-allo-threonine aldolase like protein

sRNA_AGO1_Solexa_Mi2008_85_22179_hit1
5' UAGCCAAGGAUGACUUGCCUGU
    ||||||:||||| ||||||||
   CUCGGUUUCUACUAAACGGACA 5'
AT3G05690.1     1181    1202
putative transcription factor
```

```
sRNA_AGO1_Solexa_Mi2008_1_1014_hit1
5' AAU-AGCCAAGGAUGACUUGCCUG
   ||| ||||||:||||| |||||||
   UUACUCGGUUUCUACUAAACGGAC 5'
AT3G05690.1     1183    1206
putative transcription factor

sRNA_AGO1_Solexa_Mi2008_1_13905_hit1
5' GAGAAGAGAAAU-AGGUGGUA
   |||||||||||| ||| |||
   CUCUUCUCUUUACUCC-CCAA 5'
AT3G06190.2     136     155
unknown protein

sRNA_AGO1_Solexa_Mi2008_1_44627_hit1
5' UGUCCCUCCGCACUUCAGACC
   |||||||| ||| ||||||||
   ACAGGGAGACGUAAAGUCUGG 5'
AT3G06455.1     1339    1359

sRNA_AGO1_Solexa_Mi2008_2_10859_hit1
5' CUC-CAUCUCUCUCUCUGCUU
   ||| | ||||||||||||||
   GAGAG-AGAGAGAGAGACGAA 5'
AT3G06455.1     218     237

sRNA_AGO1_Solexa_Mi2008_1_1889_hit1
5' AGAGAGAGAGAUGAAUGUGGU
   ||||||||||||| ||| ||
   UCUCUCUCUCUAC-UAC-CCC 5'
AT3G07565.1     42      60
unknown protein

sRNA_AGO1_Solexa_Mi2008_1_5280_hit1
5' CACAAGAUAUUGUACAUAUC
   |||||||||||| |||| ||
   GUGUUCUAUAAC-UGUA-AG 5'
AT3G07565.1     791     808
unknown protein

sRNA_AGO1_Solexa_Mi2008_1_5280_hit1
5' CACAAGAUAUUGUACAUAUC
   |||||||||||| |||| ||
   GUGUUCUAUAAC-UGUA-AG 5'
AT3G07565.3     796     813
unknown protein

sRNA_AGO1_Solexa_Mi2008_1_5280_hit1
5' CACAAGAUAUUGUACAUAUC
   |||||||||||| |||| ||
   GUGUUCUAUAAC-UGUA-AG 5'
AT3G07565.2     883     900
unknown protein

sRNA_AGO1_Solexa_Mi2008_1_37779_hit1
5' UGCAAGAGUUUCAGAUGUAGAU
   |||||||||||||  |||||||
   ACGUUCUCAAAGUGGACAUCUA 5'
AT3G07660.1     1163    1184
unknown protein

sRNA_AGO1_Solexa_Mi2008_1_36861_hit1
```

```
5' UGAGUUACCAGUGGUGGUGGA
   |||||||||||||||||||||
   ACUCAAUGGUCACCACCACCU 5'
AT3G07660.1     1964    1984
unknown protein

sRNA_AGO1_Solexa_Mi2008_1_13510_hit1
5' GAA-GGGGAGAAGUAUAACAUA
   ||| ||||||||||||  ||||
   CUUACCCCUCUUCAUAG-GUAU 5'
AT3G07660.1     2092    2112
unknown protein

sRNA_AGO1_Solexa_Mi2008_1_53148_hit10
5' UUGGUUA-GUAUUGGUGAAUUGUA
   ||| ||| ||||||||| ||||||
   AACGAAUUCAUAACCACGUAACAU 5'
AT3G07660.1     2127    2150
unknown protein

sRNA_AGO1_Solexa_Mi2008_1_16916_hit1
5' UAAAGU-AC-UGUUGCCCAUGGUC
   |||||| || |||||||||||||
   AUUUCAGUGCACAACGGGUACCAC 5'
AT3G07660.1     2500    2523
unknown protein

sRNA_AGO1_Solexa_Mi2008_1_15033_hit11
5' GGCCUUG-CCCGGAUGGUAC
   |:||||| ||||||||||||
   CUGGAACAGGGCCUACCAUG 5'
AT3G07660.1     2562    2581
unknown protein

sRNA_AGO1_Solexa_Mi2008_30_5875_hit1
5' CAGAAAAGGAUGGACGAAGGA
   ||||| |||||||||| |||
   GUCUUCUCCUACCUGC-UCCC 5'
AT3G07660.1     852     871
unknown protein

sRNA_AGO1_Solexa_Mi2008_1_3_hit25
5' AAA-A-AAAAAAAAAGAAAGA
   ||| | ||||||||||||||
   UUUAUCUUUUUUUUUCUUUCG 5'
AT3G07810.2     1975    1995
putative RNA-binding protein

sRNA_AGO1_Solexa_Mi2008_1_3_hit25
5' AAA-A-AAAAAAAAAGAAAGA
   ||| | ||||||||||||||
   UUUAUCUUUUUUUUUCUUUCG 5'
AT3G07810.1     2042    2062
putative RNA-binding protein

sRNA_AGO1_Solexa_Mi2008_1_23335_hit2
5' UAGGUAAUGAUUGAGGAGGCC
   |||| ||||| ||||||||||
   AUCC-UUACUUACUCCUCCGG 5'
AT3G08720.1     1655    1674
putative ribosomal-protein S6 kinase (ATPK19)

sRNA_AGO1_Solexa_Mi2008_1_23335_hit2
```

```
5' UAGGUAAUGAUUGAGGAGGCC
   |||| ||||| ||||||||||
   AUCC-UUACUUACUCCUCCGG 5'
AT3G08720.2     1843    1862
putative ribosomal-protein S6 kinase (ATPK19)

sRNA_AGO1_Solexa_Mi2008_1_23007_hit1
5' UAGGGAAA-GUCAGACAACUGCAU
   |||||||| |||||||||||:|||
   AUCCCUUUACAGUCUGUUGAUGUA 5'
AT3G09010.1     1635    1658
putative receptor ser/thr protein kinase

sRNA_AGO1_Solexa_Mi2008_1_10886_hit7
5' CUC-CCUUGAUAUCACGGUGAC
   ||| ||||||| |||||||||
   GAGCGGAACUACAGUGCCACUU 5'
AT3G09010.1     838     859
putative receptor ser/thr protein kinase

sRNA_AGO1_Solexa_Mi2008_2_14955_hit1
5' GGCAGC-AGCUGAAGCAAGAGCA
   |||||| | ||||||||| ||||
   CCGUCGAU-GACUUCGUUGUCGU 5'
AT3G09200.2     826     847
putative 60S acidic ribosomal protein P0

sRNA_AGO1_Solexa_Mi2008_2_14955_hit1
5' GGCAGC-AGCUGAAGCAAGAGCA
   |||||| | ||||||||| ||||
   CCGUCGAU-GACUUCGUUGUCGU 5'
AT3G09200.1     925     946
putative 60S acidic ribosomal protein P0

sRNA_AGO1_Solexa_Mi2008_6_1651_hit1
5' ACGGUCAGGAACAAAAGCA
   | |||||||| ||||||||
   UACCAGUCCUCGUUUUCGU 5'
AT3G09260.1     82      100
thioglucosidase 3D precursor

sRNA_AGO1_Solexa_Mi2008_7_14062_hit1
5' GAGGAGGAGGAGGU-GA-ACA
   |||||||||||||| || ||
   CUCCUCCUCCUCCACCUAUGA 5'
AT3G09520.1     56      76
hypothetical protein

sRNA_AGO1_Solexa_Mi2008_1_18666_hit1
5' UAAUCAGAGAGAGAAAGAGGAU
    ||| |||||||||||||||
   CUUACUCUCUCUCUUUCUCCUC 5'
AT3G09780.1     32      53
putative protein kinase

sRNA_AGO1_Solexa_Mi2008_3_40895_hit1
5' UGGAUGA-UGAGAGAGAGAU
   |||| || |||||||||||
   ACCU-CUUACUCUCUCUCUU 5'
AT3G09780.1     40      58
putative protein kinase

sRNA_AGO1_Solexa_Mi2008_3_13907_hit1
```

```
5' GAGAAGAGAUAGA-AUAGAAU
    |||||||||||| ||||| |
   AUCUUCUCUAUCUCUAUCUAA 5'
AT3G09860.1     443     463
unknown protein

sRNA_AGO1_Solexa_Mi2008_13_13906_hit2
5' GAGAAGAGAUAGA-AUAGAA
    |||||||||||| |||||
   AUCUUCUCUAUCUCUAUCUA 5'
AT3G09860.1     444     463
unknown protein

sRNA_AGO1_Solexa_Mi2008_1_35784_hit2
5' UGAGAAGAGAUAGA-AUAGA
     |||||||||||| |||||
   CAUCUUCUCUAUCUCUAUCU 5'
AT3G09860.1     445     464
unknown protein

sRNA_AGO1_Solexa_Mi2008_1_2_hit38
5' AAAAAAAAAAAAAAAACCAU
   |||||||||||||||| ||
   UUUUUUUUUUUUUUUUGUUA 5'
AT3G10300.4     1119    1137
unknown protein

sRNA_AGO1_Solexa_Mi2008_1_2_hit38
5' AAAAAAAAAAAAAAAACCAU
   |||||||||||||||| ||
   UUUUUUUUUUUUUUUUGUUA 5'
AT3G10300.3     1126    1144
unknown protein

sRNA_AGO1_Solexa_Mi2008_1_2_hit38
5' AAAAAAAAAAAAAAAACCAU
   |||||||||||||||| ||
   UUUUUUUUUUUUUUUUGUUA 5'
AT3G10300.2     1339    1357
unknown protein

sRNA_AGO1_Solexa_Mi2008_17_7551_hit1
5' CCAAAAAAGGAAAGACGCU-GAU
   ||||||||||||||| || ||
   GGUUUUUUCCUUUCUG-GAUCUU 5'
AT3G10300.1     799     820
unknown protein

sRNA_AGO1_Solexa_Mi2008_1_11370_hit1
5' CUGAAGCCGAGUGAUG-UGAC
    ||||| ||||||||| ||||
   UACUUCUGCUCACUACCACUG 5'
AT3G10500.1     42      62
unknown protein

sRNA_AGO1_Solexa_Mi2008_7_44312_hit1
5' UGUAGUGAUGAUUGUGUCUAG
    ||||| | ||||||||||||
   CCAUCA-U-CUAACACAGAUC 5'
AT3G10720.2     1696    1714
pectinesterase like protein

sRNA_AGO1_Solexa_Mi2008_1_10249_hit2
```

```
5' CUA-GGCAAAGCUUAGAGGUC
   ||| |||||||||||| |||
   GAUGCCGUUUCGAAUC-CCAU 5'
AT3G10720.2     295     314
pectinesterase like protein

sRNA_AGO1_Solexa_Mi2008_7_44312_hit1
5' UGUAGUGAUGAUUGUGUCUAG
    ||||| | ||||||||||||
   CCAUCA-U-CUAACACAGAUC 5'
AT3G10720.1     895     913
pectinesterase like protein

sRNA_AGO1_Solexa_Mi2008_1_3_hit25
5' AAAA-AA-AAAAAAAGAAAGA
   |||| || |||||||||||||
   UUUUCUUGUUUUUUUCUUUCU 5'
AT3G10930.1     406     426
unknown protein

sRNA_AGO1_Solexa_Mi2008_1_3188_hit1
5' AU-GCUCUAAGUCUUGAUCGU
   || | ||||||||||||||:|
   UAUC-AGAUUCAGAACUAGUA 5'
AT3G10930.1     544     563
unknown protein

sRNA_AGO1_Solexa_Mi2008_1_43410_hit1
5' UGGUGCACAGAUUUGACGUCGC
   ||||||||||||||||||||||
   ACCACGUGUCUAAACUGCAGCG 5'
AT3G10985.1     168     189
unknown protein

sRNA_AGO1_Solexa_Mi2008_7_353_hit2
5' AAAGAAGAAGAUAAAGC-AU
    ||||||||||| |||| ||
   GUUCUUCUUCUACUUCGCUA 5'
AT3G10985.1     17      36
unknown protein

sRNA_AGO1_Solexa_Mi2008_1_173_hit2
5' AA-AAGAAGAAGAUAAAGC-AU
   || ||||||||||| |||| ||
   UUCUUCUUCUUCUACUUCGCUA 5'
AT3G10985.1     17      38
unknown protein

sRNA_AGO1_Solexa_Mi2008_1_172_hit2
5' AA-AAGAAGAAGAUAAAGCA
   || ||||||||||| ||||
   UUCUUCUUCUUCUACUUCGC 5'
AT3G10985.1     19      38
unknown protein

sRNA_AGO1_Solexa_Mi2008_1_22224_hit1
5' UAGCCCCAGAAGCCCACACCG
   |||||||||||||||||||||
   AUCGGGGUCUUCGGGUGUGGC 5'
AT3G10985.1     707     727
unknown protein

sRNA_AGO1_Solexa_Mi2008_1_2_hit38
```

5' AAAAAAAAAAAAAAAACCAU
   ||||||||||||||||| |
   UUUUUUUUUUUUUUUUGAUC 5'
AT3G10985.1   747   765
unknown protein

sRNA_AGO1_Solexa_Mi2008_1_26697_hit1
5' UCAAA-GAGGGAAAUUGGAUGAG
    |||| ||||||||||||| |||
   UGUUUACUCCCUUUAACCUUCUC 5'
AT3G11340.1   170   192
hypothetical protein

sRNA_AGO1_Solexa_Mi2008_3_37720_hit1
5' UGCAACACACUUGG-AUACGAGG
   |||||||||||||| || ||||
   ACGUUGUGUGAACCUUA-ACUCC 5'
AT3G11340.1   263   284
hypothetical protein

sRNA_AGO1_Solexa_Mi2008_38_14830_hit2
5' GGACUGAAGGGAGCUCCCU
   ||| |||||||||||||
   CCUUACUUCCCUCGAGGUC 5'
AT3G11440.1   1153   1171
transcription factor like protein (MYB65)

sRNA_AGO1_Solexa_Mi2008_19_40112_hit2
5' UGGACUGAAGGGAGCUCCCU
   |||| |||||||||||||
   ACCUUACUUCCCUCGAGGUC 5'
AT3G11440.1   1153   1172
transcription factor like protein (MYB65)

sRNA_AGO1_Solexa_Mi2008_705_52065_hit2
5' UUGGACUGAAGGGAGCUCCCU
   ||||| |||||||||||||
   AACCUUACUUCCCUCGAGGUC 5'
AT3G11440.1   1153   1173
transcription factor like protein (MYB65)

sRNA_AGO1_Solexa_Mi2008_16_52069_hit1
5' UUGGACUGAAGGGAGCUCCUU
   ||||| |||||||||||||
   AACCUUACUUCCCUCGAGGUC 5'
AT3G11440.1   1153   1173
transcription factor like protein (MYB65)

sRNA_AGO1_Solexa_Mi2008_7_3717_hit1
5' AUUGGACUGAAGGGAGCUCCCU
   |||||| |||||||||||||
   UAACCUUACUUCCCUCGAGGUC 5'
AT3G11440.1   1153   1174
transcription factor like protein (MYB65)

sRNA_AGO1_Solexa_Mi2008_3349_41018_hit1
5' UGGAUUGAAGGGAGCUCUA
   |||| ||||||||||||:|
   ACCUUACUUCCCUCGAGGU 5'
AT3G11440.1   1154   1172
transcription factor like protein (MYB65)

sRNA_AGO1_Solexa_Mi2008_1621_41020_hit1

```
5' UGGAUUGAAGGGAGCUCUU
   |||| ||||||||||||:
   ACCUUACUUCCCUCGAGGU 5'
AT3G11440.1     1154    1172
transcription factor like protein (MYB65)

sRNA_AGO1_Solexa_Mi2008_24_52064_hit2
5' UUGGACUGAAGGGAGCUCCC
   ||||| |||||||||||||
   AACCUUACUUCCCUCGAGGU 5'
AT3G11440.1     1154    1173
transcription factor like protein (MYB65)

sRNA_AGO1_Solexa_Mi2008_7_52068_hit1
5' UUGGACUGAAGGGAGCUCCU
   ||||| |||||||||||||
   AACCUUACUUCCCUCGAGGU 5'
AT3G11440.1     1154    1173
transcription factor like protein (MYB65)

sRNA_AGO1_Solexa_Mi2008_1_52243_hit1
5' UUGGAUUGAAGGGAGCUCCU
   ||||| |||||||||||||
   AACCUUACUUCCCUCGAGGU 5'
AT3G11440.1     1154    1173
transcription factor like protein (MYB65)

sRNA_AGO1_Solexa_Mi2008_3416_52245_hit1
5' UUGGAUUGAAGGGAGCUCUA
   ||||| ||||||||||||:|
   AACCUUACUUCCCUCGAGGU 5'
AT3G11440.1     1154    1173
transcription factor like protein (MYB65)

sRNA_AGO1_Solexa_Mi2008_870_52246_hit1
5' UUGGAUUGAAGGGAGCUCUU
   ||||| ||||||||||||:
   AACCUUACUUCCCUCGAGGU 5'
AT3G11440.1     1154    1173
transcription factor like protein (MYB65)

sRNA_AGO1_Solexa_Mi2008_1_3716_hit1
5' AUUGGACUGAAGGGAGCUCCC
   |||||| |||||||||||||
   UAACCUUACUUCCCUCGAGGU 5'
AT3G11440.1     1154    1174
transcription factor like protein (MYB65)

sRNA_AGO1_Solexa_Mi2008_21_12906_hit1
5' CUUGGACUGAAGGGAGCUCCC
    ||||| |||||||||||||
   UAACCUUACUUCCCUCGAGGU 5'
AT3G11440.1     1154    1174
transcription factor like protein (MYB65)

sRNA_AGO1_Solexa_Mi2008_61_55550_hit1
5' UUUGGACUGAAGGGAGCUCCU
    ||||| |||||||||||||
   UAACCUUACUUCCCUCGAGGU 5'
AT3G11440.1     1154    1174
transcription factor like protein (MYB65)

sRNA_AGO1_Solexa_Mi2008_14_55632_hit1
```

```
5' UUUGGAUUGAAGGGAGCUCCU
    ||||| ||||||||||||
   UAACCUUACUUCCCUCGAGGU 5'
AT3G11440.1     1154    1174
transcription factor like protein (MYB65)

sRNA_AGO1_Solexa_Mi2008_15_52063_hit3
5' UUGGACUGAAGGGAGCUCC
   ||||| ||||||||||||
   AACCUUACUUCCCUCGAGG 5'
AT3G11440.1     1155    1173
transcription factor like protein (MYB65)

sRNA_AGO1_Solexa_Mi2008_52_52244_hit2
5' UUGGAUUGAAGGGAGCUCU
   ||||| |||||||||||:
   AACCUUACUUCCCUCGAGG 5'
AT3G11440.1     1155    1173
transcription factor like protein (MYB65)

sRNA_AGO1_Solexa_Mi2008_9_55631_hit1
5' UUUGGAUUGAAGGGAGCUCC
    ||||| ||||||||||||
   UAACCUUACUUCCCUCGAGG 5'
AT3G11440.1     1155    1174
transcription factor like protein (MYB65)

sRNA_AGO1_Solexa_Mi2008_1201_55633_hit2
5' UUUGGAUUGAAGGGAGCUCU
    ||||| |||||||||||:
   UAACCUUACUUCCCUCGAGG 5'
AT3G11440.1     1155    1174
transcription factor like protein (MYB65)

sRNA_AGO1_Solexa_Mi2008_136_14386_hit1
5' GAUUGGACUGAAGGGAGCUCC
    |||||| ||||||||||||
   AUAACCUUACUUCCCUCGAGG 5'
AT3G11440.1     1155    1175
transcription factor like protein (MYB65)

sRNA_AGO1_Solexa_Mi2008_3_55549_hit1
5' UUUGGACUGAAGGGAGCUC
    ||||| |||||||||||
   UAACCUUACUUCCCUCGAG 5'
AT3G11440.1     1156    1174
transcription factor like protein (MYB65)

sRNA_AGO1_Solexa_Mi2008_907_55630_hit3
5' UUUGGAUUGAAGGGAGCUC
    ||||| |||||||||||
   UAACCUUACUUCCCUCGAG 5'
AT3G11440.1     1156    1174
transcription factor like protein (MYB65)

sRNA_AGO1_Solexa_Mi2008_4_13220_hit2
5' CUUUGGAUUGAAGGGAGCUC
     ||||| |||||||||||
   AUAACCUUACUUCCCUCGAG 5'
AT3G11440.1     1156    1175
transcription factor like protein (MYB65)

sRNA_AGO1_Solexa_Mi2008_1_14385_hit1
```

```
5' GAUUGGACUGAAGGGAGCUC
    |||||| ||||||||||||
   AUAACCUUACUUCCCUCGAG 5'
AT3G11440.1     1156    1175
transcription factor like protein (MYB65)

sRNA_AGO1_Solexa_Mi2008_5_13219_hit2
5' CUUUGGAUUGAAGGGAGCU
     ||||| |||||||||||
   AUAACCUUACUUCCCUCGA 5'
AT3G11440.1     1157    1175
transcription factor like protein (MYB65)

sRNA_AGO1_Solexa_Mi2008_1_34009_hit1
5' UCUUUCC-AGGUUGUUGAAAGU
   ||||| | |||||||||||| |
   AGAAACGCUCCAACAACUUUGA 5'
AT3G11540.2     1789    1810
spindly (gibberellin signal transduction protein)

sRNA_AGO1_Solexa_Mi2008_1_34009_hit1
5' UCUUUCC-AGGUUGUUGAAAGU
   ||||| | |||||||||||| |
   AGAAACGCUCCAACAACUUUGA 5'
AT3G11540.1     2610    2631
spindly (gibberellin signal transduction protein)

sRNA_AGO1_Solexa_Mi2008_2_13419_hit2
5' GAAGAAGAAGAAGACACUU
   |||||||||| ||| ||||
   CUUCUUCUUCCUCU-UGAA 5'
AT3G11720.1     1287    1304
unknown protein

sRNA_AGO1_Solexa_Mi2008_2_13419_hit2
5' GAAGAAGAAGAAGACACUU
   |||||||||| ||| ||||
   CUUCUUCUUCCUCU-UGAA 5'
AT3G11720.2     1336    1353
unknown protein

sRNA_AGO1_Solexa_Mi2008_1_1510_hit1
5' ACCACCACCACCAG-CGCCGCC
   ||||||||| || | |||||||
   UGGUGGUGGCGG-CGGCGGCGG 5'
AT3G11820.2     1104    1124
syntaxin like protein

sRNA_AGO1_Solexa_Mi2008_2_5441_hit1
5' CACCACCACCACCAG-CGCCGC
   |||||||||| || | ||||||
   GUGGUGGUGGCGG-CGGCGGCG 5'
AT3G11820.2     1105    1125
syntaxin like protein

sRNA_AGO1_Solexa_Mi2008_1_1510_hit1
5' ACCACCACCACCAGC-GCCGCC
   |||||||||||| || ||||||
   UGGUGGUGGUGG-CGGCGGCGG 5'
AT3G11820.2     1107    1127
syntaxin like protein

sRNA_AGO1_Solexa_Mi2008_2_5441_hit1
```

```
5' CACCACCACCACCAG-CGCCGC
   ||||||||||||| | ||||||
   GUGGUGGUGGUGG-CGGCGGCG 5'
AT3G11820.2     1108    1128
syntaxin like protein

sRNA_AGO1_Solexa_Mi2008_1_1510_hit1
5' ACCACCACCACCAGCGCCGCC
    |||||||||||| |||||||
   GGGUGGUGGUGGUGGCGGCGG 5'
AT3G11820.2     1109    1129
syntaxin like protein

sRNA_AGO1_Solexa_Mi2008_2_5441_hit1
5' CACCACCACCACCAGCGCCGC
   | |||||||||||| ||||||
   GGGGUGGUGGUGGUGGCGGCG 5'
AT3G11820.2     1110    1130
syntaxin like protein

sRNA_AGO1_Solexa_Mi2008_1_36082_hit1
5' UGAGCAUUUGGUGGUGUACCC
   :||| |||||||||||| |||
   GCUCCUAAACCACCACA-GGG 5'
AT3G11820.2     1161    1180
syntaxin like protein

sRNA_AGO1_Solexa_Mi2008_1_36083_hit1
5' UGAGCAUUUGGUGGUGUUCCC
   :||| ||||||||||| ||||
   GCUCCUAAACCACCAC-AGGG 5'
AT3G11820.2     1161    1180
syntaxin like protein

sRNA_AGO1_Solexa_Mi2008_3_40409_hit2
5' UGGAGCAUUUGGUGGUGUACCC
   ||||| |||||||||||| |||
   ACCUCCUAAACCACCACA-GGG 5'
AT3G11820.2     1161    1181
syntaxin like protein

sRNA_AGO1_Solexa_Mi2008_1_1510_hit1
5' ACCACCACCACCAG-CGCCGCC
   ||||||||| || | |||||||
   UGGUGGUGGCGG-CGGCGGCGG 5'
AT3G11820.1     1188    1208
syntaxin like protein

sRNA_AGO1_Solexa_Mi2008_2_5441_hit1
5' CACCACCACCACCAG-CGCCGC
   |||||||||| || | ||||||
   GUGGUGGUGGCGG-CGGCGGCG 5'
AT3G11820.1     1189    1209
syntaxin like protein

sRNA_AGO1_Solexa_Mi2008_1_1510_hit1
5' ACCACCACCACCAGC-GCCGCC
   |||||||||||| || ||||||
   UGGUGGUGGUGG-CGGCGGCGG 5'
AT3G11820.1     1191    1211
syntaxin like protein

sRNA_AGO1_Solexa_Mi2008_2_5441_hit1
```

```
5' CACCACCACCACCAG-CGCCGC
   ||||||||||||| | ||||||
   GUGGUGGUGGUGG-CGGCGGCG 5'
AT3G11820.1     1192    1212
syntaxin like protein

sRNA_AGO1_Solexa_Mi2008_1_1510_hit1
5' ACCACCACCACCAGCGCCGCC
    |||||||||||| |||||||
   GGGUGGUGGUGGUGGCGGCGG 5'
AT3G11820.1     1193    1213
syntaxin like protein

sRNA_AGO1_Solexa_Mi2008_2_5441_hit1
5' CACCACCACCACCAGCGCCGC
   | |||||||||||| ||||||
   GGGGUGGUGGUGGUGGCGGCG 5'
AT3G11820.1     1194    1214
syntaxin like protein

sRNA_AGO1_Solexa_Mi2008_1_36082_hit1
5' UGAGCAUUUGGUGGUGUACCC
   :||| |||||||||||| |||
   GCUCCUAAACCACCACA-GGG 5'
AT3G11820.1     1245    1264
syntaxin like protein

sRNA_AGO1_Solexa_Mi2008_1_36083_hit1
5' UGAGCAUUUGGUGGUGUUCCC
   :||| ||||||||||| ||||
   GCUCCUAAACCACCAC-AGGG 5'
AT3G11820.1     1245    1264
syntaxin like protein

sRNA_AGO1_Solexa_Mi2008_3_40409_hit2
5' UGGAGCAUUUGGUGGUGUACCC
   ||||| |||||||||||| |||
   ACCUCCUAAACCACCACA-GGG 5'
AT3G11820.1     1245    1265
syntaxin like protein

sRNA_AGO1_Solexa_Mi2008_3_24107_hit2
5' UAGUUGAACCUUGGGAUGGGUC
   |||| |||| ||||||| ||||
   AUCA-CUUGUAACCCUA-CCAG 5'
AT3G11910.1     297     316
ubiquitin carboxyl-terminal hydrolase like protein

sRNA_AGO1_Solexa_Mi2008_2_15140_hit2
5' GGGAAAAGUUUGUAGAGAA
   : ||||||||||:||||||
   UACUUUUCAAACGUCUCUU 5'
AT3G11910.1     3705    3723
ubiquitin carboxyl-terminal hydrolase like protein

sRNA_AGO1_Solexa_Mi2008_3_7100_hit6
5' CAU-GAAAUUGAUGUUGCGC
   ||| ||| |||||||||||
   GUAACUU-AACUACAACGCA 5'
AT3G11910.1     532     550
ubiquitin carboxyl-terminal hydrolase like protein

sRNA_AGO1_Solexa_Mi2008_1_2096_hit5
```

```
5' AGCUCCCAAAGUUGAUGUC
   ||||||||||||||| |||
   UCGAGGGUUUCAACU-CAG 5'
AT3G11964.1     3114    3131
rRNA biogenesis protein, putative, 3' partial

sRNA_AGO1_Solexa_Mi2008_1_9696_hit1
5' CUAAGUACAAGGGCCACCCGA
   |||||||||||||||||||||
   GAUUCAUGUUCCCGGUGGGCU 5'
AT3G12120.2     699     719
unknown protein

sRNA_AGO1_Solexa_Mi2008_1_9696_hit1
5' CUAAGUACAAGGGCCACCCGA
   |||||||||||||||||||||
   GAUUCAUGUUCCCGGUGGGCU 5'
AT3G12120.1     738     758
unknown protein

sRNA_AGO1_Solexa_Mi2008_1_48962_hit1
5' UUCCACAAAGAUUGAUGUUGU-AG
   |||||||||||: |||||||| ||
   AAGGUGUUUCUGUCUACAACACUC 5'
AT3G12145.1     1098    1121

sRNA_AGO1_Solexa_Mi2008_1_48961_hit1
5' UUCCACAAAGAUUGAUGUUGUA
   |||||||||||: ||||||||
   AAGGUGUUUCUGUCUACAACAC 5'
AT3G12145.1     1099    1120

sRNA_AGO1_Solexa_Mi2008_3_22957_hit1
5' UAGGCGGGCCUUAUCUAGUAUUAU
   |||||||:| ||||||||| ||||
   AUCCGCCUG-AAUAGAUCACAAUA 5'
AT3G12145.1     1310    1332

sRNA_AGO1_Solexa_Mi2008_2_33035_hit1
5' UCUAUUUAGGCGGGCCUUAUC
   ||||||||||| || ||||||
   AGAUAAAUCCG-CCUGAAUAG 5'
AT3G12145.1     1319    1338

sRNA_AGO1_Solexa_Mi2008_14_4987_hit1
5' CAAGUCGAGCAGCAAAUCAG
   | |||||||| |||||||||
   GCUCAGCUCGACGUUUAGUC 5'
AT3G12570.1     511     530
unknown protein

sRNA_AGO1_Solexa_Mi2008_14_4987_hit1
5' CAAGUCGAGCAGCAAAUCAG
   | |||||||| |||||||||
   GCUCAGCUCGACGUUUAGUC 5'
AT3G12570.3     618     637
unknown protein

sRNA_AGO1_Solexa_Mi2008_14_4987_hit1
5' CAAGUCGAGCAGCAAAUCAG
   | |||||||| |||||||||
   GCUCAGCUCGACGUUUAGUC 5'
AT3G12570.4     627     646
```

unknown protein

sRNA_AGO1_Solexa_Mi2008_14_4987_hit1
5' CAAGUCGAGCAGCAAAUCAG
   | |||||||| |||||||||
   GCUCAGCUCGACGUUUAGUC 5'
AT3G12570.2   790   809
unknown protein

sRNA_AGO1_Solexa_Mi2008_4_48532_hit1
5' UUCAAGGUGAUCAAA-GGAAGA
   ||||||||||||||| ||||:|
   AAGUUCCACUAGUUUUCCUUUU 5'
AT3G12610.1   1066   1087
unknown protein

sRNA_AGO1_Solexa_Mi2008_1_26591_hit1
5' UCAAAAGCAGAA-ACAGAUGGA
   |||||||||||| | ||| |||
   AGUUUUCGUCUUCU-UCUUCCU 5'
AT3G12720.1   909   929
myb like transcription factor

sRNA_AGO1_Solexa_Mi2008_1_826_hit13
5' AAGAUCUCAGAUGAUGAUUAU
   ||||||||||||| ||||| :
   UUCUAGAGUCUACAACUAAAG 5'
AT3G13080.4   1520   1540
ABC transporter, putative

sRNA_AGO1_Solexa_Mi2008_4_5870_hit3
5' CAGAAAACAGAAGUAAUAACAG
   |||||||||:|| |||||||||
   GUCUUUUGUUUUGAUUAUUGUC 5'
AT3G13080.4   1654   1675
ABC transporter, putative

sRNA_AGO1_Solexa_Mi2008_9_51530_hit4
5' UUGC-CAUCAUCAUAAACAGGAG
   |||| |||| ||| |||||||||
   AACGUGUAGGAGUCUUUGUCCUC 5'
AT3G13080.4   2939   2961
ABC transporter, putative

sRNA_AGO1_Solexa_Mi2008_2_568_hit154
5' AAAGUGAUGACAA-AUGAUG
   ||||||||||||| || ||
   UUUCACUACUGUUGUA-UAG 5'
AT3G13080.2   3550   3568
ABC transporter, putative

sRNA_AGO1_Solexa_Mi2008_2_568_hit154
5' AAAGUGAUGACAA-AUGAUG
   ||||||||||||| || ||
   UUUCACUACUGUUGUA-UAG 5'
AT3G13080.4   3665   3683
ABC transporter, putative

sRNA_AGO1_Solexa_Mi2008_1_50661_hit2
5' UUGAGGAGUACUCAG-CACCAA
   |||||| |||||||| || |||
   AACUCCACAUGAGUCGGU-GUU 5'
AT3G13080.2   4442   4462

ABC transporter, putative

```
sRNA_AGO1_Solexa_Mi2008_1_50661_hit2
5' UUGAGGAGUACUCAG-CACCAA
   |||||| |||||||| || |||
   AACUCCACAUGAGUCGGU-GUU 5'
AT3G13080.1     4517    4537
ABC transporter, putative

sRNA_AGO1_Solexa_Mi2008_1_50661_hit2
5' UUGAGGAGUACUCAGC-ACCAA
   |||||| ||||||||| | |||
   AACUCCACAUGAGUCGGU-GUU 5'
AT3G13080.4     4816    4836
ABC transporter, putative

sRNA_AGO1_Solexa_Mi2008_1_20871_hit4
5' UAGAAGAAAACCGCAGCG-GAC
   ||||||||||| |||||| ||
   AUCUUCUUUUGCCGUCGCUCUU 5'
AT3G13175.1     296     317
unknown protein

sRNA_AGO1_Solexa_Mi2008_1_20872_hit2
5' UAGAAGAAAACCGCAGCG-GAU
   ||||||||||| |||||| ||
   AUCUUCUUUUGCCGUCGCUCUU 5'
AT3G13175.1     296     317
unknown protein

sRNA_AGO1_Solexa_Mi2008_19_875_hit12
5' AAGCUGCUUCGGAUGGGAU
   |||||||||||||  ||||
   UUCGACGAAGCCU--CCUA 5'
AT3G13230.1     15      31
unknown protein

sRNA_AGO1_Solexa_Mi2008_43_20590_hit1
5' UACUGAUGUCAGGAGAGUGUC
   |||||||||| || |||||||
   AUGACUACAG-CC-CUCACAG 5'
AT3G13230.1     371     389
unknown protein

sRNA_AGO1_Solexa_Mi2008_1_12910_hit1
5' CUUGGAGAAGCCAGUAAAUAU
    ||| |||||||||||||||
   CAACGUCUUCGGUCAUUUAUA 5'
AT3G13940.1     894     914
unknown protein

sRNA_AGO1_Solexa_Mi2008_40_6132_hit1
5' CAGAU-AUGAAAGACCGUGAU
   ||||| |||||||||||:||
   GUCUACUACUUUCUGGCAUUA 5'
AT3G15010.2     1210    1230
RNA-binding protein

sRNA_AGO1_Solexa_Mi2008_1_36946_hit4
5' UGAUAAG-GUGACAAAGCAUGGU
   ||||| | | |||||||||||||
   ACUAU-CUCUCUGUUUCGUACCA 5'
AT3G15010.2     1698    1719
```

```
RNA-binding protein

sRNA_AGO1_Solexa_Mi2008_1_2266_hit1
5' AGUCUCAAGAAAUAUAAUG
   ||| |||||||||||||:
   UCA-AGUUCUUUAUAUUGG 5'
AT3G15010.2     2180    2197
RNA-binding protein

sRNA_AGO1_Solexa_Mi2008_1_11639_hit1
5' CUGAUUACA-AAAGUAUGAU
   ||||||||| ||| |||||
   GACUAAUGUGUUU-AUACUU 5'
AT3G15356.1     993     1011

sRNA_AGO1_Solexa_Mi2008_1_19217_hit6
5' UA-CAAUGGUGAAGUGAGGU
   || ||||||||| ||||||
   AUCGUUACCACUACACUCCC 5'
AT3G15380.1     1381    1400
unknown protein

sRNA_AGO1_Solexa_Mi2008_1_24301_hit71
5' UAUAAUA-CAAUGGUGAAGUGAG
   |||| || ||||||||| |||||
   AUAU-AUCGUUACCACUACACUC 5'
AT3G15380.1     1383    1404
unknown protein

sRNA_AGO1_Solexa_Mi2008_1_3901_hit3
5' CAAAACG-AA-GAAGAUGAUGAG
   ||||| | || ||||||||||||
   GUUUUCCGUUACUUCUACUACUC 5'
AT3G15380.1     200     222
unknown protein

sRNA_AGO1_Solexa_Mi2008_1_4253_hit1
5' CA-AAUGACGUGGUGCGUGCUG
   || || || |||||||||||||
   GUCUU-CUCCACCACGCACGAC 5'
AT3G15380.1     2120    2140
unknown protein

sRNA_AGO1_Solexa_Mi2008_1_50944_hit1
5' UUGAGUUGAUGAUUAGCAAAG
   |||||||| ||||||||||||
   AACUCAACAACUAAUCGUUUC 5'
AT3G15430.1     755     775
unknown protein

sRNA_AGO1_Solexa_Mi2008_4_46099_hit5
5' UGUUUGAGUUGAUGAUUAGCA
   : ||||||||| |||||||||
   GGAAACUCAACAACUAAUCGU 5'
AT3G15430.1     758     778
unknown protein

sRNA_AGO1_Solexa_Mi2008_1_50944_hit1
5' UUGAGUUGAUGAUUAGCAAAG
   |||||||| ||||||||||||
   AACUCAACAACUAAUCGUUUC 5'
AT3G15430.2     883     903
unknown protein
```

```
sRNA_AGO1_Solexa_Mi2008_4_46099_hit5
5' UGUUUGAGUUGAUGAUUAGCA
   : ||||||||| |||||||||
   GGAAACUCAACAACUAAUCGU 5'
AT3G15430.2     886     906
unknown protein

sRNA_AGO1_Solexa_Mi2008_2_14225_hit1
5' GAUG-AAAGAAAGGAAAAGA
   |||| ||||||||:||||||
   CUACAUUUCUUUCUUUUUCU 5'
AT3G15520.1     1477    1496
cyclophilin like protein (AtCYP37)

sRNA_AGO1_Solexa_Mi2008_1_7534_hit1
5' CAUUUGUUUAGUGUUUGUGAU
   |||||||||||||||||||||
   GUAAACAAAUCACAAACACUA 5'
AT3G15570.1     1681    1701
non-phototropic hypocotyl protein, putative

sRNA_AGO1_Solexa_Mi2008_1_7006_hit1
5' CAUCAUCAUCAUCACAGAAG
   |||||||||||||| |||
   GUAGUAGUAGUAGU-UCUGG 5'
AT3G15970.1     231     249
unknown protein

sRNA_AGO1_Solexa_Mi2008_3_14780_hit1
5' GCUGAGUUGAAGGUGAAGGA
   |||| |||||||||| ||||
   CGACGCAACUUCCAC-UCCU 5'
AT3G16050.1     156     174
putative ethylene-inducible protein

sRNA_AGO1_Solexa_Mi2008_1_22925_hit5
5' UAGGCCGUCAUCUUGUCUCUCC
   ||| || || ||||||||||||
   AUC-GG-AGAAGAACAGAGAGG 5'
AT3G16150.1     119     138
L-asparaginase  like protein

sRNA_AGO1_Solexa_Mi2008_2_3790_hit1
5' AUUGUUUCACGGUGGUUUAUG
   ||| ||||||||||||| ||
   UAA-AAAGUGCCACCAA-UAG 5'
AT3G16150.1     350     368
L-asparaginase  like protein

sRNA_AGO1_Solexa_Mi2008_1_34561_hit2
5' UGAAGAGCA--GAUGGAGCAUUU
   |||||| ||  ||||||||||||
   ACUUCUGGUGUCUACCUCGUAAA 5'
AT3G16290.1     1500    1522
putative  FtsH-like metalloprotease

sRNA_AGO1_Solexa_Mi2008_1_34805_hit1
5' UGAAGGUAAUAGAUG-GAUCAA
   ||||||||||||||| ||| |
   ACUUCCAUUAUCUACACUACUA 5'
AT3G16450.3     299     320
putative lectin
```

```
sRNA_AGO1_Solexa_Mi2008_1_34805_hit1
5' UGAAGGUAAUAGAUG-GAUCAA
   ||||||||||||||| ||| |
   ACUUCCAUUAUCUACACUACUA 5'
AT3G16450.1     378     399
putative lectin

sRNA_AGO1_Solexa_Mi2008_1_34805_hit1
5' UGAAGGUAAUAGAUG-GAUCAA
   ||||||||||||||| ||| |
   ACUUCCAUUAUCUACACUACUA 5'
AT3G16450.2     387     408
putative lectin

sRNA_AGO1_Solexa_Mi2008_1_54445_hit1
5' UUUAGGUGC-UUCAACUGCGGUA
    |||||||| ||||||||||||
   CAAUCCACGGAAGUUGACGCCAC 5'
AT3G16450.3     709     731
putative lectin

sRNA_AGO1_Solexa_Mi2008_1_54445_hit1
5' UUUAGGUGC-UUCAACUGCGGUA
    |||||||| ||||||||||||
   CAAUCCACGGAAGUUGACGCCAC 5'
AT3G16450.1     788     810
putative lectin

sRNA_AGO1_Solexa_Mi2008_1_54445_hit1
5' UUUAGGUGC-UUCAACUGCGGUA
    |||||||| ||||||||||||
   CAAUCCACGGAAGUUGACGCCAC 5'
AT3G16450.2     797     819
putative lectin

sRNA_AGO1_Solexa_Mi2008_1_7584_hit16
5' CCA-AAUGCAGAAACCCAUCUU
    || |||||||||||||| |||
   UGUGUUACGUCUUUGGGU-GAA 5'
AT3G16470.1     1557    1577
putative lectin

sRNA_AGO1_Solexa_Mi2008_1_7584_hit16
5' CCA-AAUGCAGAAACCCAUCUU
    || |||||||||||||| |||
   UGUGUUACGUCUUUGGGU-GAA 5'
AT3G16470.2     1562    1582
putative lectin

sRNA_AGO1_Solexa_Mi2008_1_10010_hit16
5' CUACUCAACUUCCGAUGUG
   ||||||||| ||||| |||
   GAUGAGUUGUAGGCU-CAC 5'
AT3G16720.1     922     939
putative RING zinc finger protein

sRNA_AGO1_Solexa_Mi2008_1_17728_hit1
5' UAAGAAAUGUGGUAGG-AGUUGUA
   ||||||||||||| || ||| |||
   AUUCUUUACACCA-CCAUCAUCAU 5'
AT3G17390.1     130     152
s-adenosylmethionine synthetase like protein
```

```
sRNA_AGO1_Solexa_Mi2008_1_3030_hit2
5' AUGAGGA-GGAAGAUGGUUUG
   ||||| | ||||||||||||
   UACUC-UACCUUCUACCAAAA 5'
AT3G17390.1     890     909
s-adenosylmethionine synthetase like protein

sRNA_AGO1_Solexa_Mi2008_7_42823_hit1
5' UGGGUUGAGGUGGAAGAUGGU
   |||||||||:|||||||||||
   ACCCAACUCUACCUUCUACCA 5'
AT3G17390.1     892     912
s-adenosylmethionine synthetase like protein

sRNA_AGO1_Solexa_Mi2008_1_15600_hit1
5' GUCAGAAAAUUAGAUAUUU
   |||||||||||  ||||||
   CAGUCUUUUAA--UAUAAA 5'
AT3G17800.2     59      75
unknown protein

sRNA_AGO1_Solexa_Mi2008_2_16482_hit2
5' UAAACAAAGUGA-UAAACCCC
   |||||||||||| ||||||
   AUUUGUUUCACUGAUUUGGCC 5'
AT3G18080.1     1641    1661
beta-glucosidase like protein

sRNA_AGO1_Solexa_Mi2008_1_21386_hit1
5' UAGAG-GAGUUCAAGUUACCAA
   | ||| ||||||||||||| ||
   AGCUCUCUCAAGUUCAAUG-UU 5'
AT3G18080.1     1678    1698
beta-glucosidase like protein

sRNA_AGO1_Solexa_Mi2008_2_36326_hit1
5' UGAGGAUGAUGGAUUUAGAUC
   |||||||||||||||| | ||
   ACUCCUACUACCUAAA-C-AG 5'
AT3G18130.1     171     189
protein kinase C-receptor/G-protein, putative

sRNA_AGO1_Solexa_Mi2008_1_242_hit3
5' AAA-AGUGAAGAGAUAGAUCGA
   ||| ||||||||||||||||:
   UUUAUCACUUCUCUAUCUAGUC 5'
AT3G18190.1     1023    1044
chaperonin subunit, putative

sRNA_AGO1_Solexa_Mi2008_5_23592_hit1
5' UAGGUUUGGAUGUCUCU-UUCA
   ||||||||||||| ||| || |
   AUCCAAACCUACAAAGACAACU 5'
AT3G18290.1     3008    3029
zinc finger protein, putative

sRNA_AGO1_Solexa_Mi2008_1_44333_hit1
5' UGUAGUUCCGUUGUC-UUCACA
   ||||||||| ||| | ||||||
   ACAUCAAGGAAAC-GCAAGUGU 5'
AT3G18290.1     3956    3976
zinc finger protein, putative
```

```
sRNA_AGO1_Solexa_Mi2008_2_13419_hit2
5' GAAGAAGAAGAAGACACUU
   |||||||||||||||||
   CUUCUUCUUCUUCUGUGUC 5'
AT3G18290.1     395     413
zinc finger protein, putative

sRNA_AGO1_Solexa_Mi2008_2_51_hit1
5' AAAAAAG-AAGAGAAACAAAGA
   ||||||| || ||||| |||||
   UUUUUUCCUU-UCUUUUUUUCU 5'
AT3G18290.1     4111    4131
zinc finger protein, putative

sRNA_AGO1_Solexa_Mi2008_2_53729_hit1
5' UUGUUAACUAUUUUACACUUC
   |||||||||||| ||||||:
   AACAAUUGAUAA-AUGUGAGA 5'
AT3G18290.1     4372    4391
zinc finger protein, putative

sRNA_AGO1_Solexa_Mi2008_5_479_hit1
5' AAAGGAAGAAGAUAUGG-AGUU
    ||| |||||||||||| ||||
   GUUC-UUCUUCUAUACCCUCAA 5'
AT3G19100.1     131     151
CDPK-related kinase

sRNA_AGO1_Solexa_Mi2008_4_10938_hit2
5' CUCCUUCU-GCAGUUGUAUGUU
     |||||| |||||||||||||
   UUGGAAGAACGUCAACAUACAA 5'
AT3G19100.1     2046    2067
CDPK-related kinase

sRNA_AGO1_Solexa_Mi2008_20_28243_hit2
5' UCAGCAAAG-GAUGGUGAGGGAC
   ||| ||| | |||||||||||||
   AGUGGUUCCUCUACCACUCCCUG 5'
AT3G19160.1     334     356
tRNA isopentenyl transferase, putative

sRNA_AGO1_Solexa_Mi2008_1_32180_hit1
5' UCGGGUUAAGUGGUAUGUGGU
   |||||||||||||||||||||
   AGCCCAAUUCACCAUACACCA 5'
AT3G20810.3     50      70
unknown protein

sRNA_AGO1_Solexa_Mi2008_1_32180_hit1
5' UCGGGUUAAGUGGUAUGUGGU
   |||||||||||||||||||||
   AGCCCAAUUCACCAUACACCA 5'
AT3G20810.2     82      102
unknown protein

sRNA_AGO1_Solexa_Mi2008_10_13861_hit1
5' GACUGAGGAUUGGACCGAA
   : |||||||||||| ||||
   UGGACUCCUAACCUAGCUU 5'
AT3G21630.1     237     255
receptor like protein kinase
```

```
sRNA_AGO1_Solexa_Mi2008_1_26584_hit1
5' UCAAAAGAAGAAA-GAUGAGAAGAU
   ||||||||||||| || ||| ||||
   AGUUUUCUUCUUUACU-CUCCUCUA 5'
AT3G21670.1     30      53
nitrate transporter

sRNA_AGO1_Solexa_Mi2008_5_29024_hit1
5' UCAUGGUUUCAAGAAUGCU
    ||||||||||||||| ||
   UGUACCAAAGUUCUUA-GA 5'
AT3G22104.1     1157    1174
hypothetical protein, 3' partial

sRNA_AGO1_Solexa_Mi2008_14_49272_hit2
5' UUCGAUGGAGAUGG-ACAAGUU
   |||||||||||||| |||||
   AAGCUACCUCUACCUUGUUCUU 5'
AT3G22104.1     1331    1352
hypothetical protein, 3' partial

sRNA_AGO1_Solexa_Mi2008_1_54841_hit3
5' UUUCC-UUUCAAAGACACGCCAC
   ||||| ||| ||||||||| |||
   AAAGGGAAA-UUUCUGUGCUGUG 5'
AT3G22350.1     605     626
hypothetical protein

sRNA_AGO1_Solexa_Mi2008_1_33308_hit1
5' UCUCUCUGUUGUGAAGUCAAA
   ||||||||||||:||:|||||
   AGAGAGACAACAUUUUAGUUU 5'
AT3G22350.1     669     689
hypothetical protein

sRNA_AGO1_Solexa_Mi2008_1_43578_hit1
5' UGGUGGUGGUGAUGAUG-UGUC
   |||||||| |||||||| |||
   ACCACCACAACUACUACCACAC 5'
AT3G22440.1     1650    1671
unknown protein

sRNA_AGO1_Solexa_Mi2008_1_49525_hit4
5' UUCGGUGGUGUUGAUUGAUC
   || |||||||||||| |||
   AA-CCACCACAACUA-CUAC 5'
AT3G22440.1     1655    1672
unknown protein

sRNA_AGO1_Solexa_Mi2008_1_1171_hit3
5' AAUGGUGCUGAUGGU-GAAGUU
   |||| |||||||||| ||| ||
   UUACAACGACUACCACCUU-AA 5'
AT3G22440.1     1671    1691
unknown protein

sRNA_AGO1_Solexa_Mi2008_19_34341_hit1
5' UGAACACCAGGA-UAGUAGCAC
   |||||||||| | ||||||||
   ACUUGUGGUC-UCAUCAUCGUC 5'
AT3G22440.1     1773    1793
unknown protein
```

```
sRNA_AGO1_Solexa_Mi2008_7_11649_hit2
5' CUGAUUGAUGAUGAUGGAUCU
    |||| |||||||||||||
   AACUA-CUACUACUACCUAGU 5'
AT3G22530.1     399     418
unknown protein

sRNA_AGO1_Solexa_Mi2008_4_42709_hit1
5' UGGGUGAUGAUGAUGAUUGAU
    |||| ||||||||||| |||
   CCCCAAUACUACUACUA-CUA 5'
AT3G22530.1     405     424
unknown protein

sRNA_AGO1_Solexa_Mi2008_1_4324_hit2
5' CAACA-AAUGCAACAGUAACGA
   ||||| || |||||||||||:|
   GUUGUGUU-CGUUGUCAUUGUU 5'
AT3G22530.1     729     749
unknown protein

sRNA_AGO1_Solexa_Mi2008_9_5443_hit29
5' CACCACCAUCACCAUCACUCG
   ||||||||:|||||||| ||:
   GUGGUGGUGGUGGUAGU-AGU 5'
AT3G22530.1     796     815
unknown protein

sRNA_AGO1_Solexa_Mi2008_4_12827_hit3
5' CUUGAUUGAUUGGAUU-AAU
    |||||||||||:||| |||
   UAACUAACUAACUUAAGUUA 5'
AT3G22845.1     120     139
unknown protein

sRNA_AGO1_Solexa_Mi2008_1_4733_hit1
5' CAAGCCCAUCAUUGAUAGUUCA
   |||||||||||||| || ||||
   GUUCGGGUAGUAAC-AUGAAGU 5'
AT3G22890.1     758     778
ATP sulfurylase like protein

sRNA_AGO1_Solexa_Mi2008_2_28866_hit1
5' UCAUCAUCGCCGUCGGUUGGA
   |||||||||||||||||||||
   AGUAGUAGCGGCAGCCAACCU 5'
AT3G22970.2     1577    1597
unknown protein

sRNA_AGO1_Solexa_Mi2008_2_28866_hit1
5' UCAUCAUCGCCGUCGGUUGGA
   |||||||||||||||||||||
   AGUAGUAGCGGCAGCCAACCU 5'
AT3G22970.1     1579    1599
unknown protein

sRNA_AGO1_Solexa_Mi2008_1_11311_hit1
5' CUGAAAAAAA-AAGAGAGGG
    ||||||||| ||||||||
   AACUUUUUUUGUUCUCUCCA 5'
AT3G22970.2     1792    1811
unknown protein
```

```
sRNA_AGO1_Solexa_Mi2008_1_11311_hit1
5' CUGAAAAAAA-AAGAGAGGG
    ||||||||| ||||||||
   AACUUUUUUUGUUCUCUCCA 5'
AT3G22970.1     1794    1813
unknown protein

sRNA_AGO1_Solexa_Mi2008_1_39984_hit1
5' UGGACCAUAGAAUGCAUUUGAU
   ||||||||||||||||||||||
   ACCUGGUAUCUUACGUAAACUA 5'
AT3G23120.1     1508    1529
disease resistance protein, putative

sRNA_AGO1_Solexa_Mi2008_1_51216_hit1
5' UUGAUGUUGGACAGAAACGAG
   |||||||||||||||||||||
   AACUACAACCUGUCUUUGCUC 5'
AT3G23120.1     2039    2059
disease resistance protein, putative

sRNA_AGO1_Solexa_Mi2008_1_16206_hit2
5' GUUGUUGCAG-UUAAAAAGC
    ||||||||| ||||||||
   GAACAACGUCAAAUUUUUCU 5'
AT3G23120.1     315     334
disease resistance protein, putative

sRNA_AGO1_Solexa_Mi2008_1_18581_hit2
5' UAAGUUGUUGCAG-UUAAAAAG
   |||| |||||||| ||||||||
   AUUCUACAACGUCAAAUUUUUC 5'
AT3G23120.1     316     337
disease resistance protein, putative

sRNA_AGO1_Solexa_Mi2008_2_54009_hit2
5' UUUAAGUUGUUGCAG-UUAAAA
   |:|||| |||||||| ||||||
   AGAUUCUACAACGUCAAAUUUU 5'
AT3G23120.1     318     339
disease resistance protein, putative

sRNA_AGO1_Solexa_Mi2008_1_13417_hit1
5' GAAGAAAUAGAUGGUAAUAGU
   |||||||||||||||||||||
   CUUCUUUAUCUACCAUUAUCA 5'
AT3G23120.1     36      56
disease resistance protein, putative

sRNA_AGO1_Solexa_Mi2008_1_32978_hit1
5' UCUAGUUGGUUUAGGUUACCU
   ||||||||||||||||||||
   AGAUCAACCAAAUCCAAUGGC 5'
AT3G23120.1     461     481
disease resistance protein, putative

sRNA_AGO1_Solexa_Mi2008_3_4691_hit4
5' CAAGAUGUUUUUCUUGUGG-GA
   ||| ||||||||||||||| ||
   GUU-UACAAAAAGAACACCACU 5'
AT3G23250.2     229     249
myb-related transcription factor like protein
```

```
sRNA_AGO1_Solexa_Mi2008_3_4691_hit4
5' CAAGAUGUUUUUCUUGUGG-GA
   ||| ||||||||||||||| ||
   GUU-UACAAAAAGAACACCACU 5'
AT3G23250.1     230     250
myb-related transcription factor like protein

sRNA_AGO1_Solexa_Mi2008_2_56220_hit2
5' UUUGUUU-CGGGUCAUAACG
   ||||||| ||| ||||||||
   AAACAAAUGCC-AGUAUUGC 5'
AT3G23390.1     179     197
putative ribosomal protein

sRNA_AGO1_Solexa_Mi2008_1_3905_hit2
5' CA-AAACGGAGAGAU-GGGUUCA
   || |||||||||||| |||||||
   GUCUUUGCCUCUCUAGCCCAAGU 5'
AT3G23640.1     105     127
alpha glucosidase like protein

sRNA_AGO1_Solexa_Mi2008_1_3905_hit2
5' CA-AAACGGAGAGAU-GGGUUCA
   || |||||||||||| |||||||
   GUCUUUGCCUCUCUAGCCCAAGU 5'
AT3G23640.2     108     130
alpha glucosidase like protein

sRNA_AGO1_Solexa_Mi2008_1_17728_hit1
5' UAAGAAAUGUGGUAGGAGUUG-UA
   ||| ||||||||||||||||| ||
   AUU-UUUACACCAUCCUCAACCAU 5'
AT3G23640.1     1742    1764
alpha glucosidase like protein

sRNA_AGO1_Solexa_Mi2008_1_17728_hit1
5' UAAGAAAUGUGGUAGGAGUUG-UA
   ||| ||||||||||||||||| ||
   AUU-UUUACACCAUCCUCAACCAU 5'
AT3G23640.2     1765    1787
alpha glucosidase like protein

sRNA_AGO1_Solexa_Mi2008_1_3119_hit1
5' AUGCAAAAGAGU-AA-GAUGGAU
   |||||||||||| || | |||||
   UACGUUUUCUCACUUUCAACCUA 5'
AT3G23640.1     2862    2884
alpha glucosidase like protein

sRNA_AGO1_Solexa_Mi2008_12_3118_hit1
5' AUGCAAAAGAGU-AA-GAUGGA
   |||||||||||| || | ||||
   UACGUUUUCUCACUUUCAACCU 5'
AT3G23640.1     2863    2884
alpha glucosidase like protein

sRNA_AGO1_Solexa_Mi2008_1_3117_hit1
5' AUGCAAAAGAGU-A-AGAUGG
   |||||||||||| | || |||
   UACGUUUUCUCACUUUCAACC 5'
AT3G23640.1     2864    2884
alpha glucosidase like protein
```

```
sRNA_AGO1_Solexa_Mi2008_1_3119_hit1
5' AUGCAAAAGAGU--AAGAUGGAU
   ||||||||||||  ||| |||||
   UACGUUUUCUCACUUUCAACCUA 5'
AT3G23640.2     2885    2907
alpha glucosidase like protein

sRNA_AGO1_Solexa_Mi2008_12_3118_hit1
5' AUGCAAAAGAGU--AAGAUGGA
   ||||||||||||  ||| ||||
   UACGUUUUCUCACUUUCAACCU 5'
AT3G23640.2     2886    2907
alpha glucosidase like protein

sRNA_AGO1_Solexa_Mi2008_1_3117_hit1
5' AUGCAAAAGAGU--AAGAUGG
   ||||||||||||  ||| |||
   UACGUUUUCUCACUUUCAACC 5'
AT3G23640.2     2887    2907
alpha glucosidase like protein

sRNA_AGO1_Solexa_Mi2008_1_1743_hit1
5' ACUGGUAUAAAGAAGGAUGGUU
   |||||||||| ||| | |||||
   UGACCAUAUUGCUU-C-ACCAA 5'
AT3G23640.1     559     578
alpha glucosidase like protein

sRNA_AGO1_Solexa_Mi2008_1_1743_hit1
5' ACUGGUAUAAAGAAGGAUGGUU
   |||||||||| ||| | |||||
   UGACCAUAUUGCUU-C-ACCAA 5'
AT3G23640.2     582     601
alpha glucosidase like protein

sRNA_AGO1_Solexa_Mi2008_1_5045_hit1
5' CAAGUUAAG-UGGAUGAUUGGU
   ||||||||| ||| ||||||||
   GUUCAAUUCUACC-ACUAACCA 5'
AT3G23990.1     1857    1877
mitochondrial chaperonin hsp60

sRNA_AGO1_Solexa_Mi2008_1_15541_hit2
5' GUAGUUGUGAUACAUGGAA-GAC
   ||||||||||| ||||||| ||
   CAUCAACACUACGUACCUUUCUU 5'
AT3G25190.1     728     750
unknown protein

sRNA_AGO1_Solexa_Mi2008_2_16_hit1
5' AAAAAAAGAGAGGGACGAA
   | ||||||||||:||||||
   UCUUUUUCUCUCUCUGCUU 5'
AT3G25620.1     24      42
membrane transporter, putative

sRNA_AGO1_Solexa_Mi2008_1_6037_hit2
5' CAGAGAGCUUUGGUGUGAUU-UU
   ||||||| ||||||||| || ||
   GUCUCUCUAAACCACAC-AACAA 5'
AT3G25620.1     3       24
membrane transporter, putative
```

```
sRNA_AGO1_Solexa_Mi2008_10_6651_hit1
5' CAGGUUAGUUAGAUCAGUGAU
   |||||||||||||||||||||
   GUCCAAUCAAUCUAGUCACUA 5'
AT3G26500.1     1046    1066
unknown protein

sRNA_AGO1_Solexa_Mi2008_1_41910_hit1
5' UGGGAUCUCAAGCGGGUUUUGGU
   |||||||||||||||||||||||
   ACCCUAGAGUUCGCCCAAAACCA 5'
AT3G26500.1     1152    1174
unknown protein

sRNA_AGO1_Solexa_Mi2008_2_17160_hit1
5' UAACAACUUCAGCUCCUUGAGU
   ||||||||||||||||||||||
   AUUGUUGAAGUCGAGGAACUCA 5'
AT3G26500.1     1190    1211
unknown protein

sRNA_AGO1_Solexa_Mi2008_2_33514_hit1
5' UCUGCCAUGAUAUCACCCCAUC
   ||||||||||||||||||||||
   AGACGGUACUAUAGUGGGGUAG 5'
AT3G26500.1     1230    1251
unknown protein

sRNA_AGO1_Solexa_Mi2008_1_823_hit1
5' AAGAUAAUUUAGCAGAAGUC
   ||||||||||||||||||||
   UUCUAUUAAAUCGUCUUCAG 5'
AT3G26500.1     1434    1453
unknown protein

sRNA_AGO1_Solexa_Mi2008_1_6741_hit1
5' CAGUGGUUUUUGUGAUUCAUA
   || |||||||||||||| ||
   GU-ACCAAAAACACUAA-UAA 5'
AT3G26500.1     5       23
unknown protein

sRNA_AGO1_Solexa_Mi2008_1_10652_hit1
5' CUCAAGUGUCGUUAG-CUCA
   ||||||| ||||||| |||
   GAGUUCAAAGCAAUCAGAGG 5'
AT3G26500.1     976     995
unknown protein

sRNA_AGO1_Solexa_Mi2008_1_45425_hit9
5' UGUGGGUA-UGGAAUUCGGAAC
   |||||||| | ||||| |||||
   ACACCCAUCA-CUUAACCCUUG 5'
AT3G27060.1     295     315
ribonucleotide reductase small subunit, putative

sRNA_AGO1_Solexa_Mi2008_2_28149_hit1
5' UCAGAGUA-GUUAUGAUUGAUA
   ||| || | |||||||||||||
   AGU-UCCUUCAAUACUAACUAU 5'
AT3G27380.2     1298    1318
succinate dehydrogenase iron-protein subunit, putative
```

```
sRNA_AGO1_Solexa_Mi2008_2_28149_hit1
5' UCAGAGUA-GUUAUGAUUGAUA
   ||| || | ||||||||||||
   AGU-UCCUUCAAUACUAACUAU 5'
AT3G27380.1     1299    1319
succinate dehydrogenase iron-protein subunit, putative

sRNA_AGO1_Solexa_Mi2008_1_28630_hit1
5' UCAGUACUUGCAGUGAU-GUUAC
   |||||||||||||| ||  ||||
   AGUCAUGAACGUCAAUAUGAAUG 5'
AT3G27380.2     1336    1358
succinate dehydrogenase iron-protein subunit, putative

sRNA_AGO1_Solexa_Mi2008_1_28630_hit1
5' UCAGUACUUGCAGUGAU-GUUAC
   |||||||||||||| ||  ||||
   AGUCAUGAACGUCAAUAUGAAUG 5'
AT3G27380.1     1337    1359
succinate dehydrogenase iron-protein subunit, putative

sRNA_AGO1_Solexa_Mi2008_1_34187_hit1
5' UGAAACUAUCGUGUCUGAUUGGUA
   ||| ||| |||||| |||||||||
   ACUCUGACAGCACAAACUAACCAU 5'
AT3G27380.1     2097    2120
succinate dehydrogenase iron-protein subunit, putative

sRNA_AGO1_Solexa_Mi2008_1_34187_hit1
5' UGAAACUAUCGUGUCUGAUUGGUA
   ||| ||| |||||| |||||||||
   ACUCUGACAGCACAAACUAACCAU 5'
AT3G27380.2     2198    2221
succinate dehydrogenase iron-protein subunit, putative

sRNA_AGO1_Solexa_Mi2008_3_39684_hit1
5' UGGAACGGAAGCUUGCGGAUC
   ||||| ||||||||:||||||
   ACCUUUCCUUCGAAUGCCUAG 5'
AT3G27380.1     2349    2369
succinate dehydrogenase iron-protein subunit, putative

sRNA_AGO1_Solexa_Mi2008_3_39684_hit1
5' UGGAACGGAAGCUUGCGGAUC
   ||||| ||||||||:||||||
   ACCUUUCCUUCGAAUGCCUAG 5'
AT3G27380.2     2450    2470
succinate dehydrogenase iron-protein subunit, putative

sRNA_AGO1_Solexa_Mi2008_2_4570_hit2
5' CAAGAAGAGAUUCCAUAGU
    ||||||||||||||||
   UUUCUUCUCUAAGGUAUAC 5'
AT3G28150.1     466     484
unknown protein (At3g28150)

sRNA_AGO1_Solexa_Mi2008_1_7324_hit1
5' CAUGGUGUAGAUUUUGAUGAA
    |||||| ||||||||||||
   CUACCACUUCUAAAACUACUU 5'
AT3G28210.1     600     620
zinc finger protein (PMZ), putative
```

```
sRNA_AGO1_Solexa_Mi2008_1_42_hit14
5' AAAAAACAGAAAACAA-UGGAU
   ||||||||||| |||| ||| |
   UUUUUUGUCUUGUGUUAACCAA 5'
AT3G28220.1     1161    1182
unknown protein

sRNA_AGO1_Solexa_Mi2008_1_16297_hit1
5' UAAAAAGUGGAGCAGAUGGUU
    ||||||||||| ||||||||
   UUUUUUCACCUCUUCUACCAA 5'
AT3G28320.1     1232    1252
At14a-like protein

sRNA_AGO1_Solexa_Mi2008_1_41650_hit1
5' UGGCUGUCCAAAGUUCUUGUAA
   |||||||||||||: ||||||
   ACCGACAGGUUUCGCGAACAUC 5'
AT3G28360.1     3232    3253
P-glycoprotein like protein

sRNA_AGO1_Solexa_Mi2008_4_13945_hit10
5' GAGAGCAACAUG-UUUACCAG
   |||||||||| | ||||||||
   CUCUCGUUGU-CUAAAUGGUC 5'
AT3G28415.1     1094    1113
putative protein

sRNA_AGO1_Solexa_Mi2008_1_5286_hit1
5' CACAAGGAAGAGUAUGUUCCU
    |||||||||||| ||||||
   UUGUUCCUUCUCACACAAGGU 5'
AT3G28415.1     3584    3604
putative protein

sRNA_AGO1_Solexa_Mi2008_1_22037_hit1
5' UAGC-A-CAAGGAAGAGUAUGUUC
   |||| | ||||||||||| |||||
   AUCGAUUGUUCCUUCUCACACAAG 5'
AT3G28415.1     3587    3610
putative protein

sRNA_AGO1_Solexa_Mi2008_2_4327_hit3
5' CAA-CAACAGCAGCCACCACCU
   ||| ||||| |||||||||||
   GUUAGUUGUAGUCGGUGGUGGU 5'
AT3G28490.1     203     224
prolyl 4-hydroxylase, putative

sRNA_AGO1_Solexa_Mi2008_1_49142_hit1
5' UUCCUCAGGAAGAUAAUGGU
   ||||||||||||| || |||
   AAGGAGUCCUUCU-UU-CCA 5'
AT3G28490.1     328     345
prolyl 4-hydroxylase, putative

sRNA_AGO1_Solexa_Mi2008_1_7688_hit1
5' CCACCACCACCUCCAGCGCCAC
   |||||||||||||||| | ||
   GGUGGUGGUGGAGGUCG-G-UG 5'
AT3G28500.1     330     349
acidic ribosomal protein P2b (rpp2b), putative
```

```
sRNA_AGO1_Solexa_Mi2008_4_13685_hit6
5' GACCAUAUGGACAAUGGGC
   :||| |||||||||||||
   UUGGAAUACCUGUUACCCU 5'
AT3G28740.1     1491    1509
cytochrome P450 like protein

sRNA_AGO1_Solexa_Mi2008_3_768_hit1
5' AAGAAGAGAGAGAAUUGAG
   |||| ||||||||||| ||
   UUCU-CUCUCUCUUAA-UC 5'
AT3G28740.1     38      54
cytochrome P450 like protein

sRNA_AGO1_Solexa_Mi2008_8_4655_hit1
5' CAAGAGUGUUUGAGAGAUA
   ||||||| || ||||||||
   GUUCUCA-AA-CUCUCUAU 5'
AT3G28740.1     72      88
cytochrome P450 like protein

sRNA_AGO1_Solexa_Mi2008_1_13420_hit2
5' GAAGAAGAAGAAGACUCUU
   |||||||||||||| | ||
   CUUCUUCUUCUUCU-A-AA 5'
AT3G29000.1     22      38
unknown protein

sRNA_AGO1_Solexa_Mi2008_3_23318_hit2
5' UAG-GUAAAAACAUCUGGUCUA
   ||| |||||| ||||||||||
   AUCACAUUUU-GUAGACCAGAG 5'
AT3G44300.1     961     981
nitrilase 2

sRNA_AGO1_Solexa_Mi2008_3_23318_hit2
5' UAG-GUAAAAACAUCUGGUCUA
   ||| |||||| ||||||||||
   AUCACAUUUU-GUAGACCAGAG 5'
AT3G44310.2     861     881
nitrilase 1

sRNA_AGO1_Solexa_Mi2008_3_23318_hit2
5' UAG-GUAAAAACAUCUGGUCUA
   ||| |||||| ||||||||||
   AUCACAUUUU-GUAGACCAGAG 5'
AT3G44310.3     979     999
nitrilase 1

sRNA_AGO1_Solexa_Mi2008_2_19350_hit1
5' UACAGAAGGGAGUGUCACUAG
   |||||||||:|||||||| ||
   AUGUCUUCCUUCACAGUG-UC 5'
AT3G45040.1     278     297
unknown protein

sRNA_AGO1_Solexa_Mi2008_2_51_hit1
5' AAAAA-AGAAGAGAAACAAAGA
   ||||| ||||| ||||||||||
   UUUUUAUCUUCACUUUGUUUCU 5'
AT3G45040.1     947     968
unknown protein
```

```
sRNA_AGO1_Solexa_Mi2008_4_3519_hit1
5' AUUACUCAGUUAUACAUAGGG
   |||||||||||||||||||||
   UAAUGAGUCAAUAUGUAUCCC 5'
AT3G45160.1     519     539
unknown protein

sRNA_AGO1_Solexa_Mi2008_3_13856_hit1
5' GACUG-ACGACCAAAUUGAU
    |||| |||||||||||| |
   AUGACCUGCUGGUUUAACAA 5'
AT3G45300.1     1090    1109
isovaleryl-CoA-dehydrogenase precursor (IVD)

sRNA_AGO1_Solexa_Mi2008_3_17155_hit6
5' UAACAACC-UAGGAACUGAUGAG
   |||||| | |||:||||||||||
   AUUGUUUGUAUCUUUGACUACUC 5'
AT3G45410.1     1941    1963
receptor-like protein kinase

sRNA_AGO1_Solexa_Mi2008_5_16910_hit1
5' UAAAGUAAGAUGGGAGAGCAGU
    |||||||||||| |||||||
   UUUUCAUUCUACCGUCUCGUCU 5'
AT3G45410.1     501     522
receptor-like protein kinase

sRNA_AGO1_Solexa_Mi2008_4_34638_hit1
5' UGAAGAUUCACUGUUGA--AAGA
   ||||||||| |||||||  ||||
   ACUUCUAAGGGACAACUACUUCU 5'
AT3G45410.1     715     737
receptor-like protein kinase

sRNA_AGO1_Solexa_Mi2008_1_36072_hit2
5' UGAGCAUGGUG-UGAUGAUGAUC
   |||| |:|||| |||||||||||
   ACUC-UGCCACCACUACUACUAG 5'
AT3G45860.1     758     779
protein kinase - like

sRNA_AGO1_Solexa_Mi2008_1_28151_hit1
5' UCAGAGUCCAGAUCCCAUCAU
   || ||:|||||||||||||||
   AG-CUUAGGUCUAGGGUAGUU 5'
AT3G46620.1     524     543
unknown protein

sRNA_AGO1_Solexa_Mi2008_6_35094_hit47
5' UGAAUGUGUUUUGGGU-GAA
   |||||||||||| ||| ||
   ACUUACACAAAA-CCAUCUA 5'
AT3G47340.2     1518    1536
glutamine-dependent asparagine synthetase

sRNA_AGO1_Solexa_Mi2008_6_35094_hit47
5' UGAAUGUGUUUUGGGU-GAA
   ||||||||||||| || ||
   ACUUACACAAAAC-CAUCUA 5'
AT3G47340.1     1519    1537
glutamine-dependent asparagine synthetase
```

```
sRNA_AGO1_Solexa_Mi2008_6_35094_hit47
5' UGAAUGUGUUUUGGGU-GAA
   |||||||||||| ||| ||
   ACUUACACAAAA-CCAUCUA 5'
AT3G47340.3     1601    1619
glutamine-dependent asparagine synthetase

sRNA_AGO1_Solexa_Mi2008_22_5333_hit1
5' CA-CA-GAGAUUGAGAACGAA
   || || ||||||||||| |||
   GUAGUACUCUAACUCUUACUU 5'
AT3G47480.1     100     120
putative calcium-binding protein

sRNA_AGO1_Solexa_Mi2008_1_12752_hit1
5' CUUGACUUGAGGUGAAGAGAA
   ||| | |||||||||||||||
   GAA-U-AACUCCACUUCUCUU 5'
AT3G47480.1     185     203
putative calcium-binding protein

sRNA_AGO1_Solexa_Mi2008_3_13421_hit1
5' GAAGAAGAAGAUGAUGUUGAU
   ||||||||||||| ||| |||
   CUUCUUCUUCUAC-ACA-CUA 5'
AT3G47510.1     145     163
putative protein

sRNA_AGO1_Solexa_Mi2008_1_17742_hit1
5' UAAGAACCUUGAUGAUGUUGA
   ||| |||||||||| |||||
   AUU-UUGGAACUACAACAACA 5'
AT3G47510.1     35      54
putative protein

sRNA_AGO1_Solexa_Mi2008_1_17741_hit1
5' UAAGAACCUUGAUGAUGUUG
   ||| |||||||||| |||||
   AUU-UUGGAACUACAACAAC 5'
AT3G47510.1     36      54
putative protein

sRNA_AGO1_Solexa_Mi2008_3_18344_hit1
5' UAAG-GUCGAGGGUGUUGAAU
   |||| |||| |||||||||||
   AUUCUCAGCGCCCACAACUUA 5'
AT3G47540.1     177     197
endochitinase-like protein

sRNA_AGO1_Solexa_Mi2008_2_13419_hit2
5' GAAGAAGAAGAAGACA-CUU
   |||||||||||||| | ||
   CUUCUUCUUCUUCU-UCGAU 5'
AT3G48390.1     120     138
unknown protein

sRNA_AGO1_Solexa_Mi2008_2_36913_hit3
5' UGAGUUU-AUCAGGUGAAUCAA
   ||| ||| |||||||||| |||
   ACU-AAACUAGUCCACUUUGUU 5'
AT3G48390.1     584     604
unknown protein
```

```
sRNA_AGO1_Solexa_Mi2008_1_28025_hit79
5' UCAGAACUCCGCAGUUAAGCG
   ||||||||||||| |||| :
   AGUCUUGAGGCGUCCAUUCAU 5'
AT3G48390.1     98      118
unknown protein

sRNA_AGO1_Solexa_Mi2008_1_37529_hit1
5' UGAUUAUGAAACUGUUGAUGUU
   ||||||||||| || | |||||
   ACUAAUACUUUUAC-A-UACAA 5'
AT3G48720.1     1503    1522
unknown protein

sRNA_AGO1_Solexa_Mi2008_2_3789_hit1
5' AUUGUUUACUCUGUAUUCUGU
    ||| || ||||||||||||
   CAACCAA-GAGACAUAAGACA 5'
AT3G48870.1     272     291
AtClpC

sRNA_AGO1_Solexa_Mi2008_4_10817_hit1
5' CUCCAAGAAAUCAGAUGCC
   ||||||||| ||||| |||
   GAGGUUCUUGAGUCU-CGG 5'
AT3G49320.1     674     691
unknown protein

sRNA_AGO1_Solexa_Mi2008_1_43922_hit1
5' UGUAAAGUGAAGAUGAAGGA
    |||||||||||| |||||
   CCAUUUCACUUCUUCUUCCG 5'
AT3G49530.1     6       25
NAC2-like protein

sRNA_AGO1_Solexa_Mi2008_11_8992_hit1
5' CGGAGGUC-CUGAAUCGUUGUU
   |||||:|| | |||||||||||
   GCCUCUAGUG-CUUAGCAACAA 5'
AT3G49590.2     135     155
unknown protein

sRNA_AGO1_Solexa_Mi2008_9_14254_hit8
5' GAUGAUGAUGAUGAUGAUCUU
   |||||:|||||||||||||
   CUACUGCUACUACUACUAGUU 5'
AT3G49590.2     18      38
unknown protein

sRNA_AGO1_Solexa_Mi2008_11_8992_hit1
5' CGGAGGUC-CUGAAUCGUUGUU
   |||||:|| | |||||||||||
   GCCUCUAGUG-CUUAGCAACAA 5'
AT3G49590.1     219     239
unknown protein

sRNA_AGO1_Solexa_Mi2008_1_7006_hit1
5' CAUCAUCAUCAUCACAGAAG
   ||||||||||||| || ||
   GUAGUAGUAGUAG-GU-UUA 5'
AT3G49590.2     541     558
unknown protein
```

```
sRNA_AGO1_Solexa_Mi2008_1_7006_hit1
5' CAUCAUCAUCAUCACAGAAG
   ||||||||||||| || ||
   GUAGUAGUAGUAG-GU-UUA 5'
AT3G49590.1     625     642
unknown protein

sRNA_AGO1_Solexa_Mi2008_1_16738_hit1
5' UAAAGAUUGUAGCAUCAAGUUG
   |||||||||||||||||| :|
   AUUUCUAACAUCGUAGUUCUGC 5'
AT3G50470.1     206     227
RPW8- like protein 3 (HR3)

sRNA_AGO1_Solexa_Mi2008_1_21351_hit8
5' UAGAGGAAAUGAGUGUGUUAUUCC
   ||||:|||||||||||||||||||
   AUCUUCUUUACUCACACAAUAAGG 5'
AT3G50480.1     1006    1029
RPW8- like protein 4 (HR4)

sRNA_AGO1_Solexa_Mi2008_1_21350_hit1
5' UAGAGGAAAUGAGUGUGAUAU
   ||||:|||||||||||| |||
   AUCUUCUUUACUCACACAAUA 5'
AT3G50480.1     1009    1029
RPW8- like protein 4 (HR4)

sRNA_AGO1_Solexa_Mi2008_1_2507_hit3
5' AUAGAAGAAAUGAGUGUGUUU
    ||||||||||||||||||
   _AUCUUCUUUACUCACACAAU 5'
AT3G50480.1     1010    1030
RPW8- like protein 4 (HR4)

sRNA_AGO1_Solexa_Mi2008_1_37358_hit1
5' UGAUGGGCCUUAUAAUGGGCAUUC
   ||||||||||||||||||||||:|
   ACUACCCGGAAUAUUACCCGUAGG 5'
AT3G50480.1     905     928
RPW8- like protein 4 (HR4)

sRNA_AGO1_Solexa_Mi2008_1_37374_hit5
5' UGAUGGGUCUUAUAAUGGGCAUCC
   |||||||:||||||||||||||||
   ACUACCCGGAAUAUUACCCGUAGG 5'
AT3G50480.1     905     928
RPW8- like protein 4 (HR4)

sRNA_AGO1_Solexa_Mi2008_1_42681_hit5
5' UGGGUCUUAUAAUGGGCAU
   ||||:||||||||||||||
   ACCCGGAAUAUUACCCGUA 5'
AT3G50480.1     907     925
RPW8- like protein 4 (HR4)

sRNA_AGO1_Solexa_Mi2008_1_37373_hit5
5' UGAUGGGUCUUAUAAUGGGCAU
   |||||||:||||||||||||||
   ACUACCCGGAAUAUUACCCGUA 5'
AT3G50480.1     907     928
RPW8- like protein 4 (HR4)
```

```
sRNA_AGO1_Solexa_Mi2008_1_37356_hit1
5' UGAUGGGCCUCAUAAUGGGCA
   |||||||||| ||||||||||
   ACUACCCGGAAUAUUACCCGU 5'
AT3G50480.1     908     928
RPW8- like protein 4 (HR4)

sRNA_AGO1_Solexa_Mi2008_5_37357_hit26
5' UGAUGGGCCUUAUAAUGGGCA
   |||||||||||||||||||||
   ACUACCCGGAAUAUUACCCGU 5'
AT3G50480.1     908     928
RPW8- like protein 4 (HR4)

sRNA_AGO1_Solexa_Mi2008_1_12412_hit2
5' CUUAGAUAAUGGGCCUUAUAA
   ||||||| |||||||||||||
   GAAUCUACUACCCGGAAUAUU 5'
AT3G50480.1     914     934
RPW8- like protein 4 (HR4)

sRNA_AGO1_Solexa_Mi2008_1_42239_hit4
5' UGGGCUUAGAUGAUGGGUCUUAU
   |||||||||||||||||:|||||
   ACCCGAAUCUACUACCCGGAAUA 5'
AT3G50480.1     916     938
RPW8- like protein 4 (HR4)

sRNA_AGO1_Solexa_Mi2008_2_42236_hit1
5' UGGGCUUAGAUGAUGGACCUU
   |||||||||||||||| ||||
   ACCCGAAUCUACUACCCGGAA 5'
AT3G50480.1     918     938
RPW8- like protein 4 (HR4)

sRNA_AGO1_Solexa_Mi2008_2_42238_hit4
5' UGGGCUUAGAUGAUGGGUCUU
   |||||||||||||||||:|||
   ACCCGAAUCUACUACCCGGAA 5'
AT3G50480.1     918     938
RPW8- like protein 4 (HR4)

sRNA_AGO1_Solexa_Mi2008_2_42237_hit4
5' UGGGCUUAGAUGAUGGGUCU
   |||||||||||||||||:||
   ACCCGAAUCUACUACCCGGA 5'
AT3G50480.1     919     938
RPW8- like protein 4 (HR4)

sRNA_AGO1_Solexa_Mi2008_1_14303_hit4
5' GAUGGGCUUAGAUGAUGGGUCU
   |||||||||||||||||||:||
   CUACCCGAAUCUACUACCCGGA 5'
AT3G50480.1     919     940
RPW8- like protein 4 (HR4)

sRNA_AGO1_Solexa_Mi2008_4_37363_hit4
5' UGAUGGGCUUAGAUGAUGGGU
   ||||||||||||||||||||:
   ACUACCCGAAUCUACUACCCG 5'
AT3G50480.1     921     941
RPW8- like protein 4 (HR4)
```

```
sRNA_AGO1_Solexa_Mi2008_1_37364_hit1
5' UGAUGGGCUUAGAUGAUUGGC
   ||||||||||||||| |||
   ACUACCCGAAUCUACU-ACCC 5'
AT3G50480.1     923     942
RPW8- like protein 4 (HR4)

sRNA_AGO1_Solexa_Mi2008_3_14257_hit30
5' GAUGAUGGGCUUAGAUGAU
   |||||||||||||||||||
   CUACUACCCGAAUCUACUA 5'
AT3G50480.1     925     943
RPW8- like protein 4 (HR4)

sRNA_AGO1_Solexa_Mi2008_1_21813_hit1
5' UAGAUGAUGGACUUCGAUGAU
   |||||||||| ||| ||||||
   AUCUACUACCCGAAUCUACUA 5'
AT3G50480.1     925     945
RPW8- like protein 4 (HR4)

sRNA_AGO1_Solexa_Mi2008_6_21815_hit1
5' UAGAUGAUGGGCUUAAAUGAU
   ||||||||||||||| |||||
   AUCUACUACCCGAAUCUACUA 5'
AT3G50480.1     925     945
RPW8- like protein 4 (HR4)

sRNA_AGO1_Solexa_Mi2008_5_21816_hit30
5' UAGAUGAUGGGCUUAGAUGAU
   |||||||||||||||||||||
   AUCUACUACCCGAAUCUACUA 5'
AT3G50480.1     925     945
RPW8- like protein 4 (HR4)

sRNA_AGO1_Solexa_Mi2008_1_21817_hit1
5' UAGAUGAUGGGUUUAGAUGAU
   |||||||||||:|||||||||
   AUCUACUACCCGAAUCUACUA 5'
AT3G50480.1     925     945
RPW8- like protein 4 (HR4)

sRNA_AGO1_Solexa_Mi2008_1_10162_hit2
5' CUAGAUGAUGGACUUAGAUGA
   ||||||||||| |||||||||
   GAUCUACUACCCGAAUCUACU 5'
AT3G50480.1     926     946
RPW8- like protein 4 (HR4)

sRNA_AGO1_Solexa_Mi2008_6_10164_hit27
5' CUAGAUGAUGGGCUUAGAUGA
   |||||||||||||||||||||
   GAUCUACUACCCGAAUCUACU 5'
AT3G50480.1     926     946
RPW8- like protein 4 (HR4)

sRNA_AGO1_Solexa_Mi2008_1_32880_hit27
5' UCUAGAUGAUGGGCUUAGAUGA
   ||||||||||||||||||||||
   AGAUCUACUACCCGAAUCUACU 5'
AT3G50480.1     926     947
RPW8- like protein 4 (HR4)
```

```
sRNA_AGO1_Solexa_Mi2008_7_10163_hit27
5' CUAGAUGAUGGGCUUAGAUG
   ||||||||||||||||||||
   GAUCUACUACCCGAAUCUAC 5'
AT3G50480.1     927     946
RPW8- like protein 4 (HR4)

sRNA_AGO1_Solexa_Mi2008_2_32879_hit27
5' UCUAGAUGAUGGGCUUAGAUG
   |||||||||||||||||||||
   AGAUCUACUACCCGAAUCUAC 5'
AT3G50480.1     927     947
RPW8- like protein 4 (HR4)

sRNA_AGO1_Solexa_Mi2008_1_20757_hit22
5' UAGAAACAUCUAGAUGAUGGGCUU
   ||||||||||||||||||||||||
   AUCUUUGUAGAUCUACUACCCGAA 5'
AT3G50480.1     932     955
RPW8- like protein 4 (HR4)

sRNA_AGO1_Solexa_Mi2008_1_19030_hit3
5' UACAAACAUCUAGAUGAUGGGCU
   || ||||||||||||||||||||
   AUCUUUGUAGAUCUACUACCCGA 5'
AT3G50480.1     933     955
RPW8- like protein 4 (HR4)

sRNA_AGO1_Solexa_Mi2008_5_10081_hit25
5' CUAGAAACAUCUAGAUGAUGG
   |||||||||||||||||||||
   GAUCUUUGUAGAUCUACUACC 5'
AT3G50480.1     936     956
RPW8- like protein 4 (HR4)

sRNA_AGO1_Solexa_Mi2008_4_14880_hit24
5' GGAGUACAAGGAAAGGGUA
   |||||||||||||||||||
   CCUCAUGUUCCUUUCCCAU 5'
AT3G50480.1     962     980
RPW8- like protein 4 (HR4)

sRNA_AGO1_Solexa_Mi2008_1_23502_hit2
5' UAGGUGUACAAGGAAAGGGUA
   |||| ||||||||||||||||
   AUCCUCAUGUUCCUUUCCCAU 5'
AT3G50480.1     962     982
RPW8- like protein 4 (HR4)

sRNA_AGO1_Solexa_Mi2008_5_24584_hit2
5' UAUAGGUGUACAAGGAAAGGGU
   |||||| |||||||||||||||
   AUAUCCUCAUGUUCCUUUCCCA 5'
AT3G50480.1     963     984
RPW8- like protein 4 (HR4)

sRNA_AGO1_Solexa_Mi2008_1_22768_hit2
5' UAGGAGUACAAGGAAAAGGGU
   ||||||||||||||| |||||
   AUCCUCAUGUUCCUU-UCCCA 5'
AT3G50480.1     964     983
RPW8- like protein 4 (HR4)
```

```
sRNA_AGO1_Solexa_Mi2008_1_2590_hit4
5' AUAGGAGUACAAGGAAAAGG
   |||||||||||||||| |||
   UAUCCUCAUGUUCCUU-UCC 5'
AT3G50480.1     966     984
RPW8- like protein 4 (HR4)

sRNA_AGO1_Solexa_Mi2008_1_54542_hit29
5' UUUAUAUAGGAGUACAAGGAA
   |||||||||||||||||||||
   AAAUAUAUCCUCAUGUUCCUU 5'
AT3G50480.1     968     988
RPW8- like protein 4 (HR4)

sRNA_AGO1_Solexa_Mi2008_1_21149_hit1
5' UAG-AGAAAAAGGUAGGCAUGGG
   ||| |||||||| |||||||||
   AUCGUCUUUUUC-AUCCGUACCG 5'
AT3G50910.1     276     297
unknown protein

sRNA_AGO1_Solexa_Mi2008_1_13420_hit2
5' GAAGAAGAAGAAGAC-UCUU
   ||||||||||||:|| ||||
   CUUCUUCUUCUUUUGGAGAA 5'
AT3G50910.1     98      117
unknown protein

sRNA_AGO1_Solexa_Mi2008_1_35975_hit2
5' UGAGAUGAGUGACCAUGGCUGU
   |||||||| || |||||||||:
   ACUCUACUAACCGGUACCGACG 5'
AT3G50930.1     1239    1260
BCS1 protein-like protein

sRNA_AGO1_Solexa_Mi2008_1_24431_hit2
5' UAUA-GACAUGUGGAUGAUGCAC
   |||| |||||||||||| ||||
   AUAUGCUGUACACCUAC-ACGUA 5'
AT3G50930.1     1585    1606
BCS1 protein-like protein

sRNA_AGO1_Solexa_Mi2008_6_24430_hit2
5' UAUA-GACAUGUGGAUGAUGCA
   |||| |||||||||||| ||||
   AUAUGCUGUACACCUAC-ACGU 5'
AT3G50930.1     1586    1606
BCS1 protein-like protein

sRNA_AGO1_Solexa_Mi2008_1_24432_hit1
5' UAUA-GACAUGUGGAUGAUGCG
   |||| |||||||||||| |||:
   AUAUGCUGUACACCUAC-ACGU 5'
AT3G50930.1     1586    1606
BCS1 protein-like protein

sRNA_AGO1_Solexa_Mi2008_23_6153_hit2
5' CAGAUCUUGGUGG-UAG-UAGC
   ||||||||||||| ||| |||
   GUCUAGAACCACCGAUCUAUCC 5'
AT3G50930.1     671     692
BCS1 protein-like protein
```

```
sRNA_AGO1_Solexa_Mi2008_2_34202_hit2
5' UGAA-ACUUUAAUUUAAUUAGU
   |||| ||||| ||||||||||
   ACUUAUGAAAAUAAAUUAAUCC 5'
AT3G51590.1     431     452
lipid transfer protein-like protein

sRNA_AGO1_Solexa_Mi2008_1_46987_hit1
5' UUACCAAUCAGACCUCAUACCUU
   ||| ||||||  |||||||||||
   AAUAGUUAGU--GGAGUAUGGAA 5'
AT3G52430.1     1452    1472
putative protein

sRNA_AGO1_Solexa_Mi2008_14_5560_hit17
5' CACCUGCUCAUCAACAACU
   ||| | ||||||||||||:
   GUG-A-GAGUAGUUGUUGG 5'
AT3G52430.1     1788    1804
putative protein

sRNA_AGO1_Solexa_Mi2008_1_52646_hit1
5' UUGG-GGAUUGUUGUGGUUGUU
   :||| |||||||||| ||||||
   GACCUCCUAACAACAACAACAA 5'
AT3G52800.1     377     398
zinc finger like protein

sRNA_AGO1_Solexa_Mi2008_1_49739_hit1
5' UUCUCGACGUUGAUG-UGGCAAG
   ||||||||||||||| | |||||
   AAGAGCUGCAACUACGAGCGUUC 5'
AT3G52930.1     317     339
fructose bisphosphate aldolase - like protein

sRNA_AGO1_Solexa_Mi2008_3_13421_hit1
5' GAAGAAGAAGAUGAUG-UUGAU
   |||||||||||||| | ||||
   CUUCUUCUUCUACU-CGAACUC 5'
AT3G53810.1     61      81
serine/threonine-specific kinase like protein

sRNA_AGO1_Solexa_Mi2008_1_22094_hit1
5' UAGCAGCCUGACCAUUACCAG
   |||||||||||||||||||||
   AUCGUCGGACUGGUAAUGGUC 5'
AT3G54150.1     197     217
embryonic abundant protein -like

sRNA_AGO1_Solexa_Mi2008_5_427_hit1
5' AAAGAUGUUGUC-GCUA-UGU
   |||||||||||| || | |||
   UUUCUACAACAGACGUUUACA 5'
AT3G54640.1     1167    1187
tryptophan synthase alpha chain

sRNA_AGO1_Solexa_Mi2008_1_53131_hit2
5' UUGGUGUUGGU-GGUGUAAUGA
   ||||||| ||| |||||| |||
   AACCACA-CCAACCACAUCACU 5'
AT3G54640.1     816     836
tryptophan synthase alpha chain
```

```
sRNA_AGO1_Solexa_Mi2008_1_3_hit25
5' AAAAAAA-AAAAAAGAAAGA
   ||||||| |||||| |||||
   UUUUUUUCUUUUUU-UUUCU 5'
AT3G54810.2     1506    1524
unknown protein

sRNA_AGO1_Solexa_Mi2008_1_3_hit25
5' AAAAAAAAA-AAAAGAAAGA
   ||||||||| |||| |||:|
   UUUUUUUUUCUUUU-UUUUU 5'
AT3G54810.2     1508    1526
unknown protein

sRNA_AGO1_Solexa_Mi2008_1_3_hit25
5' AAAAAAAAAA-AAAGAAAGA
   |||||||||| ||| |||:|
   UUUUUUUUUUCUUU-UUUUU 5'
AT3G54810.2     1509    1527
unknown protein

sRNA_AGO1_Solexa_Mi2008_1_1776_hit10
5' AGAAAAAAAAAAAA-AAAAAAAU
    |||||||||||| |||||||
   ACUUUUUUUUUUUUCUUUUUUUU 5'
AT3G54810.2     1509    1530
unknown protein

sRNA_AGO1_Solexa_Mi2008_1_3_hit25
5' AAAAAAAAAAA-AAGAAAGA
   ||||||||||| || |||:|
   UUUUUUUUUUUCUU-UUUUU 5'
AT3G54810.2     1510    1528
unknown protein

sRNA_AGO1_Solexa_Mi2008_1_3_hit25
5' AAAAAAA-AAAAAAGAAAGA
   ||||||| |||||| |||||
   UUUUUUUCUUUUUU-UUUCU 5'
AT3G54810.1     1587    1605
unknown protein

sRNA_AGO1_Solexa_Mi2008_1_3_hit25
5' AAAAAAAAA-AAAAGAAAGA
   ||||||||| |||| |||:|
   UUUUUUUUUCUUUU-UUUUU 5'
AT3G54810.1     1589    1607
unknown protein

sRNA_AGO1_Solexa_Mi2008_1_3_hit25
5' AAAAAAAAAA-AAAGAAAGA
   |||||||||| ||| |||:|
   UUUUUUUUUUCUUU-UUUUU 5'
AT3G54810.1     1590    1608
unknown protein

sRNA_AGO1_Solexa_Mi2008_1_1776_hit10
5' AGAAAAAAAAAAAA-AAAAAAAU
    |||||||||||| |||||||
   ACUUUUUUUUUUUUCUUUUUUUU 5'
AT3G54810.1     1590    1611
unknown protein
```

```
sRNA_AGO1_Solexa_Mi2008_1_3_hit25
5' AAAAAAAAAAA-AAGAAAGA
   ||||||||||| || |||:|
   UUUUUUUUUUUCUU-UUUUU 5'
AT3G54810.1     1591    1609
unknown protein

sRNA_AGO1_Solexa_Mi2008_1_34602_hit1
5' UGAAGAGUGAUUGAUUGGGAA
   |||||||||||||||||||||
   ACUUCUCACUAACUAACCCUU 5'
AT3G54810.2     20      40
unknown protein

sRNA_AGO1_Solexa_Mi2008_1_17855_hit2
5' UAAGAGCUCAGGUGAAGAAU
     ||||||||||||| ||||
   UAUCUCGAGUCCACUACUUA 5'
AT3G54810.2     398     417
unknown protein

sRNA_AGO1_Solexa_Mi2008_1_17855_hit2
5' UAAGAGCUCAGGUGAAGAAU
     ||||||||||||| ||||
   UAUCUCGAGUCCACUACUUA 5'
AT3G54810.1     479     498
unknown protein

sRNA_AGO1_Solexa_Mi2008_3_768_hit1
5' AAGAAGAGAGAGAAUUGAG
   |||||||||||| ||||
   UUCUUCUCUCUC-UAACGA 5'
AT3G54810.2     82      99
unknown protein

sRNA_AGO1_Solexa_Mi2008_1_50638_hit1
5' UUGAGGACAAAAGAG-GGAAA
   ||||||| ||||||| |||||
   AACUCCUUUUUUCUCUCCUUU 5'
AT3G55050.1     1582    1602
protein phosphatase 2C - like protein

sRNA_AGO1_Solexa_Mi2008_1_50638_hit1
5' UUGAGGACAAAAGAG-GGAAA
   ||||||| ||||||| |||||
   AACUCCUUUUUUCUCUCCUUU 5'
AT3G55050.2     1699    1719
protein phosphatase 2C - like protein

sRNA_AGO1_Solexa_Mi2008_1_51840_hit3
5' UUGCUGAAGAUGGUGUUGAUGC
   ||| ||||| |||||||||||
   AACAACUUCAACCACAACUACC 5'
AT3G55560.1     281     302
unknown protein

sRNA_AGO1_Solexa_Mi2008_1_19482_hit1
5' UACAGGGAAAGCAGU-CAUGCU
   ||||||||||||||| || |||
   AUGUCCCUUUCGUCACGU-CGA 5'
AT3G55605.1     562     582
unknown protein
```

```
sRNA_AGO1_Solexa_Mi2008_1_394_hit4
5' AAAGAGGAAGAUAG-GACGCAU
   ||||||||||||| || |||
   UUUCUCCUUCUAUCUCU-CGUU 5'
AT3G55840.1     1587    1607
nematode resistance protein-like protein

sRNA_AGO1_Solexa_Mi2008_1_4082_hit2
5' CAAAGAGGAAGAUAG-GACGCA
   |||||||||||||| || |||
   GUUUCUCCUUCUAUCUCU-CGU 5'
AT3G55840.1     1588    1608
nematode resistance protein-like protein

sRNA_AGO1_Solexa_Mi2008_1_393_hit4
5' AAAGAGGAAGAUAG-GACGC
   ||||||||||||| || ||
   UUUCUCCUUCUAUCUCU-CG 5'
AT3G55840.1     1589    1607
nematode resistance protein-like protein

sRNA_AGO1_Solexa_Mi2008_1_5113_hit1
5' CAAUCCGAGAGAUUCGA-UGUCC
   |||||||||||||||| |||
   GUUAGGCUCUCUAAGCUGACACC 5'
AT3G55980.1     1623    1645
unknown protein

sRNA_AGO1_Solexa_Mi2008_1_42480_hit1
5' UGGG-GGUUGAUGAUCCGAUGG
    ||| |||||||||||| ||||
   CCCCUCCAACUACUAGG-UACC 5'
AT3G56200.1     118     138
unknown protein

sRNA_AGO1_Solexa_Mi2008_1_54251_hit1
5' UUUAGAGGUGACCAUUAGAGUA
   ||||||||||| ||  ||||||
   AAAUCUCCACU-GU-UUCUCAU 5'
AT3G56260.1     118     137
unknown protein

sRNA_AGO1_Solexa_Mi2008_1_2092_hit5
5' AGCUC-AAGAAAAUGCG-CCUCC
   ||||| ||||||||||| ||||
   UCGAGGUUCUUUUACGCAGGAGU 5'
AT3G56400.1     429     451
putative DNA-binding protein

sRNA_AGO1_Solexa_Mi2008_3_12720_hit1
5' CUUGAAACAGGAGAUAGAGUUU
   |||||||| |||||| | ||||
   GAACUUUGGCCUCUA-C-CAAA 5'
AT3G56710.1     109     128
SigA binding protein

sRNA_AGO1_Solexa_Mi2008_6_28221_hit36
5' UCAGAUGUUUACAUGUGUCA
   ||||||||||| ||||||
   AGUCUACAAAUAUACACACA 5'
AT3G56710.1     16      35
SigA binding protein
```

```
sRNA_AGO1_Solexa_Mi2008_5_1114_hit1
5' AAUG-GAACUAGUGGACAAACAA
   |||| ||||||||||| |||||
   UUACUCUUGAUCACCUAUUUGUG 5'
AT3G56970.1     840     862
putative bHLH transcription factor  (bHLH038)

sRNA_AGO1_Solexa_Mi2008_1_22326_hit1
5' UAGCGAGGAAAUUGUUGGU
   |||||||||||||||||||
   AUCGCUCCUUUAACAACCA 5'
AT3G57120.1     530     548
unknown protein

sRNA_AGO1_Solexa_Mi2008_6_11244_hit6
5' CUCUGAACCACAACG-CUUU
   ||||||| ||||||| |||:
   GAGACUUUGUGUUGCUGAAG 5'
AT3G57120.1     606     625
unknown protein

sRNA_AGO1_Solexa_Mi2008_1_48516_hit1
5' UUCAAGGAAGAUUGGACCAGU
   ||||||||||||||| |||:|
   AAGUUCCUUCUAACC-GGUUA 5'
AT3G57170.1     1048    1067
unknown protein

sRNA_AGO1_Solexa_Mi2008_2_13688_hit1
5' GACCAUUUGUGAGAAGAGA
   |||||||||||||||||||
   CUGGUAAACACUCUUCUCU 5'
AT3G57230.2     585     603
MADS-box transcription factor (AGL16)

sRNA_AGO1_Solexa_Mi2008_596_13689_hit1
5' GACCAUUUGUGAGAAGGGA
   ||||||||||||||||:||
   CUGGUAAACACUCUUCUCU 5'
AT3G57230.2     585     603
MADS-box transcription factor (AGL16)

sRNA_AGO1_Solexa_Mi2008_72_1853_hit1
5' AGACCAUUUGUGAGAAGGGA
   |||||||||||||||||:||
   UCUGGUAAACACUCUUCUCU 5'
AT3G57230.2     585     604
MADS-box transcription factor (AGL16)

sRNA_AGO1_Solexa_Mi2008_3321_21060_hit1
5' UAGACCAUUUGUGAGAAGGGA
   ||||||||||||||||||:||
   AUCUGGUAAACACUCUUCUCU 5'
AT3G57230.2     585     605
MADS-box transcription factor (AGL16)

sRNA_AGO1_Solexa_Mi2008_2_10113_hit1
5' CUAGACCAUUUGUGAGAAGGGA
   |||||||||||||||||||:||
   GAUCUGGUAAACACUCUUCUCU 5'
AT3G57230.2     585     606
MADS-box transcription factor (AGL16)
```

```
sRNA_AGO1_Solexa_Mi2008_32_21059_hit1
5' UAGACCAUUUGUGAGAAGGG
   ||||||||||||||||||:|
   AUCUGGUAAACACUCUUCUC 5'
AT3G57230.2     586     605
MADS-box transcription factor (AGL16)

sRNA_AGO1_Solexa_Mi2008_37_21058_hit1
5' UAGACCAUUUGUGAGAAGG
   ||||||||||||||||||:
   AUCUGGUAAACACUCUUCU 5'
AT3G57230.2     587     605
MADS-box transcription factor (AGL16)

sRNA_AGO1_Solexa_Mi2008_2_13688_hit1
5' GACCAUUUGUGAGAAGAGA
   |||||||||||||||||||
   CUGGUAAACACUCUUCUCU 5'
AT3G57230.1     588     606
MADS-box transcription factor (AGL16)

sRNA_AGO1_Solexa_Mi2008_596_13689_hit1
5' GACCAUUUGUGAGAAGGGA
   ||||||||||||||||:||
   CUGGUAAACACUCUUCUCU 5'
AT3G57230.1     588     606
MADS-box transcription factor (AGL16)

sRNA_AGO1_Solexa_Mi2008_72_1853_hit1
5' AGACCAUUUGUGAGAAGGGA
   |||||||||||||||||:||
   UCUGGUAAACACUCUUCUCU 5'
AT3G57230.1     588     607
MADS-box transcription factor (AGL16)

sRNA_AGO1_Solexa_Mi2008_3321_21060_hit1
5' UAGACCAUUUGUGAGAAGGGA
   ||||||||||||||||||:||
   AUCUGGUAAACACUCUUCUCU 5'
AT3G57230.1     588     608
MADS-box transcription factor (AGL16)

sRNA_AGO1_Solexa_Mi2008_2_10113_hit1
5' CUAGACCAUUUGUGAGAAGGGA
   |||||||||||||||||||:||
   GAUCUGGUAAACACUCUUCUCU 5'
AT3G57230.1     588     609
MADS-box transcription factor (AGL16)

sRNA_AGO1_Solexa_Mi2008_32_21059_hit1
5' UAGACCAUUUGUGAGAAGGG
   ||||||||||||||||||:|
   AUCUGGUAAACACUCUUCUC 5'
AT3G57230.1     589     608
MADS-box transcription factor (AGL16)

sRNA_AGO1_Solexa_Mi2008_37_21058_hit1
5' UAGACCAUUUGUGAGAAGG
   ||||||||||||||||||:
   AUCUGGUAAACACUCUUCU 5'
AT3G57230.1     590     608
MADS-box transcription factor (AGL16)
```

```
sRNA_AGO1_Solexa_Mi2008_15_2751_hit1
5' AUCAAAAUCUG-UGGUGAGGCU
   ||||| || || ||||||||||
   UAGUUGUA-ACCACCACUCCGA 5'
AT3G57260.1     58      78
beta-1,3-glucanase 2 (BG2)

sRNA_AGO1_Solexa_Mi2008_1_40423_hit1
5' UGGAGCCUGCACUGGUACU
    || |||||||||||| ||
   CCC-CGGACGUGACCA-GA 5'
AT3G57410.1     1596    1612
villin 3

sRNA_AGO1_Solexa_Mi2008_5_349_hit1
5' AAAGAACAAUGGCAGCGAGU
    |||||||||||||  ||||
   GUUCUUGUUACCGU--CUCA 5'
AT3G57550.2     575     592
guanylate kinase (GK-2)

sRNA_AGO1_Solexa_Mi2008_2_40296_hit2
5' UG-GAGAGUUCAUCAGAGA-UU
   || |||||||||||||||| ||
   ACUCUCUCAAGUAGUCUCUCAA 5'
AT3G58270.2     281     302
unknown protein

sRNA_AGO1_Solexa_Mi2008_2_40296_hit2
5' UG-GAGAGUUCAUCAGAGA-UU
   || |||||||||||||||| ||
   ACUCUCUCAAGUAGUCUCUCAA 5'
AT3G58270.1     307     328
unknown protein

sRNA_AGO1_Solexa_Mi2008_1_5432_hit1
5' CACCAAUGCUUUCUCUAUCUU
   ||| || |||||||||||||
   GUG-UU-CGAAAGAGAUAGAC 5'
AT3G58660.1     605     623
putative protein

sRNA_AGO1_Solexa_Mi2008_1_14019_hit1
5' GAGCUCCUUGAAGUUCAAUG
   ||| |||||||||||||| |
   CUCAAGGAACUUCAAGUUCC 5'
AT3G58780.2     697     716
shatterproof 1 (SHP1)/ agamous -like 1 (AGL1)

sRNA_AGO1_Solexa_Mi2008_271_14020_hit1
5' GAGCUCCUUGAAGUUCAAUGG
   ||| |||||||||||||| ||
   CUCAAGGAACUUCAAGUU-CC 5'
AT3G58780.2     698     717
shatterproof 1 (SHP1)/ agamous -like 1 (AGL1)

sRNA_AGO1_Solexa_Mi2008_1_14019_hit1
5' GAGCUCCUUGAAGUUCAAUG
   ||| |||||||||||||| |
   CUCAAGGAACUUCAAGUUCC 5'
AT3G58780.1     718     737
shatterproof 1 (SHP1)/ agamous -like 1 (AGL1)
```

```
sRNA_AGO1_Solexa_Mi2008_271_14020_hit1
5' GAGCUCCUUGAAGUUCAAUGG
   ||| ||||||||||||| ||
   CUCAAGGAACUUCAAGUU-CC 5'
AT3G58780.1     719     738
shatterproof 1 (SHP1)/ agamous -like 1 (AGL1)

sRNA_AGO1_Solexa_Mi2008_1_1059_hit1
5' AAUGAAAGUAGAGAGA-GCU
   ||| ||||||||||| ||
   UUAAUUUCAUCUCUCUUCGC 5'
AT3G59320.2     120     139
anthocyanin-related membrane protein 2 (Anm2)

sRNA_AGO1_Solexa_Mi2008_2_15803_hit1
5' GUCUGAGAAAAUUACCCAU
   |||||||||||:|| ||||
   CAGACUCUUUUGAU-GGUA 5'
AT3G59320.1     433     450
anthocyanin-related membrane protein 2 (Anm2)

sRNA_AGO1_Solexa_Mi2008_2_15803_hit1
5' GUCUGAGAAAAUUACCCAU
   |||||||||||:|| ||||
   CAGACUCUUUUGAU-GGUA 5'
AT3G59320.2     591     608
anthocyanin-related membrane protein 2 (Anm2)

sRNA_AGO1_Solexa_Mi2008_1_1059_hit1
5' AAUGAAAGUAGAGAG-AGCU
   ||| |||||||||| |||
   UUAAUUUCAUCUCUCUUCGC 5'
AT3G59320.1     74      93
anthocyanin-related membrane protein 2 (Anm2)

sRNA_AGO1_Solexa_Mi2008_2_17207_hit1
5' UAACACCACCAAGACUCCU
   :|||| ||||||||||||
   GUUGUUGUGGUUCUGAGGU 5'
AT3G59520.1     450     468
unknown protein

sRNA_AGO1_Solexa_Mi2008_1_11291_hit2
5' CUCUUAUCCAAGGGUUAUUUC
    |||||||||||||| |||
   AAGAAUAGGUUCCCAACAAAC 5'
AT3G59530.2     1067    1087
unknown protein

sRNA_AGO1_Solexa_Mi2008_1_11291_hit2
5' CUCUUAUCCAAGGGUUAUUUC
    |||||||||||||| |||
   AAGAAUAGGUUCCCAACAAAC 5'
AT3G59530.1     1131    1151
unknown protein

sRNA_AGO1_Solexa_Mi2008_1_19370_hit1
5' UACAGAGAGAUGAUGUU-GUUC
   ||||||||||||| || ||||
   AUGUCUCUCUACUA-AAUCAAG 5'
AT3G59530.2     1320    1340
unknown protein
```

```
sRNA_AGO1_Solexa_Mi2008_1_19370_hit1
5' UACAGAGAGAUGAUGUU-GUUC
   ||||||||||||| || ||||
   AUGUCUCUCUACUA-AAUCAAG 5'
AT3G59530.1     1384    1404
unknown protein

sRNA_AGO1_Solexa_Mi2008_4_7308_hit1
5' CAUGGU-CCACCGAAAAUGU
   |||| | ||||||||||||
   GUACAAUGGUGGCUUUUACA 5'
AT3G59530.2     437     456
unknown protein

sRNA_AGO1_Solexa_Mi2008_4_7308_hit1
5' CAUGGU-CCACCGAAAAUGU
   |||| | ||||||||||||
   GUACAAUGGUGGCUUUUACA 5'
AT3G59530.1     501     520
unknown protein

sRNA_AGO1_Solexa_Mi2008_1_812_hit1
5' AAGAGGAGGA-AGCAUUGUUCUU
   |||||||||| || |||| ||||
   UUCUCCUCCUUUC-UAACCAGAA 5'
AT3G59530.2     873     894
unknown protein

sRNA_AGO1_Solexa_Mi2008_1_812_hit1
5' AAGAGGAGGA-AGCAUUGUUCUU
   |||||||||| || |||| ||||
   UUCUCCUCCUUUC-UAACCAGAA 5'
AT3G59530.1     937     958
unknown protein

sRNA_AGO1_Solexa_Mi2008_10_35767_hit2
5' UGAGAAAGAUGAGAUCACA
   ||||||||||||||| |||
   ACUCUUUCUACUCUACUGU 5'
AT3G59700.1     216     234
serine/threonine-specific kinase lecRK1 precursor,lectin receptor-like

sRNA_AGO1_Solexa_Mi2008_1_23362_hit3
5' UAGGUAUA-ACUAUGUUGGUUGUU
   |||||| | ||| |||||||||||
   AUCCAUUUGUGAAACAACCAACAA 5'
AT3G60070.1     1408    1431
unknown protein

sRNA_AGO1_Solexa_Mi2008_1_18400_hit1
5' UAAGGUUCGUUGAUUGUUGUC
   |||||||||||||||||||||
   AUUCCAAGCAACUAACAACAG 5'
AT3G60140.1     647     667
beta-glucosidase

sRNA_AGO1_Solexa_Mi2008_1_24287_hit1
5' UAUAAGGUUCGUUGAUUGUUGUC
   |||||||||||||||||||||||
   AUAUUCCAAGCAACUAACAACAG 5'
AT3G60140.1     647     669
beta-glucosidase
```

```
sRNA_AGO1_Solexa_Mi2008_7_13212_hit6
5' CUUUGCCAUCUUUUUGAGAG
    |||||||||| |||| |||
   AAAACGGUAGA-AAAC-CUC 5'
AT3G60420.2     686     703
unknown protein

sRNA_AGO1_Solexa_Mi2008_1_50433_hit1
5' UU-GACUCACCGUGACGCAUUA
   || || ||||||||||||| |
   AAGCU-AGUGGCACUGCGUACU 5'
AT3G60450.1     75      95
unknown protein

sRNA_AGO1_Solexa_Mi2008_2_35370_hit1
5' UGAC-CAGAUCCCAUCUGUGUA
   | || || ||||||||||||||
   AAUGAGU-UAGGGUAGACACAU 5'
AT3G60680.1     628     648
unknown protein

sRNA_AGO1_Solexa_Mi2008_1_7416_hit27
5' CAUG-UUUGGGAUGCAUUUGGA
    ||| ||||| |||||||||||
   UUACGAAACC-UACGUAAACCU 5'
AT3G60680.1     99      119
unknown protein

sRNA_AGO1_Solexa_Mi2008_2_12536_hit1
5' CUUAUUGAUUCGAUGUG-UUU
    ||||||||||||| || |||
   UAAUAACUAAGCUA-ACUAAA 5'
AT3G61170.1     666     685
putative protein

sRNA_AGO1_Solexa_Mi2008_2_32161_hit1
5' UCGGGUCGGGUACGAUGUGUA
   |||||||||||||||||||||
   AGCCCAGCCCAUGCUACACAU 5'
AT3G61460.1     224     244
RING finger protein

sRNA_AGO1_Solexa_Mi2008_60_9189_hit1
5' CGGGUAUUCGGGUCGAGUA
   ||||||||||||||| |||
   GCCCAUAAGCCCAGCCCAU 5'
AT3G61460.1     233     251
RING finger protein

sRNA_AGO1_Solexa_Mi2008_1_54911_hit1
5' UUUCGGGUAUUCGGGUCGGGUA
   ||||||||||||||||||||||
   AAAGCCCAUAAGCCCAGCCCAU 5'
AT3G61460.1     233     254
RING finger protein

sRNA_AGO1_Solexa_Mi2008_16_36152_hit1
5' UGAGCGGGUUUCGGGUAUUCG
   |||||||||||||||||||||
   ACUCGCCCAAAGCCCAUAAGC 5'
AT3G61460.1     242     262
RING finger protein
```

```
sRNA_AGO1_Solexa_Mi2008_3_8682_hit1
5' CGAGU-UUCUACAGAGUGGACA
   || || |||||||:||||||||
   GC-CACAAGAUGUUUCACCUGU 5'
AT3G61460.1     468     488
RING finger protein

sRNA_AGO1_Solexa_Mi2008_1_30383_hit1
5' UCGAAAGGGCCAAACCUGUGA
   |||||||| |||||||| |||
   AGCUUUCCAGGUUUGGA-ACU 5'
AT3G61630.1     1243    1262
unknown protein

sRNA_AGO1_Solexa_Mi2008_2_38647_hit1
5' UGCCGUAGACGUUGUUGUUG
     |||| |||||||||||||
   CAGGCA-CUGCAACAACAAC 5'
AT3G61630.1     241     259
unknown protein

sRNA_AGO1_Solexa_Mi2008_99_5812_hit1
5' CACUGG-UCGACGGAUCUCUA
    || || ||||||||||||||
   UUG-CCGAGCUGCCUAGAGAU 5'
AT3G61630.1     454     473
unknown protein

sRNA_AGO1_Solexa_Mi2008_4_15860_hit3
5' GUGAGAGCUUUAGGU--UUUGA
   ||||| |||||||||  |||||
   CACUCGCGAAAUCCAGGAAACU 5'
AT3G61630.1     546     567
unknown protein

sRNA_AGO1_Solexa_Mi2008_28_14484_hit1
5' GCAGUGGUGGCGGUUACUCAG
   |||||||||||| ||| |||:
   CGUCACCACCGC-AAU-AGUU 5'
AT3G62130.1     386     404
unknown protein

sRNA_AGO1_Solexa_Mi2008_1_16782_hit1
5' UAAAGCU-CCAGGACCGGAUGGU
    |||||| |||| ||||||||||
   CUUUCGAAGGUC-UGGCCUACCA 5'
AT3G62260.2     405     426
unknown protein

sRNA_AGO1_Solexa_Mi2008_1_16782_hit1
5' UAAAGCU-CCAGGACCGGAUGGU
    |||||| |||| ||||||||||
   CUUUCGAAGGUC-UGGCCUACCA 5'
AT3G62260.1     416     437
unknown protein

sRNA_AGO1_Solexa_Mi2008_4_11360_hit1
5' CUGAA-CUCCGAGUCCGAUUGAU
   ||||| |||||||||||||| |:
   GACUUAGAGGCUCAGGCUAAGUG 5'
AT3G62370.1     116     138
unknown protein (At3g62370)
```

```
sRNA_AGO1_Solexa_Mi2008_2_8781_hit1
5' CGCCAACACUACAACCACGAG
    |||| | ||||||||||||
   ACGGU-GCGAUGUUGGUGCUC 5'
AT3G62370.1     40      59
unknown protein (At3g62370)

sRNA_AGO1_Solexa_Mi2008_3_27777_hit1
5' UCAC-GAAGACGGAUGAUGGCA
   |||| || ||||||||||||
   AGUGACU-CUGCCUACUACCGA 5'
AT3G62700.1     1025    1045
ABC transporter-like protein

sRNA_AGO1_Solexa_Mi2008_1_29196_hit2
5' UCCAAAGGGAUCGCAUUGUUU
   ||||||||||||||||||:|
   AGGUUUCCCUAGCGUAACAGA 5'
AT3G62980.1     1710    1730
transport inhibitor response 1 (TIR1)

sRNA_AGO1_Solexa_Mi2008_1_29193_hit2
5' UCCAAAGGGAUCGCAUUGAU
   |||||||||||||||||| :
   AGGUUUCCCUAGCGUAACAG 5'
AT3G62980.1     1711    1730
transport inhibitor response 1 (TIR1)

sRNA_AGO1_Solexa_Mi2008_14_29195_hit2
5' UCCAAAGGGAUCGCAUUGAUCC
   |||||||||||||||||| ||
   AGGUUUCCCUAGCGUAAC-AGA 5'
AT3G62980.1     1711    1731
transport inhibitor response 1 (TIR1)

sRNA_AGO1_Solexa_Mi2008_23_29194_hit2
5' UCCAAAGGGAUCGCAUUGAUC
   |||||||||||||||||| ||
   AGGUUUCCCUAGCGUAAC-AG 5'
AT3G62980.1     1712    1731
transport inhibitor response 1 (TIR1)

sRNA_AGO1_Solexa_Mi2008_2_36207_hit1
5' UGAGCUUGAUGGUUAUAUGAA
   ||||||||||||||||||||
   ACUCGAACUACCAAUAUACUU 5'
AT3G62980.1     2092    2112
transport inhibitor response 1 (TIR1)

sRNA_AGO1_Solexa_Mi2008_1_39228_hit1
5' UGCUCAUGAGCUUGAUGGUUA
   ||||||||||||||||||||
   ACGAGUACUCGAACUACCAAU 5'
AT3G62980.1     2098    2118
transport inhibitor response 1 (TIR1)

sRNA_AGO1_Solexa_Mi2008_7_33390_hit1
5' UCUGAGACGAGGCGUGGGUGG
   |||||||||||||||||  |
   AGACUCUGCUCCGCACCCCGC 5'
AT3G63090.1     543     563
unknown protein
```

```
sRNA_AGO1_Solexa_Mi2008_1_34710_hit1
5' UGAAGGAACAUGGUACA-GAAG
   ||||||||||||| | | ||||
   ACUUCCUUGUACC-UCUACUUC 5'
AT4G00300.1     59      79
null

sRNA_AGO1_Solexa_Mi2008_6_37023_hit2
5' UGAUCAAGAAGACGA-G-UGGCC
   ||||||||||||||| | ||||
   ACUAGUUCUUCUGCUGCUACCGA 5'
AT4G00300.1     81      103
null

sRNA_AGO1_Solexa_Mi2008_1_46662_hit2
5' UUAAGUGGAAAUGUGGUAUGA
    ||||||||||||||| | ||
   CAUUCACCUUUACACC-U-CU 5'
AT4G00335.2     887     905
RING-H2 finger protein RHB1a

sRNA_AGO1_Solexa_Mi2008_1_46662_hit2
5' UUAAGUGGAAAUGUGGUAUGA
    ||||||||||||||| | ||
   CAUUCACCUUUACACC-U-CU 5'
AT4G00335.1     917     935
RING-H2 finger protein RHB1a

sRNA_AGO1_Solexa_Mi2008_1_46662_hit2
5' UUAAGUGGAAAUGUGGUAUGA
    ||||||||||||||| | ||
   CAUUCACCUUUACACC-U-CU 5'
AT4G00335.3     919     937
RING-H2 finger protein RHB1a

sRNA_AGO1_Solexa_Mi2008_1_13417_hit1
5' GAAGAAAUAGA-UGGUAAUAGU
   ||||||||| | ||| ||||||
   CUUCUUUAU-UGACCCUUAUCA 5'
AT4G00755.2     1241    1261
putative F-box protein

sRNA_AGO1_Solexa_Mi2008_3_768_hit1
5' AAGA-AGAGAGAGAAUUGAG
   |||| | ||||||||||||
   UUCUGU-UCUCUCUUAACUA 5'
AT4G00755.2     132     150
putative F-box protein

sRNA_AGO1_Solexa_Mi2008_1_13417_hit1
5' GAAGAAAUAGA-UGGUAAUAGU
   ||||||||| | ||| ||||||
   CUUCUUUAU-UGACCCUUAUCA 5'
AT4G00755.1     1377    1397
putative F-box protein

sRNA_AGO1_Solexa_Mi2008_3_768_hit1
5' AAGA-AGAGAGAGAAUUGAG
   |||| | ||||||||||||
   UUCUGU-UCUCUCUUAACUA 5'
AT4G00755.1     268     286
putative F-box protein
```

```
sRNA_AGO1_Solexa_Mi2008_2_52709_hit1
5' UUGGGGUGCUCAUGAUCAUAC
   |||||||||||||||||||||
   AACCCCACGAGUACUAGUAUG 5'
AT4G00940.1     714     734
putative protein

sRNA_AGO1_Solexa_Mi2008_1_3079_hit472
5' AUGAUGAUGAUGA-UGAUGAUGA
   ||||||||||| | ||||||||
   UACUACUACUA-UGACUACUACC 5'
AT4G00940.1     91      112
putative protein

sRNA_AGO1_Solexa_Mi2008_6_14255_hit369
5' GAUGAUGAUGAUGAUGA-UGAUGAU
   ||| ||||||||||| | |||||||
   CUAGUACUACUACUA-UGACUACUA 5'
AT4G00940.1     93      116
putative protein

sRNA_AGO1_Solexa_Mi2008_1_3079_hit472
5' AUGAUGAUGAUGAUGAUGA-UGA
   ||||| ||||||||||| | |||
   UACUAGUACUACUACUA-UGACU 5'
AT4G00940.1     97      118
putative protein

sRNA_AGO1_Solexa_Mi2008_1_44627_hit1
5' UGUCCCUCCGCACUUCAGACC
   |||||||||||||||||||||
   ACAGGGAGGCGUGAAGUCUGG 5'
AT4G01000.1     1265    1285
putative protein

sRNA_AGO1_Solexa_Mi2008_1_23079_hit1
5' UAGGGCAAAAUGGUGGACU-UG
   :|||||||||||||||| | ||
   GUCCCGUUUUACCACCU-AUAC 5'
AT4G01120.1     378     398
G-box binding bZip transcription factor GBF2 / AtbZip54

sRNA_AGO1_Solexa_Mi2008_19_34341_hit1
5' UGAACACCAGGAUAGUAGCAC
   ||||||||||||| | ||||
   ACUUGUGGUCCUA-C-UCGUA 5'
AT4G01120.1     411     429
G-box binding bZip transcription factor GBF2 / AtbZip54

sRNA_AGO1_Solexa_Mi2008_9_14254_hit8
5' GAUGAUGAUGAUGAUGAUCUU
   ||| |||||||||||||||:
   CUA-UACUACUACUACUAGGU 5'
AT4G01360.1     719     738
unknown protein

sRNA_AGO1_Solexa_Mi2008_1_36323_hit13
5' UGAGGAUGAUGAUGAUGAUGA
   :||| || ||||||||||||
   GCUC-UA-UACUACUACUACU 5'
AT4G01360.1     723     741
unknown protein
```

```
sRNA_AGO1_Solexa_Mi2008_1_44646_hit1
5' UGUCCUAAACGAAGCGGUGGU
   |||||||||||||||||||||
   ACAGGAUUUGCUUCGCCACCA 5'
AT4G01410.1     538     558
putative hypoersensitive response protein

sRNA_AGO1_Solexa_Mi2008_31_7103_hit13
5' CAUGAACGUUUGUUGUGUC
   |||||||||| |||||| |
   GUACUUGCAAUCAACACCG 5'
AT4G01430.1     300     318
unknown protein

sRNA_AGO1_Solexa_Mi2008_1_30942_hit3
5' UCGAGUGAAGUGGUCUGGUCG
   ||| |||||||||||| |||
   AGC-CACUUCACCAGAACAGA 5'
AT4G01840.1     381     400
potassium channel like protein

sRNA_AGO1_Solexa_Mi2008_1_3079_hit472
5' AUGAUGAUGAUGAUGAUGAUGA
   ||||||||| || ||||||||
   UACUACUACCACAACUACUACG 5'
AT4G01840.1     795     816
potassium channel like protein

sRNA_AGO1_Solexa_Mi2008_6_14255_hit369
5' GAUGAUGAUGAUGAUGAUGAUGAU
    |||||||||||| || |||||||
   AUACUACUACUACCACAACUACUA 5'
AT4G01840.1     797     820
potassium channel like protein

sRNA_AGO1_Solexa_Mi2008_1_3079_hit472
5' AUGAUGAUGAUGAUGAUGAUGA
   |||||||||||| || ||||||
   UACUACUACUACCACAACUACU 5'
AT4G01840.1     798     819
potassium channel like protein

sRNA_AGO1_Solexa_Mi2008_1_27297_hit1
5' UCAA-GUAGCUUAUGUUUGGAG
   |||| ||||||| |||||||||
   AGUUGCAUCGAACACAAACCUC 5'
AT4G04490.1     107     128
putative receptor-like protein kinase

sRNA_AGO1_Solexa_Mi2008_1_13589_hit1
5' GACAAGAGUAAGAC-UCUAU
    ||||||||||||| || ||
   GUGUUCUCAUUCUGAAG-UA 5'
AT4G04490.1     1148    1166
putative receptor-like protein kinase

sRNA_AGO1_Solexa_Mi2008_1_51110_hit1
5' UUGAUGAUGAUCACAGUUGUGU
   |||||||||||||  |||| ||
   AACUACUACUAGUC-CAAC-CA 5'
AT4G04830.1     1       20
Unknown protein
```

```
sRNA_AGO1_Solexa_Mi2008_1_32728_hit1
5' UCUAAGGGAAAAUGUAUGAGC
   : ||||||||| |||||||||
   GUAUUCCCUUU-ACAUACUCG 5'
AT4G04920.1     3700    3719
unknown protein

sRNA_AGO1_Solexa_Mi2008_1_20752_hit15
5' UAGAAAAUGCACUGC-UA-GAUC
   |||||| |||||||| || ||||
   AUCUUUAACGUGACGUAUACUAG 5'
AT4G07410.1     1543    1565
unknown protein

sRNA_AGO1_Solexa_Mi2008_1_20752_hit15
5' UAGAAAAUGCACUGC-UA-GAUC
   |||||| |||||||| || ||||
   AUCUUUAACGUGACGUAUACUAG 5'
AT4G07410.2     1685    1707
unknown protein

sRNA_AGO1_Solexa_Mi2008_1_49909_hit1
5' UUGAAACGAACCAAG-GCUGCCU
   |||||| ||||| || |||||||
   AACUUUACUUGG-UCACGACGGA 5'
AT4G07410.1     1940    1961
unknown protein

sRNA_AGO1_Solexa_Mi2008_1_49909_hit1
5' UUGAAACGAACCAAG-GCUGCCU
   |||||| |||||| | |||||||
   AACUUUACUUGGU-CACGACGGA 5'
AT4G07410.2     2082    2103
unknown protein

sRNA_AGO1_Solexa_Mi2008_3_4568_hit12
5' CAAGAAGAAGAUGAGAACA-AUG
   ||||||||||| ||||| | |||
   GUUCUUCUUCUUCUCUU-UCUAC 5'
AT4G08330.1     244     265
unknown protein

sRNA_AGO1_Solexa_Mi2008_1_21813_hit1
5' UAGAUGAUGGACUUCGAUGAU
    || |||||||||||:|||||
   CUC-ACUACCUGAAGUUACUA 5'
AT4G08350.1     1262    1281
putative protein

sRNA_AGO1_Solexa_Mi2008_1_2916_hit1
5' AUCGUGGACUCAUAG-C-UGU
   ||||||||||||||| | |||
   UAGCACCUGAGUAUCCGCACA 5'
AT4G08350.1     2539    2559
putative protein

sRNA_AGO1_Solexa_Mi2008_44_10639_hit2
5' CUC-AAGAUGGCAGCAU-AGU
    || ||||||||||||| |||
   UAGCUUCUACCGUCGUAGUCA 5'
AT4G08390.2     1048    1068
stromal ascorbate peroxidase
```

```
sRNA_AGO1_Solexa_Mi2008_5_5124_hit2
5' CAAUCU-C-AAGAUGGCAGCAU
   |||||| | |||||||||||||
   GUUAGAAGCUUCUACCGUCGUA 5'
AT4G08390.2     1052    1073
stromal ascorbate peroxidase

sRNA_AGO1_Solexa_Mi2008_5_19199_hit2
5' UACAAUCU-C-AAGAUGGCAGCAU
   | |||||| | |||||||||||||
   AAGUUAGAAGCUUCUACCGUCGUA 5'
AT4G08390.2     1052    1075
stromal ascorbate peroxidase

sRNA_AGO1_Solexa_Mi2008_1_19198_hit2
5' UACAAUCU-C-AAGAUGGCAGCA
   | |||||| | ||||||||||||
   AAGUUAGAAGCUUCUACCGUCGU 5'
AT4G08390.2     1053    1075
stromal ascorbate peroxidase

sRNA_AGO1_Solexa_Mi2008_44_10639_hit2
5' CUC-AAGAUGGCAGCAU-AGU
    || ||||||||||||| |||
   UAGCUUCUACCGUCGUAGUCA 5'
AT4G08390.1     1058    1078
stromal ascorbate peroxidase

sRNA_AGO1_Solexa_Mi2008_5_5124_hit2
5' CAAUCU-C-AAGAUGGCAGCAU
   |||||| | |||||||||||||
   GUUAGAAGCUUCUACCGUCGUA 5'
AT4G08390.1     1062    1083
stromal ascorbate peroxidase

sRNA_AGO1_Solexa_Mi2008_5_19199_hit2
5' UACAAUCU-C-AAGAUGGCAGCAU
   | |||||| | |||||||||||||
   AAGUUAGAAGCUUCUACCGUCGUA 5'
AT4G08390.1     1062    1085
stromal ascorbate peroxidase

sRNA_AGO1_Solexa_Mi2008_1_19198_hit2
5' UACAAUCU-C-AAGAUGGCAGCA
   | |||||| | ||||||||||||
   AAGUUAGAAGCUUCUACCGUCGU 5'
AT4G08390.1     1063    1085
stromal ascorbate peroxidase

sRNA_AGO1_Solexa_Mi2008_1_50784_hit1
5' UUGAGGUAAAUGAUGUUCCCCCAU
   ||||| ||||| |||| |||||||
   AACUC-AUUUAAUACACGGGGGUA 5'
AT4G08390.3     1066    1088
stromal ascorbate peroxidase

sRNA_AGO1_Solexa_Mi2008_1_50784_hit1
5' UUGAGGUAAAUGAUGUUCCCCCAU
   ||||| ||||| |||| |||||||
   AACUC-AUUUAAUACACGGGGGUA 5'
AT4G08390.2     1203    1225
stromal ascorbate peroxidase
```

```
sRNA_AGO1_Solexa_Mi2008_1_50784_hit1
5' UUGAGGUAAAUGAUGUUCCCCCAU
   ||||| ||||| |||| |||||||
   AACUC-AUUUAAUACACGGGGGUA 5'
AT4G08390.1     1213    1235
stromal ascorbate peroxidase

sRNA_AGO1_Solexa_Mi2008_3_10123_hit2
5' CUAGAGACGAGAGAU-U-ACU
   ||||||||||||||| | ||
   GAUCUCUGCUCUCUAGACUGC 5'
AT4G08390.3     130     150
stromal ascorbate peroxidase

sRNA_AGO1_Solexa_Mi2008_3_10123_hit2
5' CUAGAGACGAGAGAU-U-ACU
   ||||||||||||||| | ||
   GAUCUCUGCUCUCUAGACUGC 5'
AT4G08390.2     264     284
stromal ascorbate peroxidase

sRNA_AGO1_Solexa_Mi2008_3_10123_hit2
5' CUAGAGACGAGAGAU-U-ACU
   ||||||||||||||| | ||
   GAUCUCUGCUCUCUAGACUGC 5'
AT4G08390.1     274     294
stromal ascorbate peroxidase

sRNA_AGO1_Solexa_Mi2008_44_10639_hit2
5' CUC-AAGAUGGCAGCAU-AGU
    || ||||||||||||| |||
   UAGCUUCUACCGUCGUAGUCA 5'
AT4G08390.3     911     931
stromal ascorbate peroxidase

sRNA_AGO1_Solexa_Mi2008_5_5124_hit2
5' CAAUCU-C-AAGAUGGCAGCAU
   |||||| | |||||||||||||
   GUUAGAAGCUUCUACCGUCGUA 5'
AT4G08390.3     915     936
stromal ascorbate peroxidase

sRNA_AGO1_Solexa_Mi2008_5_19199_hit2
5' UACAAUCU-C-AAGAUGGCAGCAU
   | |||||| | |||||||||||||
   AAGUUAGAAGCUUCUACCGUCGUA 5'
AT4G08390.3     915     938
stromal ascorbate peroxidase

sRNA_AGO1_Solexa_Mi2008_1_19198_hit2
5' UACAAUCU-C-AAGAUGGCAGCA
   | |||||| | ||||||||||||
   AAGUUAGAAGCUUCUACCGUCGU 5'
AT4G08390.3     916     938
stromal ascorbate peroxidase

sRNA_AGO1_Solexa_Mi2008_2_411_hit5
5' AAAGAUGA-AGAGAGAAAGAGA
    |||| || ||||||||||||
   GUUCU-CUCUCUCUCUUUCUCU 5'
AT4G08500.1     39      59
MEKK1/MAP kinase kinase kinase
```

```
sRNA_AGO1_Solexa_Mi2008_1_41654_hit1
5' UGGCUUA-GCUACUGGUGGUU
   ||||||| | ||||||||||:
   ACCGAAUUC-AUGACCACCAG 5'
AT4G08500.1     913     932
MEKK1/MAP kinase kinase kinase

sRNA_AGO1_Solexa_Mi2008_1_51111_hit1
5' UUGAUGAUGAUGAUGGUUUGA
     ||||||||||||||||||
   CUCUACUACUACUACCAAACA 5'
AT4G09730.1     1687    1707
putative protein

sRNA_AGO1_Solexa_Mi2008_1_40485_hit1
5' UGGAGGA-AUGAUGAUGAUGUG
   ||||||| ||||||||||||
   ACCUCCUCUACUACUACUACCA 5'
AT4G09730.1     1692    1713
putative protein

sRNA_AGO1_Solexa_Mi2008_1_36323_hit13
5' UGAGGAUGAUGAUGAUGAUGA
   :||||| |||||||||||||
   GCUCCU-CUACUACUACUACC 5'
AT4G09730.1     1693    1712
putative protein

sRNA_AGO1_Solexa_Mi2008_1_38921_hit1
5' UGC-GGACGAUGAUGAUGAUGAU
   ||| ||| || ||||||||||||
   ACGUCCUCCU-CUACUACUACUA 5'
AT4G09730.1     1695    1716
putative protein

sRNA_AGO1_Solexa_Mi2008_1_36323_hit13
5' UG-AGGAUGAUGAUGAUGAUGA
   || |||| || |||||||||||
   ACGUCCUCCU-CUACUACUACU 5'
AT4G09730.1     1696    1716
putative protein

sRNA_AGO1_Solexa_Mi2008_1_15319_hit1
5' GGUGGAAGCUGUGGUU-GUGGA
   ||||||| ||| |||| |||||
   CCACCUUAGAC-CCAAACACCU 5'
AT4G09730.1     179     199
putative protein

sRNA_AGO1_Solexa_Mi2008_1_3030_hit2
5' AUGAGGAGGAAGAUGGUU-UG
    |||||||||||| |||| ||
   GACUCCUCCUUCUCCCAACAC 5'
AT4G09730.1     34      54
putative protein

sRNA_AGO1_Solexa_Mi2008_13_5218_hit2
5' CAAU-UCAGUAGGGACCUCAAG
   |||| ||||||||:|||||||
   GUUAGAGUCAUCCUUGGAGUUA 5'
AT4G09730.1     478     499
putative protein
```

```
sRNA_AGO1_Solexa_Mi2008_1_11311_hit1
5' CUGA-A-AAAAAAAGAGAGGG
   |||| | ||||||||||||
   GACUCUGUUUUUUUCUCUCCG 5'
AT4G10970.3     20      40
putative protein

sRNA_AGO1_Solexa_Mi2008_1_11311_hit1
5' CUGA-A-AAAAAAAGAGAGGG
   |||| | ||||||||||||
   GACUCUGUUUUUUUCUCUCCG 5'
AT4G10970.4     38      58
putative protein

sRNA_AGO1_Solexa_Mi2008_1_3358_hit1
5' AUGGUA-GUUGAUGGUAGUUGU
   |||||| ||||||| ||||||
   UACCAUUCAACUACAAUCAACC 5'
AT4G11170.1     2083    2104
RPP1-WsA-like disease resistance protein

sRNA_AGO1_Solexa_Mi2008_1_173_hit2
5' AAAAGAAGAAGAUAA-AGCAU
   ||||||||||||:|| ||||
   UUUUCUUCUUCUGUUAUCGUU 5'
AT4G11890.1     1159    1179
protein kinase - like protein

sRNA_AGO1_Solexa_Mi2008_1_172_hit2
5' AAAAGAAGAAGAUAA-AGCA
   ||||||||||||:|| ||||
   UUUUCUUCUUCUGUUAUCGU 5'
AT4G11890.1     1160    1179
protein kinase - like protein

sRNA_AGO1_Solexa_Mi2008_2_16280_hit2
5' UAAAAAGAAGAAGAUAA-AGCA
    |||||||||||||:|| ||||
   CUUUUUCUUCUUCUGUUAUCGU 5'
AT4G11890.1     1160    1181
protein kinase - like protein

sRNA_AGO1_Solexa_Mi2008_1_173_hit2
5' AAAAGAAGAAGAUAA-AGCAU
   ||||||||||||:|| ||||
   UUUUCUUCUUCUGUUAUCGUU 5'
AT4G11890.3     1170    1190
protein kinase - like protein

sRNA_AGO1_Solexa_Mi2008_1_172_hit2
5' AAAAGAAGAAGAUAA-AGCA
   ||||||||||||:|| ||||
   UUUUCUUCUUCUGUUAUCGU 5'
AT4G11890.3     1171    1190
protein kinase - like protein

sRNA_AGO1_Solexa_Mi2008_2_16280_hit2
5' UAAAAAGAAGAAGAUAA-AGCA
    |||||||||||||:|| ||||
   CUUUUUCUUCUUCUGUUAUCGU 5'
AT4G11890.3     1171    1192
protein kinase - like protein
```

```
sRNA_AGO1_Solexa_Mi2008_1_173_hit2
5' AAAAGAAGAAGAUAA-AGCAU
   ||||||||||||:|| ||||
   UUUUCUUCUUCUGUUAUCGUU 5'
AT4G11890.4     1190    1210
protein kinase - like protein

sRNA_AGO1_Solexa_Mi2008_1_172_hit2
5' AAAAGAAGAAGAUAA-AGCA
   ||||||||||||:|| ||||
   UUUUCUUCUUCUGUUAUCGU 5'
AT4G11890.4     1191    1210
protein kinase - like protein

sRNA_AGO1_Solexa_Mi2008_2_16280_hit2
5' UAAAAAGAAGAAGAUAA-AGCA
    |||||||||||||:|| ||||
   CUUUUUCUUCUUCUGUUAUCGU 5'
AT4G11890.4     1191    1212
protein kinase - like protein

sRNA_AGO1_Solexa_Mi2008_1_173_hit2
5' AAAAGAAGAAGAUAA-AGCAU
   ||||||||||||:|| ||||
   UUUUCUUCUUCUGUUAUCGUU 5'
AT4G11890.2     1545    1565
protein kinase - like protein

sRNA_AGO1_Solexa_Mi2008_1_172_hit2
5' AAAAGAAGAAGAUAA-AGCA
   ||||||||||||:|| ||||
   UUUUCUUCUUCUGUUAUCGU 5'
AT4G11890.2     1546    1565
protein kinase - like protein

sRNA_AGO1_Solexa_Mi2008_2_16280_hit2
5' UAAAAAGAAGAAGAUAA-AGCA
    |||||||||||||:|| ||||
   CUUUUUCUUCUUCUGUUAUCGU 5'
AT4G11890.2     1546    1567
protein kinase - like protein

sRNA_AGO1_Solexa_Mi2008_1_29903_hit2
5' UCCGCAAAACCAAGGUCCUGC
    || ||||| |||||||||||
   CGG-GUUUUGUUCCAGGACG 5'
AT4G12120.1     1478    1497
unknown protein

sRNA_AGO1_Solexa_Mi2008_1_1044_hit2
5' AAUCCUGAUGAUG-CUGCAU
   ||||||||||||| || |||
   UUAGGACUACUACUGAGGUA 5'
AT4G12560.2     1555    1574
putative protein

sRNA_AGO1_Solexa_Mi2008_2_1701_hit3
5' ACUCAAGAGAUGAU-AUAAA
     |||||||||||| |||||
   AUAGUUCUCUACUAUUAUUU 5'
AT4G12560.2     653     672
putative protein
```

```
sRNA_AGO1_Solexa_Mi2008_1_14470_hit10
5' GCAGC-CCGACGUAAUCAGA
   |||:| |||||||||||||
   CGUUGCGGCUGCAUUAGUCU 5'
AT4G12570.1     329     348
polyubiquitin-like protein

sRNA_AGO1_Solexa_Mi2008_1_37137_hit1
5' UGAUCUGGUGAAGUGUUCGG
   |||||||||||||| |||
   ACUAGACCACUUCA-AAGAA 5'
AT4G12730.1     365     383
fasciclin-like arabinogalactan protein FLA2

sRNA_AGO1_Solexa_Mi2008_1_29609_hit1
5' UCCCAAAACAAUAUUGU-AGUA
   | ||||||||||||| | ||||
   AUGGUUUUGUUAUAA-AUUCAU 5'
AT4G13510.1     1604    1624
ammonium transport protein (AMT1)

sRNA_AGO1_Solexa_Mi2008_9_5443_hit29
5' CACCACCAUCACCAUCACUCG
   |||||||||||||| | |||
   GUGGUGGUAGUGGU-G-GAGG 5'
AT4G13850.4     431     449
glycine-rich RNA-binding protein AtGRP2 - like

sRNA_AGO1_Solexa_Mi2008_9_5443_hit29
5' CACCACCAUCACCAUCACUCG
   |||||||||||||| | |||
   GUGGUGGUAGUGGU-G-GAGG 5'
AT4G13850.3     476     494
glycine-rich RNA-binding protein AtGRP2 - like

sRNA_AGO1_Solexa_Mi2008_9_5443_hit29
5' CACCACCAUCACCAUCACUCG
   |||||||||||||| | |||
   GUGGUGGUAGUGGU-G-GAGG 5'
AT4G13850.2     503     521
glycine-rich RNA-binding protein AtGRP2 - like

sRNA_AGO1_Solexa_Mi2008_9_5443_hit29
5' CACCACCAUCACCAUCACUCG
   |||||||||||||| | |||
   GUGGUGGUAGUGGU-G-GAGG 5'
AT4G13850.1     517     535
glycine-rich RNA-binding protein AtGRP2 - like

sRNA_AGO1_Solexa_Mi2008_16_13755_hit1
5' GACGAUAAGCAUUGGCAUU
   :|||||||||||||:||||
   UUGCUAUUCGUAACUGUAA 5'
AT4G14365.1     281     299
C3HC4-type zinc finger ankyrin repeat protein - like

sRNA_AGO1_Solexa_Mi2008_1_51487_hit1
5' UUGCCACAUGUAG-GGAUGUC
    |||||||||||| || ||||
   UACGGUGUACAUCGCC-ACAG 5'
AT4G14400.2     424     443
unknown protein
```

```
sRNA_AGO1_Solexa_Mi2008_1_51487_hit1
5' UUGCCACAUGUAG-GGAUGUC
    |||||||||||| || ||||
   UACGGUGUACAUCGCC-ACAG 5'
AT4G14400.3     488     507
unknown protein

sRNA_AGO1_Solexa_Mi2008_1_51487_hit1
5' UUGCCACAUGUAG-GGAUGUC
    |||||||||||| || ||||
   UACGGUGUACAUCGCC-ACAG 5'
AT4G14400.1     507     526
unknown protein

sRNA_AGO1_Solexa_Mi2008_4_50857_hit1
5' UUGAGGUGUUGAUUAAAGUG-UU
   |||||||||||||:||| || ||
   AACUCCACAACUAGUUUGACGAA 5'
AT4G14746.1     202     224
putative protein

sRNA_AGO1_Solexa_Mi2008_3_10294_hit3
5' CUAGGGUUUCAAUGAU-UGUA
   ||||||||||||| || |||
   GAUCCCAAAGUUAGUAGACAG 5'
AT4G14900.1     268     288
hydroxyproline-rich glycoprotein homolog (Z97337.18)

sRNA_AGO1_Solexa_Mi2008_1_5287_hit5
5' CACAAGGACAAGAAAUUGG
    |||||| ||||||||||
   UUGUUCCCGUUCUUUAACG 5'
AT4G14905.1     1319    1337

sRNA_AGO1_Solexa_Mi2008_1_5287_hit5
5' CACAAGGACAAGAAAUUGG
    |||||| ||||||||||
   UUGUUCCCGUUCUUUAACG 5'
AT4G14905.2     1555    1573

sRNA_AGO1_Solexa_Mi2008_12_20785_hit2
5' UAGAAAGGGCAAUAUACACGUG
   |||||||||||| | ||| |||
   AUCUUUCCCGUU-U-UGUACAC 5'
AT4G16370.1     2273    2292
isp4 like protein

sRNA_AGO1_Solexa_Mi2008_1_13420_hit2
5' GAAGAAGAAGAAGACUCUU
   ||||||||||||||||
   CUUCUUCUUCUUCUGAGCC 5'
AT4G16745.1     406     424

sRNA_AGO1_Solexa_Mi2008_2_16_hit1
5' AAAAAAAGAGAGGGACGAA
   ||||||| ||||||| |||
   UUUUUUUGUCUCCCU-CUU 5'
AT4G16760.1     78      95
unknown protein

sRNA_AGO1_Solexa_Mi2008_1_2_hit38
5' AAAAAAAAAAAAAACCAU
```

   ||||||||||||||||
   UUUUUUUUUUUUUUUGUCU 5'
AT4G16760.1   84   102
unknown protein

sRNA_AGO1_Solexa_Mi2008_1_1776_hit10
5' AGAAAAAAAAAAAAAAAAAAU
    |||||||||||||||||||:
   CCUUUUUUUUUUUUUUUUUUG 5'
AT4G16760.1   87   107
unknown protein

sRNA_AGO1_Solexa_Mi2008_1_3_hit25
5' AAAAAAAAAAAAAGAAAGA
   ||||||||||||| |||:
   UUUUUUUUUUUUU-UUUUG 5'
AT4G16760.1   88   105
unknown protein

sRNA_AGO1_Solexa_Mi2008_1_3_hit25
5' AAAAAAAAAAAAAGAAAGA
   ||||||||||||| |||:|
   UUUUUUUUUUUUU-UUUUU 5'
AT4G16760.1   89   106
unknown protein

sRNA_AGO1_Solexa_Mi2008_1_48909_hit1
5' UUCAUUGAAUAGCACAUGUGA
   |||||| |||||||||| |||
   AAGUAAAUUAUCGUGUAAACU 5'
AT4G17660.1   714   734
NAK like protein kinase

sRNA_AGO1_Solexa_Mi2008_2_5924_hit1
5' CAGAACGGUGUGGUC-CCUCUGU
   |||||||||||| || |||||||
   GUCUUGCCACAC-AGAGGAGACA 5'
AT4G17670.1   351   372
unknown protein

sRNA_AGO1_Solexa_Mi2008_2_20775_hit1
5' UAGAAAGAGU-UUUAGGUGAAGA
   |||||||| | |||||:||||||
   AUCUUUCU-AGAAAUCUACUUCU 5'
AT4G17670.1   634   655
unknown protein

sRNA_AGO1_Solexa_Mi2008_2_34699_hit1
5' UGAAGCUG-CUUCUGGUGGUUC
   |||||||| ||| |||||:|||
   ACUUCGACGGAA-ACCACUAAG 5'
AT4G17720.1   940   960
unknown protein

sRNA_AGO1_Solexa_Mi2008_1_36919_hit1
5' UGAGUUU-GAUGCACGUUCGGA
   ||||||| ||| |||||||||
   ACUCAAAACUAAGUGCAAGCCG 5'
AT4G17720.1   973   994
unknown protein

sRNA_AGO1_Solexa_Mi2008_2_50953_hit1
5' UUGAGUUU-GAUGCACGUUCGG

```
   :||||||| ||| |||||||||
   GACUCAAAACUAAGUGCAAGCC 5'
AT4G17720.1     974     995
unknown protein

sRNA_AGO1_Solexa_Mi2008_5_24931_hit2
5' UAUC-AUUGUGAAGCAGAAUUC
   |||| |||||||||||||| :|
   AUAGCUAACACUUCGUCUUUGG 5'
AT4G18270.1     593     614
translocase I

sRNA_AGO1_Solexa_Mi2008_6_649_hit8
5' AA-CAGACGCUCUAUCCACU
   || |||||| |||||||||
   UUCGUCUGCCAGAUAGGUGC 5'
AT4G19190.1     1354    1373

sRNA_AGO1_Solexa_Mi2008_1_56030_hit2
5' UUUGUAGAGAAACAUGAGAGA
   |||| ||||||| ||||||||
   AAAC-UCUCUUUCUACUCUCU 5'
AT4G20830.2     101     120
reticuline oxidase -like protein

sRNA_AGO1_Solexa_Mi2008_1_56030_hit2
5' UUUGUAGAGAAACAUGAGAGA
   |||| ||||||| ||||||||
   AAAC-UCUCUUUCUACUCUCU 5'
AT4G20830.1     108     127
reticuline oxidase -like protein

sRNA_AGO1_Solexa_Mi2008_4_6008_hit1
5' CAGACUGAGAUUCGUCGUUGGU
   ||||| ||| ||||||||||||
   GUCUG-CUCAAAGCAGCAACCA 5'
AT4G21540.2     16      36

sRNA_AGO1_Solexa_Mi2008_4_6008_hit1
5' CAGACUGAGAUUCGUCGUUGGU
   ||||| ||| ||||||||||||
   GUCUG-CUCAAAGCAGCAACCA 5'
AT4G21540.1     2334    2354

sRNA_AGO1_Solexa_Mi2008_4_6008_hit1
5' CAGACUGAGAUUCGUCGUUGGU
   ||||| ||| ||||||||||||
   GUCUG-CUCAAAGCAGCAACCA 5'
AT4G21540.4     23      43

sRNA_AGO1_Solexa_Mi2008_1_35652_hit7
5' UGACU-C-AACAUGACCGGCGU
   ||||| | |||||||||||||
   ACUGAUGGUUGUACUGGCCGCU 5'
AT4G21910.3     301     322
unknown protein

sRNA_AGO1_Solexa_Mi2008_1_35652_hit7
5' UGACU-C-AACAUGACCGGCGU
   ||||| | |||||||||||||
   ACUGAUGGUUGUACUGGCCGCU 5'
AT4G21910.1     362     383
unknown protein
```

```
sRNA_AGO1_Solexa_Mi2008_1_38809_hit1
5' UGCGAGCUGAUGGUCCACAAG
   |||||||||||||||||||||
   ACGCUCGACUACCAGGUGUUC 5'
AT4G21960.1     957     977
peroxidase prxr1

sRNA_AGO1_Solexa_Mi2008_105_14948_hit3
5' GGCAAUAACAGGUCUGUGA
    |||||||||||||| ||
   ACGUUAUUGUCCAGA-ACA 5'
AT4G22380.1     364     381
Ribosomal protein L7Ae - like (fragment)

sRNA_AGO1_Solexa_Mi2008_3_2154_hit3
5' AGGCAAUAACAGGUCUGUG
     |||||||||||||| ||
   AACGUUAUUGUCCAGA-AC 5'
AT4G22380.1     365     382
Ribosomal protein L7Ae - like (fragment)

sRNA_AGO1_Solexa_Mi2008_1_14875_hit1
5' GGAGGUGGAGGCGGUGG-UGGU
   : ||||||||||||||| ||||
   UGUCCACCUCCGCCACCAACCA 5'
AT4G22470.1     207     228
extensin - like protein

sRNA_AGO1_Solexa_Mi2008_2_43577_hit1
5' UGGUGGUGGUGACG-UUGGUGGU
   |||||||||||| | ||| ||||
   ACCACCACCACU-CUAACAACCA 5'
AT4G22470.1     778     799
extensin - like protein

sRNA_AGO1_Solexa_Mi2008_1_43578_hit1
5' UGGUGGUGGUGAUGAUGUGUC
   |||||||||||| ||| |||
   ACCACCACCACU-CUA-ACAA 5'
AT4G22470.1     781     799
extensin - like protein

sRNA_AGO1_Solexa_Mi2008_4_10367_hit4
5' CUAGUGAGGAAUGAGAAAAGG
   |||||||||||||||||||||
   GAUCACUCCUUACUCUUUUCC 5'
AT4G23150.1     32      52
serine/threonine kinase - like protein

sRNA_AGO1_Solexa_Mi2008_2_29105_hit1
5' UCAUUGAGUACUGACCAUGCA
   |||| ||||| ||||||||||
   AGUACCUCAUAACUGGUACGU 5'
AT4G23190.1     1696    1716
serine/threonine kinase - like protein

sRNA_AGO1_Solexa_Mi2008_9_14254_hit8
5' GAUGAUGAUGAUGAUGAUCUU
   ||||||||||| ||||| ||
   CUACUACUACUCCUACU-GAU 5'
AT4G23210.2     926     945
serine/threonine kinase - like protein
```

```
sRNA_AGO1_Solexa_Mi2008_1_36323_hit13
5' UGAGGAUGAUGAUGAUGAUGA
    ||| |||||||||| |||||
   CCUC-UACUACUACUCCUACU 5'
AT4G23210.2     929     948
serine/threonine kinase - like protein

sRNA_AGO1_Solexa_Mi2008_1_7931_hit2
5' CCCGAUGAUGAUGAUGAGAC
   ||||| ||||||||||||
   GGGCU-CUACUACUACUCCU 5'
AT4G23210.2     932     950
serine/threonine kinase - like protein

sRNA_AGO1_Solexa_Mi2008_9_14254_hit8
5' GAUGAUGAUGAUGAUGAUCUU
   ||||||||||| ||||| ||
   CUACUACUACUCCUACU-GAU 5'
AT4G23210.3     987     1006
serine/threonine kinase - like protein

sRNA_AGO1_Solexa_Mi2008_1_36323_hit13
5' UGAGGAUGAUGAUGAUGAUGA
    ||| |||||||||| |||||
   CCUC-UACUACUACUCCUACU 5'
AT4G23210.3     990     1009
serine/threonine kinase - like protein

sRNA_AGO1_Solexa_Mi2008_1_7931_hit2
5' CCCGAUGAUGAUGAUGAGAC
   ||||| ||||||||||||
   GGGCU-CUACUACUACUCCU 5'
AT4G23210.3     993     1011
serine/threonine kinase - like protein

sRNA_AGO1_Solexa_Mi2008_1_1652_hit1
5' ACGGUGCGUGAAUUGUAUU
   |||||||||||||||||||
   UGCCACGCACUUAACAUAA 5'
AT4G23470.3     1161    1179
putative protein

sRNA_AGO1_Solexa_Mi2008_2_24561_hit1
5' UAUAGGGUGCGUGAAU-GUAAU
   |||| ||||||||||| ||| |
   AUAUGCCACGCACUUAACAUAA 5'
AT4G23470.3     1162    1183
putative protein

sRNA_AGO1_Solexa_Mi2008_1_1652_hit1
5' ACGGUGCGUGAAUUGUAUU
   |||||||||||||||||||
   UGCCACGCACUUAACAUAA 5'
AT4G23470.1     1227    1245
putative protein

sRNA_AGO1_Solexa_Mi2008_2_24561_hit1
5' UAUAGGGUGCGUGAA-UGUAAU
   |||| |||||||||| |||| |
   AUAUGCCACGCACUUAACAUAA 5'
AT4G23470.1     1228    1249
putative protein
```

```
sRNA_AGO1_Solexa_Mi2008_1_1652_hit1
5' ACGGUGCGUGAAUUGUAUU
   ||||||||||||||||||
   UGCCACGCACUUAACAUAA 5'
AT4G23470.2     1575    1593
putative protein

sRNA_AGO1_Solexa_Mi2008_2_24561_hit1
5' UAUAGGGUGCGUGAA-UGUAAU
   |||| |||||||||| |||| |
   AUAUGCCACGCACUUAACAUAA 5'
AT4G23470.2     1576    1597
putative protein

sRNA_AGO1_Solexa_Mi2008_2_7736_hit1
5' CCAGAA-AAGAAGAAACAAU
    ||||| ||||||||||||
   UGUCUUCUUCUUCUUUGUUC 5'
AT4G23470.2     660     679
putative protein

sRNA_AGO1_Solexa_Mi2008_2_20292_hit1
5' UACGGGU-AUGCCUCGGGGUGCU
   ||||| | ||||||||||:||||
   AUGCCAAAUACGGAGCCCUACGA 5'
AT4G23700.1     1582    1604
putative Na+/H+-exchanging protein

sRNA_AGO1_Solexa_Mi2008_1_31650_hit1
5' UCGCUUAUGAGA-UUGUGGAUC
   ||||||| |||| ||| |||||
   AGCGAAUUCUCUAAAC-CCUAG 5'
AT4G23700.1     500     520
putative Na+/H+-exchanging protein

sRNA_AGO1_Solexa_Mi2008_7_3078_hit1
5' AUGAUGAUGAGAAUGAUGAU
   || ||||||||||||| ||
   UA-UACUACUCUUACU-CUC 5'
AT4G23810.1     13      30
unknown protein

sRNA_AGO1_Solexa_Mi2008_2_56577_hit1
5' UUUUGG-UCGGUGUGUAUGU
   |||||| |||||| |||||
   AAAACCCAGCCACUCAUACU 5'
AT4G24190.2     2303    2322
HSP90-like protein

sRNA_AGO1_Solexa_Mi2008_2_56577_hit1
5' UUUUGG-UCGGUGUGUAUGU
   |||||| |||||| |||||
   AAAACCCAGCCACUCAUACU 5'
AT4G24190.1     2307    2326
HSP90-like protein

sRNA_AGO1_Solexa_Mi2008_9_24682_hit1
5' UAUAUGCAAAAGAGUAAGAUG
   |||||||||||||:| ||||
   AUAUACGUUUUCUUA-UCUAA 5'
AT4G24230.6     1156    1175
unknown protein
```

```
sRNA_AGO1_Solexa_Mi2008_9_24682_hit1
5' UAUAUGCAAAAGAGUAAGAUG
   |||||||||||||:|| |||
   AUAUACGUUUUCUUAU-CUAA 5'
AT4G24230.3     1166    1185
unknown protein

sRNA_AGO1_Solexa_Mi2008_9_24682_hit1
5' UAUAUGCAAAAGAGUAAGAUG
   |||||||||||||:|| |||
   AUAUACGUUUUCUUAU-CUAA 5'
AT4G24230.4     1170    1189
unknown protein

sRNA_AGO1_Solexa_Mi2008_9_24682_hit1
5' UAUAUGCAAAAGAGUAAGAUG
   |||||||||||||:| ||||
   AUAUACGUUUUCUUA-UCUAA 5'
AT4G24230.1     1217    1236
unknown protein

sRNA_AGO1_Solexa_Mi2008_9_24682_hit1
5' UAUAUGCAAAAGAGUAAGAUG
   |||||||||||||:| ||||
   AUAUACGUUUUCUUA-UCUAA 5'
AT4G24230.2     1261    1280
unknown protein

sRNA_AGO1_Solexa_Mi2008_2_12201_hit1
5' CUGUCCUCGAAAUAAAGAUCU
   ||||||||||| | ||||||
   GACAGGAGCUUGA-UUCUAGU 5'
AT4G24230.6     377     396
unknown protein

sRNA_AGO1_Solexa_Mi2008_2_12201_hit1
5' CUGUCCUCGAAAUAAAGAUCU
   ||||||||||| | ||||||
   GACAGGAGCUUGA-UUCUAGU 5'
AT4G24230.4     390     409
unknown protein

sRNA_AGO1_Solexa_Mi2008_2_12201_hit1
5' CUGUCCUCGAAAUAAAGAUCU
   ||||||||||| | ||||||
   GACAGGAGCUUGA-UUCUAGU 5'
AT4G24230.5     391     410
unknown protein

sRNA_AGO1_Solexa_Mi2008_2_12201_hit1
5' CUGUCCUCGAAAUAAAGAUCU
   ||||||||||| | ||||||
   GACAGGAGCUUGA-UUCUAGU 5'
AT4G24230.3     393     412
unknown protein

sRNA_AGO1_Solexa_Mi2008_2_12201_hit1
5' CUGUCCUCGAAAUAAAGAUCU
   ||||||||||| | ||||||
   GACAGGAGCUUGA-UUCUAGU 5'
AT4G24230.1     444     463
unknown protein
```

```
sRNA_AGO1_Solexa_Mi2008_2_12201_hit1
5' CUGUCCUCGAAAUAAAGAUCU
   ||||||||||| | ||||||
   GACAGGAGCUUGA-UUCUAGU 5'
AT4G24230.2     469     488
unknown protein

sRNA_AGO1_Solexa_Mi2008_1_16810_hit10
5' UAAAG-GAGAACAUGACGGUGG
   | ||| |||||||||| |||||
   AGUUCGCUCUUGUACUACCACC 5'
AT4G24330.1     153     174
unknown protein

sRNA_AGO1_Solexa_Mi2008_1_36276_hit1
5' UG-AGGAGAGAGUGAUUUUCUGU
   || |||||||| ||||||||||
   ACGUCCUCUCUGACUAAAAGACU 5'
AT4G24390.1     2235    2257
transport inhibitor response-like protein

sRNA_AGO1_Solexa_Mi2008_13_14047_hit1
5' GAGGAAGACGAGGAGGAAGAGGA
   |||||||| |||||||| |||||
   CUCCUUCUUCUCCUCCUCCUCCU 5'
AT4G24390.2     223     245
transport inhibitor response-like protein

sRNA_AGO1_Solexa_Mi2008_1_7006_hit1
5' CAUCAUCAUCAUCACAGAAG
     ||| ||||||||||||||
   CGAGU-GUAGUAGUGUCUUC 5'
AT4G24690.1     2174    2192
unknown protein

sRNA_AGO1_Solexa_Mi2008_6_22289_hit1
5' UAGC-CUGUACGAUGAUGUUGU
   |||| |:| |||||||||||||
   AUCGUGGC-UGCUACUACAACA 5'
AT4G24920.1     256     276
PROTEIN TRANSPORT PROTEIN SEC61 GAMMA SUBUNIT -like

sRNA_AGO1_Solexa_Mi2008_1_50495_hit1
5' UUGAGACCCGACGCGAUGGC
   |||||||||||| |||||
   AACUCUGGGCUG-GCUACGA 5'
AT4G25000.1     760     778
alpha-amylase  like protein

sRNA_AGO1_Solexa_Mi2008_2_28261_hit2
5' UCAGC-ACGGUUACUCGUUGGA
   ||||| | |||||||| |||||
   AGUCGAU-CCAAUGAGGAACCU 5'
AT4G25340.1     43      63
unknown protein

sRNA_AGO1_Solexa_Mi2008_3_19661_hit1
5' UACCAAAGGAUCUGCCACUCGC
   :||||||| |||||||| ||||
   GUGGUUUCGUAGACGGUUAGCG 5'
AT4G25710.1     1027    1048
putative protein
```

sRNA_AGO1_Solexa_Mi2008_2_5430_hit1
5' CACCAAUAGAUCAUAAAAU
     ||||||:||||||||||
   UCGGUUAUUUAGUAUUUUA 5'
AT4G25710.1   1250   1268
putative protein

sRNA_AGO1_Solexa_Mi2008_2_48800_hit1
5' UUCAGUAACAGGUUUCAUUGUA
   ||| ||||||||||||||||
   AAG-CAUUGUCCAAAGUAACCA 5'
AT4G25730.1   2084   2104
unknown protein

sRNA_AGO1_Solexa_Mi2008_1_9509_hit1
5' CUAAACGAUGAGGAUGAUGAA
    |||| |||||||||||||||
   AAUUUCCUACUCCUACUACUU 5'
AT4G25900.1   1116   1136
possible apospory-associated like protein

sRNA_AGO1_Solexa_Mi2008_1_33495_hit1
5' UCUGCAGAAAUGGUGGGAGAC
   |||||||||||||||||||||
   AGACGUCUUUACCACCCUCUG 5'
AT4G26400.2   64   84
unknown protein

sRNA_AGO1_Solexa_Mi2008_1_33495_hit1
5' UCUGCAGAAAUGGUGGGAGAC
   |||||||||||||||||||||
   AGACGUCUUUACCACCCUCUG 5'
AT4G26400.1   87   107
unknown protein

sRNA_AGO1_Solexa_Mi2008_1_3_hit25
5' AAAAAAAAAAAAAGAAAGA
   ||||||||||||| |||
   UUUUUUUUUUUUU-UUUAG 5'
AT4G27020.1   1723   1740
unknown protein

sRNA_AGO1_Solexa_Mi2008_19_10930_hit1
5' CUCCUCGCCAUAGUU-UUCGCCUUU
   ||||||| ||||||| ||| |||||
   GAGGAGCAGUAUCAACAAG-GGAAA 5'
AT4G27430.2   1747   1770
COP1-interacting protein 7 (CIP7)

sRNA_AGO1_Solexa_Mi2008_1_3966_hit1
5' CAAAAUUCAAAAGAUUCUUAA
    ||||| |||||||||||||
   UUUUUAUGUUUUCUAAGAAUG 5'
AT4G27430.1   3567   3587
COP1-interacting protein 7 (CIP7)

sRNA_AGO1_Solexa_Mi2008_1_50088_hit1
5' UUGAAGGAUCAUCAGUGUGAAA
   |||||||||||| |||| |||
   AACUUCCUAGUA-UCACUCUUG 5'
AT4G27710.1   1520   1540
cytochrome P450 - like protein

```
sRNA_AGO1_Solexa_Mi2008_7_8338_hit9
5' CGAAGG-UGCAUAGUGAGAA
    ||||| | ||||||||||
   UCUUCCUA-GUAUCACUCUU 5'
AT4G27710.1     1521    1539
cytochrome P450 - like protein

sRNA_AGO1_Solexa_Mi2008_1_1573_hit9
5' ACGAAGG-UGCAUAGUGAGAA
   | ||||| | ||||||||||
   UUCUUCCUA-GUAUCACUCUU 5'
AT4G27710.1     1521    1540
cytochrome P450 - like protein

sRNA_AGO1_Solexa_Mi2008_1_50087_hit1
5' UUGAAGGAUCAUCAGUGUGAA
   ||||||||||| |||| |||
   AACUUCCUAGUA-UCACUCUU 5'
AT4G27710.1     1521    1540
cytochrome P450 - like protein

sRNA_AGO1_Solexa_Mi2008_1_10208_hit1
5' CUAGC-GAGAAAACUCUUUC
   |  || |||||||||||||
   GCGCGUCUCUUUUGAGAAAG 5'
AT4G27940.1     458     477
unknown protein

sRNA_AGO1_Solexa_Mi2008_8_15580_hit6
5' GUCAAAGUGAGAU-AUGGUUUU
   ||||||||||| |  |||||||
   CAGUUUCACUC-ACGACCAAAA 5'
AT4G28250.2     476     496
putative Expansin (AtEXPB3) /allergen protein

sRNA_AGO1_Solexa_Mi2008_1_43726_hit3
5' UGGUUAAGGAGAUAGACUUGA
   ||||| || ||||||||||
   ACCAAAUC-UCUAUCUGAACC 5'
AT4G28490.1     86      105
receptor-like protein kinase 5 precursor (RLK5)

sRNA_AGO1_Solexa_Mi2008_1_5817_hit1
5' CACUGGUUGGUUGGAUAGU
   |||||||||||| ||||:|
   GUGACCAACCAAACUAUUA 5'
AT4G28940.1     321     339
putative protein

sRNA_AGO1_Solexa_Mi2008_1_49711_hit26
5' UUCUCAACAAUG-C-AUCAUA
   |||||||||||| | ||| ||
   AAGAGUUGUUACAGUUAGAAU 5'
AT4G29520.1     373     393
unknown protein

sRNA_AGO1_Solexa_Mi2008_16_13022_hit1
5' CUUGGUGGCUUAGUGGGCUAA
   ||||||||||:||| ||||||
   GAACCACCGAGUCA-CCGAUU 5'
AT4G29900.1     2680    2699
```

```
sRNA_AGO1_Solexa_Mi2008_3_5873_hit1
5' CAGAAAAGAGGUAAACGAG
    |||||||||||||||| :
   CUCUUUUCUCCAUUUGCAU 5'
AT4G29900.1     3272    3290

sRNA_AGO1_Solexa_Mi2008_8_52273_hit1
5' UUGGCAAGUUAGAGGAUGUG-UC
   |||||||||| |||||| || ||
   AACCGUUCAAGCUCCUAGACGAG 5'
AT4G30190.1     1415    1437
H+-transporting ATPase type 2, plasma membrane

sRNA_AGO1_Solexa_Mi2008_12_41443_hit21
5' UGGCGAUUUCAGCUCUUCU
   |:|||||:|||||||||||
   AUCGCUAGAGUCGAGAAGA 5'
AT4G30190.1     2869    2887
H+-transporting ATPase type 2, plasma membrane

sRNA_AGO1_Solexa_Mi2008_2_13419_hit2
5' GAAGAAGAAGAAGACACUU
   ||||||||||||||||
   CUUCUUCUUCUUCUGUUUU 5'
AT4G30190.1     3021    3039
H+-transporting ATPase type 2, plasma membrane

sRNA_AGO1_Solexa_Mi2008_7_353_hit2
5' AAAGAAGAAGAUAAAGCAU
   |||||||||||| ||| ||
   UUUCUUCUUCUAAUUC-UA 5'
AT4G30190.1     3256    3273
H+-transporting ATPase type 2, plasma membrane

sRNA_AGO1_Solexa_Mi2008_1_173_hit2
5' AAAAGAAGAAGAUAAAGCAU
   ||||||||||||| ||| ||
   UUUUCUUCUUCUAAUUC-UA 5'
AT4G30190.1     3256    3274
H+-transporting ATPase type 2, plasma membrane

sRNA_AGO1_Solexa_Mi2008_1_16281_hit2
5' UAAAAAGAAGAAGAUAAAGCAU
   :|||||||||||||| ||| ||
   GUUUUUCUUCUUCUAAUUC-UA 5'
AT4G30190.1     3256    3276
H+-transporting ATPase type 2, plasma membrane

sRNA_AGO1_Solexa_Mi2008_3_53531_hit4
5' UUGUGAAUCA-GGUGUUUUAAU
   |:||||| || |||||||||||
   AGCACUUCGUUCCACAAAAUUA 5'
AT4G30210.2     1447    1468
NADPH-ferrihemoprotein reductase (ATR2)

sRNA_AGO1_Solexa_Mi2008_1_45260_hit2
5' UGUGGACC-AAAAUAGAUGGUA
   |||| ||| |||| ||||||||
   ACACAUGGCUUUU-UCUACCAU 5'
AT4G30210.2     451     471
NADPH-ferrihemoprotein reductase (ATR2)

sRNA_AGO1_Solexa_Mi2008_1_13420_hit2
```

5' GAAG-AAGAAGAAGA-CUCUU
   |||| |||||||||| ||||:
   CUUCCUUCUUCUUCUAGAGAG 5'
AT4G30210.2   61   81
NADPH-ferrihemoprotein reductase (ATR2)

sRNA_AGO1_Solexa_Mi2008_3_11393_hit1
5' CUGA-A-GUGUGCUGUUGUUGCU
   |||| | ||||| ||||||||||
   GACUCUGCACACAACAACAACGA 5'
AT4G30250.1   253   275
putative protein

sRNA_AGO1_Solexa_Mi2008_3_1034_hit1
5' AAUCAAGGCAAGAAUCUUC
   |||||||| ||||||||
   UUAGUUCCAUUCUUAGAUC 5'
AT4G30250.1   477   495
putative protein

sRNA_AGO1_Solexa_Mi2008_3_12806_hit1
5' CUUGAUAUC-CAGAUGUAUAUU
    |||||||| ||||||||||||
   UAACUAUAGAGUCUACAUAUAA 5'
AT4G30290.1   7   28
xyloglucan endo-1,4-beta-D-glucanase-like protein

sRNA_AGO1_Solexa_Mi2008_1_53875_hit1
5' UUUAAACAGGUAAAGAAAAACAG
   ||||| |||||||| ||||| ||
   AAAUUAGUCCAUUU-UUUUU-UC 5'
AT4G30350.1   113   133
unknown protein

sRNA_AGO1_Solexa_Mi2008_2_116_hit3
5' AAA-AAGAUAAGAGAGAUAG
    || |||||||||||| |||
   GUUCUUCUAUUCUCUC-AUC 5'
AT4G30350.1   416   434
unknown protein

sRNA_AGO1_Solexa_Mi2008_2_13419_hit2
5' GAAGAAGAAGAAGAC-ACUU
   ||||||||||||||| ||
   CUUCUUCUUCUUCUGCUGCU 5'
AT4G30350.1   571   590
unknown protein

sRNA_AGO1_Solexa_Mi2008_1_1549_hit1
5' ACCGGAGAAGAAGAAGAUGAC
     ||||||||||||||| |||
   CUGCCUCUUCUUCUUCUUCUG 5'
AT4G30350.1   575   595
unknown protein

sRNA_AGO1_Solexa_Mi2008_3_13542_hit2
5' GAAGUUUUGAAGAUUUGCAGA
   |||| |||||||||||| |||
   CUUCUAAACUUCUAAACCUCU 5'
AT4G30430.1   25   45
senescence-associated protein homolog

sRNA_AGO1_Solexa_Mi2008_2_43655_hit2

```
5' UGGUGUGAAUUGCAGAAUCCCG
   |||| |||||||||||| |||
   ACCA-ACUUAACGUCUUUGGGA 5'
AT4G30800.1     63      83

sRNA_AGO1_Solexa_Mi2008_10_43654_hit2
5' UGGUGUGAAUUGCAGAAUCCC
   |||| |||||||||||| |||
   ACCA-ACUUAACGUCUUUGGG 5'
AT4G30800.1     64      83

sRNA_AGO1_Solexa_Mi2008_1_47319_hit2
5' UUAGACAAAAAAAAAGAUAUG
   ||||| ||||||||||||||:
   AAUCU-UUUUUUUUUCUAUAU 5'
AT4G31240.1     86      105
predicted protein

sRNA_AGO1_Solexa_Mi2008_1_21944_hit1
5' UAGAUUGGGAGGAUAAGCU
   ||| ||||||||||||| |
   AUC-AACCCUCCUAUUCUA 5'
AT4G31420.2     75      92
unknown protein with zinc finger

sRNA_AGO1_Solexa_Mi2008_1_21944_hit1
5' UAGAUUGGGAGGAUAAGCU
   ||| ||||||||||||| |
   AUC-AACCCUCCUAUUCUA 5'
AT4G31420.1     78      95
unknown protein with zinc finger

sRNA_AGO1_Solexa_Mi2008_3_20883_hit1
5' UAGAAGAAUGGUGAUGUACGUG
   ||||||||||| |||| || ||
   AUCUUCUUACCCCUAC-UG-AC 5'
AT4G31500.1     1124    1143
cytochrome P450 monooxygenase (CYP83B1)

sRNA_AGO1_Solexa_Mi2008_1_32802_hit1
5' UCUACCAGCAGAAACGUCCUA
   |||||||||||||||||||||
   AGAUGGUCGUCUUUGCAGGAU 5'
AT4G31500.1     1554    1574
cytochrome P450 monooxygenase (CYP83B1)

sRNA_AGO1_Solexa_Mi2008_1_27836_hit1
5' UCA-CGGUGUCUGAUUGAUCG
   ||| |||||:||||||||||:
   AGUUGCCACGGACUAACUAGU 5'
AT4G31500.1     522     542
cytochrome P450 monooxygenase (CYP83B1)

sRNA_AGO1_Solexa_Mi2008_9_14254_hit8
5' GAUGAUGAUGAUGAUGAUCUU
   |||||||||||||||||||||
   CUACUACUACUACUACUAGAA 5'
AT4G31800.2     474     494

sRNA_AGO1_Solexa_Mi2008_1_36323_hit13
5' UGAGGAUGAUGAUGAUGAUGA
   ||| ||||||||||||||
   ACUACUACUACUACUACUAGA 5'
```

```
AT4G31800.2     475     495

sRNA_AGO1_Solexa_Mi2008_1_3079_hit472
5' AUGAUGAUGAUGAUGAUGA
    |||||||||||||||||||
   GACUACUACUACUACUACUAGA 5'
AT4G31800.2     475     496

sRNA_AGO1_Solexa_Mi2008_1_45039_hit3
5' UG-UGAUGAUGAUGAUGAUGA
   || |||||||||||||||||||
   ACGACUACUACUACUACUACUAGA 5'
AT4G31800.2     476     499

sRNA_AGO1_Solexa_Mi2008_9_14254_hit8
5' GAUGAUGAUGAUGAUGAUCUU
   |||||||||||||||||||||
   CUACUACUACUACUACUAGAA 5'
AT4G31800.1     477     497

sRNA_AGO1_Solexa_Mi2008_1_38921_hit1
5' UGCGGACGAUGAUGAUGAUGAU
   ||| || |||||||||||||||
   ACGACUACUACUACUACUACUA 5'
AT4G31800.2     477     498

sRNA_AGO1_Solexa_Mi2008_7_3078_hit1
5' AUGAUGAUGA-GAAUGAUGAU
    ||||||||| || |||||||
   CACUACUACUACU-ACUACUA 5'
AT4G31800.2     478     497

sRNA_AGO1_Solexa_Mi2008_1_36323_hit13
5' UGAGGAUGAUGAUGAUGAUGA
   ||| |||||||||||||||
   ACUACUACUACUACUACUAGA 5'
AT4G31800.1     478     498

sRNA_AGO1_Solexa_Mi2008_1_3079_hit472
5' AUGAUGAUGAUGAUGAUGA
    |||||||||||||||||||
   GACUACUACUACUACUACUAGA 5'
AT4G31800.1     478     499

sRNA_AGO1_Solexa_Mi2008_3_39351_hit1
5' UGCUGAUGAUGCUGAUG-UGAC
   ||||||||||| ||||| |||
   ACGACUACUACUACUACUACUA 5'
AT4G31800.2     478     499

sRNA_AGO1_Solexa_Mi2008_1_56092_hit3
5' UUUG-UGAUGAUGAUGAUGAUGA
   :||| ||||||||||||||||||
   GAACGACUACUACUACUACUACU 5'
AT4G31800.2     479     501

sRNA_AGO1_Solexa_Mi2008_1_45039_hit3
5' UGU-GAUGAUGAUGAUGAUGAUGA
   ||| | ||||||||||||||||||
   ACAACGACUACUACUACUACUACU 5'
AT4G31800.2     479     502

sRNA_AGO1_Solexa_Mi2008_1_38921_hit1
```

```
5' UGCGGACGAUGAUGAUGAUGAU
   ||| || |||||||||||||||
   ACGACUACUACUACUACUACUA 5'
AT4G31800.1     480     501

sRNA_AGO1_Solexa_Mi2008_6_14255_hit369
5' GAUGAUGAUGAUGAUGAUGAUGAU
    ||| || ||||||||||||||||
   GUACAACGACUACUACUACUACUA 5'
AT4G31800.2     480     503

sRNA_AGO1_Solexa_Mi2008_7_3078_hit1
5' AUGAUGAUGA-GAAUGAUGAU
    ||||||||| || |||||||
   CACUACUACUACU-ACUACUA 5'
AT4G31800.1     481     500

sRNA_AGO1_Solexa_Mi2008_2_31619_hit1
5' UCGCUGAUGAUGAUUGAUGAU
     |||||||||||| ||||||
   UUCGACUACUACUA-CUACUA 5'
AT4G31800.2     481     500

sRNA_AGO1_Solexa_Mi2008_1_3079_hit472
5' AUGAUGAUGAUGAUGAUGAUGA
   ||| || |||||||||||||||
   UACAACGACUACUACUACUACU 5'
AT4G31800.2     481     502

sRNA_AGO1_Solexa_Mi2008_3_39351_hit1
5' UGCUGAUGAUGCUGAUG-UGAC
   ||||||||||| ||||| |||
   ACGACUACUACUACUACUACUA 5'
AT4G31800.1     481     502

sRNA_AGO1_Solexa_Mi2008_1_56092_hit3
5' UUUG-UGAUGAUGAUGAUGAUGA
   :||| ||||||||||||||||||
   GAACGACUACUACUACUACUACU 5'
AT4G31800.1     482     504

sRNA_AGO1_Solexa_Mi2008_1_45039_hit3
5' UGU-GAUGAUGAUGAUGAUGAUGA
   ||| | ||||||||||||||||||
   ACAACGACUACUACUACUACUACU 5'
AT4G31800.1     482     505

sRNA_AGO1_Solexa_Mi2008_6_14255_hit369
5' GAUGAUGAUGAUGAUGAUGAUGAU
    ||| || ||||||||||||||||
   GUACAACGACUACUACUACUACUA 5'
AT4G31800.1     483     506

sRNA_AGO1_Solexa_Mi2008_2_31619_hit1
5' UCGCUGAUGAUGAUUGAUGAU
     ||||||||||| |||||||
   UUCGACUACUACU-ACUACUA 5'
AT4G31800.1     484     503

sRNA_AGO1_Solexa_Mi2008_1_3079_hit472
5' AUGAUGAUGAUGAUGAUGAUGA
   ||| || |||||||||||||||
   UACAACGACUACUACUACUACU 5'
```

```
AT4G31800.1     484     505

sRNA_AGO1_Solexa_Mi2008_1_35777_hit6
5' UGAGAACAAUGAAGAAGUCGCU
    ||||| |||||||||||||:
   UCUCUUUUUACUUCUUCAGCGG 5'
AT4G32285.2     117     138
unknown protein

sRNA_AGO1_Solexa_Mi2008_1_28035_hit2
5' UCAGAAGAGAAAGACUACCCU
   ||||||||||||| || |||
   AGUCUUCUCUUUC-GAAGGGU 5'
AT4G32285.1     1566    1585
unknown protein

sRNA_AGO1_Solexa_Mi2008_15_11643_hit2
5' CUG-AUUCUGCCAAGCCCGU
   ||| ||||||||||||| :|
   GACCUAAGACGGUUCGGCUA 5'
AT4G32285.1     1598    1617
unknown protein

sRNA_AGO1_Solexa_Mi2008_1_28035_hit2
5' UCAGAAGAGAAAGACUACCCU
   ||||||||||||| || |||
   AGUCUUCUCUUUC-GAAGGGU 5'
AT4G32285.2     1668    1687
unknown protein

sRNA_AGO1_Solexa_Mi2008_15_11643_hit2
5' CUG-AUUCUGCCAAGCCCGU
   ||| ||||||||||||| :|
   GACCUAAGACGGUUCGGCUA 5'
AT4G32285.2     1700    1719
unknown protein

sRNA_AGO1_Solexa_Mi2008_2_8711_hit62
5' CGAUG-ACAAGCUUCAGAGCCUU
   ||||| |||||| | ||||||||
   GCUACCUGUUCGUAUUCUCGGAA 5'
AT4G32285.1     480     502
unknown protein

sRNA_AGO1_Solexa_Mi2008_2_8711_hit62
5' CGAUG-ACAAGCUUCAGAGCCUU
   ||||| |||||| | ||||||||
   GCUACCUGUUCGUAUUCUCGGAA 5'
AT4G32285.2     582     604
unknown protein

sRNA_AGO1_Solexa_Mi2008_1_2_hit38
5' AAA-AAAAAAAAAAAACCAU
   ||| |||||||||||| |||
   UUUAUUUUUUUUUUUUCGUA 5'
AT4G32850.6     2921    2940
poly(A) polymerase like protein

sRNA_AGO1_Solexa_Mi2008_1_2_hit38
5' AAA-AAAAAAAAAAAACCAU
   ||| |||||||||||| |||
   UUUAUUUUUUUUUUUUCGUA 5'
AT4G32850.5     2934    2953
```

```
poly(A) polymerase like protein

sRNA_AGO1_Solexa_Mi2008_1_2_hit38
5' AAA-AAAAAAAAAAAACCAU
   ||| |||||||||||| |||
   UUUAUUUUUUUUUUUUCGUA 5'
AT4G32850.7     3124    3143
poly(A) polymerase like protein

sRNA_AGO1_Solexa_Mi2008_1_2_hit38
5' AAA-AAAAAAAAAAAACCAU
   ||| |||||||||||| |||
   UUUAUUUUUUUUUUUUCGUA 5'
AT4G32850.4     3430    3449
poly(A) polymerase like protein

sRNA_AGO1_Solexa_Mi2008_3_35894_hit1
5' UGAGA-GCAGAGAAAGAGAGU
   ||||| | ||||||||||||
   ACUCUUC-UCUCUUUCUCUCU 5'
AT4G32850.9     66      85
poly(A) polymerase like protein

sRNA_AGO1_Solexa_Mi2008_2_411_hit5
5' AAAGAUGAAGAGAGAAAGAGA
     ||| |||||||||||||||
   AGUCU-CUUCUCUCUUUCUCU 5'
AT4G32850.9     68      87
poly(A) polymerase like protein

sRNA_AGO1_Solexa_Mi2008_3_35894_hit1
5' UGAGA-GCAGAGAAAGAGAGU
   ||||| | ||||||||||||
   ACUCUUC-UCUCUUUCUCUCU 5'
AT4G32850.8     74      93
poly(A) polymerase like protein

sRNA_AGO1_Solexa_Mi2008_2_411_hit5
5' AAAGAUGAAGAGAGAAAGAGA
     ||| |||||||||||||||
   AGUCU-CUUCUCUCUUUCUCU 5'
AT4G32850.8     76      95
poly(A) polymerase like protein

sRNA_AGO1_Solexa_Mi2008_1_21387_hit3
5' UAGAGGAUACAA-ACGGGAUGU
   |||||||||||| || ||| ||
   AUCUCCUAUGUUGUGGCCU-CA 5'
AT4G32910.1     172     192

sRNA_AGO1_Solexa_Mi2008_3_13421_hit1
5' GAAGAAGAAGAUGAUG-UUGAU
   ||||||||||||:||| || ||
   CUUCUUCUUCUAUUACGAA-UA 5'
AT4G32980.1     564     584
homeobox gene ATH1

sRNA_AGO1_Solexa_Mi2008_1_17946_hit13
5' UAAGAUUUCAGACAACACAAU
    ||||||||||||||||||:
   CUUCUAAAGUCUGUUGUGUUG 5'
AT4G32980.1     635     655
homeobox gene ATH1
```

```
sRNA_AGO1_Solexa_Mi2008_1_25259_hit4
5' UAU-GAAGUGCAAGUUGAUGGA
   ||| ||||||||||||| |||
   AUAUCUUCACGUUCAAC-ACCG 5'
AT4G33050.4     808     828
unknown protein

sRNA_AGO1_Solexa_Mi2008_1_25259_hit4
5' UAU-GAAGUGCAAGUUGAUGGA
   ||| ||||||||||||| |||
   AUAUCUUCACGUUCAAC-ACCG 5'
AT4G33050.2     922     942
unknown protein

sRNA_AGO1_Solexa_Mi2008_7_35034_hit1
5' UGAAUGCAGUUUCAGGAAU-AUC
   ||||||||||||||:| || |||
   ACUUACGUCAAAGUUC-UACUAG 5'
AT4G33210.1     1912    1933
unknown protein

sRNA_AGO1_Solexa_Mi2008_1_22977_hit1
5' UAGGCUCCAAUGAUGAUGAUC
   :||||||||||||||| | ||
   GUCCGAGGUUACUACU-C-AG 5'
AT4G33210.1     2335    2353
unknown protein

sRNA_AGO1_Solexa_Mi2008_4_4778_hit3
5' CAAGCUGGAGAUUGUGAUAU
   ||||| |||||||||||||:
   GUUCG-CCUCUAACACUAUG 5'
AT4G33300.2     1099    1117
putative protein

sRNA_AGO1_Solexa_Mi2008_1_27163_hit3
5' UCAAGCUGGAGAUUGUGAUA
   |||||| |||||||||||||
   AGUUCG-CCUCUAACACUAU 5'
AT4G33300.2     1100    1118
putative protein

sRNA_AGO1_Solexa_Mi2008_1_55692_hit1
5' UUUGGCGGUGG-GGCUGGUGGU
   |:||||||||| || |||||||
   AGACCGCCACCACC-ACCACCA 5'
AT4G33300.2     113     133
putative protein

sRNA_AGO1_Solexa_Mi2008_3_13895_hit1
5' GAGAAACCACCGAUGGA-GAUGGU
   |||||||||| |||||| || |||
   CUCUUUGGUGUCUACCUACUUCCA 5'
AT4G34140.1     629     652
hypothetical protein

sRNA_AGO1_Solexa_Mi2008_2_13419_hit2
5' GAAGAAGAAGAAGACACUU
   ||||||||||||||||
   CUUCUUCUUCUUCUGUUCC 5'
AT4G34160.1     385     403
cyclin delta-3
```

```
sRNA_AGO1_Solexa_Mi2008_1_46328_hit1
5' UUAAAUGGUGAUCAACGAUAACUU
   ||||||| ||||||||| || |||
   AAUUUACAACUAGUUGC-AU-GAA 5'
AT4G34610.2     1156    1177
Homeodomain - like protein

sRNA_AGO1_Solexa_Mi2008_1_16210_hit1
5' GU-UUAAAUGGUGAUCAACGAUA
   || ||||||| ||||||||| ||
   CAGAAUUUACAACUAGUUGC-AU 5'
AT4G34610.2     1159    1180
Homeodomain - like protein

sRNA_AGO1_Solexa_Mi2008_1_46328_hit1
5' UUAAAUGGUGAUCAACGAUAACUU
   ||||||| ||||||||| | ||||
   AAUUUACAACUAGUUGC-A-UGAA 5'
AT4G34610.1     1213    1234
Homeodomain - like protein

sRNA_AGO1_Solexa_Mi2008_1_16210_hit1
5' GU-UUAAAUGGUGAUCAACGAUA
   || ||||||| ||||||||| ||
   CAGAAUUUACAACUAGUUGC-AU 5'
AT4G34610.1     1216    1237
Homeodomain - like protein

sRNA_AGO1_Solexa_Mi2008_1_44135_hit7
5' UGUAGAGGCUGAGAAAUUGUA
   ||| |||||||||| |||||
   ACA-CUCCGACUCUGUAACAA 5'
AT4G34610.2     376     395
Homeodomain - like protein

sRNA_AGO1_Solexa_Mi2008_1_44135_hit7
5' UGUAGAGGCUGAGAAAUUGUA
   ||| |||||||||| |||||
   ACA-CUCCGACUCUGUAACAA 5'
AT4G34610.1     433     452
Homeodomain - like protein

sRNA_AGO1_Solexa_Mi2008_2_46553_hit2
5' UUAAGCCAUGCAUGUG-UAA
   |||||||||||||:|| ||
   AAUUCGGUACGUAUACUAUA 5'
AT4G34860.1     58      77
invertase - like protein

sRNA_AGO1_Solexa_Mi2008_1_46017_hit2
5' UGUUGGGACCAGAGUUAGCCA
   ||||:||||||||||||||||
   ACAAUCCUGGUCUCAAUCGGU 5'
AT4G34870.1     443     463
cyclophilin (AtCYP18-4)

sRNA_AGO1_Solexa_Mi2008_1_27051_hit1
5' UCAAGAAU-ACAGAAGAAGGA
   |||||||| | ||||||||||
   AGUUCUUACU-UCUUCUUCCU 5'
AT4G34950.1     28      47
unknown protein
```

```
sRNA_AGO1_Solexa_Mi2008_1_34612_hit1
5' UGAAGAUGAAGAUGAA-AUGUAU
   |||||||||||| ||| |||||:
   ACUUCUACUUCUUCUUCUACAUG 5'
AT4G34950.1     70      92
unknown protein

sRNA_AGO1_Solexa_Mi2008_2_13457_hit1
5' GAAGAUGAAGAUGAAG-UGGUA
   ||||||||||| |||| | |||
   CUUCUACUUCUUCUUCUA-CAU 5'
AT4G34950.1     71      91
unknown protein

sRNA_AGO1_Solexa_Mi2008_1_38854_hit6
5' UGCGAUUGAUG-AAGCUUCUGU
   ||||||||| | |||||:||||
   ACGCUAACU-CUUUCGAGGACA 5'
AT4G34950.1     774     794
unknown protein

sRNA_AGO1_Solexa_Mi2008_1_7010_hit1
5' CAUCAUGAACUUUGC-A-GGCU
   ||||||||||||||| | || |
   GUAGUACUUGAAACGAUGCCUA 5'
AT4G34950.1     855     876
unknown protein

sRNA_AGO1_Solexa_Mi2008_1_34418_hit4
5' UGAA-CCUCAGGCGACCAAG
   |||| |||| ||||||||||
   ACUUCGGAGGCCGCUGGUUC 5'
AT4G34970.1     352     371
actin depolymerizing factor - like protein

sRNA_AGO1_Solexa_Mi2008_29_22294_hit1
5' UAGCCUUAUGCACACUCUGUU
   ||||||||||||| | ||||
   AUCGGAAUACGUG-GCGACAU 5'
AT4G35260.1     680     699
NAD+ dependent isocitrate dehydrogenase subunit 1

sRNA_AGO1_Solexa_Mi2008_6_4720_hit1
5' CAAGCAUCACAGGAGUAAUA
   ||||||||||||||||||||
   GUUCGUAGUGUCCUCAUUAU 5'
AT4G35630.1     1006    1025
phosphoserine aminotransferase

sRNA_AGO1_Solexa_Mi2008_2_1458_hit7
5' ACAUAAGUGAUAUGAUGCGGU
   ||||||||:|||||||| |||
   UGUAUUCAUUAUACUACUCCA 5'
AT4G36020.1     1212    1232
glycine-rich protein

sRNA_AGO1_Solexa_Mi2008_3_330_hit1
5' AAA-CU-CAGAAGAACAUUCU
   ||| || |||||||||||||:
   UUUCGAUGUCUUCUUGUAAGG 5'
AT4G36020.1     933     953
glycine-rich protein
```

```
sRNA_AGO1_Solexa_Mi2008_1_44974_hit2
5' UGUGAGCGAUAACAAUGGCGU
   ||||||||||||||||||||
   ACACUCGCUAUUGUUACCGCA 5'
AT4G36130.1     445     465
putative ribosomal protein L8

sRNA_AGO1_Solexa_Mi2008_2_4548_hit7
5' CAAGA-AAUUUUAAAAGGGUUU
    |||| |||||||||||||||
   AUUCUCUUAAAAUUUUCCCAAA 5'
AT4G36380.1     8       29
cytochrome P450 (ROTUNDIFOLIA3)

sRNA_AGO1_Solexa_Mi2008_1_4625_hit1
5' CAAGAGA-UUUUAAAUGGGUUU
    |||||| ||||||| ||||||
   AUUCUCUUAAAAUUUUCCCAAA 5'
AT4G36380.1     8       29
cytochrome P450 (ROTUNDIFOLIA3)

sRNA_AGO1_Solexa_Mi2008_3_4543_hit1
5' CAAGAAAGAAUUUGCAUCU
   |||||||||| |||||| |
   GUUCUUUCUU-AACGUAAA 5'
AT4G36880.1     763     780
cysteine proteinase

sRNA_AGO1_Solexa_Mi2008_1_41920_hit17
5' UGGGAUGAUGAAGUUGAUGAU
    :||||||||||| |||||||
   CUCCUACUACUUCUACUACUA 5'
AT4G36930.1     146     166
putative bHLH transcription factor (AtbHLH024) / SPATULA (SPT)

sRNA_AGO1_Solexa_Mi2008_1_36323_hit13
5' UGAGGAUGAUGAUGAUGAUGA
    ||||||||||| ||||||||
   CCUCCUACUACUUCUACUACU 5'
AT4G36930.1     147     167
putative bHLH transcription factor (AtbHLH024) / SPATULA (SPT)

sRNA_AGO1_Solexa_Mi2008_3_40895_hit1
5' UGGAUGAUGAGAGA-GAGAU
   ||||||||||||:| ||||:
   ACCUACUACUCUUUGCUCUG 5'
AT4G36930.1     321     340
putative bHLH transcription factor (AtbHLH024) / SPATULA (SPT)

sRNA_AGO1_Solexa_Mi2008_3_4568_hit12
5' CAAGAAGAAGAUGAGAA-CAAUG
   |||||||||||||| || ||||
   GUUCUUCUUCUACU-UUAGUUAA 5'
AT4G36930.1     99      120
putative bHLH transcription factor (AtbHLH024) / SPATULA (SPT)

sRNA_AGO1_Solexa_Mi2008_1_36137_hit2
5' UGAGCGAGACCAACUUUCACG
   :||||||||||| |||| |||
   GCUCGCUCUGGUCGAAA-UGC 5'
AT4G37120.1     102     121
step II splicing factor like protein
```

```
sRNA_AGO1_Solexa_Mi2008_2_56594_hit1
5' UUUUGG-UUCGGGUCCAAUC
   |||||| ||||||| ||||
   AAAACCCAAGCCCAAGUUAA 5'
AT4G37120.1     239     258
step II splicing factor like protein

sRNA_AGO1_Solexa_Mi2008_7_25146_hit3
5' UAUCUAUUCGGGUUCGGGUUC
   ||||| || |||||||||||
   AUAGAAAAACCCAAGCCCAAG 5'
AT4G37120.1     242     262
step II splicing factor like protein

sRNA_AGO1_Solexa_Mi2008_23_6630_hit1
5' CAGGUG-GAAGACAAGAUCGA
   | |||| |||||||||||||
   GGCCACUCUUCUGUUCUAGCC 5'
AT4G37390.1     348     368
auxin-responsive GH3 homolog (CF4)

sRNA_AGO1_Solexa_Mi2008_6_14255_hit369
5' GAUGAUGAUGAUGAUGAUGAUGAU
   |||||||||| ||||| || ||||
   CUACUACUACAACUACAAC-ACUA 5'
AT4G37590.1     1042    1064
unknown protein

sRNA_AGO1_Solexa_Mi2008_1_25400_hit1
5' UAUGAUGAUGAAGUUGAUGUU
   | ||||||||| |||||||||
   AAACUACUACUACAACUACAA 5'
AT4G37590.1     1046    1066
unknown protein

sRNA_AGO1_Solexa_Mi2008_9_14254_hit8
5' GAUGAUGAUGAUGAUGA-UCUU
   ||||||||||||||||| |||
   CUACUACUACUACUACUUAGAC 5'
AT4G37590.1     1078    1099
unknown protein

sRNA_AGO1_Solexa_Mi2008_7_11649_hit2
5' CUGAUUGAUGAUGAUGGAUCU
    |||| ||||||||||:||||
   AACUA-CUACUACUACUUAGA 5'
AT4G37590.1     1079    1098
unknown protein

sRNA_AGO1_Solexa_Mi2008_1_51445_hit1
5' UUGCAUGAUGAUGAUUGAUGU
   || |||||||||||| ||||
   AA-GUACUACUACUA-CUACU 5'
AT4G37590.1     1083    1101
unknown protein

sRNA_AGO1_Solexa_Mi2008_2_4250_hit1
5' CAAAUGAAGCAGGACCACCAG
   |||||||||||||||||||||
   GUUUACUUCGUCCUGGUGGUC 5'
AT4G37590.1     1126    1146
unknown protein
```

```
sRNA_AGO1_Solexa_Mi2008_1_351_hit2
5' AAAGAAGAAAAACAGAUCU
   |||||||||||| ||| ||
   UUUCUUCUUUUU-UCU-GA 5'
AT4G37590.1     819     835
unknown protein

sRNA_AGO1_Solexa_Mi2008_4_4395_hit1
5' CAACAUGUUUGAGGUGGAACAA
   ||||| ||| |||||||||||
   GUUGUUCAA-CUCCACCUUGUC 5'
AT4G37750.1     426     446
ovule development protein aintegumenta (ANT)

sRNA_AGO1_Solexa_Mi2008_7_32730_hit2
5' UCUAAG-GGCAUCACAGACCUGU
   ||| || |||||||||||| |||
   AGACUCACCGUAGUGUCUGUACA 5'
AT4G37750.1     513     535
ovule development protein aintegumenta (ANT)

sRNA_AGO1_Solexa_Mi2008_1_7065_hit2
5' CAUCUAAG-GGCAUCACAGAC
   ||||| || ||||||||||||
   GUAGACUCACCGUAGUGUCUG 5'
AT4G37750.1     517     537
ovule development protein aintegumenta (ANT)

sRNA_AGO1_Solexa_Mi2008_19_1471_hit2
5' ACAUCUAAG-GGCAUCACAGAC
    ||||| || ||||||||||||
   AGUAGACUCACCGUAGUGUCUG 5'
AT4G37750.1     517     538
ovule development protein aintegumenta (ANT)

sRNA_AGO1_Solexa_Mi2008_12_19173_hit1
5' UACAAGGUGUUGU-GAAGUCUA
   ||||||||||||| :||||||
   AUGUUCCACAACAGUUUCAGAA 5'
AT4G37910.1     902     923
heat shock protein 70 like protein

sRNA_AGO1_Solexa_Mi2008_1_20804_hit2
5' UAGAAAUGCACCGAAAUACUU
   :|||||||||||||| || ||
   GUCUUUACGUGGCUUGAU-AA 5'
AT4G38200.1     3393    3412
guanine nucleotide-exchange protein -like

sRNA_AGO1_Solexa_Mi2008_1_45228_hit1
5' UGUGCUGGAGCAAUUUC-AUG
   ||| ||||||||||||| ||:
   ACA-GACCUCGUUAAAGGUAU 5'
AT4G38240.2     749     768
glycosyltransferase like protein

sRNA_AGO1_Solexa_Mi2008_1_172_hit2
5' AA-AAGAAGAAGAUAAAGCA
   || |||||||||||||||
   UUCUUCUUCUUCUAUUUCAA 5'
AT4G38420.1     32      51
pectinesterase like protein
```

```
sRNA_AGO1_Solexa_Mi2008_3_10033_hit2
5' CUACUGAUGCC-CGCGUCGC
   |||||||| || ||||||||
   GAUGACUA-GGUGCGCAGCG 5'
AT4G38440.1     4120    4138
unknown protein

sRNA_AGO1_Solexa_Mi2008_1_36855_hit2
5' UGAGUGUUGUGGUCAAAUGGG
     ||||||||||||| |||||
   UGUCACAACACCAGUGUACCC 5'
AT4G38540.1     692     712
monooxygenase 2 (MO2)

sRNA_AGO1_Solexa_Mi2008_1_20879_hit2
5' UAGAAGAAGGAGAUGCGAUGAC
   |||||||||:||||  ||||||
   AUCUUCUUCUUCUA--CUACUG 5'
AT4G38840.1     152     171
auxin-induced protein - like

sRNA_AGO1_Solexa_Mi2008_1_2_hit38
5' AAAAAAAAAAAAAAACCAU
    | |||||||||||||||
   AUCUUUUUUUUUUUUGGUA 5'
AT4G38860.1     622     640
putative auxin-induced protein

sRNA_AGO1_Solexa_Mi2008_1_12944_hit4
5' CUUGGGAGAGAGUA--GUACUAG
   ||||||||||||||  ||| |||
   GAACCCUCUCUCAUCCCAUUAUC 5'
AT4G38940.1     278     300
unknown protein

sRNA_AGO1_Solexa_Mi2008_1_12954_hit4
5' CUUGGGCGAGAGUA--GUAAUAG
   |||||| |||||||  |||||||
   GAACCCUCUCUCAUCCCAUUAUC 5'
AT4G38940.1     278     300
unknown protein

sRNA_AGO1_Solexa_Mi2008_2_53577_hit1
5' UUGUGCAUACGAAGAAUGGAU
    ||||||| ||||||| ||||
   CACACGUAGGCUUCUU-CCUA 5'
AT4G38940.1     353     372
unknown protein

sRNA_AGO1_Solexa_Mi2008_3_17219_hit1
5' UAACAGAAUUCCAGAUGAGAG
   :|||||||||||| ||||||
   GUUGUCUUAAGGUGUACUCUG 5'
AT4G39390.2     521     541
unknown protein

sRNA_AGO1_Solexa_Mi2008_3_17219_hit1
5' UAACAGAAUUCCAGAUGAGAG
   :|||||||||||| ||||||
   GUUGUCUUAAGGUGUACUCUG 5'
AT4G39390.1     599     619
unknown protein
```

```
sRNA_AGO1_Solexa_Mi2008_1_50104_hit2
5' UUGA-AGGUCGCGAUUCUUCCC
   |||| |||||||||| |||||
   AACUAUCCAGCGCUACGAAGGU 5'
AT4G39670.1     617     638
unknown protein

sRNA_AGO1_Solexa_Mi2008_4_3653_hit2
5' AUUGA-AGGUCGCGAUUCUUCC
   ||||| |||||||||| |||||
   UAACUAUCCAGCGCUACGAAGG 5'
AT4G39670.1     618     639
unknown protein

sRNA_AGO1_Solexa_Mi2008_12_12075_hit1
5' CUGG-UCGAGGAAUCCUGGA
    ||| || ||||||||||||
   UACCUAG-UCCUUAGGACCU 5'
AT4G39980.1     725     743
2-dehydro-3-deoxyphosphoheptonate aldolase

sRNA_AGO1_Solexa_Mi2008_1_38872_hit1
5' UGCGCCACCACGUUCCUGUGU
   |||||||||||||||||||||
   ACGCGGUGGUGCAAGGACACA 5'
AT5G01210.1     1162    1182
anthranilate N-benzoyltransferase - like protein

sRNA_AGO1_Solexa_Mi2008_1_34189_hit1
5' UGAAA-CUCCCAAGUACUGUCA
   ||||| ||||||||||||||
   ACUUUUGAGGGUUCAUGACACG 5'
AT5G01540.1     524     545
receptor like protein kinase

sRNA_AGO1_Solexa_Mi2008_1_52782_hit1
5' UUGGGUGAAGUAGUCGUCAUU
   |||| ||||| |||||||||:
   AACC-ACUUCUUCAGCAGUAG 5'
AT5G01930.1     551     570
(1-4)-beta-mannan endohydrolase-like protein

sRNA_AGO1_Solexa_Mi2008_14_41633_hit1
5' UGGCUGCGGCAGUGGUGCUUCG
   |||||||:||||||| ||| ||
   ACCGACGUCGUCACC-CGA-GC 5'
AT5G02490.1     616     635
dnaK-type molecular chaperone hsc70.1 - like

sRNA_AGO1_Solexa_Mi2008_10_5525_hit1
5' CACCGGACGAUCUUCU-CGA
    ||| ||||||||||| |||
   AUGGACUGCUAGAAGAUGCU 5'
AT5G02780.2     107     126
putative protein

sRNA_AGO1_Solexa_Mi2008_1_13977_hit25
5' GAGAUA-AGCCAUGGAAAGGAU
   |||||| ||||||||| |||||
   CUCUAUCUCGGUACCUCUCCUA 5'
AT5G02780.2     73      94
putative protein
```

```
sRNA_AGO1_Solexa_Mi2008_1_14784_hit24
5' GCUGGAGCAGUCAUAGAUGUC
    ||||||||||||| ||:|||
   AGACCUCGUCAGUA-CUGCAG 5'
AT5G02870.2     138     157
60S ribosomal protein - like

sRNA_AGO1_Solexa_Mi2008_1_5053_hit1
5' CAAGUUCUUGAAAUCA-GAAGUA
   ||||||||||||| || :|||||
   GUUCAAGAACUUU-GUAUUUCAU 5'
AT5G02960.1     619     640
unknown protein

sRNA_AGO1_Solexa_Mi2008_3_35894_hit1
5' UGAGAGCAGAGAAAGAGAGU
    ||||| ||||||||||||
   CCUCUC-UCUCUUUCUCUCC 5'
AT5G03290.1     7       25
putative isocitrate dehydrogenase (NAD+) like protein

sRNA_AGO1_Solexa_Mi2008_2_6093_hit1
5' CAGAGGUUGAGGUUGACGUGC
    |||||||||||||||| ||
   UUCUCCAACUCCAACUGC-CG 5'
AT5G03350.1     85      104
protein kinase - like

sRNA_AGO1_Solexa_Mi2008_9_14254_hit8
5' GAUGAUGAUGAUGAUGAUC-UU
   ||||||||||||||||||| ||
   CUACUACUACUACUACUAGUAA 5'
AT5G03545.1     292     313
unknown protein

sRNA_AGO1_Solexa_Mi2008_6_14255_hit369
5' GAUGAUGAUGAUGAUGAUGAUGAU
   ||||||||||||||||||||| ||
   CUACUACUACUACUACUACUAGUA 5'
AT5G03545.1     292     315
unknown protein

sRNA_AGO1_Solexa_Mi2008_1_14256_hit8
5' GAUGAUGAUGAUGAUGAUGAUGUU
   |||||||||||||||||||||  |
   CUACUACUACUACUACUACUAGUA 5'
AT5G03545.1     292     315
unknown protein

sRNA_AGO1_Solexa_Mi2008_1_36323_hit13
5' UGAGGAUGAUGAUGAUGAUGA
   ||| ||||||||||||||| |
   ACUACUACUACUACUACUAGU 5'
AT5G03545.1     293     313
unknown protein

sRNA_AGO1_Solexa_Mi2008_1_3079_hit472
5' AUGAUGAUGAUGAUGAUGAUGA
   |||||||||||||||||||| |
   UACUACUACUACUACUACUAGU 5'
AT5G03545.1     293     314
unknown protein
```

```
sRNA_AGO1_Solexa_Mi2008_1_45039_hit3
5' UG-UGAUGAUGAUGAUGAUGA
   || ||||||||||||||||||| |
   ACUACUACUACUACUACUACUAGU 5'
AT5G03545.1     294     317
unknown protein

sRNA_AGO1_Solexa_Mi2008_9_14254_hit8
5' GAUGAUGAUGAUGAUGAUCUU
   ||||||||||||||||||  |
   CUACUACUACUACUACUACUA 5'
AT5G03545.1     295     315
unknown protein

sRNA_AGO1_Solexa_Mi2008_6_14255_hit369
5' GAUGAUGAUGAUGAUGAUGAUGAU
   :|||||||||||||||||||||||
   UUACUACUACUACUACUACUACUA 5'
AT5G03545.1     295     318
unknown protein

sRNA_AGO1_Solexa_Mi2008_1_14256_hit8
5' GAUGAUGAUGAUGAUGAUGAUGUU
   :||||||||||||||||||||| |
   UUACUACUACUACUACUACUACUA 5'
AT5G03545.1     295     318
unknown protein

sRNA_AGO1_Solexa_Mi2008_7_3078_hit1
5' AUGAUGAUGA-GAAUGAUGAU
   |||||||||| | ||||||||
   UACUACUACUAC-UACUACUA 5'
AT5G03545.1     296     315
unknown protein

sRNA_AGO1_Solexa_Mi2008_1_36323_hit13
5' UGAGGAUGAUGAUGAUGAUGA
   ||| |||||||||||||||||
   ACUACUACUACUACUACUACU 5'
AT5G03545.1     296     316
unknown protein

sRNA_AGO1_Solexa_Mi2008_1_3079_hit472
5' AUGAUGAUGAUGAUGAUGAUGA
   |||||||||||||||||||||
   UACUACUACUACUACUACUACU 5'
AT5G03545.1     296     317
unknown protein

sRNA_AGO1_Solexa_Mi2008_1_45039_hit3
5' UGUGAUGAUGAUGAUGAUGAUGA
     :||||||||||||||||||||
   UUACUACUACUACUACUACUACU 5'
AT5G03545.1     296     318
unknown protein

sRNA_AGO1_Solexa_Mi2008_1_56092_hit3
5' UUUG-UGAUGAUGAUGAUGAUGA
   |||| ||||||||||||||||||
   AAACUACUACUACUACUACUACU 5'
AT5G03545.1     297     319
unknown protein
```

```
sRNA_AGO1_Solexa_Mi2008_6_14255_hit369
5' GAUGAUGAUGAUGAUGAUGAUGAU
   |||:||||||||||||||||||||
   CUAUUACUACUACUACUACUACUA 5'
AT5G03545.1     298     321
unknown protein

sRNA_AGO1_Solexa_Mi2008_1_14256_hit8
5' GAUGAUGAUGAUGAUGAUGAUGUU
   |||:|||||||||||||||||| |
   CUAUUACUACUACUACUACUACUA 5'
AT5G03545.1     298     321
unknown protein

sRNA_AGO1_Solexa_Mi2008_7_3078_hit1
5' AUGAUGAUGA-GAAUGAUGAU
   |||||||||| || |||||||
   UACUACUACUACU-ACUACUA 5'
AT5G03545.1     299     318
unknown protein

sRNA_AGO1_Solexa_Mi2008_1_3079_hit472
5' AUGAUGAUGAUGAUGAUGAUGA
     |||:||||||||||||||||
   ACCUAUUACUACUACUACUACU 5'
AT5G03545.1     302     323
unknown protein

sRNA_AGO1_Solexa_Mi2008_4_14169_hit1
5' GAUAAUGAUGAUGAAAGAUGA
   ||||||||||||||  |||||
   CUAUUACUACUACUA-CUACU 5'
AT5G03545.1     303     322
unknown protein

sRNA_AGO1_Solexa_Mi2008_1_39699_hit1
5' UGGA-ACUGAUGAUGAUGAUGA
   |||| | |||||||||||||||
   ACCUAUUACUACUACUACUACU 5'
AT5G03545.1     303     324
unknown protein

sRNA_AGO1_Solexa_Mi2008_1_45039_hit3
5' UGUGAUGAUGAUGAUGAUGAUGA
   || |||:||||||||||||||||
   AC-CUAUUACUACUACUACUACU 5'
AT5G03545.1     303     324
unknown protein

sRNA_AGO1_Solexa_Mi2008_1_2280_hit1
5' AGUGGAUGAUGAUGAUGAUG
   | |||||:||||||||||||
   UAACCUAUUACUACUACUAC 5'
AT5G03545.1     306     325
unknown protein

sRNA_AGO1_Solexa_Mi2008_1_56092_hit3
5' UUUGUGAUGAUGAUGAUGAUGA
   |||| |||:|||||||||||||
   AAAC-CUAUUACUACUACUACU 5'
AT5G03545.1     306     326
unknown protein
```

```
sRNA_AGO1_Solexa_Mi2008_1_7077_hit1
5' CAUCUCAGACCGAAUUGUC-CU
   ||||||||| ||||||| | ||
   GUAGAGUCUUGCUUAAC-GUGA 5'
AT5G04240.1     2605    2625
zinc finger protein - like

sRNA_AGO1_Solexa_Mi2008_3_28073_hit1
5' UCAGAAUUCAGUGUCUGUUGGU
   :|| |||||||||||||| |||
   GGU-UUAAGUCACAGACAUCCA 5'
AT5G04240.1     60      80
zinc finger protein - like

sRNA_AGO1_Solexa_Mi2008_2_38066_hit1
5' UGCAGCAGGUCUCGGUGUCGA
   |||| ||:|||||||||||||
   ACGU-GUUCAGAGCCACAGCU 5'
AT5G04530.1     218     237
fatty acid elongase - like protein

sRNA_AGO1_Solexa_Mi2008_1_7082_hit4
5' CAUCUCCAGGA-ACCUCUUGAU
   | ||||||||| || |||||||
   GGAGAGGUCCUAUG-AGAACUA 5'
AT5G05320.1     800     820
monooxygenase

sRNA_AGO1_Solexa_Mi2008_1_646_hit1
5' AACAGAAAAAAAACA-UGAU
   ||||:||||||||||| |||
   UUGUUUUUUUUUUGUAACUU 5'
AT5G05340.1     1138    1157
peroxidase

sRNA_AGO1_Solexa_Mi2008_13_14779_hit6
5' GCUGAGAAUCGAAAU-AGUU
    || ||||||||||| ||||
   AGA-UCUUAGCUUUAUUCAA 5'
AT5G05340.1     70      88
peroxidase

sRNA_AGO1_Solexa_Mi2008_6_11866_hit1
5' CUGGAACAG-AUUCUCGAAGGUC
   ||||||||| ||  |||||||||
   GACCUUGUCGUAU-AGCUUCCAG 5'
AT5G05520.1     1061    1082
Unknown protein (MOP10.6)

sRNA_AGO1_Solexa_Mi2008_5_4006_hit2
5' CAAACCCUUGCAGCAUCUGUU
   ||||||||||||||| || ||
   GUUUGGGAACGUCGUUGA-AA 5'
AT5G05640.1     803     822
putative protein

sRNA_AGO1_Solexa_Mi2008_1_48514_hit1
5' UUCAAGCUUCAGAUGCUCUUCUU
   |||||||||||||| | ||||||
   AAGUUCGAAGUCUA-G-GAAGAA 5'
AT5G05740.1     139     159
putative protein
```

```
sRNA_AGO1_Solexa_Mi2008_1_48514_hit1
5' UUCAAGCUUCAGAUGCUCUUCUU
   |||||||||||||| | ||||||
   AAGUUCGAAGUCUA-G-GAAGAA 5'
AT5G05740.2     294     314
putative protein

sRNA_AGO1_Solexa_Mi2008_1_28782_hit1
5' UCAUAACAGGUGAAUGUCGGU
   |||||||||||||||||||||
   AGUAUUGUCCACUUACAGCCA 5'
AT5G06100.2     1070    1090
transcription factor MYB33 - like protein

sRNA_AGO1_Solexa_Mi2008_1_28782_hit1
5' UCAUAACAGGUGAAUGUCGGU
   |||||||||||||||||||||
   AGUAUUGUCCACUUACAGCCA 5'
AT5G06100.3     1158    1178
transcription factor MYB33 - like protein

sRNA_AGO1_Solexa_Mi2008_38_14830_hit2
5' GGACUGAAGGGAGCUCCCU
   ||| |||||||||||||
   CCUUACUUCCCUCGAGGUC 5'
AT5G06100.2     1159    1177
transcription factor MYB33 - like protein

sRNA_AGO1_Solexa_Mi2008_19_40112_hit2
5' UGGACUGAAGGGAGCUCCCU
   |||| |||||||||||||
   ACCUUACUUCCCUCGAGGUC 5'
AT5G06100.2     1159    1178
transcription factor MYB33 - like protein

sRNA_AGO1_Solexa_Mi2008_705_52065_hit2
5' UUGGACUGAAGGGAGCUCCCU
   ||||| |||||||||||||
   AACCUUACUUCCCUCGAGGUC 5'
AT5G06100.2     1159    1179
transcription factor MYB33 - like protein

sRNA_AGO1_Solexa_Mi2008_16_52069_hit1
5' UUGGACUGAAGGGAGCUCCUU
   ||||| |||||||||||||
   AACCUUACUUCCCUCGAGGUC 5'
AT5G06100.2     1159    1179
transcription factor MYB33 - like protein

sRNA_AGO1_Solexa_Mi2008_7_3717_hit1
5' AUUGGACUGAAGGGAGCUCCCU
   |||||| |||||||||||||
   UAACCUUACUUCCCUCGAGGUC 5'
AT5G06100.2     1159    1180
transcription factor MYB33 - like protein

sRNA_AGO1_Solexa_Mi2008_3349_41018_hit1
5' UGGAUUGAAGGGAGCUCUA
   |||| |||||||||||:|
   ACCUUACUUCCCUCGAGGU 5'
AT5G06100.2     1160    1178
transcription factor MYB33 - like protein
```

```
sRNA_AGO1_Solexa_Mi2008_1621_41020_hit1
5' UGGAUUGAAGGGAGCUCUU
   |||| ||||||||||||:
   ACCUUACUUCCCUCGAGGU 5'
AT5G06100.2     1160    1178
transcription factor MYB33 - like protein

sRNA_AGO1_Solexa_Mi2008_24_52064_hit2
5' UUGGACUGAAGGGAGCUCCC
   ||||| ||||||||||||| 
   AACCUUACUUCCCUCGAGGU 5'
AT5G06100.2     1160    1179
transcription factor MYB33 - like protein

sRNA_AGO1_Solexa_Mi2008_7_52068_hit1
5' UUGGACUGAAGGGAGCUCCU
   ||||| ||||||||||||| 
   AACCUUACUUCCCUCGAGGU 5'
AT5G06100.2     1160    1179
transcription factor MYB33 - like protein

sRNA_AGO1_Solexa_Mi2008_1_52243_hit1
5' UUGGAUUGAAGGGAGCUCCU
   ||||| ||||||||||||| 
   AACCUUACUUCCCUCGAGGU 5'
AT5G06100.2     1160    1179
transcription factor MYB33 - like protein

sRNA_AGO1_Solexa_Mi2008_3416_52245_hit1
5' UUGGAUUGAAGGGAGCUCUA
   ||||| ||||||||||||:|
   AACCUUACUUCCCUCGAGGU 5'
AT5G06100.2     1160    1179
transcription factor MYB33 - like protein

sRNA_AGO1_Solexa_Mi2008_870_52246_hit1
5' UUGGAUUGAAGGGAGCUCUU
   ||||| ||||||||||||:
   AACCUUACUUCCCUCGAGGU 5'
AT5G06100.2     1160    1179
transcription factor MYB33 - like protein

sRNA_AGO1_Solexa_Mi2008_1_3716_hit1
5' AUUGGACUGAAGGGAGCUCCC
   |||||| ||||||||||||| 
   UAACCUUACUUCCCUCGAGGU 5'
AT5G06100.2     1160    1180
transcription factor MYB33 - like protein

sRNA_AGO1_Solexa_Mi2008_21_12906_hit1
5' CUUGGACUGAAGGGAGCUCCC
    ||||| ||||||||||||| 
   UAACCUUACUUCCCUCGAGGU 5'
AT5G06100.2     1160    1180
transcription factor MYB33 - like protein

sRNA_AGO1_Solexa_Mi2008_61_55550_hit1
5' UUUGGACUGAAGGGAGCUCCU
    ||||| ||||||||||||| 
   UAACCUUACUUCCCUCGAGGU 5'
AT5G06100.2     1160    1180
transcription factor MYB33 - like protein
```

```
sRNA_AGO1_Solexa_Mi2008_14_55632_hit1
5' UUUGGAUUGAAGGGAGCUCCU
    ||||| ||||||||||||
   UAACCUUACUUCCCUCGAGGU 5'
AT5G06100.2     1160    1180
transcription factor MYB33 - like protein

sRNA_AGO1_Solexa_Mi2008_15_52063_hit3
5' UUGGACUGAAGGGAGCUCC
   ||||| ||||||||||||
   AACCUUACUUCCCUCGAGG 5'
AT5G06100.2     1161    1179
transcription factor MYB33 - like protein

sRNA_AGO1_Solexa_Mi2008_52_52244_hit2
5' UUGGAUUGAAGGGAGCUCU
   ||||| |||||||||||:
   AACCUUACUUCCCUCGAGG 5'
AT5G06100.2     1161    1179
transcription factor MYB33 - like protein

sRNA_AGO1_Solexa_Mi2008_9_55631_hit1
5' UUUGGAUUGAAGGGAGCUCC
    ||||| ||||||||||||
   UAACCUUACUUCCCUCGAGG 5'
AT5G06100.2     1161    1180
transcription factor MYB33 - like protein

sRNA_AGO1_Solexa_Mi2008_1201_55633_hit2
5' UUUGGAUUGAAGGGAGCUCU
    ||||| |||||||||||:
   UAACCUUACUUCCCUCGAGG 5'
AT5G06100.2     1161    1180
transcription factor MYB33 - like protein

sRNA_AGO1_Solexa_Mi2008_136_14386_hit1
5' GAUUGGACUGAAGGGAGCUCC
    |||||| ||||||||||||
   AUAACCUUACUUCCCUCGAGG 5'
AT5G06100.2     1161    1181
transcription factor MYB33 - like protein

sRNA_AGO1_Solexa_Mi2008_3_55549_hit1
5' UUUGGACUGAAGGGAGCUC
    ||||| |||||||||||
   UAACCUUACUUCCCUCGAG 5'
AT5G06100.2     1162    1180
transcription factor MYB33 - like protein

sRNA_AGO1_Solexa_Mi2008_907_55630_hit3
5' UUUGGAUUGAAGGGAGCUC
    ||||| |||||||||||
   UAACCUUACUUCCCUCGAG 5'
AT5G06100.2     1162    1180
transcription factor MYB33 - like protein

sRNA_AGO1_Solexa_Mi2008_4_13220_hit2
5' CUUUGGAUUGAAGGGAGCUC
     ||||| |||||||||||
   AUAACCUUACUUCCCUCGAG 5'
AT5G06100.2     1162    1181
transcription factor MYB33 - like protein
```

```
sRNA_AGO1_Solexa_Mi2008_1_14385_hit1
5' GAUUGGACUGAAGGGAGCUC
    |||||| ||||||||||||
   AUAACCUUACUUCCCUCGAG 5'
AT5G06100.2     1162    1181
transcription factor MYB33 - like protein

sRNA_AGO1_Solexa_Mi2008_5_13219_hit2
5' CUUUGGAUUGAAGGGAGCU
     ||||| |||||||||||
   AUAACCUUACUUCCCUCGA 5'
AT5G06100.2     1163    1181
transcription factor MYB33 - like protein

sRNA_AGO1_Solexa_Mi2008_38_14830_hit2
5' GGACUGAAGGGAGCUCCCU
   ||| |||||||||||||
   CCUUACUUCCCUCGAGGUC 5'
AT5G06100.3     1247    1265
transcription factor MYB33 - like protein

sRNA_AGO1_Solexa_Mi2008_19_40112_hit2
5' UGGACUGAAGGGAGCUCCCU
   |||| |||||||||||||
   ACCUUACUUCCCUCGAGGUC 5'
AT5G06100.3     1247    1266
transcription factor MYB33 - like protein

sRNA_AGO1_Solexa_Mi2008_705_52065_hit2
5' UUGGACUGAAGGGAGCUCCCU
   ||||| |||||||||||||
   AACCUUACUUCCCUCGAGGUC 5'
AT5G06100.3     1247    1267
transcription factor MYB33 - like protein

sRNA_AGO1_Solexa_Mi2008_16_52069_hit1
5' UUGGACUGAAGGGAGCUCCUU
   ||||| |||||||||||||
   AACCUUACUUCCCUCGAGGUC 5'
AT5G06100.3     1247    1267
transcription factor MYB33 - like protein

sRNA_AGO1_Solexa_Mi2008_7_3717_hit1
5' AUUGGACUGAAGGGAGCUCCCU
   |||||| |||||||||||||
   UAACCUUACUUCCCUCGAGGUC 5'
AT5G06100.3     1247    1268
transcription factor MYB33 - like protein

sRNA_AGO1_Solexa_Mi2008_3349_41018_hit1
5' UGGAUUGAAGGGAGCUCUA
   |||| ||||||||||||:|
   ACCUUACUUCCCUCGAGGU 5'
AT5G06100.3     1248    1266
transcription factor MYB33 - like protein

sRNA_AGO1_Solexa_Mi2008_1621_41020_hit1
5' UGGAUUGAAGGGAGCUCUU
   |||| ||||||||||||:
   ACCUUACUUCCCUCGAGGU 5'
AT5G06100.3     1248    1266
transcription factor MYB33 - like protein
```

```
sRNA_AGO1_Solexa_Mi2008_24_52064_hit2
5' UUGGACUGAAGGGAGCUCCC
   ||||| ||||||||||||| 
   AACCUUACUUCCCUCGAGGU 5'
AT5G06100.3     1248    1267
transcription factor MYB33 - like protein

sRNA_AGO1_Solexa_Mi2008_7_52068_hit1
5' UUGGACUGAAGGGAGCUCCU
   ||||| ||||||||||||| 
   AACCUUACUUCCCUCGAGGU 5'
AT5G06100.3     1248    1267
transcription factor MYB33 - like protein

sRNA_AGO1_Solexa_Mi2008_1_52243_hit1
5' UUGGAUUGAAGGGAGCUCCU
   ||||| ||||||||||||| 
   AACCUUACUUCCCUCGAGGU 5'
AT5G06100.3     1248    1267
transcription factor MYB33 - like protein

sRNA_AGO1_Solexa_Mi2008_3416_52245_hit1
5' UUGGAUUGAAGGGAGCUCUA
   ||||| ||||||||||||:|
   AACCUUACUUCCCUCGAGGU 5'
AT5G06100.3     1248    1267
transcription factor MYB33 - like protein

sRNA_AGO1_Solexa_Mi2008_870_52246_hit1
5' UUGGAUUGAAGGGAGCUCUU
   ||||| ||||||||||||: 
   AACCUUACUUCCCUCGAGGU 5'
AT5G06100.3     1248    1267
transcription factor MYB33 - like protein

sRNA_AGO1_Solexa_Mi2008_1_3716_hit1
5' AUUGGACUGAAGGGAGCUCCC
   |||||| ||||||||||||| 
   UAACCUUACUUCCCUCGAGGU 5'
AT5G06100.3     1248    1268
transcription factor MYB33 - like protein

sRNA_AGO1_Solexa_Mi2008_21_12906_hit1
5' CUUGGACUGAAGGGAGCUCCC
    ||||| ||||||||||||| 
   UAACCUUACUUCCCUCGAGGU 5'
AT5G06100.3     1248    1268
transcription factor MYB33 - like protein

sRNA_AGO1_Solexa_Mi2008_61_55550_hit1
5' UUUGGACUGAAGGGAGCUCCU
    ||||| ||||||||||||| 
   UAACCUUACUUCCCUCGAGGU 5'
AT5G06100.3     1248    1268
transcription factor MYB33 - like protein

sRNA_AGO1_Solexa_Mi2008_14_55632_hit1
5' UUUGGAUUGAAGGGAGCUCCU
    ||||| ||||||||||||| 
   UAACCUUACUUCCCUCGAGGU 5'
AT5G06100.3     1248    1268
transcription factor MYB33 - like protein
```

```
sRNA_AGO1_Solexa_Mi2008_15_52063_hit3
5' UUGGACUGAAGGGAGCUCC
   ||||| |||||||||||||
   AACCUUACUUCCCUCGAGG 5'
AT5G06100.3     1249    1267
transcription factor MYB33 - like protein

sRNA_AGO1_Solexa_Mi2008_52_52244_hit2
5' UUGGAUUGAAGGGAGCUCU
   ||||| ||||||||||||:
   AACCUUACUUCCCUCGAGG 5'
AT5G06100.3     1249    1267
transcription factor MYB33 - like protein

sRNA_AGO1_Solexa_Mi2008_9_55631_hit1
5' UUUGGAUUGAAGGGAGCUCC
    ||||| |||||||||||||
   UAACCUUACUUCCCUCGAGG 5'
AT5G06100.3     1249    1268
transcription factor MYB33 - like protein

sRNA_AGO1_Solexa_Mi2008_1201_55633_hit2
5' UUUGGAUUGAAGGGAGCUCU
    ||||| ||||||||||||:
   UAACCUUACUUCCCUCGAGG 5'
AT5G06100.3     1249    1268
transcription factor MYB33 - like protein

sRNA_AGO1_Solexa_Mi2008_136_14386_hit1
5' GAUUGGACUGAAGGGAGCUCC
    |||||| |||||||||||||
   AUAACCUUACUUCCCUCGAGG 5'
AT5G06100.3     1249    1269
transcription factor MYB33 - like protein

sRNA_AGO1_Solexa_Mi2008_3_55549_hit1
5' UUUGGACUGAAGGGAGCUC
    ||||| ||||||||||||
   UAACCUUACUUCCCUCGAG 5'
AT5G06100.3     1250    1268
transcription factor MYB33 - like protein

sRNA_AGO1_Solexa_Mi2008_907_55630_hit3
5' UUUGGAUUGAAGGGAGCUC
    ||||| ||||||||||||
   UAACCUUACUUCCCUCGAG 5'
AT5G06100.3     1250    1268
transcription factor MYB33 - like protein

sRNA_AGO1_Solexa_Mi2008_4_13220_hit2
5' CUUUGGAUUGAAGGGAGCUC
     ||||| ||||||||||||
   AUAACCUUACUUCCCUCGAG 5'
AT5G06100.3     1250    1269
transcription factor MYB33 - like protein

sRNA_AGO1_Solexa_Mi2008_1_14385_hit1
5' GAUUGGACUGAAGGGAGCUC
    |||||| ||||||||||||
   AUAACCUUACUUCCCUCGAG 5'
AT5G06100.3     1250    1269
transcription factor MYB33 - like protein
```

```
sRNA_AGO1_Solexa_Mi2008_5_13219_hit2
5' CUUUGGAUUGAAGGGAGCU
     ||||| |||||||||||
   AUAACCUUACUUCCCUCGA 5'
AT5G06100.3     1251    1269
transcription factor MYB33 - like protein

sRNA_AGO1_Solexa_Mi2008_1_2_hit38
5' AAAAAAAAAA-AAAAACCAU
   |||||||||| |||| |||
   UUUUUUUUUUGUUUUAGGUC 5'
AT5G06100.2     137     156
transcription factor MYB33 - like protein

sRNA_AGO1_Solexa_Mi2008_1_2_hit38
5' AAAAAAAAAA-AAAAACCAU
   |||||||||| |||| |||
   UUUUUUUUUUGUUUUAGGUC 5'
AT5G06100.3     144     163
transcription factor MYB33 - like protein

sRNA_AGO1_Solexa_Mi2008_1_4084_hit1
5' CAAAGAGGAGAGCA--CAAUGGAA
    |||||||||||||  ||||||||
   AUUUCUCCUCUCGUAGGUUACCUU 5'
AT5G06530.1     1300    1323
ABC transporter like protein

sRNA_AGO1_Solexa_Mi2008_1_4084_hit1
5' CAAAGAGGAGAGCA--CAAUGGAA
    |||||||||||||  ||||||||
   AUUUCUCCUCUCGUAGGUUACCUU 5'
AT5G06530.3     1404    1427
ABC transporter like protein

sRNA_AGO1_Solexa_Mi2008_1_11845_hit13
5' CUG-CUUCUUGGCCUCUGUGAU
   ||| ||||||| |||||| |||
   GACCGAAGAACAGGAGAC-CUA 5'
AT5G07340.1     767     787
calnexin homolog

sRNA_AGO1_Solexa_Mi2008_1_3_hit25
5' AAAAAAAAAAAAAGAAAGA
   ||||||||||||| |||
   UUUUUUUUUUUUU-UUUAG 5'
AT5G07440.3     137     154
glutamate dehydrogenase 2 (GDH2)

sRNA_AGO1_Solexa_Mi2008_1_1776_hit10
5' AGAA-AAAAAAAAAAAAAAAAU
    ||| ||||||||||||||||
   GCUUGUUUUUUUUUUUUUUUUA 5'
AT5G07440.3     138     159
glutamate dehydrogenase 2 (GDH2)

sRNA_AGO1_Solexa_Mi2008_1_3_hit25
5' AAAAAAAAAAAAAGAAAGA
   ||||||||||||| |||
   UUUUUUUUUUUUU-UUUAG 5'
AT5G07440.1     162     179
glutamate dehydrogenase 2 (GDH2)
```

```
sRNA_AGO1_Solexa_Mi2008_1_1776_hit10
5' AGAA-AAAAAAAAAAAAAAAAU
    ||| ||||||||||||||||
   GCUUGUUUUUUUUUUUUUUUUA 5'
AT5G07440.1     163     184
glutamate dehydrogenase 2 (GDH2)

sRNA_AGO1_Solexa_Mi2008_5_5595_hit1
5' CACGAGAACGGGACU-ACAAAU
   ||||||||||||||| | ||||
   GUGCUCUUGCCCUGACU-UUUA 5'
AT5G07550.3     179     199
glycine-rich protein PUTG1

sRNA_AGO1_Solexa_Mi2008_5_5595_hit1
5' CACGAGAACGGGACU-ACAAAU
   ||||||||||||||| | ||||
   GUGCUCUUGCCCUGACU-UUUA 5'
AT5G07550.1     186     206
glycine-rich protein PUTG1

sRNA_AGO1_Solexa_Mi2008_2_46801_hit10
5' UUA-CAAAAUAACCAUGGAC
   ||| |||||| ||||||||
   AAUAGUUUUAGUGGUACCUA 5'
AT5G08139.1     209     228
unknown protein

sRNA_AGO1_Solexa_Mi2008_29_7893_hit1
5' CCCAAUCAACUUCAUCACCAG
   ||||||||| ||||||| |||
   GGGUUAGUUUAAGUAGUCGUC 5'
AT5G08139.1     647     667
unknown protein

sRNA_AGO1_Solexa_Mi2008_19_12362_hit1
5' CUUACCCAAUCAACUUCAUCAC
   ||| ||||||||| |||||||
   GAA-GGGUUAGUUUAAGUAGUC 5'
AT5G08139.1     651     671
unknown protein

sRNA_AGO1_Solexa_Mi2008_1_34074_hit1
5' UGAAAACGGGAAUGGAGCGAAGAA
   |||| ||||:||||||||:|||||
   ACUUCUGCCUUUACCUCGUUUCUU 5'
AT5G08300.1     80      103
succinyl-CoA-ligase alpha subunit

sRNA_AGO1_Solexa_Mi2008_1_20620_hit1
5' UACUGGGCAUGUGAAAUGGGU
   ||||||:||||||| ||||||
   AUGACCUGUACACUGUACCCA 5'
AT5G09220.1     635     655
amino acid transport protein AAP2

sRNA_AGO1_Solexa_Mi2008_1_28727_hit1
5' UCAG-UGUGGUGUUGGUGUCGAU
   |||| |||| | |||||||||||
   AGUCGACACAAGAACCACAGCUA 5'
AT5G09590.1     1917    1939
heat shock protein 70 (Hsc70-5)
```

```
sRNA_AGO1_Solexa_Mi2008_1_23048_hit1
5' UAGG-GAGUAGCCACUGGAUGC
   |||| ||||||||||:|||||
   AUCCUCUCAUCGGUGGCCUACC 5'
AT5G10170.1     1458    1479
myo-inositol-1-phosphate synthase -like protein

sRNA_AGO1_Solexa_Mi2008_339_11394_hit3
5' CUGAAGUGUUUGGGGGAACUC
    |||||||||||||:|||||
   AACUUCACAAACCCUCUUGAA 5'
AT5G10180.1     124     144
sulfate transporter

sRNA_AGO1_Solexa_Mi2008_1_22796_hit1
5' UAGGAUAUUGAUAUAGUGU
   |||||||||||| ||||:|
   AUCCUAUAACUA-AUCAUA 5'
AT5G10180.1     1712    1729
sulfate transporter

sRNA_AGO1_Solexa_Mi2008_1_4581_hit2
5' CAAGA-AUACUACAGCCAU-GGUC
   ||||| ||||||||||||: ||||
   GUUCUCUAUGAUGUCGGUGGCCAG 5'
AT5G10180.1     590     613
sulfate transporter

sRNA_AGO1_Solexa_Mi2008_6_50680_hit1
5' UUGAGGCAAAGAACAUCCGAA
    ||||| |||||||||||||
   CACUCC-UUUCUUGUAGGCUC 5'
AT5G10180.1     931     950
sulfate transporter

sRNA_AGO1_Solexa_Mi2008_1_53946_hit2
5' UUUAACUGCAACAACUUAAA
   |||| |||||||||||||
   AAAU-GACGUUGUUGAAUAA 5'
AT5G10210.1     736     754
putative protein

sRNA_AGO1_Solexa_Mi2008_2_43551_hit1
5' UGGUGGUGACGUUGGUGGUGGU
    |||||||| | ||||||||||
   UCCACCACUACCACCACCACCA 5'
AT5G10550.1     1289    1310
bromodomain protein - like

sRNA_AGO1_Solexa_Mi2008_2_42329_hit1
5' UGGGGAGGUGGUGAUGCG-GGU
   |||||||||||||||| | |||
   ACCCCUCCACCACUAC-CACCA 5'
AT5G10550.1     1296    1316
bromodomain protein - like

sRNA_AGO1_Solexa_Mi2008_1_20849_hit1
5' UAGAACCU-CGGUCGAGAAUG-GU
   ||| |||| |||||||||||| ||
   AUCGUGGAAGCCAGCUCUUACUCA 5'
AT5G10760.1     1046    1069
nucleoid DNA-binding protein cnd41 - like protein
```

```
sRNA_AGO1_Solexa_Mi2008_1_4400_hit1
5' CAACAUUUAGCGUCGUCUGC
   ||||||:|||||||||||||
   GUUGUAGAUCGCAGCAGACG 5'
AT5G10760.1     1367    1386
nucleoid DNA-binding protein cnd41 - like protein

sRNA_AGO1_Solexa_Mi2008_1_13420_hit2
5' GAAGAAGAAGAA-GACUCUU
   |||||||||||| |||||
   CUUCUUCUUCUUCCUGAGCC 5'
AT5G11390.1     7       26
unknown protein

sRNA_AGO1_Solexa_Mi2008_2_40093_hit1
5' UGGACUAACUACAUGCAAGGG
   |||||||||||| || ||||
   ACCUGAUUGAUG-AC-UUCCG 5'
AT5G12930.1     1236    1254
unknown protein

sRNA_AGO1_Solexa_Mi2008_1_44719_hit2
5' UGUCGAUGGACUUGCUACUGAA
    ||||||||||| :||||||||
   UCAGCUACCUGAUUGAUGACUU 5'
AT5G12930.1     1238    1259
unknown protein

sRNA_AGO1_Solexa_Mi2008_1_3917_hit1
5' CAAAAGAAGCAA-AAGUUUGUU
   ||||||||| || || ||||||
   GUUUUCUUC-UUCUUAAAACAA 5'
AT5G13080.1     50      70
WRKY-like protein

sRNA_AGO1_Solexa_Mi2008_2_22589_hit1
5' UAGGAAAUAGUGAUUGAUGCAU
   :||||||| ||||||||| |||
   GUCCUUUACCACUAACUA-GUA 5'
AT5G13170.1     145     165
senescence-associated protein (SAG29)

sRNA_AGO1_Solexa_Mi2008_1_17162_hit1
5' UAACA-AGAGGAAGAAGAACGAU
   ||||| ||||||||||||| ||
   AUUGUCUCUCCUUCUUCUU-CUU 5'
AT5G13180.1     24      45
NAM-like protein

sRNA_AGO1_Solexa_Mi2008_1_14_hit1
5' AAAAAAACAGAGAACAAGAAGA
    ||||||||||||  |||||||
   CUUUUUUGUCUCUCCUUCUUCU 5'
AT5G13180.1     27      48
NAM-like protein

sRNA_AGO1_Solexa_Mi2008_1_341_hit1
5' AAAGAAACAGAGAGGAAGAU
   ||| |||||||||||||||
   UUU-UUUGUCUCUCCUUCUU 5'
AT5G13180.1     30      48
NAM-like protein
```

```
sRNA_AGO1_Solexa_Mi2008_3_10681_hit1
5' CUCACGAUUUGAUUUCCUCU
   |: |||||||||||||||||
   GGUUGCUAAACUAAAGGAGA 5'
AT5G13180.1     383     402
NAM-like protein

sRNA_AGO1_Solexa_Mi2008_12_14067_hit2
5' GAGG-A-UCCAUUGGAGGGCA
   |||| | |||||||:||||||
   CUCCGUUAGGUAACUUCCCGU 5'
AT5G13190.1     462     482
unknown protein

sRNA_AGO1_Solexa_Mi2008_1_1732_hit1
5' ACUGAGCAGCUUGAGGUGU-GA
   |||| | |||||||||||| ||
   UGAC-CUUCGAACUCCACAACU 5'
AT5G13400.1     1374    1394
peptide transporter - like protein

sRNA_AGO1_Solexa_Mi2008_1_13917_hit1
5' GAGAAUAACGAGAAG-GAAG
    |||||||||||||| |||
   AUCUUAUUGCUCUUCUCUUG 5'
AT5G13400.1     2035    2054
peptide transporter - like protein

sRNA_AGO1_Solexa_Mi2008_1_22897_hit1
5' UAGGCAUGUCUGAGGAUUGGU
   |||| |||| |||||||||||
   AUCC-UACAUACUCCUAACCA 5'
AT5G13400.1     2       21
peptide transporter - like protein

sRNA_AGO1_Solexa_Mi2008_1_52886_hit1
5' UUGGUACUGAAG-UCGGCAAUAG
   |||||| ||||| | ||||||||
   AACCAUCACUUCUA-CCGUUAUC 5'
AT5G13400.1     557     578
peptide transporter - like protein

sRNA_AGO1_Solexa_Mi2008_1_448_hit2
5' AAAGCAUUUGCCAAGGAUGUU
   |||||||||||||| ||| ||
   UUUCGUAAACGGUU-CUA-AA 5'
AT5G13400.1     691     709
peptide transporter - like protein

sRNA_AGO1_Solexa_Mi2008_5_479_hit1
5' AA-AGGAAGAAGAUAUGGAGUU
   || ||||||||||| ||||||:
   UUCUCCUUCUUCUAAACCUCAG 5'
AT5G13420.1     149     170
transaldolase - like protein

sRNA_AGO1_Solexa_Mi2008_1_16815_hit1
5' UAAAGGAGGAAGCAACCACUG
    ||||:|||||||||| ||||
   CUUUCUUCCUUCGUUG-UGAC 5'
AT5G13420.1     892     911
transaldolase - like protein
```

```
sRNA_AGO1_Solexa_Mi2008_1_8580_hit1
5' CGAGGAUU-GGUGGUACUUAGGU
   || ||||| ||||||||||||:
   GC-CCUAAACCACCAUGAAUCCG 5'
AT5G13490.2     1198    1219
adenosine nucleotide translocator

sRNA_AGO1_Solexa_Mi2008_1_8580_hit1
5' CGAGGAUU-GGUGGUACUUAGGU
   || ||||| ||||||||||||:
   GC-CCUAAACCACCAUGAAUCCG 5'
AT5G13490.1     1262    1283
adenosine nucleotide translocator

sRNA_AGO1_Solexa_Mi2008_21_23402_hit1
5' UAGGUCGAGCUUCAUUGGA
    ||| ||||||||||||||
   CUCCUGCUCGAAGUAACCU 5'
AT5G13550.1     197     215
sulfate transporter

sRNA_AGO1_Solexa_Mi2008_1_21106_hit1
5' UAGAC-GUAAAUUGGUGGCAU
   ||||| |||||||||||| |
   AUCUGGCAUUUAACCACCUUC 5'
AT5G13550.1     426     446
sulfate transporter

sRNA_AGO1_Solexa_Mi2008_5_4303_hit1
5' CAAAUUGGUG-CUUGCUCAACAA
   ||||||| |  ||||||||||||
   GUUUAACAAAUGAACGAGUUGUU 5'
AT5G13680.1     2267    2289
putative protein

sRNA_AGO1_Solexa_Mi2008_1_6827_hit1
5' CAUAAGCCCAAUCAUAAUGUG
   || |||||||||||||| ||
   GU-UUCGGGUUAGUAUU-CAG 5'
AT5G13680.1     429     447
putative protein

sRNA_AGO1_Solexa_Mi2008_1_22432_hit10
5' UAGCUAAAAAACCACCAUCGAG
   :|||||||||||||||  |||
   GUCGAUUUUUUGGUGGUU-CUC 5'
AT5G13740.1     817     837
transporter-like protein

sRNA_AGO1_Solexa_Mi2008_1_40339_hit1
5' UGGAGAUGGAAGAUGAG-CC
   |||||||||||| |||| ||
   ACCUCUACCUUCCACUCUGG 5'
AT5G13930.1     876     895
chalcone synthase (naringenin-chalcone synthase) (testa 4 protein) (sp|P13114)

sRNA_AGO1_Solexa_Mi2008_12_10453_hit13
5' CUAUAGUCCAGAUGAAUUGCU
    |||| ||||| |||||||||
   UAUAU-AGGUCGACUUAACGA 5'
AT5G14580.1     1748    1767
polynucleotide phosphorylase
```

```
sRNA_AGO1_Solexa_Mi2008_2_56340_hit1
5' UUUUAUUAGUAGAUUAAGAAU
   |||| ||| |||||||||||
   AAAA-AAUAAUCUAAUUCUUA 5'
AT5G14800.1     1240    1259
pyrroline-5-carboxylate reductase

sRNA_AGO1_Solexa_Mi2008_2_56340_hit1
5' UUUUAUUAGUAGAUUAAGAAU
   |||| ||| |||||||||||
   AAAA-AAUAAUCUAAUUCUUA 5'
AT5G14800.2     1244    1263
pyrroline-5-carboxylate reductase

sRNA_AGO1_Solexa_Mi2008_1_8021_hit2
5' CCGCGAGUUGAUGAGCUA-UUG
   |||||||:|||||||||| |||
   GGCGCUCGACUACUCGAUGAAC 5'
AT5G14800.2     134     155
pyrroline-5-carboxylate reductase

sRNA_AGO1_Solexa_Mi2008_1_50501_hit2
5' UUGAGACGGGCGAACGGAUGAGCU
   ||||||||||||| | ||||||||
   AACUCUGCCCGCUCGACUACUCGA 5'
AT5G14800.2     138     161
pyrroline-5-carboxylate reductase

sRNA_AGO1_Solexa_Mi2008_1_50983_hit4
5' UUGAUACGGGUGAGCUGACGAGCU
   |||| |||||:||||||| |||||
   AACUCUGCCCGCUCGACUACUCGA 5'
AT5G14800.2     138     161
pyrroline-5-carboxylate reductase

sRNA_AGO1_Solexa_Mi2008_3_35861_hit32
5' UGAGACGGGUGAGCUGACGAG
   |||||||||:||||||| |||
   ACUCUGCCCGCUCGACUACUC 5'
AT5G14800.2     140     160
pyrroline-5-carboxylate reductase

sRNA_AGO1_Solexa_Mi2008_1_50502_hit3
5' UUGAGACGGGCGAACUGAUGAG
   ||||||||||||| ||||||||
   AACUCUGCCCGCUCGACUACUC 5'
AT5G14800.2     140     161
pyrroline-5-carboxylate reductase

sRNA_AGO1_Solexa_Mi2008_31_50503_hit32
5' UUGAGACGGGUGAGCUGACGAG
   ||||||||||:||||||| |||
   AACUCUGCCCGCUCGACUACUC 5'
AT5G14800.2     140     161
pyrroline-5-carboxylate reductase

sRNA_AGO1_Solexa_Mi2008_1_35500_hit29
5' UGACGAGCUGCUUGAGACGGGU
   |||||||||||||||||||||:
   ACUGCUCGACGAACUCUGCCCG 5'
AT5G14800.2     151     172
pyrroline-5-carboxylate reductase
```

```
sRNA_AGO1_Solexa_Mi2008_6_35501_hit3
5' UGACGAGCUGCUUGAUACGGAC
   ||||||||||||||| |||| |
   ACUGCUCGACGAACUCUGCCCG 5'
AT5G14800.2     151     172
pyrroline-5-carboxylate reductase

sRNA_AGO1_Solexa_Mi2008_2_31782_hit15
5' UCGGAGGGGAGAGCUGACGAG
   ||||| ||| |||||||||||
   AGCCUGCCCACUCGACUGCUC 5'
AT5G14800.2     166     186
pyrroline-5-carboxylate reductase

sRNA_AGO1_Solexa_Mi2008_3_35861_hit32
5' UGAGACGGGUGAGCUGACGAG
   |  ||||||||||||||||||
   AGCCUGCCCACUCGACUGCUC 5'
AT5G14800.2     166     186
pyrroline-5-carboxylate reductase

sRNA_AGO1_Solexa_Mi2008_7_49399_hit1
5' UUCGGACGGGUGAGCUGACAAG
   ||||||||||||||||||| ||
   AAGCCUGCCCACUCGACUGCUC 5'
AT5G14800.2     166     187
pyrroline-5-carboxylate reductase

sRNA_AGO1_Solexa_Mi2008_5_50475_hit1
5' UU-GAGAAGGGUGAGCUGACGAG
   || | || |||||||||||||||
   AAGC-CUGCCCACUCGACUGCUC 5'
AT5G14800.2     167     188
pyrroline-5-carboxylate reductase

sRNA_AGO1_Solexa_Mi2008_31_50503_hit32
5' UU-GAGACGGGUGAGCUGACGAG
   || | ||||||||||||||||||
   AAGC-CUGCCCACUCGACUGCUC 5'
AT5G14800.2     167     188
pyrroline-5-carboxylate reductase

sRNA_AGO1_Solexa_Mi2008_5_50504_hit4
5' UU-GAGACGGGUGAGCUGACGAU
   || | |||||||||||||||||
   AAGC-CUGCCCACUCGACUGCUC 5'
AT5G14800.2     167     188
pyrroline-5-carboxylate reductase

sRNA_AGO1_Solexa_Mi2008_13_12760_hit32
5' CUU-GAGACGGGUGAGCUGACGA
   ||| | |||||||||||||||||
   GAAGC-CUGCCCACUCGACUGCU 5'
AT5G14800.2     168     189
pyrroline-5-carboxylate reductase

sRNA_AGO1_Solexa_Mi2008_1_41507_hit12
5' UGGCGGGAUACUUCGGACGGG
   |||||:|||||||||||||||
   ACCGCUCUAUGAAGCCUGCCC 5'
AT5G14800.2     178     198
pyrroline-5-carboxylate reductase
```

```
sRNA_AGO1_Solexa_Mi2008_3_11956_hit15
5' CUGGCGGGAUACUUCGGA-GGG
   ||||||:|||||||||||| |||
   GACCGCUCUAUGAAGCCUGCCC 5'
AT5G14800.2     179     200
pyrroline-5-carboxylate reductase

sRNA_AGO1_Solexa_Mi2008_3_35861_hit32
5' UGAGACGGGUGAGCUGACGAG
   | ||||||||||||||||||| 
   AGUCUGCCCACUCGACUGCUC 5'
AT5G14800.2     36      56
pyrroline-5-carboxylate reductase

sRNA_AGO1_Solexa_Mi2008_31_50503_hit32
5' UUGAGACGGGUGAGCUGACGAG
   || |||||||||||||||||||
   AAGUCUGCCCACUCGACUGCUC 5'
AT5G14800.2     36      57
pyrroline-5-carboxylate reductase

sRNA_AGO1_Solexa_Mi2008_13_12760_hit32
5' CUUGAGACGGGUGAGCUGACGA
   ||| ||||||||||||||||||
   GAAGUCUGCCCACUCGACUGCU 5'
AT5G14800.2     37      58
pyrroline-5-carboxylate reductase

sRNA_AGO1_Solexa_Mi2008_1_41507_hit12
5' UGGCGGGAUACUUCGGACGGG
   |||||:|||||||||:||||||
   ACCGCUCUAUGAAGUCUGCCC 5'
AT5G14800.2     48      68
pyrroline-5-carboxylate reductase

sRNA_AGO1_Solexa_Mi2008_1_35500_hit29
5' UGACGAGCUGCUUGAGACGGGU
   |||||||||||||||||||||:
   ACUGCUCGACGAACUCUGCCCG 5'
AT5G14800.2     73      94
pyrroline-5-carboxylate reductase

sRNA_AGO1_Solexa_Mi2008_6_35501_hit3
5' UGACGAGCUGCUUGAUACGGAC
   ||||||||||||||| |||| |
   ACUGCUCGACGAACUCUGCCCG 5'
AT5G14800.2     73      94
pyrroline-5-carboxylate reductase

sRNA_AGO1_Solexa_Mi2008_3_35861_hit32
5' UGAGACGGGUGAGCUGACGAG
   | ||||||||||| ||||||||
   AGUCUGCCCACUAGACUGCUC 5'
AT5G14800.2     88      108
pyrroline-5-carboxylate reductase

sRNA_AGO1_Solexa_Mi2008_13_12760_hit32
5' CUUGAGACGGGUGAGCUGACGA
   ||| ||||||||||| |||||||
   GAAGUCUGCCCACUAGACUGCU 5'
AT5G14800.2     89      110
pyrroline-5-carboxylate reductase
```

```
sRNA_AGO1_Solexa_Mi2008_76_8501_hit1
5' CGAGACGCUUCAGACGGGUGAG
   ||||| ||||||||||||||
   GCUCUACGAAGUCUGCCCACUA 5'
AT5G14800.2     96      117
pyrroline-5-carboxylate reductase

sRNA_AGO1_Solexa_Mi2008_1_35500_hit29
5' UGACGAGCUGCUUGAGACGGGU
   ||||||| ||||| ||||||||
   ACUGCUCUACGAAGUCUGCCCA 5'
AT5G14800.2     99      120
pyrroline-5-carboxylate reductase

sRNA_AGO1_Solexa_Mi2008_1_45665_hit1
5' UGUGUGAAGAGAGAAUGAUGG
   |||||||||||||||||||||
   ACACACUUCUCUCUUACUACC 5'
AT5G14930.2     422     442
SAG101

sRNA_AGO1_Solexa_Mi2008_1_45665_hit1
5' UGUGUGAAGAGAGAAUGAUGG
   |||||||||||||||||||||
   ACACACUUCUCUCUUACUACC 5'
AT5G14930.3     423     443
SAG101

sRNA_AGO1_Solexa_Mi2008_4_12767_hit1
5' CUUGAGCGUUUGG-GUCAACUA
   ||||||||||||| | |||||
   GAACUCGCAAACCUC-GUUGAG 5'
AT5G14930.2     498     518
SAG101

sRNA_AGO1_Solexa_Mi2008_4_12767_hit1
5' CUUGAGCGUUUGG-GUCAACUA
   ||||||||||||| | |||||
   GAACUCGCAAACCUC-GUUGAG 5'
AT5G14930.3     499     519
SAG101

sRNA_AGO1_Solexa_Mi2008_5_29024_hit1
5' UC-AUGGUUUCAAGAAUGCU
   || ||||||||||||| ||:
   AGCUACCAAAGUUCUU-CGG 5'
AT5G14930.2     586     604
SAG101

sRNA_AGO1_Solexa_Mi2008_5_29024_hit1
5' UC-AUGGUUUCAAGAAUGCU
   || ||||||||||||| ||:
   AGCUACCAAAGUUCUU-CGG 5'
AT5G14930.3     587     605
SAG101

sRNA_AGO1_Solexa_Mi2008_2_51035_hit1
5' UUGAUCAGAUGGUAAAGG-UGG
   ||||||||||||| |||| ||:
   AACUAGUCUACCA-UUCCGACU 5'
AT5G15650.1     172     192
reversibly glycosylated polypeptide-2 (AtRGP)
```

```
sRNA_AGO1_Solexa_Mi2008_1_21248_hit1
5' UAGAGAUCG--UCGCGUCGGUGA
   |||||||||  |||||||||||
   AUCUCUAGCCUAGCGCAGCCACU 5'
AT5G15650.1     6       28
reversibly glycosylated polypeptide-2 (AtRGP)

sRNA_AGO1_Solexa_Mi2008_1_56083_hit1
5' UUUGUCUUGACCAAUCAUACA
   ||||||| ||||| |||||||
   AAACAGAUCUGGUCAGUAUGU 5'
AT5G15950.1     1014    1034
S-adenosylmethionine decarboxylase (adoMetDC2)

sRNA_AGO1_Solexa_Mi2008_1_56083_hit1
5' UUUGUCUUGACCAAUCAUACA
   ||||||| ||||| |||||||
   AAACAGAUCUGGUCAGUAUGU 5'
AT5G15950.2     1096    1116
S-adenosylmethionine decarboxylase (adoMetDC2)

sRNA_AGO1_Solexa_Mi2008_1_36688_hit1
5' UGAGU-AAAUGAAAAGCAAUUAG
   | ||| ||||| |||||||||||
   AAUCAGUUUACGUUUCGUUAAUC 5'
AT5G15950.2     1831    1853
S-adenosylmethionine decarboxylase (adoMetDC2)

sRNA_AGO1_Solexa_Mi2008_1_9262_hit1
5' CGGUGGGGAUGAUGGACUAA
   ||||||||||||| || ||
   GCCACCCCUACUA-CU-AUC 5'
AT5G16110.1     614     631
unknown protein

sRNA_AGO1_Solexa_Mi2008_1_32339_hit1
5' UCGGUU-CCUGAAUAUGGCU
    ||||| || ||||||||||
   CGCCAACGG-CUUAUACCGA 5'
AT5G16110.1     738     756
unknown protein

sRNA_AGO1_Solexa_Mi2008_1_47599_hit1
5' UUAG-CGACGAUGAUGAUGAAG
   :||| ||||||||||| ||||
   GAUCUGCUGCUACUACUCCUUC 5'
AT5G16110.1     774     795
unknown protein

sRNA_AGO1_Solexa_Mi2008_19_11133_hit1
5' CUC-GGUGGUGAAGAUGCAG
   ||| ||||||||||||||
   GAGUCCACCACUUCUACGGG 5'
AT5G16260.1     614     633
putative protein

sRNA_AGO1_Solexa_Mi2008_10_49988_hit1
5' UUGAACAAUUGAUGUUG-UGCU
   |:|||||| |||||||| ||||
   AGCUUGUUUACUACAACCACGA 5'
AT5G17380.1     1544    1565
2-hydroxyphytanoyl-CoA lyase-like protein
```

```
sRNA_AGO1_Solexa_Mi2008_3_4962_hit3
5' CAAGUAAUAACAUUUAACCU
   ||| |||||||||||| ||
   GUU-AUUAUUGUAAAU-GGU 5'
AT5G17380.1     1681    1698
2-hydroxyphytanoyl-CoA lyase-like protein

sRNA_AGO1_Solexa_Mi2008_1_53538_hit1
5' UUGUGAAUUGGUGGAGAAU
   | ||||| |||||||||||
   AUCACUUUACCACCUCUUA 5'
AT5G17640.1     181     199
putative protein

sRNA_AGO1_Solexa_Mi2008_1_39739_hit2
5' UGGAAGAUGG-AGUGAUGCCUC
   ||||| |||| ||||||||||
   ACCUUGUACCCUCACUACGGAC 5'
AT5G17770.1     702     723
NADH-cytochrome b5 reductase

sRNA_AGO1_Solexa_Mi2008_1_25604_hit2
5' UAUGGAAGAUGG-AGUGAUGCCU
    |||||| |||| ||||||||||
   CUACCUUGUACCCUCACUACGGA 5'
AT5G17770.1     703     725
NADH-cytochrome b5 reductase

sRNA_AGO1_Solexa_Mi2008_4_13665_hit1
5' GACAUUUUGAAGAAUGCUUGGA
   || ||:||||||||||| ||||
   CU-UAGAACUUCUUACG-ACCU 5'
AT5G18000.1     220     239
putative protein

sRNA_AGO1_Solexa_Mi2008_2_56_hit1
5' AAAAAAGAGGAAGAUUGGU
      ||||||||||||||||
   CAAUUUCUCCUUCUAACCA 5'
AT5G18170.1     13      31
glutamate dehydrogenase (EC 1.4.1.-) 1 (pir||S71217)

sRNA_AGO1_Solexa_Mi2008_1_22493_hit1
5' UAGCUCCUGAU-GGUCGAGUA
   ||||||||||| |||| |||
   AUCGAGGACUACCCAGAUCAG 5'
AT5G18470.1     778     798
unknown protein

sRNA_AGO1_Solexa_Mi2008_3_15394_hit1
5' GUAAUCAGAGUGAGCAUCC
   |||||||:||||||||||
   CAUUAGUUUCACUCGUAGC 5'
AT5G18900.1     528     546
unknown protein

sRNA_AGO1_Solexa_Mi2008_1_40806_hit1
5' UGGAUAGAUAAAUAAG-AUUGAU
   |||||||||| ||||| |||| |
   ACCUAUCUAUGUAUUCUUAACAA 5'
AT5G18900.1     592     614
unknown protein
```

```
sRNA_AGO1_Solexa_Mi2008_2_2731_hit1
5' AUAUGUUCUCUGUCUGCUUCU
   |||||||||  ||||||||||
   UAUACAAGA--CAGACGAAGA 5'
AT5G19440.1     40      58
cinnamyl-alcohol dehydrogenase - like protein

sRNA_AGO1_Solexa_Mi2008_3_17070_hit1
5' UAA-AUGGCCAAGUUG-AUGUU
   ||| |||||||||||| | |||
   AUUCUACCGGUUCAACCUCCAA 5'
AT5G19690.1     1215    1236
oligosaccharyl transferase STT3-like protein

sRNA_AGO1_Solexa_Mi2008_4_46322_hit1
5' UUAA-AUGGCCAAGUUG-AUGUU
   |||| |||||||||||| | |||
   AAUUCUACCGGUUCAACCUCCAA 5'
AT5G19690.1     1215    1237
oligosaccharyl transferase STT3-like protein

sRNA_AGO1_Solexa_Mi2008_1_40533_hit1
5' UGGAGGAUGAGAUUGCAGUGCA
   ||||| |||| ||||||||||
   ACCUC-UACUGUAACGUCACGG 5'
AT5G19690.1     1979    1999
oligosaccharyl transferase STT3-like protein

sRNA_AGO1_Solexa_Mi2008_1_17767_hit1
5' UAAGAAGUGAUAUACUGGAU
   ||||| ||||||||||||||
   AUUCU-CACUAUAUGACCUA 5'
AT5G19740.1     1519    1537
peptidase-like protein

sRNA_AGO1_Solexa_Mi2008_4_13579_hit2
5' GACAAAGUGAAGGGUU-UGGU
   ||||||||||||| || ||||
   CUGUUUCACUUCC-AAGACCA 5'
AT5G20020.1     614     633
small Ras-like GTP-binding protein

sRNA_AGO1_Solexa_Mi2008_1_8382_hit2
5' CGACAAAGUGAAGGGUU-UGGU
    ||||||||||||| || ||||
   CCUGUUUCACUUCC-AAGACCA 5'
AT5G20020.1     614     634
small Ras-like GTP-binding protein

sRNA_AGO1_Solexa_Mi2008_1_13578_hit2
5' GACAAAGUGAAGGGUU-UGG
   ||||||||||||| || |||
   CUGUUUCACUUCC-AAGACC 5'
AT5G20020.1     615     633
small Ras-like GTP-binding protein

sRNA_AGO1_Solexa_Mi2008_1_4723_hit2
5' CAAGCAUUUGUGGUCCAGUGGU
    ||| ||| ||||||||||||
   AUUCAUAA-CACCAGGUCACCA 5'
AT5G20230.1     415     435
blue copper binding protein (bcb)
```

```
sRNA_AGO1_Solexa_Mi2008_73_36978_hit1
5' UGAUAGGUGUUGUGGUCUCCC
   ||| ||||||||||||| ||
   ACU-UCCACAACACCAG-GGU 5'
AT5G20230.1     591     609
blue copper binding protein (bcb)

sRNA_AGO1_Solexa_Mi2008_9_11566_hit1
5' CUGAUAGGUGUUGUGGUCUCCC
   |||| ||||||||||||| ||
   GACU-UCCACAACACCAG-GGU 5'
AT5G20230.1     591     610
blue copper binding protein (bcb)

sRNA_AGO1_Solexa_Mi2008_32_11564_hit1
5' CUGAUAGGUGUUGUGGUCUC
   |||| |||||||||||||:|
   GACU-UCCACAACACCAGGG 5'
AT5G20230.1     592     610
blue copper binding protein (bcb)

sRNA_AGO1_Solexa_Mi2008_62_11565_hit1
5' CUGAUAGGUGUUGUGGUCUCC
   |||| ||||||||||||| ||
   GACU-UCCACAACACCAG-GG 5'
AT5G20230.1     592     610
blue copper binding protein (bcb)

sRNA_AGO1_Solexa_Mi2008_3_39344_hit1
5' UGCUGAUAGGUGUUGUGGUC
   :||||| |||||||||||||
   GCGACU-UCCACAACACCAG 5'
AT5G20230.1     594     612
blue copper binding protein (bcb)

sRNA_AGO1_Solexa_Mi2008_1_14342_hit1
5' GAUGGUUUUGAUUAAUGCU
    ||| ||||||||||| ||
   AUAC-AAAACUAAUUA-GA 5'
AT5G20240.1     7       23
PI protein (PISTILLATA)

sRNA_AGO1_Solexa_Mi2008_11_6237_hit1
5' CAGCAACACCAGAAGACGAG
    ||||||||||||||| |||
   UUCGUUGUGGUCUUCU-CUC 5'
AT5G20320.1     1531    1549
CAF-like protein

sRNA_AGO1_Solexa_Mi2008_6_7327_hit1
5' CAUGGUGUUGAAAAAGGU-UCC
   |||||||| ||||||||| ||
   GUACCACAUCUUUUUCCACAGC 5'
AT5G20320.1     3601    3622
CAF-like protein

sRNA_AGO1_Solexa_Mi2008_4_53237_hit1
5' UUGGUUUCAGUUUCGGGAUA-AC
    ||||||||||||||||| | ||
   CACCAAAGUCAAAGCCCU-UCUG 5'
AT5G20320.1     4240    4261
CAF-like protein
```

```
sRNA_AGO1_Solexa_Mi2008_2_43380_hit1
5' UGGUGAUGAUUGUGU-GUUGC
   ||||:|||||||||| |||||
   ACCAUUACUAACACAACAACG 5'
AT5G21105.1     639     659

sRNA_AGO1_Solexa_Mi2008_1_40185_hit1
5' UGGAGAAGACGCUAAUGGUGG
   |||||||||||| |||| ||:
   ACCUCUUCUGCG-UUAC-ACU 5'
AT5G21160.1     110     128
unknown protein

sRNA_AGO1_Solexa_Mi2008_2_830_hit1
5' AAGAUGGAGAAGAUACGCAA
    |||||||||||  ||||||
   CUCUACCUCUUC--UGCGUU 5'
AT5G21160.1     115     132
unknown protein

sRNA_AGO1_Solexa_Mi2008_1_47294_hit1
5' UUAGAAGGCAUCAGAUGGAGA
   || ||||| ||||||||||||
   AA-CUUCC-UAGUCUACCUCU 5'
AT5G21160.1     123     141
unknown protein

sRNA_AGO1_Solexa_Mi2008_1_4285_hit1
5' CAAAUUCAGAGUGAC-GCUU
    |||||||||||||  ||||
   AUUUAAGUCUCACUAACGAA 5'
AT5G21160.1     2814    2833
unknown protein

sRNA_AGO1_Solexa_Mi2008_1_45719_hit1
5' UGUGUGUGUGUGU-UGGUGUGU
   ||||||||||||| | |||| |
   ACACACACACACAUA-CACAAA 5'
AT5G22570.1     42      62
WRKY transcription factor 38 (WRKY38)

sRNA_AGO1_Solexa_Mi2008_26_5155_hit1
5' CA-AUGGAGCUCUGAAAUGGUU
   || ||:||||||||||||| ||
   GUCUAUCUCGAGACUUUAC-AA 5'
AT5G22690.1     2274    2294
disease resistance protein-like

sRNA_AGO1_Solexa_Mi2008_1_45919_hit1
5' UGUUCUCUCGUGUGG-UUUGGU
     ||||||||||||| ||||||
   CAAAGAGAGCACACCCAAACCA 5'
AT5G22940.1     138     159
unknown protein

sRNA_AGO1_Solexa_Mi2008_1_174_hit2
5' AA-AAGAAGAAGAUAAAGCAUAU
   || ||||||||||||||| || |
   UUCUUCUUCUUCUAUUUC-UACA 5'
AT5G22940.1     366     387
unknown protein
```

```
sRNA_AGO1_Solexa_Mi2008_7_353_hit2
5' AAAGAAGAAGAUAAAGCAU
    ||||||||||||||| ||
   GUUCUUCUUCUAUUUC-UA 5'
AT5G22940.1     368     385
unknown protein

sRNA_AGO1_Solexa_Mi2008_1_172_hit2
5' AA-AAGAAGAAGAUAAAGCA
   || |||||||||||||||
   UUCUUCUUCUUCUAUUUCUA 5'
AT5G22940.1     368     387
unknown protein

sRNA_AGO1_Solexa_Mi2008_1_173_hit2
5' AA-AAGAAGAAGAUAAAGCAU
   || ||||||||||||||| ||
   UUCUUCUUCUUCUAUUUC-UA 5'
AT5G22940.1     368     387
unknown protein

sRNA_AGO1_Solexa_Mi2008_1_30783_hit1
5' UCGAGGGAGAAGCUA-GAUGUG
   |||| ||||||| || ||||||
   AGCUGCCUCUUC-AUUCUACAC 5'
AT5G22940.1     439     459
unknown protein

sRNA_AGO1_Solexa_Mi2008_2_255_hit1
5' AAA-AUAGUGUGGGAA-UGUUA
   ||| |||||||||||| ||||
   UUUCUAUCACACCCUUAACAAA 5'
AT5G23010.1     5       26
2-isopropylmalate synthase-like; homocitrate synthase-like

sRNA_AGO1_Solexa_Mi2008_9_13465_hit1
5' GAAGAUGG-GUGGGAAUUGUUU
    |||||:| ||||||||||||
   AUUCUAUCACACCCUUAACAAA 5'
AT5G23010.1     5       26
2-isopropylmalate synthase-like; homocitrate synthase-like

sRNA_AGO1_Solexa_Mi2008_2_4771_hit1
5' CAAGCUCUUCAGGUA-UUUCU
   |||||||| |||||| |||||
   GUUCGAGAUGUCCAUAAAAGA 5'
AT5G23405.1     175     195

sRNA_AGO1_Solexa_Mi2008_2_4771_hit1
5' CAAGCUCUUCAGGUAUUU-CU
   |||||||| ||||||||| ||
   GUUCGAGAUGUCCAUAAAAGA 5'
AT5G23405.2     182     202

sRNA_AGO1_Solexa_Mi2008_1_3079_hit472
5' AUGAUGAUGAUGAUGAUGAUGA
   |||||||||||||| || |||
   UACUACUACUACUAAUAGUACC 5'
AT5G24030.1     629     650
unknown protein

sRNA_AGO1_Solexa_Mi2008_9_14254_hit8
5' GAUGAUGAUGAUGAUGAUCUU
```

```
   ||||||||||||||| ||| |
   CUACUACUACUACUAAUAGUA 5'
AT5G24030.1     631     651
unknown protein

sRNA_AGO1_Solexa_Mi2008_1_36323_hit13
5' UGAGGAUGAUGAUGAUGAUGA
   |||:||||||||||||||| |
   ACUUCUACUACUACUACUAAU 5'
AT5G24030.1     635     655
unknown protein

sRNA_AGO1_Solexa_Mi2008_1_3079_hit472
5' AUGAUGAUGAUGAUGAUGAUGA
   |||| ||||||||||||||| |
   UACUUCUACUACUACUACUAAU 5'
AT5G24030.1     635     656
unknown protein

sRNA_AGO1_Solexa_Mi2008_1_37834_hit1
5' UGCAAUAUAGAUGAUGAUGAUU
   || || || |||||||||||||
   AC-UUCUA-CUACUACUACUAA 5'
AT5G24030.1     637     656
unknown protein

sRNA_AGO1_Solexa_Mi2008_1_3079_hit472
5' AUGAUGAUGAUGAUGAUGAUGA
    :||||| ||||||||||||||
   AGCUACUUCUACUACUACUACU 5'
AT5G24030.1     638     659
unknown protein

sRNA_AGO1_Solexa_Mi2008_1_45039_hit3
5' UGU-GAUGAUGAUGAUGAUGAUGA
   ||| ||||| ||||||||||||||
   ACAGCUACUUCUACUACUACUACU 5'
AT5G24030.1     639     662
unknown protein

sRNA_AGO1_Solexa_Mi2008_12_14346_hit675
5' GAUGUCAUGUGUAUG-A-UUGA
   |||||||||||| || | ||||
   CUACAGUACACAGACUUAAACU 5'
AT5G24040.1     122     143
putative protein

sRNA_AGO1_Solexa_Mi2008_1_38851_hit1
5' UGCGAUUGAGAGCAACAAGA
   |||||||||||||| |||:
   ACGCUAACUCUCGUAGUUUC 5'
AT5G24160.1     1594    1613
squalene monooxygenase 1,2 (squalene epoxidase 1,2) (se 1,2) (sp|O65402)

sRNA_AGO1_Solexa_Mi2008_4_15252_hit1
5' GGGUACCAGAGGAAAUAUAGUA
   :|||||| |||||| |||||||
   UCCAUGGACUCCUUGAUAUCAU 5'
AT5G24160.1     905     926
squalene monooxygenase 1,2 (squalene epoxidase 1,2) (se 1,2) (sp|O65402)

sRNA_AGO1_Solexa_Mi2008_1_19693_hit1
5' UACCAAUGACGCUCGGUGGUAC
```

```
   ||||||||||||||||||||||
   AUGGUUACUGCGAGCCACCAUG 5'
AT5G24200.1     259     280
putative protein

sRNA_AGO1_Solexa_Mi2008_2_18780_hit1
5' UAAUGCGUCCCUAAGUUGUUGGU
   |||||||||||||||||||||||
   AUUACGCAGGGAUUCAACAACCA 5'
AT5G24200.1     390     412
putative protein

sRNA_AGO1_Solexa_Mi2008_1_40595_hit1
5' UGGAGGUACUAUCGAGUUGGGAGU
   ||||||||||||||||||||||||
   ACCUCCAUGAUAGCUCAACCCUCA 5'
AT5G24200.1     464     487
putative protein

sRNA_AGO1_Solexa_Mi2008_1_40594_hit1
5' UGGAGGUACUAUCGAGUUGGGA
   ||||||||||||||||||||||
   ACCUCCAUGAUAGCUCAACCCU 5'
AT5G24200.1     466     487
putative protein

sRNA_AGO1_Solexa_Mi2008_1_17535_hit1
5' UAACGUGGAGGUACUAUCGAGU
   ||||||||||||||||||||||
   AUUGCACCUCCAUGAUAGCUCA 5'
AT5G24200.1     471     492
putative protein

sRNA_AGO1_Solexa_Mi2008_2_42234_hit1
5' UGGGCUUAAGAUCGUUCCACGU
   ||||||||||||||||||||||
   ACCCGAAUUCUAGCAAGGUGCA 5'
AT5G24200.1     505     526
putative protein

sRNA_AGO1_Solexa_Mi2008_1_2672_hit1
5' AUAGUUUGUGGGCUUAAGAUC
   |||||||||||||||||||||
   UAUCAAACACCCGAAUUCUAG 5'
AT5G24200.1     514     534
putative protein

sRNA_AGO1_Solexa_Mi2008_9_26051_hit1
5' UAUGUGGCCACAAAAUAUUGCA
   ||||||||||||||||||||||
   AUACACCGGUGUUUUAUAACGU 5'
AT5G24200.1     618     639
putative protein

sRNA_AGO1_Solexa_Mi2008_1_41017_hit1
5' UGGAUUGAAGAUGUAAGCCUCGAC
   ||||||||||||||||||||||||
   ACCUAACUUCUACAUUCGGAGCUG 5'
AT5G24200.1     731     754
putative protein

sRNA_AGO1_Solexa_Mi2008_3_24953_hit3
5' UAUCCA-UUCUAUAUACUAUGU
```

```
   |||| | || ||||||||||||
   AUAG-UGAACAUAUAUGAUACA 5'
AT5G24420.1     1178    1198
6-phosphogluconolactonase-like protein

sRNA_AGO1_Solexa_Mi2008_41_6691_hit3
5' CAGUCAUAGAUAGUCUCUGCAA
    |||| |||||||||||| |||
   CUCAGAAUCUAUCAGAGAGGUU 5'
AT5G24530.1     496     517
flavanone 3-hydroxylase-like protein

sRNA_AGO1_Solexa_Mi2008_4_48531_hit1
5' UUCAAGGUGAUAUCUAGACAAUGU
   |||||||||||||||| | ||||
   AAGUUCCACUAUAGAU-U-UUACU 5'
AT5G24590.2     135     156
NAC2-like protein

sRNA_AGO1_Solexa_Mi2008_1_36065_hit1
5' UGAGCAUCCACAGUUGGUCCU
   |||||||||||||||||||||
   ACUCGUAGGUGUCAACCAGGA 5'
AT5G24810.1     1665    1685
unknown protein

sRNA_AGO1_Solexa_Mi2008_1_34056_hit1
5' UGAAAAACGGAUGAGUGAUUUAAG
   ||||||||||||||||||||||||
   ACUUUUUGCCUACUCACUAAAUUC 5'
AT5G25110.1     145     168
serine/threonine protein kinase-like protein

sRNA_AGO1_Solexa_Mi2008_1_23143_hit1
5' UAGGGGCCGAUUGAUAGCGACUGU
   ||||||||||||| ||:|||||||
   AUCCCCGGCUAACCAUUGCUGACA 5'
AT5G25110.1     174     197
serine/threonine protein kinase-like protein

sRNA_AGO1_Solexa_Mi2008_1_23146_hit1
5' UAGGGGCUGAUUGGUAGCGAGUGU
   |||||||:||||||||:||| |||
   AUCCCCGGCUAACCAUUGCUGACA 5'
AT5G25110.1     174     197
serine/threonine protein kinase-like protein

sRNA_AGO1_Solexa_Mi2008_17_8110_hit2
5' CCGUGGGUU-GUUUUAUAAGAA
    ||||| || ||||||||||||
   AGCACCAAAUCAAAAUAUUCUU 5'
AT5G25110.1     17      38
serine/threonine protein kinase-like protein

sRNA_AGO1_Solexa_Mi2008_1_4569_hit2
5' CAAGAAGAAGGUUGG-UUAGUU
   ||||||||||| ||| ||||||
   GUUCUUCUUCC-ACCAAAUCAA 5'
AT5G25110.1     26      46
serine/threonine protein kinase-like protein

sRNA_AGO1_Solexa_Mi2008_5_21867_hit1
5' UAGAUGGGUGUAAAAUGUAAC
```

```
    |||||:|||||||||||||
   CUCUACUCACAUUUUACAUUA 5'
AT5G25460.1     28      48
Unknown protein

sRNA_AGO1_Solexa_Mi2008_1_2_hit38
5' AAAA-AAAAAAAAAAACCAU
   |||| ||||||||||| |||
   UUUUAUUUUUUUUUUU-GUA 5'
AT5G25610.1     1436    1454
dehydration-induced protein RD22

sRNA_AGO1_Solexa_Mi2008_4_43921_hit1
5' UGUAAA-GGGUGUUAGUAGAAAG
   |||||| |||| |||||||||||
   ACAUUUUCCCAAAAUCAUCUUUC 5'
AT5G25610.1     22      44
dehydration-induced protein RD22

sRNA_AGO1_Solexa_Mi2008_1_50782_hit1
5' UUGAGG-GUUGGAAUAGACAUGG
   ||| || ||||||||||| ||||
   AAC-CCUCAACCUUAUCUUUACC 5'
AT5G25610.1     93      114
dehydration-induced protein RD22

sRNA_AGO1_Solexa_Mi2008_8_50781_hit1
5' UUGAGG-GUUGGAAUAGACAUG
   ||| || ||||||||||| |||
   AAC-CCUCAACCUUAUCUUUAC 5'
AT5G25610.1     94      114
dehydration-induced protein RD22

sRNA_AGO1_Solexa_Mi2008_1_1627_hit6
5' ACGGAAUAGCGAGUUGGUC
   |||||||||||||| | ||
   UGCCUUAUCGCUCA-C-AG 5'
AT5G25770.2     1034    1050
unknown protein

sRNA_AGO1_Solexa_Mi2008_1_1627_hit6
5' ACGGAAUAGCGAGUUGGUC
   ||||||||||||| | |||
   UGCCUUAUCGCUC-A-CAG 5'
AT5G25770.3     1043    1059
unknown protein

sRNA_AGO1_Solexa_Mi2008_1_1627_hit6
5' ACGGAAUAGCGAGUUGGUC
   |||||||||||||| | ||
   UGCCUUAUCGCUCA-C-AG 5'
AT5G25770.1     1277    1293
unknown protein

sRNA_AGO1_Solexa_Mi2008_1_33976_hit1
5' UCUUGGUGGACAUG-AGUGGAC
   |||||||||||||: ||||||
   AGAACCACCUGUAUAUCACCUA 5'
AT5G26000.2     1672    1693
myrosinase precursor

sRNA_AGO1_Solexa_Mi2008_1_16880_hit3
5' UAAAGGUC-U-AUCUAUGGAGCA
```

```
   ||||| || | ||||||||||||
   AUUUCGAGGAAUAGAUACCUCGU 5'
AT5G26000.2     1720    1742
myrosinase precursor

sRNA_AGO1_Solexa_Mi2008_1_23310_hit418
5' UAGGG-UUUA-GGGUUUAGGGUUU
   ||:|| |||| |||||||||||||
   AUUCCUAAAUACCCAAAUCCCAAA 5'
AT5G26030.2     119     142
ferrochelatase-I

sRNA_AGO1_Solexa_Mi2008_13_27378_hit1
5' UCAAUAGAUUGGACUAUGUAU
   ||||||||||||||||:|||
   AGUUAUCUAACCUGAUAUAUA 5'
AT5G26030.1     1674    1694
ferrochelatase-I

sRNA_AGO1_Solexa_Mi2008_2_27377_hit1
5' UCAAUAGAUUGGACUAUGUA
   ||||||||||||||||:||
   AGUUAUCUAACCUGAUAUAU 5'
AT5G26030.1     1675    1694
ferrochelatase-I

sRNA_AGO1_Solexa_Mi2008_2_55069_hit1
5' UUUGAAGA-UUUCAGGACCACC
   :|| |||| ||||||||||||
   GAA-UUCUCAAAGUCCUGGUGG 5'
AT5G26030.2     507     527
ferrochelatase-I

sRNA_AGO1_Solexa_Mi2008_9_6886_hit1
5' CAUAGAGU-UGGUUUUGGGUCU
    |||| || ||||||||||||
   CUAUCCCAGACCAAAACCCAGA 5'
AT5G26690.1     336     357
unknown protein

sRNA_AGO1_Solexa_Mi2008_22_7409_hit1
5' CA-UGUUGACCAAUUCAAGA
   || |||||||||||| ||||
   GUGACAACUGGUUAAAUUCU 5'
AT5G26770.2     110     129
unknown protein

sRNA_AGO1_Solexa_Mi2008_2_131_hit1
5' AAAA-AUAUAGUUCAGGGUU
    ||| ||| ||||||||||
   GUUUGUAUCUCAAGUCCCAA 5'
AT5G26770.3     128     147
unknown protein

sRNA_AGO1_Solexa_Mi2008_2_131_hit1
5' AAAA-AUAUAGUUCAGGGUU
    ||| ||| ||||||||||
   GUUUGUAUCUCAAGUCCCAA 5'
AT5G26770.2     29      48
unknown protein

sRNA_AGO1_Solexa_Mi2008_1_6075_hit1
5' CAGAGGCGAAGUGA-AAA-UGCU
```

```
   |||||||||||||| ||| || |
   GUCUCCGCUUCACUCUUUGACCA 5'
AT5G26850.1     43      65
unknown protein

sRNA_AGO1_Solexa_Mi2008_2_20904_hit1
5' UAGA-A-GAUGAUUGAUUGUU
   |||| | ||||||||||:|||
   AUCUCUACUACUAACUAGCAA 5'
AT5G27600.1     110     130
long chain acyl-CoA synthetase 7 (LACS7)

sRNA_AGO1_Solexa_Mi2008_1_3_hit25
5' AAA-A-AAAAAAAAAGAAAGA
   ||| | ||||||||||||| |
   UUUAUGUUUUUUUUUCUUUGU 5'
AT5G27600.1     2166    2186
long chain acyl-CoA synthetase 7 (LACS7)

sRNA_AGO1_Solexa_Mi2008_8_53727_hit2
5' UUGUUAAAGAAGAUGGAG-AAA
   ||||| |||||||| ||| |||
   AACAACUUCUUCUA-CUCGUUU 5'
AT5G27760.1     34      54
unknown protein

sRNA_AGO1_Solexa_Mi2008_3_13907_hit1
5' GAGAAGAGAUAGA-AUAGAAU
   ||||||||||||| || |||
   CUCUUCUCUAUCUCUACCUUU 5'
AT5G33290.1     326     346
unknown protein

sRNA_AGO1_Solexa_Mi2008_13_13906_hit2
5' GAGAAGAGAUAGA-AUAGAA
   ||||||||||||| || |||
   CUCUUCUCUAUCUCUACCUU 5'
AT5G33290.1     327     346
unknown protein

sRNA_AGO1_Solexa_Mi2008_4_35785_hit2
5' UGAGAAGAGAUAGA-AUAGAA
   :||||||||||||| || |||
   GCUCUUCUCUAUCUCUACCUU 5'
AT5G33290.1     327     347
unknown protein

sRNA_AGO1_Solexa_Mi2008_40_50469_hit1
5' UUGAGAAGAGAUAGA-AUAGAA
   |:||||||||||||| || |||
   AGCUCUUCUCUAUCUCUACCUU 5'
AT5G33290.1     327     348
unknown protein

sRNA_AGO1_Solexa_Mi2008_1_2422_hit4
5' AUAAUAUUCAGAUAACUCGGU
   |||||||||||| |||| ||
   UAUUAUAAGUCU-UUGA-CCU 5'
AT5G33370.2     459     477
unknown protein

sRNA_AGO1_Solexa_Mi2008_1_23511_hit1
5' UAGGUG-UGAUGAUUGGUUGUA
```

```
   :||||| |||||||||||:|||
   GUCCACCACUACUAACCAGCAU 5'
AT5G35090.1     200     221
unknown protein

sRNA_AGO1_Solexa_Mi2008_1_43578_hit1
5' UGGUGGUGGUGAUGA-UGUGUC
    |||||||||||||| || |||
   UCCACCACCACUACUAAC-CAG 5'
AT5G35090.1     203     223
unknown protein

sRNA_AGO1_Solexa_Mi2008_7_20716_hit3
5' UAC-UUUAGAGUGGACUUCU
   ||| ||||||||||| ||||
   AUGCAAAUCUCACCUAAAGA 5'
AT5G35180.3     1332    1351
unknown protein

sRNA_AGO1_Solexa_Mi2008_2_7736_hit1
5' CCAGAAAAGAAGAAACAAU
     ||||||||||||| |||
   AAUCUUUUCUUCUUU-UUA 5'
AT5G35180.1     2561    2578
unknown protein

sRNA_AGO1_Solexa_Mi2008_2_7736_hit1
5' CCAGAAAAGAAGAAACAAU
     ||||||||||||| |||
   AAUCUUUUCUUCUUU-UUA 5'
AT5G35180.2     2649    2666
unknown protein

sRNA_AGO1_Solexa_Mi2008_2_40489_hit1
5' UGGAGGACAAGCUAGUGAUCGU
   |||||||||||| |:||||||
   ACCUCCUGUUCGUUUACUAGCU 5'
AT5G36220.1     847     868
cytochrome P450 monooxygenase (CYP81D1 )

sRNA_AGO1_Solexa_Mi2008_4_16065_hit2
5' GUGUU-GAGACAUGAGAAGU
   :|||| ||||||||||||:|
   UACAAACUCUGUACUCUUUA 5'
AT5G36890.2     1103    1122
beta-glucosidase -like protein

sRNA_AGO1_Solexa_Mi2008_4_16065_hit2
5' GUGUU-GAGACAUGAGAAGU
   :|||| ||||||||||||:|
   UACAAACUCUGUACUCUUUA 5'
AT5G36890.1     1104    1123
beta-glucosidase -like protein

sRNA_AGO1_Solexa_Mi2008_1_27137_hit1
5' UCAAGCAUAUCACUGAGUAGU
   |||||||||||| |||:|||
   AGUUCGUAUAGU-ACUUAUCU 5'
AT5G36890.2     1337    1356
beta-glucosidase -like protein

sRNA_AGO1_Solexa_Mi2008_1_27137_hit1
5' UCAAGCAUAUCACUGAGUAGU
```

```
   |||||||||||| |||:|||
   AGUUCGUAUAGU-ACUUAUCU 5'
AT5G36890.1     1338    1357
beta-glucosidase -like protein

sRNA_AGO1_Solexa_Mi2008_1_11678_hit1
5' CUGCAACUGUUGAAGAU--CCA
   |||| ||||||||||||  |||
   GACGGUGACAACUUCUACCGGU 5'
AT5G37020.2     1749    1770
auxin response factor 8 (ARF8)

sRNA_AGO1_Solexa_Mi2008_1_38941_hit1
5' UGCGGAUUUCCGGGUUCUAGAG
   ||||||||||||||||||||||
   ACGCCUAAAGGCCCAAGAUCUC 5'
AT5G37020.2     2005    2026
auxin response factor 8 (ARF8)

sRNA_AGO1_Solexa_Mi2008_1_34515_hit1
5' UGAAGAAGCAAAUG-GGGUA
   |||||||||||| | ||| |
   ACUUCUUCGUUU-CGCCCCU 5'
AT5G37020.2     2085    2103
auxin response factor 8 (ARF8)

sRNA_AGO1_Solexa_Mi2008_4_2102_hit1
5' AGCUGCCAGCAUGAUCUAU
   |||||||||| |||||||
   UCGACGGUCGGACUAGAUU 5'
AT5G37020.2     2368    2386
auxin response factor 8 (ARF8)

sRNA_AGO1_Solexa_Mi2008_4_13484_hit1
5' GAAGCUGCCAGCAUGAUCUAU
    ||||||||||| |||||||
   GUUCGACGGUCGGACUAGAUU 5'
AT5G37020.2     2368    2388
auxin response factor 8 (ARF8)

sRNA_AGO1_Solexa_Mi2008_61_34692_hit1
5' UGAAGCUGCCAGCAUGAUCUAA
     ||||||||||| ||||||||
   UGUUCGACGGUCGGACUAGAUU 5'
AT5G37020.2     2368    2389
auxin response factor 8 (ARF8)

sRNA_AGO1_Solexa_Mi2008_13_873_hit2
5' AAGCUGCCAGCAUGAUCUA
   ||||||||||| |||||||
   UUCGACGGUCGGACUAGAU 5'
AT5G37020.2     2369    2387
auxin response factor 8 (ARF8)

sRNA_AGO1_Solexa_Mi2008_1_874_hit1
5' AAGCUGCCAGCAUGAUCUG
   ||||||||||| ||||||:
   UUCGACGGUCGGACUAGAU 5'
AT5G37020.2     2369    2387
auxin response factor 8 (ARF8)

sRNA_AGO1_Solexa_Mi2008_1_4776_hit1
5' CAAGCUGCCAGCCUGAUCUA
```

```
   ||||||||||||||||||||
   GUUCGACGGUCGGACUAGAU 5'
AT5G37020.2     2369    2388
auxin response factor 8 (ARF8)

sRNA_AGO1_Solexa_Mi2008_1519_13483_hit2
5' GAAGCUGCCAGCAUGAUCUA
    ||||||||||| |||||||
   GUUCGACGGUCGGACUAGAU 5'
AT5G37020.2     2369    2388
auxin response factor 8 (ARF8)

sRNA_AGO1_Solexa_Mi2008_3_13485_hit1
5' GAAGCUGCCAGCAUGAUCUG
    ||||||||||| ||||||:
   GUUCGACGGUCGGACUAGAU 5'
AT5G37020.2     2369    2388
auxin response factor 8 (ARF8)

sRNA_AGO1_Solexa_Mi2008_344873_34691_hit2
5' UGAAGCUGCCAGCAUGAUCUA
     ||||||||||| |||||||
   UGUUCGACGGUCGGACUAGAU 5'
AT5G37020.2     2369    2389
auxin response factor 8 (ARF8)

sRNA_AGO1_Solexa_Mi2008_54_13482_hit3
5' GAAGCUGCCAGCAUGAUCU
    ||||||||||| ||||||
   GUUCGACGGUCGGACUAGA 5'
AT5G37020.2     2370    2388
auxin response factor 8 (ARF8)

sRNA_AGO1_Solexa_Mi2008_7845_34690_hit3
5' UGAAGCUGCCAGCAUGAUCU
     ||||||||||| ||||||
   UGUUCGACGGUCGGACUAGA 5'
AT5G37020.2     2370    2389
auxin response factor 8 (ARF8)

sRNA_AGO1_Solexa_Mi2008_17_46569_hit1
5' UUAAGCUGCCAGCAUGAUCU
    :||||||||||| ||||||
   UGUUCGACGGUCGGACUAGA 5'
AT5G37020.2     2370    2389
auxin response factor 8 (ARF8)

sRNA_AGO1_Solexa_Mi2008_6340_34689_hit3
5' UGAAGCUGCCAGCAUGAUC
     ||||||||||| |||||
   UGUUCGACGGUCGGACUAG 5'
AT5G37020.2     2371    2389
auxin response factor 8 (ARF8)

sRNA_AGO1_Solexa_Mi2008_8_46568_hit1
5' UUAAGCUGCCAGCAUGAUC
    :||||||||||| |||||
   UGUUCGACGGUCGGACUAG 5'
AT5G37020.2     2371    2389
auxin response factor 8 (ARF8)

sRNA_AGO1_Solexa_Mi2008_1_47627_hit1
5' UUAGCUAGCAGAAGCAUGUGCA
```

```
   |||| | ||||||||||||||
   AAUCAA-CGUCUUCGUACACGU 5'
AT5G37020.2     879     899
auxin response factor 8 (ARF8)

sRNA_AGO1_Solexa_Mi2008_1_51898_hit5
5' UUGCUUUCUUGAUGGAUACUCA
   ||||||||||||| || |||
   AACGAAAGAACUAC-UA-GAGC 5'
AT5G37600.1     974     993
glutamate--ammonia ligase

sRNA_AGO1_Solexa_Mi2008_1_7253_hit1
5' CAUGGAUGGUCUGAUGGAUCGG
   ||||||:||||||||||| |||
   GUACCUGCCAGACUACCU-GCC 5'
AT5G38250.1     1575    1595
receptor serine/threonine protein kinase - like

sRNA_AGO1_Solexa_Mi2008_2_14498_hit1
5' GCCAAAGAAAUGG--ACGAGG
   |||||||||||||  ||||||
   CGGUUUCUUUACCGCUGCUCC 5'
AT5G38250.1     421     441
receptor serine/threonine protein kinase - like

sRNA_AGO1_Solexa_Mi2008_1_31483_hit3
5' UCGCCGGUU-CGAGUGACGUU
   ||||||||| || |||||| |
   AGCGGCCAAGGC-CACUGCCA 5'
AT5G38880.1     98      117
putative protein

sRNA_AGO1_Solexa_Mi2008_1_13420_hit2
5' GAAGAAGAAGAAGACU-CUU
   |||||||||||||| | |||
   CUUCUUCUUCUUCUCACGAA 5'
AT5G38900.1     895     914
frnE protein - like

sRNA_AGO1_Solexa_Mi2008_13_4545_hit1
5' CAAGAAAGAUGGUUGUCGUU
     |||||| ||||||||||
   ACUCUUUCAACCAACAGCAA 5'
AT5G39050.1     534     553
Anthocyanin acyltransferase - like protein

sRNA_AGO1_Solexa_Mi2008_1_13420_hit2
5' GAAGAAGAAGAAGACUCUU
   || |||||||||||||||
   CUCCUUCUUCUUCUGAGAA 5'
AT5G39050.1     830     848
Anthocyanin acyltransferase - like protein

sRNA_AGO1_Solexa_Mi2008_1_4090_hit1
5' CA-AAGAGUAGACAAUCAUCAA
   || |||||||:||||||| |||
   GUAUUCUCAUUUGUUAGU-GUU 5'
AT5G39050.1     888     908
Anthocyanin acyltransferase - like protein

sRNA_AGO1_Solexa_Mi2008_1_23339_hit9
5' UAGGUACAACUCGGUUGGAGGG
```

```
     |||||||||||| |||||||
   CGCCAUGUUGAGCC-ACCUCCC 5'
AT5G39080.1     390     410
acyltransferase -like protein

sRNA_AGO1_Solexa_Mi2008_13_4545_hit1
5' CAAGAAAGAUGGUUGUCGUU
     ||||| ||||||||||||
   ACUCUUUGUACCAACAGCAA 5'
AT5G39080.1     532     551
acyltransferase -like protein

sRNA_AGO1_Solexa_Mi2008_1_54777_hit1
5' UUUCCAAAAGCUGCCGGU-UCUG
   |||||||||||| ||||| |||
   AAAGGUUUUCGA-GGCCAUAGAA 5'
AT5G39090.1     1090    1111
acyltransferase -like  protein

sRNA_AGO1_Solexa_Mi2008_3_40895_hit1
5' UGGAUGAUGAGAGAGA-GAU
   :|||||| |||||||| |||
   GCCUACUCCUCUCUCUCCUA 5'
AT5G39090.1     207     226
acyltransferase -like  protein

sRNA_AGO1_Solexa_Mi2008_1_36103_hit1
5' UGAGCCCAAGA-UUGACAACAC
   ||||||||||| |||| || ||
   ACUCGGGUUCUAAACUCUU-UG 5'
AT5G39090.1     531     551
acyltransferase -like  protein

sRNA_AGO1_Solexa_Mi2008_1_36793_hit1
5' UGAGUGACGCCGACGAGUUAG
   |||||||||||||||||||||
   ACUCACUGCGGCUGCUCAAUC 5'
AT5G39090.1     61      81
acyltransferase -like  protein

sRNA_AGO1_Solexa_Mi2008_1_13420_hit2
5' GAAGAA-GAAGAAGACUCUU
   ||| || ||||||||||||:
   CUU-UUGCUUCUUCUGAGAG 5'
AT5G39090.1     785     803
acyltransferase -like  protein

sRNA_AGO1_Solexa_Mi2008_1_17319_hit1
5' UAACCAAAGCAAAUUGAUCGGA
     |||||||||||| |||||||
   UGUGGUUUCGUUUA-CUAGCCU 5'
AT5G39580.2     92      112
peroxidase ATP24a

sRNA_AGO1_Solexa_Mi2008_2_19236_hit1
5' UACACAACAUCUGAUGGACUA
    |||||| ||||||||||||
   UUGUGUUCUAGACUACCUGAA 5'
AT5G40450.2     1334    1354
unknown protein

sRNA_AGO1_Solexa_Mi2008_6_10413_hit1
5' CUAGUUCGUCGAUAUGUUG
```

```
   || ||||||||||||| ||
   GA-CAAGCAGCUAUA-AAG 5'
AT5G40450.2     2425    2441
unknown protein

sRNA_AGO1_Solexa_Mi2008_1_32975_hit1
5' UCUAGUUCGUCGAUAUGUU
   ||| |||||||||||| ||
   AGA-CAAGCAGCUAUA-AA 5'
AT5G40450.2     2426    2442
unknown protein

sRNA_AGO1_Solexa_Mi2008_1_39819_hit1
5' UGGAAUAAUUUCAGGAUAGGU
   |||||||||||| || ||||
   ACCUUAUUAAAG-CC-AUCCC 5'
AT5G40450.2     4704    4722
unknown protein

sRNA_AGO1_Solexa_Mi2008_1_27991_hit1
5' UCAGAAACCCUAAUUUCGUGGG
   ||| ||||||||||||| |||:
   AGUGUUUGGGAUUAAAGAACCU 5'
AT5G40480.1     1489    1510
nuclear pore protein -like

sRNA_AGO1_Solexa_Mi2008_1_36711_hit4
5' UGAGUAGCAAGAGAUGGAGAAA
   |||| ||||||||| |||||||
   ACUC-UCGUUCUCUUCCUCUUU 5'
AT5G40480.1     154     174
nuclear pore protein -like

sRNA_AGO1_Solexa_Mi2008_1_53211_hit1
5' UUGGUUGGUUC-UUGACAGUCA
   ||||||||| | |||||||||
   AACCAACCA-GUAACUGUCAGA 5'
AT5G40480.1     4607    4627
nuclear pore protein -like

sRNA_AGO1_Solexa_Mi2008_5_4358_hit1
5' CAACAGAAGAAUUUCAA-ACAG
   |||||||||| ||| || ||||
   GUUGUCUUCUCAAA-UUGUGUC 5'
AT5G40480.1     983     1003
nuclear pore protein -like

sRNA_AGO1_Solexa_Mi2008_2_156_hit1
5' AAAACCCGGUGGAUAAAAU
   |||||| |:||||||||||
   UUUUGG-CUACCUAUUUUA 5'
AT5G40770.1     1437    1454
prohibitin (gb|AAC49691.1)

sRNA_AGO1_Solexa_Mi2008_1_3_hit25
5' AAAAAAAAAAAAAGAAAGA
   ||||||||||||||| |||
   UUUUUUUUUUUUUCUAUCU 5'
AT5G41400.1     644     662
RING zinc finger protein-like

sRNA_AGO1_Solexa_Mi2008_1_3_hit25
5' AAAAAAAAAAAAAGAAAGA
```

```
   ||||||||||||| |||||
   UUUUUUUUUUUUU-UUUCU 5'
AT5G41400.1     649     666
RING zinc finger protein-like

sRNA_AGO1_Solexa_Mi2008_1_1776_hit10
5' AGAA-AAAAAAAAAAAAAAAAU
   | || ||||||||||||||||
   UAUUGUUUUUUUUUUUUUUUUC 5'
AT5G41400.1     650     671
RING zinc finger protein-like

sRNA_AGO1_Solexa_Mi2008_2_47828_hit1
5' UUAGGGUGUCAAAAUAUGUC-AAA
   ||||:||| ||||||||||| |||
   AAUCUCACUGUUUUAUACAGAUUU 5'
AT5G42100.2     1375    1398
beta-1,3-glucanase - like predicted GPI-anchored protein

sRNA_AGO1_Solexa_Mi2008_1_17833_hit2
5' UAAGAGAGAGAUUGAGAAUAG
   | |||||||||||||||| ||
   AGUCUCUCUCUAACUCUU-UC 5'
AT5G42440.1     13      32
serine/threonine protein kinase-like protein

sRNA_AGO1_Solexa_Mi2008_1_25234_hit1
5' UAUGAACUUGAUGACCCGCCC
   |||||||||||||||||||||
   AUACUUGAACUACUGGGCGGG 5'
AT5G42650.1     1051    1071
allene oxide synthase (emb|CAA73184.1)

sRNA_AGO1_Solexa_Mi2008_1_15261_hit5
5' GGGUCGAGUGAUGUGAUUGAG
   :|| |||||| ||||||||||
   UCC-GCUCACAACACUAACUC 5'
AT5G42650.1     4       23
allene oxide synthase (emb|CAA73184.1)

sRNA_AGO1_Solexa_Mi2008_1_12824_hit1
5' CUUGAUUCUUCUGAUAAUGGU
    |||||||||||| ||| |||
   UAACUAAGAAGACCAUU-CCA 5'
AT5G42830.1     55      74
N-hydroxycinnamoyl/benzoyltransferase-like protein

sRNA_AGO1_Solexa_Mi2008_1_38180_hit1
5' UGCAGUGUACUCUGGUAUGGU
   |||||||||||||||||||||
   ACGUCACAUGAGACCAUACCA 5'
AT5G43450.1     1094    1114
1-aminocyclopropane-1-carboxylate oxidase

sRNA_AGO1_Solexa_Mi2008_1_18828_hit1
5' UAAUGGGUGAGGAACUACUAU
    ||||| ||||||||| ||||
   CUUACCAACUCCUUGA-GAUA 5'
AT5G43450.1     1250    1269
1-aminocyclopropane-1-carboxylate oxidase

sRNA_AGO1_Solexa_Mi2008_5_6056_hit1
5' CAGAGCAUAAACAUAAGCC
```

```
   ||||||||||||| || ||
   GUCUCGUAUUUGU-UU-GG 5'
AT5G43450.1     717     733
1-aminocyclopropane-1-carboxylate oxidase

sRNA_AGO1_Solexa_Mi2008_7_36003_hit1
5' UGAGAUUUUCAAG-UC-GUCUA
   ||||||||||||| |  |||||
   ACUCUAAAAGUUCUAAACAGAU 5'
AT5G45380.1     1488    1509
urea active transporter-like protein

sRNA_AGO1_Solexa_Mi2008_3_7090_hit2
5' CAUCUGUUUUGAUCAUAUCAG
   || |||||||:|||||||||
   GU-GACAAAAUUAGUAUAGUA 5'
AT5G45510.2     1916    1935
unknown protein

sRNA_AGO1_Solexa_Mi2008_1_6039_hit2
5' CAGAGAGUCUGAUAUC-AAGAACA
   ||||||||||| || | |||||||
   GUCUCUCAGACCAUCGUUUCUUGU 5'
AT5G45510.2     348     371
unknown protein

sRNA_AGO1_Solexa_Mi2008_6_44162_hit1
5' UGUAGAUUUCACGUUCUCCUC
   ||| ||||||| |||||||||
   ACA-CUAAAGUUCAAGAGGAG 5'
AT5G45510.2     3493    3512
unknown protein

sRNA_AGO1_Solexa_Mi2008_1_29526_hit2
5' UCCA-GUGAUACUUCUCAAGUC
   |||| | |||||||||||| ||
   AGGUUCUCUAUGAAGAGUU-AG 5'
AT5G45510.2     573     593
unknown protein

sRNA_AGO1_Solexa_Mi2008_1_53286_hit1
5' UUGUAAGUCCUGGAAACCCAAA
   ||||| ||||| ||||||||||
   AACAU-CAGGAGCUUUGGGUUU 5'
AT5G45670.1     352     372
GDSL-motif lipase/hydrolase-like protein

sRNA_AGO1_Solexa_Mi2008_10_40396_hit1
5' UGGAGCAGGAUAGUGACGUGA
   |||||||:|||||||| ||||
   ACCUCGUUCUAUCACU-CACU 5'
AT5G45670.1     79      98
GDSL-motif lipase/hydrolase-like protein

sRNA_AGO1_Solexa_Mi2008_5_5834_hit1
5' CACUGUUGUGC-AAGAGAUCGA
   ||||||||||| || |||||||
   GUGACAACACGCUU-UCUAGCU 5'
AT5G45800.1     608     628
receptor kinase-like protein

sRNA_AGO1_Solexa_Mi2008_1_27959_hit1
5' UCACUGUUGUGC-AAGAGAUCGA
```

```
   |||||||||||| || |||||||
   AGUGACAACACGCUU-UCUAGCU 5'
AT5G45800.1     608     629
receptor kinase-like protein

sRNA_AGO1_Solexa_Mi2008_2_411_hit5
5' AAA-GAUGAAGAGAGAAAGAGA
   ||| | |||||||||||||| |
   UUUACCACUUCUCUCUUUCUAU 5'
AT5G45800.1     69      90
receptor kinase-like protein

sRNA_AGO1_Solexa_Mi2008_1_37976_hit1
5' UGCAGAAAUACAAAUGUAUGUG
   |||||||||||||||| || :
   ACGUCUUUAUGUUUACA-ACUU 5'
AT5G46230.1     548     568
unknown protein

sRNA_AGO1_Solexa_Mi2008_2_30665_hit1
5' UCGAGAAGAUGCUGA-AGAGAG
   ||||||||||||||| ||||||
   AGCUCUUCUACGACUCUCUCUC 5'
AT5G46410.2     127     148
unknown protein

sRNA_AGO1_Solexa_Mi2008_1_42884_hit1
5' UGG-UAACAGAUGAUGAUGACUUA
   ||| |||||||||||||| ||||
   ACCUAUUGUCUACUACUA-UGAAG 5'
AT5G46580.1     2135    2157
putative protein

sRNA_AGO1_Solexa_Mi2008_1_3344_hit1
5' AUGG-UAACAGAUGAUGAUGAC
   |||| |||||||||||||| ||
   UACCUAUUGUCUACUACUA-UG 5'
AT5G46580.1     2138    2158
putative protein

sRNA_AGO1_Solexa_Mi2008_1_53132_hit2
5' UUGGUG-UUGGUUGUGGUGGU
   :||||| ||| ||||||||||
   GACCACCAAC-AACACCACCA 5'
AT5G46760.1     111     130
putative transcription factor BHLH5

sRNA_AGO1_Solexa_Mi2008_2_10088_hit1
5' CUAGAACGAUUAUGGGUUUCA
    ||||| | ||||||||||||
   AAUCUU-C-AAUACCCAAAGU 5'
AT5G47200.1     896     914

sRNA_AGO1_Solexa_Mi2008_2_6120_hit1
5' CAGAUAC-AAAACGUAAAGAU
    |||| | |||||||||||||
   AUCUA-GCUUUUGCAUUUCUA 5'
AT5G48180.1     214     233
unknown protein

sRNA_AGO1_Solexa_Mi2008_1_29127_hit1
5' UCAUUGGGCUCAGUAUCAUA
   ||||||| ||||||||||||
```

```
   AGUAACCAGAGUCAUAGUAU 5'
AT5G48180.1     374     393
unknown protein

sRNA_AGO1_Solexa_Mi2008_1_37225_hit4
5' UGAUGAUGAUGAUGAUGAAGAAG
   |||||||||||||||||||||:
   ACUACUACUACUACUACUUCUUU 5'
AT5G48380.1     145     167
receptor-like protein kinase

sRNA_AGO1_Solexa_Mi2008_1_34620_hit1
5' UGAAGAUGAUGAUGAAGUU
   ||| |||||||||||||
   ACUACUACUACUACUUCUU 5'
AT5G48380.1     146     164
receptor-like protein kinase

sRNA_AGO1_Solexa_Mi2008_1_37225_hit4
5' UGAUGAUGAUGAUGAUGAAGAAG
   |||||||||||||||||| ||||
   ACUACUACUACUACUACUACUUC 5'
AT5G48380.1     148     170
receptor-like protein kinase

sRNA_AGO1_Solexa_Mi2008_9_14254_hit8
5' GAUGAUGAUGAUGAUGAUCUU
   ||||||||||||||||||
   CUACUACUACUACUACUACUU 5'
AT5G48380.1     149     169
receptor-like protein kinase

sRNA_AGO1_Solexa_Mi2008_6_14255_hit369
5' GAUGAUGAUGAUGAUGAUGAUGAU
   :|||||||||||||||||||||
   UUACUACUACUACUACUACUACUU 5'
AT5G48380.1     149     172
receptor-like protein kinase

sRNA_AGO1_Solexa_Mi2008_1_14256_hit8
5' GAUGAUGAUGAUGAUGAUGAUGUU
   :|||||||||||||||||||||
   UUACUACUACUACUACUACUACUU 5'
AT5G48380.1     149     172
receptor-like protein kinase

sRNA_AGO1_Solexa_Mi2008_7_3078_hit1
5' AUGAUGAUGA-GAAUGAUGAU
   |||||||||| || ||||||
   UACUACUACUACU-ACUACUU 5'
AT5G48380.1     150     169
receptor-like protein kinase

sRNA_AGO1_Solexa_Mi2008_1_36323_hit13
5' UGAGGAUGAUGAUGAUGAUGA
   ||| |||||||||||||||||
   ACUACUACUACUACUACUACU 5'
AT5G48380.1     150     170
receptor-like protein kinase

sRNA_AGO1_Solexa_Mi2008_1_3079_hit472
5' AUGAUGAUGAUGAUGAUGAUGA
   |||||||||||||||||||||
```

```
   UACUACUACUACUACUACUACU 5'
AT5G48380.1     150     171
receptor-like protein kinase

sRNA_AGO1_Solexa_Mi2008_1_45039_hit3
5' UGUGAUGAUGAUGAUGAUGA
    :||||||||||||||||||||
   UUACUACUACUACUACUACUACU 5'
AT5G48380.1     150     172
receptor-like protein kinase

sRNA_AGO1_Solexa_Mi2008_1_56092_hit3
5' UUUG-UGAUGAUGAUGAUGAUGA
   |||| |||||||||||||||||
   AAACUACUACUACUACUACUACU 5'
AT5G48380.1     151     173
receptor-like protein kinase

sRNA_AGO1_Solexa_Mi2008_7_3078_hit1
5' AUGAUGAUGA-GAAUGAUGAU
   |||||||||| | ||||||||
   UACUACUACUAC-UACUACUA 5'
AT5G48380.1     153     172
receptor-like protein kinase

sRNA_AGO1_Solexa_Mi2008_6_14255_hit369
5' GAUGA-UGAUGAUGAUGAUGAUGAU
   :|||| ||||||||||||||||||
   UUACUUACUACUACUACUACUACUA 5'
AT5G48380.1     153     177
receptor-like protein kinase

sRNA_AGO1_Solexa_Mi2008_1_45039_hit3
5' UGUGA-UGAUGAUGAUGAUGAUGA
     ||| |||||||||||||||||
   UAACUUACUACUACUACUACUACU 5'
AT5G48380.1     154     177
receptor-like protein kinase

sRNA_AGO1_Solexa_Mi2008_1_39699_hit1
5' UGGAACUGAUGAUGAUGAUGA
   |:||| |||||||||||||||
   AUCUU-ACUACUACUACUACU 5'
AT5G48380.1     157     176
receptor-like protein kinase

sRNA_AGO1_Solexa_Mi2008_1_3414_hit1
5' AUGGUUG-C-UCUGCUUUGCUG
   ||||||| | |||||||||||:
   UACCAACCGGAGACGAAACGAU 5'
AT5G48380.1     2113    2134
receptor-like protein kinase

sRNA_AGO1_Solexa_Mi2008_1_2_hit38
5' AAAAAAAAAAA-AAAACCAU
    |||||||||| |||||||
   CUUUUUUUUUUCUUUUGGUU 5'
AT5G48380.1     2693    2712
receptor-like protein kinase

sRNA_AGO1_Solexa_Mi2008_7_132_hit1
5' AAAAAUCACGGGUUCUCUA
    ||||||| |||||||||
```

```
   GUUUUAGUUCCCAAGAGAA 5'
AT5G48380.1     329     347
receptor-like protein kinase

sRNA_AGO1_Solexa_Mi2008_3_56550_hit1
5' UUUU-GGGUGCGAUUUUGGUUA
   |||| |||| ||||||:|||||
   AAAACCCCA-GCUAAAGCCAAU 5'
AT5G48540.1     464     484
33 kDa secretory protein-like

sRNA_AGO1_Solexa_Mi2008_4_46129_hit1
5' UGUUUUGAUGGUUGU-UGUA
   ||||| ||||||||| |||
   ACAAA-CUACCAACAUACAA 5'
AT5G48657.2     340     358
unknown protein

sRNA_AGO1_Solexa_Mi2008_4_46129_hit1
5' UGUUUUGAUGGUUGU-UGUA
   ||||| ||||||||| |||
   ACAAA-CUACCAACAUACAA 5'
AT5G48657.1     854     872
unknown protein

sRNA_AGO1_Solexa_Mi2008_2_501_hit1
5' AA-AGGAGGUGAAUAUAAGG-UU
   || |||||||||||| |||| ||
   UUAUCCUCCACUUAUUUUCCUAA 5'
AT5G48657.2     882     904
unknown protein

sRNA_AGO1_Solexa_Mi2008_1_6472_hit1
5' CAGGAGG-AGAAAGAGUAGGUU
   ||||||| ||||||||||||
   GUCCUCCCUCUUUCUCAUCCUC 5'
AT5G49280.1     30      51
predicted GPI-anchored protein

sRNA_AGO1_Solexa_Mi2008_1_35281_hit5
5' UGACAGUGAUGAUGAUGACGC
   ||| | |||||||||||||||
   ACU-U-ACUACUACUACUGCG 5'
AT5G49520.1     1018    1036
unknown protein

sRNA_AGO1_Solexa_Mi2008_1_13759_hit1
5' GACGA-UGAUGAUGAUGACA
   || || |||||||||||||
   CU-CUUACUACUACUACUGC 5'
AT5G49520.1     1019    1037
unknown protein

sRNA_AGO1_Solexa_Mi2008_1_40485_hit1
5' UGGAGGAAUGAUGAUGAUGUG
   ||||| |||||||||||||
   ACCUC-UUACUACUACUACUG 5'
AT5G49520.1     1020    1039
unknown protein

sRNA_AGO1_Solexa_Mi2008_1_7157_hit1
5' CAUGAUGAUGAUGGAACUGGU
    ||||||||||||| ||||
```

```
   UUACUACUACUACCUGGACCU 5'
AT5G49520.1     1148    1168
unknown protein

sRNA_AGO1_Solexa_Mi2008_1_37226_hit1
5' UGAUGAUGAUGGUACCAUGGU
   |||||||||||| ||| |||
   ACUACUACUACC-UGG-ACCU 5'
AT5G49520.1     1149    1167
unknown protein

sRNA_AGO1_Solexa_Mi2008_1_55151_hit1
5' UUUGAGAGUAGUAGCUUGAGA
   :|||| ||||| |||||||||
   GAACU-UCAUCUUCGAACUCU 5'
AT5G49520.1     1192    1211
unknown protein

sRNA_AGO1_Solexa_Mi2008_1_3079_hit472
5' AUGAUGAUGAUGAUG-AUGAUGA
   |||||||||||||||  ||| ||
   UACUACUACUACUACGAACU-CU 5'
AT5G49520.1     235     256
unknown protein

sRNA_AGO1_Solexa_Mi2008_1_51111_hit1
5' UUGAUGAUGAUGAUGGUUUGA
   ||||||||||||| |:||||
   AACUACUACUACUA-CGAACU 5'
AT5G49520.1     237     256
unknown protein

sRNA_AGO1_Solexa_Mi2008_2_22880_hit6
5' UAGGCAGAGAAGAGCAUCCA
    ||||||||||||| |||
   UUCCGUCUCUUCUCGAAGGA 5'
AT5G49610.1     1212    1231
unknown protein

sRNA_AGO1_Solexa_Mi2008_13_13620_hit1
5' GACAGAAGCAAUCAAC-CUA
    ||||||| ||||||| |||
   AUGUCUUCUUUAGUUGAGAU 5'
AT5G49610.1     1411    1430
unknown protein

sRNA_AGO1_Solexa_Mi2008_2_56650_hit2
5' UUUUUCAGGAAUGGUUGAU
   :|||||||||||| ||||
   GAAAAGUCCUUACGAACUU 5'
AT5G49610.1     507     525
unknown protein

sRNA_AGO1_Solexa_Mi2008_3_4134_hit1
5' CAAAGGACCUAUGGUAACUCAG
    |||| ||||||||||| |||
   AUUUC-UGGAUACCAUUG-GUC 5'
AT5G49610.1     681     700
unknown protein

sRNA_AGO1_Solexa_Mi2008_1_20881_hit10
5' UAGAAGAAGGUUGGUUAGUGU
   |||||||||||| ||| |||
```

```
   AUCUUCUUCCAA-CAA-CACC 5'
AT5G49630.1     917     935
amino acid permease 6 (emb|CAA65051.1)

sRNA_AGO1_Solexa_Mi2008_2_44959_hit1
5' UG-UGACUGUGAUGAAACUUCCA
   || ||||| |||||||||||| |
   ACUACUGAAACUACUUUGAAGAU 5'
AT5G49760.1     1950    1972
receptor protein kinase-like

sRNA_AGO1_Solexa_Mi2008_3_3062_hit1
5' AUGAGUUGUU-UCAGGGUCUU
   ||||||| || |||||||||:
   UACUCAA-AAGAGUCCCAGAG 5'
AT5G49760.1     2649    2668
receptor protein kinase-like

sRNA_AGO1_Solexa_Mi2008_5_1374_hit1
5' ACAGAGAAUUUGAGAUACA
      ||||||||||||||||
   AAACUCUUAAACUCUAUGU 5'
AT5G49980.1     260     278
transport inhibitor response 1 protein

sRNA_AGO1_Solexa_Mi2008_2_13419_hit2
5' GAAGAAGAAGAAGACA-CUU
   |||||||||||||| | |||
   CUUCUUCUUCUUCU-UCGAA 5'
AT5G49980.1     66      84
transport inhibitor response 1 protein

sRNA_AGO1_Solexa_Mi2008_1_27863_hit1
5' UCACUAAU-GACAAUGAGUGUC
   ||| |||| ||||||||||||
   AGU-AUUAUCUGUUACUCACAA 5'
AT5G50160.1     1170    1190
FRO1 and FRO2-like protein

sRNA_AGO1_Solexa_Mi2008_1_53198_hit1
5' UUGGUUGCGAUGAUGAUGUUG
   ||| ||| |||||||||||||
   AAC-AAC-CUACUACUACAAC 5'
AT5G50160.1     296     314
FRO1 and FRO2-like protein

sRNA_AGO1_Solexa_Mi2008_1_21052_hit1
5' UAGACCAAACCUGUGAUAGU
   |||||||||||| || |||:
   AUCUGGUUUGGA-AC-AUCG 5'
AT5G50160.1     527     544
FRO1 and FRO2-like protein

sRNA_AGO1_Solexa_Mi2008_1_13614_hit8
5' GACAGAAGAGAGAGAGCAC
   || |||||||||||| ||
   CU-UCUUCUCUCUCU-GUA 5'
AT5G50160.1     556     572
FRO1 and FRO2-like protein

sRNA_AGO1_Solexa_Mi2008_2_8245_hit1
5' CGAAAAGAAGAAGAUGAUGAG
   |||||||||||||| || |||
```

```
   GCUUUUCUUCUUCU-CU-CUC 5'
AT5G50160.1     560     578
FRO1 and FRO2-like protein

sRNA_AGO1_Solexa_Mi2008_1_24933_hit1
5' UAUCCAAAACACCACCCGGUCC
   :|||||||||| |||| |||||
   GUAGGUUUUGUUGUGGACCAGG 5'
AT5G50200.1     252     273
unknown protein

sRNA_AGO1_Solexa_Mi2008_1_24933_hit1
5' UAUCCAAAACACCACCCGGUCC
   :|||||||||| |||| |||||
   GUAGGUUUUGUUGUGGACCAGG 5'
AT5G50200.3     266     287
unknown protein

sRNA_AGO1_Solexa_Mi2008_1_24933_hit1
5' UAUCCAAAACACCACCCGGUCC
   :|||||||||| |||| |||||
   GUAGGUUUUGUUGUGGACCAGG 5'
AT5G50200.2     275     296
unknown protein

sRNA_AGO1_Solexa_Mi2008_70_10006_hit7
5' CUACUAUGU-AUGGUUACGUCA
   ||| ||| | ||||||||||||
   GAU-AUAAAGUACCAAUGCAGU 5'
AT5G50200.3     34      54
unknown protein

sRNA_AGO1_Solexa_Mi2008_4_5934_hit1
5' CAGAAGAGAGUGAGCACAU
   |||||||||| |||||||
   GUCUUCUCUCUCUCGUGUU 5'
AT5G50670.1     1099    1117
putative protein

sRNA_AGO1_Solexa_Mi2008_15_1354_hit1
5' ACAGAAGAGAGUGAGCACAU
   ||||||||||| |||||||
   UGUCUUCUCUCUCUCGUGUU 5'
AT5G50670.1     1099    1118
putative protein

sRNA_AGO1_Solexa_Mi2008_15_35242_hit1
5' UGACAGAAGAGAGUGAGCACAU
   ||||||||||||| |||||||
   ACUGUCUUCUCUCUCUCGUGUU 5'
AT5G50670.1     1099    1120
putative protein

sRNA_AGO1_Solexa_Mi2008_3_35247_hit2
5' UGACAGAAGAUAGAGAGCACUA
   |||||||||| ||||||||| |
   ACUGUCUUCUCUCUCUCGUGUU 5'
AT5G50670.1     1099    1120
putative protein

sRNA_AGO1_Solexa_Mi2008_1_1352_hit1
5' ACAGAAGAGAGAGAGCACU
   |||||||||||||||||||
```

```
   UGUCUUCUCUCUCUCGUGU 5'
AT5G50670.1     1100    1118
putative protein

sRNA_AGO1_Solexa_Mi2008_70_1353_hit7
5' ACAGAAGAGAGUGAGCACA
   ||||||||||| |||||||
   UGUCUUCUCUCUCUCGUGU 5'
AT5G50670.1     1100    1118
putative protein

sRNA_AGO1_Solexa_Mi2008_83_1355_hit2
5' ACAGAAGAUAGAGAGCACU
   |||||||| |||||||||
   UGUCUUCUCUCUCUCGUGU 5'
AT5G50670.1     1100    1118
putative protein

sRNA_AGO1_Solexa_Mi2008_1_13615_hit4
5' GACAGAAGAGAGAGAGCACA
   ||||||||||||||||||||
   CUGUCUUCUCUCUCUCGUGU 5'
AT5G50670.1     1100    1119
putative protein

sRNA_AGO1_Solexa_Mi2008_150_13617_hit7
5' GACAGAAGAGAGUGAGCACA
   |||||||||||| |||||||
   CUGUCUUCUCUCUCUCGUGU 5'
AT5G50670.1     1100    1119
putative protein

sRNA_AGO1_Solexa_Mi2008_161_13619_hit2
5' GACAGAAGAUAGAGAGCACU
   ||||||||| |||||||||
   CUGUCUUCUCUCUCUCGUGU 5'
AT5G50670.1     1100    1119
putative protein

sRNA_AGO1_Solexa_Mi2008_2_8397_hit1
5' CGACAGAAGAGAGUGAGCACA
    |||||||||||| |||||||
   ACUGUCUUCUCUCUCUCGUGU 5'
AT5G50670.1     1100    1120
putative protein

sRNA_AGO1_Solexa_Mi2008_2_35238_hit4
5' UGACAGAAGAGAGAGAGCACA
   |||||||||||||||||||||
   ACUGUCUUCUCUCUCUCGUGU 5'
AT5G50670.1     1100    1120
putative protein

sRNA_AGO1_Solexa_Mi2008_714_35241_hit6
5' UGACAGAAGAGAGUGAGCACA
   ||||||||||||| |||||||
   ACUGUCUUCUCUCUCUCGUGU 5'
AT5G50670.1     1100    1120
putative protein

sRNA_AGO1_Solexa_Mi2008_4_35245_hit2
5' UGACAGAAGAUAGAGAGCACA
   |||||||||| ||||||||||
```

```
   ACUGUCUUCUCUCUCUCGUGU 5'
AT5G50670.1     1100    1120
putative protein

sRNA_AGO1_Solexa_Mi2008_66_35246_hit2
5' UGACAGAAGAUAGAGAGCACU
   |||||||||| |||||||||
   ACUGUCUUCUCUCUCUCGUGU 5'
AT5G50670.1     1100    1120
putative protein

sRNA_AGO1_Solexa_Mi2008_2_11428_hit3
5' CUGACAGAAGAGAGUGAGCACA
    ||||||||||||| |||||||
   UACUGUCUUCUCUCUCUCGUGU 5'
AT5G50670.1     1100    1121
putative protein

sRNA_AGO1_Solexa_Mi2008_16_11431_hit1
5' CUGACAGAAGAUAGAGAGCACU
    |||||||||| |||||||||
   UACUGUCUUCUCUCUCUCGUGU 5'
AT5G50670.1     1100    1121
putative protein

sRNA_AGO1_Solexa_Mi2008_14_50261_hit1
5' UUGACAGAAGAGAGUGAGCACA
    ||||||||||||| |||||||
   UACUGUCUUCUCUCUCUCGUGU 5'
AT5G50670.1     1100    1121
putative protein

sRNA_AGO1_Solexa_Mi2008_29_50265_hit2
5' UUGACAGAAGAUAGAGAGCACA
    |||||||||| ||||||||||
   UACUGUCUUCUCUCUCUCGUGU 5'
AT5G50670.1     1100    1121
putative protein

sRNA_AGO1_Solexa_Mi2008_449_50266_hit1
5' UUGACAGAAGAUAGAGAGCACU
    |||||||||| |||||||||
   UACUGUCUUCUCUCUCUCGUGU 5'
AT5G50670.1     1100    1121
putative protein

sRNA_AGO1_Solexa_Mi2008_6_13612_hit1
5' GACAGAAGAAAGAGAGCAC
   ||||||||| |||||||||
   CUGUCUUCUCUCUCUCGUG 5'
AT5G50670.1     1101    1119
putative protein

sRNA_AGO1_Solexa_Mi2008_1_13614_hit8
5' GACAGAAGAGAGAGAGCAC
   |||||||||||||||||||
   CUGUCUUCUCUCUCUCGUG 5'
AT5G50670.1     1101    1119
putative protein

sRNA_AGO1_Solexa_Mi2008_480_13616_hit7
5' GACAGAAGAGAGUGAGCAC
   |||||||||||| ||||||
```

   CUGUCUUCUCUCUCUCGUG 5'
AT5G50670.1   1101   1119
putative protein

sRNA_AGO1_Solexa_Mi2008_855_13618_hit4
5' GACAGAAGAUAGAGAGCAC
   ||||||||| |||||||||
   CUGUCUUCUCUCUCUCGUG 5'
AT5G50670.1   1101   1119
putative protein

sRNA_AGO1_Solexa_Mi2008_3_8396_hit1
5' CGACAGAAGAGAGUGAGCAC
    |||||||||||| ||||||
   ACUGUCUUCUCUCUCUCGUG 5'
AT5G50670.1   1101   1120
putative protein

sRNA_AGO1_Solexa_Mi2008_6_35236_hit1
5' UGACAGAAGAAAGAGAGCAC
   |||||||||| |||||||||
   ACUGUCUUCUCUCUCUCGUG 5'
AT5G50670.1   1101   1120
putative protein

sRNA_AGO1_Solexa_Mi2008_12_35237_hit8
5' UGACAGAAGAGAGAGAGCAC
   ||||||||||||||||||||
   ACUGUCUUCUCUCUCUCGUG 5'
AT5G50670.1   1101   1120
putative protein

sRNA_AGO1_Solexa_Mi2008_2091_35240_hit6
5' UGACAGAAGAGAGUGAGCAC
   ||||||||||||| ||||||
   ACUGUCUUCUCUCUCUCGUG 5'
AT5G50670.1   1101   1120
putative protein

sRNA_AGO1_Solexa_Mi2008_193_35244_hit4
5' UGACAGAAGAUAGAGAGCAC
   |||||||||| |||||||||
   ACUGUCUUCUCUCUCUCGUG 5'
AT5G50670.1   1101   1120
putative protein

sRNA_AGO1_Solexa_Mi2008_28_11427_hit3
5' CUGACAGAAGAGAGUGAGCAC
    ||||||||||||| ||||||
   UACUGUCUUCUCUCUCUCGUG 5'
AT5G50670.1   1101   1121
putative protein

sRNA_AGO1_Solexa_Mi2008_79_11430_hit1
5' CUGACAGAAGAUAGAGAGCAC
    |||||||||| |||||||||
   UACUGUCUUCUCUCUCUCGUG 5'
AT5G50670.1   1101   1121
putative protein

sRNA_AGO1_Solexa_Mi2008_4_15853_hit2
5' GUGACAGAAGAGAGUGAGCAC
   :||||||||||||| ||||||

```
   UACUGUCUUCUCUCUCUCGUG 5'
AT5G50670.1     1101    1121
putative protein

sRNA_AGO1_Solexa_Mi2008_12_50256_hit1
5' UUGACAGAAGAAAGAGAGCAC
    |||||||||| |||||||||
   UACUGUCUUCUCUCUCUCGUG 5'
AT5G50670.1     1101    1121
putative protein

sRNA_AGO1_Solexa_Mi2008_14_50257_hit5
5' UUGACAGAAGAGAGAGAGCAC
    ||||||||||||||||||||
   UACUGUCUUCUCUCUCUCGUG 5'
AT5G50670.1     1101    1121
putative protein

sRNA_AGO1_Solexa_Mi2008_1778_50260_hit1
5' UUGACAGAAGAGAGUGAGCAC
    ||||||||||||| ||||||
   UACUGUCUUCUCUCUCUCGUG 5'
AT5G50670.1     1101    1121
putative protein

sRNA_AGO1_Solexa_Mi2008_4050_50264_hit3
5' UUGACAGAAGAUAGAGAGCAC
    |||||||||| |||||||||
   UACUGUCUUCUCUCUCUCGUG 5'
AT5G50670.1     1101    1121
putative protein

sRNA_AGO1_Solexa_Mi2008_1_16138_hit3
5' GUUGACAGAAGAUAGAGAGCAC
   | |||||||||| |||||||||
   CUACUGUCUUCUCUCUCUCGUG 5'
AT5G50670.1     1101    1122
putative protein

sRNA_AGO1_Solexa_Mi2008_30_35239_hit6
5' UGACAGAAGAGAGUGAGCA
   ||||||||||||| |||||
   ACUGUCUUCUCUCUCUCGU 5'
AT5G50670.1     1102    1120
putative protein

sRNA_AGO1_Solexa_Mi2008_1_11426_hit3
5' CUGACAGAAGAGAGUGAGCA
    ||||||||||||| |||||
   UACUGUCUUCUCUCUCUCGU 5'
AT5G50670.1     1102    1121
putative protein

sRNA_AGO1_Solexa_Mi2008_2_11429_hit1
5' CUGACAGAAGAUAGAGAGCA
    |||||||||| ||||||||
   UACUGUCUUCUCUCUCUCGU 5'
AT5G50670.1     1102    1121
putative protein

sRNA_AGO1_Solexa_Mi2008_16_50259_hit1
5' UUGACAGAAGAGAGUGAGCA
    ||||||||||||| |||||
```

```
   UACUGUCUUCUCUCUCUCGU 5'
AT5G50670.1     1102    1121
putative protein

sRNA_AGO1_Solexa_Mi2008_122_50263_hit3
5' UUGACAGAAGAUAGAGAGCA
    |||||||||| ||||||||
   UACUGUCUUCUCUCUCUCGU 5'
AT5G50670.1     1102    1121
putative protein

sRNA_AGO1_Solexa_Mi2008_1_50258_hit1
5' UUGACAGAAGAGAGUGAGC
    ||||||||||||| ||||
   UACUGUCUUCUCUCUCUCG 5'
AT5G50670.1     1103    1121
putative protein

sRNA_AGO1_Solexa_Mi2008_21_50262_hit3
5' UUGACAGAAGAUAGAGAGC
    |||||||||| |||||||
   UACUGUCUUCUCUCUCUCG 5'
AT5G50670.1     1103    1121
putative protein

sRNA_AGO1_Solexa_Mi2008_2_35025_hit1
5' UGAAUGAUGAUG-UGAAAGAA
   |||||||||||| | ||||||
   ACUUACUACUACUA-UUUCUU 5'
AT5G50670.1     1382    1401
putative protein

sRNA_AGO1_Solexa_Mi2008_2_2745_hit1
5' AUAUUUGUUGUAGUGAUAACA
   |||||| |||||||| |||||
   UAUAAAGAACAUCACAAUUGU 5'
AT5G50670.1     1404    1424
putative protein

sRNA_AGO1_Solexa_Mi2008_2_54927_hit2
5' UUUCGGUGUGUUAAGAAUGUC
   |||||||||||||||||||||
   AAAGCCACACAAUUCUUACAG 5'
AT5G50670.1     1426    1446
putative protein

sRNA_AGO1_Solexa_Mi2008_1_40228_hit1
5' UGGAGACAACAAGGUGAUGGU
   ||||||||||||| || |||:
   ACCUCUGUUGUUC-AC-ACCG 5'
AT5G50670.1     50      68
putative protein

sRNA_AGO1_Solexa_Mi2008_1_23609_hit1
5' UAGUAACCACCGGAGUUUUUAU
   |||||||||| |||||||||
   AUCAUUGGUGUCCUCAAAAACU 5'
AT5G50670.1     602     623
putative protein

sRNA_AGO1_Solexa_Mi2008_45_5105_hit1
5' CAAUCAAUAGUAGGGUGUCCA
   |||||||||||||||||||||
```

```
   GUUAGUUAUCAUCCCACAGGU 5'
AT5G51300.2     1188    1208
unknown protein

sRNA_AGO1_Solexa_Mi2008_45_5105_hit1
5' CAAUCAAUAGUAGGGUGUCCA
   |||||||||||||||||||||
   GUUAGUUAUCAUCCCACAGGU 5'
AT5G51300.3     1219    1239
unknown protein

sRNA_AGO1_Solexa_Mi2008_45_5105_hit1
5' CAAUCAAUAGUAGGGUGUCCA
   |||||||||||||||||||||
   GUUAGUUAUCAUCCCACAGGU 5'
AT5G51300.1     1223    1243
unknown protein

sRNA_AGO1_Solexa_Mi2008_1_17420_hit1
5' UAACCUUGAGUUGCUGAUUGAU
   ||||||||||||| || ||||
   AUUGGAACUCAACCACCAACUC 5'
AT5G51300.2     1715    1736
unknown protein

sRNA_AGO1_Solexa_Mi2008_1_17420_hit1
5' UAACCUUGAGUUGCUGAUUGAU
   ||||||||||||| || ||||
   AUUGGAACUCAACCACCAACUC 5'
AT5G51300.3     1746    1767
unknown protein

sRNA_AGO1_Solexa_Mi2008_1_17420_hit1
5' UAACCUUGAGUUGCUGAUUGAU
   ||||||||||||| || ||||
   AUUGGAACUCAACCACCAACUC 5'
AT5G51300.1     1750    1771
unknown protein

sRNA_AGO1_Solexa_Mi2008_1_1340_hit1
5' ACAC-UGAGGUGGUGGUGGU
   |||| |||||||||||||||
   UGUGUACUCCACCACCACCA 5'
AT5G51300.2     1927    1946
unknown protein

sRNA_AGO1_Solexa_Mi2008_68_26867_hit1
5' UCAACACUGAGGUGGUGGUGGU
   ||| || ||||||||||||||
   AGU-GU-ACUCCACCACCACCA 5'
AT5G51300.2     1927    1946
unknown protein

sRNA_AGO1_Solexa_Mi2008_1_48460_hit1
5' UUCAACACUGAGGUGGUGGUGGU
   :||| || ||||||||||||||
   GAGU-GU-ACUCCACCACCACCA 5'
AT5G51300.2     1927    1947
unknown protein

sRNA_AGO1_Solexa_Mi2008_1_48459_hit1
5' UUCAACACUGAGGUGGUGGU
   :||| || ||||||||||||
```

```
   GAGU-GU-ACUCCACCACCA 5'
AT5G51300.2     1930    1947
unknown protein

sRNA_AGO1_Solexa_Mi2008_1_1340_hit1
5' ACAC-UGAGGUGGUGGUGGU
   |||| |||||||||||||||
   UGUGUACUCCACCACCACCA 5'
AT5G51300.3     1958    1977
unknown protein

sRNA_AGO1_Solexa_Mi2008_68_26867_hit1
5' UCAACACUGAGGUGGUGGUGGU
   ||| || |||||||||||||||
   AGU-GU-ACUCCACCACCACCA 5'
AT5G51300.3     1958    1977
unknown protein

sRNA_AGO1_Solexa_Mi2008_1_48460_hit1
5' UUCAACACUGAGGUGGUGGUGGU
   :||| || |||||||||||||||
   GAGU-GU-ACUCCACCACCACCA 5'
AT5G51300.3     1958    1978
unknown protein

sRNA_AGO1_Solexa_Mi2008_1_48459_hit1
5' UUCAACACUGAGGUGGUGGU
   :||| || ||||||||||||
   GAGU-GU-ACUCCACCACCA 5'
AT5G51300.3     1961    1978
unknown protein

sRNA_AGO1_Solexa_Mi2008_1_1340_hit1
5' ACAC-UGAGGUGGUGGUGGU
   |||| |||||||||||||||
   UGUGUACUCCACCACCACCA 5'
AT5G51300.1     1962    1981
unknown protein

sRNA_AGO1_Solexa_Mi2008_68_26867_hit1
5' UCAACACUGAGGUGGUGGUGGU
   ||| || |||||||||||||||
   AGU-GU-ACUCCACCACCACCA 5'
AT5G51300.1     1962    1981
unknown protein

sRNA_AGO1_Solexa_Mi2008_1_48460_hit1
5' UUCAACACUGAGGUGGUGGUGGU
   :||| || |||||||||||||||
   GAGU-GU-ACUCCACCACCACCA 5'
AT5G51300.1     1962    1982
unknown protein

sRNA_AGO1_Solexa_Mi2008_1_48459_hit1
5' UUCAACACUGAGGUGGUGGU
   :||| || ||||||||||||
   GAGU-GU-ACUCCACCACCA 5'
AT5G51300.1     1965    1982
unknown protein

sRNA_AGO1_Solexa_Mi2008_6_3355_hit1
5' AUGGUAGUUCAAGUAUUCC
     :||||||||||||| ||||
```

```
   CGCCAUCAAGUUCA-AAGG 5'
AT5G51750.1     2082    2099
serine protease-like protein

sRNA_AGO1_Solexa_Mi2008_1_6206_hit1
5' CAGAUUGAGAUUAUGAA-GAUUU
   ||||| ||||||||||| ||||:
   GUCUAUCUCUAAUACUUCCUAAG 5'
AT5G52650.1     722     744
unknown protein

sRNA_AGO1_Solexa_Mi2008_1_6530_hit1
5' CAGGCUUCUUCGGGUCAAG
   |||||||||||||:|||
   GUCCGAAGAAGCCUAGUAG 5'
AT5G52740.1     228     246
putative protein

sRNA_AGO1_Solexa_Mi2008_1_41041_hit1
5' UGGAUUUAG-UCAGGUGUGGU
   :|||||| | |||||||||||
   GCCUAAA-CUAGUCCACACCA 5'
AT5G52810.1     622     641
unknown protein

sRNA_AGO1_Solexa_Mi2008_3_35894_hit1
5' UGAGAGCA-GAGAAAGAGAGU
   |||||| | |||||||||||
   ACUCUCUUUCUCUUUCUCUCU 5'
AT5G53160.2     27      47
unknown protein

sRNA_AGO1_Solexa_Mi2008_1_46130_hit14
5' UG-UUUUGAUUCAGGUACUAC
   || ||||||||||| ||||||
   ACUAAAACUAAGUCGAUGAUG 5'
AT5G53360.1     1072    1092
ring finger E3 ligase (SINAT5)

sRNA_AGO1_Solexa_Mi2008_4_55164_hit2
5' UUUGAGCUC-AGGUGGUAGUCA
   ||||||||| | |||||| |||
   AAACUCGAGCU-CACCAUAAGU 5'
AT5G53450.2     497     517
unknown protein

sRNA_AGO1_Solexa_Mi2008_4_55164_hit2
5' UUUGAGCUC-AGGUGGUAGUCA
   ||||||||| | |||||| |||
   AAACUCGAGCU-CACCAUAAGU 5'
AT5G53450.1     566     586
unknown protein

sRNA_AGO1_Solexa_Mi2008_1_33462_hit2
5' UCUGCAAA-AGAGUGAAAUGGA
   |||| ||| |||:|||||||||
   AGAC-UUUGUCUUACUUUACCU 5'
AT5G53760.2     1458    1478
unknown protein

sRNA_AGO1_Solexa_Mi2008_1_33462_hit2
5' UCUGCAAA-AGAGUGAAAUGGA
   |||| ||| |||:|||||||||
```

```
   AGAC-UUUGUCUUACUUUACCU 5'
AT5G53760.1     1596    1616
unknown protein

sRNA_AGO1_Solexa_Mi2008_3_12041_hit2
5' CUGGUAAAUUU-ACAUAAUUU
    |||||| ||| |||||||||
   AACCAUU-AAACUGUAUUAAA 5'
AT5G55620.1     563     582
unknown protein

sRNA_AGO1_Solexa_Mi2008_6_13612_hit1
5' GACAGAAGAAAGAGA-GCAC
   || |||||||||||| |||
   CU-UCUUCUUUCUCUUCGUU 5'
AT5G55620.1     76      94
unknown protein

sRNA_AGO1_Solexa_Mi2008_6_35236_hit1
5' UGACAGAAGAAAGAGA-GCAC
   ||| |||||||||||| |||
   ACU-UCUUCUUUCUCUUCGUU 5'
AT5G55620.1     76      95
unknown protein

sRNA_AGO1_Solexa_Mi2008_1_34109_hit2
5' UGAAAAGGUC-AGGGUUUAGA
   ||||||| || |||||||||
   ACUUUUC-AGGUCCCAAAUCG 5'
AT5G55940.1     757     776
unknown protein

sRNA_AGO1_Solexa_Mi2008_1_53720_hit1
5' UUG-UGUUGUUGGGCUUGUU
   ||| ||||||||||||| ||
   AACUACAACAACCCGAAGAA 5'
AT5G56010.1     1084    1103
heat shock protein 90

sRNA_AGO1_Solexa_Mi2008_1_53720_hit1
5' UUG-UGUUGUUGGGCUUGUU
   ||| ||||||||||||| ||
   AACUACAACAACCCGAAGAA 5'
AT5G56030.1     1100    1119
HEAT SHOCK PROTEIN 81-2 (HSP81-2) (sp|P55737)

sRNA_AGO1_Solexa_Mi2008_1_35788_hit1
5' UGAGAAGAGCGGU-UCACAG-GG
   ||||||||||||| ||| || ||
   ACUCUUCUCGCCAGAGUUUCUCC 5'
AT5G56030.1     2029    2051
HEAT SHOCK PROTEIN 81-2 (HSP81-2) (sp|P55737)

sRNA_AGO1_Solexa_Mi2008_1_2_hit38
5' AAAA-AAAAAAA-AAAACCAU
   |||| ||||||| ||||||||
   UUUUCUUUUUUUCUUUUGGUA 5'
AT5G56030.1     2268    2288
HEAT SHOCK PROTEIN 81-2 (HSP81-2) (sp|P55737)

sRNA_AGO1_Solexa_Mi2008_1_5366_hit3
5' CACAGUCAUAGAUAGUC-UCUG
   ||||| ||||||||||| |||
```

```
   GUGUCCGUAUCUAUCAGCAGAG 5'
AT5G56240.1     1894    1915
unknown protein

sRNA_AGO1_Solexa_Mi2008_1_39442_hit1
5' UGCUUAAAUGGAUGA-UUGGUU
   ||||:|||||||||| |||| |
   ACGAGUUUACCUACUAAACCUA 5'
AT5G56240.1     2853    2874
unknown protein

sRNA_AGO1_Solexa_Mi2008_1_52192_hit1
5' UUGGAUCAACAAACUGAUGG
   :||||||||||||||| ||
   GACCUAGUUGUUUGACU-CC 5'
AT5G56240.1     3062    3080
unknown protein

sRNA_AGO1_Solexa_Mi2008_6_733_hit1
5' AACUGUU-CAUUAAGUAGAA
   ||||||| | |||||||||
   UUGACAAAG-AAUUCAUCUC 5'
AT5G56240.1     3116    3134
unknown protein

sRNA_AGO1_Solexa_Mi2008_1_1800_hit1
5' AGAAGCAAGAGAUUGAGAGAG
   ||||| |||||||||||||||
   UCUUCUUUCUCUAACUCUCUC 5'
AT5G56240.1     79      99
unknown protein

sRNA_AGO1_Solexa_Mi2008_3_9236_hit1
5' CGGUAUUGAUCUCAGGAAGCA
   |||| ||||||||||||| ||
   GCCAAAACUAGAGUCCUU-GU 5'
AT5G56350.1     1473    1492
pyruvate kinase

sRNA_AGO1_Solexa_Mi2008_1_45939_hit1
5' UG-UUGACACAUGAUCCAUGGA
   || ||| |||||:|||||||||
   ACGAAC-GUGUAUUAGGUACCU 5'
AT5G56870.1     733     753
beta-galactosidase (emb|CAB64740.1)

sRNA_AGO1_Solexa_Mi2008_1_37225_hit4
5' UGAUGAUGAUGAUGAUGAAGAAG
   |||||||||||||||| |||||
   ACUACUACUACUACUAAUUCUUA 5'
AT5G57655.2     72      94
xylose isomerase

sRNA_AGO1_Solexa_Mi2008_1_29234_hit17
5' UCCAACUUCAGAUGACUGGUC
   |||||||||||| | ||||||
   AGGUUGAAGUCU-C-GACCAG 5'
AT5G57655.1     742     760
xylose isomerase

sRNA_AGO1_Solexa_Mi2008_1_48945_hit17
5' UUCCAACUUCAGAUGACUGGUC
    |||||||||||| | ||||||
```

```
   CAGGUUGAAGUCU-C-GACCAG 5'
AT5G57655.1     742     761
xylose isomerase

sRNA_AGO1_Solexa_Mi2008_9_14254_hit8
5' GAUGAUGAUGAUGAUGAUCUU
   ||||||||||||||||||
   CUACUACUACUACUACUAAUU 5'
AT5G57655.2     76      96
xylose isomerase

sRNA_AGO1_Solexa_Mi2008_1_36323_hit13
5' UGAGGAUGAUGAUGAUGAUGA
   ||| |||||||||||||| |
   ACUACUACUACUACUACUAAU 5'
AT5G57655.2     77      97
xylose isomerase

sRNA_AGO1_Solexa_Mi2008_1_3079_hit472
5' AUGAUGAUGAUGAUGAUGAUGA
    ||||||||||||||||||| |
   CACUACUACUACUACUACUAAU 5'
AT5G57655.2     77      98
xylose isomerase

sRNA_AGO1_Solexa_Mi2008_1_45039_hit3
5' UGUGAUGAUGAUGAUGAUGAUGA
    |||||||||||||||||||| |
   UCACUACUACUACUACUACUAAU 5'
AT5G57655.2     77      99
xylose isomerase

sRNA_AGO1_Solexa_Mi2008_1_56092_hit3
5' UUUGUGAUGAUGAUGAUGAUGA
   |  |||||||||||||||||||
   AUUCACUACUACUACUACUACU 5'
AT5G57655.2     80      101
xylose isomerase

sRNA_AGO1_Solexa_Mi2008_7_3078_hit1
5' AUGAUGAUGA-GAAUGAUGAU
    ||||||||| || |||||||
   GACUACUACUACU-ACUACUA 5'
AT5G57655.2     80      99
xylose isomerase

sRNA_AGO1_Solexa_Mi2008_1_36323_hit13
5' UGAG-GAUGAUGAUGAUGAUGA
     || ||||||||||||||||
   UAUCACUACUACUACUACUACU 5'
AT5G57655.2     81      102
xylose isomerase

sRNA_AGO1_Solexa_Mi2008_1_2280_hit1
5' AGUGGAUGAUGAUGAUGAUG
   |||| ||||||||||||||
   UCAC-UACUACUACUACUAC 5'
AT5G57655.2     82      100
xylose isomerase

sRNA_AGO1_Solexa_Mi2008_1_29234_hit17
5' UCCAACUUCAGAUGACUGGUC
   |||||||||||| | ||||||
```

```
   AGGUUGAAGUCU-C-GACCAG 5'
AT5G57655.2     858     876
xylose isomerase

sRNA_AGO1_Solexa_Mi2008_1_48945_hit17
5' UUCCAACUUCAGAUGACUGGUC
    |||||||||||| | ||||||
   CAGGUUGAAGUCU-C-GACCAG 5'
AT5G57655.2     858     877
xylose isomerase

sRNA_AGO1_Solexa_Mi2008_2_48112_hit1
5' UUA-UA-UCGGGUUUGAAUGGGU
   ||| || ||||||||:|||||||
   AAUCAUUAGCCCAAAUUUACCCA 5'
AT5G57900.1     1063    1085
unknown protein

sRNA_AGO1_Solexa_Mi2008_1_47310_hit1
5' UUAGAA-UGAGGUUGAUGAGUAUC
   || ||| ||| |||||||||||||
   AA-CUUUACUACAACUACUCAUAG 5'
AT5G57900.1     60      82
unknown protein

sRNA_AGO1_Solexa_Mi2008_1_21349_hit7
5' UAGAGGAAAGAGAGCAAUGAC
   :||||||||||||| |||||
   GUCUCCUUUCUCUCUUUACUC 5'
AT5G57910.1     65      85
unknown protein

sRNA_AGO1_Solexa_Mi2008_1_23549_hit4
5' UAGGUUCAACAAACACGAGGAG
    ||||||||||| ||| |||||
   CUCCAAGUUGUUGGUG-UCCUC 5'
AT5G58210.1     1050    1070
unknown protein

sRNA_AGO1_Solexa_Mi2008_1_37315_hit1
5' UGAUGGAGGCAAGUGUGCAGG
   ||||||||:|||||||| |||
   ACUACCUCUGUUCACAC-UCC 5'
AT5G58210.3     647     666
unknown protein

sRNA_AGO1_Solexa_Mi2008_1_37315_hit1
5' UGAUGGAGGCAAGUGUGCAGG
   ||||||||:|||||||| |||
   ACUACCUCUGUUCACAC-UCC 5'
AT5G58210.2     672     691
unknown protein

sRNA_AGO1_Solexa_Mi2008_1_37315_hit1
5' UGAUGGAGGCAAGUGUGCAGG
   ||||||||:|||||||| |||
   ACUACCUCUGUUCACAC-UCC 5'
AT5G58210.4     691     710
unknown protein

sRNA_AGO1_Solexa_Mi2008_1_37315_hit1
5' UGAUGGAGGCAAGUGUGCAGG
   ||||||||:|||||||| |||
```

```
   ACUACCUCUGUUCACAC-UCC 5'
AT5G58210.1     786     805
unknown protein

sRNA_AGO1_Solexa_Mi2008_1_23549_hit4
5' UAGGUUCAACAAACACGAGGAG
    ||||||||||| ||| |||||
   CUCCAAGUUGUUGGUG-UCCUC 5'
AT5G58210.3     911     931
unknown protein

sRNA_AGO1_Solexa_Mi2008_1_23549_hit4
5' UAGGUUCAACAAACACGAGGAG
    ||||||||||| ||| |||||
   CUCCAAGUUGUUGGUG-UCCUC 5'
AT5G58210.2     936     956
unknown protein

sRNA_AGO1_Solexa_Mi2008_1_23549_hit4
5' UAGGUUCAACAAACACGAGGAG
    ||||||||||| ||| |||||
   CUCCAAGUUGUUGGUG-UCCUC 5'
AT5G58210.4     955     975
unknown protein

sRNA_AGO1_Solexa_Mi2008_1_40627_hit1
5' UGGAGGUGCCGAUGAUGCUAU
   ||||| ||||:||||||||||
   ACCUC-ACGGUUACUACGAUA 5'
AT5G58350.1     1255    1274
MAP kinase

sRNA_AGO1_Solexa_Mi2008_1_13759_hit1
5' GACGAUGAUGAUGAUGACA
   |||||||||||| ||||
   CUGCUACUACUAGUACUCC 5'
AT5G58350.1     1916    1934
MAP kinase

sRNA_AGO1_Solexa_Mi2008_5_13760_hit2
5' GACGAUGAUGAUGAUGAGC
   |||||||||||| |||||
   CUGCUACUACUAGUACUCC 5'
AT5G58350.1     1916    1934
MAP kinase

sRNA_AGO1_Solexa_Mi2008_1_13759_hit1
5' GACGAUGAUGAUGAUGA-CA
   :|||||||:|||||||| ||
   UUGCUACUGCUACUACUAGU 5'
AT5G58350.1     1922    1941
MAP kinase

sRNA_AGO1_Solexa_Mi2008_1_38921_hit1
5' UGCGG-ACGAUGAUGAUGAUGAU
   |:||| |||||||:|||||||||
   AUGCCAUGCUACUGCUACUACUA 5'
AT5G58350.1     1924    1946
MAP kinase

sRNA_AGO1_Solexa_Mi2008_1_40903_hit2
5' UGGAUGAUUUCACAGGAUUGU
   ||||||||||||||  |||||
```

```
   ACCUACUAAAGUGU--UAACA 5'
AT5G58720.2     1624    1642
putative PRL1 associated protein

sRNA_AGO1_Solexa_Mi2008_2_18688_hit10
5' UAAUCCGCCACAAGUUCCAUCC
    ||||| ||| |||||||||||
   CUUAGG-GGUUUUCAAGGUAGG 5'
AT5G58930.1     1430    1450
unknown protein (At5g58930)

sRNA_AGO1_Solexa_Mi2008_1_14852_hit1
5' GGAGAGACAGAGGCGUUGA
   |||||||||||||| ||
   CCUCUCUGUCUCCG-AAAG 5'
AT5G59440.2     900     917
thymidylate kinase - like protein

sRNA_AGO1_Solexa_Mi2008_1_14852_hit1
5' GGAGAGACAGAGGCGUUGA
   |||||||||||||| ||
   CCUCUCUGUCUCCG-AAAG 5'
AT5G59440.1     911     928
thymidylate kinase - like protein

sRNA_AGO1_Solexa_Mi2008_1_14852_hit1
5' GGAGAGACAGAGGCGUUGA
   |||||||||||||| ||
   CCUCUCUGUCUCCG-AAAG 5'
AT5G59440.3     951     968
thymidylate kinase - like protein

sRNA_AGO1_Solexa_Mi2008_2_49343_hit1
5' UUCGCCGUCUUGUUCUAGUAAACC
   || ||| ||||||| |||||||||
   AA-CGGAAGAACAACAUCAUUUGG 5'
AT5G59500.1     1169    1191
unknown protein

sRNA_AGO1_Solexa_Mi2008_2_44392_hit1
5' UGUAUGGACCGUGGCAUGUCU
   |||| ||||| ||||||||||
   ACAU-CCUGGAACCGUACAGA 5'
AT5G60020.1     1227    1246
laccase - like protein

sRNA_AGO1_Solexa_Mi2008_1_54567_hit1
5' UUUAUCUGCAUCAAGAA-AAGA
   ||||||| ||||||||| |||
   AAAUAGAGGUAGUUCUUGUUCG 5'
AT5G60020.1     1741    1762
laccase - like protein

sRNA_AGO1_Solexa_Mi2008_2_52985_hit50
5' UUGGUCGU-UUUAGGUGGUUGA
   |||||| | ||||||||||| |
   AACCAG-ACAAAUCCACCAAGU 5'
AT5G60020.1     994     1014
laccase - like protein

sRNA_AGO1_Solexa_Mi2008_4_2943_hit3
5' AUCUUGAUGAUGCUGCAUC
   |||:||||||||||||||
```

```
   UAGGACUACUACGACGUAA 5'
AT5G60120.1     1645    1663
APETALA2 protein - like

sRNA_AGO1_Solexa_Mi2008_3_1834_hit2
5' AGAAUCUUGAUGAUGCUGCAUC
   ||||||:|||||||||||||
   UCUUAGGACUACUACGACGUAA 5'
AT5G60120.1     1645    1666
APETALA2 protein - like

sRNA_AGO1_Solexa_Mi2008_1_1044_hit2
5' AAUCCUGAUGAUGCUGCAU
   |||||||||||||||||||
   UUAGGACUACUACGACGUA 5'
AT5G60120.1     1646    1664
APETALA2 protein - like

sRNA_AGO1_Solexa_Mi2008_3_1051_hit2
5' AAUCUUGAUGAUGCUGCAG
   ||||:|||||||||||||
   UUAGGACUACUACGACGUA 5'
AT5G60120.1     1646    1664
APETALA2 protein - like

sRNA_AGO1_Solexa_Mi2008_54_1052_hit3
5' AAUCUUGAUGAUGCUGCAU
   ||||:||||||||||||||
   UUAGGACUACUACGACGUA 5'
AT5G60120.1     1646    1664
APETALA2 protein - like

sRNA_AGO1_Solexa_Mi2008_1_13547_hit1
5' GAAUCCUGAUGAUGCUGCAG
   |||||||||||||||||||
   CUUAGGACUACUACGACGUA 5'
AT5G60120.1     1646    1665
APETALA2 protein - like

sRNA_AGO1_Solexa_Mi2008_1_13548_hit2
5' GAAUCCUGAUGAUGCUGCAU
   ||||||||||||||||||||
   CUUAGGACUACUACGACGUA 5'
AT5G60120.1     1646    1665
APETALA2 protein - like

sRNA_AGO1_Solexa_Mi2008_55_13552_hit2
5' GAAUCUUGAUGAUGCUGCAG
   |||||:|||||||||||||
   CUUAGGACUACUACGACGUA 5'
AT5G60120.1     1646    1665
APETALA2 protein - like

sRNA_AGO1_Solexa_Mi2008_495_13553_hit3
5' GAAUCUUGAUGAUGCUGCAU
   |||||:||||||||||||||
   CUUAGGACUACUACGACGUA 5'
AT5G60120.1     1646    1665
APETALA2 protein - like

sRNA_AGO1_Solexa_Mi2008_1_1827_hit1
5' AGAAUCCUGAUGAUGCUGCAG
   ||||||||||||||||||||
```

```
   UCUUAGGACUACUACGACGUA 5'
AT5G60120.1     1646    1666
APETALA2 protein - like

sRNA_AGO1_Solexa_Mi2008_13_1828_hit2
5' AGAAUCCUGAUGAUGCUGCAU
   |||||||||||||||||||||
   UCUUAGGACUACUACGACGUA 5'
AT5G60120.1     1646    1666
APETALA2 protein - like

sRNA_AGO1_Solexa_Mi2008_834_1831_hit2
5' AGAAUCUUGAUGAUGCUGCAG
   ||||||:|||||||||||||
   UCUUAGGACUACUACGACGUA 5'
AT5G60120.1     1646    1666
APETALA2 protein - like

sRNA_AGO1_Solexa_Mi2008_6188_1833_hit2
5' AGAAUCUUGAUGAUGCUGCAU
   ||||||:||||||||||||||
   UCUUAGGACUACUACGACGUA 5'
AT5G60120.1     1646    1666
APETALA2 protein - like

sRNA_AGO1_Solexa_Mi2008_374_14811_hit1
5' GGAAUCUUGAUGAUGCUGCAU
   :|||||:||||||||||||||
   UCUUAGGACUACUACGACGUA 5'
AT5G60120.1     1646    1666
APETALA2 protein - like

sRNA_AGO1_Solexa_Mi2008_4_13922_hit2
5' GAGAAUCUUGAUGAUGCUGCAG
   |||||||:|||||||||||||
   CUCUUAGGACUACUACGACGUA 5'
AT5G60120.1     1646    1667
APETALA2 protein - like

sRNA_AGO1_Solexa_Mi2008_25_13923_hit2
5' GAGAAUCUUGAUGAUGCUGCAU
   |||||||:||||||||||||||
   CUCUUAGGACUACUACGACGUA 5'
AT5G60120.1     1646    1667
APETALA2 protein - like

sRNA_AGO1_Solexa_Mi2008_1_15149_hit1
5' GGGAAUCUUGAUGAUGCUGCAU
   |:|||||:||||||||||||||
   CUCUUAGGACUACUACGACGUA 5'
AT5G60120.1     1646    1667
APETALA2 protein - like

sRNA_AGO1_Solexa_Mi2008_117_13551_hit5
5' GAAUCUUGAUGAUGCUGCA
   |||||:|||||||||||||
   CUUAGGACUACUACGACGU 5'
AT5G60120.1     1647    1665
APETALA2 protein - like

sRNA_AGO1_Solexa_Mi2008_8_1826_hit3
5' AGAAUCCUGAUGAUGCUGCA
   ||||||||||||||||||||
```

```
   UCUUAGGACUACUACGACGU 5'
AT5G60120.1     1647    1666
APETALA2 protein - like

sRNA_AGO1_Solexa_Mi2008_2484_1830_hit4
5' AGAAUCUUGAUGAUGCUGCA
   ||||||:|||||||||||||
   UCUUAGGACUACUACGACGU 5'
AT5G60120.1     1647    1666
APETALA2 protein - like

sRNA_AGO1_Solexa_Mi2008_1_14807_hit1
5' GGAAUCCUGAUGAUGCUGCA
   :|||||||||||||||||||
   UCUUAGGACUACUACGACGU 5'
AT5G60120.1     1647    1666
APETALA2 protein - like

sRNA_AGO1_Solexa_Mi2008_102_14810_hit1
5' GGAAUCUUGAUGAUGCUGCA
   :|||||:|||||||||||||
   UCUUAGGACUACUACGACGU 5'
AT5G60120.1     1647    1666
APETALA2 protein - like

sRNA_AGO1_Solexa_Mi2008_2_13921_hit4
5' GAGAAUCUUGAUGAUGCUGCA
   |||||||:|||||||||||||
   CUCUUAGGACUACUACGACGU 5'
AT5G60120.1     1647    1667
APETALA2 protein - like

sRNA_AGO1_Solexa_Mi2008_25_1829_hit4
5' AGAAUCUUGAUGAUGCUGC
   ||||||:||||||||||||
   UCUUAGGACUACUACGACG 5'
AT5G60120.1     1648    1666
APETALA2 protein - like

sRNA_AGO1_Solexa_Mi2008_3_14809_hit1
5' GGAAUCUUGAUGAUGCUGC
   :|||||:||||||||||||
   UCUUAGGACUACUACGACG 5'
AT5G60120.1     1648    1666
APETALA2 protein - like

sRNA_AGO1_Solexa_Mi2008_1_13920_hit4
5' GAGAAUCUUGAUGAUGCUGC
   |||||||:||||||||||||
   CUCUUAGGACUACUACGACG 5'
AT5G60120.1     1648    1667
APETALA2 protein - like

sRNA_AGO1_Solexa_Mi2008_1_35822_hit4
5' UGAGAAUCUUGAUGAUGCUGC
   ||||||||:||||||||||||
   ACUCUUAGGACUACUACGACG 5'
AT5G60120.1     1648    1668
APETALA2 protein - like

sRNA_AGO1_Solexa_Mi2008_1_5081_hit1
5' CAAUAACGAAAUUGAUGUCAG
   |||||||||||||||||||||
```

```
   GUUAUUGCUUUAACUACAGUC 5'
AT5G60120.1     998     1018
APETALA2 protein - like

sRNA_AGO1_Solexa_Mi2008_8_34422_hit1
5' UGAAC-CUUGGAAGAAAACUUU
   ||||| ||||||||||| ||||
   ACUUGAGAACCUUCUUUCGAAA 5'
AT5G60160.1     1478    1499
aspartyl aminopeptidase - like protein

sRNA_AGO1_Solexa_Mi2008_1_27291_hit3
5' UCAAGGUUGGUGGACCUCUCA
   ||||||||||| |||||||||
   AGUUCCAACCAACUGGAGAGU 5'
AT5G60390.3     664     684
translation elongation factor eEF-1 alpha chain (gene A4)

sRNA_AGO1_Solexa_Mi2008_1_27291_hit3
5' UCAAGGUUGGUGGACCUCUCA
   ||||||||||| |||||||||
   AGUUCCAACCAACUGGAGAGU 5'
AT5G60390.2     674     694
translation elongation factor eEF-1 alpha chain (gene A4)

sRNA_AGO1_Solexa_Mi2008_7_12746_hit1
5' CUUGACCUUGUAAGACCCC
   ||||||||||:||||||
   GAACUGGAACGUUCUGGAA 5'
AT5G60450.1     1873    1891
auxin response factor 4

sRNA_AGO1_Solexa_Mi2008_7_49852_hit1
5' UUCUUGACCUUGUAAGACCCC
   ||||||||||||:||||||
   AAGAACUGGAACGUUCUGGAA 5'
AT5G60450.1     1873    1893
auxin response factor 4

sRNA_AGO1_Solexa_Mi2008_4_55012_hit1
5' UUUCUUGACCUUGUAAGACCCC
   |||||||||||||:||||||
   AAAGAACUGGAACGUUCUGGAA 5'
AT5G60450.1     1873    1894
auxin response factor 4

sRNA_AGO1_Solexa_Mi2008_1_55011_hit1
5' UUUCUUGACCUUGUAAGACCC
   |||||||||||||:||||||
   AAAGAACUGGAACGUUCUGGA 5'
AT5G60450.1     1874    1894
auxin response factor 4

sRNA_AGO1_Solexa_Mi2008_19_29991_hit1
5' UCCGGCGGUUCAUAACAUCAA
   ||||||||||||||||||||
   AGGCCGCCAAGUAUUGUAGUU 5'
AT5G60450.1     1925    1945
auxin response factor 4

sRNA_AGO1_Solexa_Mi2008_2_24987_hit1
5' UAUCCGGCGGUUCAUAACAUC
   ||||||||||||||||||||
```

```
   AUAGGCCGCCAAGUAUUGUAG 5'
AT5G60450.1     1927    1947
auxin response factor 4

sRNA_AGO1_Solexa_Mi2008_8_4564_hit1
5' CAAGAACUGGAUUUGCAUGAGA
   ||||||||||||||||||||||
   GUUCUUGACCUAAACGUACUCU 5'
AT5G60450.1     1966    1987
auxin response factor 4

sRNA_AGO1_Solexa_Mi2008_7_4563_hit1
5' CAAGAACUGGAUUUGCAUGAG
   |||||||||||||||||||||
   GUUCUUGACCUAAACGUACUC 5'
AT5G60450.1     1967    1987
auxin response factor 4

sRNA_AGO1_Solexa_Mi2008_1_34330_hit1
5' UGAACAAGCUGGGUUCACGCC
   |||||||||||||||||||||
   ACUUGUUCGACCCAAGUGCGG 5'
AT5G60450.1     2035    2055
auxin response factor 4

sRNA_AGO1_Solexa_Mi2008_7_33918_hit1
5' UCUUGACCUUGUAAGACCCCA
   |||||||||||:|||||||
   AGAACUGGAACGUUCUGGGAA 5'
AT5G60450.1     2082    2102
auxin response factor 4

sRNA_AGO1_Solexa_Mi2008_7_49853_hit1
5' UUCUUGACCUUGUAAGACCCCA
   ||||||||||||:|||||||
   AAGAACUGGAACGUUCUGGGAA 5'
AT5G60450.1     2082    2103
auxin response factor 4

sRNA_AGO1_Solexa_Mi2008_7_12746_hit1
5' CUUGACCUUGUAAGACCCC
   ||||||||||:|||||||
   GAACUGGAACGUUCUGGGA 5'
AT5G60450.1     2083    2101
auxin response factor 4

sRNA_AGO1_Solexa_Mi2008_7_49852_hit1
5' UUCUUGACCUUGUAAGACCCC
   ||||||||||||:|||||||
   AAGAACUGGAACGUUCUGGGA 5'
AT5G60450.1     2083    2103
auxin response factor 4

sRNA_AGO1_Solexa_Mi2008_4_55012_hit1
5' UUUCUUGACCUUGUAAGACCCC
   |||||||||||||:|||||||
   AAAGAACUGGAACGUUCUGGGA 5'
AT5G60450.1     2083    2104
auxin response factor 4

sRNA_AGO1_Solexa_Mi2008_1_55011_hit1
5' UUUCUUGACCUUGUAAGACCC
   |||||||||||||:|||||||
```

```
   AAAGAACUGGAACGUUCUGGG 5'
AT5G60450.1     2084    2104
auxin response factor 4

sRNA_AGO1_Solexa_Mi2008_2_34797_hit1
5' UGAAGGGGGACCCGAGGAUUG
   |||||||||||||||||||||
   ACUUCCCCCUGGGCUCCUAAC 5'
AT5G60450.1     2266    2286
auxin response factor 4

sRNA_AGO1_Solexa_Mi2008_1_7242_hit1
5' CAUGGAGAAGAAGAAUGA-GGU
   ||||:||||||||||| || |||
   GUACUUCUUCUUCUU-CUACCA 5'
AT5G60450.1     625     645
auxin response factor 4

sRNA_AGO1_Solexa_Mi2008_1_25289_hit1
5' UAUGACGAUGAUGAUGACGGA
   :||||||||||||||  ||||
   GUACUGCUACUACUA-GGCCU 5'
AT5G60450.1     650     669
auxin response factor 4

sRNA_AGO1_Solexa_Mi2008_9_14254_hit8
5' GAUGAUGAUGAUGAUGAUCUU
   ||||||||:||||||||||:
   CUACUACUGCUACUACUAGGC 5'
AT5G60450.1     651     671
auxin response factor 4

sRNA_AGO1_Solexa_Mi2008_7_3078_hit1
5' AUGAUGAUGA-GAAUGAUGAU
    ||||||||| || |||||||
   AACUACUACUGCU-ACUACUA 5'
AT5G60450.1     655     674
auxin response factor 4

sRNA_AGO1_Solexa_Mi2008_1_45039_hit3
5' UGU-GAUGAUGAUGAUGAUGAUGA
   ||| || ||||||||:||||||||
   ACAUCUUCUACUACUGCUACUACU 5'
AT5G60450.1     656     679
auxin response factor 4

sRNA_AGO1_Solexa_Mi2008_1_10092_hit1
5' CUAGAAGAUGAUGACGAUUGA
   ||||||||||||||||| ||
   GAUCUUCUACUACUGCUA-CU 5'
AT5G60450.1     659     678
auxin response factor 4

sRNA_AGO1_Solexa_Mi2008_3_35894_hit1
5' UGAGAGCAGAGAAAGAGAGU
   |||||| ||||||||||||
   ACUCUC-UCUCUUUCUCUCC 5'
AT5G60450.1     67      85
auxin response factor 4

sRNA_AGO1_Solexa_Mi2008_1_11311_hit1
5' CUGAAAAAAAAAGAGAGGG
   || |||||||||||||||
```

```
   GA-UUUUUUUUUCUCUCCA 5'
AT5G60850.1     1218    1235
zinc finger protein OBP4 - like

sRNA_AGO1_Solexa_Mi2008_1_1776_hit10
5' AGAAAAAAAAAAAAAAAAAAU
     |||||||||||||||||
   GGUUUUUUUUUUUUUUUUUUC 5'
AT5G61020.2     1673    1693
unknown protein

sRNA_AGO1_Solexa_Mi2008_1_3_hit25
5' AAAAAAAAAAAAAGAAAGA
   ||||||||||||| |||:
   UUUUUUUUUUUUU-UUUUC 5'
AT5G61020.2     1674    1691
unknown protein

sRNA_AGO1_Solexa_Mi2008_1_3_hit25
5' AAAAAAAAAAAAAGAAAGA
   ||||||||||||| |||:|
   UUUUUUUUUUUUU-UUUUU 5'
AT5G61020.2     1675    1692
unknown protein

sRNA_AGO1_Solexa_Mi2008_1_1776_hit10
5' AGAAAAAAAAAAAAAAAAAAU
     |||||||||||||||||
   GGUUUUUUUUUUUUUUUUUUC 5'
AT5G61020.1     1678    1698
unknown protein

sRNA_AGO1_Solexa_Mi2008_1_3_hit25
5' AAAAAAAAAAAAAGAAAGA
   ||||||||||||| |||:
   UUUUUUUUUUUUU-UUUUC 5'
AT5G61020.1     1679    1696
unknown protein

sRNA_AGO1_Solexa_Mi2008_1_3_hit25
5' AAAAAAAAAAAAAGAAAGA
   ||||||||||||| |||:|
   UUUUUUUUUUUUU-UUUUU 5'
AT5G61020.1     1680    1697
unknown protein

sRNA_AGO1_Solexa_Mi2008_1_352_hit1
5' AAAGAAGAAGAUA-AAAGGA
    |||||||||| | ||||||
   GUUCUUCUUCU-UCUUUCCU 5'
AT5G61210.1     107     125
snap25a

sRNA_AGO1_Solexa_Mi2008_14_6261_hit1
5' CAGCAGGUAGUCCUCAUAGAU
   ||| ||||||||||| ||||
   GUC-UCCAUCAGGAGAAUCUU 5'
AT5G61210.1     427     446
snap25a

sRNA_AGO1_Solexa_Mi2008_3_39824_hit1
5' UGGAAUACUUGAAC-UACCAUCU
   ||||||||| |||  ||||||||
```

```
   ACCUUAUGA-CUUUUAUGGUAGA 5'
AT5G61600.1     719     740
DNA binding protein - like

sRNA_AGO1_Solexa_Mi2008_1_50782_hit1
5' UUGAGGGUUGGAAUAG-ACA-UGG
   ||||||||||| |||| ||| |||
   AACUCCCAACCGUAUCAUGUAACC 5'
AT5G61620.1     739     762
transcriptional activator - like protein

sRNA_AGO1_Solexa_Mi2008_8_50781_hit1
5' UUGAGGGUUGGAAUAG-ACAUG
   ||||||||||| |||| ||||
   AACUCCCAACCGUAUCAUGUAA 5'
AT5G61620.1     741     762
transcriptional activator - like protein

sRNA_AGO1_Solexa_Mi2008_15_12780_hit1
5' CUUGAGGGUUGGAAUAG-ACA
   |||||||||||| |||| |||
   GAACUCCCAACCGUAUCAUGU 5'
AT5G61620.1     743     763
transcriptional activator - like protein

sRNA_AGO1_Solexa_Mi2008_1_33924_hit1
5' UCUUGAGGGUUGGAAUAG-ACA
   ||||||||||||| |||| |||
   AGAACUCCCAACCGUAUCAUGU 5'
AT5G61620.1     743     764
transcriptional activator - like protein

sRNA_AGO1_Solexa_Mi2008_2_5441_hit1
5' CACCACCACCACCAGCGCCGC
   |||||||||||||| ||||:|
   GUGGUGGUGGUGGUGGCGGUG 5'
AT5G61660.1     399     419
unknown protein

sRNA_AGO1_Solexa_Mi2008_1_7688_hit1
5' CCACCACCACCUCCAGCGCCAC
   ||||||||||| ||| ||||||
   GGUGGUGGUGGUGGUGGCGGUG 5'
AT5G61660.1     399     420
unknown protein

sRNA_AGO1_Solexa_Mi2008_1_1510_hit1
5' ACCACCACCACCAGCGCCGCC
   ||||||||||||| |:|||||
   UGGUGGUGGUGGUGGUGGCGG 5'
AT5G61660.1     401     421
unknown protein

sRNA_AGO1_Solexa_Mi2008_8_13469_hit2
5' GAAGAUUCAGGAGAACAUGAU
   ||||||||||:|||| |||||
   CUUCUAAGUCUUCUUUUACUA 5'
AT5G61900.3     3       23
copine - like protein

sRNA_AGO1_Solexa_Mi2008_7_33918_hit1
5' UCUUGACCUUGUAAGACCCCA
   |||||||||||:|||||||
```

```
   AGAACUGGAACGUUCUGGGAA 5'
AT5G62000.3     1709    1729
auxin response factor - like protein

sRNA_AGO1_Solexa_Mi2008_7_49853_hit1
5' UUCUUGACCUUGUAAGACCCCA
   ||||||||||||:|||||||
   AAGAACUGGAACGUUCUGGGAA 5'
AT5G62000.3     1709    1730
auxin response factor - like protein

sRNA_AGO1_Solexa_Mi2008_7_12746_hit1
5' CUUGACCUUGUAAGACCCC
   ||||||||||:|||||||
   GAACUGGAACGUUCUGGGA 5'
AT5G62000.3     1710    1728
auxin response factor - like protein

sRNA_AGO1_Solexa_Mi2008_7_49852_hit1
5' UUCUUGACCUUGUAAGACCCC
   ||||||||||||:|||||||
   AAGAACUGGAACGUUCUGGGA 5'
AT5G62000.3     1710    1730
auxin response factor - like protein

sRNA_AGO1_Solexa_Mi2008_4_55012_hit1
5' UUUCUUGACCUUGUAAGACCCC
    ||||||||||||:|||||||
   UAAGAACUGGAACGUUCUGGGA 5'
AT5G62000.3     1710    1731
auxin response factor - like protein

sRNA_AGO1_Solexa_Mi2008_1_55011_hit1
5' UUUCUUGACCUUGUAAGACCC
    ||||||||||||:|||||||
   UAAGAACUGGAACGUUCUGGG 5'
AT5G62000.3     1711    1731
auxin response factor - like protein

sRNA_AGO1_Solexa_Mi2008_7_33918_hit1
5' UCUUGACCUUGUAAGACCCCA
   |||||||||||:|||||||
   AGAACUGGAACGUUCUGGGAA 5'
AT5G62000.1     1835    1855
auxin response factor - like protein

sRNA_AGO1_Solexa_Mi2008_7_49853_hit1
5' UUCUUGACCUUGUAAGACCCCA
   ||||||||||||:|||||||
   AAGAACUGGAACGUUCUGGGAA 5'
AT5G62000.1     1835    1856
auxin response factor - like protein

sRNA_AGO1_Solexa_Mi2008_7_12746_hit1
5' CUUGACCUUGUAAGACCCC
   ||||||||||:|||||||
   GAACUGGAACGUUCUGGGA 5'
AT5G62000.1     1836    1854
auxin response factor - like protein

sRNA_AGO1_Solexa_Mi2008_7_49852_hit1
5' UUCUUGACCUUGUAAGACCCC
   ||||||||||||:|||||||
```

```
   AAGAACUGGAACGUUCUGGGA 5'
AT5G62000.1     1836    1856
auxin response factor - like protein

sRNA_AGO1_Solexa_Mi2008_4_55012_hit1
5' UUUCUUGACCUUGUAAGACCCC
    ||||||||||||:|||||||
   UAAGAACUGGAACGUUCUGGGA 5'
AT5G62000.1     1836    1857
auxin response factor - like protein

sRNA_AGO1_Solexa_Mi2008_1_55011_hit1
5' UUUCUUGACCUUGUAAGACCC
    ||||||||||||:|||||||
   UAAGAACUGGAACGUUCUGGG 5'
AT5G62000.1     1837    1857
auxin response factor - like protein

sRNA_AGO1_Solexa_Mi2008_1_7208_hit1
5' CAUGCCUCAACUCCCAACUUG
   ||| ||||||| ||||||||:
   GUA-GGAGUUGCGGGUUGAAU 5'
AT5G62190.1     343     362
putative RNA helicase (MMI9.2)

sRNA_AGO1_Solexa_Mi2008_10_35767_hit2
5' UGAGAAAGAUGAGA-UCACA
   :||||||||||||| ||| |
   GCUCUUUCUACUCUUAGUCU 5'
AT5G62530.1     99      118
delta-1-pyrroline-5-carboxylate dehydrogenase precursor (P5CDH)

sRNA_AGO1_Solexa_Mi2008_1_34628_hit3
5' UGAAGAUGGAACCAGCGAAA
    :|||||||| |||||||||
   UUUUCUACCUAGGUCGCUUU 5'
AT5G62680.1     1674    1693
peptide transporter

sRNA_AGO1_Solexa_Mi2008_1_9527_hit1
5' CUAAAGGCGUUAAUGAUGGUU
   |||||||||||||||||||||
   GAUUUCCGCAAUUACUACCAA 5'
AT5G62680.1     381     401
peptide transporter

sRNA_AGO1_Solexa_Mi2008_1_3006_hit1
5' AUGACGUUAAGGGCCAAUGGUA
   ||||||||||||||||||||||
   UACUGCAAUUCCCGGUUACCAU 5'
AT5G63020.1     1045    1066
NBS/LRR disease resistance like protein

sRNA_AGO1_Solexa_Mi2008_5_14408_hit1
5' GCAAGAGU-UCCAUAGUAGCAA
   :||||| | |||||||||||||
   UGUUCUGACAGGUAUCAUCGUU 5'
AT5G63020.1     2096    2117
NBS/LRR disease resistance like protein

sRNA_AGO1_Solexa_Mi2008_1_6562_hit1
5' CAGGGGACCCCGGUGGAUGUG
   |||||||||||||||||||||
```

```
   GUCCCCUGGGGCCACCUACAC 5'
AT5G63020.1     2421    2441
NBS/LRR disease resistance like protein

sRNA_AGO1_Solexa_Mi2008_1_4416_hit1
5' CAACCCACAUCACAAUAUCAA
   ||| ||| |||||||||||||
   GUUAGGUUUAGUGUUAUAGUU 5'
AT5G63020.1     613     633
NBS/LRR disease resistance like protein

sRNA_AGO1_Solexa_Mi2008_3_13626_hit2
5' GACAGACUGA-GAGCUCUUU
   ||||||| || |||||||||
   CUGUCUG-CUUCUCGAGAAA 5'
AT5G63600.2     1460    1478
1-aminocyclopropane-1-carboxylic acid oxidase-like protein

sRNA_AGO1_Solexa_Mi2008_5_36780_hit5
5' UGAGUCUUUGGCUUUGUUUC
   || | ||||||||||||||
   AC-C-GAAACCGAAACAAAC 5'
AT5G63680.1     1294    1311
pyruvate kinase

sRNA_AGO1_Solexa_Mi2008_1_166_hit3
5' AAAAGAAACAGAAGAGAAUGCU
   |||||||| |||||| |||||:
   UUUUCUUUCUCUUCU-UUACGG 5'
AT5G63680.1     1761    1781
pyruvate kinase

sRNA_AGO1_Solexa_Mi2008_6_10055_hit66
5' CUACUGUUGUUUACUGC-AUCUU
   ||||||||||||| ||| |||||
   GAUGACAACAAAU-ACGAUAGAA 5'
AT5G63920.1     2570    2591
DNA topoisomerase III

sRNA_AGO1_Solexa_Mi2008_1_355_hit2
5' AAAGAAGAGAACAAUUAAU
   ||||||||||| || ||||
   UUUCUUCUCUU-UUUAUUA 5'
AT5G64310.1     563     580
arabinogalactan-protein AGP1 (gb|AAC77823.1)

sRNA_AGO1_Solexa_Mi2008_1_340_hit1
5' AAAGAAAAAUGA-UCAAAGU
   |||||||||| | ||||||
   UUUCUUUUUA-UAAGUUUCU 5'
AT5G64310.1     590     608
arabinogalactan-protein AGP1 (gb|AAC77823.1)

sRNA_AGO1_Solexa_Mi2008_1_18966_hit2
5' UAAUUGUUGUUGUGUAUGGA
    |||||||||||||| ||||
   UUUAACAACAACACA-ACCU 5'
AT5G64620.1     114     132
invertase inhibitor homolog (emb|CAA73335.1)

sRNA_AGO1_Solexa_Mi2008_1_351_hit2
5' AAAGAA-GAAAAACAGAUCU
   |||||| |||||||||| |
```

```
   UUUCUUUCUUUUUGUCUAAA 5'
AT5G65020.1     1178    1197
annexin

sRNA_AGO1_Solexa_Mi2008_2_46703_hit1
5' UUAAUCAAGGUCGUACAUUGC
   ||||||||||| |||||||:
   AAUUAGUUCCAUCAUGUAAUU 5'
AT5G65310.2     37      57
homeobox-leucine zipper protein ATHB-5 (HD-zip protein ATHB-5) (sp|P46667)

sRNA_AGO1_Solexa_Mi2008_1_20899_hit1
5' UAGAAGAUAGAGAAUGAUGGA
   :|||||| ||||||||| |||
   GUCUUCUCUCUCUUACU-CCU 5'
AT5G67070.1     126     145
unknown protein

sRNA_AGO1_Solexa_Mi2008_1_35784_hit2
5' UGAGAAGAGAUAGAAU-AGA
   |||||||||| ||||| ||
   ACUCUUCUCUCUCUUACUCC 5'
AT5G67070.1     127     146
unknown protein

sRNA_AGO1_Solexa_Mi2008_3_768_hit1
5' AAGAAGAGAGAGAAUUGAG
    ||||||||||||| ||||
   GUCUUCUCUCUCUU-ACUC 5'
AT5G67070.1     128     145
unknown protein

sRNA_AGO1_Solexa_Mi2008_1_43909_hit1
5' UGUAAAGAGGUGAUUGGGGUU
   |||||||||||||||||||||
   ACAUUUCUCCACUAACCCCAA 5'
AT5G67070.1     4       24
unknown protein

sRNA_AGO1_Solexa_Mi2008_7_12746_hit1
5' CUUGACCUUGUAAG-ACCCC
   ||| |||||||||| ||||
   GAAGUGGAACAUUCUUGGGU 5'
AT5G67370.1     703     722
putative protein

sRNA_AGO1_Solexa_Mi2008_1_431_hit1
5' AAAGAUUGAUGAUAAUGAA
     ||||||||||| |||||
   GCUCUAACUACUACUACUU 5'
AT5G67580.2     1449    1467
telomere repeat binding factor 2 (TRB2)

sRNA_AGO1_Solexa_Mi2008_2_52774_hit1
5' UUGGGUCUAGAAGAAUUC-AGC
   |||||||||||||||:|| ||
   AACCCAGAUCUUCUUGAGCUCU 5'
AT5G67580.2     1464    1485
telomere repeat binding factor 2 (TRB2)

sRNA_AGO1_Solexa_Mi2008_1_431_hit1
5' AAAGAUUGAUGAUAAUGAA
     ||||||||||| |||||
```

```
   GCUCUAACUACUACUACUU 5'
AT5G67580.1     1591    1609
telomere repeat binding factor 2 (TRB2)

sRNA_AGO1_Solexa_Mi2008_2_52774_hit1
5' UUGGGUCUAGAAGAAUUC-AGC
   |||||||||||||||:|| ||
   AACCCAGAUCUUCUUGAGCUCU 5'
AT5G67580.1     1606    1627
telomere repeat binding factor 2 (TRB2)

sRNA_AGO1_Solexa_Mi2008_1_50655_hit1
5' UUGAGGAGCGUUCUGGUCUUG
   ||||||||| |||| ||||||
   AACUCCUCGGAAGA-CAGAAC 5'
AT5G67580.2     699     718
telomere repeat binding factor 2 (TRB2)

sRNA_AGO1_Solexa_Mi2008_1_50655_hit1
5' UUGAGGAGCGUUCUGGUCUUG
   ||||||||| |||| ||||||
   AACUCCUCGGAAGA-CAGAAC 5'
AT5G67580.1     841     860
telomere repeat binding factor 2 (TRB2)
```

```
sRNA_AGO1_Solexa_Mi2008_2_19092_hit1
5' UACAACAGGAAGGUCGAUGGUU
   ||||||||||||||||||||||
   AUGUUGUCCUUCCAGCUACCAA 5'
AT1G01470.1     487     508
unknown protein

sRNA_AGO1_Solexa_Mi2008_3_768_hit1
5' AAGA-AGAGAGAGAAUUGAG
    ||| |||||||||| ||||
   GUCUCUCUCUCUCUUUACUC 5'
AT1G01490.1     191     210
unknown protein

sRNA_AGO1_Solexa_Mi2008_2_13419_hit2
5' GAAGAAGAAGAAGACACUU
   ||||||||||||||||  :
   CUUCUUCUUCUUCUGUUCG 5'
AT1G04310.1     206     224
putative ethylene receptor ERS2

sRNA_AGO1_Solexa_Mi2008_1_13420_hit2
5' GAAGAAGAAGAAGACUCUU
   |||||||||||||  |||
   CUUCUUCUUCUUC--AGAU 5'
AT1G04310.1     90      106
putative ethylene receptor ERS2

sRNA_AGO1_Solexa_Mi2008_1_49223_hit1
5' UUCGAGAUCAGGUACACCGGA
    ||||||||||| ||| ||||
   CAGCUCUAGUCCCUGUAGCCU 5'
AT1G05010.1     463     483
1-aminocyclopropane-1-carboxylate oxidase

sRNA_AGO1_Solexa_Mi2008_3_36293_hit2
5' UGAGGAGGAAGAUGG-UUUGGAU
   ||||||||||| ||| ||||||:
   ACUCCUCCUUCCACCAAAACCUG 5'
AT1G06620.1     169     191
oxidoreductase-like protein

sRNA_AGO1_Solexa_Mi2008_2_36292_hit2
5' UGAGGAGGAAGAUGG-UUUGGA
   ||||||||||| ||| ||||||
   ACUCCUCCUUCCACCAAAACCU 5'
AT1G06620.1     170     191
oxidoreductase-like protein

sRNA_AGO1_Solexa_Mi2008_18_6978_hit1
5' CAUCAACUUC-AGGAUUCGCCA
   ||| |||| | |||||||||||
   GUAUUUGA-GCUCCUAAGCGGU 5'
AT1G06620.1     964     984
oxidoreductase-like protein

sRNA_AGO1_Solexa_Mi2008_23_8104_hit1
5' CCGUGAGCCAUCUCU-UAGAUCU
   |||||||| |||||| | |||||
   GGCACUCGUUAGAGACA-CUAGA 5'
AT1G07240.1     1091    1112
unknown protein
```

```
sRNA_AGO1_Solexa_Mi2008_2_45261_hit1
5' UGUGGACCAAGAUAUCCAGUUG
   ||||| |||||||||||| |||
   ACACCCGGUUCUAUAGGUGAAC 5'
AT1G07240.1     1248    1269
unknown protein

sRNA_AGO1_Solexa_Mi2008_1_34289_hit2
5' UGAAAUCGAUGUUGUAAGUCC
   ||||||||| | ||||||||
   ACUUUAGCUCCUACAUUCAGA 5'
AT1G07240.1     454     474
unknown protein

sRNA_AGO1_Solexa_Mi2008_9_14254_hit8
5' GAUGAUGAUGAUGAUGAUCUU
   |||||||||||||| || ||
   CUACUACUACUACU-CU-GAU 5'
AT1G07240.1     518     536
unknown protein

sRNA_AGO1_Solexa_Mi2008_1_7931_hit2
5' CCCGAUGAUGAUGAUGAGAC
      ||||||||||||||||
   CUUCUACUACUACUACUCUG 5'
AT1G07240.1     519     538
unknown protein

sRNA_AGO1_Solexa_Mi2008_1_34723_hit1
5' UGAAGGAAGGUAUACCUAACGGCC
    |||||||||| ||||| ||||||
   CCUUCCUUCCAAAUGGAGUGCCGG 5'
AT1G07240.1     606     629
unknown protein

sRNA_AGO1_Solexa_Mi2008_1_53805_hit1
5' UUGUUGGUAC-AGAAAGACAUG
   ||||||| |  |||||||||||
   AACAACC-UCAUCUUUCUGUAC 5'
AT1G07350.1     1366    1386
transformer-SR ribonucleoprotein like protein

sRNA_AGO1_Solexa_Mi2008_3_34916_hit1
5' UGAAGUUGAAGAAGGAA-UCAA
   |||||||||||| | || ||||
   ACUUCAACUUCUGCAUUGAGUU 5'
AT1G08630.5     34      55
unknown protein

sRNA_AGO1_Solexa_Mi2008_3_34916_hit1
5' UGAAGUUGAAGAAGGAA-UCAA
   |||||||||||| | || ||||
   ACUUCAACUUCUGCAUUGAGUU 5'
AT1G08630.4     3       24
unknown protein

sRNA_AGO1_Solexa_Mi2008_8_56168_hit1
5' UUUGUGUUCUCAGGUCACCCCUU
   |||||| |||||||  |||||||
   AAACACUAGAGUCCUUUGGGGAA 5'
AT1G08830.1     116     138
superoxidase dismutase
```

```
sRNA_AGO1_Solexa_Mi2008_1_13516_hit1
5' GAAGGUGACAAAGUCAGACAA
    ||||||||||||||| |||
   AUUCCACUGUUUCAGU-UGUG 5'
AT1G09070.1     1240    1259
unknown protein

sRNA_AGO1_Solexa_Mi2008_1_29353_hit1
5' UCCACCCUGUUGCUGAGGCGGA
   ||||||||||||||||||||||
   AGGUGGGACAACGACUCCGCCU 5'
AT1G09070.1     753     774
unknown protein

sRNA_AGO1_Solexa_Mi2008_4_11921_hit1
5' CUGGAUCGAAGCAUUUCUC
    |||| ||||||||||||
   AACCUUGCUUCGUAAAGAA 5'
AT1G12010.1     272     290
putative amino-cyclopropane-carboxylic acid oxidase (ACC oxidase)

sRNA_AGO1_Solexa_Mi2008_2_10859_hit1
5' CUCCAUCUCUCUCUCUGCUU
   |||||||||||||||| ||:
   GAGGUAGAGAGAGAGA-GAG 5'
AT1G12010.1     55      73
putative amino-cyclopropane-carboxylic acid oxidase (ACC oxidase)

sRNA_AGO1_Solexa_Mi2008_1_6621_hit1
5' CA-GG-UGAAGAAGAUAACGU
   || || ||||||||| |||||
   GUACCUACUUCUUCUCUUGCA 5'
AT1G13340.1     296     316
unknown protein

sRNA_AGO1_Solexa_Mi2008_1_8242_hit1
5' CGAAAACAGAUCGCCGGAGUC
   |||||||||||||||||||||
   GCUUUUGUCUAGCGGCCUCAG 5'
AT1G13360.1     614     634
unknown protein (At1g13360)

sRNA_AGO1_Solexa_Mi2008_7_13130_hit1
5' CUUUAACAAAACGAUAUCGUU
   |||||||||||||||||||||
   GAAAUUGUUUUGCUAUAGCAA 5'
AT1G13360.1     705     725
unknown protein (At1g13360)

sRNA_AGO1_Solexa_Mi2008_3_5252_hit2
5' CACAAAACCGCAGCUACAU-UAG
   || ||||| |||||||||| |||
   GU-UUUUGACGUCGAUGUACAUC 5'
AT1G14480.1     268     289
hypothetical protein

sRNA_AGO1_Solexa_Mi2008_1_8623_hit1
5' CGAGGUGGCUAUGAGCGGUCG
   |||||||||||||||||||||
   GCUCCACCGAUACUCGCCAGC 5'
AT1G14920.1     1745    1765
signal response protein (GAI)
```

```
sRNA_AGO1_Solexa_Mi2008_9_14254_hit8
5' GAUGAUGAUGAUGAUGAUCUU
   ||||||||||||||||||||:
   CUACUACUACUACUACUAGAG 5'
AT1G14920.1     195     215
signal response protein (GAI)

sRNA_AGO1_Solexa_Mi2008_6_14255_hit369
5' GAUGAUGAUGAUGAUGAUGAUGAU
   |||||||||||||||||||||  :
   CUACUACUACUACUACUACUAGAG 5'
AT1G14920.1     195     218
signal response protein (GAI)

sRNA_AGO1_Solexa_Mi2008_1_14256_hit8
5' GAUGAUGAUGAUGAUGAUGAUGUU
   ||||||||||||||||||||| |:
   CUACUACUACUACUACUACUAGAG 5'
AT1G14920.1     195     218
signal response protein (GAI)

sRNA_AGO1_Solexa_Mi2008_1_36323_hit13
5' UGAGGAUGAUGAUGAUGAUGA
   ||| |||||||||||||||
   ACUACUACUACUACUACUAGA 5'
AT1G14920.1     196     216
signal response protein (GAI)

sRNA_AGO1_Solexa_Mi2008_1_3079_hit472
5' AUGAUGAUGAUGAUGAUGAUGA
   ||||||||||||||||||||
   UACUACUACUACUACUACUAGA 5'
AT1G14920.1     196     217
signal response protein (GAI)

sRNA_AGO1_Solexa_Mi2008_1_45039_hit3
5' UG-UGAUGAUGAUGAUGAUGAUGA
   || |||||||||||||||||||
   ACUACUACUACUACUACUACUAGA 5'
AT1G14920.1     197     220
signal response protein (GAI)

sRNA_AGO1_Solexa_Mi2008_9_14254_hit8
5' GAUGAUGAUGAUGAUGAUCUU
   ||||||||||||||||||  |
   CUACUACUACUACUACUACUA 5'
AT1G14920.1     198     218
signal response protein (GAI)

sRNA_AGO1_Solexa_Mi2008_6_14255_hit369
5' GAUGAUGAUGAUGAUGAUGAUGAU
     ||||||||||||||||||||||
   GAACUACUACUACUACUACUACUA 5'
AT1G14920.1     198     221
signal response protein (GAI)

sRNA_AGO1_Solexa_Mi2008_7_3078_hit1
5' AUGAUGAUGA-GAAUGAUGAU
   |||||||||| || |||||||
   UACUACUACUACU-ACUACUA 5'
AT1G14920.1     199     218
signal response protein (GAI)
```

```
sRNA_AGO1_Solexa_Mi2008_1_36323_hit13
5' UGAGGAUGAUGAUGAUGAUGA
   ||| |||||||||||||||||
   ACUACUACUACUACUACUACU 5'
AT1G14920.1     199     219
signal response protein (GAI)

sRNA_AGO1_Solexa_Mi2008_1_3079_hit472
5' AUGAUGAUGAUGAUGAUGAUGA
    |||||||||||||||||||||
   AACUACUACUACUACUACUACU 5'
AT1G14920.1     199     220
signal response protein (GAI)

sRNA_AGO1_Solexa_Mi2008_1_45039_hit3
5' UGUGAUGAUGAUGAUGAUGAUGA
   : |||||||||||||||||||||
   GAACUACUACUACUACUACUACU 5'
AT1G14920.1     199     221
signal response protein (GAI)

sRNA_AGO1_Solexa_Mi2008_1_56092_hit3
5' UUUG-UGAUGAUGAUGAUGAUGA
    ||| ||||||||||||||||||
   CAACUACUACUACUACUACUACU 5'
AT1G14920.1     200     222
signal response protein (GAI)

sRNA_AGO1_Solexa_Mi2008_7_3078_hit1
5' AUGAUGAUGA-GAAUGAUGAU
   |||||||||| || |||||||
   UACUACUACUACU-ACUACUA 5'
AT1G14920.1     202     221
signal response protein (GAI)

sRNA_AGO1_Solexa_Mi2008_1_13731_hit1
5' GACGAAGAAGCCGAAUCGA
   |||||||||||||||||||
   CUGCUUCUUCGGCUUAGCU 5'
AT1G14920.1     534     552
signal response protein (GAI)

sRNA_AGO1_Solexa_Mi2008_5_24931_hit2
5' UAUCAUUGUGAAGCAGAAUUC
   ||||||||||||  |||||||
   AUAGUAACACUUA-UCUUAAG 5'
AT1G16950.1     408     427
unknown protein

sRNA_AGO1_Solexa_Mi2008_1_4590_hit1
5' CAAGACAAAGAUGGUAUUG
   |||||||||||||| || :
   GUUCUGUUUCUACC-UACU 5'
AT1G17210.1     2517    2534
hypothetical protein

sRNA_AGO1_Solexa_Mi2008_2_39987_hit1
5' UGGACCAUGAGGAUGGAGGCU
   ||||||||| ||| |||||||
   ACCUGGUACCCCUCCCUCCGA 5'
AT1G17420.1     1694    1714
lipoxygenase
```

```
sRNA_AGO1_Solexa_Mi2008_4_24200_hit5
5' UAUAAACGAAGGAAUUUGUA
   ||||| |||||:|||||||
   AUAUU-GCUUCUUUAAACAG 5'
AT1G18470.1     107     125
unknown protein

sRNA_AGO1_Solexa_Mi2008_9_6689_hit1
5' CAGUCAGAAGUUUGGGGAA
   || ||||||||||||| ||
   GU-AGUCUUCAAACCC-UU 5'
AT1G18470.1     1623    1639
unknown protein

sRNA_AGO1_Solexa_Mi2008_9_6689_hit1
5' CAGUCAGAAGUUUGGGGAA
   || ||||||||||| ||||
   GU-AGUCUUCAAAC-CCUU 5'
AT1G18470.2     1741    1757
unknown protein

sRNA_AGO1_Solexa_Mi2008_1_19649_hit1
5' UACCAAAAAUGAGAUGGACUA
   ||| ||||| |||||||| ||
   AUGUUUUU-CUCUACCU-AU 5'
AT1G18470.1     1770    1788
unknown protein

sRNA_AGO1_Solexa_Mi2008_1_19649_hit1
5' UACCAAAAAUGAGAUGGACUA
   ||| ||||| |||||||| ||
   AUGUUUUU-CUCUACCU-AU 5'
AT1G18470.2     1888    1906
unknown protein

sRNA_AGO1_Solexa_Mi2008_1_40868_hit1
5' UGGAUGAACUUGAUUGGACCAU
   ||||| | || |||||||||||
   ACCUAAU-GACCUAACCUGGUA 5'
AT1G18470.2     709     729
unknown protein

sRNA_AGO1_Solexa_Mi2008_1_40868_hit1
5' UGGAUGAACUUGAUUGGACCAU
   ||||| | || |||||||||||
   ACCUAAU-GACCUAACCUGGUA 5'
AT1G18470.1     735     755
unknown protein

sRNA_AGO1_Solexa_Mi2008_4_24200_hit5
5' UAUAAACGAAGGAAUUUGUA
   ||||| |||||:|||||||
   AUAUU-GCUUCUUUAAACAG 5'
AT1G18470.2     81      99
unknown protein

sRNA_AGO1_Solexa_Mi2008_1_40_hit1
5' AAAAAACACAACACAAAAAU
   |||||| | ||||||||||:
   UUUUUU-U-UUGUGUUUUG 5'
AT1G18570.1     1571    1588
unknown protein
```

```
sRNA_AGO1_Solexa_Mi2008_1_40_hit1
5' AAAAAACACAACACAAAAAU
   ||||||||||| | |||||
   UUUUUUGUGUU-U-UUUUUU 5'
AT1G18570.1     264     281
unknown protein

sRNA_AGO1_Solexa_Mi2008_1_35874_hit2
5' UGAGAGACA-AAAUGAGAAGGU
   ||||||||| ||| ||||||||
   ACUCUCUGUGUUU-CUCUUCCA 5'
AT1G18570.1     48      68
unknown protein

sRNA_AGO1_Solexa_Mi2008_1_6226_hit1
5' CAGCAAAAGAAUCAG--GAAU
   ||||||||| |||||  ||||
   GUCGUUUUCCUAGUCUUCUUA 5'
AT1G18590.1     1138    1158
unknown protein

sRNA_AGO1_Solexa_Mi2008_1_6801_hit1
5' CAUAAACAGCAGGACGAUCCU
   ||||||||||||||||||| |
   GUAUUUGUCGUCCUGCUAGAA 5'
AT1G18590.1     940     960
unknown protein

sRNA_AGO1_Solexa_Mi2008_4_42709_hit1
5' UGGGUGAUGAUGAUGAUUGAU
    |:||||||||||||||||||
   UCUCACUACUACUACUAACUA 5'
AT1G18740.1     0       20
unknown protein

sRNA_AGO1_Solexa_Mi2008_1_36884_hit32
5' UGAGUUGAUGAGUCAA-UUGGU
   |||||||||||||||| |||||
   ACUCAACUACUCAGUUGAACCA 5'
AT1G18740.1     190     211
unknown protein

sRNA_AGO1_Solexa_Mi2008_1_21856_hit3
5' UAGAUGGGAAUCUCUCUGAUG
   |||||||||||||||||||||
   AUCUACCCUUAGAGAGACUAC 5'
AT1G18740.1     1939    1959
unknown protein

sRNA_AGO1_Solexa_Mi2008_3_37174_hit2
5' UGAUGACUAAUGUCUAGAUGG
   |||||||||||||||||||||
   ACUACUGAUUACAGAUCUACC 5'
AT1G18740.1     1953    1973
unknown protein

sRNA_AGO1_Solexa_Mi2008_1_21856_hit3
5' UAGAUGGGAAUCUCUCUGAUG
   |||||||||||||||||||||
   AUCUACCCUUAGAGAGACUAC 5'
AT1G18740.1     2102    2122
unknown protein
```

```
sRNA_AGO1_Solexa_Mi2008_3_37174_hit2
5' UGAUGACUAAUGUCUAGAUGG
   |||||||||||||||||||||
   ACUACUGAUUACAGAUCUACC 5'
AT1G18740.1     2116    2136
unknown protein

sRNA_AGO1_Solexa_Mi2008_1_37815_hit1
5' UGCAAGUGAUGAUAAAAAGA
   ||||||||||||| ||||||
   ACGUUCACUACUACUUUUCU 5'
AT1G18740.1     2173    2192
unknown protein

sRNA_AGO1_Solexa_Mi2008_1_80_hit1
5' AAAAAAUCAACUCUGAGGCA
   ||||||||||||||||||||
   UUUUUUAGUUGAGACUCCGU 5'
AT1G18740.1     2310    2329
unknown protein

sRNA_AGO1_Solexa_Mi2008_1_33_hit1
5' AAAAAAAUCAACUCUGAGGCA
   |||||||||||||||||||||
   UUUUUUUAGUUGAGACUCCGU 5'
AT1G18740.1     2310    2330
unknown protein

sRNA_AGO1_Solexa_Mi2008_1_13420_hit2
5' GAAGAAGAAGAAGACUCUU
    || |||||||||||| ||
   AUU-UUCUUCUUCUGA-AA 5'
AT1G18740.1     45      61
unknown protein

sRNA_AGO1_Solexa_Mi2008_1_50511_hit7
5' UUGAGAGACAGGGAAGAUGAUGAU
   |||||||| || || |||||||||
   AACUCUCUCUCACUACUACUACUA 5'
AT1G18740.1     4       27
unknown protein

sRNA_AGO1_Solexa_Mi2008_2_50515_hit4
5' UUGAGAGAUAGGGAAGAUGAUGAU
   |||||||| || || |||||||||
   AACUCUCUCUCACUACUACUACUA 5'
AT1G18740.1     4       27
unknown protein

sRNA_AGO1_Solexa_Mi2008_1_42552_hit1
5' UGGGGUUGGUUGG-UUGGUUGG
   ||||||||||||| |||||||
   ACCCCAACCAACCAAACCAACG 5'
AT1G19180.1     27      48
unknown protein

sRNA_AGO1_Solexa_Mi2008_1_42552_hit1
5' UGGGGUUGGUUGG-UUGGUUGG
   ||||||||||||| |||||||
   ACCCCAACCAACCAAACCAACG 5'
AT1G19180.2     7       28
unknown protein
```

```
sRNA_AGO1_Solexa_Mi2008_6_14778_hit1
5' GCUGACCAUUUGGCCGAUCCAGUU
   ||||||||||||||| || ||||
   CGACUGGUAAACCGGCCAGAUCAA 5'
AT1G19660.2     1002    1025
unknown protein

sRNA_AGO1_Solexa_Mi2008_12_5493_hit2
5' CACCCAUUUGAUACAUAGU
   |||| |:||||||||||||
   GUGG-UGAACUAUGUAUCA 5'
AT1G19660.1     660     677
unknown protein

sRNA_AGO1_Solexa_Mi2008_12_5493_hit2
5' CACCCAUUUGAUACAUAGU
   |||| |:||||||||||||
   GUGG-UGAACUAUGUAUCA 5'
AT1G19660.2     705     722
unknown protein

sRNA_AGO1_Solexa_Mi2008_1_8820_hit17
5' CGCCUCGUGCUCUCUUGUGGU
    ||||||||  ||||||||||
   ACGGAGCACUUGAGAACACCA 5'
AT1G19660.1     725     745
unknown protein

sRNA_AGO1_Solexa_Mi2008_1_8820_hit17
5' CGCCUCGUGCUCUCUUGUGGU
    ||||||||  ||||||||||
   ACGGAGCACUUGAGAACACCA 5'
AT1G19660.2     770     790
unknown protein

sRNA_AGO1_Solexa_Mi2008_6_14778_hit1
5' GCUGACCAUUUGGCCGAUCCAGUU
   ||||||||||||||| || ||||
   CGACUGGUAAACCGGCCAGAUCAA 5'
AT1G19660.1     957     980
unknown protein

sRNA_AGO1_Solexa_Mi2008_3_2080_hit1
5' AGCGAUGAUGAC-AAUGAUGA
    ||||||||||| || |||||
   ACGCUACUACUGCUU-CUACU 5'
AT1G19770.1     237     256
unknown protein

sRNA_AGO1_Solexa_Mi2008_1_23826_hit1
5' UAGUGAAGAAGAGCUAUGCAUG
   :|||||||||||||| ||| ||
   GUCACUUCUUCUCGACACG-AC 5'
AT1G19770.1     582     602
unknown protein

sRNA_AGO1_Solexa_Mi2008_2_3216_hit4
5' AUGGAAGCUGCGAAAAUA-GAG
   |||:|||||||||| ||| |||
   UACUUUCGACGCUUCUAUCCUC 5'
AT1G19770.1     600     621
unknown protein
```

```
sRNA_AGO1_Solexa_Mi2008_1_53503_hit1
5' UUGUCUGAGCAACGCGAUUGA
   |||||||||||||||||||||
   AACAGACUCGUUGCGCUAACU 5'
AT1G20070.1     581     601
unknown protein

sRNA_AGO1_Solexa_Mi2008_1_37225_hit4
5' UGAUGAUGAUGAUGAU-GA-AGAAG
   |||||||||||||||| ||  ||||
   ACUACUACUACUACUAGCUCACUUC 5'
AT1G20190.1     55      79
Expansin (AtEXPA11)

sRNA_AGO1_Solexa_Mi2008_6_14255_hit369
5' GAUGAUGAUGAUGAUGAU-GA-UGAU
   |||||||||||||||||| || |||
   CUACUACUACUACUACUAGCUCACUU 5'
AT1G20190.1     56      81
Expansin (AtEXPA11)

sRNA_AGO1_Solexa_Mi2008_1_36323_hit13
5' UGAGGAUGAUGAUGAU-GA-UGA
   ||| |||||||||||| || |||
   ACUACUACUACUACUAGCUCACU 5'
AT1G20190.1     57      79
Expansin (AtEXPA11)

sRNA_AGO1_Solexa_Mi2008_1_3079_hit472
5' AUGAUGAUGAUGAUGAU-GA-UGA
   ||||||||||||||||| || |||
   UACUACUACUACUACUAGCUCACU 5'
AT1G20190.1     57      80
Expansin (AtEXPA11)

sRNA_AGO1_Solexa_Mi2008_9_14254_hit8
5' GAUGAUGAUGAUGAUGAUCUU
   |||||||||||||||||||
   CUACUACUACUACUACUAGCU 5'
AT1G20190.1     60      80
Expansin (AtEXPA11)

sRNA_AGO1_Solexa_Mi2008_1_3079_hit472
5' AUGAUGAUGAUGAUGAUGAU-GA
    ||||||||||||||||||| ||
   GACUACUACUACUACUACUAGCU 5'
AT1G20190.1     61      83
Expansin (AtEXPA11)

sRNA_AGO1_Solexa_Mi2008_1_45039_hit3
5' UGUGAUGAUGAUGAUGAUGAU-GA
   | ||||||||||||||||||| ||
   AGACUACUACUACUACUACUAGCU 5'
AT1G20190.1     61      84
Expansin (AtEXPA11)

sRNA_AGO1_Solexa_Mi2008_1_2280_hit1
5' AG-UGGAUGAUGAUGAUGAUG
   || || ||||||||||||||
   UCUAC-UACUACUACUACUAG 5'
AT1G20190.1     63      82
Expansin (AtEXPA11)
```

```
sRNA_AGO1_Solexa_Mi2008_7_3078_hit1
5' AUGAUGAUGA-GAAUGAUGAU
    ||||||||| || |||||||
   GACUACUACUACU-ACUACUA 5'
AT1G20190.1     64      83
Expansin (AtEXPA11)

sRNA_AGO1_Solexa_Mi2008_1_36898_hit1
5' UGAGUUGGAUGAUGAUGAUGA
   |||| || |||||||||||||
   ACUCUAC-UACUACUACUACU 5'
AT1G20190.1     65      84
Expansin (AtEXPA11)

sRNA_AGO1_Solexa_Mi2008_1_39699_hit1
5' UGGA-ACUGAUGAUGAUGAUGA
   | || | |||||||||||||||
   AACUCU-ACUACUACUACUACU 5'
AT1G20190.1     65      85
Expansin (AtEXPA11)

sRNA_AGO1_Solexa_Mi2008_1_36323_hit13
5' UGAG-GAUGAUGAUGAUGAUGA
   | || |||||||||||||||||
   AAUCUCUACUACUACUACUACU 5'
AT1G20190.1     65      86
Expansin (AtEXPA11)

sRNA_AGO1_Solexa_Mi2008_1_13759_hit1
5' GACGAUGAUGAUGAUGACA
   || ||||||||||||||
   CU-CUACUACUACUACUAC 5'
AT1G20190.1     66      83
Expansin (AtEXPA11)

sRNA_AGO1_Solexa_Mi2008_5_13760_hit2
5' GACGAUGAUGAUGAUGAGC
   || ||||||||||||||
   CU-CUACUACUACUACUAC 5'
AT1G20190.1     66      83
Expansin (AtEXPA11)

sRNA_AGO1_Solexa_Mi2008_3_13421_hit1
5' GAAGAAGA-AGAUGAUGUUGAU
   |||||| | |||||||| ||||
   CUUCUU-UCUCUACUACUACUA 5'
AT1G20190.1     70      90
Expansin (AtEXPA11)

sRNA_AGO1_Solexa_Mi2008_1_36931_hit2
5' UGAUAACCGUAGA-GCCGAUGG
   ||||||||| ||| ||||||||
   ACUAUUGGC-UCUUCGGCUACC 5'
AT1G21270.1     1076    1096
putative protein

sRNA_AGO1_Solexa_Mi2008_2_35299_hit10
5' UGACAUCAACAUUUAAUGGCC
   |||||||||||||| ||||||
   ACUGUAGUUGUAAACUACCGG 5'
AT1G21270.1     1213    1233
putative protein
```

```
sRNA_AGO1_Solexa_Mi2008_3_20869_hit2
5' UA-GAACUUGAUUAUUCAUGAU
   || |||| |||||||| |||||
   AUACUUGUACUAAUAACUACUA 5'
AT1G21270.1     2369    2390
putative protein

sRNA_AGO1_Solexa_Mi2008_1_13420_hit2
5' GAAGAAGAAGAAGA-CUCUU
   ||||||||||||||  ||||
   CUUCUUCUUCUUCUCAAGAA 5'
AT1G22530.1     34      53
unknown protein

sRNA_AGO1_Solexa_Mi2008_1_43580_hit1
5' UGGUGGUGGUGGCGGCGCUGGU
   |||||||||:|| ||||| |||
   ACCACCACCGCCACCGCG-CCA 5'
AT1G22530.1     459     479
unknown protein

sRNA_AGO1_Solexa_Mi2008_11_14876_hit4
5' GGAGGUGGUGGUGGUGGUGGU
   |||| |||||||||:|||||:
   CCUCGACCACCACCGCCACCG 5'
AT1G22530.1     463     483
unknown protein

sRNA_AGO1_Solexa_Mi2008_1_3_hit25
5' AAAAAAAAA-A-AAAGAAAGA
   ||||||||| | |||||||:|
   UUUUUUUUUCUCUUUCUUUUU 5'
AT1G23000.1     1385    1405
unknown protein

sRNA_AGO1_Solexa_Mi2008_1_44585_hit1
5' UGUCCACCAUU-GAUGUUCUGC
   |||| ||| || ||||||||||
   ACAG-UGGAAAACUACAAGACG 5'
AT1G23000.1     349     369
unknown protein

sRNA_AGO1_Solexa_Mi2008_1_37103_hit1
5' UGAUCGCUCAGGUCUGGUGGG
   |||||||||||||||||||||
   ACUAGCGAGUCCAGACCACCC 5'
AT1G27340.1     1243    1263
unknown protein

sRNA_AGO1_Solexa_Mi2008_1_55653_hit2
5' UUUGGCAUUCUGUCCACCUCC
   |||||||||||||| ||||||
   AAACCGUAAGACAGUUGGAGG 5'
AT1G27340.1     1372    1392
unknown protein

sRNA_AGO1_Solexa_Mi2008_1_43831_hit1
5' UGGUUGUGGCUC-UGGUGGUAA
   |||| ||||| | |||||||||
   ACCACCACCG-GCACCACCAUU 5'
AT1G28290.2     158     178
proline-rich protein, putative
```

```
sRNA_AGO1_Solexa_Mi2008_1_35934_hit2
5' UGAG-AGUGAUGUGGGUGGUGGU
   |||| || | |||||||||||||
   ACUCCUCCC-ACACCCACCACCA 5'
AT1G28290.2     172     193
proline-rich protein, putative

sRNA_AGO1_Solexa_Mi2008_1_43831_hit1
5' UGGUUGUGGCUC-UGGUGGUAA
   |||| ||||| | |||||||||
   ACCACCACCG-GCACCACCAUU 5'
AT1G28290.1     276     296
proline-rich protein, putative

sRNA_AGO1_Solexa_Mi2008_1_35934_hit2
5' UGAG-AGUGAUGUGGGUGGUGGU
   |||| || | |||||||||||||
   ACUCCUCCC-ACACCCACCACCA 5'
AT1G28290.1     290     311
proline-rich protein, putative

sRNA_AGO1_Solexa_Mi2008_1_43744_hit2
5' UGGUUAGAGGGUAAAUUGGUC
   ||||| |||||||||:||||
   ACCAACCUCCCAUUUGACCAC 5'
AT1G28290.2     396     416
proline-rich protein, putative

sRNA_AGO1_Solexa_Mi2008_1_43744_hit2
5' UGGUUAGAGGGUAAAUUGGUC
   ||||| |||||||||:||||
   ACCAACCUCCCAUUUGACCAC 5'
AT1G28290.2     456     476
proline-rich protein, putative

sRNA_AGO1_Solexa_Mi2008_1_43744_hit2
5' UGGUUAGAGGGUAAAUUGGUC
   ||||| |||||||||:||||
   ACCAACCUCCCAUUUGACCAC 5'
AT1G28290.1     514     534
proline-rich protein, putative

sRNA_AGO1_Solexa_Mi2008_1_43744_hit2
5' UGGUUAGAGGGUAAAUUGGUC
   ||||| |||||||||:||||
   ACCAACCUCCCAUUUGACCAC 5'
AT1G28290.1     574     594
proline-rich protein, putative

sRNA_AGO1_Solexa_Mi2008_1_43744_hit2
5' UGGUUAGAGGGUAAAUUGGUC
   ||||| |||||||||:||||
   ACCAACCUCCCAUUUGACCAC 5'
AT1G28290.1     634     654
proline-rich protein, putative

sRNA_AGO1_Solexa_Mi2008_1_336_hit2
5' AAAGAAAAAAAAAGUAUUGA
   ||| ||||||||| |||||
   UUU-UUUUUUUUUAAUAACC 5'
AT1G28370.1     687     705
putative ethylene responsive element binding factor 4 protein
```

```
sRNA_AGO1_Solexa_Mi2008_1_1776_hit10
5' AGAAAAAAAAAAAAAAAAAAU
   |  ||||||||||||||||||
   UGGUUUUUUUUUUUUUUUUUA 5'
AT1G28370.1     692     712
putative ethylene responsive element binding factor 4 protein

sRNA_AGO1_Solexa_Mi2008_1_3_hit25
5' AAAAAAAAAAAAAGAAAGA
   ||||||||||||| |||:
   UUUUUUUUUUUUU-UUUUA 5'
AT1G28370.1     693     710
putative ethylene responsive element binding factor 4 protein

sRNA_AGO1_Solexa_Mi2008_3_8177_hit1
5' CCUGA-UAAAGUAAAAUCUCCAU
   ||||| |||||||  ||||||||
   GGACUUAUUUCAUAAUAGAGGUA 5'
AT1G28370.1     950     972
putative ethylene responsive element binding factor 4 protein

sRNA_AGO1_Solexa_Mi2008_1_1549_hit1
5' ACC-GGAGAAGAAGAAGAUGAC
   ||| ||||| |||||||||||
   UGGUCCUCU-CUUCUUCUACUA 5'
AT1G30220.1     1422    1442
unknown protein

sRNA_AGO1_Solexa_Mi2008_1_47831_hit2
5' UUAGGGUUAAAUGAGUUAUGA
   || |||||||| |||||| ||
   AA-CCCAAUUUUCUCAAU-CU 5'
AT1G30220.1     8       26
unknown protein

sRNA_AGO1_Solexa_Mi2008_1_28030_hit4
5' UCAGAAGAAGCCAC-GUCAGAUGA
   ||||||||||  || |||||||||
   AGUCUUCUUCU-UGGCAGUCUACU 5'
AT1G35210.1     259     281
unknown protein

sRNA_AGO1_Solexa_Mi2008_1_919_hit1
5' AAGGCUAAUGUUGUGAUCUUC
    |||| | |||||||||||||
   GUCCG-U-ACAACACUAGAAG 5'
AT1G35310.1     137     155
unknown protein

sRNA_AGO1_Solexa_Mi2008_1_14083_hit2
5' GAGGCCAAUGUUGUGAUCUA
   ||||| | |||||||||||
   CUCCG-U-ACAACACUAGAA 5'
AT1G35310.1     138     155
unknown protein

sRNA_AGO1_Solexa_Mi2008_1_26497_hit1
5' UAUUUGUGA-GAGUUCGUGAAA
   | ||| ||| ||||||||||||
   AGAAA-ACUACUCAAGCACUUU 5'
AT1G35710.1     129     149
protein kinase, putative
```

```
sRNA_AGO1_Solexa_Mi2008_1_19304_hit2
5' UACACGUUCAUCGGAAAUGCU
   |||||||||||||||||||||
   AUGUGCAAGUAGCCUUUACGA 5'
AT1G35710.1     3219    3239
protein kinase, putative

sRNA_AGO1_Solexa_Mi2008_2_5215_hit1
5' CAAUUAG-UGGAGAAGUUCA
    |||| | ||||||||||||
   AUUAA-CUACCUCUUCAAGU 5'
AT1G35710.1     3368    3386
protein kinase, putative

sRNA_AGO1_Solexa_Mi2008_10_17146_hit1
5' UAACAACAACAAC-AAAGGUGAA
   ||||||||||||| |||| ||||
   AUUGUUGUUGUUGUUUUCGACUU 5'
AT1G35710.1     3487    3509
protein kinase, putative

sRNA_AGO1_Solexa_Mi2008_2_35306_hit1
5' UGACAUCGAUGA-UUGGUUUCG
   |||||| || || |||||||||
   ACUGUAUCU-CUUAACCAAAGC 5'
AT1G44750.2     336     356
Unknown protein (At1g44750)

sRNA_AGO1_Solexa_Mi2008_2_35306_hit1
5' UGACAUCGAUGA-UUGGUUUCG
   |||||| || || |||||||||
   ACUGUAUCU-CUUAACCAAAGC 5'
AT1G44750.3     88      108
Unknown protein (At1g44750)

sRNA_AGO1_Solexa_Mi2008_2_44202_hit1
5' UGUAGGACGAAUGCUUUGGUA
   |||||||||||||||||||||
   ACAUCCUGCUUACGAAACCAU 5'
AT1G48410.1     1250    1270
Argonaute protein (AGO1)

sRNA_AGO1_Solexa_Mi2008_2_44202_hit1
5' UGUAGGACGAAUGCUUUGGUA
   |||||||||||||||||||||
   ACAUCCUGCUUACGAAACCAU 5'
AT1G48410.2     1256    1276
Argonaute protein (AGO1)

sRNA_AGO1_Solexa_Mi2008_13_6596_hit1
5' CAGGUAACCUUCAGCAAAGCA
   |||||||||||||||||||||
   GUCCAUUGGAAGUCGUUUCGU 5'
AT1G48410.1     1711    1731
Argonaute protein (AGO1)

sRNA_AGO1_Solexa_Mi2008_13_6596_hit1
5' CAGGUAACCUUCAGCAAAGCA
   |||||||||||||||||||||
   GUCCAUUGGAAGUCGUUUCGU 5'
AT1G48410.2     1717    1737
Argonaute protein (AGO1)
```

```
sRNA_AGO1_Solexa_Mi2008_16_26870_hit1
5' UCAACAGAAGCCAGAGAAGUA
   |||||||||||||||||||||
   AGUUGUCUUCGGUCUCUUCAU 5'
AT1G48410.1     1831    1851
Argonaute protein (AGO1)

sRNA_AGO1_Solexa_Mi2008_16_26870_hit1
5' UCAACAGAAGCCAGAGAAGUA
   |||||||||||||||||||||
   AGUUGUCUUCGGUCUCUUCAU 5'
AT1G48410.2     1837    1857
Argonaute protein (AGO1)

sRNA_AGO1_Solexa_Mi2008_3_40523_hit1
5' UGGAGGAGGCAGUAUACGAGC
   |||||||||||||||||||||
   ACCUCCUCCGUCAUAUGCUCG 5'
AT1G48410.1     1853    1873
Argonaute protein (AGO1)

sRNA_AGO1_Solexa_Mi2008_3_40523_hit1
5' UGGAGGAGGCAGUAUACGAGC
   |||||||||||||||||||||
   ACCUCCUCCGUCAUAUGCUCG 5'
AT1G48410.2     1859    1879
Argonaute protein (AGO1)

sRNA_AGO1_Solexa_Mi2008_2_28543_hit1
5' UCAGGGCGAGCACUGACUGGU
   |||||||||||||||||||||
   AGUCCCGCUCGUGACUGACCA 5'
AT1G48410.1     2098    2118
Argonaute protein (AGO1)

sRNA_AGO1_Solexa_Mi2008_2_28543_hit1
5' UCAGGGCGAGCACUGACUGGU
   |||||||||||||||||||||
   AGUCCCGCUCGUGACUGACCA 5'
AT1G48410.2     2104    2124
Argonaute protein (AGO1)

sRNA_AGO1_Solexa_Mi2008_2_36850_hit22
5' UGAGUGUGGUCCUCCUCUUCC
   |||| | ||||||||||||||
   ACUC-CUCCAGGAGGAGAAGG 5'
AT1G48410.2     417     436
Argonaute protein (AGO1)

sRNA_AGO1_Solexa_Mi2008_1_55475_hit2
5' UUUG-CUC-GCGGUGGUCCAGAC
   |||| ||| ||||||||||||||
   AAACAGAGACGCCACCAGGUCUU 5'
AT1G48410.2     485     507
Argonaute protein (AGO1)

sRNA_AGO1_Solexa_Mi2008_46_8918_hit2
5' CGCUUGGUGCAGGUCGGGAAC
    |||||:||||| ||||||||
   UCGAACUACGUCGAGCCCUUG 5'
AT1G48410.2     509     529
Argonaute protein (AGO1)
```

```
sRNA_AGO1_Solexa_Mi2008_1_39168_hit1
5' UGCUAGAAGAAGGUAUAGGCU
   |||||||||||||||||||||
   ACGAUCUUCUUCCAUAUCCGA 5'
AT1G48410.2     669     689
Argonaute protein (AGO1)

sRNA_AGO1_Solexa_Mi2008_5_6229_hit1
5' CAGCAAAGAAAUGGUUAGCC
   ||||||||||||||||||||
   GUCGUUUCUUUACCAAUCGG 5'
AT1G48410.2     751     770
Argonaute protein (AGO1)

sRNA_AGO1_Solexa_Mi2008_1_28240_hit1
5' UCAGCAAAGAAAUGGUUAGCC
   |||||||||||||||||||||
   AGUCGUUUCUUUACCAAUCGG 5'
AT1G48410.2     751     771
Argonaute protein (AGO1)

sRNA_AGO1_Solexa_Mi2008_1_28621_hit15
5' UC-AGGUUGUCUGGUU-GAGUG
   || ||||||||||||| ||| |
   AGAUCCAACAGACCAAACUCUC 5'
AT1G49470.1     293     314
unknown protein

sRNA_AGO1_Solexa_Mi2008_1_18744_hit1
5' UAAUGAGGUGGAGGAACAUGGA
   ||||||||||||||||||||||
   AUUACUCCACCUCCUUGUACCU 5'
AT1G49470.1     321     342
unknown protein

sRNA_AGO1_Solexa_Mi2008_1_53557_hit1
5' UUGUGAGUAAUA--GUGUAUGUGA
   ||||||||||||  |||| |||
   AACACUCAUUAUCGCACACACA 5'
AT1G50420.1     -1      22
scarecrow 3 -like protein

sRNA_AGO1_Solexa_Mi2008_1_12127_hit1
5' CUGGUGGUUAUCUUGUGAGUA
   |||||||||||||||||||||
   GACCACCAAUAGAACACUCAU 5'
AT1G50420.1     13      33
scarecrow 3 -like protein

sRNA_AGO1_Solexa_Mi2008_1_14022_hit2
5' GAGCUCU-UUC-UUGAUUCUA
   | ||||| ||| |||||||||
   CCCGAGAGAAGGAACUAAGAU 5'
AT1G50420.1     1613    1633
scarecrow 3 -like protein

sRNA_AGO1_Solexa_Mi2008_2_654_hit1
5' AACAGCAUGUGAUAUUAUCA
     |||| |||||||||||||
   ACGUCG-ACACUAUAAUAGU 5'
AT1G51940.1     702     720
protein kinase like protein
```

```
sRNA_AGO1_Solexa_Mi2008_2_2818_hit2
5' AUCAUUGCAAUUGUU-GGUCUU
   ||||||||| | ||| ||||||
   UAGUAACGUGA-CAAACCAGAA 5'
AT1G51950.1     1338    1358
IAA18 early auxin-induced protein

sRNA_AGO1_Solexa_Mi2008_3_13373_hit1
5' GAAC-A-AGAAGAAUCUUGCAU
   |||| | ||||||||||||| |
   CUUGAUGUCUUCUUAGAACGCA 5'
AT1G51950.1     317     338
IAA18 early auxin-induced protein

sRNA_AGO1_Solexa_Mi2008_1_34289_hit2
5' UGAAAUCGAUGUUGU-AAGUCC
   :|||||||||||| || ||||||
   GCUUUAGCUACAUCAUUUCAGG 5'
AT1G52400.2     1567    1588
beta-glucosidase, putative

sRNA_AGO1_Solexa_Mi2008_2_23683_hit1
5' UAGUA-UGGU-GUAGUAGUAGUA
   :|||| |||| ||||||||||||
   GUCAUCACCAGCAUCAUCAUCAU 5'
AT1G52690.2     565     587
unknown protein

sRNA_AGO1_Solexa_Mi2008_1_33431_hit1
5' UCUGAUCUGUGGUCGCUUGAA
   |||:|||||||||||||||||
   AGAUUAGACACCAGCGAACUU 5'
AT1G53160.1     558     578
squamosa promoter binding protein-like 4 (spl4)

sRNA_AGO1_Solexa_Mi2008_1_33431_hit1
5' UCUGAUCUGUGGUCGCUUGAA
   |||:|||||||||||||||||
   AGAUUAGACACCAGCGAACUU 5'
AT1G53160.2     560     580
squamosa promoter binding protein-like 4 (spl4)

sRNA_AGO1_Solexa_Mi2008_1_1352_hit1
5' ACAGAAGAGAGAGAGCACU
   |||||||||||||||||
   UGUCUUCUCUCUCUCGUCU 5'
AT1G53160.1     590     608
squamosa promoter binding protein-like 4 (spl4)

sRNA_AGO1_Solexa_Mi2008_70_1353_hit7
5' ACAGAAGAGAGUGAGCACA
   ||||||||||| ||||| |
   UGUCUUCUCUCUCUCGUCU 5'
AT1G53160.1     590     608
squamosa promoter binding protein-like 4 (spl4)

sRNA_AGO1_Solexa_Mi2008_83_1355_hit2
5' ACAGAAGAUAGAGAGCACU
   |||||||| ||||||||
   UGUCUUCUCUCUCUCGUCU 5'
AT1G53160.1     590     608
squamosa promoter binding protein-like 4 (spl4)
```

```
sRNA_AGO1_Solexa_Mi2008_1_13615_hit4
5' GACAGAAGAGAGAGAGCACA
   |||||||||||||||||| |
   CUGUCUUCUCUCUCUCGUCU 5'
AT1G53160.1     590     609
squamosa promoter binding protein-like 4 (spl4)

sRNA_AGO1_Solexa_Mi2008_150_13617_hit7
5' GACAGAAGAGAGUGAGCACA
   |||||||||||| ||||| |
   CUGUCUUCUCUCUCUCGUCU 5'
AT1G53160.1     590     609
squamosa promoter binding protein-like 4 (spl4)

sRNA_AGO1_Solexa_Mi2008_161_13619_hit2
5' GACAGAAGAUAGAGAGCACU
   ||||||||| ||||||||
   CUGUCUUCUCUCUCUCGUCU 5'
AT1G53160.1     590     609
squamosa promoter binding protein-like 4 (spl4)

sRNA_AGO1_Solexa_Mi2008_2_35238_hit4
5' UGACAGAAGAGAGAGAGCACA
   ||||||||||||||||||| |
   ACUGUCUUCUCUCUCUCGUCU 5'
AT1G53160.1     590     610
squamosa promoter binding protein-like 4 (spl4)

sRNA_AGO1_Solexa_Mi2008_714_35241_hit6
5' UGACAGAAGAGAGUGAGCACA
   ||||||||||||| ||||| |
   ACUGUCUUCUCUCUCUCGUCU 5'
AT1G53160.1     590     610
squamosa promoter binding protein-like 4 (spl4)

sRNA_AGO1_Solexa_Mi2008_4_35245_hit2
5' UGACAGAAGAUAGAGAGCACA
   |||||||||| |||||||| |
   ACUGUCUUCUCUCUCUCGUCU 5'
AT1G53160.1     590     610
squamosa promoter binding protein-like 4 (spl4)

sRNA_AGO1_Solexa_Mi2008_66_35246_hit2
5' UGACAGAAGAUAGAGAGCACU
   |||||||||| ||||||||
   ACUGUCUUCUCUCUCUCGUCU 5'
AT1G53160.1     590     610
squamosa promoter binding protein-like 4 (spl4)

sRNA_AGO1_Solexa_Mi2008_6_13612_hit1
5' GACAGAAGAAAGAGAGCAC
   ||||||||| ||||||||
   CUGUCUUCUCUCUCUCGUC 5'
AT1G53160.1     591     609
squamosa promoter binding protein-like 4 (spl4)

sRNA_AGO1_Solexa_Mi2008_1_13614_hit8
5' GACAGAAGAGAGAGAGCAC
   ||||||||||||||||||
   CUGUCUUCUCUCUCUCGUC 5'
AT1G53160.1     591     609
squamosa promoter binding protein-like 4 (spl4)
```

```
sRNA_AGO1_Solexa_Mi2008_480_13616_hit7
5' GACAGAAGAGAGUGAGCAC
   |||||||||||| |||||
   CUGUCUUCUCUCUCUCGUC 5'
AT1G53160.1     591     609
squamosa promoter binding protein-like 4 (spl4)

sRNA_AGO1_Solexa_Mi2008_855_13618_hit4
5' GACAGAAGAUAGAGAGCAC
   ||||||||| ||||||||
   CUGUCUUCUCUCUCUCGUC 5'
AT1G53160.1     591     609
squamosa promoter binding protein-like 4 (spl4)

sRNA_AGO1_Solexa_Mi2008_3_8396_hit1
5' CGACAGAAGAGAGUGAGCAC
    |||||||||||| |||||
   ACUGUCUUCUCUCUCUCGUC 5'
AT1G53160.1     591     610
squamosa promoter binding protein-like 4 (spl4)

sRNA_AGO1_Solexa_Mi2008_6_35236_hit1
5' UGACAGAAGAAAGAGAGCAC
   |||||||||| ||||||||
   ACUGUCUUCUCUCUCUCGUC 5'
AT1G53160.1     591     610
squamosa promoter binding protein-like 4 (spl4)

sRNA_AGO1_Solexa_Mi2008_12_35237_hit8
5' UGACAGAAGAGAGAGAGCAC
   |||||||||||||||||||
   ACUGUCUUCUCUCUCUCGUC 5'
AT1G53160.1     591     610
squamosa promoter binding protein-like 4 (spl4)

sRNA_AGO1_Solexa_Mi2008_2091_35240_hit6
5' UGACAGAAGAGAGUGAGCAC
   ||||||||||||| |||||
   ACUGUCUUCUCUCUCUCGUC 5'
AT1G53160.1     591     610
squamosa promoter binding protein-like 4 (spl4)

sRNA_AGO1_Solexa_Mi2008_193_35244_hit4
5' UGACAGAAGAUAGAGAGCAC
   |||||||||| ||||||||
   ACUGUCUUCUCUCUCUCGUC 5'
AT1G53160.1     591     610
squamosa promoter binding protein-like 4 (spl4)

sRNA_AGO1_Solexa_Mi2008_28_11427_hit3
5' CUGACAGAAGAGAGUGAGCAC
    ||||||||||||| |||||
   UACUGUCUUCUCUCUCUCGUC 5'
AT1G53160.1     591     611
squamosa promoter binding protein-like 4 (spl4)

sRNA_AGO1_Solexa_Mi2008_79_11430_hit1
5' CUGACAGAAGAUAGAGAGCAC
    |||||||||| ||||||||
   UACUGUCUUCUCUCUCUCGUC 5'
AT1G53160.1     591     611
squamosa promoter binding protein-like 4 (spl4)
```

```
sRNA_AGO1_Solexa_Mi2008_4_15853_hit2
5' GUGACAGAAGAGAGUGAGCAC
   :||||||||||||| |||||
   UACUGUCUUCUCUCUCUCGUC 5'
AT1G53160.1     591     611
squamosa promoter binding protein-like 4 (spl4)

sRNA_AGO1_Solexa_Mi2008_12_50256_hit1
5' UUGACAGAAGAAAGAGAGCAC
    |||||||||| ||||||||
   UACUGUCUUCUCUCUCUCGUC 5'
AT1G53160.1     591     611
squamosa promoter binding protein-like 4 (spl4)

sRNA_AGO1_Solexa_Mi2008_14_50257_hit5
5' UUGACAGAAGAGAGAGAGCAC
    |||||||||||||||||||
   UACUGUCUUCUCUCUCUCGUC 5'
AT1G53160.1     591     611
squamosa promoter binding protein-like 4 (spl4)

sRNA_AGO1_Solexa_Mi2008_1778_50260_hit1
5' UUGACAGAAGAGAGUGAGCAC
    ||||||||||||| |||||
   UACUGUCUUCUCUCUCUCGUC 5'
AT1G53160.1     591     611
squamosa promoter binding protein-like 4 (spl4)

sRNA_AGO1_Solexa_Mi2008_4050_50264_hit3
5' UUGACAGAAGAUAGAGAGCAC
    |||||||||| ||||||||
   UACUGUCUUCUCUCUCUCGUC 5'
AT1G53160.1     591     611
squamosa promoter binding protein-like 4 (spl4)

sRNA_AGO1_Solexa_Mi2008_30_35239_hit6
5' UGACAGAAGAGAGUGAGCA
   ||||||||||||| |||||
   ACUGUCUUCUCUCUCUCGU 5'
AT1G53160.1     592     610
squamosa promoter binding protein-like 4 (spl4)

sRNA_AGO1_Solexa_Mi2008_1_11426_hit3
5' CUGACAGAAGAGAGUGAGCA
    ||||||||||||| |||||
   UACUGUCUUCUCUCUCUCGU 5'
AT1G53160.1     592     611
squamosa promoter binding protein-like 4 (spl4)

sRNA_AGO1_Solexa_Mi2008_2_11429_hit1
5' CUGACAGAAGAUAGAGAGCA
    |||||||||| ||||||||
   UACUGUCUUCUCUCUCUCGU 5'
AT1G53160.1     592     611
squamosa promoter binding protein-like 4 (spl4)

sRNA_AGO1_Solexa_Mi2008_16_50259_hit1
5' UUGACAGAAGAGAGUGAGCA
    ||||||||||||| |||||
   UACUGUCUUCUCUCUCUCGU 5'
AT1G53160.1     592     611
squamosa promoter binding protein-like 4 (spl4)
```

```
sRNA_AGO1_Solexa_Mi2008_122_50263_hit3
5' UUGACAGAAGAUAGAGAGCA
    |||||||||| ||||||||
   UACUGUCUUCUCUCUCUCGU 5'
AT1G53160.1     592     611
squamosa promoter binding protein-like 4 (spl4)

sRNA_AGO1_Solexa_Mi2008_1_50258_hit1
5' UUGACAGAAGAGAGUGAGC
    ||||||||||||| ||||
   UACUGUCUUCUCUCUCUCG 5'
AT1G53160.1     593     611
squamosa promoter binding protein-like 4 (spl4)

sRNA_AGO1_Solexa_Mi2008_21_50262_hit3
5' UUGACAGAAGAUAGAGAGC
    |||||||||| |||||||
   UACUGUCUUCUCUCUCUCG 5'
AT1G53160.1     593     611
squamosa promoter binding protein-like 4 (spl4)

sRNA_AGO1_Solexa_Mi2008_1_37308_hit1
5' UGAUGGAGAAGAUCAAACCUAA
   |||||| || ||||||||| ||
   ACUACCACUCCUAGUUUGG-UU 5'
AT1G53230.1     1226    1246
flower development cycloidea like protein

sRNA_AGO1_Solexa_Mi2008_6_14255_hit369
5' GAUGAUGAUGAUGAUGAUGAUGAU
   ||||||||||||||| ||| |||
   CUACUACUACUACUACCACUCCUA 5'
AT1G53230.1     1233    1256
flower development cycloidea like protein

sRNA_AGO1_Solexa_Mi2008_6_14255_hit369
5' GAUGAUGAUGAUGAUGAUGAUGAU
   |||||||||||||||||| |||
   CUACUACUACUACUACUACCACUC 5'
AT1G53230.1     1236    1259
flower development cycloidea like protein

sRNA_AGO1_Solexa_Mi2008_1_36323_hit13
5' UGAGGAUGAUGAUGAUGAUGA
   ||| ||||||||||||| |||
   ACUACUACUACUACUACCACU 5'
AT1G53230.1     1237    1257
flower development cycloidea like protein

sRNA_AGO1_Solexa_Mi2008_1_3079_hit472
5' AUGAUGAUGAUGAUGAUGAUGA
   ||||||||||||||||| |||
   UACUACUACUACUACUACCACU 5'
AT1G53230.1     1237    1258
flower development cycloidea like protein

sRNA_AGO1_Solexa_Mi2008_1_56092_hit3
5' UUUG-UGAUGAUGAUGAUGAUGA
   :||| ||||||||||||| |||
   GAACUACUACUACUACUACCACU 5'
AT1G53230.1     1238    1260
flower development cycloidea like protein
```

```
sRNA_AGO1_Solexa_Mi2008_1_45039_hit3
5' UG-UGAUGAUGAUGAUGAUGAUGA
   || ||||||||||||||||| |||
   ACUACUACUACUACUACUACCACU 5'
AT1G53230.1     1238    1261
flower development cycloidea like protein

sRNA_AGO1_Solexa_Mi2008_9_14254_hit8
5' GAUGAUGAUGAUGAUGAUCUU
   ||||||||||||||||||  |
   CUACUACUACUACUACUACCA 5'
AT1G53230.1     1239    1259
flower development cycloidea like protein

sRNA_AGO1_Solexa_Mi2008_7_3078_hit1
5' AUGAUGAUGA-GAAUGAUGAU
   |||||||||| | |||||| |
   UACUACUACUAC-UACUACCA 5'
AT1G53230.1     1240    1259
flower development cycloidea like protein

sRNA_AGO1_Solexa_Mi2008_1_36323_hit13
5' UGAGGAUGAUGAUGAUGAUGA
   ||| ||||||||||||||||
   ACUACUACUACUACUACUACC 5'
AT1G53230.1     1240    1260
flower development cycloidea like protein

sRNA_AGO1_Solexa_Mi2008_1_3079_hit472
5' AUGAUGAUGAUGAUGAUGAUGA
    ||||||||||||||||||||
   GACUACUACUACUACUACUACC 5'
AT1G53230.1     1240    1261
flower development cycloidea like protein

sRNA_AGO1_Solexa_Mi2008_1_45039_hit3
5' UG-UGAUGAUGAUGAUGAUGAUGA
   || ||||||||||||||||||||
   ACGACUACUACUACUACUACUACC 5'
AT1G53230.1     1241    1264
flower development cycloidea like protein

sRNA_AGO1_Solexa_Mi2008_1_38921_hit1
5' UGCGGACGAUGAUGAUGAUGAU
   ||| || |||||||||||||||
   ACGACUACUACUACUACUACUA 5'
AT1G53230.1     1242    1263
flower development cycloidea like protein

sRNA_AGO1_Solexa_Mi2008_7_3078_hit1
5' AUGAUGAUGA-GAAUGAUGAU
    ||||||||| || |||||||
   CACUACUACUACU-ACUACUA 5'
AT1G53230.1     1243    1262
flower development cycloidea like protein

sRNA_AGO1_Solexa_Mi2008_3_39351_hit1
5' UGCUGAUGAUGCUGAUG-UGAC
   ||||||||||| ||||| |||
   ACGACUACUACUACUACUACUA 5'
AT1G53230.1     1243    1264
flower development cycloidea like protein
```

```
sRNA_AGO1_Solexa_Mi2008_6_14255_hit369
5' GA-UGAUGAUGAUGAUGAUGAUGAU
   || || |||||||||||||||||||
   CUGACGACUACUACUACUACUACUA 5'
AT1G53230.1     1243    1267
flower development cycloidea like protein

sRNA_AGO1_Solexa_Mi2008_1_56092_hit3
5' UUUG-UGAUGAUGAUGAUGAUGA
   | || ||||||||||||||||||
   ACACGACUACUACUACUACUACU 5'
AT1G53230.1     1244    1266
flower development cycloidea like protein

sRNA_AGO1_Solexa_Mi2008_2_31619_hit1
5' UCGCUGAUGAUGAUUGAUGAU
     |||||||||||| ||||||
   CUCGACUACUACUA-CUACUA 5'
AT1G53230.1     1246    1265
flower development cycloidea like protein

sRNA_AGO1_Solexa_Mi2008_1_39699_hit1
5' UGGAACUGAUGAUGAUGAUGA
   |||| ||| ||||||||||||
   ACCU-GACGACUACUACUACU 5'
AT1G53230.1     1250    1269
flower development cycloidea like protein

sRNA_AGO1_Solexa_Mi2008_1_28327_hit1
5' UCAGCCGCAACCACCACCUGUG
   || ||||||||||||||| ||
   AG-CGGCGUUGGUGGUGG-CAA 5'
AT1G53230.1     372     391
flower development cycloidea like protein

sRNA_AGO1_Solexa_Mi2008_1_13897_hit2
5' GAGAAAUCAAAGUUUUUGGGUU
   ||||||| ||||||||  ||||
   CUCUUUAAUUUCAAAA--CCAA 5'
AT1G53230.1     689     708
flower development cycloidea like protein

sRNA_AGO1_Solexa_Mi2008_1_19666_hit1
5' UACCAACAGCAGAAUC-CACCAU
   |||||||||||:|||| || |||
   AUGGUUGUCGUUUUAGUGU-GUA 5'
AT1G54100.2     487     508
unknown protein

sRNA_AGO1_Solexa_Mi2008_1_19666_hit1
5' UACCAACAGCAGAAUC-CACCAU
   |||||||||||:|||| || |||
   AUGGUUGUCGUUUUAGUGU-GUA 5'
AT1G54100.1     538     559
unknown protein

sRNA_AGO1_Solexa_Mi2008_12_46414_hit1
5' UUAACGCCGUGAUUGUU-UGGU
   |||||| |||||||||| ||:|
   AAUUGCUGCACUAACAACACUA 5'
AT1G55020.1     340     361
unknown protein
```

```
sRNA_AGO1_Solexa_Mi2008_1_28726_hit1
5' UCAGUGUGGAAGCUAAGUCUC
   ||| |||||||| |||||||
   AGU-ACACCUUCUAUUCAGAA 5'
AT1G55020.1     577     596
unknown protein

sRNA_AGO1_Solexa_Mi2008_1_46444_hit1
5' UUAACUCUGUGAUUGUUU-GGU
   |||||||||||||| ||| ||:
   AAUUGAGACACUAAGAAAUCCG 5'
AT1G56010.1     1000    1021
NAC1

sRNA_AGO1_Solexa_Mi2008_1_46444_hit1
5' UUAACUCUGUGAUUGUUU-GGU
   |||||||||||||| ||| ||:
   AAUUGAGACACUAAGAAAUCCG 5'
AT1G56010.2     1025    1046
NAC1

sRNA_AGO1_Solexa_Mi2008_1_13477_hit1
5' GAAGCAGGGCACGUG--CAUU
   ||||||||| |||||  ||||
   CUUCGUCCCAUGCACGAGUAA 5'
AT1G56010.1     773     793
NAC1

sRNA_AGO1_Solexa_Mi2008_2_14844_hit1
5' GGAGAAGCAGGGCACGUG--CAUU
   |||||||||||| |||||  ||||
   CCUCUUCGUCCCAUGCACGAGUAA 5'
AT1G56010.1     773     796
NAC1

sRNA_AGO1_Solexa_Mi2008_91_40198_hit1
5' UGGAGAAGCAGGGCACGUG--CAUU
   ||||||||||||| |||||  ||||
   ACCUCUUCGUCCCAUGCACGAGUAA 5'
AT1G56010.1     773     797
NAC1

sRNA_AGO1_Solexa_Mi2008_2_13910_hit1
5' GAGAAGCAGGGCACGUGCAU
   ||||||||||| |||||| :
   CUCUUCGUCCCAUGCACGAG 5'
AT1G56010.1     775     794
NAC1

sRNA_AGO1_Solexa_Mi2008_1_14842_hit1
5' GGAGAAGCAGGGCACGUGCAA
   |||||||||||| ||||||
   CCUCUUCGUCCCAUGCACGAG 5'
AT1G56010.1     775     795
NAC1

sRNA_AGO1_Solexa_Mi2008_4_14843_hit1
5' GGAGAAGCAGGGCACGUGCAU
   |||||||||||| |||||| :
   CCUCUUCGUCCCAUGCACGAG 5'
AT1G56010.1     775     795
NAC1
```

```
sRNA_AGO1_Solexa_Mi2008_1_14846_hit1
5' GGAGAAGCAGGGCACGUGCGA
   |||||||||||| ||||||
   CCUCUUCGUCCCAUGCACGAG 5'
AT1G56010.1     775     795
NAC1

sRNA_AGO1_Solexa_Mi2008_3_40196_hit1
5' UGGAGAAGCAGGGCACGUGCAA
   ||||||||||||| ||||||
   ACCUCUUCGUCCCAUGCACGAG 5'
AT1G56010.1     775     796
NAC1

sRNA_AGO1_Solexa_Mi2008_284_40197_hit1
5' UGGAGAAGCAGGGCACGUGCAU
   ||||||||||||| |||||| :
   ACCUCUUCGUCCCAUGCACGAG 5'
AT1G56010.1     775     796
NAC1

sRNA_AGO1_Solexa_Mi2008_10440_13909_hit2
5' GAGAAGCAGGGCACGUGCA
   ||||||||||| ||||||
   CUCUUCGUCCCAUGCACGA 5'
AT1G56010.1     776     794
NAC1

sRNA_AGO1_Solexa_Mi2008_110_13911_hit1
5' GAGAAGCAGGGCACGUGCG
   ||||||||||| ||||||
   CUCUUCGUCCCAUGCACGA 5'
AT1G56010.1     776     794
NAC1

sRNA_AGO1_Solexa_Mi2008_41322_14841_hit2
5' GGAGAAGCAGGGCACGUGCA
   |||||||||||| ||||||
   CCUCUUCGUCCCAUGCACGA 5'
AT1G56010.1     776     795
NAC1

sRNA_AGO1_Solexa_Mi2008_422_14845_hit1
5' GGAGAAGCAGGGCACGUGCG
   |||||||||||| ||||||
   CCUCUUCGUCCCAUGCACGA 5'
AT1G56010.1     776     795
NAC1

sRNA_AGO1_Solexa_Mi2008_370931_40195_hit2
5' UGGAGAAGCAGGGCACGUGCA
   ||||||||||||| ||||||
   ACCUCUUCGUCCCAUGCACGA 5'
AT1G56010.1     776     796
NAC1

sRNA_AGO1_Solexa_Mi2008_7589_40199_hit1
5' UGGAGAAGCAGGGCACGUGCG
   ||||||||||||| ||||||
   ACCUCUUCGUCCCAUGCACGA 5'
AT1G56010.1     776     796
NAC1
```

```
sRNA_AGO1_Solexa_Mi2008_2_40202_hit1
5' UGGAGAAGCAGGGUACGUGCU
   |||||||||||||||||||||
   ACCUCUUCGUCCCAUGCACGA 5'
AT1G56010.1     776     796
NAC1

sRNA_AGO1_Solexa_Mi2008_1214_3232_hit1
5' AUGGAGAAGCAGGGCACGUGCA
    ||||||||||||| ||||||
   AACCUCUUCGUCCCAUGCACGA 5'
AT1G56010.1     776     797
NAC1

sRNA_AGO1_Solexa_Mi2008_24_3233_hit1
5' AUGGAGAAGCAGGGCACGUGCG
    ||||||||||||| ||||||
   AACCUCUUCGUCCCAUGCACGA 5'
AT1G56010.1     776     797
NAC1

sRNA_AGO1_Solexa_Mi2008_9_52081_hit1
5' UUGGAGAAGCAGGGCACGUGCA
   |||||||||||||| ||||||
   AACCUCUUCGUCCCAUGCACGA 5'
AT1G56010.1     776     797
NAC1

sRNA_AGO1_Solexa_Mi2008_23_14840_hit3
5' GGAGAAGCAGGGCACGUGC
   ||||||||||| ||||||
   CCUCUUCGUCCCAUGCACG 5'
AT1G56010.1     777     795
NAC1

sRNA_AGO1_Solexa_Mi2008_1_40190_hit1
5' UGGAGAAGCAGGGCACGUAA
   ||||||||||||| ||||
   ACCUCUUCGUCCCAUGCACG 5'
AT1G56010.1     777     796
NAC1

sRNA_AGO1_Solexa_Mi2008_404_40194_hit3
5' UGGAGAAGCAGGGCACGUGC
   ||||||||||||| ||||||
   ACCUCUUCGUCCCAUGCACG 5'
AT1G56010.1     777     796
NAC1

sRNA_AGO1_Solexa_Mi2008_2_40201_hit1
5' UGGAGAAGCAGGGUACGUGC
   ||||||||||||||||||||
   ACCUCUUCGUCCCAUGCACG 5'
AT1G56010.1     777     796
NAC1

sRNA_AGO1_Solexa_Mi2008_9_40189_hit2
5' UGGAGAAGCAGGGCACGUA
   ||||||||||||| ||||
   ACCUCUUCGUCCCAUGCAC 5'
AT1G56010.1     778     796
NAC1
```

```
sRNA_AGO1_Solexa_Mi2008_366_40193_hit3
5' UGGAGAAGCAGGGCACGUG
   ||||||||||||| |||||
   ACCUCUUCGUCCCAUGCAC 5'
AT1G56010.1     778     796
NAC1

sRNA_AGO1_Solexa_Mi2008_1_40200_hit1
5' UGGAGAAGCAGGGUACGUG
   |||||||||||||||||||
   ACCUCUUCGUCCCAUGCAC 5'
AT1G56010.1     778     796
NAC1

sRNA_AGO1_Solexa_Mi2008_2_3231_hit2
5' AUGGAGAAGCAGGGCACGUG
    ||||||||||||| |||||
   AACCUCUUCGUCCCAUGCAC 5'
AT1G56010.1     778     797
NAC1

sRNA_AGO1_Solexa_Mi2008_1_13477_hit1
5' GAAGCAGGGCACGUG--CAUU
   ||||||||| |||||  ||||
   CUUCGUCCCAUGCACGAGUAA 5'
AT1G56010.2     798     818
NAC1

sRNA_AGO1_Solexa_Mi2008_2_14844_hit1
5' GGAGAAGCAGGGCACGUG--CAUU
   |||||||||||| |||||  ||||
   CCUCUUCGUCCCAUGCACGAGUAA 5'
AT1G56010.2     798     821
NAC1

sRNA_AGO1_Solexa_Mi2008_91_40198_hit1
5' UGGAGAAGCAGGGCACGUG--CAUU
   ||||||||||||| |||||  ||||
   ACCUCUUCGUCCCAUGCACGAGUAA 5'
AT1G56010.2     798     822
NAC1

sRNA_AGO1_Solexa_Mi2008_2_13910_hit1
5' GAGAAGCAGGGCACGUGCAU
   ||||||||||| |||||| :
   CUCUUCGUCCCAUGCACGAG 5'
AT1G56010.2     800     819
NAC1

sRNA_AGO1_Solexa_Mi2008_1_14842_hit1
5' GGAGAAGCAGGGCACGUGCAA
   |||||||||||| ||||||
   CCUCUUCGUCCCAUGCACGAG 5'
AT1G56010.2     800     820
NAC1

sRNA_AGO1_Solexa_Mi2008_4_14843_hit1
5' GGAGAAGCAGGGCACGUGCAU
   |||||||||||| |||||| :
   CCUCUUCGUCCCAUGCACGAG 5'
AT1G56010.2     800     820
NAC1
```

```
sRNA_AGO1_Solexa_Mi2008_1_14846_hit1
5' GGAGAAGCAGGGCACGUGCGA
   |||||||||||| ||||||
   CCUCUUCGUCCCAUGCACGAG 5'
AT1G56010.2     800     820
NAC1

sRNA_AGO1_Solexa_Mi2008_3_40196_hit1
5' UGGAGAAGCAGGGCACGUGCAA
   ||||||||||||| ||||||
   ACCUCUUCGUCCCAUGCACGAG 5'
AT1G56010.2     800     821
NAC1

sRNA_AGO1_Solexa_Mi2008_284_40197_hit1
5' UGGAGAAGCAGGGCACGUGCAU
   ||||||||||||| |||||| :
   ACCUCUUCGUCCCAUGCACGAG 5'
AT1G56010.2     800     821
NAC1

sRNA_AGO1_Solexa_Mi2008_10440_13909_hit2
5' GAGAAGCAGGGCACGUGCA
   ||||||||||| ||||||
   CUCUUCGUCCCAUGCACGA 5'
AT1G56010.2     801     819
NAC1

sRNA_AGO1_Solexa_Mi2008_110_13911_hit1
5' GAGAAGCAGGGCACGUGCG
   ||||||||||| ||||||
   CUCUUCGUCCCAUGCACGA 5'
AT1G56010.2     801     819
NAC1

sRNA_AGO1_Solexa_Mi2008_41322_14841_hit2
5' GGAGAAGCAGGGCACGUGCA
   |||||||||||| ||||||
   CCUCUUCGUCCCAUGCACGA 5'
AT1G56010.2     801     820
NAC1

sRNA_AGO1_Solexa_Mi2008_422_14845_hit1
5' GGAGAAGCAGGGCACGUGCG
   |||||||||||| ||||||
   CCUCUUCGUCCCAUGCACGA 5'
AT1G56010.2     801     820
NAC1

sRNA_AGO1_Solexa_Mi2008_370931_40195_hit2
5' UGGAGAAGCAGGGCACGUGCA
   ||||||||||||| ||||||
   ACCUCUUCGUCCCAUGCACGA 5'
AT1G56010.2     801     821
NAC1

sRNA_AGO1_Solexa_Mi2008_7589_40199_hit1
5' UGGAGAAGCAGGGCACGUGCG
   ||||||||||||| ||||||
   ACCUCUUCGUCCCAUGCACGA 5'
AT1G56010.2     801     821
NAC1
```

```
sRNA_AGO1_Solexa_Mi2008_2_40202_hit1
5' UGGAGAAGCAGGGUACGUGCU
   |||||||||||||||||||||
   ACCUCUUCGUCCCAUGCACGA 5'
AT1G56010.2     801     821
NAC1

sRNA_AGO1_Solexa_Mi2008_1214_3232_hit1
5' AUGGAGAAGCAGGGCACGUGCA
    ||||||||||||| ||||||
   AACCUCUUCGUCCCAUGCACGA 5'
AT1G56010.2     801     822
NAC1

sRNA_AGO1_Solexa_Mi2008_24_3233_hit1
5' AUGGAGAAGCAGGGCACGUGCG
    ||||||||||||| ||||||
   AACCUCUUCGUCCCAUGCACGA 5'
AT1G56010.2     801     822
NAC1

sRNA_AGO1_Solexa_Mi2008_9_52081_hit1
5' UUGGAGAAGCAGGGCACGUGCA
   |||||||||||||| ||||||
   AACCUCUUCGUCCCAUGCACGA 5'
AT1G56010.2     801     822
NAC1

sRNA_AGO1_Solexa_Mi2008_23_14840_hit3
5' GGAGAAGCAGGGCACGUGC
   |||||||||||| ||||||
   CCUCUUCGUCCCAUGCACG 5'
AT1G56010.2     802     820
NAC1

sRNA_AGO1_Solexa_Mi2008_1_40190_hit1
5' UGGAGAAGCAGGGCACGUAA
   ||||||||||||| ||||
   ACCUCUUCGUCCCAUGCACG 5'
AT1G56010.2     802     821
NAC1

sRNA_AGO1_Solexa_Mi2008_404_40194_hit3
5' UGGAGAAGCAGGGCACGUGC
   ||||||||||||| ||||||
   ACCUCUUCGUCCCAUGCACG 5'
AT1G56010.2     802     821
NAC1

sRNA_AGO1_Solexa_Mi2008_2_40201_hit1
5' UGGAGAAGCAGGGUACGUGC
   ||||||||||||||||||||
   ACCUCUUCGUCCCAUGCACG 5'
AT1G56010.2     802     821
NAC1

sRNA_AGO1_Solexa_Mi2008_9_40189_hit2
5' UGGAGAAGCAGGGCACGUA
   ||||||||||||| ||||
   ACCUCUUCGUCCCAUGCAC 5'
AT1G56010.2     803     821
NAC1
```

```
sRNA_AGO1_Solexa_Mi2008_366_40193_hit3
5' UGGAGAAGCAGGGCACGUG
   ||||||||||||| |||||
   ACCUCUUCGUCCCAUGCAC 5'
AT1G56010.2     803     821
NAC1

sRNA_AGO1_Solexa_Mi2008_1_40200_hit1
5' UGGAGAAGCAGGGUACGUG
   |||||||||||||||||||
   ACCUCUUCGUCCCAUGCAC 5'
AT1G56010.2     803     821
NAC1

sRNA_AGO1_Solexa_Mi2008_2_3231_hit2
5' AUGGAGAAGCAGGGCACGUG
    ||||||||||||| |||||
   AACCUCUUCGUCCCAUGCAC 5'
AT1G56010.2     803     822
NAC1

sRNA_AGO1_Solexa_Mi2008_1_21205_hit1
5' UAGAGACGAUUGAUAAGAGUA
    ||||||||||||| ||||||
   UUCUCUGCUAACUACUCUCAU 5'
AT1G58270.1     1171    1191
unknown protein

sRNA_AGO1_Solexa_Mi2008_2_5090_hit1
5' CAAUAGGUGAAAUCAUAGAUU
   ||||| ||||||||  |||||
   GUUAUACACUUUAG--UCUAA 5'
AT1G58270.1     1335    1353
unknown protein

sRNA_AGO1_Solexa_Mi2008_1_2_hit38
5' AAAAAAAAAAAAAAACCAU
    |||||||||||||| |||
   GUUUUUUUUUUUUUUUGUA 5'
AT1G58270.1     1392    1410
unknown protein

sRNA_AGO1_Solexa_Mi2008_7_14756_hit2
5' GCUACCUU-AAGAGAGUCA-UAG
   | |||||| |||||||||| |||
   CCAUGGAAAUUCUCUCAGUUAUC 5'
AT1G58340.1     972     994
putative protein

sRNA_AGO1_Solexa_Mi2008_9_2828_hit2
5' AUCCAGCGCACGGUAGCUU
   |||| ||||||||||||
   UAGGCCGCGUGCCAUCGUU 5'
AT1G58360.1     368     386
unknown protein

sRNA_AGO1_Solexa_Mi2008_1_172_hit2
5' AA-AAGAAGAAGAUAAAGCA
   || ||||||||||||||: |
   UUGUUCUUCUUCUAUUUUAU 5'
AT1G58360.1     49      68
unknown protein
```

```
sRNA_AGO1_Solexa_Mi2008_2_6628_hit1
5' CAGGUGAUGGUGUAAUCAA
   ||| |||||||||||:|||
   GUC-ACUACCACAUUGGUU 5'
AT1G58360.1     503     520
unknown protein

sRNA_AGO1_Solexa_Mi2008_1_14_hit1
5' AAA-A-AAACAGAGAACAAGAAGA
   ||| | ||| |||||||||||||
   UUUCUCUUUCUCUCUUGUUCUUCU 5'
AT1G58360.1     59      82
unknown protein

sRNA_AGO1_Solexa_Mi2008_3_35894_hit1
5' UGAGAGCA-GAGAAAGAGAGU
   |||||| | |||||||||||
   ACUCUCUUUCUCUUUCUCUCU 5'
AT1G58360.1     68      88
unknown protein

sRNA_AGO1_Solexa_Mi2008_1_35311_hit3
5' UGAC-AUGAUGUUGAUGUGCAUA
   |||| |||||||||||||| ||
   ACUGGUACUACAACUACAC-UAC 5'
AT1G62360.1     104     125
homeobox transcription factor shootmeristemless (stm)

sRNA_AGO1_Solexa_Mi2008_1_35310_hit3
5' UGAC-AUGAUGUUGAUGUGCA
   |||| ||||||||||||||
   ACUGGUACUACAACUACACUA 5'
AT1G62360.1     105     125
homeobox transcription factor shootmeristemless (stm)

sRNA_AGO1_Solexa_Mi2008_1_2990_hit3
5' AUGAC-AUGAUGUUGAUGUGCAU
   ||||| |||||||||||||| ||
   UACUGGUACUACAACUACAC-UA 5'
AT1G62360.1     105     126
homeobox transcription factor shootmeristemless (stm)

sRNA_AGO1_Solexa_Mi2008_2_7102_hit1
5' CAUGAACAUGAUGAUGAUG-GA
   ||||| ||||||| ||||| ||
   GUACUGGUACUACAACUACACU 5'
AT1G62360.1     106     127
homeobox transcription factor shootmeristemless (stm)

sRNA_AGO1_Solexa_Mi2008_2_13419_hit2
5' GAAGAAGAAGAAG-ACACUU
   ||||||||||||| | ||||
   CUUCUUCUUCUUCGU-UGAA 5'
AT1G62360.1     239     257
homeobox transcription factor shootmeristemless (stm)

sRNA_AGO1_Solexa_Mi2008_4_26725_hit129
5' UCAAAGCUUUCA-UGGUGUAGC
   |||||||||||| |:|| ||||
   AGUUUCGAAAGUUAUCAAAUCG 5'
AT1G62540.1     1547    1568
unknown protein
```

```
sRNA_AGO1_Solexa_Mi2008_1_3891_hit1
5' CAAAACCAGAAGGAUGAUAAA
   |||||| |||||||||| |||
   GUUUUGCUCUUCCUACU-UUU 5'
AT1G62570.1     1421    1440
similar to glutamate synthase

sRNA_AGO1_Solexa_Mi2008_3_21212_hit1
5' UAGAGAGAGAAAAAGAUGGC
   |||||||||||| ||| |||
   AUCUCUCUCUUUCUCU-CCG 5'
AT1G62710.1     26      44
beta-VPE

sRNA_AGO1_Solexa_Mi2008_30_14853_hit1
5' GGAGAGAGCAAAGUUGAGA
   | ||||||||||||| |||
   CGUCUCUCGUUUCAAAUCU 5'
AT1G63010.1     190     208
tetracycline resistance efflux protein like protein

sRNA_AGO1_Solexa_Mi2008_30_14853_hit1
5' GGAGAGAGCAAAGUUGAGA
   | ||||||||||||| |||
   CGUCUCUCGUUUCAAAUCU 5'
AT1G63010.4     310     328
tetracycline resistance efflux protein like protein

sRNA_AGO1_Solexa_Mi2008_30_14853_hit1
5' GGAGAGAGCAAAGUUGAGA
   | ||||||||||||| |||
   CGUCUCUCGUUUCAAAUCU 5'
AT1G63010.2     409     427
tetracycline resistance efflux protein like protein

sRNA_AGO1_Solexa_Mi2008_30_14853_hit1
5' GGAGAGAGCAAAGUUGAGA
   | ||||||||||||| |||
   CGUCUCUCGUUUCAAAUCU 5'
AT1G63010.3     410     428
tetracycline resistance efflux protein like protein

sRNA_AGO1_Solexa_Mi2008_1_43726_hit3
5' UGGUUAAGGAGAUAGACUUGA
   |||||||| |||||||  |||
   ACCAAUUCAUCUAUCU--ACU 5'
AT1G63720.1     683     701
unknown protein

sRNA_AGO1_Solexa_Mi2008_1_50002_hit1
5' UUGAACAUUGUGGUG-UGAGUG
   ||||||||||||| | |||||
   AACUUGUAACACCUCUACUCAA 5'
AT1G63840.1     399     420
putative RING zinc finger protein

sRNA_AGO1_Solexa_Mi2008_1_3384_hit1
5' AUGGUGAAGAUGUUUCUAGU
    |||||| |||||||||||
   CACCACUACUACAAAGAUCU 5'
AT1G63840.1     73      92
putative RING zinc finger protein
```

```
sRNA_AGO1_Solexa_Mi2008_1_27754_hit1
5' UCACCUCAUGAUGAACUUGGA
    |||| | ||||||||||||
   CGUGGUG-ACUACUUGAACCU 5'
AT1G64660.1     798     817
similar to O-succinylhomoserine sulfhydrylase

sRNA_AGO1_Solexa_Mi2008_3_13421_hit1
5' GAAGAAGAAGAUGAUGUUGAU
   :||||||||||||||| |||
   UUUCUUCUUCUACUACUACUU 5'
AT1G66090.1     101     121
unknown protein

sRNA_AGO1_Solexa_Mi2008_6_29484_hit1
5' UCCAGGACCAAACCACGUUGU
   |||||||||| ||||| |||
   AGGUCCUGGUAUGGUG-AACU 5'
AT1G66090.1     1049    1068
unknown protein

sRNA_AGO1_Solexa_Mi2008_1_48999_hit1
5' UUCCAGGACCAAACCACGUUG
   ||||||||||| ||||| |||
   AAGGUCCUGGUAUGGUG-AAC 5'
AT1G66090.1     1050    1069
unknown protein

sRNA_AGO1_Solexa_Mi2008_1_1763_hit2
5' ACUUCCAGGACCAAACCACGU
   ||||||||||||| ||||| |
   UGAAGGUCCUGGUAUGGUGAA 5'
AT1G66090.1     1050    1070
unknown protein

sRNA_AGO1_Solexa_Mi2008_26_20692_hit2
5' UACUUCCAGGACCAAACCACGU
   |||||||||||||| ||||| |
   AUGAAGGUCCUGGUAUGGUGAA 5'
AT1G66090.1     1050    1071
unknown protein

sRNA_AGO1_Solexa_Mi2008_12_20691_hit2
5' UACUUCCAGGACCAAACCACG
   |||||||||||||| |||||
   AUGAAGGUCCUGGUAUGGUGA 5'
AT1G66090.1     1051    1071
unknown protein

sRNA_AGO1_Solexa_Mi2008_2_8245_hit1
5' CGA-AAAGAAGAAGAUGAUGAG
    || |||||||||||||||||
   ACUAUUUCUUCUUCUACUACUA 5'
AT1G66090.1     105     126
unknown protein

sRNA_AGO1_Solexa_Mi2008_1_24884_hit1
5' UAUCAGGUGCAGAGCUUUGUC
   :|||||||| |||| ||||||
   GUAGUCCACCUCUCCAAACAG 5'
AT1G66090.1     1192    1212
unknown protein
```

```
sRNA_AGO1_Solexa_Mi2008_1_48406_hit1
5' UUCAAAAUACACAAGUUC-AAA
   |||| |||| |||||||| |||
   AAGUAUUAUCUGUUCAAGAUUU 5'
AT1G66090.1     1518    1539
unknown protein

sRNA_AGO1_Solexa_Mi2008_3_27133_hit1
5' UCAAG-CACCAGCUCGAAGAAGC
   ||||| ||||||||||| |||||
   AGUUCUGUGGUCGAGCUACUUCG 5'
AT1G66090.1     346     368
unknown protein

sRNA_AGO1_Solexa_Mi2008_1_27132_hit1
5' UCAAG-CACCAGCUCGAAGAAG
   ||||| ||||||||||| ||||
   AGUUCUGUGGUCGAGCUACUUC 5'
AT1G66090.1     347     368
unknown protein

sRNA_AGO1_Solexa_Mi2008_1_48406_hit1
5' UUCAAAAUACACAAGUUCAAA
   |||||||||||  | ||||||
   AAGUUUUAUGU-CU-AAGUUU 5'
AT1G66090.1     50      68
unknown protein

sRNA_AGO1_Solexa_Mi2008_3_39171_hit1
5' UGCUAGAGAGUAGGCUU-UGU
   |||||||||||||| || |||
   ACGAUCUCUCAUCC-AAUACA 5'
AT1G66090.1     795     814
unknown protein

sRNA_AGO1_Solexa_Mi2008_1_34620_hit1
5' UGAAGAUGAUGAUGAAGUU
    ||||||||||||||||
   UCUUCUACUACUACUUCUU 5'
AT1G66090.1     98      116
unknown protein

sRNA_AGO1_Solexa_Mi2008_1_2360_hit1
5' AUAACAAGUCCUGUUGGGUC
   | |||||||||| |||||||
   UCUUGUUCAGGAAAACCCAG 5'
AT1G68550.1     319     338
putative AP2 domain transcription factor

sRNA_AGO1_Solexa_Mi2008_1_2360_hit1
5' AUAACAAGUCCUGUUGGGUC
   | |||||||||| |||||||
   UCUUGUUCAGGAAAACCCAG 5'
AT1G68550.2     329     348
putative AP2 domain transcription factor

sRNA_AGO1_Solexa_Mi2008_1_20838_hit1
5' UAGAACCAAUUAUUUAUUGAA
   || ||| |||||||||||||
   AU-UUGCUUAAUAAAUAACUA 5'
AT1G68620.1     1274    1293
putative carboxylesterase
```

```
sRNA_AGO1_Solexa_Mi2008_1_4194_hit1
5' CAAAGGUGUG-AUCAGGAUGGU
   ||||||| || |||||||||||
   GUUUCCA-ACCUAGUCCUACCA 5'
AT1G68620.1     675     695
putative carboxylesterase

sRNA_AGO1_Solexa_Mi2008_1_18801_hit1
5' UAAUGGAGUUG-AUCACAUGA
   ||||||||| | |||||||||
   AUUACCUCA-CGUAGUGUACU 5'
AT1G68875.1     301     320

sRNA_AGO1_Solexa_Mi2008_4_21672_hit1
5' UAGAGUUGGGUAUGAUAGCUU
   :||||||||||||| || |||
   GUCUCAACCCAUAC-AU-GAA 5'
AT1G69180.1     582     600
transcription factor CRC

sRNA_AGO1_Solexa_Mi2008_2_7120_hit1
5' CAUGAAUUUGAGGUUUACAG
   ||||||||||||:||| |||
   GUACUUAAACUCUAAA-GUC 5'
AT1G69410.1     56      74
eukaryotic initiation factor 5A (eIF-5A) like protein

sRNA_AGO1_Solexa_Mi2008_1_24278_hit24
5' UA-UAAGGAGGAGACAAUCGAG
   || |||||||||||||| |||:
   AUCAUUCCUCCUCUGUUCGCUU 5'
AT1G69450.1     1205    1226
hypothetical protein

sRNA_AGO1_Solexa_Mi2008_1_55724_hit1
5' UUUGGGAGUUACGACUUUGA
   :|||| || |||||||||||
   GAACC-UCCAUGCUGAAACU 5'
AT1G69450.1     1380    1398
hypothetical protein

sRNA_AGO1_Solexa_Mi2008_2_36326_hit1
5' UGAGGAUGAUGGAUUU-AGAUC
   ||| |||||||| ||| |||||
   ACUGCUACUACC-AAAUUCUAG 5'
AT1G69490.1     862     882
unknown protein

sRNA_AGO1_Solexa_Mi2008_8_15630_hit1
5' GUCAUGGAAUAAGCUUGUUGG
    ||||||||  ||||||||||
   AAGUACCUUCGUCGAACAACC 5'
AT1G69850.1     1171    1191
nitrate transporter (NTL1)

sRNA_AGO1_Solexa_Mi2008_1_22549_hit1
5' UAGCUUCUUGAACCAGUUGAU
   |||||||||||||||||||||
   AUCGAAGAACUUGGUCAACUA 5'
AT1G69850.1     1801    1821
nitrate transporter (NTL1)

sRNA_AGO1_Solexa_Mi2008_1_1110_hit1
```

```
5' AAUGCUCAGGUCGAGGUGGUCC
   ||| |||||||||||||| |||
   UUA-GAGUCCAGCUCCAC-AGG 5'
AT1G69880.1     129     148
thioredoxin like protein

sRNA_AGO1_Solexa_Mi2008_1_1109_hit1
5' AAUGCUCAGGUCGAGGUGGUC
   ||| |||||||||||||| ||
   UUA-GAGUCCAGCUCCAC-AG 5'
AT1G69880.1     130     148
thioredoxin like protein

sRNA_AGO1_Solexa_Mi2008_1_20843_hit2
5' UAGAACCGUGAUUGCUCUGA
   |||||||| |||||||| ||
   AUCUUGGCUCUAACGAG-CU 5'
AT1G71200.1     501     519
hypothetical protein

sRNA_AGO1_Solexa_Mi2008_1_45596_hit2
5' UGUGUACA-GGCUAAGAUCUGG
    ||||| | ||||||||||||
   CCACAU-UACCGAUUCUAGACC 5'
AT1G72070.1     206     226
hypothetical protein

sRNA_AGO1_Solexa_Mi2008_1_51229_hit14
5' UUGAUUAUUGGAGGGUUUAGU
   |||||| ||||||||  ||||
   AACUAAAAACCUCCC--AUCA 5'
AT1G72520.1     1291    1309
putative lipoxygenase

sRNA_AGO1_Solexa_Mi2008_1_20232_hit22
5' UACGGAUAAGCCAUCAGAGUG
   ||||| |||||||||| |||
   AUGCCAAUUCGGUAGU-UCAA 5'
AT1G72520.1     1523    1542
putative lipoxygenase

sRNA_AGO1_Solexa_Mi2008_2_51167_hit1
5' UUGAUGGAGGAUUGUGUUC-CU
   |||||||||:|:||||||| ||
   AACUACCUCUUGACACAAGAGA 5'
AT1G72520.1     200     221
putative lipoxygenase

sRNA_AGO1_Solexa_Mi2008_11_14524_hit19
5' GCCAUUUGUUCUUGAGUAG
   ||||||||||||| | ||
   CGGUAAACAAGAA-U-AUG 5'
AT1G72520.1     2064    2080
putative lipoxygenase

sRNA_AGO1_Solexa_Mi2008_1_4981_hit1
5' CAAGUA-UUUAAGCCCAUUUA
   |||||| ||||||||||| |
   GUUCAUUAAAUUCGGUACAC 5'
AT1G72520.1     2150    2170
putative lipoxygenase

sRNA_AGO1_Solexa_Mi2008_1_20416_hit2
```

```
5' UACGUUGAUGAUAUGGUCA
   || ||||||||||||||
   AU-CAACUACUAUACCAUC 5'
AT1G72520.1     63      80
putative lipoxygenase

sRNA_AGO1_Solexa_Mi2008_1_54673_hit1
5' UUUCAAGUG-GUCCAAACAUAUCAG
   ||||| ||| ||||||| |||||||
   AAAGU-CACACAGGUUUCUAUAGUC 5'
AT1G72830.3     1238    1261
unknown protein

sRNA_AGO1_Solexa_Mi2008_1_54673_hit1
5' UUUCAAGUG-GUCCAAACAUAUCAG
   ||||| ||| ||||||| |||||||
   AAAGU-CACACAGGUUUCUAUAGUC 5'
AT1G72830.1     1314    1337
unknown protein

sRNA_AGO1_Solexa_Mi2008_1_54673_hit1
5' UUUCAAGUG-GUCCAAACAUAUCAG
   ||||| ||| ||||||| |||||||
   AAAGU-CACACAGGUUUCUAUAGUC 5'
AT1G72830.2     1317    1340
unknown protein

sRNA_AGO1_Solexa_Mi2008_17_12252_hit1
5' CUGUGGUGGGAACAUGAACUC
   || |||||||||||||||| |
   GA-ACCACCCUUGUACUUGUG 5'
AT1G72830.3     1391    1410
unknown protein

sRNA_AGO1_Solexa_Mi2008_26_14509_hit4
5' GCCAAGGAUGACUUGCCGG
   |||||||||||||| ||
   CGGUUCCUACUGAA-GGGG 5'
AT1G72830.3     1413    1430
unknown protein

sRNA_AGO1_Solexa_Mi2008_4_2048_hit4
5' AGCCAAGGAUGACUUGCCGG
   ||||||||||||||| ||
   UCGGUUCCUACUGAA-GGGG 5'
AT1G72830.3     1413    1431
unknown protein

sRNA_AGO1_Solexa_Mi2008_400_22176_hit7
5' UAGCCAAGGAUGACUUGCCUG
   |||||||||||||||| ||:
   AUCGGUUCCUACUGAA-GGGG 5'
AT1G72830.3     1413    1432
unknown protein

sRNA_AGO1_Solexa_Mi2008_2_15465_hit6
5' GUAGCCAAGGAUGACUUGCCUG
   ||||||||||||||||| ||:
   CAUCGGUUCCUACUGAA-GGGG 5'
AT1G72830.3     1413    1433
unknown protein

sRNA_AGO1_Solexa_Mi2008_78_6282_hit3
```

5' CAGCCAAGGAUGACUUGCCG
    ||||||||||||||| ||
   UUCGGUUCCUACUGAA-GGG 5'
AT1G72830.3   1414   1432
unknown protein

sRNA_AGO1_Solexa_Mi2008_9_22175_hit7
5' UAGCCAAGGAUGACUUGCCU
   |||||||||||||||| ||:
   AUCGGUUCCUACUGAA-GGG 5'
AT1G72830.3   1414   1432
unknown protein

sRNA_AGO1_Solexa_Mi2008_20_6281_hit3
5' CAGCCAAGGAUGACUUGCC
    ||||||||||||||| ||
   UUCGGUUCCUACUGAA-GG 5'
AT1G72830.3   1415   1432
unknown protein

sRNA_AGO1_Solexa_Mi2008_7_22174_hit7
5' UAGCCAAGGAUGACUUGCC
   |||||||||||||||| ||
   AUCGGUUCCUACUGAA-GG 5'
AT1G72830.3   1415   1432
unknown protein

sRNA_AGO1_Solexa_Mi2008_1_28302_hit1
5' UCAGCCAAGGAUGACUUGCC
   : ||||||||||||||| ||
   GUUCGGUUCCUACUGAA-GG 5'
AT1G72830.3   1415   1433
unknown protein

sRNA_AGO1_Solexa_Mi2008_4_36086_hit4
5' UGAGCCAAGGAUGACUUGCC
   ::||||||||||||||| ||
   GUUCGGUUCCUACUGAA-GG 5'
AT1G72830.3   1415   1433
unknown protein

sRNA_AGO1_Solexa_Mi2008_17_12252_hit1
5' CUGUGGUGGGAACAUGAACUC
   || |||||||||||||||| |
   GA-ACCACCCUUGUACUUGUG 5'
AT1G72830.1   1467   1486
unknown protein

sRNA_AGO1_Solexa_Mi2008_17_12252_hit1
5' CUGUGGUGGGAACAUGAACUC
   || |||||||||||||||| |
   GA-ACCACCCUUGUACUUGUG 5'
AT1G72830.2   1470   1489
unknown protein

sRNA_AGO1_Solexa_Mi2008_26_14509_hit4
5' GCCAAGGAUGACUUGCCGG
   |||||||||||||| ||
   CGGUUCCUACUGAA-GGGG 5'
AT1G72830.1   1489   1506
unknown protein

sRNA_AGO1_Solexa_Mi2008_4_2048_hit4

```
5' AGCCAAGGAUGACUUGCCGG
   ||||||||||||||| ||
   UCGGUUCCUACUGAA-GGGG 5'
AT1G72830.1     1489    1507
unknown protein

sRNA_AGO1_Solexa_Mi2008_400_22176_hit7
5' UAGCCAAGGAUGACUUGCCUG
   |||||||||||||||| ||:
   AUCGGUUCCUACUGAA-GGGG 5'
AT1G72830.1     1489    1508
unknown protein

sRNA_AGO1_Solexa_Mi2008_2_15465_hit6
5' GUAGCCAAGGAUGACUUGCCUG
   ||||||||||||||||| ||:
   CAUCGGUUCCUACUGAA-GGGG 5'
AT1G72830.1     1489    1509
unknown protein

sRNA_AGO1_Solexa_Mi2008_78_6282_hit3
5' CAGCCAAGGAUGACUUGCCG
    ||||||||||||||| ||
   UUCGGUUCCUACUGAA-GGG 5'
AT1G72830.1     1490    1508
unknown protein

sRNA_AGO1_Solexa_Mi2008_9_22175_hit7
5' UAGCCAAGGAUGACUUGCCU
   |||||||||||||||| ||:
   AUCGGUUCCUACUGAA-GGG 5'
AT1G72830.1     1490    1508
unknown protein

sRNA_AGO1_Solexa_Mi2008_20_6281_hit3
5' CAGCCAAGGAUGACUUGCC
    ||||||||||||||| ||
   UUCGGUUCCUACUGAA-GG 5'
AT1G72830.1     1491    1508
unknown protein

sRNA_AGO1_Solexa_Mi2008_7_22174_hit7
5' UAGCCAAGGAUGACUUGCC
   |||||||||||||||| ||
   AUCGGUUCCUACUGAA-GG 5'
AT1G72830.1     1491    1508
unknown protein

sRNA_AGO1_Solexa_Mi2008_1_28302_hit1
5' UCAGCCAAGGAUGACUUGCC
   : ||||||||||||||| ||
   GUUCGGUUCCUACUGAA-GG 5'
AT1G72830.1     1491    1509
unknown protein

sRNA_AGO1_Solexa_Mi2008_4_36086_hit4
5' UGAGCCAAGGAUGACUUGCC
   ::||||||||||||||| ||
   GUUCGGUUCCUACUGAA-GG 5'
AT1G72830.1     1491    1509
unknown protein

sRNA_AGO1_Solexa_Mi2008_26_14509_hit4
```

```
5' GCCAAGGAUGACUUGCCGG
   |||||||||||||| ||
   CGGUUCCUACUGAA-GGGG 5'
AT1G72830.2     1492    1509
unknown protein

sRNA_AGO1_Solexa_Mi2008_4_2048_hit4
5' AGCCAAGGAUGACUUGCCGG
   ||||||||||||||| ||
   UCGGUUCCUACUGAA-GGGG 5'
AT1G72830.2     1492    1510
unknown protein

sRNA_AGO1_Solexa_Mi2008_400_22176_hit7
5' UAGCCAAGGAUGACUUGCCUG
   |||||||||||||||| ||:
   AUCGGUUCCUACUGAA-GGGG 5'
AT1G72830.2     1492    1511
unknown protein

sRNA_AGO1_Solexa_Mi2008_2_15465_hit6
5' GUAGCCAAGGAUGACUUGCCUG
   ||||||||||||||||| ||:
   CAUCGGUUCCUACUGAA-GGGG 5'
AT1G72830.2     1492    1512
unknown protein

sRNA_AGO1_Solexa_Mi2008_78_6282_hit3
5' CAGCCAAGGAUGACUUGCCG
    ||||||||||||||| ||
   UUCGGUUCCUACUGAA-GGG 5'
AT1G72830.2     1493    1511
unknown protein

sRNA_AGO1_Solexa_Mi2008_9_22175_hit7
5' UAGCCAAGGAUGACUUGCCU
   |||||||||||||||| ||:
   AUCGGUUCCUACUGAA-GGG 5'
AT1G72830.2     1493    1511
unknown protein

sRNA_AGO1_Solexa_Mi2008_20_6281_hit3
5' CAGCCAAGGAUGACUUGCC
    ||||||||||||||| ||
   UUCGGUUCCUACUGAA-GG 5'
AT1G72830.2     1494    1511
unknown protein

sRNA_AGO1_Solexa_Mi2008_7_22174_hit7
5' UAGCCAAGGAUGACUUGCC
   |||||||||||||||| ||
   AUCGGUUCCUACUGAA-GG 5'
AT1G72830.2     1494    1511
unknown protein

sRNA_AGO1_Solexa_Mi2008_1_28302_hit1
5' UCAGCCAAGGAUGACUUGCC
   : ||||||||||||||| ||
   GUUCGGUUCCUACUGAA-GG 5'
AT1G72830.2     1494    1512
unknown protein

sRNA_AGO1_Solexa_Mi2008_4_36086_hit4
```

```
5' UGAGCCAAGGAUGACUUGCC
   ::||||||||||||||| ||
   GUUCGGUUCCUACUGAA-GG 5'
AT1G72830.2     1494    1512
unknown protein

sRNA_AGO1_Solexa_Mi2008_5_24767_hit1
5' UAUCAAAAGAAUCAU-UGCACU
   ||||| ||||||||| | ||||
   AUAGUCUUCUUAGUAUA-GUGA 5'
AT1G72830.3     1579    1599
unknown protein

sRNA_AGO1_Solexa_Mi2008_5_24767_hit1
5' UAUCAAAAGAAUCAU-UGCACU
   ||||| ||||||||| | ||||
   AUAGUCUUCUUAGUAUA-GUGA 5'
AT1G72830.1     1655    1675
unknown protein

sRNA_AGO1_Solexa_Mi2008_5_24767_hit1
5' UAUCAAAAGAAUCAU-UGCACU
   ||||| ||||||||| | ||||
   AUAGUCUUCUUAGUAUA-GUGA 5'
AT1G72830.2     1658    1678
unknown protein

sRNA_AGO1_Solexa_Mi2008_1_40813_hit2
5' UGGAUAUAAGAGCUGGGCUGU
   |||||||||||| ||| |||
   ACCUAUAUUCUC-ACCAGACU 5'
AT1G73220.1     336     355
putative transporter

sRNA_AGO1_Solexa_Mi2008_2_14040_hit1
5' GAGGAAAUAUAUUUGAUUU
    |||||||||||||| |||
   GUCCUUUAUAUAAAC-AAA 5'
AT1G73220.1     75      92
putative transporter

sRNA_AGO1_Solexa_Mi2008_1_38353_hit4
5' UGCCAAGGAGUCGCAACUCCGA
   ||||| | |||||||| |||||
   ACGGU-C-UCAGCGUUCAGGCU 5'
AT1G73260.1     631     650
putative trypsin inhibitor (At1g73260)

sRNA_AGO1_Solexa_Mi2008_6_35858_hit1
5' UGAGACGCAAGGCGAUGAUGAG
   ||||||| |||| ||||||||
   ACUCUGCUUUCCUCUACUACUA 5'
AT1G73325.1     236     257

sRNA_AGO1_Solexa_Mi2008_10_4341_hit1
5' CAACAAUGGAGCUUCAAGAGGA
   |||||||||||| | || ||||
   GUUGUUACCUCG-A-UUAUCCU 5'
AT1G73325.1     495     514

sRNA_AGO1_Solexa_Mi2008_1_37225_hit4
5' UGAUGAUGAUGAUGAUG-AAGAAG
   |||||||||||||||| ||||||
```

```
   ACUACUACUACUACUACUUUCUUC 5'
AT1G73500.1     129     152
unknown protein

sRNA_AGO1_Solexa_Mi2008_1_45036_hit1
5' UGUGAUGAGGAGGAUGAUGAA
    ||||||| || |||||||||
   UCACUACUACUACUACUACUU 5'
AT1G73500.1     133     153
unknown protein

sRNA_AGO1_Solexa_Mi2008_1_56092_hit3
5' UUUGUGAUGAUGAUGAUGAUGA
      ||||||||||||||||||
   UCUCACUACUACUACUACUACU 5'
AT1G73500.1     134     155
unknown protein

sRNA_AGO1_Solexa_Mi2008_6_14255_hit369
5' GAUGA-UGAUGAUGAUGAUGAUGAU
   || || |||||||||||||||||
   CU-CUCACUACUACUACUACUACUU 5'
AT1G73500.1     134     157
unknown protein

sRNA_AGO1_Solexa_Mi2008_1_36323_hit13
5' UGAG-GAUGAUGAUGAUGAUGA
   |||| ||||||||||||||||
   ACUCACUACUACUACUACUACU 5'
AT1G73500.1     135     156
unknown protein

sRNA_AGO1_Solexa_Mi2008_1_3079_hit472
5' AUGA-UGAUGAUGAUGAUGAUGA
    ||| |||||||||||||||||
   GACUCACUACUACUACUACUACU 5'
AT1G73500.1     135     157
unknown protein

sRNA_AGO1_Solexa_Mi2008_1_45039_hit3
5' UGUGA-UGAUGAUGAUGAUGAUGA
   | ||| |||||||||||||||||
   AGACUCACUACUACUACUACUACU 5'
AT1G73500.1     135     158
unknown protein

sRNA_AGO1_Solexa_Mi2008_1_2280_hit1
5' AGUGGAUGAUGAUGAUGAUG
   |||| ||||||||||||||
   UCAC-UACUACUACUACUAC 5'
AT1G73500.1     136     154
unknown protein

sRNA_AGO1_Solexa_Mi2008_1_6801_hit1
5' CAUAAACAGCAGGACGAUCCU
   ||||||||||||||||||||
   GUAUUUGUCGUCCUGCUAGGA 5'
AT1G74100.1     1051    1071
putative flavonol sulfotransferase

sRNA_AGO1_Solexa_Mi2008_2_56163_hit1
5' UUUGUGU-GUCUUUGAUUC-AUU
   |||| || ||||||||||| |||
```

```
   AAACCCAACAGAAACUAAGGUAA 5'
AT1G74100.1     189     211
putative flavonol sulfotransferase

sRNA_AGO1_Solexa_Mi2008_1_36550_hit1
5' UGAGGGUUUCGUUUGAGGAGU
   |||||||||||||||||||||
   ACUCCCAAAGCAAACUCCUCA 5'
AT1G74100.1     506     526
putative flavonol sulfotransferase

sRNA_AGO1_Solexa_Mi2008_1_18261_hit1
5' UAAGGGACAAACUCGUGAGGG
   |||||||||||||||||||||
   AUUCCCUGUUUGAGCACUCCC 5'
AT1G74100.1     521     541
putative flavonol sulfotransferase

sRNA_AGO1_Solexa_Mi2008_1_4144_hit1
5' CAAAGGAUUGGCCCUCAUGGUC
   ||||||||||||||||||||||
   GUUUCCUAACCGGGAGUACCAG 5'
AT1G74100.1     884     905
putative flavonol sulfotransferase

sRNA_AGO1_Solexa_Mi2008_1_36898_hit1
5' UGAGUUGGAUGAUGAUGAUGA
   ||||| | |||||||||||||
   ACUCA-C-UACUACUACUACU 5'
AT1G74450.1     100     118
unknown protein

sRNA_AGO1_Solexa_Mi2008_1_3079_hit472
5' AUGAUGA-UGAUGAUGAUGAUGA
    ||| || |||||||||||||||
   GACU-CUCACUACUACUACUACU 5'
AT1G74450.1     100     121
unknown protein

sRNA_AGO1_Solexa_Mi2008_1_36323_hit13
5' UGAG-GA-UGAUGAUGAUGAUGA
   |||| || |||||||||||||||
   ACUCUCUCACUACUACUACUACU 5'
AT1G74450.1     100     122
unknown protein

sRNA_AGO1_Solexa_Mi2008_1_14208_hit2
5' GAUCGAUAAACCUCUGCAUC
   ||||||||||||||| || |
   CUAGCUAUUUGGAGA-GUCG 5'
AT1G74450.1     376     394
unknown protein

sRNA_AGO1_Solexa_Mi2008_3_8704_hit1
5' CGAUCGAUAAACCUCUGCAU
    ||||||||||||||| ||
   ACUAGCUAUUUGGAGA-GUC 5'
AT1G74450.1     377     395
unknown protein

sRNA_AGO1_Solexa_Mi2008_2_21756_hit1
5' UAGAUCGAUAAACCUCUGCAU
   :|||||||||||||||| ||
```

```
   GUCUAGCUAUUUGGAGA-GUC 5'
AT1G74450.1     377     396
unknown protein

sRNA_AGO1_Solexa_Mi2008_1_37225_hit4
5' UGA-UGAUGAUGAUGAUGAAGAAG
   ||| ||||||||||||||| ||
   ACUCACUACUACUACUACUU-UUA 5'
AT1G74450.1     96      118
unknown protein

sRNA_AGO1_Solexa_Mi2008_1_2280_hit1
5' AGUGGAUGAUGAUGAUGAUG
   |||| ||||||||||||| :
   UCAC-UACUACUACUACUUU 5'
AT1G74450.1     98      116
unknown protein

sRNA_AGO1_Solexa_Mi2008_9_14254_hit8
5' GAUGAUGAUGAUGAUGAUCUU
   || |||||||||| ||||||
   CU-CUACUACUACCACUAGAU 5'
AT1G76580.1     615     634
Squamosa promoter binding protein-like 16 (SPL16)

sRNA_AGO1_Solexa_Mi2008_1_49672_hit1
5' UUCUACAGCAAAAAAUCUAUAA
   || |||||||||||| |||||
   AA-AUGUCGUUUUUUUGAUAUA 5'
AT1G76590.1     1135    1155
unknown protein

sRNA_AGO1_Solexa_Mi2008_3_4962_hit3
5' CAAGUAAUAACAUUUAACCU
   |||||||| |||||||||
   GUUCAUUAGUGUAAAUUGCC 5'
AT1G76590.1     773     792
unknown protein

sRNA_AGO1_Solexa_Mi2008_21_8641_hit2
5' CGAGUCAGCAUUCCAGAACCUA
   ||| |||| ||||||||||||
   GCUAAGUC-UAAGGUCUUGGAC 5'
AT1G76600.1     202     222
unknown protein

sRNA_AGO1_Solexa_Mi2008_1_37225_hit4
5' UGAUGAUGAUGAUGAUGAAGAAG
    |||||||||||||| |||||||
   UCUACUACUACUACUUCUUCUUC 5'
AT1G76600.1     226     248
unknown protein

sRNA_AGO1_Solexa_Mi2008_3_13421_hit1
5' GAAGAAGAAGAUGAUGUUGAU
   |||||||| ||||||| |||
   CUUCUUCUACUACUACUACUU 5'
AT1G76600.1     233     253
unknown protein

sRNA_AGO1_Solexa_Mi2008_3_13421_hit1
5' GAAGAAGAAGAUGAUGUUGAU
    ||||||||||||||| ||||
```

```
   AUUCUUCUUCUACUACUACUA 5'
AT1G76600.1     236     256
unknown protein

sRNA_AGO1_Solexa_Mi2008_1_53872_hit1
5' UUUAA-AAUGAAGAUGAUGAUGA
   ||||| || |||||||||||||
   AAAUUCUU-CUUCUACUACUACU 5'
AT1G76600.1     238     259
unknown protein

sRNA_AGO1_Solexa_Mi2008_4_12045_hit2
5' CUGGUAAUUGGAAUG-AGUAC
   ||||||||||||| | ||||
   GACCAUUAACCUU-CUUCAUC 5'
AT1G77120.1     2       21
alcohol dehydrogenase

sRNA_AGO1_Solexa_Mi2008_5_12044_hit2
5' CUGGUAAUUGGAAUG-AGUA
   ||||||||||||| | ||||
   GACCAUUAACCUU-CUUCAU 5'
AT1G77120.1     3       21
alcohol dehydrogenase

sRNA_AGO1_Solexa_Mi2008_1_3_hit25
5' AAAAAAAAAAAAAGAAAGA
     ||||||||||| |||||
   CCUUUUUUUUUUUGUUUCU 5'
AT1G78370.1     789     807
unknown protein

sRNA_AGO1_Solexa_Mi2008_1_50384_hit1
5' UUGACGGACGAAGUAAGUGGA
   |||||||||||||||| ||||
   AACUGCCUGCUUCAUUAACCU 5'
AT1G78820.1     1017    1037
Strong similarity to glycoprotein EP1

sRNA_AGO1_Solexa_Mi2008_1_50384_hit1
5' UUGACGGACGAAGUAAGUGGA
   |||||||||||||||||||||
   AACUGCCUGCUUCAUUCACCU 5'
AT1G78830.1     1016    1036
unknown protein (At1g78830)

sRNA_AGO1_Solexa_Mi2008_2_35025_hit1
5' UGAAUGAUGAUGUGAAAGAA
   :||||| ||||||| |||||
   GCUUACCACUACAC-UUCUU 5'
AT1G78830.1     219     237
unknown protein (At1g78830)

sRNA_AGO1_Solexa_Mi2008_1_13416_hit2
5' GAAGAAAGCGAUGAUGACUCAG
   ||||||| ||||||||| | ||
   CUUCUUUGGCUACUACUAA-UC 5'
AT1G79900.1     257     277
putative carnitine/acylcarnitine translocase

sRNA_AGO1_Solexa_Mi2008_1_22821_hit1
5' UAGGAUGAAACCGAUGAUGAG
   |||:| ||||||||||||||
```

```
   AUCUU-CUUUGGCUACUACUA 5'
AT1G79900.1     260     279
putative carnitine/acylcarnitine translocase

sRNA_AGO1_Solexa_Mi2008_3_38618_hit1
5' UGCCGGAGAUGAUGGACGG-GA
   ||||||||||||| | ||| ||
   ACGGCCUCUACUA-CGGCCGCU 5'
AT1G79900.1     408     428
putative carnitine/acylcarnitine translocase

sRNA_AGO1_Solexa_Mi2008_37_14464_hit3
5' GCAGCAAGGCCACUCUGCCAC
   ||||||||||||| | |||||
   CGUCGUUCCGGUG-GUCGGUG 5'
AT1G79900.1     950     969
putative carnitine/acylcarnitine translocase

sRNA_AGO1_Solexa_Mi2008_1_41610_hit1
5' UGGCUGAACGAGUUUGGCAGG
   ||||||||||||:| ||| ||
   ACCGACUUGCUCGA-CCG-CC 5'
AT1G80160.1     566     584
unknown protein

sRNA_AGO1_Solexa_Mi2008_1_5313_hit2
5' CACACGCAAU-AAAGUAGACAAG
   |||||||||| |||| ||||||
   GUGUGCGUUAGUUUC-UCUGUUA 5'
AT1G80160.1     658     679
unknown protein

sRNA_AGO1_Solexa_Mi2008_1_41610_hit1
5' UGGCUGAACGAGUUUGGCAGG
   |||||||||||| :|||| ||
   ACCGACUUGCUC-GACCG-CC 5'
AT1G80160.2     664     682
unknown protein

sRNA_AGO1_Solexa_Mi2008_1_5313_hit2
5' CACACGCAAU-AAAGUAGACAAG
   |||||||||| |||| ||||||
   GUGUGCGUUAGUUUC-UCUGUUA 5'
AT1G80160.2     756     777
unknown protein

sRNA_AGO1_Solexa_Mi2008_17_5479_hit1
5' CACCAUGGAAAGGCUGAAG
     |||||| ||||||||||
   UCGGUACCCUUCCGACUUC 5'
AT1G80300.1     167     185
adenine nucleotide translocase

sRNA_AGO1_Solexa_Mi2008_2_17207_hit1
5' UAACACCACCAAGAC-UCCU
     ||||||||||||| ||||
   CGUGUGGUGGUUCUGUAGGA 5'
AT1G80300.1     508     527
adenine nucleotide translocase

sRNA_AGO1_Solexa_Mi2008_8_4655_hit1
5' CAAGAGUGUUUGAGAGAUA
   |||||||||||| | |||
```

```
   GUUCUCACAAAC-C-CUAG 5'
AT2G05910.1     325     341
unknown protein

sRNA_AGO1_Solexa_Mi2008_1_55155_hit1
5' UUUGA-GAUGAUGAUGACUAU
   :|||| |||||||||||||:
   GAACUACUACUACUACUGAUG 5'
AT2G05940.1     1242    1262
putative protein kinase

sRNA_AGO1_Solexa_Mi2008_9_14254_hit8
5' GAUGAUGAUGAUGAUGAUCUU
   |||||||||||||||| ||
   CUACUACUACUACUACU-GAU 5'
AT2G05940.1     1243    1262
putative protein kinase

sRNA_AGO1_Solexa_Mi2008_7_3078_hit1
5' AUGAUGAUGA-GAAUGAUGAU
   |||||||||| || ||||||:
   UACUACUACUACU-ACUACUG 5'
AT2G05940.1     1245    1264
putative protein kinase

sRNA_AGO1_Solexa_Mi2008_1_7931_hit2
5' CCCGAUGAUGAUGAUGA-GAC
   |  |||||||||||||| |||
   GUUCUACUACUACUACUACUG 5'
AT2G05940.1     1245    1265
putative protein kinase

sRNA_AGO1_Solexa_Mi2008_1_56092_hit3
5' UUUGU-GAUGAUGAUGAUGAUGA
   :|||| ||||||||||||||||
   GAACAACUACUACUACUACUACU 5'
AT2G05940.1     1246    1268
putative protein kinase

sRNA_AGO1_Solexa_Mi2008_2_4635_hit1
5' CAAGAGGACCCAAAGUUGCGA
   |||||||||||||||||||||
   GUUCUCCUGGGUUUCAACGCU 5'
AT2G15090.1     1077    1097
putative fatty acid elongase

sRNA_AGO1_Solexa_Mi2008_1_40614_hit1
5' UGGAGGUCGAGUGAUGGCGUUG
   |||||| |||||||||| ||||
   ACCUCC-GCUCACUACCUCAAC 5'
AT2G15090.1     634     654
putative fatty acid elongase

sRNA_AGO1_Solexa_Mi2008_1_40847_hit1
5' UGGAUCCGUACGGUGUGAACA
   |||||||||||||||||||||
   ACCUAGGCAUGCCACACUUGU 5'
AT2G15090.1     934     954
putative fatty acid elongase

sRNA_AGO1_Solexa_Mi2008_1_22510_hit1
5' UAGCU-GCCGGAGACAACAUU
   ||| | ||||||||| |||||
```

```
   AUC-AGCGGCCUCUGCUGUAA 5'
AT2G17230.1     33      52
unknown protein

sRNA_AGO1_Solexa_Mi2008_1_56662_hit4
5' UUUUUGAAGGCAAGA-GA-GAGA
   ||||| ||||||||| || ||||
   AAAAAGUUCCGUUCUACUACUCU 5'
AT2G21050.1     48      70
AUX1-like amino acid permease

sRNA_AGO1_Solexa_Mi2008_10_30296_hit1
5' UCCUGUAGUUGUUGGUAGCGA
   |||||||||||| || || ||
   AGGACAUCAACA-CCUUC-CU 5'
AT2G21050.1     746     764
AUX1-like amino acid permease

sRNA_AGO1_Solexa_Mi2008_1_51316_hit1
5' UUGCAACAGUAGCG-CCGAAA
    ||||||||||||| | ||||
   CACGUUGUCAUCGCUGUCUUU 5'
AT2G21620.1     789     809
RD2 protein (RD2)

sRNA_AGO1_Solexa_Mi2008_1_51316_hit1
5' UUGCAACAGUAGCG-CCGAAA
    ||||||||||||| | ||||
   CACGUUGUCAUCGCUGUCUUU 5'
AT2G21620.2     807     827
RD2 protein (RD2)

sRNA_AGO1_Solexa_Mi2008_1_23262_hit1
5' UAGGGUCUUGAAACGUUGUGAG
   ||||| |||||| |||||||||
   AUCCC-GAACUUCGCAACACUC 5'
AT2G22330.1     129     149
putative cytochrome P450

sRNA_AGO1_Solexa_Mi2008_1_21384_hit1
5' UAGAGGAGGCGCGUUGACGGU
   |||||||||||||||||||||
   AUCUCCUCCGCGCAACUGCCA 5'
AT2G22500.1     438     458
putative mitochondrial dicarboxylate carrier protein

sRNA_AGO1_Solexa_Mi2008_2_4570_hit2
5' CAAGAAGAGAUUCCAU-AGU
   |||||||||| ||||| |||
   GUUCUUCUCUUAGGUAGUCA 5'
AT2G22840.1     233     252
unknown protein

sRNA_AGO1_Solexa_Mi2008_3_5131_hit1
5' CAAUGAAAAAGGGCCUAUU-CUC
   |||||||||||| ||| || |||
   GUUACUUUUUCCAGGAAAAUGAG 5'
AT2G22840.1     471     493
unknown protein

sRNA_AGO1_Solexa_Mi2008_9_5355_hit1
5' CACAG-CUUUCUUGAACUUU
   ||||| |||||||||||  |
```

```
   GUGUCCGAAAGAACUUGCUA 5'
AT2G22840.1     780     799
unknown protein

sRNA_AGO1_Solexa_Mi2008_138_7679_hit1
5' CCACAG-CUUUCUUGAACUG
   |||||| ||||||||||| :
   GGUGUCCGAAAGAACUUGCU 5'
AT2G22840.1     781     800
unknown protein

sRNA_AGO1_Solexa_Mi2008_54_7680_hit1
5' CCACAG-CUUUCUUGAACUU
   |||||| |||||||||||
   GGUGUCCGAAAGAACUUGCU 5'
AT2G22840.1     781     800
unknown protein

sRNA_AGO1_Solexa_Mi2008_18_29330_hit1
5' UCCACAG-CUUUCUUGAACUG
   ||||||| ||||||||||| :
   AGGUGUCCGAAAGAACUUGCU 5'
AT2G22840.1     781     801
unknown protein

sRNA_AGO1_Solexa_Mi2008_11_29331_hit1
5' UCCACAG-CUUUCUUGAACUU
   ||||||| |||||||||||
   AGGUGUCCGAAAGAACUUGCU 5'
AT2G22840.1     781     801
unknown protein

sRNA_AGO1_Solexa_Mi2008_1449_48967_hit1
5' UUCCACAG-CUUUCUUGAACUG
   |||||||| ||||||||||| :
   AAGGUGUCCGAAAGAACUUGCU 5'
AT2G22840.1     781     802
unknown protein

sRNA_AGO1_Solexa_Mi2008_809_48968_hit1
5' UUCCACAG-CUUUCUUGAACUU
   |||||||| |||||||||||
   AAGGUGUCCGAAAGAACUUGCU 5'
AT2G22840.1     781     802
unknown protein

sRNA_AGO1_Solexa_Mi2008_9_12589_hit1
5' CUUCCACAG-CUUUCUUGAACUG
   ||||||||| ||||||||||| :
   GAAGGUGUCCGAAAGAACUUGCU 5'
AT2G22840.1     781     803
unknown protein

sRNA_AGO1_Solexa_Mi2008_8_48966_hit2
5' UUCCACAG-CUUUCUUGAACU
   |||||||| |||||||||||
   AAGGUGUCCGAAAGAACUUGC 5'
AT2G22840.1     782     802
unknown protein

sRNA_AGO1_Solexa_Mi2008_5_12588_hit1
5' CUUCCACAG-CUUUCUUGAACU
   ||||||||| |||||||||||
```

```
   GAAGGUGUCCGAAAGAACUUGC 5'
AT2G22840.1     782     803
unknown protein

sRNA_AGO1_Solexa_Mi2008_1_42987_hit1
5' UGGUAUGAUGAUGUGUUACUCA
   |||||||||| |||||| | ||
   ACCAUACUACAACACAA-G-GU 5'
AT2G22840.1     949     968
unknown protein

sRNA_AGO1_Solexa_Mi2008_1_28594_hit1
5' UCA-GGUGAGAUUAGAGGAGAUU
   ||| ||||||||| | |||||||
   AGUGCCACUCUAAACCCCUCUAA 5'
AT2G22900.1     264     286
unknown protein

sRNA_AGO1_Solexa_Mi2008_1_1776_hit10
5' AGAAAAAAA-AAAAAA-AAAAAU
   | ||||||| |||||| ||||||
   UAUUUUUUUAUUUUUUAUUUUUA 5'
AT2G23790.1     1293    1315
unknown protein

sRNA_AGO1_Solexa_Mi2008_1_1776_hit10
5' AGAAA-AAAAAAA-AAAAAAAAU
   | ||| ||||||| |||||||||
   UAUUUAUUUUUUUAUUUUUUUUA 5'
AT2G23790.1     1306    1328
unknown protein

sRNA_AGO1_Solexa_Mi2008_1_21596_hit4
5' UAGAGUGA-ACAAUGAUGAG
    ||||| | |||||||||||
   CUCUCA-UCUGUUACUACUC 5'
AT2G23810.1     942     960
similar to senescence-associated protein

sRNA_AGO1_Solexa_Mi2008_1_12714_hit5
5' CUUCUUGUUGAGUGCCGCGAG
    ||||||||  ||||||||||
   UAAGAACAAAGCACGGCGCUC 5'
AT2G24600.3     842     862
unknown protein

sRNA_AGO1_Solexa_Mi2008_1_10750_hit1
5' CUCAGUGCCUUCAUCUUCGUC
   ||| | ||||||| |||||||
   GAG-C-CGGAAGUCGAAGCAG 5'
AT2G24850.1     391     409
putative tyrosine aminotransferase

sRNA_AGO1_Solexa_Mi2008_1_33497_hit1
5' UCUGCAGCACUUGGUGAAGUA
   ||| | | |||||||||||||
   AGA-G-CUUGAACCACUUCAU 5'
AT2G25450.1     1019    1037
putative dioxygenase

sRNA_AGO1_Solexa_Mi2008_3_49087_hit1
5' UUCCGAGGUGGUGUGGUCU
   ||||||||||||||  |||
```

```
   AAGGCUCCACCACAG-AGA 5'
AT2G25450.1     1089    1106
putative dioxygenase

sRNA_AGO1_Solexa_Mi2008_9_52832_hit1
5' UUGGGUUGGU-UCGGGUAUAGAA
   |||| || || |||||||||||
   AACCAAAACAGAGCCCAUAUCUU 5'
AT2G25450.1     450     472
putative dioxygenase

sRNA_AGO1_Solexa_Mi2008_1_2638_hit1
5' AUAGGUUGUGCAGUUUGAGCU
   ||||| | ||||||||||||
   UAUCCUAGACGUCAAACUCGA 5'
AT2G25490.1     1962    1982
putative glucose regulated repressor protein

sRNA_AGO1_Solexa_Mi2008_1_21036_hit2
5' UA-GACAGUUGCCG-CACCCACA
   || ||||||||||| ||||| ||
   AUGCUGUCAACGGCGGUGGGGGU 5'
AT2G25625.2     254     276
unknown protein

sRNA_AGO1_Solexa_Mi2008_1_21036_hit2
5' UA-GACAGUUGCCGC-ACCCACA
   || |||||||||||| |||| ||
   AUGCUGUCAACGGCGGUGGGGGU 5'
AT2G25625.1     289     311
unknown protein

sRNA_AGO1_Solexa_Mi2008_2_26713_hit3
5' UC-AAAGCCAAAUCAAUAUCAC
   || ||||| |||||||||||
   AGCUUUCGCUUUAGUUAUAGUA 5'
AT2G26530.2     431     452
AR781, similar to yeast pheromone receptor

sRNA_AGO1_Solexa_Mi2008_1_10600_hit3
5' CUC-AAAGCCAAAUCAAUAUCAC
   ||| ||||| |||||||||||
   GAGCUUUCGCUUUAGUUAUAGUA 5'
AT2G26530.2     431     453
AR781, similar to yeast pheromone receptor

sRNA_AGO1_Solexa_Mi2008_3_26712_hit3
5' UC-AAAGCCAAAUCAAUAUCA
   || ||||| |||||||||||
   AGCUUUCGCUUUAGUUAUAGU 5'
AT2G26530.2     432     452
AR781, similar to yeast pheromone receptor

sRNA_AGO1_Solexa_Mi2008_2_26713_hit3
5' UC-AAAGCCAAAUCAAUAUCAC
   || ||||| |||||||||||
   AGCUUUCGCUUUAGUUAUAGUA 5'
AT2G26530.1     449     470
AR781, similar to yeast pheromone receptor

sRNA_AGO1_Solexa_Mi2008_1_10600_hit3
5' CUC-AAAGCCAAAUCAAUAUCAC
   ||| ||||| |||||||||||
```

```
   GAGCUUUCGCUUUAGUUAUAGUA 5'
AT2G26530.1     449     471
AR781, similar to yeast pheromone receptor

sRNA_AGO1_Solexa_Mi2008_3_26712_hit3
5' UC-AAAGCCAAAUCAAUAUCA
   || ||||| ||||||||||||
   AGCUUUCGCUUUAGUUAUAGU 5'
AT2G26530.1     450     470
AR781, similar to yeast pheromone receptor

sRNA_AGO1_Solexa_Mi2008_1_14490_hit1
5' GCAUCAAUGAUAGUGUUGUAG
   ||||||||||||| | |||||
   CGUAGUUACUAUC-C-ACAUC 5'
AT2G26560.1     961     979
similar to latex allergen from Hevea brasiliensis

sRNA_AGO1_Solexa_Mi2008_1_806_hit1
5' AAGAGCAUCAAUGAUAG-UGUU
   || |||||||||||||| |||
   UU-UCGUAGUUACUAUCCACAU 5'
AT2G26560.1     962     982
similar to latex allergen from Hevea brasiliensis

sRNA_AGO1_Solexa_Mi2008_1_5212_hit1
5' CAAUUAAGUUUUAUGGGCUCUC
   |||||||||||:||  ||||||
   GUUAAUUCAAAGUA--CGAGAG 5'
AT2G27310.1     514     533
Unknown protein (At2g27310; F12K2.11)

sRNA_AGO1_Solexa_Mi2008_2_15799_hit1
5' GUCUGAAGGAUUAGAGGAAC
   || ||||||||| ||||||
   CA-ACUUCCUAACCUCCUUC 5'
AT2G28190.1     109     127
putative copper/zinc superoxide dismutase

sRNA_AGO1_Solexa_Mi2008_1_11584_hit1
5' CUGAUGACAUAAA-UUUACAU
   ||||||||||||| |||| ||
   GACUACUGUAUUUGAAAU-UA 5'
AT2G28510.1     1045    1064
DOF zinc finger like protein

sRNA_AGO1_Solexa_Mi2008_3_35894_hit1
5' UGAGAGCAGAGAAAGAGAGU
   |||||| ||||||||||||
   ACUCUC-UCUCUUUCUCUCU 5'
AT2G28510.1     24      42
DOF zinc finger like protein

sRNA_AGO1_Solexa_Mi2008_8_13576_hit1
5' GACAAACUAAUGUUAUAUAGU
   ||||||||| ||||||| |||
   CUGUUUGAUAACAAUAU-UCA 5'
AT2G28510.1     709     728
DOF zinc finger like protein

sRNA_AGO1_Solexa_Mi2008_3_29723_hit1
5' UCCCCUUACAAUGUCGAG-UAA
   | ||||| |||||||||| |||
```

```
   AUGGGAAGGUUACAGCUCAAUU 5'
AT2G30140.2     1103    1124
putative glucosyltransferase

sRNA_AGO1_Solexa_Mi2008_3_29723_hit1
5' UCCCCUUACAAUGUCGAGU-AA
   | ||||| ||||||||||| ||
   AUGGGAAGGUUACAGCUCAAUU 5'
AT2G30140.1     1106    1127
putative glucosyltransferase

sRNA_AGO1_Solexa_Mi2008_1_53677_hit1
5' UUGUGUAGAGUGUGAU-UUGGU
   ||||| || ||||||| |||||
   AACACUUCACACACUAAAACCA 5'
AT2G30140.2     35      56
putative glucosyltransferase

sRNA_AGO1_Solexa_Mi2008_1_43489_hit1
5' UGGUGGCGUUGUUGGUCUUGAA
   |||||| ||||||| ||||||
   ACCACCACAACAACAAGAACUC 5'
AT2G31070.1     1037    1058
unknown protein

sRNA_AGO1_Solexa_Mi2008_1_51213_hit1
5' UUGAUGUUCUGAUGAG-AUCCU
   ||||||||||||||| ||   |
   AACUACAAGACUACUCCUACUA 5'
AT2G31070.1     1215    1236
unknown protein

sRNA_AGO1_Solexa_Mi2008_2_51261_hit1
5' UUGAUUGAUGUUCAGAUGAUGAU
   ||| ||||||||| ||||| |||
   AAC-AACUACAAGACUACUCCUA 5'
AT2G31070.1     1218    1239
unknown protein

sRNA_AGO1_Solexa_Mi2008_7_3078_hit1
5' AUGAUGAUGAGAAUGAUGAU
   |||||| |||| ||||||||
   UACUACCACUCCUACUACUA 5'
AT2G31070.1     1279    1298
unknown protein

sRNA_AGO1_Solexa_Mi2008_6_14255_hit369
5' GAUGAUGAUGAUGAUGAUGAUGAU
   |||||||||| ||| |||||||||
   CUACUACUACCACUCCUACUACUA 5'
AT2G31070.1     1279    1302
unknown protein

sRNA_AGO1_Solexa_Mi2008_1_3079_hit472
5' AUGAUGAUGAUGAUGAUGAUGA
   ||||||||| ||| ||||||||
   UACUACUACCACUCCUACUACU 5'
AT2G31070.1     1280    1301
unknown protein

sRNA_AGO1_Solexa_Mi2008_1_45039_hit3
5' UG-UGAUGAUGAUGAUGAUGAUGA
   || |||||||| ||| ||||||||
```

```
   ACUACUACUACCACUCCUACUACU 5'
AT2G31070.1     1281    1304
unknown protein

sRNA_AGO1_Solexa_Mi2008_6_14255_hit369
5' GAUGAUGAUGAUGAUGAUGAUGAU
   ||||||||||||| ||| ||||||
   CUACUACUACUACCACUCCUACUA 5'
AT2G31070.1     1282    1305
unknown protein

sRNA_AGO1_Solexa_Mi2008_1_3079_hit472
5' AUGAUGAUGAUGAUGAUGAUGA
   |||||||||||| ||| |||||
   UACUACUACUACCACUCCUACU 5'
AT2G31070.1     1283    1304
unknown protein

sRNA_AGO1_Solexa_Mi2008_7_11649_hit2
5' CUGAUUGAUGAUGAUGGAUCU
   ||||| |||||||||| |||:
   GACUA-CUACUACUAC-UAGG 5'
AT2G31070.1     1449    1467
unknown protein

sRNA_AGO1_Solexa_Mi2008_1_36898_hit1
5' UGAGU-UGGAUGAUGAUGAUGA
   ||| | || |||||||||||||
   ACUAAGAC-UACUACUACUACU 5'
AT2G31070.1     1452    1472
unknown protein

sRNA_AGO1_Solexa_Mi2008_1_14057_hit1
5' GAGGAGAAGCAGAAGCAAGUU
   ||||||||| |||||  ||||
   CUCCUCUUCUUCUUC--UCAA 5'
AT2G31070.1     1591    1609
unknown protein

sRNA_AGO1_Solexa_Mi2008_5_12942_hit1
5' CUUGGGACAGAGUGUG-CUUAC
   ||||| |||||||||| ||| |
   GAACC-UGUCUCACACAGAAGG 5'
AT2G31560.2     95      115
unknown protein

sRNA_AGO1_Solexa_Mi2008_1_40688_hit1
5' UGGAGUCCCAGUCAAGAAUG-UC
   ||| |||||||||||| ||| ||
   ACC-CAGGGUCAGUUCGUACUAG 5'
AT2G31610.1     643     664
40S ribosomal protein; contains C-terminal domain

sRNA_AGO1_Solexa_Mi2008_8_4777_hit1
5' CAAGCUGCCAUGUCAUCAGAG
    ||||| ||||||||||||||
   UUUCGAGGGUACAGUAGUCUC 5'
AT2G31660.1     2038    2058
importin (nuclear transport factor ) like protein

sRNA_AGO1_Solexa_Mi2008_2_37314_hit1
5' UGAUGGAG-GACCUUGCUGAUA
   ||||| || |||||||||||||
```

```
   ACUAC-UCACUGGAACGACUAG 5'
AT2G31660.1     3747    3767
importin (nuclear transport factor ) like protein

sRNA_AGO1_Solexa_Mi2008_7_3090_hit1
5' AUGAUGGAG-GACCUUGCUGAU
    ||||| || |||||||||||
   CACUAC-UCACUGGAACGACUA 5'
AT2G31660.1     3748    3768
importin (nuclear transport factor ) like protein

sRNA_AGO1_Solexa_Mi2008_2_5577_hit1
5' CACGAAGAGCAUGAU-GGGUC
   || ||||||||||| ||||
   GU-CUUCUCGUACUAUCCCAA 5'
AT2G31660.1     74      93
importin (nuclear transport factor ) like protein

sRNA_AGO1_Solexa_Mi2008_1_7415_hit1
5' CAUGUUUGGAUACUUGUUCUG
   ||||||||| |||||| |||
   GUACAAACCAAUGAAC-AGAA 5'
AT2G31890.1     2071    2090
unknown protein

sRNA_AGO1_Solexa_Mi2008_1_25400_hit1
5' UAUGAUGAUGAAGUUGAUGUU
    ||||||||| ||||| ||||
   CUACUACUACAUCAACAACAA 5'
AT2G31945.1     367     387
unknown protein

sRNA_AGO1_Solexa_Mi2008_1_33665_hit5
5' UCUGGUCCUGAUUGAAUCCAA
   ||||||| ||||:|||||||
   AGACCAGAACUAGCUUAGGUA 5'
AT2G32870.1     1274    1294
unknown protein

sRNA_AGO1_Solexa_Mi2008_11_14876_hit4
5' GGAGGUGGUGG-UGG-UGGUGGU
   ||||||||||| ||| ||||| |
   CCUCCACCACCAACCUACCACAA 5'
AT2G33770.1     108     130
ubiquitin-conjugating enzyme E2 -like protein

sRNA_AGO1_Solexa_Mi2008_24_502_hit1
5' AAAGGAGGUGGUGGUUG-AU
   | ||||||||||||||| ||
   UCUCCUCCACCACCAACCUA 5'
AT2G33770.1     114     133
ubiquitin-conjugating enzyme E2 -like protein

sRNA_AGO1_Solexa_Mi2008_1_28360_hit2
5' UCAGCGGCGGAUCC-CACAAUGU
   |||| |||||| || ||||||||
   AGUCACCGCCU-GGCGUGUUACA 5'
AT2G33770.1     1331    1352
ubiquitin-conjugating enzyme E2 -like protein

sRNA_AGO1_Solexa_Mi2008_2_10859_hit1
5' CUCCAUCUCUCUCUCUGCUU
   |||| |||||||||||| ||
```

```
   GAGGAAGAGAGAGAGAC-AA 5'
AT2G33770.1     175     193
ubiquitin-conjugating enzyme E2 -like protein

sRNA_AGO1_Solexa_Mi2008_4_38337_hit1
5' UGCCAAAGGAGAUUUGCCCUGU
   ||||||||:|||||||||||:
   ACGGUUUCUUCUAAACGGGAUU 5'
AT2G33770.1     605     626
ubiquitin-conjugating enzyme E2 -like protein

sRNA_AGO1_Solexa_Mi2008_1041_38329_hit2
5' UGCCAAAGGAGAGUUGCCCUG
   ||||||||:||| |||||||:
   ACGGUUUCUUCUAAACGGGAU 5'
AT2G33770.1     606     626
ubiquitin-conjugating enzyme E2 -like protein

sRNA_AGO1_Solexa_Mi2008_2_38332_hit1
5' UGCCAAAGGAGAUUUGCCCCG
   ||||||||:|||||||||| :
   ACGGUUUCUUCUAAACGGGAU 5'
AT2G33770.1     606     626
ubiquitin-conjugating enzyme E2 -like protein

sRNA_AGO1_Solexa_Mi2008_92_38333_hit1
5' UGCCAAAGGAGAUUUGCCCGG
   ||||||||:|||||||||| :
   ACGGUUUCUUCUAAACGGGAU 5'
AT2G33770.1     606     626
ubiquitin-conjugating enzyme E2 -like protein

sRNA_AGO1_Solexa_Mi2008_101_38336_hit1
5' UGCCAAAGGAGAUUUGCCCUG
   ||||||||:|||||||||||:
   ACGGUUUCUUCUAAACGGGAU 5'
AT2G33770.1     606     626
ubiquitin-conjugating enzyme E2 -like protein

sRNA_AGO1_Solexa_Mi2008_74_51466_hit1
5' UUGCCAAAGGAGAGUUGCCCUG
   |||||||||:||| |||||||:
   AACGGUUUCUUCUAAACGGGAU 5'
AT2G33770.1     606     627
ubiquitin-conjugating enzyme E2 -like protein

sRNA_AGO1_Solexa_Mi2008_7_38328_hit2
5' UGCCAAAGGAGAGUUGCCCU
   ||||||||:||| |||||||
   ACGGUUUCUUCUAAACGGGA 5'
AT2G33770.1     607     626
ubiquitin-conjugating enzyme E2 -like protein

sRNA_AGO1_Solexa_Mi2008_2_38335_hit1
5' UGCCAAAGGAGAUUUGCCCU
   ||||||||:|||||||||||
   ACGGUUUCUUCUAAACGGGA 5'
AT2G33770.1     607     626
ubiquitin-conjugating enzyme E2 -like protein

sRNA_AGO1_Solexa_Mi2008_1_51465_hit1
5' UUGCCAAAGGAGAGUUGCCCU
   |||||||||:||| |||||||
```

```
   AACGGUUUCUUCUAAACGGGA 5'
AT2G33770.1     607     627
ubiquitin-conjugating enzyme E2 -like protein

sRNA_AGO1_Solexa_Mi2008_1_38331_hit4
5' UGCCAAAGGAGAUUUGCCC
   ||||||||:||||||||||
   ACGGUUUCUUCUAAACGGG 5'
AT2G33770.1     608     626
ubiquitin-conjugating enzyme E2 -like protein

sRNA_AGO1_Solexa_Mi2008_4_38337_hit1
5' UGCCAAAGGAGAUUUGCCCUGU
   ||||||||||||| ||||||:
   ACGGUUUCCUCUAUACGGGAUC 5'
AT2G33770.1     738     759
ubiquitin-conjugating enzyme E2 -like protein

sRNA_AGO1_Solexa_Mi2008_2_38332_hit1
5' UGCCAAAGGAGAUUUGCCCCG
   ||||||||||||| ||||| :
   ACGGUUUCCUCUAUACGGGAU 5'
AT2G33770.1     739     759
ubiquitin-conjugating enzyme E2 -like protein

sRNA_AGO1_Solexa_Mi2008_92_38333_hit1
5' UGCCAAAGGAGAUUUGCCCGG
   ||||||||||||| ||||| :
   ACGGUUUCCUCUAUACGGGAU 5'
AT2G33770.1     739     759
ubiquitin-conjugating enzyme E2 -like protein

sRNA_AGO1_Solexa_Mi2008_101_38336_hit1
5' UGCCAAAGGAGAUUUGCCCUG
   ||||||||||||| ||||||:
   ACGGUUUCCUCUAUACGGGAU 5'
AT2G33770.1     739     759
ubiquitin-conjugating enzyme E2 -like protein

sRNA_AGO1_Solexa_Mi2008_2_7575_hit2
5' CCAAAGGAGAGU-UGCCCUG
   |||||||||| | ||||||:
   GGUUUCCUCU-AUACGGGAU 5'
AT2G33770.1     740     758
ubiquitin-conjugating enzyme E2 -like protein

sRNA_AGO1_Solexa_Mi2008_26_14499_hit2
5' GCCAAAGGAGAGU-UGCCCUG
   ||||||||||| | ||||||:
   CGGUUUCCUCU-AUACGGGAU 5'
AT2G33770.1     740     759
ubiquitin-conjugating enzyme E2 -like protein

sRNA_AGO1_Solexa_Mi2008_2_38335_hit1
5' UGCCAAAGGAGAUUUGCCCU
   ||||||||||||| ||||||
   ACGGUUUCCUCUAUACGGGA 5'
AT2G33770.1     740     759
ubiquitin-conjugating enzyme E2 -like protein

sRNA_AGO1_Solexa_Mi2008_1041_38329_hit2
5' UGCCAAAGGAGAGU-UGCCCUG
   |||||||||||| | ||||||:
```

```
   ACGGUUUCCUCU-AUACGGGAU 5'
AT2G33770.1     740     760
ubiquitin-conjugating enzyme E2 -like protein

sRNA_AGO1_Solexa_Mi2008_74_51466_hit1
5' UUGCCAAAGGAGAGU-UGCCCUG
   ||||||||||||| | ||||||:
   AACGGUUUCCUCU-AUACGGGAU 5'
AT2G33770.1     740     761
ubiquitin-conjugating enzyme E2 -like protein

sRNA_AGO1_Solexa_Mi2008_1_38331_hit4
5' UGCCAAAGGAGAUUUGCCC
   ||||||||||||| |||||
   ACGGUUUCCUCUAUACGGG 5'
AT2G33770.1     741     759
ubiquitin-conjugating enzyme E2 -like protein

sRNA_AGO1_Solexa_Mi2008_7_38328_hit2
5' UGCCAAAGGAGAGU-UGCCCU
   |||||||||||| | ||||||
   ACGGUUUCCUCU-AUACGGGA 5'
AT2G33770.1     741     760
ubiquitin-conjugating enzyme E2 -like protein

sRNA_AGO1_Solexa_Mi2008_1_51465_hit1
5' UUGCCAAAGGAGAGU-UGCCCU
   ||||||||||||| | ||||||
   AACGGUUUCCUCU-AUACGGGA 5'
AT2G33770.1     741     761
ubiquitin-conjugating enzyme E2 -like protein

sRNA_AGO1_Solexa_Mi2008_3_38327_hit2
5' UGCCAAAGGAGAGU-UGCCC
   |||||||||||| | |||||
   ACGGUUUCCUCU-AUACGGG 5'
AT2G33770.1     742     760
ubiquitin-conjugating enzyme E2 -like protein

sRNA_AGO1_Solexa_Mi2008_1_26738_hit1
5' UCAAAGGGGAACCCAAGAUGUG
   ||||||||||||||||||||||
   AGUUUCCCCUUGGGUUCUACAC 5'
AT2G33770.1     774     795
ubiquitin-conjugating enzyme E2 -like protein

sRNA_AGO1_Solexa_Mi2008_6_14501_hit1
5' GCCAAAGGAGAUUUGCCCGGU
   ||||||||||||||||||:::
   CGGUUUCCUCUAAACGGGUUG 5'
AT2G33770.1     827     847
ubiquitin-conjugating enzyme E2 -like protein

sRNA_AGO1_Solexa_Mi2008_4_38334_hit1
5' UGCCAAAGGAGAUUUGCCCGGU
   |||||||||||||||||||:::
   ACGGUUUCCUCUAAACGGGUUG 5'
AT2G33770.1     827     848
ubiquitin-conjugating enzyme E2 -like protein

sRNA_AGO1_Solexa_Mi2008_4_38337_hit1
5' UGCCAAAGGAGAUUUGCCCUGU
   |||||||||||||||||||| ::
```

```
   ACGGUUUCCUCUAAACGGGUUG 5'
AT2G33770.1     827     848
ubiquitin-conjugating enzyme E2 -like protein

sRNA_AGO1_Solexa_Mi2008_2_7575_hit2
5' CCAAAGGAGAGUUGCCCUG
   |||||||||| |||||| :
   GGUUUCCUCUAAACGGGUU 5'
AT2G33770.1     828     846
ubiquitin-conjugating enzyme E2 -like protein

sRNA_AGO1_Solexa_Mi2008_26_14499_hit2
5' GCCAAAGGAGAGUUGCCCUG
   ||||||||||| |||||| :
   CGGUUUCCUCUAAACGGGUU 5'
AT2G33770.1     828     847
ubiquitin-conjugating enzyme E2 -like protein

sRNA_AGO1_Solexa_Mi2008_1041_38329_hit2
5' UGCCAAAGGAGAGUUGCCCUG
   |||||||||||| |||||| :
   ACGGUUUCCUCUAAACGGGUU 5'
AT2G33770.1     828     848
ubiquitin-conjugating enzyme E2 -like protein

sRNA_AGO1_Solexa_Mi2008_2_38332_hit1
5' UGCCAAAGGAGAUUUGCCCCG
   ||||||||||||||||||| :
   ACGGUUUCCUCUAAACGGGUU 5'
AT2G33770.1     828     848
ubiquitin-conjugating enzyme E2 -like protein

sRNA_AGO1_Solexa_Mi2008_92_38333_hit1
5' UGCCAAAGGAGAUUUGCCCGG
   |||||||||||||||||||::
   ACGGUUUCCUCUAAACGGGUU 5'
AT2G33770.1     828     848
ubiquitin-conjugating enzyme E2 -like protein

sRNA_AGO1_Solexa_Mi2008_101_38336_hit1
5' UGCCAAAGGAGAUUUGCCCUG
   ||||||||||||||||||| :
   ACGGUUUCCUCUAAACGGGUU 5'
AT2G33770.1     828     848
ubiquitin-conjugating enzyme E2 -like protein

sRNA_AGO1_Solexa_Mi2008_7_38328_hit2
5' UGCCAAAGGAGAGUUGCCCU
   |||||||||||| ||||||
   ACGGUUUCCUCUAAACGGGU 5'
AT2G33770.1     829     848
ubiquitin-conjugating enzyme E2 -like protein

sRNA_AGO1_Solexa_Mi2008_2_38335_hit1
5' UGCCAAAGGAGAUUUGCCCU
   |||||||||||||||||||
   ACGGUUUCCUCUAAACGGGU 5'
AT2G33770.1     829     848
ubiquitin-conjugating enzyme E2 -like protein

sRNA_AGO1_Solexa_Mi2008_1_51465_hit1
5' UUGCCAAAGGAGAGUUGCCCU
    |||||||||||| ||||||
```

```
   UACGGUUUCCUCUAAACGGGU 5'
AT2G33770.1     829     849
ubiquitin-conjugating enzyme E2 -like protein

sRNA_AGO1_Solexa_Mi2008_3_38327_hit2
5' UGCCAAAGGAGAGUUGCCC
   |||||||||||| ||||||
   ACGGUUUCCUCUAAACGGG 5'
AT2G33770.1     830     848
ubiquitin-conjugating enzyme E2 -like protein

sRNA_AGO1_Solexa_Mi2008_1_38331_hit4
5' UGCCAAAGGAGAUUUGCCC
   |||||||||||||||||||
   ACGGUUUCCUCUAAACGGG 5'
AT2G33770.1     830     848
ubiquitin-conjugating enzyme E2 -like protein

sRNA_AGO1_Solexa_Mi2008_2_38335_hit1
5' UGCCAAAGGAGAUUUGCCCU
   |||||||||||||||||| |
   ACGGUUUCCUCUAAACGAGC 5'
AT2G33770.1     886     905
ubiquitin-conjugating enzyme E2 -like protein

sRNA_AGO1_Solexa_Mi2008_3_38327_hit2
5' UGCCAAAGGAGAGUUGCCC
   |||||||||||| |||| |
   ACGGUUUCCUCUAAACGAG 5'
AT2G33770.1     887     905
ubiquitin-conjugating enzyme E2 -like protein

sRNA_AGO1_Solexa_Mi2008_1_38331_hit4
5' UGCCAAAGGAGAUUUGCCC
   ||||||||||||||||| |
   ACGGUUUCCUCUAAACGAG 5'
AT2G33770.1     887     905
ubiquitin-conjugating enzyme E2 -like protein

sRNA_AGO1_Solexa_Mi2008_2_38335_hit1
5' UGCCAAAGGAGAUUUGCCCU
   ||||||||||||||||| ||
   ACGGUUUCCUCUAAACGAGA 5'
AT2G33770.1     943     962
ubiquitin-conjugating enzyme E2 -like protein

sRNA_AGO1_Solexa_Mi2008_3_38327_hit2
5' UGCCAAAGGAGAGUUGCCC
   |||||||||||| |||| |
   ACGGUUUCCUCUAAACGAG 5'
AT2G33770.1     944     962
ubiquitin-conjugating enzyme E2 -like protein

sRNA_AGO1_Solexa_Mi2008_1_38331_hit4
5' UGCCAAAGGAGAUUUGCCC
   ||||||||||||||||| |
   ACGGUUUCCUCUAAACGAG 5'
AT2G33770.1     944     962
ubiquitin-conjugating enzyme E2 -like protein

sRNA_AGO1_Solexa_Mi2008_1_39748_hit1
5' UGGAAGCAGAUGGUUCGUU
   ||||| ||||||||||| |
```

```
   ACCUUGGUCUACCAAGCUA 5'
AT2G33860.1     1523    1541
auxin response transcription factor 3 (ETTIN/ARF3)

sRNA_AGO1_Solexa_Mi2008_1_35469_hit1
5' UGACCUUGUAAGACCCCAUCU
   ||||||||:||||| || |||
   ACUGGAACGUUCUG-GG-AGA 5'
AT2G33860.1     1672    1690
auxin response transcription factor 3 (ETTIN/ARF3)

sRNA_AGO1_Solexa_Mi2008_7_33918_hit1
5' UCUUGACCUUGUAAGACCCCA
   |||||||||||:|||||||
   AGAACUGGAACGUUCUGGGAG 5'
AT2G33860.1     1672    1692
auxin response transcription factor 3 (ETTIN/ARF3)

sRNA_AGO1_Solexa_Mi2008_7_49853_hit1
5' UUCUUGACCUUGUAAGACCCCA
   ||||||||||||:|||||||
   AAGAACUGGAACGUUCUGGGAG 5'
AT2G33860.1     1672    1693
auxin response transcription factor 3 (ETTIN/ARF3)

sRNA_AGO1_Solexa_Mi2008_7_12746_hit1
5' CUUGACCUUGUAAGACCCC
   ||||||||||:|||||||
   GAACUGGAACGUUCUGGGA 5'
AT2G33860.1     1673    1691
auxin response transcription factor 3 (ETTIN/ARF3)

sRNA_AGO1_Solexa_Mi2008_7_49852_hit1
5' UUCUUGACCUUGUAAGACCCC
   ||||||||||||:|||||||
   AAGAACUGGAACGUUCUGGGA 5'
AT2G33860.1     1673    1693
auxin response transcription factor 3 (ETTIN/ARF3)

sRNA_AGO1_Solexa_Mi2008_4_55012_hit1
5' UUUCUUGACCUUGUAAGACCCC
   |||||||||||||:|||||||
   AAAGAACUGGAACGUUCUGGGA 5'
AT2G33860.1     1673    1694
auxin response transcription factor 3 (ETTIN/ARF3)

sRNA_AGO1_Solexa_Mi2008_1_55011_hit1
5' UUUCUUGACCUUGUAAGACCC
   |||||||||||||:|||||||
   AAAGAACUGGAACGUUCUGGG 5'
AT2G33860.1     1674    1694
auxin response transcription factor 3 (ETTIN/ARF3)

sRNA_AGO1_Solexa_Mi2008_1_3_hit25
5' AAAAAAAAAAAA--AGAAAGA
   ||||||||||||  |||||:|
   UUUUUUUUUUUUGAUCUUUUU 5'
AT2G33860.1     173     193
auxin response transcription factor 3 (ETTIN/ARF3)

sRNA_AGO1_Solexa_Mi2008_1_2_hit38
5' AAA-AAAAAAAAAAAAACCAU
   ||| ||||||||||||| |
```

```
   UUUCUUUUUUUUUUUUGAUC 5'
AT2G33860.1     178     197
auxin response transcription factor 3 (ETTIN/ARF3)

sRNA_AGO1_Solexa_Mi2008_7_12746_hit1
5' CUUGACCUUGUAAGACCCC
   ||||||||||:||||||
   GAACUGGAACGUUCUGGAA 5'
AT2G33860.1     1793    1811
auxin response transcription factor 3 (ETTIN/ARF3)

sRNA_AGO1_Solexa_Mi2008_7_49852_hit1
5' UUCUUGACCUUGUAAGACCCC
   ||||||||||||:||||||
   AAGAACUGGAACGUUCUGGAA 5'
AT2G33860.1     1793    1813
auxin response transcription factor 3 (ETTIN/ARF3)

sRNA_AGO1_Solexa_Mi2008_4_55012_hit1
5' UUUCUUGACCUUGUAAGACCCC
   |||||||||||||:||||||
   AAAGAACUGGAACGUUCUGGAA 5'
AT2G33860.1     1793    1814
auxin response transcription factor 3 (ETTIN/ARF3)

sRNA_AGO1_Solexa_Mi2008_1_55011_hit1
5' UUUCUUGACCUUGUAAGACCC
   |||||||||||||:||||||
   AAAGAACUGGAACGUUCUGGA 5'
AT2G33860.1     1794    1814
auxin response transcription factor 3 (ETTIN/ARF3)

sRNA_AGO1_Solexa_Mi2008_1_42435_hit1
5' UGGGGGGAGGAUACGUGUACU
   |||||||||||||||||||||
   ACCCCCCUCCUAUGCACAUGA 5'
AT2G33860.1     1901    1921
auxin response transcription factor 3 (ETTIN/ARF3)

sRNA_AGO1_Solexa_Mi2008_2_52_hit1
5' AAAAA-AGAGAGAUACAGAGAU
   ||||| ||||||| ||||||||
   UUUUUCUCUCUCUUUGUCUCUA 5'
AT2G33860.1     345     366
auxin response transcription factor 3 (ETTIN/ARF3)

sRNA_AGO1_Solexa_Mi2008_1_43638_hit1
5' UGGUGUCAGAUGUGUGUG-UGU
   ||||| | |||||||||| |||
   ACCACUGACUACACACACCACA 5'
AT2G33860.1     39      60
auxin response transcription factor 3 (ETTIN/ARF3)

sRNA_AGO1_Solexa_Mi2008_1_146_hit4
5' AAA-ACAAAAGAGGAAGAAGCU
   ||| | |||| |||||||||||
   UUUAUCUUUUAUCCUUCUUCGA 5'
AT2G37180.1     1001    1022
aquaporin (plasma membrane intrinsic protein 2C)

sRNA_AGO1_Solexa_Mi2008_1_44212_hit1
5' UGUAGGCAAGCACGAGAAUGU
   |||||:||||||||| | |||
```

```
   ACAUCUGUUCGUGCU-U-ACA 5'
AT2G37180.1     597     615
aquaporin (plasma membrane intrinsic protein 2C)

sRNA_AGO1_Solexa_Mi2008_2_7000_hit28
5' CAUCAGGUACUUCCAGCGUUCU
   |||||||| |||||| || |||
   GUAGUCCAGGAAGGU-GC-AGA 5'
AT2G37180.1     80      99
aquaporin (plasma membrane intrinsic protein 2C)

sRNA_AGO1_Solexa_Mi2008_1_29116_hit2
5' UCAUUGCAAUUGUUGGUCUUCA
   || ||||||||||| ||||||
   AG-AACGUUAACAA-CAGAAGC 5'
AT2G38790.1     164     183
unknown protein

sRNA_AGO1_Solexa_Mi2008_7_29115_hit2
5' UCAUUGCAAUUGUUGGUCUUC
   || ||||||||||| ||||||
   AG-AACGUUAACAA-CAGAAG 5'
AT2G38790.1     165     183
unknown protein

sRNA_AGO1_Solexa_Mi2008_2_2818_hit2
5' AUCAUUGCAAUUGUUGGUCUU
   ||| ||||||||||| |||||
   UAG-AACGUUAACAA-CAGAA 5'
AT2G38790.1     166     184
unknown protein

sRNA_AGO1_Solexa_Mi2008_1_29114_hit2
5' UCAUUGCAAUUGUUGGUCU
   || ||||||||||| ||||
   AG-AACGUUAACAA-CAGA 5'
AT2G38790.1     167     183
unknown protein

sRNA_AGO1_Solexa_Mi2008_1_12211_hit1
5' CUGU-CUAUGCAAUUGUUGUG
   |||| || ||||||||||||
   GACAAGA-ACGUUAACAACAG 5'
AT2G38790.1     168     187
unknown protein

sRNA_AGO1_Solexa_Mi2008_2_6944_hit1
5' CAUAUACUCGAAUACCUAU
   ||||||||| |||||| ||
   GUAUAUGAGAUUAUGG-UA 5'
AT2G38790.1     375     392
unknown protein

sRNA_AGO1_Solexa_Mi2008_2_33689_hit1
5' UCUGGUGGAUUACGGAUUGUG
   |||||||||||| |  |||||
   AGACCACCUAAU-CG-AACAC 5'
AT2G39030.1     148     166
unknown protein

sRNA_AGO1_Solexa_Mi2008_3_40000_hit2
5' UGGACCCGGUCGAUGAAGUCU
   ||| || ||||| ||||||||
```

```
   ACC-GG-CCAGCCACUUCAGA 5'
AT2G39030.1     77      95
unknown protein

sRNA_AGO1_Solexa_Mi2008_1_17143_hit1
5' UAACAAAUGAUGUGGCACGUG
   |||||||||| ||||| ||||
   AUUGUUUACUUCACCGCGCAC 5'
AT2G39800.1     117     137
delta-1-pyrroline 5-carboxylase synthetase (P5C1)

sRNA_AGO1_Solexa_Mi2008_1_2355_hit1
5' AUAACAAAUGACGUGGCACGUG
    |||||||||| ||||| ||||
   GAUUGUUUACUUCACCGCGCAC 5'
AT2G39800.1     117     138
delta-1-pyrroline 5-carboxylase synthetase (P5C1)

sRNA_AGO1_Solexa_Mi2008_8_48955_hit2
5' UUCCAAUAAUGAGUAGUGGUAA
   ||| ||||||:||||||:||||
   AAG-UUAUUAUUCAUCAUCAUU 5'
AT2G39800.2     2287    2307
delta-1-pyrroline 5-carboxylase synthetase (P5C1)

sRNA_AGO1_Solexa_Mi2008_8_48955_hit2
5' UUCCAAUAAUGAGUAGUGGUAA
   ||| ||||||:||||||:||||
   AAG-UUAUUAUUCAUCAUCAUU 5'
AT2G39800.1     2651    2671
delta-1-pyrroline 5-carboxylase synthetase (P5C1)

sRNA_AGO1_Solexa_Mi2008_2_380_hit1
5' AAAGAGAGAGAUGAUGCCAU
    || |||||||||||| |||
   GUU-UCUCUCUACUACCGUA 5'
AT2G40940.1     1322    1340
ethylene response sensor (ERS)

sRNA_AGO1_Solexa_Mi2008_1_38224_hit4
5' UGCAUCAUUGGUGGUGUGAG
   ||| | ||||||||||||||
   ACG-A-UAACCACCACACUC 5'
AT2G40940.1     174     191
ethylene response sensor (ERS)

sRNA_AGO1_Solexa_Mi2008_1_5010_hit1
5' CAAGUGCAUUAAGAACAUC-AU
   ||| ||||| ||||||||| ||
   GUU-ACGUACUUCUUGUAGGUA 5'
AT2G40940.1     462     482
ethylene response sensor (ERS)

sRNA_AGO1_Solexa_Mi2008_1_4591_hit1
5' CAAGACAAGGAGUUUUACC
   ||||||||:||||||||||
   GUUCUGUUUCUCAAAAUGG 5'
AT2G40940.1     753     771
ethylene response sensor (ERS)

sRNA_AGO1_Solexa_Mi2008_1_3647_hit1
5' AUUGAAC-GAGAUGAUGAUU-GG
   ||||| | |||||||||||| ||
```

```
   UAACUAGGCUCUACUACUAAUCC 5'
AT2G40970.1     304     326
unknown protein

sRNA_AGO1_Solexa_Mi2008_1_35931_hit1
5' UGAGAGUG-AGGAGAUGAUGCGU
   |||||| | ||||:|||||||||
   ACUCUC-CAUCCUUUACUACGCA 5'
AT2G41190.1     1126    1147
unknown protein

sRNA_AGO1_Solexa_Mi2008_1_20735_hit4
5' UACUUUUUCG-GGCAUUUUUGUG
   |||||||||| || |||| ||||
   AUGAAAAAGCUCC-UAAACACAC 5'
AT2G41190.1     27      48
unknown protein

sRNA_AGO1_Solexa_Mi2008_6_53710_hit1
5' UUGUGU-UCAGGAUGAUCCAGU
   ||| || ||||||| |||||||
   AAC-CACAGUCCUAGUAGGUCA 5'
AT2G42790.1     1408    1428
putative citrate synthase

sRNA_AGO1_Solexa_Mi2008_1_56383_hit1
5' UUU-UCCCACAAACUUGAUCGCU
   ||| |||| |||||||||||| |
   AAAUAGGGGGUUUGAACUAGCCA 5'
AT2G42790.1     2263    2285
putative citrate synthase

sRNA_AGO1_Solexa_Mi2008_4_56382_hit1
5' UUU-UCCCACAAACUUGAUCGC
   ||| |||| ||||||||||||
   AAAUAGGGGGUUUGAACUAGCC 5'
AT2G42790.1     2264    2285
putative citrate synthase

sRNA_AGO1_Solexa_Mi2008_1_189_hit14
5' AAAAGAGGAGAAGA-CAAAGUGU
    ||||||||||||| |||| |||
   GUUUCUCCUCUUCUCGUUU-ACA 5'
AT2G42890.1     154     175
MEI2-like protein (MEI2)

sRNA_AGO1_Solexa_Mi2008_3_21212_hit1
5' UAGAGAGAGAAAAAGAUGGC
   :||||||||||||| ||||
   GUCUCUCUCUUUUU-UACCU 5'
AT2G42890.1     25      43
MEI2-like protein (MEI2)

sRNA_AGO1_Solexa_Mi2008_3_24633_hit1
5' UAUAG-UUUCAGGAGUUGUUGGA
   ||||| |||  |||||||||||
   AUAUCGAAAAACCUCAACAACCU 5'
AT2G44460.1     1386    1408
putative beta-glucosidase

sRNA_AGO1_Solexa_Mi2008_1_1776_hit10
5' AGAAAAAAAAAAAAAAA-A-AAU
   ||||| ||||||||||| | |||
```

```
   UCUUUCUUUUUUUUUUUCUGUUA 5'
AT2G45210.1     109     131
putative auxin-regulated protein

sRNA_AGO1_Solexa_Mi2008_6_733_hit1
5' AACUGUUCAUUAAGUAGAA
   || |||||||||||| ||
   UU-ACAAGUAAUUCA-CUA 5'
AT2G46680.2     811     827
homeodomain transcription factor  (ATHB-7)

sRNA_AGO1_Solexa_Mi2008_6_733_hit1
5' AACUGUUCAUUAAGUAGAA
   || |||||||||||| ||
   UU-ACAAGUAAUUCA-CUA 5'
AT2G46680.1     817     833
homeodomain transcription factor  (ATHB-7)

sRNA_AGO1_Solexa_Mi2008_1_37959_hit1
5' UGCACUGCCUCUUCCCUGGCUC
   ||||||||||||||||||||||
   ACGUGACGGAGAAGGGACCGAG 5'
AT2G47020.2     1487    1508
peptide chain release factor  like protein

sRNA_AGO1_Solexa_Mi2008_11_37958_hit1
5' UGCACUGCCUCUUCCCUGGCU
   |||||||||||||||||||||
   ACGUGACGGAGAAGGGACCGA 5'
AT2G47020.2     1488    1508
peptide chain release factor  like protein

sRNA_AGO1_Solexa_Mi2008_3_37957_hit1
5' UGCACUGCCUCUUCCCUGGC
   ||||||||||||||||||||
   ACGUGACGGAGAAGGGACCG 5'
AT2G47020.2     1489    1508
peptide chain release factor  like protein

sRNA_AGO1_Solexa_Mi2008_6_3128_hit1
5' AUGCACUGCCUCUUCCCUGGC
   |||||||||||||||||||||
   UACGUGACGGAGAAGGGACCG 5'
AT2G47020.2     1489    1509
peptide chain release factor  like protein

sRNA_AGO1_Solexa_Mi2008_1_6534_hit1
5' CAGGGAACAAGCAGAGCAUGG
   |||||||||||||||||||||
   GUCCCUUGUUCGUCUCGUACC 5'
AT2G47020.2     1555    1575
peptide chain release factor  like protein

sRNA_AGO1_Solexa_Mi2008_3_2164_hit1
5' AGGGAACAAGCAGAGCAUG
   |||||||||||||||||||
   UCCCUUGUUCGUCUCGUAC 5'
AT2G47020.2     1556    1574
peptide chain release factor  like protein

sRNA_AGO1_Solexa_Mi2008_18_6533_hit1
5' CAGGGAACAAGCAGAGCAUG
   ||||||||||||||||||||
```

```
   GUCCCUUGUUCGUCUCGUAC 5'
AT2G47020.2     1556    1575
peptide chain release factor  like protein

sRNA_AGO1_Solexa_Mi2008_175_1428_hit1
5' ACAGGGAACAAGCAGAGCAUG
   |||||||||||||||||||||
   UGUCCCUUGUUCGUCUCGUAC 5'
AT2G47020.2     1556    1576
peptide chain release factor  like protein

sRNA_AGO1_Solexa_Mi2008_7_12617_hit1
5' CU-UCGAAGUUUGAGCUUG-AGA
   || || ||||||||||||| |||
   GAGAGGUUCAAACUCGAACAUCU 5'
AT2G47170.1     269     291
ADP-ribosylation factor 1

sRNA_AGO1_Solexa_Mi2008_1_16455_hit1
5' UAAAAUGAUCUGCGUUUGAGA
   |||||||||||||||||||||
   AUUUUACUAGACGCAAACUCU 5'
AT2G47170.1     824     844
ADP-ribosylation factor 1

sRNA_AGO1_Solexa_Mi2008_1_230_hit1
5' AAAAGGGGAAGUACAAAGUA
     |||||||||||||||||
   ACUUCCCCUUCAUGUUUCAC 5'
AT2G47170.1     845     864
ADP-ribosylation factor 1

sRNA_AGO1_Solexa_Mi2008_5_4043_hit2
5' CAAACUGCC-AUGGUCGUCCAA
    |||| ||| ||||||||||||
   UUUUG-CGGCUACCAGCAGGUU 5'
AT2G47170.1     93      113
ADP-ribosylation factor 1

sRNA_AGO1_Solexa_Mi2008_1_13420_hit2
5' GAAGAAGAAGAAGACUCUU
   |||||||||||||| | ||
   CUUCUUCUUCUUCU-A-AA 5'
AT2G47730.1     125     141
glutathione S-transferase (GST6)

sRNA_AGO1_Solexa_Mi2008_6_44397_hit1
5' UGUAUGG-GCCACAUAGGUGGG
   |||| || |||||| |||||||
   ACAU-CCUCGGUGUCUCCACCC 5'
AT2G47770.1     489     509
unknown protein

sRNA_AGO1_Solexa_Mi2008_1_3384_hit1
5' AUGGUGAAGAUGU-UUC-UAGU
   ||||||||||||| ||| || |
   UACCACUUCUACAGAAGCAUGA 5'
AT3G01420.1     291     312
feebly like protein

sRNA_AGO1_Solexa_Mi2008_1_51959_hit1
5' UUGGAAGAACGGGAUUGAGA
   ||||| || |||||||||||
```

```
   AACCUGCU-GCCCUAACUCU 5'
AT3G01470.1     438     456
homeobox protein (HAT5)

sRNA_AGO1_Solexa_Mi2008_1_30071_hit1
5' UCCGUCUGCGGUGGUCCGAUU
   |||||||||||||||||||||
   AGGCAGACGCCACCAGGCUAA 5'
AT3G03450.1     1086    1106
RGA1-like protein

sRNA_AGO1_Solexa_Mi2008_1_55233_hit1
5' UUUGAGUAUUCGGGUCGGGUAUU
   ||| || |||||||||||| ||
   AAAGUC-UAAGCCCAGCCCA-AA 5'
AT3G03450.1     1233    1253
RGA1-like protein

sRNA_AGO1_Solexa_Mi2008_1_48488_hit1
5' UUCAACUGCGGUAACAUCAGAU
   :|||||||||||||| || |||
   GAGUUGACGCCAUUGAAGACUA 5'
AT3G04070.2     932     953
NAM-like protein (no apical meristem)

sRNA_AGO1_Solexa_Mi2008_1_48488_hit1
5' UUCAACUGCGGUAACAUCAGAU
   :|||||||||||||| || |||
   GAGUUGACGCCAUUGAAGACUA 5'
AT3G04070.1     980     1001
NAM-like protein (no apical meristem)

sRNA_AGO1_Solexa_Mi2008_1_4534_hit3
5' CAAGA-A-AAAAAGAGAUGAG
   ||||| | ||||||||||| |
   GUUCUCUAUUUUUCUCUACAC 5'
AT3G04520.1     14      34
L-allo-threonine aldolase like protein

sRNA_AGO1_Solexa_Mi2008_2_6737_hit1
5' CAG-UGGUGCUUCGGGUUACUC
   ||| || |||||||||| ||||
   GUCUAC-ACGAAGCCCACUGAG 5'
AT3G04520.1     1       21
L-allo-threonine aldolase like protein

sRNA_AGO1_Solexa_Mi2008_2_44800_hit1
5' UGUCUACGU-UGAUGAUGUUGU
   ||||||||| | |||||||||
   ACAGAUGCAUA-UACUACAACC 5'
AT3G04520.1     965     985
L-allo-threonine aldolase like protein

sRNA_AGO1_Solexa_Mi2008_4_11415_hit2
5' CUGAAUUCGUUCAUACUUACA
   ||||||| |||| |||||||
   GACUUAACCAAGAAUGAAUGG 5'
AT3G05155.1     1265    1285
putative protein

sRNA_AGO1_Solexa_Mi2008_85_22179_hit1
5' UAGCCAAGGAUGACUUGCCUGU
    ||||||:||||| ||||||||
```

```
   CUCGGUUUCUACUAAACGGACA 5'
AT3G05690.1     1181    1202
putative transcription factor

sRNA_AGO1_Solexa_Mi2008_400_22176_hit7
5' UAGCCAAGGAUGACUUGCCUG
    ||||||:||||| |||||||
   CUCGGUUUCUACUAAACGGAC 5'
AT3G05690.1     1182    1202
putative transcription factor

sRNA_AGO1_Solexa_Mi2008_30_36087_hit4
5' UGAGCCAAGGAUGACUUGCCG
   ||||||||:||||| |||||
   ACUCGGUUUCUACUAAACGGA 5'
AT3G05690.1     1183    1203
putative transcription factor

sRNA_AGO1_Solexa_Mi2008_1_1014_hit1
5' AAU-AGCCAAGGAUGACUUGCCUG
   ||| ||||||:||||| |||||||
   UUACUCGGUUUCUACUAAACGGAC 5'
AT3G05690.1     1183    1206
putative transcription factor

sRNA_AGO1_Solexa_Mi2008_4_36086_hit4
5' UGAGCCAAGGAUGACUUGCC
   ||||||||:||||| |||||
   ACUCGGUUUCUACUAAACGG 5'
AT3G05690.1     1184    1203
putative transcription factor

sRNA_AGO1_Solexa_Mi2008_1_50390_hit1
5' UUGACGGAUACGGAUACGGAU
      |||||||||||||||||
   CCAUGCCUAUGCCUAUGCCUA 5'
AT3G06130.1     1597    1617
unknown protein

sRNA_AGO1_Solexa_Mi2008_8_6832_hit1
5' CAUAAGGUGGAGGAUAUGGAU
   |||||||||||||||||||||
   GUAUUCCACCUCCUAUACCUA 5'
AT3G06130.1     1621    1641
unknown protein

sRNA_AGO1_Solexa_Mi2008_1_341_hit1
5' AAAGAAACAGA-GAGGAAGAU
   ||||||||||| ||||||||
   UUUCUUUGUCUCCUCCUUCUU 5'
AT3G06130.1     162     182
unknown protein

sRNA_AGO1_Solexa_Mi2008_1_26337_hit1
5' UAU-U-GCAGCUUGAUGUGUUCU
   ||| | ||||||||||||||||
   AUAUAGCGUCGAACUACACAAGA 5'
AT3G06130.1     1676    1698
unknown protein

sRNA_AGO1_Solexa_Mi2008_1_40229_hit3
5' UGGAGACAAGUGAUGAUGAUGAU
   |||||||||||| |||| ||||
```

```
   ACCUCUGUUCACAACUA-UACUC 5'
AT3G06390.1     373     394
unknown protein

sRNA_AGO1_Solexa_Mi2008_1_10771_hit1
5' CUCAUAUGGAGAGUGUUGACC
   ||| | |||||||||:|||||
   GAG-A-ACCUCUCACGACUGG 5'
AT3G06390.1     647     665
unknown protein

sRNA_AGO1_Solexa_Mi2008_1_23335_hit2
5' UAGGUAAUGAUUGAGGAGGCC
   |||| ||||| ||||||||||
   AUCC-UUACUUACUCCUCCGG 5'
AT3G08720.1     1655    1674
putative ribosomal-protein S6 kinase (ATPK19)

sRNA_AGO1_Solexa_Mi2008_1_23335_hit2
5' UAGGUAAUGAUUGAGGAGGCC
   |||| ||||| ||||||||||
   AUCC-UUACUUACUCCUCCGG 5'
AT3G08720.2     1843    1862
putative ribosomal-protein S6 kinase (ATPK19)

sRNA_AGO1_Solexa_Mi2008_3_53977_hit1
5' UUUAAGGGUGCGGUGUAG-AGU
   |||||||||| | ||||| |||
   AAAUUCCCACACAACAUCUUCA 5'
AT3G08860.1     304     325
aminotransferase like protein

sRNA_AGO1_Solexa_Mi2008_1_2_hit38
5' AAAAA-AAAAAAAAAACCAU
   ||||| ||||||||||| |
   UUUUUCUUUUUUUUUUGAUC 5'
AT3G09440.1     2114    2133
heat-shock protein (At-hsc70-3)

sRNA_AGO1_Solexa_Mi2008_1_1776_hit10
5' AGAAA-AAAAA-AAAAAAAAAAU
   ||||| ||||| ||||||||||:
   UCUUUCUUUUUCUUUUUUUUUUG 5'
AT3G09440.1     2117    2139
heat-shock protein (At-hsc70-3)

sRNA_AGO1_Solexa_Mi2008_1_45820_hit1
5' UGUUAAUGAUGGAACUGUGCGA
   |||| ||||||| |||||| ||
   ACAACUACUACCGUGACAC-CU 5'
AT3G09810.1     1075    1095
putative dehydrogenase

sRNA_AGO1_Solexa_Mi2008_1_40348_hit1
5' UGGAGAUGGUCUAAGGAUGAG
   :|||| |||| ||||||||||
   GCCUCCACCACAUUCCUACUC 5'
AT3G10040.1     299     319
unknown protein

sRNA_AGO1_Solexa_Mi2008_1_2_hit38
5' AAAAAAAAAAAAAAACCAU
   ||||||||||||||| ||
```

```
   UUUUUUUUUUUUUUUGUUA 5'
AT3G10300.4     1119    1137
unknown protein

sRNA_AGO1_Solexa_Mi2008_1_2_hit38
5' AAAAAAAAAAAAAAACCAU
   ||||||||||||||| ||
   UUUUUUUUUUUUUUUGUUA 5'
AT3G10300.3     1126    1144
unknown protein

sRNA_AGO1_Solexa_Mi2008_1_2_hit38
5' AAAAAAAAAAAAAAACCAU
   ||||||||||||||| ||
   UUUUUUUUUUUUUUUGUUA 5'
AT3G10300.2     1339    1357
unknown protein

sRNA_AGO1_Solexa_Mi2008_17_7551_hit1
5' CCAAAAAAGGAAAGACGCU-GAU
   |||||||||||||||| || ||
   GGUUUUUUCCUUUCUG-GAUCUU 5'
AT3G10300.1     799     820
unknown protein

sRNA_AGO1_Solexa_Mi2008_1_11370_hit1
5' CUGAAGCCGAGUGAUG-UGAC
    ||||| ||||||||| ||||
   UACUUCUGCUCACUACCACUG 5'
AT3G10500.1     42      62
unknown protein

sRNA_AGO1_Solexa_Mi2008_1_3_hit25
5' AAAA-AA-AAAAAAAGAAAGA
   |||| || ||||||||||||
   UUUUCUUGUUUUUUUCUUUCU 5'
AT3G10930.1     406     426
unknown protein

sRNA_AGO1_Solexa_Mi2008_1_3188_hit1
5' AU-GCUCUAAGUCUUGAUCGU
   || | ||||||||||||||:|
   UAUC-AGAUUCAGAACUAGUA 5'
AT3G10930.1     544     563
unknown protein

sRNA_AGO1_Solexa_Mi2008_38_14830_hit2
5' GGACUGAAGGGAGCUCCCU
   ||| |||||||||||||
   CCUUACUUCCCUCGAGGUC 5'
AT3G11440.1     1153    1171
transcription factor like protein (MYB65)

sRNA_AGO1_Solexa_Mi2008_19_40112_hit2
5' UGGACUGAAGGGAGCUCCCU
   |||| |||||||||||||
   ACCUUACUUCCCUCGAGGUC 5'
AT3G11440.1     1153    1172
transcription factor like protein (MYB65)

sRNA_AGO1_Solexa_Mi2008_705_52065_hit2
5' UUGGACUGAAGGGAGCUCCCU
   ||||| |||||||||||||
```

```
   AACCUUACUUCCCUCGAGGUC 5'
AT3G11440.1     1153    1173
transcription factor like protein (MYB65)

sRNA_AGO1_Solexa_Mi2008_16_52069_hit1
5' UUGGACUGAAGGGAGCUCCUU
   ||||| |||||||||||||
   AACCUUACUUCCCUCGAGGUC 5'
AT3G11440.1     1153    1173
transcription factor like protein (MYB65)

sRNA_AGO1_Solexa_Mi2008_7_3717_hit1
5' AUUGGACUGAAGGGAGCUCCCU
   |||||| ||||||||||||
   UAACCUUACUUCCCUCGAGGUC 5'
AT3G11440.1     1153    1174
transcription factor like protein (MYB65)

sRNA_AGO1_Solexa_Mi2008_3349_41018_hit1
5' UGGAUUGAAGGGAGCUCUA
   |||| ||||||||||||:|
   ACCUUACUUCCCUCGAGGU 5'
AT3G11440.1     1154    1172
transcription factor like protein (MYB65)

sRNA_AGO1_Solexa_Mi2008_1621_41020_hit1
5' UGGAUUGAAGGGAGCUCUU
   |||| ||||||||||||:
   ACCUUACUUCCCUCGAGGU 5'
AT3G11440.1     1154    1172
transcription factor like protein (MYB65)

sRNA_AGO1_Solexa_Mi2008_24_52064_hit2
5' UUGGACUGAAGGGAGCUCCC
   ||||| |||||||||||||
   AACCUUACUUCCCUCGAGGU 5'
AT3G11440.1     1154    1173
transcription factor like protein (MYB65)

sRNA_AGO1_Solexa_Mi2008_7_52068_hit1
5' UUGGACUGAAGGGAGCUCCU
   ||||| |||||||||||||
   AACCUUACUUCCCUCGAGGU 5'
AT3G11440.1     1154    1173
transcription factor like protein (MYB65)

sRNA_AGO1_Solexa_Mi2008_1_52243_hit1
5' UUGGAUUGAAGGGAGCUCCU
   ||||| |||||||||||||
   AACCUUACUUCCCUCGAGGU 5'
AT3G11440.1     1154    1173
transcription factor like protein (MYB65)

sRNA_AGO1_Solexa_Mi2008_3416_52245_hit1
5' UUGGAUUGAAGGGAGCUCUA
   ||||| ||||||||||||:|
   AACCUUACUUCCCUCGAGGU 5'
AT3G11440.1     1154    1173
transcription factor like protein (MYB65)

sRNA_AGO1_Solexa_Mi2008_870_52246_hit1
5' UUGGAUUGAAGGGAGCUCUU
   ||||| ||||||||||||:
```

```
   AACCUUACUUCCCUCGAGGU 5'
AT3G11440.1     1154    1173
transcription factor like protein (MYB65)

sRNA_AGO1_Solexa_Mi2008_1_3716_hit1
5' AUUGGACUGAAGGGAGCUCCC
   |||||| |||||||||||||
   UAACCUUACUUCCCUCGAGGU 5'
AT3G11440.1     1154    1174
transcription factor like protein (MYB65)

sRNA_AGO1_Solexa_Mi2008_21_12906_hit1
5' CUUGGACUGAAGGGAGCUCCC
    ||||| |||||||||||||
   UAACCUUACUUCCCUCGAGGU 5'
AT3G11440.1     1154    1174
transcription factor like protein (MYB65)

sRNA_AGO1_Solexa_Mi2008_61_55550_hit1
5' UUUGGACUGAAGGGAGCUCCU
    ||||| |||||||||||||
   UAACCUUACUUCCCUCGAGGU 5'
AT3G11440.1     1154    1174
transcription factor like protein (MYB65)

sRNA_AGO1_Solexa_Mi2008_14_55632_hit1
5' UUUGGAUUGAAGGGAGCUCCU
    ||||| |||||||||||||
   UAACCUUACUUCCCUCGAGGU 5'
AT3G11440.1     1154    1174
transcription factor like protein (MYB65)

sRNA_AGO1_Solexa_Mi2008_15_52063_hit3
5' UUGGACUGAAGGGAGCUCC
   ||||| |||||||||||||
   AACCUUACUUCCCUCGAGG 5'
AT3G11440.1     1155    1173
transcription factor like protein (MYB65)

sRNA_AGO1_Solexa_Mi2008_52_52244_hit2
5' UUGGAUUGAAGGGAGCUCU
   ||||| ||||||||||||:
   AACCUUACUUCCCUCGAGG 5'
AT3G11440.1     1155    1173
transcription factor like protein (MYB65)

sRNA_AGO1_Solexa_Mi2008_9_55631_hit1
5' UUUGGAUUGAAGGGAGCUCC
    ||||| |||||||||||||
   UAACCUUACUUCCCUCGAGG 5'
AT3G11440.1     1155    1174
transcription factor like protein (MYB65)

sRNA_AGO1_Solexa_Mi2008_1201_55633_hit2
5' UUUGGAUUGAAGGGAGCUCU
    ||||| ||||||||||||:
   UAACCUUACUUCCCUCGAGG 5'
AT3G11440.1     1155    1174
transcription factor like protein (MYB65)

sRNA_AGO1_Solexa_Mi2008_136_14386_hit1
5' GAUUGGACUGAAGGGAGCUCC
    |||||| |||||||||||||
```

```
   AUAACCUUACUUCCCUCGAGG 5'
AT3G11440.1     1155    1175
transcription factor like protein (MYB65)

sRNA_AGO1_Solexa_Mi2008_3_55549_hit1
5' UUUGGACUGAAGGGAGCUC
    ||||| ||||||||||||
   UAACCUUACUUCCCUCGAG 5'
AT3G11440.1     1156    1174
transcription factor like protein (MYB65)

sRNA_AGO1_Solexa_Mi2008_907_55630_hit3
5' UUUGGAUUGAAGGGAGCUC
    ||||| ||||||||||||
   UAACCUUACUUCCCUCGAG 5'
AT3G11440.1     1156    1174
transcription factor like protein (MYB65)

sRNA_AGO1_Solexa_Mi2008_4_13220_hit2
5' CUUUGGAUUGAAGGGAGCUC
     ||||| ||||||||||||
   AUAACCUUACUUCCCUCGAG 5'
AT3G11440.1     1156    1175
transcription factor like protein (MYB65)

sRNA_AGO1_Solexa_Mi2008_1_14385_hit1
5' GAUUGGACUGAAGGGAGCUC
    |||||| ||||||||||||
   AUAACCUUACUUCCCUCGAG 5'
AT3G11440.1     1156    1175
transcription factor like protein (MYB65)

sRNA_AGO1_Solexa_Mi2008_5_13219_hit2
5' CUUUGGAUUGAAGGGAGCU
     ||||| |||||||||||
   AUAACCUUACUUCCCUCGA 5'
AT3G11440.1     1157    1175
transcription factor like protein (MYB65)

sRNA_AGO1_Solexa_Mi2008_1_1257_hit1
5' ACA-ACGACAAUGAUGGAGAA
   ||| | ||||| |||||||||
   UGUCU-CUGUUCCUACCUCUU 5'
AT3G11440.1     35      54
transcription factor like protein (MYB65)

sRNA_AGO1_Solexa_Mi2008_2_13419_hit2
5' GAAGAAGAAGAAGACACUU
   |||||||||| ||| ||||
   CUUCUUCUUCCUCU-UGAA 5'
AT3G11720.1     1287    1304
unknown protein

sRNA_AGO1_Solexa_Mi2008_2_13419_hit2
5' GAAGAAGAAGAAGACACUU
   |||||||||| ||| ||||
   CUUCUUCUUCCUCU-UGAA 5'
AT3G11720.2     1336    1353
unknown protein

sRNA_AGO1_Solexa_Mi2008_1_20871_hit4
5' UAGAAGAAAACCGCAGCG-GAC
   ||||||||||| |||||| ||
```

```
   AUCUUCUUUUGCCGUCGCUCUU 5'
AT3G13175.1     296     317
unknown protein

sRNA_AGO1_Solexa_Mi2008_1_20872_hit2
5' UAGAAGAAAACCGCAGCG-GAU
   ||||||||||| |||||| ||
   AUCUUCUUUUGCCGUCGCUCUU 5'
AT3G13175.1     296     317
unknown protein

sRNA_AGO1_Solexa_Mi2008_40_6132_hit1
5' CAGAU-AUGAAAGACCGUGAU
   ||||| ||||||||||||:||
   GUCUACUACUUUCUGGCAUUA 5'
AT3G15010.2     1210    1230
RNA-binding protein

sRNA_AGO1_Solexa_Mi2008_1_36946_hit4
5' UGAUAAG-GUGACAAAGCAUGGU
   ||||| | | ||||||||||||
   ACUAU-CUCUCUGUUUCGUACCA 5'
AT3G15010.2     1698    1719
RNA-binding protein

sRNA_AGO1_Solexa_Mi2008_1_2266_hit1
5' AGUCUCAAGAAAUAUAAUG
   ||| |||||||||||||:
   UCA-AGUUCUUUAUAUUGG 5'
AT3G15010.2     2180    2197
RNA-binding protein

sRNA_AGO1_Solexa_Mi2008_1_33431_hit1
5' UCUGAUCUGUGGUCGCUUGAA
   |||||||||||||||||||||
   AGACUAGACACCAGCGAACUU 5'
AT3G15270.1     614     634
squamosa promoter binding protein-like 5

sRNA_AGO1_Solexa_Mi2008_1_1352_hit1
5' ACAGAAGAGAGAGAGCACU
   ||||||||||||||||
   UGUCUUCUCUCUCUCGCCC 5'
AT3G15270.1     643     661
squamosa promoter binding protein-like 5

sRNA_AGO1_Solexa_Mi2008_1_13615_hit4
5' GACAGAAGAGAGAGAGCACA
   |||||||||||||||||
   CUGUCUUCUCUCUCUCGCCC 5'
AT3G15270.1     643     662
squamosa promoter binding protein-like 5

sRNA_AGO1_Solexa_Mi2008_2_35238_hit4
5' UGACAGAAGAGAGAGAGCACA
   ||||||||||||||||||
   ACUGUCUUCUCUCUCUCGCCC 5'
AT3G15270.1     643     663
squamosa promoter binding protein-like 5

sRNA_AGO1_Solexa_Mi2008_6_13612_hit1
5' GACAGAAGAAAGAGAGCAC
   ||||||||| |||||||
```

```
   CUGUCUUCUCUCUCUCGCC 5'
AT3G15270.1     644     662
squamosa promoter binding protein-like 5

sRNA_AGO1_Solexa_Mi2008_1_13614_hit8
5' GACAGAAGAGAGAGAGCAC
   |||||||||||||||||
   CUGUCUUCUCUCUCUCGCC 5'
AT3G15270.1     644     662
squamosa promoter binding protein-like 5

sRNA_AGO1_Solexa_Mi2008_480_13616_hit7
5' GACAGAAGAGAGUGAGCAC
   |||||||||||| ||||
   CUGUCUUCUCUCUCUCGCC 5'
AT3G15270.1     644     662
squamosa promoter binding protein-like 5

sRNA_AGO1_Solexa_Mi2008_855_13618_hit4
5' GACAGAAGAUAGAGAGCAC
   ||||||||| |||||||
   CUGUCUUCUCUCUCUCGCC 5'
AT3G15270.1     644     662
squamosa promoter binding protein-like 5

sRNA_AGO1_Solexa_Mi2008_6_35236_hit1
5' UGACAGAAGAAAGAGAGCAC
   |||||||||| |||||||
   ACUGUCUUCUCUCUCUCGCC 5'
AT3G15270.1     644     663
squamosa promoter binding protein-like 5

sRNA_AGO1_Solexa_Mi2008_12_35237_hit8
5' UGACAGAAGAGAGAGAGCAC
   ||||||||||||||||||
   ACUGUCUUCUCUCUCUCGCC 5'
AT3G15270.1     644     663
squamosa promoter binding protein-like 5

sRNA_AGO1_Solexa_Mi2008_2091_35240_hit6
5' UGACAGAAGAGAGUGAGCAC
   ||||||||||||| ||||
   ACUGUCUUCUCUCUCUCGCC 5'
AT3G15270.1     644     663
squamosa promoter binding protein-like 5

sRNA_AGO1_Solexa_Mi2008_193_35244_hit4
5' UGACAGAAGAUAGAGAGCAC
   |||||||||| |||||||
   ACUGUCUUCUCUCUCUCGCC 5'
AT3G15270.1     644     663
squamosa promoter binding protein-like 5

sRNA_AGO1_Solexa_Mi2008_14_50257_hit5
5' UUGACAGAAGAGAGAGAGCAC
    ||||||||||||||||||
   UACUGUCUUCUCUCUCUCGCC 5'
AT3G15270.1     644     664
squamosa promoter binding protein-like 5

sRNA_AGO1_Solexa_Mi2008_30_35239_hit6
5' UGACAGAAGAGAGUGAGCA
   ||||||||||||| ||||
```

```
   ACUGUCUUCUCUCUCUCGC 5'
AT3G15270.1     645     663
squamosa promoter binding protein-like 5

sRNA_AGO1_Solexa_Mi2008_1_11426_hit3
5' CUGACAGAAGAGAGUGAGCA
    ||||||||||||| ||||
   UACUGUCUUCUCUCUCUCGC 5'
AT3G15270.1     645     664
squamosa promoter binding protein-like 5

sRNA_AGO1_Solexa_Mi2008_2_11429_hit1
5' CUGACAGAAGAUAGAGAGCA
    |||||||||| |||||||
   UACUGUCUUCUCUCUCUCGC 5'
AT3G15270.1     645     664
squamosa promoter binding protein-like 5

sRNA_AGO1_Solexa_Mi2008_16_50259_hit1
5' UUGACAGAAGAGAGUGAGCA
    ||||||||||||| ||||
   UACUGUCUUCUCUCUCUCGC 5'
AT3G15270.1     645     664
squamosa promoter binding protein-like 5

sRNA_AGO1_Solexa_Mi2008_122_50263_hit3
5' UUGACAGAAGAUAGAGAGCA
    |||||||||| |||||||
   UACUGUCUUCUCUCUCUCGC 5'
AT3G15270.1     645     664
squamosa promoter binding protein-like 5

sRNA_AGO1_Solexa_Mi2008_1_50258_hit1
5' UUGACAGAAGAGAGUGAGC
    ||||||||||||| ||||
   UACUGUCUUCUCUCUCUCG 5'
AT3G15270.1     646     664
squamosa promoter binding protein-like 5

sRNA_AGO1_Solexa_Mi2008_21_50262_hit3
5' UUGACAGAAGAUAGAGAGC
    |||||||||| |||||||
   UACUGUCUUCUCUCUCUCG 5'
AT3G15270.1     646     664
squamosa promoter binding protein-like 5

sRNA_AGO1_Solexa_Mi2008_1_11639_hit1
5' CUGAUUACA-AAAGUAUGAU
   ||||||||| ||| |||||
   GACUAAUGUGUUU-AUACUU 5'
AT3G15356.1     993     1011

sRNA_AGO1_Solexa_Mi2008_1_28834_hit1
5' UCAUCAAGCUUGGGUGCGUUU
   ||||||||||||| ||| ||:
   AGUAGUUCGAACC-ACG-AAG 5'
AT3G15500.1     554     572
putative jasmonic acid regulatory protein

sRNA_AGO1_Solexa_Mi2008_1_7006_hit1
5' CAUCAUCAUCAUCACAGAAG
   |||||||||||||| |||
   GUAGUAGUAGUAGU-UCUGG 5'
```

```
AT3G15970.1     231     249
unknown protein

sRNA_AGO1_Solexa_Mi2008_1_7584_hit16
5' CCA-AAUGCAGAAACCCAUCUU
    || |||||||||||||| |||
   UGUGUUACGUCUUUGGGU-GAA 5'
AT3G16470.1     1557    1577
putative lectin

sRNA_AGO1_Solexa_Mi2008_1_7584_hit16
5' CCA-AAUGCAGAAACCCAUCUU
    || |||||||||||||| |||
   UGUGUUACGUCUUUGGGU-GAA 5'
AT3G16470.2     1562    1582
putative lectin

sRNA_AGO1_Solexa_Mi2008_1_40210_hit1
5' UGGAGAAGGAAGAUGGUGAGAA
   ||| |||||||||||:||| ||
   ACCACUUCCUUCUACUACU-UU 5'
AT3G16720.1     69      89
putative RING zinc finger protein

sRNA_AGO1_Solexa_Mi2008_1_10010_hit16
5' CUACUCAACUUCCGAUGUG
   ||||||||| ||||| |||
   GAUGAGUUGUAGGCU-CAC 5'
AT3G16720.1     922     939
putative RING zinc finger protein

sRNA_AGO1_Solexa_Mi2008_1_15600_hit1
5' GUCAGAAAAUUAGAUAUUU
   |||||||||||  ||||||
   CAGUCUUUUAA--UAUAAA 5'
AT3G17800.2     59      75
unknown protein

sRNA_AGO1_Solexa_Mi2008_2_15560_hit2
5' GUAUGAGAGGGAUAACAGUU
   || |||||| |||||||||
   CA-ACUCUCGCUAUUGUCAC 5'
AT3G18215.1     226     244
unknown protein

sRNA_AGO1_Solexa_Mi2008_5_23592_hit1
5' UAGGUUUGGAUGUCUCU-UUCA
   ||||||||||||| ||| || |
   AUCCAAACCUACAAAGACAACU 5'
AT3G18290.1     3008    3029
zinc finger protein, putative

sRNA_AGO1_Solexa_Mi2008_1_44333_hit1
5' UGUAGUUCCGUUGUC-UUCACA
   ||||||||| ||| | ||||||
   ACAUCAAGGAAAC-GCAAGUGU 5'
AT3G18290.1     3956    3976
zinc finger protein, putative

sRNA_AGO1_Solexa_Mi2008_2_13419_hit2
5' GAAGAAGAAGAAGACACUU
   |||||||||||||||||
   CUUCUUCUUCUUCUGUGUC 5'
```

```
AT3G18290.1     395     413
zinc finger protein, putative

sRNA_AGO1_Solexa_Mi2008_2_51_hit1
5' AAAAAAG-AAGAGAAACAAAGA
   ||||||| || ||||| |||||
   UUUUUUCCUU-UCUUUUUUUCU 5'
AT3G18290.1     4111    4131
zinc finger protein, putative

sRNA_AGO1_Solexa_Mi2008_2_53729_hit1
5' UUGUUAACUAUUUUACACUUC
   |||||||||||| ||||||:
   AACAAUUGAUAA-AUGUGAGA 5'
AT3G18290.1     4372    4391
zinc finger protein, putative

sRNA_AGO1_Solexa_Mi2008_1_39170_hit3
5' UGCUAGAGAGA--AAACGACGUC
   |||| ||||||  ||||||||||
   ACGACCUCUCUACUUUGCUGCAG 5'
AT3G18490.1     191     213
chloroplast nucleoid DNA-binding protein like

sRNA_AGO1_Solexa_Mi2008_2_411_hit5
5' AAAGAUG-AAGAGAGAAAGAGA
   |||| || |||||||||||||
   UUUC-ACCUUCUCUCUUUCUCA 5'
AT3G18490.1     30      50
chloroplast nucleoid DNA-binding protein like

sRNA_AGO1_Solexa_Mi2008_1_49212_hit1
5' UUCGACGGCGAAACGGAUCUU
   |||||||||||||||||||||
   AAGCUGCCGCUUUGCCUAGAA 5'
AT3G18490.1     430     450
chloroplast nucleoid DNA-binding protein like

sRNA_AGO1_Solexa_Mi2008_1_50527_hit1
5' UUGAGAGGCUCCGGAGACGAC
   |||||||||||||||||||||
   AACUCUCCGAGGCCUCUGCUG 5'
AT3G18490.1     526     546
chloroplast nucleoid DNA-binding protein like

sRNA_AGO1_Solexa_Mi2008_10_52860_hit1
5' UU-GGUAAGGGAAGAUAUCCUC
   || ||||| ||||||| |||||
   AAGCCAUUGCCUUCUAGAGGAG 5'
AT3G19620.1     1208    1229
beta-xylosidase, putative

sRNA_AGO1_Solexa_Mi2008_1_269_hit2
5' AAAAUGGUGGAGAAAGAAGAG
   |||| | | ||||||||||||
   UUUU-C-AGCUCUUUCUUCUC 5'
AT3G19620.1     1884    1902
beta-xylosidase, putative

sRNA_AGO1_Solexa_Mi2008_5_29024_hit1
5' UCAUGGUUUCAAGAAUGCU
    ||||||||||||||| ||
   UGUACCAAAGUUCUUA-GA 5'
```

```
AT3G22104.1     1157    1174
hypothetical protein, 3' partial

sRNA_AGO1_Solexa_Mi2008_1_806_hit1
5' AAGAGCAUCAAUGAUAGUGUU
   ||||| ||||||||| | |||
   UUCUCAUAGUUACUA-C-CAA 5'
AT3G22104.1     1167    1185
hypothetical protein, 3' partial

sRNA_AGO1_Solexa_Mi2008_14_49272_hit2
5' UUCGAUGGAGAUGG-ACAAGUU
   |||||||||||||| |||||
   AAGCUACCUCUACCUUGUUCUU 5'
AT3G22104.1     1331    1352
hypothetical protein, 3' partial

sRNA_AGO1_Solexa_Mi2008_1_38236_hit3
5' UGCAUCUGUUGUUGGUGUUGC
   ||| |||||||||| |||||
   ACGAAGACAACAACAACAACA 5'
AT3G22104.1     1739    1759
hypothetical protein, 3' partial

sRNA_AGO1_Solexa_Mi2008_3_4691_hit4
5' CAAGAUGUUUUUCUUGUGG-GA
   ||| ||||||||||||||| ||
   GUU-UACAAAAAGAACACCACU 5'
AT3G23250.2     229     249
myb-related transcription factor like protein

sRNA_AGO1_Solexa_Mi2008_3_4691_hit4
5' CAAGAUGUUUUUCUUGUGG-GA
   ||| ||||||||||||||| ||
   GUU-UACAAAAAGAACACCACU 5'
AT3G23250.1     230     250
myb-related transcription factor like protein

sRNA_AGO1_Solexa_Mi2008_1_23588_hit1
5' UAGGUUUAGGUGGUUAAUAAA
   ||||||||| ||||| ||||
   AUCCAAAUCGACCAACUAUUA 5'
AT3G23250.1     635     655
myb-related transcription factor like protein

sRNA_AGO1_Solexa_Mi2008_1_23588_hit1
5' UAGGUUUAGGUGGUUAAUAAA
   ||||||||| ||||| ||||
   AUCCAAAUCGACCAACUAUUA 5'
AT3G23250.2     713     733
myb-related transcription factor like protein

sRNA_AGO1_Solexa_Mi2008_1_45425_hit9
5' UGUGGGUA-UGGAAUUCGGAAC
   |||||||| | ||||| |||||
   ACACCCAUCA-CUUAACCCUUG 5'
AT3G27060.1     295     315
ribonucleotide reductase small subunit, putative

sRNA_AGO1_Solexa_Mi2008_1_42_hit14
5' AAAAAACAGAAAACAA-UGGAU
   ||||||||||| |||| ||| |
   UUUUUUGUCUUGUGUUAACCAA 5'
```

```
AT3G28220.1     1161    1182
unknown protein

sRNA_AGO1_Solexa_Mi2008_1_379_hit1
5' AAAGAGA-AGUAUGAAAACUCA
   ||||| | ||||||| ||||||
   UUUCU-UGUCAUACUGUUGAGU 5'
AT3G28600.1     1134    1154
unknown protein

sRNA_AGO1_Solexa_Mi2008_2_3516_hit21
5' AUUACCUUCACGUGCUCUUCC
   |||||||:||||||| ||||
   UAAUGGAGGUGCACG-GAAGU 5'
AT3G29670.1     937     956
anthocyanin 5-aromatic acyltransferase, putative

sRNA_AGO1_Solexa_Mi2008_1_1776_hit10
5' AGAAAAAAAA-AAAAAAAAAAU
     |||||||| |||||||||||
   CAUUUUUUUUAUUUUUUUUUUA 5'
AT3G44260.1     916     937
CCR4-associated factor 1-like protein

sRNA_AGO1_Solexa_Mi2008_3_23318_hit2
5' UAG-GUAAAAACAUCUGGUCUA
   ||| |||||| ||||||||||
   AUCACAUUUU-GUAGACCAGAG 5'
AT3G44300.1     961     981
nitrilase 2

sRNA_AGO1_Solexa_Mi2008_3_23318_hit2
5' UAG-GUAAAAACAUCUGGUCUA
   ||| |||||| ||||||||||
   AUCACAUUUU-GUAGACCAGAG 5'
AT3G44310.2     861     881
nitrilase 1

sRNA_AGO1_Solexa_Mi2008_3_23318_hit2
5' UAG-GUAAAAACAUCUGGUCUA
   ||| |||||| ||||||||||
   AUCACAUUUU-GUAGACCAGAG 5'
AT3G44310.3     979     999
nitrilase 1

sRNA_AGO1_Solexa_Mi2008_1_22606_hit1
5' UAGGAACGACGUGUACUCUUG
   |||||||||||||||||||||
   AUCCUUGCUGCACAUGAGAAC 5'
AT3G44720.1     1295    1315
chloroplast prephenate dehydratase like protein

sRNA_AGO1_Solexa_Mi2008_1_15995_hit1
5' GUGGUAAAUGAUGGUAAGA
   :|||||| |||||||||||
   UACCAUU-ACUACCAUUCU 5'
AT3G44720.1     87      104
chloroplast prephenate dehydratase like protein

sRNA_AGO1_Solexa_Mi2008_1_28151_hit1
5' UCAGAGUCCAGAUCCCAUCAU
   || ||:||||||||||||||
   AG-CUUAGGUCUAGGGUAGUU 5'
```

AT3G46620.1   524   543
unknown protein

sRNA_AGO1_Solexa_Mi2008_1_14149_hit1
5' GAGUGUGUACAC-GACGGUGU
   |||||||||||| | |||| |
   CUCACACAUGUGUC-GCCAAA 5'
AT3G46900.1   287   306
copper transport protein - like protein

sRNA_AGO1_Solexa_Mi2008_1_37226_hit1
5' UGAUGAUGAUGGUACCAUGGU
   ||||||||||||| |||||
   ACUACUACUACCAC--UACCA 5'
AT3G46900.1   45   63
copper transport protein - like protein

sRNA_AGO1_Solexa_Mi2008_1_7157_hit1
5' CAUGAUGAUGAUGG-AACUGGU
   |||||||||||||| | ||||
   GUACUACUACUACCACU-ACCA 5'
AT3G46900.1   45   65
copper transport protein - like protein

sRNA_AGO1_Solexa_Mi2008_1_3079_hit472
5' AU-GAUGAUGAUGAUGAUGAUGA
   || ||||||||||||| |||||
   UAGCUACUACUACUACCACUACC 5'
AT3G46900.1   46   68
copper transport protein - like protein

sRNA_AGO1_Solexa_Mi2008_1_3_hit25
5' AAAAAAAAAAAAAGAAAGA
   | ||||||||||| |||||
   UAUUUUUUUUUUUGUUUCU 5'
AT3G46900.1   503   521
copper transport protein - like protein

sRNA_AGO1_Solexa_Mi2008_6_35094_hit47
5' UGAAUGUGUUUUGGGU-GAA
   |||||||||||| ||| ||
   ACUUACACAAAA-CCAUCUA 5'
AT3G47340.2   1518   1536
glutamine-dependent asparagine synthetase

sRNA_AGO1_Solexa_Mi2008_6_35094_hit47
5' UGAAUGUGUUUUGGGU-GAA
   ||||||||||||| || ||
   ACUUACACAAAAC-CAUCUA 5'
AT3G47340.1   1519   1537
glutamine-dependent asparagine synthetase

sRNA_AGO1_Solexa_Mi2008_6_35094_hit47
5' UGAAUGUGUUUUGGGU-GAA
   |||||||||||| ||| ||
   ACUUACACAAAA-CCAUCUA 5'
AT3G47340.3   1601   1619
glutamine-dependent asparagine synthetase

sRNA_AGO1_Solexa_Mi2008_1_2556_hit1
5' AUAGAUUCAGAUCUUCAG-GGA
   ||| ||||||||||||| |||
   UAUGUAAGUCUAGAAGUAUCCU 5'

AT3G47340.3   2224   2245
glutamine-dependent asparagine synthetase

sRNA_AGO1_Solexa_Mi2008_2_4447_hit2
5' CAACGAGCUGUGUUGGUCCAG
   ||||||||||||| | ||||:
   GUUGCUCGACACA-CAAGGUU 5'
AT3G47420.1   1522   1541
putative sugar transporter protein

sRNA_AGO1_Solexa_Mi2008_1_21351_hit8
5' UAGAGGAAAUGAGUGUGUUAUUCC
   ||||:|||||||||||||||||||
   AUCUUCUUUACUCACACAAUAAGG 5'
AT3G50480.1   1006   1029
RPW8- like protein 4 (HR4)

sRNA_AGO1_Solexa_Mi2008_1_21350_hit1
5' UAGAGGAAAUGAGUGUGAUAU
   ||||:|||||||||||| |||
   AUCUUCUUUACUCACACAAUA 5'
AT3G50480.1   1009   1029
RPW8- like protein 4 (HR4)

sRNA_AGO1_Solexa_Mi2008_1_2507_hit3
5' AUAGAAGAAAUGAGUGUGUUU
    |||||||||||||||||||
   _AUCUUCUUUACUCACACAAU 5'
AT3G50480.1   1010   1030
RPW8- like protein 4 (HR4)

sRNA_AGO1_Solexa_Mi2008_1_37358_hit1
5' UGAUGGGCCUUAUAAUGGGCAUUC
   ||||||||||||||||||||||:|
   ACUACCCGGAAUAUUACCCGUAGG 5'
AT3G50480.1   905   928
RPW8- like protein 4 (HR4)

sRNA_AGO1_Solexa_Mi2008_1_37374_hit5
5' UGAUGGGUCUUAUAAUGGGCAUCC
   |||||||:||||||||||||||||
   ACUACCCGGAAUAUUACCCGUAGG 5'
AT3G50480.1   905   928
RPW8- like protein 4 (HR4)

sRNA_AGO1_Solexa_Mi2008_1_42681_hit5
5' UGGGUCUUAUAAUGGGCAU
   ||||:||||||||||||||
   ACCCGGAAUAUUACCCGUA 5'
AT3G50480.1   907   925
RPW8- like protein 4 (HR4)

sRNA_AGO1_Solexa_Mi2008_1_37373_hit5
5' UGAUGGGUCUUAUAAUGGGCAU
   |||||||:||||||||||||||
   ACUACCCGGAAUAUUACCCGUA 5'
AT3G50480.1   907   928
RPW8- like protein 4 (HR4)

sRNA_AGO1_Solexa_Mi2008_1_37356_hit1
5' UGAUGGGCCUCAUAAUGGGCA
   |||||||||| ||||||||||
   ACUACCCGGAAUAUUACCCGU 5'

AT3G50480.1   908   928
RPW8- like protein 4 (HR4)

sRNA_AGO1_Solexa_Mi2008_5_37357_hit26
5' UGAUGGGCCUUAUAAUGGGCA
   |||||||||||||||||||||
   ACUACCCGGAAUAUUACCCGU 5'
AT3G50480.1   908   928
RPW8- like protein 4 (HR4)

sRNA_AGO1_Solexa_Mi2008_1_12412_hit2
5' CUUAGAUAAUGGGCCUUAUAA
   ||||||| |||||||||||||
   GAAUCUACUACCCGGAAUAUU 5'
AT3G50480.1   914   934
RPW8- like protein 4 (HR4)

sRNA_AGO1_Solexa_Mi2008_1_42239_hit4
5' UGGGCUUAGAUGAUGGGUCUUAU
   |||||||||||||||||:|||||
   ACCCGAAUCUACUACCCGGAAUA 5'
AT3G50480.1   916   938
RPW8- like protein 4 (HR4)

sRNA_AGO1_Solexa_Mi2008_2_42236_hit1
5' UGGGCUUAGAUGAUGGACCUU
   |||||||||||||||| ||||
   ACCCGAAUCUACUACCCGGAA 5'
AT3G50480.1   918   938
RPW8- like protein 4 (HR4)

sRNA_AGO1_Solexa_Mi2008_2_42238_hit4
5' UGGGCUUAGAUGAUGGGUCUU
   |||||||||||||||||:|||
   ACCCGAAUCUACUACCCGGAA 5'
AT3G50480.1   918   938
RPW8- like protein 4 (HR4)

sRNA_AGO1_Solexa_Mi2008_2_42237_hit4
5' UGGGCUUAGAUGAUGGGUCU
   |||||||||||||||||:||
   ACCCGAAUCUACUACCCGGA 5'
AT3G50480.1   919   938
RPW8- like protein 4 (HR4)

sRNA_AGO1_Solexa_Mi2008_1_14303_hit4
5' GAUGGGCUUAGAUGAUGGGUCU
   |||||||||||||||||||:||
   CUACCCGAAUCUACUACCCGGA 5'
AT3G50480.1   919   940
RPW8- like protein 4 (HR4)

sRNA_AGO1_Solexa_Mi2008_4_37363_hit4
5' UGAUGGGCUUAGAUGAUGGGU
   ||||||||||||||||||||:
   ACUACCCGAAUCUACUACCCG 5'
AT3G50480.1   921   941
RPW8- like protein 4 (HR4)

sRNA_AGO1_Solexa_Mi2008_1_37364_hit1
5' UGAUGGGCUUAGAUGAUUGGC
   |||||||||||||||| |||
   ACUACCCGAAUCUACU-ACCC 5'

```
AT3G50480.1     923     942
RPW8- like protein 4 (HR4)

sRNA_AGO1_Solexa_Mi2008_3_14257_hit30
5' GAUGAUGGGCUUAGAUGAU
   |||||||||||||||||||
   CUACUACCCGAAUCUACUA 5'
AT3G50480.1     925     943
RPW8- like protein 4 (HR4)

sRNA_AGO1_Solexa_Mi2008_1_21813_hit1
5' UAGAUGAUGGACUUCGAUGAU
   |||||||||| ||| ||||||
   AUCUACUACCCGAAUCUACUA 5'
AT3G50480.1     925     945
RPW8- like protein 4 (HR4)

sRNA_AGO1_Solexa_Mi2008_6_21815_hit1
5' UAGAUGAUGGGCUUAAAUGAU
   ||||||||||||||| |||||
   AUCUACUACCCGAAUCUACUA 5'
AT3G50480.1     925     945
RPW8- like protein 4 (HR4)

sRNA_AGO1_Solexa_Mi2008_5_21816_hit30
5' UAGAUGAUGGGCUUAGAUGAU
   |||||||||||||||||||||
   AUCUACUACCCGAAUCUACUA 5'
AT3G50480.1     925     945
RPW8- like protein 4 (HR4)

sRNA_AGO1_Solexa_Mi2008_1_21817_hit1
5' UAGAUGAUGGGUUUAGAUGAU
   |||||||||||:|||||||||
   AUCUACUACCCGAAUCUACUA 5'
AT3G50480.1     925     945
RPW8- like protein 4 (HR4)

sRNA_AGO1_Solexa_Mi2008_1_10162_hit2
5' CUAGAUGAUGGACUUAGAUGA
   ||||||||||| |||||||||
   GAUCUACUACCCGAAUCUACU 5'
AT3G50480.1     926     946
RPW8- like protein 4 (HR4)

sRNA_AGO1_Solexa_Mi2008_6_10164_hit27
5' CUAGAUGAUGGGCUUAGAUGA
   |||||||||||||||||||||
   GAUCUACUACCCGAAUCUACU 5'
AT3G50480.1     926     946
RPW8- like protein 4 (HR4)

sRNA_AGO1_Solexa_Mi2008_1_32880_hit27
5' UCUAGAUGAUGGGCUUAGAUGA
   ||||||||||||||||||||||
   AGAUCUACUACCCGAAUCUACU 5'
AT3G50480.1     926     947
RPW8- like protein 4 (HR4)

sRNA_AGO1_Solexa_Mi2008_7_10163_hit27
5' CUAGAUGAUGGGCUUAGAUG
   ||||||||||||||||||||
   GAUCUACUACCCGAAUCUAC 5'
```

```
AT3G50480.1     927     946
RPW8- like protein 4 (HR4)

sRNA_AGO1_Solexa_Mi2008_2_32879_hit27
5' UCUAGAUGAUGGGCUUAGAUG
   |||||||||||||||||||||
   AGAUCUACUACCCGAAUCUAC 5'
AT3G50480.1     927     947
RPW8- like protein 4 (HR4)

sRNA_AGO1_Solexa_Mi2008_1_20757_hit22
5' UAGAAACAUCUAGAUGAUGGGCUU
   ||||||||||||||||||||||||
   AUCUUUGUAGAUCUACUACCCGAA 5'
AT3G50480.1     932     955
RPW8- like protein 4 (HR4)

sRNA_AGO1_Solexa_Mi2008_1_19030_hit3
5' UACAAACAUCUAGAUGAUGGGCU
   || ||||||||||||||||||||
   AUCUUUGUAGAUCUACUACCCGA 5'
AT3G50480.1     933     955
RPW8- like protein 4 (HR4)

sRNA_AGO1_Solexa_Mi2008_5_10081_hit25
5' CUAGAAACAUCUAGAUGAUGG
   |||||||||||||||||||||
   GAUCUUUGUAGAUCUACUACC 5'
AT3G50480.1     936     956
RPW8- like protein 4 (HR4)

sRNA_AGO1_Solexa_Mi2008_4_14880_hit24
5' GGAGUACAAGGAAAGGGUA
   |||||||||||||||||||
   CCUCAUGUUCCUUUCCCAU 5'
AT3G50480.1     962     980
RPW8- like protein 4 (HR4)

sRNA_AGO1_Solexa_Mi2008_1_23502_hit2
5' UAGGUGUACAAGGAAAGGGUA
   |||| ||||||||||||||||
   AUCCUCAUGUUCCUUUCCCAU 5'
AT3G50480.1     962     982
RPW8- like protein 4 (HR4)

sRNA_AGO1_Solexa_Mi2008_5_24584_hit2
5' UAUAGGUGUACAAGGAAAGGGU
   |||||| |||||||||||||||
   AUAUCCUCAUGUUCCUUUCCCA 5'
AT3G50480.1     963     984
RPW8- like protein 4 (HR4)

sRNA_AGO1_Solexa_Mi2008_1_22768_hit2
5' UAGGAGUACAAGGAAAAGGGU
   ||||||||||||||| |||||
   AUCCUCAUGUUCCUU-UCCCA 5'
AT3G50480.1     964     983
RPW8- like protein 4 (HR4)

sRNA_AGO1_Solexa_Mi2008_1_2590_hit4
5' AUAGGAGUACAAGGAAAAGG
   |||||||||||||||| |||
   UAUCCUCAUGUUCCUU-UCC 5'
```

```
AT3G50480.1     966     984
RPW8- like protein 4 (HR4)

sRNA_AGO1_Solexa_Mi2008_1_54542_hit29
5' UUUAUAUAGGAGUACAAGGAA
   |||||||||||||||||||||
   AAAUAUAUCCUCAUGUUCCUU 5'
AT3G50480.1     968     988
RPW8- like protein 4 (HR4)

sRNA_AGO1_Solexa_Mi2008_1_35975_hit2
5' UGAGAUGAGUGACCAUGGCUGU
   |||||||| || |||||||||:
   ACUCUACUAACCGGUACCGACG 5'
AT3G50930.1     1239    1260
BCS1 protein-like protein

sRNA_AGO1_Solexa_Mi2008_2_35974_hit2
5' UGAGAUGAGUGACCAUGGCU
   |||||||| || ||||||||
   ACUCUACUAACCGGUACCGA 5'
AT3G50930.1     1241    1260
BCS1 protein-like protein

sRNA_AGO1_Solexa_Mi2008_1_24431_hit2
5' UAUA-GACAUGUGGAUGAUGCAC
   |||| |||||||||||| ||||
   AUAUGCUGUACACCUAC-ACGUA 5'
AT3G50930.1     1585    1606
BCS1 protein-like protein

sRNA_AGO1_Solexa_Mi2008_6_24430_hit2
5' UAUA-GACAUGUGGAUGAUGCA
   |||| |||||||||||| ||||
   AUAUGCUGUACACCUAC-ACGU 5'
AT3G50930.1     1586    1606
BCS1 protein-like protein

sRNA_AGO1_Solexa_Mi2008_1_24432_hit1
5' UAUA-GACAUGUGGAUGAUGCG
   |||| |||||||||||| |||:
   AUAUGCUGUACACCUAC-ACGU 5'
AT3G50930.1     1586    1606
BCS1 protein-like protein

sRNA_AGO1_Solexa_Mi2008_23_6153_hit2
5' CAGAUCUUGGUGG-UAG-UAGC
   ||||||||||||| ||| |||
   GUCUAGAACCACCGAUCUAUCC 5'
AT3G50930.1     671     692
BCS1 protein-like protein

sRNA_AGO1_Solexa_Mi2008_2_43473_hit2
5' UGGUGGAUGUAUUUGAAU-CAU
   ||| |||||| ||||||| |||
   ACCUCCUACACAAACUUAGGUA 5'
AT3G51730.1     955     976
unknown protein

sRNA_AGO1_Solexa_Mi2008_1_3398_hit2
5' AUGGUGGAUGUAUUUGAAU-CAU
   |||| |||||| ||||||| |||
   UACCUCCUACACAAACUUAGGUA 5'
```

```
AT3G51730.1     955     977
unknown protein

sRNA_AGO1_Solexa_Mi2008_1_49005_hit4
5' UUCCA-GUGAAGCAAGAAACUGA
   ||||| || | ||||||||||||
   AAGGUGCAGU-CGUUCUUUGACU 5'
AT3G53950.1     694     715
unknown protein

sRNA_AGO1_Solexa_Mi2008_1_50463_hit1
5' UUGAGAAACAAG-UUGUCUGCA
   |||||||||||| |||| |||
   AACUCUUUGUUCGAACA-ACGA 5'
AT3G55610.2     1578    1598
delta-1-pyrroline-5-carboxylate synthetase

sRNA_AGO1_Solexa_Mi2008_9_14634_hit2
5' GCCU-AUCCUCAGAAUCGCU
   |||| |||||||| ||||||
   CGGAGUAGGAGUCGUAGCGA 5'
AT3G55610.2     530     549
delta-1-pyrroline-5-carboxylate synthetase

sRNA_AGO1_Solexa_Mi2008_3_6076_hit1
5' CA-GAGGGAGAUGAAAGAAUU
   || | ||||||| ||||||||
   GUAC-CCCUCUAAUUUCUUAA 5'
AT3G55970.1     377     396
leucoanthocyanidin dioxygenase -like protein

sRNA_AGO1_Solexa_Mi2008_1_42962_hit1
5' UGGUAGUAGAGAUAAUAGUAU
   |||||:| ||||||||||||
   ACCAUUACCUCUAUUAUCAUC 5'
AT3G55970.1     491     511
leucoanthocyanidin dioxygenase -like protein

sRNA_AGO1_Solexa_Mi2008_1_5113_hit1
5' CAAUCCGAGAGAUUCGA-UGUCC
   |||||||||||||||| |||
   GUUAGGCUCUCUAAGCUGACACC 5'
AT3G55980.1     1623    1645
unknown protein

sRNA_AGO1_Solexa_Mi2008_1_35795_hit1
5' UGAG-AAGAUUAUGGGUUUCGG
   |||| ||||||||:||||| ||
   ACUCCUUCUAAUAUCCAAA-CC 5'
AT3G56060.1     7       27
mandelonitrile lyase-like protein

sRNA_AGO1_Solexa_Mi2008_5_1114_hit1
5' AAUG-GAACUAGUGGACAAACAA
   |||| ||||||||||| |||||
   UUACUCUUGAUCACCUAUUUGUG 5'
AT3G56970.1     840     862
putative bHLH transcription factor  (bHLH038)

sRNA_AGO1_Solexa_Mi2008_1_48516_hit1
5' UUCAAGGAAGAUUGGACCAGU
   |||||||||||||| |||:|
   AAGUUCCUUCUAACC-GGUUA 5'
```

```
AT3G57170.1     1048    1067
unknown protein

sRNA_AGO1_Solexa_Mi2008_3_45808_hit1
5' UGUUAAAGAAGAUGGAACAAG
   |||||||||||||  | ||||
   ACAAUUUCUUCUA--UAGUUC 5'
AT3G57170.1     857     875
unknown protein

sRNA_AGO1_Solexa_Mi2008_2_13688_hit1
5' GACCAUUUGUGAGAAGAGA
   |||||||||||||||||||
   CUGGUAAACACUCUUCUCU 5'
AT3G57230.2     585     603
MADS-box transcription factor (AGL16)

sRNA_AGO1_Solexa_Mi2008_596_13689_hit1
5' GACCAUUUGUGAGAAGGGA
   ||||||||||||||||:||
   CUGGUAAACACUCUUCUCU 5'
AT3G57230.2     585     603
MADS-box transcription factor (AGL16)

sRNA_AGO1_Solexa_Mi2008_72_1853_hit1
5' AGACCAUUUGUGAGAAGGGA
   |||||||||||||||||:||
   UCUGGUAAACACUCUUCUCU 5'
AT3G57230.2     585     604
MADS-box transcription factor (AGL16)

sRNA_AGO1_Solexa_Mi2008_3321_21060_hit1
5' UAGACCAUUUGUGAGAAGGGA
   ||||||||||||||||||:||
   AUCUGGUAAACACUCUUCUCU 5'
AT3G57230.2     585     605
MADS-box transcription factor (AGL16)

sRNA_AGO1_Solexa_Mi2008_2_10113_hit1
5' CUAGACCAUUUGUGAGAAGGGA
   |||||||||||||||||||:||
   GAUCUGGUAAACACUCUUCUCU 5'
AT3G57230.2     585     606
MADS-box transcription factor (AGL16)

sRNA_AGO1_Solexa_Mi2008_32_21059_hit1
5' UAGACCAUUUGUGAGAAGGG
   ||||||||||||||||||:|
   AUCUGGUAAACACUCUUCUC 5'
AT3G57230.2     586     605
MADS-box transcription factor (AGL16)

sRNA_AGO1_Solexa_Mi2008_37_21058_hit1
5' UAGACCAUUUGUGAGAAGG
   ||||||||||||||||||:
   AUCUGGUAAACACUCUUCU 5'
AT3G57230.2     587     605
MADS-box transcription factor (AGL16)

sRNA_AGO1_Solexa_Mi2008_2_13688_hit1
5' GACCAUUUGUGAGAAGAGA
   |||||||||||||||||||
   CUGGUAAACACUCUUCUCU 5'
```

```
AT3G57230.1     588     606
MADS-box transcription factor (AGL16)

sRNA_AGO1_Solexa_Mi2008_596_13689_hit1
5' GACCAUUUGUGAGAAGGGA
   ||||||||||||||||:||
   CUGGUAAACACUCUUCUCU 5'
AT3G57230.1     588     606
MADS-box transcription factor (AGL16)

sRNA_AGO1_Solexa_Mi2008_72_1853_hit1
5' AGACCAUUUGUGAGAAGGGA
   |||||||||||||||||:||
   UCUGGUAAACACUCUUCUCU 5'
AT3G57230.1     588     607
MADS-box transcription factor (AGL16)

sRNA_AGO1_Solexa_Mi2008_3321_21060_hit1
5' UAGACCAUUUGUGAGAAGGGA
   ||||||||||||||||||:||
   AUCUGGUAAACACUCUUCUCU 5'
AT3G57230.1     588     608
MADS-box transcription factor (AGL16)

sRNA_AGO1_Solexa_Mi2008_2_10113_hit1
5' CUAGACCAUUUGUGAGAAGGGA
   |||||||||||||||||||:||
   GAUCUGGUAAACACUCUUCUCU 5'
AT3G57230.1     588     609
MADS-box transcription factor (AGL16)

sRNA_AGO1_Solexa_Mi2008_32_21059_hit1
5' UAGACCAUUUGUGAGAAGGG
   ||||||||||||||||||:|
   AUCUGGUAAACACUCUUCUC 5'
AT3G57230.1     589     608
MADS-box transcription factor (AGL16)

sRNA_AGO1_Solexa_Mi2008_37_21058_hit1
5' UAGACCAUUUGUGAGAAGG
   ||||||||||||||||||:
   AUCUGGUAAACACUCUUCU 5'
AT3G57230.1     590     608
MADS-box transcription factor (AGL16)

sRNA_AGO1_Solexa_Mi2008_1_37480_hit2
5' UGAUGUG-G-GAGAUGAGAAUGU
    |||||| | |||||||||||||
   UCUACACUCACUCUACUCUUACA 5'
AT3G57510.1     33      55
endo-polygalacturonase

sRNA_AGO1_Solexa_Mi2008_1_27213_hit1
5' UCAAGGCUAGAGAACAUUUGGA
   ||||:| |||| ||||||||||
   AGUUUC-AUCUGUUGUAAACCU 5'
AT3G59210.1     1073    1093
unknown protein

sRNA_AGO1_Solexa_Mi2008_2_13650_hit1
5' GACAUCCAGAUAGAAGCUUU
   |||| ||||| |||||||||
   CUGUUGGUCUUUCUUCGAAA 5'
```

```
AT3G59210.1     913     932
unknown protein

sRNA_AGO1_Solexa_Mi2008_4_35304_hit1
5' UGACAUCCAGAUAGAAGCUUU
    |||| ||||| |||||||||
   CCUGUUGGUCUUUCUUCGAAA 5'
AT3G59210.1     913     933
unknown protein

sRNA_AGO1_Solexa_Mi2008_1_52192_hit1
5' UUGGAUCAACAAACUGAUGG
   ||||||||| |||||| ||
   AACCUAGUUCUUUGAC-ACA 5'
AT3G59900.1     35      53
putative protein

sRNA_AGO1_Solexa_Mi2008_1_18400_hit1
5' UAAGGUUCGUUGAUUGUUGUC
   |||||||||||||||||||||
   AUUCCAAGCAACUAACAACAG 5'
AT3G60140.1     647     667
beta-glucosidase

sRNA_AGO1_Solexa_Mi2008_1_24287_hit1
5' UAUAAGGUUCGUUGAUUGUUGUC
   |||||||||||||||||||||||
   AUAUUCCAAGCAACUAACAACAG 5'
AT3G60140.1     647     669
beta-glucosidase

sRNA_AGO1_Solexa_Mi2008_2_44860_hit1
5' UGUCUUUAGAGAUCUCAAACGU
   ||||||| ||||| ||||| ||
   ACAGAAAACUCUAAAGUUU-CA 5'
AT3G60140.1     6       26
beta-glucosidase

sRNA_AGO1_Solexa_Mi2008_1_47622_hit1
5' UUAGCUACACAA-AGUAUUGAU
   ||||||||| || ||||||||
   AAUCGAUGU-UUCUCAUAACUU 5'
AT3G61890.1     919     939
homeobox-leucine zipper protein ATHB-12

sRNA_AGO1_Solexa_Mi2008_1_49005_hit4
5' UUCCAGUGAAGCAAGAAACUGA
   ||| | |||||||||||||||
   AAG-UGACUUCGUUCUUUGACG 5'
AT4G01080.1     466     486
unknown protein (At4g01080)

sRNA_AGO1_Solexa_Mi2008_21_12055_hit2
5' CUGGUAGUGUGAAGUU-UGA
   |||:|||||||||||| |||
   GACUAUCACACUUCAACACU 5'
AT4G01080.1     628     647
unknown protein (At4g01080)

sRNA_AGO1_Solexa_Mi2008_1_45014_hit1
5' UGUGAUAGUGUGAAGUUUGUGA
   ||||||||||||||||| ||||
   ACACUAUCACACUUCAA-CACU 5'
```

```
AT4G01080.1     628     648
unknown protein (At4g01080)

sRNA_AGO1_Solexa_Mi2008_1_44646_hit1
5' UGUCCUAAACGAAGCGGUGGU
   |||||||||||||||||||||
   ACAGGAUUUGCUUCGCCACCA 5'
AT4G01410.1     538     558
putative hypoersensitive response protein

sRNA_AGO1_Solexa_Mi2008_1_44726_hit1
5' UGUCGAUUGAUUUACCGU--GGA
   ||||||||||||||||||  |||
   ACAGCUAACUAAAUGGCAGGCCU 5'
AT4G01870.1     1374    1396
unknown protein

sRNA_AGO1_Solexa_Mi2008_5_11906_hit6
5' CUGGAGCUACGGGAA-CUGCUG
   |||||||| |||||| ||||||
   GACCUCGAGGCCCUUAGACGAG 5'
AT4G01870.1     247     268
unknown protein

sRNA_AGO1_Solexa_Mi2008_33_11834_hit6
5' CUGCUGGAGCUACGGGAA-CUG
   || |||||||| |||||| |||
   GA-GACCUCGAGGCCCUUAGAC 5'
AT4G01870.1     250     270
unknown protein

sRNA_AGO1_Solexa_Mi2008_1_13362_hit1
5' GAAAUGCUCUAAGUCUCCUAG
   :||| ||| ||||||||||||
   UUUU-CGACAUUCAGAGGAUC 5'
AT4G02280.1     1858    1877
putative sucrose synthetase

sRNA_AGO1_Solexa_Mi2008_11_14066_hit1
5' GA-GGAGUUGAAUAUCUGUUCA
   || ||| |||||||| ||||||
   CUACCUGAACUUAUAAACAAGU 5'
AT4G02520.1     489     510
Atpm24.1 glutathione S transferase

sRNA_AGO1_Solexa_Mi2008_1_15664_hit1
5' GUCCGGUUUUGGAUACGUG
   |||||||||||||:|||||
   CAGGCCAAAACCUGUGCAC 5'
AT4G03060.1     1166    1184
putative oxidoreductase

sRNA_AGO1_Solexa_Mi2008_1_23752_hit1
5' UAGUCCGGUUUUGGAUACGUG
   |||||||||||||||:|||||
   AUCAGGCCAAAACCUGUGCAC 5'
AT4G03060.1     1166    1186
putative oxidoreductase

sRNA_AGO1_Solexa_Mi2008_30_46350_hit1
5' UUAACACCAACAACGAGAUC-GU
   |||||||||||| |:||||| ||
   AAUUGUGGUUGUGGUUCUAGUCA 5'
```

AT4G03060.1   826   848
putative oxidoreductase

sRNA_AGO1_Solexa_Mi2008_1_14952_hit2
5' GGCACGUGCUGAUUGUU-GUU
   :|||| ||||||||||| |||
   UCGUG-ACGACUAACAACCAA 5'
AT4G04460.1   1315   1334
putative aspartic protease

sRNA_AGO1_Solexa_Mi2008_1_13343_hit1
5' GAA-AGGAGCAAGAUUGAUUGG
   ||| | |||||||| |||||||
   CUUGUGCUCGUUCUUACUAACC 5'
AT4G04460.1   72   93
putative aspartic protease

sRNA_AGO1_Solexa_Mi2008_1_1786_hit1
5' AGAA-AGGAGCAAGAUUGAUUGG
   |||| | |||||||| |||||||
   UCUUGUGCUCGUUCUUACUAACC 5'
AT4G04460.1   72   94
putative aspartic protease

sRNA_AGO1_Solexa_Mi2008_1_51110_hit1
5' UUGAUGAUGAUCACAGUUGUGU
   ||||||||||||| |||| ||
   AACUACUACUAGUC-CAAC-CA 5'
AT4G04830.1   1   20
Unknown protein

sRNA_AGO1_Solexa_Mi2008_1_30428_hit1
5' UCGAACUUCGUGUGUAGAUA
   |||||||||||| || |||
   AGCUUGAAGCAC-CAACUAA 5'
AT4G04830.1   222   240
Unknown protein

sRNA_AGO1_Solexa_Mi2008_1_14057_hit1
5' GAGGAGAAGCAGAAGCAAG-UU
   ||||| |||||||||| || ||
   CUCCUGUUCGUCUUCG-UCGAA 5'
AT4G08950.1   848   868
putative phi-1-like phosphate-induced protein

sRNA_AGO1_Solexa_Mi2008_1_24907_hit1
5' UAUCAUCUACGAGGUCAGUCA
   ||||||| |||||||||||
   AUAGUAGUUGCUCCAGUCAAA 5'
AT4G11130.1   3024   3044
putative RNA-directed RNA polymerase

sRNA_AGO1_Solexa_Mi2008_1_1044_hit2
5' AAUCCUGAUGAUG-CUGCAU
   ||||||||||||| || |||
   UUAGGACUACUACUGAGGUA 5'
AT4G12560.2   1555   1574
putative protein

sRNA_AGO1_Solexa_Mi2008_1_13548_hit2
5' GAAUCCUGAUGAUG-CUGCAU
   :||||||||||||| || |||
   UUUAGGACUACUACUGAGGUA 5'

```
AT4G12560.2     1555    1575
putative protein

sRNA_AGO1_Solexa_Mi2008_2_1701_hit3
5' ACUCAAGAGAUGAU-AUAAA
     |||||||||||| |||||
   AUAGUUCUCUACUAUUAUUU 5'
AT4G12560.2     653     672
putative protein

sRNA_AGO1_Solexa_Mi2008_1_14470_hit10
5' GCAGC-CCGACGUAAUCAGA
   |||:| |||||||||||||
   CGUUGCGGCUGCAUUAGUCU 5'
AT4G12570.1     329     348
polyubiquitin-like protein

sRNA_AGO1_Solexa_Mi2008_1_34211_hit3
5' UGAAAGAGAGAUGAGAGCUUU
     ||||| ||||||||||||
   UAUUUCU-UCUACUCUCGAAA 5'
AT4G14130.1     18      37
xyloglucan endotransglycosylase-related protein XTR-7

sRNA_AGO1_Solexa_Mi2008_1_41698_hit1
5' UGGGAAAGGA-CAGCCUAGCUUC
   |||||||||| | ||| ||||||
   ACCCUUUCCUUG-CGGCUCGAAG 5'
AT4G14130.1     601     622
xyloglucan endotransglycosylase-related protein XTR-7

sRNA_AGO1_Solexa_Mi2008_4_11303_hit6
5' CUCUUGGGGAAAGGAAUUCGC
   ||||| ||||||||||  |||
   GAGAAACCCUUUCCUU--GCG 5'
AT4G14130.1     609     627
xyloglucan endotransglycosylase-related protein XTR-7

sRNA_AGO1_Solexa_Mi2008_16_13755_hit1
5' GACGAUAAGCAUUGGCAUU
   :|||||||||||||:||||
   UUGCUAUUCGUAACUGUAA 5'
AT4G14365.1     281     299
C3HC4-type zinc finger ankyrin repeat protein - like

sRNA_AGO1_Solexa_Mi2008_1_166_hit3
5' AAAAGAAACAGAAGAGAAUGCU
   ||||||||||| |||:||| ||
   UUUUCUUUGUC-UCUUUUA-GA 5'
AT4G14880.2     1255    1274
cytosolic O-acetylserine(thiol)lyase (EC 4.2.99.8)

sRNA_AGO1_Solexa_Mi2008_1_166_hit3
5' AAAAGAAACAGAAGAGAAUGCU
   ||||||||||| |||:||| ||
   UUUUCUUUGUC-UCUUUUA-GA 5'
AT4G14880.1     1258    1277
cytosolic O-acetylserine(thiol)lyase (EC 4.2.99.8)

sRNA_AGO1_Solexa_Mi2008_2_7120_hit1
5' CAUGAAUUUGAGGUUUACAG
   ||| ||:||||||||||||
   GUA-UUGAACUCCAAAUGUG 5'
```

```
AT4G16000.1     323     341
unknown protein

sRNA_AGO1_Solexa_Mi2008_1_29469_hit1
5' UCCAGCGGCUAUUAGGAUGGG
   ||||| |||||||||| |||
   AGGUCCCCGAUAAUCCCACCA 5'
AT4G16370.1     1711    1731
isp4 like protein

sRNA_AGO1_Solexa_Mi2008_1_29468_hit1
5' UCCAGCGGCUAUUAGGAUGG
   ||||| |||||||||| |||
   AGGUCCCCGAUAAUCCCACC 5'
AT4G16370.1     1712    1731
isp4 like protein

sRNA_AGO1_Solexa_Mi2008_12_20785_hit2
5' UAGAAAGGGCAAUAUACACGUG
   |||||||||||| | ||| |||
   AUCUUUCCCGUU-U-UGUACAC 5'
AT4G16370.1     2273    2292
isp4 like protein

sRNA_AGO1_Solexa_Mi2008_1_26506_hit1
5' UAUU-U-UAGUGUUCAGAAGA
   |||| | | ||||||||||||
   AUAACAGAGCACAAGUCUUCU 5'
AT4G17470.1     995     1015
putative protein

sRNA_AGO1_Solexa_Mi2008_5_2843_hit1
5' AUCCGCCGUUACCGCCGCC
     |||||||:|||||||||
   GCGGCGGCAGUGGCGGCGG 5'
AT4G17500.1     491     509
ethylene responsive element binding factor 1 (frameshift !)

sRNA_AGO1_Solexa_Mi2008_1_34614_hit1
5' UGAAGAUGAAGUCAUCAUUG
   |||||||:||||||| ||||
   ACUUCUAUUUCAGUACUAAC 5'
AT4G18360.2     352     371
glycolate oxidase - like protein

sRNA_AGO1_Solexa_Mi2008_2_56179_hit1
5' UUUGUUACUGGAGAACGUUUCU
   ||||| | ||||||||||| ||
   AAACACUAACCUCUUGCAA-GA 5'
AT4G19170.1     166     186
neoxanthin cleavage enzyme-like protein

sRNA_AGO1_Solexa_Mi2008_1_336_hit2
5' AAAGAAAAAAA-AAGUAUUGA
   ||||||||||| || |||| |
   UUUCUUUUUUUAUU-AUAAGU 5'
AT4G19170.1     1996    2015
neoxanthin cleavage enzyme-like protein

sRNA_AGO1_Solexa_Mi2008_2_52173_hit1
5' UUGGAGUGUCGUGGGUUUGUU
    ||||||||  ||||||||||
   CACCUCACAAAACCCAAACAA 5'
```

```
AT4G19170.1     211     231
neoxanthin cleavage enzyme-like protein

sRNA_AGO1_Solexa_Mi2008_1_45665_hit1
5' UGUG-UGAAGAGAGAAUGAUGG
   |||| ||||||||||||| |||
   ACACUACUUCUCUCUUAC-ACC 5'
AT4G19170.1     84      104
neoxanthin cleavage enzyme-like protein

sRNA_AGO1_Solexa_Mi2008_1_53254_hit1
5' UUGGUUUGGGUUUUGUU-CUAA
   ||||||||||| ||||| |||
   AACCAAACCCA-AACAAUGAUA 5'
AT4G19430.1     143     163
unknown protein

sRNA_AGO1_Solexa_Mi2008_1_14875_hit1
5' GGAGGUGGAGGCGGUGG-UGGU
   : ||||||||||||||| ||||
   UGUCCACCUCCGCCACCAACCA 5'
AT4G22470.1     207     228
extensin - like protein

sRNA_AGO1_Solexa_Mi2008_2_43577_hit1
5' UGGUGGUGGUGACG-UUGGUGGU
   |||||||||||| | ||| ||||
   ACCACCACCACU-CUAACAACCA 5'
AT4G22470.1     778     799
extensin - like protein

sRNA_AGO1_Solexa_Mi2008_1_43578_hit1
5' UGGUGGUGGUGAUGAUGUGUC
   |||||||||||| ||| |||
   ACCACCACCACU-CUA-ACAA 5'
AT4G22470.1     781     799
extensin - like protein

sRNA_AGO1_Solexa_Mi2008_1_7157_hit1
5' CAUGAUGAUGAUGGAACUGGU
    |||||||||| |||| ||||
   CUACUACUACUCCCUUUACCA 5'
AT4G22490.1     57      77
RCc3- like protein

sRNA_AGO1_Solexa_Mi2008_1_3079_hit472
5' AUGAUGAUGAUGAUGAUGAUGA
    |||||| || |||||||||||
   GACUACUCCUCCUACUACUACU 5'
AT4G22490.1     67      88
RCc3- like protein

sRNA_AGO1_Solexa_Mi2008_1_45036_hit1
5' UGUGAUGAGGAGGAUGAUGAA
     ||||||||||||||||||
   CGACUACUCCUCCUACUACUA 5'
AT4G22490.1     69      89
RCc3- like protein

sRNA_AGO1_Solexa_Mi2008_1_50495_hit1
5' UUGAGACCCGACGCGAUGGC
   |||||||||||| |||||
   AACUCUGGGCUG-GCUACGA 5'
```

AT4G25000.1   760   778
alpha-amylase like protein

sRNA_AGO1_Solexa_Mi2008_1_9617_hit1
```
5' CUAAGAAACAACGAGGUGUCU
   |||||||||||| || || ||
   GAUUCUUUGUUG-UCGAC-GA 5'
```
AT4G27080.1   45   63
unknown protein

sRNA_AGO1_Solexa_Mi2008_1_14399_hit1
```
5' GCAAAAUGCGAAUCGAUGG
   | ||||||||||| |||||
   CCUUUUACGCUUACCUACC 5'
```
AT4G27080.1   747   765
unknown protein

sRNA_AGO1_Solexa_Mi2008_1_50088_hit1
```
5' UUGAAGGAUCAUCAGUGUGAAA
   |||||||||||| |||| |||
   AACUUCCUAGUA-UCACUCUUG 5'
```
AT4G27710.1   1520   1540
cytochrome P450 - like protein

sRNA_AGO1_Solexa_Mi2008_7_8338_hit9
```
5' CGAAGG-UGCAUAGUGAGAA
    ||||| | |||||||||||
   UCUUCCUA-GUAUCACUCUU 5'
```
AT4G27710.1   1521   1539
cytochrome P450 - like protein

sRNA_AGO1_Solexa_Mi2008_1_1573_hit9
```
5' ACGAAGG-UGCAUAGUGAGAA
   | ||||| | |||||||||||
   UUCUUCCUA-GUAUCACUCUU 5'
```
AT4G27710.1   1521   1540
cytochrome P450 - like protein

sRNA_AGO1_Solexa_Mi2008_1_50087_hit1
```
5' UUGAAGGAUCAUCAGUGUGAA
   |||||||||||| |||| |||
   AACUUCCUAGUA-UCACUCUU 5'
```
AT4G27710.1   1521   1540
cytochrome P450 - like protein

sRNA_AGO1_Solexa_Mi2008_1_15358_hit4
```
5' GUAAAUGGGAUGGAGAACAAA
    |||||||:|| |||||||||
   AAUUUACCUUAGCUCUUGUUU 5'
```
AT4G27810.1   100   120
hypothetical protein

sRNA_AGO1_Solexa_Mi2008_1_43726_hit3
```
5' UGGUUAAGGAGAUAGACUUGA
   ||||| || |||||||||||
   ACCAAAUC-UCUAUCUGAACC 5'
```
AT4G28490.1   86   105
receptor-like protein kinase 5 precursor (RLK5)

sRNA_AGO1_Solexa_Mi2008_3_36318_hit1
```
5' UGAGGAUGACUAUGGUGAUGAG
   ||| |||||| ||||||||||
   ACUACUACUGUUACCACUACUA 5'
```

```
AT4G28530.2     798     819
NAM / CUC2 -like protein

sRNA_AGO1_Solexa_Mi2008_3_36318_hit1
5' UGAGGAUGACUAUGGUGAUGAG
   ||| |||||| ||||||||||
   ACUACUACUGUUACCACUACUA 5'
AT4G28530.1     909     930
NAM / CUC2 -like protein

sRNA_AGO1_Solexa_Mi2008_1_5420_hit1
5' CACCAAGCAAUAUCGUUUG
   ||||||||| |||||||
   GUGGUUCGUGAUAGCAACA 5'
AT4G28610.1     468     486
unknown protein

sRNA_AGO1_Solexa_Mi2008_1_20963_hit5
5' UAGAA-UAGAAAGUACAUAUAGA
   ||||| ||||||||| ||| |||
   AUCUUUAUCUUUCAUCUAU-UCU 5'
AT4G29030.1     32      53
glycine-rich protein like

sRNA_AGO1_Solexa_Mi2008_6_19010_hit1
5' UACAAAAGAAGGUGA-AAUGCA
   ||||||||||||:|| | ||||
   AUGUUUUCUUCCGCUGUCACGU 5'
AT4G29360.1     1850    1871
beta-1,3-glucanase-like protein

sRNA_AGO1_Solexa_Mi2008_6_19010_hit1
5' UACAAAAGAAGGUGA-AAUGCA
   ||||||||||||:|| | ||||
   AUGUUUUCUUCCGCUGUCACGU 5'
AT4G29360.2     1941    1962
beta-1,3-glucanase-like protein

sRNA_AGO1_Solexa_Mi2008_5_29024_hit1
5' UCAU-GGUUUCAAGAAUGCU
   :||| |||||||||||:|||
   GGUAACCAAAGUUCUUGCGA 5'
AT4G29360.2     765     784
beta-1,3-glucanase-like protein

sRNA_AGO1_Solexa_Mi2008_1_55382_hit1
5' UUUGCAUAGGGUGUGUUGUGAC
    |||||||| ||||| ||||||
   UAACGUAUCACACACUACACUG 5'
AT4G29700.1     1637    1658
nucleotide pyrophosphatase -like protein

sRNA_AGO1_Solexa_Mi2008_5_2929_hit1
5' AUCUAUUGAACAUCGUGUUU
    ||| || ||||||||||||
   GAGAGAA-UUGUAGCACAAA 5'
AT4G29780.1     869     887
unknown protein

sRNA_AGO1_Solexa_Mi2008_16_13022_hit1
5' CUUGGUGGCUUAGUGGGCUAA
   ||||||||||:||| ||||||
   GAACCACCGAGUCA-CCGAUU 5'
```

```
AT4G29900.1     2680    2699

sRNA_AGO1_Solexa_Mi2008_3_5873_hit1
5' CAGAAAAGAGGUAAACGAG
    |||||||||||||||| :
   CUCUUUUCUCCAUUUGCAU 5'
AT4G29900.1     3272    3290

sRNA_AGO1_Solexa_Mi2008_5_4358_hit1
5' CAACAGAAGAAUUUCAAACAG
   ||||||||||| |:||||||
   GUUGUCUUCUUUAGGUUUGUG 5'
AT4G30430.1     1009    1029
senescence-associated protein homolog

sRNA_AGO1_Solexa_Mi2008_3_13542_hit2
5' GAAGUUUUGAAGAUUUGCAGA
   |||| |||||||||||| |||
   CUUCUAAACUUCUAAACCUCU 5'
AT4G30430.1     25      45
senescence-associated protein homolog

sRNA_AGO1_Solexa_Mi2008_3_20883_hit1
5' UAGAAGAAUGGUGAUGUACGUG
   ||||||||||| |||| || ||
   AUCUUCUUACCCCUAC-UG-AC 5'
AT4G31500.1     1124    1143
cytochrome P450 monooxygenase (CYP83B1)

sRNA_AGO1_Solexa_Mi2008_1_32802_hit1
5' UCUACCAGCAGAAACGUCCUA
   |||||||||||||||||||||
   AGAUGGUCGUCUUUGCAGGAU 5'
AT4G31500.1     1554    1574
cytochrome P450 monooxygenase (CYP83B1)

sRNA_AGO1_Solexa_Mi2008_1_27836_hit1
5' UCA-CGGUGUCUGAUUGAUCG
   ||| |||||:||||||||||:
   AGUUGCCACGGACUAACUAGU 5'
AT4G31500.1     522     542
cytochrome P450 monooxygenase (CYP83B1)

sRNA_AGO1_Solexa_Mi2008_1_7340_hit1
5' CAUGUAAAACACUCUCUUU
   ||||||| | ||||||||:
   GUACAUU-U-UGAGAGAAG 5'
AT4G31620.1     1039    1055

sRNA_AGO1_Solexa_Mi2008_1_37209_hit1
5' UGAUGAGUGUAUGAUUCGCCA
   |||| |||| |||||||||||
   ACUA-UCACCUACUAAGCGGU 5'
AT4G31620.1     25      44

sRNA_AGO1_Solexa_Mi2008_9_14254_hit8
5' GAUGAUGAUGAUGAUGAUCUU
   |||||||||||||||||||||
   CUACUACUACUACUACUAGAA 5'
AT4G31800.2     474     494

sRNA_AGO1_Solexa_Mi2008_1_36323_hit13
5' UGAGGAUGAUGAUGAUGAUGA
```

```
   ||| ||||||||||||||
   ACUACUACUACUACUACUAGA 5'
AT4G31800.2     475     495

sRNA_AGO1_Solexa_Mi2008_1_3079_hit472
5' AUGAUGAUGAUGAUGAUGA
    ||||||||||||||||||
   GACUACUACUACUACUACUAGA 5'
AT4G31800.2     475     496

sRNA_AGO1_Solexa_Mi2008_1_45039_hit3
5' UG-UGAUGAUGAUGAUGAUGA
   || ||||||||||||||||||
   ACGACUACUACUACUACUACUAGA 5'
AT4G31800.2     476     499

sRNA_AGO1_Solexa_Mi2008_9_14254_hit8
5' GAUGAUGAUGAUGAUGAUCUU
   |||||||||||||||||||||
   CUACUACUACUACUACUAGAA 5'
AT4G31800.1     477     497

sRNA_AGO1_Solexa_Mi2008_1_38921_hit1
5' UGCGGACGAUGAUGAUGAUGAU
   ||| || ||||||||||||||
   ACGACUACUACUACUACUACUA 5'
AT4G31800.2     477     498

sRNA_AGO1_Solexa_Mi2008_7_3078_hit1
5' AUGAUGAUGA-GAAUGAUGAU
    ||||||||| || |||||||
   CACUACUACUACU-ACUACUA 5'
AT4G31800.2     478     497

sRNA_AGO1_Solexa_Mi2008_1_36323_hit13
5' UGAGGAUGAUGAUGAUGAUGA
   ||| ||||||||||||||
   ACUACUACUACUACUACUAGA 5'
AT4G31800.1     478     498

sRNA_AGO1_Solexa_Mi2008_1_3079_hit472
5' AUGAUGAUGAUGAUGAUGA
    ||||||||||||||||||
   GACUACUACUACUACUACUAGA 5'
AT4G31800.1     478     499

sRNA_AGO1_Solexa_Mi2008_3_39351_hit1
5' UGCUGAUGAUGCUGAUG-UGAC
   ||||||||||| ||||| |||
   ACGACUACUACUACUACUACUA 5'
AT4G31800.2     478     499

sRNA_AGO1_Solexa_Mi2008_1_56092_hit3
5' UUUG-UGAUGAUGAUGAUGAUGA
   :||| |||||||||||||||||
   GAACGACUACUACUACUACUACU 5'
AT4G31800.2     479     501

sRNA_AGO1_Solexa_Mi2008_1_45039_hit3
5' UGU-GAUGAUGAUGAUGAUGAUGA
   ||| | |||||||||||||||||
   ACAACGACUACUACUACUACUACU 5'
AT4G31800.2     479     502
```

```
sRNA_AGO1_Solexa_Mi2008_1_38921_hit1
5' UGCGGACGAUGAUGAUGAUGAU
   ||| || ||||||||||||||
   ACGACUACUACUACUACUACUA 5'
AT4G31800.1     480     501

sRNA_AGO1_Solexa_Mi2008_6_14255_hit369
5' GAUGAUGAUGAUGAUGAUGAUGAU
    ||| || ||||||||||||||||
   GUACAACGACUACUACUACUACUA 5'
AT4G31800.2     480     503

sRNA_AGO1_Solexa_Mi2008_7_3078_hit1
5' AUGAUGAUGA-GAAUGAUGAU
    ||||||||| || |||||||
   CACUACUACUACU-ACUACUA 5'
AT4G31800.1     481     500

sRNA_AGO1_Solexa_Mi2008_2_31619_hit1
5' UCGCUGAUGAUGAUUGAUGAU
     |||||||||||| ||||||
   UUCGACUACUACUA-CUACUA 5'
AT4G31800.2     481     500

sRNA_AGO1_Solexa_Mi2008_1_3079_hit472
5' AUGAUGAUGAUGAUGAUGAUGA
   ||| || ||||||||||||||
   UACAACGACUACUACUACUACU 5'
AT4G31800.2     481     502

sRNA_AGO1_Solexa_Mi2008_3_39351_hit1
5' UGCUGAUGAUGCUGAUG-UGAC
   ||||||||||| ||||| |||
   ACGACUACUACUACUACUACUA 5'
AT4G31800.1     481     502

sRNA_AGO1_Solexa_Mi2008_1_56092_hit3
5' UUUG-UGAUGAUGAUGAUGAUGA
   :||| ||||||||||||||||||
   GAACGACUACUACUACUACUACU 5'
AT4G31800.1     482     504

sRNA_AGO1_Solexa_Mi2008_1_45039_hit3
5' UGU-GAUGAUGAUGAUGAUGAUGA
   ||| | ||||||||||||||||||
   ACAACGACUACUACUACUACUACU 5'
AT4G31800.1     482     505

sRNA_AGO1_Solexa_Mi2008_6_14255_hit369
5' GAUGAUGAUGAUGAUGAUGAUGAU
    ||| || ||||||||||||||||
   GUACAACGACUACUACUACUACUA 5'
AT4G31800.1     483     506

sRNA_AGO1_Solexa_Mi2008_2_31619_hit1
5' UCGCUGAUGAUGAUUGAUGAU
     ||||||||||| |||||||
   UUCGACUACUACU-ACUACUA 5'
AT4G31800.1     484     503

sRNA_AGO1_Solexa_Mi2008_1_3079_hit472
5' AUGAUGAUGAUGAUGAUGAUGA
```

```
   ||| || |||||||||||||||
   UACAACGACUACUACUACUACU 5'
AT4G31800.1     484     505

sRNA_AGO1_Solexa_Mi2008_1_33805_hit1
5' UC-UGUUGCUGUUGAUGUUGAU
   || |||||||| ||||| ||||
   AGUACAACGACUACUACUACUA 5'
AT4G31800.2     484     505

sRNA_AGO1_Solexa_Mi2008_1_33805_hit1
5' UC-UGUUGCUGUUGAUGUUGAU
   || |||||||| ||||| ||||
   AGUACAACGACUACUACUACUA 5'
AT4G31800.1     487     508

sRNA_AGO1_Solexa_Mi2008_1_33062_hit1
5' UCUCAACGGCUCGGAAAUGGA
   |||||||||||||||||||||
   AGAGUUGCCGAGCCUUUACCU 5'
AT4G32020.1     30      50
unknown protein

sRNA_AGO1_Solexa_Mi2008_1_5354_hit1
5' CACAG-CUUUCUUGAACUGC
   | ||| ||||||||||| ||
   GGGUCCGAAAGAACUUG-CG 5'
AT4G32250.1     1123    1141
unknown protein

sRNA_AGO1_Solexa_Mi2008_1_5354_hit1
5' CACAG-CUUUCUUGAACUGC
   | ||| ||||||||||| ||
   GGGUCCGAAAGAACUUG-CG 5'
AT4G32250.3     1190    1208
unknown protein

sRNA_AGO1_Solexa_Mi2008_1_5354_hit1
5' CACAG-CUUUCUUGAACUGC
   | ||| ||||||||||| ||
   GGGUCCGAAAGAACUUG-CG 5'
AT4G32250.2     1278    1296
unknown protein

sRNA_AGO1_Solexa_Mi2008_2_13776_hit2
5' GAC-G-GAAGAAAGUUAGAGU
   ||| | ||||||||||||||
   CUGACUCUUCUUUCAAUCUCC 5'
AT4G32250.1     591     611
unknown protein

sRNA_AGO1_Solexa_Mi2008_2_13776_hit2
5' GAC-G-GAAGAAAGUUAGAGU
   ||| | ||||||||||||||
   CUGACUCUUCUUUCAAUCUCC 5'
AT4G32250.3     658     678
unknown protein

sRNA_AGO1_Solexa_Mi2008_2_13776_hit2
5' GAC-G-GAAGAAAGUUAGAGU
   ||| | ||||||||||||||
   CUGACUCUUCUUUCAAUCUCC 5'
AT4G32250.2     746     766
```

```
unknown protein

sRNA_AGO1_Solexa_Mi2008_2_50423_hit1
5' UUGACUACAAAAGAUGGAGCA
    ||||| ||| ||||||||||
   CACUGAGGUUGUCUACCUCGU 5'
AT4G34110.1     2112    2132
poly(A)-binding protein

sRNA_AGO1_Solexa_Mi2008_67_35933_hit1
5' UGAGAGUGAUCCGUGUG-UGUU
   |||||||||||| | || ||||
   ACUCUCACUAGGGAAACGACAA 5'
AT4G34540.1     2576    2597
isoflavone reductase - like protein

sRNA_AGO1_Solexa_Mi2008_6_33555_hit1
5' UCUGCUCGGUGCUUUAGGGUC
   |||||||||||||||||||||
   AGACGAGCCACGAAAUCCCAG 5'
AT4G34710.2     2279    2299
arginine decarboxylase (spe2)

sRNA_AGO1_Solexa_Mi2008_6_33555_hit1
5' UCUGCUCGGUGCUUUAGGGUC
   |||||||||||||||||||||
   AGACGAGCCACGAAAUCCCAG 5'
AT4G34710.1     2414    2434
arginine decarboxylase (spe2)

sRNA_AGO1_Solexa_Mi2008_1_2984_hit1
5' AUGACAACAACCAACCACACGU
   ||| ||||||| |||||||||
   UAC-GUUGUUGUUUGGUGUGCU 5'
AT4G34710.2     2534    2554
arginine decarboxylase (spe2)

sRNA_AGO1_Solexa_Mi2008_1_2984_hit1
5' AUGACAACAACCAACCACACGU
   ||| ||||||| |||||||||
   UAC-GUUGUUGUUUGGUGUGCU 5'
AT4G34710.1     2669    2689
arginine decarboxylase (spe2)

sRNA_AGO1_Solexa_Mi2008_1_34418_hit4
5' UGAA-CCUCAGGCGACCAAG
   |||| |||| ||||||||||
   ACUUCGGAGGCCGCUGGUUC 5'
AT4G34970.1     352     371
actin depolymerizing factor - like protein

sRNA_AGO1_Solexa_Mi2008_1_41556_hit1
5' UGGCUAACGACAAAGAUGUG
   :|||| | ||||||||||||
   GCCGACU-CUGUUUCUACAC 5'
AT4G35190.1     627     645
putative protein

sRNA_AGO1_Solexa_Mi2008_4_36955_hit4
5' UGAUAAUU-CAGGUUGUGCAUUC
   ||||| || |||||||||||||
   ACUAUAAAAGUCCAACACGUAAA 5'
AT4G35190.1     959     981
```

```
putative protein

sRNA_AGO1_Solexa_Mi2008_1_46232_hit15
5' UUAAACUAUGUUUCAG-G-AUA
   ||||||||||||| || | |||
   AAUUUGAUACAAAAUCUCUUAU 5'
AT4G35770.1     763     784
senescence-associated protein sen1

sRNA_AGO1_Solexa_Mi2008_1_46232_hit15
5' UUAAACUAUGUUUCAG-G-AUA
   ||||||||||||| || | |||
   AAUUUGAUACAAAAUCUCUUAU 5'
AT4G35770.3     767     788
senescence-associated protein sen1

sRNA_AGO1_Solexa_Mi2008_11_51755_hit1
5' UUGCGGUGUUCGAAGUUCGGC
   ||| ||||||||||||||||
   AACACCACAAGCUUCAAGCCA 5'
AT4G35770.1     813     833
senescence-associated protein sen1

sRNA_AGO1_Solexa_Mi2008_1_51754_hit1
5' UUGCGGUGUUCGAAGUUCGG
   ||| ||||||||||||||||
   AACACCACAAGCUUCAAGCC 5'
AT4G35770.1     814     833
senescence-associated protein sen1

sRNA_AGO1_Solexa_Mi2008_11_51755_hit1
5' UUGCGGUGUUCGAAGUUCGGC
   ||| ||||||||||||||||
   AACACCACAAGCUUCAAGCCA 5'
AT4G35770.3     817     837
senescence-associated protein sen1

sRNA_AGO1_Solexa_Mi2008_1_51754_hit1
5' UUGCGGUGUUCGAAGUUCGG
   ||| ||||||||||||||||
   AACACCACAAGCUUCAAGCC 5'
AT4G35770.3     818     837
senescence-associated protein sen1

sRNA_AGO1_Solexa_Mi2008_3_7026_hit4
5' CAUCCC-GUCCUUAAUUGGUCC
   |||||| ||||||||||||||
   GUAGGGGCAGGAAUUAACCAGG 5'
AT4G35770.1     842     863
senescence-associated protein sen1

sRNA_AGO1_Solexa_Mi2008_1_46232_hit15
5' UUAAACUAUGUUUCAG-G-AUA
   ||||||||||||| || | |||
   AAUUUGAUACAAAAUCUCUUAU 5'
AT4G35770.2     843     864
senescence-associated protein sen1

sRNA_AGO1_Solexa_Mi2008_3_7026_hit4
5' CAUCCC-GUCCUUAAUUGGUCC
   |||||| ||||||||||||||
   GUAGGGGCAGGAAUUAACCAGG 5'
AT4G35770.3     846     867
```

```
senescence-associated protein sen1

sRNA_AGO1_Solexa_Mi2008_11_51755_hit1
5' UUGCGGUGUUCGAAGUUCGGC
   ||| ||||||||||||||||
   AACACCACAAGCUUCAAGCCA 5'
AT4G35770.2     893     913
senescence-associated protein sen1

sRNA_AGO1_Solexa_Mi2008_1_51754_hit1
5' UUGCGGUGUUCGAAGUUCGG
   ||| ||||||||||||||||
   AACACCACAAGCUUCAAGCC 5'
AT4G35770.2     894     913
senescence-associated protein sen1

sRNA_AGO1_Solexa_Mi2008_3_7026_hit4
5' CAUCCC-GUCCUUAAUUGGUCC
   |||||| |||||||||||||||
   GUAGGGGCAGGAAUUAACCAGG 5'
AT4G35770.2     922     943
senescence-associated protein sen1

sRNA_AGO1_Solexa_Mi2008_2_16158_hit3
5' GUUGAUGUUGAGCCAAAAA
    ||||||||||||||||
   AAACUACAACUCGGUUUAG 5'
AT4G36850.1     896     914
unknown protein

sRNA_AGO1_Solexa_Mi2008_13_10755_hit1
5' CUCAGUUCGGGCGAGGCAU
   |||||||| ||||| ||||
   GAGUCAAGGCCGCU-CGUA 5'
AT4G37430.1     1024    1041
cytochrome P450 monooxygenase (CYP91A2)

sRNA_AGO1_Solexa_Mi2008_4_44164_hit1
5' UGUAGCAACGGUUGUGGUGGU
   |||| |||||||||| |||||
   ACAUGGUUGCCAACA-CACCA 5'
AT4G37430.1     455     474
cytochrome P450 monooxygenase (CYP91A2)

sRNA_AGO1_Solexa_Mi2008_2_44633_hit3
5' UGUCCG-AGGAGUGGUCGAGGG
   ||| || ||||| |||||||||
   ACACGCCUCCUCUCCAGCUCCC 5'
AT4G37520.1     198     219
peroxidase, prxr2

sRNA_AGO1_Solexa_Mi2008_1_36855_hit2
5' UGAGUGUUGUGGUCAAAUGGG
     ||||||||||||| |||||
   UGUCACAACACCAGUGUACCC 5'
AT4G38540.1     692     712
monooxygenase 2 (MO2)

sRNA_AGO1_Solexa_Mi2008_1_43581_hit1
5' UGGUGGUGGUGGU-AAUGGUC
   ||||||||||||| |||||:
   ACCACCACCACCACUUACCGA 5'
AT4G38550.1     1017    1037
```

```
Phospholipase like protein

sRNA_AGO1_Solexa_Mi2008_1_30443_hit1
5' UCGA-AGAUGGUAAUGGAGUCAG
   |||| ||||: ||||||||||||
   AGCUAUCUAU-AUUACCUCAGUC 5'
AT4G38550.1     6       27
Phospholipase like protein

sRNA_AGO1_Solexa_Mi2008_1_55562_hit1
5' UUUGGAGCAAAAACGU-UUUA
   |||||||||||||| | ||||
   AAACCUCGUUUUUG-AGAAAU 5'
AT4G39940.1     1154    1173
adenosine-5'-phosphosulfate-kinase

sRNA_AGO1_Solexa_Mi2008_1_31918_hit1
5' UCGGCCGGUUGAUGGAUUGU-UG
   ||||| | |||||||||||| ||
   AGCCG-CAAACUACCUAACAGAC 5'
AT4G39950.1     1078    1099
cytochrome P450 like protein

sRNA_AGO1_Solexa_Mi2008_1_53083_hit1
5' UUGG-UGGUGUUCCUCACUGUG
   |||| |||||||||||| |||
   AACCUACCACAAGGAGU-ACAA 5'
AT5G02500.2     1378    1398
dnaK-type molecular chaperone hsc70.1

sRNA_AGO1_Solexa_Mi2008_1_53083_hit1
5' UUGG-UGGUGUUCCUCACUGUG
   |||| |||||||||||| |||
   AACCUACCACAAGGAGU-ACAA 5'
AT5G02500.1     1768    1788
dnaK-type molecular chaperone hsc70.1

sRNA_AGO1_Solexa_Mi2008_1_5228_hit1
5' CAA-UUGGGGAAGAUGUUGUU
    || ||||||||||||||||:
   UUUGAACCCCUUCUACAACAG 5'
AT5G02760.1     391     411
protein phosphatase - like protein

sRNA_AGO1_Solexa_Mi2008_9_14254_hit8
5' GAUGAUGAUGAUGAUGAUC-UU
   ||||||||||||||||||| ||
   CUACUACUACUACUACUAGUAA 5'
AT5G03545.1     292     313
unknown protein

sRNA_AGO1_Solexa_Mi2008_6_14255_hit369
5' GAUGAUGAUGAUGAUGAUGAUGAU
   |||||||||||||||||||| ||
   CUACUACUACUACUACUACUAGUA 5'
AT5G03545.1     292     315
unknown protein

sRNA_AGO1_Solexa_Mi2008_1_14256_hit8
5' GAUGAUGAUGAUGAUGAUGAUGUU
   ||||||||||||||||||||  |
   CUACUACUACUACUACUACUAGUA 5'
AT5G03545.1     292     315
```

```
unknown protein

sRNA_AGO1_Solexa_Mi2008_1_36323_hit13
5' UGAGGAUGAUGAUGAUGAUGA
   ||| ||||||||||||||| |
   ACUACUACUACUACUACUAGU 5'
AT5G03545.1     293     313
unknown protein

sRNA_AGO1_Solexa_Mi2008_1_3079_hit472
5' AUGAUGAUGAUGAUGAUGAUGA
   |||||||||||||||||||| |
   UACUACUACUACUACUACUAGU 5'
AT5G03545.1     293     314
unknown protein

sRNA_AGO1_Solexa_Mi2008_1_45039_hit3
5' UG-UGAUGAUGAUGAUGAUGAUGA
   || ||||||||||||||||||| |
   ACUACUACUACUACUACUACUAGU 5'
AT5G03545.1     294     317
unknown protein

sRNA_AGO1_Solexa_Mi2008_9_14254_hit8
5' GAUGAUGAUGAUGAUGAUCUU
   ||||||||||||||||||  |
   CUACUACUACUACUACUACUA 5'
AT5G03545.1     295     315
unknown protein

sRNA_AGO1_Solexa_Mi2008_6_14255_hit369
5' GAUGAUGAUGAUGAUGAUGAUGAU
   :|||||||||||||||||||||||
   UUACUACUACUACUACUACUACUA 5'
AT5G03545.1     295     318
unknown protein

sRNA_AGO1_Solexa_Mi2008_1_14256_hit8
5' GAUGAUGAUGAUGAUGAUGAUGUU
   :||||||||||||||||||||| |
   UUACUACUACUACUACUACUACUA 5'
AT5G03545.1     295     318
unknown protein

sRNA_AGO1_Solexa_Mi2008_7_3078_hit1
5' AUGAUGAUGA-GAAUGAUGAU
   |||||||||| | ||||||||
   UACUACUACUAC-UACUACUA 5'
AT5G03545.1     296     315
unknown protein

sRNA_AGO1_Solexa_Mi2008_1_36323_hit13
5' UGAGGAUGAUGAUGAUGAUGA
   ||| |||||||||||||||||
   ACUACUACUACUACUACUACU 5'
AT5G03545.1     296     316
unknown protein

sRNA_AGO1_Solexa_Mi2008_1_3079_hit472
5' AUGAUGAUGAUGAUGAUGAUGA
   ||||||||||||||||||||||
   UACUACUACUACUACUACUACU 5'
AT5G03545.1     296     317
```

```
unknown protein

sRNA_AGO1_Solexa_Mi2008_1_45039_hit3
5' UGUGAUGAUGAUGAUGAUGAUGA
    :||||||||||||||||||||
   UUACUACUACUACUACUACUACU 5'
AT5G03545.1     296     318
unknown protein

sRNA_AGO1_Solexa_Mi2008_1_56092_hit3
5' UUUG-UGAUGAUGAUGAUGAUGA
   |||| |||||||||||||||||
   AAACUACUACUACUACUACUACU 5'
AT5G03545.1     297     319
unknown protein

sRNA_AGO1_Solexa_Mi2008_6_14255_hit369
5' GAUGAUGAUGAUGAUGAUGAUGAU
   |||:||||||||||||||||||||
   CUAUUACUACUACUACUACUACUA 5'
AT5G03545.1     298     321
unknown protein

sRNA_AGO1_Solexa_Mi2008_1_14256_hit8
5' GAUGAUGAUGAUGAUGAUGAUGUU
   |||:|||||||||||||||||| |
   CUAUUACUACUACUACUACUACUA 5'
AT5G03545.1     298     321
unknown protein

sRNA_AGO1_Solexa_Mi2008_7_3078_hit1
5' AUGAUGAUGA-GAAUGAUGAU
   |||||||||| || |||||||
   UACUACUACUACU-ACUACUA 5'
AT5G03545.1     299     318
unknown protein

sRNA_AGO1_Solexa_Mi2008_1_3079_hit472
5' AUGAUGAUGAUGAUGAUGAUGA
     |||:||||||||||||||||
   ACCUAUUACUACUACUACUACU 5'
AT5G03545.1     302     323
unknown protein

sRNA_AGO1_Solexa_Mi2008_4_14169_hit1
5' GAUAAUGAUGAUGAAAGAUGA
   ||||||||||||||  |||||
   CUAUUACUACUACUA-CUACU 5'
AT5G03545.1     303     322
unknown protein

sRNA_AGO1_Solexa_Mi2008_1_39699_hit1
5' UGGA-ACUGAUGAUGAUGAUGA
   |||| | ||||||||||||||
   ACCUAUUACUACUACUACUACU 5'
AT5G03545.1     303     324
unknown protein

sRNA_AGO1_Solexa_Mi2008_1_45039_hit3
5' UGUGAUGAUGAUGAUGAUGAUGA
   || |||:||||||||||||||||
   AC-CUAUUACUACUACUACUACU 5'
AT5G03545.1     303     324
```

```
unknown protein

sRNA_AGO1_Solexa_Mi2008_1_52789_hit1
5' UUGGGUGAUGAUGAUGAUUGAU
   ||||:|:||||||||||| |||
   AACCUAUUACUACUACUA-CUA 5'
AT5G03545.1     305     325
unknown protein

sRNA_AGO1_Solexa_Mi2008_1_2280_hit1
5' AGUGGAUGAUGAUGAUGAUG
   | |||||:||||||||||||
   UAACCUAUUACUACUACUAC 5'
AT5G03545.1     306     325
unknown protein

sRNA_AGO1_Solexa_Mi2008_1_56092_hit3
5' UUUGUGAUGAUGAUGAUGAUGA
   |||| |||:||||||||||||
   AAAC-CUAUUACUACUACUACU 5'
AT5G03545.1     306     326
unknown protein

sRNA_AGO1_Solexa_Mi2008_1_7106_hit1
5' CAUGAAGGGCAAGGCGCGU-AU
    ||||||||||||||:||| ||
   CUACUUCCCGUUCCGUGCACUA 5'
AT5G04200.1     646     667
latex-abundant protein - like

sRNA_AGO1_Solexa_Mi2008_1_7082_hit4
5' CAUCUCCAGGA-ACCUCUUGAU
   | ||||||||| || |||||||
   GGAGAGGUCCUAUG-AGAACUA 5'
AT5G05320.1     800     820
monooxygenase

sRNA_AGO1_Solexa_Mi2008_1_646_hit1
5' AACAGAAAAAAAACA-UGAU
   ||||:|||||||||| |||
   UUGUUUUUUUUUUGUAACUU 5'
AT5G05340.1     1138    1157
peroxidase

sRNA_AGO1_Solexa_Mi2008_1_21951_hit1
5' UAGAUUUACAGUUGAUUGUUU
   ||||:||| |||||||||||
   AUCUGAAUCUCAACUAACAAC 5'
AT5G05340.1     5       25
peroxidase

sRNA_AGO1_Solexa_Mi2008_13_14779_hit6
5' GCUGAGAAUCGAAAU-AGUU
    || ||||||||||| ||||
   AGA-UCUUAGCUUUAUUCAA 5'
AT5G05340.1     70      88
peroxidase

sRNA_AGO1_Solexa_Mi2008_2_45191_hit1
5' UGUGCGU-UGGUUUCAGGAUGGA
   |||| || ||||||| |||||||
   ACACCCACACCAAAGCCCUACCU 5'
AT5G06370.1     372     394
```

```
unknown protein

sRNA_AGO1_Solexa_Mi2008_7_41670_hit11
5' UGGCUUGCCAAUGGAGGAUCC
   ||||| ||||||||||||||
   ACCGA-CGGUUACCUCCUAGU 5'
AT5G06370.1     918     937
unknown protein

sRNA_AGO1_Solexa_Mi2008_1_29050_hit1
5' UCAUG-UGGGUUGGAUUGAUA
    |||| |||||||||||| ||
   CGUACCACCCAACCUAAC-AU 5'
AT5G06860.1     350     369
polygalacturonase inhibiting protein 1; PGIP1 (gb|AAF69827.1)

sRNA_AGO1_Solexa_Mi2008_6_20075_hit1
5' UACGAGCCACUGGAAACUGAA
   || ||| | ||||||||||||
   AU-CUCUG-GACCUUUGACUU 5'
AT5G07580.1     1013    1031
transcription factor-like protein (emb|CAB87947.1)

sRNA_AGO1_Solexa_Mi2008_1_39180_hit4
5' UGCUAGUGAUGAUGGUACAAU
   ||| ||||||||||| ||||
   ACGUUCACUACUACCUUGUUU 5'
AT5G07580.1     178     198
transcription factor-like protein (emb|CAB87947.1)

sRNA_AGO1_Solexa_Mi2008_1_28254_hit1
5' UCAGCAAGUGAUGAUGGAAC
   || |||||||||||||||||
   AGACGUUCACUACUACCUUG 5'
AT5G07580.1     181     200
transcription factor-like protein (emb|CAB87947.1)

sRNA_AGO1_Solexa_Mi2008_9_14254_hit8
5' GAUGAUGAUGAUGAUGAUCUU
   |||||||||||||||||| ::
   CUACUACUACUACUACUAAGG 5'
AT5G07580.1     437     457
transcription factor-like protein (emb|CAB87947.1)

sRNA_AGO1_Solexa_Mi2008_1_36323_hit13
5' UGAGGAUGAUGAUGAUGAUGA
   ||| |||||||||||||||
   ACUACUACUACUACUACUAAG 5'
AT5G07580.1     438     458
transcription factor-like protein (emb|CAB87947.1)

sRNA_AGO1_Solexa_Mi2008_1_3079_hit472
5' AUGAUGAUGAUGAUGAUGAUGA
    |||||||||||||||||||
   CACUACUACUACUACUACUAAG 5'
AT5G07580.1     438     459
transcription factor-like protein (emb|CAB87947.1)

sRNA_AGO1_Solexa_Mi2008_1_45039_hit3
5' UGUGAUGAUGAUGAUGAUGAUGA
    ||||||||||||||||||||
   CCACUACUACUACUACUACUAAG 5'
AT5G07580.1     438     460
```

```
transcription factor-like protein (emb|CAB87947.1)

sRNA_AGO1_Solexa_Mi2008_7_3078_hit1
5' AUGAUGAUGA-GAAUGAUGAU
    ||||||||| || |||||||
   GACUACUACUACU-ACUACUA 5'
AT5G07580.1     441     460
transcription factor-like protein (emb|CAB87947.1)

sRNA_AGO1_Solexa_Mi2008_1_38921_hit1
5' UGCG-GACGAUGAUGAUGAUGAU
   || | || ||||||||||||||
   AC-CACUACUACUACUACUACUA 5'
AT5G07580.1     441     462
transcription factor-like protein (emb|CAB87947.1)

sRNA_AGO1_Solexa_Mi2008_1_41920_hit17
5' UGG-GAUGAUGAAGUUGAUGAU
   ||| |||||||| | |||||||
   ACCACUACUACUACUACUACUA 5'
AT5G07580.1     441     462
transcription factor-like protein (emb|CAB87947.1)

sRNA_AGO1_Solexa_Mi2008_1_56092_hit3
5' UUUGUGAUGAUGAUGAUGAUGA
   :| ||||||||||||||||||
   GACCACUACUACUACUACUACU 5'
AT5G07580.1     441     462
transcription factor-like protein (emb|CAB87947.1)

sRNA_AGO1_Solexa_Mi2008_1_36323_hit13
5' UGAG-GAUGAUGAUGAUGAUGA
   || | ||||||||||||||||
   AC-CACUACUACUACUACUACU 5'
AT5G07580.1     442     462
transcription factor-like protein (emb|CAB87947.1)

sRNA_AGO1_Solexa_Mi2008_1_45039_hit3
5' UGUGAUGAUGAUGAUGAUGAUGA
   || | |||||||||||||||||
   AC-C-ACUACUACUACUACUACU 5'
AT5G07580.1     442     462
transcription factor-like protein (emb|CAB87947.1)

sRNA_AGO1_Solexa_Mi2008_1_2280_hit1
5' AGUGGAUGAUGAUGAUGAUG
    ||| |||||||||||||||
   GCAC-UACUACUACUACUAC 5'
AT5G07580.1     443     461
transcription factor-like protein (emb|CAB87947.1)

sRNA_AGO1_Solexa_Mi2008_4_42709_hit1
5' UGGGUGAUGAUGAUGAUUGAU
    :|||||||||||||| |||
   CUCCACUACUACUACUA-CUA 5'
AT5G07580.1     444     463
transcription factor-like protein (emb|CAB87947.1)

sRNA_AGO1_Solexa_Mi2008_4_11252_hit1
5' CUC-UGAGUGUGUGAUCAACUU
   ||| ||||| ||||||||| ||
   GAGUACUCAGACACUAGUU-AA 5'
AT5G07580.1     6       26
```

```
transcription factor-like protein (emb|CAB87947.1)

sRNA_AGO1_Solexa_Mi2008_1_34613_hit1
5' UGAAGAUGAAGAUGAGUUGU
    ||||||||||||||:||||:
   CCUUCUACUUCUACUUAACG 5'
AT5G07580.1     811     830
transcription factor-like protein (emb|CAB87947.1)

sRNA_AGO1_Solexa_Mi2008_339_11394_hit3
5' CUGAAGUGUUUGGGGGAACUC
    |||||||||||||:|||||
   AACUUCACAAACCCUCUUGAA 5'
AT5G10180.1     124     144
sulfate transporter

sRNA_AGO1_Solexa_Mi2008_1_22796_hit1
5' UAGGAUAUUGAUAUAGUGU
   |||||||||||| ||||:|
   AUCCUAUAACUA-AUCAUA 5'
AT5G10180.1     1712    1729
sulfate transporter

sRNA_AGO1_Solexa_Mi2008_1_4581_hit2
5' CAAGA-AUACUACAGCCAU-GGUC
   ||||| ||||||||||||: ||||
   GUUCUCUAUGAUGUCGGUGGCCAG 5'
AT5G10180.1     590     613
sulfate transporter

sRNA_AGO1_Solexa_Mi2008_6_50680_hit1
5' UUGAGGCAAAGAACAUCCGAA
    ||||| |||||||||||||
   CACUCC-UUUCUUGUAGGCUC 5'
AT5G10180.1     931     950
sulfate transporter

sRNA_AGO1_Solexa_Mi2008_1_20849_hit1
5' UAGAACCU-CGGUCGAGAAUG-GU
   ||| |||| |||||||||||| ||
   AUCGUGGAAGCCAGCUCUUACUCA 5'
AT5G10760.1     1046    1069
nucleoid DNA-binding protein cnd41 - like protein

sRNA_AGO1_Solexa_Mi2008_1_4400_hit1
5' CAACAUUUAGCGUCGUCUGC
   ||||||:|||||||||||||
   GUUGUAGAUCGCAGCAGACG 5'
AT5G10760.1     1367    1386
nucleoid DNA-binding protein cnd41 - like protein

sRNA_AGO1_Solexa_Mi2008_1_44071_hit1
5' UGUACGGAAGUUUUGGUGGCGA
   || ||||||||  |||||||||
   AC-UGCCUUCACUACCACCGCU 5'
AT5G11420.1     149     169
unknown protein

sRNA_AGO1_Solexa_Mi2008_1_3917_hit1
5' CAAAAGAAGCAA-AAGUUUGUU
   ||||||||| || || ||||||
   GUUUUCUUC-UUCUUAAAACAA 5'
AT5G13080.1     50      70
```

```
WRKY-like protein

sRNA_AGO1_Solexa_Mi2008_1_17162_hit1
5' UAACA-AGAGGAAGAAGAACGAU
   ||||| ||||||||||||| ||
   AUUGUCUCUCCUUCUUCUU-CUU 5'
AT5G13180.1     24      45
NAM-like protein

sRNA_AGO1_Solexa_Mi2008_1_14_hit1
5' AAAAAAACAGAGAACAAGAAGA
    ||||||||||||  |||||||
   CUUUUUUGUCUCUCCUUCUUCU 5'
AT5G13180.1     27      48
NAM-like protein

sRNA_AGO1_Solexa_Mi2008_1_341_hit1
5' AAAGAAACAGAGAGGAAGAU
   ||| |||||||||||||||
   UUU-UUUGUCUCUCCUUCUU 5'
AT5G13180.1     30      48
NAM-like protein

sRNA_AGO1_Solexa_Mi2008_3_10681_hit1
5' CUCACGAUUUGAUUUCCUCU
   |: ||||||||||||||||| 
   GGUUGCUAAACUAAAGGAGA 5'
AT5G13180.1     383     402
NAM-like protein

sRNA_AGO1_Solexa_Mi2008_12_14067_hit2
5' GAGG-A-UCCAUUGGAGGGCA
   |||| | |||||||:||||||
   CUCCGUUAGGUAACUUCCCGU 5'
AT5G13190.1     462     482
unknown protein

sRNA_AGO1_Solexa_Mi2008_1_22432_hit10
5' UAGCUAAAAAACCACCAUCGAG
   :||||||||||||||||  |||
   GUCGAUUUUUUGGUGGUU-CUC 5'
AT5G13740.1     817     837
transporter-like protein

sRNA_AGO1_Solexa_Mi2008_1_40339_hit1
5' UGGAGAUGGAAGAUGAG-CC
   |||||||||||| |||| ||
   ACCUCUACCUUCCACUCUGG 5'
AT5G13930.1     876     895
chalcone synthase (naringenin-chalcone synthase) (testa 4 protein) (sp|P13114)

sRNA_AGO1_Solexa_Mi2008_2_2279_hit1
5' AGUG-GAAAUCAGUAAUUUA
   |||| |||||||||||||
   UCACACUUUAGUCAUUAACC 5'
AT5G14780.1     29      48
formate dehydrogenase (FDH)

sRNA_AGO1_Solexa_Mi2008_2_22965_hit1
5' UAGG-CGUAAAGAUCCGGGAUG
   |||| ||| ||||||||||||:
   AUCCUGCACUUCUAGGCCCUAU 5'
AT5G14780.1     376     397
```

```
formate dehydrogenase (FDH)

sRNA_AGO1_Solexa_Mi2008_1_35784_hit2
5' UGAGAAGAGAUAGAAUAGA
   |||||||||||| ||||
   ACUCUUCUCUAU-UUAUAG 5'
AT5G14780.1     80      97
formate dehydrogenase (FDH)

sRNA_AGO1_Solexa_Mi2008_10_49988_hit1
5' UUGAACAAUUGAUGUUG-UGCU
   |:|||||| |||||||| ||||
   AGCUUGUUUACUACAACCACGA 5'
AT5G17380.1     1544    1565
2-hydroxyphytanoyl-CoA lyase-like protein

sRNA_AGO1_Solexa_Mi2008_3_4962_hit3
5' CAAGUAAUAACAUUUAACCU
   ||| |||||||||||| ||
   GUU-AUUAUUGUAAAU-GGU 5'
AT5G17380.1     1681    1698
2-hydroxyphytanoyl-CoA lyase-like protein

sRNA_AGO1_Solexa_Mi2008_2_56_hit1
5' AAAAAAGAGGAAGAUUGGU
       ||||||||||||||||
   CAAUUUCUCCUUCUAACCA 5'
AT5G18170.1     13      31
glutamate dehydrogenase (EC 1.4.1.-) 1 (pir||S71217)

sRNA_AGO1_Solexa_Mi2008_1_22493_hit1
5' UAGCUCCUGAU-GGUCGAGUA
   ||||||||||| |||| |||
   AUCGAGGACUACCCAGAUCAG 5'
AT5G18470.1     778     798
unknown protein

sRNA_AGO1_Solexa_Mi2008_1_54615_hit4
5' UUUAUUG-GGUACACAAAUAA
   ||||:|| ||||||||||||
   AAAUGACACCAUGUGUUUAUA 5'
AT5G23010.1     1621    1641
2-isopropylmalate synthase-like; homocitrate synthase-like

sRNA_AGO1_Solexa_Mi2008_2_255_hit1
5' AAA-AUAGUGUGGGAA-UGUUA
   ||| |||||||||||| ||||
   UUUCUAUCACACCCUUAACAAA 5'
AT5G23010.1     5       26
2-isopropylmalate synthase-like; homocitrate synthase-like

sRNA_AGO1_Solexa_Mi2008_9_13465_hit1
5' GAAGAUGG-GUGGGAAUUGUUU
    |||||:| |||||||||||||
   AUUCUAUCACACCCUUAACAAA 5'
AT5G23010.1     5       26
2-isopropylmalate synthase-like; homocitrate synthase-like

sRNA_AGO1_Solexa_Mi2008_1_45985_hit1
5' UGUUGAUGUUGUUGUCGUUAG
   ||||| ||||||||| ||||:
   ACAACAACAACAACAACAAUU 5'
AT5G23280.1     53      73
```

```
unknown protein

sRNA_AGO1_Solexa_Mi2008_1_51621_hit1
5' UUGCCUUGAUGUUGAUGUUGU
   |||| | |||| |||||||||
   AACG-A-CUACUACUACAACA 5'
AT5G23280.1     625     643
unknown protein

sRNA_AGO1_Solexa_Mi2008_1_53198_hit1
5' UUGGUUGC-GAUGAUGAUGUUG
   |||||||| ||||||||||||
   AACCAACGACUACUACUACAAC 5'
AT5G23280.1     626     647
unknown protein

sRNA_AGO1_Solexa_Mi2008_1_38236_hit3
5' UGCAUCU-GUUGUUGGUGUUGC
   |||| || ||||||| ||||||
   ACGUCGAACAACAACGACAACG 5'
AT5G23280.1     661     682
unknown protein

sRNA_AGO1_Solexa_Mi2008_1_37145_hit2
5' UGAUCUUUGGUUCGCGGGUC
    |||||||||||| |||||
   CCUAGAAACCAAG-GCCCAA 5'
AT5G23660.1     105     123
MtN3-like protein

sRNA_AGO1_Solexa_Mi2008_6_37144_hit2
5' UGAUCUUUGGUUCGCGGGU
    |||||||||||| |||||
   CCUAGAAACCAAG-GCCCA 5'
AT5G23660.1     106     123
MtN3-like protein

sRNA_AGO1_Solexa_Mi2008_3_43372_hit2
5' UGGU-GAUCUUUGGUUCGCGGG
   |:|| |||||||||||| ||||
   AUCAGCUAGAAACCAAG-GCCC 5'
AT5G23660.1     107     127
MtN3-like protein

sRNA_AGO1_Solexa_Mi2008_1_27384_hit1
5' UCAA-UAGGAAAAUAUUUACA
   |||| | |||||||||||| |
   AGUUGAACCUUUUAUAAAUAU 5'
AT5G23660.1     1226    1246
MtN3-like protein

sRNA_AGO1_Solexa_Mi2008_1_629_hit1
5' AACAAAGAUGGUGAUGGGUAU
   ||||||||||| |||  ||||
   UUGUUUCUACCUCUA--CAUA 5'
AT5G23660.1     387     405
MtN3-like protein

sRNA_AGO1_Solexa_Mi2008_1_33058_hit1
5' UCUCAACCAAAUCCGACGGCGU
   |||||||||||||||||||||
   AGAGUUGGUUUAGGCUGCCGCA 5'
AT5G23660.1     783     804
```

MtN3-like protein

sRNA_AGO1_Solexa_Mi2008_3_5851_hit1
5' CACUUCUGGCAAUUUCGCAGC
   |||||||||||||||||||||
   GUGAAGACCGUUAAAGCGUCG 5'
AT5G23660.1   816   836
MtN3-like protein

sRNA_AGO1_Solexa_Mi2008_1_14213_hit1
5' GAUCGGACGACGGUGAUCGCUA
   ||||||||||||||||||||||
   CUAGCCUGCUGCCACUAGCGAU 5'
AT5G23660.1   883   904
MtN3-like protein

sRNA_AGO1_Solexa_Mi2008_28_46962_hit1
5' UUACAUGUGUUCACCGAUCGGA
   ||||||||||||||||||||||
   AAUGUACACAAGUGGCUAGCCU 5'
AT5G23660.1   898   919
MtN3-like protein

sRNA_AGO1_Solexa_Mi2008_31_31178_hit1
5' UCGAUCGUUACAGUUACAUGUGUU
   ||||||||||||||||||||||||
   AGCUAGCAAUGUCAAUGUACACAA 5'
AT5G23660.1   909   932
MtN3-like protein

sRNA_AGO1_Solexa_Mi2008_1_28297_hit1
5' UCAGCAUUUCGAUCGUUACAGU
   ||||||||||||||||||||||
   AGUCGUAAAGCUAGCAAUGUCA 5'
AT5G23660.1   919   940
MtN3-like protein

sRNA_AGO1_Solexa_Mi2008_1_34056_hit1
5' UGAAAAACGGAUGAGUGAUUUAAG
   ||||||||||||||||||||||||
   ACUUUUUGCCUACUCACUAAAUUC 5'
AT5G25110.1   145   168
serine/threonine protein kinase-like protein

sRNA_AGO1_Solexa_Mi2008_1_23143_hit1
5' UAGGGGCCGAUUGAUAGCGACUGU
   ||||||||||||| ||:|||||||
   AUCCCCGGCUAACCAUUGCUGACA 5'
AT5G25110.1   174   197
serine/threonine protein kinase-like protein

sRNA_AGO1_Solexa_Mi2008_1_23146_hit1
5' UAGGGGCUGAUUGGUAGCGAGUGU
   |||||||:||||||||:||| |||
   AUCCCCGGCUAACCAUUGCUGACA 5'
AT5G25110.1   174   197
serine/threonine protein kinase-like protein

sRNA_AGO1_Solexa_Mi2008_17_8110_hit2
5' CCGUGGGUU-GUUUUAUAAGAA
    ||||| || ||||||||||||
   AGCACCAAAUCAAAAUAUUCUU 5'
AT5G25110.1   17   38

serine/threonine protein kinase-like protein

```
sRNA_AGO1_Solexa_Mi2008_1_4569_hit2
5' CAAGAAGAAGGUUGG-UUAGUU
   ||||||||||| ||| ||||||
   GUUCUUCUUCC-ACCAAAUCAA 5'
AT5G25110.1     26      46
serine/threonine protein kinase-like protein

sRNA_AGO1_Solexa_Mi2008_1_36886_hit1
5' UGAG-UUGAUGAUUAGGAAG-GA
   |||| |||||||:||||||| ||
   ACUCUAACUACUGAUCCUUCUCU 5'
AT5G25340.1     145     167
unknown protein

sRNA_AGO1_Solexa_Mi2008_2_8432_hit1
5' CGACCGUUAGAAGAAGCGCGG
   |||||||||||||||||||||
   GCUGGCAAUCUUCUUCGCGCC 5'
AT5G26340.1     706     726
hexose transporter - like protein

sRNA_AGO1_Solexa_Mi2008_22_7409_hit1
5' CA-UGUUGACCAAUUCAAGA
   || |||||||||||| ||||
   GUGACAACUGGUUAAAUUCU 5'
AT5G26770.2     110     129
unknown protein

sRNA_AGO1_Solexa_Mi2008_2_131_hit1
5' AAAA-AUAUAGUUCAGGGUU
    ||| ||| |||||||||||
   GUUUGUAUCUCAAGUCCCAA 5'
AT5G26770.3     128     147
unknown protein

sRNA_AGO1_Solexa_Mi2008_2_131_hit1
5' AAAA-AUAUAGUUCAGGGUU
    ||| ||| |||||||||||
   GUUUGUAUCUCAAGUCCCAA 5'
AT5G26770.2     29      48
unknown protein

sRNA_AGO1_Solexa_Mi2008_2_6120_hit1
5' CAGAUACAAAACGUAAAGAU
    ||| ||||| |||||||||
   AUCU-UGUUUAGCAUUUCUA 5'
AT5G26770.2     70      88
unknown protein

sRNA_AGO1_Solexa_Mi2008_1_21732_hit1
5' UAGAUCACAAUGG-UGUAUGC
    |||||||||||| | |||||
   CUCUAGUGUUACCUA-AUACG 5'
AT5G35980.2     1298    1317
protein kinase-like

sRNA_AGO1_Solexa_Mi2008_2_34753_hit1
5' UGAAGGAUCGAGGUCGAGGCAC
   ||| |||| ||||||||||||
   ACU-CCUA-CUCCAGCUCCGUA 5'
AT5G35980.1     3026    3045
```

```
protein kinase-like

sRNA_AGO1_Solexa_Mi2008_157_34752_hit1
5' UGAAGGAUCGAGGUCGAGGCA
   ||| |||| |||||||||||
   ACU-CCUA-CUCCAGCUCCGU 5'
AT5G35980.1     3027    3045
protein kinase-like

sRNA_AGO1_Solexa_Mi2008_3_51268_hit1
5' UUGAUUGGGAGCCUAUCUCGC
   |||||||||||| ||||||
   AACUAACCCUCGUAUAGAGGA 5'
AT5G35980.1     3060    3080
protein kinase-like

sRNA_AGO1_Solexa_Mi2008_1_34620_hit1
5' UGAAGAUGAUGAUGAAGUU
   ||||||||||||||| |||
   ACUUCUACUACUACU-CAA 5'
AT5G35980.2     54      71
protein kinase-like

sRNA_AGO1_Solexa_Mi2008_1_50630_hit7
5' UUGAGGAAGAAGAUGAGGAUGAU
   :||||||||||||||| |||||
   GACUCCUUCUUCUACUACUACUC 5'
AT5G35980.2     55      77
protein kinase-like

sRNA_AGO1_Solexa_Mi2008_1_36323_hit13
5' UGAGGAUGAUGAUGAUGAUGA
   |||||| || |||||||||||
   ACUCCUUCUUCUACUACUACU 5'
AT5G35980.2     56      76
protein kinase-like

sRNA_AGO1_Solexa_Mi2008_2_40489_hit1
5' UGGAGGACAAGCUAGUGAUCGU
   |||||||||||| |:||||||
   ACCUCCUGUUCGUUUACUAGCU 5'
AT5G36220.1     847     868
cytochrome P450 monooxygenase (CYP81D1 )

sRNA_AGO1_Solexa_Mi2008_2_35253_hit1
5' UGACAGACUGAUGUGUUAGA
   |||| | ||||||||||||
   ACUGAC-GACUACACAAUCA 5'
AT5G36220.1     940     958
cytochrome P450 monooxygenase (CYP81D1 )

sRNA_AGO1_Solexa_Mi2008_1_51898_hit5
5' UUGCUUUCUUGAUGGAUACUCA
   |||||||||||||| || |||
   AACGAAAGAACUAC-UA-GAGC 5'
AT5G37600.1     974     993
glutamate--ammonia ligase

sRNA_AGO1_Solexa_Mi2008_13_4545_hit1
5' CAAGAAAGAUGGUUGUCGUU
     |||||| |||||||||||
   ACUCUUUCAACCAACAGCAA 5'
AT5G39050.1     534     553
```

```
Anthocyanin acyltransferase - like protein

sRNA_AGO1_Solexa_Mi2008_1_13420_hit2
5' GAAGAAGAAGAAGACUCUU
   || ||||||||||||||||
   CUCCUUCUUCUUCUGAGAA 5'
AT5G39050.1     830     848
Anthocyanin acyltransferase - like protein

sRNA_AGO1_Solexa_Mi2008_1_4090_hit1
5' CA-AAGAGUAGACAAUCAUCAA
   || |||||||:||||||| |||
   GUAUUCUCAUUUGUUAGU-GUU 5'
AT5G39050.1     888     908
Anthocyanin acyltransferase - like protein

sRNA_AGO1_Solexa_Mi2008_10440_13909_hit2
5' GAGAAGCAGGGCACGUGCA
   |||||||||| ||||||
   CUCUUCGUCCAGUGCACUC 5'
AT5G39610.1     769     787
NAM / CUC2 - like protein

sRNA_AGO1_Solexa_Mi2008_110_13911_hit1
5' GAGAAGCAGGGCACGUGCG
   |||||||||| |||||| |
   CUCUUCGUCCAGUGCACUC 5'
AT5G39610.1     769     787
NAM / CUC2 - like protein

sRNA_AGO1_Solexa_Mi2008_41322_14841_hit2
5' GGAGAAGCAGGGCACGUGCA
   ||||||||||| ||||||
   CCUCUUCGUCCAGUGCACUC 5'
AT5G39610.1     769     788
NAM / CUC2 - like protein

sRNA_AGO1_Solexa_Mi2008_422_14845_hit1
5' GGAGAAGCAGGGCACGUGCG
   ||||||||||| |||||| |
   CCUCUUCGUCCAGUGCACUC 5'
AT5G39610.1     769     788
NAM / CUC2 - like protein

sRNA_AGO1_Solexa_Mi2008_3_40192_hit1
5' UGGAGAAGCAGGGCACGU-AGA
    ||||||||||| ||||| |||
   CCCUCUUCGUCCAGUGCACUCU 5'
AT5G39610.1     769     790
NAM / CUC2 - like protein

sRNA_AGO1_Solexa_Mi2008_23_14840_hit3
5' GGAGAAGCAGGGCACGUGC
   ||||||||||| ||||||
   CCUCUUCGUCCAGUGCACU 5'
AT5G39610.1     770     788
NAM / CUC2 - like protein

sRNA_AGO1_Solexa_Mi2008_404_40194_hit3
5' UGGAGAAGCAGGGCACGUGC
   :||||||||||| ||||||
   GCCUCUUCGUCCAGUGCACU 5'
AT5G39610.1     770     789
```

```
NAM / CUC2 - like protein

sRNA_AGO1_Solexa_Mi2008_9_40189_hit2
5' UGGAGAAGCAGGGCACGUA
   :||||||||||| |||||
   GCCUCUUCGUCCAGUGCAC 5'
AT5G39610.1     771     789
NAM / CUC2 - like protein

sRNA_AGO1_Solexa_Mi2008_366_40193_hit3
5' UGGAGAAGCAGGGCACGUG
   :||||||||||| ||||||
   GCCUCUUCGUCCAGUGCAC 5'
AT5G39610.1     771     789
NAM / CUC2 - like protein

sRNA_AGO1_Solexa_Mi2008_2_3231_hit2
5' AUGGAGAAGCAGGGCACGUG
    :||||||||||| ||||||
   AGCCUCUUCGUCCAGUGCAC 5'
AT5G39610.1     771     790
NAM / CUC2 - like protein

sRNA_AGO1_Solexa_Mi2008_1_40200_hit1
5' UGGAGAAGCAGGGU-ACGUG
    ||||||||| ||| |||||
   CCCUCUUCGU-CCAGUGCAC 5'
AT5G39610.1     772     790
NAM / CUC2 - like protein

sRNA_AGO1_Solexa_Mi2008_1_2655_hit2
5' AUAGUGCUGAAACAUGA-GAU
   |||||| |||||||||| ||
   UAUCACAACUUUGUACUUCUC 5'
AT5G40670.1     957     977
unknown protein

sRNA_AGO1_Solexa_Mi2008_1_3_hit25
5' AAAAAAAAAAAAAGAAAGA
   ||||||||||||||| |||
   UUUUUUUUUUUUUCUAUCU 5'
AT5G41400.1     644     662
RING zinc finger protein-like

sRNA_AGO1_Solexa_Mi2008_1_3_hit25
5' AAAAAAAAAAAAAGAAAGA
   ||||||||||||| |||||
   UUUUUUUUUUUUU-UUUCU 5'
AT5G41400.1     649     666
RING zinc finger protein-like

sRNA_AGO1_Solexa_Mi2008_1_1776_hit10
5' AGAA-AAAAAAAAAAAAAAAU
   | || |||||||||||||||
   UAUUGUUUUUUUUUUUUUUUC 5'
AT5G41400.1     650     671
RING zinc finger protein-like

sRNA_AGO1_Solexa_Mi2008_1_46486_hit1
5' UUAAGAAGUGACUG-AUGCACA
   |||| ||||||||| |||| ||
   AAUUAUUCACUGACUUACG-GU 5'
AT5G42080.2     1117    1137
```

```
dynamin-like protein (pir||S59558)

sRNA_AGO1_Solexa_Mi2008_1_46486_hit1
5' UUAAGAAGUGACUG-AUGCACA
   |||| ||||||||| |||| ||
   AAUUAUUCACUGACUUACG-GU 5'
AT5G42080.1     1120    1140
dynamin-like protein (pir||S59558)

sRNA_AGO1_Solexa_Mi2008_12_11898_hit1
5' CUGGAGACAAUGGAAGACU
    ||||||||||:|||||||
   UACCUCUGUUAUCUUCUGA 5'
AT5G42080.2     1451    1469
dynamin-like protein (pir||S59558)

sRNA_AGO1_Solexa_Mi2008_12_11898_hit1
5' CUGGAGACAAUGGAAGACU
    ||||||||||:|||||||
   UACCUCUGUUAUCUUCUGA 5'
AT5G42080.1     1454    1472
dynamin-like protein (pir||S59558)

sRNA_AGO1_Solexa_Mi2008_1_32995_hit1
5' UCUAUCGCCGGAUUUAUGUCA
   |||||||||| | || |||||
   AGAUAGCGGCGU-AA-ACAGU 5'
AT5G42080.1     1590    1608
dynamin-like protein (pir||S59558)

sRNA_AGO1_Solexa_Mi2008_2_14498_hit1
5' GCCAAAGAAAUGGACGAGG
     ||||||||||| |||||
   GCGUUUCUUUACCAGCUCC 5'
AT5G42080.1     1887    1905
dynamin-like protein (pir||S59558)

sRNA_AGO1_Solexa_Mi2008_1_25234_hit1
5' UAUGAACUUGAUGACCCGCCC
   |||||||||||||||||||||
   AUACUUGAACUACUGGGCGGG 5'
AT5G42650.1     1051    1071
allene oxide synthase (emb|CAA73184.1)

sRNA_AGO1_Solexa_Mi2008_1_15261_hit5
5' GGGUCGAGUGAUGUGAUUGAG
   :|| |||||| ||||||||||
   UCC-GCUCACAACACUAACUC 5'
AT5G42650.1     4       23
allene oxide synthase (emb|CAA73184.1)

sRNA_AGO1_Solexa_Mi2008_2_13457_hit1
5' GAAGAUGAAGAUGAAGUGGUA
   ||||| ||||||||| || ||
   CUUCUUCUUCUACUU-AC-AU 5'
AT5G43060.1     17      35
cysteine protease component of protease-inhibitor complex

sRNA_AGO1_Solexa_Mi2008_1_42997_hit306
5' UGGUCA-AA-AUGUGUCACAAUC
   |||| | || |||||||||||||
   ACCAAUAUUCUACACAGUGUUAG 5'
AT5G43060.1     621     643
```

```
cysteine protease component of protease-inhibitor complex

sRNA_AGO1_Solexa_Mi2008_1_27432_hit1
5' UCAAUGAUGGCC-CUGUUUGUU
   |||||||||||| | ||||||
   AGUUACUACCGGAG-CAAACAC 5'
AT5G43830.1     387     407
aluminum-induced protein-like

sRNA_AGO1_Solexa_Mi2008_3_30917_hit1
5' UCGAGUCAUGUGAUCGAGUGGU
   ||| | |||||||||:||||||
   AGC-CUGUACACUAGUUCACCA 5'
AT5G44680.1     1105    1125
unknown protein

sRNA_AGO1_Solexa_Mi2008_1_1904_hit1
5' AGAGAUUAGGGAGAUUGGU-UU
   |||||||||:||:|||||| ||
   UCUCUAAUCUCUUUAACCAGAA 5'
AT5G44680.1     269     290
unknown protein

sRNA_AGO1_Solexa_Mi2008_3_29136_hit1
5' UCAU-UG-GUGUGAUGAUGUGUU
   |||| || ||||||||||| |||
   AGUAGACACACACUACUACCCAA 5'
AT5G45340.2     806     828
cytochrome P450

sRNA_AGO1_Solexa_Mi2008_7_1062_hit1
5' AAUGAACAGUUGAUGAUA-CA
   ||||||||||||| |||| ||
   UUACUUGUCAACUUCUAUAGU 5'
AT5G46330.1     1413    1433
receptor protein kinase

sRNA_AGO1_Solexa_Mi2008_1_20517_hit1
5' UACUCCUAAAUGUG-CGUUGGA
   |||||||||| | | |||||||
   AUGAGGAUUUCC-CAGCAACCU 5'
AT5G46330.1     190     210
receptor protein kinase

sRNA_AGO1_Solexa_Mi2008_6_16103_hit1
5' GUUCAAGAGAUUGCCAGAAUCA
   |||||||||||| |||:||||
   CAAGUUCUCUAAAGGUUUUAGA 5'
AT5G46330.1     767     788
receptor protein kinase

sRNA_AGO1_Solexa_Mi2008_2_6120_hit1
5' CAGAUAC-AAAACGUAAAGAU
    |||| | |||||||||||||
   AUCUA-GCUUUUGCAUUUCUA 5'
AT5G48180.1     214     233
unknown protein

sRNA_AGO1_Solexa_Mi2008_1_29127_hit1
5' UCAUUGGGCUCAGUAUCAUA
   ||||||| ||||||||||||
   AGUAACCAGAGUCAUAGUAU 5'
AT5G48180.1     374     393
```

```
unknown protein

sRNA_AGO1_Solexa_Mi2008_3_56550_hit1
5' UUUU-GGGUGCGAUUUUGGUUA
   |||| |||| ||||||:|||||
   AAAACCCCA-GCUAAAGCCAAU 5'
AT5G48540.1     464     484
33 kDa secretory protein-like

sRNA_AGO1_Solexa_Mi2008_1_27863_hit1
5' UCACUAAU-GACAAUGAGUGUC
   ||| |||| ||||||||||||
   AGU-AUUAUCUGUUACUCACAA 5'
AT5G50160.1     1170    1190
FRO1 and FRO2-like protein

sRNA_AGO1_Solexa_Mi2008_1_53198_hit1
5' UUGGUUGCGAUGAUGAUGUUG
   ||| ||| |||||||||||||
   AAC-AAC-CUACUACUACAAC 5'
AT5G50160.1     296     314
FRO1 and FRO2-like protein

sRNA_AGO1_Solexa_Mi2008_1_21052_hit1
5' UAGACCAAACCUGUGAUAGU
   |||||||||||| || |||:
   AUCUGGUUUGGA-AC-AUCG 5'
AT5G50160.1     527     544
FRO1 and FRO2-like protein

sRNA_AGO1_Solexa_Mi2008_1_13614_hit8
5' GACAGAAGAGAGAGAGCAC
   || |||||||||||| ||
   CU-UCUUCUCUCUCU-GUA 5'
AT5G50160.1     556     572
FRO1 and FRO2-like protein

sRNA_AGO1_Solexa_Mi2008_2_8245_hit1
5' CGAAAAGAAGAAGAUGAUGAG
   |||||||||||||| || |||
   GCUUUUCUUCUUCU-CU-CUC 5'
AT5G50160.1     560     578
FRO1 and FRO2-like protein

sRNA_AGO1_Solexa_Mi2008_3_3428_hit1
5' AUGGUUUUUGACAGGCAG-GAA
   ||||||||||||||| || ||
   UACCAAAAACUGUCC-UCACUG 5'
AT5G50260.1     700     720
cysteine endopeptidase

sRNA_AGO1_Solexa_Mi2008_1_98_hit1
5' AA-AAAAUUGUUGCACUUGUU
   || ||||| ||||||||||||
   UUGUUUUA-CAACGUGAACAA 5'
AT5G52390.1     1003    1022
photoassimilate-responsive protein PAR-like protein

sRNA_AGO1_Solexa_Mi2008_2_41008_hit1
5' UG-GAUUCCAACGAUGCGGUCC
   || |||||||||:|||| ||||
   ACUCUAAGGUUGUUACG-CAGG 5'
AT5G52390.1     382     402
```

```
photoassimilate-responsive protein PAR-like protein

sRNA_AGO1_Solexa_Mi2008_4_55164_hit2
5' UUUGAGCUC-AGGUGGUAGUCA
   ||||||||| | |||||| |||
   AAACUCGAGCU-CACCAUAAGU 5'
AT5G53450.2     497     517
unknown protein

sRNA_AGO1_Solexa_Mi2008_4_55164_hit2
5' UUUGAGCUC-AGGUGGUAGUCA
   ||||||||| | |||||| |||
   AAACUCGAGCU-CACCAUAAGU 5'
AT5G53450.1     566     586
unknown protein

sRNA_AGO1_Solexa_Mi2008_1_13489_hit1
5' GAAGGAAGAAAGUUAGAGU
   |||| |||||||||||||
   CUUC-UUCUUUCAAUCUCC 5'
AT5G53550.1     1595    1612
EspB-like protein

sRNA_AGO1_Solexa_Mi2008_2_56650_hit2
5' UUUUUCAGGAAUG-GUUGAU
   :|||||||||| | ||||||
   GAAAAGUCCUU-CUCAACUA 5'
AT5G53550.1     970     988
EspB-like protein

sRNA_AGO1_Solexa_Mi2008_1_3901_hit3
5' CAAAACGAAGAAGAUGAUGAG
   | |||||| |||||||||||
   GGUUUGCUCCUUCUACUACUC 5'
AT5G53730.1     214     234
putative protein

sRNA_AGO1_Solexa_Mi2008_1_37225_hit4
5' UGAUGAUGAUGAUGAUGAAGAAG
   ||| ||||||||||||||||||
   ACU-CUACUACUACUACUUCUUC 5'
AT5G53730.1     68      89
putative protein

sRNA_AGO1_Solexa_Mi2008_1_13759_hit1
5' GACGAUGAUGAUGAUGACA
   || ||||||||||||||
   CU-CUACUACUACUACUUC 5'
AT5G53730.1     71      88
putative protein

sRNA_AGO1_Solexa_Mi2008_5_13760_hit2
5' GACGAUGAUGAUGAUGAGC
   || ||||||||||||||:
   CU-CUACUACUACUACUUC 5'
AT5G53730.1     71      88
putative protein

sRNA_AGO1_Solexa_Mi2008_1_36323_hit13
5' UGAG-GAUGAUGAUGAUGAUGA
   |||| || |||||||||||||
   ACUCUCU-CUACUACUACUACU 5'
AT5G53730.1     73      93
```

```
putative protein

sRNA_AGO1_Solexa_Mi2008_1_41417_hit1
5' UGGCGAGAGUGAUGAUGAUGU
   ||| ||||| ||||||||||
   ACCUCUCUCUCUACUACUACU 5'
AT5G53730.1     75      95
putative protein

sRNA_AGO1_Solexa_Mi2008_1_36498_hit1
5' UGAGGGUACUUUGGUCAUUU
   |||||||||||| ||| |||
   ACUCCCAUGAAAGCAG-AAA 5'
AT5G53950.1     625     643
CUC2 (dbj|BAA19529.1)

sRNA_AGO1_Solexa_Mi2008_2_22079_hit1
5' UAGCAGAGGGUAGUGUUC-GAU
   |||| |||||||||| || |||
   AUCGGCUCCCAUCACCAGCCUA 5'
AT5G53950.1     711     732
CUC2 (dbj|BAA19529.1)

sRNA_AGO1_Solexa_Mi2008_370931_40195_hit2
5' UGGAGAAGCAGGGCACGUGCA
   |||||||:||||:|||||||
   ACCUCUUUGUCCUGUGCACGA 5'
AT5G53950.1     800     820
CUC2 (dbj|BAA19529.1)

sRNA_AGO1_Solexa_Mi2008_7589_40199_hit1
5' UGGAGAAGCAGGGCACGUGCG
   |||||||:||||:|||||||
   ACCUCUUUGUCCUGUGCACGA 5'
AT5G53950.1     800     820
CUC2 (dbj|BAA19529.1)

sRNA_AGO1_Solexa_Mi2008_404_40194_hit3
5' UGGAGAAGCAGGGCACGUGC
   |||||||:||||:|||||||
   ACCUCUUUGUCCUGUGCACG 5'
AT5G53950.1     801     820
CUC2 (dbj|BAA19529.1)

sRNA_AGO1_Solexa_Mi2008_3_12041_hit2
5' CUGGUAAAUUU-ACAUAAUUU
    |||||| ||| |||||||||
   AACCAUU-AAACUGUAUUAAA 5'
AT5G55620.1     563     582
unknown protein

sRNA_AGO1_Solexa_Mi2008_6_13612_hit1
5' GACAGAAGAAAGAGA-GCAC
   || |||||||||||| |||
   CU-UCUUCUUUCUCUUCGUU 5'
AT5G55620.1     76      94
unknown protein

sRNA_AGO1_Solexa_Mi2008_6_35236_hit1
5' UGACAGAAGAAAGAGA-GCAC
   ||| |||||||||||| |||
   ACU-UCUUCUUUCUCUUCGUU 5'
AT5G55620.1     76      95
```

```
unknown protein

sRNA_AGO1_Solexa_Mi2008_1_45939_hit1
5' UG-UUGACACAUGAUCCAUGGA
   || ||| |||||:|||||||||
   ACGAAC-GUGUAUUAGGUACCU 5'
AT5G56870.1     733     753
beta-galactosidase (emb|CAB64740.1)

sRNA_AGO1_Solexa_Mi2008_1_51535_hit1
5' UUGCCAUUAGAGUAUACACAUG
   ||||| || |||||||||||
   AACGG-AA-CUCAUAUGUGUAA 5'
AT5G57550.1     404     423
endoxyloglucan transferase (gb|AAD45127.1)

sRNA_AGO1_Solexa_Mi2008_2_34651_hit1
5' UGAAGCAGAAGUAGUGGUAAA
    ||||||||||| ||||:|||
   UCUUCGUCUUCAACACCGUUU 5'
AT5G58220.2     272     292
unknown protein (At5g58220)

sRNA_AGO1_Solexa_Mi2008_2_34651_hit1
5' UGAAGCAGAAGUAGUGGUAAA
    ||||||||||| ||||:|||
   UCUUCGUCUUCAACACCGUUU 5'
AT5G58220.3     274     294
unknown protein (At5g58220)

sRNA_AGO1_Solexa_Mi2008_1_40627_hit1
5' UGGAGGUGCCGAUGAUGCUAU
   ||||| ||||:||||||||||
   ACCUC-ACGGUUACUACGAUA 5'
AT5G58350.1     1255    1274
MAP kinase

sRNA_AGO1_Solexa_Mi2008_1_13759_hit1
5' GACGAUGAUGAUGAUGACA
   |||||||||||| ||||
   CUGCUACUACUAGUACUCC 5'
AT5G58350.1     1916    1934
MAP kinase

sRNA_AGO1_Solexa_Mi2008_5_13760_hit2
5' GACGAUGAUGAUGAUGAGC
   |||||||||||| |||||
   CUGCUACUACUAGUACUCC 5'
AT5G58350.1     1916    1934
MAP kinase

sRNA_AGO1_Solexa_Mi2008_1_13759_hit1
5' GACGAUGAUGAUGAUGA-CA
   :|||||||:|||||||| ||
   UUGCUACUGCUACUACUAGU 5'
AT5G58350.1     1922    1941
MAP kinase

sRNA_AGO1_Solexa_Mi2008_1_38921_hit1
5' UGCGG-ACGAUGAUGAUGAUGAU
   |:||| |||||||:|||||||||
   AUGCCAUGCUACUGCUACUACUA 5'
AT5G58350.1     1924    1946
```

```
MAP kinase

sRNA_AGO1_Solexa_Mi2008_1_23760_hit1
5' UAGUCG-ACAAACGAAUGAUGG
   |||| | | ||||||||||||
   AUCA-CAUCUUUGCUUACUACC 5'
AT5G58350.1     952     972
MAP kinase

sRNA_AGO1_Solexa_Mi2008_2_4149_hit1
5' CAAAGGCAGAAGAUGAUGACAG
   |||||||| |||||||||| :
   GUUUCCGUAUUCUACUACUGAU 5'
AT5G59780.1     1022    1043
MYB27 protein - like

sRNA_AGO1_Solexa_Mi2008_1_29192_hit1
5' UCCAAAGGCAGAAGAUGAUGA
     |||||||| |||||||||
   UUGUUUCCGUAUUCUACUACU 5'
AT5G59780.1     1025    1045
MYB27 protein - like

sRNA_AGO1_Solexa_Mi2008_1_42751_hit2
5' UGGGUGGUGA-UUGUGAGUGUAA
   |||||||||| || ||| |||||
   ACCCACCACUAAAAACU-ACAUU 5'
AT5G59780.3     865     886
MYB27 protein - like

sRNA_AGO1_Solexa_Mi2008_1_42751_hit2
5' UGGGUGGUGA-UUGUGAGUGUAA
   |||||||||| || ||| |||||
   ACCCACCACUAAAAACU-ACAUU 5'
AT5G59780.2     890     911
MYB27 protein - like

sRNA_AGO1_Solexa_Mi2008_2_4149_hit1
5' CAAAGGCAGAAGAUGAUGACAG
   |||||||| |||||||||| :
   GUUUCCGUAUUCUACUACUGAU 5'
AT5G59780.3     904     925
MYB27 protein - like

sRNA_AGO1_Solexa_Mi2008_1_29192_hit1
5' UCCAAAGGCAGAAGAUGAUGA
     |||||||| |||||||||
   UUGUUUCCGUAUUCUACUACU 5'
AT5G59780.3     907     927
MYB27 protein - like

sRNA_AGO1_Solexa_Mi2008_2_4149_hit1
5' CAAAGGCAGAAGAUGAUGACAG
   |||||||| |||||||||| :
   GUUUCCGUAUUCUACUACUGAU 5'
AT5G59780.2     929     950
MYB27 protein - like

sRNA_AGO1_Solexa_Mi2008_1_29192_hit1
5' UCCAAAGGCAGAAGAUGAUGA
     |||||||| |||||||||
   UUGUUUCCGUAUUCUACUACU 5'
AT5G59780.2     932     952
```

MYB27 protein - like

```
sRNA_AGO1_Solexa_Mi2008_1_42751_hit2
5' UGGGUGGUGA-UUGUGAGUGUAA
   |||||||||| || ||| |||||
   ACCCACCACUAAAAACU-ACAUU 5'
AT5G59780.1     983     1004
MYB27 protein - like

sRNA_AGO1_Solexa_Mi2008_1_41800_hit8
5' UGGGACUAGGAUGCGUACUGA
   |||||| |||||||| || ||
   ACCCUGUUCCUACGC-UG-CU 5'
AT5G59820.1     339     357
zinc finger protein Zat12

sRNA_AGO1_Solexa_Mi2008_7_12746_hit1
5' CUUGACCUUGUAAGACCCC
   ||||||||||:||||||
   GAACUGGAACGUUCUGGAA 5'
AT5G60450.1     1873    1891
auxin response factor 4

sRNA_AGO1_Solexa_Mi2008_7_49852_hit1
5' UUCUUGACCUUGUAAGACCCC
   ||||||||||||:||||||
   AAGAACUGGAACGUUCUGGAA 5'
AT5G60450.1     1873    1893
auxin response factor 4

sRNA_AGO1_Solexa_Mi2008_4_55012_hit1
5' UUUCUUGACCUUGUAAGACCCC
   |||||||||||||:||||||
   AAAGAACUGGAACGUUCUGGAA 5'
AT5G60450.1     1873    1894
auxin response factor 4

sRNA_AGO1_Solexa_Mi2008_1_55011_hit1
5' UUUCUUGACCUUGUAAGACCC
   |||||||||||||:||||||
   AAAGAACUGGAACGUUCUGGA 5'
AT5G60450.1     1874    1894
auxin response factor 4

sRNA_AGO1_Solexa_Mi2008_19_29991_hit1
5' UCCGGCGGUUCAUAACAUCAA
   |||||||||||||||||||||
   AGGCCGCCAAGUAUUGUAGUU 5'
AT5G60450.1     1925    1945
auxin response factor 4

sRNA_AGO1_Solexa_Mi2008_2_24987_hit1
5' UAUCCGGCGGUUCAUAACAUC
   |||||||||||||||||||||
   AUAGGCCGCCAAGUAUUGUAG 5'
AT5G60450.1     1927    1947
auxin response factor 4

sRNA_AGO1_Solexa_Mi2008_8_4564_hit1
5' CAAGAACUGGAUUUGCAUGAGA
   ||||||||||||||||||||||
   GUUCUUGACCUAAACGUACUCU 5'
AT5G60450.1     1966    1987
```

auxin response factor 4

```
sRNA_AGO1_Solexa_Mi2008_7_4563_hit1
5' CAAGAACUGGAUUUGCAUGAG
   |||||||||||||||||||||
   GUUCUUGACCUAAACGUACUC 5'
AT5G60450.1     1967    1987
auxin response factor 4

sRNA_AGO1_Solexa_Mi2008_1_34330_hit1
5' UGAACAAGCUGGGUUCACGCC
   |||||||||||||||||||||
   ACUUGUUCGACCCAAGUGCGG 5'
AT5G60450.1     2035    2055
auxin response factor 4

sRNA_AGO1_Solexa_Mi2008_7_33918_hit1
5' UCUUGACCUUGUAAGACCCCA
   |||||||||||:|||||||
   AGAACUGGAACGUUCUGGGAA 5'
AT5G60450.1     2082    2102
auxin response factor 4

sRNA_AGO1_Solexa_Mi2008_7_49853_hit1
5' UUCUUGACCUUGUAAGACCCCA
   ||||||||||||:|||||||
   AAGAACUGGAACGUUCUGGGAA 5'
AT5G60450.1     2082    2103
auxin response factor 4

sRNA_AGO1_Solexa_Mi2008_7_12746_hit1
5' CUUGACCUUGUAAGACCCC
   ||||||||||:|||||||
   GAACUGGAACGUUCUGGGA 5'
AT5G60450.1     2083    2101
auxin response factor 4

sRNA_AGO1_Solexa_Mi2008_7_49852_hit1
5' UUCUUGACCUUGUAAGACCCC
   ||||||||||||:|||||||
   AAGAACUGGAACGUUCUGGGA 5'
AT5G60450.1     2083    2103
auxin response factor 4

sRNA_AGO1_Solexa_Mi2008_4_55012_hit1
5' UUUCUUGACCUUGUAAGACCCC
   |||||||||||||:|||||||
   AAAGAACUGGAACGUUCUGGGA 5'
AT5G60450.1     2083    2104
auxin response factor 4

sRNA_AGO1_Solexa_Mi2008_1_55011_hit1
5' UUUCUUGACCUUGUAAGACCC
   |||||||||||||:|||||||
   AAAGAACUGGAACGUUCUGGG 5'
AT5G60450.1     2084    2104
auxin response factor 4

sRNA_AGO1_Solexa_Mi2008_2_34797_hit1
5' UGAAGGGGGACCCGAGGAUUG
   |||||||||||||||||||||
   ACUUCCCCCUGGGCUCCUAAC 5'
AT5G60450.1     2266    2286
```

```
auxin response factor 4

sRNA_AGO1_Solexa_Mi2008_3_13421_hit1
5' GAAGAAGAAGAUGAUGUUGAU
   ||||||||||| |||| |||
   CUUCUUCUUCUUCUACCACUU 5'
AT5G60450.1     621     641
auxin response factor 4

sRNA_AGO1_Solexa_Mi2008_1_7242_hit1
5' CAUGGAGAAGAAGAAUGA-GGU
   ||||:|||||||||| || |||
   GUACUUCUUCUUCUU-CUACCA 5'
AT5G60450.1     625     645
auxin response factor 4

sRNA_AGO1_Solexa_Mi2008_1_25289_hit1
5' UAUGACGAUGAUGAUGACGGA
   :|||||||||||||  ||||
   GUACUGCUACUACUA-GGCCU 5'
AT5G60450.1     650     669
auxin response factor 4

sRNA_AGO1_Solexa_Mi2008_9_14254_hit8
5' GAUGAUGAUGAUGAUGAUCUU
   ||||||||:||||||||||:
   CUACUACUGCUACUACUAGGC 5'
AT5G60450.1     651     671
auxin response factor 4

sRNA_AGO1_Solexa_Mi2008_7_3078_hit1
5' AUGAUGAUGA-GAAUGAUGAU
    ||||||||| || |||||||
   AACUACUACUGCU-ACUACUA 5'
AT5G60450.1     655     674
auxin response factor 4

sRNA_AGO1_Solexa_Mi2008_1_45039_hit3
5' UGU-GAUGAUGAUGAUGAUGAUGA
   ||| || ||||||||:||||||||
   ACAUCUUCUACUACUGCUACUACU 5'
AT5G60450.1     656     679
auxin response factor 4

sRNA_AGO1_Solexa_Mi2008_1_10092_hit1
5' CUAGAAGAUGAUGACGAUUGA
   ||||||||||||||||| ||
   GAUCUUCUACUACUGCUA-CU 5'
AT5G60450.1     659     678
auxin response factor 4

sRNA_AGO1_Solexa_Mi2008_3_35894_hit1
5' UGAGAGCAGAGAAAGAGAGU
   |||||| ||||||||||||
   ACUCUC-UCUCUUUCUCUCC 5'
AT5G60450.1     67      85
auxin response factor 4

sRNA_AGO1_Solexa_Mi2008_2_34470_hit2
5' UGAACUCGUUGAAUACAUCAG
    ||| ||||||||| ||||||
   UCUUAAGCAACUUAAGUAGUC 5'
AT5G60890.1     1008    1028
```

```
Myb transcription factor homolog (ATR1)

sRNA_AGO1_Solexa_Mi2008_31_14606_hit1
5' GCCGGGGUUUUGUGAUAUC
   |||||:||||||||| |||
   CGGCCUCAAAACACU-UAG 5'
AT5G60890.1     885     902
Myb transcription factor homolog (ATR1)

sRNA_AGO1_Solexa_Mi2008_24_32530_hit1
5' UCGU-GCCGGGGUUUUGUGAUAU
   |||| |||||:||||||||| ||
   AGCAGCGGCCUCAAAACACU-UA 5'
AT5G60890.1     886     907
Myb transcription factor homolog (ATR1)

sRNA_AGO1_Solexa_Mi2008_1_20014_hit1
5' UACGAAAGGAACAACAUUUAUU
   || ||||| ||||||||| |||
   AU-CUUUCGUUGUUGUAACUAA 5'
AT5G61520.1     1007    1027
monosaccharide transporter STP3

sRNA_AGO1_Solexa_Mi2008_1_20014_hit1
5' UACGAAAGGAACAACAUUUAUU
   || ||||| ||||||||| |||
   AU-CUUUCGUUGUUGUAACUAA 5'
AT5G61520.2     1155    1175
monosaccharide transporter STP3

sRNA_AGO1_Solexa_Mi2008_1_3_hit25
5' AAAAA-AAAAAAAAGAAAGA
     ||| ||||||||||||||
   ACUUUGUUUUUUUUCUUUCU 5'
AT5G61520.2     223     242
monosaccharide transporter STP3

sRNA_AGO1_Solexa_Mi2008_1_16934_hit1
5' UAAAGUGAGGA-UCAAGC-GGU
   ||||||||||| | |||| |||
   AUUUCACUCCUAACUUCGUCCA 5'
AT5G61520.1     740     761
monosaccharide transporter STP3

sRNA_AGO1_Solexa_Mi2008_1_16934_hit1
5' UAAAGUGAGGA-UCAAGC-GGU
   ||||||||||| | |||| |||
   AUUUCACUCCUAACUUCGUCCA 5'
AT5G61520.2     888     909
monosaccharide transporter STP3

sRNA_AGO1_Solexa_Mi2008_3_39824_hit1
5' UGGAAUACUUGAAC-UACCAUCU
   ||||||||| |||  ||||||||
   ACCUUAUGA-CUUUUAUGGUAGA 5'
AT5G61600.1     719     740
DNA binding protein - like

sRNA_AGO1_Solexa_Mi2008_2_5441_hit1
5' CACCACCACCACCAGCGCCGC
   ||||||||||||| ||||:|
   GUGGUGGUGGUGGUGGCGGUG 5'
AT5G61660.1     399     419
```

```
unknown protein

sRNA_AGO1_Solexa_Mi2008_1_7688_hit1
5' CCACCACCACCUCCAGCGCCAC
   ||||||||||| ||| ||||||
   GGUGGUGGUGGUGGUGGCGGUG 5'
AT5G61660.1     399     420
unknown protein

sRNA_AGO1_Solexa_Mi2008_1_1510_hit1
5' ACCACCACCACCAGCGCCGCC
   ||||||||||||| |:|||||
   UGGUGGUGGUGGUGGUGGCGG 5'
AT5G61660.1     401     421
unknown protein

sRNA_AGO1_Solexa_Mi2008_2_5441_hit1
5' CACCACCACCACCAGCGCCGC
    ||||||||||||| |:||||
   UUGGUGGUGGUGGUGGUGGCG 5'
AT5G61660.1     402     422
unknown protein

sRNA_AGO1_Solexa_Mi2008_10_35767_hit2
5' UGAGAAAGAUGAGA-UCACA
   :||||||||||||| ||| |
   GCUCUUUCUACUCUUAGUCU 5'
AT5G62530.1     99      118
delta-1-pyrroline-5-carboxylate dehydrogenase precursor (P5CDH)

sRNA_AGO1_Solexa_Mi2008_1_17123_hit1
5' UAAAUUUUCAGAUGGUUAUU
   |||||||:||||||||| ||
   AUUUAAAGGUCUACCAA-AA 5'
AT5G63800.1     2308    2326
beta-galactosidase like protein

sRNA_AGO1_Solexa_Mi2008_1_47432_hit1
5' UUAGAUAAACAAAUGGA-UGAA
   |||||||||||||| || |||
   AAUCUAUUUGUUUAGCUAACUG 5'
AT5G63800.1     43      64
beta-galactosidase like protein

sRNA_AGO1_Solexa_Mi2008_2_9726_hit2
5' CUAAGUUUGGAUGCUCU-UUGA
    ||||||||||| |||| ||||
   AAUUCAAACCUAGGAGACAACU 5'
AT5G63800.1     7       28
beta-galactosidase like protein

sRNA_AGO1_Solexa_Mi2008_1_35874_hit2
5' UGAGAGACAAAAUGAGAAGGU
   |||||||||||| |||||| |
   ACUCUCUGUUUU-CUCUUCAA 5'
AT5G64090.1     20      39
unknown protein

sRNA_AGO1_Solexa_Mi2008_1_18966_hit2
5' UAAUUGUUGUUGUGUAUGGA
    |||||||||||||| ||||
   UUUAACAACAACACA-ACCU 5'
AT5G64620.1     114     132
```

```
invertase inhibitor homolog (emb|CAA73335.1)

sRNA_AGO1_Solexa_Mi2008_2_46703_hit1
5' UUAAUCAAGGUCGUACAUUGC
   ||||||||||| |||||||:
   AAUUAGUUCCAUCAUGUAAUU 5'
AT5G65310.2     37      57
homeobox-leucine zipper protein ATHB-5 (HD-zip protein ATHB-5) (sp|P46667)

sRNA_AGO1_Solexa_Mi2008_7_12746_hit1
5' CUUGACCUUGUAAG-ACCCC
   ||| |||||||||| ||||
   GAAGUGGAACAUUCUUGGGU 5'
AT5G67370.1     703     722
putative protein
```

```
sRNA_AGO1_Solexa_Mi2008_3_23994_hit1
5' UAGUGGUGUCAAUGUAACAUG
   |||||||| ||||||||||||
   AUCACCAC-GUUACAUUGUAC 5'
AT1G02850.5     479     498
Similar to beta-glucosidases (At1g02850)

sRNA_AGO1_Solexa_Mi2008_1_1909_hit1
5' AGAGCAAUCGUGAAGAUG-GAU
   |||||||||| ||||| | |||
   UCUCGUUAGCUCUUCU-CUCUA 5'
AT1G04120.1     124     144
multi-drug resistance protein

sRNA_AGO1_Solexa_Mi2008_3_768_hit1
5' AAGAAGAGAGAGAAUUGAG
   ||||||| |||||| ||||
   UUCUUCUAUCUCUU-ACUC 5'
AT1G04120.1     187     204
multi-drug resistance protein

sRNA_AGO1_Solexa_Mi2008_3_13733_hit1
5' GACGAAGAUCAUGAACGACA
   :|||||||||||||| |||
   UUGCUUCUAGUACUU-CUGG 5'
AT1G04120.1     4347    4365
multi-drug resistance protein

sRNA_AGO1_Solexa_Mi2008_1_27166_hit1
5' UCAAGCUGG-UCAAGAUGUGCU
   ||||| ||| ||||||| ||||
   AGUUC-ACCCAGUUCUAAACGA 5'
AT1G04120.1     4418    4438
multi-drug resistance protein

sRNA_AGO1_Solexa_Mi2008_2_27439_hit1
5' UCAAUG-CAUGUGGCUGUCAACA
   |||| | |||||:||||||||||
   AGUU-CAGUACAUCGACAGUUGU 5'
AT1G05570.1     2658    2679
putative glucan synthase

sRNA_AGO1_Solexa_Mi2008_7_3892_hit1
5' CAAAACCAGAAGGA-UGAUAAAG
   |||||||||| ||| ||| ||||
   GUUUUGGUCUGCCUAACU-UUUC 5'
AT1G05570.1     5113    5134
putative glucan synthase

sRNA_AGO1_Solexa_Mi2008_1_3891_hit1
5' CAAAACCAGAAGGA-UGAUAAA
   |||||||||| ||| ||| |||
   GUUUUGGUCUGCCUAACU-UUU 5'
AT1G05570.1     5114    5134
putative glucan synthase

sRNA_AGO1_Solexa_Mi2008_3_6611_hit1
5' CAGGUCAACAACAAUCUUUC
   ||| ||| |||||||||||
   GUC-AGUAGUUGUUAGAAAA 5'
AT1G05570.1     5141    5159
putative glucan synthase
```

```
sRNA_AGO1_Solexa_Mi2008_1_54576_hit1
5' UUUAUGAGCUCUAAAACGACGUC
   ||| || ||| |||||||||||
   AAA-ACGCGA-AUUUUGCUGCAG 5'
AT1G05710.3     136     156
unknown protein

sRNA_AGO1_Solexa_Mi2008_1_34211_hit3
5' UGAAAGAGAGAUGAGAGCUUU
   |||||||||||| ||| |||
   ACUUUCUCUCUAGUCU-GAAC 5'
AT1G05710.4     149     168
unknown protein

sRNA_AGO1_Solexa_Mi2008_1_34211_hit3
5' UGAAAGAGAGAUGAGAGCUUU
   |||||||||||| ||| |||
   ACUUUCUCUCUAGUCU-GAAC 5'
AT1G05710.1     260     279
unknown protein

sRNA_AGO1_Solexa_Mi2008_1_34211_hit3
5' UGAAAGAGAGAUGAGAGCUUU
   |||||||||||| ||| |||
   ACUUUCUCUCUAGUCU-GAAC 5'
AT1G05710.3     521     540
unknown protein

sRNA_AGO1_Solexa_Mi2008_2_41651_hit1
5' UGGCUUAACACAACGUAU-GUAG
   ||||||||||||| | || ||||
   ACCGAAUUGUGUUUC-UAGCAUC 5'
AT1G05710.4     536     557
unknown protein

sRNA_AGO1_Solexa_Mi2008_2_41651_hit1
5' UGGCUUAACACAACGUAU-GUAG
   ||||||||||||| | || ||||
   ACCGAAUUGUGUUUC-UAGCAUC 5'
AT1G05710.1     647     668
unknown protein

sRNA_AGO1_Solexa_Mi2008_2_41651_hit1
5' UGGCUUAACACAACGUAU-GUAG
   ||||||||||||| | || ||||
   ACCGAAUUGUGUUUC-UAGCAUC 5'
AT1G05710.3     908     929
unknown protein

sRNA_AGO1_Solexa_Mi2008_2_41651_hit1
5' UGGCUUAACACAACGUAU-GUAG
   ||||||||||||| | || ||||
   ACCGAAUUGUGUUUC-UAGCAUC 5'
AT1G05710.2     974     995
unknown protein

sRNA_AGO1_Solexa_Mi2008_1_27132_hit1
5' UCAAGCACC-AGCUCGAAGAAG
   |||| | || |||||||||||
   AGUUGG-GGAUCGAGCUUCUUC 5'
AT1G06150.1     217     237
unknown protein
```

```
sRNA_AGO1_Solexa_Mi2008_1_44921_hit1
5' UGUGAAUGAUGCUAUAUUGUG
     |||||| |||||||||||
   UGACUUACAACGAUAUAACAC 5'
AT1G06150.1     2946    2966
unknown protein

sRNA_AGO1_Solexa_Mi2008_1_34211_hit3
5' UGAAAGAGAGAUGAGA-GCUUU
   |||||||||||||| | :||||
   ACUUUCUCUCUACU-UGUGAAA 5'
AT1G06150.1     42      62
unknown protein

sRNA_AGO1_Solexa_Mi2008_1_3874_hit3
5' CAAAAAUGAUUGAUG-A-GAA
   || |||||||||||| | |||
   GUCUUUACUAACUACAUUCUU 5'
AT1G06160.1     142     162
ethylene response factor, putative

sRNA_AGO1_Solexa_Mi2008_1_13420_hit2
5' GAAGAAGAAGAAGACUCUU
   |||||||||||||| | ||
   CUUCUUCUUCUUCU-A-AA 5'
AT1G06160.1     580     596
ethylene response factor, putative

sRNA_AGO1_Solexa_Mi2008_8_56168_hit1
5' UUUGUGUUCUCAGGUCACCCCUU
   |||||| |||||||  |||||||
   AAACACUAGAGUCCUUUGGGGAA 5'
AT1G08830.1     116     138
superoxidase dismutase

sRNA_AGO1_Solexa_Mi2008_1_26464_hit13
5' UAUUUCAGGAAAUCAGA-UGGCA
   | |||| |||||||||| |||||
   ACAAAGACCUUUAGUCUUACCGU 5'
AT1G10450.1     1510    1532
unknown protein

sRNA_AGO1_Solexa_Mi2008_1_369_hit5
5' AAAGAAUGCCAAUGAUGAGGU
   |||||||||||| |||| ||:
   UUUCUUACGGUUUCUAC-CCG 5'
AT1G10450.1     1854    1873
unknown protein

sRNA_AGO1_Solexa_Mi2008_1_36675_hit1
5' UG-AGGUUGCAGAUGCUUGG
   || || ||||||| ||||||
   ACAUC-AACGUCUUCGAACC 5'
AT1G10450.1     2981    2999
unknown protein

sRNA_AGO1_Solexa_Mi2008_1_8542_hit1
5' CGAGCAAGUGACAACGGUU-UC
   |||||||||||||| |:|| ||
   GCUCGUUCACUGUU-CUAAGAG 5'
AT1G10450.1     841     861
unknown protein
```

```
sRNA_AGO1_Solexa_Mi2008_1_40683_hit1
5' UGGAGUCAGACG-AGCAAGUGA
   :|||||||| || |||||||||
   GCCUCAGUC-GCCUCGUUCACU 5'
AT1G10450.1     851     871
unknown protein

sRNA_AGO1_Solexa_Mi2008_1_56685_hit2
5' UUUUUUCAGUUGUAUUUG-UGU
   ||||||||||| |||:|| |||
   AAAAAAGUCAAGAUAGACGACA 5'
AT1G10450.1     937     958
unknown protein

sRNA_AGO1_Solexa_Mi2008_1_13075_hit1
5' CUUGUGAUUUCAGAUAUGCUU
    |||||||||| ||||| |||
   UAACACUAAAGGCUAUA-GAA 5'
AT1G10450.1     977     996
unknown protein

sRNA_AGO1_Solexa_Mi2008_8_1538_hit1
5' ACCGAUGAUGAUU-AUUGCUA
   ||||||||||||| | ||| |
   UGGCUACUACUAACUCACGCU 5'
AT1G10970.1     999     1019
zinc transporter like protein (ZIP4)

sRNA_AGO1_Solexa_Mi2008_1_18608_hit1
5' UAAUAAGCUGAGCGAGGAUCA
   |||||| ||||||||||| ||
   AUUAUUAGACUCGCUCCU-GU 5'
AT1G11670.1     823     842
unknown protein

sRNA_AGO1_Solexa_Mi2008_1_1059_hit1
5' AAU-GAAAGUAGAGAGAGCU
   ||| ||||||| |||||| |
   UUAACUUUCAUGUCUCUCAA 5'
AT1G12520.1     1109    1128
Cu/Zn superoxide dismutase copper chaperone like protein

sRNA_AGO1_Solexa_Mi2008_1_1059_hit1
5' AAU-GAAAGUAGAGAGAGCU
   ||| ||||||| |||||| |
   UUAACUUUCAUGUCUCUCAA 5'
AT1G12520.2     1178    1197
Cu/Zn superoxide dismutase copper chaperone like protein

sRNA_AGO1_Solexa_Mi2008_1_1059_hit1
5' AAU-GAAAGUAGAGAGAGCU
   ||| ||||||| |||||| |
   UUAACUUUCAUGUCUCUCAA 5'
AT1G12520.3     1194    1213
Cu/Zn superoxide dismutase copper chaperone like protein

sRNA_AGO1_Solexa_Mi2008_1_27462_hit1
5' UCAAUGGAGGCAAUGAAUCGGU
   ||||||||||||||||||||||
   AGUUACCUCCGUUACUUAGCCA 5'
AT1G13640.1     104     125
unknown protein
```

```
sRNA_AGO1_Solexa_Mi2008_4_14108_hit2
5' GAGGUAAA-GAUGAAAAGGA
    |:||||| |||||||||||
   AUUCAUUUUCUACUUUUCCU 5'
AT1G15125.1     124     143
putative protein

sRNA_AGO1_Solexa_Mi2008_4_4868_hit1
5' CAAG-GGAGCUUGGAUGAUGA
   |||| || |||||||||||||
   GUUCUCC-CGAACCUACUACU 5'
AT1G15125.1     194     213
putative protein

sRNA_AGO1_Solexa_Mi2008_7_50046_hit3
5' UUGAAGAGGACUUGGAACU
   |||||||:||||||:||||
   AACUUCUUCUGAACUUUGA 5'
AT1G15125.1     253     271
putative protein

sRNA_AGO1_Solexa_Mi2008_26_5458_hit1
5' CACCAGAGUAUCCUGCAAG
   ||||||||:| ||||||||
   GUGGUCUCGUCGGACGUUC 5'
AT1G15750.1     1384    1402
unknown protein (At1g15750)

sRNA_AGO1_Solexa_Mi2008_26_5458_hit1
5' CACCAGAGUAUCCUGCAAG
   ||||||||:| ||||||||
   GUGGUCUCGUCGGACGUUC 5'
AT1G15750.4     1385    1403
unknown protein (At1g15750)

sRNA_AGO1_Solexa_Mi2008_26_5458_hit1
5' CACCAGAGUAUCCUGCAAG
   ||||||||:| ||||||||
   GUGGUCUCGUCGGACGUUC 5'
AT1G15750.2     1524    1542
unknown protein (At1g15750)

sRNA_AGO1_Solexa_Mi2008_2_13419_hit2
5' GAAGAAGAAGAAGAC-ACUU
   ||||||||||||||| ||
   CUUCUUCUUCUUCUGGUGUC 5'
AT1G15750.2     154     173
unknown protein (At1g15750)

sRNA_AGO1_Solexa_Mi2008_26_5458_hit1
5' CACCAGAGUAUCCUGCAAG
   ||||||||:| ||||||||
   GUGGUCUCGUCGGACGUUC 5'
AT1G15750.3     1657    1675
unknown protein (At1g15750)

sRNA_AGO1_Solexa_Mi2008_1_20_hit1
5' AAAAAAAGAGGUAAUUU-GGA
   ||||||||||| ||||| ||
   UUUUUUUCUCCUUUAAAUCCA 5'
AT1G15750.3     188     208
unknown protein (At1g15750)
```

```
sRNA_AGO1_Solexa_Mi2008_1_20_hit1
5' AAAAAAAGAGGUAAUUU-GGA
   ||||||||||| ||||| ||
   UUUUUUUCUCCUUUAAAUCCA 5'
AT1G15750.1     22      42
unknown protein (At1g15750)

sRNA_AGO1_Solexa_Mi2008_2_13419_hit2
5' GAAGAAGAAGAAGAC-ACUU
   ||||||||||||||| ||
   CUUCUUCUUCUUCUGGUGUC 5'
AT1G15750.4     50      69
unknown protein (At1g15750)

sRNA_AGO1_Solexa_Mi2008_10_30419_hit1
5' UCGAACGCUUCACGGAUCUCUA
   ||||| || ||||||||||||
   AGCUUUCGCAGUGCCUAGAGAC 5'
AT1G18210.2     314     335
unknown protein

sRNA_AGO1_Solexa_Mi2008_1_14930_hit1
5' GGAUUGAAGGGAGCUCUAC
     ||||| |||||||||||
   GGUAACUACCCUCGAGAUG 5'
AT1G18250.2     130     148
putative protein

sRNA_AGO1_Solexa_Mi2008_1_14930_hit1
5' GGAUUGAAGGGAGCUCUAC
     ||||| |||||||||||
   GGUAACUACCCUCGAGAUG 5'
AT1G18250.1     25      43
putative protein

sRNA_AGO1_Solexa_Mi2008_1_42552_hit1
5' UGGGGUUGGUUGG-UUGGUUGG
   ||||||||||||| |||||||
   ACCCCAACCAACCAAACCAACG 5'
AT1G19180.1     27      48
unknown protein

sRNA_AGO1_Solexa_Mi2008_1_42552_hit1
5' UGGGGUUGGUUGG-UUGGUUGG
   ||||||||||||| |||||||
   ACCCCAACCAACCAAACCAACG 5'
AT1G19180.2     7       28
unknown protein

sRNA_AGO1_Solexa_Mi2008_1_36931_hit2
5' UGAUAACCGUAGA-GCCGAUGG
   ||||||||| ||| ||||||||
   ACUAUUGGC-UCUUCGGCUACC 5'
AT1G21270.1     1076    1096
putative protein

sRNA_AGO1_Solexa_Mi2008_2_35299_hit10
5' UGACAUCAACAUUUAAUGGCC
   |||||||||||||| ||||||
   ACUGUAGUUGUAAACUACCGG 5'
AT1G21270.1     1213    1233
putative protein
```

```
sRNA_AGO1_Solexa_Mi2008_3_20869_hit2
5' UA-GAACUUGAUUAUUCAUGAU
   || |||| |||||||| |||||
   AUACUUGUACUAAUAACUACUA 5'
AT1G21270.1     2369    2390
putative protein

sRNA_AGO1_Solexa_Mi2008_12_5493_hit2
5' CACCCAUUUGAUACAUAGU
   |||||||||||| |||| |
   GUGGGUAAACUA-GUAUAA 5'
AT1G23850.1     1142    1159
unknown protein

sRNA_AGO1_Solexa_Mi2008_1_55587_hit1
5' UUUGGAGUGAAAGGU-GAAGAU
   ||||||||||||| | |||||
   AAACCUCACUUUCGAGCUUCUU 5'
AT1G23850.1     317     338
unknown protein

sRNA_AGO1_Solexa_Mi2008_1_43578_hit1
5' UGGUGGUGGUGAUGAUGUGUC
   |||||||||||| || | |||
   ACCACCACCACUUCU-C-CAG 5'
AT1G23850.1     488     506
unknown protein

sRNA_AGO1_Solexa_Mi2008_1_3079_hit472
5' AUGAUGAUGAUGAUGAUGAUGA
   |||||||| |||||| |||||
   UACUACUAAUACUACAACUACC 5'
AT1G24575.1     89      110
unknown protein

sRNA_AGO1_Solexa_Mi2008_1_3079_hit472
5' AUGAUGAUGAUGAUGAUGAUGA
    |||||||||| |||||| |||
   CACUACUACUAAUACUACAACU 5'
AT1G24575.1     92      113
unknown protein

sRNA_AGO1_Solexa_Mi2008_1_45039_hit3
5' UGUGAUGAUGAUGAUGAUGAUGA
    ||||||||||| |||||| |||
   CCACUACUACUAAUACUACAACU 5'
AT1G24575.1     92      114
unknown protein

sRNA_AGO1_Solexa_Mi2008_1_2280_hit1
5' AGUGGAUGAUGAUGAUGAUG
    ||| |||||||| ||||||
   GCAC-UACUACUAAUACUAC 5'
AT1G24575.1     97      115
unknown protein

sRNA_AGO1_Solexa_Mi2008_2_45177_hit1
5' UGUGCGGCCGUCGCUGGUGGU
   ||||||||||||||||||||
   ACACGCCGGCAGCGACCACCA 5'
AT1G26770.2     712     732
Expansin (AtEXPA10)
```

```
sRNA_AGO1_Solexa_Mi2008_2_45177_hit1
5' UGUGCGGCCGUCGCUGGUGGU
   ||||||||||||||||||||
   ACACGCCGGCAGCGACCACCA 5'
AT1G26770.1     752     772
Expansin (AtEXPA10)

sRNA_AGO1_Solexa_Mi2008_1_43831_hit1
5' UGGUUGUGGCUC-UGGUGGUAA
   |||| ||||| | |||||||||
   ACCACCACCG-GCACCACCAUU 5'
AT1G28290.2     158     178
proline-rich protein, putative

sRNA_AGO1_Solexa_Mi2008_1_35934_hit2
5' UGAG-AGUGAUGUGGGUGGUGGU
   |||| || | |||||||||||||
   ACUCCUCCC-ACACCCACCACCA 5'
AT1G28290.2     172     193
proline-rich protein, putative

sRNA_AGO1_Solexa_Mi2008_1_43831_hit1
5' UGGUUGUGGCUC-UGGUGGUAA
   |||| ||||| | |||||||||
   ACCACCACCG-GCACCACCAUU 5'
AT1G28290.1     276     296
proline-rich protein, putative

sRNA_AGO1_Solexa_Mi2008_1_35934_hit2
5' UGAG-AGUGAUGUGGGUGGUGGU
   |||| || | |||||||||||||
   ACUCCUCCC-ACACCCACCACCA 5'
AT1G28290.1     290     311
proline-rich protein, putative

sRNA_AGO1_Solexa_Mi2008_1_43744_hit2
5' UGGUUAGAGGGUAAAUUGGUC
   ||||| |||||||||:||||
   ACCAACCUCCCAUUUGACCAC 5'
AT1G28290.2     396     416
proline-rich protein, putative

sRNA_AGO1_Solexa_Mi2008_1_43744_hit2
5' UGGUUAGAGGGUAAAUUGGUC
   ||||| |||||||||:||||
   ACCAACCUCCCAUUUGACCAC 5'
AT1G28290.2     456     476
proline-rich protein, putative

sRNA_AGO1_Solexa_Mi2008_1_43744_hit2
5' UGGUUAGAGGGUAAAUUGGUC
   ||||| |||||||||:||||
   ACCAACCUCCCAUUUGACCAC 5'
AT1G28290.1     514     534
proline-rich protein, putative

sRNA_AGO1_Solexa_Mi2008_1_43744_hit2
5' UGGUUAGAGGGUAAAUUGGUC
   ||||| |||||||||:||||
   ACCAACCUCCCAUUUGACCAC 5'
AT1G28290.1     574     594
proline-rich protein, putative
```

```
sRNA_AGO1_Solexa_Mi2008_1_43744_hit2
5' UGGUUAGAGGGUAAAUUGGUC
   ||||| |||||||||:||||
   ACCAACCUCCCAUUUGACCAC 5'
AT1G28290.1     634     654
proline-rich protein, putative

sRNA_AGO1_Solexa_Mi2008_1_12727_hit1
5' CUUGAACCUUGGA-AGAAAAC
    ||||||||||||  ||||||
   UAACUUGGAACCUGACUUUUG 5'
AT1G30400.1     1603    1623
AtMRP1

sRNA_AGO1_Solexa_Mi2008_1_12727_hit1
5' CUUGAACCUUGGA-AGAAAAC
    ||||||||||||  ||||||
   UAACUUGGAACCUGACUUUUG 5'
AT1G30400.2     1673    1693
AtMRP1

sRNA_AGO1_Solexa_Mi2008_2_37709_hit1
5' UGCAAAUGAUUGUUGGUUCGA
   |||| ||| |||||||||||
   ACGUAUACGAACAACCAAGCG 5'
AT1G30400.1     3599    3619
AtMRP1

sRNA_AGO1_Solexa_Mi2008_1_3121_hit1
5' AUGCAAAUGAUUGUUGGUU
   ||||| ||| |||||||||
   UACGUAUACGAACAACCAA 5'
AT1G30400.1     3602    3620
AtMRP1

sRNA_AGO1_Solexa_Mi2008_2_37709_hit1
5' UGCAAAUGAUUGUUGGUUCGA
   |||| ||| |||||||||||
   ACGUAUACGAACAACCAAGCG 5'
AT1G30400.2     3669    3689
AtMRP1

sRNA_AGO1_Solexa_Mi2008_1_3121_hit1
5' AUGCAAAUGAUUGUUGGUU
   ||||| ||| |||||||||
   UACGUAUACGAACAACCAA 5'
AT1G30400.2     3672    3690
AtMRP1

sRNA_AGO1_Solexa_Mi2008_1_6844_hit1
5' CAUACAACGCCACAUAGGGAG
   |||| |||||| |||||||||
   GUAU-UUGCGGAGUAUCCCUC 5'
AT1G30400.1     427     446
AtMRP1

sRNA_AGO1_Solexa_Mi2008_1_6844_hit1
5' CAUACAACGCCACAUAGGGAG
   |||| |||||| |||||||||
   GUAU-UUGCGGAGUAUCCCUC 5'
AT1G30400.2     497     516
AtMRP1
```

```
sRNA_AGO1_Solexa_Mi2008_1_40743_hit3
5' UGGAGUUGCUCAGAAAGAUGG
   ||||||||||| || |||||
   ACCUCAACGAGACUCUCUACG 5'
AT1G30490.1     1711    1731
HD-Zip protein

sRNA_AGO1_Solexa_Mi2008_11_7246_hit3
5' CAUGGAGUUGCUCAGAAAGAU
    |||||||||||| || ||||
   AUACCUCAACGAGACUCUCUA 5'
AT1G30490.1     1713    1733
HD-Zip protein

sRNA_AGO1_Solexa_Mi2008_1_9282_hit3
5' CGGUUUUUUCGGGU-UUUUUC
   | ||||||| |||| ||||||
   GACAAAAAA-CCCAAAAAAAG 5'
AT1G30490.1     17      36
HD-Zip protein

sRNA_AGO1_Solexa_Mi2008_1_39355_hit2
5' UGCUGAUGUG-UGGGCUUUUGG
   |||||||||| |||| |||||
   ACGACUACACAACCC-AAAACG 5'
AT1G30490.1     664     684
HD-Zip protein

sRNA_AGO1_Solexa_Mi2008_2_8957_hit1
5' CGGACCAGGCUUCAUCCCCCC
   ||||||||||||||||||
   GCCUGGUCCGAAGUAGGGUUA 5'
AT1G30490.1     793     813
HD-Zip protein

sRNA_AGO1_Solexa_Mi2008_153_14822_hit2
5' GGACCAGGCUUCAUCCCCC
   |||||||||||||||||
   CCUGGUCCGAAGUAGGGUU 5'
AT1G30490.1     794     812
HD-Zip protein

sRNA_AGO1_Solexa_Mi2008_219_8956_hit2
5' CGGACCAGGCUUCAUCCCCC
   ||||||||||||||||||
   GCCUGGUCCGAAGUAGGGUU 5'
AT1G30490.1     794     813
HD-Zip protein

sRNA_AGO1_Solexa_Mi2008_1063_31727_hit2
5' UCGGACCAGGCUUCAUCCCCC
   :||||||||||||||||||
   GGCCUGGUCCGAAGUAGGGUU 5'
AT1G30490.1     794     814
HD-Zip protein

sRNA_AGO1_Solexa_Mi2008_4_8955_hit2
5' CGGACCAGGCUUCAUCCCC
   ||||||||||||||||||
   GCCUGGUCCGAAGUAGGGU 5'
AT1G30490.1     795     813
HD-Zip protein
```

```
sRNA_AGO1_Solexa_Mi2008_8_8960_hit4
5' CGGACCAGGCUUCAUUCCCCC
   |||||||||||||| ||||
   GCCUGGUCCGAAGU-AGGGUU 5'
AT1G30490.1     795     814
HD-Zip protein

sRNA_AGO1_Solexa_Mi2008_47_31726_hit2
5' UCGGACCAGGCUUCAUCCCC
   :||||||||||||||||||
   GGCCUGGUCCGAAGUAGGGU 5'
AT1G30490.1     795     814
HD-Zip protein

sRNA_AGO1_Solexa_Mi2008_310_14823_hit7
5' GGACCAGGCUUCAUUCCCC
   ||||||||||||| ||||
   CCUGGUCCGAAGU-AGGGU 5'
AT1G30490.1     796     813
HD-Zip protein

sRNA_AGO1_Solexa_Mi2008_641_8959_hit7
5' CGGACCAGGCUUCAUUCCCC
   |||||||||||||| ||||
   GCCUGGUCCGAAGU-AGGGU 5'
AT1G30490.1     796     814
HD-Zip protein

sRNA_AGO1_Solexa_Mi2008_2_31725_hit2
5' UCGGACCAGGCUUCAUCCC
   :||||||||||||||||||
   GGCCUGGUCCGAAGUAGGG 5'
AT1G30490.1     796     814
HD-Zip protein

sRNA_AGO1_Solexa_Mi2008_4138_31731_hit7
5' UCGGACCAGGCUUCAUUCCCC
    |||||||||||||| ||||
   CGCCUGGUCCGAAGU-AGGGU 5'
AT1G30490.1     796     815
HD-Zip protein

sRNA_AGO1_Solexa_Mi2008_1_8958_hit7
5' CGGACCAGGCUUCAUUCCC
   |||||||||||||| ||||
   GCCUGGUCCGAAGU-AGGG 5'
AT1G30490.1     797     814
HD-Zip protein

sRNA_AGO1_Solexa_Mi2008_15_11085_hit1
5' CUCGGACCAGGCUUCAUCC
    :|||||||||||||||||
   AGGCCUGGUCCGAAGUAGG 5'
AT1G30490.1     797     815
HD-Zip protein

sRNA_AGO1_Solexa_Mi2008_98_31730_hit7
5' UCGGACCAGGCUUCAUUCCC
    |||||||||||||| ||||
   CGCCUGGUCCGAAGU-AGGG 5'
AT1G30490.1     797     815
HD-Zip protein
```

```
sRNA_AGO1_Solexa_Mi2008_1_11087_hit3
5' CUCGGACCAGGCUUCAUUCCC
     |||||||||||||| ||||
   UCGCCUGGUCCGAAGU-AGGG 5'
AT1G30490.1     797     816
HD-Zip protein

sRNA_AGO1_Solexa_Mi2008_33_15725_hit3
5' GUCGGACCAGGCUUCAUUCCC
   : |||||||||||||| ||||
   UCGCCUGGUCCGAAGU-AGGG 5'
AT1G30490.1     797     816
HD-Zip protein

sRNA_AGO1_Solexa_Mi2008_68_49390_hit1
5' UUCGGACCAGGCUUCAUUCCC
     |||||||||||||| ||||
   UCGCCUGGUCCGAAGU-AGGG 5'
AT1G30490.1     797     816
HD-Zip protein

sRNA_AGO1_Solexa_Mi2008_6_31729_hit7
5' UCGGACCAGGCUUCAUUCC
    |||||||||||||| |||
   CGCCUGGUCCGAAGU-AGG 5'
AT1G30490.1     798     815
HD-Zip protein

sRNA_AGO1_Solexa_Mi2008_1_49389_hit1
5' UUCGGACCAGGCUUCAUUCC
     |||||||||||||| |||
   UCGCCUGGUCCGAAGU-AGG 5'
AT1G30490.1     798     816
HD-Zip protein

sRNA_AGO1_Solexa_Mi2008_3_40720_hit1
5' UGGAGUGGGACGGCGAGAUCAU
   ||||||||:|||| |||| |||
   ACCUCACCUUGCCUCUCU-GUA 5'
AT1G33270.2     224     244
unknown protein

sRNA_AGO1_Solexa_Mi2008_3_17114_hit1
5' UAA-AUU-GUUGUAUGGAGAA
   ||| ||| |||| ||||||||
   AUUAUAAACAACUUACCUCUU 5'
AT1G33560.1     1269    1289
ADR1 (activated disease resistance)

sRNA_AGO1_Solexa_Mi2008_73_9994_hit1
5' CUACUAGAGCGAACGAGCA
   |||||||||||| | |||
   GAUGAUCUCGCUCG-UCGA 5'
AT1G33560.1     377     394
ADR1 (activated disease resistance)

sRNA_AGO1_Solexa_Mi2008_1_28030_hit4
5' UCAGAAGAAGCCAC-GUCAGAUGA
   ||||||||||  || |||||||||
   AGUCUUCUUCU-UGGCAGUCUACU 5'
AT1G35210.1     259     281
unknown protein
```

```
sRNA_AGO1_Solexa_Mi2008_1_919_hit1
5' AAGGCUAAUGUUGUGAUCUUC
    |||| | ||||||||||||
   GUCCG-U-ACAACACUAGAAG 5'
AT1G35310.1     137     155
unknown protein

sRNA_AGO1_Solexa_Mi2008_1_14083_hit2
5' GAGGCCAAUGUUGUGAUCUA
   ||||| | |||||||||||
   CUCCG-U-ACAACACUAGAA 5'
AT1G35310.1     138     155
unknown protein

sRNA_AGO1_Solexa_Mi2008_1_52302_hit3
5' UUGGCAGGGUCAUCCUUGGAG
   ||| ||||:||||||||||||
   AAC-GUCCUAGUAGGAACCUC 5'
AT1G35550.1     162     181
elongation factor, putative

sRNA_AGO1_Solexa_Mi2008_1_26497_hit1
5' UAUUUGUGA-GAGUUCGUGAAA
   | ||| ||| ||||||||||||
   AGAAA-ACUACUCAAGCACUUU 5'
AT1G35710.1     129     149
protein kinase, putative

sRNA_AGO1_Solexa_Mi2008_1_19304_hit2
5' UACACGUUCAUCGGAAAUGCU
   |||||||||||||||||||||
   AUGUGCAAGUAGCCUUUACGA 5'
AT1G35710.1     3219    3239
protein kinase, putative

sRNA_AGO1_Solexa_Mi2008_2_5215_hit1
5' CAAUUAG-UGGAGAAGUUCA
    |||| | |||||||||||
   AUUAA-CUACCUCUUCAAGU 5'
AT1G35710.1     3368    3386
protein kinase, putative

sRNA_AGO1_Solexa_Mi2008_10_17146_hit1
5' UAACAACAACAAC-AAAGGUGAA
   ||||||||||||| |||| ||||
   AUUGUUGUUGUUGUUUCGACUU 5'
AT1G35710.1     3487    3509
protein kinase, putative

sRNA_AGO1_Solexa_Mi2008_1_28845_hit2
5' UCAUCACUAGGAAGCGUUGGA
   |||| ||:||||||||||||
   AGUAAUGGUCCUUCGCAACCU 5'
AT1G37130.1     1592    1612
nitrate reductase (At1g37130)

sRNA_AGO1_Solexa_Mi2008_2_44202_hit1
5' UGUAGGACGAAUGCUUUGGUA
   |||||||||||||||||||||
   ACAUCCUGCUUACGAAACCAU 5'
AT1G48410.1     1250    1270
Argonaute protein (AGO1)
```

```
sRNA_AGO1_Solexa_Mi2008_2_44202_hit1
5' UGUAGGACGAAUGCUUUGGUA
   |||||||||||||||||||||
   ACAUCCUGCUUACGAAACCAU 5'
AT1G48410.2     1256    1276
Argonaute protein (AGO1)

sRNA_AGO1_Solexa_Mi2008_13_6596_hit1
5' CAGGUAACCUUCAGCAAAGCA
   |||||||||||||||||||||
   GUCCAUUGGAAGUCGUUUCGU 5'
AT1G48410.1     1711    1731
Argonaute protein (AGO1)

sRNA_AGO1_Solexa_Mi2008_13_6596_hit1
5' CAGGUAACCUUCAGCAAAGCA
   |||||||||||||||||||||
   GUCCAUUGGAAGUCGUUUCGU 5'
AT1G48410.2     1717    1737
Argonaute protein (AGO1)

sRNA_AGO1_Solexa_Mi2008_16_26870_hit1
5' UCAACAGAAGCCAGAGAAGUA
   |||||||||||||||||||||
   AGUUGUCUUCGGUCUCUUCAU 5'
AT1G48410.1     1831    1851
Argonaute protein (AGO1)

sRNA_AGO1_Solexa_Mi2008_16_26870_hit1
5' UCAACAGAAGCCAGAGAAGUA
   |||||||||||||||||||||
   AGUUGUCUUCGGUCUCUUCAU 5'
AT1G48410.2     1837    1857
Argonaute protein (AGO1)

sRNA_AGO1_Solexa_Mi2008_3_40523_hit1
5' UGGAGGAGGCAGUAUACGAGC
   |||||||||||||||||||||
   ACCUCCUCCGUCAUAUGCUCG 5'
AT1G48410.1     1853    1873
Argonaute protein (AGO1)

sRNA_AGO1_Solexa_Mi2008_3_40523_hit1
5' UGGAGGAGGCAGUAUACGAGC
   |||||||||||||||||||||
   ACCUCCUCCGUCAUAUGCUCG 5'
AT1G48410.2     1859    1879
Argonaute protein (AGO1)

sRNA_AGO1_Solexa_Mi2008_2_28543_hit1
5' UCAGGGCGAGCACUGACUGGU
   |||||||||||||||||||||
   AGUCCCGCUCGUGACUGACCA 5'
AT1G48410.1     2098    2118
Argonaute protein (AGO1)

sRNA_AGO1_Solexa_Mi2008_2_28543_hit1
5' UCAGGGCGAGCACUGACUGGU
   |||||||||||||||||||||
   AGUCCCGCUCGUGACUGACCA 5'
AT1G48410.2     2104    2124
Argonaute protein (AGO1)
```

```
sRNA_AGO1_Solexa_Mi2008_2_36850_hit22
5' UGAGUGUGGUCCUCCUCUUCC
   |||| | |||||||||||||
   ACUC-CUCCAGGAGGAGAAGG 5'
AT1G48410.2     417     436
Argonaute protein (AGO1)

sRNA_AGO1_Solexa_Mi2008_1_55475_hit2
5' UUUG-CUC-GCGGUGGUCCAGAC
   |||| ||| |||||||||||||
   AAACAGAGACGCCACCAGGUCUU 5'
AT1G48410.2     485     507
Argonaute protein (AGO1)

sRNA_AGO1_Solexa_Mi2008_46_8918_hit2
5' CGCUUGGUGCAGGUCGGGAAC
    |||||:||||| ||||||||
   UCGAACUACGUCGAGCCCUUG 5'
AT1G48410.2     509     529
Argonaute protein (AGO1)

sRNA_AGO1_Solexa_Mi2008_280_14793_hit2
5' GCUUGGUGCAGGUCGGGAA
   |||||:||||| |||||||
   CGAACUACGUCGAGCCCUU 5'
AT1G48410.2     510     528
Argonaute protein (AGO1)

sRNA_AGO1_Solexa_Mi2008_1_39168_hit1
5' UGCUAGAAGAAGGUAUAGGCU
   |||||||||||||||||||||
   ACGAUCUUCUUCCAUAUCCGA 5'
AT1G48410.2     669     689
Argonaute protein (AGO1)

sRNA_AGO1_Solexa_Mi2008_5_6229_hit1
5' CAGCAAAGAAAUGGUUAGCC
   ||||||||||||||||||||
   GUCGUUUCUUUACCAAUCGG 5'
AT1G48410.2     751     770
Argonaute protein (AGO1)

sRNA_AGO1_Solexa_Mi2008_1_28240_hit1
5' UCAGCAAAGAAAUGGUUAGCC
   |||||||||||||||||||||
   AGUCGUUUCUUUACCAAUCGG 5'
AT1G48410.2     751     771
Argonaute protein (AGO1)

sRNA_AGO1_Solexa_Mi2008_1_140_hit1
5' AA-AAAUGGGCCAGAACUUGA
   || ||| |:||||||||||||
   UUGUUU-CUCGGUCUUGAACU 5'
AT1G48760.3     1762    1781
putative protein

sRNA_AGO1_Solexa_Mi2008_1_37249_hit1
5' UGAUGAUUGAUG-UUGGAUGAG
   |||||||||||| || |||||
   ACUACUAACUACUAA-CUACUA 5'
AT1G48760.3     2973    2993
putative protein
```

```
sRNA_AGO1_Solexa_Mi2008_1_51445_hit1
5' UUGCAUGAU-GAUGAUUGAUGU
   ||| ||||| |||||||||||
   AAC-UACUAACUACUAACUACU 5'
AT1G48760.3     2974    2994
putative protein

sRNA_AGO1_Solexa_Mi2008_1_37249_hit1
5' UGAUGAUUGAUG-UUGGAUGAG
   |||||||||||| || |||||
   ACUACUAACUACUAA-CUACUA 5'
AT1G48760.3     2980    3000
putative protein

sRNA_AGO1_Solexa_Mi2008_1_7745_hit1
5' CCAGAAGCAUAACCGGUGGUC
    ||||||||||||||: ||||
   UGUCUUCGUAUUGGCU-CCAG 5'
AT1G48760.3     459     478
putative protein

sRNA_AGO1_Solexa_Mi2008_15_14868_hit2
5' GGAGGAGGACAUGGCGGAGG
   |||||||| ||||| |||||
   CCUCCUCCCGUACCUCCUCC 5'
AT1G49750.1     220     239
unknown protein

sRNA_AGO1_Solexa_Mi2008_3_3203_hit1
5' AUGCUUUCGGAAAUUAGGACU
   |||||||||| |||| ||||:
   UACGAAAGCCCUUAA-CCUGG 5'
AT1G49750.1     938     957
unknown protein

sRNA_AGO1_Solexa_Mi2008_2_4537_hit1
5' CAAGAAAAAGAGA-AUAAUG
   ||||||||||||| ||||::
   GUUCUUUUUCUCUAUAUUGU 5'
AT1G50360.1     169     188
myosin, putative

sRNA_AGO1_Solexa_Mi2008_2_13419_hit2
5' GAAGAAGAAGAAG-ACACUU
   ||||||||||| | | ||||
   CUUCUUCUUCUACGU-UGAA 5'
AT1G50360.1     61      79
myosin, putative

sRNA_AGO1_Solexa_Mi2008_1_53557_hit1
5' UUGUGAGUAAUA--GUGUAUGUGA
   ||||||||||||  |||| |||
   AACACUCAUUAUCGCACACACA 5'
AT1G50420.1     -1      22
scarecrow 3 -like protein

sRNA_AGO1_Solexa_Mi2008_1_12127_hit1
5' CUGGUGGUUAUCUUGUGAGUA
   |||||||||||||||||||||
   GACCACCAAUAGAACACUCAU 5'
AT1G50420.1     13      33
scarecrow 3 -like protein
```

```
sRNA_AGO1_Solexa_Mi2008_1_14022_hit2
5' GAGCUCU-UUC-UUGAUUCUA
   | ||||| ||| |||||||||
   CCCGAGAGAAGGAACUAAGAU 5'
AT1G50420.1     1613    1633
scarecrow 3 -like protein

sRNA_AGO1_Solexa_Mi2008_7_5017_hit1
5' CAAGUGCUGGGAAACAGUUUC
   ||| || ||| ||||||||||
   GUU-ACAACC-UUUGUCAAAG 5'
AT1G52000.1     888     906
null

sRNA_AGO1_Solexa_Mi2008_1_5010_hit1
5' CAAGUGCAUUAAGAACAUCAU
   |||| |||| ||||||||||:
   GUUCUCGUAUUUCUUGUAGUG 5'
AT1G52030.1     1580    1600
unknown protein

sRNA_AGO1_Solexa_Mi2008_2_9562_hit1
5' CUAAC-AAACGCUACACCGUCGU
   ||||| ||| ||||||||:||||
   GAUUGCUUU-CGAUGUGGUAGCA 5'
AT1G52030.1     1749    1770
unknown protein

sRNA_AGO1_Solexa_Mi2008_1_5010_hit1
5' CAAGUGCAUUAAGAACAUCAU
   |||| |||| ||||||||||:
   GUUCUCGUAUUUCUUGUAGUG 5'
AT1G52030.2     1779    1799
unknown protein

sRNA_AGO1_Solexa_Mi2008_2_9562_hit1
5' CUAAC-AAACGCUACACCGUCGU
   ||||| ||| ||||||||:||||
   GAUUGCUUU-CGAUGUGGUAGCA 5'
AT1G52030.2     1948    1969
unknown protein

sRNA_AGO1_Solexa_Mi2008_5_14577_hit1
5' GCC-GAGAGAUGGAAACGUU
    || ||:||||||||||||
   AGGACUUUCUACCUUUGCAA 5'
AT1G52030.2     340     359
unknown protein

sRNA_AGO1_Solexa_Mi2008_2_4629_hit1
5' CAAGAGCAUCACCAGUAAG
   ||||||| ||||||| ||
   GUUCUCGAAGUGGUC-UUG 5'
AT1G52030.1     597     614
unknown protein

sRNA_AGO1_Solexa_Mi2008_2_4629_hit1
5' CAAGAGCAUCACCAGUAAG
   ||||||| ||||||| ||
   GUUCUCGAAGUGGUC-UUG 5'
AT1G52030.2     796     813
unknown protein
```

```
sRNA_AGO1_Solexa_Mi2008_1_34289_hit2
5' UGAAAUCGAUGUUGU-AAGUCC
   :||||||||||| || ||||||
   GCUUUAGCUACAUCAUUUCAGG 5'
AT1G52400.2     1567    1588
beta-glucosidase, putative

sRNA_AGO1_Solexa_Mi2008_1_7741_hit1
5' CCAGAAAUAUUUACGUGUU
   || ||||| |||||||||:
   GG-CUUUAGAAAUGCACAG 5'
AT1G52410.1     1804    1821
myosin-like protein

sRNA_AGO1_Solexa_Mi2008_1_7741_hit1
5' CCAGAAAUAUUUACGUGUU
   || ||||| |||||||||:
   GG-CUUUAGAAAUGCACAG 5'
AT1G52410.2     1816    1833
myosin-like protein

sRNA_AGO1_Solexa_Mi2008_1_24174_hit1
5' UAGUUUUCUGAGAUCAUGUAUA
   :||||||||||||||| ||||
   GUCAAAAGACUCUAGU-CAUAC 5'
AT1G52420.1     2456    2476
glycosyl transferase, putative

sRNA_AGO1_Solexa_Mi2008_5_13886_hit2
5' GACUUGAGAGGUGUAGGAUA
   ||||||||||||| |:||||
   CUGAACUCUCCAC-UUCUAU 5'
AT1G53070.1     80      98
protein kinase, putative

sRNA_AGO1_Solexa_Mi2008_13_35730_hit2
5' UGACUUGAGAGGUGUAGGAUA
   |||||||||||||| |:||||
   ACUGAACUCUCCAC-UUCUAU 5'
AT1G53070.1     80      99
protein kinase, putative

sRNA_AGO1_Solexa_Mi2008_5_35729_hit2
5' UGACUUGAGAGGUGUAGGAU
   |||||||||||||| |:|||
   ACUGAACUCUCCAC-UUCUA 5'
AT1G53070.1     81      99
protein kinase, putative

sRNA_AGO1_Solexa_Mi2008_1_37308_hit1
5' UGAUGGAGAAGAUCAAACCUAA
   |||||| || ||||||||| ||
   ACUACCACUCCUAGUUUGG-UU 5'
AT1G53230.1     1226    1246
flower development cycloidea like protein

sRNA_AGO1_Solexa_Mi2008_6_14255_hit369
5' GAUGAUGAUGAUGAUGAUGAUGAU
   |||||||||||||||| ||| |||
   CUACUACUACUACUACCACUCCUA 5'
AT1G53230.1     1233    1256
flower development cycloidea like protein
```

```
sRNA_AGO1_Solexa_Mi2008_6_14255_hit369
5' GAUGAUGAUGAUGAUGAUGAUGAU
   ||||||||||||||||||| |||
   CUACUACUACUACUACUACCACUC 5'
AT1G53230.1     1236    1259
flower development cycloidea like protein

sRNA_AGO1_Solexa_Mi2008_1_36323_hit13
5' UGAGGAUGAUGAUGAUGAUGA
   ||| ||||||||||||| |||
   ACUACUACUACUACUACCACU 5'
AT1G53230.1     1237    1257
flower development cycloidea like protein

sRNA_AGO1_Solexa_Mi2008_1_3079_hit472
5' AUGAUGAUGAUGAUGAUGAUGA
   |||||||||||||||||| |||
   UACUACUACUACUACUACCACU 5'
AT1G53230.1     1237    1258
flower development cycloidea like protein

sRNA_AGO1_Solexa_Mi2008_1_56092_hit3
5' UUUG-UGAUGAUGAUGAUGAUGA
   :||| |||||||||||||| |||
   GAACUACUACUACUACUACCACU 5'
AT1G53230.1     1238    1260
flower development cycloidea like protein

sRNA_AGO1_Solexa_Mi2008_1_45039_hit3
5' UG-UGAUGAUGAUGAUGAUGAUGA
   || ||||||||||||||||| |||
   ACUACUACUACUACUACUACCACU 5'
AT1G53230.1     1238    1261
flower development cycloidea like protein

sRNA_AGO1_Solexa_Mi2008_9_14254_hit8
5' GAUGAUGAUGAUGAUGAUCUU
   ||||||||||||||||||  |
   CUACUACUACUACUACUACCA 5'
AT1G53230.1     1239    1259
flower development cycloidea like protein

sRNA_AGO1_Solexa_Mi2008_7_3078_hit1
5' AUGAUGAUGA-GAAUGAUGAU
   |||||||||| | |||||| |
   UACUACUACUAC-UACUACCA 5'
AT1G53230.1     1240    1259
flower development cycloidea like protein

sRNA_AGO1_Solexa_Mi2008_1_36323_hit13
5' UGAGGAUGAUGAUGAUGAUGA
   ||| ||||||||||||||||
   ACUACUACUACUACUACUACC 5'
AT1G53230.1     1240    1260
flower development cycloidea like protein

sRNA_AGO1_Solexa_Mi2008_1_3079_hit472
5' AUGAUGAUGAUGAUGAUGAUGA
    ||||||||||||||||||||
   GACUACUACUACUACUACUACC 5'
AT1G53230.1     1240    1261
flower development cycloidea like protein
```

```
sRNA_AGO1_Solexa_Mi2008_1_45039_hit3
5' UG-UGAUGAUGAUGAUGAUGAUGA
   || ||||||||||||||||||||
   ACGACUACUACUACUACUACUACC 5'
AT1G53230.1     1241    1264
flower development cycloidea like protein

sRNA_AGO1_Solexa_Mi2008_1_38921_hit1
5' UGCGGACGAUGAUGAUGAUGAU
   ||| || |||||||||||||||
   ACGACUACUACUACUACUACUA 5'
AT1G53230.1     1242    1263
flower development cycloidea like protein

sRNA_AGO1_Solexa_Mi2008_7_3078_hit1
5' AUGAUGAUGA-GAAUGAUGAU
    ||||||||| || |||||||
   CACUACUACUACU-ACUACUA 5'
AT1G53230.1     1243    1262
flower development cycloidea like protein

sRNA_AGO1_Solexa_Mi2008_3_39351_hit1
5' UGCUGAUGAUGCUGAUG-UGAC
   ||||||||||| ||||| |||
   ACGACUACUACUACUACUACUA 5'
AT1G53230.1     1243    1264
flower development cycloidea like protein

sRNA_AGO1_Solexa_Mi2008_6_14255_hit369
5' GA-UGAUGAUGAUGAUGAUGAUGAU
   || || |||||||||||||||||||
   CUGACGACUACUACUACUACUACUA 5'
AT1G53230.1     1243    1267
flower development cycloidea like protein

sRNA_AGO1_Solexa_Mi2008_1_56092_hit3
5' UUUG-UGAUGAUGAUGAUGAUGA
   | || ||||||||||||||||||
   ACACGACUACUACUACUACUACU 5'
AT1G53230.1     1244    1266
flower development cycloidea like protein

sRNA_AGO1_Solexa_Mi2008_1_8179_hit1
5' CCUGAUGAUGAUGCUGCAU
   ||||||||||||| || ||
   GGACUACUACUACUAC-UA 5'
AT1G53230.1     1246    1263
flower development cycloidea like protein

sRNA_AGO1_Solexa_Mi2008_2_31619_hit1
5' UCGCUGAUGAUGAUUGAUGAU
     |||||||||||| ||||||
   CUCGACUACUACUA-CUACUA 5'
AT1G53230.1     1246    1265
flower development cycloidea like protein

sRNA_AGO1_Solexa_Mi2008_1_39699_hit1
5' UGGAACUGAUGAUGAUGAUGA
   |||| ||| ||||||||||||
   ACCU-GACGACUACUACUACU 5'
AT1G53230.1     1250    1269
flower development cycloidea like protein
```

```
sRNA_AGO1_Solexa_Mi2008_1_28327_hit1
5' UCAGCCGCAACCACCACCUGUG
   || ||||||||||||||| ||
   AG-CGGCGUUGGUGGUGG-CAA 5'
AT1G53230.1     372     391
flower development cycloidea like protein

sRNA_AGO1_Solexa_Mi2008_1_13897_hit2
5' GAGAAAUCAAAGUUUUGGGUU
   ||||||| ||||||||  ||||
   CUCUUUAAUUUCAAAA--CCAA 5'
AT1G53230.1     689     708
flower development cycloidea like protein

sRNA_AGO1_Solexa_Mi2008_1_46444_hit1
5' UUAACUCUGUGAUUGUUU-GGU
   |||||||||||||| ||| ||:
   AAUUGAGACACUAAGAAAUCCG 5'
AT1G56010.1     1000    1021
NAC1

sRNA_AGO1_Solexa_Mi2008_1_46444_hit1
5' UUAACUCUGUGAUUGUUU-GGU
   |||||||||||||| ||| ||:
   AAUUGAGACACUAAGAAAUCCG 5'
AT1G56010.2     1025    1046
NAC1

sRNA_AGO1_Solexa_Mi2008_1_13477_hit1
5' GAAGCAGGGCACGUG--CAUU
   ||||||||| |||||  ||||
   CUUCGUCCCAUGCACGAGUAA 5'
AT1G56010.1     773     793
NAC1

sRNA_AGO1_Solexa_Mi2008_2_14844_hit1
5' GGAGAAGCAGGGCACGUG--CAUU
   |||||||||||| |||||  ||||
   CCUCUUCGUCCCAUGCACGAGUAA 5'
AT1G56010.1     773     796
NAC1

sRNA_AGO1_Solexa_Mi2008_91_40198_hit1
5' UGGAGAAGCAGGGCACGUG--CAUU
   ||||||||||||| |||||  ||||
   ACCUCUUCGUCCCAUGCACGAGUAA 5'
AT1G56010.1     773     797
NAC1

sRNA_AGO1_Solexa_Mi2008_2_13910_hit1
5' GAGAAGCAGGGCACGUGCAU
   ||||||||||| |||||| :
   CUCUUCGUCCCAUGCACGAG 5'
AT1G56010.1     775     794
NAC1

sRNA_AGO1_Solexa_Mi2008_1_14842_hit1
5' GGAGAAGCAGGGCACGUGCAA
   |||||||||||| ||||||
   CCUCUUCGUCCCAUGCACGAG 5'
AT1G56010.1     775     795
NAC1
```

```
sRNA_AGO1_Solexa_Mi2008_4_14843_hit1
5' GGAGAAGCAGGGCACGUGCAU
   |||||||||||| |||||| :
   CCUCUUCGUCCCAUGCACGAG 5'
AT1G56010.1     775     795
NAC1

sRNA_AGO1_Solexa_Mi2008_1_14846_hit1
5' GGAGAAGCAGGGCACGUGCGA
   |||||||||||| ||||||
   CCUCUUCGUCCCAUGCACGAG 5'
AT1G56010.1     775     795
NAC1

sRNA_AGO1_Solexa_Mi2008_3_40196_hit1
5' UGGAGAAGCAGGGCACGUGCAA
   ||||||||||||| ||||||
   ACCUCUUCGUCCCAUGCACGAG 5'
AT1G56010.1     775     796
NAC1

sRNA_AGO1_Solexa_Mi2008_284_40197_hit1
5' UGGAGAAGCAGGGCACGUGCAU
   ||||||||||||| |||||| :
   ACCUCUUCGUCCCAUGCACGAG 5'
AT1G56010.1     775     796
NAC1

sRNA_AGO1_Solexa_Mi2008_10440_13909_hit2
5' GAGAAGCAGGGCACGUGCA
   ||||||||||| ||||||
   CUCUUCGUCCCAUGCACGA 5'
AT1G56010.1     776     794
NAC1

sRNA_AGO1_Solexa_Mi2008_110_13911_hit1
5' GAGAAGCAGGGCACGUGCG
   ||||||||||| ||||||
   CUCUUCGUCCCAUGCACGA 5'
AT1G56010.1     776     794
NAC1

sRNA_AGO1_Solexa_Mi2008_41322_14841_hit2
5' GGAGAAGCAGGGCACGUGCA
   |||||||||||| ||||||
   CCUCUUCGUCCCAUGCACGA 5'
AT1G56010.1     776     795
NAC1

sRNA_AGO1_Solexa_Mi2008_422_14845_hit1
5' GGAGAAGCAGGGCACGUGCG
   |||||||||||| ||||||
   CCUCUUCGUCCCAUGCACGA 5'
AT1G56010.1     776     795
NAC1

sRNA_AGO1_Solexa_Mi2008_370931_40195_hit2
5' UGGAGAAGCAGGGCACGUGCA
   ||||||||||||| ||||||
   ACCUCUUCGUCCCAUGCACGA 5'
AT1G56010.1     776     796
NAC1
```

```
sRNA_AGO1_Solexa_Mi2008_7589_40199_hit1
5' UGGAGAAGCAGGGCACGUGCG
   ||||||||||||| ||||||
   ACCUCUUCGUCCCAUGCACGA 5'
AT1G56010.1     776     796
NAC1

sRNA_AGO1_Solexa_Mi2008_2_40202_hit1
5' UGGAGAAGCAGGGUACGUGCU
   ||||||||||||||||||||
   ACCUCUUCGUCCCAUGCACGA 5'
AT1G56010.1     776     796
NAC1

sRNA_AGO1_Solexa_Mi2008_1214_3232_hit1
5' AUGGAGAAGCAGGGCACGUGCA
    ||||||||||||| ||||||
   AACCUCUUCGUCCCAUGCACGA 5'
AT1G56010.1     776     797
NAC1

sRNA_AGO1_Solexa_Mi2008_24_3233_hit1
5' AUGGAGAAGCAGGGCACGUGCG
    ||||||||||||| ||||||
   AACCUCUUCGUCCCAUGCACGA 5'
AT1G56010.1     776     797
NAC1

sRNA_AGO1_Solexa_Mi2008_9_52081_hit1
5' UUGGAGAAGCAGGGCACGUGCA
   |||||||||||||| ||||||
   AACCUCUUCGUCCCAUGCACGA 5'
AT1G56010.1     776     797
NAC1

sRNA_AGO1_Solexa_Mi2008_23_14840_hit3
5' GGAGAAGCAGGGCACGUGC
   |||||||||||| ||||||
   CCUCUUCGUCCCAUGCACG 5'
AT1G56010.1     777     795
NAC1

sRNA_AGO1_Solexa_Mi2008_1_40190_hit1
5' UGGAGAAGCAGGGCACGUAA
   ||||||||||||| ||||
   ACCUCUUCGUCCCAUGCACG 5'
AT1G56010.1     777     796
NAC1

sRNA_AGO1_Solexa_Mi2008_404_40194_hit3
5' UGGAGAAGCAGGGCACGUGC
   ||||||||||||| ||||||
   ACCUCUUCGUCCCAUGCACG 5'
AT1G56010.1     777     796
NAC1

sRNA_AGO1_Solexa_Mi2008_2_40201_hit1
5' UGGAGAAGCAGGGUACGUGC
   |||||||||||||||||||
   ACCUCUUCGUCCCAUGCACG 5'
AT1G56010.1     777     796
NAC1
```

```
sRNA_AGO1_Solexa_Mi2008_9_40189_hit2
5' UGGAGAAGCAGGGCACGUA
   ||||||||||||| ||||
   ACCUCUUCGUCCCAUGCAC 5'
AT1G56010.1     778     796
NAC1

sRNA_AGO1_Solexa_Mi2008_366_40193_hit3
5' UGGAGAAGCAGGGCACGUG
   ||||||||||||| |||||
   ACCUCUUCGUCCCAUGCAC 5'
AT1G56010.1     778     796
NAC1

sRNA_AGO1_Solexa_Mi2008_1_40200_hit1
5' UGGAGAAGCAGGGUACGUG
   |||||||||||||||||||
   ACCUCUUCGUCCCAUGCAC 5'
AT1G56010.1     778     796
NAC1

sRNA_AGO1_Solexa_Mi2008_2_3231_hit2
5' AUGGAGAAGCAGGGCACGUG
    ||||||||||||| |||||
   AACCUCUUCGUCCCAUGCAC 5'
AT1G56010.1     778     797
NAC1

sRNA_AGO1_Solexa_Mi2008_1_13477_hit1
5' GAAGCAGGGCACGUG--CAUU
   ||||||||| |||||  ||||
   CUUCGUCCCAUGCACGAGUAA 5'
AT1G56010.2     798     818
NAC1

sRNA_AGO1_Solexa_Mi2008_2_14844_hit1
5' GGAGAAGCAGGGCACGUG--CAUU
   |||||||||||| |||||  ||||
   CCUCUUCGUCCCAUGCACGAGUAA 5'
AT1G56010.2     798     821
NAC1

sRNA_AGO1_Solexa_Mi2008_91_40198_hit1
5' UGGAGAAGCAGGGCACGUG--CAUU
   ||||||||||||| |||||  ||||
   ACCUCUUCGUCCCAUGCACGAGUAA 5'
AT1G56010.2     798     822
NAC1

sRNA_AGO1_Solexa_Mi2008_2_13910_hit1
5' GAGAAGCAGGGCACGUGCAU
   ||||||||||| |||||| :
   CUCUUCGUCCCAUGCACGAG 5'
AT1G56010.2     800     819
NAC1

sRNA_AGO1_Solexa_Mi2008_1_14842_hit1
5' GGAGAAGCAGGGCACGUGCAA
   |||||||||||| ||||||
   CCUCUUCGUCCCAUGCACGAG 5'
AT1G56010.2     800     820
NAC1
```

```
sRNA_AGO1_Solexa_Mi2008_4_14843_hit1
5' GGAGAAGCAGGGCACGUGCAU
   |||||||||||| |||||| :
   CCUCUUCGUCCCAUGCACGAG 5'
AT1G56010.2     800     820
NAC1

sRNA_AGO1_Solexa_Mi2008_1_14846_hit1
5' GGAGAAGCAGGGCACGUGCGA
   |||||||||||| ||||||
   CCUCUUCGUCCCAUGCACGAG 5'
AT1G56010.2     800     820
NAC1

sRNA_AGO1_Solexa_Mi2008_3_40196_hit1
5' UGGAGAAGCAGGGCACGUGCAA
   ||||||||||||| ||||||
   ACCUCUUCGUCCCAUGCACGAG 5'
AT1G56010.2     800     821
NAC1

sRNA_AGO1_Solexa_Mi2008_284_40197_hit1
5' UGGAGAAGCAGGGCACGUGCAU
   ||||||||||||| |||||| :
   ACCUCUUCGUCCCAUGCACGAG 5'
AT1G56010.2     800     821
NAC1

sRNA_AGO1_Solexa_Mi2008_10440_13909_hit2
5' GAGAAGCAGGGCACGUGCA
   ||||||||||| ||||||
   CUCUUCGUCCCAUGCACGA 5'
AT1G56010.2     801     819
NAC1

sRNA_AGO1_Solexa_Mi2008_110_13911_hit1
5' GAGAAGCAGGGCACGUGCG
   ||||||||||| ||||||
   CUCUUCGUCCCAUGCACGA 5'
AT1G56010.2     801     819
NAC1

sRNA_AGO1_Solexa_Mi2008_41322_14841_hit2
5' GGAGAAGCAGGGCACGUGCA
   |||||||||||| ||||||
   CCUCUUCGUCCCAUGCACGA 5'
AT1G56010.2     801     820
NAC1

sRNA_AGO1_Solexa_Mi2008_422_14845_hit1
5' GGAGAAGCAGGGCACGUGCG
   |||||||||||| ||||||
   CCUCUUCGUCCCAUGCACGA 5'
AT1G56010.2     801     820
NAC1

sRNA_AGO1_Solexa_Mi2008_370931_40195_hit2
5' UGGAGAAGCAGGGCACGUGCA
   ||||||||||||| ||||||
   ACCUCUUCGUCCCAUGCACGA 5'
AT1G56010.2     801     821
NAC1
```

```
sRNA_AGO1_Solexa_Mi2008_7589_40199_hit1
5' UGGAGAAGCAGGGCACGUGCG
   ||||||||||||| ||||||
   ACCUCUUCGUCCCAUGCACGA 5'
AT1G56010.2     801     821
NAC1

sRNA_AGO1_Solexa_Mi2008_2_40202_hit1
5' UGGAGAAGCAGGGUACGUGCU
   |||||||||||||||||||||
   ACCUCUUCGUCCCAUGCACGA 5'
AT1G56010.2     801     821
NAC1

sRNA_AGO1_Solexa_Mi2008_1214_3232_hit1
5' AUGGAGAAGCAGGGCACGUGCA
    ||||||||||||| ||||||
   AACCUCUUCGUCCCAUGCACGA 5'
AT1G56010.2     801     822
NAC1

sRNA_AGO1_Solexa_Mi2008_24_3233_hit1
5' AUGGAGAAGCAGGGCACGUGCG
    ||||||||||||| ||||||
   AACCUCUUCGUCCCAUGCACGA 5'
AT1G56010.2     801     822
NAC1

sRNA_AGO1_Solexa_Mi2008_9_52081_hit1
5' UUGGAGAAGCAGGGCACGUGCA
   |||||||||||||| ||||||
   AACCUCUUCGUCCCAUGCACGA 5'
AT1G56010.2     801     822
NAC1

sRNA_AGO1_Solexa_Mi2008_23_14840_hit3
5' GGAGAAGCAGGGCACGUGC
   |||||||||||| ||||||
   CCUCUUCGUCCCAUGCACG 5'
AT1G56010.2     802     820
NAC1

sRNA_AGO1_Solexa_Mi2008_1_40190_hit1
5' UGGAGAAGCAGGGCACGUAA
   ||||||||||||| ||||
   ACCUCUUCGUCCCAUGCACG 5'
AT1G56010.2     802     821
NAC1

sRNA_AGO1_Solexa_Mi2008_404_40194_hit3
5' UGGAGAAGCAGGGCACGUGC
   ||||||||||||| ||||||
   ACCUCUUCGUCCCAUGCACG 5'
AT1G56010.2     802     821
NAC1

sRNA_AGO1_Solexa_Mi2008_2_40201_hit1
5' UGGAGAAGCAGGGUACGUGC
   ||||||||||||||||||||
   ACCUCUUCGUCCCAUGCACG 5'
AT1G56010.2     802     821
NAC1
```

```
sRNA_AGO1_Solexa_Mi2008_9_40189_hit2
5' UGGAGAAGCAGGGCACGUA
   ||||||||||||| ||||
   ACCUCUUCGUCCCAUGCAC 5'
AT1G56010.2     803     821
NAC1

sRNA_AGO1_Solexa_Mi2008_366_40193_hit3
5' UGGAGAAGCAGGGCACGUG
   ||||||||||||| |||||
   ACCUCUUCGUCCCAUGCAC 5'
AT1G56010.2     803     821
NAC1

sRNA_AGO1_Solexa_Mi2008_1_40200_hit1
5' UGGAGAAGCAGGGUACGUG
   |||||||||||||||||||
   ACCUCUUCGUCCCAUGCAC 5'
AT1G56010.2     803     821
NAC1

sRNA_AGO1_Solexa_Mi2008_2_3231_hit2
5' AUGGAGAAGCAGGGCACGUG
    ||||||||||||| |||||
   AACCUCUUCGUCCCAUGCAC 5'
AT1G56010.2     803     822
NAC1

sRNA_AGO1_Solexa_Mi2008_5_12305_hit2
5' CUU-AAAGGAAUUGACGGAAGG
   ||| |||| |||||| ||||||
   GAAGUUUC-UUAACUACCUUCC 5'
AT1G60960.1     1077    1097
putative iron-regulated transporter

sRNA_AGO1_Solexa_Mi2008_1_14896_hit1
5' GGAG-UUUACGCAAAGGAAAGA
   :||| |||| ||||||||||||
   UCUCUAAAU-CGUUUCCUUUCU 5'
AT1G64390.1     1341    1361
endo-beta-1,4-glucanase-like protein

sRNA_AGO1_Solexa_Mi2008_1_13420_hit2
5' GAAGAAGAAGAAGACUCUU
   |||||||| ||||||||||
   CUUCUUCU-CUUCUGAGAA 5'
AT1G64390.1     304     321
endo-beta-1,4-glucanase-like protein

sRNA_AGO1_Solexa_Mi2008_1_27754_hit1
5' UCACCUCAUGAUGAACUUGGA
    |||| | |||||||||||||
   CGUGGUG-ACUACUUGAACCU 5'
AT1G64660.1     798     817
similar to O-succinylhomoserine sulfhydrylase

sRNA_AGO1_Solexa_Mi2008_1_14104_hit1
5' GAGGGUAAUUGGGUCAUUU
    |||||||||||||  |||
   AUCCCAUUAACCCA--AAA 5'
AT1G64710.1     1132    1148
alcohol dehydrogenase (EC 1.1.1.1) like protein
```

```
sRNA_AGO1_Solexa_Mi2008_2_41105_hit1
5' UGGCAAGUUGGUCAGGGUUGAU
   ||| || ||| |||||||||||
   ACC-UUGAAC-AGUCCCAACUA 5'
AT1G64710.2     684     703
alcohol dehydrogenase (EC 1.1.1.1) like protein

sRNA_AGO1_Solexa_Mi2008_2_41105_hit1
5' UGGCAAGUUGGUCAGGGUUGAU
   ||| || ||| |||||||||||
   ACC-UUGAAC-AGUCCCAACUA 5'
AT1G64710.1     829     848
alcohol dehydrogenase (EC 1.1.1.1) like protein

sRNA_AGO1_Solexa_Mi2008_1_14104_hit1
5' GAGGGUAAUUGGGUCAUUU
    |||||||||||||  |||
   AUCCCAUUAACCCA--AAA 5'
AT1G64710.2     987     1003
alcohol dehydrogenase (EC 1.1.1.1) like protein

sRNA_AGO1_Solexa_Mi2008_1_36546_hit1
5' UGAGGGUUGCAAGGAGUUGGU
   |||||||||| |||| ||||
   ACUCCCAACGAUCCUGAACCU 5'
AT1G65480.1     303     323
flowering time locus T (FT)

sRNA_AGO1_Solexa_Mi2008_1_37716_hit3
5' UGCAACAAAACAGGUGGAUUCU
   || |||||| ||||||| ||||
   AC-UUGUUU-GUCCACCAAAGA 5'
AT1G65480.1     54      73
flowering time locus T (FT)

sRNA_AGO1_Solexa_Mi2008_1_7340_hit1
5' CAUGUAAAACACUCUC-UUU
     |||||||||||||| |||
   UAACAUUUUGUGAGAGUAAA 5'
AT1G65480.1     774     793
flowering time locus T (FT)

sRNA_AGO1_Solexa_Mi2008_1_15859_hit1
5' GUGAGAGAGAGGUGUAA-UU
   |:||||||||:|||||| ||
   CGCUCUCUCUUCACAUUCAA 5'
AT1G66970.1     1591    1610
Glycerophosphodiesterase-like (GPDL3)

sRNA_AGO1_Solexa_Mi2008_1_341_hit1
5' AAAGAAACAGAGAGGAAGAU
   ||| ||||||||||| |||
   UUU-UUUGUCUCUCC-UCUU 5'
AT1G66970.1     2364    2381
Glycerophosphodiesterase-like (GPDL3)

sRNA_AGO1_Solexa_Mi2008_1_11311_hit1
5' CUGAA-AAAAAAAGAGAGGG
    |||| |||||||||||||
   UACUUCUUUUUUUCUCUCCA 5'
AT1G67360.2     1026    1045
unknown protein
```

```
sRNA_AGO1_Solexa_Mi2008_3_492_hit1
5' AAAGGACCAGGACAGAAUCAA
   |||| ||||||| |||||||
   UUUC-UGGUCCUCUCUUAGUC 5'
AT1G67360.1     332     351
unknown protein

sRNA_AGO1_Solexa_Mi2008_3_492_hit1
5' AAAGGACCAGGACAGAAUCAA
   |||| ||||||| |||||||
   UUUC-UGGUCCUCUCUUAGUC 5'
AT1G67360.2     430     449
unknown protein

sRNA_AGO1_Solexa_Mi2008_1_11311_hit1
5' CUGAA-AAAAAAAGAGAGGG
    |||| |||||||||||||
   UACUUCUUUUUUUCUCUCCA 5'
AT1G67360.1     928     947
unknown protein

sRNA_AGO1_Solexa_Mi2008_2_2764_hit1
5' AUCAAGAUCCAUCUUACU-CU
   ||| | |||||||||||| ||
   UAGCU-UAGGUAGAAUGAUGA 5'
AT1G67750.1     1439    1458
pectate lyase 1-like protein

sRNA_AGO1_Solexa_Mi2008_1_36753_hit1
5' UGAGUCAUGGU-CGUUUUCUGG
   ||||||||||| || ||||||
   ACUCAGUACCAGGC-AAAGACG 5'
AT1G67810.1     603     623
putative protein

sRNA_AGO1_Solexa_Mi2008_1_55298_hit1
5' UUUGAUGCGCCAC-UCUUGUAGC
   ||||||||||||| ||  |||||
   AAACUACGCGGUGCAG-UCAUCG 5'
AT1G67810.1     628     649
putative protein

sRNA_AGO1_Solexa_Mi2008_1_2360_hit1
5' AUAACAAGUCCUGUUGGGUC
   | |||||||||| |||||||
   UCUUGUUCAGGAAAACCCAG 5'
AT1G68550.1     319     338
putative AP2 domain transcription factor

sRNA_AGO1_Solexa_Mi2008_1_2360_hit1
5' AUAACAAGUCCUGUUGGGUC
   | |||||||||| |||||||
   UCUUGUUCAGGAAAACCCAG 5'
AT1G68550.2     329     348
putative AP2 domain transcription factor

sRNA_AGO1_Solexa_Mi2008_2_13457_hit1
5' GAAGAUGAAGAUGAAGU-GGUA
   |||||||| |||||||| ||:|
   CUUCUACUGCUACUUCAACCGU 5'
AT1G69295.1     1236    1257
predicted GPI-anchored protein
```

```
sRNA_AGO1_Solexa_Mi2008_1_1242_hit1
5' ACAA-AUGACGAUGAUGUUGGC
    ||| |||||||||| ||||||
   CGUUCUACUGCUACUUCAACCG 5'
AT1G69295.1     1237    1258
predicted GPI-anchored protein

sRNA_AGO1_Solexa_Mi2008_1_34620_hit1
5' UGAAGAUGAUGAUGAAGUU
   :||||||||:|||||||||
   GCUUCUACUGCUACUUCAA 5'
AT1G69295.1     1239    1257
predicted GPI-anchored protein

sRNA_AGO1_Solexa_Mi2008_1_46690_hit3
5' UUAAUA-CUUUAUGAUUAUUUA
    ||||| || ||||||||||||
   UAUUAUCGAUAUACUAAUAAAU 5'
AT1G69295.1     252     273
predicted GPI-anchored protein

sRNA_AGO1_Solexa_Mi2008_2_9402_hit1
5' CGUGGU-AGAGAUGAGUUUUUAU
   |||||| ||||||||||||||
   GCACCACUCUCUACUCAAAAACC 5'
AT1G69295.1     625     647
predicted GPI-anchored protein

sRNA_AGO1_Solexa_Mi2008_2_7120_hit1
5' CAUGAAUUUGAGGUUUACAG
   ||||||||||||:||| |||
   GUACUUAAACUCUAAA-GUC 5'
AT1G69410.1     56      74
eukaryotic initiation factor 5A (eIF-5A) like protein

sRNA_AGO1_Solexa_Mi2008_1_1110_hit1
5' AAUGCUCAGGUCGAGGUGGUCC
   ||| |||||||||||||| |||
   UUA-GAGUCCAGCUCCAC-AGG 5'
AT1G69880.1     129     148
thioredoxin like protein

sRNA_AGO1_Solexa_Mi2008_1_1109_hit1
5' AAUGCUCAGGUCGAGGUGGUC
   ||| |||||||||||||| ||
   UUA-GAGUCCAGCUCCAC-AG 5'
AT1G69880.1     130     148
thioredoxin like protein

sRNA_AGO1_Solexa_Mi2008_1_45596_hit2
5' UGUGUACA-GGCUAAGAUCUGG
    ||||| | ||||||||||||
   CCACAU-UACCGAUUCUAGACC 5'
AT1G72070.1     206     226
hypothetical protein

sRNA_AGO1_Solexa_Mi2008_23_12874_hit1
5' CUUGCGGGUGUUCUUGAUGUC
    ||| ||| |||||||||||
   AAACUCCCUCAAGAACUACAG 5'
AT1G72680.1     1184    1204
putative cinnamyl-alcohol dehydrogenase
```

```
sRNA_AGO1_Solexa_Mi2008_1_10750_hit1
5' CUC-AGUGCCUUCAUCUUCGUC
   ||| |||||||||||||  |||
   GAGAUCACGGAAGUAGAC-CAG 5'
AT1G72680.1     849     869
putative cinnamyl-alcohol dehydrogenase

sRNA_AGO1_Solexa_Mi2008_1_38353_hit4
5' UGCCAAGGAGUCGCAACUCCGA
   ||||| | |||||||| |||||
   ACGGU-C-UCAGCGUUCAGGCU 5'
AT1G73260.1     631     650
putative trypsin inhibitor (At1g73260)

sRNA_AGO1_Solexa_Mi2008_9_49410_hit1
5' UUC-GGA-GCCACUUGUGUUUGA
   ||| ||| |||||||| ||||||
   AAGACCUUCGGUGAACCCAAACU 5'
AT1G73330.1     466     488
unknown protein

sRNA_AGO1_Solexa_Mi2008_1_47836_hit2
5' UUAGG-GUUGCUGCGUUCUGGA
   || || |||||||| |||||||
   AA-CCUCAACGACGGAAGACCU 5'
AT1G73330.1     632     652
unknown protein

sRNA_AGO1_Solexa_Mi2008_1_23512_hit1
5' UAGGU-GUGAUGGAUAUGGCUG
   ||||| ||||||||||||| ||
   AUCCAUCACUACCUAUACC-AC 5'
AT1G73330.1     75      95
unknown protein

sRNA_AGO1_Solexa_Mi2008_10_14179_hit22
5' GAUAGGUGUGUAUGUGAGAAG
   ||||||||| |||||| |||
   CUAUCCACAAAUACAC-CUUG 5'
AT1G74210.1     1263    1282
glycerophosphodiester phosphodiesterase like protein

sRNA_AGO1_Solexa_Mi2008_16_36976_hit22
5' UGAUAGGUGUGUAUGUGAGAA
   |||||||||| |||||| |||
   ACUAUCCACAAAUACAC-CUU 5'
AT1G74210.1     1264    1283
glycerophosphodiester phosphodiesterase like protein

sRNA_AGO1_Solexa_Mi2008_4_36977_hit1
5' UGAUAGGUGUGUAUGUGGGAA
   |||||||||| ||||| ||||
   ACUAUCCACAAAUACA-CCUU 5'
AT1G74210.1     1264    1283
glycerophosphodiester phosphodiesterase like protein

sRNA_AGO1_Solexa_Mi2008_1_51006_hit22
5' UUGAUAGGUGUGUAUGUGAGAA
    |||||||||| |||||| |||
   CACUAUCCACAAAUACAC-CUU 5'
AT1G74210.1     1264    1284
glycerophosphodiester phosphodiesterase like protein
```

```
sRNA_AGO1_Solexa_Mi2008_1_51005_hit24
5' UUGAUAGGUGUGUAUGUGAGA
    |||||||||| |||||| ||
   CACUAUCCACAAAUACAC-CU 5'
AT1G74210.1     1265    1284
glycerophosphodiester phosphodiesterase like protein

sRNA_AGO1_Solexa_Mi2008_3_53844_hit1
5' UUGUUUCGUGGAGAAUA-AAU
   ||||||||| ||||||| |||
   AACAAAGCAGCUCUUAUGUUA 5'
AT1G74210.1     357     377
glycerophosphodiester phosphodiesterase like protein

sRNA_AGO1_Solexa_Mi2008_1_3_hit25
5' AAAAAAAAAAAAAGAAAGA
     ||||||||||| |||||
   CCUUUUUUUUUUUGUUUCU 5'
AT1G78370.1     789     807
unknown protein

sRNA_AGO1_Solexa_Mi2008_1_41610_hit1
5' UGGCUGAACGAGUUUGGCAGG
   ||||||||||||:| ||| ||
   ACCGACUUGCUCGA-CCG-CC 5'
AT1G80160.1     566     584
unknown protein

sRNA_AGO1_Solexa_Mi2008_1_5313_hit2
5' CACACGCAAU-AAAGUAGACAAG
   |||||||||| |||| ||||||
   GUGUGCGUUAGUUUC-UCUGUUA 5'
AT1G80160.1     658     679
unknown protein

sRNA_AGO1_Solexa_Mi2008_1_41610_hit1
5' UGGCUGAACGAGUUUGGCAGG
   |||||||||||| :|||| ||
   ACCGACUUGCUC-GACCG-CC 5'
AT1G80160.2     664     682
unknown protein

sRNA_AGO1_Solexa_Mi2008_1_5313_hit2
5' CACACGCAAU-AAAGUAGACAAG
   |||||||||| |||| ||||||
   GUGUGCGUUAGUUUC-UCUGUUA 5'
AT1G80160.2     756     777
unknown protein

sRNA_AGO1_Solexa_Mi2008_2_49995_hit1
5' UUGAACAGAAGUCGUCGAGGAU
   ||||||||||||||||||||||
   AACUUGUCUUCAGCAGCUCCUA 5'
AT2G04160.1     1693    1714
subtilisin-like serine protease AIR3

sRNA_AGO1_Solexa_Mi2008_1_17635_hit2
5' UAACUGAGAGGUGUUGC-UUACA
   |||||||:||||||||| || ||
   AUUGACUUUCCACAACGGAA-GU 5'
AT2G04160.1     1880    1901
subtilisin-like serine protease AIR3
```

```
sRNA_AGO1_Solexa_Mi2008_1_41483_hit1
5' UGGCGGCCAGCAGACUUUGUA
   |||||||||||||||||||||
   ACCGCCGGUCGUCUGAAACAU 5'
AT2G04160.1     831     851
subtilisin-like serine protease AIR3

sRNA_AGO1_Solexa_Mi2008_2_36903_hit1
5' UGAGUUGGGUUUCACGGUGGC
   |||||||||||||||||||||
   ACUCAACCCAAAGUGCCACCG 5'
AT2G15320.1     125     145
putative leucine-rich repeat disease resistance protein

sRNA_AGO1_Solexa_Mi2008_1_40683_hit1
5' UGGAGUCAGACGAGCAAGUGA
   |||||||||||||||||||||
   ACCUCAGUCUGCUCGUUCACU 5'
AT2G15320.1     234     254
putative leucine-rich repeat disease resistance protein

sRNA_AGO1_Solexa_Mi2008_7_14062_hit1
5' GAGGAGGAGGAGGUGAACA
   ||||||||||||| ||| |
   CUCCUCCUCCUCCUCUUCU 5'
AT2G15320.1     46      64
putative leucine-rich repeat disease resistance protein

sRNA_AGO1_Solexa_Mi2008_13_14047_hit1
5' GAGGAAGACGAGGAGGAAGAGGA
   ||||| || |||||||| |||||
   CUCCU-CUUCUCCUCCUCCUCCU 5'
AT2G15320.1     52      73
putative leucine-rich repeat disease resistance protein

sRNA_AGO1_Solexa_Mi2008_2_55158_hit1
5' UUUGAGAUGGGUCCUGAUAAA
   |||||||||||||||||||||
   AAACUCUACCCAGGACUAUUU 5'
AT2G15320.1     640     660
putative leucine-rich repeat disease resistance protein

sRNA_AGO1_Solexa_Mi2008_10_3059_hit1
5' AUGAGUUCUCCGCGAUAUU
   |||||||||| ||||| ||
   UACUCAAGAGUCGCUA-AA 5'
AT2G15320.1     700     717
putative leucine-rich repeat disease resistance protein

sRNA_AGO1_Solexa_Mi2008_2_34868_hit1
5' UGAAGUC-CGGUUCUCGCAUGGU
   ||||||| ||||||||||||  |
   ACUUCAGUGCCAAGAGCGUAGGA 5'
AT2G15490.2     355     377
putative glucosyltransferase

sRNA_AGO1_Solexa_Mi2008_1_48518_hit2
5' UUCAAGGACAAGUCACAUGAGC
   ||||||:|||||||| |||||
   AAGUUCUUGUUCAGUUUACUCA 5'
AT2G15490.2     398     419
putative glucosyltransferase
```

```
sRNA_AGO1_Solexa_Mi2008_3_11240_hit1
5' CUCUCUCAUGUUAUACAGA
    || |||||||||||| ||
   AAG-GAGUACAAUAUG-CU 5'
AT2G15490.2     593     609
putative glucosyltransferase

sRNA_AGO1_Solexa_Mi2008_10_8366_hit1
5' CGAAUGGAGUUUGC-ACGUU
   |||||||||||| |  ||||
   GCUUACCUCAAA-GAAGCAA 5'
AT2G15490.1     710     728
putative glucosyltransferase

sRNA_AGO1_Solexa_Mi2008_1_45020_hit16
5' UGUGAU-CAGCAAAGACCAGCUG
   |||| | ||| ||||||||||||
   ACACAAUGUC-UUUCUGGUCGAC 5'
AT2G16640.1     3270    3291
putative chloroplast outer membrane protein

sRNA_AGO1_Solexa_Mi2008_1_10880_hit1
5' CUCCC-GUAUUCUCGAUAAG
   ||||| | |||||||||||:
   GAGGGGC-UAAGAGCUAUUU 5'
AT2G17040.1     159     177
NAM (no apical meristem)-like protein

sRNA_AGO1_Solexa_Mi2008_1_33653_hit1
5' UCUGGUAAACCUCCAAGAUGGU
   |||||||||| ||||| |:|||
   AGACCAUUUGAAGGUU-UGCCA 5'
AT2G17040.1     714     734
NAM (no apical meristem)-like protein

sRNA_AGO1_Solexa_Mi2008_1_2_hit38
5' AAAAA-AAAA-AAAAAACCAU
   ||||| |||| ||||||||||
   UUUUUGUUUUGUUUUUUGGUA 5'
AT2G17130.2     1319    1339
NAD+ dependent isocitrate dehydrogenase subunit 2 like, IDH2

sRNA_AGO1_Solexa_Mi2008_1_2_hit38
5' AAAAA-AAAA-AAAAAACCAU
   ||||| |||| ||||||||||
   UUUUUGUUUUGUUUUUUGGUA 5'
AT2G17130.1     1331    1351
NAD+ dependent isocitrate dehydrogenase subunit 2 like, IDH2

sRNA_AGO1_Solexa_Mi2008_1_34613_hit1
5' UGAAGAUGAAGAUGAGUUGU
    ||||||||||| ||||||:
   UCUUCUACUUCUUCUCAACG 5'
AT2G17130.2     13      32
NAD+ dependent isocitrate dehydrogenase subunit 2 like, IDH2

sRNA_AGO1_Solexa_Mi2008_1_19461_hit1
5' UACAGCUGUGUCAUGGUACCU
   ||||||||||||||||||||
   AUGUCGACACAGUACCAUGGC 5'
AT2G17500.4     1216    1236
unknown protein
```

```
sRNA_AGO1_Solexa_Mi2008_1_19461_hit1
5' UACAGCUGUGUCAUGGUACCU
   ||||||||||||||||||||
   AUGUCGACACAGUACCAUGGC 5'
AT2G17500.3     1223    1243
unknown protein

sRNA_AGO1_Solexa_Mi2008_1_19461_hit1
5' UACAGCUGUGUCAUGGUACCU
   ||||||||||||||||||||
   AUGUCGACACAGUACCAUGGC 5'
AT2G17500.1     1278    1298
unknown protein

sRNA_AGO1_Solexa_Mi2008_1_19461_hit1
5' UACAGCUGUGUCAUGGUACCU
   ||||||||||||||||||||
   AUGUCGACACAGUACCAUGGC 5'
AT2G17500.2     1295    1315
unknown protein

sRNA_AGO1_Solexa_Mi2008_26_44780_hit1
5' UGUCGUGUCUUUGAGUGUCCAA
   ||||||||||| ||| || |||
   ACAGCACAGAACCUCCCA-GUU 5'
AT2G18960.1     1684    1704
plasma membrane proton ATPase  (PMA)

sRNA_AGO1_Solexa_Mi2008_1_51592_hit1
5' UUGCCGUAGUUGAU-GAAGAGC
   ||||||:||||| | |||||||
   AACGGCGUCAAC-AGCUUCUCG 5'
AT2G20610.2     255     275
tyrosine aminotransferase like protein

sRNA_AGO1_Solexa_Mi2008_1_52954_hit1
5' UUGGUCCAUGGUGUUUU-GACA
   ||| ||||||||||||| || |
   AAC-AGGUACCACAAAAACUUU 5'
AT2G21640.1     238     258
unknown protein

sRNA_AGO1_Solexa_Mi2008_1_21384_hit1
5' UAGAGGAGGCGCGUUGACGGU
   |||||||||||||||||||||
   AUCUCCUCCGCGCAACUGCCA 5'
AT2G22500.1     438     458
putative mitochondrial dicarboxylate carrier protein

sRNA_AGO1_Solexa_Mi2008_2_4570_hit2
5' CAAGAAGAGAUUCCAU-AGU
   |||||||||| ||||| |||
   GUUCUUCUCUUAGGUAGUCA 5'
AT2G22840.1     233     252
unknown protein

sRNA_AGO1_Solexa_Mi2008_3_5131_hit1
5' CAAUGAAAAAGGGCCUAUU-CUC
   |||||||||||| ||| || |||
   GUUACUUUUUCCAGGAAAAUGAG 5'
AT2G22840.1     471     493
unknown protein
```

```
sRNA_AGO1_Solexa_Mi2008_9_5355_hit1
5' CACAG-CUUUCUUGAACUUU
   ||||| |||||||||||  |
   GUGUCCGAAAGAACUUGCUA 5'
AT2G22840.1     780     799
unknown protein

sRNA_AGO1_Solexa_Mi2008_138_7679_hit1
5' CCACAG-CUUUCUUGAACUG
   |||||| ||||||||||| :
   GGUGUCCGAAAGAACUUGCU 5'
AT2G22840.1     781     800
unknown protein

sRNA_AGO1_Solexa_Mi2008_54_7680_hit1
5' CCACAG-CUUUCUUGAACUU
   |||||| |||||||||||
   GGUGUCCGAAAGAACUUGCU 5'
AT2G22840.1     781     800
unknown protein

sRNA_AGO1_Solexa_Mi2008_18_29330_hit1
5' UCCACAG-CUUUCUUGAACUG
   ||||||| ||||||||||| :
   AGGUGUCCGAAAGAACUUGCU 5'
AT2G22840.1     781     801
unknown protein

sRNA_AGO1_Solexa_Mi2008_11_29331_hit1
5' UCCACAG-CUUUCUUGAACUU
   ||||||| |||||||||||
   AGGUGUCCGAAAGAACUUGCU 5'
AT2G22840.1     781     801
unknown protein

sRNA_AGO1_Solexa_Mi2008_1449_48967_hit1
5' UUCCACAG-CUUUCUUGAACUG
   |||||||| ||||||||||| :
   AAGGUGUCCGAAAGAACUUGCU 5'
AT2G22840.1     781     802
unknown protein

sRNA_AGO1_Solexa_Mi2008_809_48968_hit1
5' UUCCACAG-CUUUCUUGAACUU
   |||||||| |||||||||||
   AAGGUGUCCGAAAGAACUUGCU 5'
AT2G22840.1     781     802
unknown protein

sRNA_AGO1_Solexa_Mi2008_9_12589_hit1
5' CUUCCACAG-CUUUCUUGAACUG
   ||||||||| ||||||||||| :
   GAAGGUGUCCGAAAGAACUUGCU 5'
AT2G22840.1     781     803
unknown protein

sRNA_AGO1_Solexa_Mi2008_8_48966_hit2
5' UUCCACAG-CUUUCUUGAACU
   |||||||| |||||||||||
   AAGGUGUCCGAAAGAACUUGC 5'
AT2G22840.1     782     802
unknown protein
```

```
sRNA_AGO1_Solexa_Mi2008_5_12588_hit1
5' CUUCCACAG-CUUUCUUGAACU
   ||||||||| ||||||||||| 
   GAAGGUGUCCGAAAGAACUUGC 5'
AT2G22840.1     782     803
unknown protein

sRNA_AGO1_Solexa_Mi2008_1_42987_hit1
5' UGGUAUGAUGAUGUGUUACUCA
   |||||||||| |||||| | ||
   ACCAUACUACAACACAA-G-GU 5'
AT2G22840.1     949     968
unknown protein

sRNA_AGO1_Solexa_Mi2008_6_14255_hit369
5' GAUGAUGAUGAUGAUGAUGAUGAU
   ||||||||||||||||||:||| :
   CUACUACUACUACUACUAUUACCG 5'
AT2G25110.1     157     180
unknown protein

sRNA_AGO1_Solexa_Mi2008_1_14256_hit8
5' GAUGAUGAUGAUGAUGAUGAUGUU
   ||||||||||||||||||:||| :
   CUACUACUACUACUACUAUUACCG 5'
AT2G25110.1     157     180
unknown protein

sRNA_AGO1_Solexa_Mi2008_1_36323_hit13
5' UGAGGAUGAUGAUGAUGAUGA
   ||| ||||||||||||:|||
   ACUACUACUACUACUAUUACC 5'
AT2G25110.1     158     178
unknown protein

sRNA_AGO1_Solexa_Mi2008_1_3079_hit472
5' AUGAUGAUGAUGAUGAUGAUGA
   |||||||||||||||||:|||
   UACUACUACUACUACUAUUACC 5'
AT2G25110.1     158     179
unknown protein

sRNA_AGO1_Solexa_Mi2008_9_14254_hit8
5' GAUGAUGAUGAUGAUGAUCUU
   |||||||||||||||||  |
   CUACUACUACUACUACUAUUA 5'
AT2G25110.1     160     180
unknown protein

sRNA_AGO1_Solexa_Mi2008_1_34544_hit1
5' UGAAGACGAAGAUGAUGAUGAUA
   |||||| || |||||||||||||
   ACUUCUACUACUACUACUACUAU 5'
AT2G25110.1     162     184
unknown protein

sRNA_AGO1_Solexa_Mi2008_1_2280_hit1
5' AG-UGGAUGAUGAUGAUGAUG
   || || ||||||||||||||:
   UCUAC-UACUACUACUACUAU 5'
AT2G25110.1     163     182
unknown protein
```

```
sRNA_AGO1_Solexa_Mi2008_6_14255_hit369
5' GAUGAUGAUGAUGAUGAUGAUGAU
   ||||| ||||||||||||||||||
   CUACUUCUACUACUACUACUACUA 5'
AT2G25110.1     163     186
unknown protein

sRNA_AGO1_Solexa_Mi2008_1_14256_hit8
5' GAUGAUGAUGAUGAUGAUGAUGUU
   ||||| |||||||||||||||| |
   CUACUUCUACUACUACUACUACUA 5'
AT2G25110.1     163     186
unknown protein

sRNA_AGO1_Solexa_Mi2008_7_3078_hit1
5' AUGAUGAUGA-GAAUGAUGAU
    ||||||||| || |||||||
   AACUACUACUACU-ACUACUA 5'
AT2G25110.1     164     183
unknown protein

sRNA_AGO1_Solexa_Mi2008_1_36323_hit13
5' UGAGGAUGAUGAUGAUGAUGA
   |||:|||||||||||||||||
   ACUUCUACUACUACUACUACU 5'
AT2G25110.1     164     184
unknown protein

sRNA_AGO1_Solexa_Mi2008_1_3079_hit472
5' AUGAUGAUGAUGAUGAUGAUGA
   |||| |||||||||||||||||
   UACUUCUACUACUACUACUACU 5'
AT2G25110.1     164     185
unknown protein

sRNA_AGO1_Solexa_Mi2008_1_36898_hit1
5' UGA-GUUGGAUGAUGAUGAUGA
   ||| | || |||||||||||||
   ACUUCUAC-UACUACUACUACU 5'
AT2G25110.1     165     185
unknown protein

sRNA_AGO1_Solexa_Mi2008_1_45039_hit3
5' UG-UGAUGAUGAUGAUGAUGAUGA
   || ||| |||||||||||||||||
   ACUACUUCUACUACUACUACUACU 5'
AT2G25110.1     165     188
unknown protein

sRNA_AGO1_Solexa_Mi2008_6_14255_hit369
5' GAUGAUGAUGAUGAUGAUGAUGAU
   |||||||| |||||||||||||||
   CUACUACUUCUACUACUACUACUA 5'
AT2G25110.1     166     189
unknown protein

sRNA_AGO1_Solexa_Mi2008_1_14256_hit8
5' GAUGAUGAUGAUGAUGAUGAUGUU
   |||||||| ||||||||||||| |
   CUACUACUUCUACUACUACUACUA 5'
AT2G25110.1     166     189
unknown protein
```

```
sRNA_AGO1_Solexa_Mi2008_7_3078_hit1
5' AUGAUGAUGA-GAAUGAUGAU
   |||| ||||| || |||||||
   UACUUCUACUACU-ACUACUA 5'
AT2G25110.1     167     186
unknown protein

sRNA_AGO1_Solexa_Mi2008_1_3079_hit472
5' AUGAUGAUGAUGAUGAUGAUGA
   ||||||| ||||||||||||||
   UACUACUUCUACUACUACUACU 5'
AT2G25110.1     167     188
unknown protein

sRNA_AGO1_Solexa_Mi2008_1_38238_hit1
5' UGCAUGAACAUGAUGAUGAUGG
   || ||||| ||||||||||||:
   AC-UACUUCUACUACUACUACU 5'
AT2G25110.1     168     188
unknown protein

sRNA_AGO1_Solexa_Mi2008_3_14271_hit18
5' GAUGCUGAAUAUGAUGAUGA-GA
   |||| |||| |||||||||| ||
   CUACUACUUCUACUACUACUACU 5'
AT2G25110.1     168     190
unknown protein

sRNA_AGO1_Solexa_Mi2008_1_56092_hit3
5' UUUG-UGAUGAUGAUGAUGAUGA
   :||| ||| ||||||||||||||
   GAACUACUUCUACUACUACUACU 5'
AT2G25110.1     168     190
unknown protein

sRNA_AGO1_Solexa_Mi2008_1_3079_hit472
5' AUGAUGAUGAUGAUGAUGAUGA
     |||||||| |||||||||||
   CUCUACUACUUCUACUACUACU 5'
AT2G25110.1     170     191
unknown protein

sRNA_AGO1_Solexa_Mi2008_1_2280_hit1
5' AG-UGGAUGAUGAUGAUGAUG
   || || |||| ||||||||||
   UCUAC-UACUUCUACUACUAC 5'
AT2G25110.1     172     191
unknown protein

sRNA_AGO1_Solexa_Mi2008_1_41920_hit17
5' UGG-GAUGAUGAAGUUGAUGAU
   ||| |||||||||| |||||||
   ACCUCUACUACUUCUACUACUA 5'
AT2G25110.1     173     194
unknown protein

sRNA_AGO1_Solexa_Mi2008_1_2638_hit1
5' AUAGGUUGUGCAGUUUGAGCU
   ||||| | |||||||||||||
   UAUCCUAGACGUCAAACUCGA 5'
AT2G25490.1     1962    1982
putative glucose regulated repressor protein
```

```
sRNA_AGO1_Solexa_Mi2008_2_26713_hit3
5' UC-AAAGCCAAAUCAAUAUCAC
   || ||||| |||||||||||
   AGCUUUCGCUUUAGUUAUAGUA 5'
AT2G26530.2     431     452
AR781, similar to yeast pheromone receptor

sRNA_AGO1_Solexa_Mi2008_1_10600_hit3
5' CUC-AAAGCCAAAUCAAUAUCAC
   ||| ||||| |||||||||||
   GAGCUUUCGCUUUAGUUAUAGUA 5'
AT2G26530.2     431     453
AR781, similar to yeast pheromone receptor

sRNA_AGO1_Solexa_Mi2008_3_26712_hit3
5' UC-AAAGCCAAAUCAAUAUCA
   || ||||| |||||||||||
   AGCUUUCGCUUUAGUUAUAGU 5'
AT2G26530.2     432     452
AR781, similar to yeast pheromone receptor

sRNA_AGO1_Solexa_Mi2008_2_26713_hit3
5' UC-AAAGCCAAAUCAAUAUCAC
   || ||||| |||||||||||
   AGCUUUCGCUUUAGUUAUAGUA 5'
AT2G26530.1     449     470
AR781, similar to yeast pheromone receptor

sRNA_AGO1_Solexa_Mi2008_1_10600_hit3
5' CUC-AAAGCCAAAUCAAUAUCAC
   ||| ||||| |||||||||||
   GAGCUUUCGCUUUAGUUAUAGUA 5'
AT2G26530.1     449     471
AR781, similar to yeast pheromone receptor

sRNA_AGO1_Solexa_Mi2008_3_26712_hit3
5' UC-AAAGCCAAAUCAAUAUCA
   || ||||| |||||||||||
   AGCUUUCGCUUUAGUUAUAGU 5'
AT2G26530.1     450     470
AR781, similar to yeast pheromone receptor

sRNA_AGO1_Solexa_Mi2008_1_14490_hit1
5' GCAUCAAUGAUAGUGUUGUAG
   ||||||||||||| | |||||
   CGUAGUUACUAUC-C-ACAUC 5'
AT2G26560.1     961     979
similar to latex allergen from Hevea brasiliensis

sRNA_AGO1_Solexa_Mi2008_1_806_hit1
5' AAGAGCAUCAAUGAUAG-UGUU
   || |||||||||||||| |||
   UU-UCGUAGUUACUAUCCACAU 5'
AT2G26560.1     962     982
similar to latex allergen from Hevea brasiliensis

sRNA_AGO1_Solexa_Mi2008_1_50014_hit1
5' UUGAA-CGACCGAGUAAGGAGU
   ||||| || ||||| |||||||
   AACUUUGCCGGCUCCUUCCUCA 5'
AT2G26690.1     346     367
putative nitrate transporter
```

```
sRNA_AGO1_Solexa_Mi2008_2_15799_hit1
5' GUCUGAAGGAUUAGAGGAAC
   || ||||||||| ||||||
   CA-ACUUCCUAACCUCCUUC 5'
AT2G28190.1     109     127
putative copper/zinc superoxide dismutase

sRNA_AGO1_Solexa_Mi2008_3_29723_hit1
5' UCCCCUUACAAUGUCGAG-UAA
   | ||||| |||||||||| |||
   AUGGGAAGGUUACAGCUCAAUU 5'
AT2G30140.2     1103    1124
putative glucosyltransferase

sRNA_AGO1_Solexa_Mi2008_3_29723_hit1
5' UCCCCUUACAAUGUCGAGU-AA
   | ||||| ||||||||||| ||
   AUGGGAAGGUUACAGCUCAAUU 5'
AT2G30140.1     1106    1127
putative glucosyltransferase

sRNA_AGO1_Solexa_Mi2008_1_53677_hit1
5' UUGUGUAGAGUGUGAU-UUGGU
   ||||| || ||||||| |||||
   AACACUUCACACACUAAAACCA 5'
AT2G30140.2     35      56
putative glucosyltransferase

sRNA_AGO1_Solexa_Mi2008_1_19407_hit2
5' UACAGAGUCGCCGGAGAUGGU
   || ||||||||||||| |||
   AU-UCUCAGCGGCCUCAACCU 5'
AT2G31190.1     26      45
unknown protein

sRNA_AGO1_Solexa_Mi2008_1_38236_hit3
5' UGCAU-CUGUUGUUGGUGUUGC
   ||| | |||:||||||||||||
   ACG-ACGACGACAACCACAACG 5'
AT2G31370.4     1254    1274
bZIP transcription factor PosF21 / AtbZip59

sRNA_AGO1_Solexa_Mi2008_1_38236_hit3
5' UGCAU-CUGUUGUUGGUGUUGC
   ||| | |||:||||||||||||
   ACG-ACGACGACAACCACAACG 5'
AT2G31370.3     1299    1319
bZIP transcription factor PosF21 / AtbZip59

sRNA_AGO1_Solexa_Mi2008_1_38236_hit3
5' UGCAU-CUGUUGUUGGUGUUGC
   ||| | |||:||||||||||||
   ACG-ACGACGACAACCACAACG 5'
AT2G31370.1     1351    1371
bZIP transcription factor PosF21 / AtbZip59

sRNA_AGO1_Solexa_Mi2008_1_27099_hit1
5' UCAAGAUGCUUGAGGUUGAUG
   ||||||||||||||||||||
   AGUUCUACGAACUCCAACUAC 5'
AT2G32680.1     1016    1036
putative disease resistance protein
```

```
sRNA_AGO1_Solexa_Mi2008_1_32535_hit1
5' UCGUGCGUGAUGAUGCUUUCC
   |||||||||||||||||||||
   AGCACGCACUACUACGAAAGG 5'
AT2G32680.1     1951    1971
putative disease resistance protein

sRNA_AGO1_Solexa_Mi2008_1_48497_hit1
5' UUCAA-GAGGCAACUGUUUGGA
    |||| ||||| ||||||||||
   CAGUUUCUCCGAUGACAAACCU 5'
AT2G32680.1     561     582
putative disease resistance protein

sRNA_AGO1_Solexa_Mi2008_11_14876_hit4
5' GGAGGUGGUGG-UGG-UGGUGGU
   ||||||||||| ||| ||||| |
   CCUCCACCACCAACCUACCACAA 5'
AT2G33770.1     108     130
ubiquitin-conjugating enzyme E2 -like protein

sRNA_AGO1_Solexa_Mi2008_24_502_hit1
5' AAAGGAGGUGGUGGUUG-AU
   | ||||||||||||||| ||
   UCUCCUCCACCACCAACCUA 5'
AT2G33770.1     114     133
ubiquitin-conjugating enzyme E2 -like protein

sRNA_AGO1_Solexa_Mi2008_1_28360_hit2
5' UCAGCGGCGGAUCC-CACAAUGU
   |||| |||||| || ||||||||
   AGUCACCGCCU-GGCGUGUUACA 5'
AT2G33770.1     1331    1352
ubiquitin-conjugating enzyme E2 -like protein

sRNA_AGO1_Solexa_Mi2008_2_10859_hit1
5' CUCCAUCUCUCUCUCUGCUU
   |||| |||||||||||| ||
   GAGGAAGAGAGAGAGAC-AA 5'
AT2G33770.1     175     193
ubiquitin-conjugating enzyme E2 -like protein

sRNA_AGO1_Solexa_Mi2008_4_38337_hit1
5' UGCCAAAGGAGAUUUGCCCUGU
   ||||||||:|||||||||||:
   ACGGUUUCUUCUAAACGGGAUU 5'
AT2G33770.1     605     626
ubiquitin-conjugating enzyme E2 -like protein

sRNA_AGO1_Solexa_Mi2008_1041_38329_hit2
5' UGCCAAAGGAGAGUUGCCCUG
   ||||||||:||| |||||||:
   ACGGUUUCUUCUAAACGGGAU 5'
AT2G33770.1     606     626
ubiquitin-conjugating enzyme E2 -like protein

sRNA_AGO1_Solexa_Mi2008_2_38332_hit1
5' UGCCAAAGGAGAUUUGCCCCG
   ||||||||:|||||||||| :
   ACGGUUUCUUCUAAACGGGAU 5'
AT2G33770.1     606     626
ubiquitin-conjugating enzyme E2 -like protein
```

```
sRNA_AGO1_Solexa_Mi2008_92_38333_hit1
5' UGCCAAAGGAGAUUUGCCCGG
   ||||||||:|||||||||| :
   ACGGUUUCUUCUAAACGGGAU 5'
AT2G33770.1     606     626
ubiquitin-conjugating enzyme E2 -like protein

sRNA_AGO1_Solexa_Mi2008_101_38336_hit1
5' UGCCAAAGGAGAUUUGCCCUG
   ||||||||:|||||||||||:
   ACGGUUUCUUCUAAACGGGAU 5'
AT2G33770.1     606     626
ubiquitin-conjugating enzyme E2 -like protein

sRNA_AGO1_Solexa_Mi2008_74_51466_hit1
5' UUGCCAAAGGAGAGUUGCCCUG
   |||||||||:||| |||||||:
   AACGGUUUCUUCUAAACGGGAU 5'
AT2G33770.1     606     627
ubiquitin-conjugating enzyme E2 -like protein

sRNA_AGO1_Solexa_Mi2008_7_38328_hit2
5' UGCCAAAGGAGAGUUGCCCU
   ||||||||:||| |||||||
   ACGGUUUCUUCUAAACGGGA 5'
AT2G33770.1     607     626
ubiquitin-conjugating enzyme E2 -like protein

sRNA_AGO1_Solexa_Mi2008_2_38335_hit1
5' UGCCAAAGGAGAUUUGCCCU
   ||||||||:|||||||||||
   ACGGUUUCUUCUAAACGGGA 5'
AT2G33770.1     607     626
ubiquitin-conjugating enzyme E2 -like protein

sRNA_AGO1_Solexa_Mi2008_1_51465_hit1
5' UUGCCAAAGGAGAGUUGCCCU
   |||||||||:||| |||||||
   AACGGUUUCUUCUAAACGGGA 5'
AT2G33770.1     607     627
ubiquitin-conjugating enzyme E2 -like protein

sRNA_AGO1_Solexa_Mi2008_3_38327_hit2
5' UGCCAAAGGAGAGUUGCCC
   ||||||||:||| ||||||
   ACGGUUUCUUCUAAACGGG 5'
AT2G33770.1     608     626
ubiquitin-conjugating enzyme E2 -like protein

sRNA_AGO1_Solexa_Mi2008_1_38331_hit4
5' UGCCAAAGGAGAUUUGCCC
   ||||||||:||||||||||
   ACGGUUUCUUCUAAACGGG 5'
AT2G33770.1     608     626
ubiquitin-conjugating enzyme E2 -like protein

sRNA_AGO1_Solexa_Mi2008_4_38337_hit1
5' UGCCAAAGGAGAUUUGCCCUGU
   ||||||||||||| ||||||:
   ACGGUUUCCUCUAUACGGGAUC 5'
AT2G33770.1     738     759
ubiquitin-conjugating enzyme E2 -like protein
```

```
sRNA_AGO1_Solexa_Mi2008_2_38332_hit1
5' UGCCAAAGGAGAUUUGCCCCG
   ||||||||||||| ||||| :
   ACGGUUUCCUCUAUACGGGAU 5'
AT2G33770.1     739     759
ubiquitin-conjugating enzyme E2 -like protein

sRNA_AGO1_Solexa_Mi2008_92_38333_hit1
5' UGCCAAAGGAGAUUUGCCCGG
   ||||||||||||| ||||| :
   ACGGUUUCCUCUAUACGGGAU 5'
AT2G33770.1     739     759
ubiquitin-conjugating enzyme E2 -like protein

sRNA_AGO1_Solexa_Mi2008_101_38336_hit1
5' UGCCAAAGGAGAUUUGCCCUG
   ||||||||||||| ||||||:
   ACGGUUUCCUCUAUACGGGAU 5'
AT2G33770.1     739     759
ubiquitin-conjugating enzyme E2 -like protein

sRNA_AGO1_Solexa_Mi2008_2_7575_hit2
5' CCAAAGGAGAGU-UGCCCUG
   |||||||||| | ||||||:
   GGUUUCCUCU-AUACGGGAU 5'
AT2G33770.1     740     758
ubiquitin-conjugating enzyme E2 -like protein

sRNA_AGO1_Solexa_Mi2008_26_14499_hit2
5' GCCAAAGGAGAGU-UGCCCUG
   ||||||||||| | ||||||:
   CGGUUUCCUCU-AUACGGGAU 5'
AT2G33770.1     740     759
ubiquitin-conjugating enzyme E2 -like protein

sRNA_AGO1_Solexa_Mi2008_2_38335_hit1
5' UGCCAAAGGAGAUUUGCCCU
   ||||||||||||| ||||||
   ACGGUUUCCUCUAUACGGGA 5'
AT2G33770.1     740     759
ubiquitin-conjugating enzyme E2 -like protein

sRNA_AGO1_Solexa_Mi2008_1041_38329_hit2
5' UGCCAAAGGAGAGU-UGCCCUG
   |||||||||||| | ||||||:
   ACGGUUUCCUCU-AUACGGGAU 5'
AT2G33770.1     740     760
ubiquitin-conjugating enzyme E2 -like protein

sRNA_AGO1_Solexa_Mi2008_74_51466_hit1
5' UUGCCAAAGGAGAGU-UGCCCUG
   ||||||||||||| | ||||||:
   AACGGUUUCCUCU-AUACGGGAU 5'
AT2G33770.1     740     761
ubiquitin-conjugating enzyme E2 -like protein

sRNA_AGO1_Solexa_Mi2008_1_38331_hit4
5' UGCCAAAGGAGAUUUGCCC
   ||||||||||||| |||||
   ACGGUUUCCUCUAUACGGG 5'
AT2G33770.1     741     759
ubiquitin-conjugating enzyme E2 -like protein
```

```
sRNA_AGO1_Solexa_Mi2008_7_38328_hit2
5' UGCCAAAGGAGAGU-UGCCCU
   |||||||||||| | ||||||
   ACGGUUUCCUCU-AUACGGGA 5'
AT2G33770.1     741     760
ubiquitin-conjugating enzyme E2 -like protein

sRNA_AGO1_Solexa_Mi2008_1_51465_hit1
5' UUGCCAAAGGAGAGU-UGCCCU
   ||||||||||||| | ||||||
   AACGGUUUCCUCU-AUACGGGA 5'
AT2G33770.1     741     761
ubiquitin-conjugating enzyme E2 -like protein

sRNA_AGO1_Solexa_Mi2008_3_38327_hit2
5' UGCCAAAGGAGAGU-UGCCC
   |||||||||||| | |||||
   ACGGUUUCCUCU-AUACGGG 5'
AT2G33770.1     742     760
ubiquitin-conjugating enzyme E2 -like protein

sRNA_AGO1_Solexa_Mi2008_1_26738_hit1
5' UCAAAGGGGAACCCAAGAUGUG
   ||||||||||||||||||||||
   AGUUUCCCCUUGGGUUCUACAC 5'
AT2G33770.1     774     795
ubiquitin-conjugating enzyme E2 -like protein

sRNA_AGO1_Solexa_Mi2008_6_14501_hit1
5' GCCAAAGGAGAUUUGCCCGGU
   ||||||||||||||||||:::
   CGGUUUCCUCUAAACGGGUUG 5'
AT2G33770.1     827     847
ubiquitin-conjugating enzyme E2 -like protein

sRNA_AGO1_Solexa_Mi2008_4_38334_hit1
5' UGCCAAAGGAGAUUUGCCCGGU
   |||||||||||||||||||:::
   ACGGUUUCCUCUAAACGGGUUG 5'
AT2G33770.1     827     848
ubiquitin-conjugating enzyme E2 -like protein

sRNA_AGO1_Solexa_Mi2008_4_38337_hit1
5' UGCCAAAGGAGAUUUGCCCUGU
   ||||||||||||||||||| ::
   ACGGUUUCCUCUAAACGGGUUG 5'
AT2G33770.1     827     848
ubiquitin-conjugating enzyme E2 -like protein

sRNA_AGO1_Solexa_Mi2008_2_7575_hit2
5' CCAAAGGAGAGUUGCCCUG
   |||||||||| |||||| :
   GGUUUCCUCUAAACGGGUU 5'
AT2G33770.1     828     846
ubiquitin-conjugating enzyme E2 -like protein

sRNA_AGO1_Solexa_Mi2008_26_14499_hit2
5' GCCAAAGGAGAGUUGCCCUG
   ||||||||||| |||||| :
   CGGUUUCCUCUAAACGGGUU 5'
AT2G33770.1     828     847
ubiquitin-conjugating enzyme E2 -like protein
```

```
sRNA_AGO1_Solexa_Mi2008_1041_38329_hit2
5' UGCCAAAGGAGAGUUGCCCUG
   |||||||||||| |||||| :
   ACGGUUUCCUCUAAACGGGUU 5'
AT2G33770.1     828     848
ubiquitin-conjugating enzyme E2 -like protein

sRNA_AGO1_Solexa_Mi2008_2_38332_hit1
5' UGCCAAAGGAGAUUUGCCCCG
   |||||||||||||||||| :
   ACGGUUUCCUCUAAACGGGUU 5'
AT2G33770.1     828     848
ubiquitin-conjugating enzyme E2 -like protein

sRNA_AGO1_Solexa_Mi2008_92_38333_hit1
5' UGCCAAAGGAGAUUUGCCCGG
   |||||||||||||||||||::
   ACGGUUUCCUCUAAACGGGUU 5'
AT2G33770.1     828     848
ubiquitin-conjugating enzyme E2 -like protein

sRNA_AGO1_Solexa_Mi2008_101_38336_hit1
5' UGCCAAAGGAGAUUUGCCCUG
   |||||||||||||||||| :
   ACGGUUUCCUCUAAACGGGUU 5'
AT2G33770.1     828     848
ubiquitin-conjugating enzyme E2 -like protein

sRNA_AGO1_Solexa_Mi2008_7_38328_hit2
5' UGCCAAAGGAGAGUUGCCCU
   |||||||||||| ||||||
   ACGGUUUCCUCUAAACGGGU 5'
AT2G33770.1     829     848
ubiquitin-conjugating enzyme E2 -like protein

sRNA_AGO1_Solexa_Mi2008_2_38335_hit1
5' UGCCAAAGGAGAUUUGCCCU
   ||||||||||||||||||
   ACGGUUUCCUCUAAACGGGU 5'
AT2G33770.1     829     848
ubiquitin-conjugating enzyme E2 -like protein

sRNA_AGO1_Solexa_Mi2008_1_51465_hit1
5' UUGCCAAAGGAGAGUUGCCCU
    |||||||||||| ||||||
   UACGGUUUCCUCUAAACGGGU 5'
AT2G33770.1     829     849
ubiquitin-conjugating enzyme E2 -like protein

sRNA_AGO1_Solexa_Mi2008_3_38327_hit2
5' UGCCAAAGGAGAGUUGCCC
   |||||||||||| ||||||
   ACGGUUUCCUCUAAACGGG 5'
AT2G33770.1     830     848
ubiquitin-conjugating enzyme E2 -like protein

sRNA_AGO1_Solexa_Mi2008_1_38331_hit4
5' UGCCAAAGGAGAUUUGCCC
   ||||||||||||||||||
   ACGGUUUCCUCUAAACGGG 5'
AT2G33770.1     830     848
ubiquitin-conjugating enzyme E2 -like protein
```

sRNA_AGO1_Solexa_Mi2008_2_38335_hit1
5' UGCCAAAGGAGAUUUGCCCU
   ||||||||||||||||| |
   ACGGUUUCCUCUAAACGAGC 5'
AT2G33770.1   886   905
ubiquitin-conjugating enzyme E2 -like protein

sRNA_AGO1_Solexa_Mi2008_3_38327_hit2
5' UGCCAAAGGAGAGUUGCCC
   |||||||||||| |||| |
   ACGGUUUCCUCUAAACGAG 5'
AT2G33770.1   887   905
ubiquitin-conjugating enzyme E2 -like protein

sRNA_AGO1_Solexa_Mi2008_1_38331_hit4
5' UGCCAAAGGAGAUUUGCCC
   ||||||||||||||||| |
   ACGGUUUCCUCUAAACGAG 5'
AT2G33770.1   887   905
ubiquitin-conjugating enzyme E2 -like protein

sRNA_AGO1_Solexa_Mi2008_2_38335_hit1
5' UGCCAAAGGAGAUUUGCCCU
   ||||||||||||||||| ||
   ACGGUUUCCUCUAAACGAGA 5'
AT2G33770.1   943   962
ubiquitin-conjugating enzyme E2 -like protein

sRNA_AGO1_Solexa_Mi2008_3_38327_hit2
5' UGCCAAAGGAGAGUUGCCC
   |||||||||||| |||| |
   ACGGUUUCCUCUAAACGAG 5'
AT2G33770.1   944   962
ubiquitin-conjugating enzyme E2 -like protein

sRNA_AGO1_Solexa_Mi2008_1_38331_hit4
5' UGCCAAAGGAGAUUUGCCC
   ||||||||||||||||| |
   ACGGUUUCCUCUAAACGAG 5'
AT2G33770.1   944   962
ubiquitin-conjugating enzyme E2 -like protein

sRNA_AGO1_Solexa_Mi2008_70_1353_hit7
5' ACAGAAGAGAGUGAGCACA
   ||||||||||||:|||| |
   UGUCUUCUCUCAUUCGUUU 5'
AT2G33810.1   785   803
squamosa-promoter binding protein-like 3

sRNA_AGO1_Solexa_Mi2008_150_13617_hit7
5' GACAGAAGAGAGUGAGCACA
   |||||||||||||:|||| |
   CUGUCUUCUCUCAUUCGUUU 5'
AT2G33810.1   785   804
squamosa-promoter binding protein-like 3

sRNA_AGO1_Solexa_Mi2008_714_35241_hit6
5' UGACAGAAGAGAGUGAGCACA
   ||||||||||||||:|||| |
   ACUGUCUUCUCUCAUUCGUUU 5'
AT2G33810.1   785   805
squamosa-promoter binding protein-like 3

```
sRNA_AGO1_Solexa_Mi2008_2_11428_hit3
5' CUGACAGAAGAGAGUGAGCACA
   |||||||||||||||:|||| |
   GACUGUCUUCUCUCAUUCGUUU 5'
AT2G33810.1     785     806
squamosa-promoter binding protein-like 3

sRNA_AGO1_Solexa_Mi2008_480_13616_hit7
5' GACAGAAGAGAGUGAGCAC
   |||||||||||||:||||
   CUGUCUUCUCUCAUUCGUU 5'
AT2G33810.1     786     804
squamosa-promoter binding protein-like 3

sRNA_AGO1_Solexa_Mi2008_3_8396_hit1
5' CGACAGAAGAGAGUGAGCAC
    |||||||||||||:||||
   ACUGUCUUCUCUCAUUCGUU 5'
AT2G33810.1     786     805
squamosa-promoter binding protein-like 3

sRNA_AGO1_Solexa_Mi2008_1_13615_hit4
5' GACAGAAGAGAG-AGAGCACA
   |||||||||||| | |||| |
   CUGUCUUCUCUCAU-UCGUUU 5'
AT2G33810.1     786     805
squamosa-promoter binding protein-like 3

sRNA_AGO1_Solexa_Mi2008_2091_35240_hit6
5' UGACAGAAGAGAGUGAGCAC
   ||||||||||||||:||||
   ACUGUCUUCUCUCAUUCGUU 5'
AT2G33810.1     786     805
squamosa-promoter binding protein-like 3

sRNA_AGO1_Solexa_Mi2008_28_11427_hit3
5' CUGACAGAAGAGAGUGAGCAC
   |||||||||||||||:||||
   GACUGUCUUCUCUCAUUCGUU 5'
AT2G33810.1     786     806
squamosa-promoter binding protein-like 3

sRNA_AGO1_Solexa_Mi2008_4_15853_hit2
5' GUGACAGAAGAGAGUGAGCAC
    ||||||||||||||:||||
   GACUGUCUUCUCUCAUUCGUU 5'
AT2G33810.1     786     806
squamosa-promoter binding protein-like 3

sRNA_AGO1_Solexa_Mi2008_2_35238_hit4
5' UGACAGAAGAGAG-AGAGCACA
   ||||||||||||| | |||| |
   ACUGUCUUCUCUCAU-UCGUUU 5'
AT2G33810.1     786     806
squamosa-promoter binding protein-like 3

sRNA_AGO1_Solexa_Mi2008_1778_50260_hit1
5' UUGACAGAAGAGAGUGAGCAC
   :||||||||||||||:||||
   GACUGUCUUCUCUCAUUCGUU 5'
AT2G33810.1     786     806
squamosa-promoter binding protein-like 3
```

```
sRNA_AGO1_Solexa_Mi2008_1_13614_hit8
5' GACAGAAGAGAG-AGAGCAC
   |||||||||||| | ||||
   CUGUCUUCUCUCAU-UCGUU 5'
AT2G33810.1     787     805
squamosa-promoter binding protein-like 3

sRNA_AGO1_Solexa_Mi2008_30_35239_hit6
5' UGACAGAAGAGAGUGAGCA
   ||||||||||||||:||||
   ACUGUCUUCUCUCAUUCGU 5'
AT2G33810.1     787     805
squamosa-promoter binding protein-like 3

sRNA_AGO1_Solexa_Mi2008_1_11426_hit3
5' CUGACAGAAGAGAGUGAGCA
   |||||||||||||||:||||
   GACUGUCUUCUCUCAUUCGU 5'
AT2G33810.1     787     806
squamosa-promoter binding protein-like 3

sRNA_AGO1_Solexa_Mi2008_12_35237_hit8
5' UGACAGAAGAGAG-AGAGCAC
   ||||||||||||| | ||||
   ACUGUCUUCUCUCAU-UCGUU 5'
AT2G33810.1     787     806
squamosa-promoter binding protein-like 3

sRNA_AGO1_Solexa_Mi2008_16_50259_hit1
5' UUGACAGAAGAGAGUGAGCA
   :||||||||||||||:||||
   GACUGUCUUCUCUCAUUCGU 5'
AT2G33810.1     787     806
squamosa-promoter binding protein-like 3

sRNA_AGO1_Solexa_Mi2008_1_50258_hit1
5' UUGACAGAAGAGAGUGAGC
   :||||||||||||||:|||
   GACUGUCUUCUCUCAUUCG 5'
AT2G33810.1     788     806
squamosa-promoter binding protein-like 3

sRNA_AGO1_Solexa_Mi2008_1_39748_hit1
5' UGGAAGCAGAUGGUUCGUU
   ||||| ||||||||||| |
   ACCUUGGUCUACCAAGCUA 5'
AT2G33860.1     1523    1541
auxin response transcription factor 3 (ETTIN/ARF3)

sRNA_AGO1_Solexa_Mi2008_1_35469_hit1
5' UGACCUUGUAAGACCCCAUCU
   ||||||||:||||| || |||
   ACUGGAACGUUCUG-GG-AGA 5'
AT2G33860.1     1672    1690
auxin response transcription factor 3 (ETTIN/ARF3)

sRNA_AGO1_Solexa_Mi2008_7_33918_hit1
5' UCUUGACCUUGUAAGACCCCA
   |||||||||||:|||||||
   AGAACUGGAACGUUCUGGGAG 5'
AT2G33860.1     1672    1692
auxin response transcription factor 3 (ETTIN/ARF3)
```

```
sRNA_AGO1_Solexa_Mi2008_7_49853_hit1
5' UUCUUGACCUUGUAAGACCCCA
   ||||||||||||:|||||||
   AAGAACUGGAACGUUCUGGGAG 5'
AT2G33860.1     1672    1693
auxin response transcription factor 3 (ETTIN/ARF3)

sRNA_AGO1_Solexa_Mi2008_7_12746_hit1
5' CUUGACCUUGUAAGACCCC
   ||||||||||:|||||||
   GAACUGGAACGUUCUGGGA 5'
AT2G33860.1     1673    1691
auxin response transcription factor 3 (ETTIN/ARF3)

sRNA_AGO1_Solexa_Mi2008_7_49852_hit1
5' UUCUUGACCUUGUAAGACCCC
   ||||||||||||:|||||||
   AAGAACUGGAACGUUCUGGGA 5'
AT2G33860.1     1673    1693
auxin response transcription factor 3 (ETTIN/ARF3)

sRNA_AGO1_Solexa_Mi2008_4_55012_hit1
5' UUUCUUGACCUUGUAAGACCCC
   |||||||||||||:|||||||
   AAAGAACUGGAACGUUCUGGGA 5'
AT2G33860.1     1673    1694
auxin response transcription factor 3 (ETTIN/ARF3)

sRNA_AGO1_Solexa_Mi2008_1_55011_hit1
5' UUUCUUGACCUUGUAAGACCC
   |||||||||||||:|||||||
   AAAGAACUGGAACGUUCUGGG 5'
AT2G33860.1     1674    1694
auxin response transcription factor 3 (ETTIN/ARF3)

sRNA_AGO1_Solexa_Mi2008_1_3_hit25
5' AAAAAAAAAAAA--AGAAAGA
   ||||||||||||  |||||:|
   UUUUUUUUUUUUGAUCUUUUU 5'
AT2G33860.1     173     193
auxin response transcription factor 3 (ETTIN/ARF3)

sRNA_AGO1_Solexa_Mi2008_1_2_hit38
5' AAA-AAAAAAAAAAAACCAU
   ||| |||||||||||| |
   UUUCUUUUUUUUUUUUGAUC 5'
AT2G33860.1     178     197
auxin response transcription factor 3 (ETTIN/ARF3)

sRNA_AGO1_Solexa_Mi2008_7_12746_hit1
5' CUUGACCUUGUAAGACCCC
   ||||||||||:||||||
   GAACUGGAACGUUCUGGAA 5'
AT2G33860.1     1793    1811
auxin response transcription factor 3 (ETTIN/ARF3)

sRNA_AGO1_Solexa_Mi2008_7_49852_hit1
5' UUCUUGACCUUGUAAGACCCC
   ||||||||||||:||||||
   AAGAACUGGAACGUUCUGGAA 5'
AT2G33860.1     1793    1813
auxin response transcription factor 3 (ETTIN/ARF3)
```

```
sRNA_AGO1_Solexa_Mi2008_4_55012_hit1
5' UUUCUUGACCUUGUAAGACCCC
   |||||||||||||:||||||
   AAAGAACUGGAACGUUCUGGAA 5'
AT2G33860.1     1793    1814
auxin response transcription factor 3 (ETTIN/ARF3)

sRNA_AGO1_Solexa_Mi2008_1_55011_hit1
5' UUUCUUGACCUUGUAAGACCC
   |||||||||||||:||||||
   AAAGAACUGGAACGUUCUGGA 5'
AT2G33860.1     1794    1814
auxin response transcription factor 3 (ETTIN/ARF3)

sRNA_AGO1_Solexa_Mi2008_1_42435_hit1
5' UGGGGGGAGGAUACGUGUACU
   |||||||||||||||||||||
   ACCCCCCUCCUAUGCACAUGA 5'
AT2G33860.1     1901    1921
auxin response transcription factor 3 (ETTIN/ARF3)

sRNA_AGO1_Solexa_Mi2008_2_52_hit1
5' AAAAA-AGAGAGAUACAGAGAU
   ||||| ||||||| ||||||||
   UUUUUCUCUCUCUUUGUCUCUA 5'
AT2G33860.1     345     366
auxin response transcription factor 3 (ETTIN/ARF3)

sRNA_AGO1_Solexa_Mi2008_1_43638_hit1
5' UGGUGUCAGAUGUGUGUG-UGU
   ||||| | |||||||||| |||
   ACCACUGACUACACACACCACA 5'
AT2G33860.1     39      60
auxin response transcription factor 3 (ETTIN/ARF3)

sRNA_AGO1_Solexa_Mi2008_2_8957_hit1
5' CGGACCAGGCUUCAUCCCCC
   ||||||||||||||||||
   GCCUGGUCCGAAGUAGGGUUA 5'
AT2G34710.1     868     888
homeodomain transcription factor (ATHB-14)

sRNA_AGO1_Solexa_Mi2008_153_14822_hit2
5' GGACCAGGCUUCAUCCCCC
   |||||||||||||||||
   CCUGGUCCGAAGUAGGGUU 5'
AT2G34710.1     869     887
homeodomain transcription factor (ATHB-14)

sRNA_AGO1_Solexa_Mi2008_219_8956_hit2
5' CGGACCAGGCUUCAUCCCCC
   ||||||||||||||||||
   GCCUGGUCCGAAGUAGGGUU 5'
AT2G34710.1     869     888
homeodomain transcription factor (ATHB-14)

sRNA_AGO1_Solexa_Mi2008_1063_31727_hit2
5' UCGGACCAGGCUUCAUCCCCC
   :||||||||||||||||||
   GGCCUGGUCCGAAGUAGGGUU 5'
AT2G34710.1     869     889
homeodomain transcription factor (ATHB-14)
```

```
sRNA_AGO1_Solexa_Mi2008_4_8955_hit2
5' CGGACCAGGCUUCAUCCCC
   |||||||||||||||||
   GCCUGGUCCGAAGUAGGGU 5'
AT2G34710.1     870     888
homeodomain transcription factor (ATHB-14)

sRNA_AGO1_Solexa_Mi2008_8_8960_hit4
5' CGGACCAGGCUUCAUUCCCCC
   |||||||||||||| ||||
   GCCUGGUCCGAAGU-AGGGUU 5'
AT2G34710.1     870     889
homeodomain transcription factor (ATHB-14)

sRNA_AGO1_Solexa_Mi2008_47_31726_hit2
5' UCGGACCAGGCUUCAUCCCC
   :||||||||||||||||||
   GGCCUGGUCCGAAGUAGGGU 5'
AT2G34710.1     870     889
homeodomain transcription factor (ATHB-14)

sRNA_AGO1_Solexa_Mi2008_310_14823_hit7
5' GGACCAGGCUUCAUUCCCC
   ||||||||||||| ||||
   CCUGGUCCGAAGU-AGGGU 5'
AT2G34710.1     871     888
homeodomain transcription factor (ATHB-14)

sRNA_AGO1_Solexa_Mi2008_641_8959_hit7
5' CGGACCAGGCUUCAUUCCCC
   |||||||||||||| ||||
   GCCUGGUCCGAAGU-AGGGU 5'
AT2G34710.1     871     889
homeodomain transcription factor (ATHB-14)

sRNA_AGO1_Solexa_Mi2008_2_31725_hit2
5' UCGGACCAGGCUUCAUCCC
   :||||||||||||||||||
   GGCCUGGUCCGAAGUAGGG 5'
AT2G34710.1     871     889
homeodomain transcription factor (ATHB-14)

sRNA_AGO1_Solexa_Mi2008_4138_31731_hit7
5' UCGGACCAGGCUUCAUUCCCC
    |||||||||||||| ||||
   CGCCUGGUCCGAAGU-AGGGU 5'
AT2G34710.1     871     890
homeodomain transcription factor (ATHB-14)

sRNA_AGO1_Solexa_Mi2008_1_8958_hit7
5' CGGACCAGGCUUCAUUCCC
   |||||||||||||| ||||
   GCCUGGUCCGAAGU-AGGG 5'
AT2G34710.1     872     889
homeodomain transcription factor (ATHB-14)

sRNA_AGO1_Solexa_Mi2008_15_11085_hit1
5' CUCGGACCAGGCUUCAUCC
    :|||||||||||||||||
   AGGCCUGGUCCGAAGUAGG 5'
AT2G34710.1     872     890
homeodomain transcription factor (ATHB-14)
```

```
sRNA_AGO1_Solexa_Mi2008_98_31730_hit7
5' UCGGACCAGGCUUCAUUCCC
    ||||||||||||| ||||
   CGCCUGGUCCGAAGU-AGGG 5'
AT2G34710.1     872     890
homeodomain transcription factor (ATHB-14)

sRNA_AGO1_Solexa_Mi2008_1_11087_hit3
5' CUCGGACCAGGCUUCAUUCCC
     ||||||||||||| ||||
   UCGCCUGGUCCGAAGU-AGGG 5'
AT2G34710.1     872     891
homeodomain transcription factor (ATHB-14)

sRNA_AGO1_Solexa_Mi2008_33_15725_hit3
5' GUCGGACCAGGCUUCAUUCCC
   : ||||||||||||| ||||
   UCGCCUGGUCCGAAGU-AGGG 5'
AT2G34710.1     872     891
homeodomain transcription factor (ATHB-14)

sRNA_AGO1_Solexa_Mi2008_68_49390_hit1
5' UUCGGACCAGGCUUCAUUCCC
     ||||||||||||| ||||
   UCGCCUGGUCCGAAGU-AGGG 5'
AT2G34710.1     872     891
homeodomain transcription factor (ATHB-14)

sRNA_AGO1_Solexa_Mi2008_6_31729_hit7
5' UCGGACCAGGCUUCAUUCC
    ||||||||||||| |||
   CGCCUGGUCCGAAGU-AGG 5'
AT2G34710.1     873     890
homeodomain transcription factor (ATHB-14)

sRNA_AGO1_Solexa_Mi2008_1_49389_hit1
5' UUCGGACCAGGCUUCAUUCC
     ||||||||||||| |||
   UCGCCUGGUCCGAAGU-AGG 5'
AT2G34710.1     873     891
homeodomain transcription factor (ATHB-14)

sRNA_AGO1_Solexa_Mi2008_1_37677_hit4
5' UGCAAAGACCAUCAAUGAUUG
   ||||| |||||||||| |||:
   ACGUUCCUGGUAGUUA-UAAU 5'
AT2G37130.1     1055    1074
putative peroxidase ATP2a

sRNA_AGO1_Solexa_Mi2008_1_1352_hit1
5' ACAGAAGAGAGAGAG-CACU
   ||||| ||||||||| || |
   UGUCUACUCUCUCUCAGUAA 5'
AT2G37130.2     481     500
putative peroxidase ATP2a

sRNA_AGO1_Solexa_Mi2008_1_1352_hit1
5' ACAGAAGAGAGAGAG-CACU
   ||||| ||||||||| || |
   UGUCUACUCUCUCUCAGUAA 5'
AT2G37130.1     581     600
putative peroxidase ATP2a
```

```
sRNA_AGO1_Solexa_Mi2008_1_37677_hit4
5' UGCAAAGACCAUCAAUGAUUG
   ||||| |||||||||| |||:
   ACGUUCCUGGUAGUUA-UAAU 5'
AT2G37130.2     955     974
putative peroxidase ATP2a

sRNA_AGO1_Solexa_Mi2008_1_10933_hit5
5' CUCCUGAAUCUUCCGACAA
   ||||| | |||||||||||
   GAGGA-U-AGAAGGCUGUU 5'
AT2G37640.1     527     543
Expansin (AtEXPA3)

sRNA_AGO1_Solexa_Mi2008_2_25192_hit1
5' UAUCUUAUGCGUCCUAUCUUCC
    ||||||| | |||||||||||
   UUAGAAUAGGGAGGAUAGAAGG 5'
AT2G37640.1     531     552
Expansin (AtEXPA3)

sRNA_AGO1_Solexa_Mi2008_4_15695_hit1
5' GUCGAGAAGAAUGAAAAGGU
   ||||||||| |||||| |||
   CAGCUCUUCCUACUUUGCCA 5'
AT2G37710.1     283     302
putative receptor-like protein kinase

sRNA_AGO1_Solexa_Mi2008_1_1205_hit1
5' AAUUGAAUGGACCUUUCUC
     |||||| ||||||||||
   AGAACUUAACUGGAAAGAG 5'
AT2G37710.1     580     598
putative receptor-like protein kinase

sRNA_AGO1_Solexa_Mi2008_1_174_hit2
5' AAAAGAAGAAGAUAAAGCAUAU
   |||||||||||| ||| ||||
   UUUUCUUCUUCUUUUUAGUAUC 5'
AT2G38290.1     1514    1535
putative ammonium transporter

sRNA_AGO1_Solexa_Mi2008_1_174_hit2
5' AAAAGAAGAAGAUAAAGCAUAU
   |||||||||||| ||| ||||
   UUUUCUUCUUCUUUUUAGUAUC 5'
AT2G38290.2     1593    1614
putative ammonium transporter

sRNA_AGO1_Solexa_Mi2008_1_17161_hit1
5' UAACAAGACCUGGCCACACAUC
   |||||||||||||| | || ||
   AUUGUUCUGGACCG-UAUG-AG 5'
AT2G38290.2     194     213
putative ammonium transporter

sRNA_AGO1_Solexa_Mi2008_1_4535_hit1
5' CAAGAAAAAACGAAGAUUUAU
    ||||||||||||| | ||||
   AUUCUUUUUUGCUU-UUAAUA 5'
AT2G38940.1     1802    1821
phosphate transporter (AtPT2)
```

```
sRNA_AGO1_Solexa_Mi2008_2_33689_hit1
5' UCUGGUGGAUUACGGAUUGUG
   |||||||||||| |  |||||
   AGACCACCUAAU-CG-AACAC 5'
AT2G39030.1     148     166
unknown protein

sRNA_AGO1_Solexa_Mi2008_3_40000_hit2
5' UGGACCCGGUCGAUGAAGUCU
   ||| || ||||| ||||||||
   ACC-GG-CCAGCCACUUCAGA 5'
AT2G39030.1     77      95
unknown protein

sRNA_AGO1_Solexa_Mi2008_1_33572_hit3
5' UCUGGACAA-GAUGAAGAUGGU
   ||||:| || ||||||||||||
   AGACUU-UUACUACUUCUACCA 5'
AT2G39510.1     1047    1067
nodulin-like protein

sRNA_AGO1_Solexa_Mi2008_1_4534_hit3
5' CAAGAAAAAAAGAGAUGAG
    ||||| |||||||||||:
   UUUCUUCUUUUCUCUACUU 5'
AT2G40360.1     2345    2363
putative WD-40 repeat protein

sRNA_AGO1_Solexa_Mi2008_2_51040_hit1
5' UUGAUCCAGAUGUCGAAUACAA
   |||||| ||||||||||| ||
   AACUAG-UCUACAGCUUA-GUA 5'
AT2G40360.1     945     964
putative WD-40 repeat protein

sRNA_AGO1_Solexa_Mi2008_2_380_hit1
5' AAAGAGAGAGAUGAUGCCAU
    || |||||||||||| |||
   GUU-UCUCUCUACUACCGUA 5'
AT2G40940.1     1322    1340
ethylene response sensor (ERS)

sRNA_AGO1_Solexa_Mi2008_1_38224_hit4
5' UGCAUCAUUGGUGGUGUGAG
   ||| | ||||||||||||||
   ACG-A-UAACCACCACACUC 5'
AT2G40940.1     174     191
ethylene response sensor (ERS)

sRNA_AGO1_Solexa_Mi2008_1_5010_hit1
5' CAAGUGCAUUAAGAACAUC-AU
   ||| ||||| ||||||||| ||
   GUU-ACGUACUUCUUGUAGGUA 5'
AT2G40940.1     462     482
ethylene response sensor (ERS)

sRNA_AGO1_Solexa_Mi2008_1_4591_hit1
5' CAAGACAAGGAGUUUUACC
   ||||||||:||||||||||
   GUUCUGUUUCUCAAAAUGG 5'
AT2G40940.1     753     771
ethylene response sensor (ERS)
```

```
sRNA_AGO1_Solexa_Mi2008_2_28315_hit7
5' UCAGCCAUGGUGGUGUCU-UUA
   ||||||||||| ||:||| |||
   AGUCGGUACCAACAUAGAUAAU 5'
AT2G46420.2     1136    1157
unknown protein

sRNA_AGO1_Solexa_Mi2008_2_48750_hit7
5' UUCAGCCAUGGUGGUGUCU-UUA
   |||||||||||| ||:||| |||
   AAGUCGGUACCAACAUAGAUAAU 5'
AT2G46420.2     1136    1158
unknown protein

sRNA_AGO1_Solexa_Mi2008_1_11854_hit1
5' CUGGAAAA-AUAGGAGUGUUU
   |: ||||| ||||||||||||
   GGACUUUUCUAUCCUCACAAA 5'
AT2G46970.1     1216    1236
PIF3 like basic Helix Loop Helix protein (PIL1)

sRNA_AGO1_Solexa_Mi2008_4_36271_hit2
5' UG-AGGAGACACCGGUGCCAAA
   || ||||||||||||| ||| |
   ACAUCCUCUGUGGCCA-GGUGU 5'
AT2G47160.1     1093    1113
putative anion exchange protein

sRNA_AGO1_Solexa_Mi2008_3_14060_hit2
5' GAGGAGACACCGGUGCCAA
   :||||||||||||| |||
   UUCCUCUGUGGCCA-GGUG 5'
AT2G47160.1     1094    1111
putative anion exchange protein

sRNA_AGO1_Solexa_Mi2008_1_11489_hit2
5' CUG-AGGAGACACCGGUGCCA
    || ||||||||||||| |||
   AACAUCCUCUGUGGCCA-GGU 5'
AT2G47160.1     1095    1114
putative anion exchange protein

sRNA_AGO1_Solexa_Mi2008_2_33399_hit2
5' UCUG-AGGAGACACCGGUGCCA
   | || ||||||||||||| |||
   AAACAUCCUCUGUGGCCA-GGU 5'
AT2G47160.1     1095    1115
putative anion exchange protein

sRNA_AGO1_Solexa_Mi2008_4_36271_hit2
5' UG-AGGAGACACCGGUGCCAAA
   || ||||||||||||| ||| |
   ACAUCCUCUGUGGCCA-GGUGU 5'
AT2G47160.2     1172    1192
putative anion exchange protein

sRNA_AGO1_Solexa_Mi2008_3_14060_hit2
5' GAGGAGACACCGGUGCCAA
   :||||||||||||| |||
   UUCCUCUGUGGCCA-GGUG 5'
AT2G47160.2     1173    1190
putative anion exchange protein
```

```
sRNA_AGO1_Solexa_Mi2008_1_11489_hit2
5' CUG-AGGAGACACCGGUGCCA
    || ||||||||||||| |||
   AACAUCCUCUGUGGCCA-GGU 5'
AT2G47160.2     1174    1193
putative anion exchange protein

sRNA_AGO1_Solexa_Mi2008_2_33399_hit2
5' UCUG-AGGAGACACCGGUGCCA
   | || ||||||||||||| |||
   AAACAUCCUCUGUGGCCA-GGU 5'
AT2G47160.2     1174    1194
putative anion exchange protein

sRNA_AGO1_Solexa_Mi2008_1_13420_hit2
5' GAAGAAGAAGAAGACUCUU
   |||||||||||||| ||
   CUUCUUCUUCUUCU-AGCU 5'
AT2G47160.1     142     159
putative anion exchange protein

sRNA_AGO1_Solexa_Mi2008_1_13420_hit2
5' GAAGAAGAAGAAGACUCUU
   |||||||||||||| ||
   CUUCUUCUUCUUCU-AGCU 5'
AT2G47160.2     146     163
putative anion exchange protein

sRNA_AGO1_Solexa_Mi2008_3_768_hit1
5' AAGAAGAGAGAGAAUUG-AG
   ||||| |||||||| || ||
   UUCUUAUCUCUCUU-ACGUC 5'
AT2G47160.1     185     203
putative anion exchange protein

sRNA_AGO1_Solexa_Mi2008_3_768_hit1
5' AAGAAGAGAGAGAAUUG-AG
   ||||| |||||||| || ||
   UUCUUAUCUCUCUU-ACGUC 5'
AT2G47160.2     189     207
putative anion exchange protein

sRNA_AGO1_Solexa_Mi2008_7_15838_hit1
5' GUGAAGAUGAAGAAUAAUGUU
   || |||||||||||:||||||
   CA-UUCUACUUCUUGUUACAA 5'
AT2G47510.2     1680    1699
putative fumarase

sRNA_AGO1_Solexa_Mi2008_7_15838_hit1
5' GUGAAGAUGAAGAAUAAUGUU
   || |||||||||||:||||||
   CA-UUCUACUUCUUGUUACAA 5'
AT2G47510.1     1736    1755
putative fumarase

sRNA_AGO1_Solexa_Mi2008_1_41846_hit1
5' UGG-GAGCUGAGAGAA-ACGUCG
   ||| |||||||||||| ||| ||
   ACCUCUCGACUCUCUUCUGCUGC 5'
AT2G47510.2     40      62
putative fumarase
```

```
sRNA_AGO1_Solexa_Mi2008_1_41846_hit1
5' UGG-GAGCUGAGAGAA-ACGUCG
   ||| |||||||||||| ||| ||
   ACCUCUCGACUCUCUUCUGCUGC 5'
AT2G47510.1     84      106
putative fumarase

sRNA_AGO1_Solexa_Mi2008_1_37225_hit4
5' UGAUGAUGAUGAUGAUGAAGAAG
    |||||:||||| ||||||||||
   CCUACUGCUACUUCUACUUCUUC 5'
AT3G01830.1     614     636
unknown protein

sRNA_AGO1_Solexa_Mi2008_6_33450_hit2
5' UCUGAUGGUGUGCAUCCCAAU
   |||||||||||| | |||||:
   AGACUACCACAC-UCGGGUUG 5'
AT3G01970.1     737     756
putative WRKY-like transcriptional regulator protein

sRNA_AGO1_Solexa_Mi2008_1_48488_hit1
5' UUCAACUGCGGUAACAUCAGAU
   :|||||||||||||| || |||
   GAGUUGACGCCAUUGAAGACUA 5'
AT3G04070.2     932     953
NAM-like protein (no apical meristem)

sRNA_AGO1_Solexa_Mi2008_1_48488_hit1
5' UUCAACUGCGGUAACAUCAGAU
   :|||||||||||||| || |||
   GAGUUGACGCCAUUGAAGACUA 5'
AT3G04070.1     980     1001
NAM-like protein (no apical meristem)

sRNA_AGO1_Solexa_Mi2008_1_4534_hit3
5' CAAGA-A-AAAAAGAGAUGAG
   ||||| | ||||||||||| |
   GUUCUCUAUUUUUCUCUACAC 5'
AT3G04520.1     14      34
L-allo-threonine aldolase like protein

sRNA_AGO1_Solexa_Mi2008_2_6737_hit1
5' CAG-UGGUGCUUCGGGUUACUC
   ||| || |||||||||| ||||
   GUCUAC-ACGAAGCCCACUGAG 5'
AT3G04520.1     1       21
L-allo-threonine aldolase like protein

sRNA_AGO1_Solexa_Mi2008_2_44800_hit1
5' UGUCUACGU-UGAUGAUGUUGU
   ||||||||| | |||||||||
   ACAGAUGCAUA-UACUACAACC 5'
AT3G04520.1     965     985
L-allo-threonine aldolase like protein

sRNA_AGO1_Solexa_Mi2008_85_22179_hit1
5' UAGCCAAGGAUGACUUGCCUGU
    ||||||:||||| ||||||||
   CUCGGUUUCUACUAAACGGACA 5'
AT3G05690.1     1181    1202
putative transcription factor
```

```
sRNA_AGO1_Solexa_Mi2008_400_22176_hit7
5' UAGCCAAGGAUGACUUGCCUG
    ||||||:||||| |||||||
   CUCGGUUUCUACUAAACGGAC 5'
AT3G05690.1     1182    1202
putative transcription factor

sRNA_AGO1_Solexa_Mi2008_30_36087_hit4
5' UGAGCCAAGGAUGACUUGCCG
   ||||||||:||||| |||||
   ACUCGGUUUCUACUAAACGGA 5'
AT3G05690.1     1183    1203
putative transcription factor

sRNA_AGO1_Solexa_Mi2008_1_1014_hit1
5' AAU-AGCCAAGGAUGACUUGCCUG
   ||| ||||||:||||| |||||||
   UUACUCGGUUUCUACUAAACGGAC 5'
AT3G05690.1     1183    1206
putative transcription factor

sRNA_AGO1_Solexa_Mi2008_4_36086_hit4
5' UGAGCCAAGGAUGACUUGCC
   ||||||||:||||| |||||
   ACUCGGUUUCUACUAAACGG 5'
AT3G05690.1     1184    1203
putative transcription factor

sRNA_AGO1_Solexa_Mi2008_1_3_hit25
5' AAAAAAAAAAAAAG-AAAGA
   |||||| |||||||  ||||
   UUUUUU-UUUUUUCACUUCU 5'
AT3G07010.1     1515    1533
pectate lyase like protein

sRNA_AGO1_Solexa_Mi2008_1_36258_hit1
5' UGAGGA-CCAAAGAGUUGCUGA
   |||||| |||| |||||||||
   ACUCCUCGGUU-CUCAACGACG 5'
AT3G08640.1     632     652
unknown protein

sRNA_AGO1_Solexa_Mi2008_1_6861_hit1
5' CAUACG-CUUGAUGGACAUGUU
   |||| | ||||||||||| |||
   GUAUACCGAACUACCUGU-CAA 5'
AT3G08640.1     677     697
unknown protein

sRNA_AGO1_Solexa_Mi2008_3_15159_hit2
5' GGGAUAACAUCAUAGGAUU
   |||||||||:||| |||||
   CCCUAUUGUGGUACCCUAA 5'
AT3G08860.1     1005    1023
aminotransferase like protein

sRNA_AGO1_Solexa_Mi2008_3_53977_hit1
5' UUUAAGGGUGCGGUGUAG-AGU
   |||||||||| | ||||| |||
   AAAUUCCCACACAACAUCUUCA 5'
AT3G08860.1     304     325
aminotransferase like protein
```

```
sRNA_AGO1_Solexa_Mi2008_2_14955_hit1
5' GGCAGC-AGCUGAAGCAAGAGCA
   |||||| | ||||||||| ||||
   CCGUCGAU-GACUUCGUUGUCGU 5'
AT3G09200.2     826     847
putative 60S acidic ribosomal protein P0

sRNA_AGO1_Solexa_Mi2008_2_14955_hit1
5' GGCAGC-AGCUGAAGCAAGAGCA
   |||||| | ||||||||| ||||
   CCGUCGAU-GACUUCGUUGUCGU 5'
AT3G09200.1     925     946
putative 60S acidic ribosomal protein P0

sRNA_AGO1_Solexa_Mi2008_1_1666_hit1
5' ACGUUGUUCAGUAGCUGGUA
   || ||| ||||||||||||
   UG-AACUAGUCAUCGACCAA 5'
AT3G10660.1     149     167
calmodulin-domain protein kinase CDPK isoform 2

sRNA_AGO1_Solexa_Mi2008_17_9865_hit4
5' CUACAGAAACGGAUUUGAC
   ||||||||||:||||| ||
   GAUGUCUUUGUCUAAA-UG 5'
AT3G10660.1     2057    2074
calmodulin-domain protein kinase CDPK isoform 2

sRNA_AGO1_Solexa_Mi2008_1_2542_hit2
5' AUAGAUAUAACA-CCAGGAU
   ||||||||||||  | ||||
   UAUCUAUAUUGUAAG-CCUA 5'
AT3G10660.1     731     749
calmodulin-domain protein kinase CDPK isoform 2

sRNA_AGO1_Solexa_Mi2008_1_45296_hit1
5' UGUGGAGUAAUUGAUGUUGUCC
   ||||||||||||||||||||||
   ACACCUCAUUAACUACAACAGG 5'
AT3G11680.1     1290    1311
unknown protein

sRNA_AGO1_Solexa_Mi2008_1_1310_hit1
5' ACACAUCCCCUGUGGAGUAAU
   |||||||||||||||||||||
   UGUGUAGGGGACACCUCAUUA 5'
AT3G11680.1     1301    1321
unknown protein

sRNA_AGO1_Solexa_Mi2008_8_49355_hit1
5' UUCGCGGUGGCCGUCGGUA
   |||||||||||||||||||
   AAGCGCCACCGGCAGCCAU 5'
AT3G11680.1     1405    1423
unknown protein

sRNA_AGO1_Solexa_Mi2008_1_20871_hit4
5' UAGAAGAAAACCGCAGCG-GAC
   ||||||||||| |||||| ||
   AUCUUCUUUUGCCGUCGCUCUU 5'
AT3G13175.1     296     317
unknown protein
```

```
sRNA_AGO1_Solexa_Mi2008_1_20872_hit2
5' UAGAAGAAAACCGCAGCG-GAU
   ||||||||||| |||||| ||
   AUCUUCUUUUGCCGUCGCUCUU 5'
AT3G13175.1     296     317
unknown protein

sRNA_AGO1_Solexa_Mi2008_1_4102_hit1
5' CAAAGAUGGAGUGUAG-GAA
   ||||||||||||| || ||
   GUUUCUACCUCACUUCUCUC 5'
AT3G13175.1     74      93
unknown protein

sRNA_AGO1_Solexa_Mi2008_1_19217_hit6
5' UA-CAAUGGUGAAGUGAGGU
   || ||||||||| ||||||
   AUCGUUACCACUACACUCCC 5'
AT3G15380.1     1381    1400
unknown protein

sRNA_AGO1_Solexa_Mi2008_1_24301_hit71
5' UAUAAUA-CAAUGGUGAAGUGAG
   |||| || ||||||||| |||||
   AUAU-AUCGUUACCACUACACUC 5'
AT3G15380.1     1383    1404
unknown protein

sRNA_AGO1_Solexa_Mi2008_1_3901_hit3
5' CAAAACG-AA-GAAGAUGAUGAG
   ||||| | || ||||||||||||
   GUUUUCCGUUACUUCUACUACUC 5'
AT3G15380.1     200     222
unknown protein

sRNA_AGO1_Solexa_Mi2008_1_4253_hit1
5' CA-AAUGACGUGGUGCGUGCUG
   || || || |||||||||||||
   GUCUU-CUCCACCACGCACGAC 5'
AT3G15380.1     2120    2140
unknown protein

sRNA_AGO1_Solexa_Mi2008_1_7584_hit16
5' CCA-AAUGCAGAAACCCAUCUU
    || |||||||||||||| |||
   UGUGUUACGUCUUUGGGU-GAA 5'
AT3G16470.1     1557    1577
putative lectin

sRNA_AGO1_Solexa_Mi2008_1_7584_hit16
5' CCA-AAUGCAGAAACCCAUCUU
    || |||||||||||||| |||
   UGUGUUACGUCUUUGGGU-GAA 5'
AT3G16470.2     1562    1582
putative lectin

sRNA_AGO1_Solexa_Mi2008_1_15600_hit1
5' GUCAGAAAAUUAGAUAUUU
   |||||||||||  ||||||
   CAGUCUUUUAA--UAUAAA 5'
AT3G17800.2     59      75
unknown protein
```

```
sRNA_AGO1_Solexa_Mi2008_5_479_hit1
5' AAAGGAAGAAGAUAUGG-AGUU
    ||| |||||||||||| ||||
   GUUC-UUCUUCUAUACCCUCAA 5'
AT3G19100.1     131     151
CDPK-related kinase

sRNA_AGO1_Solexa_Mi2008_4_10938_hit2
5' CUCCUUCU-GCAGUUGUAUGUU
     |||||| ||||||||||||
   UUGGAAGAACGUCAACAUACAA 5'
AT3G19100.1     2046    2067
CDPK-related kinase

sRNA_AGO1_Solexa_Mi2008_1_32180_hit1
5' UCGGGUUAAGUGGUAUGUGGU
   |||||||||||||||||||||
   AGCCCAAUUCACCAUACACCA 5'
AT3G20810.3     50      70
unknown protein

sRNA_AGO1_Solexa_Mi2008_1_32180_hit1
5' UCGGGUUAAGUGGUAUGUGGU
   |||||||||||||||||||||
   AGCCCAAUUCACCAUACACCA 5'
AT3G20810.2     82      102
unknown protein

sRNA_AGO1_Solexa_Mi2008_3_4691_hit4
5' CAAGAUGUUUUUCUUGUGG-GA
   ||| ||||||||||||||| ||
   GUU-UACAAAAAGAACACCACU 5'
AT3G23250.2     229     249
myb-related transcription factor like protein

sRNA_AGO1_Solexa_Mi2008_3_4691_hit4
5' CAAGAUGUUUUUCUUGUGG-GA
   ||| ||||||||||||||| ||
   GUU-UACAAAAAGAACACCACU 5'
AT3G23250.1     230     250
myb-related transcription factor like protein

sRNA_AGO1_Solexa_Mi2008_1_23588_hit1
5' UAGGUUUAGGUGGUUAAUAAA
   ||||||||| ||||| ||||
   AUCCAAAUCGACCAACUAUUA 5'
AT3G23250.1     635     655
myb-related transcription factor like protein

sRNA_AGO1_Solexa_Mi2008_1_23588_hit1
5' UAGGUUUAGGUGGUUAAUAAA
   ||||||||| ||||| ||||
   AUCCAAAUCGACCAACUAUUA 5'
AT3G23250.2     713     733
myb-related transcription factor like protein

sRNA_AGO1_Solexa_Mi2008_2_35284_hit4
5' UGACAGUGGCAGUAGUACAG
   ||||||||||||:||| |||
   ACUGUCACCGUCGUCA-GUC 5'
AT3G23550.1     1438    1456
unknown protein
```

```
sRNA_AGO1_Solexa_Mi2008_13_7081_hit3
5' CAUCUCAUGCCGAAUU-GUCCUA
    ||||||||||||||  ||||||
   AUAGAGUACGGCUUACGCAGGAU 5'
AT3G24660.1     1327    1349
putative kinase-like protein TMKL1 precursor

sRNA_AGO1_Solexa_Mi2008_1_48336_hit1
5' UUAUUAGUCUAUUGU-GUGA
   :||||||||:||||| ||||
   GAUAAUCAGGUAACACCACU 5'
AT3G24660.1     318     337
putative kinase-like protein TMKL1 precursor

sRNA_AGO1_Solexa_Mi2008_1_46521_hit1
5' UUAAGA-UCAGGAAACUCAU
   |||| | ||||||||||||:
   AAUU-UGAGUCCUUUGAGUG 5'
AT3G24660.1     703     721
putative kinase-like protein TMKL1 precursor

sRNA_AGO1_Solexa_Mi2008_7_353_hit2
5' AAAGAAGAAGAUAAAGCAU
   |||||||||||| || |||
   UUUCUUCUUCUA-UU-GUA 5'
AT3G24660.1     94      110
putative kinase-like protein TMKL1 precursor

sRNA_AGO1_Solexa_Mi2008_1_173_hit2
5' AAAAGAAGAAGAUAAAGCAU
    |||||||||||| || |||
   CUUUCUUCUUCUA-UU-GUA 5'
AT3G24660.1     94      111
putative kinase-like protein TMKL1 precursor

sRNA_AGO1_Solexa_Mi2008_1_4774_hit23
5' CAAGCUGCAGAAGUGG-UGCAA
   ||||| |||||||||| ||||
   GUUCGUCGUCUUCACCGACGUC 5'
AT3G25890.2     1244    1265
unknown protein

sRNA_AGO1_Solexa_Mi2008_2_4775_hit6
5' CAAGCUGCAGAAGUGG-UGCAU
   ||||| |||||||||| ||||
   GUUCGUCGUCUUCACCGACGUC 5'
AT3G25890.2     1244    1265
unknown protein

sRNA_AGO1_Solexa_Mi2008_1_173_hit2
5' AA-AAGAAGAAGAUAAAGCAU
   || ||||||||||||||| |
   UUCUUCUUCUUCUAUUUCAUU 5'
AT3G25890.2     156     176
unknown protein

sRNA_AGO1_Solexa_Mi2008_1_172_hit2
5' AA-AAGAAGAAGAUAAAGCA
   || ||||||||||||||| |
   UUCUUCUUCUUCUAUUUCAU 5'
AT3G25890.2     157     176
unknown protein
```

```
sRNA_AGO1_Solexa_Mi2008_1_4774_hit23
5' CAAGCUGCAGAAGUGG-UGCAA
   ||||| |||||||||| ||||
   GUUCGUCGUCUUCACCGACGUC 5'
AT3G25890.1     919     940
unknown protein

sRNA_AGO1_Solexa_Mi2008_2_4775_hit6
5' CAAGCUGCAGAAGUGG-UGCAU
   ||||| |||||||||| ||||
   GUUCGUCGUCUUCACCGACGUC 5'
AT3G25890.1     919     940
unknown protein

sRNA_AGO1_Solexa_Mi2008_2_16789_hit1
5' UAAAGCUGGUGGUGGUUU-ACU
   |||||||||| ||| ||| |||
   AUUUCGACCAACACAAAAUUGA 5'
AT3G26380.1     1508    1529
unknown protein

sRNA_AGO1_Solexa_Mi2008_1_379_hit1
5' AAAGAGA-AGUAUGAAAACUCA
   ||||| | ||||||| ||||||
   UUUCU-UGUCAUACUGUUGAGU 5'
AT3G28600.1     1134    1154
unknown protein

sRNA_AGO1_Solexa_Mi2008_4_13685_hit6
5' GACCAUAUGGACAAUGGGC
   :||| |||||||||||||
   UUGGAAUACCUGUUACCCU 5'
AT3G28740.1     1491    1509
cytochrome P450 like protein

sRNA_AGO1_Solexa_Mi2008_3_768_hit1
5' AAGAAGAGAGAGAAUUGAG
   |||| ||||||||||| ||
   UUCU-CUCUCUCUUAA-UC 5'
AT3G28740.1     38      54
cytochrome P450 like protein

sRNA_AGO1_Solexa_Mi2008_8_4655_hit1
5' CAAGAGUGUUUGAGAGAUA
   ||||||| || ||||||||
   GUUCUCA-AA-CUCUCUAU 5'
AT3G28740.1     72      88
cytochrome P450 like protein

sRNA_AGO1_Solexa_Mi2008_1_13420_hit2
5' GAAGAAGAAGAAGACUCUU
   |||||||||||||| | ||
   CUUCUUCUUCUUCU-A-AA 5'
AT3G29000.1     22      38
unknown protein

sRNA_AGO1_Solexa_Mi2008_3_21212_hit1
5' UAGAGAGAGAAAAAGAUGGC
   :||||||||||| ||||||
   GUCUCUCUCUUUCUCUACCA 5'
AT3G30180.1     55      74
cytochrome P450 homolog, putative
```

```
sRNA_AGO1_Solexa_Mi2008_1_18666_hit1
5' UAAUCAGAGAGAGAAAGAGGAU
   ||| |||||||||||||| ||
   AUU-GUCUCUCUCUUUCUC-UA 5'
AT3G30180.1     59      78
cytochrome P450 homolog, putative

sRNA_AGO1_Solexa_Mi2008_2_411_hit5
5' AAAGAUGAAGAGAGAAAGAGA
   ||| | || |||||||||||
   UUUGU-CU-CUCUCUUUCUCU 5'
AT3G30180.1     60      78
cytochrome P450 homolog, putative

sRNA_AGO1_Solexa_Mi2008_3_23318_hit2
5' UAG-GUAAAAACAUCUGGUCUA
   ||| |||||| ||||||||||
   AUCACAUUUU-GUAGACCAGAG 5'
AT3G44300.1     961     981
nitrilase 2

sRNA_AGO1_Solexa_Mi2008_3_23318_hit2
5' UAG-GUAAAAACAUCUGGUCUA
   ||| |||||| ||||||||||
   AUCACAUUUU-GUAGACCAGAG 5'
AT3G44310.2     861     881
nitrilase 1

sRNA_AGO1_Solexa_Mi2008_3_23318_hit2
5' UAG-GUAAAAACAUCUGGUCUA
   ||| |||||| ||||||||||
   AUCACAUUUU-GUAGACCAGAG 5'
AT3G44310.3     979     999
nitrilase 1

sRNA_AGO1_Solexa_Mi2008_1_29347_hit1
5' UCCACCACUUUCUUAGCCUUA
    |||||| ||||||||||||
   CGGUGGU-AAAGAAUCGGAAU 5'
AT3G46610.1     1430    1449
unknown protein

sRNA_AGO1_Solexa_Mi2008_2_26842_hit13
5' UCAACAACUGCAGCCAUUGU-GU
   |||| ||||| ||||||||| ||
   AGUU-UUGACUUCGGUAACAACA 5'
AT3G46610.1     1848    1869
unknown protein

sRNA_AGO1_Solexa_Mi2008_1_3_hit25
5' AAAAAAAAAAAAAGAAAGA
    ||||||||||||| |||
   GUUUUUUUUUUUUCUGUCU 5'
AT3G48740.1     1193    1211
MTN3-like protein

sRNA_AGO1_Solexa_Mi2008_1_1352_hit1
5' ACAGAAGAGAGAGAGCACU
     |||||||||| ||||||
   CUUCUUCUCUCUAUCGUGA 5'
AT3G48740.1     47      65
MTN3-like protein
```

```
sRNA_AGO1_Solexa_Mi2008_1_13615_hit4
5' GACAGAAGAGAGAGAGCACA
   || |||||||||| |||||
   CU-UCUUCUCUCUAUCGUGA 5'
AT3G48740.1     48      66
MTN3-like protein

sRNA_AGO1_Solexa_Mi2008_1_13614_hit8
5' GACAGAAGAGAGAGAGCAC
   || |||||||||| |||||
   CU-UCUUCUCUCUAUCGUG 5'
AT3G48740.1     49      66
MTN3-like protein

sRNA_AGO1_Solexa_Mi2008_12_35237_hit8
5' UGACAGAAGAGAGAGAGCAC
   ||| |||||||||| |||||
   ACU-UCUUCUCUCUAUCGUG 5'
AT3G48740.1     49      67
MTN3-like protein

sRNA_AGO1_Solexa_Mi2008_7_9719_hit1
5' CUAAG-UUCAACAUAUCGACG
   ||| | ||||||||||||| |
   GAU-CGAAGUUGUAUAGCUCC 5'
AT3G48740.1     803     822
MTN3-like protein

sRNA_AGO1_Solexa_Mi2008_1_49818_hit1
5' UUCUGGUGUUCAUGAUUUUCA
   ||| | | |||||||||||||
   AAG-C-AGAAGUACUAAAAGU 5'
AT3G50280.1     240     258
anthranilate N-hydroxycinnamoyl/benzoyltransferase - like protein

sRNA_AGO1_Solexa_Mi2008_56_12285_hit1
5' CUGUUGCUGCAUGUGCUGU-UU
    |||||||||||||| ||| ||
   UACAACGACGUACACCACACAA 5'
AT3G50440.1     128     149
unknown protein

sRNA_AGO1_Solexa_Mi2008_6_5721_hit1
5' CACGUGGACAAAGA-GAAGC
   | |||||||||||| ||||
   GCGCACCUGUUUCUGCUUCA 5'
AT3G50440.1     160     179
unknown protein

sRNA_AGO1_Solexa_Mi2008_1_21351_hit8
5' UAGAGGAAAUGAGUGUGUUAUUCC
   ||||:|||||||||||||||||||
   AUCUUCUUUACUCACACAAUAAGG 5'
AT3G50480.1     1006    1029
RPW8- like protein 4 (HR4)

sRNA_AGO1_Solexa_Mi2008_1_21350_hit1
5' UAGAGGAAAUGAGUGUGAUAU
   ||||:|||||||||||| |||
   AUCUUCUUUACUCACACAAUA 5'
AT3G50480.1     1009    1029
RPW8- like protein 4 (HR4)
```

```
sRNA_AGO1_Solexa_Mi2008_1_2507_hit3
5' AUAGAAGAAAUGAGUGUUU
    ||||||||||||||||||
   _AUCUUCUUUACUCACACAAU 5'
AT3G50480.1     1010    1030
RPW8- like protein 4 (HR4)

sRNA_AGO1_Solexa_Mi2008_1_37358_hit1
5' UGAUGGGCCUUAUAAUGGGCAUUC
   ||||||||||||||||||||||:|
   ACUACCCGGAAUAUUACCCGUAGG 5'
AT3G50480.1     905     928
RPW8- like protein 4 (HR4)

sRNA_AGO1_Solexa_Mi2008_1_37374_hit5
5' UGAUGGGUCUUAUAAUGGGCAUCC
   |||||||:||||||||||||||||
   ACUACCCGGAAUAUUACCCGUAGG 5'
AT3G50480.1     905     928
RPW8- like protein 4 (HR4)

sRNA_AGO1_Solexa_Mi2008_1_42681_hit5
5' UGGGUCUUAUAAUGGGCAU
   ||||:||||||||||||||
   ACCCGGAAUAUUACCCGUA 5'
AT3G50480.1     907     925
RPW8- like protein 4 (HR4)

sRNA_AGO1_Solexa_Mi2008_1_37373_hit5
5' UGAUGGGUCUUAUAAUGGGCAU
   |||||||:||||||||||||||
   ACUACCCGGAAUAUUACCCGUA 5'
AT3G50480.1     907     928
RPW8- like protein 4 (HR4)

sRNA_AGO1_Solexa_Mi2008_1_37356_hit1
5' UGAUGGGCCUCAUAAUGGGCA
   |||||||||| ||||||||||
   ACUACCCGGAAUAUUACCCGU 5'
AT3G50480.1     908     928
RPW8- like protein 4 (HR4)

sRNA_AGO1_Solexa_Mi2008_5_37357_hit26
5' UGAUGGGCCUUAUAAUGGGCA
   |||||||||||||||||||||
   ACUACCCGGAAUAUUACCCGU 5'
AT3G50480.1     908     928
RPW8- like protein 4 (HR4)

sRNA_AGO1_Solexa_Mi2008_1_12412_hit2
5' CUUAGAUAAUGGGCCUUAUAA
   ||||||| |||||||||||||
   GAAUCUACUACCCGGAAUAUU 5'
AT3G50480.1     914     934
RPW8- like protein 4 (HR4)

sRNA_AGO1_Solexa_Mi2008_1_42239_hit4
5' UGGGCUUAGAUGAUGGGUCUUAU
   |||||||||||||||||:|||||
   ACCCGAAUCUACUACCCGGAAUA 5'
AT3G50480.1     916     938
RPW8- like protein 4 (HR4)
```

```
sRNA_AGO1_Solexa_Mi2008_2_42236_hit1
5' UGGGCUUAGAUGAUGGACCUU
   |||||||||||||||| ||||
   ACCCGAAUCUACUACCCGGAA 5'
AT3G50480.1     918     938
RPW8- like protein 4 (HR4)

sRNA_AGO1_Solexa_Mi2008_2_42238_hit4
5' UGGGCUUAGAUGAUGGGUCUU
   |||||||||||||||||:|||
   ACCCGAAUCUACUACCCGGAA 5'
AT3G50480.1     918     938
RPW8- like protein 4 (HR4)

sRNA_AGO1_Solexa_Mi2008_2_42237_hit4
5' UGGGCUUAGAUGAUGGGUCU
   |||||||||||||||||:||
   ACCCGAAUCUACUACCCGGA 5'
AT3G50480.1     919     938
RPW8- like protein 4 (HR4)

sRNA_AGO1_Solexa_Mi2008_1_14303_hit4
5' GAUGGGCUUAGAUGAUGGGUCU
   |||||||||||||||||||:||
   CUACCCGAAUCUACUACCCGGA 5'
AT3G50480.1     919     940
RPW8- like protein 4 (HR4)

sRNA_AGO1_Solexa_Mi2008_4_37363_hit4
5' UGAUGGGCUUAGAUGAUGGGU
   ||||||||||||||||||||:
   ACUACCCGAAUCUACUACCCG 5'
AT3G50480.1     921     941
RPW8- like protein 4 (HR4)

sRNA_AGO1_Solexa_Mi2008_1_37364_hit1
5' UGAUGGGCUUAGAUGAUUGGC
   ||||||||||||||||| |||
   ACUACCCGAAUCUACU-ACCC 5'
AT3G50480.1     923     942
RPW8- like protein 4 (HR4)

sRNA_AGO1_Solexa_Mi2008_3_14257_hit30
5' GAUGAUGGGCUUAGAUGAU
   |||||||||||||||||||
   CUACUACCCGAAUCUACUA 5'
AT3G50480.1     925     943
RPW8- like protein 4 (HR4)

sRNA_AGO1_Solexa_Mi2008_1_21813_hit1
5' UAGAUGAUGGACUUCGAUGAU
   |||||||||| ||| ||||||
   AUCUACUACCCGAAUCUACUA 5'
AT3G50480.1     925     945
RPW8- like protein 4 (HR4)

sRNA_AGO1_Solexa_Mi2008_6_21815_hit1
5' UAGAUGAUGGGCUUAAAUGAU
   ||||||||||||||| |||||
   AUCUACUACCCGAAUCUACUA 5'
AT3G50480.1     925     945
RPW8- like protein 4 (HR4)
```

```
sRNA_AGO1_Solexa_Mi2008_5_21816_hit30
5' UAGAUGAUGGGCUUAGAUGAU
   |||||||||||||||||||||
   AUCUACUACCCGAAUCUACUA 5'
AT3G50480.1     925     945
RPW8- like protein 4 (HR4)

sRNA_AGO1_Solexa_Mi2008_1_21817_hit1
5' UAGAUGAUGGGUUUAGAUGAU
   |||||||||||:|||||||||
   AUCUACUACCCGAAUCUACUA 5'
AT3G50480.1     925     945
RPW8- like protein 4 (HR4)

sRNA_AGO1_Solexa_Mi2008_1_10162_hit2
5' CUAGAUGAUGGACUUAGAUGA
   ||||||||||| |||||||||
   GAUCUACUACCCGAAUCUACU 5'
AT3G50480.1     926     946
RPW8- like protein 4 (HR4)

sRNA_AGO1_Solexa_Mi2008_6_10164_hit27
5' CUAGAUGAUGGGCUUAGAUGA
   |||||||||||||||||||||
   GAUCUACUACCCGAAUCUACU 5'
AT3G50480.1     926     946
RPW8- like protein 4 (HR4)

sRNA_AGO1_Solexa_Mi2008_1_32880_hit27
5' UCUAGAUGAUGGGCUUAGAUGA
   ||||||||||||||||||||||
   AGAUCUACUACCCGAAUCUACU 5'
AT3G50480.1     926     947
RPW8- like protein 4 (HR4)

sRNA_AGO1_Solexa_Mi2008_7_10163_hit27
5' CUAGAUGAUGGGCUUAGAUG
   ||||||||||||||||||||
   GAUCUACUACCCGAAUCUAC 5'
AT3G50480.1     927     946
RPW8- like protein 4 (HR4)

sRNA_AGO1_Solexa_Mi2008_2_32879_hit27
5' UCUAGAUGAUGGGCUUAGAUG
   |||||||||||||||||||||
   AGAUCUACUACCCGAAUCUAC 5'
AT3G50480.1     927     947
RPW8- like protein 4 (HR4)

sRNA_AGO1_Solexa_Mi2008_1_20757_hit22
5' UAGAAACAUCUAGAUGAUGGGCUU
   ||||||||||||||||||||||||
   AUCUUUGUAGAUCUACUACCCGAA 5'
AT3G50480.1     932     955
RPW8- like protein 4 (HR4)

sRNA_AGO1_Solexa_Mi2008_1_19030_hit3
5' UACAAACAUCUAGAUGAUGGGCU
   || ||||||||||||||||||||
   AUCUUUGUAGAUCUACUACCCGA 5'
AT3G50480.1     933     955
RPW8- like protein 4 (HR4)
```

```
sRNA_AGO1_Solexa_Mi2008_5_10081_hit25
5' CUAGAAACAUCUAGAUGAUGG
   |||||||||||||||||||||
   GAUCUUUGUAGAUCUACUACC 5'
AT3G50480.1     936     956
RPW8- like protein 4 (HR4)

sRNA_AGO1_Solexa_Mi2008_4_14880_hit24
5' GGAGUACAAGGAAAGGGUA
   |||||||||||||||||||
   CCUCAUGUUCCUUUCCCAU 5'
AT3G50480.1     962     980
RPW8- like protein 4 (HR4)

sRNA_AGO1_Solexa_Mi2008_1_23502_hit2
5' UAGGUGUACAAGGAAAGGGUA
   |||| ||||||||||||||||
   AUCCUCAUGUUCCUUUCCCAU 5'
AT3G50480.1     962     982
RPW8- like protein 4 (HR4)

sRNA_AGO1_Solexa_Mi2008_5_24584_hit2
5' UAUAGGUGUACAAGGAAAGGGU
   |||||| |||||||||||||||
   AUAUCCUCAUGUUCCUUUCCCA 5'
AT3G50480.1     963     984
RPW8- like protein 4 (HR4)

sRNA_AGO1_Solexa_Mi2008_1_22768_hit2
5' UAGGAGUACAAGGAAAAGGGU
   ||||||||||||||| |||||
   AUCCUCAUGUUCCUU-UCCCA 5'
AT3G50480.1     964     983
RPW8- like protein 4 (HR4)

sRNA_AGO1_Solexa_Mi2008_1_2590_hit4
5' AUAGGAGUACAAGGAAAAGG
   |||||||||||||||| |||
   UAUCCUCAUGUUCCUU-UCC 5'
AT3G50480.1     966     984
RPW8- like protein 4 (HR4)

sRNA_AGO1_Solexa_Mi2008_1_54542_hit29
5' UUUAUAUAGGAGUACAAGGAA
   |||||||||||||||||||||
   AAAUAUAUCCUCAUGUUCCUU 5'
AT3G50480.1     968     988
RPW8- like protein 4 (HR4)

sRNA_AGO1_Solexa_Mi2008_1_35975_hit2
5' UGAGAUGAGUGACCAUGGCUGU
   |||||||| || |||||||||:
   ACUCUACUAACCGGUACCGACG 5'
AT3G50930.1     1239    1260
BCS1 protein-like protein

sRNA_AGO1_Solexa_Mi2008_2_35974_hit2
5' UGAGAUGAGUGACCAUGGCU
   |||||||| || ||||||||
   ACUCUACUAACCGGUACCGA 5'
AT3G50930.1     1241    1260
BCS1 protein-like protein
```

```
sRNA_AGO1_Solexa_Mi2008_1_24431_hit2
5' UAUA-GACAUGUGGAUGAUGCAC
   |||| |||||||||||| ||||
   AUAUGCUGUACACCUAC-ACGUA 5'
AT3G50930.1     1585    1606
BCS1 protein-like protein

sRNA_AGO1_Solexa_Mi2008_6_24430_hit2
5' UAUA-GACAUGUGGAUGAUGCA
   |||| |||||||||||| ||||
   AUAUGCUGUACACCUAC-ACGU 5'
AT3G50930.1     1586    1606
BCS1 protein-like protein

sRNA_AGO1_Solexa_Mi2008_1_24432_hit1
5' UAUA-GACAUGUGGAUGAUGCG
   |||| |||||||||||| |||:
   AUAUGCUGUACACCUAC-ACGU 5'
AT3G50930.1     1586    1606
BCS1 protein-like protein

sRNA_AGO1_Solexa_Mi2008_23_6153_hit2
5' CAGAUCUUGGUGG-UAG-UAGC
   ||||||||||||| ||| |||
   GUCUAGAACCACCGAUCUAUCC 5'
AT3G50930.1     671     692
BCS1 protein-like protein

sRNA_AGO1_Solexa_Mi2008_1_11865_hit1
5' CUGGAAAUUGACGCC-UUCU
   |||||| |||||||| || |
   GACCUUAAACUGCGGUAACA 5'
AT3G50930.1     785     804
BCS1 protein-like protein

sRNA_AGO1_Solexa_Mi2008_3_6076_hit1
5' CA-GAGGGAGAUGAAAGAAUU
   || | ||||||| ||||||||
   GUAC-CCCUCUAAUUUCUUAA 5'
AT3G55970.1     377     396
leucoanthocyanidin dioxygenase -like protein

sRNA_AGO1_Solexa_Mi2008_1_42962_hit1
5' UGGUAGUAGAGAUAAUAGUAU
   |||||:| ||||||||||||
   ACCAUUACCUCUAUUAUCAUC 5'
AT3G55970.1     491     511
leucoanthocyanidin dioxygenase -like protein

sRNA_AGO1_Solexa_Mi2008_7_14062_hit1
5' GAGGAGGAGGAGGUGAACA
   ||||||||||||| ||| |
   CUCCUCCUCCUCCUCUUCU 5'
AT3G56090.1     121     139
unknown protein

sRNA_AGO1_Solexa_Mi2008_3_12720_hit1
5' CUUGAAACAGGAGAUAGAGUUU
   |||||||| |||||| | ||||
   GAACUUUGGCCUCUA-C-CAAA 5'
AT3G56710.1     109     128
SigA binding protein
```

```
sRNA_AGO1_Solexa_Mi2008_6_28221_hit36
5' UCAGAUGUUUACAUGUGUCA
   ||||||||||| ||||||
   AGUCUACAAAUAUACACACA 5'
AT3G56710.1     16      35
SigA binding protein

sRNA_AGO1_Solexa_Mi2008_2_13688_hit1
5' GACCAUUUGUGAGAAGAGA
   |||||||||||||||||||
   CUGGUAAACACUCUUCUCU 5'
AT3G57230.2     585     603
MADS-box transcription factor (AGL16)

sRNA_AGO1_Solexa_Mi2008_596_13689_hit1
5' GACCAUUUGUGAGAAGGGA
   ||||||||||||||||:||
   CUGGUAAACACUCUUCUCU 5'
AT3G57230.2     585     603
MADS-box transcription factor (AGL16)

sRNA_AGO1_Solexa_Mi2008_72_1853_hit1
5' AGACCAUUUGUGAGAAGGGA
   |||||||||||||||||:||
   UCUGGUAAACACUCUUCUCU 5'
AT3G57230.2     585     604
MADS-box transcription factor (AGL16)

sRNA_AGO1_Solexa_Mi2008_3321_21060_hit1
5' UAGACCAUUUGUGAGAAGGGA
   ||||||||||||||||||:||
   AUCUGGUAAACACUCUUCUCU 5'
AT3G57230.2     585     605
MADS-box transcription factor (AGL16)

sRNA_AGO1_Solexa_Mi2008_2_10113_hit1
5' CUAGACCAUUUGUGAGAAGGGA
   |||||||||||||||||||:||
   GAUCUGGUAAACACUCUUCUCU 5'
AT3G57230.2     585     606
MADS-box transcription factor (AGL16)

sRNA_AGO1_Solexa_Mi2008_32_21059_hit1
5' UAGACCAUUUGUGAGAAGGG
   ||||||||||||||||||:|
   AUCUGGUAAACACUCUUCUC 5'
AT3G57230.2     586     605
MADS-box transcription factor (AGL16)

sRNA_AGO1_Solexa_Mi2008_37_21058_hit1
5' UAGACCAUUUGUGAGAAGG
   ||||||||||||||||||:
   AUCUGGUAAACACUCUUCU 5'
AT3G57230.2     587     605
MADS-box transcription factor (AGL16)

sRNA_AGO1_Solexa_Mi2008_2_13688_hit1
5' GACCAUUUGUGAGAAGAGA
   |||||||||||||||||||
   CUGGUAAACACUCUUCUCU 5'
AT3G57230.1     588     606
MADS-box transcription factor (AGL16)
```

```
sRNA_AGO1_Solexa_Mi2008_596_13689_hit1
5' GACCAUUUGUGAGAAGGGA
   ||||||||||||||||:||
   CUGGUAAACACUCUUCUCU 5'
AT3G57230.1     588     606
MADS-box transcription factor (AGL16)

sRNA_AGO1_Solexa_Mi2008_72_1853_hit1
5' AGACCAUUUGUGAGAAGGGA
   |||||||||||||||||:||
   UCUGGUAAACACUCUUCUCU 5'
AT3G57230.1     588     607
MADS-box transcription factor (AGL16)

sRNA_AGO1_Solexa_Mi2008_3321_21060_hit1
5' UAGACCAUUUGUGAGAAGGGA
   ||||||||||||||||||:||
   AUCUGGUAAACACUCUUCUCU 5'
AT3G57230.1     588     608
MADS-box transcription factor (AGL16)

sRNA_AGO1_Solexa_Mi2008_2_10113_hit1
5' CUAGACCAUUUGUGAGAAGGGA
   |||||||||||||||||||:||
   GAUCUGGUAAACACUCUUCUCU 5'
AT3G57230.1     588     609
MADS-box transcription factor (AGL16)

sRNA_AGO1_Solexa_Mi2008_32_21059_hit1
5' UAGACCAUUUGUGAGAAGGG
   ||||||||||||||||||:|
   AUCUGGUAAACACUCUUCUC 5'
AT3G57230.1     589     608
MADS-box transcription factor (AGL16)

sRNA_AGO1_Solexa_Mi2008_37_21058_hit1
5' UAGACCAUUUGUGAGAAGG
   ||||||||||||||||||:
   AUCUGGUAAACACUCUUCU 5'
AT3G57230.1     590     608
MADS-box transcription factor (AGL16)

sRNA_AGO1_Solexa_Mi2008_15_2751_hit1
5' AUCAAAAUCUG-UGGUGAGGCU
   ||||| || || ||||||||||
   UAGUUGUA-ACCACCACUCCGA 5'
AT3G57260.1     58      78
beta-1,3-glucanase 2 (BG2)

sRNA_AGO1_Solexa_Mi2008_1_14019_hit1
5' GAGCUCCUUGAAGUUCAAUG
   ||| |||||||||||||| |
   CUCAAGGAACUUCAAGUUCC 5'
AT3G58780.2     697     716
shatterproof 1 (SHP1)/ agamous -like 1 (AGL1)

sRNA_AGO1_Solexa_Mi2008_271_14020_hit1
5' GAGCUCCUUGAAGUUCAAUGG
   ||| |||||||||||||| ||
   CUCAAGGAACUUCAAGUU-CC 5'
AT3G58780.2     698     717
shatterproof 1 (SHP1)/ agamous -like 1 (AGL1)
```

```
sRNA_AGO1_Solexa_Mi2008_1_14019_hit1
5' GAGCUCCUUGAAGUUCAAUG
   ||| ||||||||||||| |
   CUCAAGGAACUUCAAGUUCC 5'
AT3G58780.1     718     737
shatterproof 1 (SHP1)/ agamous -like 1 (AGL1)

sRNA_AGO1_Solexa_Mi2008_271_14020_hit1
5' GAGCUCCUUGAAGUUCAAUGG
   ||| ||||||||||||| ||
   CUCAAGGAACUUCAAGUU-CC 5'
AT3G58780.1     719     738
shatterproof 1 (SHP1)/ agamous -like 1 (AGL1)

sRNA_AGO1_Solexa_Mi2008_1_52192_hit1
5' UUGGAUCAACAAACUGAUGG
   ||||||||| |||||| ||
   AACCUAGUUCUUUGAC-ACA 5'
AT3G59900.1     35      53
putative protein

sRNA_AGO1_Solexa_Mi2008_1_18400_hit1
5' UAAGGUUCGUUGAUUGUUGUC
   ||||||||||||||||||||
   AUUCCAAGCAACUAACAACAG 5'
AT3G60140.1     647     667
beta-glucosidase

sRNA_AGO1_Solexa_Mi2008_1_24287_hit1
5' UAUAAGGUUCGUUGAUUGUUGUC
   ||||||||||||||||||||||
   AUAUUCCAAGCAACUAACAACAG 5'
AT3G60140.1     647     669
beta-glucosidase

sRNA_AGO1_Solexa_Mi2008_2_44860_hit1
5' UGUCUUUAGAGAUCUCAAACGU
   ||||||| ||||| ||||| ||
   ACAGAAAACUCUAAAGUUU-CA 5'
AT3G60140.1     6       26
beta-glucosidase

sRNA_AGO1_Solexa_Mi2008_2_32161_hit1
5' UCGGGUCGGGUACGAUGUGUA
   ||||||||||||||||||||
   AGCCCAGCCCAUGCUACACAU 5'
AT3G61460.1     224     244
RING finger protein

sRNA_AGO1_Solexa_Mi2008_60_9189_hit1
5' CGGGUAUUCGGGUCGAGUA
   |||||||||||||| |||
   GCCCAUAAGCCCAGCCCAU 5'
AT3G61460.1     233     251
RING finger protein

sRNA_AGO1_Solexa_Mi2008_1_54911_hit1
5' UUUCGGGUAUUCGGGUCGGGUA
   |||||||||||||||||||||
   AAAGCCCAUAAGCCCAGCCCAU 5'
AT3G61460.1     233     254
RING finger protein
```

```
sRNA_AGO1_Solexa_Mi2008_16_36152_hit1
5' UGAGCGGGUUUCGGGUAUUCG
   |||||||||||||||||||||
   ACUCGCCCAAAGCCCAUAAGC 5'
AT3G61460.1     242     262
RING finger protein

sRNA_AGO1_Solexa_Mi2008_3_8682_hit1
5' CGAGU-UUCUACAGAGUGGACA
   || || |||||||:||||||||
   GC-CACAAGAUGUUUCACCUGU 5'
AT3G61460.1     468     488
RING finger protein

sRNA_AGO1_Solexa_Mi2008_1_29196_hit2
5' UCCAAAGGGAUCGCAUUGUUU
   |||||||||||||||||||:|
   AGGUUUCCCUAGCGUAACAGA 5'
AT3G62980.1     1710    1730
transport inhibitor response 1 (TIR1)

sRNA_AGO1_Solexa_Mi2008_1_29193_hit2
5' UCCAAAGGGAUCGCAUUGAU
   |||||||||||||||||| :
   AGGUUUCCCUAGCGUAACAG 5'
AT3G62980.1     1711    1730
transport inhibitor response 1 (TIR1)

sRNA_AGO1_Solexa_Mi2008_14_29195_hit2
5' UCCAAAGGGAUCGCAUUGAUCC
   |||||||||||||||||| ||
   AGGUUUCCCUAGCGUAAC-AGA 5'
AT3G62980.1     1711    1731
transport inhibitor response 1 (TIR1)

sRNA_AGO1_Solexa_Mi2008_23_29194_hit2
5' UCCAAAGGGAUCGCAUUGAUC
   |||||||||||||||||| ||
   AGGUUUCCCUAGCGUAAC-AG 5'
AT3G62980.1     1712    1731
transport inhibitor response 1 (TIR1)

sRNA_AGO1_Solexa_Mi2008_2_36207_hit1
5' UGAGCUUGAUGGUUAUAUGAA
   |||||||||||||||||||||
   ACUCGAACUACCAAUAUACUU 5'
AT3G62980.1     2092    2112
transport inhibitor response 1 (TIR1)

sRNA_AGO1_Solexa_Mi2008_1_39228_hit1
5' UGCUCAUGAGCUUGAUGGUUA
   |||||||||||||||||||||
   ACGAGUACUCGAACUACCAAU 5'
AT3G62980.1     2098    2118
transport inhibitor response 1 (TIR1)

sRNA_AGO1_Solexa_Mi2008_1_51840_hit3
5' UUGCU-GAAGAUGGUGUUGAUGC
   ||| | ||||||||||||| |||
   AAC-AUCUUCUACCACAACGACG 5'
AT4G00040.1     1045    1066
chalcone synthase like protein
```

```
sRNA_AGO1_Solexa_Mi2008_4_35117_hit1
5' UGAAUUGGAUCAUAUAAAGCU
   ||||||| ||||| |||||||
   ACUUAACGUAGUAGAUUUCGA 5'
AT4G00710.1     1966    1986
unknown protein

sRNA_AGO1_Solexa_Mi2008_2_52709_hit1
5' UUGGGGUGCUCAUGAUCAUAC
   |||||||||||||||||||||
   AACCCCACGAGUACUAGUAUG 5'
AT4G00940.1     714     734
putative protein

sRNA_AGO1_Solexa_Mi2008_1_3079_hit472
5' AUGAUGAUGAUGA-UGAUGAUGA
   ||||||||||||| | ||||||||
   UACUACUACUACUA-UGACUACUACC 5'
AT4G00940.1     91      112
putative protein

sRNA_AGO1_Solexa_Mi2008_6_14255_hit369
5' GAUGAUGAUGAUGAUGA-UGAUGAU
   ||| ||||||||||| | |||||||
   CUAGUACUACUACUACUA-UGACUACUA 5'
AT4G00940.1     93      116
putative protein

sRNA_AGO1_Solexa_Mi2008_1_3079_hit472
5' AUGAUGAUGAUGAUGAUGA-UGA
   ||||| ||||||||||| | |||
   UACUAGUACUACUACUACUA-UGACU 5'
AT4G00940.1     97      118
putative protein

sRNA_AGO1_Solexa_Mi2008_11_14066_hit1
5' GA-GGAGUUGAAUAUCUGUUCA
   || ||| |||||||| ||||||
   CUACCUGAACUUAUAAACAAGU 5'
AT4G02520.1     489     510
Atpm24.1 glutathione S transferase

sRNA_AGO1_Solexa_Mi2008_1_32728_hit1
5' UCUAAGGGAAAAUGUAUGAGC
   : ||||||||| |||||||||
   GUAUUCCCUUU-ACAUACUCG 5'
AT4G04920.1     3700    3719
unknown protein

sRNA_AGO1_Solexa_Mi2008_44_10639_hit2
5' CUC-AAGAUGGCAGCAU-AGU
    || ||||||||||||| |||
   UAGCUUCUACCGUCGUAGUCA 5'
AT4G08390.2     1048    1068
stromal ascorbate peroxidase

sRNA_AGO1_Solexa_Mi2008_5_5124_hit2
5' CAAUCU-C-AAGAUGGCAGCAU
   |||||| | |||||||||||||
   GUUAGAAGCUUCUACCGUCGUA 5'
AT4G08390.2     1052    1073
stromal ascorbate peroxidase
```

```
sRNA_AGO1_Solexa_Mi2008_5_19199_hit2
5' UACAAUCU-C-AAGAUGGCAGCAU
   | |||||| | |||||||||||||
   AAGUUAGAAGCUUCUACCGUCGUA 5'
AT4G08390.2     1052    1075
stromal ascorbate peroxidase

sRNA_AGO1_Solexa_Mi2008_1_19198_hit2
5' UACAAUCU-C-AAGAUGGCAGCA
   | |||||| | ||||||||||||
   AAGUUAGAAGCUUCUACCGUCGU 5'
AT4G08390.2     1053    1075
stromal ascorbate peroxidase

sRNA_AGO1_Solexa_Mi2008_44_10639_hit2
5' CUC-AAGAUGGCAGCAU-AGU
    || ||||||||||||| |||
   UAGCUUCUACCGUCGUAGUCA 5'
AT4G08390.1     1058    1078
stromal ascorbate peroxidase

sRNA_AGO1_Solexa_Mi2008_5_5124_hit2
5' CAAUCU-C-AAGAUGGCAGCAU
   |||||| | |||||||||||||
   GUUAGAAGCUUCUACCGUCGUA 5'
AT4G08390.1     1062    1083
stromal ascorbate peroxidase

sRNA_AGO1_Solexa_Mi2008_5_19199_hit2
5' UACAAUCU-C-AAGAUGGCAGCAU
   | |||||| | |||||||||||||
   AAGUUAGAAGCUUCUACCGUCGUA 5'
AT4G08390.1     1062    1085
stromal ascorbate peroxidase

sRNA_AGO1_Solexa_Mi2008_1_19198_hit2
5' UACAAUCU-C-AAGAUGGCAGCA
   | |||||| | ||||||||||||
   AAGUUAGAAGCUUCUACCGUCGU 5'
AT4G08390.1     1063    1085
stromal ascorbate peroxidase

sRNA_AGO1_Solexa_Mi2008_1_50784_hit1
5' UUGAGGUAAAUGAUGUUCCCCCAU
   ||||| ||||| |||| |||||||
   AACUC-AUUUAAUACACGGGGGUA 5'
AT4G08390.3     1066    1088
stromal ascorbate peroxidase

sRNA_AGO1_Solexa_Mi2008_1_50784_hit1
5' UUGAGGUAAAUGAUGUUCCCCCAU
   ||||| ||||| |||| |||||||
   AACUC-AUUUAAUACACGGGGGUA 5'
AT4G08390.2     1203    1225
stromal ascorbate peroxidase

sRNA_AGO1_Solexa_Mi2008_1_50784_hit1
5' UUGAGGUAAAUGAUGUUCCCCCAU
   ||||| ||||| |||| |||||||
   AACUC-AUUUAAUACACGGGGGUA 5'
AT4G08390.1     1213    1235
stromal ascorbate peroxidase
```

```
sRNA_AGO1_Solexa_Mi2008_3_10123_hit2
5' CUAGAGACGAGAGAU-U-ACU
   ||||||||||||||| | ||
   GAUCUCUGCUCUCUAGACUGC 5'
AT4G08390.3     130     150
stromal ascorbate peroxidase

sRNA_AGO1_Solexa_Mi2008_3_10123_hit2
5' CUAGAGACGAGAGAU-U-ACU
   ||||||||||||||| | ||
   GAUCUCUGCUCUCUAGACUGC 5'
AT4G08390.2     264     284
stromal ascorbate peroxidase

sRNA_AGO1_Solexa_Mi2008_3_10123_hit2
5' CUAGAGACGAGAGAU-U-ACU
   ||||||||||||||| | ||
   GAUCUCUGCUCUCUAGACUGC 5'
AT4G08390.1     274     294
stromal ascorbate peroxidase

sRNA_AGO1_Solexa_Mi2008_44_10639_hit2
5' CUC-AAGAUGGCAGCAU-AGU
    || ||||||||||||| |||
   UAGCUUCUACCGUCGUAGUCA 5'
AT4G08390.3     911     931
stromal ascorbate peroxidase

sRNA_AGO1_Solexa_Mi2008_5_5124_hit2
5' CAAUCU-C-AAGAUGGCAGCAU
   |||||| | |||||||||||||
   GUUAGAAGCUUCUACCGUCGUA 5'
AT4G08390.3     915     936
stromal ascorbate peroxidase

sRNA_AGO1_Solexa_Mi2008_5_19199_hit2
5' UACAAUCU-C-AAGAUGGCAGCAU
   | |||||| | |||||||||||||
   AAGUUAGAAGCUUCUACCGUCGUA 5'
AT4G08390.3     915     938
stromal ascorbate peroxidase

sRNA_AGO1_Solexa_Mi2008_1_19198_hit2
5' UACAAUCU-C-AAGAUGGCAGCA
   | |||||| | ||||||||||||
   AAGUUAGAAGCUUCUACCGUCGU 5'
AT4G08390.3     916     938
stromal ascorbate peroxidase

sRNA_AGO1_Solexa_Mi2008_1_51487_hit1
5' UUGCCACAUGUAG-GGAUGUC
    |||||||||||| || ||||
   UACGGUGUACAUCGCC-ACAG 5'
AT4G14400.2     424     443
unknown protein

sRNA_AGO1_Solexa_Mi2008_1_51487_hit1
5' UUGCCACAUGUAG-GGAUGUC
    |||||||||||| || ||||
   UACGGUGUACAUCGCC-ACAG 5'
AT4G14400.3     488     507
unknown protein
```

```
sRNA_AGO1_Solexa_Mi2008_1_51487_hit1
5' UUGCCACAUGUAG-GGAUGUC
    |||||||||||| || ||||
   UACGGUGUACAUCGCC-ACAG 5'
AT4G14400.1     507     526
unknown protein

sRNA_AGO1_Solexa_Mi2008_3_10294_hit3
5' CUAGGGUUUCAAUGAU-UGUA
   ||||||||||||| || |||
   GAUCCCAAAGUUAGUAGACAG 5'
AT4G14900.1     268     288
hydroxyproline-rich glycoprotein homolog (Z97337.18)

sRNA_AGO1_Solexa_Mi2008_1_21596_hit4
5' UAGAGUGAACAAUGAUGAG
   ||| | ||||||||||||
   AUC-CUCUUGUUACUACUG 5'
AT4G18130.1     1201    1218
phytochrome E

sRNA_AGO1_Solexa_Mi2008_1_12237_hit1
5' CUGUGCUGCGAACUGAAGU
   ||||| ||| |||||||||
   GACACUACGAUUGACUUCA 5'
AT4G18130.1     1291    1309
phytochrome E

sRNA_AGO1_Solexa_Mi2008_10_859_hit2
5' AAGCCUGCGCGUGAUUACCUCU
    ||| ||||||||||| |||||
   GUCG-ACGCGCACUAA-GGAGA 5'
AT4G18130.1     2078    2097
phytochrome E

sRNA_AGO1_Solexa_Mi2008_1_35722_hit6
5' UGACUU-CA-GACCUGCGCGUGAUU
   |||||| || || ||||||||||||
   ACUGAAAGUUCUCGACGCGCACUAA 5'
AT4G18130.1     2083    2107
phytochrome E

sRNA_AGO1_Solexa_Mi2008_76_35721_hit6
5' UGACUU-CA-GACCUGCGCGUGAU
   |||||| || || |||||||||||
   ACUGAAAGUUCUCGACGCGCACUA 5'
AT4G18130.1     2084    2107
phytochrome E

sRNA_AGO1_Solexa_Mi2008_21_35738_hit1
5' UGACUUU-A-GACCUGCGCGUGAU
   ||||||| | || |||||||||||
   ACUGAAAGUUCUCGACGCGCACUA 5'
AT4G18130.1     2084    2107
phytochrome E

sRNA_AGO1_Solexa_Mi2008_2_13419_hit2
5' GAAGAAGAAGAAGACA-CUU
   |||||||||||| ||| |||
   CUUCUUCUUCUU-UGUAGAA 5'
AT4G18130.1     2669    2687
phytochrome E
```

```
sRNA_AGO1_Solexa_Mi2008_1_13420_hit2
5' GAAGAAGAAGAAGAC-UCUU
   |||||||||||| || ||||
   CUUCUUCUUCUU-UGUAGAA 5'
AT4G18130.1     2669    2687
phytochrome E

sRNA_AGO1_Solexa_Mi2008_2_13419_hit2
5' GAAGAAGAAGAAGACA-CUU
   |||||||||||||| | ||
   CUUCUUCUUCUUCU-UCGAC 5'
AT4G18130.1     79      97
phytochrome E

sRNA_AGO1_Solexa_Mi2008_1_35652_hit7
5' UGACU-C-AACAUGACCGGCGU
   ||||| | |||||||||||||
   ACUGAUGGUUGUACUGGCCGCU 5'
AT4G21910.3     301     322
unknown protein

sRNA_AGO1_Solexa_Mi2008_1_35652_hit7
5' UGACU-C-AACAUGACCGGCGU
   ||||| | |||||||||||||
   ACUGAUGGUUGUACUGGCCGCU 5'
AT4G21910.1     362     383
unknown protein

sRNA_AGO1_Solexa_Mi2008_105_14948_hit3
5' GGCAAUAACAGGUCUGUGA
    |||||||||||||| ||
   ACGUUAUUGUCCAGA-ACA 5'
AT4G22380.1     364     381
Ribosomal protein L7Ae - like (fragment)

sRNA_AGO1_Solexa_Mi2008_3_2154_hit3
5' AGGCAAUAACAGGUCUGUG
     |||||||||||||| ||
   AACGUUAUUGUCCAGA-AC 5'
AT4G22380.1     365     382
Ribosomal protein L7Ae - like (fragment)

sRNA_AGO1_Solexa_Mi2008_1_14875_hit1
5' GGAGGUGGAGGCGGUGG-UGGU
   : |||||||||||||| ||||
   UGUCCACCUCCGCCACCAACCA 5'
AT4G22470.1     207     228
extensin - like protein

sRNA_AGO1_Solexa_Mi2008_2_43577_hit1
5' UGGUGGUGGUGACG-UUGGUGGU
   |||||||||||| | ||| ||||
   ACCACCACCACU-CUAACAACCA 5'
AT4G22470.1     778     799
extensin - like protein

sRNA_AGO1_Solexa_Mi2008_1_43578_hit1
5' UGGUGGUGGUGAUGAUGUGUC
   |||||||||||| ||| |||
   ACCACCACCACU-CUA-ACAA 5'
AT4G22470.1     781     799
extensin - like protein
```

```
sRNA_AGO1_Solexa_Mi2008_1_44600_hit1
5' UGUCCAUCUCCAUCUCGUUGUU
   ||||||| ||||||| | ||||
   ACAGGUAAAGGUAGAAC-ACAA 5'
AT4G23470.2     1069    1089
putative protein

sRNA_AGO1_Solexa_Mi2008_1_1652_hit1
5' ACGGUGCGUGAAUUGUAUU
   |||||||||||||||||||
   UGCCACGCACUUAACAUAA 5'
AT4G23470.3     1161    1179
putative protein

sRNA_AGO1_Solexa_Mi2008_2_24561_hit1
5' UAUAGGGUGCGUGAAU-GUAAU
   |||| ||||||||||| ||| |
   AUAUGCCACGCACUUAACAUAA 5'
AT4G23470.3     1162    1183
putative protein

sRNA_AGO1_Solexa_Mi2008_1_1652_hit1
5' ACGGUGCGUGAAUUGUAUU
   |||||||||||||||||||
   UGCCACGCACUUAACAUAA 5'
AT4G23470.1     1227    1245
putative protein

sRNA_AGO1_Solexa_Mi2008_2_24561_hit1
5' UAUAGGGUGCGUGAA-UGUAAU
   |||| |||||||||| |||| |
   AUAUGCCACGCACUUAACAUAA 5'
AT4G23470.1     1228    1249
putative protein

sRNA_AGO1_Solexa_Mi2008_1_1652_hit1
5' ACGGUGCGUGAAUUGUAUU
   |||||||||||||||||||
   UGCCACGCACUUAACAUAA 5'
AT4G23470.2     1575    1593
putative protein

sRNA_AGO1_Solexa_Mi2008_2_24561_hit1
5' UAUAGGGUGCGUGAA-UGUAAU
   |||| |||||||||| |||| |
   AUAUGCCACGCACUUAACAUAA 5'
AT4G23470.2     1576    1597
putative protein

sRNA_AGO1_Solexa_Mi2008_2_7736_hit1
5' CCAGAA-AAGAAGAAACAAU
    ||||| ||||||||||||
   UGUCUUCUUCUUCUUUGUUC 5'
AT4G23470.2     660     679
putative protein

sRNA_AGO1_Solexa_Mi2008_1_44600_hit1
5' UGUCCAUCUCCAUCUCGUUGUU
   ||||||| ||||||| | ||||
   ACAGGUAAAGGUAGAAC-ACAA 5'
AT4G23470.3     721     741
putative protein
```

```
sRNA_AGO1_Solexa_Mi2008_2_56577_hit1
5' UUUUGG-UCGGUGUGUAUGU
   |||||| |||||| |||||
   AAAACCCAGCCACUCAUACU 5'
AT4G24190.2     2303    2322
HSP90-like protein

sRNA_AGO1_Solexa_Mi2008_2_56577_hit1
5' UUUUGG-UCGGUGUGUAUGU
   |||||| |||||| |||||
   AAAACCCAGCCACUCAUACU 5'
AT4G24190.1     2307    2326
HSP90-like protein

sRNA_AGO1_Solexa_Mi2008_1_16810_hit10
5' UAAAG-GAGAACAUGACGGUGG
   | ||| |||||||||| |||||
   AGUUCGCUCUUGUACUACCACC 5'
AT4G24330.1     153     174
unknown protein

sRNA_AGO1_Solexa_Mi2008_1_7006_hit1
5' CAUCAUCAUCAUCACAGAAG
     ||| ||||||||||||||
   CGAGU-GUAGUAGUGUCUUC 5'
AT4G24690.1     2174    2192
unknown protein

sRNA_AGO1_Solexa_Mi2008_1_50495_hit1
5' UUGAGACCCGACGCGAUGGC
   |||||||||||| |||||
   AACUCUGGGCUG-GCUACGA 5'
AT4G25000.1     760     778
alpha-amylase  like protein

sRNA_AGO1_Solexa_Mi2008_8_15580_hit6
5' GUCAAAGUGAGAU-AUGGUUUU
   ||||||||||| |  |||||||
   CAGUUUCACUC-ACGACCAAAA 5'
AT4G28250.2     476     496
putative Expansin (AtEXPB3) /allergen protein

sRNA_AGO1_Solexa_Mi2008_1_43726_hit3
5' UGGUUAAGGAGAUAGACUUGA
   ||||| || |||||||||||
   ACCAAAUC-UCUAUCUGAACC 5'
AT4G28490.1     86      105
receptor-like protein kinase 5 precursor (RLK5)

sRNA_AGO1_Solexa_Mi2008_3_36318_hit1
5' UGAGGAUGACUAUGGUGAUGAG
   ||| |||||| ||||||||||
   ACUACUACUGUUACCACUACUA 5'
AT4G28530.2     798     819
NAM / CUC2 -like protein

sRNA_AGO1_Solexa_Mi2008_3_36318_hit1
5' UGAGGAUGACUAUGGUGAUGAG
   ||| |||||| ||||||||||
   ACUACUACUGUUACCACUACUA 5'
AT4G28530.1     909     930
NAM / CUC2 -like protein
```

```
sRNA_AGO1_Solexa_Mi2008_4_24200_hit5
5' UAUAAACGAAGGAA-UUUGUA
    ||||||||| ||| ||||||
   UUAUUUGCUU-CUUGAAACAU 5'
AT4G29100.1     1486    1505
putative bHLH transcription factor  (bHLH068)

sRNA_AGO1_Solexa_Mi2008_1_34531_hit1
5' UGAAGACAAGACAUAGCCAUU
    ||||| | ||||||||||||
   CCUUCUAUACUGUAUCGGUAA 5'
AT4G29100.1     659     679
putative bHLH transcription factor  (bHLH068)

sRNA_AGO1_Solexa_Mi2008_1_45985_hit1
5' UGUUGAUGUUGUUGUCGUUAG
   ||||||||||:|:|||||||
   ACAACUACAAUAGCAGCAAUG 5'
AT4G29100.1     738     758
putative bHLH transcription factor  (bHLH068)

sRNA_AGO1_Solexa_Mi2008_5_53772_hit1
5' UUGUUCUGA-AAGUGAAGACCA
     ||||||| ||||||||||||
   CCCAAGACUGUUCACUUCUGGU 5'
AT4G29100.1     797     818
putative bHLH transcription factor  (bHLH068)

sRNA_AGO1_Solexa_Mi2008_18_22571_hit1
5' UAG-GAAAAACGAAUGGUGGUA
   ||| |||||| | |||||||||
   AUCACUUUUUUCCUACCACCAU 5'
AT4G29410.2     619     640
unknown protein

sRNA_AGO1_Solexa_Mi2008_18_22571_hit1
5' UAG-GAAAAACGAAUGGUGGUA
   ||| |||||| | |||||||||
   AUCACUUUUUUCCUACCACCAU 5'
AT4G29410.1     629     650
unknown protein

sRNA_AGO1_Solexa_Mi2008_1_55382_hit1
5' UUUGCAUAGGGUGUGUUGUGAC
    |||||||| ||||| ||||||
   UAACGUAUCACACACUACACUG 5'
AT4G29700.1     1637    1658
nucleotide pyrophosphatase -like protein

sRNA_AGO1_Solexa_Mi2008_8_52273_hit1
5' UUGGCAAGUUAGAGGAUGUG-UC
   |||||||||| |||||| || ||
   AACCGUUCAAGCUCCUAGACGAG 5'
AT4G30190.1     1415    1437
H+-transporting ATPase type 2, plasma membrane

sRNA_AGO1_Solexa_Mi2008_12_41443_hit21
5' UGGCGAUUUCAGCUCUUCU
   |:|||||:|||||||||||
   AUCGCUAGAGUCGAGAAGA 5'
AT4G30190.1     2869    2887
H+-transporting ATPase type 2, plasma membrane
```

```
sRNA_AGO1_Solexa_Mi2008_2_13419_hit2
5' GAAGAAGAAGAAGACACUU
   |||||||||||||||
   CUUCUUCUUCUUCUGUUUU 5'
AT4G30190.1     3021    3039
H+-transporting ATPase type 2, plasma membrane

sRNA_AGO1_Solexa_Mi2008_7_353_hit2
5' AAAGAAGAAGAUAAAGCAU
   |||||||||||| ||| ||
   UUUCUUCUUCUAAUUC-UA 5'
AT4G30190.1     3256    3273
H+-transporting ATPase type 2, plasma membrane

sRNA_AGO1_Solexa_Mi2008_1_173_hit2
5' AAAAGAAGAAGAUAAAGCAU
   ||||||||||||| ||| ||
   UUUUCUUCUUCUAAUUC-UA 5'
AT4G30190.1     3256    3274
H+-transporting ATPase type 2, plasma membrane

sRNA_AGO1_Solexa_Mi2008_1_16281_hit2
5' UAAAAAGAAGAAGAUAAAGCAU
   :|||||||||||||| ||| ||
   GUUUUUCUUCUUCUAAUUC-UA 5'
AT4G30190.1     3256    3276
H+-transporting ATPase type 2, plasma membrane

sRNA_AGO1_Solexa_Mi2008_1_53875_hit1
5' UUUAAACAGGUAAAGAAAAACAG
   ||||| |||||||| ||||| ||
   AAAUUAGUCCAUUU-UUUUU-UC 5'
AT4G30350.1     113     133
unknown protein

sRNA_AGO1_Solexa_Mi2008_2_116_hit3
5' AAA-AAGAUAAGAGAGAUAG
    || |||||||||||| |||
   GUUCUUCUAUUCUCUC-AUC 5'
AT4G30350.1     416     434
unknown protein

sRNA_AGO1_Solexa_Mi2008_2_13419_hit2
5' GAAGAAGAAGAAGAC-ACUU
   ||||||||||||||| ||
   CUUCUUCUUCUUCUGCUGCU 5'
AT4G30350.1     571     590
unknown protein

sRNA_AGO1_Solexa_Mi2008_1_1549_hit1
5' ACCGGAGAAGAAGAAGAUGAC
     ||||||||||||||| |||
   CUGCCUCUUCUUCUUCUUCUG 5'
AT4G30350.1     575     595
unknown protein

sRNA_AGO1_Solexa_Mi2008_1_49131_hit1
5' UUCCGUGGUUGUGAUUGUGGC
   ||||||||||||||||||||
   AAGGCACCAACACUAACACCG 5'
AT4G30960.1     1220    1240
CBL-interacting protein kinase 6 (CIPK6)
```

```
sRNA_AGO1_Solexa_Mi2008_2_10634_hit2
5' CUCAAGAGAAUCAACAUCCUC
     ||||| ||||||||||||
   ACGUUCU-UUAGUUGUAGGAG 5'
AT4G30960.1     1242    1261
CBL-interacting protein kinase 6 (CIPK6)

sRNA_AGO1_Solexa_Mi2008_3_20883_hit1
5' UAGAAGAAUGGUGAUGUACGUG
   ||||||||||| |||| || ||
   AUCUUCUUACCCCUAC-UG-AC 5'
AT4G31500.1     1124    1143
cytochrome P450 monooxygenase (CYP83B1)

sRNA_AGO1_Solexa_Mi2008_1_32802_hit1
5' UCUACCAGCAGAAACGUCCUA
   |||||||||||||||||||||
   AGAUGGUCGUCUUUGCAGGAU 5'
AT4G31500.1     1554    1574
cytochrome P450 monooxygenase (CYP83B1)

sRNA_AGO1_Solexa_Mi2008_1_27836_hit1
5' UCA-CGGUGUCUGAUUGAUCG
   ||| |||||:||||||||||:
   AGUUGCCACGGACUAACUAGU 5'
AT4G31500.1     522     542
cytochrome P450 monooxygenase (CYP83B1)

sRNA_AGO1_Solexa_Mi2008_3_13895_hit1
5' GAGAAACCACCGAUGGA-GAUGGU
   |||||||||| |||||| || |||
   CUCUUUGGUGUCUACCUACUUCCA 5'
AT4G34140.1     629     652
hypothetical protein

sRNA_AGO1_Solexa_Mi2008_6_4720_hit1
5' CAAGCAUCACAGGAGUAAUA
   ||||||||||||||||||||
   GUUCGUAGUGUCCUCAUUAU 5'
AT4G35630.1     1006    1025
phosphoserine aminotransferase

sRNA_AGO1_Solexa_Mi2008_5_15466_hit1
5' GUAGCCACCCUGAUUGUUGUU
   |||||| || ||||||||||
   CAUCGGCGGUACUAACAACAC 5'
AT4G35630.1     136     156
phosphoserine aminotransferase

sRNA_AGO1_Solexa_Mi2008_1_30321_hit1
5' UCCUUCUCUUGUUCAAGUGCA
   |||||||||||||||||||||
   AGGAAGAGAACAAGUUCACGU 5'
AT4G36520.1     2272    2292
trichohyalin like protein

sRNA_AGO1_Solexa_Mi2008_13_10755_hit1
5' CUCAGUUCGGGCGAGGCAU
   |||||||| ||||| ||||
   GAGUCAAGGCCGCU-CGUA 5'
AT4G37430.1     1024    1041
cytochrome P450 monooxygenase (CYP91A2)
```

```
sRNA_AGO1_Solexa_Mi2008_4_44164_hit1
5' UGUAGCAACGGUUGUGGUGGU
   |||| |||||||||| |||||
   ACAUGGUUGCCAACA-CACCA 5'
AT4G37430.1     455     474
cytochrome P450 monooxygenase (CYP91A2)

sRNA_AGO1_Solexa_Mi2008_12_19173_hit1
5' UACAAGGUGUUGU-GAAGUCUA
   ||||||||||||| :||||||
   AUGUUCCACAACAGUUUCAGAA 5'
AT4G37910.1     902     923
heat shock protein 70 like protein

sRNA_AGO1_Solexa_Mi2008_1_36855_hit2
5' UGAGUGUUGUGGUCAAAUGGG
     ||||||||||||| |||||
   UGUCACAACACCAGUGUACCC 5'
AT4G38540.1     692     712
monooxygenase 2 (MO2)

sRNA_AGO1_Solexa_Mi2008_1_31918_hit1
5' UCGGCCGGUUGAUGGAUUGU-UG
   ||||| | |||||||||||| ||
   AGCCG-CAAACUACCUAACAGAC 5'
AT4G39950.1     1078    1099
cytochrome P450 like protein

sRNA_AGO1_Solexa_Mi2008_5_8180_hit2
5' CCUGAUGAUGAUGUACAA-CAC
   |||||||||||||| ||| |||
   GGACUACUACUACAGGUUAGUG 5'
AT5G02190.1     1354    1375
unknown protein

sRNA_AGO1_Solexa_Mi2008_3_30265_hit2
5' UCCUGAUGAUGAUGUACAA-CAC
    |||||||||||||| ||| |||
   UGGACUACUACUACAGGUUAGUG 5'
AT5G02190.1     1354    1376
unknown protein

sRNA_AGO1_Solexa_Mi2008_7_353_hit2
5' AAAGAAGAAGAUAAAGCAU
    ||||||||||| ||| ||
   GUUCUUCUUCUACUUC-UA 5'
AT5G02190.1     177     194
unknown protein

sRNA_AGO1_Solexa_Mi2008_1_173_hit2
5' AA-AAGAAGAAGAUAAAGCAU
   || ||||||||||| ||| ||
   UUCUUCUUCUUCUACUUC-UA 5'
AT5G02190.1     177     196
unknown protein

sRNA_AGO1_Solexa_Mi2008_2_6093_hit1
5' CAGAGGUUGAGGUUGACGUGC
    |||||||||||||||| ||
   UUCUCCAACUCCAACUGC-CG 5'
AT5G03350.1     85      104
protein kinase - like
```

```
sRNA_AGO1_Solexa_Mi2008_9_14254_hit8
5' GAUGAUGAUGAUGAUGAUC-UU
   ||||||||||||||||||| ||
   CUACUACUACUACUACUAGUAA 5'
AT5G03545.1     292     313
unknown protein

sRNA_AGO1_Solexa_Mi2008_6_14255_hit369
5' GAUGAUGAUGAUGAUGAUGAUGAU
   ||||||||||||||||||||| ||
   CUACUACUACUACUACUACUAGUA 5'
AT5G03545.1     292     315
unknown protein

sRNA_AGO1_Solexa_Mi2008_1_14256_hit8
5' GAUGAUGAUGAUGAUGAUGAUGUU
   |||||||||||||||||||||  |
   CUACUACUACUACUACUACUAGUA 5'
AT5G03545.1     292     315
unknown protein

sRNA_AGO1_Solexa_Mi2008_1_36323_hit13
5' UGAGGAUGAUGAUGAUGAUGA
   ||| ||||||||||||||| |
   ACUACUACUACUACUACUAGU 5'
AT5G03545.1     293     313
unknown protein

sRNA_AGO1_Solexa_Mi2008_1_3079_hit472
5' AUGAUGAUGAUGAUGAUGAUGA
   |||||||||||||||||||| |
   UACUACUACUACUACUACUAGU 5'
AT5G03545.1     293     314
unknown protein

sRNA_AGO1_Solexa_Mi2008_1_45039_hit3
5' UG-UGAUGAUGAUGAUGAUGAUGA
   || ||||||||||||||||||| |
   ACUACUACUACUACUACUACUAGU 5'
AT5G03545.1     294     317
unknown protein

sRNA_AGO1_Solexa_Mi2008_9_14254_hit8
5' GAUGAUGAUGAUGAUGAUCUU
   ||||||||||||||||||  |
   CUACUACUACUACUACUACUA 5'
AT5G03545.1     295     315
unknown protein

sRNA_AGO1_Solexa_Mi2008_6_14255_hit369
5' GAUGAUGAUGAUGAUGAUGAUGAU
   :|||||||||||||||||||||||
   UUACUACUACUACUACUACUACUA 5'
AT5G03545.1     295     318
unknown protein

sRNA_AGO1_Solexa_Mi2008_1_14256_hit8
5' GAUGAUGAUGAUGAUGAUGAUGUU
   :||||||||||||||||||||| |
   UUACUACUACUACUACUACUACUA 5'
AT5G03545.1     295     318
unknown protein
```

sRNA_AGO1_Solexa_Mi2008_7_3078_hit1
```
5' AUGAUGAUGA-GAAUGAUGAU
   |||||||||| | ||||||||
   UACUACUACUAC-UACUACUA 5'
```
AT5G03545.1   296   315
unknown protein

sRNA_AGO1_Solexa_Mi2008_1_36323_hit13
```
5' UGAGGAUGAUGAUGAUGAUGA
   ||| ||||||||||||||||
   ACUACUACUACUACUACUACU 5'
```
AT5G03545.1   296   316
unknown protein

sRNA_AGO1_Solexa_Mi2008_1_3079_hit472
```
5' AUGAUGAUGAUGAUGAUGAUGA
   |||||||||||||||||||||
   UACUACUACUACUACUACUACU 5'
```
AT5G03545.1   296   317
unknown protein

sRNA_AGO1_Solexa_Mi2008_1_45039_hit3
```
5' UGUGAUGAUGAUGAUGAUGAUGA
     :||||||||||||||||||||
   UUACUACUACUACUACUACUACU 5'
```
AT5G03545.1   296   318
unknown protein

sRNA_AGO1_Solexa_Mi2008_1_56092_hit3
```
5' UUUG-UGAUGAUGAUGAUGAUGA
   |||| ||||||||||||||||||
   AAACUACUACUACUACUACUACU 5'
```
AT5G03545.1   297   319
unknown protein

sRNA_AGO1_Solexa_Mi2008_6_14255_hit369
```
5' GAUGAUGAUGAUGAUGAUGAUGAU
   |||:||||||||||||||||||||
   CUAUUACUACUACUACUACUACUA 5'
```
AT5G03545.1   298   321
unknown protein

sRNA_AGO1_Solexa_Mi2008_1_14256_hit8
```
5' GAUGAUGAUGAUGAUGAUGAUGUU
   |||:|||||||||||||||||| |
   CUAUUACUACUACUACUACUACUA 5'
```
AT5G03545.1   298   321
unknown protein

sRNA_AGO1_Solexa_Mi2008_7_3078_hit1
```
5' AUGAUGAUGA-GAAUGAUGAU
   |||||||||| || |||||||
   UACUACUACUACU-ACUACUA 5'
```
AT5G03545.1   299   318
unknown protein

sRNA_AGO1_Solexa_Mi2008_1_3079_hit472
```
5' AUGAUGAUGAUGAUGAUGAUGA
     |||:||||||||||||||||
   ACCUAUUACUACUACUACUACU 5'
```
AT5G03545.1   302   323
unknown protein

```
sRNA_AGO1_Solexa_Mi2008_4_14169_hit1
5' GAUAAUGAUGAUGAAAGAUGA
   |||||||||||||  |||||
   CUAUUACUACUACUA-CUACU 5'
AT5G03545.1     303     322
unknown protein

sRNA_AGO1_Solexa_Mi2008_1_39699_hit1
5' UGGA-ACUGAUGAUGAUGAUGA
   |||| | ||||||||||||||
   ACCUAUUACUACUACUACUACU 5'
AT5G03545.1     303     324
unknown protein

sRNA_AGO1_Solexa_Mi2008_1_45039_hit3
5' UGUGAUGAUGAUGAUGAUGAUGA
   || |||:|||||||||||||||
   AC-CUAUUACUACUACUACUACU 5'
AT5G03545.1     303     324
unknown protein

sRNA_AGO1_Solexa_Mi2008_1_52789_hit1
5' UUGGGUGAUGAUGAUGAUUGAU
   ||||:|:||||||||||| |||
   AACCUAUUACUACUACUA-CUA 5'
AT5G03545.1     305     325
unknown protein

sRNA_AGO1_Solexa_Mi2008_1_2280_hit1
5' AGUGGAUGAUGAUGAUGAUG
   | |||||:||||||||||||
   UAACCUAUUACUACUACUAC 5'
AT5G03545.1     306     325
unknown protein

sRNA_AGO1_Solexa_Mi2008_1_56092_hit3
5' UUUGUGAUGAUGAUGAUGAUGA
   |||| |||:|||||||||||||
   AAAC-CUAUUACUACUACUACU 5'
AT5G03545.1     306     326
unknown protein

sRNA_AGO1_Solexa_Mi2008_1_7082_hit4
5' CAUCUCCAGGA-ACCUCUUGAU
   | ||||||||| || |||||||
   GGAGAGGUCCUAUG-AGAACUA 5'
AT5G05320.1     800     820
monooxygenase

sRNA_AGO1_Solexa_Mi2008_1_646_hit1
5' AACAGAAAAAAAACA-UGAU
   ||||:|||||||||| |||
   UUGUUUUUUUUUGUAACUU 5'
AT5G05340.1     1138    1157
peroxidase

sRNA_AGO1_Solexa_Mi2008_1_21951_hit1
5' UAGAUUUACAGUUGAUUGUUU
   ||||:||| |||||||||||
   AUCUGAAUCUCAACUAACAAC 5'
AT5G05340.1     5       25
peroxidase
```

```
sRNA_AGO1_Solexa_Mi2008_13_14779_hit6
5' GCUGAGAAUCGAAAU-AGUU
    || ||||||||||| ||||
   AGA-UCUUAGCUUUAUUCAA 5'
AT5G05340.1     70      88
peroxidase

sRNA_AGO1_Solexa_Mi2008_6_11866_hit1
5' CUGGAACAG-AUUCUCGAAGGUC
   ||||||||| ||  |||||||||
   GACCUUGUCGUAU-AGCUUCCAG 5'
AT5G05520.1     1061    1082
Unknown protein (MOP10.6)

sRNA_AGO1_Solexa_Mi2008_7_6238_hit2
5' CAGCAACAGGUGGAAAGUCAA
    || ||| |||||||||||||
   AUC-UUGGCCACCUUUCAGUU 5'
AT5G06600.2     1253    1272
ubiquitin carboxyl-terminal hydrolase

sRNA_AGO1_Solexa_Mi2008_7_6238_hit2
5' CAGCAACAGGUGGAAAGUCAA
    || ||| |||||||||||||
   AUC-UUGGCCACCUUUCAGUU 5'
AT5G06600.3     1254    1273
ubiquitin carboxyl-terminal hydrolase

sRNA_AGO1_Solexa_Mi2008_3_7100_hit6
5' CAU-GAAAUUGAUGUUGCGC
   ||| ||| |||||||||||
   GUAACUU-AACUACAACGCA 5'
AT5G06600.2     520     538
ubiquitin carboxyl-terminal hydrolase

sRNA_AGO1_Solexa_Mi2008_3_7100_hit6
5' CAU-GAAAUUGAUGUUGCGC
   ||| ||| |||||||||||
   GUAACUU-AACUACAACGCA 5'
AT5G06600.3     521     539
ubiquitin carboxyl-terminal hydrolase

sRNA_AGO1_Solexa_Mi2008_1_11845_hit13
5' CUG-CUUCUUGGCCUCUGUGAU
   ||| ||||||| |||||| |||
   GACCGAAGAACAGGAGAC-CUA 5'
AT5G07340.1     767     787
calnexin homolog

sRNA_AGO1_Solexa_Mi2008_1_44503_hit1
5' UGUCACUGUU-GAUGAUGAGAC
   ||| | |||| |||||||||||
   ACA-UAACAAACUACUACUCUG 5'
AT5G07460.1     101     121
peptide methionine sulfoxide reductase-like protein

sRNA_AGO1_Solexa_Mi2008_5_50473_hit1
5' UUGAGAAGGAGAAGUUUCA
   : |||||||||||||||||
   GCCUCUUCCUCUUCAAAGG 5'
AT5G07460.1     166     184
peptide methionine sulfoxide reductase-like protein
```

```
sRNA_AGO1_Solexa_Mi2008_1_28303_hit1
5' UCA-GCCAAGGAUUCUCUGAGAA
   ||| |||| |||||||||| |||
   AGUGCGGUCCCUAAGAGAC-CUU 5'
AT5G07460.1     330     351
peptide methionine sulfoxide reductase-like protein

sRNA_AGO1_Solexa_Mi2008_2_3654_hit1
5' AUUGAAGGUGGUAGGUACUUA
   ||| ||||||||||| | |||
   UAAGUUCCACCAUCC-U-AAU 5'
AT5G07460.1     509     527
peptide methionine sulfoxide reductase-like protein

sRNA_AGO1_Solexa_Mi2008_339_11394_hit3
5' CUGAAGUGUUUGGGGGAACUC
    |||||||||||||:|||||
   AACUUCACAAACCCUCUUGAA 5'
AT5G10180.1     124     144
sulfate transporter

sRNA_AGO1_Solexa_Mi2008_1_22796_hit1
5' UAGGAUAUUGAUAUAGUGU
   |||||||||||| ||||:|
   AUCCUAUAACUA-AUCAUA 5'
AT5G10180.1     1712    1729
sulfate transporter

sRNA_AGO1_Solexa_Mi2008_1_4581_hit2
5' CAAGA-AUACUACAGCCAU-GGUC
   ||||| ||||||||||||: ||||
   GUUCUCUAUGAUGUCGGUGGCCAG 5'
AT5G10180.1     590     613
sulfate transporter

sRNA_AGO1_Solexa_Mi2008_15_14657_hit1
5' GCGAAGAAGGAUCUGGUUA
   |||||||||| | ||||||
   CGCUUCUUCCAACACCAAU 5'
AT5G10180.1     682     700
sulfate transporter

sRNA_AGO1_Solexa_Mi2008_6_50680_hit1
5' UUGAGGCAAAGAACAUCCGAA
    ||||| |||||||||||||
   CACUCC-UUUCUUGUAGGCUC 5'
AT5G10180.1     931     950
sulfate transporter

sRNA_AGO1_Solexa_Mi2008_1_20849_hit1
5' UAGAACCU-CGGUCGAGAAUG-GU
   ||| |||| |||||||||||| ||
   AUCGUGGAAGCCAGCUCUUACUCA 5'
AT5G10760.1     1046    1069
nucleoid DNA-binding protein cnd41 - like protein

sRNA_AGO1_Solexa_Mi2008_1_4400_hit1
5' CAACAUUUAGCGUCGUCUGC
   ||||||:|||||||||||||
   GUUGUAGAUCGCAGCAGACG 5'
AT5G10760.1     1367    1386
nucleoid DNA-binding protein cnd41 - like protein
```

```
sRNA_AGO1_Solexa_Mi2008_1_3917_hit1
5' CAAAAGAAGCAA-AAGUUUGUU
   ||||||||| || || ||||||
   GUUUUCUUC-UUCUUAAAACAA 5'
AT5G13080.1     50      70
WRKY-like protein

sRNA_AGO1_Solexa_Mi2008_1_17162_hit1
5' UAACA-AGAGGAAGAAGAACGAU
   ||||| |||||||||||| ||
   AUUGUCUCUCCUUCUUCUU-CUU 5'
AT5G13180.1     24      45
NAM-like protein

sRNA_AGO1_Solexa_Mi2008_1_14_hit1
5' AAAAAAACAGAGAACAAGAAGA
    ||||||||||||  |||||||
   CUUUUUUGUCUCUCCUUCUUCU 5'
AT5G13180.1     27      48
NAM-like protein

sRNA_AGO1_Solexa_Mi2008_1_341_hit1
5' AAAGAAACAGAGAGGAAGAU
   ||| |||||||||||||||
   UUU-UUUGUCUCUCCUUCUU 5'
AT5G13180.1     30      48
NAM-like protein

sRNA_AGO1_Solexa_Mi2008_3_10681_hit1
5' CUCACGAUUUGAUUUCCUCU
   |: |||||||||||||||||
   GGUUGCUAAACUAAAGGAGA 5'
AT5G13180.1     383     402
NAM-like protein

sRNA_AGO1_Solexa_Mi2008_21_23402_hit1
5' UAGGUCGAGCUUCAUUGGA
    ||| ||||||||||||||
   CUCCUGCUCGAAGUAACCU 5'
AT5G13550.1     197     215
sulfate transporter

sRNA_AGO1_Solexa_Mi2008_1_21106_hit1
5' UAGAC-GUAAAUUGGUGGCAU
   ||||| |||||||||||| |
   AUCUGGCAUUUAACCACCUUC 5'
AT5G13550.1     426     446
sulfate transporter

sRNA_AGO1_Solexa_Mi2008_1_40339_hit1
5' UGGAGAUGGAAGAUGAG-CC
   |||||||||||| |||| ||
   ACCUCUACCUUCCACUCUGG 5'
AT5G13930.1     876     895
chalcone synthase (naringenin-chalcone synthase) (testa 4 protein) (sp|P13114)

sRNA_AGO1_Solexa_Mi2008_1_45665_hit1
5' UGUGUGAAGAGAGAAUGAUGG
   |||||||||||||||||||||
   ACACACUUCUCUCUUACUACC 5'
AT5G14930.2     422     442
SAG101
```

```
sRNA_AGO1_Solexa_Mi2008_1_45665_hit1
5' UGUGUGAAGAGAGAAUGAUGG
   |||||||||||||||||||||
   ACACACUUCUCUCUUACUACC 5'
AT5G14930.3     423     443
SAG101

sRNA_AGO1_Solexa_Mi2008_4_12767_hit1
5' CUUGAGCGUUUGG-GUCAACUA
   ||||||||||||| | |||||
   GAACUCGCAAACCUC-GUUGAG 5'
AT5G14930.2     498     518
SAG101

sRNA_AGO1_Solexa_Mi2008_4_12767_hit1
5' CUUGAGCGUUUGG-GUCAACUA
   ||||||||||||| | |||||
   GAACUCGCAAACCUC-GUUGAG 5'
AT5G14930.3     499     519
SAG101

sRNA_AGO1_Solexa_Mi2008_5_29024_hit1
5' UC-AUGGUUUCAAGAAUGCU
   || ||||||||||||| ||:
   AGCUACCAAAGUUCUU-CGG 5'
AT5G14930.2     586     604
SAG101

sRNA_AGO1_Solexa_Mi2008_5_29024_hit1
5' UC-AUGGUUUCAAGAAUGCU
   || ||||||||||||| ||:
   AGCUACCAAAGUUCUU-CGG 5'
AT5G14930.3     587     605
SAG101

sRNA_AGO1_Solexa_Mi2008_1_34515_hit1
5' UGAAG-AAGCAAAUGGGUA
    |||| |||||||| |||||
   UCUUCCUUCGUUUA-CCCAU 5'
AT5G17860.1     12      30
potassium-dependent sodium-calcium exchanger - like protein

sRNA_AGO1_Solexa_Mi2008_7_30129_hit1
5' UCCUA-CACAGAUGGGCUUAUC
   ||||| ||| |||||||||||
   AGGAUCGUGCCUACCCGAAUAU 5'
AT5G17860.1     1338    1359
potassium-dependent sodium-calcium exchanger - like protein

sRNA_AGO1_Solexa_Mi2008_1_1962_hit2
5' AGAGUGCUCAAAGCAAGCCU
    :|||||:||||||||||||
   GUUCACGGGUUUCGUUCGGA 5'
AT5G17860.1     1598    1617
potassium-dependent sodium-calcium exchanger - like protein

sRNA_AGO1_Solexa_Mi2008_1_27200_hit1
5' UCAAGG-CAAGGAAACAGAGGUC
   |||||| |||| ||||||||||
   AGUUCCUGUUC-UUUGUCUCCAU 5'
AT5G17860.1     1681    1702
potassium-dependent sodium-calcium exchanger - like protein
```

```
sRNA_AGO1_Solexa_Mi2008_3_13421_hit1
5' GAAGAAGAAGAUGAUGUUGAU
   :|||| ||||| |||||||||
   UUUCUACUUCUUCUACAACUA 5'
AT5G17860.1     81      101
potassium-dependent sodium-calcium exchanger - like protein

sRNA_AGO1_Solexa_Mi2008_1_22493_hit1
5' UAGCUCCUGAU-GGUCGAGUA
   ||||||||||| |||| |||
   AUCGAGGACUACCCAGAUCAG 5'
AT5G18470.1     778     798
unknown protein

sRNA_AGO1_Solexa_Mi2008_2_2731_hit1
5' AUAUGUUCUCUGUCUGCUUCU
   |||||||||  ||||||||||
   UAUACAAGA--CAGACGAAGA 5'
AT5G19440.1     40      58
cinnamyl-alcohol dehydrogenase - like protein

sRNA_AGO1_Solexa_Mi2008_3_17070_hit1
5' UAA-AUGGCCAAGUUG-AUGUU
   ||| |||||||||||| | |||
   AUUCUACCGGUUCAACCUCCAA 5'
AT5G19690.1     1215    1236
oligosaccharyl transferase STT3-like protein

sRNA_AGO1_Solexa_Mi2008_4_46322_hit1
5' UUAA-AUGGCCAAGUUG-AUGUU
   |||| |||||||||||| | |||
   AAUUCUACCGGUUCAACCUCCAA 5'
AT5G19690.1     1215    1237
oligosaccharyl transferase STT3-like protein

sRNA_AGO1_Solexa_Mi2008_1_40533_hit1
5' UGGAGGAUGAGAUUGCAGUGCA
   ||||| |||| ||||||||||
   ACCUC-UACUGUAACGUCACGG 5'
AT5G19690.1     1979    1999
oligosaccharyl transferase STT3-like protein

sRNA_AGO1_Solexa_Mi2008_3_22541_hit1
5' UAGCUUCAGGACAGAAAGUA
   |||||||||:|||||||| |
   AUCGAAGUCUUGUCUUUCCU 5'
AT5G20280.1     1748    1767
sucrose-phosphate synthase-like protein

sRNA_AGO1_Solexa_Mi2008_2_49282_hit2
5' UUCGAUGUCGGCUCUUCCUAUC
   |||||| | | |||||||||||
   AAGCUAAAAC-GAGAAGGAUAG 5'
AT5G20280.1     2327    2347
sucrose-phosphate synthase-like protein

sRNA_AGO1_Solexa_Mi2008_1_54615_hit4
5' UUUAUUG-GGUACACAAAUAA
   ||||:|| ||||||||||||
   AAAUGACACCAUGUGUUUAUA 5'
AT5G23010.1     1621    1641
2-isopropylmalate synthase-like; homocitrate synthase-like
```

```
sRNA_AGO1_Solexa_Mi2008_2_255_hit1
5' AAA-AUAGUGUGGGAA-UGUUA
   ||| |||||||||||| ||||
   UUUCUAUCACACCCUUAACAAA 5'
AT5G23010.1     5       26
2-isopropylmalate synthase-like; homocitrate synthase-like

sRNA_AGO1_Solexa_Mi2008_9_13465_hit1
5' GAAGAUGG-GUGGGAAUUGUUU
    |||||:| |||||||||||||
   AUUCUAUCACACCCUUAACAAA 5'
AT5G23010.1     5       26
2-isopropylmalate synthase-like; homocitrate synthase-like

sRNA_AGO1_Solexa_Mi2008_2_13247_hit1
5' CUUUGUGGUGAUGUUUUUGUUA
   ||||||||| |||| ||||||
   GAAACACCAAUACACAAACAAA 5'
AT5G24090.1     51      72
acidic endochitinase (dbj|BAA21861.1)

sRNA_AGO1_Solexa_Mi2008_2_13246_hit1
5' CUUUGUGGUGAUGUUUUUGUU
   ||||||||| |||| ||||||
   GAAACACCAAUACACAAACAA 5'
AT5G24090.1     52      72
acidic endochitinase (dbj|BAA21861.1)

sRNA_AGO1_Solexa_Mi2008_41_6691_hit3
5' CAGUCAUAGAUAGUCUCUGCAA
    |||| |||||||||||| |||
   CUCAGAAUCUAUCAGAGAGGUU 5'
AT5G24530.1     496     517
flavanone 3-hydroxylase-like protein

sRNA_AGO1_Solexa_Mi2008_1_2_hit38
5' AAAA-AAAAAAAAAAACCAU
   |||| ||||||||||| |||
   UUUUAUUUUUUUUUUU-GUA 5'
AT5G25610.1     1436    1454
dehydration-induced protein RD22

sRNA_AGO1_Solexa_Mi2008_4_43921_hit1
5' UGUAAA-GGGUGUUAGUAGAAAG
   |||||| |||| |||||||||||
   ACAUUUUCCCAAAAUCAUCUUUC 5'
AT5G25610.1     22      44
dehydration-induced protein RD22

sRNA_AGO1_Solexa_Mi2008_1_50782_hit1
5' UUGAGG-GUUGGAAUAGACAUGG
   ||| || ||||||||||| ||||
   AAC-CCUCAACCUUAUCUUUACC 5'
AT5G25610.1     93      114
dehydration-induced protein RD22

sRNA_AGO1_Solexa_Mi2008_8_50781_hit1
5' UUGAGG-GUUGGAAUAGACAUG
   ||| || ||||||||||| |||
   AAC-CCUCAACCUUAUCUUUAC 5'
AT5G25610.1     94      114
dehydration-induced protein RD22
```

```
sRNA_AGO1_Solexa_Mi2008_1_1627_hit6
5' ACGGAAUAGCGAGUUGGUC
   |||||||||||||| | ||
   UGCCUUAUCGCUCA-C-AG 5'
AT5G25770.2     1034    1050
unknown protein

sRNA_AGO1_Solexa_Mi2008_1_1627_hit6
5' ACGGAAUAGCGAGUUGGUC
   ||||||||||||| | |||
   UGCCUUAUCGCUC-A-CAG 5'
AT5G25770.3     1043    1059
unknown protein

sRNA_AGO1_Solexa_Mi2008_1_1627_hit6
5' ACGGAAUAGCGAGUUGGUC
   |||||||||||||| | ||
   UGCCUUAUCGCUCA-C-AG 5'
AT5G25770.1     1277    1293
unknown protein

sRNA_AGO1_Solexa_Mi2008_1_33976_hit1
5' UCUUGGUGGACAUG-AGUGGAC
   |||||||||||||: ||||||
   AGAACCACCUGUAUAUCACCUA 5'
AT5G26000.2     1672    1693
myrosinase precursor

sRNA_AGO1_Solexa_Mi2008_1_16880_hit3
5' UAAAGGUC-U-AUCUAUGGAGCA
   ||||| || | ||||||||||||
   AUUUCGAGGAAUAGAUACCUCGU 5'
AT5G26000.2     1720    1742
myrosinase precursor

sRNA_AGO1_Solexa_Mi2008_1_45612_hit67
5' UGUGUAUAGCUCGUAGAUGGG
   ||||||||||| || |||||
   ACACAUAUCGACCAACUACCA 5'
AT5G26000.2     640     660
myrosinase precursor

sRNA_AGO1_Solexa_Mi2008_8_53727_hit2
5' UUGUUAAAGAAGAUGGAG-AAA
   ||||| |||||||| ||| |||
   AACAACUUCUUCUA-CUCGUUU 5'
AT5G27760.1     34      54
unknown protein

sRNA_AGO1_Solexa_Mi2008_1_56174_hit2
5' UUUGUUAAAGAAGAUGGAG-AA
   |||||| |||||||| ||| ||
   AAACAACUUCUUCUA-CUCGUU 5'
AT5G27760.1     35      55
unknown protein

sRNA_AGO1_Solexa_Mi2008_3_13907_hit1
5' GAGAAGAGAUAGA-AUAGAAU
   ||||||||||||| || |||
   CUCUUCUCUAUCUCUACCUUU 5'
AT5G33290.1     326     346
unknown protein
```

```
sRNA_AGO1_Solexa_Mi2008_13_13906_hit2
5' GAGAAGAGAUAGA-AUAGAA
   ||||||||||||| || |||
   CUCUUCUCUAUCUCUACCUU 5'
AT5G33290.1     327     346
unknown protein

sRNA_AGO1_Solexa_Mi2008_4_35785_hit2
5' UGAGAAGAGAUAGA-AUAGAA
   :||||||||||||| || |||
   GCUCUUCUCUAUCUCUACCUU 5'
AT5G33290.1     327     347
unknown protein

sRNA_AGO1_Solexa_Mi2008_40_50469_hit1
5' UUGAGAAGAGAUAGA-AUAGAA
   |:||||||||||||| || |||
   AGCUCUUCUCUAUCUCUACCUU 5'
AT5G33290.1     327     348
unknown protein

sRNA_AGO1_Solexa_Mi2008_1_2422_hit4
5' AUAAUAUUCAGAUAACUCGGU
   |||||||||||| |||| ||
   UAUUAUAAGUCU-UUGA-CCU 5'
AT5G33370.2     459     477
unknown protein

sRNA_AGO1_Solexa_Mi2008_2_40489_hit1
5' UGGAGGACAAGCUAGUGAUCGU
   |||||||||||| |:||||||
   ACCUCCUGUUCGUUUACUAGCU 5'
AT5G36220.1     847     868
cytochrome P450 monooxygenase (CYP81D1 )

sRNA_AGO1_Solexa_Mi2008_2_35253_hit1
5' UGACAGACUGAUGUGUUAGA
   |||| | ||||||||||||
   ACUGAC-GACUACACAAUCA 5'
AT5G36220.1     940     958
cytochrome P450 monooxygenase (CYP81D1 )

sRNA_AGO1_Solexa_Mi2008_2_11884_hit1
5' CUG-GAAUGUUCGAGGUUUC
    || |||| |||||||||||
   AACUCUUAAAAGCUCCAAAG 5'
AT5G36930.1     332     351
disease resistance like protein

sRNA_AGO1_Solexa_Mi2008_2_11884_hit1
5' CUG-GAAUGUUCGAGGUUUC
    || |||| |||||||||||
   AACUCUUAAAAGCUCCAAAG 5'
AT5G36930.2     356     375
disease resistance like protein

sRNA_AGO1_Solexa_Mi2008_1_21284_hit1
5' UAGAGAUUGGUGGUGA-GCAUC
   |||| ||||||||||| || ||
   AUCUAUAACCACCACUCCG-AG 5'
AT5G37020.2     1513    1533
auxin response factor 8 (ARF8)
```

```
sRNA_AGO1_Solexa_Mi2008_1_11678_hit1
5' CUGCAACUGUUGAAGAU--CCA
   |||| ||||||||||||  |||
   GACGGUGACAACUUCUACCGGU 5'
AT5G37020.2     1749    1770
auxin response factor 8 (ARF8)

sRNA_AGO1_Solexa_Mi2008_1_38941_hit1
5' UGCGGAUUUCCGGGUUCUAGAG
   ||||||||||||||||||||||
   ACGCCUAAAGGCCCAAGAUCUC 5'
AT5G37020.2     2005    2026
auxin response factor 8 (ARF8)

sRNA_AGO1_Solexa_Mi2008_1_34515_hit1
5' UGAAGAAGCAAAUG-GGGUA
   |||||||||||| | ||| |
   ACUUCUUCGUUU-CGCCCCU 5'
AT5G37020.2     2085    2103
auxin response factor 8 (ARF8)

sRNA_AGO1_Solexa_Mi2008_4_2102_hit1
5' AGCUGCCAGCAUGAUCUAU
   |||||||||| |||||||
   UCGACGGUCGGACUAGAUU 5'
AT5G37020.2     2368    2386
auxin response factor 8 (ARF8)

sRNA_AGO1_Solexa_Mi2008_4_13484_hit1
5' GAAGCUGCCAGCAUGAUCUAU
    ||||||||||| |||||||
   GUUCGACGGUCGGACUAGAUU 5'
AT5G37020.2     2368    2388
auxin response factor 8 (ARF8)

sRNA_AGO1_Solexa_Mi2008_61_34692_hit1
5' UGAAGCUGCCAGCAUGAUCUAA
     ||||||||||| ||||||||
   UGUUCGACGGUCGGACUAGAUU 5'
AT5G37020.2     2368    2389
auxin response factor 8 (ARF8)

sRNA_AGO1_Solexa_Mi2008_13_873_hit2
5' AAGCUGCCAGCAUGAUCUA
   ||||||||||| |||||||
   UUCGACGGUCGGACUAGAU 5'
AT5G37020.2     2369    2387
auxin response factor 8 (ARF8)

sRNA_AGO1_Solexa_Mi2008_1_874_hit1
5' AAGCUGCCAGCAUGAUCUG
   ||||||||||| ||||||:
   UUCGACGGUCGGACUAGAU 5'
AT5G37020.2     2369    2387
auxin response factor 8 (ARF8)

sRNA_AGO1_Solexa_Mi2008_1_4776_hit1
5' CAAGCUGCCAGCCUGAUCUA
   ||||||||||||||||||||
   GUUCGACGGUCGGACUAGAU 5'
AT5G37020.2     2369    2388
auxin response factor 8 (ARF8)
```

```
sRNA_AGO1_Solexa_Mi2008_1519_13483_hit2
5' GAAGCUGCCAGCAUGAUCUA
    ||||||||||| |||||||
   GUUCGACGGUCGGACUAGAU 5'
AT5G37020.2     2369    2388
auxin response factor 8 (ARF8)

sRNA_AGO1_Solexa_Mi2008_3_13485_hit1
5' GAAGCUGCCAGCAUGAUCUG
    ||||||||||| ||||||:
   GUUCGACGGUCGGACUAGAU 5'
AT5G37020.2     2369    2388
auxin response factor 8 (ARF8)

sRNA_AGO1_Solexa_Mi2008_344873_34691_hit2
5' UGAAGCUGCCAGCAUGAUCUA
     ||||||||||| |||||||
   UGUUCGACGGUCGGACUAGAU 5'
AT5G37020.2     2369    2389
auxin response factor 8 (ARF8)

sRNA_AGO1_Solexa_Mi2008_54_13482_hit3
5' GAAGCUGCCAGCAUGAUCU
    ||||||||||| ||||||
   GUUCGACGGUCGGACUAGA 5'
AT5G37020.2     2370    2388
auxin response factor 8 (ARF8)

sRNA_AGO1_Solexa_Mi2008_7845_34690_hit3
5' UGAAGCUGCCAGCAUGAUCU
     ||||||||||| ||||||
   UGUUCGACGGUCGGACUAGA 5'
AT5G37020.2     2370    2389
auxin response factor 8 (ARF8)

sRNA_AGO1_Solexa_Mi2008_17_46569_hit1
5' UUAAGCUGCCAGCAUGAUCU
    :||||||||||| ||||||
   UGUUCGACGGUCGGACUAGA 5'
AT5G37020.2     2370    2389
auxin response factor 8 (ARF8)

sRNA_AGO1_Solexa_Mi2008_6340_34689_hit3
5' UGAAGCUGCCAGCAUGAUC
     ||||||||||| |||||
   UGUUCGACGGUCGGACUAG 5'
AT5G37020.2     2371    2389
auxin response factor 8 (ARF8)

sRNA_AGO1_Solexa_Mi2008_8_46568_hit1
5' UUAAGCUGCCAGCAUGAUC
    :||||||||||| |||||
   UGUUCGACGGUCGGACUAG 5'
AT5G37020.2     2371    2389
auxin response factor 8 (ARF8)

sRNA_AGO1_Solexa_Mi2008_1_38269_hit1
5' UGCAUGUGCAGUAGCAUCAAG
    ||||||||||:|||| ||||
   UCGUACACGUCGUCGUCGUUC 5'
AT5G37020.2     867     887
auxin response factor 8 (ARF8)
```

```
sRNA_AGO1_Solexa_Mi2008_1_47627_hit1
5' UUAGCUAGCAGAAGCAUGUGCA
   |||| | |||||||||||||||
   AAUCAA-CGUCUUCGUACACGU 5'
AT5G37020.2     879     899
auxin response factor 8 (ARF8)

sRNA_AGO1_Solexa_Mi2008_1_51898_hit5
5' UUGCUUUCUUGAUGGAUACUCA
   |||||||||||||| || |||
   AACGAAAGAACUAC-UA-GAGC 5'
AT5G37600.1     974     993
glutamate--ammonia ligase

sRNA_AGO1_Solexa_Mi2008_13_4545_hit1
5' CAAGAAAGAUGGUUGUCGUU
     |||||| |||||||||||
   ACUCUUUCAACCAACAGCAA 5'
AT5G39050.1     534     553
Anthocyanin acyltransferase - like protein

sRNA_AGO1_Solexa_Mi2008_1_13420_hit2
5' GAAGAAGAAGAAGACUCUU
   || ||||||||||||||||
   CUCCUUCUUCUUCUGAGAA 5'
AT5G39050.1     830     848
Anthocyanin acyltransferase - like protein

sRNA_AGO1_Solexa_Mi2008_1_4090_hit1
5' CA-AAGAGUAGACAAUCAUCAA
   || |||||||:||||||| |||
   GUAUUCUCAUUUGUUAGU-GUU 5'
AT5G39050.1     888     908
Anthocyanin acyltransferase - like protein

sRNA_AGO1_Solexa_Mi2008_1_10_hit1
5' AAAAAAAAGUAGAUGUUGGU
   |||||||||||||||||  |
   UUUUUUUUCAUCUACAAAGA 5'
AT5G39220.1     1254    1273
unknown protein

sRNA_AGO1_Solexa_Mi2008_1_1790_hit2
5' AGAACAAGUGGUUAGAUACAC
    |||||||||||| ||||||
   ACUUGUUCACCAAACUAUGUC 5'
AT5G39220.1     306     326
unknown protein

sRNA_AGO1_Solexa_Mi2008_1_41315_hit1
5' UGGCCGCAACAGAGGUG-AUGAG
   ||||||||| ||||||| | |||
   ACCGGCGUUAUCUCCACGUUCUC 5'
AT5G39680.1     2193    2215
unknown protein

sRNA_AGO1_Solexa_Mi2008_2_19236_hit1
5' UACACAACAUCUGAUGGACUA
    |||||| ||||||||||||
   UUGUGUUCUAGACUACCUGAA 5'
AT5G40450.2     1334    1354
unknown protein
```

```
sRNA_AGO1_Solexa_Mi2008_6_10413_hit1
5' CUAGUUCGUCGAUAUGUUG
   || |||||||||||| ||
   GA-CAAGCAGCUAUA-AAG 5'
AT5G40450.2     2425    2441
unknown protein

sRNA_AGO1_Solexa_Mi2008_1_32975_hit1
5' UCUAGUUCGUCGAUAUGUU
   ||| |||||||||||| ||
   AGA-CAAGCAGCUAUA-AA 5'
AT5G40450.2     2426    2442
unknown protein

sRNA_AGO1_Solexa_Mi2008_1_39819_hit1
5' UGGAAUAAUUUCAGGAUAGGU
   |||||||||||| || ||||
   ACCUUAUUAAAG-CC-AUCCC 5'
AT5G40450.2     4704    4722
unknown protein

sRNA_AGO1_Solexa_Mi2008_2_38233_hit2
5' UGCAUCUACCACAUUCAUCUA
   |||| ||||| |||||||||
   ACGUUGAUGGAGUAAGUAGAG 5'
AT5G40450.2     6330    6350
unknown protein

sRNA_AGO1_Solexa_Mi2008_3_13459_hit1
5' GAAGAUGAUAUGUACAAUG-UA
   |||| |||||||||||| | ||
   CUUCCACUAUACAUGUU-CUAU 5'
AT5G40480.1     1134    1154
nuclear pore protein -like

sRNA_AGO1_Solexa_Mi2008_1_27991_hit1
5' UCAGAAACCCUAAUUUCGUGGG
   ||| ||||||||||||| |||:
   AGUGUUUGGGAUUAAAGAACCU 5'
AT5G40480.1     1489    1510
nuclear pore protein -like

sRNA_AGO1_Solexa_Mi2008_1_36711_hit4
5' UGAGUAGCAAGAGAUGGAGAAA
   |||| ||||||||| |||||||
   ACUC-UCGUUCUCUUCCUCUUU 5'
AT5G40480.1     154     174
nuclear pore protein -like

sRNA_AGO1_Solexa_Mi2008_1_53211_hit1
5' UUGGUUGGUUC-UUGACAGUCA
   ||||||||| | |||||||||
   AACCAACCA-GUAACUGUCAGA 5'
AT5G40480.1     4607    4627
nuclear pore protein -like

sRNA_AGO1_Solexa_Mi2008_1_37419_hit1
5' UGAUGGUGUUCGUGGUAGGUUC
   ||||||||||| ||| ||:|||
   ACUACCACAAGAACC-UCUAAG 5'
AT5G40480.1     5773    5793
nuclear pore protein -like
```

```
sRNA_AGO1_Solexa_Mi2008_2_37420_hit1
5' UGAUGGUGUUGUUGGUGACUUC
   |||||||||| |||| || |||
   ACUACCACAAGAACCUCU-AAG 5'
AT5G40480.1     5773    5793
nuclear pore protein -like

sRNA_AGO1_Solexa_Mi2008_5_4358_hit1
5' CAACAGAAGAAUUUCAA-ACAG
   |||||||||| ||| || ||||
   GUUGUCUUCUCAAA-UUGUGUC 5'
AT5G40480.1     983     1003
nuclear pore protein -like

sRNA_AGO1_Solexa_Mi2008_1_3_hit25
5' AAAAAAAAAAAAAGAAAGA
   ||||||||||||||| |||
   UUUUUUUUUUUUUCUAUCU 5'
AT5G41400.1     644     662
RING zinc finger protein-like

sRNA_AGO1_Solexa_Mi2008_1_3_hit25
5' AAAAAAAAAAAAAGAAAGA
   ||||||||||||| |||||
   UUUUUUUUUUUUU-UUUCU 5'
AT5G41400.1     649     666
RING zinc finger protein-like

sRNA_AGO1_Solexa_Mi2008_1_1776_hit10
5' AGAA-AAAAAAAAAAAAAAAAU
   | || |||||||||||||||
   UAUUGUUUUUUUUUUUUUUUUC 5'
AT5G41400.1     650     671
RING zinc finger protein-like

sRNA_AGO1_Solexa_Mi2008_23_26355_hit1
5' UAU-UG-GAUCCGCUGUGCUGAG
   ||| || |||||||||| |||||
   AUAGACUCUAGGCGACAAGACUC 5'
AT5G41790.1     1866    1888
myosin heavy chain-like protein

sRNA_AGO1_Solexa_Mi2008_1_25234_hit1
5' UAUGAACUUGAUGACCCGCCC
   |||||||||||||||||||||
   AUACUUGAACUACUGGGCGGG 5'
AT5G42650.1     1051    1071
allene oxide synthase (emb|CAA73184.1)

sRNA_AGO1_Solexa_Mi2008_1_15261_hit5
5' GGGUCGAGUGAUGUGAUUGAG
   :|| |||||| ||||||||||
   UCC-GCUCACAACACUAACUC 5'
AT5G42650.1     4       23
allene oxide synthase (emb|CAA73184.1)

sRNA_AGO1_Solexa_Mi2008_2_7083_hit1
5' CAUCUCGC-CGGUUGACAUGAU
   ||||| || |||||||||||||
   GUAGA-CGUGCCAACUGUACUA 5'
AT5G45060.1     2644    2664
disease resistance protein-like
```

```
sRNA_AGO1_Solexa_Mi2008_1_8040_hit1
5' CCGGAGAAGCAGCUGA-UUGGU
   ||| |||||||||||| |||:|
   GGC-UCUUCGUCGACUCAACUA 5'
AT5G45060.1     2954    2974
disease resistance protein-like

sRNA_AGO1_Solexa_Mi2008_1_6472_hit1
5' CAGGAGG-AGAAAGAGUAGGUU
   ||||||| ||||||||||||
   GUCCUCCCUCUUUCUCAUCCUC 5'
AT5G49280.1     30      51
predicted GPI-anchored protein

sRNA_AGO1_Solexa_Mi2008_1_27863_hit1
5' UCACUAAU-GACAAUGAGUGUC
   ||| |||| ||||||||||||
   AGU-AUUAUCUGUUACUCACAA 5'
AT5G50160.1     1170    1190
FRO1 and FRO2-like protein

sRNA_AGO1_Solexa_Mi2008_1_53198_hit1
5' UUGGUUGCGAUGAUGAUGUUG
   ||| ||| |||||||||||||
   AAC-AAC-CUACUACUACAAC 5'
AT5G50160.1     296     314
FRO1 and FRO2-like protein

sRNA_AGO1_Solexa_Mi2008_1_21052_hit1
5' UAGACCAAACCUGUGAUAGU
   |||||||||||| || |||:
   AUCUGGUUUGGA-AC-AUCG 5'
AT5G50160.1     527     544
FRO1 and FRO2-like protein

sRNA_AGO1_Solexa_Mi2008_1_13614_hit8
5' GACAGAAGAGAGAGAGCAC
   || |||||||||||| ||
   CU-UCUUCUCUCUCU-GUA 5'
AT5G50160.1     556     572
FRO1 and FRO2-like protein

sRNA_AGO1_Solexa_Mi2008_2_8245_hit1
5' CGAAAAGAAGAAGAUGAUGAG
   |||||||||||||| || |||
   GCUUUUCUUCUUCU-CU-CUC 5'
AT5G50160.1     560     578
FRO1 and FRO2-like protein

sRNA_AGO1_Solexa_Mi2008_24_11800_hit1
5' CUGCUAGAGGUAACCAUUUGCU
   |||| ||| ||||||||||||
   GACGCUCUACAUUGGUAAACGC 5'
AT5G50400.1     1248    1269
putative protein

sRNA_AGO1_Solexa_Mi2008_2_5417_hit28
5' CACCAAGAAGAUGAGUCUC
   ||| |||||||||||||
   GUG-UUCUUCUACUCAGCU 5'
AT5G50400.1     498     515
putative protein
```

```
sRNA_AGO1_Solexa_Mi2008_6_3355_hit1
5' AUGGUAGUUCAAGUAUUCC
    :||||||||||| ||||
   CGCCAUCAAGUUCA-AAGG 5'
AT5G51750.1     2082    2099
serine protease-like protein

sRNA_AGO1_Solexa_Mi2008_2_4398_hit4
5' CAACAUUGGUGCUAGA-GAUGC
   |||||||||||||| | | |||
   GUUGUAACCACGAU-UACAACG 5'
AT5G51950.2     142     162
unknown protein

sRNA_AGO1_Solexa_Mi2008_2_4398_hit4
5' CAACAUUGGUGCUAGA-GAUGC
   |||||||||||||| | | |||
   GUUGUAACCACGAU-UACAACG 5'
AT5G51950.1     155     175
unknown protein

sRNA_AGO1_Solexa_Mi2008_1_45652_hit1
5' UGUGUCUAUGAGCAGCAUGGU
   ||||||||||| |||||:|||
   ACACAGAUACUGGUCGUGCCA 5'
AT5G51950.2     646     666
unknown protein

sRNA_AGO1_Solexa_Mi2008_1_45652_hit1
5' UGUGUCUAUGAGCAGCAUGGU
   ||||||||||| |||||:|||
   ACACAGAUACUGGUCGUGCCA 5'
AT5G51950.1     659     679
unknown protein

sRNA_AGO1_Solexa_Mi2008_1_42409_hit1
5' UGGGGGACAGUUUUGCAUGUA
   |||||||||||||||||||||
   ACCCCCUGUCAAAACGUACAU 5'
AT5G53420.3     188     208
unknown protein

sRNA_AGO1_Solexa_Mi2008_1_31134_hit1
5' UCGAUCAACGGUUGAGA-UGGU
    ||||||||||||:||| ||||
   UGCUAGUUGCCAAUUCUAACCA 5'
AT5G53420.2     194     215
unknown protein

sRNA_AGO1_Solexa_Mi2008_1_36778_hit1
5' UGAG-UCUUCGAAUUG-UGAACA
   |||| ||||||||||| |||| |
   ACUCCAGAAGCUUAACAACUUAU 5'
AT5G53420.3     212     234
unknown protein

sRNA_AGO1_Solexa_Mi2008_1_31134_hit1
5' UCGAUCAACGGUUGAGA-UGGU
    ||||||||||||:||| ||||
   UGCUAGUUGCCAAUUCUAACCA 5'
AT5G53420.3     356     377
unknown protein
```

```
sRNA_AGO1_Solexa_Mi2008_6_14059_hit1
5' GAG-GA-GAAUGUUGAGAAUG
   ||| || ||| ||||||||||
   CUCUCUUCUUUCAACUCUUAC 5'
AT5G53730.1     186     206
putative protein

sRNA_AGO1_Solexa_Mi2008_1_3901_hit3
5' CAAAACGAAGAAGAUGAUGAG
   | |||||| ||||||||||||
   GGUUUGCUCCUUCUACUACUC 5'
AT5G53730.1     214     234
putative protein

sRNA_AGO1_Solexa_Mi2008_1_37225_hit4
5' UGAUGAUGAUGAUGAUGAAGAAG
   ||| |||||||||||||||||||
   ACU-CUACUACUACUACUUCUUC 5'
AT5G53730.1     68      89
putative protein

sRNA_AGO1_Solexa_Mi2008_1_13759_hit1
5' GACGAUGAUGAUGAUGACA
   || ||||||||||||||
   CU-CUACUACUACUACUUC 5'
AT5G53730.1     71      88
putative protein

sRNA_AGO1_Solexa_Mi2008_5_13760_hit2
5' GACGAUGAUGAUGAUGAGC
   || ||||||||||||||:
   CU-CUACUACUACUACUUC 5'
AT5G53730.1     71      88
putative protein

sRNA_AGO1_Solexa_Mi2008_1_36323_hit13
5' UGAG-GAUGAUGAUGAUGAUGA
   |||| || ||||||||||||||
   ACUCUCU-CUACUACUACUACU 5'
AT5G53730.1     73      93
putative protein

sRNA_AGO1_Solexa_Mi2008_1_41417_hit1
5' UGGCGAGAGUGAUGAUGAUGU
   ||| ||||| ||||||||||
   ACCUCUCUCUCUACUACUACU 5'
AT5G53730.1     75      95
putative protein

sRNA_AGO1_Solexa_Mi2008_4_15683_hit7
5' GUCGAAAUCAGGAAAUCUCC
   || |||||||| |||||||
   CA-CUUUAGUCAUUUAGAGC 5'
AT5G57340.1     1197    1215
unknown protein

sRNA_AGO1_Solexa_Mi2008_1_26111_hit1
5' UAUGUUCUCUAAAUCGAUUGGA
   ||||||| ||||||| | ||||
   AUACAAGUGAUUUAG-UUACCU 5'
AT5G57340.2     455     475
unknown protein
```

```
sRNA_AGO1_Solexa_Mi2008_2_4149_hit1
5' CAAAGGCAGAAGAUGAUGACAG
   |||||||| ||||||||||| :
   GUUUCCGUAUUCUACUACUGAU 5'
AT5G59780.1     1022    1043
MYB27 protein - like

sRNA_AGO1_Solexa_Mi2008_1_29192_hit1
5' UCCAAAGGCAGAAGAUGAUGA
     |||||||| ||||||||||
   UUGUUUCCGUAUUCUACUACU 5'
AT5G59780.1     1025    1045
MYB27 protein - like

sRNA_AGO1_Solexa_Mi2008_1_42751_hit2
5' UGGGUGGUGA-UUGUGAGUGUAA
   |||||||||| || ||| |||||
   ACCCACCACUAAAAACU-ACAUU 5'
AT5G59780.3     865     886
MYB27 protein - like

sRNA_AGO1_Solexa_Mi2008_1_42751_hit2
5' UGGGUGGUGA-UUGUGAGUGUAA
   |||||||||| || ||| |||||
   ACCCACCACUAAAAACU-ACAUU 5'
AT5G59780.2     890     911
MYB27 protein - like

sRNA_AGO1_Solexa_Mi2008_2_4149_hit1
5' CAAAGGCAGAAGAUGAUGACAG
   |||||||| ||||||||||| :
   GUUUCCGUAUUCUACUACUGAU 5'
AT5G59780.3     904     925
MYB27 protein - like

sRNA_AGO1_Solexa_Mi2008_1_29192_hit1
5' UCCAAAGGCAGAAGAUGAUGA
     |||||||| ||||||||||
   UUGUUUCCGUAUUCUACUACU 5'
AT5G59780.3     907     927
MYB27 protein - like

sRNA_AGO1_Solexa_Mi2008_2_4149_hit1
5' CAAAGGCAGAAGAUGAUGACAG
   |||||||| ||||||||||| :
   GUUUCCGUAUUCUACUACUGAU 5'
AT5G59780.2     929     950
MYB27 protein - like

sRNA_AGO1_Solexa_Mi2008_1_29192_hit1
5' UCCAAAGGCAGAAGAUGAUGA
     |||||||| ||||||||||
   UUGUUUCCGUAUUCUACUACU 5'
AT5G59780.2     932     952
MYB27 protein - like

sRNA_AGO1_Solexa_Mi2008_1_42751_hit2
5' UGGGUGGUGA-UUGUGAGUGUAA
   |||||||||| || ||| |||||
   ACCCACCACUAAAAACU-ACAUU 5'
AT5G59780.1     983     1004
MYB27 protein - like
```

```
sRNA_AGO1_Solexa_Mi2008_1_27291_hit3
5' UCAAGGUUGGUGGACCUCUCA
   ||||||||||| |||||||||
   AGUUCCAACCAACUGGAGAGU 5'
AT5G60390.3     664     684
translation elongation factor eEF-1 alpha chain (gene A4)

sRNA_AGO1_Solexa_Mi2008_1_27291_hit3
5' UCAAGGUUGGUGGACCUCUCA
   ||||||||||| |||||||||
   AGUUCCAACCAACUGGAGAGU 5'
AT5G60390.2     674     694
translation elongation factor eEF-1 alpha chain (gene A4)

sRNA_AGO1_Solexa_Mi2008_7_12746_hit1
5' CUUGACCUUGUAAGACCCC
   ||||||||||:||||||
   GAACUGGAACGUUCUGGAA 5'
AT5G60450.1     1873    1891
auxin response factor 4

sRNA_AGO1_Solexa_Mi2008_7_49852_hit1
5' UUCUUGACCUUGUAAGACCCC
   ||||||||||||:||||||
   AAGAACUGGAACGUUCUGGAA 5'
AT5G60450.1     1873    1893
auxin response factor 4

sRNA_AGO1_Solexa_Mi2008_4_55012_hit1
5' UUUCUUGACCUUGUAAGACCCC
   |||||||||||||:||||||
   AAAGAACUGGAACGUUCUGGAA 5'
AT5G60450.1     1873    1894
auxin response factor 4

sRNA_AGO1_Solexa_Mi2008_1_55011_hit1
5' UUUCUUGACCUUGUAAGACCC
   |||||||||||||:||||||
   AAAGAACUGGAACGUUCUGGA 5'
AT5G60450.1     1874    1894
auxin response factor 4

sRNA_AGO1_Solexa_Mi2008_19_29991_hit1
5' UCCGGCGGUUCAUAACAUCAA
   |||||||||||||||||||||
   AGGCCGCCAAGUAUUGUAGUU 5'
AT5G60450.1     1925    1945
auxin response factor 4

sRNA_AGO1_Solexa_Mi2008_2_24987_hit1
5' UAUCCGGCGGUUCAUAACAUC
   |||||||||||||||||||||
   AUAGGCCGCCAAGUAUUGUAG 5'
AT5G60450.1     1927    1947
auxin response factor 4

sRNA_AGO1_Solexa_Mi2008_8_4564_hit1
5' CAAGAACUGGAUUUGCAUGAGA
   ||||||||||||||||||||||
   GUUCUUGACCUAAACGUACUCU 5'
AT5G60450.1     1966    1987
auxin response factor 4
```

```
sRNA_AGO1_Solexa_Mi2008_7_4563_hit1
5' CAAGAACUGGAUUUGCAUGAG
   |||||||||||||||||||||
   GUUCUUGACCUAAACGUACUC 5'
AT5G60450.1     1967    1987
auxin response factor 4

sRNA_AGO1_Solexa_Mi2008_1_34330_hit1
5' UGAACAAGCUGGGUUCACGCC
   |||||||||||||||||||||
   ACUUGUUCGACCCAAGUGCGG 5'
AT5G60450.1     2035    2055
auxin response factor 4

sRNA_AGO1_Solexa_Mi2008_7_33918_hit1
5' UCUUGACCUUGUAAGACCCCA
   |||||||||||:|||||||
   AGAACUGGAACGUUCUGGGAA 5'
AT5G60450.1     2082    2102
auxin response factor 4

sRNA_AGO1_Solexa_Mi2008_7_49853_hit1
5' UUCUUGACCUUGUAAGACCCCA
   ||||||||||||:|||||||
   AAGAACUGGAACGUUCUGGGAA 5'
AT5G60450.1     2082    2103
auxin response factor 4

sRNA_AGO1_Solexa_Mi2008_7_12746_hit1
5' CUUGACCUUGUAAGACCCC
   ||||||||||:|||||||
   GAACUGGAACGUUCUGGGA 5'
AT5G60450.1     2083    2101
auxin response factor 4

sRNA_AGO1_Solexa_Mi2008_7_49852_hit1
5' UUCUUGACCUUGUAAGACCCC
   ||||||||||||:|||||||
   AAGAACUGGAACGUUCUGGGA 5'
AT5G60450.1     2083    2103
auxin response factor 4

sRNA_AGO1_Solexa_Mi2008_4_55012_hit1
5' UUUCUUGACCUUGUAAGACCCC
   |||||||||||||:|||||||
   AAAGAACUGGAACGUUCUGGGA 5'
AT5G60450.1     2083    2104
auxin response factor 4

sRNA_AGO1_Solexa_Mi2008_1_55011_hit1
5' UUUCUUGACCUUGUAAGACCC
   |||||||||||||:|||||||
   AAAGAACUGGAACGUUCUGGG 5'
AT5G60450.1     2084    2104
auxin response factor 4

sRNA_AGO1_Solexa_Mi2008_2_34797_hit1
5' UGAAGGGGGACCCGAGGAUUG
   |||||||||||||||||||||
   ACUUCCCCCUGGGCUCCUAAC 5'
AT5G60450.1     2266    2286
auxin response factor 4
```

```
sRNA_AGO1_Solexa_Mi2008_3_13421_hit1
5' GAAGAAGAAGAUGAUGUUGAU
   ||||||||||| |||| |||
   CUUCUUCUUCUUCUACCACUU 5'
AT5G60450.1     621     641
auxin response factor 4

sRNA_AGO1_Solexa_Mi2008_1_7242_hit1
5' CAUGGAGAAGAAGAAUGA-GGU
   ||||:|||||||||| || |||
   GUACUUCUUCUUCUU-CUACCA 5'
AT5G60450.1     625     645
auxin response factor 4

sRNA_AGO1_Solexa_Mi2008_1_25289_hit1
5' UAUGACGAUGAUGAUGACGGA
   :||||||||||||||  ||||
   GUACUGCUACUACUA-GGCCU 5'
AT5G60450.1     650     669
auxin response factor 4

sRNA_AGO1_Solexa_Mi2008_9_14254_hit8
5' GAUGAUGAUGAUGAUGAUCUU
   ||||||||:||||||||||:
   CUACUACUGCUACUACUAGGC 5'
AT5G60450.1     651     671
auxin response factor 4

sRNA_AGO1_Solexa_Mi2008_7_3078_hit1
5' AUGAUGAUGA-GAAUGAUGAU
    ||||||||| || |||||||
   AACUACUACUGCU-ACUACUA 5'
AT5G60450.1     655     674
auxin response factor 4

sRNA_AGO1_Solexa_Mi2008_1_45039_hit3
5' UGU-GAUGAUGAUGAUGAUGAUGA
   ||| || ||||||||:||||||||
   ACAUCUUCUACUACUGCUACUACU 5'
AT5G60450.1     656     679
auxin response factor 4

sRNA_AGO1_Solexa_Mi2008_1_10092_hit1
5' CUAGAAGAUGAUGACGAUUGA
   |||||||||||||||||| ||
   GAUCUUCUACUACUGCUA-CU 5'
AT5G60450.1     659     678
auxin response factor 4

sRNA_AGO1_Solexa_Mi2008_3_35894_hit1
5' UGAGAGCAGAGAAAGAGAGU
   |||||| ||||||||||||
   ACUCUC-UCUCUUUCUCUCC 5'
AT5G60450.1     67      85
auxin response factor 4

sRNA_AGO1_Solexa_Mi2008_2_16_hit1
5' AAAAAAA-GAGAGGGACGAA
   ||||||| |||||||| ||
   UUUUUUUACUCUCCCU-CUC 5'
AT5G61960.1     101     119
Mei2-like protein
```

```
sRNA_AGO1_Solexa_Mi2008_5_39770_hit1
5' UGGAAGGCAGAUGAUUCUACA
   |||||| |||||||||||| |
   ACCUUCGGUCUACUAAGAUCU 5'
AT5G61960.2     2070    2090
Mei2-like protein

sRNA_AGO1_Solexa_Mi2008_5_39770_hit1
5' UGGAAGGCAGAUGAUUCUACA
   |||||| |||||||||||| |
   ACCUUCGGUCUACUAAGAUCU 5'
AT5G61960.1     2547    2567
Mei2-like protein
```

```
sRNA_AGO1_Solexa_Mi2008_1_13735_hit2
5' GACGAAGGUAUGGAACUCU
    |||||||| ||| |||||
   GUGCUUCCAAACCCUGAGA 5'
AT1G02360.1     615     633
hypothetical protein

sRNA_AGO1_Solexa_Mi2008_1_307_hit1
5' AAACCGCCAUCUAUU-UAAUU
   |||||||||| |||| | |||
   UUUGGCGGUAAAUAAUA-UAA 5'
AT1G02360.1     717     736
hypothetical protein

sRNA_AGO1_Solexa_Mi2008_6_22295_hit8
5' UAGC-CU-UCAAGUGUUUUCUGAUU
    ||| || |||||||||||||||||
   CUCGAGAGAGUUCACAAAAGACUAA 5'
AT1G08430.1     40      64
hypothetical protein

sRNA_AGO1_Solexa_Mi2008_273_45756_hit1
5' UGUGUUCUCAGGUCACCCCUUU
   |||| |||||||  ||||||||
   ACACUAGAGUCCUUUGGGGAAA 5'
AT1G08830.1     115     136
superoxidase dismutase

sRNA_AGO1_Solexa_Mi2008_65276_45755_hit1
5' UGUGUUCUCAGGUCACCCCUU
   |||| |||||||  |||||||
   ACACUAGAGUCCUUUGGGGAA 5'
AT1G08830.1     116     136
superoxidase dismutase

sRNA_AGO1_Solexa_Mi2008_75_53715_hit1
5' UUGUGUUCUCAGGUCACCCCUU
   ||||| |||||||  |||||||
   AACACUAGAGUCCUUUGGGGAA 5'
AT1G08830.1     116     137
superoxidase dismutase

sRNA_AGO1_Solexa_Mi2008_8_56168_hit1
5' UUUGUGUUCUCAGGUCACCCCUU
   |||||| |||||||  |||||||
   AAACACUAGAGUCCUUUGGGGAA 5'
AT1G08830.1     116     138
superoxidase dismutase

sRNA_AGO1_Solexa_Mi2008_689_53714_hit1
5' UUGUGUUCUCAGGUCACCCCU
   ||||| |||||||  ||||||
   AACACUAGAGUCCUUUGGGGA 5'
AT1G08830.1     117     137
superoxidase dismutase

sRNA_AGO1_Solexa_Mi2008_19_56167_hit1
5' UUUGUGUUCUCAGGUCACCCCU
   |||||| |||||||  ||||||
   AAACACUAGAGUCCUUUGGGGA 5'
AT1G08830.1     117     138
superoxidase dismutase
```

```
sRNA_AGO1_Solexa_Mi2008_67_56166_hit1
5' UUUGUGUUCUCAGGUCACCCC
   |||||| |||||||  |||||
   AAACACUAGAGUCCUUUGGGG 5'
AT1G08830.1     118     138
superoxidase dismutase

sRNA_AGO1_Solexa_Mi2008_2_20015_hit3
5' UACGAAAGGAGAAGCGGUUGA
   |||:||||:||||:|||||||
   AUGUUUUCUUCUUUGCCAACU 5'
AT1G12110.1     676     696
nitrate/chlorate transporter CHL1

sRNA_AGO1_Solexa_Mi2008_1_1909_hit1
5' AGAGCA-AUCGUGAAGAUGGAU
   |||| | ||| |||||||||||
   UCUC-UGUAGAACUUCUACCUA 5'
AT1G12110.1     96      116
nitrate/chlorate transporter CHL1

sRNA_AGO1_Solexa_Mi2008_1_1059_hit1
5' AAU-GAAAGUAGAGAGAGCU
   ||| ||||||| |||||| |
   UUAACUUUCAUGUCUCUCAA 5'
AT1G12520.1     1109    1128
Cu/Zn superoxide dismutase copper chaperone like protein

sRNA_AGO1_Solexa_Mi2008_1_1059_hit1
5' AAU-GAAAGUAGAGAGAGCU
   ||| ||||||| |||||| |
   UUAACUUUCAUGUCUCUCAA 5'
AT1G12520.2     1178    1197
Cu/Zn superoxide dismutase copper chaperone like protein

sRNA_AGO1_Solexa_Mi2008_1_1059_hit1
5' AAU-GAAAGUAGAGAGAGCU
   ||| ||||||| |||||| |
   UUAACUUUCAUGUCUCUCAA 5'
AT1G12520.3     1194    1213
Cu/Zn superoxide dismutase copper chaperone like protein

sRNA_AGO1_Solexa_Mi2008_2_36207_hit1
5' UGAGCUUGAUGGUUAUAUGAA
   |||||||||| |::|||||||
   ACUCGAACUAACGGUAUACUU 5'
AT1G30700.1     92      112
putative reticuline oxidase-like protein

sRNA_AGO1_Solexa_Mi2008_13_50200_hit2
5' UUGAAUUGAAGUGCUUGAA
   ||||||| ||||||||| |
   AACUUAAGUUCACGAACGU 5'
AT1G52060.1     355     373
null

sRNA_AGO1_Solexa_Mi2008_2_13262_hit1
5' CUUUUAGUAGUCGAUGUU-AUA
    |||| |||||||||||| |||
   UAAAAGCAUCAGCUACAACUAU 5'
AT1G52060.1     639     660
null
```

```
sRNA_AGO1_Solexa_Mi2008_1_34289_hit2
5' UGAAAUCGAUGUUGU-AAGUCC
   :||||||||||| || ||||||
   GCUUUAGCUACAUCAUUUCAGG 5'
AT1G52400.2     1567    1588
beta-glucosidase, putative

sRNA_AGO1_Solexa_Mi2008_4_11921_hit1
5' CUGGAUCGAAGCAUUUCUC
    ||||||| || |||||||
   UACCUAGCAUCAUAAAGAG 5'
AT1G52790.1     447     465
putative oxidoreductase

sRNA_AGO1_Solexa_Mi2008_1_20703_hit1
5' UACUUGAUGAGUUGCAUGGUU
   ||||||| ||||| ||| |||
   AUGAACUUCUCAAGGUAACAA 5'
AT1G52790.1     780     800
putative oxidoreductase

sRNA_AGO1_Solexa_Mi2008_7_7022_hit1
5' CAUCCAUCUAAUUCAAGUGCU
   ||||||| |  ||||||||||
   GUAGGUAUAAGAAGUUCACGA 5'
AT1G52790.1     92      112
putative oxidoreductase

sRNA_AGO1_Solexa_Mi2008_1_49761_hit2
5' UUCUGAAAUUAAUGUGAAUUU
   || |||||||| || ||||||
   AA-ACUUUAAUAACUCUUAAA 5'
AT1G52820.1     1015    1034
oxidoreductase-like protein

sRNA_AGO1_Solexa_Mi2008_1_46059_hit1
5' UGUUGUGCUGAAAAUGAUGUU
   ||||||||||||| |||||||
   ACAACACGACUUUGACUACAA 5'
AT1G52820.1     402     422
oxidoreductase-like protein

sRNA_AGO1_Solexa_Mi2008_1_699_hit1
5' AACGCCGUGAUUGUU-UGGU
   |||| |||||||||| ||:|
   UUGCUGCACUAACAACACUA 5'
AT1G55020.1     340     359
unknown protein

sRNA_AGO1_Solexa_Mi2008_12_46414_hit1
5' UUAACGCCGUGAUUGUU-UGGU
   |||||| |||||||||| ||:|
   AAUUGCUGCACUAACAACACUA 5'
AT1G55020.1     340     361
unknown protein

sRNA_AGO1_Solexa_Mi2008_1_28726_hit1
5' UCAGUGUGGAAGCUAAGUCUC
   ||| |||||||| |||||||
   AGU-ACACCUUCUAUUCAGAA 5'
AT1G55020.1     577     596
unknown protein
```

```
sRNA_AGO1_Solexa_Mi2008_2_51_hit1
5' AAAAAAGAAGAGAAACAAAGA
    ||||||||||:||| |||||
   AUUUUUCUUCUUUUUCUUUCU 5'
AT1G66200.2     1100    1120
glutamine synthetase like protein

sRNA_AGO1_Solexa_Mi2008_1_3_hit25
5' AAAAAA-AA-AAAAAGAAAGA
   |||||| || |||||||||||
   UUUUUUCUUCUUUUUCUUUCU 5'
AT1G66200.2     1101    1121
glutamine synthetase like protein

sRNA_AGO1_Solexa_Mi2008_1_44416_hit1
5' UGUAUUAUUAGAACCAAUUA
   ||||||||||| ||||||
   ACAUAAUAAUCAUGGUUAUG 5'
AT1G66200.2     1192    1211
glutamine synthetase like protein

sRNA_AGO1_Solexa_Mi2008_2_51_hit1
5' AAAAAAGAAGAGAAACAAAGA
    ||||||||||:||| |||||
   AUUUUUCUUCUUUUUCUUUCU 5'
AT1G66200.1     1320    1340
glutamine synthetase like protein

sRNA_AGO1_Solexa_Mi2008_1_3_hit25
5' AAAAAA-AA-AAAAAGAAAGA
   |||||| || |||||||||||
   UUUUUUCUUCUUUUUCUUUCU 5'
AT1G66200.1     1321    1341
glutamine synthetase like protein

sRNA_AGO1_Solexa_Mi2008_1_44416_hit1
5' UGUAUUAUUAGAACCAAUUA
   ||||||||||| ||||||
   ACAUAAUAAUCAUGGUUAUG 5'
AT1G66200.1     1412    1431
glutamine synthetase like protein

sRNA_AGO1_Solexa_Mi2008_28_5799_hit1
5' CACUGAUGUUGGUUCC-GCU
   |||||||||||:|||| ||
   GUGACUACAACUAAGGUCGU 5'
AT1G66200.2     661     680
glutamine synthetase like protein

sRNA_AGO1_Solexa_Mi2008_6_4713_hit1
5' CAAGCAGAAACAAGUAGAGAU
   |||| ||||||||| | ||||
   GUUCUUCUUUGUUC-U-UCUA 5'
AT1G66200.2     95      113
glutamine synthetase like protein

sRNA_AGO1_Solexa_Mi2008_2_10380_hit1
5' CUAGUGGACCUUUGAUAUCUA
   |||||||||||||||||||||
   GAUCACCUGGAAACUAUAGAU 5'
AT1G77760.1     2356    2376
nitrate reductase 1 (NR1)
```

```
sRNA_AGO1_Solexa_Mi2008_1_39739_hit2
5' UGGAAGAU-GGAGUGAUGCCUC
   ||| |||| ||||||||||||
   ACCAUCUAUCCUCACUACGGAC 5'
AT1G77760.1     2465    2486
nitrate reductase 1 (NR1)

sRNA_AGO1_Solexa_Mi2008_93_9914_hit1
5' CUACCAGCCGUUGA-UCAUGCU
   ||| |||||||||| |||| ||
   GAUAGUCGGCAACUAAGUA-GA 5'
AT1G77760.1     384     404
nitrate reductase 1 (NR1)

sRNA_AGO1_Solexa_Mi2008_3_40895_hit1
5' UGGAUGAUGAGAGAGAGAU
    || ||||:||||||||||
   CCC-ACUAUUCUCUCUCUA 5'
AT1G77760.1     45      62
nitrate reductase 1 (NR1)

sRNA_AGO1_Solexa_Mi2008_5_10942_hit1
5' CUCCUUUGUAAAAAG-UGUC
   |||||||||||| || |||
   GAGGAAACAUUU-UCAACAC 5'
AT2G19500.1     774     792
cytokinin oxidase (CKX2)

sRNA_AGO1_Solexa_Mi2008_9_724_hit1
5' AACUCGUGUUGCUUGACUU
   ||| ||||||||||||||
   UUG-GCACAACGAACUGAU 5'
AT2G19500.1     920     937
cytokinin oxidase (CKX2)

sRNA_AGO1_Solexa_Mi2008_6_14198_hit2
5' GAUCAUUGCAAUUGUUGGU
    ||| ||||| ||||||||
   GUAGAAACGUAAACAACCA 5'
AT2G19970.1     399     417
putative pathogenesis-related protein

sRNA_AGO1_Solexa_Mi2008_2_3828_hit2
5' AUUUGUCUGGUUAAUUCCGUU
   |||||| |||||||||  |||
   UAAACACACCAAUUAA--CAA 5'
AT2G19990.1     70      88
pathogenesis-related protein (PR-1)

sRNA_AGO1_Solexa_Mi2008_1_52127_hit1
5' UUGGAGGACCAGGUGGAA-AUU
   |||| ||||| ||||||| |||
   AACCACCUGGACCACCUUCUAA 5'
AT2G24180.1     146     167
cytochrome P450  like protein

sRNA_AGO1_Solexa_Mi2008_1_14490_hit1
5' GCAUCAAUGAUAGUGUUGUAG
   ||||||||||||| | |||||
   CGUAGUUACUAUC-C-ACAUC 5'
AT2G26560.1     961     979
similar to latex allergen from Hevea brasiliensis
```

```
sRNA_AGO1_Solexa_Mi2008_1_806_hit1
5' AAGAGCAUCAAUGAUAG-UGUU
   || |||||||||||||| |||
   UU-UCGUAGUUACUAUCCACAU 5'
AT2G26560.1     962     982
similar to latex allergen from Hevea brasiliensis

sRNA_AGO1_Solexa_Mi2008_3_52435_hit2
5' UUGGCUGCCGUAUAUG-ACUG
   |||||||||| ||||| ||||
   AACCGACGGCUUAUACGUGAC 5'
AT2G26820.1     343     363
similar to avrRpt2-induced protein 1

sRNA_AGO1_Solexa_Mi2008_2_15799_hit1
5' GUCUGAAGGAUUAGAGGAAC
   || ||||||||| ||||||
   CA-ACUUCCUAACCUCCUUC 5'
AT2G28190.1     109     127
putative copper/zinc superoxide dismutase

sRNA_AGO1_Solexa_Mi2008_1_18078_hit67
5' UAAGCUCAUGGUCACGCACU
   |||||||||||:||| | ||
   AUUCGAGUACCGGUG-G-GA 5'
AT2G28190.1     619     636
putative copper/zinc superoxide dismutase

sRNA_AGO1_Solexa_Mi2008_11_14876_hit4
5' GGAGGUGGUGG-UGG-UGGUGGU
   ||||||||||| ||| ||||| |
   CCUCCACCACCAACCUACCACAA 5'
AT2G33770.1     108     130
ubiquitin-conjugating enzyme E2 -like protein

sRNA_AGO1_Solexa_Mi2008_24_502_hit1
5' AAAGGAGGUGGUGGUUG-AU
   | ||||||||||||||| ||
   UCUCCUCCACCACCAACCUA 5'
AT2G33770.1     114     133
ubiquitin-conjugating enzyme E2 -like protein

sRNA_AGO1_Solexa_Mi2008_1_28360_hit2
5' UCAGCGGCGGAUCC-CACAAUGU
   |||| |||||| || ||||||||
   AGUCACCGCCU-GGCGUGUUACA 5'
AT2G33770.1     1331    1352
ubiquitin-conjugating enzyme E2 -like protein

sRNA_AGO1_Solexa_Mi2008_2_10859_hit1
5' CUCCAUCUCUCUCUCUGCUU
   |||| |||||||||||| ||
   GAGGAAGAGAGAGAGAC-AA 5'
AT2G33770.1     175     193
ubiquitin-conjugating enzyme E2 -like protein

sRNA_AGO1_Solexa_Mi2008_4_38337_hit1
5' UGCCAAAGGAGAUUUGCCCUGU
   ||||||||:|||||||||||:
   ACGGUUUCUUCUAAACGGGAUU 5'
AT2G33770.1     605     626
ubiquitin-conjugating enzyme E2 -like protein
```

```
sRNA_AGO1_Solexa_Mi2008_2_7575_hit2
5' CCAAAGGAGAGUUGCCCUG
   ||||||:||| |||||||:
   GGUUUCUUCUAAACGGGAU 5'
AT2G33770.1     606     624
ubiquitin-conjugating enzyme E2 -like protein

sRNA_AGO1_Solexa_Mi2008_26_14499_hit2
5' GCCAAAGGAGAGUUGCCCUG
   |||||||:||| |||||||:
   CGGUUUCUUCUAAACGGGAU 5'
AT2G33770.1     606     625
ubiquitin-conjugating enzyme E2 -like protein

sRNA_AGO1_Solexa_Mi2008_1041_38329_hit2
5' UGCCAAAGGAGAGUUGCCCUG
   ||||||||:||| |||||||:
   ACGGUUUCUUCUAAACGGGAU 5'
AT2G33770.1     606     626
ubiquitin-conjugating enzyme E2 -like protein

sRNA_AGO1_Solexa_Mi2008_2_38332_hit1
5' UGCCAAAGGAGAUUUGCCCCG
   ||||||||:|||||||||| :
   ACGGUUUCUUCUAAACGGGAU 5'
AT2G33770.1     606     626
ubiquitin-conjugating enzyme E2 -like protein

sRNA_AGO1_Solexa_Mi2008_92_38333_hit1
5' UGCCAAAGGAGAUUUGCCCGG
   ||||||||:|||||||||| :
   ACGGUUUCUUCUAAACGGGAU 5'
AT2G33770.1     606     626
ubiquitin-conjugating enzyme E2 -like protein

sRNA_AGO1_Solexa_Mi2008_101_38336_hit1
5' UGCCAAAGGAGAUUUGCCCUG
   ||||||||:|||||||||||:
   ACGGUUUCUUCUAAACGGGAU 5'
AT2G33770.1     606     626
ubiquitin-conjugating enzyme E2 -like protein

sRNA_AGO1_Solexa_Mi2008_74_51466_hit1
5' UUGCCAAAGGAGAGUUGCCCUG
   |||||||||:||| |||||||:
   AACGGUUUCUUCUAAACGGGAU 5'
AT2G33770.1     606     627
ubiquitin-conjugating enzyme E2 -like protein

sRNA_AGO1_Solexa_Mi2008_7_38328_hit2
5' UGCCAAAGGAGAGUUGCCCU
   ||||||||:||| |||||||
   ACGGUUUCUUCUAAACGGGA 5'
AT2G33770.1     607     626
ubiquitin-conjugating enzyme E2 -like protein

sRNA_AGO1_Solexa_Mi2008_2_38335_hit1
5' UGCCAAAGGAGAUUUGCCCU
   ||||||||:|||||||||||
   ACGGUUUCUUCUAAACGGGA 5'
AT2G33770.1     607     626
ubiquitin-conjugating enzyme E2 -like protein
```

```
sRNA_AGO1_Solexa_Mi2008_1_51465_hit1
5' UUGCCAAAGGAGAGUUGCCCU
   |||||||||:||| |||||||
   AACGGUUUCUUCUAAACGGGA 5'
AT2G33770.1     607     627
ubiquitin-conjugating enzyme E2 -like protein

sRNA_AGO1_Solexa_Mi2008_3_38327_hit2
5' UGCCAAAGGAGAGUUGCCC
   ||||||||:||| ||||||
   ACGGUUUCUUCUAAACGGG 5'
AT2G33770.1     608     626
ubiquitin-conjugating enzyme E2 -like protein

sRNA_AGO1_Solexa_Mi2008_1_38331_hit4
5' UGCCAAAGGAGAUUUGCCC
   ||||||||:||||||||||
   ACGGUUUCUUCUAAACGGG 5'
AT2G33770.1     608     626
ubiquitin-conjugating enzyme E2 -like protein

sRNA_AGO1_Solexa_Mi2008_4_38337_hit1
5' UGCCAAAGGAGAUUUGCCCUGU
   ||||||||||||| ||||||:
   ACGGUUUCCUCUAUACGGGAUC 5'
AT2G33770.1     738     759
ubiquitin-conjugating enzyme E2 -like protein

sRNA_AGO1_Solexa_Mi2008_2_38332_hit1
5' UGCCAAAGGAGAUUUGCCCCG
   ||||||||||||| ||||| :
   ACGGUUUCCUCUAUACGGGAU 5'
AT2G33770.1     739     759
ubiquitin-conjugating enzyme E2 -like protein

sRNA_AGO1_Solexa_Mi2008_92_38333_hit1
5' UGCCAAAGGAGAUUUGCCCGG
   ||||||||||||| ||||| :
   ACGGUUUCCUCUAUACGGGAU 5'
AT2G33770.1     739     759
ubiquitin-conjugating enzyme E2 -like protein

sRNA_AGO1_Solexa_Mi2008_101_38336_hit1
5' UGCCAAAGGAGAUUUGCCCUG
   ||||||||||||| ||||||:
   ACGGUUUCCUCUAUACGGGAU 5'
AT2G33770.1     739     759
ubiquitin-conjugating enzyme E2 -like protein

sRNA_AGO1_Solexa_Mi2008_22_15117_hit2
5' GGCUACCACAUCCAAGGAAGG
   || || |||||| ||||||||
   CC-AUAGUGUAGCUUCCUUCC 5'
AT2G33770.1     73      92
ubiquitin-conjugating enzyme E2 -like protein

sRNA_AGO1_Solexa_Mi2008_2_7575_hit2
5' CCAAAGGAGAGU-UGCCCUG
   |||||||||| | ||||||:
   GGUUUCCUCU-AUACGGGAU 5'
AT2G33770.1     740     758
ubiquitin-conjugating enzyme E2 -like protein
```

```
sRNA_AGO1_Solexa_Mi2008_26_14499_hit2
5' GCCAAAGGAGAGU-UGCCCUG
   ||||||||||| | ||||||:
   CGGUUUCCUCU-AUACGGGAU 5'
AT2G33770.1     740     759
ubiquitin-conjugating enzyme E2 -like protein

sRNA_AGO1_Solexa_Mi2008_2_38335_hit1
5' UGCCAAAGGAGAUUUGCCCU
   ||||||||||||| ||||||
   ACGGUUUCCUCUAUACGGGA 5'
AT2G33770.1     740     759
ubiquitin-conjugating enzyme E2 -like protein

sRNA_AGO1_Solexa_Mi2008_1041_38329_hit2
5' UGCCAAAGGAGAGU-UGCCCUG
   |||||||||||| | ||||||:
   ACGGUUUCCUCU-AUACGGGAU 5'
AT2G33770.1     740     760
ubiquitin-conjugating enzyme E2 -like protein

sRNA_AGO1_Solexa_Mi2008_74_51466_hit1
5' UUGCCAAAGGAGAGU-UGCCCUG
   ||||||||||||| | ||||||:
   AACGGUUUCCUCU-AUACGGGAU 5'
AT2G33770.1     740     761
ubiquitin-conjugating enzyme E2 -like protein

sRNA_AGO1_Solexa_Mi2008_1_38331_hit4
5' UGCCAAAGGAGAUUUGCCC
   ||||||||||||| |||||
   ACGGUUUCCUCUAUACGGG 5'
AT2G33770.1     741     759
ubiquitin-conjugating enzyme E2 -like protein

sRNA_AGO1_Solexa_Mi2008_7_38328_hit2
5' UGCCAAAGGAGAGU-UGCCCU
   |||||||||||| | ||||||
   ACGGUUUCCUCU-AUACGGGA 5'
AT2G33770.1     741     760
ubiquitin-conjugating enzyme E2 -like protein

sRNA_AGO1_Solexa_Mi2008_1_51465_hit1
5' UUGCCAAAGGAGAGU-UGCCCU
   ||||||||||||| | ||||||
   AACGGUUUCCUCU-AUACGGGA 5'
AT2G33770.1     741     761
ubiquitin-conjugating enzyme E2 -like protein

sRNA_AGO1_Solexa_Mi2008_3_38327_hit2
5' UGCCAAAGGAGAGU-UGCCC
   |||||||||||| | |||||
   ACGGUUUCCUCU-AUACGGG 5'
AT2G33770.1     742     760
ubiquitin-conjugating enzyme E2 -like protein

sRNA_AGO1_Solexa_Mi2008_1_26738_hit1
5' UCAAAGGGGAACCCAAGAUGUG
   ||||||||||||||||||||||
   AGUUUCCCCUUGGGUUCUACAC 5'
AT2G33770.1     774     795
ubiquitin-conjugating enzyme E2 -like protein
```

```
sRNA_AGO1_Solexa_Mi2008_6_14501_hit1
5' GCCAAAGGAGAUUUGCCCGGU
   ||||||||||||||||||:::
   CGGUUUCCUCUAAACGGGUUG 5'
AT2G33770.1     827     847
ubiquitin-conjugating enzyme E2 -like protein

sRNA_AGO1_Solexa_Mi2008_4_38334_hit1
5' UGCCAAAGGAGAUUUGCCCGGU
   |||||||||||||||||||:::
   ACGGUUUCCUCUAAACGGGUUG 5'
AT2G33770.1     827     848
ubiquitin-conjugating enzyme E2 -like protein

sRNA_AGO1_Solexa_Mi2008_4_38337_hit1
5' UGCCAAAGGAGAUUUGCCCUGU
   ||||||||||||||||||| ::
   ACGGUUUCCUCUAAACGGGUUG 5'
AT2G33770.1     827     848
ubiquitin-conjugating enzyme E2 -like protein

sRNA_AGO1_Solexa_Mi2008_2_7575_hit2
5' CCAAAGGAGAGUUGCCCUG
   |||||||||| |||||| :
   GGUUUCCUCUAAACGGGUU 5'
AT2G33770.1     828     846
ubiquitin-conjugating enzyme E2 -like protein

sRNA_AGO1_Solexa_Mi2008_26_14499_hit2
5' GCCAAAGGAGAGUUGCCCUG
   ||||||||||| |||||| :
   CGGUUUCCUCUAAACGGGUU 5'
AT2G33770.1     828     847
ubiquitin-conjugating enzyme E2 -like protein

sRNA_AGO1_Solexa_Mi2008_1041_38329_hit2
5' UGCCAAAGGAGAGUUGCCCUG
   |||||||||||| |||||| :
   ACGGUUUCCUCUAAACGGGUU 5'
AT2G33770.1     828     848
ubiquitin-conjugating enzyme E2 -like protein

sRNA_AGO1_Solexa_Mi2008_2_38332_hit1
5' UGCCAAAGGAGAUUUGCCCCG
   ||||||||||||||||||| :
   ACGGUUUCCUCUAAACGGGUU 5'
AT2G33770.1     828     848
ubiquitin-conjugating enzyme E2 -like protein

sRNA_AGO1_Solexa_Mi2008_92_38333_hit1
5' UGCCAAAGGAGAUUUGCCCGG
   |||||||||||||||||||::
   ACGGUUUCCUCUAAACGGGUU 5'
AT2G33770.1     828     848
ubiquitin-conjugating enzyme E2 -like protein

sRNA_AGO1_Solexa_Mi2008_101_38336_hit1
5' UGCCAAAGGAGAUUUGCCCUG
   ||||||||||||||||||| :
   ACGGUUUCCUCUAAACGGGUU 5'
AT2G33770.1     828     848
ubiquitin-conjugating enzyme E2 -like protein
```

```
sRNA_AGO1_Solexa_Mi2008_7_38328_hit2
5' UGCCAAAGGAGAGUUGCCCU
   |||||||||||| ||||||
   ACGGUUUCCUCUAAACGGGU 5'
AT2G33770.1     829     848
ubiquitin-conjugating enzyme E2 -like protein

sRNA_AGO1_Solexa_Mi2008_2_38335_hit1
5' UGCCAAAGGAGAUUUGCCCU
   |||||||||||||||||||
   ACGGUUUCCUCUAAACGGGU 5'
AT2G33770.1     829     848
ubiquitin-conjugating enzyme E2 -like protein

sRNA_AGO1_Solexa_Mi2008_1_51465_hit1
5' UUGCCAAAGGAGAGUUGCCCU
    |||||||||||| ||||||
   UACGGUUUCCUCUAAACGGGU 5'
AT2G33770.1     829     849
ubiquitin-conjugating enzyme E2 -like protein

sRNA_AGO1_Solexa_Mi2008_3_38327_hit2
5' UGCCAAAGGAGAGUUGCCC
   |||||||||||| ||||||
   ACGGUUUCCUCUAAACGGG 5'
AT2G33770.1     830     848
ubiquitin-conjugating enzyme E2 -like protein

sRNA_AGO1_Solexa_Mi2008_1_38331_hit4
5' UGCCAAAGGAGAUUUGCCC
   |||||||||||||||||||
   ACGGUUUCCUCUAAACGGG 5'
AT2G33770.1     830     848
ubiquitin-conjugating enzyme E2 -like protein

sRNA_AGO1_Solexa_Mi2008_2_38335_hit1
5' UGCCAAAGGAGAUUUGCCCU
   ||||||||||||||||| |
   ACGGUUUCCUCUAAACGAGC 5'
AT2G33770.1     886     905
ubiquitin-conjugating enzyme E2 -like protein

sRNA_AGO1_Solexa_Mi2008_3_38327_hit2
5' UGCCAAAGGAGAGUUGCCC
   |||||||||||| |||| |
   ACGGUUUCCUCUAAACGAG 5'
AT2G33770.1     887     905
ubiquitin-conjugating enzyme E2 -like protein

sRNA_AGO1_Solexa_Mi2008_1_38331_hit4
5' UGCCAAAGGAGAUUUGCCC
   ||||||||||||||||| |
   ACGGUUUCCUCUAAACGAG 5'
AT2G33770.1     887     905
ubiquitin-conjugating enzyme E2 -like protein

sRNA_AGO1_Solexa_Mi2008_2_38335_hit1
5' UGCCAAAGGAGAUUUGCCCU
   ||||||||||||||||| ||
   ACGGUUUCCUCUAAACGAGA 5'
AT2G33770.1     943     962
ubiquitin-conjugating enzyme E2 -like protein
```

```
sRNA_AGO1_Solexa_Mi2008_3_38327_hit2
5' UGCCAAAGGAGAGUUGCCC
   |||||||||||| |||| |
   ACGGUUUCCUCUAAACGAG 5'
AT2G33770.1     944     962
ubiquitin-conjugating enzyme E2 -like protein

sRNA_AGO1_Solexa_Mi2008_1_38331_hit4
5' UGCCAAAGGAGAUUUGCCC
   ||||||||||||||||| |
   ACGGUUUCCUCUAAACGAG 5'
AT2G33770.1     944     962
ubiquitin-conjugating enzyme E2 -like protein

sRNA_AGO1_Solexa_Mi2008_14_21346_hit1
5' UAGA-GCU-UUGAAGAACGUUGGU
   |||| ||| ||||||||||:||||
   AUCUACGACAACUUCUUGCGACCA 5'
AT2G43610.1     319     342
endochitinase like protein

sRNA_AGO1_Solexa_Mi2008_1_56576_hit1
5' UUUUG-GUCGCCAUGUUUUU-CUU
   ||||| |||||||| ||||| |||
   AAAACACAGCGGUAAAAAAAGGAA 5'
AT2G43610.1     31      54
endochitinase like protein

sRNA_AGO1_Solexa_Mi2008_3_12867_hit1
5' CUUGCCUCUUGCUAUUUCCUCU
   ||| ||||| |||||||| |||
   GAA-GGAGACCGAUAAAGAAGA 5'
AT2G43610.1     505     525
endochitinase like protein

sRNA_AGO1_Solexa_Mi2008_1_3384_hit1
5' AUGGUGAAGAUGU-UUC-UAGU
   ||||||||||||| ||| || |
   UACCACUUCUACAGAAGCAUGA 5'
AT3G01420.1     291     312
feebly like protein

sRNA_AGO1_Solexa_Mi2008_3_13839_hit1
5' GACUCAGAGAUCAGGAAAA
   ||| |||||| |||||||
   CUGUGUCUCUUGUCCUUUG 5'
AT3G13620.1     439     457
unknown protein

sRNA_AGO1_Solexa_Mi2008_1_741_hit2
5' AAGAAA-AAAAAAGUAUUGAC
   || ||| |||||||||||||
   UU-UUUGUUUUUUCAUAACUU 5'
AT3G24503.1     1651    1670
aldehyde dehydrogenase

sRNA_AGO1_Solexa_Mi2008_1_336_hit2
5' AAAGAAA-AAAAAAGUAUUGA
    || ||| |||||||||||||
   CUU-UUUGUUUUUUCAUAACU 5'
AT3G24503.1     1652    1671
aldehyde dehydrogenase
```

```
sRNA_AGO1_Solexa_Mi2008_2_49752_hit2
5' UUCUCUACAAACUUUUCCCACA
   |||||||| |||||:| |||||
   AAGAGAUGAUUGAAGA-GGUGU 5'
AT3G24503.1     998     1018
aldehyde dehydrogenase

sRNA_AGO1_Solexa_Mi2008_1_15541_hit2
5' GUAGUUGUGAUACAUGGAA-GAC
   ||||||||||| ||||||| ||
   CAUCAACACUACGUACCUUUCUU 5'
AT3G25190.1     728     750
unknown protein

sRNA_AGO1_Solexa_Mi2008_3_23318_hit2
5' UAG-GUAAAAACAUCUGGUCUA
   ||| |||||| ||||||||||
   AUCACAUUUU-GUAGACCAGAG 5'
AT3G44300.1     961     981
nitrilase 2

sRNA_AGO1_Solexa_Mi2008_18_30815_hit1
5' UCGAGGGUGUUGAAUAUGUUUU
   ||| ||||||||||| | ||||
   AGCGCCCACAACUUAGA-AAAA 5'
AT3G47540.1     171     191
endochitinase-like protein

sRNA_AGO1_Solexa_Mi2008_3_18344_hit1
5' UAAG-GUCGAGGGUGUUGAAU
   |||| |||| |||||||||||
   AUUCUCAGCGCCCACAACUUA 5'
AT3G47540.1     177     197
endochitinase-like protein

sRNA_AGO1_Solexa_Mi2008_6_14033_hit2
5' GAGGAAACUCUGGUGGAAGC
   :|||||| | ||||||||||
   UUCCUUUAAAACCACCUUCG 5'
AT3G47540.1     18      37
endochitinase-like protein

sRNA_AGO1_Solexa_Mi2008_5_427_hit1
5' AAAGAUGUUGUC-GCUA-UGU
   |||||||||||| || | |||
   UUUCUACAACAGACGUUUACA 5'
AT3G54640.1     1167    1187
tryptophan synthase alpha chain

sRNA_AGO1_Solexa_Mi2008_1_53131_hit2
5' UUGGUGUUGGU-GGUGUAAUGA
   ||||||| ||| |||||| |||
   AACCACA-CCAACCACAUCACU 5'
AT3G54640.1     816     836
tryptophan synthase alpha chain

sRNA_AGO1_Solexa_Mi2008_1_2108_hit2
5' AGGAAACGUGGUGGUGGUCGGAA
   ||||||||||||||  |||||
   UCCUUUGCACCACCA--AGCCUC 5'
AT4G02850.1     232     252
unknown protein
```

```
sRNA_AGO1_Solexa_Mi2008_2_6203_hit1
5' CAGAUUCUCAGGAUGACAGCAC
   |||||||:||:||||| |||||
   GUCUAAGGGUUCUACUUUCGUG 5'
AT4G02850.1     613     634
unknown protein

sRNA_AGO1_Solexa_Mi2008_1_14022_hit2
5' GAGCUCUUUCUUGA--UUCUA
   ||||||||||||||  ||||
   CUCGAGAAAGAACUGAAAGAG 5'
AT4G12400.2     1176    1196
stress-induced protein sti1 -like protein

sRNA_AGO1_Solexa_Mi2008_1_14022_hit2
5' GAGCUCUUUCUUGA-UU-CUA
   |||||||||||||| || ||
   CUCGAGAAAGAACUGAAAGAG 5'
AT4G12400.1     1178    1198
stress-induced protein sti1 -like protein

sRNA_AGO1_Solexa_Mi2008_1_53208_hit1
5' UUGGUUGGUGAUAGGCUGAUAGCU
   ||||||||||| ||| |||| |||
   AACCAACCACUUUCCAACUAACGA 5'
AT4G12400.2     202     225
stress-induced protein sti1 -like protein

sRNA_AGO1_Solexa_Mi2008_1_53208_hit1
5' UUGGUUGGUGAUAGGCUGAUAGCU
   ||||||||||| ||| |||| |||
   AACCAACCACUUUCCAACUAACGA 5'
AT4G12400.1     204     227
stress-induced protein sti1 -like protein

sRNA_AGO1_Solexa_Mi2008_19_53207_hit1
5' UUGGUUGGUGAUAGGCUGAUA
   ||||||||||| ||| ||||
   AACCAACCACUUUCCAACUAA 5'
AT4G12400.2     205     225
stress-induced protein sti1 -like protein

sRNA_AGO1_Solexa_Mi2008_1_53206_hit1
5' UUGGUUGGUGAUAGGCUGAU
   ||||||||||| ||| ||||
   AACCAACCACUUUCCAACUA 5'
AT4G12400.2     206     225
stress-induced protein sti1 -like protein

sRNA_AGO1_Solexa_Mi2008_19_53207_hit1
5' UUGGUUGGUGAUAGGCUGAUA
   ||||||||||| ||| ||||
   AACCAACCACUUUCCAACUAA 5'
AT4G12400.1     207     227
stress-induced protein sti1 -like protein

sRNA_AGO1_Solexa_Mi2008_1_53206_hit1
5' UUGGUUGGUGAUAGGCUGAU
   ||||||||||| ||| ||||
   AACCAACCACUUUCCAACUA 5'
AT4G12400.1     208     227
stress-induced protein sti1 -like protein
```

```
sRNA_AGO1_Solexa_Mi2008_1_173_hit2
5' AAAAGAAGAAGAUAAAGCAU
   ||||||||||||  || |||
   UUUUCUUCUUCU-GUU-GUA 5'
AT4G13180.1     853     870
short-chain alcohol dehydrogenase like protein

sRNA_AGO1_Solexa_Mi2008_1_29609_hit1
5' UCCCAAAACAAUAUUGU-AGUA
   | ||||||||||||| | ||||
   AUGGUUUUGUUAUAA-AUUCAU 5'
AT4G13510.1     1604    1624
ammonium transport protein (AMT1)

sRNA_AGO1_Solexa_Mi2008_1_40276_hit1
5' UGGAGAGGAAUCACUCGAUUC
   ||||||||||||||| | |||
   ACCUCUCCUUAGUGA-CCAAG 5'
AT4G13770.1     1119    1138
cytochrome P450 monooxygenase (CYP83A1)

sRNA_AGO1_Solexa_Mi2008_5_4586_hit1
5' CAAGAAUGAGGGAUAAGG-GA
   |||| |:||||||||||| ||
   GUUCGUGCUCCCUAUUCCUCU 5'
AT4G13770.1     1133    1153
cytochrome P450 monooxygenase (CYP83A1)

sRNA_AGO1_Solexa_Mi2008_6_19507_hit1
5' UACAGUAAACUCAGAAGCGAA
   |||||||||||||||||||||
   AUGUCAUUUGAGUCUUCGCUU 5'
AT4G13770.1     869     889
cytochrome P450 monooxygenase (CYP83A1)

sRNA_AGO1_Solexa_Mi2008_2_50423_hit1
5' UUGACUACAAAAGAUGGAGCA
    ||||| ||| ||||||||||
   CACUGAGGUUGUCUACCUCGU 5'
AT4G34110.1     2112    2132
poly(A)-binding protein

sRNA_AGO1_Solexa_Mi2008_6_33555_hit1
5' UCUGCUCGGUGCUUUAGGGUC
   |||||||||||||||||||||
   AGACGAGCCACGAAAUCCCAG 5'
AT4G34710.2     2279    2299
arginine decarboxylase (spe2)

sRNA_AGO1_Solexa_Mi2008_6_33555_hit1
5' UCUGCUCGGUGCUUUAGGGUC
   |||||||||||||||||||||
   AGACGAGCCACGAAAUCCCAG 5'
AT4G34710.1     2414    2434
arginine decarboxylase (spe2)

sRNA_AGO1_Solexa_Mi2008_1_2984_hit1
5' AUGACAACAACCAACCACACGU
   ||| ||||||| |||||||||
   UAC-GUUGUUGUUUGGUGUGCU 5'
AT4G34710.2     2534    2554
arginine decarboxylase (spe2)
```

```
sRNA_AGO1_Solexa_Mi2008_1_2984_hit1
5' AUGACAACAACCAACCACACGU
   ||| ||||||| |||||||||
   UAC-GUUGUUGUUUGGUGUGCU 5'
AT4G34710.1     2669    2689
arginine decarboxylase (spe2)

sRNA_AGO1_Solexa_Mi2008_1_35545_hit1
5' UGACGGCAAACCUGUAGGUCC
   |||||||||||||||||||||
   ACUGCCGUUUGGACAUCCAGG 5'
AT4G34980.1     1667    1687
subtilisin proteinase - like

sRNA_AGO1_Solexa_Mi2008_6_13612_hit1
5' GACAGAAGAAAGAGAGCAC
   || ||||||||||||||
   CU-UCUUCUUUCUCUCGCU 5'
AT4G35000.1     136     153
L-ascorbate peroxidase

sRNA_AGO1_Solexa_Mi2008_6_35236_hit1
5' UGACAGAAGAAAGAGAGCAC
   ||| ||||||||||||||
   ACU-UCUUCUUUCUCUCGCU 5'
AT4G35000.1     136     154
L-ascorbate peroxidase

sRNA_AGO1_Solexa_Mi2008_22_5333_hit1
5' CACAGAGAUUGAGAACGAA
   |||||||||:|||| |||
   GUGUCUCUAGCUCUCGCUA 5'
AT4G35000.1     396     414
L-ascorbate peroxidase

sRNA_AGO1_Solexa_Mi2008_6_4720_hit1
5' CAAGCAUCACAGGAGUAAUA
   ||||||||||||||||||||
   GUUCGUAGUGUCCUCAUUAU 5'
AT4G35630.1     1006    1025
phosphoserine aminotransferase

sRNA_AGO1_Solexa_Mi2008_5_15466_hit1
5' GUAGCCACCCUGAUUGUUGUU
   |||||| || ||||||||||
   CAUCGGCGGUACUAACAACAC 5'
AT4G35630.1     136     156
phosphoserine aminotransferase

sRNA_AGO1_Solexa_Mi2008_23_6630_hit1
5' CAGGUG-GAAGACAAGAUCGA
   | |||| ||||||||||||
   GGCCACUCUUCUGUUCUAGCC 5'
AT4G37390.1     348     368
auxin-responsive GH3 homolog (CF4)

sRNA_AGO1_Solexa_Mi2008_2_35755_hit1
5' UGAGAAACACGGUCAUGGU
   |||||||| |||| ||||
   ACUCUUUGAGCCACUACCC 5'
AT4G37390.1     365     383
auxin-responsive GH3 homolog (CF4)
```

```
sRNA_AGO1_Solexa_Mi2008_1_55562_hit1
5' UUUGGAGCAAAAACGU-UUUA
   |||||||||||||| | ||||
   AAACCUCGUUUUUG-AGAAAU 5'
AT4G39940.1     1154    1173
adenosine-5'-phosphosulfate-kinase

sRNA_AGO1_Solexa_Mi2008_10_5525_hit1
5' CACCGGACGAUCUUCU-CGA
    ||| ||||||||||| |||
   AUGGACUGCUAGAAGAUGCU 5'
AT5G02780.2     107     126
putative protein

sRNA_AGO1_Solexa_Mi2008_1_28867_hit2
5' UCAUCAUCUAGCACACCCUUG
   || |||||| ||| |||||||
   AG-AGUAGAACGUCUGGGAAC 5'
AT5G02780.2     234     253
putative protein

sRNA_AGO1_Solexa_Mi2008_1_13977_hit25
5' GAGAUA-AGCCAUGGAAAGGAU
   |||||| ||||||||| |||||
   CUCUAUCUCGGUACCUCUCCUA 5'
AT5G02780.2     73      94
putative protein

sRNA_AGO1_Solexa_Mi2008_1_37628_hit1
5' UGCAAAAACGGAUAAU-CU-AGC
   |||||||||||||| | || |||
   ACGUUUUUGCCUAUAACGAUUCG 5'
AT5G13750.2     534     556
transporter-like protein

sRNA_AGO1_Solexa_Mi2008_1_52493_hit1
5' UUGGGAGAGAUAUGAUGUC
   ||||| | ||||||||||
   AACCCCCGCUAUACUACAC 5'
AT5G13750.3     67      85
transporter-like protein

sRNA_AGO1_Solexa_Mi2008_1_37628_hit1
5' UGCAAAAACGGAUAAU-CUA-GC
   |||||||||||||| | ||| ||
   ACGUUUUUGCCUAUAACGAUUCG 5'
AT5G13750.3     759     781
transporter-like protein

sRNA_AGO1_Solexa_Mi2008_1_40339_hit1
5' UGGAGAUGGAAGAUGAG-CC
   |||||||||||| |||| ||
   ACCUCUACCUUCCACUCUGG 5'
AT5G13930.1     876     895
chalcone synthase (naringenin-chalcone synthase) (testa 4 protein) (sp|P13114)

sRNA_AGO1_Solexa_Mi2008_1_146_hit4
5' AAAACAAAAGAGGAAGAAGCU
   ||||| |||||: ||||||||
   UUUUGGUUUCUUAUUCUUCGA 5'
AT5G14120.1     2132    2152
nodulin-like protein
```

```
sRNA_AGO1_Solexa_Mi2008_9_40544_hit1
5' UGGAGGCAGCUUGUGUGAUGGA
   ||||| |||||||||| |||||
   ACCUC-GUCGAACACAAUACCU 5'
AT5G19110.1     1178    1198
dermal glycoprotein - like

sRNA_AGO1_Solexa_Mi2008_4_40543_hit1
5' UGGAGGCAGCUUGUGUGAUGG
   ||||| |||||||||| ||||
   ACCUC-GUCGAACACAAUACC 5'
AT5G19110.1     1179    1198
dermal glycoprotein - like

sRNA_AGO1_Solexa_Mi2008_7_15186_hit1
5' GGGCGUUUGGUUUCUUGUAA
   :|| |||||||| |||||||
   UCC-CAAACCAACGAACAUU 5'
AT5G19110.1     303     321
dermal glycoprotein - like

sRNA_AGO1_Solexa_Mi2008_17_45019_hit1
5' UGU-GAUCACCGGUUUGAAGUA
   ||| ||| | |||||||||||
   ACAUCUA-UAGCCAAACUUCAU 5'
AT5G19110.1     800     820
dermal glycoprotein - like

sRNA_AGO1_Solexa_Mi2008_1_54615_hit4
5' UUUAUUG-GGUACACAAAUAA
   ||||:|| ||||||||||||
   AAAUGACACCAUGUGUUUAUA 5'
AT5G23010.1     1621    1641
2-isopropylmalate synthase-like; homocitrate synthase-like

sRNA_AGO1_Solexa_Mi2008_2_255_hit1
5' AAA-AUAGUGUGGGAA-UGUUA
   ||| |||||||||||| ||||
   UUUCUAUCACACCCUUAACAAA 5'
AT5G23010.1     5       26
2-isopropylmalate synthase-like; homocitrate synthase-like

sRNA_AGO1_Solexa_Mi2008_9_13465_hit1
5' GAAGAUGG-GUGGGAAUUGUUU
    |||||:| ||||||||||||
   AUUCUAUCACACCCUUAACAAA 5'
AT5G23010.1     5       26
2-isopropylmalate synthase-like; homocitrate synthase-like

sRNA_AGO1_Solexa_Mi2008_13_4545_hit1
5' CAAGAAAGAUGGUUGUCGUU
     |||||| |||||||||||
   ACUCUUUCAACCAACAGCAA 5'
AT5G39050.1     534     553
Anthocyanin acyltransferase - like protein

sRNA_AGO1_Solexa_Mi2008_1_13420_hit2
5' GAAGAAGAAGAAGACUCUU
   || ||||||||||||||||
   CUCCUUCUUCUUCUGAGAA 5'
AT5G39050.1     830     848
Anthocyanin acyltransferase - like protein
```

```
sRNA_AGO1_Solexa_Mi2008_1_4090_hit1
5' CA-AAGAGUAGACAAUCAUCAA
   || |||||||:||||||| |||
   GUAUUCUCAUUUGUUAGU-GUU 5'
AT5G39050.1     888     908
Anthocyanin acyltransferase - like protein

sRNA_AGO1_Solexa_Mi2008_6_13767_hit2
5' GA-CGCAGAUUUAGGGAUUCU
   || ||||||||:||||||| |
   CUAGCGUCUAAGUCCCUAAAA 5'
AT5G44380.1     1338    1358
berberine bridge enzyme-like protein

sRNA_AGO1_Solexa_Mi2008_13_6614_hit40
5' CAGGUCGAGUAACAGUAAA
   |||||||||||| | ||||
   GUCCAGCUCAUUAU-AUUU 5'
AT5G44380.1     474     491
berberine bridge enzyme-like protein

sRNA_AGO1_Solexa_Mi2008_1_8282_hit1
5' CGAAAUCAGAUCCAAUA-GCUU
    ||||| |||||||||| ||||
   ACUUUAAUCUAGGUUAUACGAA 5'
AT5G45070.1     1147    1168
unknown protein

sRNA_AGO1_Solexa_Mi2008_34_10628_hit1
5' CUCAAGAAAGCUGUGGGAAA
   |||||||||||| |:||||
   GAGUUCUUUCGA-AUCCUUC 5'
AT5G45070.1     153     171
unknown protein

sRNA_AGO1_Solexa_Mi2008_4_14764_hit1
5' GCUCAAGAAAGCUGUGGGAAA
   ||||||||||||| |:||||
   CGAGUUCUUUCGA-AUCCUUC 5'
AT5G45070.1     153     172
unknown protein

sRNA_AGO1_Solexa_Mi2008_1_14763_hit1
5' GCUCAAGAAAGCUGUGGGAA
   ||||||||||||| |:||||
   CGAGUUCUUUCGA-AUCCUU 5'
AT5G45070.1     154     172
unknown protein

sRNA_AGO1_Solexa_Mi2008_25_30803_hit10
5' UCGAGGGGGCGUAUUAGGGA
   |||||||||||||||||||
   AGCUCCCCCGCAUAAUCCC_ 5'
AT5G48000.1     -1      18
cytochrome P450-like protein

sRNA_AGO1_Solexa_Mi2008_25_29907_hit10
5' UCCGCACAUACUCAACCCGA
   ||||||||||||||||||||
   AGGCGUGUAUGAGUUGGGCU 5'
AT5G48000.1     100     119
cytochrome P450-like protein
```

```
sRNA_AGO1_Solexa_Mi2008_3_25861_hit1
5' UAUGGUAGCAGAGCCCGGUCC
   ||||||| |||||||||:|||
   AUACCAUAGUCUCGGGCUAGG 5'
AT5G48000.1     117     137
cytochrome P450-like protein

sRNA_AGO1_Solexa_Mi2008_1_53933_hit2
5' UUUAACAUGGUAUCAGAGC
   |:||| |||||||||||||
   AGAUUAUACCAUAGUCUCG 5'
AT5G48000.1     124     142
cytochrome P450-like protein

sRNA_AGO1_Solexa_Mi2008_2_20709_hit8
5' UACUUGGCCCGCCGAUCCAUG
   |||||| |||| |||||||||
   AUGAACGGGGCAGCUAGGUAC 5'
AT5G48000.1     59      79
cytochrome P450-like protein

sRNA_AGO1_Solexa_Mi2008_1_18254_hit1
5' UAAGGCUUUAGGUGGAUAGUAA
   ||||||||||||||||||||||
   AUUCCGAAAUCCACCUAUCAUU 5'
AT5G48430.1     116     137
dermal glycoprotein precursor, extracellular-like

sRNA_AGO1_Solexa_Mi2008_1_53515_hit1
5' UUGUCUUCUG-UUGUCACGAUA
   ||||||| || ||||||||||
   AACAGAA-ACUAACAGUGCUAG 5'
AT5G54370.1     666     686
root cap protein 2-like protein

sRNA_AGO1_Solexa_Mi2008_3_1877_hit4
5' AGAGAAGGAGAGUUGAGUUUC
   |||||||||:||||| ||||
   UCUCUUCCUUUCAAC-CAAAC 5'
AT5G54370.1     8       27
root cap protein 2-like protein

sRNA_AGO1_Solexa_Mi2008_2_13246_hit1
5' CUUUGUGGUGAUGUUUUUGUU
   ||||||| |||| ||||| ||
   GAAACACAACUA-AAAAA-AA 5'
AT5G57220.1     44      62
cytochrome P450

sRNA_AGO1_Solexa_Mi2008_7_12746_hit1
5' CUUGACCUUGUAAGACCCC
   ||||||||||:||||||
   GAACUGGAACGUUCUGGAA 5'
AT5G60450.1     1873    1891
auxin response factor 4

sRNA_AGO1_Solexa_Mi2008_7_49852_hit1
5' UUCUUGACCUUGUAAGACCCC
   ||||||||||||:||||||
   AAGAACUGGAACGUUCUGGAA 5'
AT5G60450.1     1873    1893
auxin response factor 4
```

```
sRNA_AGO1_Solexa_Mi2008_4_55012_hit1
5' UUUCUUGACCUUGUAAGACCCC
   |||||||||||||:||||||
   AAAGAACUGGAACGUUCUGGAA 5'
AT5G60450.1     1873    1894
auxin response factor 4

sRNA_AGO1_Solexa_Mi2008_1_55011_hit1
5' UUUCUUGACCUUGUAAGACCC
   |||||||||||||:||||||
   AAAGAACUGGAACGUUCUGGA 5'
AT5G60450.1     1874    1894
auxin response factor 4

sRNA_AGO1_Solexa_Mi2008_19_29991_hit1
5' UCCGGCGGUUCAUAACAUCAA
   |||||||||||||||||||||
   AGGCCGCCAAGUAUUGUAGUU 5'
AT5G60450.1     1925    1945
auxin response factor 4

sRNA_AGO1_Solexa_Mi2008_2_24987_hit1
5' UAUCCGGCGGUUCAUAACAUC
   |||||||||||||||||||||
   AUAGGCCGCCAAGUAUUGUAG 5'
AT5G60450.1     1927    1947
auxin response factor 4

sRNA_AGO1_Solexa_Mi2008_8_4564_hit1
5' CAAGAACUGGAUUUGCAUGAGA
   ||||||||||||||||||||||
   GUUCUUGACCUAAACGUACUCU 5'
AT5G60450.1     1966    1987
auxin response factor 4

sRNA_AGO1_Solexa_Mi2008_7_4563_hit1
5' CAAGAACUGGAUUUGCAUGAG
   |||||||||||||||||||||
   GUUCUUGACCUAAACGUACUC 5'
AT5G60450.1     1967    1987
auxin response factor 4

sRNA_AGO1_Solexa_Mi2008_1_34330_hit1
5' UGAACAAGCUGGGUUCACGCC
   |||||||||||||||||||||
   ACUUGUUCGACCCAAGUGCGG 5'
AT5G60450.1     2035    2055
auxin response factor 4

sRNA_AGO1_Solexa_Mi2008_7_33918_hit1
5' UCUUGACCUUGUAAGACCCCA
   |||||||||||:|||||||
   AGAACUGGAACGUUCUGGGAA 5'
AT5G60450.1     2082    2102
auxin response factor 4

sRNA_AGO1_Solexa_Mi2008_7_49853_hit1
5' UUCUUGACCUUGUAAGACCCCA
   ||||||||||||:|||||||
   AAGAACUGGAACGUUCUGGGAA 5'
AT5G60450.1     2082    2103
auxin response factor 4
```

```
sRNA_AGO1_Solexa_Mi2008_7_12746_hit1
5' CUUGACCUUGUAAGACCCC
   ||||||||||:||||||||
   GAACUGGAACGUUCUGGGA 5'
AT5G60450.1     2083    2101
auxin response factor 4

sRNA_AGO1_Solexa_Mi2008_7_49852_hit1
5' UUCUUGACCUUGUAAGACCCC
   ||||||||||||:||||||||
   AAGAACUGGAACGUUCUGGGA 5'
AT5G60450.1     2083    2103
auxin response factor 4

sRNA_AGO1_Solexa_Mi2008_4_55012_hit1
5' UUUCUUGACCUUGUAAGACCCC
   |||||||||||||:||||||||
   AAAGAACUGGAACGUUCUGGGA 5'
AT5G60450.1     2083    2104
auxin response factor 4

sRNA_AGO1_Solexa_Mi2008_1_55011_hit1
5' UUUCUUGACCUUGUAAGACCC
   |||||||||||||:|||||||
   AAAGAACUGGAACGUUCUGGG 5'
AT5G60450.1     2084    2104
auxin response factor 4

sRNA_AGO1_Solexa_Mi2008_2_34797_hit1
5' UGAAGGGGGACCCGAGGAUUG
   |||||||||||||||||||||
   ACUUCCCCCUGGGCUCCUAAC 5'
AT5G60450.1     2266    2286
auxin response factor 4

sRNA_AGO1_Solexa_Mi2008_3_13421_hit1
5' GAAGAAGAAGAUGAUGUUGAU
   ||||||||||| |||| |||
   CUUCUUCUUCUUCUACCACUU 5'
AT5G60450.1     621     641
auxin response factor 4

sRNA_AGO1_Solexa_Mi2008_1_7242_hit1
5' CAUGGAGAAGAAGAAUGA-GGU
   ||||:|||||||||| || |||
   GUACUUCUUCUUCUU-CUACCA 5'
AT5G60450.1     625     645
auxin response factor 4

sRNA_AGO1_Solexa_Mi2008_1_25289_hit1
5' UAUGACGAUGAUGAUGACGGA
   :||||||||||||||  ||||
   GUACUGCUACUACUA-GGCCU 5'
AT5G60450.1     650     669
auxin response factor 4

sRNA_AGO1_Solexa_Mi2008_9_14254_hit8
5' GAUGAUGAUGAUGAUGAUCUU
   ||||||||:||||||||||:
   CUACUACUGCUACUACUAGGC 5'
AT5G60450.1     651     671
auxin response factor 4
```

```
sRNA_AGO1_Solexa_Mi2008_7_3078_hit1
5' AUGAUGAUGA-GAAUGAUGAU
    ||||||||| || |||||||
   AACUACUACUGCU-ACUACUA 5'
AT5G60450.1     655     674
auxin response factor 4

sRNA_AGO1_Solexa_Mi2008_1_45039_hit3
5' UGU-GAUGAUGAUGAUGAUGAUGA
   ||| || ||||||||:||||||||
   ACAUCUUCUACUACUGCUACUACU 5'
AT5G60450.1     656     679
auxin response factor 4

sRNA_AGO1_Solexa_Mi2008_1_10092_hit1
5' CUAGAAGAUGAUGACGAUUGA
   |||||||||||||||||| ||
   GAUCUUCUACUACUGCUA-CU 5'
AT5G60450.1     659     678
auxin response factor 4

sRNA_AGO1_Solexa_Mi2008_3_35894_hit1
5' UGAGAGCAGAGAAAGAGAGU
   |||||| |||||||||||||
   ACUCUC-UCUCUUUCUCUCC 5'
AT5G60450.1     67      85
auxin response factor 4

sRNA_AGO1_Solexa_Mi2008_4_30551_hit1
5' UCGACCACCACAGCUCUUUGU
   ||||||||||| ||| ||||
   AGCUGGUGGUGGCGACAAACU 5'
AT5G64100.1     145     165
peroxidase ATP3a
```
