# Supplementary material for: Misregulation of AUXIN RESPONSE FACTOR 8 Underlies the Developmental Abnormalities Caused by Three Distinct Viral Silencing Suppressors in Arabidopsis
Source: PLoS Pathog. 2011 May 12;7(5):e1002035. doi: 10.1371/journal.ppat.1002035 (PMC3093370; doi:10.1371/journal.ppat.1002035)
Supplement: Text S2 — Limits and possible implements to the VSR microarray/AGO1-IP approach to silencing target identification in Arabidopsis. (DOC) [file ppat.1002035.s017.doc]

**Text S2*.*** **Possible refinements to the VSR microarray/AGO1-IP approach to small RNA target discovery in Arabidopsis**

A drawback of the method is its intrinsic reliance on sufficient VSR expression levels in the tissues of interest. This may partly explain its poor performances in roots (Figure S5), where the 35S promoter is reputed to be only weakly active in several cell layers. Constitutive VSR expression might also contribute to the overall modest variations in target transcript accumulation seen in transgenic plants (Figure 1A; Figure S3-6), as this presumably imposes strong selection against high VSR dosage during early embryogenesis; the comparable, modest effects seen in *dcl1-9* plants are, likewise, probably accounted for by the hypomorphic nature of this mutation, as null alleles are embryonic-lethal. Use of conditional, as opposed to constitutive, expression of VSRs is thus an anticipated refinement of the method that might lead to much more tractable effects on target gene accumulation. The scope of the method might also be further broadened by decreasing the stringency of the threshold (≥20) used for AGO1-IP read values (Figure S7); pairwise analysis of VSRs (as opposed to analyzing genes altered in common in the three VSR lines) might also be exploited. Additional filters may also be applied to the data set, including queries of natural antisense (Nat-si) RNA databases or ‘degradome’ libraries that document the slicing activity of endogenous small RNAs at a transcriptome-wide level.
