# Supplementary material for: Misregulation of AUXIN RESPONSE FACTOR 8 Underlies the Developmental Abnormalities Caused by Three Distinct Viral Silencing Suppressors in Arabidopsis
Source: PLoS Pathog. 2011 May 12;7(5):e1002035. doi: 10.1371/journal.ppat.1002035 (PMC3093370; doi:10.1371/journal.ppat.1002035)
Supplement: Text S3 — Details on statistical analysis of microarray data. (DOC) [file ppat.1002035.s018.doc]

**Text S3**. **Statistical microarray data analysis**

Statistic methods were developed under the software R (R Development Core Team, http://www.R-project.org) in collaboration with the group ‘Statistics and Genome’ at UMR AgroParisTech/INRA MIA 518 and are available in the R package ‘Anapuce’ on their web site (http://cran.r-project.org/web/packages/anapuce/index.html). For each CATMA array, the raw data include the logarithm of median feature pixel intensity at wavelengths 635nm (red) and 532nm (green), no background is subtracted. A normalization per array is performed to remove systematic biases. First, spots that are considered badly formed features are excluded. Then, a global intensity dependent normalization is performed using the lowess procedures [1] to correct the dye bias. Finally, for each block, the log-ratio median calculated over the values for the entire block is subtracted from each individual logratio value to correct effects on each block, as well as print-tip, washing and/or drying effects. At the end of the normalization step, a normalized log-ratio, which is equivalent to an expression difference (in log base 2) between the two samples co-hybridized on the same array, is given for each spot. It is equal to the raw log-ratio minus the lowess correction minus the block correction. A normalized logarithm intensity for each sample is also calculated. It is done according to the within-array correction proposed by Yang and Thorne [2], which is a redistribution of the correction calculated for the log2-ratio normalization on each channel. To determine differentially expressed genes from a dye-swap, a paired t-test is performed on the log2-ratios. Since the number of observations per spot equals two, it is inadequate for calculating a specific variance. For this reason, it is assumed that the variance of the log2-ratios is the same for all spots. This solution has the main advantage to calculate an estimator over a large number of data, leading to a robust estimation of the variance and to a gain in the power of the test. Nevertheless, this solution should be applied with some precautions since some spots display an extreme specific variance (too small or too large) and prevent that the assumption of common variance is verified. Indeed spots with a too small specific variance decrease wrongly the estimate of the common variance and hence it could lead to increase the number of false positives, and spot with a too large variance increase wrongly the estimate of the common variance and hence it could lead to decrease the test power. For the above reasons, spots with extreme specific variance are excluded from the statistical analysis. The spots that are excluded are those with a ‘specific variance/common variance’ ratio smaller than the ‘alphaquantile of a chi-squared distribution of one degree of liberty’ or greater than the ‘1-alpha-quantile of a chi-squared distribution of one degree of liberty’ with alpha equal to 0.0001. This rule stems from a direct application of Cochran’s theorem. The raw P-values are adjusted by the Bonferroni method, which controls the Family Wise Error Rate (FWER) [3]. When the Bonferroni P-value is lower than 0.05, the spot is declared differentially expressed. Spots with a missing P-value are spots with an extreme variance or genes for which one observation only is available. That is, when for one of the two arrays, the spot corresponding to the gene was a badly formed feature.

1. Yang YH, Dudoit S, Luu P, Lin DM, Peng V, Ngai J, Speed TP (2002). Normalization for cDNA microarray data: a robust composite method addressing single and multiple slide systematic variation. Nucleic Acids Res.30, e15.

2. Yang YH, Thorne NP; Goldstein DR, editor (2003). Normalization for two-color cDNA microarray data. Science and Statistics: A Festschrift for Terry Speed, IMS Lecture Notes–Monograph Series 40, 403-418.

3. Ge Y, Dudoit S, Speed TP (2003). Resampling-based multiple testing for microarray data analysis. TEST 12,1-44.
