# Supplementary figures and images for: Correction: Misregulation of AUXIN RESPONSE FACTOR 8 Underlies the Developmental Abnormalities Caused by Three Distinct Viral Silencing Suppressors in Arabidopsis
Source: PLoS Pathog. 2016 May 5;12(5):e1005627. doi: 10.1371/journal.ppat.1005627 (PMC4858414; doi:10.1371/journal.ppat.1005627)

## Slide 1
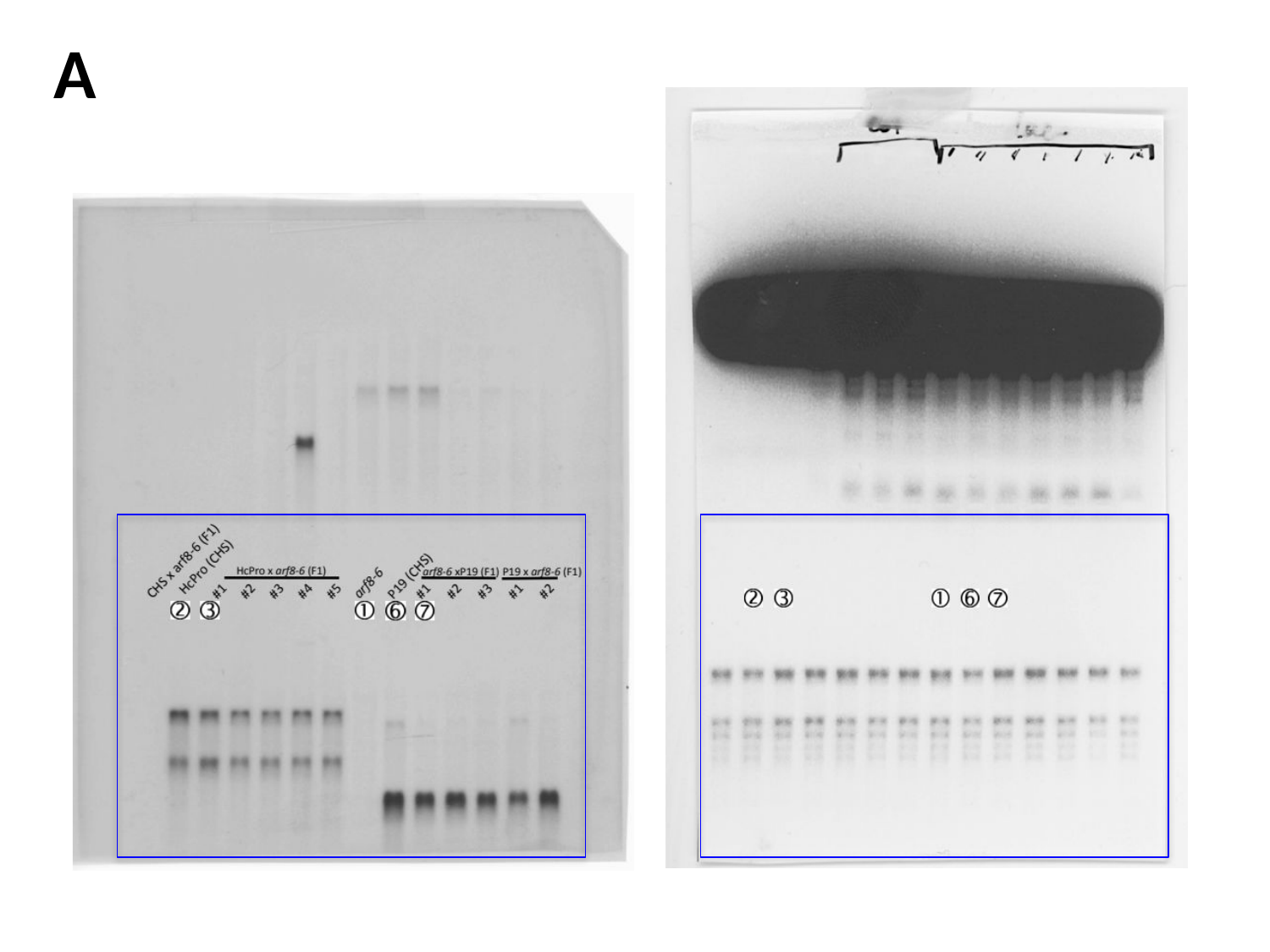

A

## Slide 2
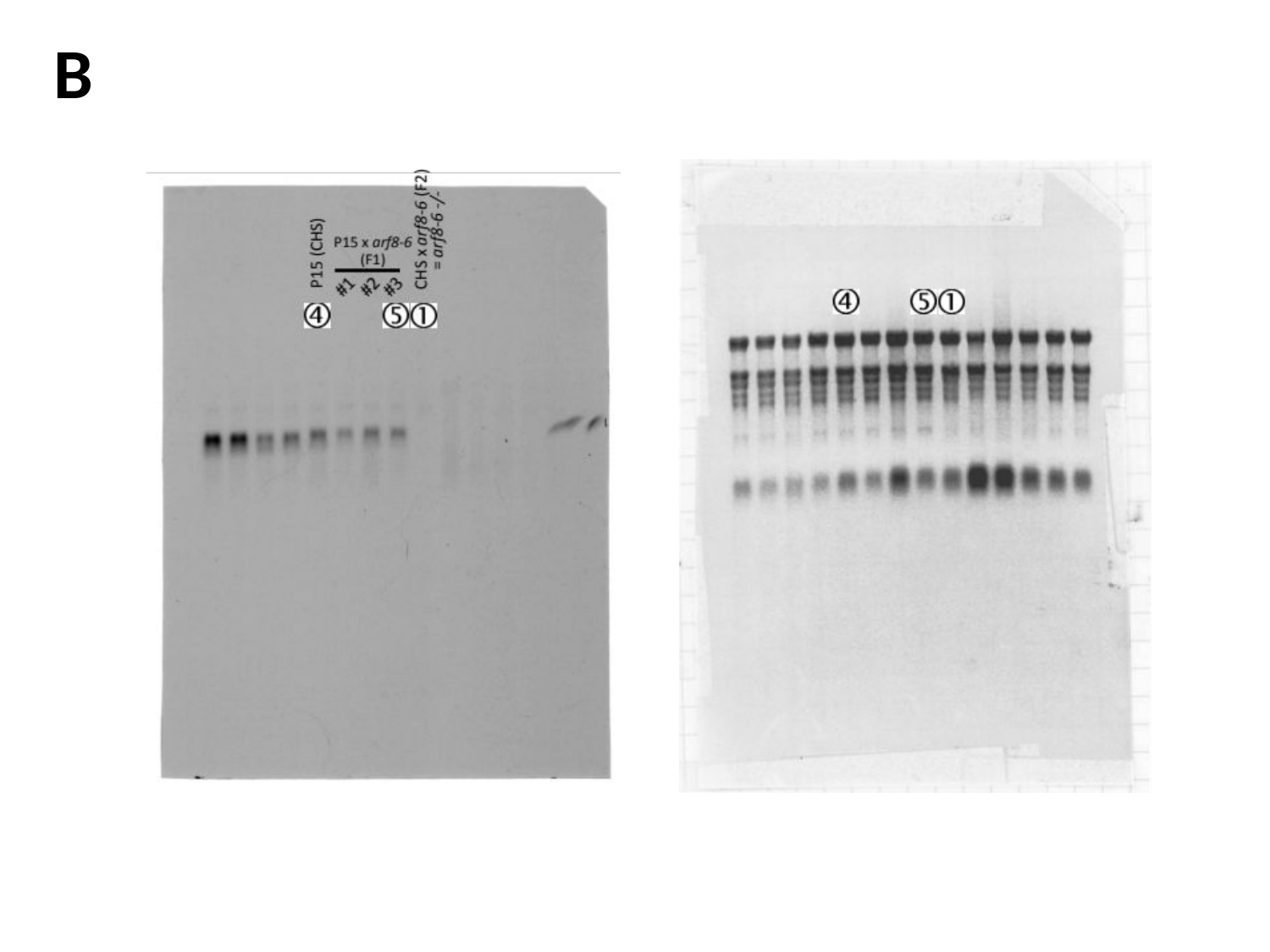

B

## Slide 3
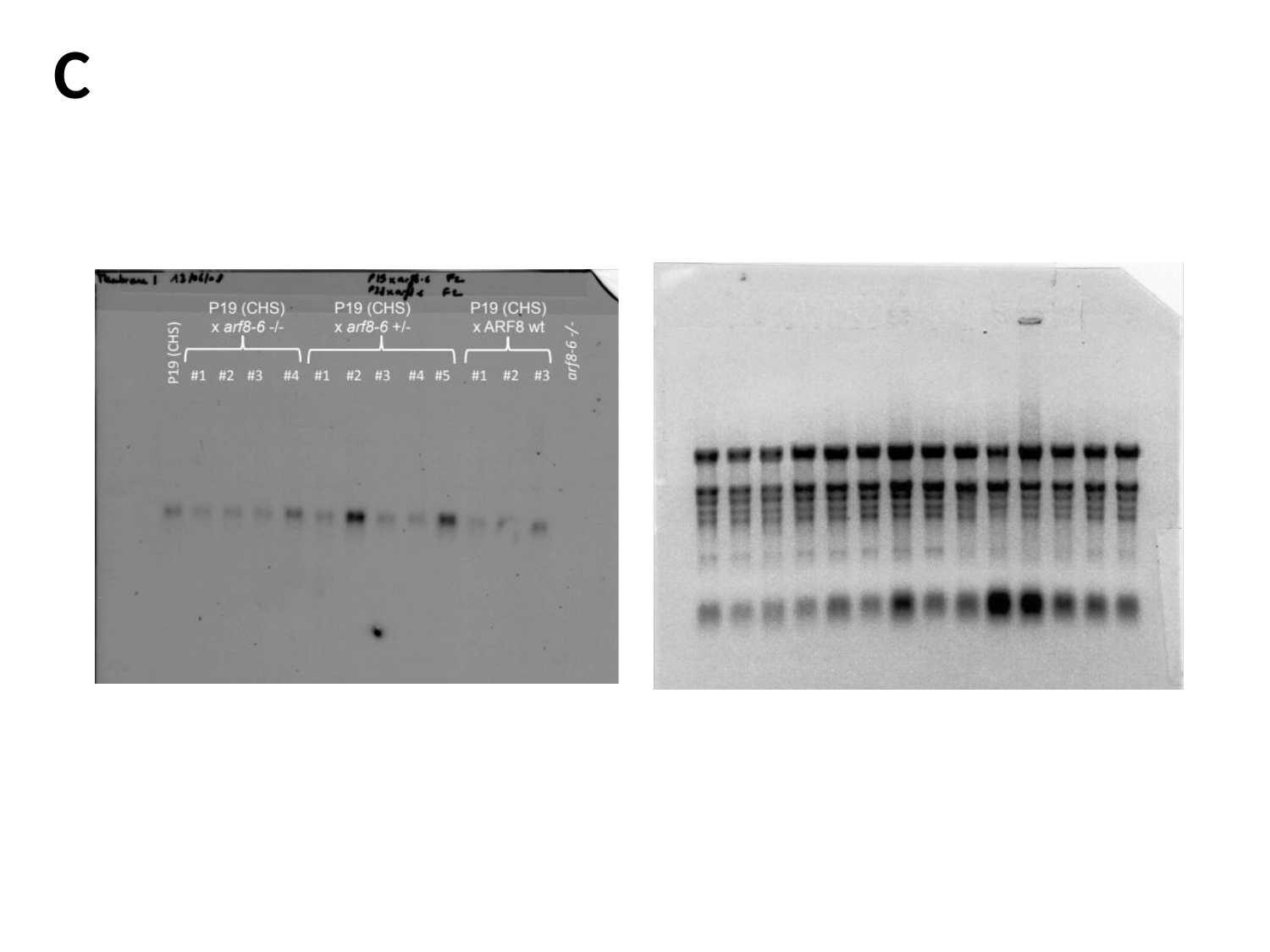

C

## Slide 4
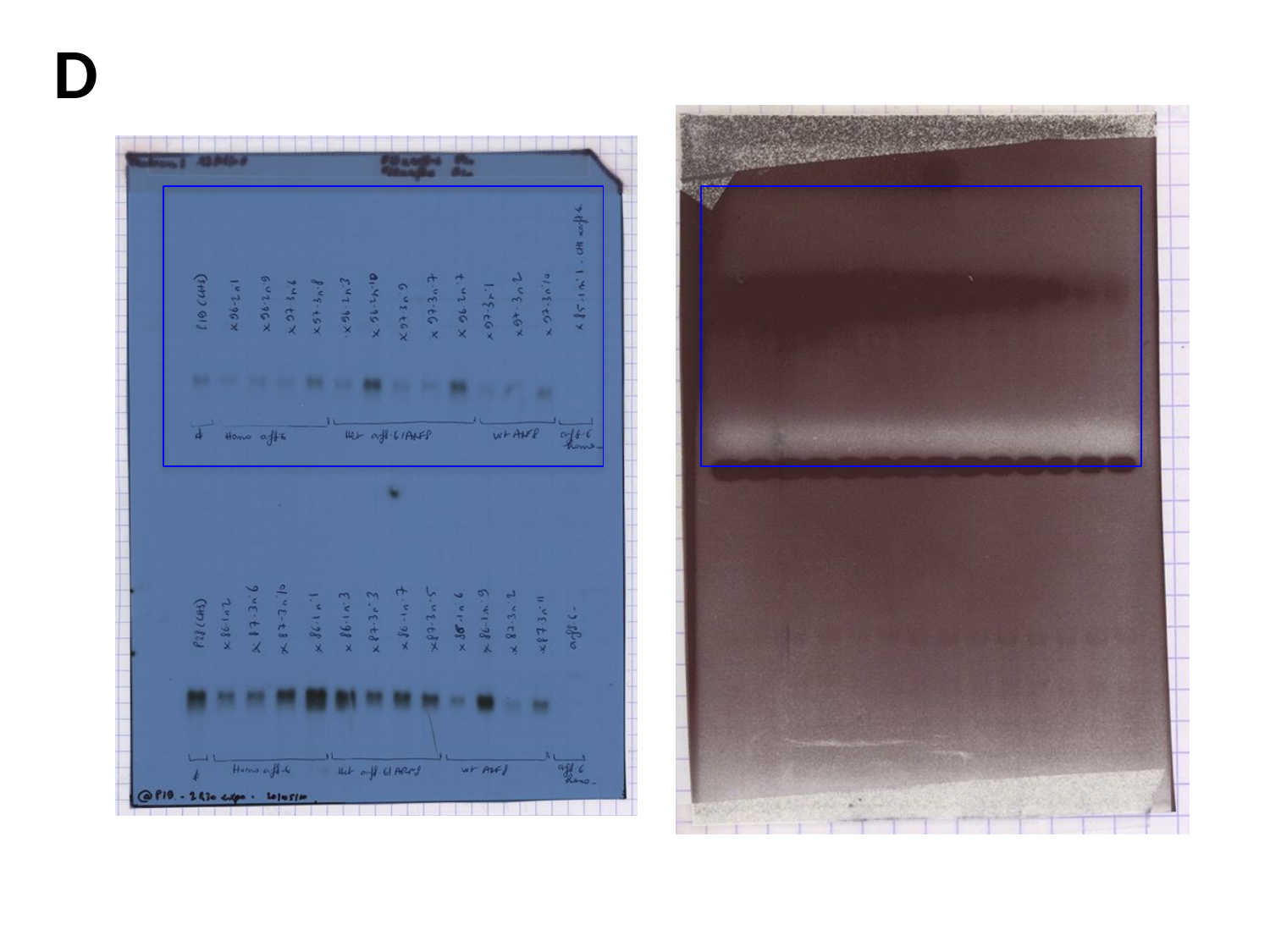

D

## Slide 5
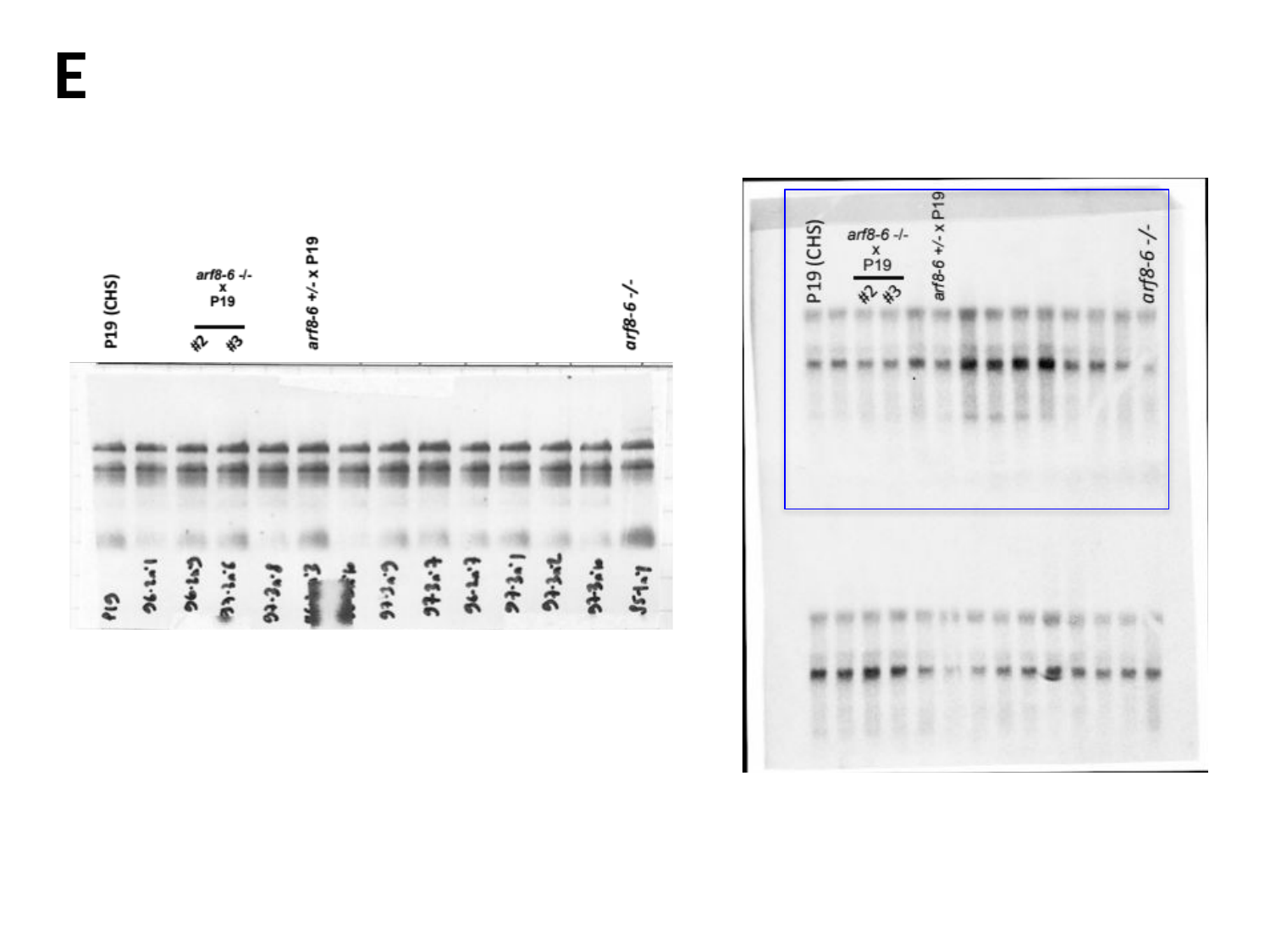

E

Supplement: S1 Fig — (A) Original film (left) and ethidium bromide staining (right) used for mounting Fig 4C and Fig 4G. Both figures were mounted from the same blot, hybridized with a mix of random-labeled PCR products corresponding to the 35S terminator and HcPro, allowing simultaneous detection of the P19 and HcPro transcripts (bottom lane), respectively. Blue rectangles indicate the part of the blot used for mounting these figures; samples were loaded according to the track labels; surrounded numbers correspond to the annotated samples on the original Fig 4B and 4F. (B) Original film (left) and ethidium bromide staining (right) used for mounting Fig 4E. The blot was hybridized with random-labeled PCR products corresponding to the 35S terminator, allowing detection of the P15 transcripts. Samples were loaded according to the track labels; surrounded numbers correspond to the annotated samples on the original Fig 4D. (C) Original scan (left) and inappropriate ethidium bromide staining (right) used for mounting Fig 4I. The blot was hybridized with random-labeled PCR products corresponding to the 35S terminator, allowing detection of the P19 transcripts. Samples were loaded according to the track labels. (D) Original film (left) and original ethidium bromide staining (right) corresponding to samples presented in Fig 4I. The blot was hybridized with random-labeled PCR products corresponding to the 35S terminator, allowing detection of the P19 transcripts. Blue rectangles indicate the part of the blot with the samples used in Fig 4I. (E) Left panel: original pre-loading control corresponding to the ethidium bromide staining of 1 μg of total RNA loaded on a 1% agarose gel to check quality and equal loading prior loading of the high molecular Northern blot. Right panel: original film of the original membrane used for mounting Fig 4I, re-probed with non-specific random-labeled PCR products that provides an independent loading control. Blue rectangle indicates the part of the blot with the sa [file ppat.1005627.s001.pptx]

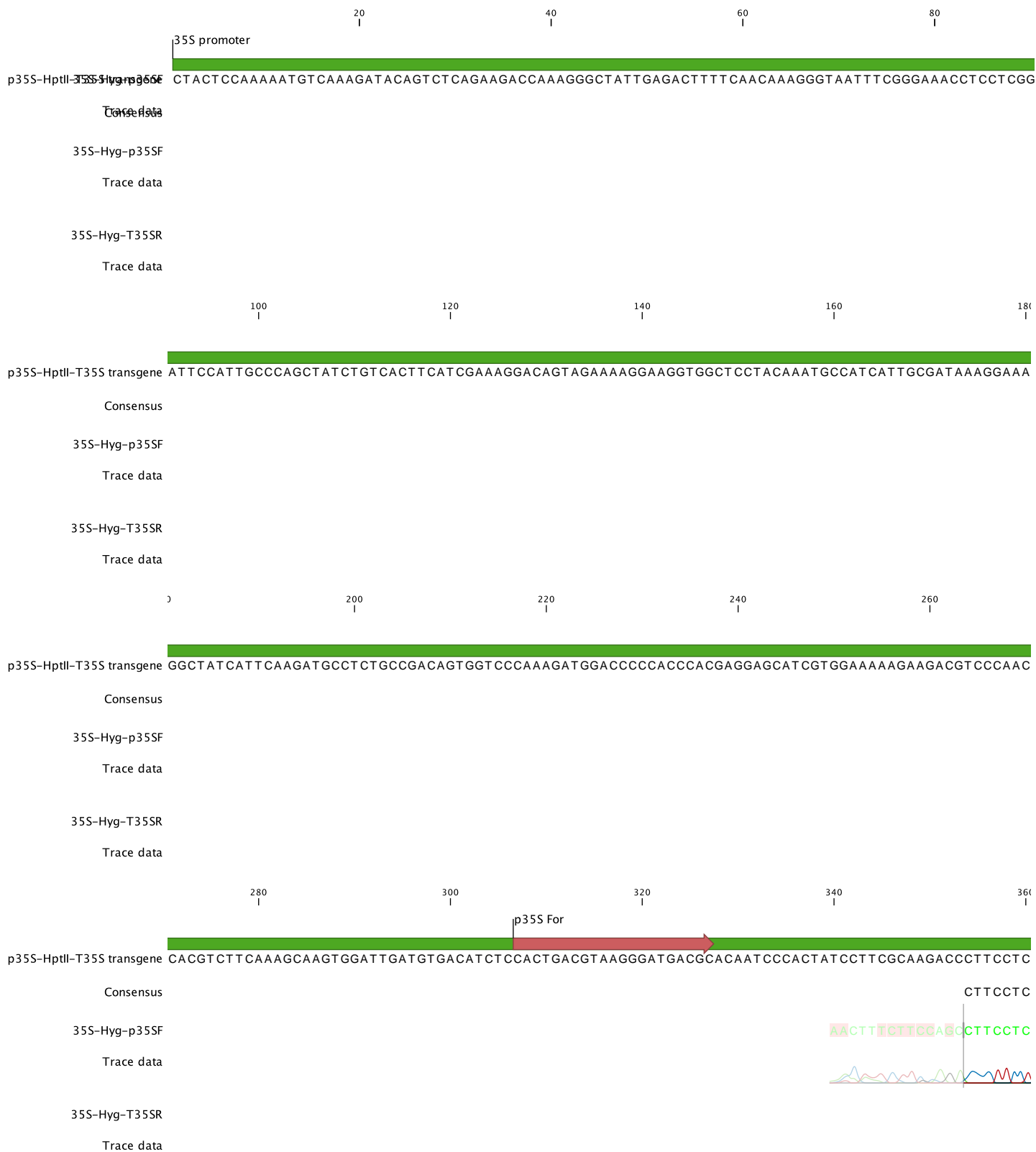

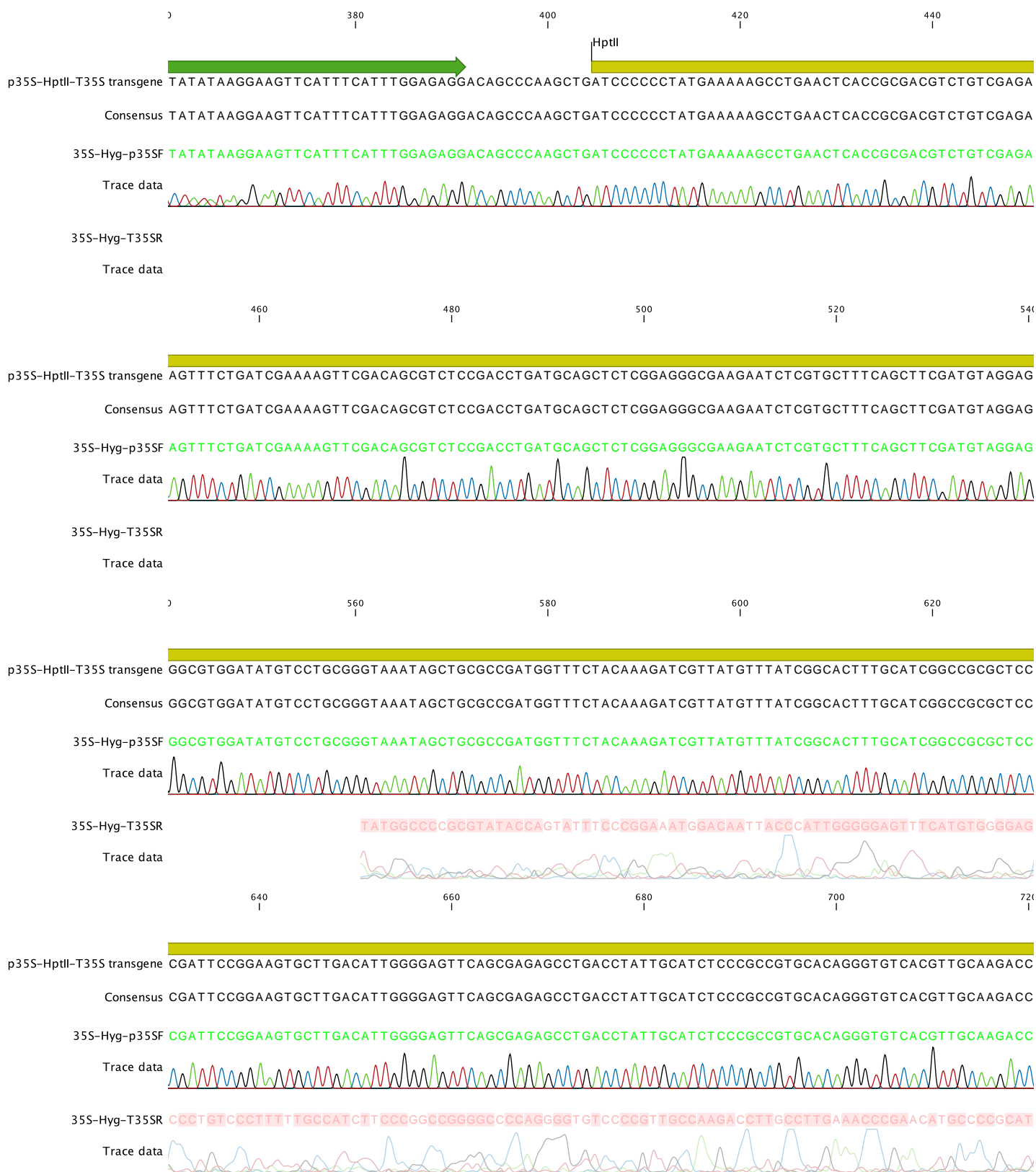

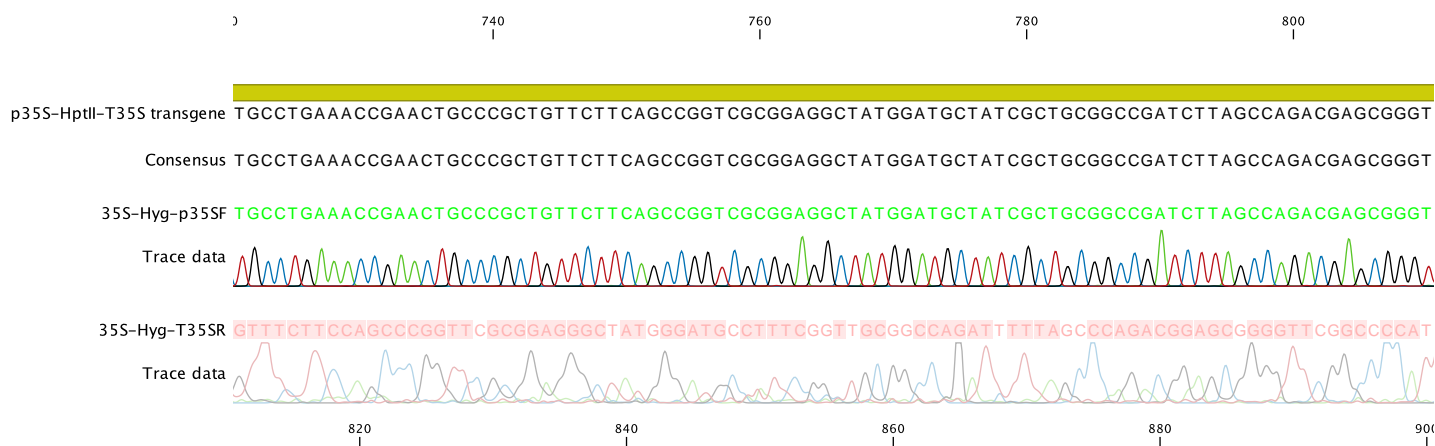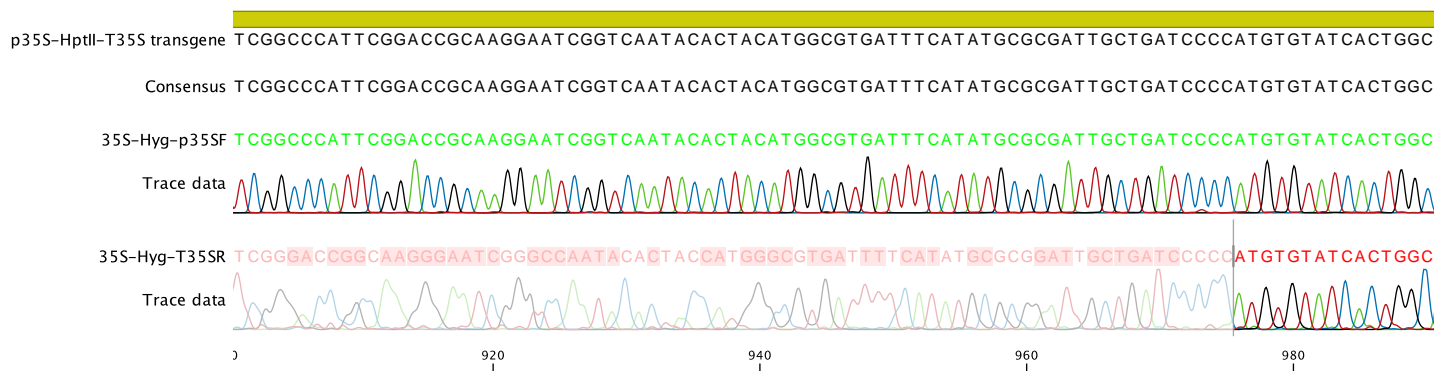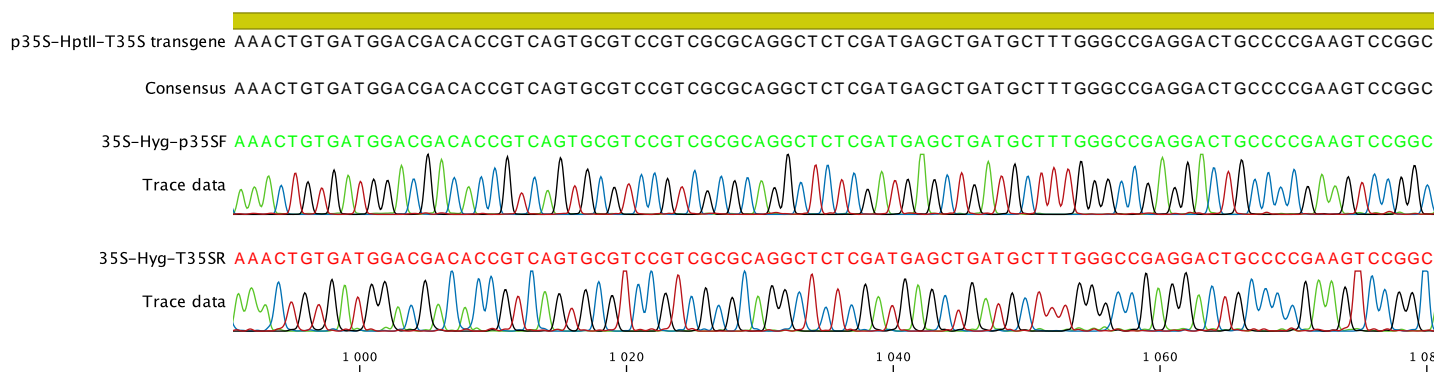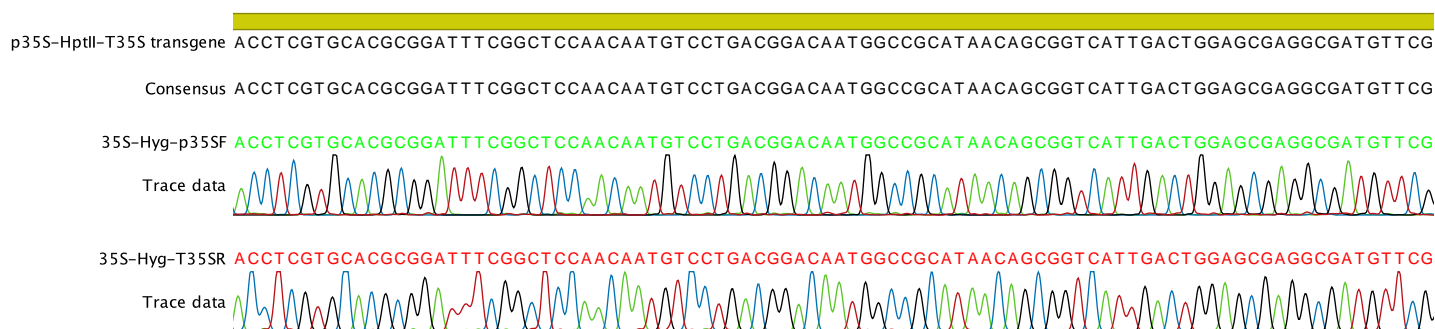

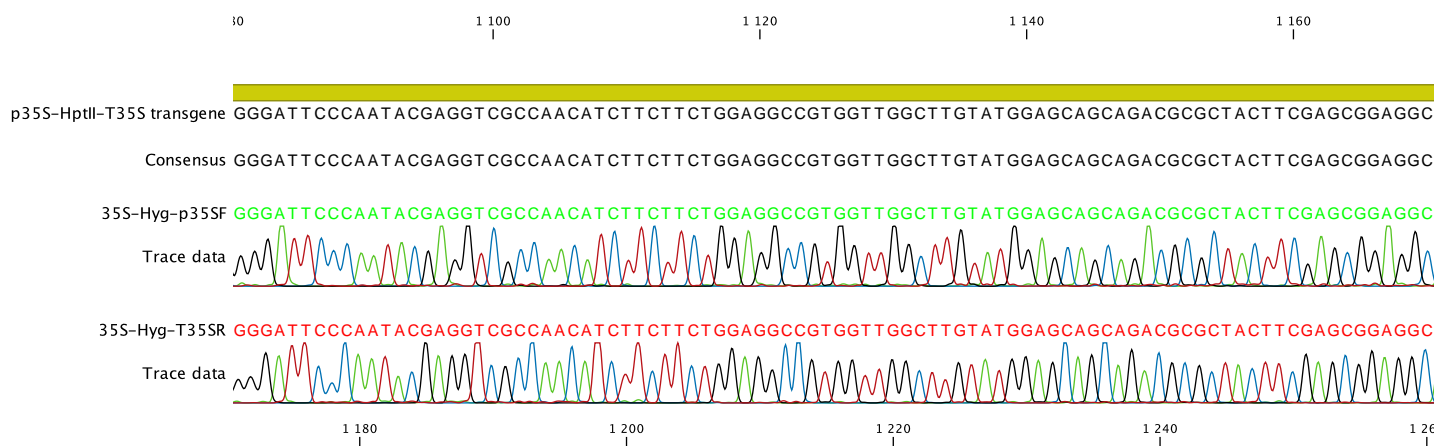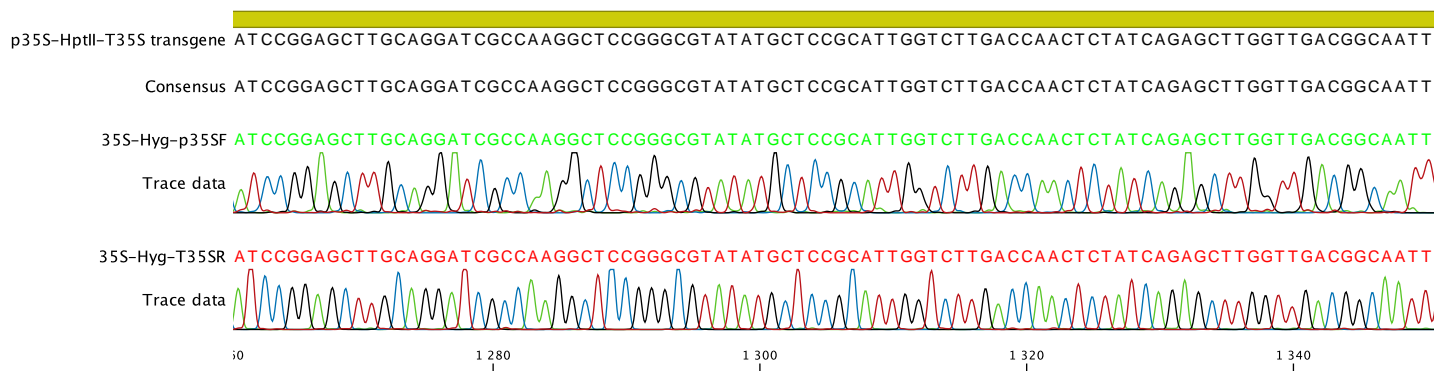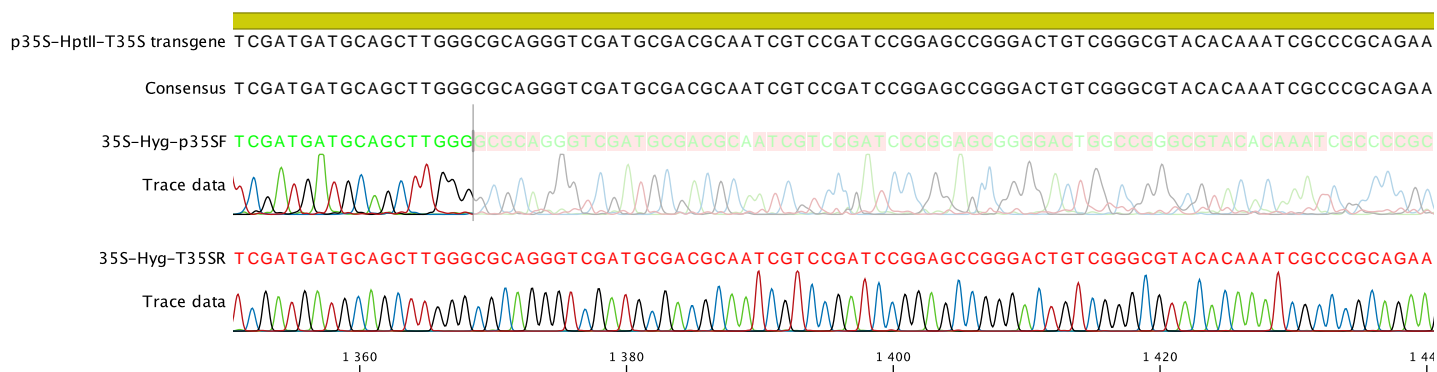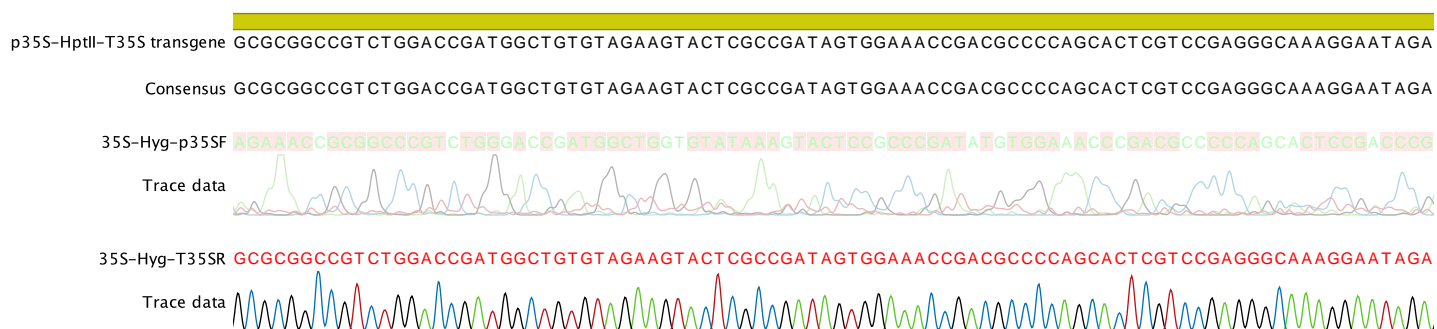

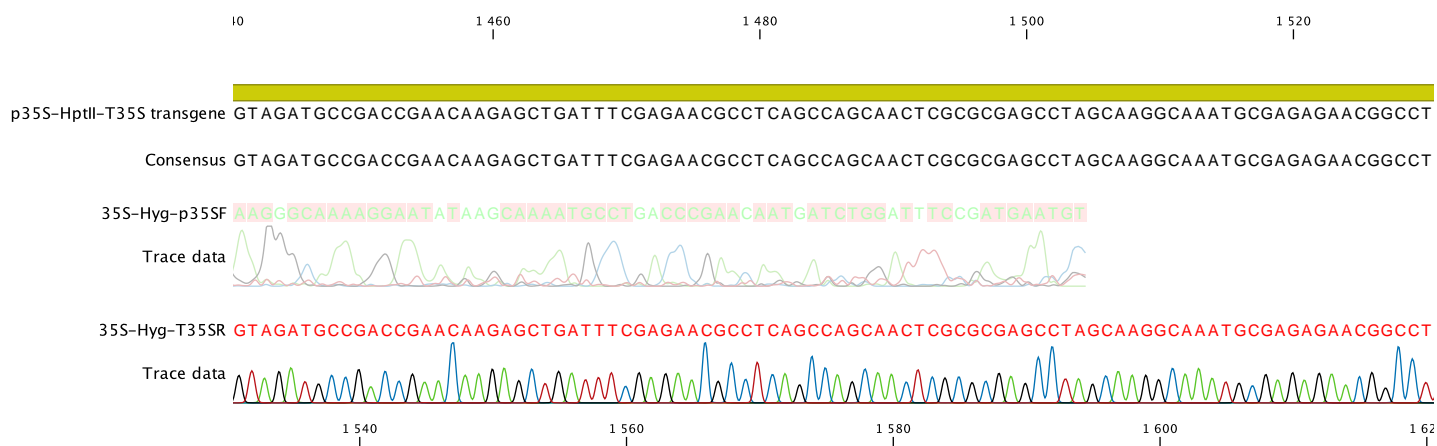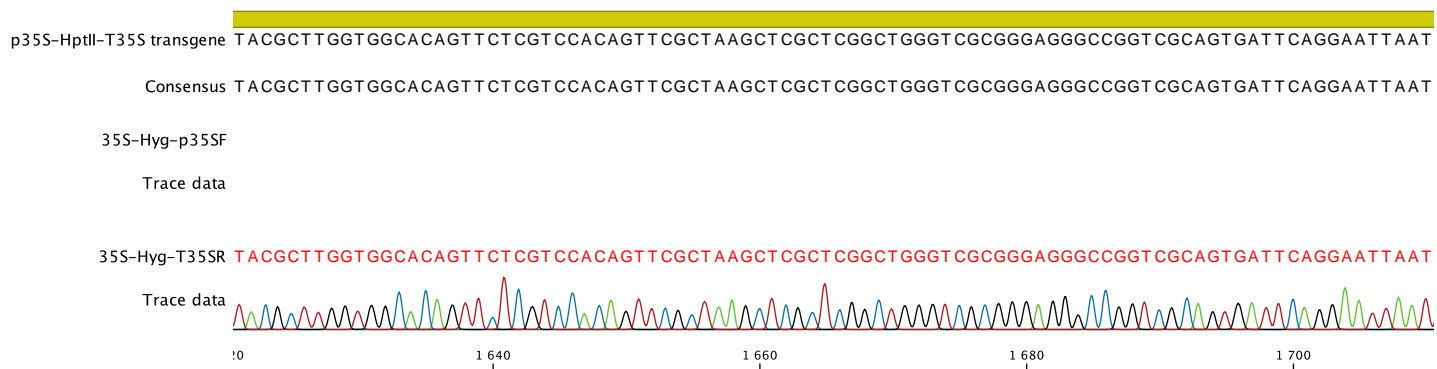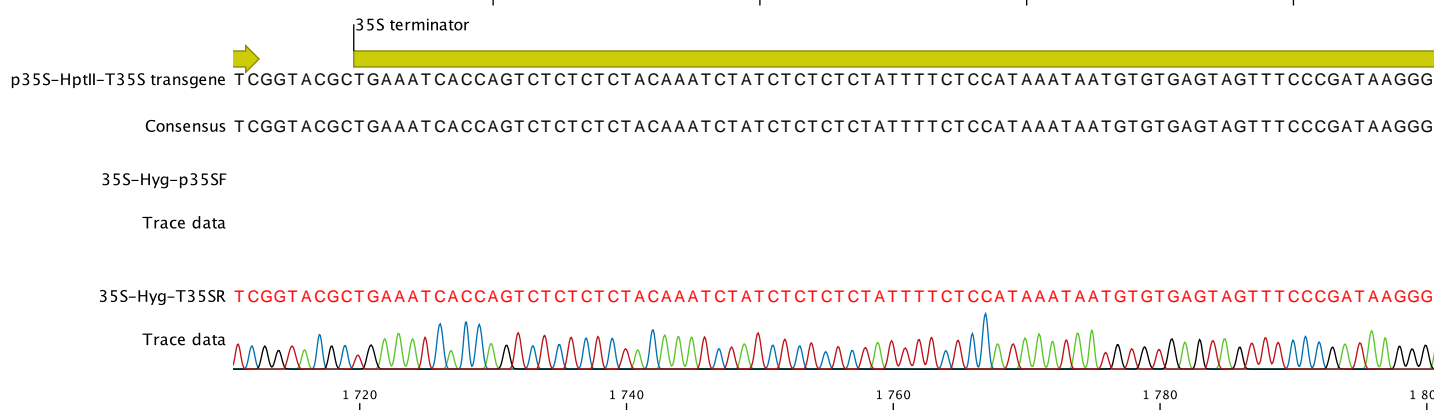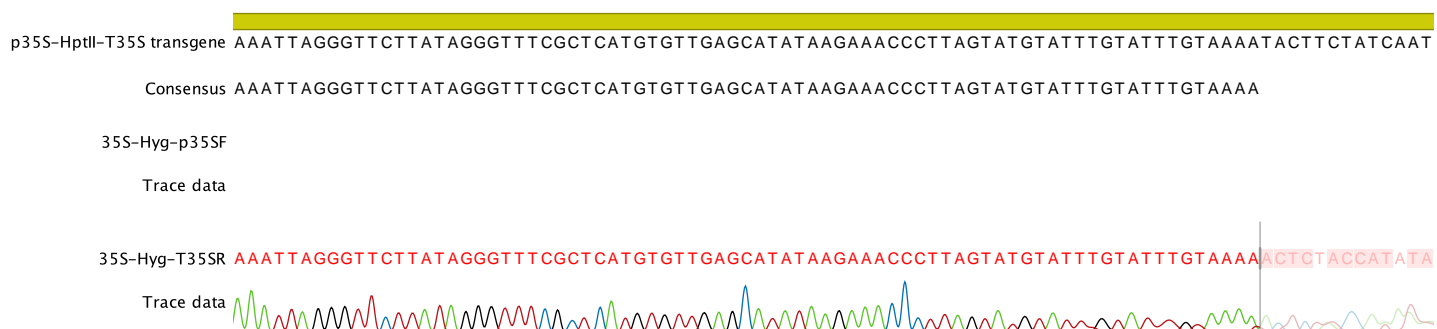

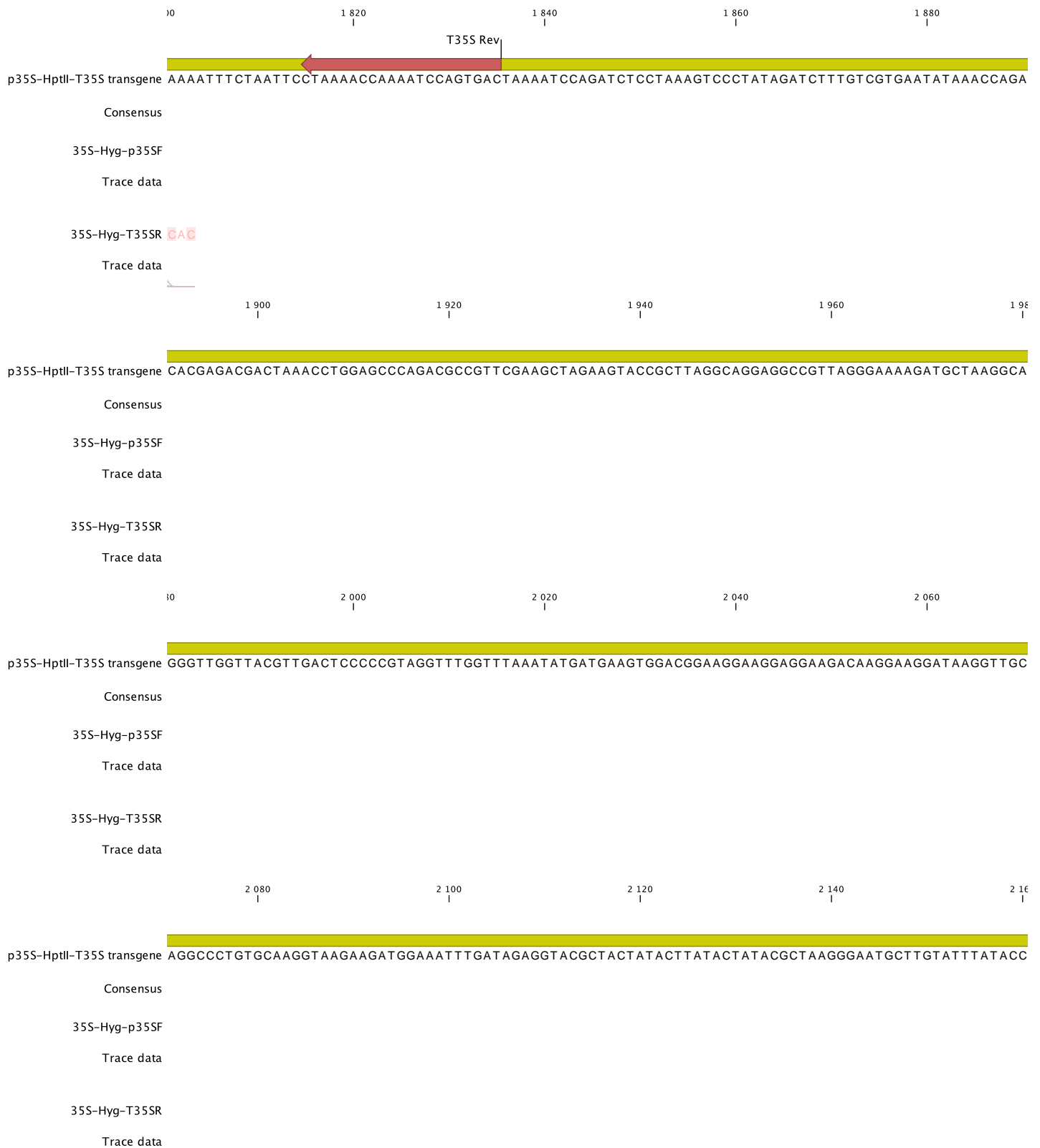

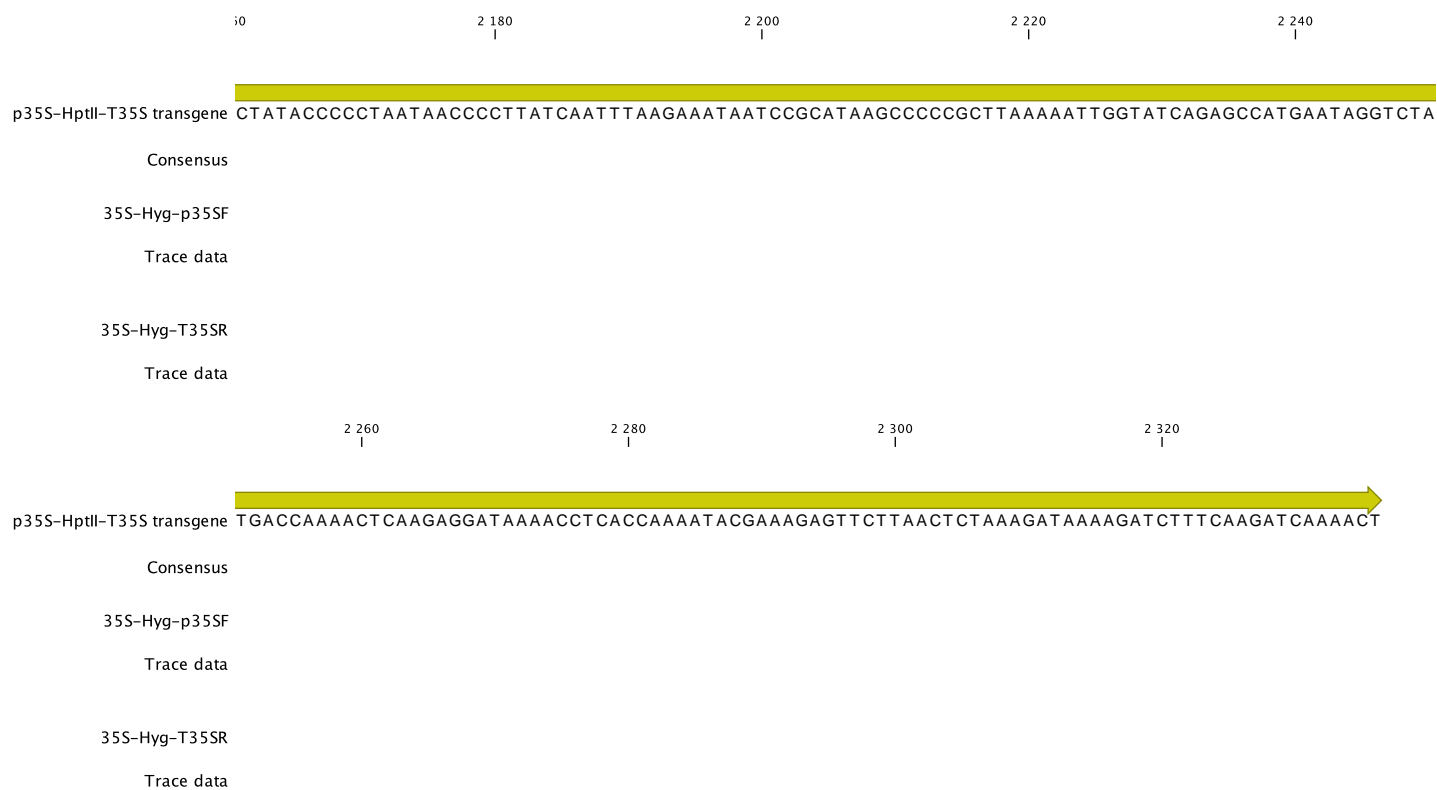

Supplement: S8 Fig — The hygromycin selection gene present in the HcPro transgenic line was also assembled. (ZIP) [file ppat.1005627.s008.zip › contig 35S-HptII.compressed.pdf]
