# Supplementary material for: Correction: Misregulation of AUXIN RESPONSE FACTOR 8 Underlies the Developmental Abnormalities Caused by Three Distinct Viral Silencing Suppressors in Arabidopsis
Source: PLoS Pathog. 2016 May 5;12(5):e1005627. doi: 10.1371/journal.ppat.1005627 (PMC4858414; doi:10.1371/journal.ppat.1005627)
Supplement: S2 Fig — (A) Original films and coomassie stainings used for mounting Fig 5A. Left panels: Western analysis of DCL1. Right panels: Western analysis of AGO1. (B) Original films used for mounting Fig 5B. Fig 5B was mounted from films obtained by sequentially stripping and re-hybridizing a single Northern blot membrane with several distinct miRNA probes. U6 and miR168 were hybridized at the same time. The long film exposure (2h30) was selected for miR168, and the short exposure (1h) was selected for U6, to avoid a saturated loading signal. Col-0 and arf8-6 -/- are respectively on tracks #7 and #6. rRNA, not used for the mounting of Fig 5B, provides an additional loading control. (C) Original films used for mounting Fig 5E. Fig 5E was mounted from films obtained by sequentially stripping and re-hybridizing two Northern blot membranes with several distinct miRNA/siRNA probes. U6 and miR168 were hybridized at the same time. The long film exposure (4h30) was selected for miR168, and the short exposure (1h30) was selected for U6, to avoid a saturated loading signal. arf8-6 +/- (CHS-RNAi) control (-), crossed with Hc, P19 or P15 are respectively on tracks #2, #3, #4 and #5. Left panels: films of the two membranes re-hybridized with CHS siRNA in June 2015. (PPTX) [file ppat.1005627.s002.pptx]

## Slide 1
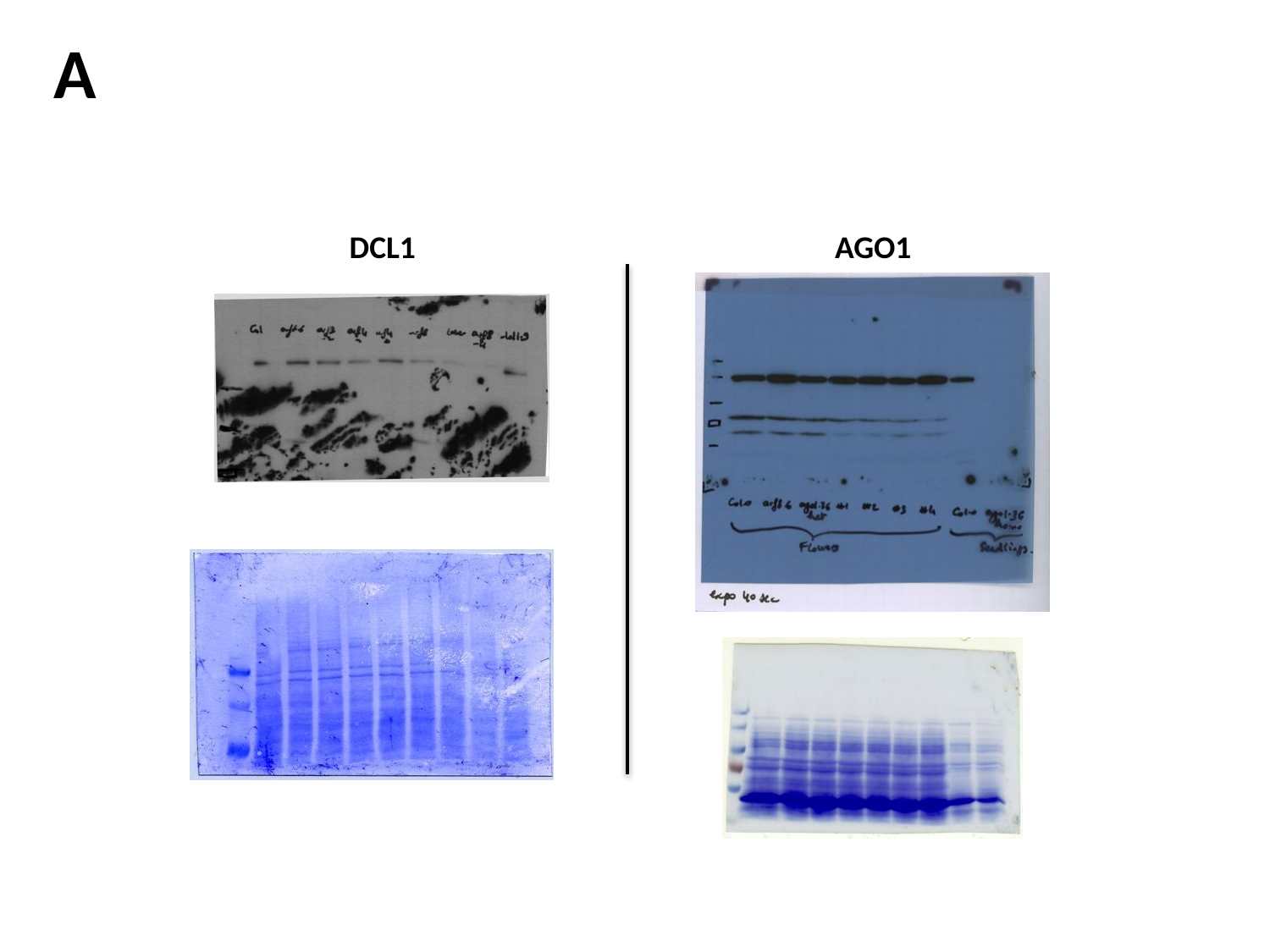

A
DCL1
AGO1

## Slide 2
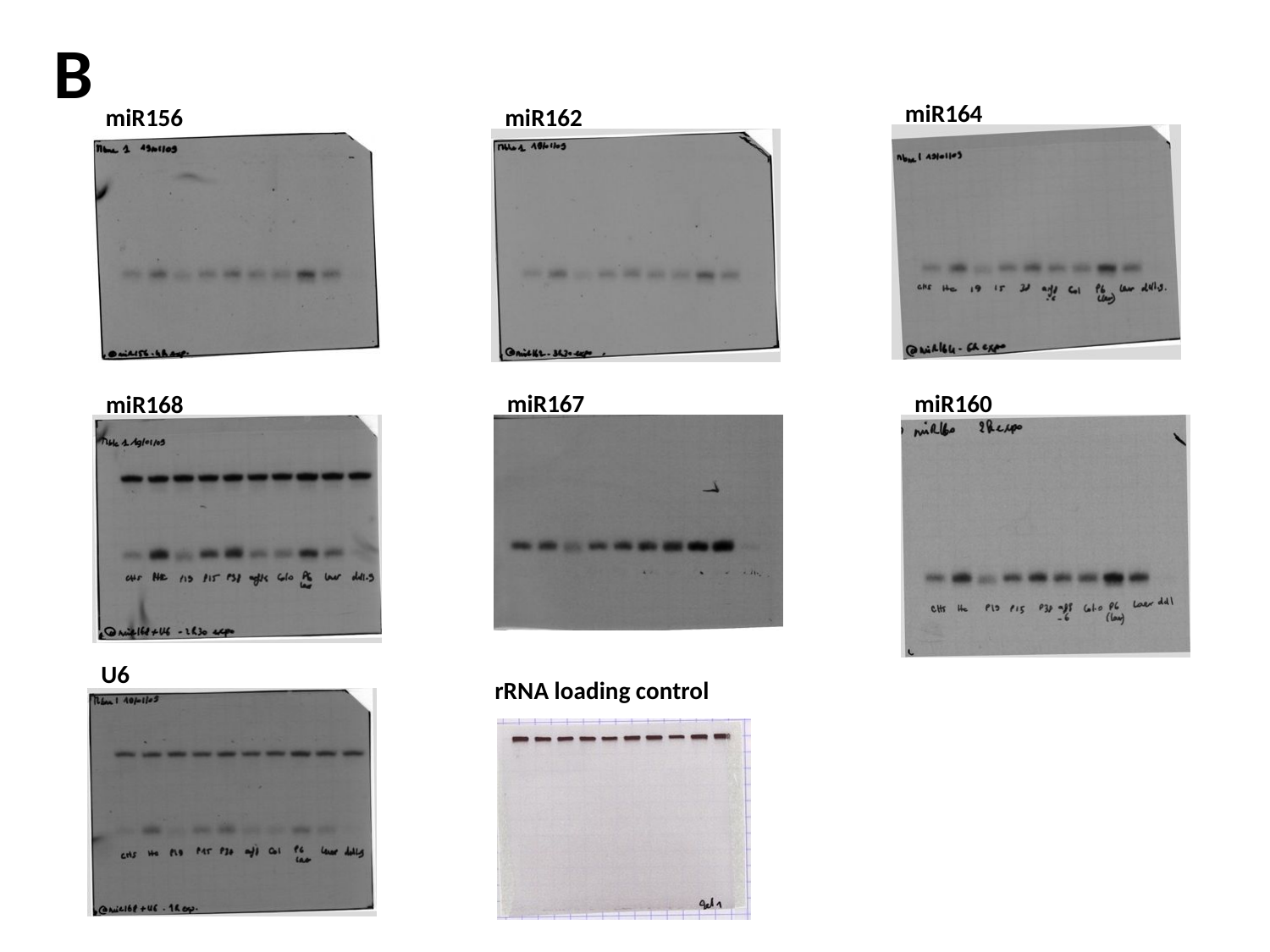

B
miR164
miR156
miR162
miR167
miR160
miR168
U6
rRNA loading control

## Slide 3
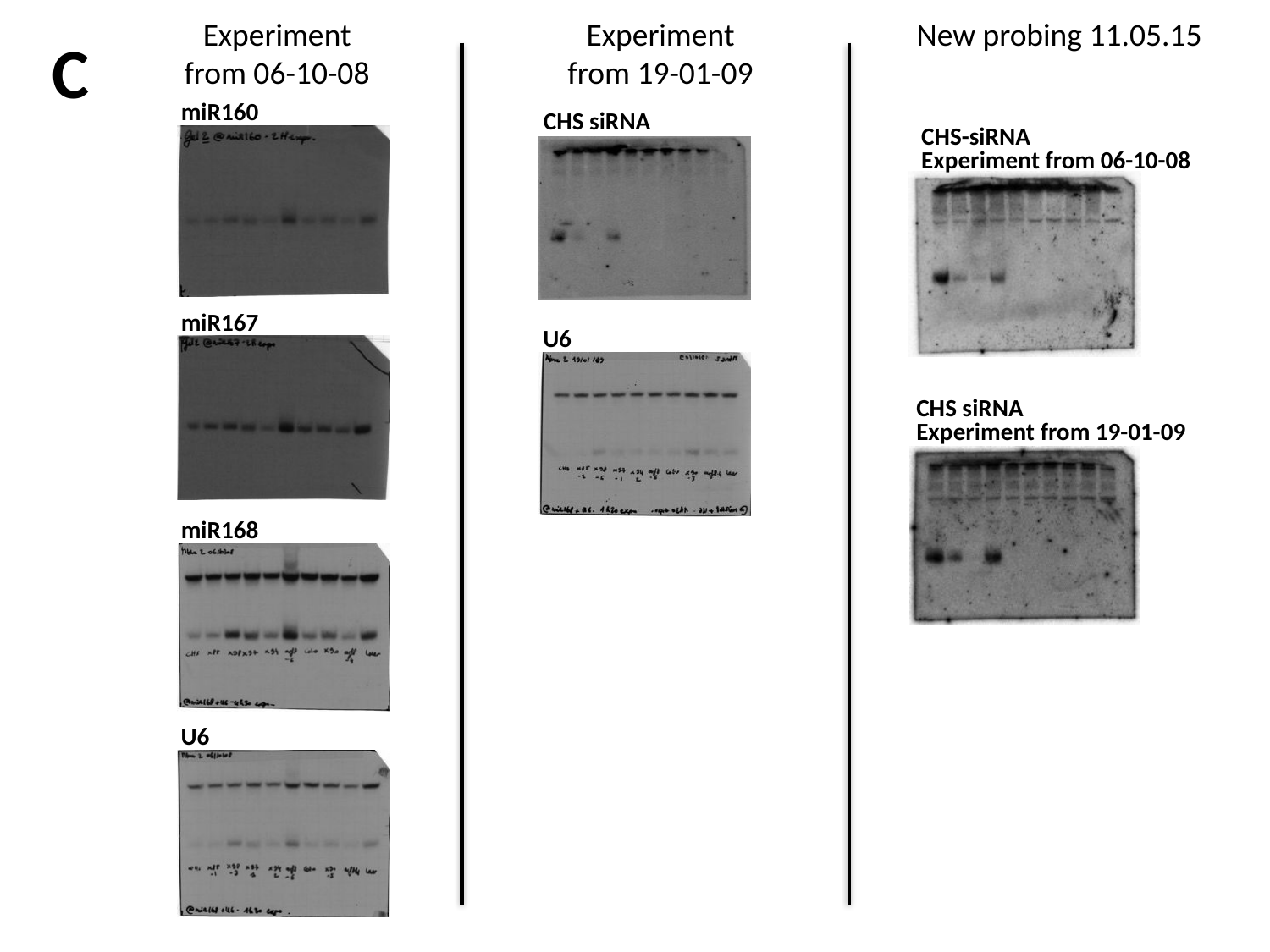

Experiment
from 06-10-08
Experiment
from 19-01-09
New probing 11.05.15
C
miR160
CHS siRNA
CHS-siRNA
Experiment from 06-10-08
miR167
U6
CHS siRNA
Experiment from 19-01-09
miR168
U6
