# Supplementary material for: Correction: Misregulation of AUXIN RESPONSE FACTOR 8 Underlies the Developmental Abnormalities Caused by Three Distinct Viral Silencing Suppressors in Arabidopsis
Source: PLoS Pathog. 2016 May 5;12(5):e1005627. doi: 10.1371/journal.ppat.1005627 (PMC4858414; doi:10.1371/journal.ppat.1005627)
Supplement: S3 Fig — (A) Original film (left) used for mounting Fig 6B and original ethidium bromide staining of the corresponding high molecular Northern gel (right). The blot was hybridized with a mix of random-labeled PCR products corresponding to the 35S terminator and HcPro, allowing detection of the P6 transcripts (upper lane). Blue rectangle indicates the part of the blot used for mounting this figure; samples were loaded according to the track labels; surrounded numbers correspond to the annotated samples on the original Fig 6B. (B) Crop and uncropped original scans of the unrelated ethidium bromide staining used for mounting Fig 6B. Blue rectangles indicate the three tracks erroneously used for mounting Fig 6B. (C) Scan obtained by re-probing the original membrane used in Fig 6B to detect the Actin2 housekeeping mRNA. Samples were loaded according to the track labels; surrounded numbers correspond to the annotated samples on the original Fig 6B. (D) Original pre-loading control corresponding to the ethidium bromide stainings of 1 μg of total RNA loaded on a 1% agarose gel to check quality and equal loading prior loading of the high molecular Northern blot used in Fig 6B. (E) Original film (left) and ethidium bromide staining (right) used for mounting Fig 6D. The blot was hybridized with random-labeled PCR products corresponding to HcPro. Samples were loaded according to the tracks labels. (PPTX) [file ppat.1005627.s003.pptx]

## Slide 1
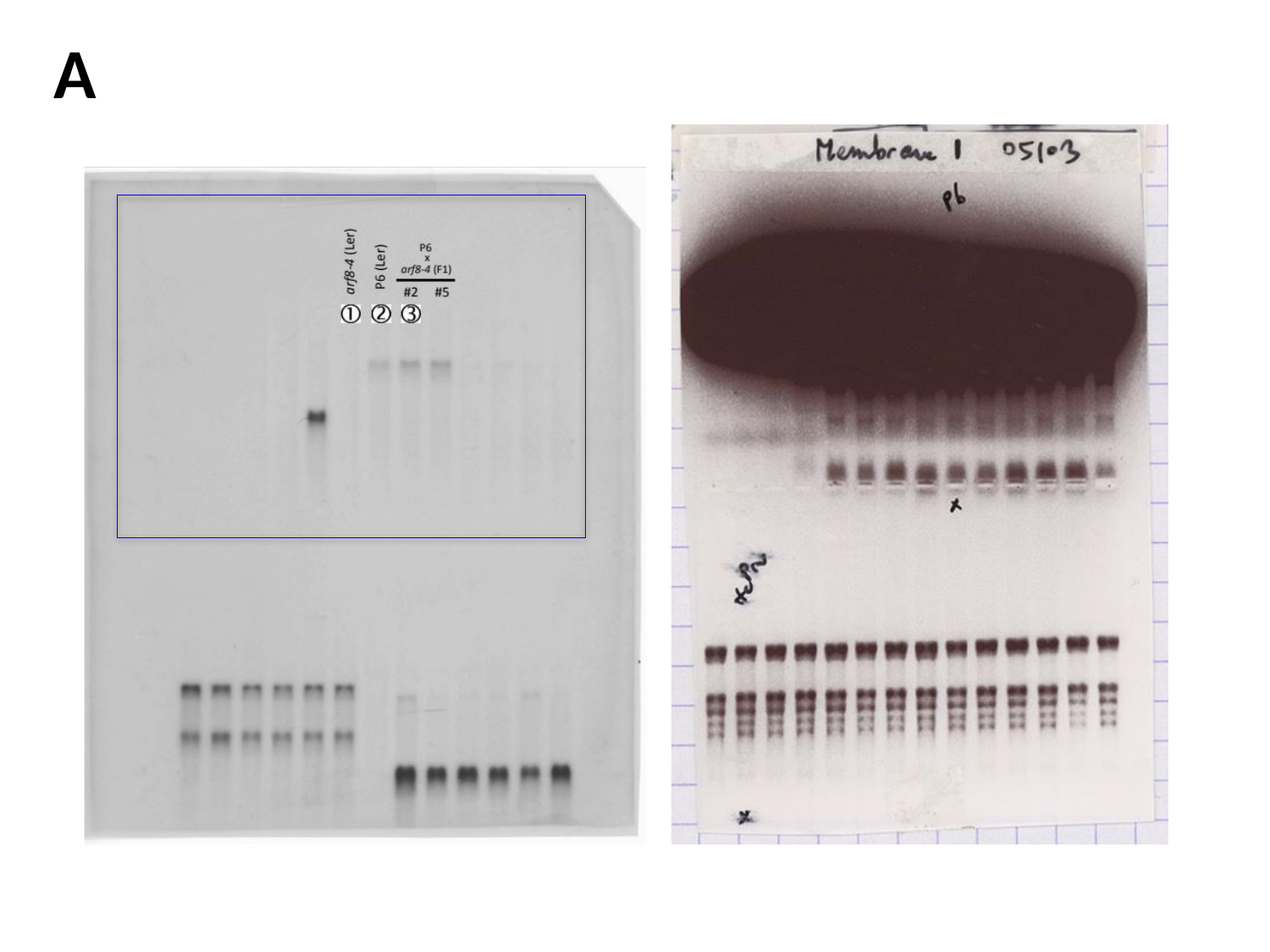

A

## Slide 2
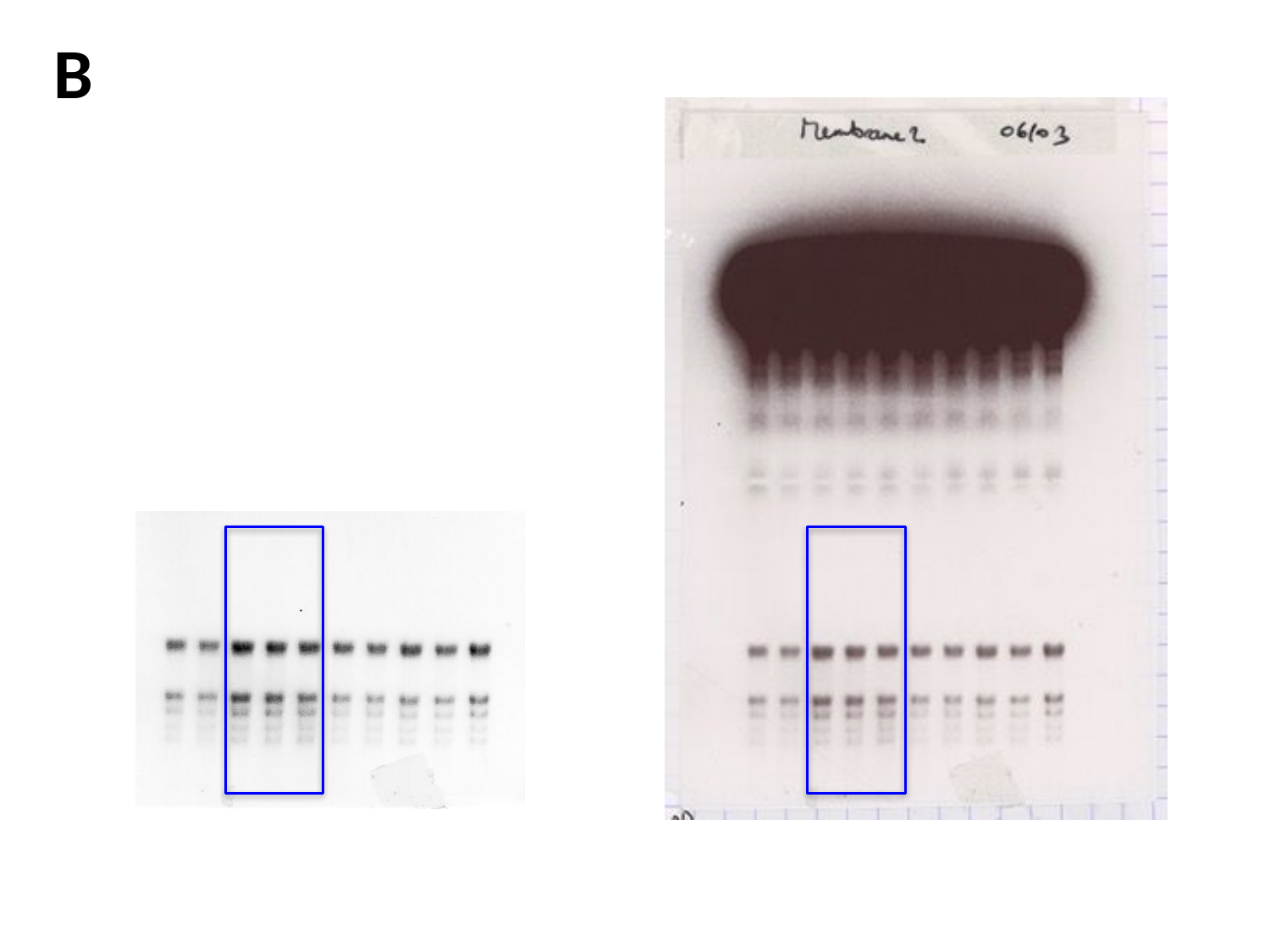

B

## Slide 3
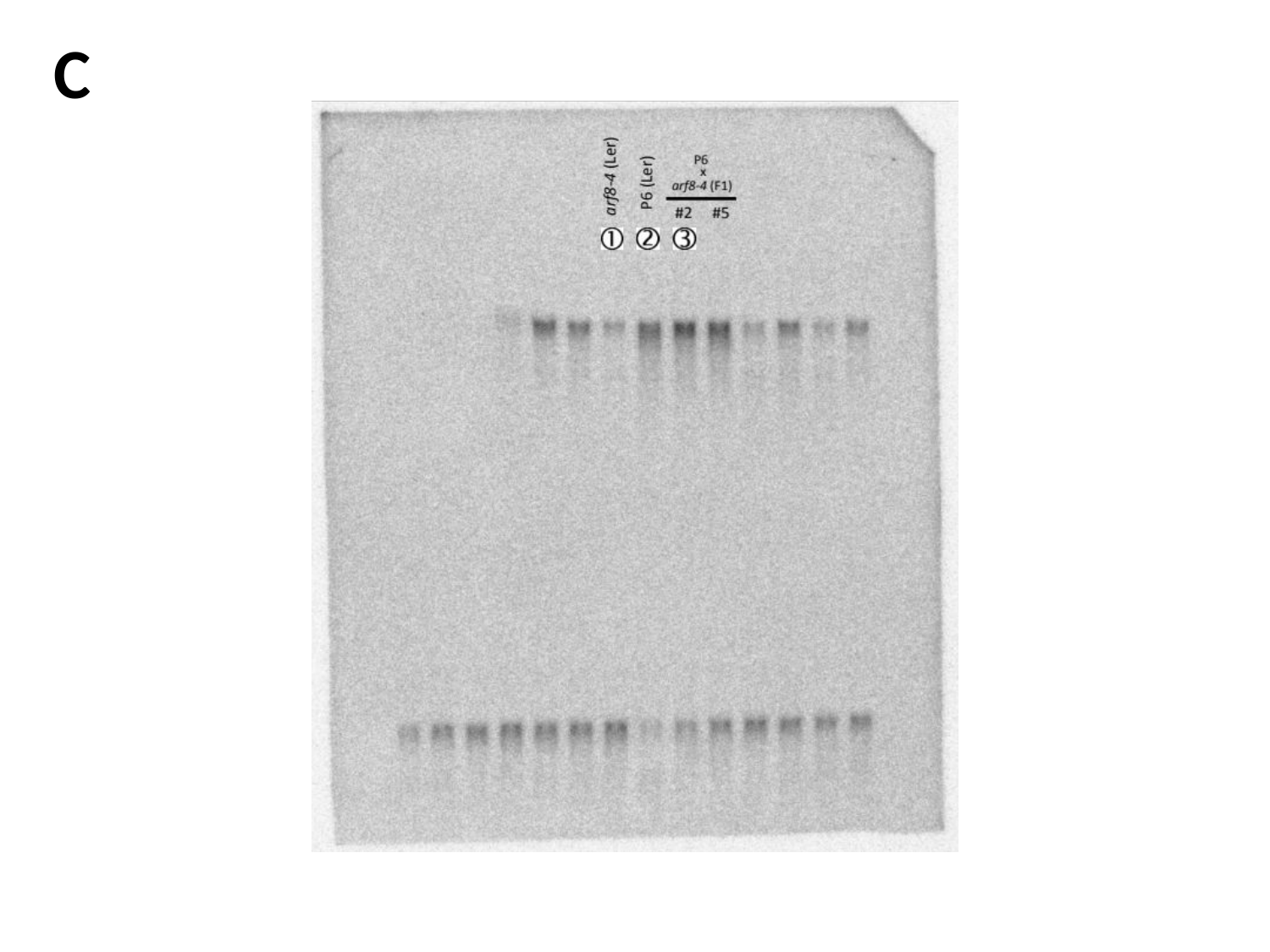

C

## Slide 4
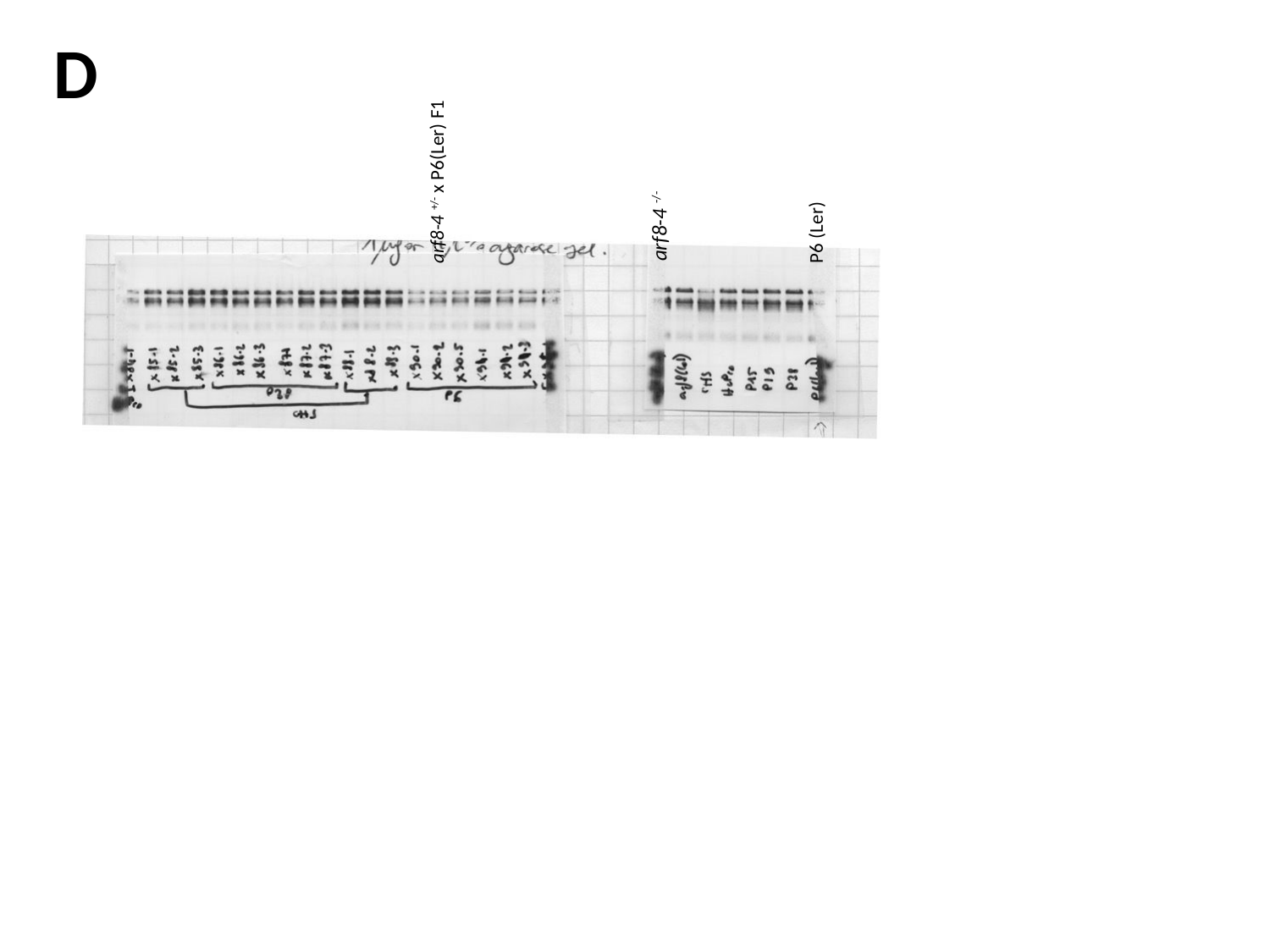

D
arf8-4 +/- x P6(Ler) F1
arf8-4 -/-
P6 (Ler)

## Slide 5
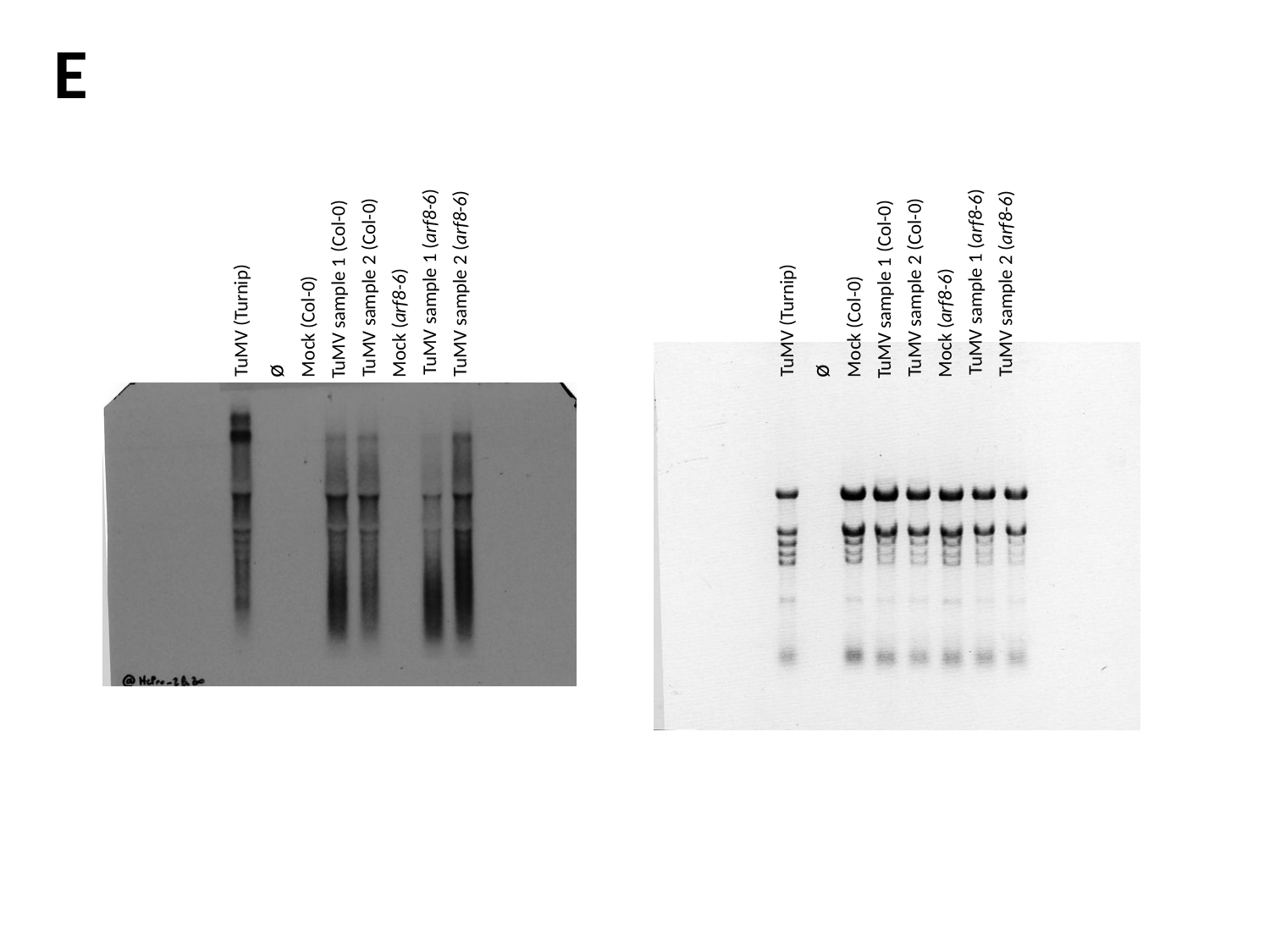

E
TuMV sample 1 (arf8-6)
TuMV sample 2 (arf8-6)
TuMV sample 1 (Col-0)
TuMV sample 2 (Col-0)
TuMV (Turnip)
Mock (arf8-6)
Mock (Col-0)
ø
TuMV sample 1 (arf8-6)
TuMV sample 2 (arf8-6)
TuMV sample 1 (Col-0)
TuMV sample 2 (Col-0)
TuMV (Turnip)
Mock (arf8-6)
Mock (Col-0)
ø
