# Supplementary material for: Correction: Misregulation of AUXIN RESPONSE FACTOR 8 Underlies the Developmental Abnormalities Caused by Three Distinct Viral Silencing Suppressors in Arabidopsis
Source: PLoS Pathog. 2016 May 5;12(5):e1005627. doi: 10.1371/journal.ppat.1005627 (PMC4858414; doi:10.1371/journal.ppat.1005627)
Supplement: S4 Fig — (A) Total gDNA loaded on 1.2% agarose gel. (B) Primers used for PCR and sequencing. (C) ACTIN2 amplification (PCR of 28 cycles using primers Actin2-For and Actin2-Rev) loaded on 1.2% agarose gel. (D) PCR amplification of the CHS RNAi transgene (PCR of 30 cycles using primers 35S-For and CHS-Rev) loaded on 1.2% agarose gel. E) P15, P19 and HC-Pro transgene amplification (PCR of 40 cycles using primers 35S-For and T35S-Rev), loaded on 1.2% agarose gel, used for sequencing. Bands extracted for sequencing are indicated for each line. The HC-Pro line presents two bands, one corresponding to the HC-Pro sequence, the second corresponding to the hygromycin resistance gene (HptII), also amplified with the primers used. (PDF) [file ppat.1005627.s004.pdf]

A

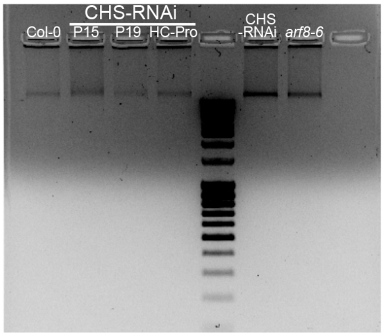

B

Primer name	Sequence
Actin2-For	GCACCCTGTTCTTCTTACCG
Actin2-Rev	AACCCTCGTAGATTGGCACA
35S-For	CACTGACGTAAGGGATGACGC
T35S-Rev	GTCACTGGATTTTGGTTTTAG
P1/H-Pro_seq1F	TGGAAGCCTTGACCCAGTAG
P1/H-Pro_seq2F	ACGAACGAAATTCGCAAGAA
P1/H-Pro_seq3R	CATGCATGGACGAAGTCAC
P1/H-Pro_seq4R	CTTGACTCCAAATTACATCTCGTG
CHS-Rev	GCAACCTTGCTGGTACATCAT

C

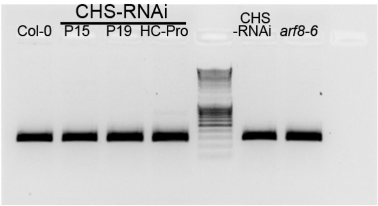

D

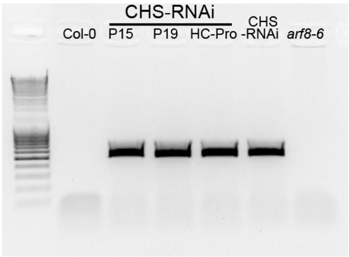

E

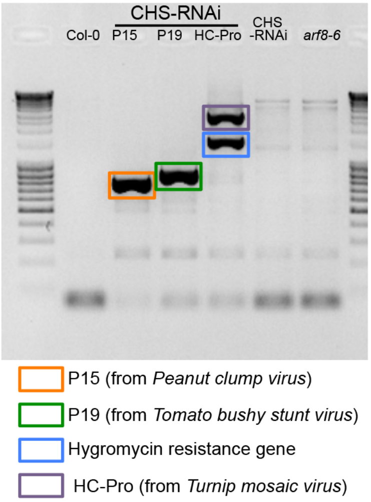

Figure 1. Genotyping of the suppressor lines used in Jay *et al.*, 2011. A) Total gDNA loaded on 1.2 % agarose gel. **B**) Primers used for PCR and sequencing. **C**) *ACTIN2* amplification (PCR of 28 cycles using primers Actin2-For and Actin2-Rev) loaded on 1.2 % agarose gel. **D**) *CHS-RNAi* amplification (PCR of 30 cycles using primers 35S-For and CHS-Rev) loaded on 1.2 % agarose gel. **E**) *P15*, *P19* and *HC-Pro* transgene amplification (PCR of 40 cycles using primers 35S-For and T35S-Rev), loaded on 1.2 % agarose gel, used for sequencing. Bands extracted for sequencing are indicated for each line. The HC-Pro line presents two bands, one corresponding to the HC-Pro sequence, the second one corresponding to the hygromycin resistance gene (*HptII*), also amplified with the primers used.
