# Supplementary material for: Correction: Misregulation of AUXIN RESPONSE FACTOR 8 Underlies the Developmental Abnormalities Caused by Three Distinct Viral Silencing Suppressors in Arabidopsis
Source: PLoS Pathog. 2016 May 5;12(5):e1005627. doi: 10.1371/journal.ppat.1005627 (PMC4858414; doi:10.1371/journal.ppat.1005627)
Supplement: S5 Fig — P15 was detected from 20 μg of total proteins from seedlings. The P15 antibody was used at a 1/10 000 dilution. P19 was detected from 100 μg of total proteins from seedlings. The P19 antibody was used at a 1/5000 dilution. Hc-Pro was detected from 20 μg of total proteins from seedlings. The Hc-Pro antibody was used at a 1/8000 dilution. The red arrow indicates the position of the P19 signal. (PDF) [file ppat.1005627.s005.pdf]

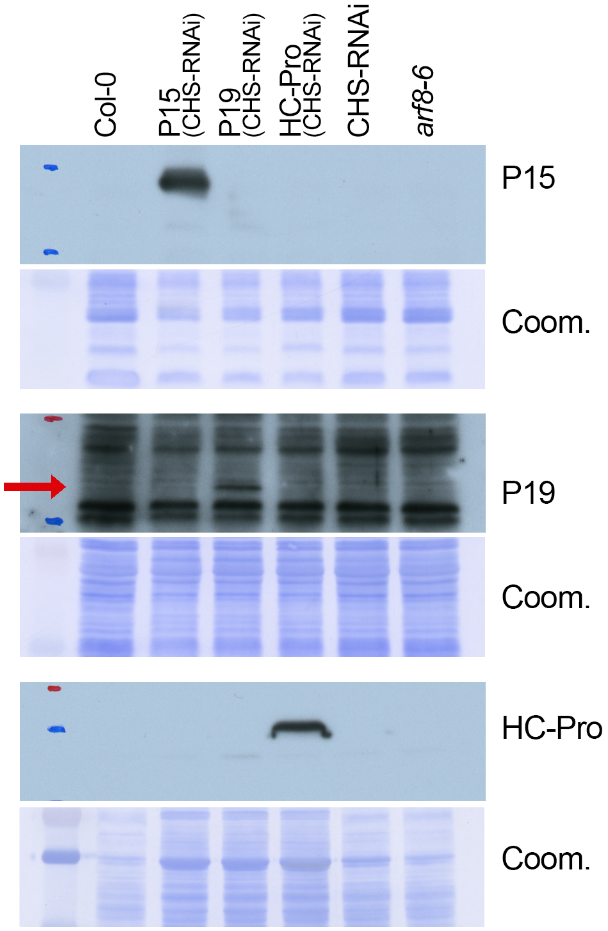

Figure 2. Western blot analysis of the suppressor lines used in Jay et al., 2011. P15 was detected from 20 µg of total proteins from seedlings. P15 antibody was used at the 1/10 000 dilution. P19 was detected from 100 µg of total proteins from seedlings. P19 antibody was used at the 1/5000 dilution. Hc-Pro was detected from 20 µg of total proteins from seedlings. HC-Pro antibody was used at the 1/8000 dilution. Red arrow indicates the position of P19 signal.
