# Supplementary material for: Correction: Misregulation of AUXIN RESPONSE FACTOR 8 Underlies the Developmental Abnormalities Caused by Three Distinct Viral Silencing Suppressors in Arabidopsis
Source: PLoS Pathog. 2016 May 5;12(5):e1005627. doi: 10.1371/journal.ppat.1005627 (PMC4858414; doi:10.1371/journal.ppat.1005627)
Supplement: S6 Text — (PDF) [file ppat.1005627.s006.pdf]

Plant material and growth conditions

The *CHS*-RNAi transgenic line and *arf8-6* mutant are in the Col-0 background and were described previously (Wesley *et al.*, 2001; Goetz *et al.*, 2006). P15, P19 and P1-HC-Pro expressing lines (in the *CHS*-RNAi background) were compared with the Col-0 or CHS-RNAi background alone. Seedlings were grown on MS media with 1 % sucrose in a growth chamber under a 12 hour light/12 hour dark regimen.

gDNA extraction, amplification and sequencing

Total gDNA was extracted from 10 days old seedlings and used for PCR amplifications with Phusion HF DNA Polymerase (Thermo Scientific). Primers used for amplification and/or sequencing are presented in Fig. 1B of the document Supp lines characterization". Amplified PCR products were purified from 1.2 % agarose gels (GeneJet PCR purification kit, Thermo Scientific) and sent to GATC for Sänger sequencing (https://www.gatc-biotech.com, Deutschland). For long sequences such as P1-HcPro, several PCR amplifications were necessary to establish overlapping contigs. All raw sequencing files have been provided alongside the contigs annotation.

Western-blot analysis

The epitopes used for raising peptide-based polyclonal antibodies against the various suppressors were chosen according to the EuroGentech specification and injected into rabbits, as follows:

For P1-HcPro injection:

```
EP111852: HcPro TuMV-1
H2N  -  SEL SQG QAS GPS MKH C  -  CONH2 (16 AA)

EP111853: HcPro TuMV-2
H2N  -  DRY EQS LSS ANE NYQ D  -  CONH2 (16 AA)
```

For purification of the anti-P1-Hcpro antibody:

```
EP111852: HcPro TuMV-1
H2N  -  SEL SQG QAS GPS MKH C  -  CONH2 (16 AA)
```

For P19 injection:

```
EP111850: P19 TBSV-1
H2N  -  RLH NDE TNS NQD NPL G  -  CONH2 (16 AA)

EP111851: P19 TBSV-2
H2N  -  QGN DAR EQA NSE RWD G  -  CONH2 (16 AA)
```

For purification of the anti-P19 antibody:

```
EP111850: P19 TBSV-1
H2N  -  RLH NDE TNS NQD NPL G  -  CONH2 (16 AA
```

For P15 injection:

```
 EP1310885:  P15 aa1-15
 H  -  MPK SEF FRE ERK RRV C  -  NH2  (16AA)

 EP1310886:  P15 aa110-124
 H  -  CIA LLS EKF KNL RSK L  -  OH  (16AA)
```

For purification of the anti-P15 antibody:

```
 EP1310885:  P15 aa1-15
 H  -  MPK SEF FRE ERK RRV C  -  NH2  (16AA)
```

These antibodies were validated in 2013-2014 by our laboratory technician, Gregory Schott and the corresponding data are available upon request. For western analysis, total proteins were isolated from the phenol-chloroform phase left from the first step of RNA extraction using Trizol by precipitation with 4 volumes of ammonium acetate 0.1M in methanol. Proteins were resolved by SDS-PAGE and electrotransferred to Immobilon-P PVDF membrane (Millipore). Following one

hour blocking step in PBS + 0.1% Tween-20 supplemented with 1% BSA, antibody incubations were carried out overnight at 4°C with constant shaking. Primary antibodies were used with the following dilutions P15 (1/10 000), P19 (1/5000), HC-Pro (1/8000). Following three washes in PBS + 0.1% Tween-20, membranes were incubated for 1 hour in HRP-conjugated goat anti-rabbit antibodies then rinsed three times before detection with ECL Western Blotting Detection Kit (GE Healthcare).

References

Goetz M, Vivian-Smith A, Johnson SD, Koltunow AM (2006). AUXIN RESPONSE FACTOR8 is a negative regulator of fruit initiation in Arabidopsis. *Plant Cell* **18**: 1873–1886.

Wesley SV, Helliwell CA, Smith NA, Wang M, Rouse DT, Liu Q, Gooding PS, Singh SP, Abbott D, Stoutjesdijk PA, Robinson SP, Gleave AP, Green AG, Waterhouse P (2001). Construct design for efficient, effective and high-throughput gene silencing in plants. *Plant J* **27**: 581–590
