# Supplementary material for: Correction: Misregulation of AUXIN RESPONSE FACTOR 8 Underlies the Developmental Abnormalities Caused by Three Distinct Viral Silencing Suppressors in Arabidopsis
Source: PLoS Pathog. 2016 May 5;12(5):e1005627. doi: 10.1371/journal.ppat.1005627 (PMC4858414; doi:10.1371/journal.ppat.1005627)
Supplement: S8 Fig — The hygromycin selection gene present in the HcPro transgenic line was also assembled. (ZIP) [file ppat.1005627.s008.zip › contig 35S-p19.compressed.pdf]

20 40 60 80

35S promoter

p35S-p19-T35S transgene / 35S-P19-T35S-Rev CTACTCCAAAAATGTCAAAGATACAGTCTCAGAAGACCAAAGGGCTATTGAGACTTTTCAACAAAGGGTAATTTCGGGAAACCTCCTCGG

Trace data / Consensus

35S-P19-T35S-Rev

Trace data

35S-P19-35S-For

Trace data

100 120 140 160 180

p35S-p19-T35S transgene ATTCCATTGCCCAGCTATCTGTCACTTCATCGAAAGGACAGTAGAAAAGGAAGGTGGCTCCTACAAATGCCATCATTGCGATAAAGGAAA

Consensus

35S-P19-T35S-Rev

Trace data

35S-P19-35S-For

Trace data

0 200 220 240 260

p35S-p19-T35S transgene GGCTATCATTCAAGATGCCTCTGCCGACAGTGGTCCCAAAGATGGACCCCCACCCACGAGGAGCATCGTGGAAAAAGAAGACGTCCCAAC

Consensus

35S-P19-T35S-Rev

Trace data

35S-P19-35S-For

Trace data

280 300 320 340 360

p35S For

p35S-p19-T35S transgene CACGTCTTCAAAGCAAGTGGATTGATGTGACATCTCCACTGACGTAAGGGATGACGCACAATCCCACTATCCTTCGCAAGACCCTTCCTC

Consensus GGATGACGCACAATCCCACTATCCTTCGCAAGACCCTTCCTC

35S-P19-T35S-Rev CCTTGTTTCTTTTTTCCCATGAACGTTAAGGATGACGCACAATCCCACTATCCTTCGCAAGACCCTTCCTC

Trace data

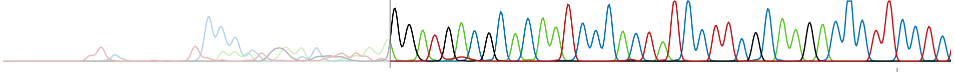

35S-P19-35S-For TATCATCCTCGCAGACCCTTCCCTC

Trace data

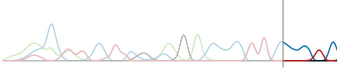

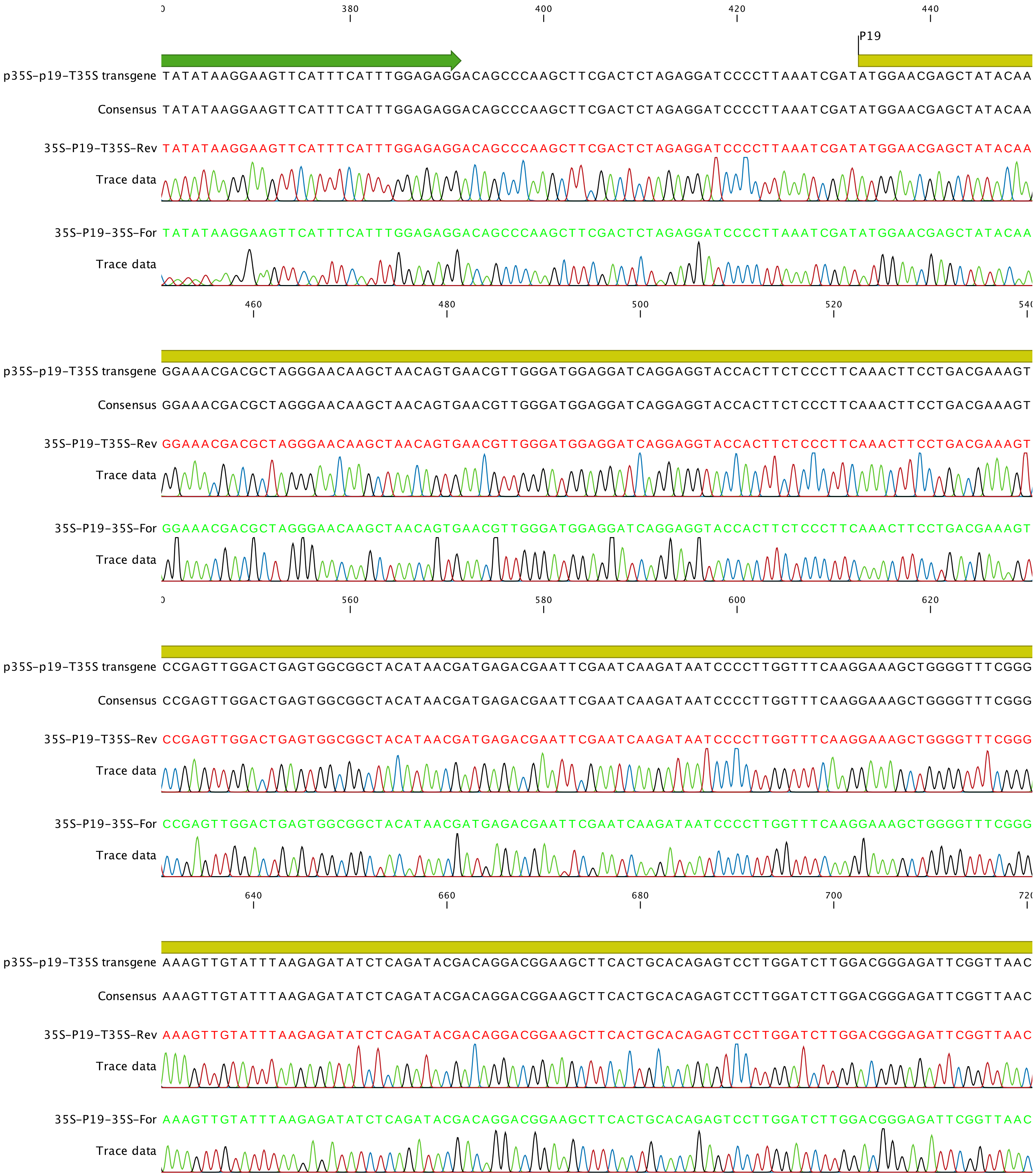
380
400
420
440
P19
p35S-p19-T35S transgene TATATAAGGAAGTTCATTTCATTTGGAGAGGACAGCCCAAGCTTCGACTCTAGAGGATCCCCTTAAATCGATATGGAACGAGCTATACAA
Consensus TATATAAGGAAGTTCATTTCATTTGGAGAGGACAGCCCAAGCTTCGACTCTAGAGGATCCCCTTAAATCGATATGGAACGAGCTATACAA
35S-P19-T35S-Rev TATATAAGGAAGTTCATTTCATTTGGAGAGGACAGCCCAAGCTTCGACTCTAGAGGATCCCCTTAAATCGATATGGAACGAGCTATACAA
Trace data
35S-P19-35S-For TATATAAGGAAGTTCATTTCATTTGGAGAGGACAGCCCAAGCTTCGACTCTAGAGGATCCCCTTAAATCGATATGGAACGAGCTATACAA
Trace data
460
480
500
520
p35S-p19-T35S transgene GGAAACGACGCTAGGGAACAAGCTAACAGTGAACGTTGGGATGGAGGATCAGGAGGTACCACTTCTCCCTTCAAACTTCCTGACGAAAGT
Consensus GGAAACGACGCTAGGGAACAAGCTAACAGTGAACGTTGGGATGGAGGATCAGGAGGTACCACTTCTCCCTTCAAACTTCCTGACGAAAGT
35S-P19-T35S-Rev GGAAACGACGCTAGGGAACAAGCTAACAGTGAACGTTGGGATGGAGGATCAGGAGGTACCACTTCTCCCTTCAAACTTCCTGACGAAAGT
Trace data
35S-P19-35S-For GGAAACGACGCTAGGGAACAAGCTAACAGTGAACGTTGGGATGGAGGATCAGGAGGTACCACTTCTCCCTTCAAACTTCCTGACGAAAGT
Trace data
560
580
600
620
p35S-p19-T35S transgene CCGAGTTGGACTGAGTGGCGGCTACATAACGATGAGACGAATTCGAATCAAGATAATCCCCTTGGTTTCAAGGAAAGCTGGGGTTTCGGG
Consensus CCGAGTTGGACTGAGTGGCGGCTACATAACGATGAGACGAATTCGAATCAAGATAATCCCCTTGGTTTCAAGGAAAGCTGGGGTTTCGGG
35S-P19-T35S-Rev CCGAGTTGGACTGAGTGGCGGCTACATAACGATGAGACGAATTCGAATCAAGATAATCCCCTTGGTTTCAAGGAAAGCTGGGGTTTCGGG
Trace data
35S-P19-35S-For CCGAGTTGGACTGAGTGGCGGCTACATAACGATGAGACGAATTCGAATCAAGATAATCCCCTTGGTTTCAAGGAAAGCTGGGGTTTCGGG
Trace data
640
660
680
700
p35S-p19-T35S transgene AAAGTTGTATTTAAGAGATATCTCAGATACGACAGGACGGAAGCTTCACTGCACAGAGTCCTTGGATCTTGGACGGGAGATTCGGTTAAC
Consensus AAAGTTGTATTTAAGAGATATCTCAGATACGACAGGACGGAAGCTTCACTGCACAGAGTCCTTGGATCTTGGACGGGAGATTCGGTTAAC
35S-P19-T35S-Rev AAAGTTGTATTTAAGAGATATCTCAGATACGACAGGACGGAAGCTTCACTGCACAGAGTCCTTGGATCTTGGACGGGAGATTCGGTTAAC
Trace data
35S-P19-35S-For AAAGTTGTATTTAAGAGATATCTCAGATACGACAGGACGGAAGCTTCACTGCACAGAGTCCTTGGATCTTGGACGGGAGATTCGGTTAAC
Trace data

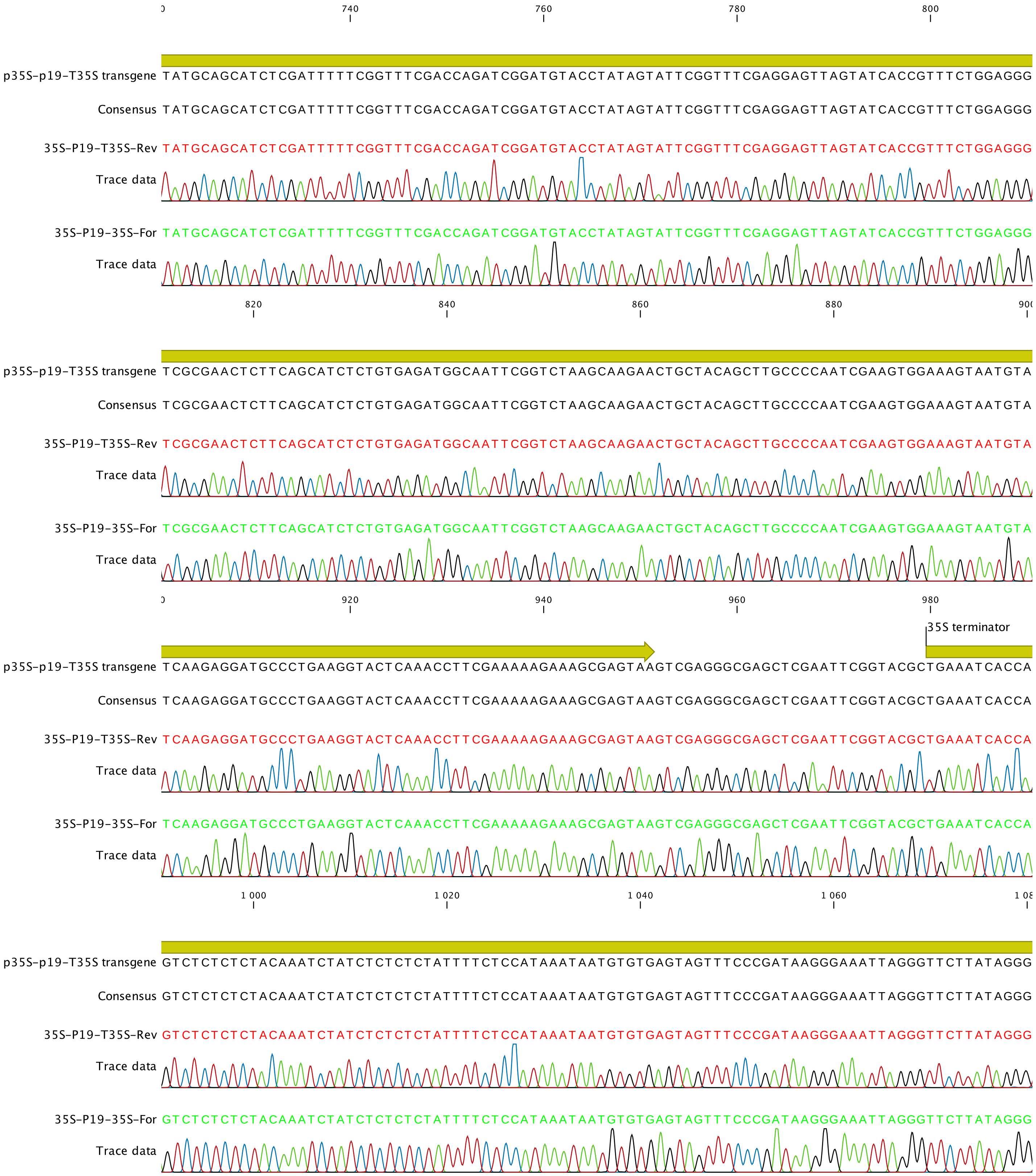

740
760
780
800
p35S-p19-T35S transgene TATGCAGCATCTCGATTTTTCGGTTTCGACCAGATCGGATGTACCTATAGTATTCGGTTTCGAGGAGTTAGTATCACCGTTTCTGGAGGG
Consensus TATGCAGCATCTCGATTTTTCGGTTTCGACCAGATCGGATGTACCTATAGTATTCGGTTTCGAGGAGTTAGTATCACCGTTTCTGGAGGG
35S-P19-T35S-Rev TATGCAGCATCTCGATTTTTCGGTTTCGACCAGATCGGATGTACCTATAGTATTCGGTTTCGAGGAGTTAGTATCACCGTTTCTGGAGGG
Trace data
35S-P19-35S-For TATGCAGCATCTCGATTTTTCGGTTTCGACCAGATCGGATGTACCTATAGTATTCGGTTTCGAGGAGTTAGTATCACCGTTTCTGGAGGG
Trace data
820
840
860
880
p35S-p19-T35S transgene TCGCGAACTCTTCAGCATCTCTGTGAGATGGCAATTCGGTCTAAGCAAGAACTGCTACAGCTTGCCCCAATCGAAGTGGAAAGTAATGTA
Consensus TCGCGAACTCTTCAGCATCTCTGTGAGATGGCAATTCGGTCTAAGCAAGAACTGCTACAGCTTGCCCCAATCGAAGTGGAAAGTAATGTA
35S-P19-T35S-Rev TCGCGAACTCTTCAGCATCTCTGTGAGATGGCAATTCGGTCTAAGCAAGAACTGCTACAGCTTGCCCCAATCGAAGTGGAAAGTAATGTA
Trace data
35S-P19-35S-For TCGCGAACTCTTCAGCATCTCTGTGAGATGGCAATTCGGTCTAAGCAAGAACTGCTACAGCTTGCCCCAATCGAAGTGGAAAGTAATGTA
Trace data
920
940
960
980
35S terminator
p35S-p19-T35S transgene TCAAGAGGATGCCCTGAAGGTACTCAAACCTTCGAAAAAGAAAGCGAGTAAGTCGAGGGCGAGCTCGAATTCGGTACGCTGAAATCACCA
Consensus TCAAGAGGATGCCCTGAAGGTACTCAAACCTTCGAAAAAGAAAGCGAGTAAGTCGAGGGCGAGCTCGAATTCGGTACGCTGAAATCACCA
35S-P19-T35S-Rev TCAAGAGGATGCCCTGAAGGTACTCAAACCTTCGAAAAAGAAAGCGAGTAAGTCGAGGGCGAGCTCGAATTCGGTACGCTGAAATCACCA
Trace data
35S-P19-35S-For TCAAGAGGATGCCCTGAAGGTACTCAAACCTTCGAAAAAGAAAGCGAGTAAGTCGAGGGCGAGCTCGAATTCGGTACGCTGAAATCACCA
Trace data
1 000
1 020
1 040
1 060
p35S-p19-T35S transgene GTCTCTCTCTACAAATCTATCTCTCTCTATTTTCTCCATAAATAATGTGTGAGTAGTTTCCCGATAAGGGAAATTAGGGTTCTTATAGGG
Consensus GTCTCTCTCTACAAATCTATCTCTCTCTATTTTCTCCATAAATAATGTGTGAGTAGTTTCCCGATAAGGGAAATTAGGGTTCTTATAGGG
35S-P19-T35S-Rev GTCTCTCTCTACAAATCTATCTCTCTCTATTTTCTCCATAAATAATGTGTGAGTAGTTTCCCGATAAGGGAAATTAGGGTTCTTATAGGG
Trace data
35S-P19-35S-For GTCTCTCTCTACAAATCTATCTCTCTCTATTTTCTCCATAAATAATGTGTGAGTAGTTTCCCGATAAGGGAAATTAGGGTTCTTATAGGG
Trace data

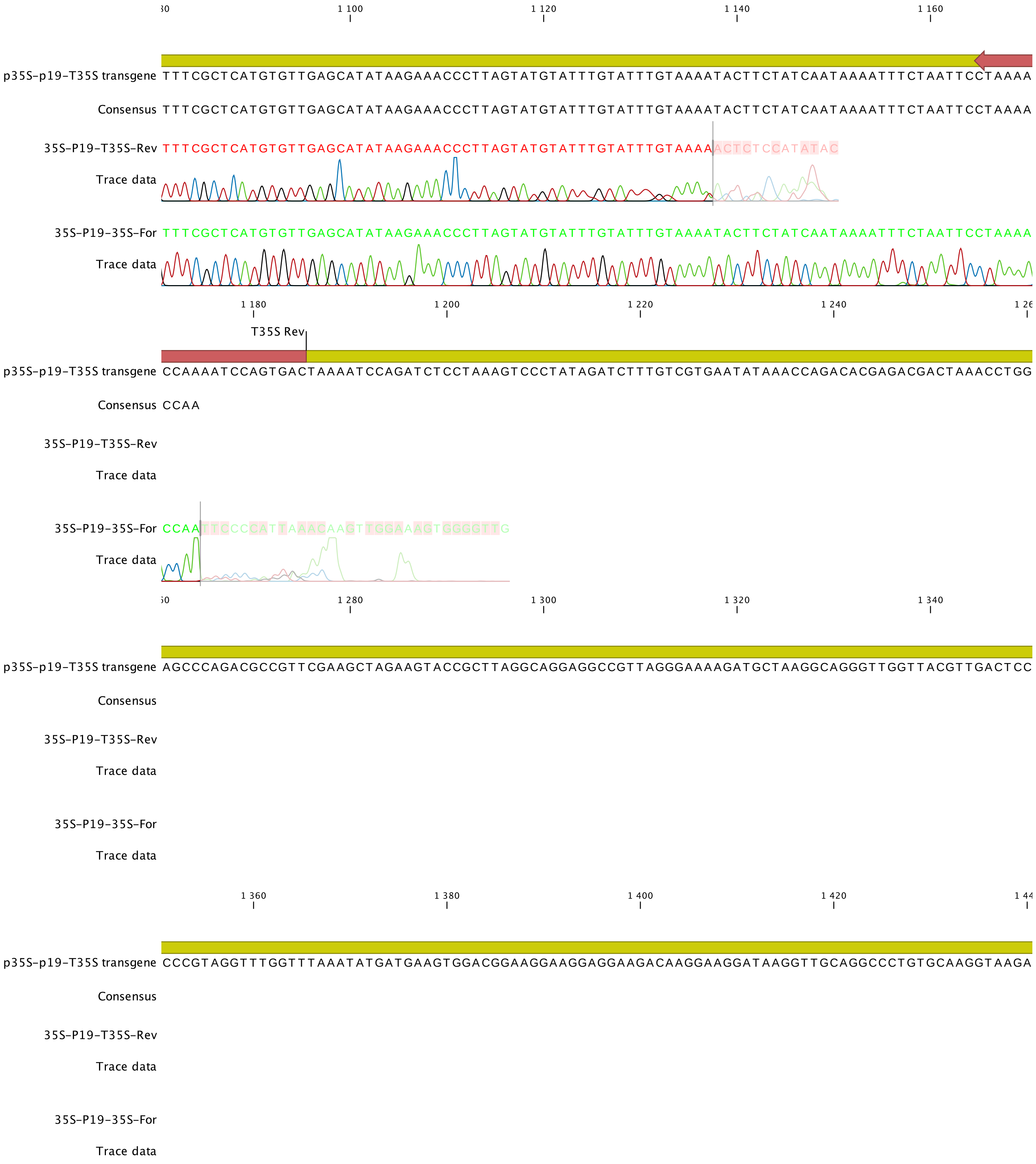
1 100
1 120
1 140
1 160
p35S-p19-T35S transgene TTTCGCTCATGTGTTGAGCATATAAGAAACCCTTAGTATGTATTTGTATTTGTAAAATACTTCTATCAATAAAATTTCTAATTCCTAAAA
Consensus TTTCGCTCATGTGTTGAGCATATAAGAAACCCTTAGTATGTATTTGTATTTGTAAAATACTTCTATCAATAAAATTTCTAATTCCTAAAA
35S-P19-T35S-Rev TTTCGCTCATGTGTTGAGCATATAAGAAACCCTTAGTATGTATTTGTATTTGTAAAAACTCTCCATATAC
Trace data
35S-P19-35S-For TTTCGCTCATGTGTTGAGCATATAAGAAACCCTTAGTATGTATTTGTATTTGTAAAATACTTCTATCAATAAAATTTCTAATTCCTAAAA
Trace data
1 180
1 200
1 220
1 240
T35S Rev
p35S-p19-T35S transgene CCAAAATCCAGTGACTAAAATCCAGATCTCCTAAAGTCCCTATAGATCTTTGTCGTGAATATAAACCAGACACGAGACGACTAAACCTGG
Consensus CCAA
35S-P19-T35S-Rev
Trace data
35S-P19-35S-For CCAATTCCCCATTAAACAAGTTGGAAAGTGGGGTTG
Trace data
1 280
1 300
1 320
1 340
p35S-p19-T35S transgene AGCCCAGACGCCGTTCGAAGCTAGAAGTACCGCTTAGGCAGGAGGCCGTTAGGGAAAAGATGCTAAGGCAGGGTTGGTTACGTTGACTCC
Consensus
35S-P19-T35S-Rev
Trace data
35S-P19-35S-For
Trace data
1 360
1 380
1 400
1 420
p35S-p19-T35S transgene CCCGTAGGTTTGGTTTAAATATGATGAAGTGGACGGAAGGAAGGAGGAAGACAAGGAAGGATAAGGTTGCAGGCCCTGTGCAAGGTAAGA
Consensus
35S-P19-T35S-Rev
Trace data
35S-P19-35S-For
Trace data

```
            40                  1 460               1 480               1 500               1 520
p35S-p19-T35S transgene  AGATGGAAATTTGATAGAGGTACGCTACTATACTTATACTATACGCTAAGGGAATGCTTGTATTTATACCCTATACCCCCTAATAACCCC
Consensus
35S-P19-T35S-Rev
Trace data

35S-P19-35S-For
Trace data

                    1 540               1 560               1 580               1 600               1 62
p35S-p19-T35S transgene  TTATCAATTTAAGAAATAATCCGCATAAGCCCCCGCTTAAAAATTGGTATCAGAGCCATGAATAGGTCTATGACCAAAACTCAAGAGGAT
Consensus
35S-P19-T35S-Rev
Trace data

35S-P19-35S-For
Trace data

            20                  1 640               1 660               1 680
p35S-p19-T35S transgene  AAAACCTCACCAAAATACGAAAGAGTTCTTAACTCTAAAGATAAAAGATCTTTCAAGATCAAAACT
Consensus
35S-P19-T35S-Rev
Trace data

35S-P19-35S-For
Trace data
```
