# Supplementary material for: Correction: Misregulation of AUXIN RESPONSE FACTOR 8 Underlies the Developmental Abnormalities Caused by Three Distinct Viral Silencing Suppressors in Arabidopsis
Source: PLoS Pathog. 2016 May 5;12(5):e1005627. doi: 10.1371/journal.ppat.1005627 (PMC4858414; doi:10.1371/journal.ppat.1005627)
Supplement: S8 Fig — The hygromycin selection gene present in the HcPro transgenic line was also assembled. (ZIP) [file ppat.1005627.s008.zip › contig 35S-HcPro.compressed.pdf]

20 40 60 80

35S promoter

p35S-HcPro-T35S transgene CTACTCCAAAAATGTCAAAGATACAGTCTCAGAAGACCAAAGGGCTATTGAGACTTTTCAACAAAGGGTAATTTCGGGAAACCTCCTCG

Consensus

Trace data

35S-HcPro-35S-For

Trace data

35S-HcPro-P1H-Pro_seq1F

Trace data

35S-HcPro-P1H-Pro_seq2F

Trace data

35S-HcPro-P1H-Pro_seq3R

Trace data

35S-HcPro-P1H-Pro_seq4R

Trace data

35S-HcPro-T35S-Rev

Trace data

100 120 140 160

p35S-HcPro-T35S transgene GATTCCATTGCCCAGCTATCTGTCACTTCATCGAAAGGACAGTAGAAAAGGAAGGTGGCTCCTACAAATGCCATCATTGCGATAAAGGA

Consensus

35S-HcPro-35S-For

Trace data

35S-HcPro-P1H-Pro_seq1F

Trace data

35S-HcPro-P1H-Pro_seq2F

Trace data

35S-HcPro-P1H-Pro_seq3R

Trace data

35S-HcPro-P1H-Pro_seq4R

Trace data

35S-HcPro-T35S-Rev

Trace data

	180 · 200 · 220 · 240 · 260
p35S-HcPro-T35S transgene	AAGGCTATCATTCAAGATGCCTCTGCCGACAGTGGTCCCAAAGATGGACCCCCACCCACGAGGAGCATCGTGGAAAAAGAAGACGTCCC
Consensus	
35S-HcPro-35S-For	
Trace data	
35S-HcPro-P1H-Pro_seq1F	
Trace data	
35S-HcPro-P1H-Pro_seq2F	
Trace data	
35S-HcPro-P1H-Pro_seq3R	
Trace data	
35S-HcPro-P1H-Pro_seq4R	
Trace data	
35S-HcPro-T35S-Rev	
Trace data	

	280 · 300 · 320 · 340 (p35S For)
p35S-HcPro-T35S transgene	AACCACGTCTTCAAAGCAAGTGGATTGATGTGACATCTCCACTGACGTAAGGGATGACGCACAATCCCACTATCCTTCGCAAGACCCTT
Consensus	
35S-HcPro-35S-For	CACATCCTCGCAGACCTTC
Trace data	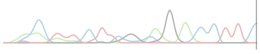
35S-HcPro-P1H-Pro_seq1F	
Trace data	
35S-HcPro-P1H-Pro_seq2F	
Trace data	
35S-HcPro-P1H-Pro_seq3R	
Trace data	
35S-HcPro-P1H-Pro_seq4R	
Trace data	
35S-HcPro-T35S-Rev	
Trace data	

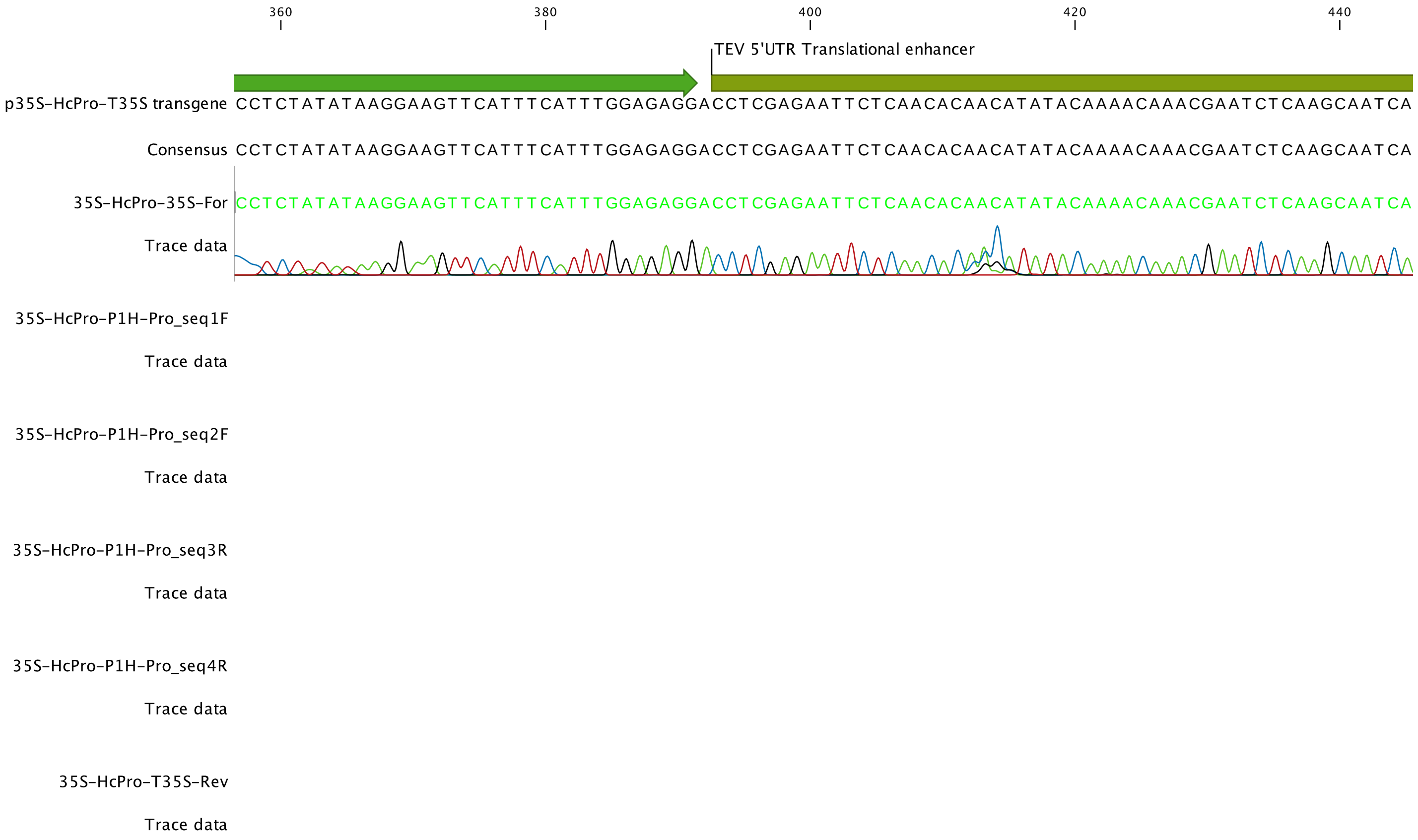
360
380
400
420
440
TEV 5'UTR Translational enhancer
p35S-HcPro-T35S transgene CCTCTATATAAGGAAGTTCATTTCATTTGGAGAGGACCTCGAGAATTCTCAACACAACATATACAAAACAAACGAATCTCAAGCAATCA
Consensus CCTCTATATAAGGAAGTTCATTTCATTTGGAGAGGACCTCGAGAATTCTCAACACAACATATACAAAACAAACGAATCTCAAGCAATCA
35S-HcPro-35S-For CCTCTATATAAGGAAGTTCATTTCATTTGGAGAGGACCTCGAGAATTCTCAACACAACATATACAAAACAAACGAATCTCAAGCAATCA
Trace data
35S-HcPro-P1H-Pro_seq1F
Trace data
35S-HcPro-P1H-Pro_seq2F
Trace data
35S-HcPro-P1H-Pro_seq3R
Trace data
35S-HcPro-P1H-Pro_seq4R
Trace data
35S-HcPro-T35S-Rev
Trace data

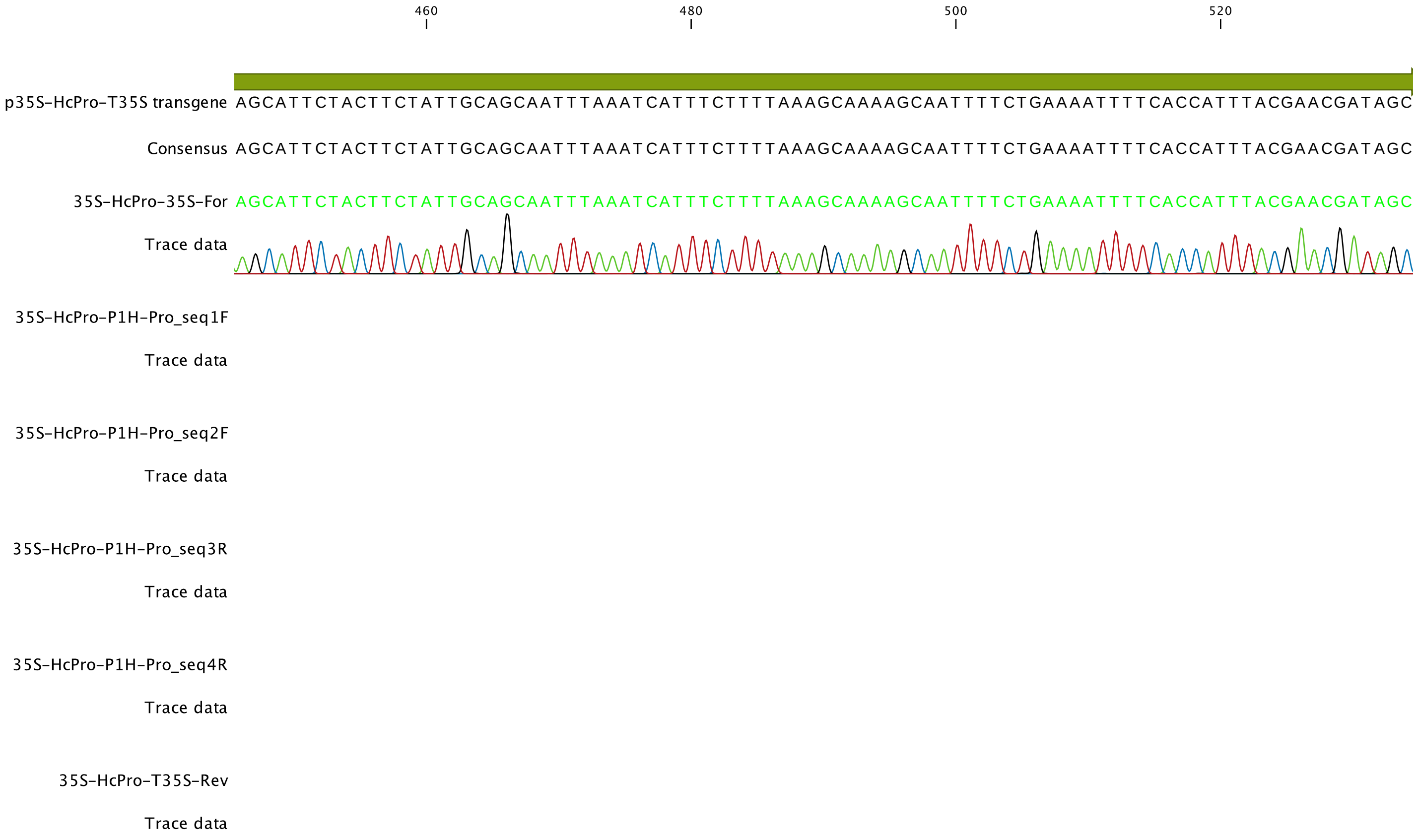
460
480
500
520
p35S-HcPro-T35S transgene AGCATTCTACTTCTATTGCAGCAATTTAAATCATTTCTTTTAAAGCAAAAGCAATTTTCTGAAAATTTTCACCATTTACGAACGATAGC
Consensus AGCATTCTACTTCTATTGCAGCAATTTAAATCATTTCTTTTAAAGCAAAAGCAATTTTCTGAAAATTTTCACCATTTACGAACGATAGC
35S-HcPro-35S-For AGCATTCTACTTCTATTGCAGCAATTTAAATCATTTCTTTTAAAGCAAAAGCAATTTTCTGAAAATTTTCACCATTTACGAACGATAGC
Trace data
35S-HcPro-P1H-Pro_seq1F
Trace data
35S-HcPro-P1H-Pro_seq2F
Trace data
35S-HcPro-P1H-Pro_seq3R
Trace data
35S-HcPro-P1H-Pro_seq4R
Trace data
35S-HcPro-T35S-Rev
Trace data

540 560 580 600 620

P1/HcPro

p35S-HcPro-T35S transgene CATGGCAGCAGTTACATTTGCAACCGCTATCACCAACGCCATCACCAGCAAACCAGCACTCACCGGAATGGTGCAGTTTGGGAGTTTCC

Consensus CATGGCAGCAGTTACATTTGCAACCGCTATCACCAACGCCATCACCAGCAAACCAGCACTCACCGGAATGGTGCAGTTTGGGAGTTTCC

35S-HcPro-35S-For CATGGCAGCAGTTACATTTGCAACCGCTATCACCAACGCCATCACCAGCAAACCAGCACTCACCGGAATGGTGCAGTTTGGGAGTTTCC

Trace data

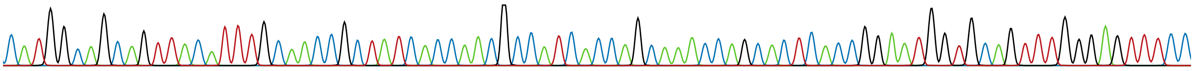

35S-HcPro-P1H-Pro_seq1F

Trace data

35S-HcPro-P1H-Pro_seq2F

Trace data

35S-HcPro-P1H-Pro_seq3R

Trace data

35S-HcPro-P1H-Pro_seq4R

Trace data

35S-HcPro-T35S-Rev

Trace data

640 660 680 700

p35S-HcPro-T35S transgene CACCAATGCCATTGCGATCCACCACCGTCACCACAGTCGCCACTTCAGTGGCGCAACCTAAACTGTACACAGTGCAGTTTGGAAGCCTT

Consensus CACCAATGCCATTGCGATCCACCACCGTCACCACAGTCGCCACTTCAGTGGCGCAACCTAAACTGTACACAGTGCAGTTTGGAAGCCTT

35S-HcPro-35S-For CACCAATGCCATTGCGATCCACCACCGTCACCACAGTCGCCACTTCAGTGGCGCAACCTAAACTGTACACAGTGCAGTTTGGAAGCCTT

Trace data

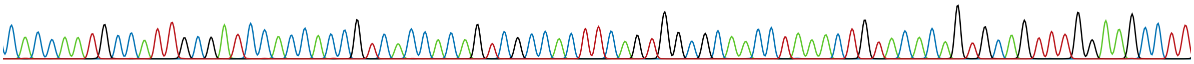

35S-HcPro-P1H-Pro_seq1F

Trace data

35S-HcPro-P1H-Pro_seq2F

Trace data

35S-HcPro-P1H-Pro_seq3R

Trace data

35S-HcPro-P1H-Pro_seq4R

Trace data

35S-HcPro-T35S-Rev

Trace data

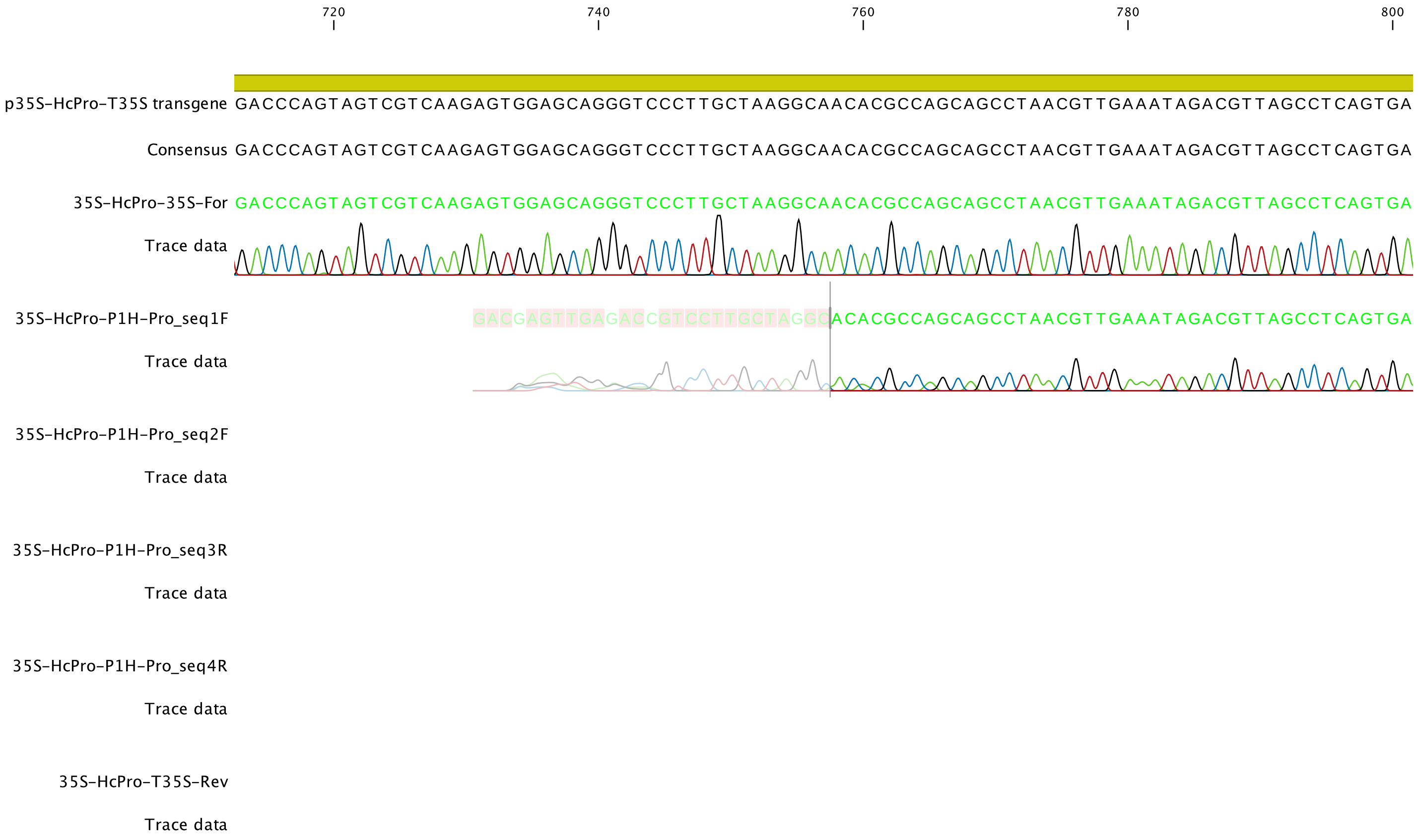
720
740
760
780
800
p35S-HcPro-T35S transgene GACCCAGTAGTCGTCAAGAGTGGAGCAGGGTCCCTTGCTAAGGCAACACGCCAGCAGCCTAACGTTGAAATAGACGTTAGCCTCAGTGA
Consensus GACCCAGTAGTCGTCAAGAGTGGAGCAGGGTCCCTTGCTAAGGCAACACGCCAGCAGCCTAACGTTGAAATAGACGTTAGCCTCAGTGA
35S-HcPro-35S-For GACCCAGTAGTCGTCAAGAGTGGAGCAGGGTCCCTTGCTAAGGCAACACGCCAGCAGCCTAACGTTGAAATAGACGTTAGCCTCAGTGA
Trace data
35S-HcPro-P1H-Pro_seq1F GACGAGTTGAGACCGTCCTTGCTAGGCACACGCCAGCAGCCTAACGTTGAAATAGACGTTAGCCTCAGTGA
Trace data
35S-HcPro-P1H-Pro_seq2F
Trace data
35S-HcPro-P1H-Pro_seq3R
Trace data
35S-HcPro-P1H-Pro_seq4R
Trace data
35S-HcPro-T35S-Rev
Trace data

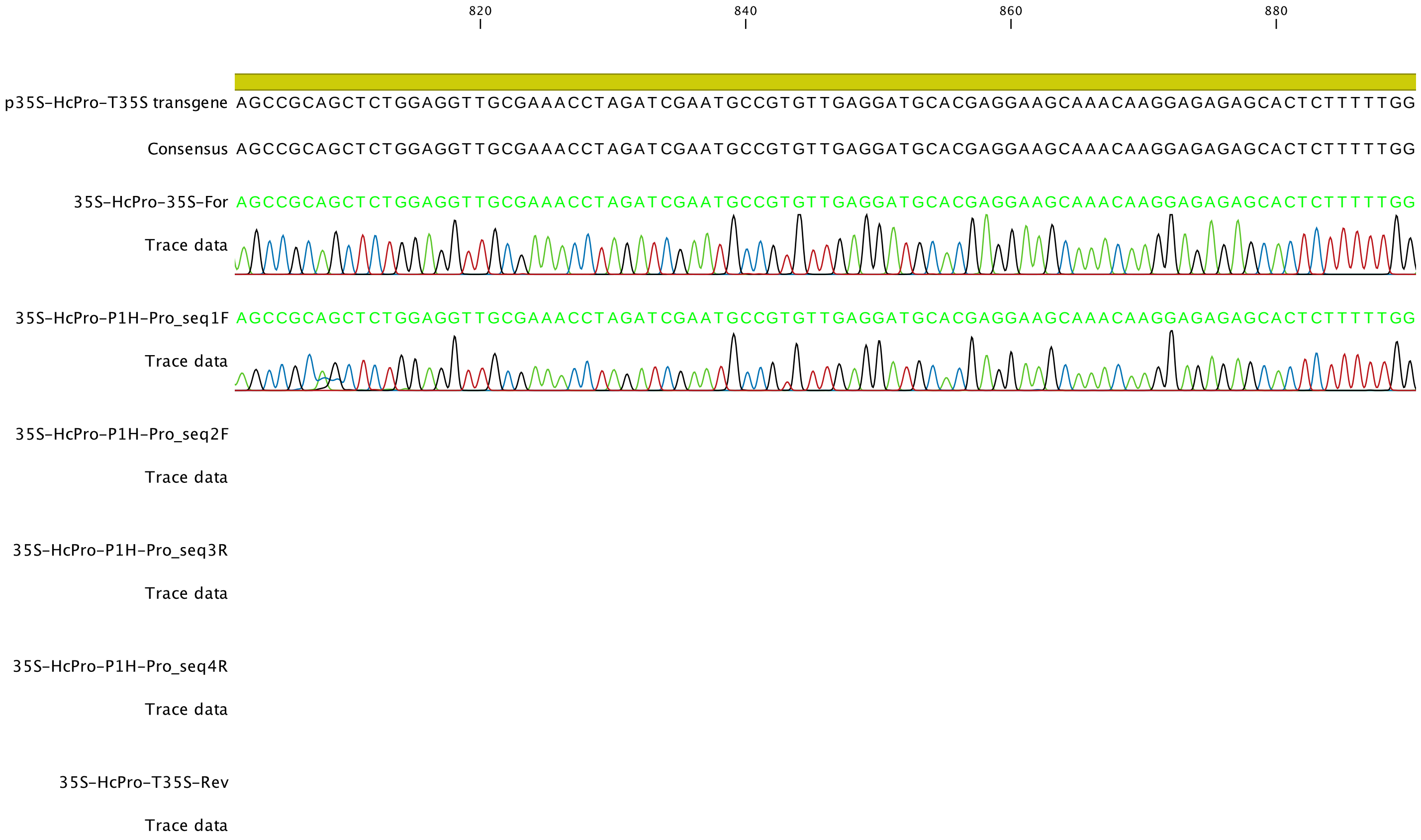
820
840
860
880
p35S-HcPro-T35S transgene AGCCGCAGCTCTGGAGGTTGCGAAACCTAGATCGAATGCCGTGTTGAGGATGCACGAGGAAGCAAACAAGGAGAGAGCACTCTTTTTGG
Consensus AGCCGCAGCTCTGGAGGTTGCGAAACCTAGATCGAATGCCGTGTTGAGGATGCACGAGGAAGCAAACAAGGAGAGAGCACTCTTTTTGG
35S-HcPro-35S-For AGCCGCAGCTCTGGAGGTTGCGAAACCTAGATCGAATGCCGTGTTGAGGATGCACGAGGAAGCAAACAAGGAGAGAGCACTCTTTTTGG
Trace data
35S-HcPro-P1H-Pro_seq1F AGCCGCAGCTCTGGAGGTTGCGAAACCTAGATCGAATGCCGTGTTGAGGATGCACGAGGAAGCAAACAAGGAGAGAGCACTCTTTTTGG
Trace data
35S-HcPro-P1H-Pro_seq2F
Trace data
35S-HcPro-P1H-Pro_seq3R
Trace data
35S-HcPro-P1H-Pro_seq4R
Trace data
35S-HcPro-T35S-Rev
Trace data

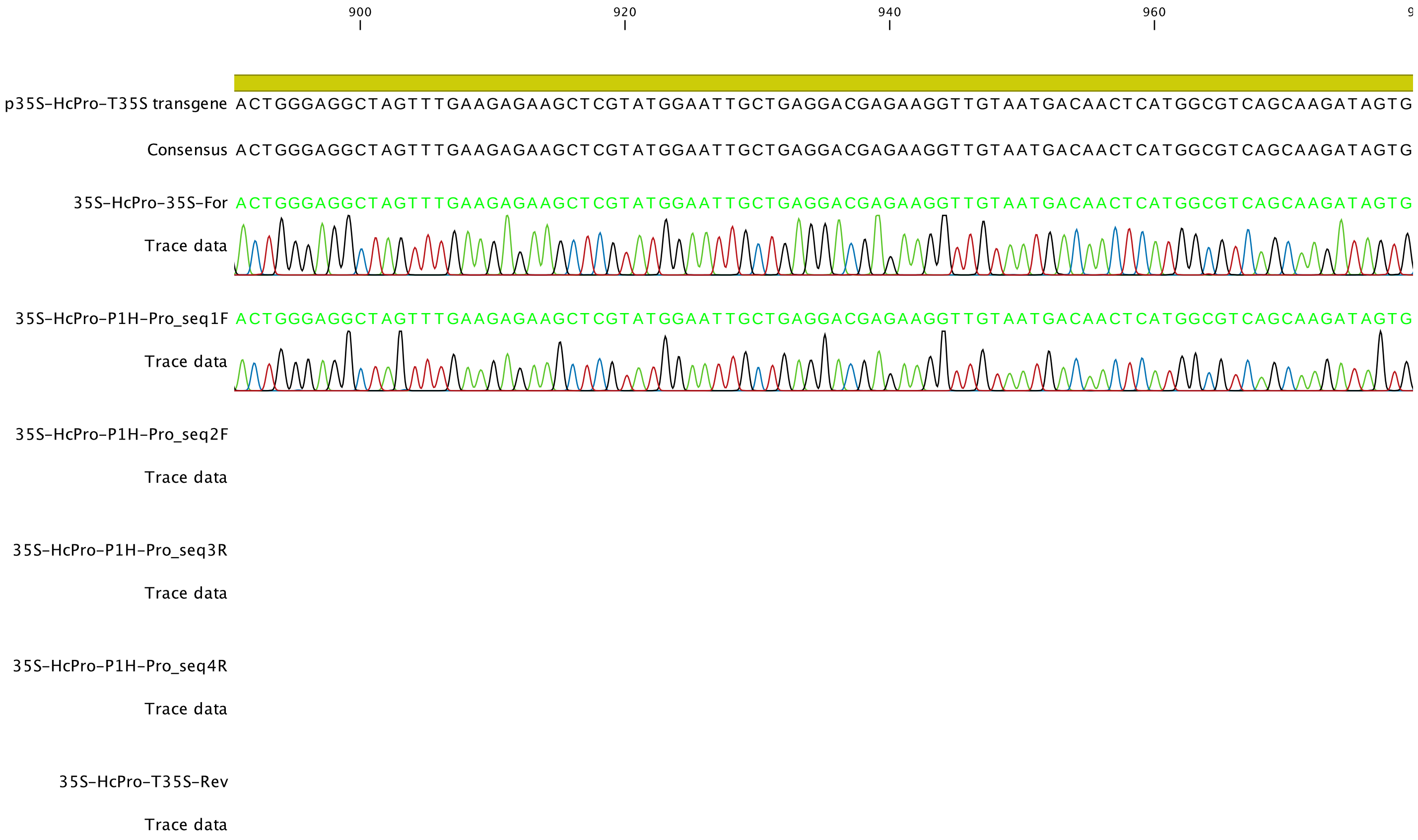
900
920
940
960
9
p35S-HcPro-T35S transgene ACTGGGAGGCTAGTTTGAAGAGAAGCTCGTATGGAATTGCTGAGGACGAGAAGGTTGTAATGACAACTCATGGCGTCAGCAAGATAGTG
Consensus ACTGGGAGGCTAGTTTGAAGAGAAGCTCGTATGGAATTGCTGAGGACGAGAAGGTTGTAATGACAACTCATGGCGTCAGCAAGATAGTG
35S-HcPro-35S-For ACTGGGAGGCTAGTTTGAAGAGAAGCTCGTATGGAATTGCTGAGGACGAGAAGGTTGTAATGACAACTCATGGCGTCAGCAAGATAGTG
Trace data
35S-HcPro-P1H-Pro_seq1F ACTGGGAGGCTAGTTTGAAGAGAAGCTCGTATGGAATTGCTGAGGACGAGAAGGTTGTAATGACAACTCATGGCGTCAGCAAGATAGTG
Trace data
35S-HcPro-P1H-Pro_seq2F
Trace data
35S-HcPro-P1H-Pro_seq3R
Trace data
35S-HcPro-P1H-Pro_seq4R
Trace data
35S-HcPro-T35S-Rev
Trace data

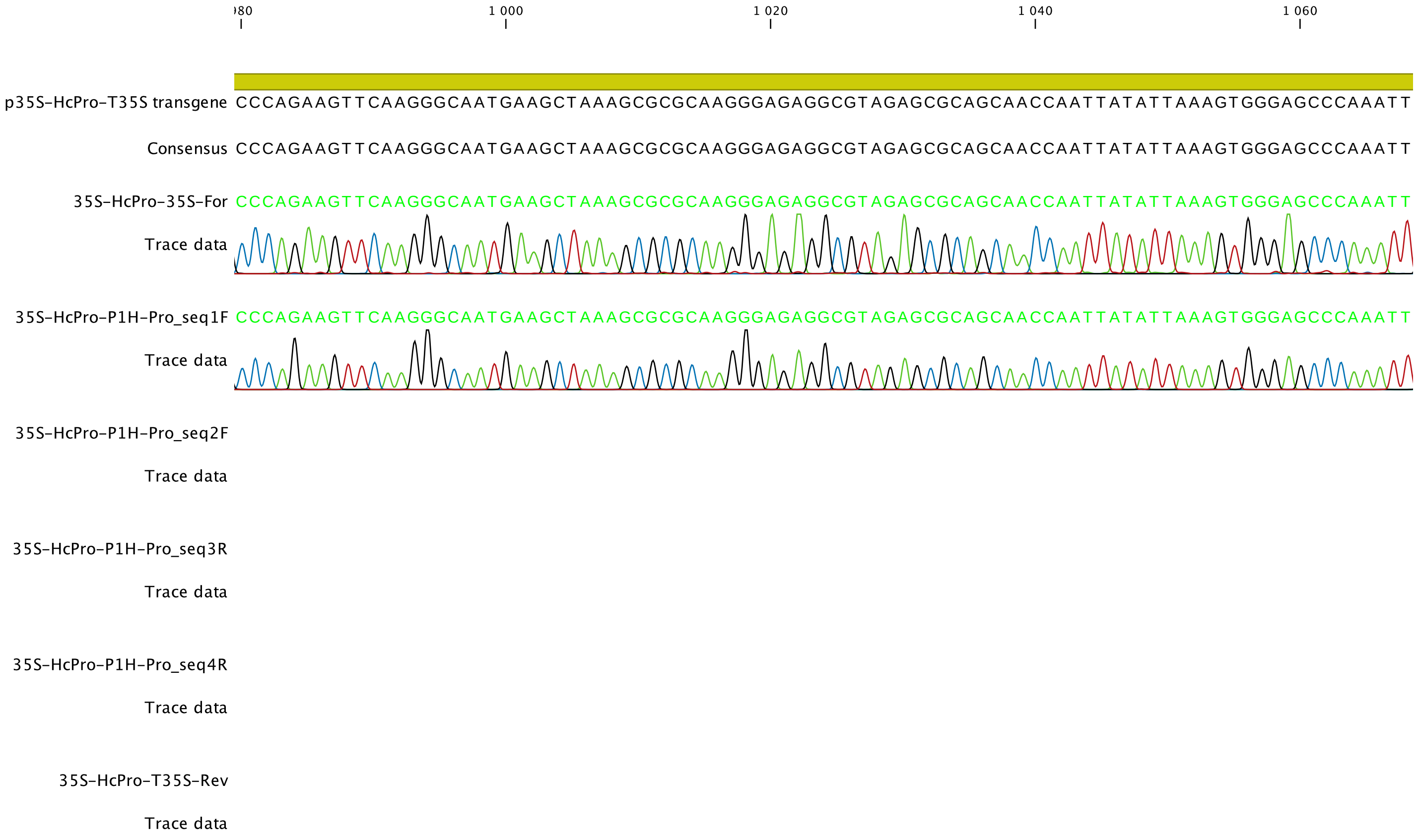
)80
1 000
1 020
1 040
1 060
p35S-HcPro-T35S transgene CCCAGAAGTTCAAGGGCAATGAAGCTAAAGCGCGCAAGGGAGAGGCGTAGAGCGCAGCAACCAATTATATTAAAGTGGGAGCCCAAATT
Consensus CCCAGAAGTTCAAGGGCAATGAAGCTAAAGCGCGCAAGGGAGAGGCGTAGAGCGCAGCAACCAATTATATTAAAGTGGGAGCCCAAATT
35S-HcPro-35S-For CCCAGAAGTTCAAGGGCAATGAAGCTAAAGCGCGCAAGGGAGAGGCGTAGAGCGCAGCAACCAATTATATTAAAGTGGGAGCCCAAATT
Trace data
35S-HcPro-P1H-Pro_seq1F CCCAGAAGTTCAAGGGCAATGAAGCTAAAGCGCGCAAGGGAGAGGCGTAGAGCGCAGCAACCAATTATATTAAAGTGGGAGCCCAAATT
Trace data
35S-HcPro-P1H-Pro_seq2F
Trace data
35S-HcPro-P1H-Pro_seq3R
Trace data
35S-HcPro-P1H-Pro_seq4R
Trace data
35S-HcPro-T35S-Rev
Trace data

1 080 1 100 1 120 1 140

p35S-HcPro-T35S transgene GAGCGGGATCTCAATCGGAGGAGGGCTCTCTGCGAGCGTAATCGAAGCAGAAGAGGTTCGCACAAAGTGGCCGCTTCATAAGACACCGT

Consensus GAGCGGGATCTCAATCGGAGGAGGGCTCTCTGCGAGCGTAATCGAAGCAGAAGAGGTTCGCACAAAGTGGCCGCTTCATAAGACACCGT

35S-HcPro-35S-For GAGCGGGATCTCAATCGGAGGAGGGCTCTCTGCGAGCGTAATCGAAGCAGAAGAGGTTCGCACAAAGTGGCCGCTTCATAAGACACCGT

Trace data 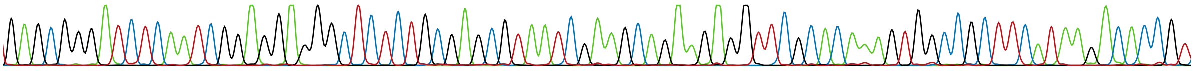

35S-HcPro-P1H-Pro_seq1F GAGCGGGATCTCAATCGGAGGAGGGCTCTCTGCGAGCGTAATCGAAGCAGAAGAGGTTCGCACAAAGTGGCCGCTTCATAAGACACCGT

Trace data 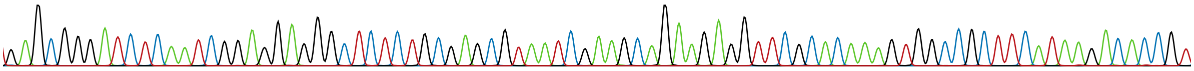

35S-HcPro-P1H-Pro_seq2F

Trace data

35S-HcPro-P1H-Pro_seq3R

Trace data

35S-HcPro-P1H-Pro_seq4R

Trace data

35S-HcPro-T35S-Rev

Trace data

1 160 1 180 1 200 1 220 1 240

p35S-HcPro-T35S transgene CAATGAAGAAGAGGACGGTGCACAGAATATGCAAGATGAACGACCAAGGAGTTGACATGTTGACACGATCCCTGGTTAAGATTTTCAAG

Consensus CAATGAAGAAGAGGACGGTGCACAGAATATGCAAGATGAACGACCAAGGAGTTGACATGTTGACACGATCCCTGGTTAAGATTTTCAAG

35S-HcPro-35S-For CAATGAAGAAGAGGACGGTGCACAGAATATGCAAGATGAACGACCAAGGAGTTGACATGTTGACACGATCCCTGGTTAAGATTTTCAAG

Trace data 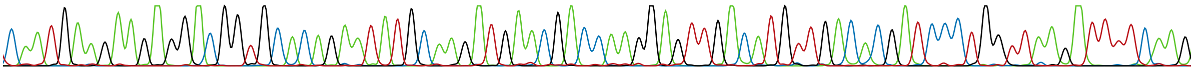

35S-HcPro-P1H-Pro_seq1F CAATGAAGAAGAGGACGGTGCACAGAATATGCAAGATGAACGACCAAGGAGTTGACATGTTGACACGATCCCTGGTTAAGATTTTCAAG

Trace data 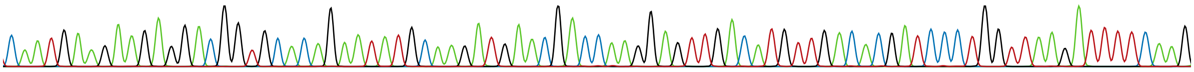

35S-HcPro-P1H-Pro_seq2F

Trace data

35S-HcPro-P1H-Pro_seq3R

Trace data

35S-HcPro-P1H-Pro_seq4R

Trace data

35S-HcPro-T35S-Rev

Trace data

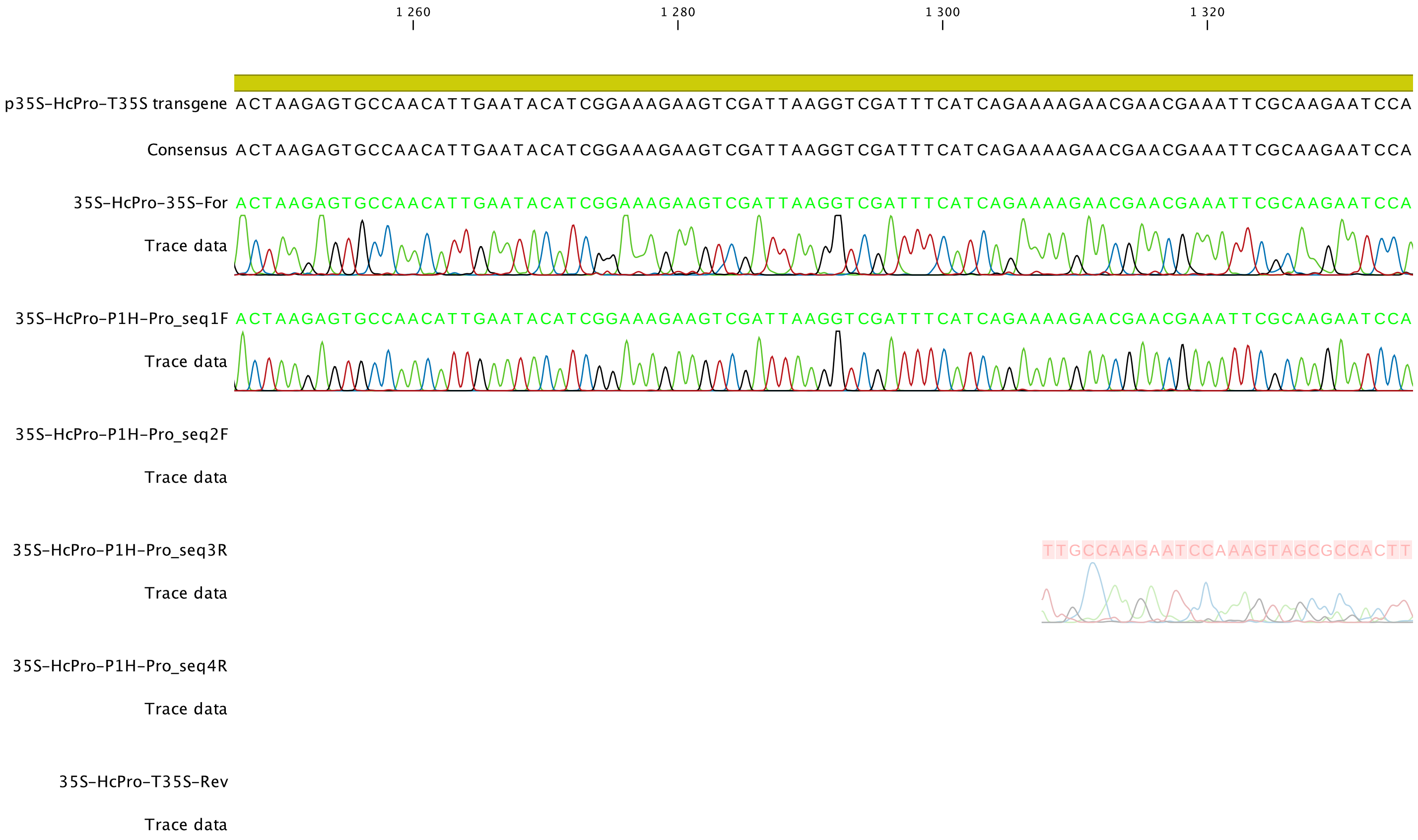
1 260
1 280
1 300
1 320
p35S-HcPro-T35S transgene ACTAAGAGTGCCAACATTGAATACATCGGAAAGAAGTCGATTAAGGTCGATTTCATCAGAAAAGAACGAACGAAATTCGCAAGAATCCA
Consensus ACTAAGAGTGCCAACATTGAATACATCGGAAAGAAGTCGATTAAGGTCGATTTCATCAGAAAAGAACGAACGAAATTCGCAAGAATCCA
35S-HcPro-35S-For ACTAAGAGTGCCAACATTGAATACATCGGAAAGAAGTCGATTAAGGTCGATTTCATCAGAAAAGAACGAACGAAATTCGCAAGAATCCA
Trace data
35S-HcPro-P1H-Pro_seq1F ACTAAGAGTGCCAACATTGAATACATCGGAAAGAAGTCGATTAAGGTCGATTTCATCAGAAAAGAACGAACGAAATTCGCAAGAATCCA
Trace data
35S-HcPro-P1H-Pro_seq2F
Trace data
35S-HcPro-P1H-Pro_seq3R
Trace data
35S-HcPro-P1H-Pro_seq4R
Trace data
35S-HcPro-T35S-Rev
Trace data

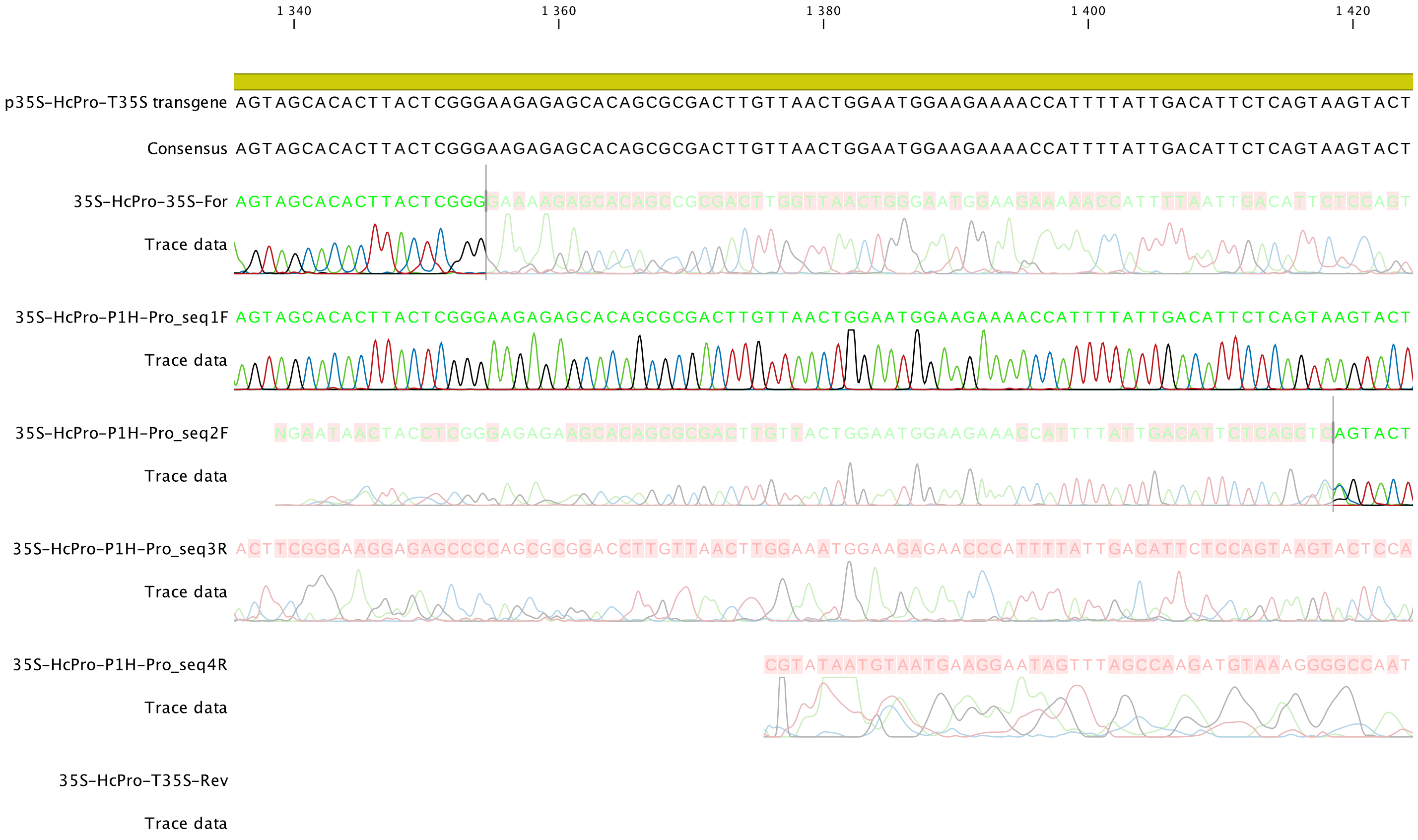
1 340
1 360
1 380
1 400
1 420
p35S-HcPro-T35S transgene AGTAGCACACTTACTCGGGAAGAGAGCACAGCGCGACTTGTTAACTGGAATGGAAGAAAACCATTTTATTGACATTCTCAGTAAGTACT
Consensus AGTAGCACACTTACTCGGGAAGAGAGCACAGCGCGACTTGTTAACTGGAATGGAAGAAAACCATTTTATTGACATTCTCAGTAAGTACT
35S-HcPro-35S-For AGTAGCACACTTACTCGGG
Trace data
35S-HcPro-P1H-Pro_seq1F AGTAGCACACTTACTCGGGAAGAGAGCACAGCGCGACTTGTTAACTGGAATGGAAGAAAACCATTTTATTGACATTCTCAGTAAGTACT
Trace data
35S-HcPro-P1H-Pro_seq2F AGTACT
Trace data
35S-HcPro-P1H-Pro_seq3R
Trace data
35S-HcPro-P1H-Pro_seq4R
Trace data
35S-HcPro-T35S-Rev
Trace data

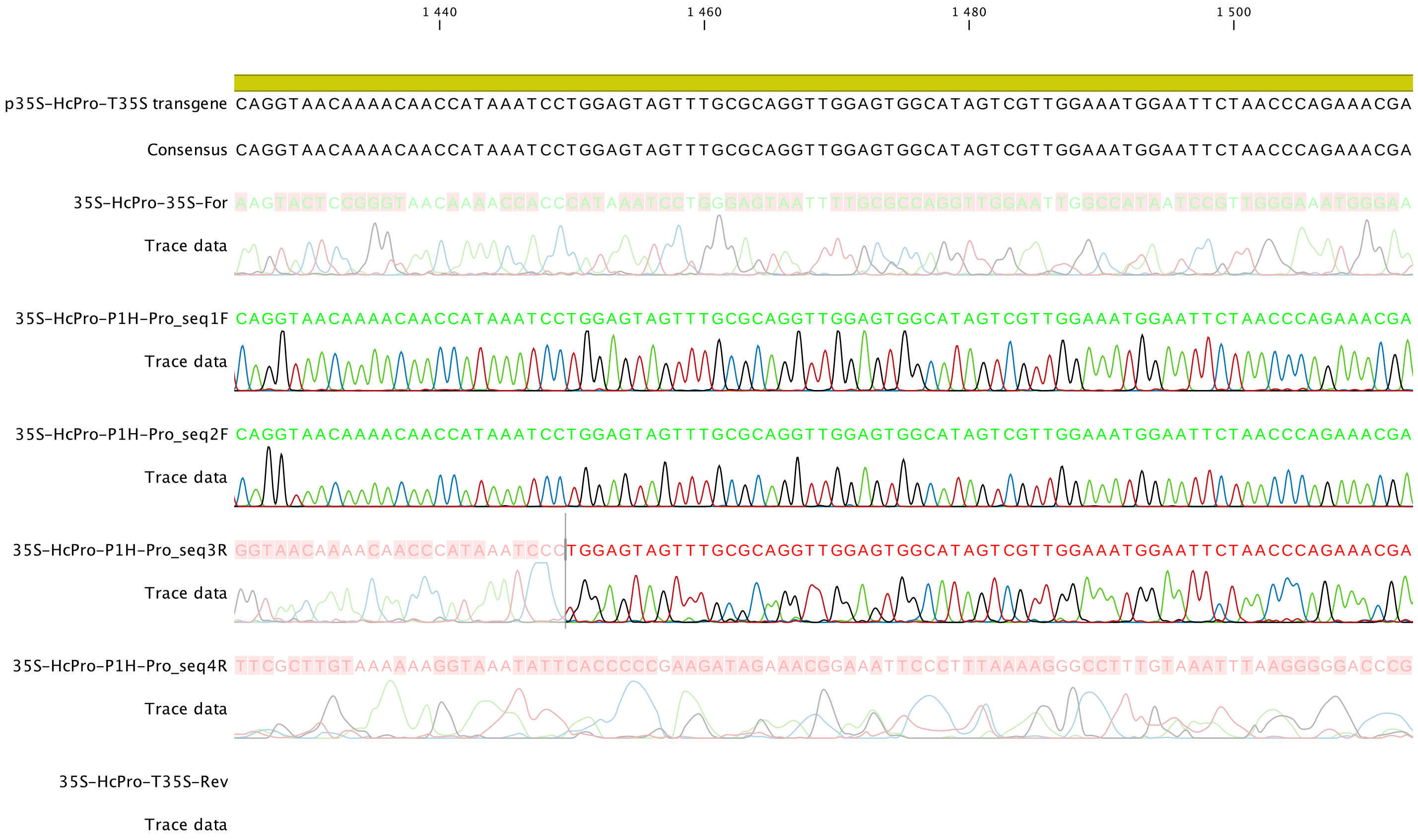

1 440
1 460
1 480
1 500
p35S-HcPro-T35S transgene CAGGTAACAAAACAACCATAAATCCTGGAGTAGTTTGCGCAGGTTGGAGTGGCATAGTCGTTGGAAATGGAATTCTAACCCAGAAACGA
Consensus CAGGTAACAAAACAACCATAAATCCTGGAGTAGTTTGCGCAGGTTGGAGTGGCATAGTCGTTGGAAATGGAATTCTAACCCAGAAACGA
35S-HcPro-35S-For AAGTACTCGGGTAACAAAACCACCCATAAATCCTGGGAGTAATTTTGGCGCAGGTTGGAATTGGCCATAATCCGTTGGGAAATGGGAA
Trace data
35S-HcPro-P1H-Pro_seq1F CAGGTAACAAAACAACCATAAATCCTGGAGTAGTTTGCGCAGGTTGGAGTGGCATAGTCGTTGGAAATGGAATTCTAACCCAGAAACGA
Trace data
35S-HcPro-P1H-Pro_seq2F CAGGTAACAAAACAACCATAAATCCTGGAGTAGTTTGCGCAGGTTGGAGTGGCATAGTCGTTGGAAATGGAATTCTAACCCAGAAACGA
Trace data
35S-HcPro-P1H-Pro_seq3R GGTAACAAAACAACCCATAAATCCCTGGAGTAGTTTGCGCAGGTTGGAGTGGCATAGTCGTTGGAAATGGAATTCTAACCCAGAAACGA
Trace data
35S-HcPro-P1H-Pro_seq4R TTCGCTTGTAAAAAAGGTAAATATTCACCCCCGAAGATAGAAACGGAAATTCCCTTTAAAAGGGCCTTTGTAAATTTAAGGGGGACCCG
Trace data
35S-HcPro-T35S-Rev
Trace data

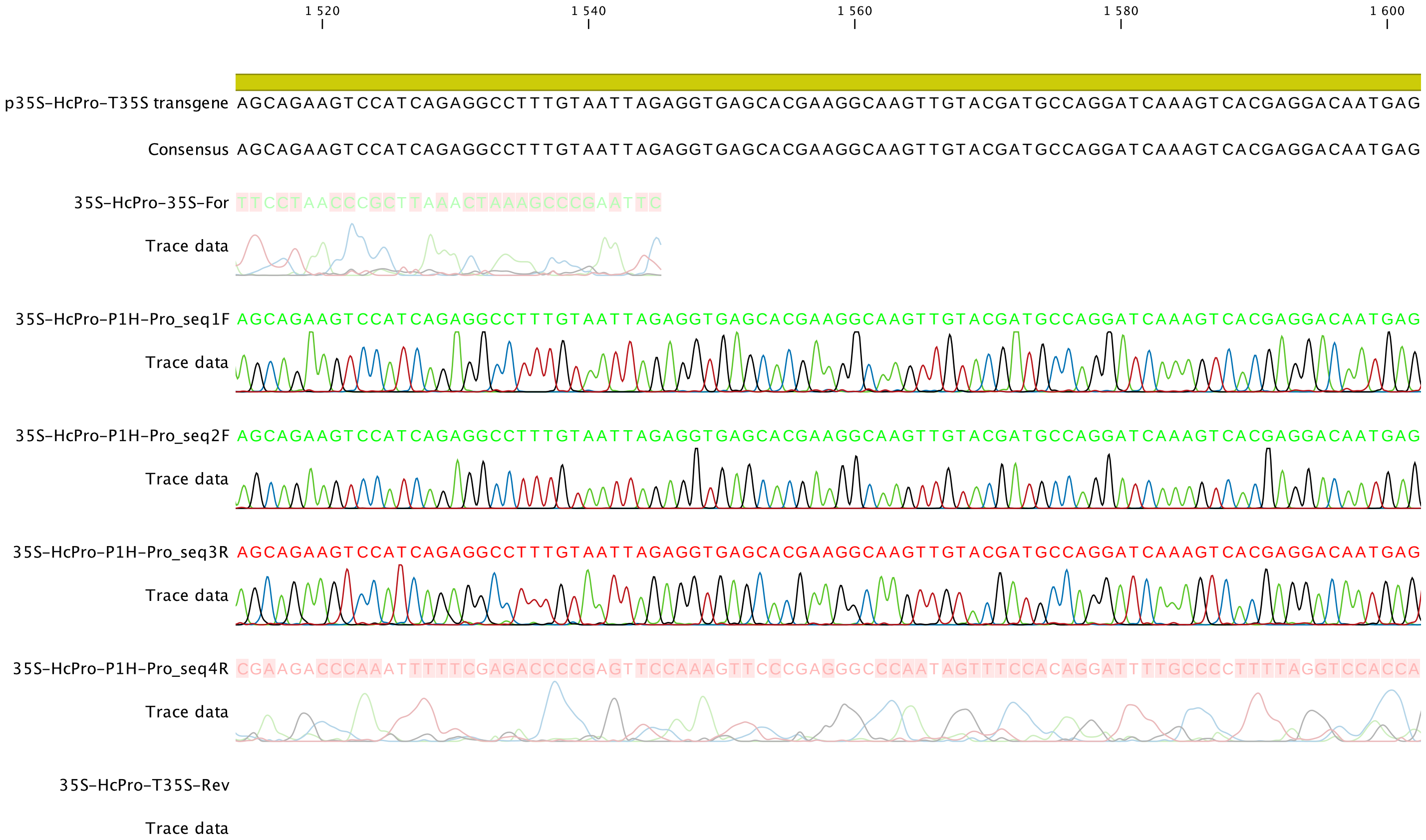

1 520
1 540
1 560
1 580
1 600
p35S-HcPro-T35S transgene AGCAGAAGTCCATCAGAGGCCTTTGTAATTAGAGGTGAGCACGAAGGCAAGTTGTACGATGCCAGGATCAAAGTCACGAGGACAATGAG
Consensus AGCAGAAGTCCATCAGAGGCCTTTGTAATTAGAGGTGAGCACGAAGGCAAGTTGTACGATGCCAGGATCAAAGTCACGAGGACAATGAG
35S-HcPro-35S-For TTCGTAACCCGCTTAAACTAAAGCGCGAATTC
Trace data
35S-HcPro-P1H-Pro_seq1F AGCAGAAGTCCATCAGAGGCCTTTGTAATTAGAGGTGAGCACGAAGGCAAGTTGTACGATGCCAGGATCAAAGTCACGAGGACAATGAG
Trace data
35S-HcPro-P1H-Pro_seq2F AGCAGAAGTCCATCAGAGGCCTTTGTAATTAGAGGTGAGCACGAAGGCAAGTTGTACGATGCCAGGATCAAAGTCACGAGGACAATGAG
Trace data
35S-HcPro-P1H-Pro_seq3R AGCAGAAGTCCATCAGAGGCCTTTGTAATTAGAGGTGAGCACGAAGGCAAGTTGTACGATGCCAGGATCAAAGTCACGAGGACAATGAG
Trace data
35S-HcPro-P1H-Pro_seq4R CGAAGACCCAAATTTTTCGAGACCCCGAGTTCCAAAGTTCCCGAGGGCCCAATAGTTTCCACAGGATTTTGCCCCTTTTAGGTCCACCA
Trace data
35S-HcPro-T35S-Rev
Trace data

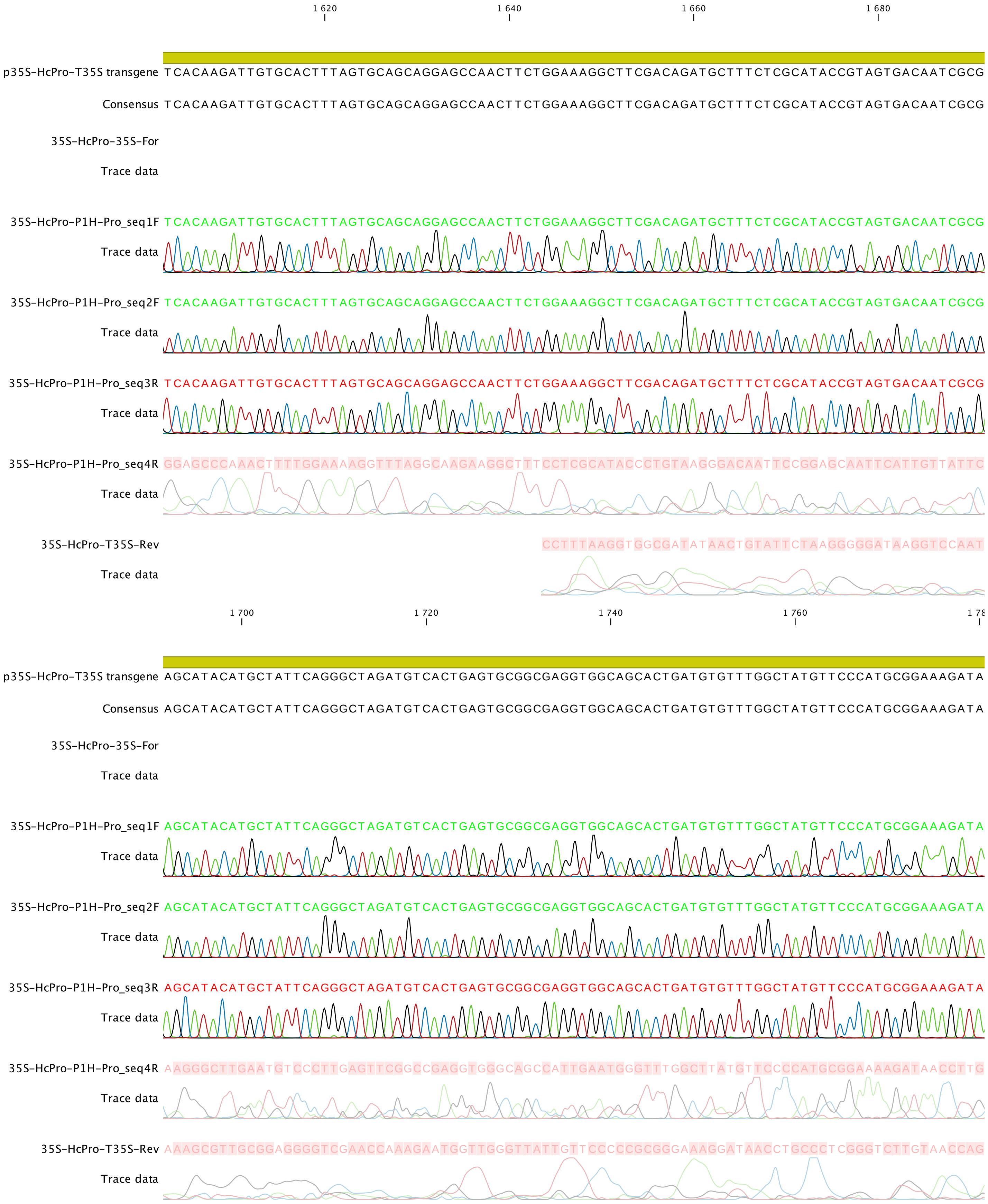

1 620
1 640
1 660
1 680
p35S-HcPro-T35S transgene TCACAAGATTGTGCACTTTAGTGCAGCAGGAGCCAACTTCTGGAAAGGCTTCGACAGATGCTTTCTCGCATACCGTAGTGACAATCGCG
Consensus TCACAAGATTGTGCACTTTAGTGCAGCAGGAGCCAACTTCTGGAAAGGCTTCGACAGATGCTTTCTCGCATACCGTAGTGACAATCGCG
35S-HcPro-35S-For
Trace data
35S-HcPro-P1H-Pro_seq1F TCACAAGATTGTGCACTTTAGTGCAGCAGGAGCCAACTTCTGGAAAGGCTTCGACAGATGCTTTCTCGCATACCGTAGTGACAATCGCG
Trace data
35S-HcPro-P1H-Pro_seq2F TCACAAGATTGTGCACTTTAGTGCAGCAGGAGCCAACTTCTGGAAAGGCTTCGACAGATGCTTTCTCGCATACCGTAGTGACAATCGCG
Trace data
35S-HcPro-P1H-Pro_seq3R TCACAAGATTGTGCACTTTAGTGCAGCAGGAGCCAACTTCTGGAAAGGCTTCGACAGATGCTTTCTCGCATACCGTAGTGACAATCGCG
Trace data
35S-HcPro-P1H-Pro_seq4R
Trace data
35S-HcPro-T35S-Rev
Trace data
1 700
1 720
1 740
1 760
p35S-HcPro-T35S transgene AGCATACATGCTATTCAGGGCTAGATGTCACTGAGTGCGGCGAGGTGGCAGCACTGATGTGTTTGGCTATGTTCCCATGCGGAAAGATA
Consensus AGCATACATGCTATTCAGGGCTAGATGTCACTGAGTGCGGCGAGGTGGCAGCACTGATGTGTTTGGCTATGTTCCCATGCGGAAAGATA
35S-HcPro-35S-For
Trace data
35S-HcPro-P1H-Pro_seq1F AGCATACATGCTATTCAGGGCTAGATGTCACTGAGTGCGGCGAGGTGGCAGCACTGATGTGTTTGGCTATGTTCCCATGCGGAAAGATA
Trace data
35S-HcPro-P1H-Pro_seq2F AGCATACATGCTATTCAGGGCTAGATGTCACTGAGTGCGGCGAGGTGGCAGCACTGATGTGTTTGGCTATGTTCCCATGCGGAAAGATA
Trace data
35S-HcPro-P1H-Pro_seq3R AGCATACATGCTATTCAGGGCTAGATGTCACTGAGTGCGGCGAGGTGGCAGCACTGATGTGTTTGGCTATGTTCCCATGCGGAAAGATA
Trace data
35S-HcPro-P1H-Pro_seq4R
Trace data
35S-HcPro-T35S-Rev
Trace data

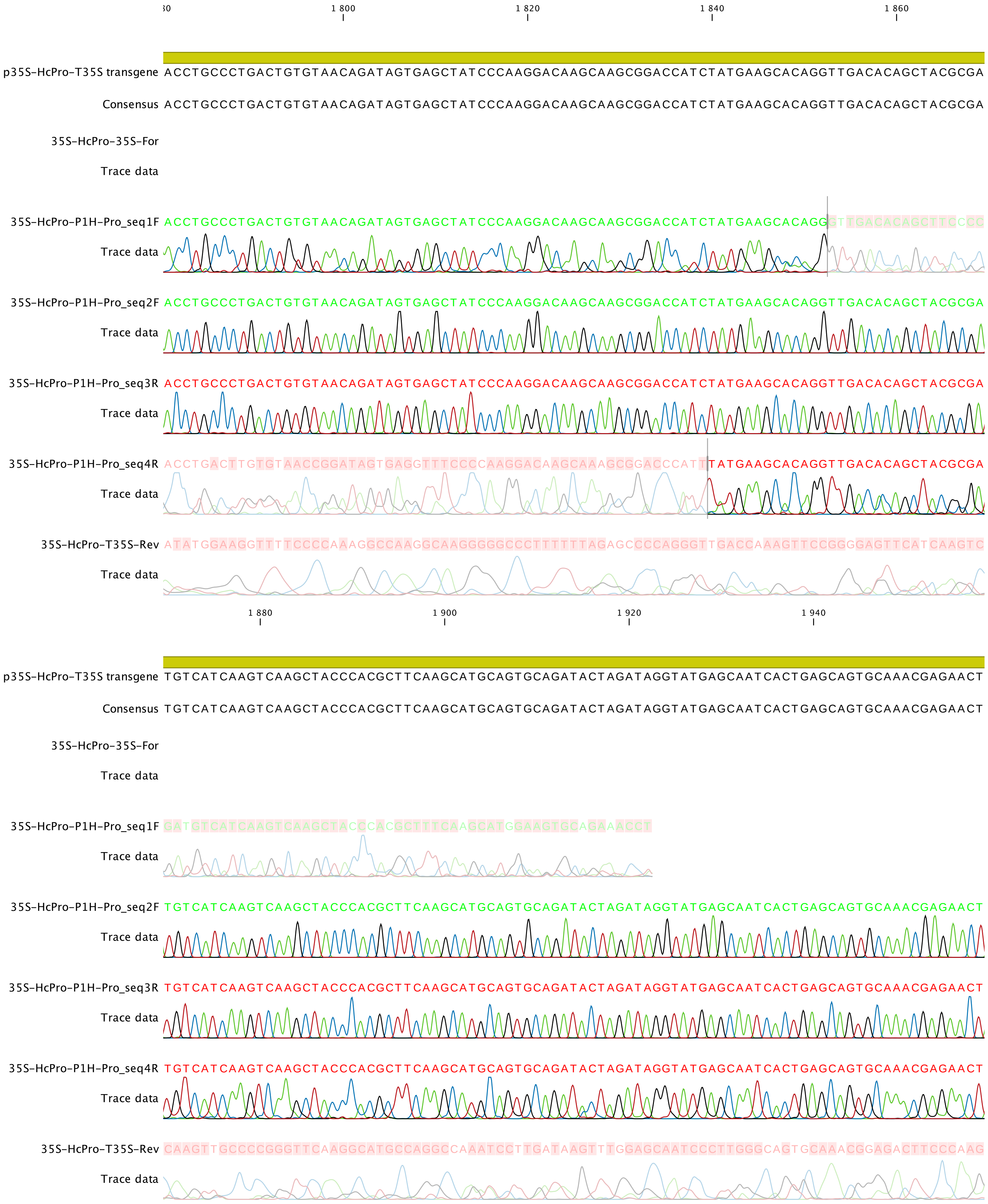
1 800
1 820
1 840
1 860
p35S-HcPro-T35S transgene ACCTGCCCTGACTGTGTAACAGATAGTGAGCTATCCCAAGGACAAGCAAGCGGACCATCTATGAAGCACAGGTTGACACAGCTACGCGA
Consensus ACCTGCCCTGACTGTGTAACAGATAGTGAGCTATCCCAAGGACAAGCAAGCGGACCATCTATGAAGCACAGGTTGACACAGCTACGCGA
35S-HcPro-35S-For
Trace data
35S-HcPro-P1H-Pro_seq1F ACCTGCCCTGACTGTGTAACAGATAGTGAGCTATCCCAAGGACAAGCAAGCGGACCATCTATGAAGCACAGGGTTGACACAGCTTCCCC
Trace data
35S-HcPro-P1H-Pro_seq2F ACCTGCCCTGACTGTGTAACAGATAGTGAGCTATCCCAAGGACAAGCAAGCGGACCATCTATGAAGCACAGGTTGACACAGCTACGCGA
Trace data
35S-HcPro-P1H-Pro_seq3R ACCTGCCCTGACTGTGTAACAGATAGTGAGCTATCCCAAGGACAAGCAAGCGGACCATCTATGAAGCACAGGTTGACACAGCTACGCGA
Trace data
35S-HcPro-P1H-Pro_seq4R ACCTGACTTGTGTAACCGGATAGTGAGGTTTCCCCAAGGACAAGCAAAGCGGACCCATTTATGAAGCACAGGTTGACACAGCTACGCGA
Trace data
35S-HcPro-T35S-Rev ATATGGAAGGTTTTCCCCAAAGGCCAAGGCAAGGGGGCCCTTTTTTAGAGCCCCAGGGTTGACCAAAGTTCCGGGGAGTTCATCAAGTC
Trace data
1 880
1 900
1 920
1 940
p35S-HcPro-T35S transgene TGTCATCAAGTCAAGCTACCCACGCTTCAAGCATGCAGTGCAGATACTAGATAGGTATGAGCAATCACTGAGCAGTGCAAACGAGAACT
Consensus TGTCATCAAGTCAAGCTACCCACGCTTCAAGCATGCAGTGCAGATACTAGATAGGTATGAGCAATCACTGAGCAGTGCAAACGAGAACT
35S-HcPro-35S-For
Trace data
35S-HcPro-P1H-Pro_seq1F GATGTCATCAAGTCAAGCTACCCACGCTTTCAAGCATGGAAGTGCAGAAACCT
Trace data
35S-HcPro-P1H-Pro_seq2F TGTCATCAAGTCAAGCTACCCACGCTTCAAGCATGCAGTGCAGATACTAGATAGGTATGAGCAATCACTGAGCAGTGCAAACGAGAACT
Trace data
35S-HcPro-P1H-Pro_seq3R TGTCATCAAGTCAAGCTACCCACGCTTCAAGCATGCAGTGCAGATACTAGATAGGTATGAGCAATCACTGAGCAGTGCAAACGAGAACT
Trace data
35S-HcPro-P1H-Pro_seq4R TGTCATCAAGTCAAGCTACCCACGCTTCAAGCATGCAGTGCAGATACTAGATAGGTATGAGCAATCACTGAGCAGTGCAAACGAGAACT
Trace data
35S-HcPro-T35S-Rev CAAGTTGCCCCGGGTTCAAGGCATGCCAGGCCAAATCCTTGATAAGTTTGGAGCAATCCCTTGGGCAGTGCAAACGGAGACTTCCCAAG
Trace data

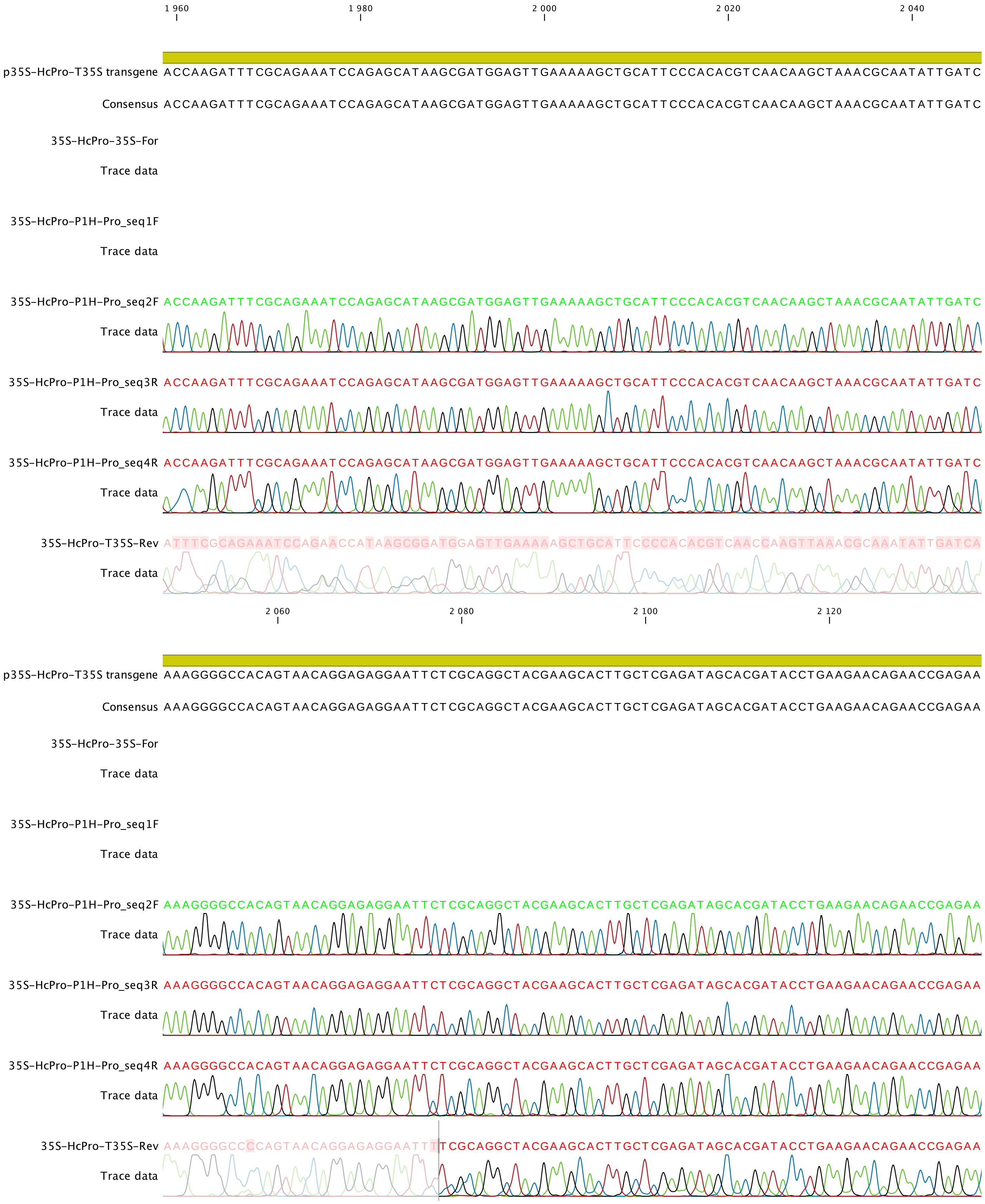
1 960
1 980
2 000
2 020
2 040
p35S-HcPro-T35S transgene ACCAAGATTTCGCAGAAATCCAGAGCATAAGCGATGGAGTTGAAAAAGCTGCATTCCCACACGTCAACAAGCTAAACGCAATATTGATC
Consensus ACCAAGATTTCGCAGAAATCCAGAGCATAAGCGATGGAGTTGAAAAAGCTGCATTCCCACACGTCAACAAGCTAAACGCAATATTGATC
35S-HcPro-35S-For
Trace data
35S-HcPro-P1H-Pro_seq1F
Trace data
35S-HcPro-P1H-Pro_seq2F ACCAAGATTTCGCAGAAATCCAGAGCATAAGCGATGGAGTTGAAAAAGCTGCATTCCCACACGTCAACAAGCTAAACGCAATATTGATC
Trace data
35S-HcPro-P1H-Pro_seq3R ACCAAGATTTCGCAGAAATCCAGAGCATAAGCGATGGAGTTGAAAAAGCTGCATTCCCACACGTCAACAAGCTAAACGCAATATTGATC
Trace data
35S-HcPro-P1H-Pro_seq4R ACCAAGATTTCGCAGAAATCCAGAGCATAAGCGATGGAGTTGAAAAAGCTGCATTCCCACACGTCAACAAGCTAAACGCAATATTGATC
Trace data
35S-HcPro-T35S-Rev ATTTCGCAGAAATCCAGAACCATAAGCGGATGGAGTTGAAAAAGCTGCATTCCCCACACGTCAACCAAGTTAAACGCAAATATTGATCA
Trace data
2 060
2 080
2 100
2 120
p35S-HcPro-T35S transgene AAAGGGGCCACAGTAACAGGAGAGGAATTCTCGCAGGCTACGAAGCACTTGCTCGAGATAGCACGATACCTGAAGAACAGAACCGAGAA
Consensus AAAGGGGCCACAGTAACAGGAGAGGAATTCTCGCAGGCTACGAAGCACTTGCTCGAGATAGCACGATACCTGAAGAACAGAACCGAGAA
35S-HcPro-35S-For
Trace data
35S-HcPro-P1H-Pro_seq1F
Trace data
35S-HcPro-P1H-Pro_seq2F AAAGGGGCCACAGTAACAGGAGAGGAATTCTCGCAGGCTACGAAGCACTTGCTCGAGATAGCACGATACCTGAAGAACAGAACCGAGAA
Trace data
35S-HcPro-P1H-Pro_seq3R AAAGGGGCCACAGTAACAGGAGAGGAATTCTCGCAGGCTACGAAGCACTTGCTCGAGATAGCACGATACCTGAAGAACAGAACCGAGAA
Trace data
35S-HcPro-P1H-Pro_seq4R AAAGGGGCCACAGTAACAGGAGAGGAATTCTCGCAGGCTACGAAGCACTTGCTCGAGATAGCACGATACCTGAAGAACAGAACCGAGAA
Trace data
35S-HcPro-T35S-Rev AAAGGGGCCCCAGTAACAGGAGAGGAATTTTCGCAGGCTACGAAGCACTTGCTCGAGATAGCACGATACCTGAAGAACAGAACCGAGAA
Trace data

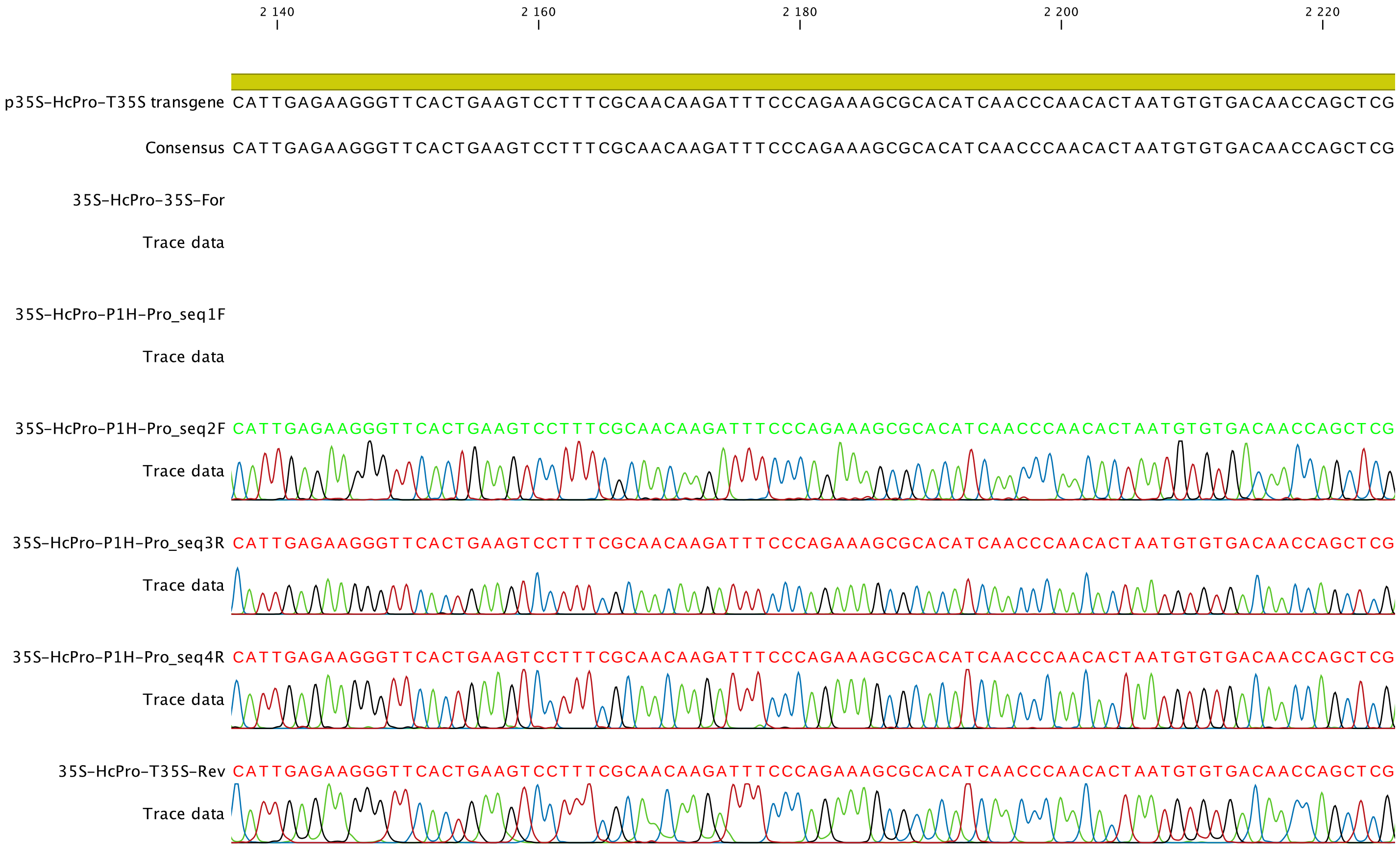
2 140
2 160
2 180
2 200
2 220
p35S-HcPro-T35S transgene CATTGAGAAGGGTTCACTGAAGTCCTTTCGCAACAAGATTTCCCAGAAAGCGCACATCAACCCAACACTAATGTGTGACAACCAGCTCG
Consensus CATTGAGAAGGGTTCACTGAAGTCCTTTCGCAACAAGATTTCCCAGAAAGCGCACATCAACCCAACACTAATGTGTGACAACCAGCTCG
35S-HcPro-35S-For
Trace data
35S-HcPro-P1H-Pro_seq1F
Trace data
35S-HcPro-P1H-Pro_seq2F CATTGAGAAGGGTTCACTGAAGTCCTTTCGCAACAAGATTTCCCAGAAAGCGCACATCAACCCAACACTAATGTGTGACAACCAGCTCG
Trace data
35S-HcPro-P1H-Pro_seq3R CATTGAGAAGGGTTCACTGAAGTCCTTTCGCAACAAGATTTCCCAGAAAGCGCACATCAACCCAACACTAATGTGTGACAACCAGCTCG
Trace data
35S-HcPro-P1H-Pro_seq4R CATTGAGAAGGGTTCACTGAAGTCCTTTCGCAACAAGATTTCCCAGAAAGCGCACATCAACCCAACACTAATGTGTGACAACCAGCTCG
Trace data
35S-HcPro-T35S-Rev CATTGAGAAGGGTTCACTGAAGTCCTTTCGCAACAAGATTTCCCAGAAAGCGCACATCAACCCAACACTAATGTGTGACAACCAGCTCG
Trace data

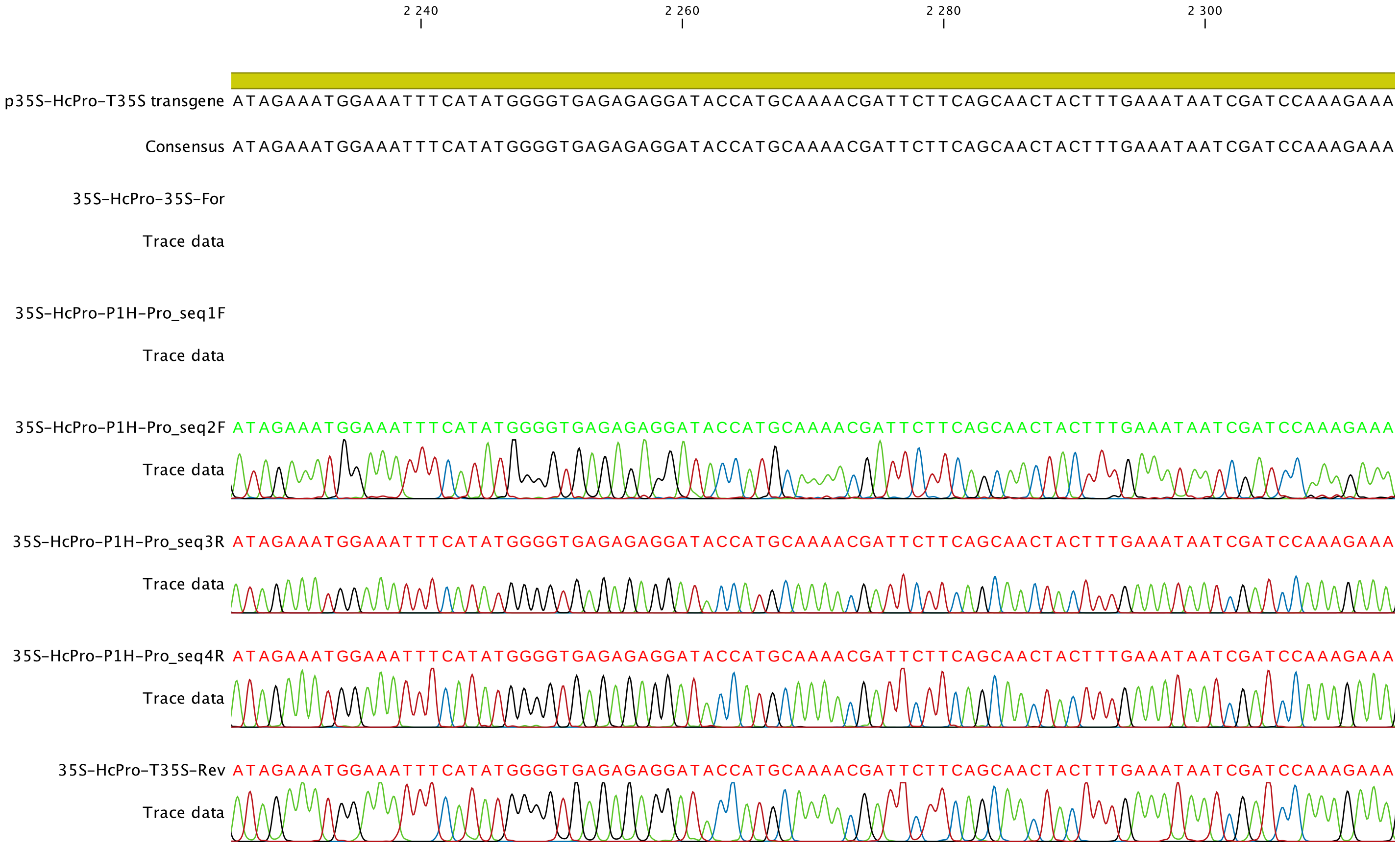
2 240
2 260
2 280
2 300
p35S-HcPro-T35S transgene ATAGAAATGGAAATTTCATATGGGGTGAGAGAGGATACCATGCAAAACGATTCTTCAGCAACTACTTTGAAATAATCGATCCAAAGAAA
Consensus ATAGAAATGGAAATTTCATATGGGGTGAGAGAGGATACCATGCAAAACGATTCTTCAGCAACTACTTTGAAATAATCGATCCAAAGAAA
35S-HcPro-35S-For
Trace data
35S-HcPro-P1H-Pro_seq1F
Trace data
35S-HcPro-P1H-Pro_seq2F ATAGAAATGGAAATTTCATATGGGGTGAGAGAGGATACCATGCAAAACGATTCTTCAGCAACTACTTTGAAATAATCGATCCAAAGAAA
Trace data
35S-HcPro-P1H-Pro_seq3R ATAGAAATGGAAATTTCATATGGGGTGAGAGAGGATACCATGCAAAACGATTCTTCAGCAACTACTTTGAAATAATCGATCCAAAGAAA
Trace data
35S-HcPro-P1H-Pro_seq4R ATAGAAATGGAAATTTCATATGGGGTGAGAGAGGATACCATGCAAAACGATTCTTCAGCAACTACTTTGAAATAATCGATCCAAAGAAA
Trace data
35S-HcPro-T35S-Rev ATAGAAATGGAAATTTCATATGGGGTGAGAGAGGATACCATGCAAAACGATTCTTCAGCAACTACTTTGAAATAATCGATCCAAAGAAA
Trace data

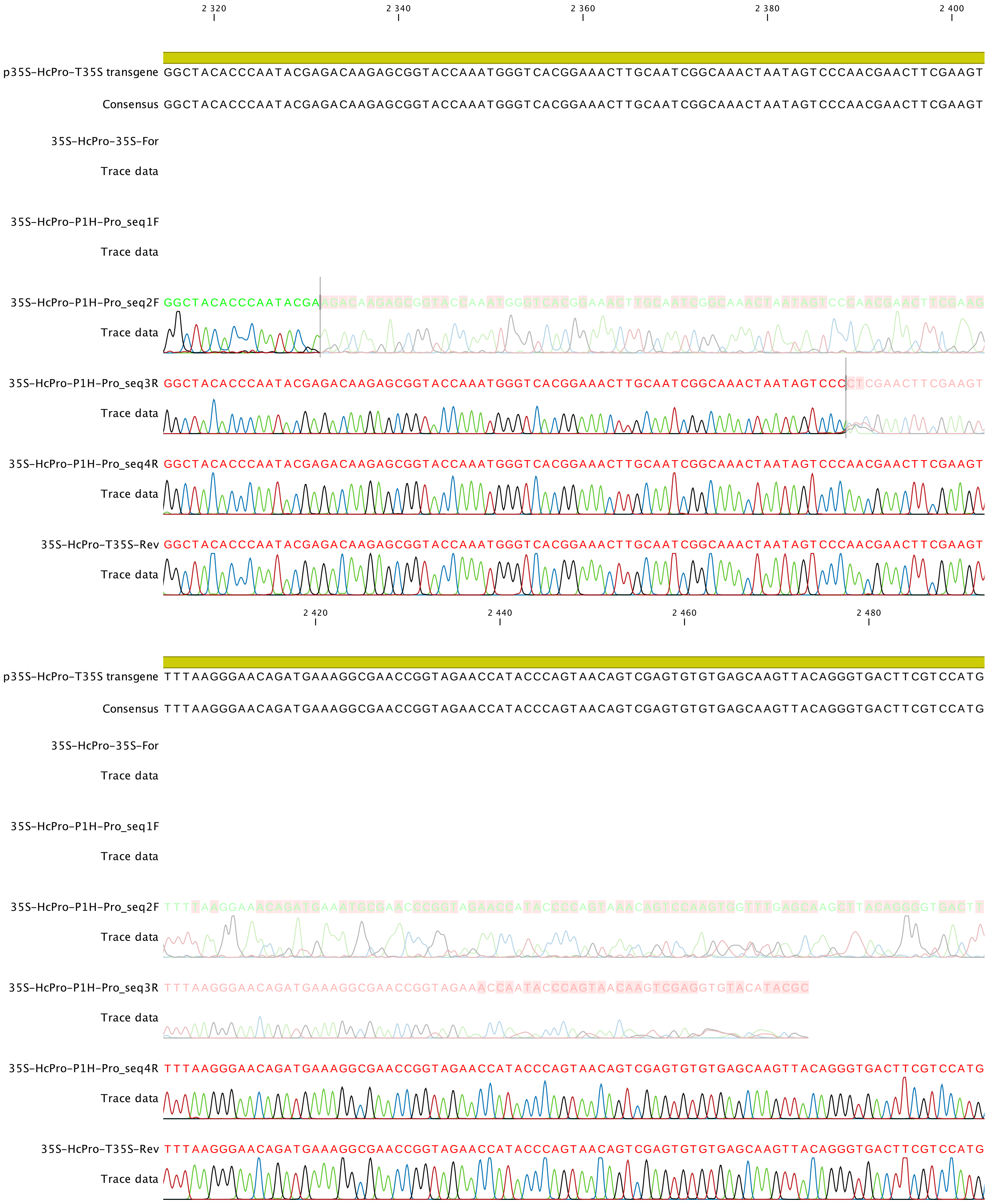
2 320
2 340
2 360
2 380
2 400
p35S-HcPro-T35S transgene GGCTACACCCAATACGAGACAAGAGCGGTACCAAATGGGTCACGGAAACTTGCAATCGGCAAACTAATAGTCCCAACGAACTTCGAAGT
Consensus GGCTACACCCAATACGAGACAAGAGCGGTACCAAATGGGTCACGGAAACTTGCAATCGGCAAACTAATAGTCCCAACGAACTTCGAAGT
35S-HcPro-35S-For
Trace data
35S-HcPro-P1H-Pro_seq1F
Trace data
35S-HcPro-P1H-Pro_seq2F GGCTACACCCAATACGAAGACAAGAGCGGTACCAAATGGGTCACGGAAACTTGCAATCGGCAAACTAATAGTCCCAACGAACTTCGAAG
Trace data
35S-HcPro-P1H-Pro_seq3R GGCTACACCCAATACGAGACAAGAGCGGTACCAAATGGGTCACGGAAACTTGCAATCGGCAAACTAATAGTCCCCTCGAACTTCGAAGT
Trace data
35S-HcPro-P1H-Pro_seq4R GGCTACACCCAATACGAGACAAGAGCGGTACCAAATGGGTCACGGAAACTTGCAATCGGCAAACTAATAGTCCCAACGAACTTCGAAGT
Trace data
35S-HcPro-T35S-Rev GGCTACACCCAATACGAGACAAGAGCGGTACCAAATGGGTCACGGAAACTTGCAATCGGCAAACTAATAGTCCCAACGAACTTCGAAGT
Trace data
2 420
2 440
2 460
2 480
p35S-HcPro-T35S transgene TTTAAGGGAACAGATGAAAGGCGAACCGGTAGAACCATACCCAGTAACAGTCGAGTGTGTGAGCAAGTTACAGGGTGACTTCGTCCATG
Consensus TTTAAGGGAACAGATGAAAGGCGAACCGGTAGAACCATACCCAGTAACAGTCGAGTGTGTGAGCAAGTTACAGGGTGACTTCGTCCATG
35S-HcPro-35S-For
Trace data
35S-HcPro-P1H-Pro_seq1F
Trace data
35S-HcPro-P1H-Pro_seq2F TTTTAAGGAAACAGATGAAATGCGAACCCGGTAGAACCATACCCCAGTAAACAGTCCAAGTGGTTTGAGCAAGCTTACAGGGGTGACTT
Trace data
35S-HcPro-P1H-Pro_seq3R TTTAAGGGAACAGATGAAAGGCGAACCGGTAGAAACCAATACCCAGTAACAAGTCGAGGTGTACATACGC
Trace data
35S-HcPro-P1H-Pro_seq4R TTTAAGGGAACAGATGAAAGGCGAACCGGTAGAACCATACCCAGTAACAGTCGAGTGTGTGAGCAAGTTACAGGGTGACTTCGTCCATG
Trace data
35S-HcPro-T35S-Rev TTTAAGGGAACAGATGAAAGGCGAACCGGTAGAACCATACCCAGTAACAGTCGAGTGTGTGAGCAAGTTACAGGGTGACTTCGTCCATG
Trace data

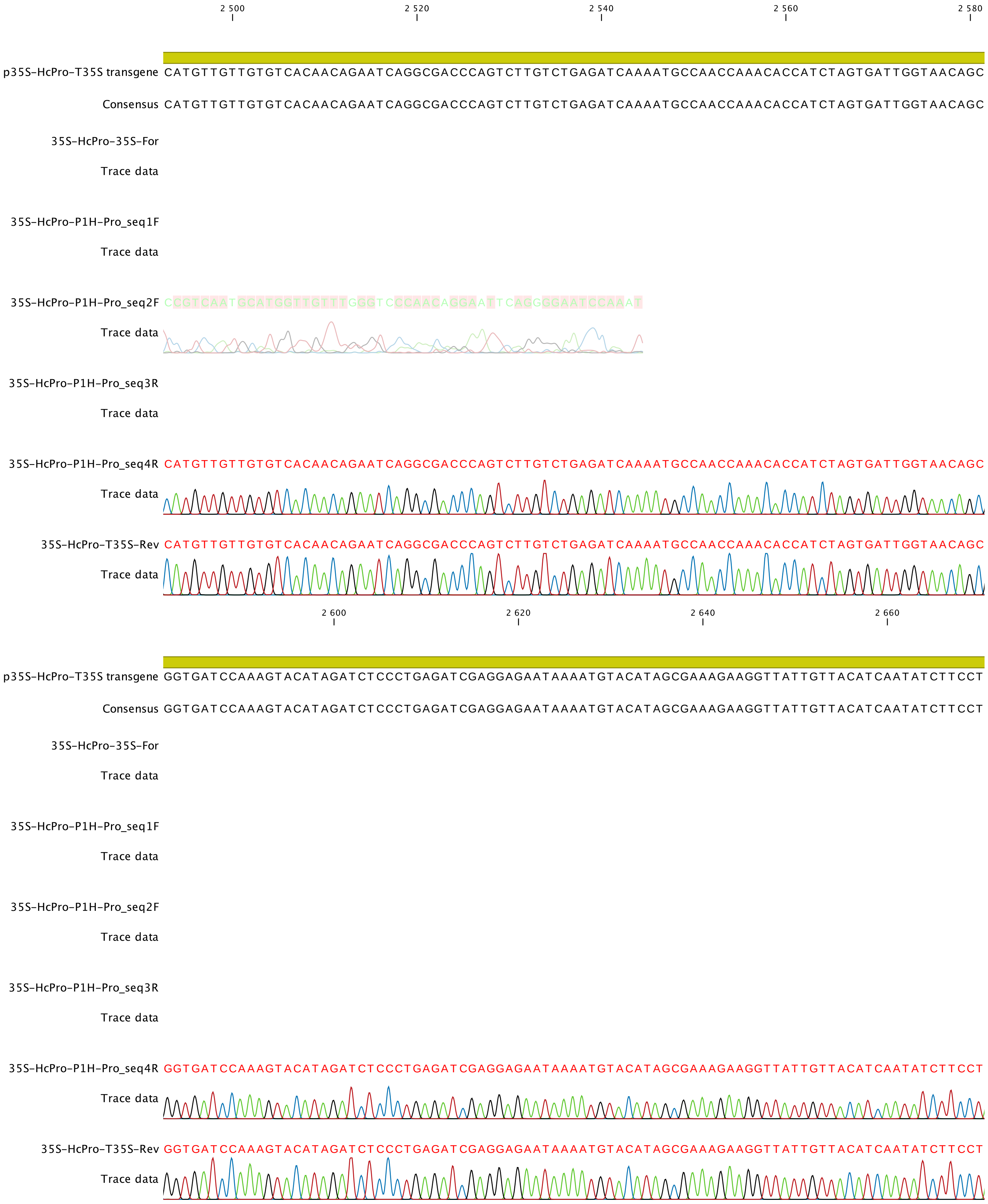
2 500
2 520
2 540
2 560
2 580
p35S-HcPro-T35S transgene CATGTTGTTGTGTCACAACAGAATCAGGCGACCCAGTCTTGTCTGAGATCAAAATGCCAACCAAACACCATCTAGTGATTGGTAACAGC
Consensus CATGTTGTTGTGTCACAACAGAATCAGGCGACCCAGTCTTGTCTGAGATCAAAATGCCAACCAAACACCATCTAGTGATTGGTAACAGC
35S-HcPro-35S-For
Trace data
35S-HcPro-P1H-Pro_seq1F
Trace data
35S-HcPro-P1H-Pro_seq2F CCGTCAATGCATGGTTGTTTGGGTCCCAACAGGAATTCAGGGGAATCCAAAT
Trace data
35S-HcPro-P1H-Pro_seq3R
Trace data
35S-HcPro-P1H-Pro_seq4R CATGTTGTTGTGTCACAACAGAATCAGGCGACCCAGTCTTGTCTGAGATCAAAATGCCAACCAAACACCATCTAGTGATTGGTAACAGC
Trace data
35S-HcPro-T35S-Rev CATGTTGTTGTGTCACAACAGAATCAGGCGACCCAGTCTTGTCTGAGATCAAAATGCCAACCAAACACCATCTAGTGATTGGTAACAGC
Trace data
2 600
2 620
2 640
2 660
p35S-HcPro-T35S transgene GGTGATCCAAAGTACATAGATCTCCCTGAGATCGAGGAGAATAAAATGTACATAGCGAAAGAAGGTTATTGTTACATCAATATCTTCCT
Consensus GGTGATCCAAAGTACATAGATCTCCCTGAGATCGAGGAGAATAAAATGTACATAGCGAAAGAAGGTTATTGTTACATCAATATCTTCCT
35S-HcPro-35S-For
Trace data
35S-HcPro-P1H-Pro_seq1F
Trace data
35S-HcPro-P1H-Pro_seq2F
Trace data
35S-HcPro-P1H-Pro_seq3R
Trace data
35S-HcPro-P1H-Pro_seq4R GGTGATCCAAAGTACATAGATCTCCCTGAGATCGAGGAGAATAAAATGTACATAGCGAAAGAAGGTTATTGTTACATCAATATCTTCCT
Trace data
35S-HcPro-T35S-Rev GGTGATCCAAAGTACATAGATCTCCCTGAGATCGAGGAGAATAAAATGTACATAGCGAAAGAAGGTTATTGTTACATCAATATCTTCCT
Trace data

2 680 | 2 700 | 2 720 | 2 740 | 2

p35S-HcPro-T35S transgene AGCCATGTTGGTAAATGTCAAGGAGTCGCAGGCAAAGGAGTTCACGAAAGTTGTTAGGGACAAACTAGTTGGCGAACTTGGCAAGTGGC

Consensus AGCCATGTTGGTAAATGTCAAGGAGTCGCAGGCAAAGGAGTTCACGAAAGTTGTTAGGGACAAACTAGTTGGCGAACTTGGCAAGTGGC

35S-HcPro-35S-For

Trace data

35S-HcPro-P1H-Pro_seq1F

Trace data

35S-HcPro-P1H-Pro_seq2F

Trace data

35S-HcPro-P1H-Pro_seq3R

Trace data

35S-HcPro-P1H-Pro_seq4R AGCCATGTTGGTAAATGTCAAGGAGTCGCAGGCAAAGGAGTTCACGAAAGTTGTTAGGGACAAACTAGTTGGCGAACTTGGCAAGTGGC

Trace data

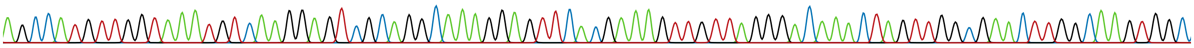

35S-HcPro-T35S-Rev AGCCATGTTGGTAAATGTCAAGGAGTCGCAGGCAAAGGAGTTCACGAAAGTTGTTAGGGACAAACTAGTTGGCGAACTTGGCAAGTGGC

Trace data

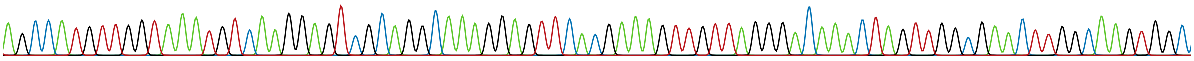

760 | 2 780 | 2 800 | 2 820 | 2 840

p35S-HcPro-T35S transgene CCACTCTGTTAGATGTAGCAACCGCTTGTTATTTCCTGAAAGTATTTTACCCAGACGTTGCTAACGCCGAATTGCCACGCATGCTAGTG

Consensus CCACTCTGTTAGATGTAGCAACCGCTTGTTATTTCCTGAAAGTATTTTACCCAGACGTTGCTAACGCCGAATTGCCACGCATGCTAGTG

35S-HcPro-35S-For

Trace data

35S-HcPro-P1H-Pro_seq1F

Trace data

35S-HcPro-P1H-Pro_seq2F

Trace data

35S-HcPro-P1H-Pro_seq3R

Trace data

35S-HcPro-P1H-Pro_seq4R CCACTCTGTTAGATGTAGCAACCGCTTGTTATTTCCTGAAAGTATTTTACCCAGACGTTGCTAACGCCGAATTGCCACGCATGCTAGTG

Trace data

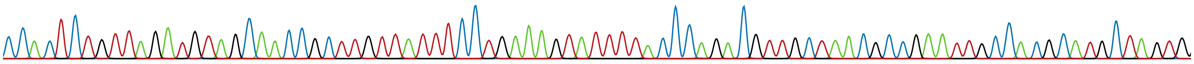

35S-HcPro-T35S-Rev CCACTCTGTTAGATGTAGCAACCGCTTGTTATTTCCTGAAAGTATTTTACCCAGACGTTGCTAACGCCGAATTGCCACGCATGCTAGTG

Trace data

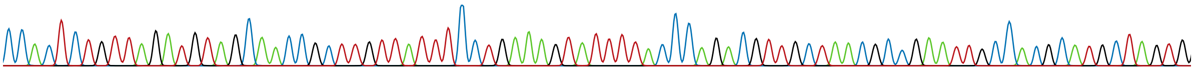

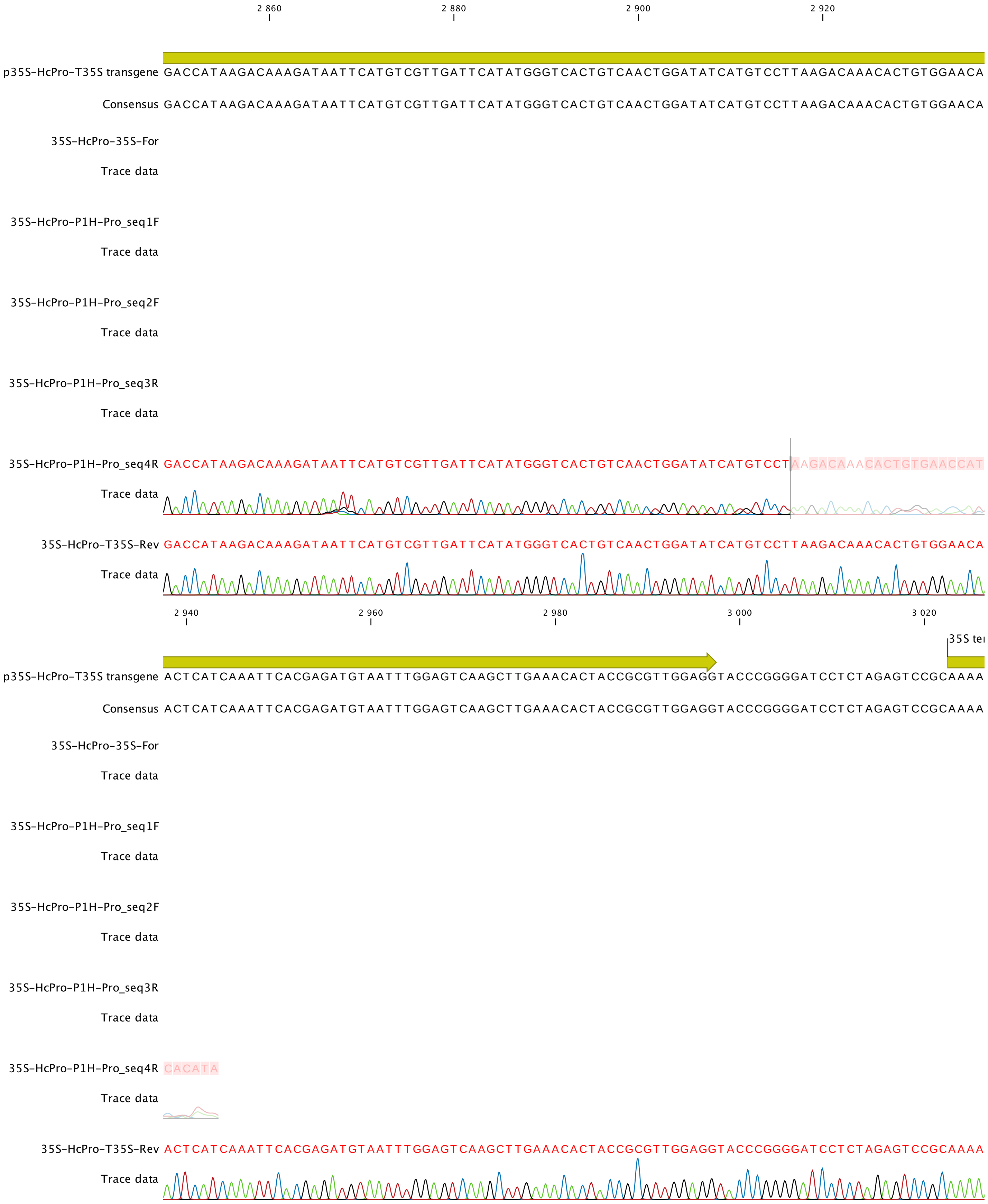
2 860
2 880
2 900
2 920
p35S-HcPro-T35S transgene GACCATAAGACAAAGATAATTCATGTCGTTGATTCATATGGGTCACTGTCAACTGGATATCATGTCCTTAAGACAAACACTGTGGAACA
Consensus GACCATAAGACAAAGATAATTCATGTCGTTGATTCATATGGGTCACTGTCAACTGGATATCATGTCCTTAAGACAAACACTGTGGAACA
35S-HcPro-35S-For
Trace data
35S-HcPro-P1H-Pro_seq1F
Trace data
35S-HcPro-P1H-Pro_seq2F
Trace data
35S-HcPro-P1H-Pro_seq3R
Trace data
35S-HcPro-P1H-Pro_seq4R GACCATAAGACAAAGATAATTCATGTCGTTGATTCATATGGGTCACTGTCAACTGGATATCATGTCCTAAGACAAACACTGTGAACCAT
Trace data
35S-HcPro-T35S-Rev GACCATAAGACAAAGATAATTCATGTCGTTGATTCATATGGGTCACTGTCAACTGGATATCATGTCCTTAAGACAAACACTGTGGAACA
Trace data
2 940
2 960
2 980
3 000
3 020
35S te
p35S-HcPro-T35S transgene ACTCATCAAATTCACGAGATGTAATTTGGAGTCAAGCTTGAAACACTACCGCGTTGGAGGTACCCGGGGATCCTCTAGAGTCCGCAAAA
Consensus ACTCATCAAATTCACGAGATGTAATTTGGAGTCAAGCTTGAAACACTACCGCGTTGGAGGTACCCGGGGATCCTCTAGAGTCCGCAAAA
35S-HcPro-35S-For
Trace data
35S-HcPro-P1H-Pro_seq1F
Trace data
35S-HcPro-P1H-Pro_seq2F
Trace data
35S-HcPro-P1H-Pro_seq3R
Trace data
35S-HcPro-P1H-Pro_seq4R CACATA
Trace data
35S-HcPro-T35S-Rev ACTCATCAAATTCACGAGATGTAATTTGGAGTCAAGCTTGAAACACTACCGCGTTGGAGGTACCCGGGGATCCTCTAGAGTCCGCAAAA
Trace data

3 040 | 3 060 | 3 080 | 3 100

rminator

p35S-HcPro-T35S transgene ATCACCAGTCTCTCTCTACAAATCTATCTCTCTCTATTTTTCTCCAGAATAATGTGTGAGTAGTTCCCAGATAAGGGAATTAGGGTTCT

Consensus ATCACCAGTCTCTCTCTACAAATCTATCTCTCTCTATTTTTCTCCAGAATAATGTGTGAGTAGTTCCCAGATAAGGGAATTAGGGTTCT

35S-HcPro-35S-For

Trace data

35S-HcPro-P1H-Pro_seq1F

Trace data

35S-HcPro-P1H-Pro_seq2F

Trace data

35S-HcPro-P1H-Pro_seq3R

Trace data

35S-HcPro-P1H-Pro_seq4R

Trace data

35S-HcPro-T35S-Rev ATCACCAGTCTCTCTCTACAAATCTATCTCTCTCTATTTTTCTCCAGAATAATGTGTGAGTAGTTCCCAGATAAGGGAATTAGGGTTCT

Trace data

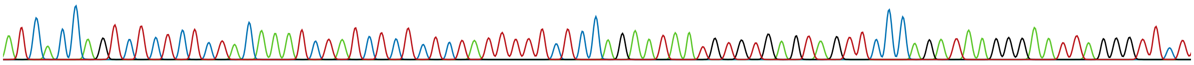

3 120 | 3 140 | 3 160 | 3 180 | 3 200

p35S-HcPro-T35S transgene TATAGGGTTTCGCTCATGTGTTGAGCATATAAGAAACCCTTAGTATGTATTTGTATTGTAAAATACTTCTATCAATAAAATTTCTAATT

Consensus TATAGGGTTTCGCTCATGTGTTGAGCATATAAGAAACCCTTAGTATGTATTTGTATTGTAAAATACT

35S-HcPro-35S-For

Trace data

35S-HcPro-P1H-Pro_seq1F

Trace data

35S-HcPro-P1H-Pro_seq2F

Trace data

35S-HcPro-P1H-Pro_seq3R

Trace data

35S-HcPro-P1H-Pro_seq4R

Trace data

35S-HcPro-T35S-Rev TATAGGGTTTCGCTCATGTGTTGAGCATATAAGAAACCCTTAGTATGTATTTGTATTGTAAAATACTCTACATATAAC

Trace data

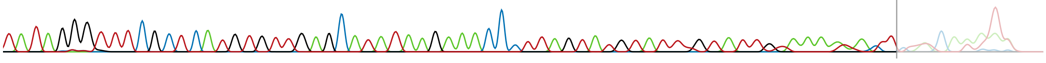

3 220 | 3 240 | 3 260 | 3 280

T35S Rev

p35S-HcPro-T35S transgene CCTAAAACCAAAATCCAGTGACTAAAATCCAGATCTCCTAAAGTCCCTATAGATCTTTGTCGTGAATATAAACCAGACACGAGACGACT

Consensus

35S-HcPro-35S-For

Trace data

35S-HcPro-P1H-Pro_seq1F

Trace data

35S-HcPro-P1H-Pro_seq2F

Trace data

35S-HcPro-P1H-Pro_seq3R

Trace data

35S-HcPro-P1H-Pro_seq4R

Trace data

35S-HcPro-T35S-Rev

Trace data

3 300 | 3 320 | 3 340 | 3 360 | 3 380

p35S-HcPro-T35S transgene AAACCTGGAGCCCAGACGCCGTTCGAAGCTAGAAGTACCGCTTAGGCAGGAGGCCGTTAGGGAAAAGATGCTAAGGCAGGGTTGGTTAC

Consensus

35S-HcPro-35S-For

Trace data

35S-HcPro-P1H-Pro_seq1F

Trace data

35S-HcPro-P1H-Pro_seq2F

Trace data

35S-HcPro-P1H-Pro_seq3R

Trace data

35S-HcPro-P1H-Pro_seq4R

Trace data

35S-HcPro-T35S-Rev

Trace data

3 400 3 420 3 440 3 460

p35S-HcPro-T35S transgene GTTGACTCCCCCGTAGGTTTGGTTTAAATATGATGAAGTGGACGGAAGGAAGGAGGAAGACAAGGAAGGATAAGGTTGCAGGCCCTGTG

Consensus

35S-HcPro-35S-For

Trace data

35S-HcPro-P1H-Pro_seq1F

Trace data

35S-HcPro-P1H-Pro_seq2F

Trace data

35S-HcPro-P1H-Pro_seq3R

Trace data

35S-HcPro-P1H-Pro_seq4R

Trace data

35S-HcPro-T35S-Rev

Trace data

3 480 3 500 3 520 3 540 3 56

p35S-HcPro-T35S transgene CAAGGTAAGAAGATGGAAATTTGATAGAGGTACGCTACTATACTTATACTATACGCTAAGGGAATGCTTGTATTTATACCCTATACCCC

Consensus

35S-HcPro-35S-For

Trace data

35S-HcPro-P1H-Pro_seq1F

Trace data

35S-HcPro-P1H-Pro_seq2F

Trace data

35S-HcPro-P1H-Pro_seq3R

Trace data

35S-HcPro-P1H-Pro_seq4R

Trace data

35S-HcPro-T35S-Rev

Trace data

	;0 3 580 3 600 3 620 3 640
p35S-HcPro-T35S transgene	CTAATAACCCCTTATCAATTTAAGAAATAATCCGCATAAGCCCCCGCTTAAAAATTGGTATCAGAGCCATGAATAGGTCTATGACCAAA
Consensus	
35S-HcPro-35S-For	
Trace data	
35S-HcPro-P1H-Pro_seq1F	
Trace data	
35S-HcPro-P1H-Pro_seq2F	
Trace data	
35S-HcPro-P1H-Pro_seq3R	
Trace data	
35S-HcPro-P1H-Pro_seq4R	
Trace data	
35S-HcPro-T35S-Rev	
Trace data	

	3 660 3 680 3 700 3 720
p35S-HcPro-T35S transgene	ACTCAAGAGGATAAAACCTCACCAAAATACGAAAGAGTTCTTAACTCTAAAGATAAAAGATCTTTCAAGATCAAAACT
Consensus	
35S-HcPro-35S-For	
Trace data	
35S-HcPro-P1H-Pro_seq1F	
Trace data	
35S-HcPro-P1H-Pro_seq2F	
Trace data	
35S-HcPro-P1H-Pro_seq3R	
Trace data	
35S-HcPro-P1H-Pro_seq4R	
Trace data	
35S-HcPro-T35S-Rev	
Trace data	
