# Supplementary material for: Correction: Misregulation of AUXIN RESPONSE FACTOR 8 Underlies the Developmental Abnormalities Caused by Three Distinct Viral Silencing Suppressors in Arabidopsis
Source: PLoS Pathog. 2016 May 5;12(5):e1005627. doi: 10.1371/journal.ppat.1005627 (PMC4858414; doi:10.1371/journal.ppat.1005627)
Supplement: S8 Fig — The hygromycin selection gene present in the HcPro transgenic line was also assembled. (ZIP) [file ppat.1005627.s008.zip › contig 35S-p15.compressed.pdf]

20 40 60 80

35S Promoter

p35S-p15-T35S transgene CTACTCCAAAAATGTCAAAGATACAGTCTCAGAAGACCAAAGGGCTATTGAGACTTTTCAACAAAGGGTAATTTCGGGAAACCTCCTCGG

Consensus

35S-P15-T35S-Rev

Trace data

35S-P15-35S-For

Trace data

100 120 140 160 180

p35S-p15-T35S transgene ATTCCATTGCCCAGCTATCTGTCACTTCATCGAAAGGACAGTAGAAAAGGAAGGTGGCTCCTACAAATGCCATCATTGCGATAAAGGAAA

Consensus

35S-P15-T35S-Rev

Trace data

35S-P15-35S-For

Trace data

200 220 240 260

p35S-p15-T35S transgene GGCTATCATTCAAGATGCCTCTGCCGACAGTGGTCCCAAAGATGGACCCCCACCCACGAGGAGCATCGTGGAAAAAGAAGACGTCCCAAC

Consensus

35S-P15-T35S-Rev

Trace data

35S-P15-35S-For

Trace data

280 300 320 340 360

p35S For

p35S-p15-T35S transgene CACGTCTTCAAAGCAAGTGGATTGATGTGACATCTCCACTGACGTAAGGGATGACGCACAATCCCACTATCCTTCGCAAGACCCTTCCTC

Consensus GGATGACGCACAATCCCACTATCCTTCGCAAGACCCTTCCTC

35S-P15-T35S-Rev TTTTTTTTTTTCTCCACGAAAGTTTAGGATGACGCACAATCCCACTATCCTTCGCAAGACCCTTCCTC

Trace data

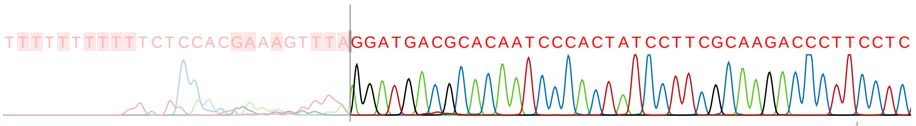

35S-P15-35S-For AACTTTCTCGAGAGCCTTCCCTC

Trace data

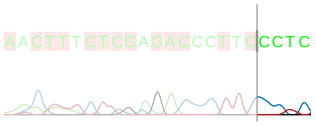

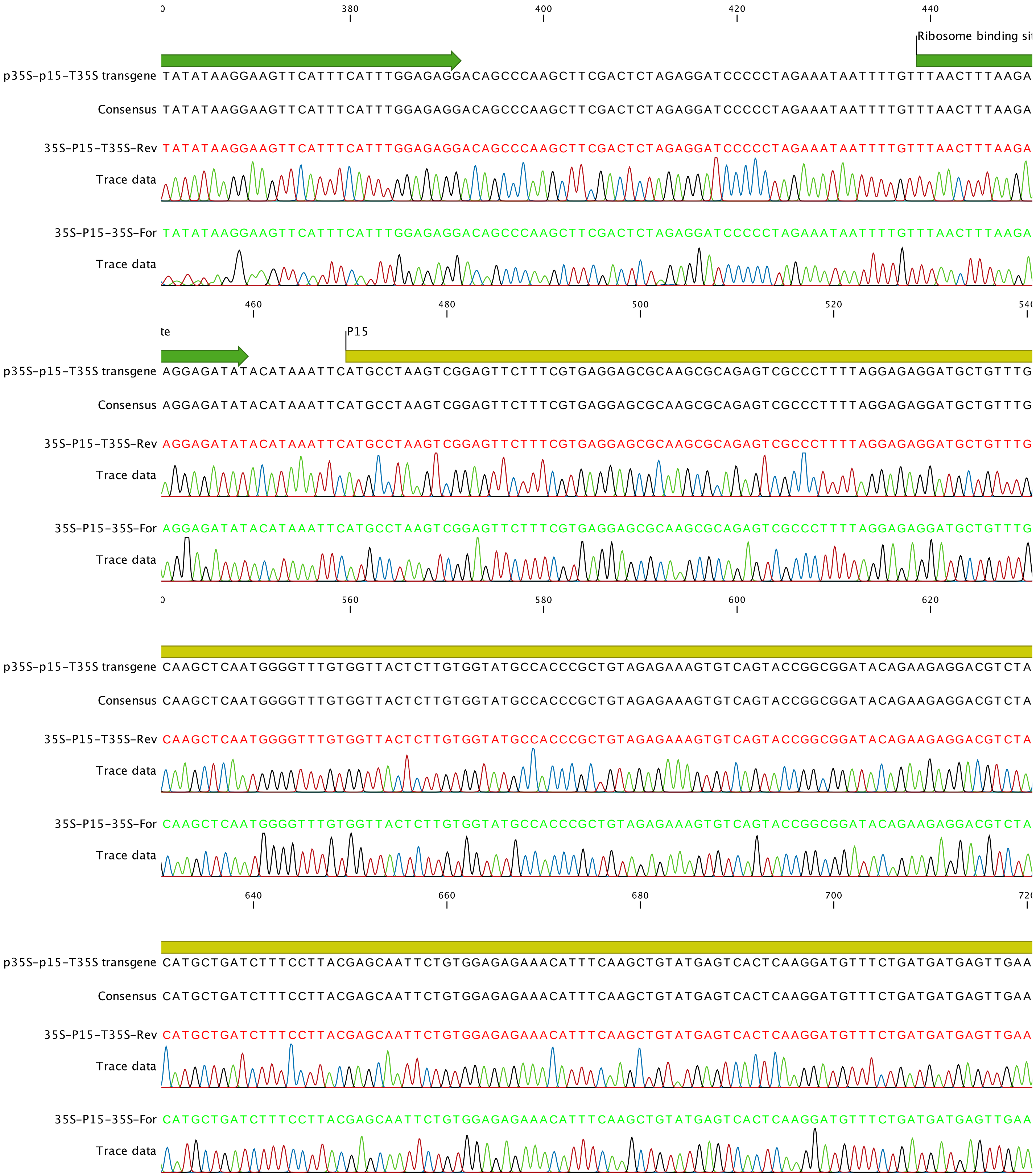
380
400
420
440
Ribosome binding si
p35S-p15-T35S transgene TATATAAGGAAGTTCATTTCATTTGGAGAGGACAGCCCAAGCTTCGACTCTAGAGGATCCCCCTAGAAATAATTTTGTTTAACTTTAAGA
Consensus TATATAAGGAAGTTCATTTCATTTGGAGAGGACAGCCCAAGCTTCGACTCTAGAGGATCCCCCTAGAAATAATTTTGTTTAACTTTAAGA
35S-P15-T35S-Rev TATATAAGGAAGTTCATTTCATTTGGAGAGGACAGCCCAAGCTTCGACTCTAGAGGATCCCCCTAGAAATAATTTTGTTTAACTTTAAGA
Trace data
35S-P15-35S-For TATATAAGGAAGTTCATTTCATTTGGAGAGGACAGCCCAAGCTTCGACTCTAGAGGATCCCCCTAGAAATAATTTTGTTTAACTTTAAGA
Trace data
460
480
500
520
te
P15
p35S-p15-T35S transgene AGGAGATATACATAAATTCATGCCTAAGTCGGAGTTCTTTCGTGAGGAGCGCAAGCGCAGAGTCGCCCTTTTAGGAGAGGATGCTGTTTG
Consensus AGGAGATATACATAAATTCATGCCTAAGTCGGAGTTCTTTCGTGAGGAGCGCAAGCGCAGAGTCGCCCTTTTAGGAGAGGATGCTGTTTG
35S-P15-T35S-Rev AGGAGATATACATAAATTCATGCCTAAGTCGGAGTTCTTTCGTGAGGAGCGCAAGCGCAGAGTCGCCCTTTTAGGAGAGGATGCTGTTTG
Trace data
35S-P15-35S-For AGGAGATATACATAAATTCATGCCTAAGTCGGAGTTCTTTCGTGAGGAGCGCAAGCGCAGAGTCGCCCTTTTAGGAGAGGATGCTGTTTG
Trace data
560
580
600
620
p35S-p15-T35S transgene CAAGCTCAATGGGGTTTGTGGTTACTCTTGTGGTATGCCACCCGCTGTAGAGAAAGTGTCAGTACCGGCGGATACAGAAGAGGACGTCTA
Consensus CAAGCTCAATGGGGTTTGTGGTTACTCTTGTGGTATGCCACCCGCTGTAGAGAAAGTGTCAGTACCGGCGGATACAGAAGAGGACGTCTA
35S-P15-T35S-Rev CAAGCTCAATGGGGTTTGTGGTTACTCTTGTGGTATGCCACCCGCTGTAGAGAAAGTGTCAGTACCGGCGGATACAGAAGAGGACGTCTA
Trace data
35S-P15-35S-For CAAGCTCAATGGGGTTTGTGGTTACTCTTGTGGTATGCCACCCGCTGTAGAGAAAGTGTCAGTACCGGCGGATACAGAAGAGGACGTCTA
Trace data
640
660
680
700
p35S-p15-T35S transgene CATGCTGATCTTTCCTTACGAGCAATTCTGTGGAGAGAAACATTTCAAGCTGTATGAGTCACTCAAGGATGTTTCTGATGATGAGTTGAA
Consensus CATGCTGATCTTTCCTTACGAGCAATTCTGTGGAGAGAAACATTTCAAGCTGTATGAGTCACTCAAGGATGTTTCTGATGATGAGTTGAA
35S-P15-T35S-Rev CATGCTGATCTTTCCTTACGAGCAATTCTGTGGAGAGAAACATTTCAAGCTGTATGAGTCACTCAAGGATGTTTCTGATGATGAGTTGAA
Trace data
35S-P15-35S-For CATGCTGATCTTTCCTTACGAGCAATTCTGTGGAGAGAAACATTTCAAGCTGTATGAGTCACTCAAGGATGTTTCTGATGATGAGTTGAA
Trace data

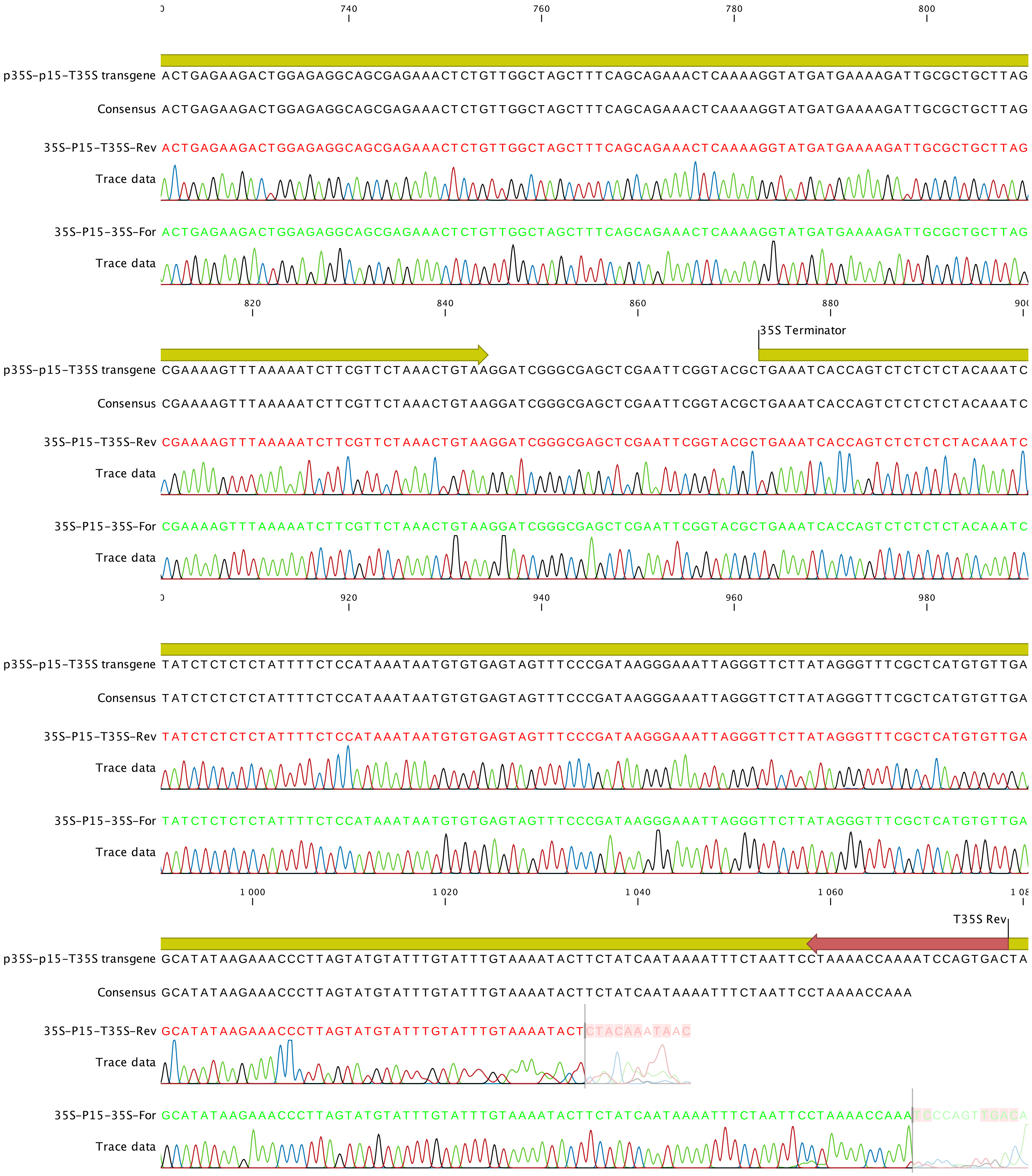
740
760
780
800
p35S-p15-T35S transgene ACTGAGAAGACTGGAGAGGCAGCGAGAAACTCTGTTGGCTAGCTTTCAGCAGAAACTCAAAAGGTATGATGAAAAGATTGCGCTGCTTAG
Consensus ACTGAGAAGACTGGAGAGGCAGCGAGAAACTCTGTTGGCTAGCTTTCAGCAGAAACTCAAAAGGTATGATGAAAAGATTGCGCTGCTTAG
35S-P15-T35S-Rev ACTGAGAAGACTGGAGAGGCAGCGAGAAACTCTGTTGGCTAGCTTTCAGCAGAAACTCAAAAGGTATGATGAAAAGATTGCGCTGCTTAG
Trace data
35S-P15-35S-For ACTGAGAAGACTGGAGAGGCAGCGAGAAACTCTGTTGGCTAGCTTTCAGCAGAAACTCAAAAGGTATGATGAAAAGATTGCGCTGCTTAG
Trace data
820
840
860
880
35S Terminator
p35S-p15-T35S transgene CGAAAAGTTTAAAAATCTTCGTTCTAAACTGTAAGGATCGGGCGAGCTCGAATTCGGTACGCTGAAATCACCAGTCTCTCTCTACAAATC
Consensus CGAAAAGTTTAAAAATCTTCGTTCTAAACTGTAAGGATCGGGCGAGCTCGAATTCGGTACGCTGAAATCACCAGTCTCTCTCTACAAATC
35S-P15-T35S-Rev CGAAAAGTTTAAAAATCTTCGTTCTAAACTGTAAGGATCGGGCGAGCTCGAATTCGGTACGCTGAAATCACCAGTCTCTCTCTACAAATC
Trace data
35S-P15-35S-For CGAAAAGTTTAAAAATCTTCGTTCTAAACTGTAAGGATCGGGCGAGCTCGAATTCGGTACGCTGAAATCACCAGTCTCTCTCTACAAATC
Trace data
920
940
960
980
p35S-p15-T35S transgene TATCTCTCTCTATTTTCTCCATAAATAATGTGTGAGTAGTTTCCCGATAAGGGAAATTAGGGTTCTTATAGGGTTTCGCTCATGTGTTGA
Consensus TATCTCTCTCTATTTTCTCCATAAATAATGTGTGAGTAGTTTCCCGATAAGGGAAATTAGGGTTCTTATAGGGTTTCGCTCATGTGTTGA
35S-P15-T35S-Rev TATCTCTCTCTATTTTCTCCATAAATAATGTGTGAGTAGTTTCCCGATAAGGGAAATTAGGGTTCTTATAGGGTTTCGCTCATGTGTTGA
Trace data
35S-P15-35S-For TATCTCTCTCTATTTTCTCCATAAATAATGTGTGAGTAGTTTCCCGATAAGGGAAATTAGGGTTCTTATAGGGTTTCGCTCATGTGTTGA
Trace data
1 000
1 020
1 040
1 060
T35S Rev
p35S-p15-T35S transgene GCATATAAGAAACCCTTAGTATGTATTTGTATTTGTAAAATACTTCTATCAATAAAATTTCTAATTCCTAAAACCAAAATCCAGTGACTA
Consensus GCATATAAGAAACCCTTAGTATGTATTTGTATTTGTAAAATACTTCTATCAATAAAATTTCTAATTCCTAAAACCAAA
35S-P15-T35S-Rev GCATATAAGAAACCCTTAGTATGTATTTGTATTTGTAAAATACTCTACAAATAAC
Trace data
35S-P15-35S-For GCATATAAGAAACCCTTAGTATGTATTTGTATTTGTAAAATACTTCTATCAATAAAATTTCTAATTCCTAAAACCAAATCCCAGTTGACA
Trace data

30 1 100 1 120 1 140 1 160

p35S-p15-T35S transgene AAATCCAGATCTCCTAAAGTCCCTATAGATCTTTGTCGTGAATATAAACCAGACACGAGACGACTAAACCTGGAGCCCAGACGCCGTTCG

Consensus

35S-P15-T35S-Rev

Trace data

35S-P15-35S-For AAAAAAAGGAATGAATATCACTATC

Trace data

1 180 1 200 1 220 1 240 1 26

p35S-p15-T35S transgene AAGCTAGAAGTACCGCTTAGGCAGGAGGCCGTTAGGGAAAAGATGCTAAGGCAGGGTTGGTTACGTTGACTCCCCCGTAGGTTTGGTTTA

Consensus

35S-P15-T35S-Rev

Trace data

35S-P15-35S-For

Trace data

60 1 280 1 300 1 320 1 340

p35S-p15-T35S transgene AATATGATGAAGTGGACGGAAGGAAGGAGGAAGACAAGGAAGGATAAGGTTGCAGGCCCTGTGCAAGGTAAGAAGATGGAAATTTGATAG

Consensus

35S-P15-T35S-Rev

Trace data

35S-P15-35S-For

Trace data

1 360 1 380 1 400 1 420 1 44

p35S-p15-T35S transgene AGGTACGCTACTATACTTATACTATACGCTAAGGGAATGCTTGTATTTATACCCTATACCCCCTAATAACCCCTTATCAATTTAAGAAAT

Consensus

35S-P15-T35S-Rev

Trace data

35S-P15-35S-For

Trace data

```
                        40                  1 460               1 480               1 500               1 520
p35S-p15-T35S transgene AATCCGCATAAGCCCCCGCTTAAAAATTGGTATCAGAGCCATGAATAGGTCTATGACCAAAACTCAAGAGGATAAAACCTCACCAAAATA
Consensus
35S-P15-T35S-Rev
Trace data

35S-P15-35S-For
Trace data
```

```
                              1 540               1 560
p35S-p15-T35S transgene CGAAAGAGTTCTTAACTCTAAAGATAAAAGATCTTTCAAGATCAAAACT
Consensus
35S-P15-T35S-Rev
Trace data

35S-P15-35S-For
Trace data
```
