# Supplementary material for: Correction: Misregulation of AUXIN RESPONSE FACTOR 8 Underlies the Developmental Abnormalities Caused by Three Distinct Viral Silencing Suppressors in Arabidopsis
Source: PLoS Pathog. 2016 May 5;12(5):e1005627. doi: 10.1371/journal.ppat.1005627 (PMC4858414; doi:10.1371/journal.ppat.1005627)

New Figure 4

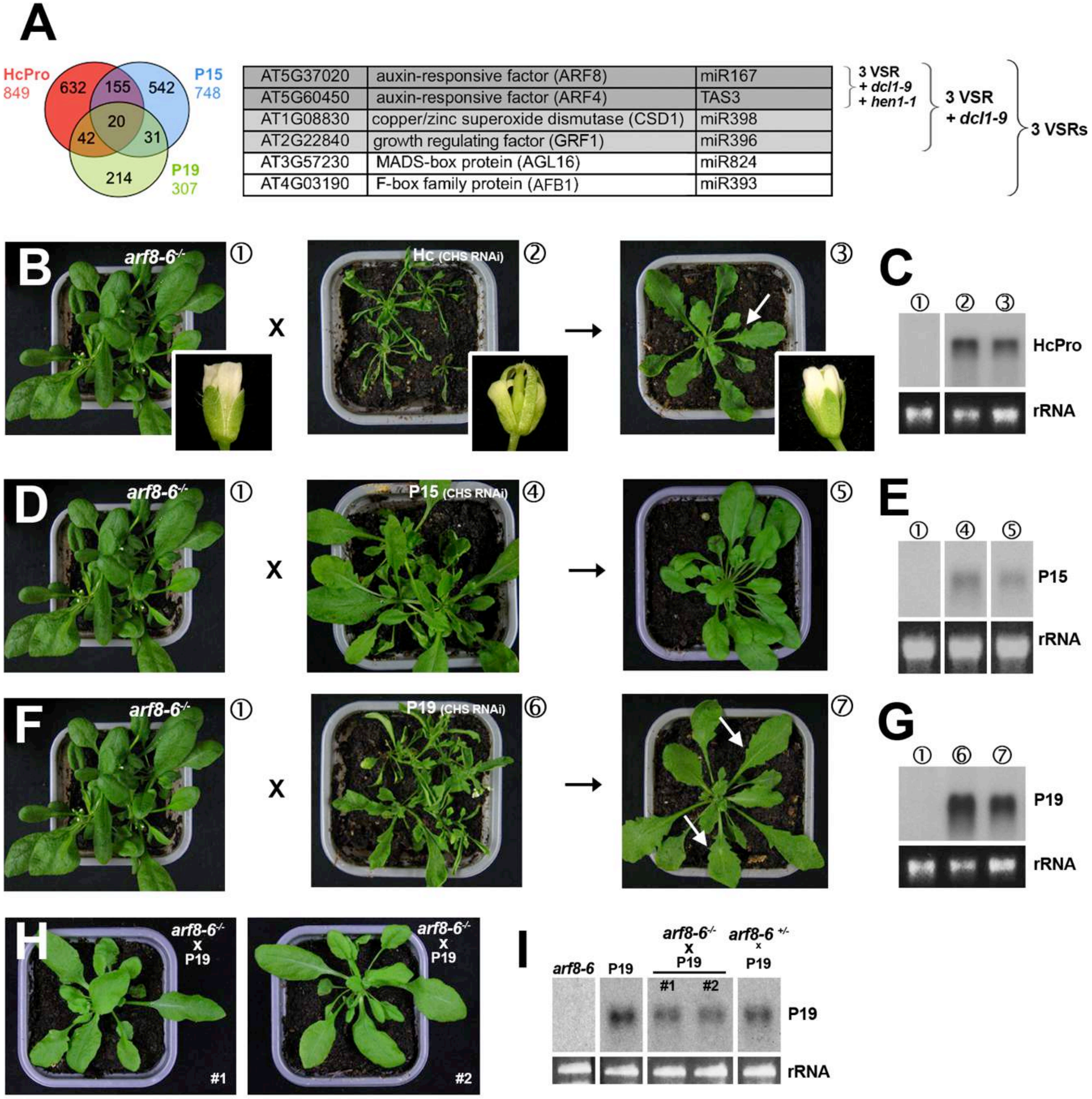

AT5G37020	auxin-responsive factor (ARF8)	miR167
AT5G60450	auxin-responsive factor (ARF4)	TAS3
AT1G08830	copper/zinc superoxide dismutase (CSD1)	miR398
AT2G22840	growth regulating factor (GRF1)	miR396
AT3G57230	MADS-box protein (AGL16)	miR824
AT4G03190	F-box family protein (AFB1)	miR393

Panel 4C

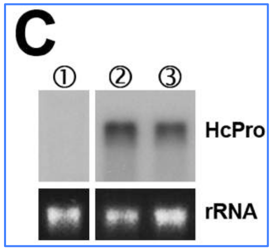

HcPro

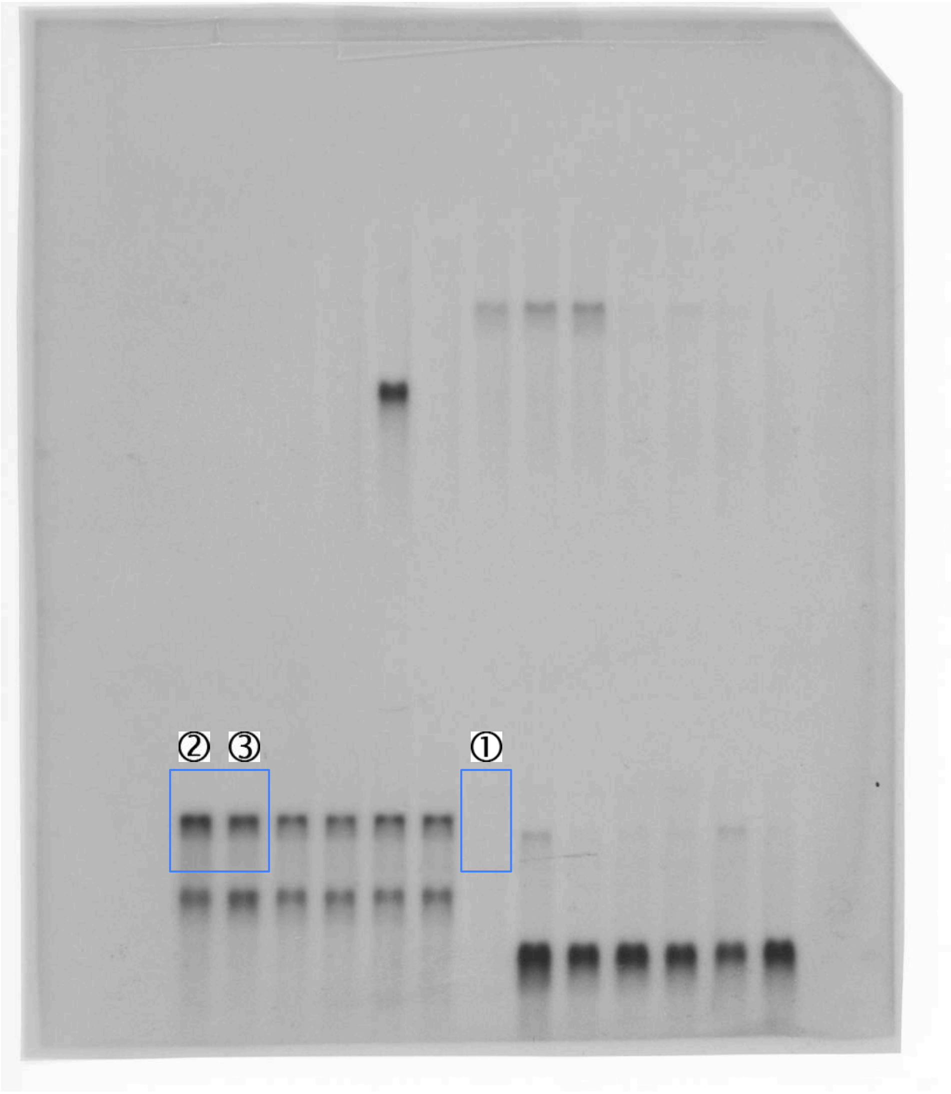

rRNA

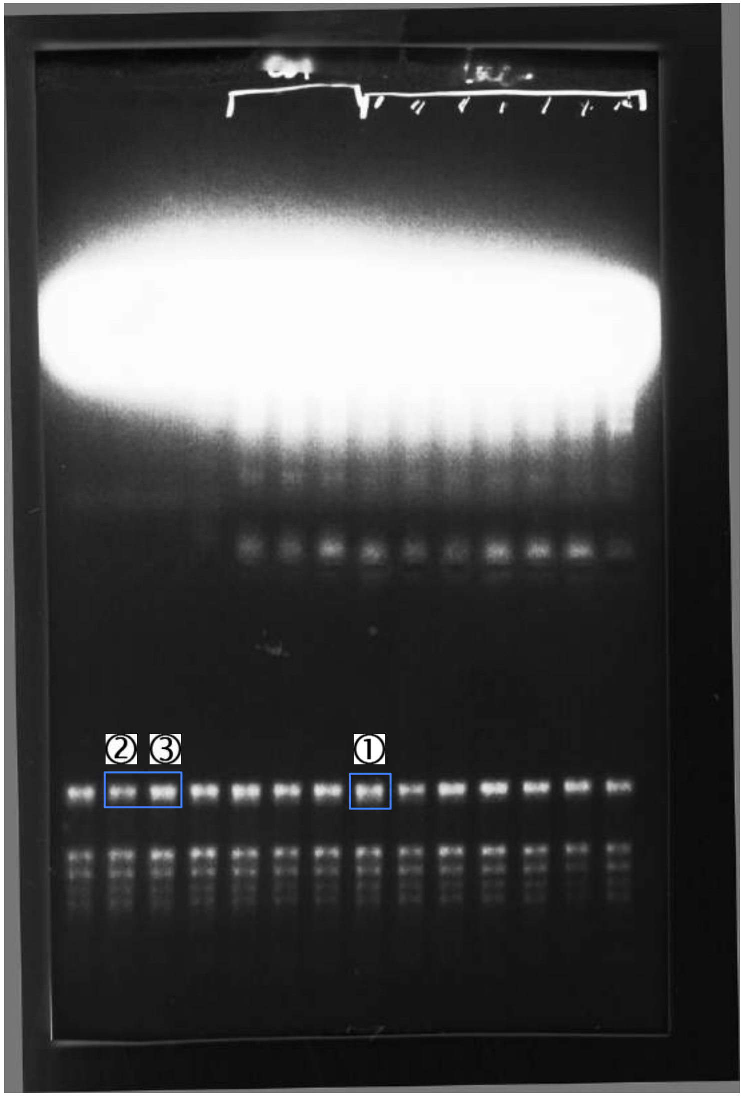

Panel 4E

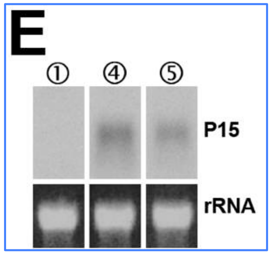

P15

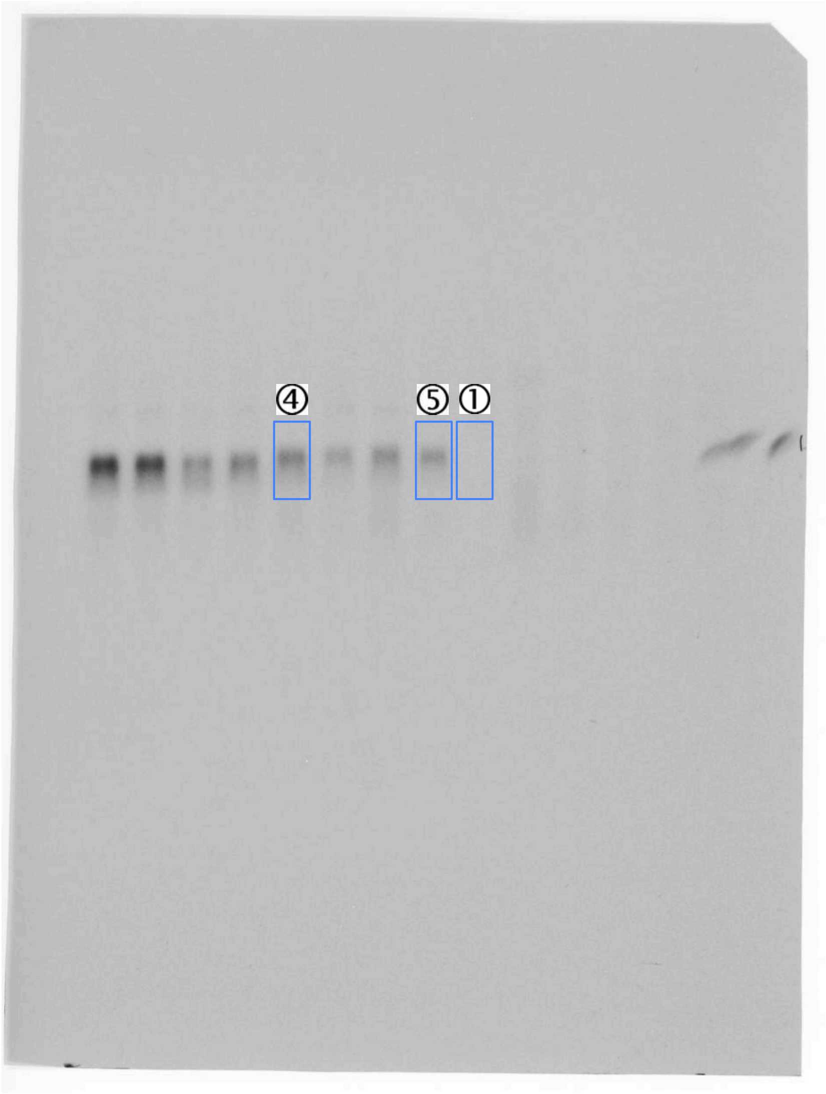

rRNA

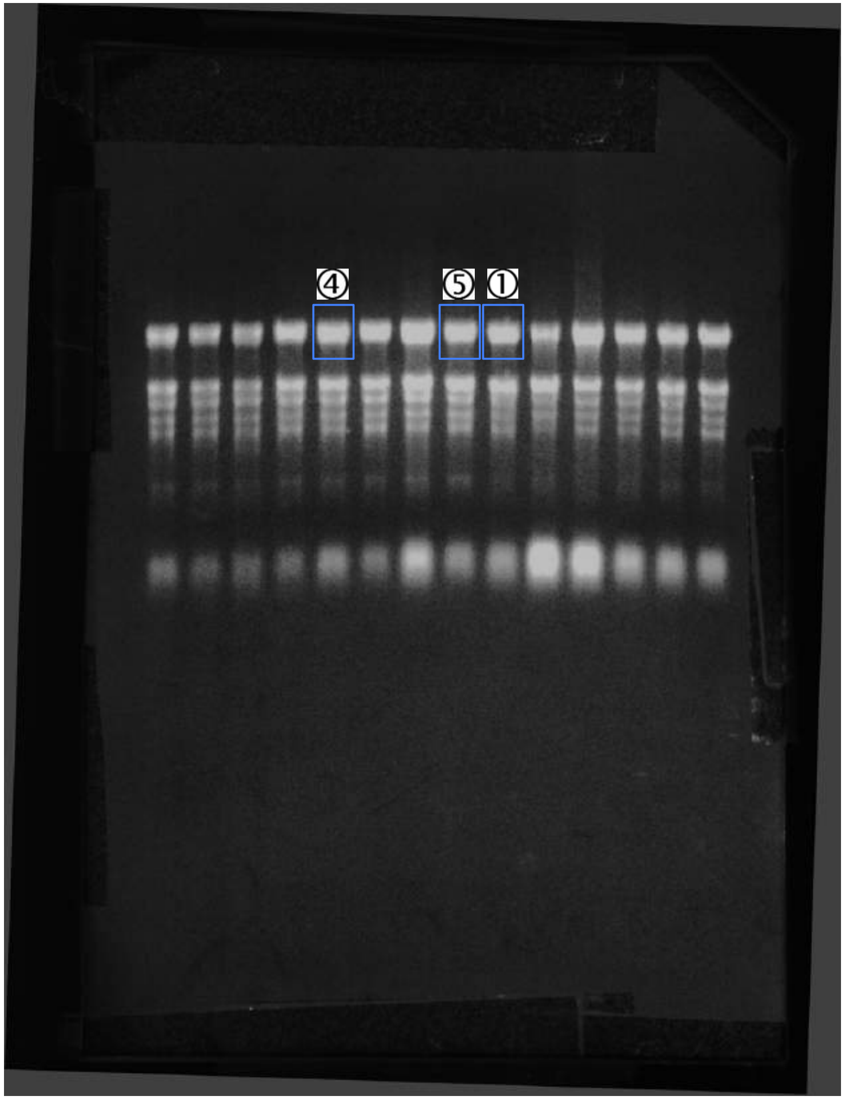

Panel 4G

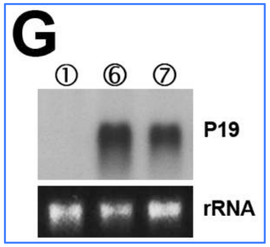

P19

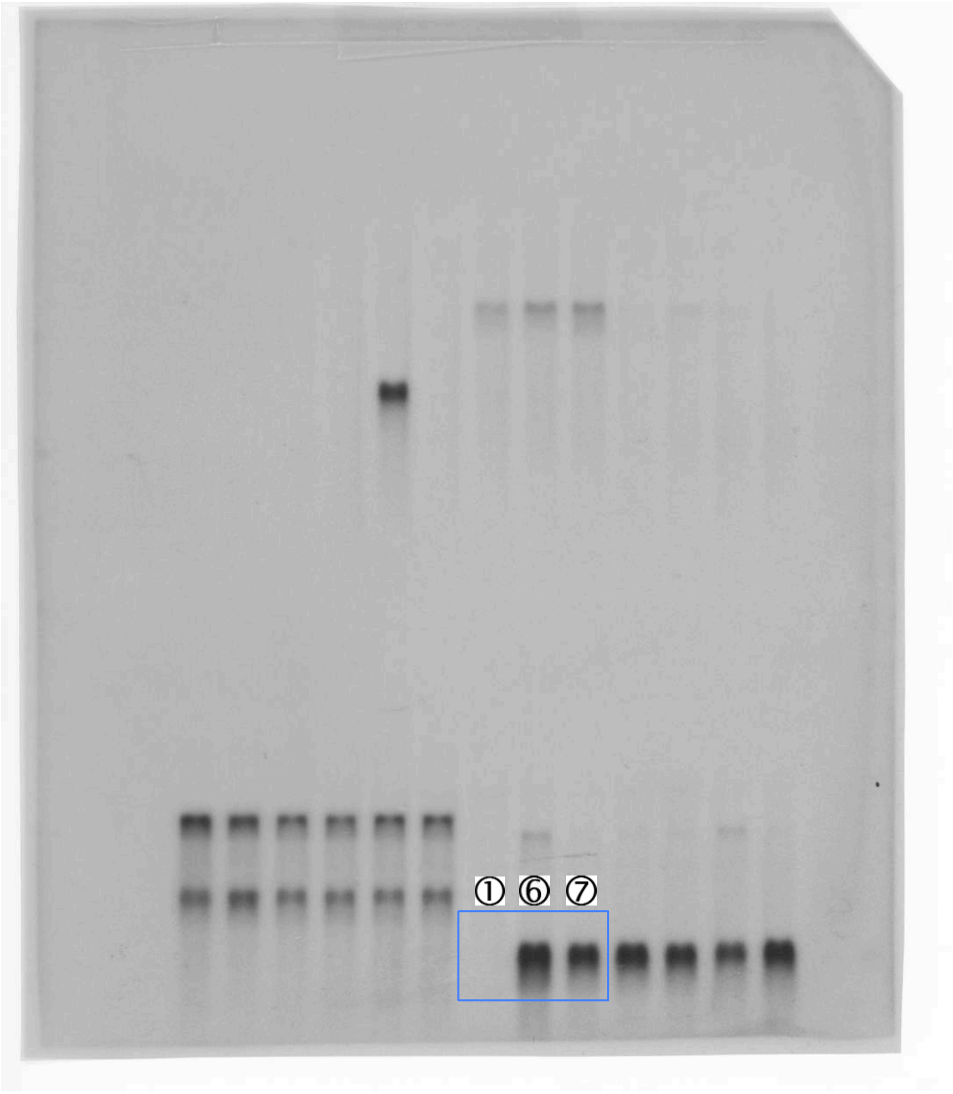

rRNA

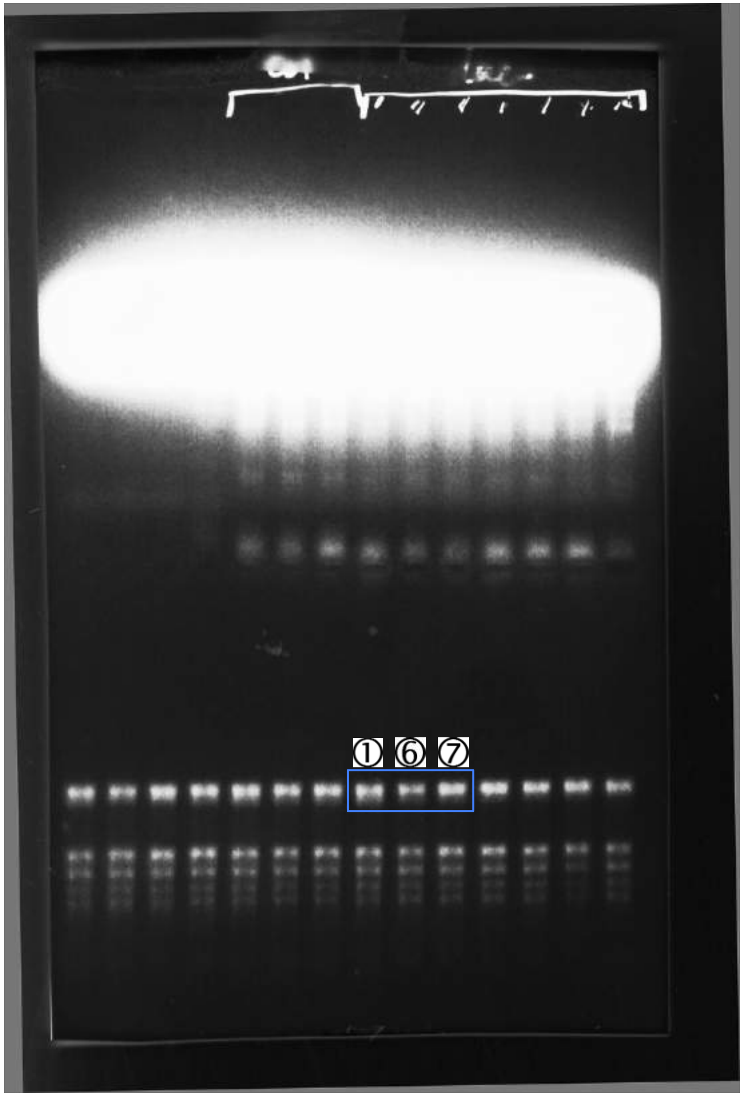

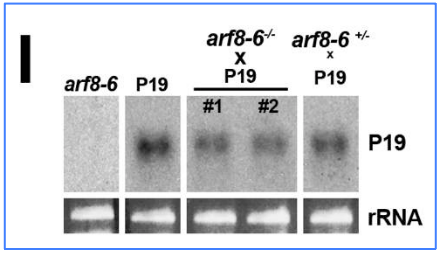

Panel 4I

P19

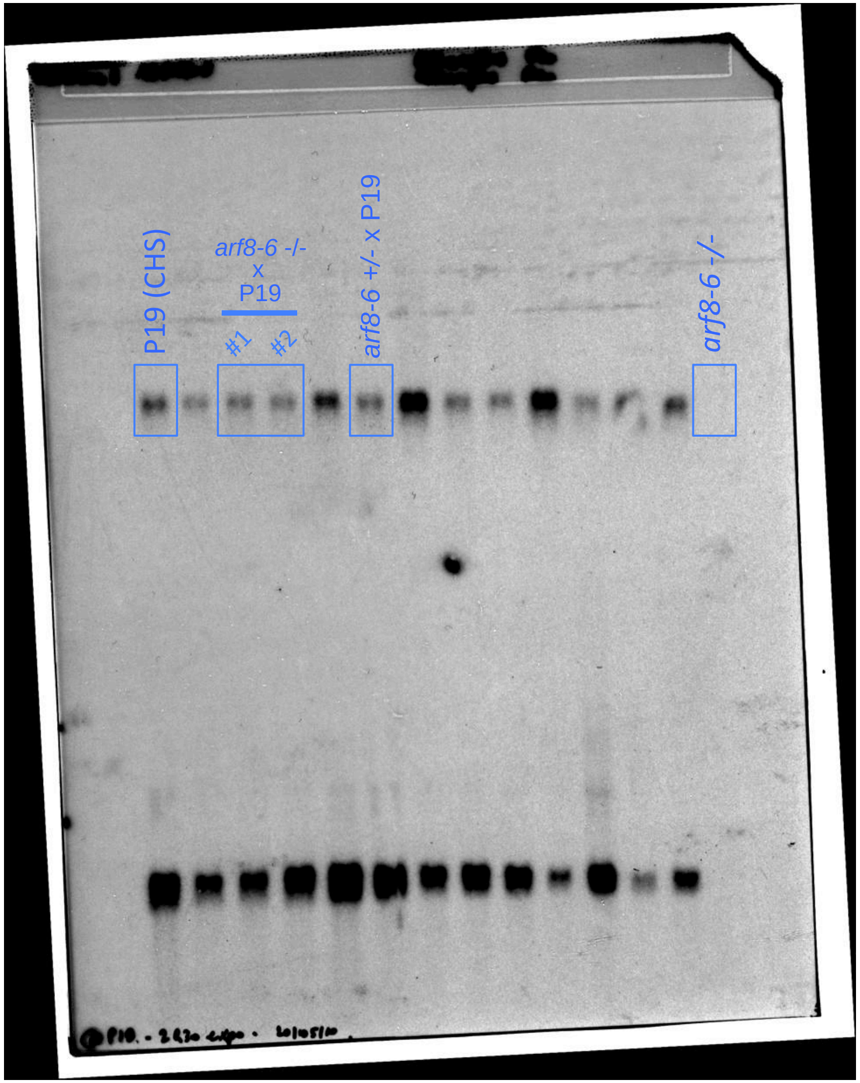

rRNA (pre-loading)

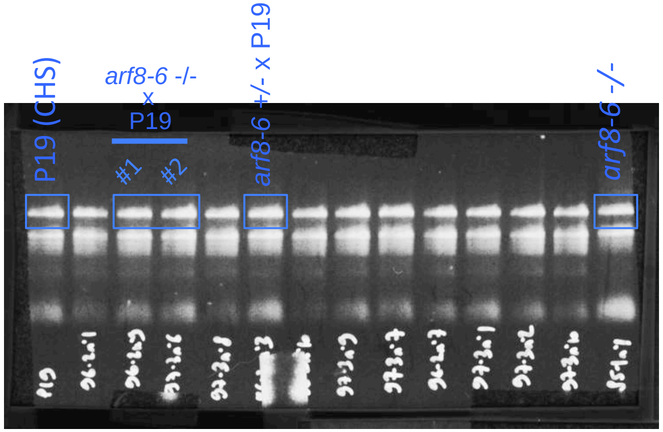

New Figure 5

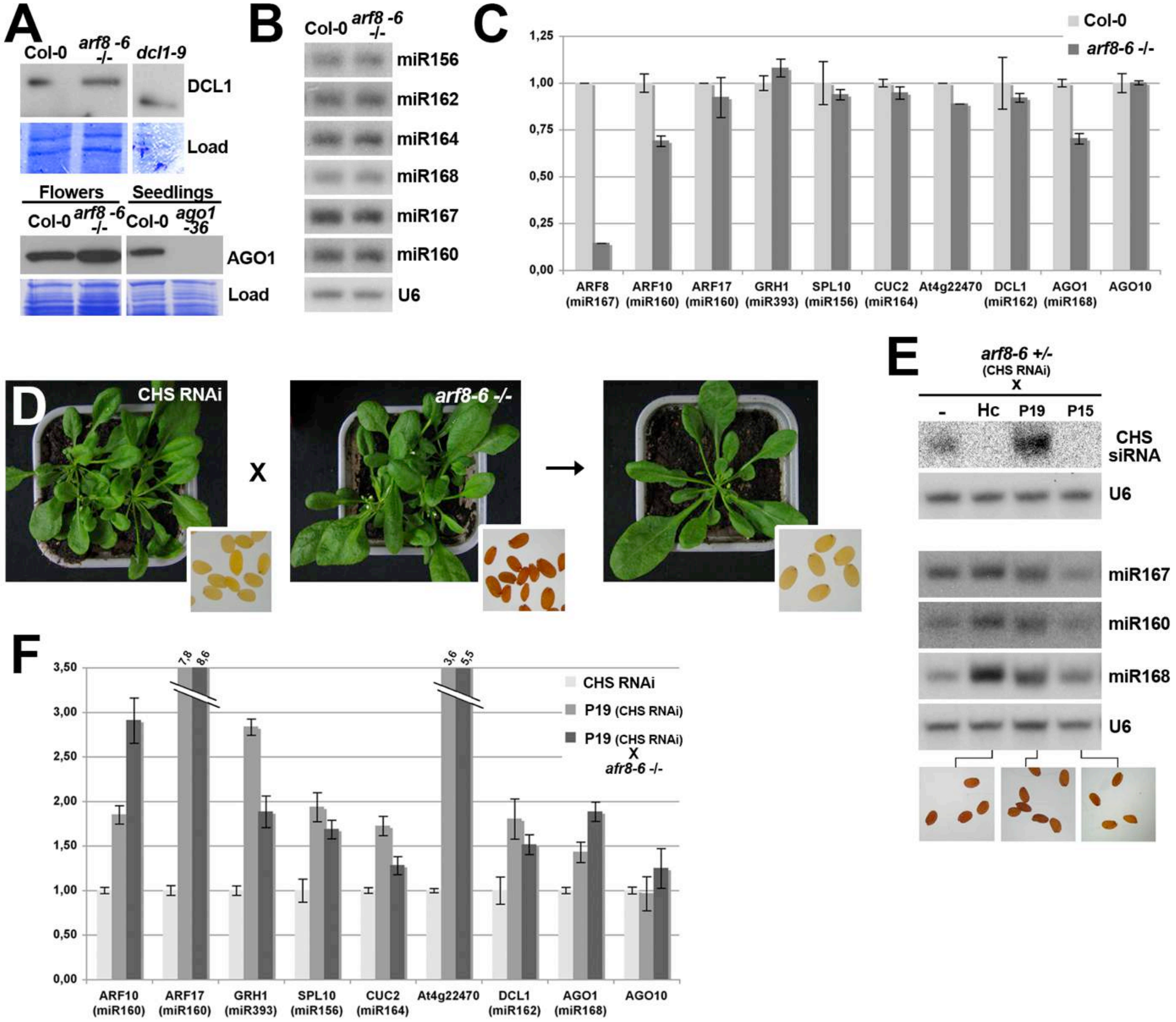
A
Col-0
arf8 -6 -/-
dcl1-9
DCL1
Load
Flowers
Seedlings
Col-0
arf8 -6 -/-
Col-0
ago1 -36
AGO1
Load
B
Col-0
arf8 -6 -/-
miR156
miR162
miR164
miR168
miR167
miR160
U6
C
Col-0
arf8-6 -/-
1,25
1,00
0,75
0,50
0,25
0,00
ARF8 (miR167)
ARF10 (miR160)
ARF17 (miR160)
GRH1 (miR393)
SPL10 (miR156)
CUC2 (miR164)
At4g22470
DCL1 (miR162)
AGO1 (miR168)
AGO10
D
CHS RNAi
x
arf8-6 -/-
E
arf8-6 +/-
(CHS RNAi)
x
-
Hc
P19
P15
CHS siRNA
U6
miR167
miR160
miR168
U6
F
3,50
3,00
2,50
2,00
1,50
1,00
0,50
0,00
7,8
8,6
3,6
5,5
CHS RNAi
P19 (CHS RNAi)
P19 (CHS RNAi) X afr8-6 -/-
ARF10 (miR160)
ARF17 (miR160)
GRH1 (miR393)
SPL10 (miR156)
CUC2 (miR164)
At4g22470
DCL1 (miR162)
AGO1 (miR168)
AGO10

Panel 5A

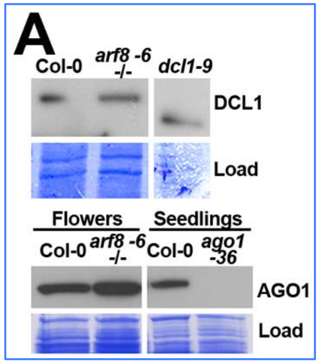

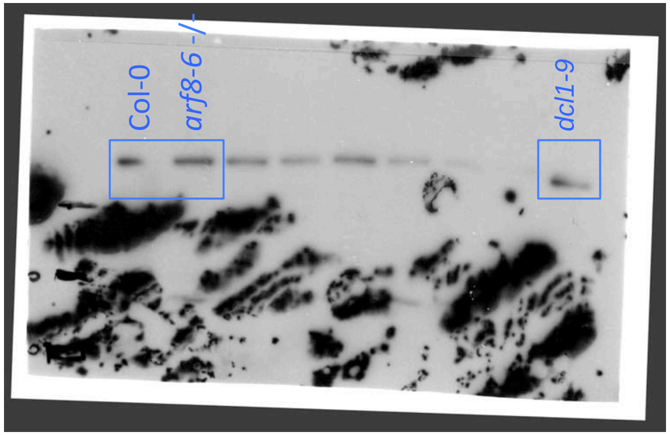

DCL1

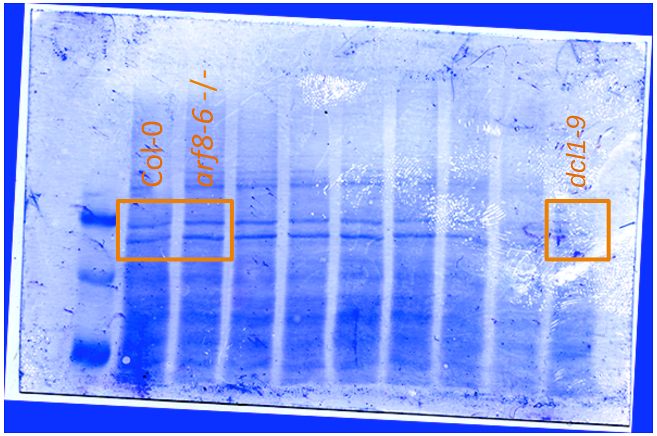

Coom.

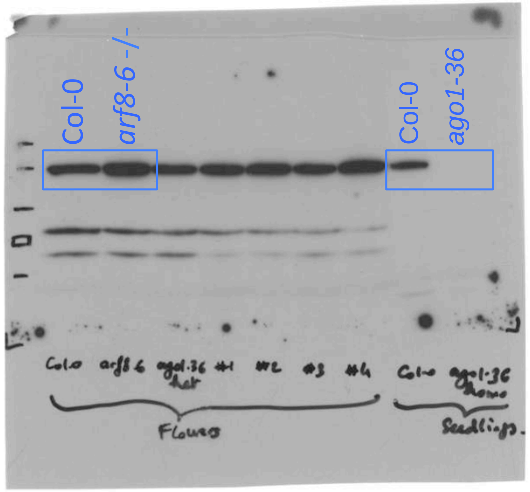

AGO1

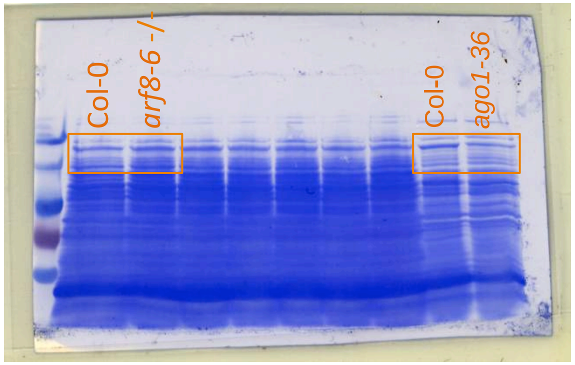

Coom.

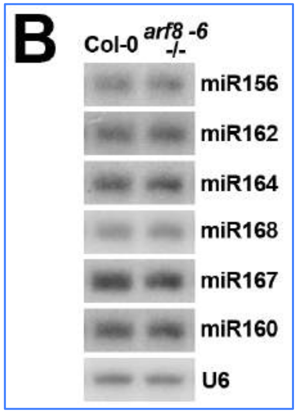

Panel 5B

miR156

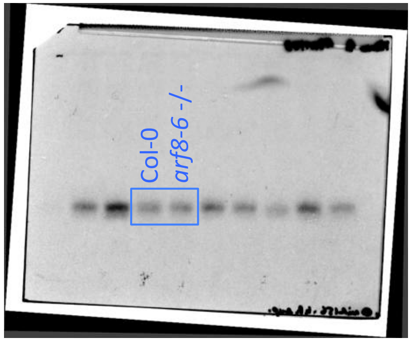

miR162

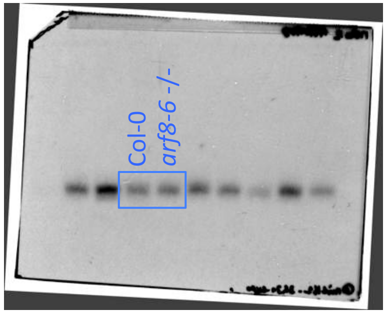

miR164

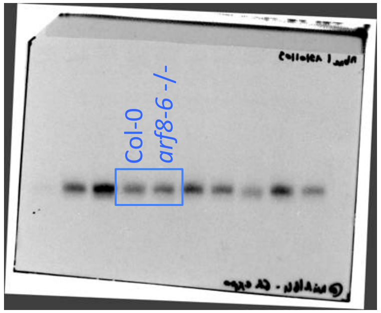

miR168

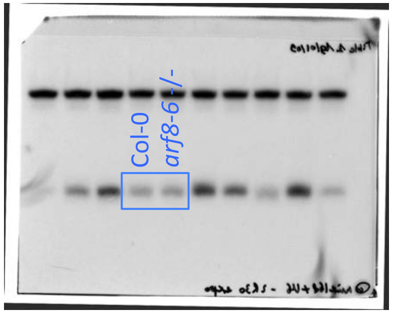

miR167

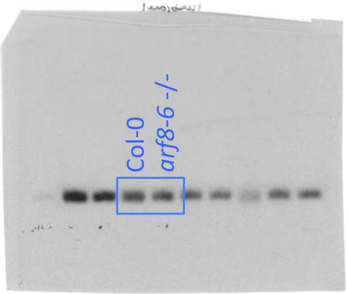

miR160

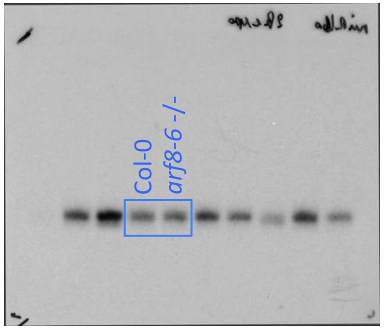

U6

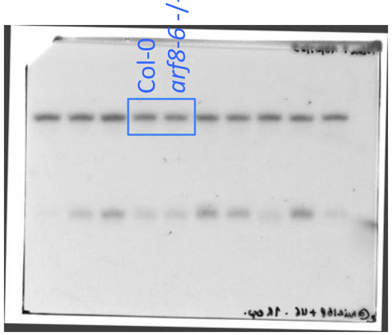

Panel 5E

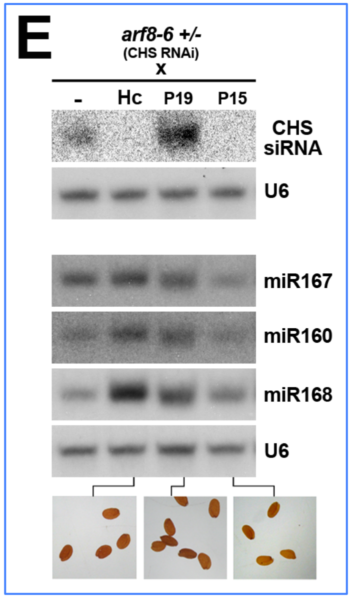

miR167
Membrane 06-10-08-10

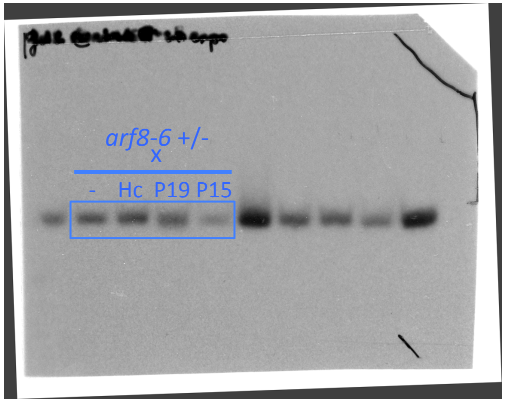

miR168
Membrane 06-10-08-10

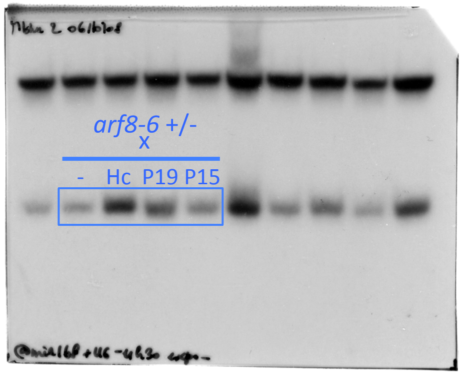

CHS-siRNA
Membrane 19-01-09

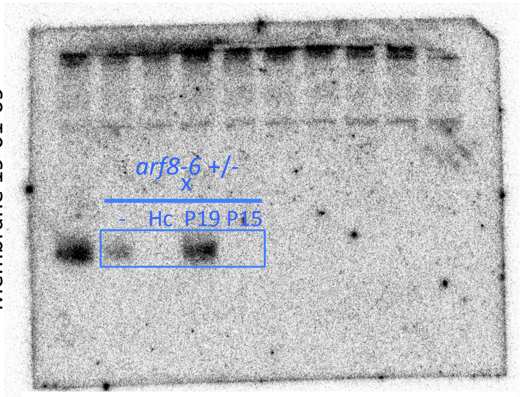

U6
Membrane 19-01-09

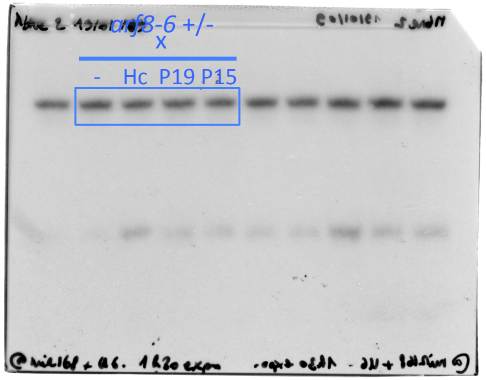

miR160
Membrane 06-10-08-10

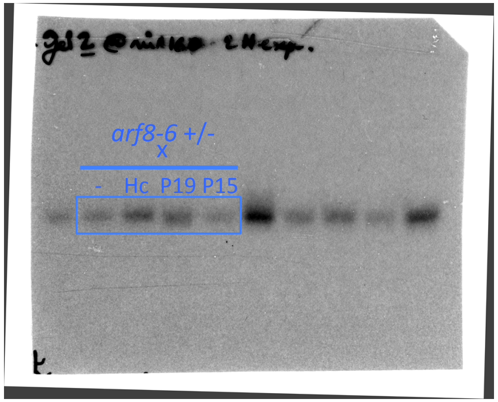

U6
Membrane 06-10-08-10

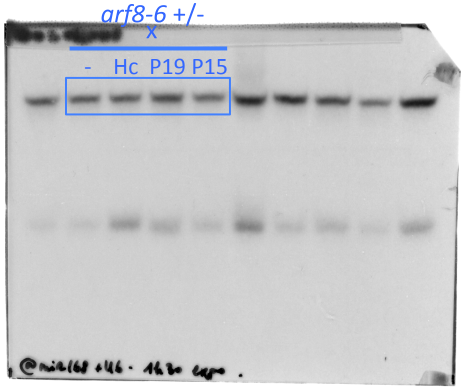

New Figure 6

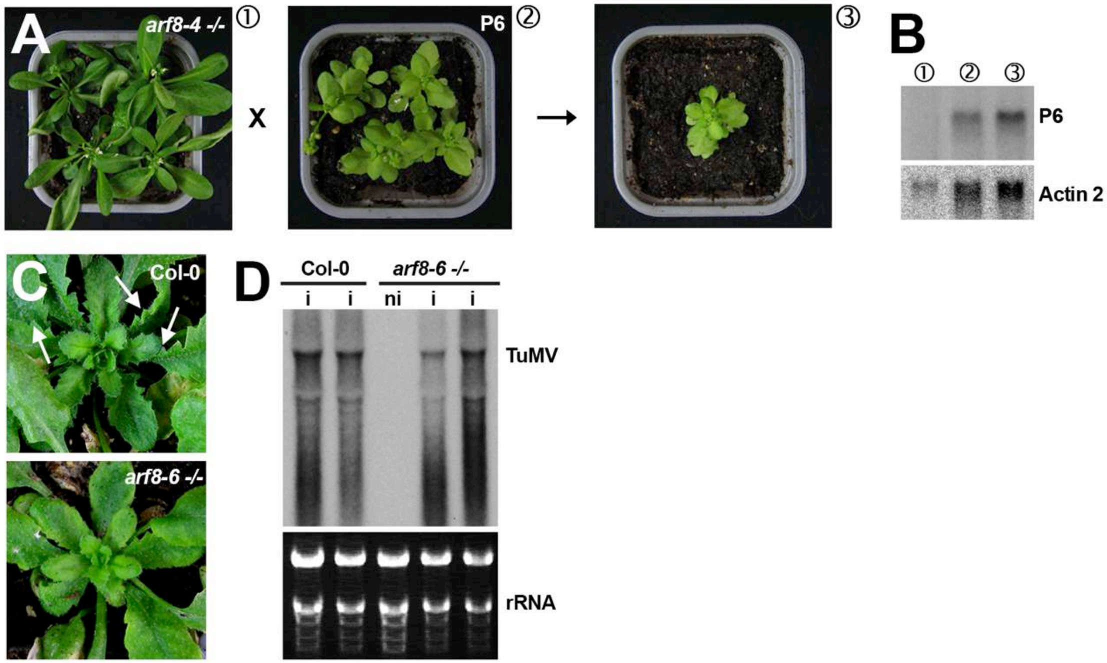
A
arf8-4 -/-
①
x
P6
②
③
B
① ② ③
P6
Actin 2
C
Col-0
arf8-6 -/-
D
Col-0
arf8-6 -/-
i i ni i i
TuMV
rRNA

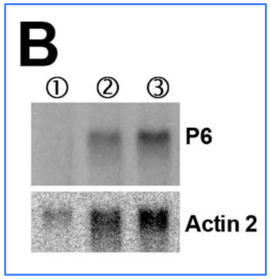

Panel 6B

P6

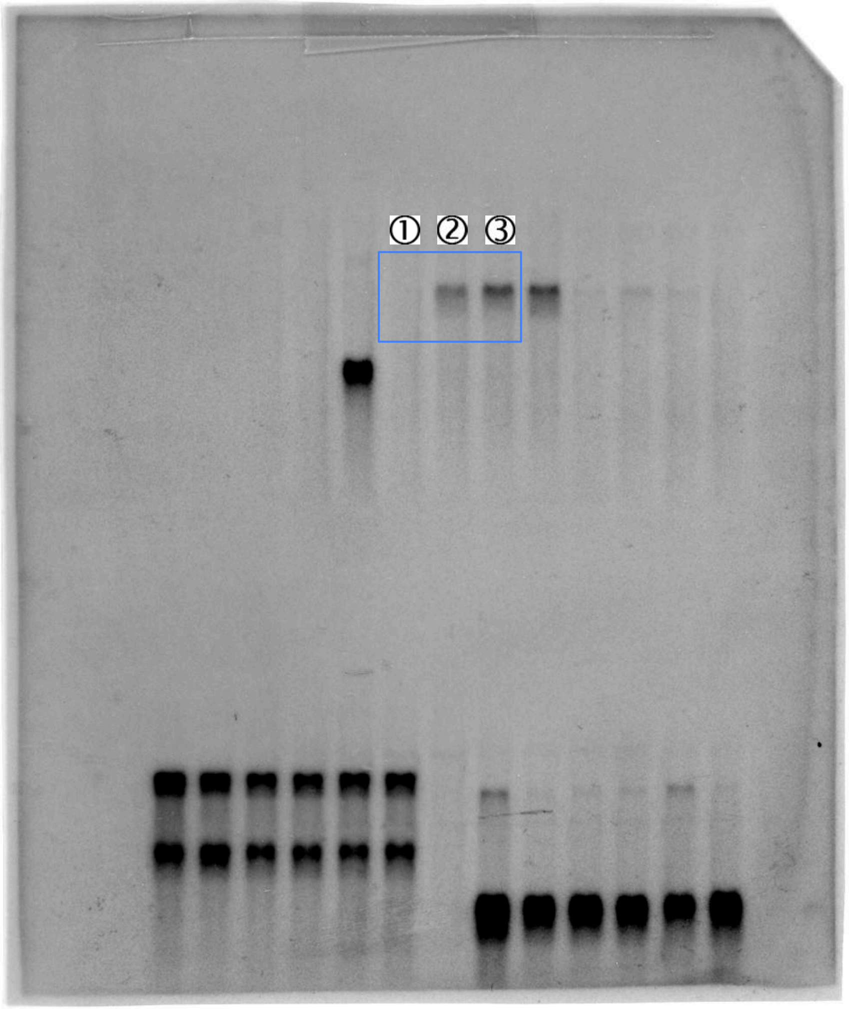

Actin 2

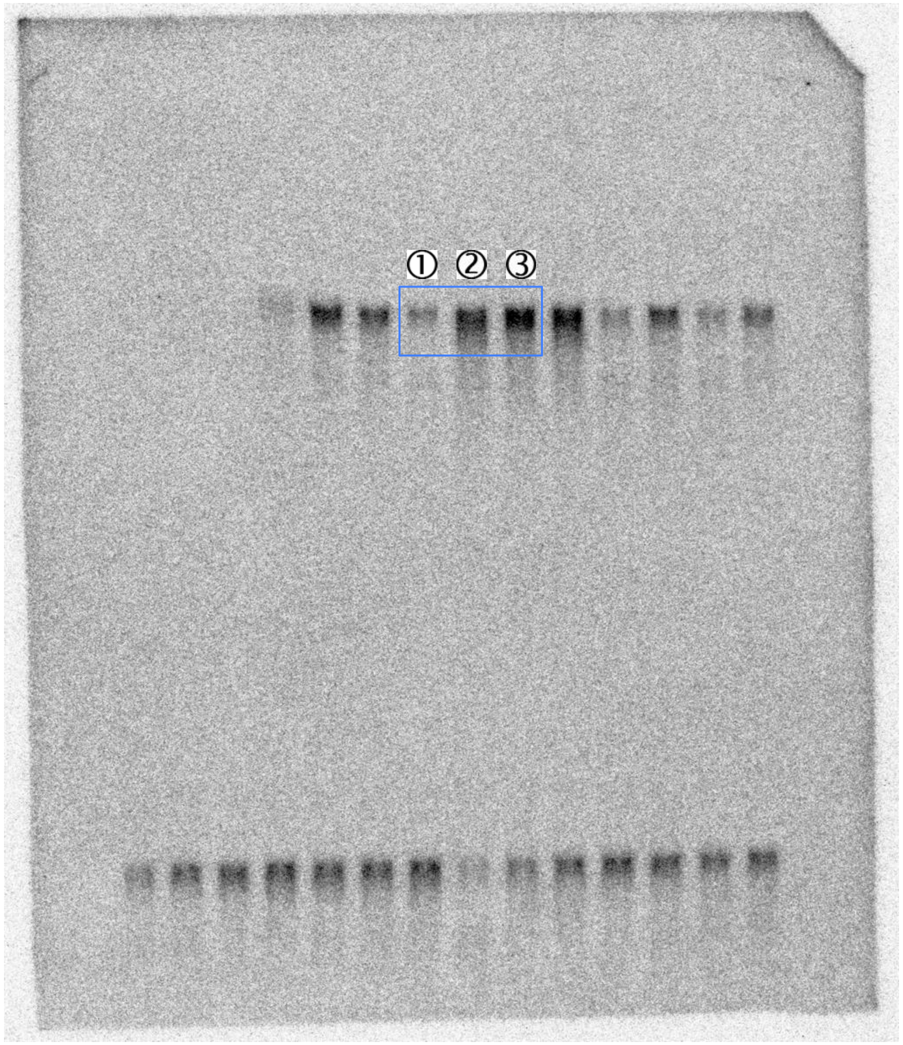

Supplement: S9 File — (PDF) [file ppat.1005627.s009.pdf]
